# Tissue-specific endothelial cell heterogeneity contributes to unequal inflammatory responses

**DOI:** 10.1038/s41598-020-80102-w

**Published:** 2021-01-21

**Authors:** Hasitha Gunawardana, Tahmineh Romero, Ning Yao, Sebastiaan Heidt, Arend Mulder, David A. Elashoff, Nicole M. Valenzuela

**Affiliations:** 1 Department of Pathology and Laboratory Medicine, David Geffen School of Medicine, University of California Los Angeles, 1000 Veteran Avenue, Room 1-520, Los Angeles, CA 90095 USA; 2 Statistics Core, David Geffen School of Medicine, University of California, Los Angeles, CA USA; 3 Department of Immunohaematology and Blood Transfusion, Leiden University Medical Center, Leiden, The Netherlands

**Keywords:** Inflammation, Allotransplantation

## Abstract

Endothelial cells (EC) coordinate vascular homeostasis and inflammation. In organ transplantation, EC are a direct alloimmune target. We posited that tissue specific heterogeneity of vascular EC may partly underlie the disparate organ-specific alloimmune risk. We examined the vascular endothelial response to inflammation across six primary endothelial beds from four major transplanted organs: the heart, lung, kidney and liver. First, we reanalyzed a public dataset of cardiac allograft rejection and found that endothelial inflammatory response genes were elevated in human cardiac allograft biopsies undergoing rejection compared with stable grafts. Next, the inducible inflammatory phenotypes of EC from heart, lung, kidney, and liver were characterized in vitro, focused on expression of adhesion molecules and chemokines, and recruitment of allogeneic peripheral blood mononuclear immune cells. Large vessel cardiac EC most highly upregulated VCAM-1, particularly compared with hepatic EC, supported greater leukocyte adhesion and had distinct chemokine profiles after stimulation with cytokines and complement. Differentially expressed gene candidates that are known regulators of cytokine signaling and inflammatory programming were verified in publicly available datasets of organ-specific endothelial transcriptomes. In summary, differential baseline expression of immune regulating genes may contribute to differential vascular inflammatory responses depending on organ.

## Introduction

Solid organ transplantation is the definitive therapy for patients with end-stage organ failure, patients who would otherwise die without vital organ function. Nearly 40,000 patients received kidney, heart, liver, lung or visceral transplants in the United States in 2019 alone, and more than three-quarters of a million people have been transplanted in the US since 1988. In the last two decades, advances in immunosuppression and surgical techniques have improved short-term and long-term graft, and patient survival post-transplant. However, the expected length of function of transplanted organs still does not match patient life-expectancy. Consequently, many patients will require multiple transplants in their lifetime, further contributing to the shortage of organs. Moreover, patient management and graft survival are disparate across transplanted organ types. The expected half-life of a kidney transplant is 8–12 years and of a heart allograft 10–13 years, while lung and bowel grafts fare particularly poorly with 50% failure around 5 years. One major limitation to long-term transplant success is rejection of the donor organ due to recognition of alloantigen, via cellular or antibody/complement-mediated immune attack of the donor tissue. Cardiac and lung transplant recipients experience higher rates of acute and chronic rejection and rejection-attributable graft failure compared with liver allograft recipients, despite lower immunosuppression burdens in liver transplantation^1–3^.

Many cell types are involved in the alloimmune process of allograft rejection, including recipient innate and adaptive immune cells, donor parenchymal cells, and donor vascular cells^4–6^. Modern mainstay immunosuppression primarily targets activation (calcineurin-dependent) and proliferative signaling in the recipient’s T cell compartment, but does not prevent function of these other cellular players^7^. The infiltration of recipient leukocytes into the donor tissue is an important early manifestation and pathogenic response in transplant rejection, and is a process tightly regulated by endothelial cell function. Vascular endothelial cells (EC) actively regulate local inflammation, by upregulating adhesion molecules and secreting chemokines which promote leukocyte trafficking^8^. Donor endothelial cells are the first allogeneic cell encountered by the recipient’s immune system, at the direct interface in contact with the recipient’s perfused blood. Although the liver is generally considered to be “tolerogenic,” specific mechanisms therein contributing to lower transplant rejection rates have not been fully elucidated. Recent work has revealed that vascular EC exhibit tissue-specific molecular and functional heterogeneity^9–16^. Yet, whether there are divergent mechanisms by which EC govern leukocyte recruitment to the site of inflammation are not well-established. The majority of research investigating endothelial inflammation and leukocyte trafficking has employed cells from the descending aorta, neonatal foreskin, muscle or umbilical vein (HUVEC), which are not representative of organ-resident endothelium. Therefore, it is of high interest to determine whether there is also tissue-specific variation in endothelial regulation of inflammation.

We posited that the positional identity of thoracic endothelium differs from that of the liver endothelium in response to inflammatory stimuli and resultant leukocyte recruitment. EC from different anatomic locations exhibited distinct inflammatory phenotypes with respect to the magnitude and kinetics of inducible gene expression, particularly comparing endothelium of the heart to liver microvasculature. In addition, differential endothelial cell responses occurred to anti-HLA antibodies and human complement in an in vitro model of acute antibody-mediated injury. Finally, we identify 37 differentially expressed immune-related genes (DEGs) across ECs from different anatomic origins, which were confirmed in public datasets of murine and human endothelial gene expression.

## Results

### Endothelial cell inflammatory markers are increased in cardiac allograft rejection

**Figure 1:**
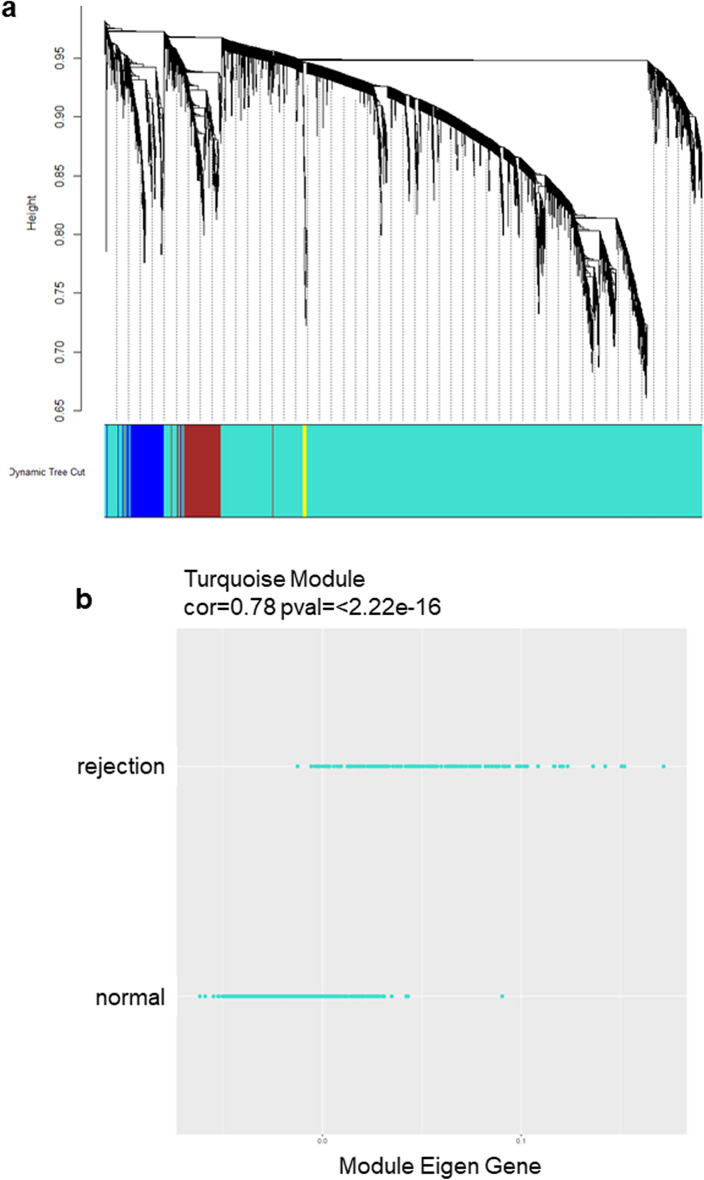

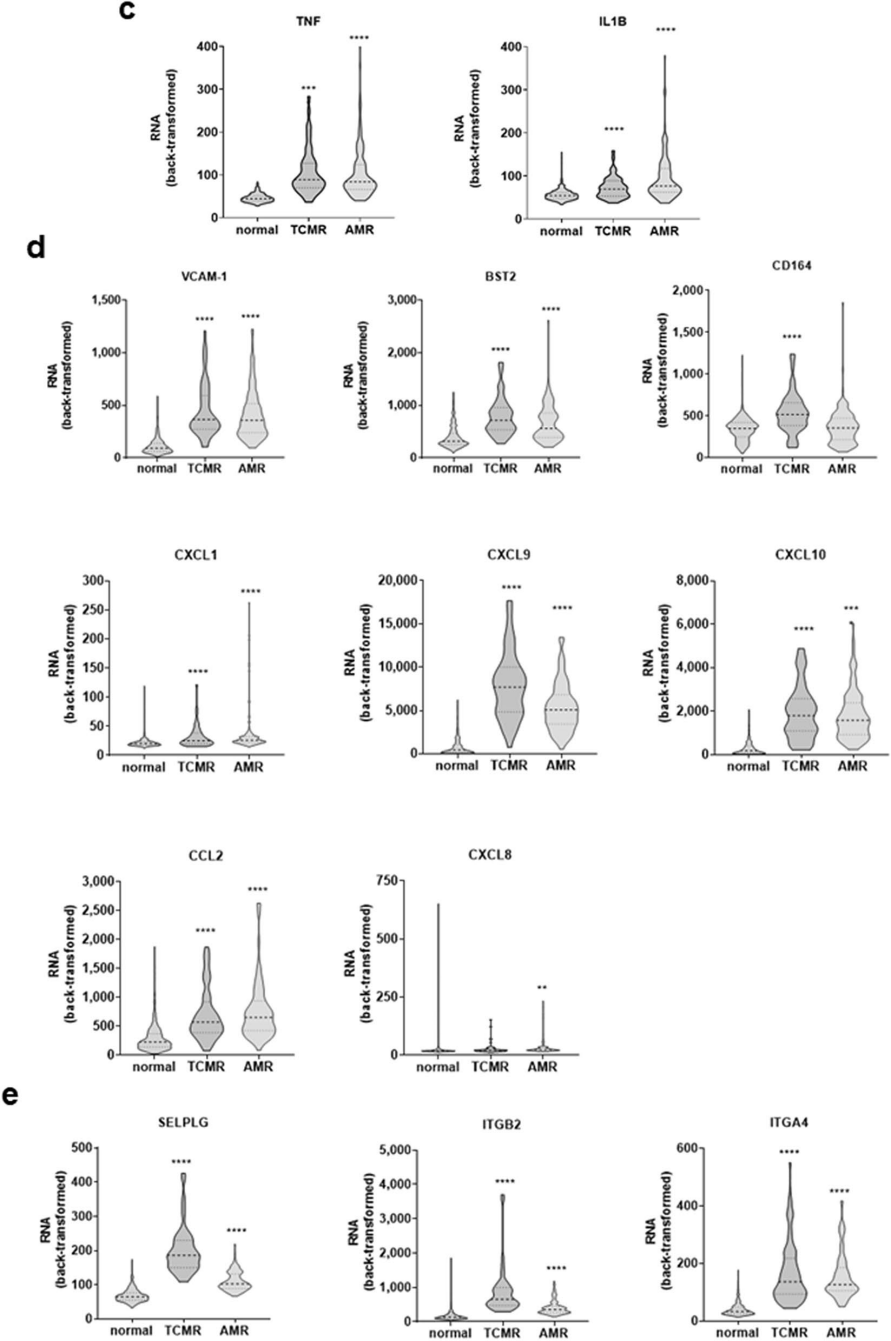
Weighted Correlation Network Analysis of transcript changes within cardiac allograft biopsies with rejection versus stable [GSE124897]. (**a**) Gene dendrogram and module colors for the top 5,000 genes with the most significant Wilcoxon test *p* value, comparing rejection to non-rejection/normal. The top 4 modules (turquoise, yellow, blue and brown) are shown. The branches indicate modules of interconnected gene groups. (**b**) Distribution of expression of genes within the Turquoise Module in biopsies with rejection versus non-rejection/normal. (**c**–**e**) Violin plots show the expression of inflammatory effector molecules and of leukocyte receptors for endothelial adhesion molecules in normal cardiac transplant biopsies and those with TCMR or ABMR. The backtransformed values for intragraft (**c**) proinflammatory cytokines TNF and IL1B. (**d**) endothelial adhesion and recruitment genes VCAM1, BST2, CD164/endolyn, CXCL1/GROα, CXCL9/MIG, CXCL10/IP-10, CCL2/MCP-1, CXCL8/IL-8; and (**e**) cognate leukocyte receptors SELPG/PSGL-1, ITGB2/LFA-1/Mac-1, and ITGA4/VLA-4 are shown.

In order to understand the inflammatory changes during transplant rejection, we performed an analysis of bulk transcript expression from publicly available datasets of clinical heart allograft biopsies. Grafts with histological diagnosis of rejection (acute cellular rejection (ACR) and antibody-mediated rejection (AMR)) were compared to normal grafts [GSE124897^17^]. Cardiac allograft biopsy data was divided into training (75%) and test (25%) sets based on the distribution of rejection/normal and rejection type (Table 1). Next, weighted gene co-expression network analyses (WGCNA) procedure grouped the top 5,000 genes into 4 modules and 1 unassigned (Grey) module (Fig. 1a). The Turquoise Module is the largest module with 4,314 genes and showed the highest correlation with rejection versus normal (r = 0.77, *p* < 0.001). Genes in this module were enriched in immune response and leukocyte activation, and were increased in rejection compared to normal biopsies (Figs. 1b, S1). Gene ontology analysis^18^ showed that 50 genes within the Turquoise Module were involved in cell–cell adhesion, and 11 in leukocyte cell–cell adhesion. The full gene list is available in Supplementary File 1.

**Table 1:** Description of dataset.

Type	Sample size N=889	Training set (75%) N = 634	Test set (25%) N = 207	Status
Normal	626 (70%)	472 (74.4%)	154	Normal
Rejection -1 (T cell mediated)	55 (6%)	42 (6.6%)	13	Rejection
Rejection -2 (antibody mediated)	160 (18%)	120 (20%)	40	
Injury	48 (5%)	X	X	Excluded
***Total analytical sample***	***841***	***634***	***207***

To gain insight into the vascular component of the rejection response, we delved further into this gene module to understand whether inflammatory cytokines and their known inducible endothelial immune response genes were altered during rejection. Transcripts for the inflammatory cytokines TNFα and IL-1β were significantly increased in rejection compared with normal biopsies (TNFα: TCMR, 2.31-fold; AMR, 2.25-fold; IL-1β: TCMR, 1.30-fold; AMR, 1.68-fold; *p* < 0.0001) (Fig. 1c). Since TNFα and IL-1β activate inflammatory responses in EC and initiate recruitment of leukocytes, we assessed endothelial adhesion molecules and chemokines. The endothelial adhesion molecule VCAM-1 was elevated 4.08-fold in TCMR and 3.70-fold in ABMR (*p* < 0.0001 compared to normal). Other adhesion molecules, SELE (E-selectin), ICAM1, BST2 and CD164 (endolyn), and chemokines CCL2/MCP-1, CXCL8/IL-8, CXCL1/GROα, CXCL10/IP-10, and CXCL9/MIG, were also higher in rejection biopsies (Fig. 1d). Further, leukocyte receptors for endothelial adhesion mediators, ITGA4/VLA-4, CX3CR1, SELPLG/PSGL-1 and ITGB2/LFA-1/Mac-1 were significantly enriched in abnormal biopsies (Fig. 1e). An inflammation and immune response module (Turquoise) correlated with rejection included many genes involved in vascular inflammation and leukocyte recruitment. These data show that cardiac allografts undergoing rejection had increased expression of transcripts associated not only with quiescent endothelial markers, as reported^17^, but also effector molecules commonly upregulated during endothelial inflammatory activation and leukocyte recruitment.

### Cytokine-induced endothelial activation

#### Allogeneic PBMC adhesion

**Figure 2:**
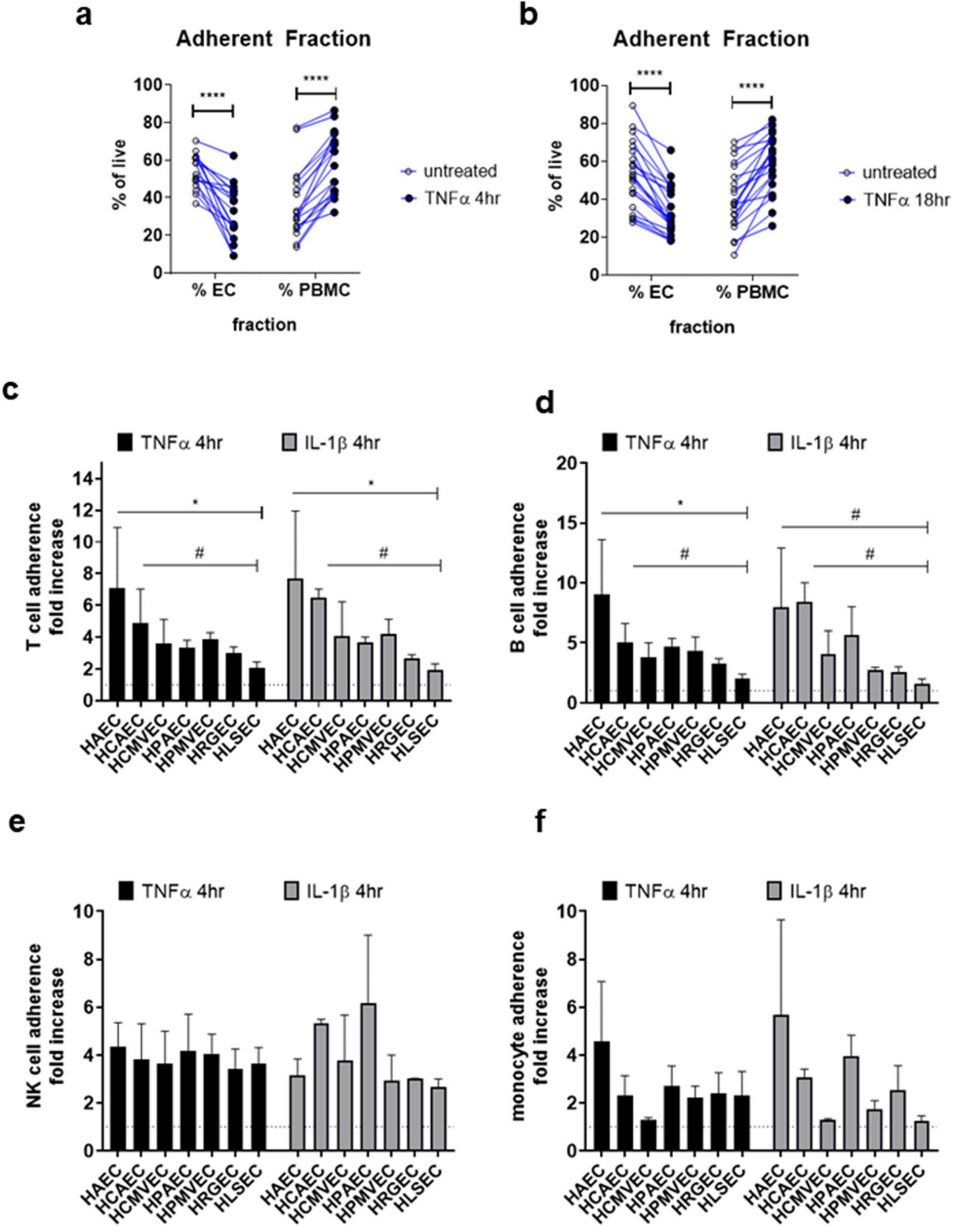
Differential adherence of allogeneic PBMC to cytokine-activated endothelial cells. Endothelial monolayers were stimulated with TNFα (20 ng/mL) or IL-1β (20 ng/mL) for 4 h or 18 h. Stimulation medium was removed, and whole PBMC fractions were added at a ratio of 3 PBMC to 1 endothelial cell and allowed to adhere for 45 min. Nonadherent cells were removed by two washes with HBSS with Ca^2+^ and Mg^2+^, and adherent cells were detached by a third wash with PBS without Ca^2+^ and Mg^2+^ followed by treatment Accutase. Adherent cells were stained with an immunophenotyping panel and acquired by flow cytometry. (**a**) Endothelial cells and total PBMC as a percent of live cells in the adherent fraction is shown in the spaghetti plots, comparing untreated endothelial cells with (**a**) TNFα 4 h-treated EC and (**b**) TNFα 18 h-treated EC. Each pair represents a unique experiment (n = 21). ****p < 0.0001. (**c**) Adherence of T cells (gated on CD105^neg^ CD3^+^) to endothelial cells (gated on CD11a^neg^ CD105^+^) from different vascular beds after pre-activation with TNFα at 4 h (black bars) or IL-1β at 4 h (grey bars). #*p* < 0.1, *p < 0.05 comparing HAEC or HCAEC to HLSEC. (**d**) Adherence of B cells (gated on CD105^neg^ CD19^+^) to endothelial cells (gated on CD11a^neg^ CD105^+^) from different vascular beds after pre-activation with TNFα at 4 h (black bars) or IL-1β at 4 h (grey bars). #*p* < 0.1, *p < 0.05 comparing HAEC or HCAEC to HLSEC. (**e**) Adherence of NK cells (gated on CD105^neg^ CD3^neg^ CD19^neg^ CD56^+^) to endothelial cells (gated on CD11a^neg^ CD105^+^) from different vascular beds after pre-activation with TNFα at 4 h (black bars) or IL-1β at 4 h (grey bars). #*p* < 0.1, *p < 0.05 comparing HAEC or HCAEC to HLSEC. (**f**) Adherence of monocytes (gated on CD105^neg^ CD3^neg^ CD19^neg^ CD14^+^) to endothelial cells (gated on CD11a^neg^ CD105^+^) from different vascular beds after pre-activation with TNFα at 4 h (black bars) or IL-1β at 4 h (grey bars). #*p* < 0.1, *p < 0.05 comparing HAEC or HCAEC to HLSEC. Results are presented as the fold increase in the ratio of PBMC to endothelial cells as mean ± SEM (n = 3 unique EC-PBMC donor combinations).

Both TNFα and IL-1β transcripts were significantly increased in cardiac allografts with rejection compared with stable grafts. These proinflammatory cytokines have well-known effects on vascular endothelial cells. Therefore, primary EC from human heart, lung, kidney and liver were left untreated or stimulated with TNFα or IL-1β for 4 h or 18 h in vitro. We measured subsequent adherence of primary allogeneic peripheral blood mononuclear cells (PBMC) to endothelial cells from different vascular beds, using an immunophenotyping adherence assay we developed. The gating strategy is presented in Figure S2. Binding of PBMC increased by 1.51-fold (*p* < 0.0001) when EC were pre-treated with TNFα for 4 h, and 1.48-fold at 18 h (Figs. 2a,b show aggregate results across EC types).

We next used immunophenotyping by flow cytometry to determine the relative proportions of T cells, B cells, NK cells and monocytes adherent to endothelium. The proportion of T cells in the adherent fraction increased an average of 3.76-fold by endothelial activation with TNFα (4 h) (Figure S3a). Similarly, the proportions of adherent B cells, NK cells and monocytes were also significantly increased when endothelial cells were pre-activated with TNFα (Figure S3a). Cycloheximide (CHX) pre-treatment of EC prior to TNFα activation abrogated leukocyte adhesion, demonstrating that recruitment of PBMC was dependent on new endothelial transcription (*data not shown*).

We then specifically compared allogeneic PBMC adhesion to EC from the heart, lung, liver and kidney. We quantitated the ratio of adherent T cells (Fig. 2c), B cells (Fig. 2d), NK cells (Fig. 2e) and monocytes (Fig. 2f) to endothelial cells from different tissue sources in each experiment. Liver endothelium supported the lowest recruitment of overall PBMC to TNFα or IL-1β, as well as individual subsets of T cells and B cells, but not NK cells and monocytes. For example, there was a 7.1 ± 3.8-fold increase in T cells adherent to HAEC with TNFα 4 h compared with only a 2.03 ± 0.4-fold increase in PBMC adherent to HLSEC under the same conditions (Fig. 2c). Similarly, B cell adhesion to TNFα activated HAEC was increased by 9.03 ± 4.5-fold, but only 1.99 ± 0.39-fold for HLSEC. The differences were most pronounced at 4 h (Figure S3b–e), although 18 h stimulation of endothelial cells with TNFα or IL-1β also showed lower adherence of PBMC to HLSEC compared with other EC types (Figure S3f.–i). These results show that endothelial cells from the liver exhibit lower adherence of allogeneic lymphocytes after cytokine activation, illustrating the heterogeneity of EC in response to inflammatory stimuli.

#### Chemokines

**Figure 3:**
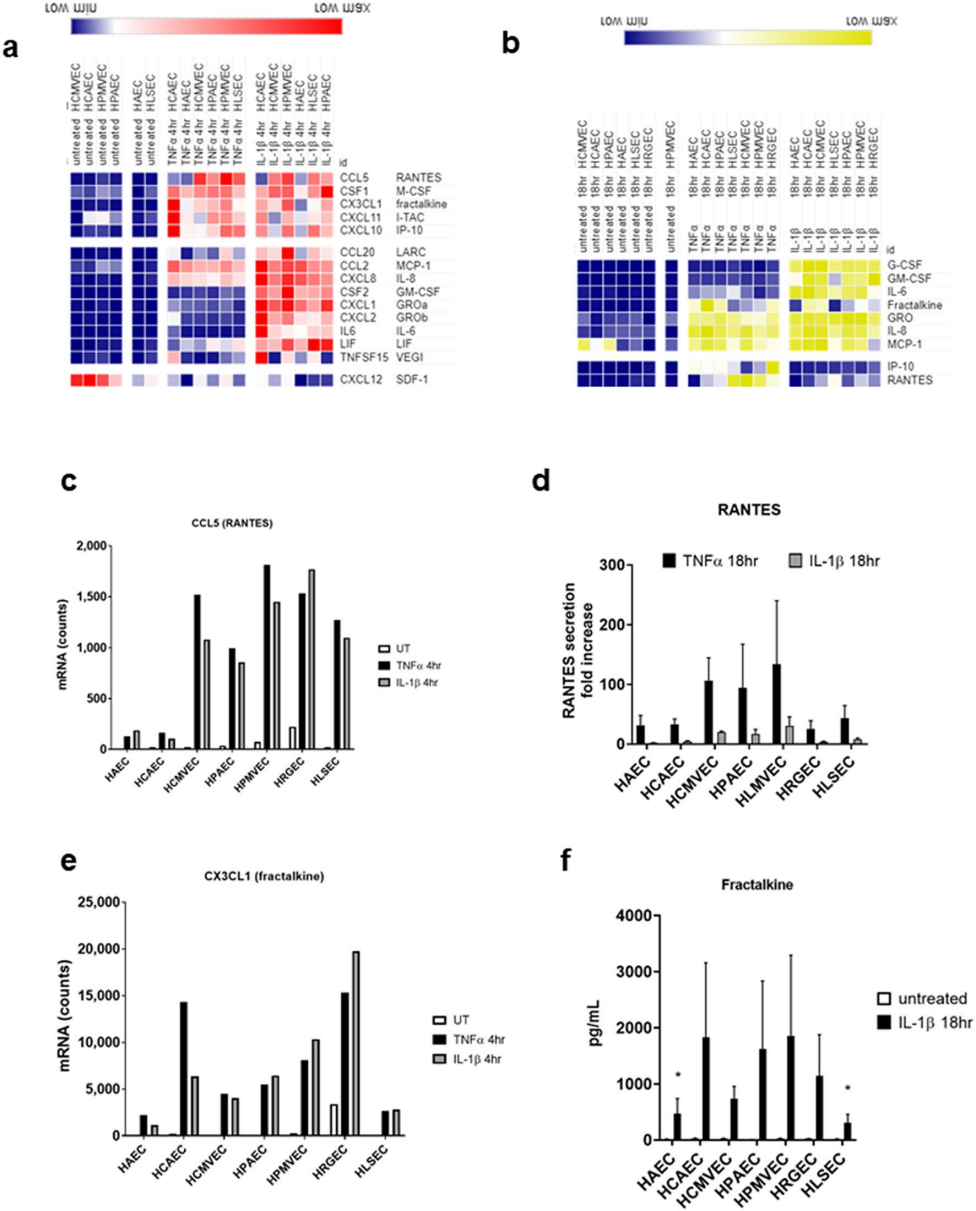
Chemokine and cytokine production by cytokine-activated endothelial cells. Endothelial monolayers were stimulated with TNFα (20 ng/mL) or IL-1β (20 ng/mL). (**a**) After 4 h, stimulated endothelial cells were lysed in RLT buffer, and mRNA for immune response genes was measured by Nanostring. Results are presented in the heat map, with hierarchical clustering and color scale representing relative mRNA counts of each chemokine across conditions. Heat maps is transformed by row min/max with hierarchical clustering using one minus Pearson correlation and grouped by stimulus. (**b**) After 18 h, conditioned media were tested for secreted factors by Luminex and ELISA. Results are presented in the heat map, with hierarchical clustering and color scale normalized to represent relative protein concentrations (pg/mL) of each secreted chemokine protein across conditions (n = 5). (**c**) Normalized mRNA counts for CCL5 (RANTES) gene expression across endothelial cells stimulated for 4 h with TNFα or IL-1β. (**d**) Fold increase in the concentration of secreted RANTES (CCL5) protein after 18 h across endothelial cells (n = 3). Results are presented as mean ± SEM. TNFα-induced RANTES was significantly lower from HAEC and HCAEC compared with HCMVEC, HPMVEC, and HLSEC (all *p* < 0.001, by two way ANOVA followed by Tukey’s multiple comparison’s test). IL-1β-induced RANTES was significantly lower from HAEC than microvascular EC (*p* < 0.05). (**e**) Normalized mRNA counts for CX3CL1 (fractalkine) gene expression across endothelial cells stimulated for 4 h with TNFα or IL-1β. (**f**) Concentration of secreted fractalkine (CX3CL1) protein after 18 h across endothelial cells measured by Luminex (n = 4). Results are presented as mean ± SEM. IL-1β-induced fractalkine was significantly lower from HAEC and HLSEC compared with HCAEC and HPMVEC (all *p* < 0.05, by two way ANOVA followed by uncorrected Fisher’s LSD).

We focused on chemokine mRNA induction (4 h, Fig. 3a) and secretion (18 h, Fig. 3b) in thoracic and hepatic EC activated with TNFα or IL-1β. Comparing across endothelial cell types, there was no overt impairment of liver endothelium to produce the canonically TNFα or IL-1β-responsive chemokines like CXCL8/IL-8, CCL2/MCP-1 and CXCL1/GROα (Fig. 3a,b). Yet, we did observe differential patterns of production of other chemokines. For example, unstimulated heart and lung EC constitutively expressed CXCL12/SDF-1, while liver EC expressed comparatively lower levels (Fig. 3a). Aortic, coronary artery and pulmonary artery EC produced less CCL5/RANTES in response to TNFα compared with microvascular endothelium, which was observed at both the mRNA level and in secreted protein (mRNA: Fig. 3c, protein: Fig. 3d). Moreover, CX3CL1/fractalkine production after cytokine stimulation was low in both HAEC and HLSEC, compared with other vascular beds (mRNA: Fig. 3e, protein: Fig. 3f). Discrete mRNA counts for all chemokine genes are presented in Figure S4a–o.

#### Adhesion molecules

**Figure 4:**
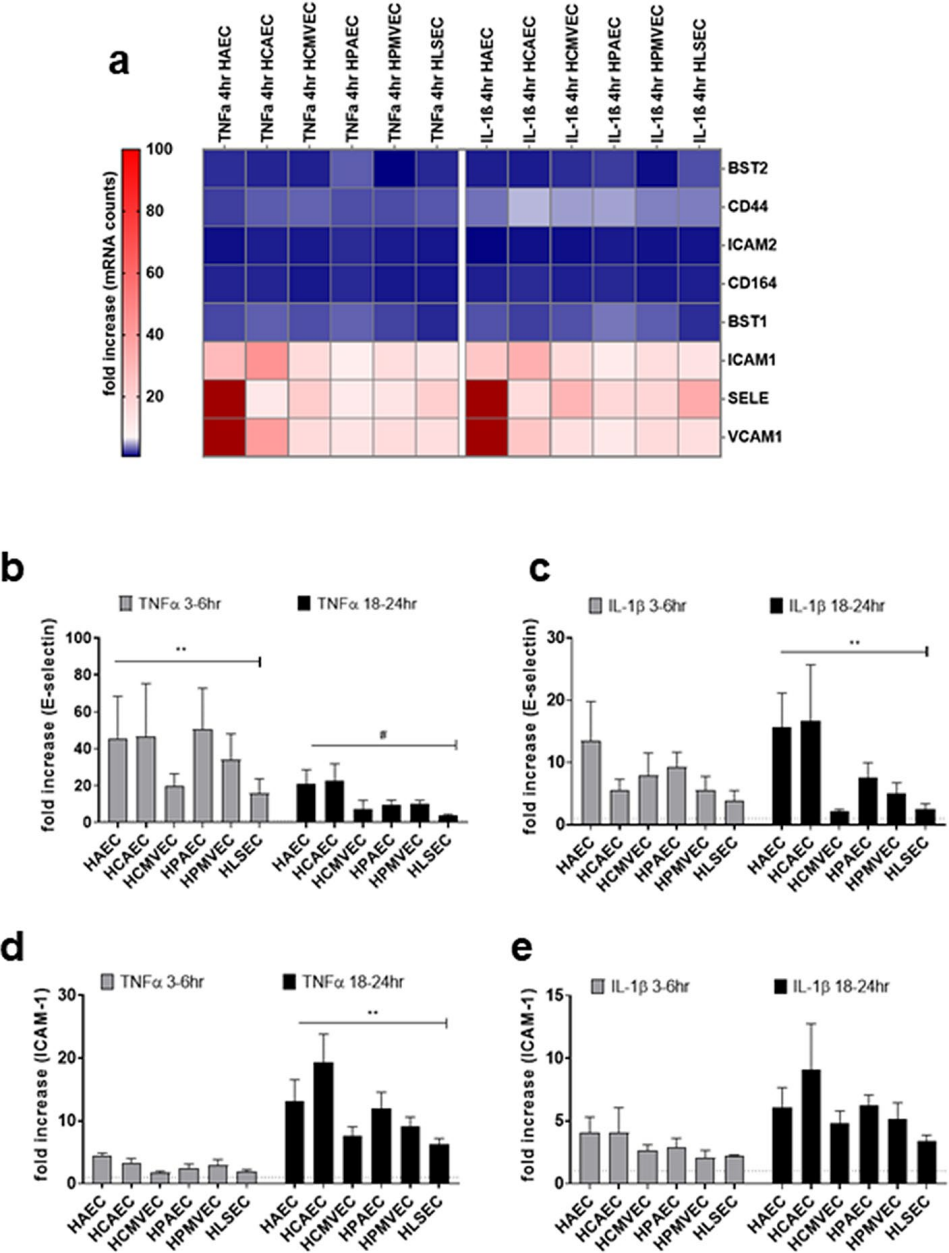

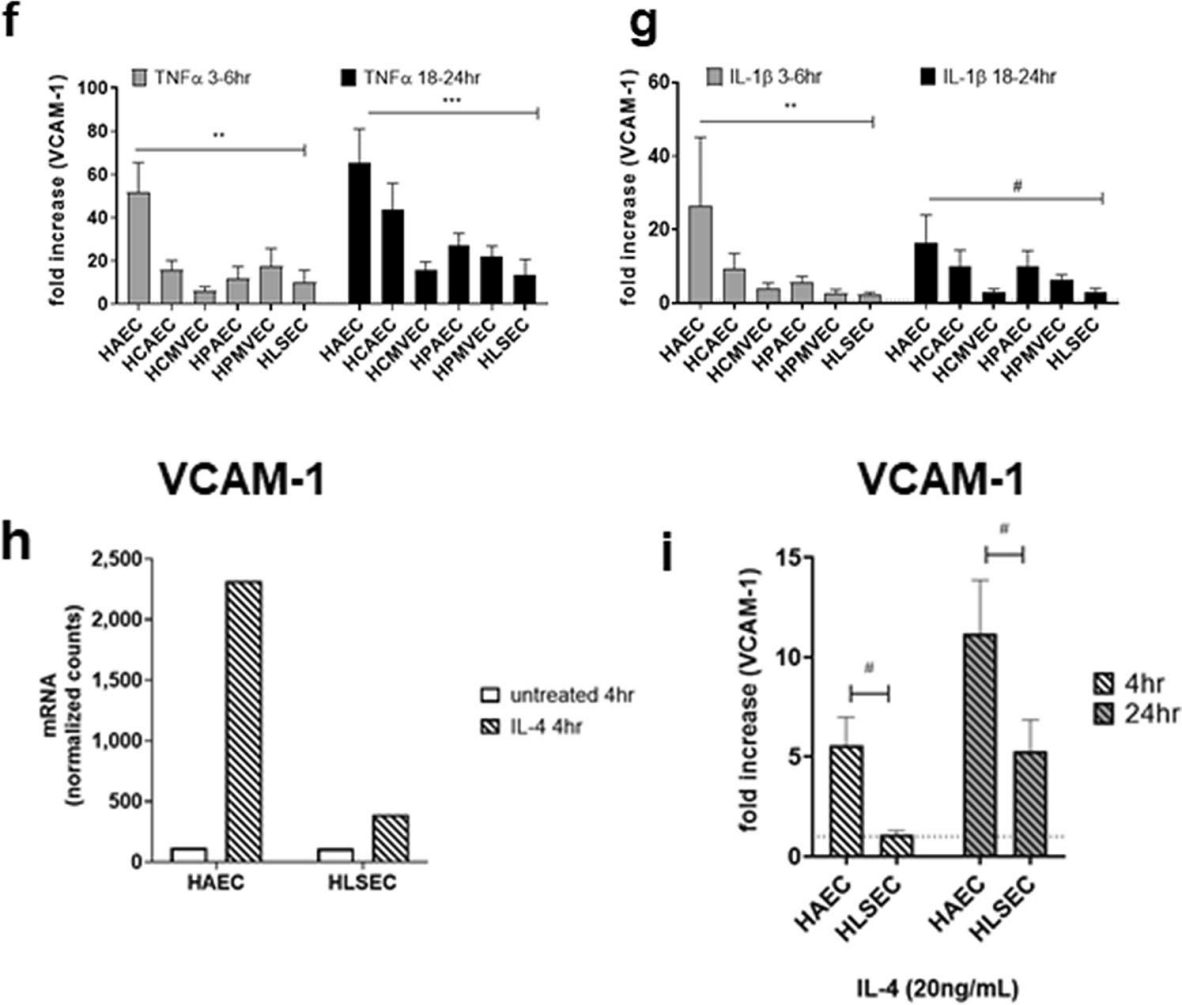
Adhesion molecule expression by cytokine-activated endothelial cells. (**a**) After 4 h activation with TNFα (20 ng/mL) or IL-1β (20 ng/mL), stimulated endothelial cells were lysed in RLT buffer, and mRNA for immune response genes was measured by nanostring. Absolute mRNA counts for endothelial adhesion molecule genes are presented in the heat map. (**b**, **c**) After 4 h or 18-24 h, stimulated endothelial cells were detached and TNFα (**b**) and IL-1β (**c**) induced cell surface E-selectin was measured by flow cytometry. Results are presented as fold increase in the median fluorescence intensity of cell surface E-selectin, normalized within each experiment to the untreated condition for each cell type.(**d**, **e**) After 4 h or 18-24 h, stimulated endothelial cells were detached and TNFα (**d**) and IL-1β (**e**) induced cell surface ICAM-1 was measured by flow cytometry. Results are presented as fold increase in the median fluorescence intensity of cell surface ICAM-1, normalized within each experiment to the untreated condition for each cell type. (**f**, **g**) After 4 h or 18-24 h, stimulated endothelial cells were detached and TNFα (**f**) and IL-1β (**g**) induced cell surface VCAM-1 was measured by flow cytometry. Results are presented as fold increase in the median fluorescence intensity of cell surface VCAM-1, normalized within each experiment to the untreated condition for each cell type. (TNFα and IL-1β 4 h, n = 4; 18-24 h, n = 5–6 donors per endothelial cell type). #*p* < 0.1, *p < 0.05, **p < 0.001 comparing HAEC and HCAEC to HLSEC by two way ANOVA followed by uncorrected Fisher’s LSD. (**h**) mRNA and (**i**) fold increase in the MFI of cell surface expression of VCAM-1 was measured on aortic HAEC and liver HLSEC endothelial cells stimulated with IL-4 (20 ng/mL) for 4 h or 24 h (n = 3 donors per endothelial cell type). Results are presented as mean ± SEM. #*p* < 0.1 comparing HAEC to HLSEC, by two way ANOVA and uncorrected Fishers LSD.

Because there was no apparent defect in liver endothelium to produce chemokines in response to inflammatory cytokine activation to account for the reduced adherence of PBMC, we next compared induction of adhesion molecules in vitro at the mRNA (4 h, Fig. 4a) and protein (4–24 h, Fig. 4b–g) levels. Normalized mRNA counts for adhesion molecule genes can be found in Figure S5. All EC expressed a low but detectable level of cell surface ICAM-1, ICAM-2 and BST2. Untreated endothelium was negative for VCAM-1, E-selectin and CD164/endolyn.

Neither TNFα nor IL-1β changed expression of BST1, ICAM2 or BST2 at 4 h (Fig. 4a, S4f.–h). Although CD44 and CD164/endolyn were modestly enhanced by TNFα stimulation, there was no notable difference across endothelial cell types at the times assessed (Fig. 4a).

E-selectin expression was transiently increased by TNFα, peaking around 6 h and declining nearly to baseline after 18 h of TNFα stimulation. Across EC types, liver endothelium consistently exhibited the lowest inducible E-selectin expression (HAEC 45.6 ± 22.74-fold; HCAEC 46.5 ± 28.7-fold; HLSEC 15.9 ± 7.81-fold; *p* < 0.0001 comparing HLSEC versus others; n = 5 donors at 4 h) in response to both TNFα and IL-1β (Fig. 4b,c). To gain insight into whether the diminished E-selectin induction in liver endothelial cells was specific to the stimulus, we tested whether there were differences in thoracic versus hepatic endothelial cells to respond to another stimulus of E-selectin. It has also been reported that endothelial cells upregulate E-selectin in response to IL-10. We observed that IL-10 stimulated increased E-selectin expression on aortic EC, but no E-selectin was detected on liver endothelium stimulated with IL-10 (*data not shown*).

ICAM-1 was expressed at low levels on untreated endothelial cells and highly upregulated by either TNFα or IL-1β at 18 h. Unlike E-selectin, there was no difference across endothelial cell types in the kinetics or magnitude of ICAM-1 induction (Fig. 4d,e).

VCAM-1 was not detected on untreated endothelial cells, and its TNFα or IL-1β inducible expression was maximal after 18–24 h of stimulation. Notably, endothelium from the aorta and coronary artery upregulated VCAM-1 earliest (by 4 h), and the magnitude and absolute expression of VCAM-1 was also higher in large vessel cardiac endothelium than other EC types (fold increase at 4 h, HAEC 51.8 ± 13.8-fold, HCAEC 15.70 ± 4.5-fold, HLSEC 10.11 ± 5.6-fold (n = 4); at 18 h, HAEC 65.32 ± 15.7-fold, HCAEC 43.64 ± 12.29-fold, HLSEC 13.68 ± 7.04-fold; *p* < 0.01 HAEC vs. HLSEC, n = 5) (Fig. 4f,g). We also tested whether cardiac and hepatic endothelial cells differentially responded to another cytokine, IL-4, which is known to stimulate endothelial expression of VCAM-1. Like with TNFα, VCAM-1 mRNA and protein was increased in aortic EC early (19.6-fold 4 h) after IL-4 stimulation, but did not substantially rise in HLSEC at this time point (3.46-fold) (mRNA: Fig. 4h, protein: Fig. 4i). Moreover, total expression and fold induction of VCAM-1 in response to IL-4 was significantly higher on aortic and coronary artery EC compared with liver EC (HAEC 5.59 ± 1.34-fold 4 h, 11.19 ± 2.68-fold 24 h; HLSEC 1.1 ± 0.2-fold 4 h, 5.26 ± 1.58-fold 24 h, *p* < 0.1, n = 3).

In summary, liver sinusoidal endothelium exhibited the lowest induction of adhesion molecules E-selectin and VCAM-1, which may contribute to lower adhesion of allogeneic PBMC. Moreover, large vessel cardiac EC upregulated VCAM-1 earlier, and to a greater magnitude, compared with EC from other anatomic origins.

### Complement-induced endothelial cell activation

Transplant recipients may also develop anti-donor antibodies binding to polymorphic HLA molecules. Such HLA antibodies are deleterious to the allograft, in part because they can activate the classical complement cascade. Complement activation at the target cell surface initiates sequential enzymatic cleavage events, generating inflammatory “split products.” The cascade culminates in deposition of terminal complement split products C5b-9, which are proinflammatory and insert into the target cell membrane. Endothelial cells (HUVEC) are known to respond to complement split products through activation of intracellular inflammatory signaling^19–23^. However, liver allografts are notably resistant to this type of injury in the transplant setting, and whether there exists heterogeneity in the sensitivity of different vascular beds to complement activation has not been determined. We therefore questioned whether endothelial responses to complement mediated injury were unequal.

First, endothelial monolayers were treated with a polyclonal mixture of chimeric and fully human monoclonal antibodies against HLA class I, or with sera from highly sensitized transplant patients, to mimic donor specific HLA antibodies seen in transplant patients; and with normal human serum as a source of exogenous complement (HLA Ab + C). We confirmed that binding of anti-HLA IgG triggered complement activation by measuring deposition of terminal complement SC5b-9 on the cell surface after 20 min and 1 h (Figure S6a–d) which was similar across HAEC, HCAEC and HLSEC, and production of C5a in the supernatant (*data not shown*).

### Chemokines and adhesion molecules

We used this stimulation system to model endothelial cell activation by antibody-mediated complement damage. When exposed to HLA antibodies and human complement (HLA Ab + C), endothelial cells enhanced expression of inflammatory genes over time (Figure S6e). As a control, HLA antibodies added with inactivated complement had a lower response compared with antibodies added in active complement (Figure S6e). Similarly, exposure to a control monoclonal chimeric antibody to endoglin (anti-CD105 hIgG1) or isotype control non-binding anti-β-gal hIgG1 had no effect (Figure S6f.).

**Figure 5:**
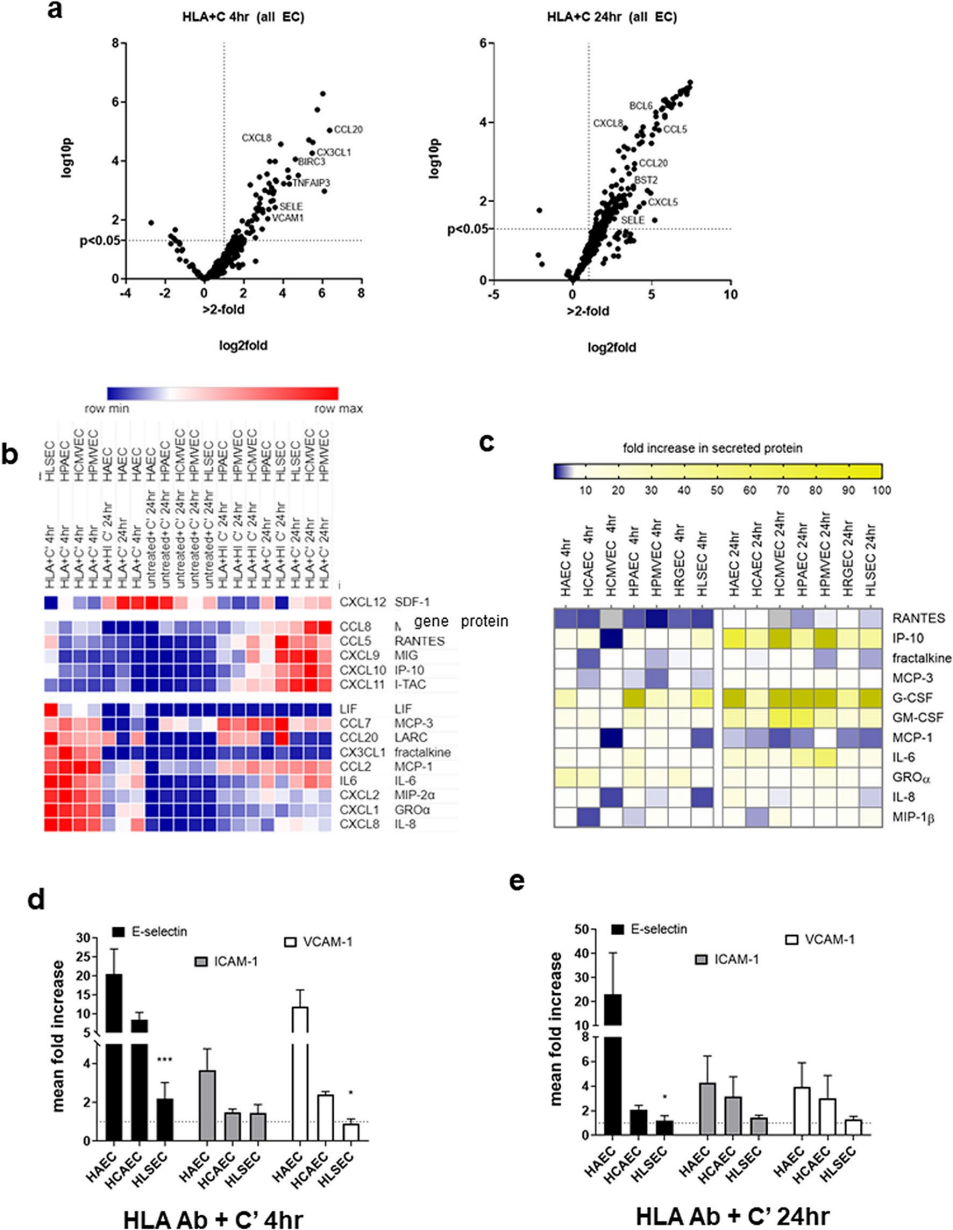
Endothelial cell activation by HLA antibody and complement. Endothelial cell monolayers were treated with a polyclonal mixture of anti-HLA I antibodies in the presence of 25% intact human serum complement for 4 h or 24 h. (**a**) Stimulated endothelial cells were lysed in RLT buffer, and mRNA for immune response genes was measured by nanostring. Volcano plots demonstrate changes in gene expression across the 6 primary endothelial cells stimulated with HLA + C’ for 4 h (left panel) and 24 h (right panel). (**b**) After 4 h and 24 h exposure to HLA + C, mRNA expression of chemokines was measured by Nanostring (n = 1). Results are presented in the heat map, with hierarchical clustering and color scale representing relative mRNA counts of each chemokine across conditions. (**c**) After 4 h or 24 h treatment with DSA + C, conditioned media were tested for secreted factors by Luminex and ELISA. Results are presented in the heat map, with color scale representing fold increase in protein concentrations (pg/mL) of each chemokine compared to untreated. (**d**) Mean fold increase in the MFI of cell surface E-selectin, ICAM-1 and VCAM-1 across HAEC, HCAEC and HLSEC is shown, after stimulation with HLA monoclonal antibodies and intact human complement for 4 h (n = 3 donors per endothelial cell type). Results are presented as mean ± SEM. *p < 0.05, ***p < 0.0001 comparing HLSEC to HAEC, by two way ANOVA and uncorrected Fisher’s LSD. E-selectin: HAEC versus HLSEC *p* < 0.001; HCAEC versus HLSEC *p* = 0.038. VCAM-1: HAEC versus HLSEC *p* = 0.0212; HCAEC versus HLSEC *p* = 0.0175. (**e**) Mean fold increase in the MFI of cell surface E-selectin, ICAM-1 and VCAM-1 across HAEC, HCAEC and HLSEC is shown, after stimulation with HLA monoclonal antibodies and intact human complement for 24 h (n = 3 donors per endothelial cell type). Results are presented as mean ± SEM. *p < 0.05, ***p < 0.0001 comparing HLSEC to HAEC, by two way ANOVA and uncorrected Fisher’s LSD.

EC upregulated 78 and 76 genes at 4 h and 24 h, respectively, of stimulation with monoclonal HLA Ab + C (Fig. 5a). We focused on induction of proinflammatory genes that contribute to adhesion of leukocytes. Comparing primary cardiac and liver endothelium, there were both universal and cell-type specific patterns of chemokine production by EC activated with HLA antibodies and human complement. For example, secreted levels of IL-6, GROα, CXCL8/IL-8, CCL2/MCP-1, and CCL4/MIP-1β were comparable across EC types, but HAEC upregulated chemokines CCL2/MCP-1 and CXCL1/GROα to a greater extent than HLSEC (mRNA: Fig. 5b, protein: Fig. 5c), and adhesion molecules E-selectin, VCAM-1 as well (mRNA: Fig. 5d,e). HLSEC again had the lowest overall induction of E-selectin and VCAM-1 among all EC types tested (Fig. 5d, S7a,b). In order to confirm the relevance of these results, we also tested endothelial cells stimulated with sera from allosensitized transplant patients (Figure S8). Here, too, liver endothelial cells showed a dampened response to complement-induced inflammation. As a control, negative serum without HLA antibodies did not bind to or activate EC (Figure S8).

These results demonstrate that cardiac EC respond to HLA Ab + C by upregulation of adhesion molecules and chemokines, while liver endothelium exhibits a lower response.

### Endothelial heterogeneity of immune-related gene expression

**Figure 6:**
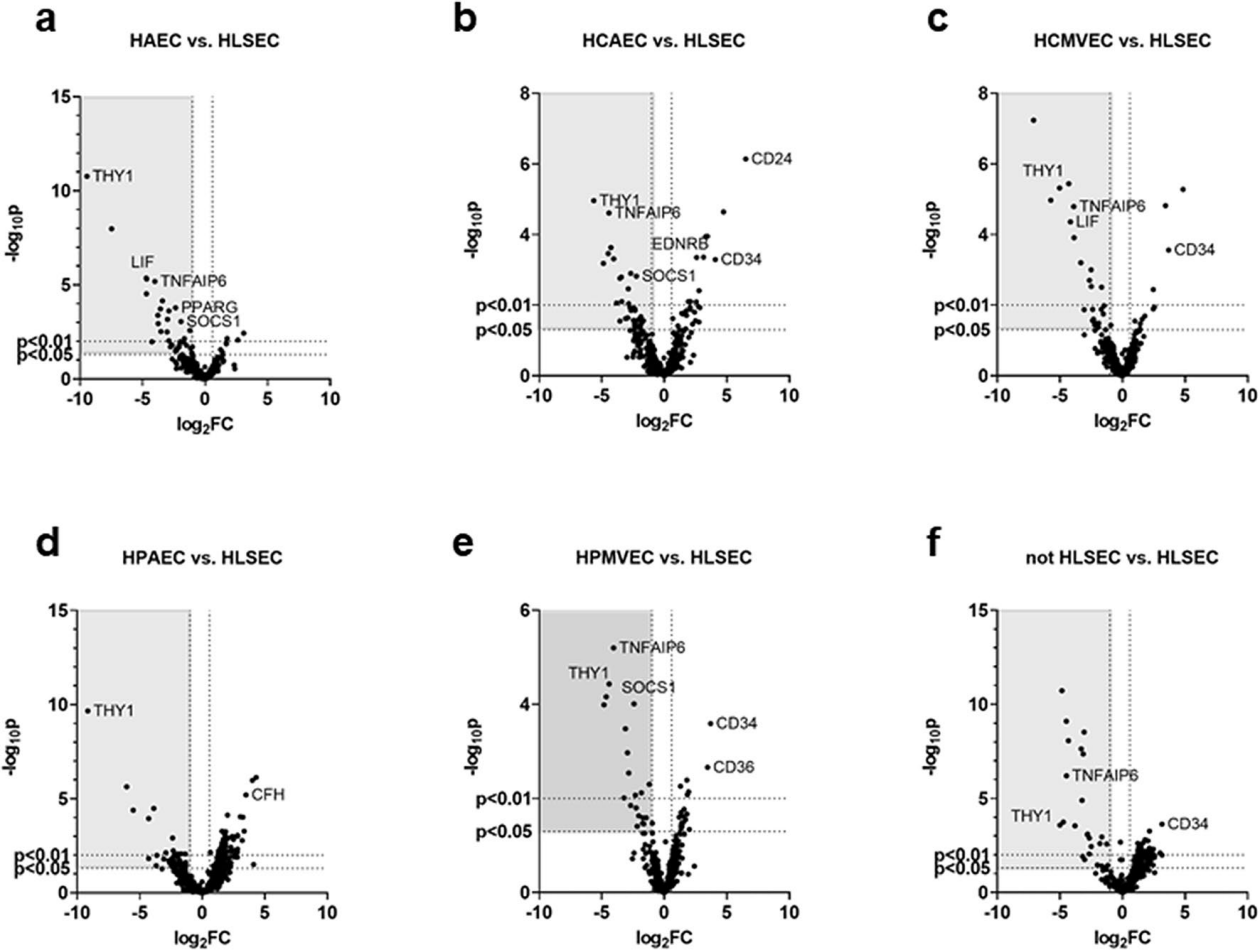
Differential immune gene expression across unstimulated endothelial cells. Volcano plots compare gene expression within each cell type to HLSEC (**a**–**e**) and compare HLSEC to all other non-liver endothelium as a group (**f**). (HAEC, n = 8; HCAEC, n = 4; HCMVEC, n = 7; HPAEC, n = 6; HPMVEC, n = 7; HLSEC, n = 7).

Finally, we sought to determine whether heterogeneity of baseline gene expression across endothelial cell types could contribute to tissue specific chemokine and adhesion molecule induction patterns. Therefore, we analyzed gene expression across untreated primary endothelial cell types (≥ 5 different donors per cell type) to identify possible mediators of differential inflammatory responses. Liver endothelial cells were compared against the five other types independently (Fig. 6a–e) and to the other six endothelial cell types as a group (Fig. 6f, Table S6). Thirty-seven of 579 immune-related genes were differentially expressed at baseline comparing at least one endothelial cell against liver endothelium or HLSEC to all other EC as a group (*p* < 0.05; FC > 1.5; minimum count 250).

We leveraged publicly available datasets of murine and human endothelial gene expression to corroborate our findings^11–13,15,16,24–26^. Eighteen of the 37 candidate DEGs were also confirmed in public datasets, and ten intersected with transcripts found in rejecting human cardiac allograft biopsies [GSE124897,^17^] (Table 2). Two of these DEGs were suppressors of cytokine signaling (SOCS) family proteins, SOCS1 and SOCS3, and two other genes, TNFAIP3/A20 and BCL6, are known repressors of NFκB and JNK signaling.

**Table 2:** List of differentially expressed genes among human endothelial cells from different anatomic origins, and results of confirmatory analysis in public datasets of endothelial cell heterogeneity.

Gene	Comparison vs HLSEC	log_2_fold	*p* value	DEG in turquoise module heart biopsy?	Confirmed DEG in > 2 datasets ?	Public data notes	Data supporting
BCL6	HRGEC	1.85	0.00169		**Yes**	Enriched heart (large vessel), lowest in liver	(11, 17, 18, 19)
C1R	not HLSEC	− 2.58	0.0013		**YES**	Liver	(7, 18, 19)
	HAEC	− 3.47	7e−05				
	HCMVEC	− 4.32	3.7e−06				
	HPAEC	− 3.87	3.3e−05				
	HPMVEC	− 3.05	3.3e−04				
	HRGEC	− 3.47	1.3e−04				
C1S	not HLSEC	− 2.55	0.026				(18, 19)
	HAEC	− 3.84	0.00114				
	HCAEC	− 5.05	0.00663				
	HCMVEC	− 5.64	1.1e−05				
	HPMVEC	− 4.64	0.00010				
CCL20	HAEC	− 3.47	0.00303				
	HCMVEC	− 5.64	0.0133				
	HRGEC	2.52	0.00748				
CD24	HCAEC	6.51	7.2e−07		**Yes**	Kidney	(18, 19)
	HCMVEC	2.45	0.0129				
CD34	not HLSEC	2.47	0.00817		**Yes**	Lowest liver	(12, 18, 19)
	HCAEC	4.07	0.00051				
	HCMVEC	3.68	0.00027				
	HRGEC	3.07	0.0027				
CD36	HAEC	− 2.32	0.0313		**Yes**	Lowest kidney	(9, 12, 18, 19)
	HCAEC	2.76	0.0305				
	HPMVEC	3.45	0.00217				
	HRGEC	− 3.18	0.00696				
CD55	not HLSEC	0.926	0.143		**Yes**	Liver	(9, 18, 19, GSE48209)
	HRGEC	2.68	0.000018				
CDH5	not HLSEC	0.889	0.117	**Yes**			(18)
	HRGEC	2.61	0.0001				
CEBPB	HAEC	− 1.51	0.0164				(17, 18, 19)
	HCMVEC	− 1.51	0.0177				
	HPMVEC	− 1.43	0.0256				
CFH	not HLSEC	2.04	0.00179		**Yes**	Heart	(7, 18, 19)
	HAEC	1.76	0.00713				
	HCAEC	2.10	0.00796				
	HPAEC	3.49	6.3e−06				
	HRGEC	2.05	0.00387				
FCGR2B	HAEC	− 2.94	0.00929	Yes*	**Yes**	LIVER	(9, 18, GSE48209)
	HPAEC	− 2.37	0.00122				
	HRGEC	− 3.05	0.00486				
IFIT2	HRGEC	3.58	0.000394	**Yes**	**Yes**	Kidney	(9, 18, 19)
IFITM1	HAEC	− 3.64	0.000191	**Yes**		Heart IFN signature	(9, 18)
	HCAEC	− 4.32	0.000233				
	HCMVEC	− 3.32	0.000625				
	HPMVEC	− 2.25	0.0158				
IL1A/IL1B	not HLSEC	− 3.14	0.0013	Yes*	**Yes**	Liver	(12, 18, 19, GSE48209)
	HAEC	− 2.0	0.108				
	HCAEC	− 3.47	0.00173				
	HCMVEC	− 2.64	0.002				
	HPAEC	− 5.52	0.000041				
	HPMVEC	− 2.94	0.00108				
	HRGEC	3.64	0.000272				
IL6ST	HRGEC	2.11	3.9e−05		Yes	Liver	(9, 17, 18, 19)
LIF	HAEC	− 4.64	4.9e−06				(12)
	HCAEC	− 4.05	4.9e−04				
	HCMVEC	− 4.05	4.3e−05				
	HPMVEC	− 2.83	2.8e−03				
LITAF	HAEC	− 1.68	0.0097	**Yes**			
	HCAEC	− 2.64	0.00126				
	HCMVEC	− 1.69	0.0133				
	HPMVEC	− 1.83	0.00755				
	HRGEC	− 3.32	2.4e−05				
NFATC1	HAEC	1.23	0.0274	**Yes**			(18, 19)
	HPAEC	1.75	0.0044				
	HRGEC	2.91	1.24e−05				
PPARG	HAEC	− 2.39	0.00016		Yes	Heart/muscle	(9, 18, 19)
SOCS1	HAEC	− 1.94	0.000891	**YES**			
	HCAEC	− 2.25	0.00151				
	HCMVEC	− 1.47	0.0104				
	HPMVEC	− 2.39	9.7e−05				
TAL1	not HLSEC	1.35	0.0263		Yes	Lowest in liver	(18, 19)
	HCAEC	1.78	0.0194				
	HPAEC	2.28	0.00111				
	HPMVEC	1.34	0.0379				
	HRGEC	2.84	8.4e−05				
TCF4	HCAEC	1.49	0.0178		Yes	lowest in liver	(18, 19)
	HRGEC	2.23	0.000171				
TGFBI	HCAEC	− 2.84	0.00349	**Yes**			
	HCMVEC	− 2.47	0.00295				
	HPMVEC	− 1.73	0.0337				
THY1	not HLSEC	− 4.04	0.00821	Yes*			(18)
	HAEC	− 6.64	1.7e−11				
	HCAEC	− 5.64	1.1e−05				
	HCMVEC	− 5.05	4.8e−06				
	HPAEC	− 9.15	2.2e−10				
	HPMVEC	− 4.32	3.7e−05				
	HRGEC	− 4.32	8.5e−05				
TNFAIP3	HRGEC	2.34	0.000871	**Yes**	Yes	Liver	(17, 18, GDS4777, GSE47067)
TNFAIP6	not HLSEC	− 3.94	9.8e−05	No	Yes	Liver	(18, GDS4777)
	HAEC	− 4.05	6.5e−06				
	HCAEC	− 4.32	2.4e−05				
	HCMVEC	− 3.34	1.6e−05				
	HPMVEC	− 4.05	6.3e−06				
	HRGEC	− 3.18	5.3e−04				
TNFSF10 (trail)	HCAEC	2.08	0.0132	**Yes**	Yes	Lowest in liver	(9, 18, 19)
	HPMVEC	1.94	0.00724				
	HRGEC	1.67	0.0245				

*Indicates enrichment may not be due to Endothelial cells, as gene may likely be expressed within leukocyte compartment.

"Yes" in bold indicates gene may be endothelial-associated.

Of the 37 DEGs initially identified in our data, 28 were differentially expressed in two or more EC versus HLSEC, and 17 were confirmed as significantly differentially expressed across EC in at least two other data sets of endothelial cell heterogeneity. Results are presented in Table 2. DEGs included markers of liver sinusoids (FCGR2B), as well as endothelial genes more lowly expressed in the hepatic microvasculature (PECAM1, CDH5). Other DEGs were regulators of cytokine signaling or complement activation, including BCL6 and CFH (highest in cardiac EC), CD55, IL6ST, IL1A, TNFAIP3, and TNFAIP6 (highest in liver), SOCS3 (highest in lung) and TNFSF10 (lowest in liver). CD55, TNFAIP3 and TNFAIP6 were confirmed enriched in liver compared with non-liver EC in multiple public datasets of endothelial transcriptomes [GDS4777, GSE47067^13,26^]. Similarly, BCL6 and TNFSF10 (TRAIL) were confirmed lower in liver EC^25,26^, and were specifically enriched in cardiac endothelium but not hepatic EC^24^. Ten genes were also present in the Turquoise immune response module in cardiac allograft biopsies, distinguishing rejection from stable allografts.

## Discussion

The divergent responses of endothelial cells from different organs and vascular beds remain incompletely characterized, and the underlying mechanisms are poorly understood. We hypothesized that heterogeneity of endogenous negative regulators of inflammatory signaling controls the diversity of endothelial response to inflammatory stimuli. The aim of this study was to characterize the basal and inducible inflammatory phenotypes of EC from heart, lung, kidney, and liver (arterial and microvascular), including expression of adhesion molecules and chemokines, and recruitment of allogeneic peripheral blood mononuclear immune cells. We provide evidence that EC from different anatomic locations exhibited distinct inflammatory phenotypes with respect to the magnitude, kinetics and durability of inducible gene expression, particularly comparing large vessels of the heart to liver microvasculature. We further demonstrate that differential endothelial cell responses occur to transplant-relevant chimeric human anti-HLA antibodies and human complement in an in vitro model of acute antibody-mediated rejection.

Early histology studies showed that endothelial adhesion molecules are increased in human and rodent allografts during rejection^8,27,28^. Our analysis of cardiac allograft biopsies confirmed at the molecular level that endothelial inflammatory genes are increased in rejection. Twenty-five years ago, Steinhoff et al. observed that endothelial adhesion molecule expression in normal and rejecting heart and lung allografts varied not only between organs, but also across vascular beds within each organ^29^. In contrast, sinusoids in liver allografts failed to upregulate E-selectin or VCAM-1^30^. However, these observations were not explained by follow-up mechanistic studies. Our findings suggest that organotypic differences of endothelial cells (EC) may contribute to the relative resistance or susceptibility of transplanted tissue. In particular, liver endothelial cells are intrinsically less likely to promote inflammation. Identification of the protective mechanisms within the liver vascular compartment would reveal new therapeutic targets to reduce rejection in other organs.

EC are key targets of alloimmunity and coordinate access of allogeneic leukocytes to the allograft. It is clear from expanding literature on the heterogeneity of endothelial cell phenotype and function that EC from different vascular beds exhibit important variation in their gene expression, morphology and response to stimuli^11–16,25,31–33^. Our central hypothesis was that EC from different organs, and vascular beds within each organ, exhibit functional diversity that contributes to response to injurious stimuli in transplantation.

Some limited prior work has shown unequal inflammatory responses comparing select EC types^34–36^. For example, differential patterns of adhesion molecule expression by endothelium from different organs have been observed in response to LPS/shock^11,12,37^ and inflammatory cytokines^9,36,38,39^. Although these studies provide evidence of differential gene expression at baseline and some inducible conditions, most compared only a few vascular beds and gave little insight into the mechanisms of differential responses. Our results further resolve that EC from the large vessels in the heart (aorta and coronary artery) exhibit the highest VCAM-1 induction by TNFα and IL-1β, and provide novel evidence that liver EC have the lowest response to not only inflammatory cytokines, but also HLA antibody-triggered complement activation.

Interestingly, our results show differential chemokine production by endothelium from different vascular beds. Chemokines show preferential recruitment of different immune cell subsets depending on leukocyte expression of the chemokine receptor^40^ as well as local tissue specific patterns. Chemokines promote firm adhesion of leukocytes to endothelial cells, augmenting the signals from adhesion molecules^41^. Endothelial MCP-1 and IL-8 are widely and strongly induced by both TNFα and IL-1β, and promote infiltration of innate leukocytes such as monocytes and neutrophils. On the other hand, CCL5 (RANTES) can also recruit CCR5-bearing T lymphocytes. The specific arrays of chemokine elaboration by different vascular beds are therefore intriguing in the broader context of endothelial control of the quality of local immune responses.

In clinical transplantation, cardiac allografts are more subject to hyperacute, acute and chronic rejection caused by donor specific antibodies compared with liver transplants. We reported that complement anaphylatoxins enhance aortic EC activation^22^. Sublytic MAC, C3a or C5a-treated HUVEC also produced chemokines^42^, ICAM-1^43^, VCAM-1 and E-selectin^20^, mediated in part by NFκB/NLRP3. Although differential complement activation between renal and brain EC was observed^44^, different responses to complement had not yet been evaluated for EC resident within transplanted organs. Using high titer monoclonal HLA antibodies, patient sera and exogenous complement, we show that thoracic EC exhibited greater expression of adhesion molecules and unique patterns of chemokine production in response to complement activation, compared with liver endothelium. Future studies are needed to understand the activation threshold of EC to lower titers or fewer clones of HLA antibodies and complement.

Bulk endothelial transcriptomes from human fetal heart, lung, liver, kidney^14^; and from mouse heart, liver, kidney and lung^11,12,16^ have revealed both shared characteristic endothelial signatures and tissue specific gene expression. A major recent breakthrough in understanding of endothelial diversity across organs in vivo, predominantly performed in mice, has arisen from single cell sequencing, for example the Tabula Muris^26^, and followed with even greater resolution of cells throughout the body by Kalucka and colleagues^13^. We capitalized on these data to examine differential expression of immune-related genes among EC from different anatomic origins. Several studies have compared the transcriptional profiles of freshly isolated and cultured EC^10,11,45^. Some of the differential signatures were lost after extended culture, indicating a contribution of microenvironment in site-specific identity or convergence due to culture conditions. Yet, a subset of markers were retained even after culture, which may represent the intrinsically specified differences^10,11^. Bulk endothelial transcriptomes from human fetal organs^14^ and mouse organs^11,12,16^, and single cell sequencing in mice^13,26^ have revealed characteristic endothelial signatures and tissue specific gene expression. We leveraged these multiple public transcriptome datasets to confirm differentially expressed immune-related genes among both cultured and freshly isolated EC.

Certainly multiple cell sources could contribute to gene expression in biopsies, and cell type deconvolution of the transcript signatures of rejection will be an important next step to drill down on compartmentalized responses during alloimmune injury. Additional work is also ongoing to elucidate the role of the candidates identified by our DEG analysis in the regulation of endothelial cell inflammation.

Our results add to the existing literature that EC from the large vessels in the heart (aorta and coronary artery) exhibit the highest VCAM-1 induction by TNFα and IL-1β, and provide novel evidence that liver EC have the lowest response to not only inflammatory cytokines, but also HLA antibody-triggered complement activation. Large vessel arteries experience higher shear stress, which may account in part for the biological need of higher VCAM-1 expression to support adhesion in the aorta and arteries compared with lower shear flow microvascular endothelium. Although differential complement activation between renal and brain EC was reported^44^, this question had not yet been evaluated for EC resident within transplanted organs. Our experiments expand beyond these previous studies to describe the long-term differential responses of tissue resident EC depending on anatomic origin, with carefully controlled conditions with monoclonal HLA antibodies, patient sera and exogenous complement .

Among the candidate genes we identified, complement regulatory factors like CFH, which encodes a soluble inhibitor of complement C3b generation, and CD55, also known as decay accelerating factor, were significantly differentially expressed by liver endothelium compared to thoracic EC. Other differentially expressed genes included transcriptional modifiers, repressors and negative feedback regulators of inflammatory and cytokine signaling, including BIRC3, BCL6, SOCS1, SOCS3, TNFAIP3 and TNFAIP6. Interestingly, many of the differentially expressed genes were also inducible by inflammatory cytokines and/or DSA + C, and were modified by NFκB inhibition. It is tempting to speculate that some differentially expressed genes function in negative feedback regulation of pro-inflammatory signaling in endothelium.

## Materials and methods

### Ethics statement

All experimental protocols in this study were approved by the UCLA Institutional Review Board (#17–000,477, #12–001,597, #10–001,689). IRB waived the requirement for informed consent under 45 CFR 46.116(d) for the study using discarded samples; informed consent was obtained from volunteers. All methods were carried out in accordance with institutional, state and federal guidelines and regulations.

### Cells

The immortalized human dermal microvascular cell line HMEC-1 was obtained from ATCC. Primary human aortic endothelial cells (HAEC, Ao) were isolated from the aortic rings of deceased donors as described^46^ (kindly provided by Dr. EF Reed, UCLA). Primary HAEC, human coronary artery (HCAEC, CA), cardiac microvascular (HCMVEC, CM), pulmonary artery (HPAEC, PA), pulmonary microvascular (HPMVEC, PM), hepatic sinusoidal (HLSEC, L), and renal glomerular (HRGEC, RG) endothelial cells were obtained from commercial sources (see Supplemental Table 1). Commercial endothelial cells were initially expanded from passage 2–3 in respective vendor-recommended media, then switched to the same complete medium for subculturing. Expression of endothelial markers CD31/PECAM-1, CD105/endoglin and CD146/MCAM was verified by flow cytometry. All cells were cultured in tissue culture-treated vessels coated with 0.2% porcine gelatin (Sigma-Aldrich) in 37 °C, and 5% CO_2_. EC were subcultured at 90% confluence using trypsin/EDTA. Cells were used for experiments between passages 3–8.

For experiments, cells were cultured to confluence in tissue culture-treated, gelatin-coated plates and allowed to rest overnight in complete media. Multiple endothelial cells types were tested in parallel in the same experiment. All endothelial cells were switched to M199 + 10% heat inactivated FBS (HI-FBS) (Hyclone) for the duration of experiments. For 96 well plates, 100μL of medium was added; for 48-well plates, 250μL of medium was added; and for 24-well plates, 500μL medium was added. The next day, cells were stimulated in M199 + 10% HI-FBS alone or with TNFα or IL-1β at the concentrations and times indicated.

Primary peripheral blood mononuclear cells were prepared from the blood of healthy volunteers using Ficoll-Paque density centrifugation. Briefly, whole blood was collected into yellow top ACD tubes or a CPDA-1 blood collection bag and diluted 1:1 in without Ca^2+^ or Mg^2+^. 25–30 mL of diluted whole blood was overlaid onto 15 mL of Ficoll-Paque Premium (1.078 g/mL, GE Healthcare) and centrifuged at 1800RPM for 20 min with no brake. The white blood cell interface was collected and washed twice with PBS, centrifuging at 300x*g* for 7 min to remove residual platelets. PBMC were either resuspended in RPMI + 10% HI-FBS and used in experiments on the same day, or frozen in 90% FBS with 10% DMSO. Frozen PBMC were thawed, washed once with RPMI + 10% HI-FBS and allowed to recover in a 37 °C water bath before use in experiments.

### Reagents

TNFα and IL-1β were obtained from Sigma Aldrich (#H8916, #I9401). Recombinant human carrier-free IL-6, IL-10 and IL-4 were purchased from R&D (#285-IF, #206-IL-010/CF, #204-IL-010/CF, 217-IL-005/CF). Based on the results of preliminary dose–response experiments, the following concentrations of each stimulus were used: TNFα 20 ng/mL, IL-1β 20 ng/mL, IL-4 (20 ng/mL), IL-10 (20 ng/mL). Cycloheximide (CHX) was obtained from Sigma-Aldrich.

Control antibodies were anti-β-galactosidase hIgG1 (Invivogen #bgal-mab1) and anti-CD105 hIgG1 (MediMabs #MM-0300). Chimeric HLA I human IgG1 (derived from murine clone W6/32) was obtained from Invivogen, and HLA I hIgG1, hIgG3 were kindly provided by One Lambda/ThermoFisher. Fully human anti-HLA-A2/A28 (clone SN607D8), anti-HLA-A2/B17 (clone SN230G6), anti-HLA-A1/A3/11 (clones MUL4C8 and MUL2C6), and anti-Bw4 (clone MUS4H4) IgG1 were kindly provided by Drs. Mulder and Heidt (Leiden University Medical Center).

Broadly reactive sera from transplant candidates with a cPRA 99–100% and strong > 10,000 MFI antibodies to common HLA-A and HLA-B antigens were heat-inactivated at 56 °C for 1 h and frozen in aliquots. Discarded patient serum without HLA antibodies by single antigen bead based detection was used as a negative control.

Human complement was obtained from Complement Technologies (cat#NHS). Human serum complement that was heat-inactivated at 56 °C for ≥ 30 min, or C1q-depleted or C3-depleted serum was used as a control.

### Flow cytometry immunophenotyping adherent PBMC

Endothelial cells were seeded at confluence in a tissue culture-treated, gelatin-coated 24 well plate and allowed to rest overnight in complete medium. Peripheral blood mononuclear cells prepared from whole blood as above were added to stimulated endothelial cells at a ratio of 3:1 (based on initial experiments testing 1:1, 3:1 and 5:1 and at concentration consistent with that in whole blood 10^6^/mL). After 45 min, nonadherent cells were removed by gentle washing with HBSS with Ca^2+^ or Mg^2+^ followed by one wash with PBS without Ca^2+^ or Mg^2+^. Remaining adherent cells were detached with Accutase, then stained with Panel 4 (Table S4) in FACS buffer (PBS + 2% HI-FBS) for 45 min at 4 °C, washed and analyzed by flow cytometry (BD Fortessa). Compensation was performed using compensation beads (BD Biosciences). The gating strategy is shown in Figure S2a–e. Gating controls showing reproducibility and relative frequencies of endothelial cells and PBMC subsets in the input fractions are provided in Figure S2f.–h. After gating out debris by FSC/SSC, leukocytes were distinguished from endothelium by gating CD11a (negative on endothelial cells) and CD31 or CD105 (bright on endothelial cells). Non-endothelial cells were then subset gated based on CD3 (T cells), CD56 (NK cells), CD19 (B cells), CD14 (monocytes), and HLA-DR (B cells and monocytes). We observed that monocyte-endothelial cell doublets were formed when endothelium was activated with TNFα, but which were not present in the input fractions or in cocultures with unstimulated endothelial cells, forming double positive CD11a + CD105 + events that were CD14^high^.

### HLA antibody and complement stimulation

Fully human and chimeric human HLA monoclonal antibodies^47^ and patient sera were tested for binding against EC by flow cytometry (Figure S6). Based on these experiments, human and chimeric anti-HLA IgG were diluted as a mixture in M199 to final concentration of 0.5 μg/mL per antibody (HLA I IgG1 + HLA-A2/A28 IgG1 + HLA-A3/A11 IgG1 + HLA-A2/B17 IgG1). Nonsensitized and alloantibody-containing sera were diluted into M199. Human serum complement was stored at − 80 °C, and on the day of experiments rapidly thawed at 37 °C until only a sliver of ice remained, then transferred immediately to ice. Freshly thawed human serum complement (intact or inactivated) was added to HLA antibody diluted in M199 to a final concentration of 25% of complement within 20 min of thawing (based on preliminary experiments). Then, HLA antibodies which bind to cells and human serum complement together were added to endothelial monolayers and experimental incubations were carried out at 37 °C. For 48-well plates, a total volume of 250μL was added; for 24-well plates, 500μL was added.

### Targeted gene expression analysis

RNA was diluted to 20 ng/μL in RNase and DNase-free water. To determine inducible changes in gene expression in cultured cells, confluent endothelial cells were stimulated for 4 h in M199 + 10% FBS with each stimulus, or left untreated in M199 + 10% FBS. Lysates of cultured endothelial cells were prepared by pelleting at 300x*g*, aspirating all supernatant, resuspending in RLT buffer (Qiagen) in nuclease-free tubes and homogenized by vortexing for 1 min. A ratio of 1μL of RLT buffer per approximately 6,500 cells was used.

mRNA expression of immunology-related genes was assessed using nCounter (NanoString, Human Immunology Panel, 579 genes), performed by the UCLA Center for Systems Biomedicine. Data were analyzed using NSolver software (NanoString Technologies, Seattle, WA). Differential gene expression data comparing each EC type to HLSEC are available in Table S6.

### Chemokine detection

Supernatants from stimulated endothelial cells were collected at the end of each experiment. 38 cytokines and chemokines were measured in conditioned media using the multiplexed Milliplex Human Cytokine/Chemokine panel (Millipore) and performed by the UCLA Immune Assessment Core. Secretion of IP-10 (CXCL10, R&D DY266-05), I-TAC (CXCL11, R&D Systems #DY672), CCL5 (RANTES, R&D Systems #DRN00B), CCL20 (LARC, R&D Systems #DY360-05) and CXCL8 (IL-8, R&D Systems #D8000C) into conditioned media were measured by ELISA according to the manufacturer’s recommendations. Generation of C5a in cell culture supernatants was measured by ELISA (R&D Systems #DY2037) according to the vendor’s protocol.

### Flow cytometry

Endothelial cells were seeded at confluence and allowed to rest overnight in complete medium. Stimulated endothelial cells were washed once with PBS without Ca^2+^ or Mg^2+^, detached with room temperature Accutase (Innovative Cell Technologies), and resuspended in FACS buffer (cold PBS + 2% FBS + 1% NaN_3_). Cell surface expression of adhesion molecules was determined using Panel 1 or Panel 2 described in Table S4. For complement detection, confluent EC exposed to antibodies and human complement at 37 °C were detached with Accutase and stained with Panel 3 (Table S4) first with rabbit anti-human SC5b-9, followed by anti-rabbit-PE and mouse anti-human IgG-BV510 secondary antibodies. Cells were then acquired by flow cytometry (BD Fortessa). SC5b-9 is an integral component of the terminal complement membrane attack complex; deposition of this protein complex on the surface of cells represents evidence for advanced complement activation at the level of the target cell.

### Publicly available datasets and data availability

Cardiac allograft biopsy microarray data [GSE124897]^17^ was downloaded from the NCBI GEO website (https://www.ncbi.nlm.nih.gov/gds). Other data were also downloaded from GEO, from investigator-developed databases or using the Bioinformatics Array Research Tool for microarray data (http://igc1.salk.edu:3838/bart/)^48^ and analyzed using GEO2R. Other datasets referenced in this study were also downloaded from GEO (http://igc1.salk.edu:3838/bart/)^48^, or from investigator-developed databases (Table S5, S7 and S8).

Cardiac allograft biopsy microarray data [GSE124897] included 889 subjects and 49,395 probes. The test and training datasets had the same distribution of rejection versus normal and the type of rejection (Table 1).

Bulk transcriptome data from 1) freshly isolated endothelium from mouse heart, liver and brain [GSE48209,^49^]; 2) ribo-tagged mouse EC from heart and lung [GSE136848,^12^]; 3) mouse translatome EC from heart, kidney, liver and lung [GSE138629,^11^] were downloaded using BART and analyzed. Genes enriched in endothelial cells from mouse kidney, liver, and lung were queried from (https://markfsabbagh.shinyapps.io/vectrdb/)^25^. Single cell RNA sequencing from whole mouse organs: Gene expression data of heart endothelial cells, endocardial cells, kidney capillary endothelial cells, lung endothelial cells and endothelial cell of hepatic sinusoids were downloaded from [https://tabula-muris.ds.czbiohub.org/^26^]. Genes that were specific markers of heart, lung, kidney and liver endothelial cells compared with all other organs were queried; and genes enriched within vascular subcompartments within each organ, using the Marker Set tool at (https://endotheliomics.shinyapps.io/ec_atlas/)^13^.

Genes that were found in human aortic endothelial cells but not in liver endothelial cells were queried by selecting for HAoEC—Heart—Normal and Specific; and HHSEC—Liver—Normal and Specific. This yielded 1946 genes specific in HAEC but not HLSEC; and 1451 genes specific in HLSEC but not HAEC, with no gene overlap. The lists can be found in Table S7 and Table S8, respectively. http://angiogenes.uni-frankfurt.de/^24^. Other studies of human endothelial cells included: Microarray study of cultured (mix of commercial and fresh isolate) human aortic, coronary artery, iliac arterial, pulmonary artery, iliac vein, and pulmonary vein ECs, human hepatic artery, hepatic vein ECs GSE43475 (GDS4777)^45^; and GSE114607 human fetal organs^14^. Differentially expressed genes comparing heart (coronary + aortic) versus not heart (pulmonary and hepatic artery) and liver (hepatic artery) versus not liver (coronary, aortic and pulmonary artery) were queried for the top 250 DEGs using Geo2R.

### Analyses

Heat maps and hierarchical clustering were generated using Morpheus (https://software.broadinstitute.org/morpheus). Graphs were generated in Prism (GraphPad Software, San Diego, CA). Differences between groups were determined by one way ANOVA followed by *t* test (GraphPad).

Cardiac allograft biopsy microarray data [GSE124897] were back transformed. Probes with standard deviation ≥ 0.5 or within the top 75 percentile mean were selected for further analyses, and the data was divided into training (75%) and test (25%) sets based on the distribution of rejection/normal and rejection type (Table 1). Next, a series of Wilcoxon testing was performed to find genes that are differentially expressed among rejection (y/n), next the *p* values were adjusted for false discovery rate (fdr), and the top 5000 probes with lowest q-value were selected for weighted gene co-expression network analyses (WGCNA) analyses. Backtransformed data were also analyzed for differences in individual gene expression by one way ANOVA followed by uncorrected Fisher’s LSD *t* test (GraphPad).

## Supplementary Information

Supplementary Information.

## Abbreviations

AMR: Antibody-mediated rejection
DEG: Differentially expressed gene(s)
DSA: Donor specific HLA antibody
EC: Endothelial cell
HAEC: Human aortic endothelial cell
HCAEC: Human coronary artery endothelial cell
HCMVEC: Human cardiac microvascular endothelial cell
HLSEC: Human liver sinusoidal endothelial cell
HLA: Human leukocyte antigen
HLA Ab + C’: HLA antibodies and complement
HPAEC: Human pulmonary artery endothelial cell
HPMVEC: Human pulmonary microvascular endothelial cell
HRGEC: Human renal glomerular endothelial cell
HUVEC: Human umbilical vein endothelial cells
PBMC: Peripheral blood mononuclear cell
TCMR: T cell-mediated rejection
