# Supplementary material for: Tissue-specific endothelial cell heterogeneity contributes to unequal inflammatory responses
Source: Sci Rep. 2021 Jan 21;11:1949. doi: 10.1038/s41598-020-80102-w (PMC7820348; doi:10.1038/s41598-020-80102-w)
Supplement: Supplementary file 1 — Supplementary Information. [file 41598_2020_80102_MOESM1_ESM.pdf]

Supplementary File

Tissue-specific endothelial cell heterogeneity contributes to unequal inflammatory responses

Hasitha Gunawardana, Tahmineh Romero, Ning Yao, Sebastiaan Heidt, Arend Mulder, David A Elashoff, Nicole M Valenzuela

Supplemental Figure Legends

Figure S1. Weighted Correlation Network Analysis of transcript changes within cardiac allograft biopsies with rejection vs. stable. Heat map of genes assigned to the Turquoise Module by WCGNA, comparing rejection to non-rejection/normal [GSE124897].

Figure S2. Gating strategy for assessing differential adherence of PBMC subsets. Endothelial cells alone, PBMC alone, or endothelial cells mixed with PBMC were stained and acquired by flow cytometry.

a) Debris was gated out by forward scatter and side scatter to gate the live fraction.

b) Endothelial cells were gated as $CD105^{bright}$, $CD11a^{negative}$. Doublets of EC and leukocytes were $CD105^{bright}$, $CD11a^{bright}$. Subsequent gating showed that the doublets were also $CD14^{high}$.

c) Non-endothelial cells ($CD105^{neg}$) were first gated as follows: $CD105^{-}CD3^{+}$ T cells, $CD105^{-}CD19^{+}$ B cells, and not T or B cells $CD105^{-}CD3^{-}CD19^{-}$.

d) Monocytes were gated from the not T or B fraction as $CD105^{-}CD3^{-}CD19^{-}CD56^{-}CD14^{+}$ monocytes.

e) NK cells were gated as $CD105^{-}CD3^{-}CD19^{-}CD14^{-}CD56^{+}$ NK cells.

f) As a control, endothelial cells alone were stained with the immunophenotyping panel. Gating results for the input fraction of EC alone are shown (n=3), demonstrating >90% of cells fell within the endothelial gate.

g) For each PBMC donor, as a control, PBMC alone were stained with the immunophenotyping panel. Gating results for the input fraction of PBMC alone are shown (n=6), with less than 5% of events falling into the endothelial cell gate. PBMC subsets T cells, B cells, NK cells and monocytes were represented with expected proportions for healthy controls.

h) For each experiment, as a control, total input of PBMC and endothelial cells was stained with the immunophenotyping panel. Gating results for the control input fraction are shown (n=6).

Figure S3. Differential adherence of allogeneic PBMC to cytokine-activated endothelial cells. Endothelial monolayers were stimulated with TNFα (20ng/mL) or IL-1β (20ng/mL) for 4hr or 18hr. Stimulation medium was removed, and whole PBMC fractions were added at a ratio of 3 PBMC to 1 endothelial cell and allowed to adhere for 45 minutes. Nonadherent cells were removed by two washes with HBSS with Ca^{2+} and Mg^{2+}, and adherent cells were detached by a third wash

with PBS without Ca^{2+} and Mg^{2+} followed by treatment Accutase. Adherent cells were stained with an immunophenotyping panel and acquired by flow cytometry.

a) Each subset as a percent of live cells is shown in the bar graphs. Summary results of the adherent fraction to untreated EC (open circles) and to TNFα 4hr-treated EC (black circles) are shown. ** $p<0.01$, **** $p<0.0001$ comparing untreated to TNFα.

b) The ratio of T cells to endothelial cells within each experiment is shown. Untreated conditions are graphed in the white bars, TNFα 4hr activated endothelial cells in the black bars, and IL-1β in the gray bars. Results are presented as mean ± SEM.

c) The ratio of B cells to endothelial cells within each experiment is shown. Untreated conditions are graphed in the white bars, TNFα 4hr activated endothelial cells in the black bars, and IL-1β in the gray bars. Results are presented as mean ± SEM.

d) The ratio of NK cells to endothelial cells within each experiment is shown. Untreated conditions are graphed in the white bars, TNFα 4hr activated endothelial cells in the black bars, and IL-1β in the gray bars. Results are presented as mean ± SEM.

e) The ratio of monocytes to endothelial cells within each experiment is shown. Untreated conditions are graphed in the white bars, TNFα 4hr activated endothelial cells in the black bars, and IL-1β in the gray bars. Results are presented as mean ± SEM.

f) The ratio of T cells to endothelial cells within each experiment is shown. Untreated conditions are graphed in the white bars, TNFα 18hr activated endothelial cells in the black bars, and IL-1β in the gray bars. Results are presented as mean ± SEM.

g) The ratio of B cells to endothelial cells within each experiment is shown. Untreated conditions are graphed in the white bars, TNFα 18hr activated endothelial cells in the black bars, and IL-1β in the gray bars. Results are presented as mean ± SEM.

h) The ratio of NK cells to endothelial cells within each experiment is shown. Untreated conditions are graphed in the white bars, TNFα 18hr activated endothelial cells in the black bars, and IL-1β in the gray bars. Results are presented as mean ± SEM.

i) The ratio of monocytes to endothelial cells within each experiment is shown. Untreated conditions are graphed in the white bars, TNFα 18hr activated endothelial cells in the black bars, and IL-1β in the gray bars. Results are presented as mean ± SEM.

Figure S4. Expression of chemokines. After activation with TNFα (20ng/mL) or IL-1β (20ng/mL), stimulated endothelial cells were lysed in RLT buffer, and mRNA for immune response genes was measured by Nanostring.

a-o) Normalized mRNA counts for each chemokine gene are shown in the bar graphs, for primary endothelial cells left untreated (green bars) or stimulated for 4hr with TNFα (red bars) or IL-1β (blue bars). One representative experiment is shown.

Figure S5. Adhesion molecule expression after TNFα stimulation.

a-f) Normalized mRNA counts for each adhesion molecule gene are shown in the bar graphs, for primary endothelial cells left untreated (green bars) or stimulated for 4hr with TNFα (red bars) or IL-1β (blue bars).

Figure S6. HLA antibody binding and induced complement deposition on endothelial cells.

a) HAEC, HCMVEC, HPAEC, HLMVEC, and HLSEC were treated with a monoclonal HLA antibody mixture (HLA-A2/A28+A2/B17+A3/A11+HLA I hIgG1, each at 0.5ug/mL) in the presence of intact (light pink and dark pink bars) or inactive (blue bars) human serum complement (25% or 50%) for 4hr or 18hr. Cells were detached with Accutase, then stained for bound human IgG and measured by flow cytometry.

b) The fold increase in hIgG bound to HAEC, HCAEC and HLSEC is shown (mean +/- SEM, n=3). Control anti-βgal hIgG1 did not bind to cells. There was no significant difference in HLA antibody bound to each cell type, when exposed to the mixture of HLA class I monoclonal antibodies.

c, d) HAEC were treated with monoclonal HLA antibodies (HLA-A2/A28+A2/B17+A3/A11 hIgG1, each at 0.5ug/mL) in the presence of intact (pink bars) or inactive (blue bars) human serum complement (25% or 50%) for 20min. Deposition of hIgG (c) and SC5b-9 (MAC) (d) were detected with rabbit anti-SC5b-9 followed by anti-hIgG-BV510 and anti-rb-PE.

e) Gene expression in endothelial cells following HLA antibody-induced complement activation. Scatter plot shows gene expression changes in HAEC stimulated with monoclonal HLA antibody mixture and complement (HLA+C') for 4hr or 24hr, or HLA antibodies and inactivated complement (HLA+iC') for 24hr. Log_2 mRNA counts are normalized against untreated (black line).

f) Representative experiment showing no induction of adhesion molecules on HAEC compared with non-HLA mAbs at 6hr and 24hr. A chimeric hIgG1 antibody that binds to CD105 on endothelial cells does not trigger adhesion molecule expression in the presence of intact

human complement. Primary endothelial cells were stimulated with a single monoclonal anti-CD105 hIgG (1μg/mL) for 4hr or 18hr, and cell surface ICAM-1, E-selectin and VCAM-1 expression was measured by flow cytometry (n=6 independent experiments). Results are presented as the mean fold increase in MFI +/- SEM.

Figure S7.

a) Mean fold increase in the MFI of cell surface E-selectin, ICAM-1 and VCAM-1 across HAEC, HCAEC, HCMVEC, HPAEC, HPMVEC and HLSEC is shown, after stimulation with HLA monoclonal antibody mixture and intact human complement for 4hr (n=3 donors per endothelial cell type). HLSEC vs. HAEC $p<0.002$ for E-selectin; HLSEC vs. HCMVEC $p<0.1$ for E-selectin; HLSEC vs. HAEC $p=0.05$ for VCAM-1; HLSEC vs. HCMVEC $p<0.05$ for VCAM-1 by two way ANOVA followed by uncorrected Fisher's LSD. Results are plotted as mean fold increase in each adhesion molecule ± SEM.
b) Mean fold increase in the MFI of cell surface E-selectin, ICAM-1 and VCAM-1 across HAEC, HCAEC, HCMVEC, HPAEC, HPMVEC and HLSEC is shown, after stimulation with HLA monoclonal antibody mixture and intact human complement for 24hr (n=3 donors per endothelial cell type). HLSEC vs. HAEC $p=0.012$ for E-selectin; HLSEC vs. HCMVEC $p<0.1$ for VCAM-1. Results are plotted as mean fold increase in each adhesion molecule ± SEM.

Figure S8. Differential activation of HAEC, HCAEC and HLSEC by HLA allosera and complement.

HAEC (a-c), HCAEC (d-f) and HLSEC (g-i) were left untreated, stimulated with negative serum from non-sensitized patients, or stimulated with HLA sera from highly sensitized transplant candidates, in the presence of 25% human complement. Cell surface expression of E-selectin (a, d, g), ICAM-1 (b, e, h), and VCAM-1 (c, f, i) was measured by flow cytometry. One representative experiment is shown (n=3 total).

Supplemental Figures

a

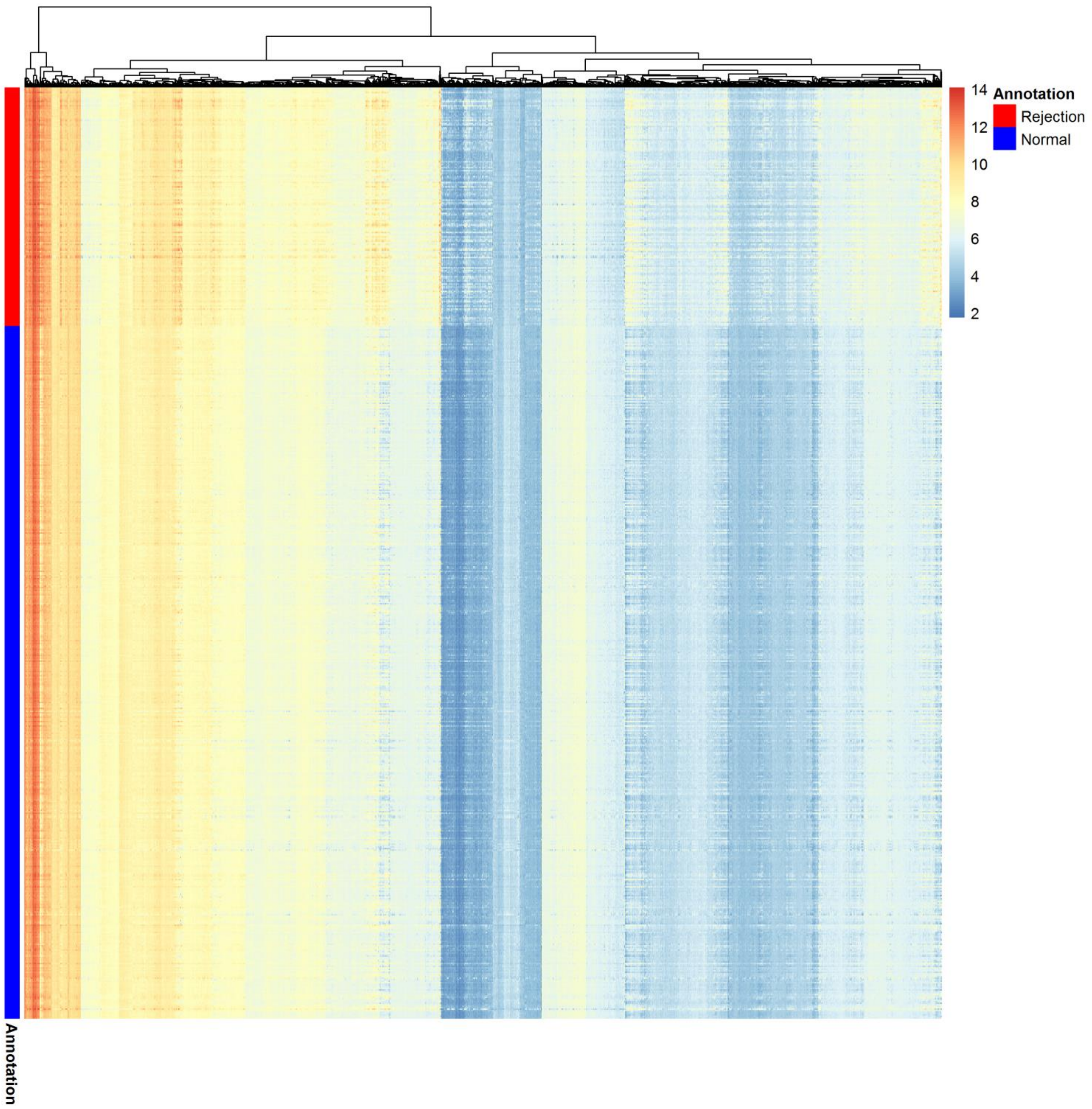

Figure S1.

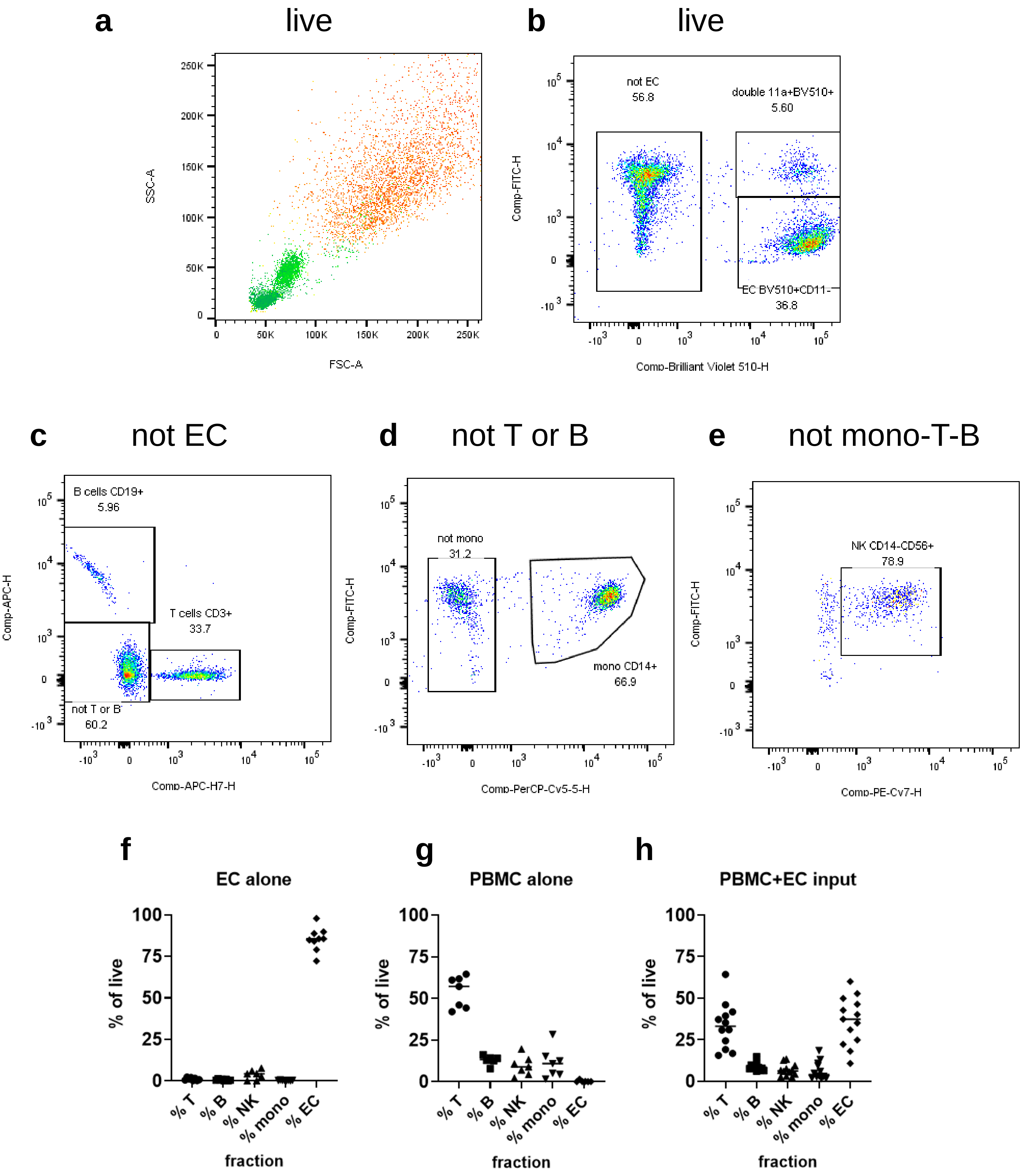
a
live
SSC-A
FSC-A
b
live
not EC
56.8
double 11a+BV510+
5.60
EC BV510+CD11-
36.8
Comp-FITC-H
Comp-Brilliant Violet 510-H
c
not EC
B cells CD19+
5.96
T cells CD3+
33.7
not T or B
60.2
Comp-APC-H
Comp-APC-H7-H
d
not T or B
not mono
31.2
mono CD14+
66.9
Comp-FITC-H
Comp-PerCP-Cy5-5-H
e
not mono-T-B
NK CD14-CD56+
78.9
Comp-FITC-H
Comp-PE-Cy7-H
f
EC alone
g
PBMC alone
h
PBMC+EC input
% of live
% T
% B
% NK
% mono
% EC
fraction

Figure S2.

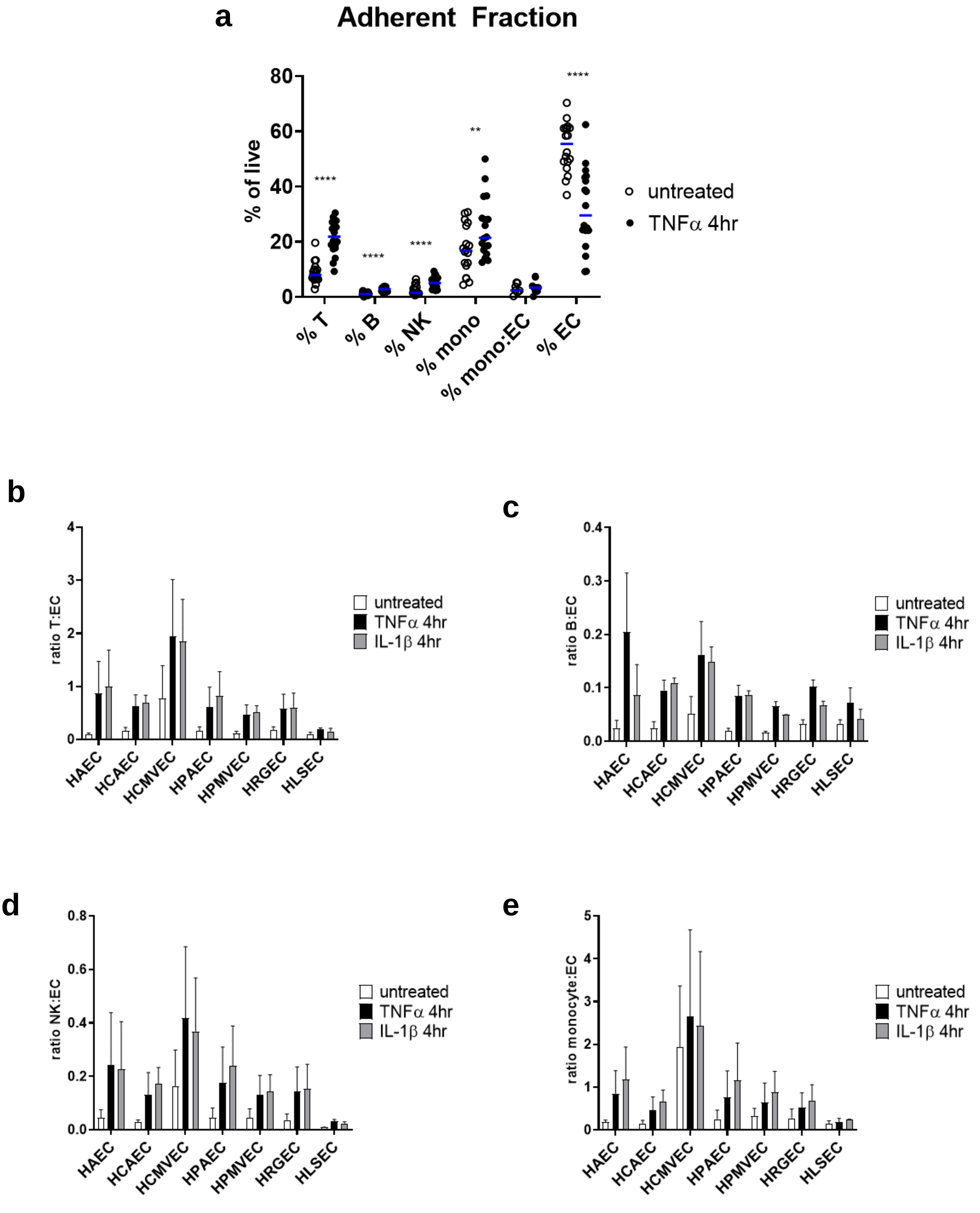
a
Adherent Fraction
% of live
****
****
****
**
****
% T
% B
% NK
% mono
% mono:EC
% EC
untreated
TNFα 4hr
b
ratio T:EC
c
ratio B:EC
d
ratio NK:EC
e
ratio monocyte:EC
untreated
TNFα 4hr
IL-1β 4hr
HAEC
HCAEC
HCMVEC
HPAEC
HPMVEC
HRGEC
HLSEC

Figure S3.

f

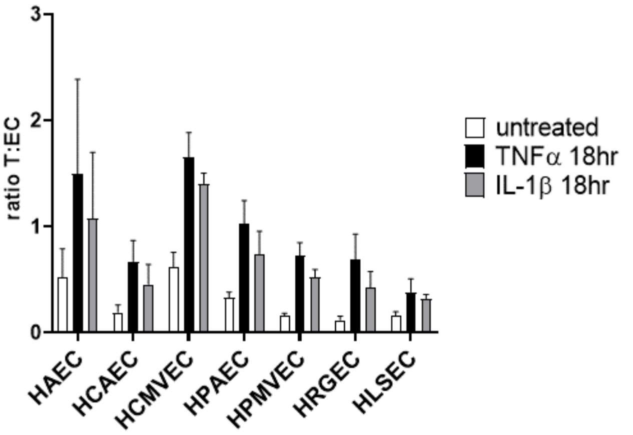

g

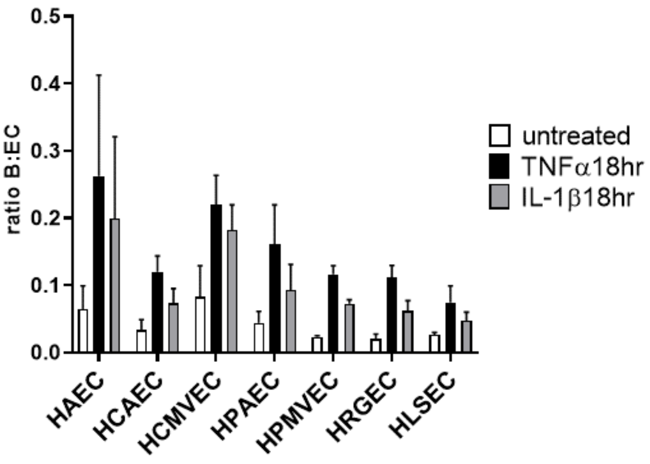

h

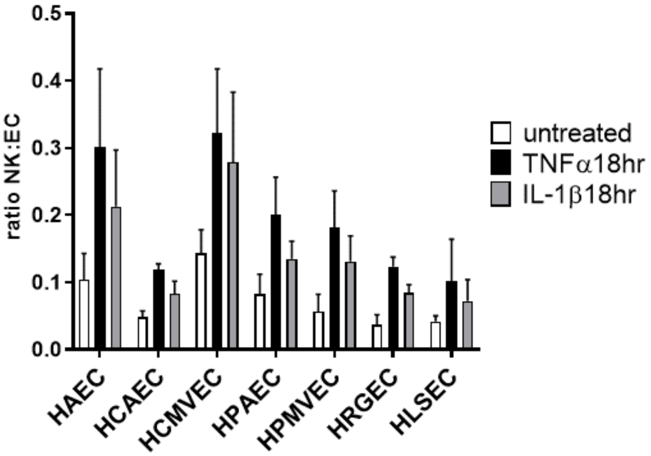

i

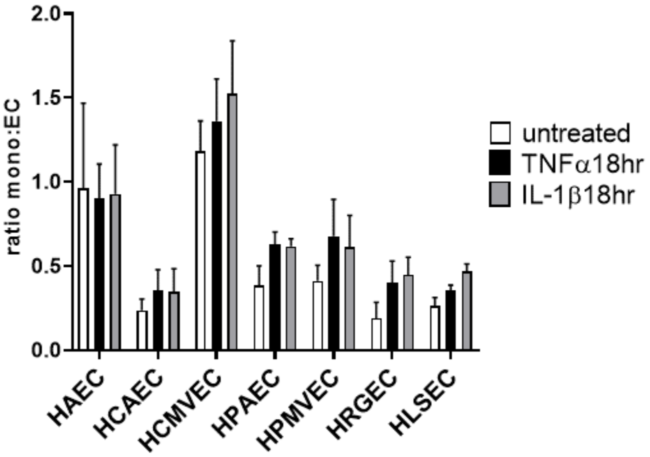

Figure S3. (continued)

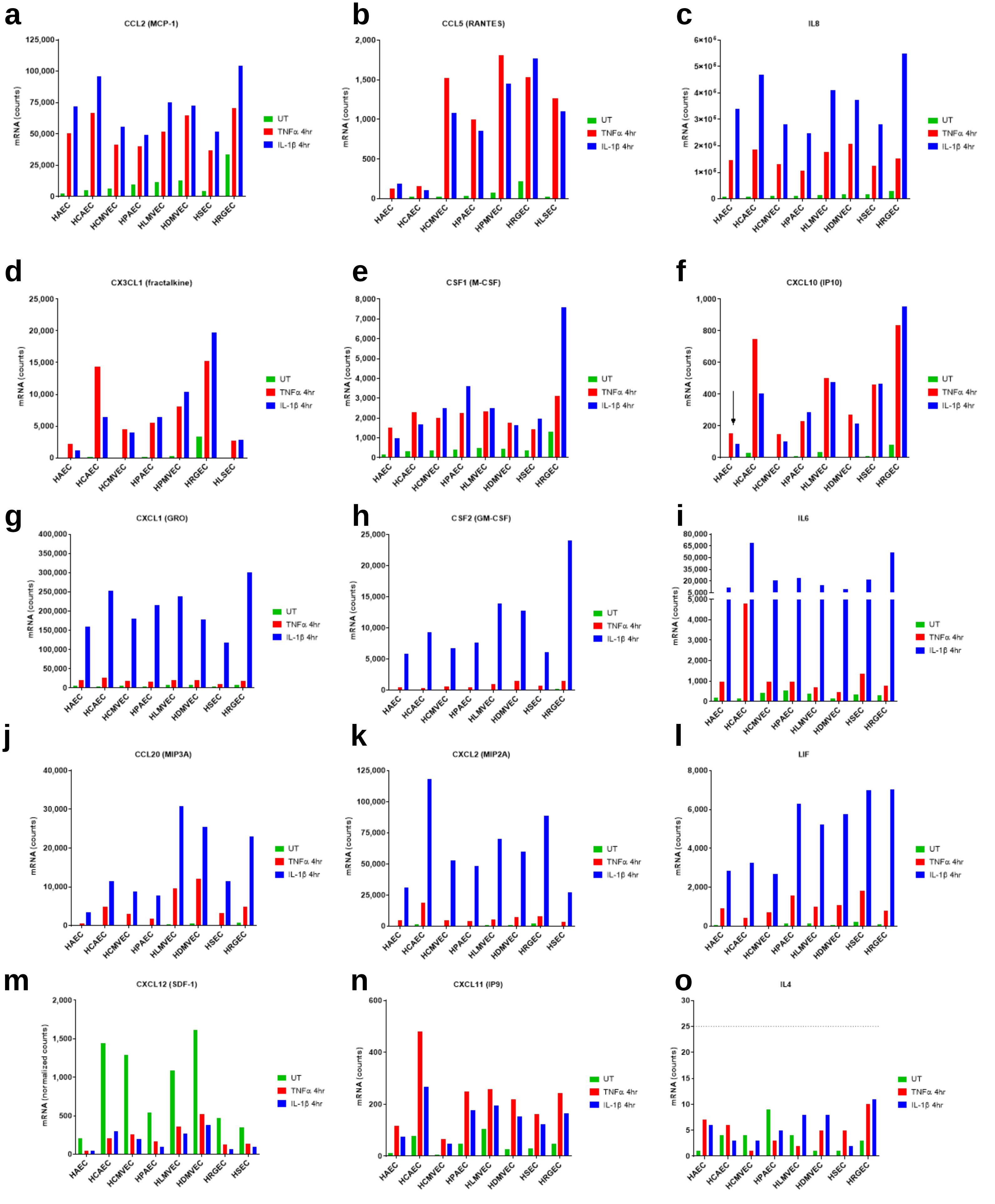
a
CCL2 (MCP-1)
mRNA (counts)
HAEC HCAEC HCMVEC HPAEC HLMVEC HDMVEC HSEC HRGEC
b
CCL5 (RANTES)
mRNA (counts)
HAEC HCAEC HCMVEC HPAEC HPMVEC HRGEC HLSEC
c
IL8
mRNA (counts)
HAEC HCAEC HCMVEC HPAEC HLMVEC HDMVEC HSEC HRGEC
d
CX3CL1 (fractalkine)
mRNA (counts)
HAEC HCAEC HCMVEC HPAEC HPMVEC HRGEC HLSEC
e
CSF1 (M-CSF)
mRNA (counts)
HAEC HCAEC HCMVEC HPAEC HLMVEC HDMVEC HSEC HRGEC
f
CXCL10 (IP10)
mRNA (counts)
HAEC HCAEC HCMVEC HPAEC HLMVEC HDMVEC HSEC HRGEC
g
CXCL1 (GRO)
mRNA (counts)
HAEC HCAEC HCMVEC HPAEC HLMVEC HDMVEC HSEC HRGEC
h
CSF2 (GM-CSF)
mRNA (counts)
HAEC HCAEC HCMVEC HPAEC HLMVEC HDMVEC HSEC HRGEC
i
IL6
mRNA (counts)
HAEC HCAEC HCMVEC HPAEC HLMVEC HDMVEC HSEC HRGEC
j
CCL20 (MIP3A)
mRNA (counts)
HAEC HCAEC HCMVEC HPAEC HLMVEC HDMVEC HSEC HRGEC
k
CXCL2 (MIP2A)
mRNA (counts)
HAEC HCAEC HCMVEC HPAEC HLMVEC HDMVEC HRGEC HSEC
l
LIF
mRNA (counts)
HAEC HCAEC HCMVEC HPAEC HLMVEC HDMVEC HSEC HRGEC
m
CXCL12 (SDF-1)
mRNA (normalized counts)
HAEC HCAEC HCMVEC HPAEC HLMVEC HDMVEC HRGEC HSEC
n
CXCL11 (IP9)
mRNA (counts)
HAEC HCAEC HCMVEC HPAEC HLMVEC HDMVEC HSEC HRGEC
o
IL4
mRNA (counts)
HAEC HCAEC HCMVEC HPAEC HLMVEC HDMVEC HSEC HRGEC
UT
TNFα 4hr
IL-1β 4hr

Figure S4.

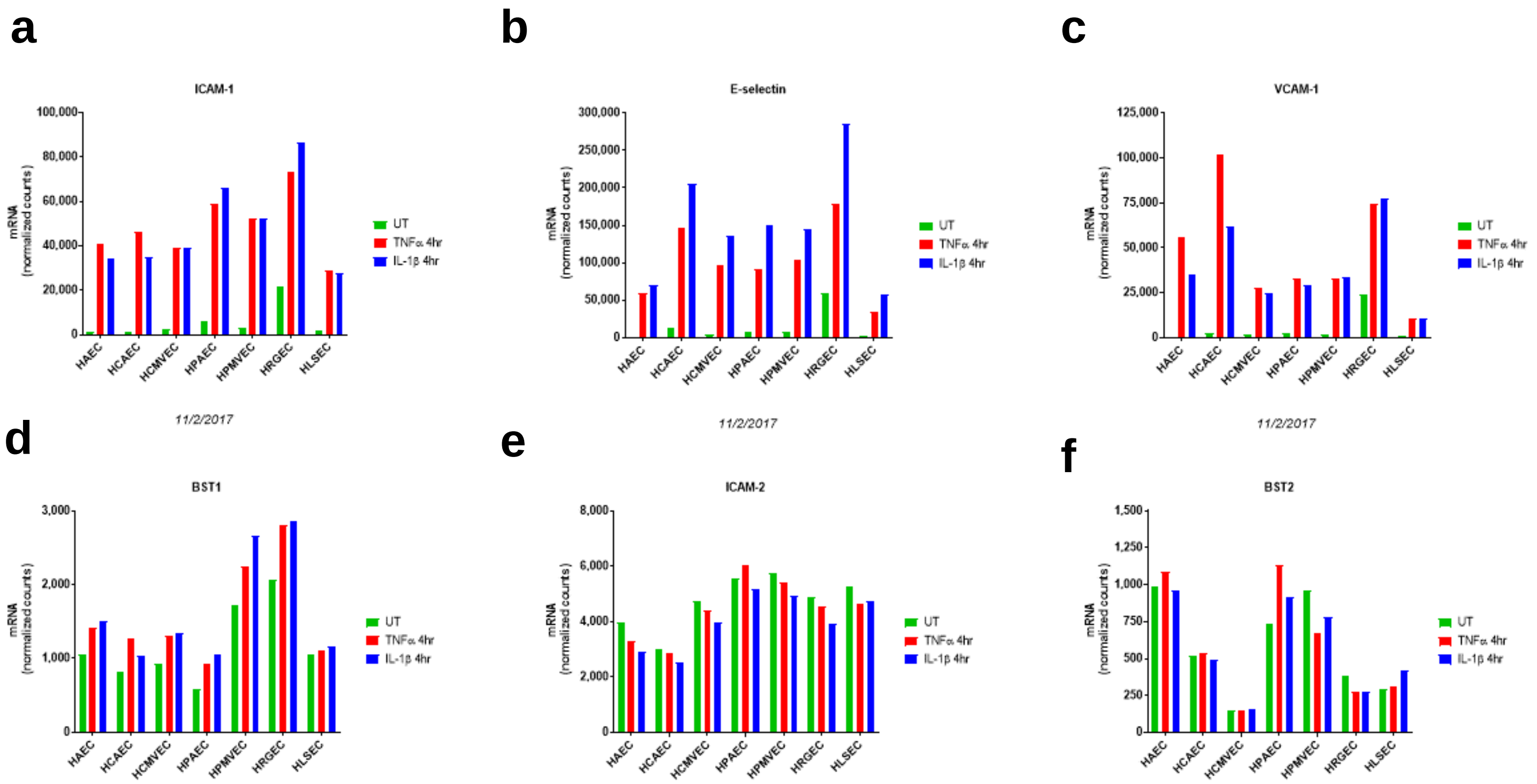
a
ICAM-1
100,000
80,000
60,000
40,000
20,000
0
mRNA (normalized counts)
UT
TNFα 4hr
IL-1β 4hr
HAEC
HCAEC
HCMVEC
HPAEC
HPMVEC
HRGEC
HLSEC
11/2/2017
b
E-selectin
300,000
250,000
200,000
150,000
100,000
50,000
0
mRNA (normalized counts)
UT
TNFα 4hr
IL-1β 4hr
HAEC
HCAEC
HCMVEC
HPAEC
HPMVEC
HRGEC
HLSEC
11/2/2017
c
VCAM-1
125,000
100,000
75,000
50,000
25,000
0
mRNA (normalized counts)
UT
TNFα 4hr
IL-1β 4hr
HAEC
HCAEC
HCMVEC
HPAEC
HPMVEC
HRGEC
HLSEC
11/2/2017
d
BST1
3,000
2,000
1,000
0
mRNA (normalized counts)
UT
TNFα 4hr
IL-1β 4hr
HAEC
HCAEC
HCMVEC
HPAEC
HPMVEC
HRGEC
HLSEC
e
ICAM-2
8,000
6,000
4,000
2,000
0
mRNA (normalized counts)
UT
TNFα 4hr
IL-1β 4hr
HAEC
HCAEC
HCMVEC
HPAEC
HPMVEC
HRGEC
HLSEC
f
BST2
1,500
1,250
1,000
750
500
250
0
mRNA (normalized counts)
UT
TNFα 4hr
IL-1β 4hr
HAEC
HCAEC
HCMVEC
HPAEC
HPMVEC
HRGEC
HLSEC

Figure S5.

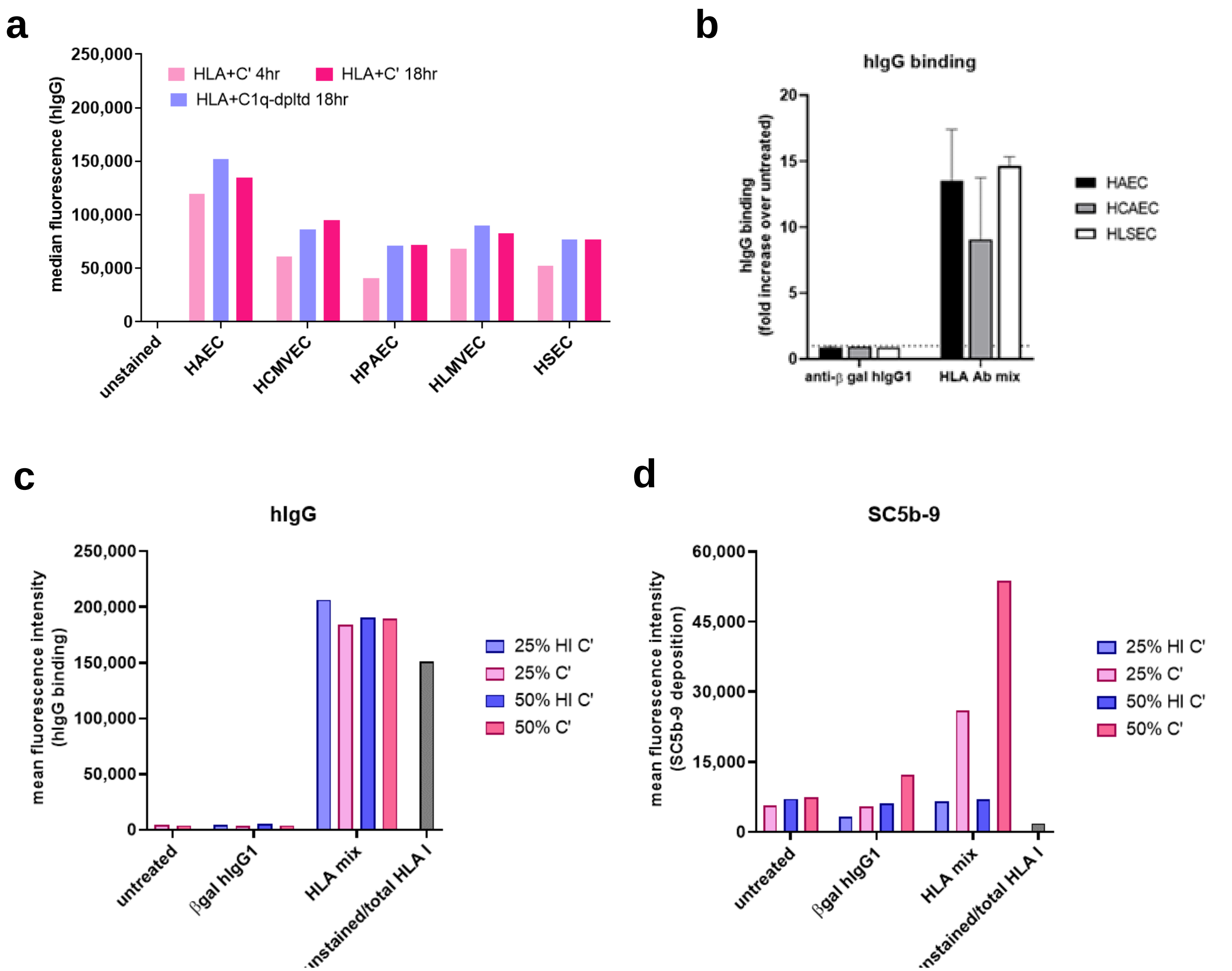
a
250,000
200,000
150,000
100,000
50,000
0
median fluorescence (hIgG)
HLA+C' 4hr
HLA+C' 18hr
HLA+C1q-dpltd 18hr
unstained
HAEC
HCMVEC
HPAEC
HLMVEC
HSEC
b
hIgG binding
hIgG binding (fold increase over untreated)
20
15
10
5
0
anti-β gal hIgG1
HLA Ab mix
HAEC
HCAEC
HLSEC
c
hIgG
mean fluorescence intensity (hIgG binding)
250,000
200,000
150,000
100,000
50,000
0
untreated
βgal hIgG1
HLA mix
unstained/total HLA I
25% HI C'
25% C'
50% HI C'
50% C'
d
SC5b-9
mean fluorescence intensity (SC5b-9 deposition)
60,000
45,000
30,000
15,000
0
untreated
βgal hIgG1
HLA mix
unstained/total HLA I
25% HI C'
25% C'
50% HI C'
50% C'

Figure S6.

e

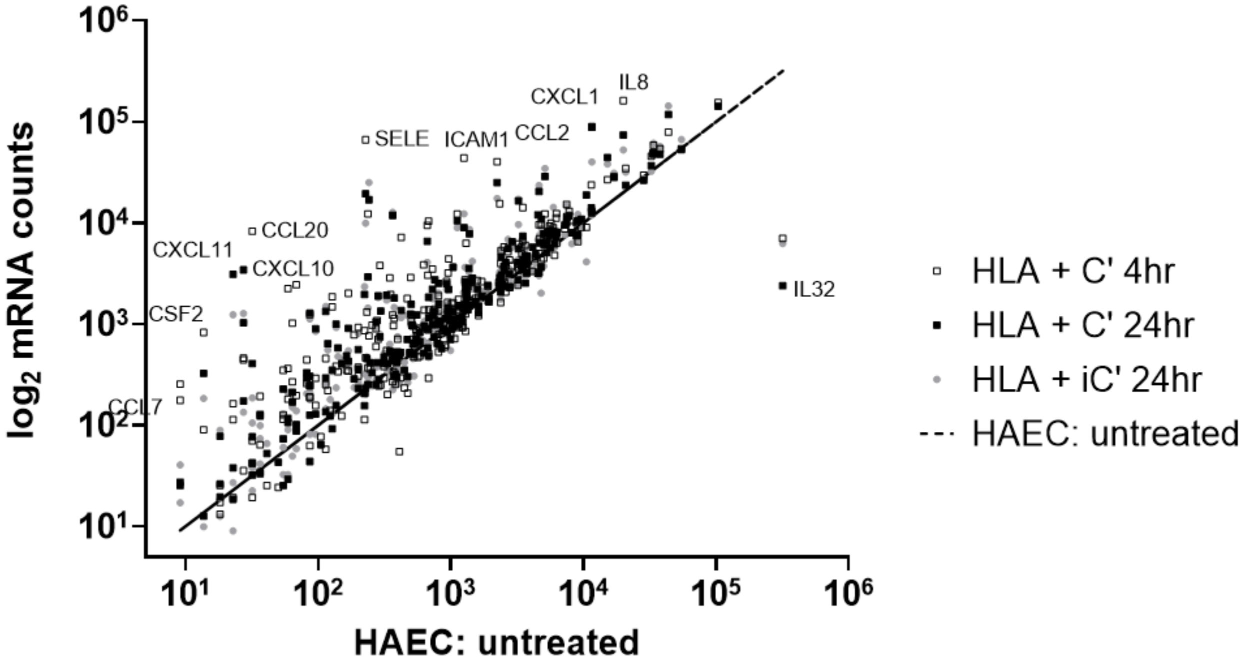

Figure S6. (continued)

f

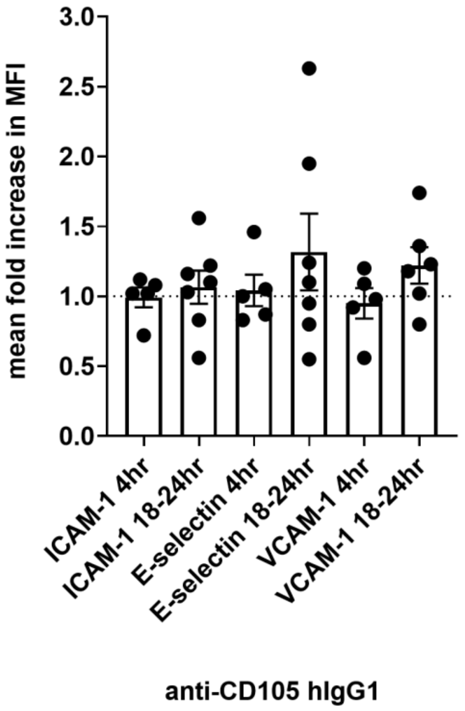

Figure S6. (continued)

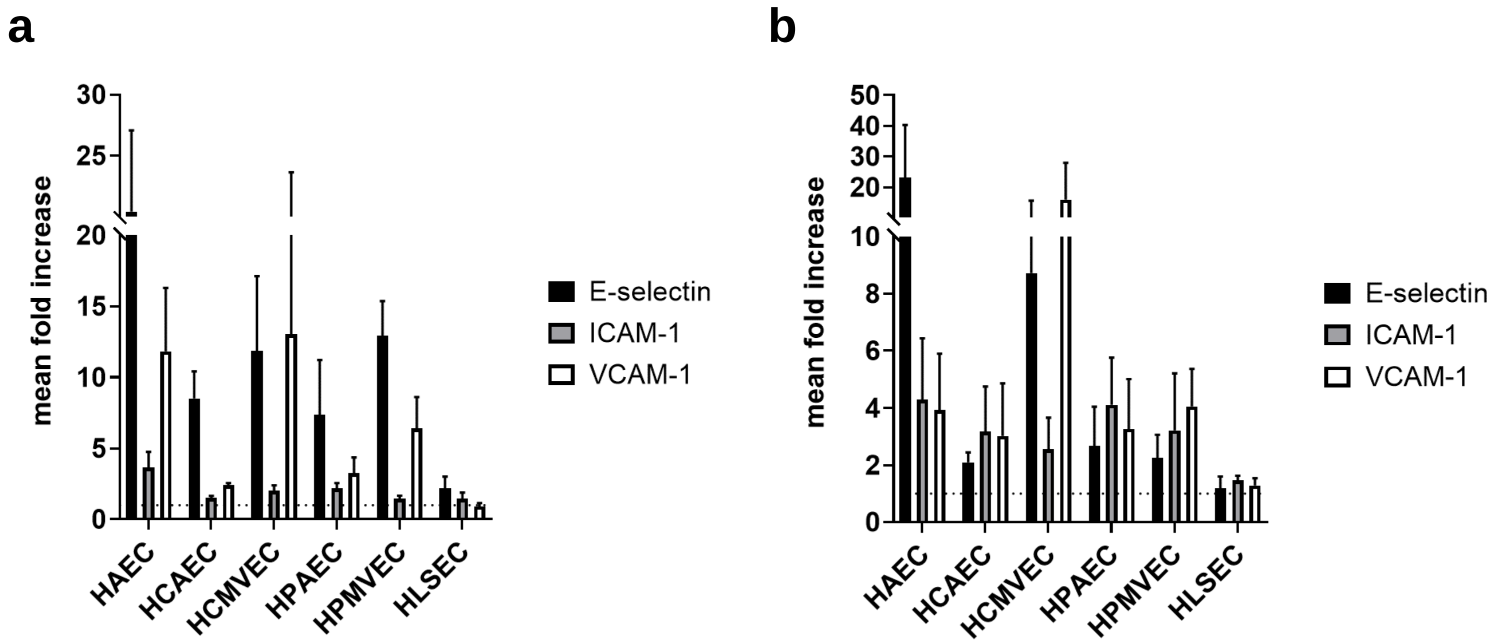
a
b
mean fold increase
E-selectin
ICAM-1
VCAM-1
HAEC
HCAEC
HCMVEC
HPAEC
HPMVEC
HLSEC
0
5
10
15
20
25
30
0
2
4
6
8
10
20
30
40
50

Figure S7.

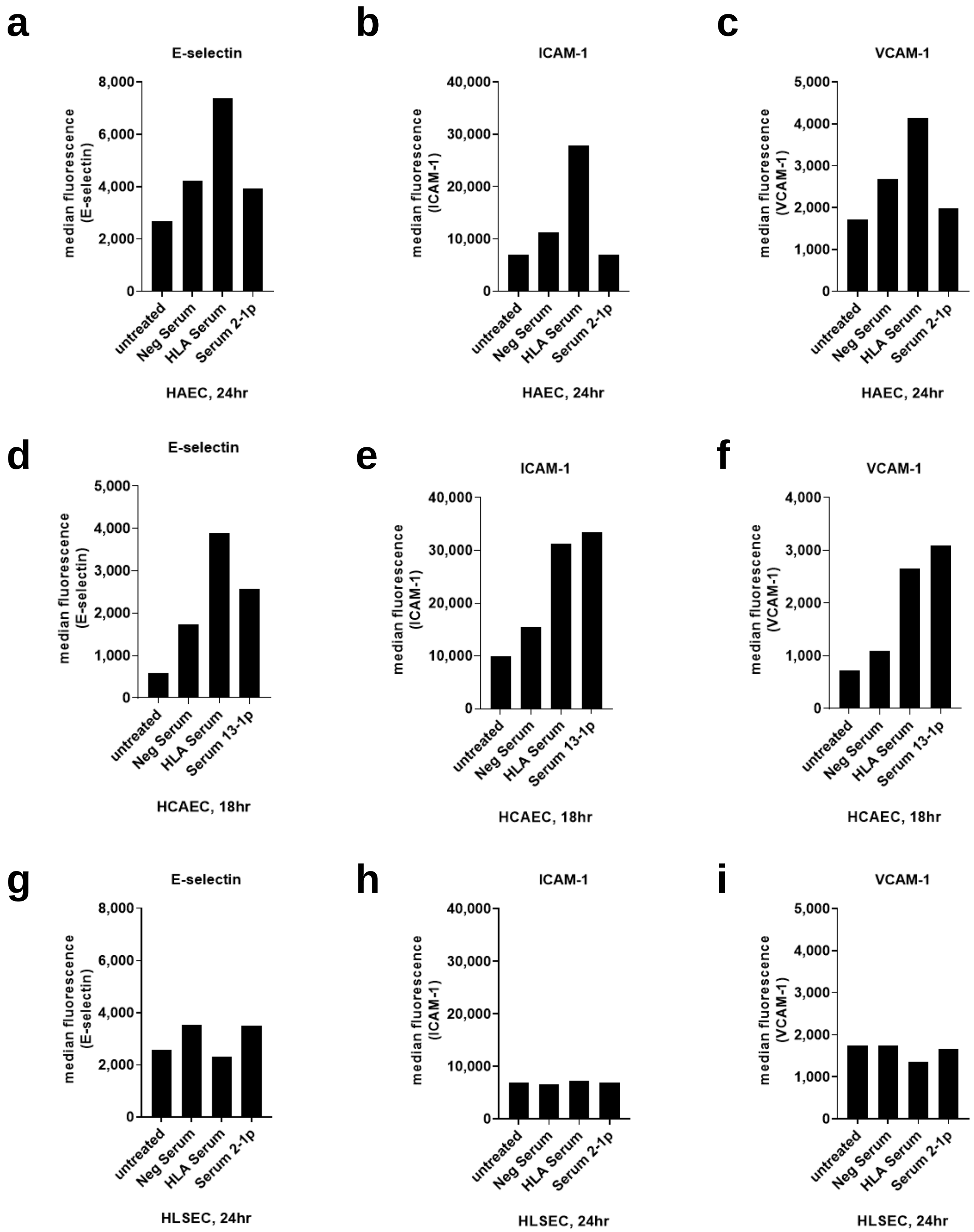
a
E-selectin
median fluorescence (E-selectin)
8,000
6,000
4,000
2,000
0
untreated
Neg Serum
HLA Serum
Serum 2-1p
HAEC, 24hr
b
ICAM-1
median fluorescence (ICAM-1)
40,000
30,000
20,000
10,000
0
untreated
Neg Serum
HLA Serum
Serum 2-1p
HAEC, 24hr
c
VCAM-1
median fluorescence (VCAM-1)
5,000
4,000
3,000
2,000
1,000
0
untreated
Neg Serum
HLA Serum
Serum 2-1p
HAEC, 24hr
d
E-selectin
median fluorescence (E-selectin)
5,000
4,000
3,000
2,000
1,000
0
untreated
Neg Serum
HLA Serum
Serum 13-1p
HCAEC, 18hr
e
ICAM-1
median fluorescence (ICAM-1)
40,000
30,000
20,000
10,000
0
untreated
Neg Serum
HLA Serum
Serum 13-1p
HCAEC, 18hr
f
VCAM-1
median fluorescence (VCAM-1)
4,000
3,000
2,000
1,000
0
untreated
Neg Serum
HLA Serum
Serum 13-1p
HCAEC, 18hr
g
E-selectin
median fluorescence (E-selectin)
8,000
6,000
4,000
2,000
0
untreated
Neg Serum
HLA Serum
Serum 2-1p
HLSEC, 24hr
h
ICAM-1
median fluorescence (ICAM-1)
40,000
30,000
20,000
10,000
0
untreated
Neg Serum
HLA Serum
Serum 2-1p
HLSEC, 24hr
i
VCAM-1
median fluorescence (VCAM-1)
5,000
4,000
3,000
2,000
1,000
0
untreated
Neg Serum
HLA Serum
Serum 2-1p
HLSEC, 24hr

Figure S8.

Supplemental Tables

Table S1

List of human endothelial cell sources and donor demographics (where available) used in this study.
HAEC: human aortic endothelial cells; HCAEC: human coronary artery endothelial cells; HCMVEC: human cardiac microvascular endothelial cells; HPAEC: human pulmonary artery endothelial cells; HPMVEC: human pulmonary microvascular endothelial cells; HRGEC: human renal glomerular endothelial cells; HLSEC: human liver sinusoid endothelial cells

Table S2

List of antibody reagents used for flow cytometry and endothelial cell stimulation in this study, including vendor, isotype and catalog numbers.

Table S3

List of non-antibody reagents used in this study, including vendor and catalog numbers.

Table S4

Flow cytometry panels used in this study, with fluorophores and antibody concentrations during staining.

Table S5

List of public datasets of murine and human endothelial cells reanalyzed for the study, with GEO accession numbers (where available) and original data source URLs.

Table S6

Data for significantly differentially expressed genes among untreated primary endothelial cells, compared with HLSEC.

Table S1.

Cell	Species	Type	Origin	Source	Catalog #	Lot #	Vendor Donor#	Internal ID
HMEC-1	Human	Immortalized	Dermal	ATCC	CRL-3243	64334159		
HAEC	Human	Primary	Aortic	Cell Biologics	H-6052	M011218Y49HAM	donor 1	CB1
HAEC	Human	Primary	Aortic	Cell Biologics	H6052	021514F14/17		CB2/#5
HAEC	Human	Primary	Aortic	ScienCell	6100	4283	4/20/2009	SC #6
HAEC	Human	Primary	Aortic	UCLA	n/a	n/a		5555
HAEC	Human	Primary	Aortic	UCLA	n/a	n/a		3F1153
HCAEC	Human	Primary	Coronary Artery	Lonza	CC-2585	0000547320	29790	#1
HCAEC	Human	Primary	Coronary Artery	PromoCell	C-12221	421Z006.3		#2
HCAEC	Human	Primary	Coronary Artery	PromoCell	C-12221	411Z027.6		#3
HCAEC	Human	Primary	Coronary Artery	Cell Biologics	H-6093	M021618	donor 1	#4
HCAEC	Human	Primary	Coronary Artery	Cell Biologics	H6093	021814M19/17		#5 CB
HCAEC	Human	Primary	Coronary Artery	ScienCell	6020	14410	7/18/2014	SC #6
HCMVEC	Human	Primary	Cardiac Microvascular	Lonza	CC-7030	0000473674	28760	#1
HCMVEC	Human	Primary	Cardiac Microvascular	PromoCell	C-12285	419Z014		#2
HCMVEC	Human	Primary	Cardiac Microvascular	PromoCell	C-12285	423Z054		#3
HCMVEC	Human	Primary	Cardiac Microvascular	Cell Biologics	H-6024	M07311Y49	donor 1	#4
HCMVEC	Human	Primary	Cardiac Microvascular	ScienCell	6000	15363	12/1/2014	SC #6
HPAEC	Human	Primary	Pulmonary Artery	Lonza	CC-2530	0000598033	31116	#1
HPAEC	Human	Primary	Pulmonary Artery	PromoCell	C-12241	415Z031		#2
HPAEC	Human	Primary	Pulmonary Artery	PromoCell	C-12241	418Z018		#3
HPAEC	Human	Primary	Pulmonary Artery	Cell Biologics	H-6059	122516BN/061917	donor 2	#4
HPAEC	Human	Primary	Pulmonary Artery	ScienCell	3100	4434	5/19/2009	SC #6
HPMVEC	Human	Primary	Pulmonary Microvascular	Lonza	CC-2527	0000547317	29774	#1
HPMVEC	Human	Primary	Pulmonary Microvascular	Lonza	CC-2527	0000549135	29812	#2
HPMVEC	Human	Primary	Pulmonary Microvascular	PromoCell	C-12281	421Z007.2		#3
HPMVEC	Human	Primary	Pulmonary Microvascular	PromoCell	C-12281	1071902.3		#4
HPMVEC	Human	Primary	Pulmonary Microvascular	Cell Biologics	H-6011	F19/091517	donor 2	#5
HPMVEC	Human	Primary	Pulmonary Microvascular	Cell Biologics	H-6011	F072517Y58		#6
HPMVEC	Human	Primary	Pulmonary Microvascular	ScienCell	3000	15902	2/22/2015	SC #7
HRGEC	Human	Primary	Renal Glomerular	ScienCell	4000	15361	0003448	#1
HRGEC	Human	Primary	Renal Glomerular	Cell Systems	ACBRI128			#2
HRGEC	Human	Primary	Renal Glomerular	Cell Biologics	H-6014G	F19mmp6/120117		#4
HRGEC	Human	Primary	Renal Glomerular	Cell Biologics	H6014G	111613F19		#5
HRGEC	Human	Primary	Renal Glomerular	ScienCell	4000	25430	7/8/2018	SC #6
HLSEC	Human	Primary	Liver Sinusoidal	ScienCell	5000	15777	0003206	#1

HLSEC	Human	Primary	Liver Sinusoidal	ScienCell	5000	16597	0003202	#2
HLSEC	Human	Primary	Liver Sinusoidal	Cell Systems	ACBRI566			#3
HLSEC	Human	Primary	Liver Sinusoidal	Cell Biologics	H-6017	M080117Y72		#4
HLSEC	Human	Primary	Liver Sinusoidal	Cell Biologics	H-6017			#5
HLSEC	Human	Primary	Liver Sinusoidal	ScienCell	5000	21045	1/26/2017	SC #6
HRGEC RNA	Human	Primary	Renal Glomerular	ScienCell	4005			total RNA
HCMVEC RNA	Human	Primary	Cardiac Microvascular	ScienCell	6005			total RNA
HAEC RNA	Human	Primary	Aortic	ScienCell	6105			total RNA
HPAEC RNA	Human	Primary	Pulmonary Artery	ScienCell	3105			total RNA
HPMVEC RNA	Human	Primary	Pulmonary Microvascular	ScienCell	3005			total RNA
HHSEC RNA	Human	Primary	Liver Sinusoidal	ScienCell	5005			total RNA
HCAEC RNA	Human	Primary	Coronary Artery	ScienCell	6025			total RNA

Table S2.

Target	Format	Host	Isotype	Vendor	City, State	Catalog #
ICAM-1	AF488	mouse	IgG1	Biolegend	San Diego, CA	322720
E-selectin	PE	mouse	IgG2a	Biolegend	San Diego, CA	322606
VCAM-1	APC	mouse	IgG1	Biolegend	San Diego, CA	305810
BST2	PE/Cy7	mouse	IgG1	Biolegend	San Diego, CA	348416
hIgG	BV510	rat	IgG2a	Biolegend	San Diego, CA	410716
HLA I	BV510	mouse	IgG2a	Biolegend	San Diego, CA	311436
BST1	AF594	mouse	IgG1	R&D Systems	Minneapolis, MN	FAB47361T-100UG
Human SC5b-9 Neoantigen	no conjugate	rabbit	IgG	Complement Technology	Tyler, Texas	A227
Rabbit IgG (H+L)	PE	goat	F(ab')2	Jackson Immuno	West Grove, PA	111-116-144
CD11a	FITC	mouse	IgG1	BD Biosciences	San Jose, CA	555383
CD56	PE/Cy7	mouse	IgG1	Biolegend	San Diego, CA	318318
CD14	PerCP/Cy5.5	mouse	IgG1	Biolegend	San Diego, CA	325622
CD19	APC	mouse	IgG1	Biolegend	San Diego, CA	392504
CD3	APC/H7 or APC/Fire750	mouse	IgG1	Biolegend	San Diego, CA	317352
Endoglin (CD105)	BV510	mouse	IgG1	BD Biosciences	San Jose, CA	563264
HLA-DR	BV421	mouse	IgG2a	Biolegend	San Diego, CA	307636
MCAM (CD146)	PerCP/Cy5.5	mouse	IgG2a	Biolegend	San Diego, CA	342013
β-galactosidase	no conjugate	chimeric human	IgG1	Invivogen	San Diego, CA	bgal-mab1
HLA I	no conjugate	chimeric human	IgG1	Invivogen	San Diego, CA	hla-c1
Endoglin (CD105)	no conjugate	chimeric human	IgG1	MediMabs	Montreal, QC	MM-0300
MCAM (CD146)	no conjugate	chimeric human	IgG1	MediMabs	Montreal, QC	MM-0302
HLA I	no conjugate	chimeric human	IgG1	One Lambda/ThermoFisher	West Hills, CA	n/a
HLA I	no conjugate	chimeric human	IgG3	One Lambda/ThermoFisher	West Hills, CA	n/a
HLA-A2/A68/A69 (clone SN607D8	no conjugate	human	IgG1	Leiden		n/a
HLA-A2/B57/B58 (clone SN230G6	no conjugate	human	IgG1	Leiden		n/a
HLA-A3/A11/A1/A36 (clone MUL4	no conjugate	human	IgG1	Leiden		n/a
HLA-A1/A3/A11/A36/A80 (clone M	no conjugate	human	IgG1	Leiden		n/a
HLA-Bw4 (clone MUS4H4rec)	no conjugate	human	IgG1	Leiden		n/a

Table S3.

Reagent	Vendor	City, State	Catalog #
Compensation Beads	BD Biosciences	San Jose, CA	552843
TNFα (recombinant, Humankine)	Sigma Aldrich	St. Louis, MO	H8916-10UG
IFNγ	R&D Systems	Minneapolis, MN	285IF-100/CF
Recombinant human IL-4, CF	R&D Systems	Minneapolis, MN	204-IL-010/CF
Recombinant human IL-10, CF	R&D Systems	Minneapolis, MN	217-IL-005/CF
IL-1β, recombinant human	Sigma-Aldrich	St. Louis, MO	I9401-5UG
Normal Human Complement Serum	Complement Technology	Tyler, Texas	NHS
Human C1q-depleted Complement Serum	Complement Technology	Tyler, Texas	A300
Human C3-depleted Complement Serum	Complement Technology	Tyler, Texas	A314
Human CCL5/RANTES Quantikine ELISA Kit	R&D Systems	Minneapolis, MN	DRN00B
CXCL11/I-TAC DuoSet ELISA, 15 plate	R&D Systems	Minneapolis, MN	DY672
CXCL10/IP-10 DuoSet ELISA, 5 plate	R&D Systems	Minneapolis, MN	DY266-05
CCL20/MIP3a DuoSet ELISA, 5 plate	R&D Systems	Minneapolis, MN	DY360-05
DuoSet ELISA Ancillary Reagent Kit 2	R&D Systems	Minneapolis, MN	DY008
Ficoll-Paque Premium 1.078g/mL, GE Healthcare	Fisher	Waltham, MA	45-001-751
gelatin type B solution	Sigma-Aldrich	St. Louis, MO	G1393-100ML
RLT Buffer	Qiagen	Germantown, MD	79216
Endothelial Growth Medium	PromoCell	Heidelberg, Germany	C-22020

Table S4

Panel	Target	Format	Dilution Factor
1	ICAM-1	AF488	2.5:100
1	E-selectin	PE	5:100
1	VCAM-1	APC	5:100
1	BST2	PE/Cy7	2.5:100
2	ICAM-1	AF488	2.5:100
2	E-selectin	PE	5:100
2	VCAM-1	APC	5:100
2	BST2	PE/Cy7	2.5:100
2	hIgG	BV510	5:100
3	anti-Human SC5b-9 Neoantigen	no conjugate	1:200
3	anti-rabbit IgG (H+L)	PE	1:100
3	hIgG	BV510	5:100
4	CD11a	FITC	5:100
4	CD56	PE/Cy7	5:100
4	CD14	PerCP/Cy5.5	5:100
4	CD19	APC	5:100
4	CD3	APC/H7 or APC/Fire750	5:100
4	Endoglin (CD105)	BV510	2.5:100
4	HLA-DR	BV421	5:100

Table S5.

PMID	Species	Description	GEO Dataset	Available at
25561514	mouse	heart and lung bulk endothelial cells (Tie2+)	GSE48209	https://www.ncbi.nlm.nih.gov/geo/query/acc.cgi?acc=GSE48209
30283141	mouse	scRNA-Seq of 20 mouse organs	GSE109774	https://tabula-muris.ds.czbiohub.org/
32059779	mouse	scRNA-Seq of heart, kidney, lung and liver endothelial cells (CD31+)		https://endotheliomics.shinyapps.io/ec_atlas/
30188322	mouse	kidney, lung and liver bulk endothelial cells	GSE111839	https://markfsabbagh.shinyapps.io/vectrdb/
31944177	mouse	heart and lung bulk endothelial cells (Cdh5+)	GSE136848	https://www.ncbi.nlm.nih.gov/geo/query/acc.cgi?acc=GSE136848
31712416	mouse	heart, kidney and liver bulk endothelial cells (Tek+)	GSE138629	https://www.ncbi.nlm.nih.gov/geo/query/acc.cgi?acc=GSE138629
27582018	human	aortic (normal), hepatic sinusoid (normal); enriched and specific transcripts		http://angiogenes.uni-frankfurt.de/
24108462	human	hepatic artery (n=3); aortic (n=2); coronary artery (n=2); pulmonary artery (n=3)	GSE43475	https://www.ncbi.nlm.nih.gov/geo/query/acc.cgi?acc=GSE43475
30240741	human	fetal (3 month gestation) heart, kidney, lung and liver bulk endothelial cells	GSE114607	https://www.ncbi.nlm.nih.gov/geo/query/acc.cgi?acc=GSE114607
30795962	human	human cardiac allograft biopsies	GSE124897	https://www.ncbi.nlm.nih.gov/geo/query/acc.cgi?acc=GSE124897

Table S6

HAEC	log2fc	P-value
THY1	-6.64386	1.69E-11
PDGFRB	-6.64386	1.04E-08
LCP2	-4.64386	4.35E-06
LIF	-4.64386	4.90E-06
TNFAIP6	-4.05889	6.54E-06
CX3CL1	-4.64386	2.98E-05
C1R	-3.47393	7.03E-05
PPARG	-2.39593	0.000165
IFITM1	-3.64386	0.000191
ITGAX	-2.94342	0.00024
VCAM1	-3.8365	0.000413
TNFRSF9	-3.05889	0.00067
SOCS1	-1.94342	0.000891
C1S	-3.8365	0.00114
CCL7	-1.21759	0.00263
CCL20	-3.47393	0.00303
IL7R	-3.05889	0.00318
ABCB1	3.080658	0.00374
CFH	1.761285	0.00713
CD97	-1.64386	0.00731
CCRL1	2.620586	0.0086
FCGR2B	-2.94342	0.00929
LITAF	-1.68966	0.0097
SERPING1	-4.32193	0.0106
IL1B	-2	0.0108
FKBP5	-1.73697	0.0109
STAT1	1.691534	0.0114
SELL	1.691534	0.0137
C1QB	-2.73697	0.0151
CD3D	-2.64386	0.0158
CEBPB	-1.51457	0.0164
EGR1	-2.05889	0.0166
CRADD	-1.02915	0.0193
CD4	-2.73697	0.0196
CARD9	-2.12029	0.0209
CSF1	-1.152	0.024
CD1D	-2.32193	0.0255
NFATC1	1.238787	0.0274
CD36	-2.32193	0.0313
CD8B	-2.39593	0.0321
TNFRSF11/	1.378512	0.0381
NFIL3	-1.05889	0.0416
PSMB9	-1.05889	0.0443

HCAEC	log2fc	P-value
CD24	6.519951	7.26E-07
THY1	-5.64386	1.10E-05
STAT4	4.739848	2.27E-05
TNFAIP6	-4.32193	2.47E-05
C7	3.450221	0.000111
STAT1	3.31034	0.000116
IFITM1	-4.32193	0.000233
LCP2	-4.32193	3.44E-04
LEF1	3.140779	0.000435
EDNRB	2.589763	0.000451
LIF	-4.05889	4.91E-04
CD34	4.079805	0.000517
C1S	-5.05889	0.000663
LITAF	-2.64386	0.00126
SOCS1	-2.25154	0.00151
CCL7	-3.47393	0.00163
IL1B	-3.47393	0.00173
TGFBI	-2.8365	0.00349
CLU	2.78031	0.00393
CFH	2.100978	0.00796
ARG2	1.929791	0.00798
PDGFRB	-3.47393	8.02E-03
BCL2L11	2.080658	0.00802
ENTPD1	2.560715	0.00808
IDO1	-3.8365	0.00881
IL5	-2.8365	0.00903
CCL16	2.729009	0.0109
TNFRSF11/	2.060047	0.0115
LILRB3	-2.73697	0.0116
MSR1	2.849999	0.0118
CD247	-2.39593	0.0123
MS4A1	-2.39593	0.0126
TNFSF10	2.080658	0.0132
KLRF2	-2.32193	0.014
PTAFR	-2.64386	0.014
TLR4	1.748461	0.0144
LAMP3	2.430285	0.0158
NOD1	1.400538	0.0171
TCF4	1.49057	0.0178
TAL1	1.778209	0.0194
C8B	-1.8365	0.0214
CD1D	-2.94342	0.0228
IRF8	-3.05889	0.024
HLA-DRA	0.678072	0.0242
IFNB1	-1.94342	0.0255
TNFRSF13I	-2.12029	0.0261
IL27	-2.47393	0.0269
NCR1	2.49057	0.0273
CD70	-1.68966	0.0275
CD4	-3.47393	0.0284
CD3D	-1.78588	0.0302

HCMVEC	log2fc	P-value
PDGFRB	-6.64386	5.69E-08
C1R	-4.32193	3.67E-06
THY1	-5.05889	4.80E-06
SPP1	4.83996	5.19E-06
C1S	-5.64386	1.07E-05
ITLN1	3.439623	1.52E-05
TNFAIP6	-3.8365	1.62E-05
LIF	-4.05889	4.36E-05
LCP2	-3.8365	1.22E-04
CD34	3.680324	0.000278
IFITM1	-3.32193	0.000625
IL1R1	-2.47393	0.000993
IL1B	-2.64386	0.002
TGFBI	-2.47393	0.00295
CCL7	-1.68966	0.00308
IL16	2.459432	0.00366
SOCS1	-1.47393	0.0104
KIT	2.541019	0.0115
CD24	2.459432	0.0129
CCL20	-2.39593	0.0133
LITAF	-1.68966	0.0133
CD4	-3.05889	0.0134
CEBPB	-1.51457	0.0177
PRDM1	1.778209	0.0202
CFP	1.560715	0.025
IL7R	-2.32193	0.0254
CD19	-2.39593	0.0274
STAT5A	1	0.0284
VCAM1	-2.25154	0.0317
BST2	-1.88897	0.0326
HLA-DPB1	1.422233	0.0337
CD1D	-2.25154	0.0363
CRADD	-0.94342	0.0374
IL21R	-1.94342	0.0424
SELL	1.389567	0.047
FKBP5	-1.32193	0.0496

HPAEC	log2fc	P-value
THY1	-9.15	2.21E-10
C7	4.31	7.33E-07
ITLN1	4.01	1.08E-06
PDGFRB	-6.04	2.36E-06
CFH	3.49	6.29E-06
C1R	-3.87	3.32E-05
IL1B	-5.52	0.000041
STAT5A	2.02	7.49E-05
CD80	3.04	0.000091
KIR_Activa	3.25	9.65E-05
LCP2	-4.28	1.16E-04
STAT4	3.35	0.000532
STAT5B	1.58	0.000538
CASP2	1.87	0.000582
MCL1	1.77	0.00077
ITLN2	2.82	0.000891
CSF3R	2.83	0.000972
IFNAR2	1.87	0.000983
STAT1	2.43	0.00103
TAL1	2.28	0.00111
FCGR2B	-2.37	0.00122
MAPKAPK:	1.73	0.00135
ATG12	1.99	0.00135
CCR1	2.49	0.00144
ARHGDIB	1.82	0.00146
ILF3	1.68	0.00146
MAPK1	1.71	0.00155
CD1A	3.19	0.00167
ABL1	1.57	0.00229
NOTCH1	1.73	0.00232
CD3EAP	1.87	0.00242
PSMD7	1.66	0.00269
C14orf166	1.66	0.003
NFKB1	1.57	0.00308
TLR3	1.88	0.00335
CD99	1.62	0.00337
TNFSF12	1.77	0.00369
C1QBP	1.49	0.00377
TNFRSF1B	2.29	0.00383
PSMC2	1.57	0.00407
ITGAE	1.53	0.00435
NFATC1	1.75	0.0044
PSMB7	1.54	0.00445
SRC	1.69	0.00469
CCL23	2.82	0.00478
STAT3	1.44	0.00479
PSMB5	1.52	0.00494

HPMVEC	log2fc	P-value
TNFAIP6	-4.05889	6.25E-06
THY1	-4.32193	3.69E-05
PDGFRB	-4.64386	6.84E-05
SOCS1	-2.39593	9.69E-05
C1S	-4.64386	0.000103
CD34	3.710393	0.000258
C1R	-3.05889	3.30E-04
IL1B	-2.94342	0.00108
CD36	3.459432	0.00217
LIF	-2.8365	2.87E-03
ARG2	1.799087	0.00408
CCL7	-1.18442	0.00497
STAT5A	1.292782	0.00553
TNFSF10	1.941106	0.00724
LITAF	-1.8365	0.00755
TNFRSF11/	1.851999	0.00834
TNFRSF9	-2.32193	0.00847
CD4	-3.18442	0.0097
CD1D	-2.64386	0.0141
IFITM1	-2.25154	0.0158
BCL2L11	1.580145	0.017
CD40	1.339137	0.0201
KLRG1	1.811471	0.0215
CARD9	-2.05889	0.0237
IL6R	1.480265	0.0238
NOTCH1	1.189034	0.0253
CEBPB	-1.4344	0.0256
ITGAX	-1.68966	0.026
CTSS	1.70044	0.0279
SELL	1.521051	0.0327
CEACAM1	1.378512	0.0334
TGFBI	-1.73697	0.0337
CRADD	-0.94342	0.0338
CD14	-1.64386	0.0344
TAL1	1.339137	0.0379
VCAM1	-2.18442	0.0387
TNFRSF10(	1.422233	0.04
HLA-DPB1	1.350497	0.0432
KIT	2	0.0457

HRGEC	log2fc	P-value
CEACAM8	5.249825	5.28E-08
IDO1	5.219943	3.65E-07
LY96	4.450221	5.19E-07
IRAK2	4.169925	5.43E-07
B2M	3.439623	1.03E-06
IL20	5.309976	1.24E-06
NT5E	3.140779	1.33E-06
FYN	2.920293	1.52E-06
MYD88	2.929791	1.76E-06
TAP1	3.279471	2.48E-06
IFIH1	3.469886	2.81E-06
IL8	4.909773	2.85E-06
TGFBR2	3.279471	3.2E-06
CTSS	4.22033	3.23E-06
CCL5	5.219943	3.48E-06
STAT2	2.889474	3.72E-06
CD99	2.78031	3.99E-06
TICAM1	3.49057	4.06E-06
ICOSLG	3.600508	4.68E-06
IFNGR1	2.599318	4.72E-06
TRAF3	3.070389	4.85E-06
CD46	2.970854	5.2E-06
IRAK1	2.819668	6.05E-06
IL4R	2.440952	6.36E-06
CD81	2.739848	6.76E-06
CCL2	4.230357	6.99E-06
RELA	3.289834	7.05E-06
IFI35	3.200065	7.3E-06
PTK2	2.599318	7.36E-06
CSF2	3.609991	7.52E-06
MX1	4.82985	7.71E-06
CD58	2.889474	8.25E-06
ITGB1	2.629939	8.44E-06
PSMB5	2.629939	8.45E-06
MAP4K4	2.82985	8.79E-06
ICAM1	3.82985	9.8E-06
HLA-A	3.089159	1.01E-05
TOLLIP	3.249445	1.01E-05
ATG5	2.408712	1.04E-05
MAPK1	2.531069	1.09E-05
NFKB1	2.510962	1.11E-05
BCAP31	2.70044	1.24E-05
NFATC1	2.910733	1.24E-05
CASP3	2.980025	1.25E-05
ICAM3	2.950468	1.35E-05
BCL10	2.729009	1.41E-05
ARHGDIB	2.639232	1.45E-05
TLR3	2.989139	1.45E-05
ABL1	2.381283	1.48E-05
TAPBP	2.939227	1.53E-05
PSMB7	2.521051	1.62E-05

CD36	2.769772	0.0305
IKZF3	-2.32193	0.0324
IL2	-2.32193	0.0339
CD74	2.269033	0.0349
CD14	-1.8365	0.0354
CARD9	-2.39593	0.0366
STAT5A	1.097611	0.0392
CD2	-2.12029	0.0393
TNFRSF9	-2.05889	0.0421
LILRB1	-1.88897	0.0461
TNFRSF1B	1.718088	0.0466
IL7R	-2.39593	0.0477

TNFRSF14	3.190615	1.62E-05
PML	3.099295	1.71E-05
SMAD3	2.799087	1.73E-05
IFNAR2	2.570463	1.76E-05
CD55	2.680324	0.000018
IRF7	3.629939	1.88E-05
STAT6	2.910733	1.89E-05
CD164	2.669027	1.93E-05
PRKCD	2.580145	1.95E-05
TAP2	2.989139	1.97E-05
NCAM1	3.980025	0.00002
RAF1	2.381283	2.02E-05
SRC	2.729009	2.11E-05
IFNAR1	2.950468	2.14E-05
ITGAE	2.430285	2.22E-05
TRAF2	2.620586	2.23E-05
LITAF	-3.32193	2.24E-05
APP	2.799087	2.47E-05
GPI	2.709291	2.57E-05
MAPKAPK2	2.389567	2.68E-05
TP53	2.22033	2.79E-05
IL18R1	3.22033	0.000028
PSMC2	2.430285	2.99E-05
NFKBIA	2.500802	3.02E-05
ITGA5	2.639232	3.11E-05
ATG12	2.709291	3.19E-05
PYCARD	2.580145	3.27E-05
BAX	2.31904	3.28E-05
NFKB2	2.669027	3.29E-05
IKBKG	2.211012	3.45E-05
UBE2L3	2.179511	3.45E-05
TNFSF12	2.689299	3.46E-05
HLA-B	3.350497	3.84E-05
IL6ST	2.111031	3.95E-05
TYK2	2.370164	4.22E-05
TBK1	2.250962	4.49E-05
CD9	3.600508	4.87E-05
PDGFRB	-5.05889	5.04E-05
MIF	2.550901	5.11E-05
PDCD1LG2	2.85997	5.12E-05
BST1	3.049631	5.22E-05
CTNNB1	2.169925	5.22E-05
CCRL1	4.609991	5.36E-05
RELB	2.550901	5.53E-05
IGF2R	2.729009	5.56E-05
CD59	2.939227	5.63E-05
FCGRT	2.82985	5.83E-05
ETS1	2.49057	5.92E-05
TMEM173	3.129283	5.92E-05
PSMD7	2.330558	6.31E-05
IRAK4	2.140779	6.55E-05
MAP4K2	2.809414	6.59E-05
SKI	2.330558	6.64E-05

TRAF6	2.85997	6.68E-05
IL13RA1	2.269033	6.77E-05
PSMB10	2.370164	6.98E-05
IKBKB	2.879706	7.37E-05
IRF1	2.280956	8.03E-05
PTGER4	3.64039	8.04E-05
STAT3	2.130931	8.05E-05
IL12B	3.389567	8.09E-05
MAPK14	2.250962	8.16E-05
CASP2	2.189034	8.34E-05
TAL1	2.83996	8.41E-05
PSMB8	2.469886	8.45E-05
THY1	-4.32193	8.49E-05
CXCL1	3.350497	9.01E-05
STAT5B	1.819668	9.44E-05
TIRAP	2.639232	9.53E-05
SMAD5	2.15056	9.62E-05
CDH5	2.611172	0.0001
ZEB1	2.469886	0.000116
HLA-C	3.529821	0.000119
CHUK	2.211012	0.000122
LTBR	2.480265	0.000123
CD3EAP	2.459432	0.000129
C1R	-3.47393	1.30E-04
MCL1	2.060047	0.000132
JAK1	2.49057	0.000133
CFB	3.769772	0.000139
IL12A	3.620586	0.000146
CCL24	3.099295	0.000151
TNFRSF10(	2.920293	0.000152
PECAM1	2.709291	0.000164
HLA-DRB1	3.109361	0.000164
CTSC	2.611172	0.000166
CCL4	3.140779	0.000169
TCF4	2.229588	0.000171
CCND3	2.70044	0.000174
STAT1	2.83996	0.000174
PLAU	3.64039	0.000183
CD40	2.389567	0.000185
PDCD2	2.358959	0.000196
PTPN2	1.978196	0.000197
PSMB9	2.269033	0.000205
IRF3	2.531069	0.000222
EOMES	3.119356	0.000224
IKBKE	2.510962	0.000229
ILF3	2	0.00023
IL1A	3.64039	0.000272
IFI16	2.289834	0.000276
TGFBR1	2.260026	0.000284
DEFB1	3.179511	0.000299
TLR4	2.400538	0.00032
JAK2	1.910733	0.000322
CCL26	-5.05889	0.000357

HRAS	2.179511	0.000364
S1PR1	2.729009	0.000365
IL2RB	3.229588	0.000365
LILRA3	2.849999	0.000367
BCL3	2.130931	0.000368
IFIT2	3.580145	0.000394
CD276	2.469886	0.000396
GBP1	2.419539	0.000396
ATG16L1	2.02148	0.00042
CD82	2.869871	0.000435
LTF	-3.05889	0.000439
CDKN1A	2.589763	0.000441
C14orf166	2.01078	0.000453
CCRL2	3.340562	0.000476
TFRC	2.31034	0.000479
CX3CL1	3.83996	4.92E-04
NOD2	2.560715	0.000495
C8B	-3.05889	0.00051
TNFAIP6	-3.18442	5.32E-04
IKBKAP	1.918386	0.000537
ATG7	1.769772	0.000544
C1QBP	1.819668	0.000549
ABCB1	4.040016	0.000552
NFKBIZ	2.090853	0.000574
POU2F2	3.920293	0.000578
NOTCH2	2.669027	0.000596
KCNJ2	3.039138	0.000597
ICAM2	3.519793	0.000623
IL6	2.750607	0.000649
MAPK11	2.869871	0.000672
CEACAM1	2.400538	0.000687
C1QB	-3.05889	0.000741
ITGAM	3.330558	0.000746
TNFSF13B	3.109361	0.000795
CD83	2.750607	0.000828
TRAF5	1.899176	0.000838
ITGA6	2.639232	0.000843
ADA	1.459432	0.000849
TNFAIP3	2.350497	0.000871
NFATC3	1.618239	0.000887
BST2	3.209453	0.0009
CD2	-2.94342	0.000965
KLRG1	2.769772	0.00113
CD79A	2.920293	0.00118
B3GAT1	-2.47393	0.00123
CXCR1	-2.8365	0.00125
CCR10	3.260026	0.00128
IL5	-2.8365	0.00134
NOTCH1	1.831877	0.00137
CYBB	-3.05889	0.00137
TLR2	2.659925	0.00138
CD44	3.529821	0.00139
IL29	3.300124	0.00141

CD3D	-2.39593	0.00149
HAMP	-2.73697	0.00149
CD4	-5.05889	0.00158
NFATC2	2.350497	0.00163
MME	2.950468	0.00163
CSF1	1.769772	0.00165
BCL6	1.851999	0.00169
IL17F	3.279471	0.00173
RUNX1	2.280956	0.00174
PLA2G2E	-2.64386	0.00175
NOD1	1.678072	0.00178
CD8B	-2.73697	0.00178
KLRF2	-2.64386	0.0018
LILRB3	-2.73697	0.00183
IL15	2.419539	0.0019
LTB4R	2.22033	0.00192
PLAUR	2.298658	0.00193
LGALS3	1.929791	0.00197
CASP1	1.831877	0.002
IRF8	-4.64386	0.00201
KLRK1	-2.8365	0.00201
BCL2	2.31904	0.00206
ZAP70	-2.73697	0.00215
IL10	-2.05889	0.00217
CFI	2.550901	0.00221
CD19	-3.32193	0.00223
IL9	-2.73697	0.00233
RORC	2.950468	0.00242
KLRF1	-2.64386	0.00245
CD14	-3.05889	0.00253
CSF2RB	3.15056	0.00264
ATG10	1.580145	0.00267
IL1RL1	5.910013	0.00268
CSF1R	-2.64386	0.00269
CD34	3.070389	0.0027
BLNK	-2.55639	0.00276
NFIL3	1.709291	0.00282
TRAF1	5.130107	0.00289
TRAF4	1.819668	0.00321
CD247	-2.25154	0.00327
CD45RA	-2.64386	0.00347
EBI3	2.260026	0.0038
CFH	2.049631	0.00387
GZMK	-2.55639	0.00418
HLA-DQB1	-2.64386	0.00424
PRF1	-2.55639	0.00426
TGFB1	1.641546	0.00472
ATM	2.580145	0.00476
FCGR2B	-3.05889	0.00486
MS4A1	-2.8365	0.00497
MARCO	-2.12029	0.00503
FCGR1A/B	-2.39593	0.00518
TNF	2.459432	0.00519

LILRB1	-2.32193	0.00526
CXCL2	2.430285	0.00595
KIR3DL1	-2.47393	0.00614
PTAFR	-2.32193	0.00618
IL7	-2.47393	0.00652
GFI1	1.899176	0.00658
PTGS2	2.790772	0.00671
ITGAX	-2.8365	0.00686
CD36	-3.18442	0.00696
CD45R0	-2.64386	0.007
CCL20	2.521051	0.00748
TNFRSF13I	-2.39593	0.00772
IKZF3	-2.39593	0.00788
STAT5A	1.280956	0.00796
FN1	1.632268	0.00807
SLAMF1	-2.47393	0.00874
PLA2G2A	-2.8365	0.00889
CCL19	-1.68966	0.00897
XBP1	1.378512	0.00903
CD274	1.641546	0.00904
TNFSF15	6.179909	0.00938
RARRES3	2	0.00959
TLR1	2.189034	0.00997
CCL18	-2.18442	0.0101
CCR7	-2.25154	0.0106
ARG2	1.641546	0.0109
AHR	1.298658	0.0111
IFNB1	-2.55639	0.0115
IL2RA	-2.47393	0.013
CD1D	-3.47393	0.0132
MALT1	1.378512	0.015
FCGR2A	-2.12029	0.0155
IL27	-2.39593	0.0164
FAS	1.778209	0.0166
SELE	4.550285	0.0175
ITLN2	-2.94342	0.0188
IL21	-2.05889	0.0192
TNFSF4	2.121015	0.0206
PDGFB	2.080658	0.0207
IL2	-1.94342	0.0216
S100A8	-2.05889	0.0221
FADD	0.879706	0.0226
IL23R	-1.73697	0.0232
KIT	2.358959	0.0233
IL22RA2	-2	0.024
ITLN1	-2.47393	0.0243
TNFSF10	1.669027	0.0245
CCL7	-3.8365	0.0245
DEFB4A	-2.25154	0.0246
CASP8	0.9855	0.0249
CFD	-2.94342	0.0255
ICAM5	1.871844	0.026
PRDM1	1.739848	0.0269

IL1B	-1.8365	0.0277
MASP1	-2.55639	0.0287
IL17B	-2.32193	0.0293
AICDA	1.978196	0.0305
CD209	-2.47393	0.0327
TIGIT	-3.47393	0.034
FCGR2A/C	-3.8365	0.036
LEF1	-3.05889	0.039
HLA-DPA1	1.778209	0.0391
KLRB1	-1.59946	0.0393
DPP4	1.819668	0.0397
CR2	-1.73697	0.0411
CIITA	-1.94342	0.0412
KLRC1	2.090853	0.0428
IL32	3.060047	0.0429
CD74	1.948601	0.044
NLRP3	-1.94342	0.0443
XCL1	-3.18442	0.0443
BCL2L11	1.350497	0.0479
CD160	2.130931	0.0487
CXCL10	3.430285	0.0499

Table S7. Angiogenes, enriched Heart not Liver

Accession	Name	Description	Biotype	Tax ID	Location	# of Detections
ENST0000(	AAAS-001	achalasia, adrenocortical insufficiency, alacrimia	protein_co	9606	12:533074	40
ENST0000(	AARS2-001	alanyl-tRNA synthetase 2, mitochondrial	protein_co	9606	6:4429965	41
ENST0000(	AASDH-001	aminoadipate-semialdehyde dehydrogenase	protein_co	9606	4:5633829	24
ENST0000(	ABCC10-007	ATP-binding cassette, sub-family C (CFTR/MRP), member 10	protein_co	9606	6:4343175	6
ENST0000(	ABCC6-001	ATP-binding cassette, sub-family C (CFTR/MRP), member 6	protein_co	9606	16:161489	11
ENST0000(	ABCF2-003	ATP-binding cassette, sub-family F (GCN20), member 2	protein_co	9606	7:1512078	21
ENST0000(	ABCG2-001	ATP-binding cassette, sub-family G (WHITE), member 2 (Junior blood group)	protein_co	9606	4:8809026	30
ENST0000(	ABHD11-001	abhydrolase domain containing 11	protein_co	9606	7:7373609	25
ENST0000(	ABHD8-001	abhydrolase domain containing 8	protein_co	9606	19:172921	33
ENST0000(	ABI3-001	ABI family, member 3	protein_co	9606	17:492102	43
ENST0000(	ACADS-001	acyl-CoA dehydrogenase, C-2 to C-3 short chain	protein_co	9606	12:120725	43
ENST0000(	ACO2-001	aconitase 2, mitochondrial	protein_co	9606	22:414691	42
ENST0000(	ACOT2-001	acyl-CoA thioesterase 2	protein_co	9606	14:735690	43
ENST0000(	ACOT8-001	acyl-CoA thioesterase 8	protein_co	9606	20:458417	40
ENST0000(	ACP5-002	acid phosphatase 5, tartrate resistant	protein_co	9606	19:115746	4
ENST0000(	ACTA2-001	actin, alpha 2, smooth muscle, aorta	protein_co	9606	10:889350	43
ENST0000(	ACTN1-001	actinin, alpha 1	protein_co	9606	14:688741	39
ENST0000(	ACVR2A-001	activin A receptor, type IIA	protein_co	9606	2:1478445	19
ENST0000(	ADCK1-001	aarF domain containing kinase 1	protein_co	9606	14:778000	38
ENST0000(	ADCK2-001	aarF domain containing kinase 2	protein_co	9606	7:1406731	25
ENST0000(	ADSL-017	adenylosuccinate lyase	protein_co	9606	22:403465	30
ENST0000(	AEBP1-001	AE binding protein 1	protein_co	9606	7:4410436	41
ENST0000(	AES-001	amino-terminal enhancer of split	protein_co	9606	19:305291	5
ENST0000(	AGO2-001	argonaute RISC catalytic component 2	protein_co	9606	8:1405311	22
ENST0000(	AGO3-002	argonaute RISC catalytic component 3	protein_co	9606	1:3593125	36
ENST0000(	AHCY-002	adenosylhomocysteinase	protein_co	9606	20:342802	44
ENST0000(	AHR-002	aryl hydrocarbon receptor	protein_co	9606	7:1729862	41
ENST0000(	AHSA1-001	AHA1, activator of heat shock 90kDa protein ATPase homolog 1 (yeast)	protein_co	9606	14:774580	40
ENST0000(	AIF1L-001	allograft inflammatory factor 1-like	protein_co	9606	9:1310965	37
ENST0000(	AIMP2-001	aminoacyl tRNA synthetase complex-interacting multifunctional protein 2	protein_co	9606	7:6009245	26
ENST0000(	AK6-003	adenylate kinase 6	protein_co	9606	5:6936474	41
ENST0000(	AKAP10-001	A kinase (PRKA) anchor protein 10	protein_co	9606	17:199043	33
ENST0000(	AKAP11-001	A kinase (PRKA) anchor protein 11	protein_co	9606	13:422721	41
ENST0000(	AKAP3-201	A kinase (PRKA) anchor protein 3	protein_co	9606	12:461550	7
ENST0000(	AKR7A2-001	aldo-keto reductase family 7, member A2 (aflatoxin aldehyde reductase)	protein_co	9606	1:1930396	44
ENST0000(	ALDH3A1-001	aldehyde dehydrogenase 3 family, member A1	protein_co	9606	17:197379	4
ENST0000(	ALDH3A2-001	aldehyde dehydrogenase 3 family, member A2	protein_co	9606	17:196485	6
ENST0000(	ALDOC-001	aldolase C, fructose-bisphosphate	protein_co	9606	17:285731	13
ENST0000(	ALG2-001	ALG2, alpha-1,3/1,6-mannosyltransferase	nonsense_	9606	9:9921642	21
ENST0000(	ALG5-001	ALG5, dolichyl-phosphate beta-glucosyltransferase	protein_co	9606	13:369498	44
ENST0000(	ALPK1-001	alpha-kinase 1	protein_co	9606	4:1122973	15
ENST0000(	ANKRD16-002	ankyrin repeat domain 16	protein_co	9606	10:587812	14
ENST0000(	AP4S1-001	adaptor-related protein complex 4, sigma 1 subunit	protein_co	9606	14:310256	11
ENST0000(	AP5S1-201	adaptor-related protein complex 5, sigma 1 subunit	protein_co	9606	20:382052	5
ENST0000(	APEX1-001	APEX nuclease (multifunctional DNA repair enzyme) 1	protein_co	9606	14:204551	26
ENST0000(	APLP1-002	amyloid beta (A4) precursor-like protein 1	protein_co	9606	19:358684	12
ENST0000(	APMAP-001	adipocyte plasma membrane associated protein	protein_co	9606	20:249629	42
ENST0000(	APOB-001	apolipoprotein B	protein_co	9606	2:2100142	9
ENST0000(	APOBEC3D-001	apolipoprotein B mRNA editing enzyme, catalytic polypeptide-like 3D	protein_co	9606	22:390211	8
ENST0000(	APPBP2-001	amyloid beta precursor protein (cytoplasmic tail) binding protein 2	protein_co	9606	17:604431	35
ENST0000(	ARHGAP33-201	Rho GTPase activating protein 33	protein_co	9606	19:357756	5
ENST0000(	ARHGDIB-001	Rho GDP dissociation inhibitor (GDI) beta	protein_co	9606	12:149420	42
ENST0000(	ARHGEF5-001	Rho guanine nucleotide exchange factor (GEF) 5	protein_co	9606	7:1443553	9
ENST0000(	ARL2BP-001	ADP-ribosylation factor-like 2 binding protein	protein_co	9606	16:572450	25
ENST0000(	ARMC2-004	armadillo repeat containing 2	protein_co	9606	6:1088486	22
ENST0000(	ARMCX5-004	armadillo repeat containing, X-linked 5	protein_co	9606	X:1025991	35
ENST0000(	ARSA-001	arylsulfatase A	protein_co	9606	22:506250	34
ENST0000(	ASIC2-001	acid-sensing (proton-gated) ion channel 2	protein_co	9606	17:330130	7
ENST0000(	ASPHD2-001	aspartate beta-hydroxylase domain containing 2	protein_co	9606	22:264292	34
ENST0000(	ATAD2B-001	ATPase family, AAA domain containing 2B	protein_co	9606	2:2374866	40
ENST0000(	ATG14-001	autophagy related 14	protein_co	9606	14:553663	39
ENST0000(	ATIC-001	5-aminoimidazole-4-carboxamide ribonucleotide formyltransferase/IMP cyclohydrolase	protein_co	9606	2:2153118	37
ENST0000(	ATP5SL-007	ATP5S-like	protein_co	9606	19:414313	37
ENST0000(	ATP6V0B-004	ATPase, H+ transporting, lysosomal 21kDa, V0 subunit b	protein_co	9606	1:4397494	30
ENST0000(	ATP6V1B1-001	ATPase, H+ transporting, lysosomal 56/58kDa, V1 subunit B1	protein_co	9606	2:7093588	12
ENST0000(	ATP6V1D-001	ATPase, H+ transporting, lysosomal 34kDa, V1 subunit D	protein_co	9606	14:673380	14
ENST0000(	ATXN1-001	ataxin 1	protein_co	9606	6:1629911	3
ENST0000(	B4GALT6-003	UDP-Gal:betaGlcNAc beta 1,4- galactosyltransferase, polypeptide 6	protein_co	9606	18:316246	12
ENST0000(	BAG6-001	BCL2-associated athanogene 6	protein_co	9606	6:3163902	4
ENST0000(	BATF3-001	basic leucine zipper transcription factor, ATF-like 3	protein_co	9606	1:2126864	32
ENST0000(	BBS2-001	Bardet-Biedl syndrome 2	protein_co	9606	16:564843	24
ENST0000(	BBS9-004	Bardet-Biedl syndrome 9	protein_co	9606	7:3312953	33
ENST0000(	BCAR1-002	breast cancer anti-estrogen resistance 1	protein_co	9606	16:752290	35
ENST0000(	BCKDK-001	branched chain ketoacid dehydrogenase kinase	protein_co	9606	16:311082	36
ENST0000(	BCL2L12-009	BCL2-like 12 (proline rich)	protein_co	9606	19:496660	33
ENST0000(	BCL6-004	B-cell CLL/lymphoma 6	protein_co	9606	3:1877223	8
ENST0000(	BCL7B-001	B-cell CLL/lymphoma 7B	protein_co	9606	7:7353635	19
ENST0000(	BCL9-001	B-cell CLL/lymphoma 9	protein_co	9606	1:1475414	16
ENST0000(	BCORL1-001	BCL6 corepressor-like 1	protein_co	9606	X:1299826	27
ENST0000(	BEST2-201	bestrophin 2	protein_co	9606	19:127525	11
ENST0000(	BET1-001	Bet1 golgi vesicular membrane trafficking protein	protein_co	9606	7:9399324	41
ENST0000(	BIRC2-001	baculoviral IAP repeat containing 2	protein_co	9606	11:102347	38

ENST0000(	BLOC1S6-001	biogenesis of lysosomal organelles complex-1, subunit 6, pallidin	protein_co	9606	15:455871	30
ENST0000(	BMP4-001	bone morphogenetic protein 4	protein_co	9606	14:539497	40
ENST0000(	BNIP1-002	BCL2/adenovirus E1B 19kDa interacting protein 1	protein_co	9606	5:1731444	5
ENST0000(	BOD1L1-001	biorientation of chromosomes in cell division 1-like 1	protein_co	9606	4:1356873	39
ENST0000(	BRD1-002	bromodomain containing 1	protein_co	9606	22:497732	4
ENST0000(	BTN3A1-003	butyrophilin, subfamily 3, member A1	retained_ir	9606	6:2640230	7
ENST0000(	BTN3A3-001	butyrophilin, subfamily 3, member A3	protein_co	9606	6:2644047	32
ENST0000(	BYSL-001	bystin-like	protein_co	9606	6:4192118	26
ENST0000(	C10orf95-201	chromosome 10 open reading frame 95	protein_co	9606	10:102449	15
ENST0000(	C11orf63-201	chromosome 11 open reading frame 63	protein_co	9606	11:122882	19
ENST0000(	C12orf5-001	chromosome 12 open reading frame 5	protein_co	9606	12:432120	43
ENST0000(	C14orf105-001	chromosome 14 open reading frame 105	protein_co	9606	14:574693	17
ENST0000(	C16orf70-001	chromosome 16 open reading frame 70	protein_co	9606	16:671099	11
ENST0000(	C17orf53-003	chromosome 17 open reading frame 53	protein_co	9606	17:441420	17
ENST0000(	C19orf43-001	chromosome 19 open reading frame 43	protein_co	9606	19:127306	43
ENST0000(	C1QBP-001	complement component 1, q subcomponent binding protein	protein_co	9606	17:543277	39
ENST0000(	C20orf27-002	chromosome 20 open reading frame 27	protein_co	9606	20:375350	37
ENST0000(	C2orf43-002	chromosome 2 open reading frame 43	protein_co	9606	2:2068505	41
ENST0000(	C3orf14-006	chromosome 3 open reading frame 14	protein_co	9606	3:6231902	30
ENST0000(	C4orf6-201	chromosome 4 open reading frame 6	protein_co	9606	4:5525156	4
ENST0000(	C5orf15-001	chromosome 5 open reading frame 15	protein_co	9606	5:1339555	42
ENST0000(	CACNG1-001	calcium channel, voltage-dependent, gamma subunit 1	protein_co	9606	17:670445	6
ENST0000(	CACTIN-202	cactin, spliceosome C complex subunit	protein_co	9606	19:361065	29
ENST0000(	CAMKK1-002	calcium/calmodulin-dependent protein kinase kinase 1, alpha	protein_co	9606	17:386515	4
ENST0000(	CAMSAP2-001	calmodulin regulated spectrin-associated protein family, member 2	protein_co	9606	1:2007397	19
ENST0000(	CANX-001	calnexin	protein_co	9606	5:1796989	39
ENST0000(	CAP2-001	CAP, adenylate cyclase-associated protein, 2 (yeast)	protein_co	9606	6:1739321	22
ENST0000(	CAPN15-001	calpain 15	protein_co	9606	16:527856	34
ENST0000(	CAT-001	catalase	protein_co	9606	11:344389	43
ENST0000(	CAV2-001	caveolin 2	protein_co	9606	7:1164993	38
ENST0000(	CBLL1-014	Cbl proto-oncogene-like 1, E3 ubiquitin protein ligase	protein_co	9606	7:1077441	37
ENST0000(	CBX1-001	chromobox homolog 1	protein_co	9606	17:480700	43
ENST0000(	CBX5-002	chromobox homolog 5	protein_co	9606	12:542309	44
ENST0000(	CBX6-003	chromobox homolog 6	protein_co	9606	22:388642	11
ENST0000(	CBX7-001	chromobox homolog 7	protein_co	9606	22:391307	9
ENST0000(	CCDC130-004	coiled-coil domain containing 130	protein_co	9606	19:137477	37
ENST0000(	CCDC169-013	coiled-coil domain containing 169	protein_co	9606	13:362271	4
ENST0000(	CCDC47-001	coiled-coil domain containing 47	protein_co	9606	17:637452	35
ENST0000(	CCDC53-001	coiled-coil domain containing 53	protein_co	9606	12:102012	34
ENST0000(	CCDC70-001	coiled-coil domain containing 70	protein_co	9606	13:518619	14
ENST0000(	CCDC86-001	coiled-coil domain containing 86	protein_co	9606	11:608420	44
ENST0000(	CCDC92-001	coiled-coil domain containing 92	protein_co	9606	12:123935	9
ENST0000(	CCL24-001	chemokine (C-C motif) ligand 24	protein_co	9606	7:7581166	14
ENST0000(	CCL26-001	chemokine (C-C motif) ligand 26	protein_co	9606	7:7576953	13
ENST0000(	CCND1-001	cyclin D1	protein_co	9606	11:696410	44
ENST0000(	CCNI-001	cyclin I	protein_co	9606	4:7704715	41
ENST0000(	CCSER2-002	coiled-coil serine-rich protein 2	protein_co	9606	10:843286	36
ENST0000(	CD4-001	CD4 molecule	protein_co	9606	12:678947	18
ENST0000(	CD79B-001	CD79b molecule, immunoglobulin-associated beta	protein_co	9606	17:639287	9
ENST0000(	CD93-002	CD93 molecule	protein_co	9606	20:230793	43
ENST0000(	CDC27-001	cell division cycle 27	protein_co	9606	17:471177	31
ENST0000(	CDC6-001	cell division cycle 6	protein_co	9606	17:402876	42
ENST0000(	CDC7-001	cell division cycle 7	protein_co	9606	1:9150089	32
ENST0000(	CDCA3-006	cell division cycle associated 3	protein_co	9606	12:684881	23
ENST0000(	CDH17-001	cadherin 17, LI cadherin (liver-intestine)	protein_co	9606	8:9412717	7
ENST0000(	CDK13-001	cyclin-dependent kinase 13	protein_co	9606	7:3995003	31
ENST0000(	CDKN1A-001	cyclin-dependent kinase inhibitor 1A (p21, Cip1)	protein_co	9606	6:3667865	42
ENST0000(	CECR5-001	cat eye syndrome chromosome region, candidate 5	protein_co	9606	22:171375	17
ENST0000(	CENPM-001	centromere protein M	protein_co	9606	22:419387	43
ENST0000(	CEP41-001	centrosomal protein 41kDa	protein_co	9606	7:1303937	21
ENST0000(	CERK-001	ceramide kinase	protein_co	9606	22:466844	43
ENST0000(	CETP-001	cholesteryl ester transfer protein, plasma	protein_co	9606	16:569618	33
ENST0000(	CFTR-001	cystic fibrosis transmembrane conductance regulator (ATP-binding cassette sub-family C, member 7)	protein_co	9606	7:1174799	3
ENST0000(	CGRRF1-001	cell growth regulator with ring finger domain 1	protein_co	9606	14:545098	33
ENST0000(	CHERP-003	calcium homeostasis endoplasmic reticulum protein	protein_co	9606	19:165178	5
ENST0000(	CHN2-001	chimerin 2	protein_co	9606	7:2919441	15
ENST0000(	CHPF2-001	chondroitin polymerizing factor 2	protein_co	9606	7:1512324	41
ENST0000(	CHPT1-001	choline phosphotransferase 1	protein_co	9606	12:101697	41
ENST0000(	CHRAC1-001	chromatin accessibility complex 1	protein_co	9606	8:1405112	41
ENST0000(	CINP-001	cyclin-dependent kinase 2 interacting protein	protein_co	9606	14:102348	40
ENST0000(	CLDN11-001	claudin 11	protein_co	9606	3:1704188	42
ENST0000(	CLDN18-002	claudin 18	protein_co	9606	3:1380100	10
ENST0000(	CLIC5-002	chloride intracellular channel 5	protein_co	9606	6:4589845	10
ENST0000(	CLIP2-201	CAP-GLY domain containing linker protein 2	protein_co	9606	7:7428947	10
ENST0000(	CMAS-001	cytidine monophosphate N-acetylneuraminic acid synthetase	protein_co	9606	12:220461	38
ENST0000(	CMTM6-001	CKLF-like MARVEL transmembrane domain containing 6	protein_co	9606	3:3248131	36
ENST0000(	CNIH1-001	cornichon family AMPA receptor auxiliary protein 1	protein_co	9606	14:544235	40
ENST0000(	COA1-201	cytochrome c oxidase assembly factor 1 homolog (S. cerevisiae)	protein_co	9606	7:4363924	35
ENST0000(	COIL-001	coilin	protein_co	9606	17:569381	42
ENST0000(	COL21A1-001	collagen, type XXI, alpha 1	protein_co	9606	6:5605659	17
ENST0000(	COPA-002	coatomer protein complex, subunit alpha	protein_co	9606	1:1602892	41
ENST0000(	COPS7A-006	COP9 signalosome subunit 7A	protein_co	9606	12:672407	22
ENST0000(	COX15-001	cytochrome c oxidase assembly homolog 15 (yeast)	protein_co	9606	10:997135	32
ENST0000(	COX6A1-001	cytochrome c oxidase subunit VIa polypeptide 1	protein_co	9606	12:120438	43

ENST0000(	COX7A2L-201	cytochrome c oxidase subunit VIIa polypeptide 2 like	protein_co	9606	2:4235050	43
ENST0000(	CPD-001	carboxypeptidase D	protein_co	9606	17:303789	40
ENST0000(	CPNE5-001	copine V	protein_co	9606	6:3674077	35
ENST0000(	CPSF3-001	cleavage and polyadenylation specific factor 3, 73kDa	protein_co	9606	2:9423568	36
ENST0000(	CPXM2-002	carboxypeptidase X (M14 family), member 2	protein_co	9606	10:123745	29
ENST0000(	CSK-001	c-src tyrosine kinase	protein_co	9606	15:747820	36
ENST0000(	CSNK2A1-002	casein kinase 2, alpha 1 polypeptide	protein_co	9606	20:481097	31
ENST0000(	CSRNP2-001	cysteine-serine-rich nuclear protein 2	protein_co	9606	12:510612	36
ENST0000(	CST4-001	cystatin S	protein_co	9606	20:236856	24
ENST0000(	CSTF1-001	cleavage stimulation factor, 3' pre-RNA, subunit 1, 50kDa	protein_co	9606	20:563923	41
ENST0000(	CTDP1-002	CTD (carboxy-terminal domain, RNA polymerase II, polypeptide A) phosphatase, subunit 1	protein_co	9606	18:796798	33
ENST0000(	CTSA-004	cathepsin A	protein_co	9606	20:458913	40
ENST0000(	CTSC-001	cathepsin C	protein_co	9606	11:882935	41
ENST0000(	CTSZ-001	cathepsin Z	protein_co	9606	20:589951	44
ENST0000(	CXCR4-001	chemokine (C-X-C motif) receptor 4	protein_co	9606	2:1361143	44
ENST0000(	CYB561D2-001	cytochrome b561 family, member D2	protein_co	9606	3:5035084	4
ENST0000(	CYP26B1-001	cytochrome P450, family 26, subfamily B, polypeptide 1	protein_co	9606	2:7212923	23
ENST0000(	CYP4F2-201	cytochrome P450, family 4, subfamily F, polypeptide 2	protein_co	9606	19:158783	2
ENST0000(	CYP4F3-001	cytochrome P450, family 4, subfamily F, polypeptide 3	protein_co	9606	19:156409	22
ENST0000(	CYP51A1-001	cytochrome P450, family 51, subfamily A, polypeptide 1	protein_co	9606	7:9211215	41
ENST0000(	DAP-001	death-associated protein	protein_co	9606	5:1067923	42
ENST0000(	DAZAP1-001	DAZ associated protein 1	protein_co	9606	19:140761	36
ENST0000(	DBNDD1-001	dysbindin (dystrobrevin binding protein 1) domain containing 1	protein_co	9606	16:900048	41
ENST0000(	DCAF10-201	DDB1 and CUL4 associated factor 10	protein_co	9606	9:3780055	22
ENST0000(	DDX1-201	DEAD (Asp-Glu-Ala-Asp) box helicase 1	protein_co	9606	2:1559162	41
ENST0000(	DDX17-003	DEAD (Asp-Glu-Ala-Asp) box helicase 17	retained_i	9606	22:384856	17
ENST0000(	DDX49-001	DEAD (Asp-Glu-Ala-Asp) box polypeptide 49	protein_co	9606	19:189196	43
ENST0000(	DECR1-001	2,4-dienoyl CoA reductase 1, mitochondrial	protein_co	9606	8:9000140	42
ENST0000(	DENND1B-004	DENN/MADD domain containing 1B	protein_co	9606	1:1975522	9
ENST0000(	DHDDS-011	dehydrodolichyl diphosphate synthase	protein_co	9606	1:2643233	8
ENST0000(	DHPS-001	deoxyhypusine synthase	protein_co	9606	19:126757	10
ENST0000(	DHRS12-001	dehydrogenase/reductase (SDR family) member 12	protein_co	9606	13:517679	8
ENST0000(	DLGAP5-001	discs, large (Drosophila) homolog-associated protein 5	protein_co	9606	14:551481	43
ENST0000(	DLL3-002	delta-like 3 (Drosophila)	protein_co	9606	19:394989	17
ENST0000(	DMC1-001	DNA meiotic recombinase 1	protein_co	9606	22:385189	11
ENST0000(	DNAJC12-001	DnaJ (Hsp40) homolog, subfamily C, member 12	protein_co	9606	10:677966	39
ENST0000(	DNASE2-001	deoxyribonuclease II, lysosomal	protein_co	9606	19:128752	43
ENST0000(	DNPH1-001	2'-deoxynucleoside 5'-phosphate N-hydrolase 1	protein_co	9606	6:4322562	43
ENST0000(	DRG2-001	developmentally regulated GTP binding protein 2	protein_co	9606	17:180878	38
ENST0000(	DUSP3-001	dual specificity phosphatase 3	protein_co	9606	17:437661	44
ENST0000(	DUSP4-001	dual specificity phosphatase 4	protein_co	9606	8:2933306	43
ENST0000(	DYNLL1-001	dynein, light chain, LC8-type 1	protein_co	9606	12:120496	43
ENST0000(	EBNA1BP2-001	EBNA1 binding protein 2	protein_co	9606	1:4316418	42
ENST0000(	EBPL-002	emopamil binding protein-like	protein_co	9606	13:496607	41
ENST0000(	ECT2-001	epithelial cell transforming 2	protein_co	9606	3:1727506	32
ENST0000(	EFNB2-001	ephrin-B2	protein_co	9606	13:106489	42
ENST0000(	EHHADH-001	enoyl-CoA, hydratase/3-hydroxyacyl CoA dehydrogenase	protein_co	9606	3:1851906	42
ENST0000(	EIF1B-001	eukaryotic translation initiation factor 1B	protein_co	9606	3:4030968	41
ENST0000(	EIF2AK1-001	eukaryotic translation initiation factor 2-alpha kinase 1	protein_co	9606	7:6022244	43
ENST0000(	EIF2AK2-001	eukaryotic translation initiation factor 2-alpha kinase 2	protein_co	9606	2:3709921	27
ENST0000(	EIF3D-001	eukaryotic translation initiation factor 3, subunit D	protein_co	9606	22:365108	33
ENST0000(	EIF3E-001	eukaryotic translation initiation factor 3, subunit E	protein_co	9606	8:1082017	40
ENST0000(	EIF3K-001	eukaryotic translation initiation factor 3, subunit K	protein_co	9606	19:386190	41
ENST0000(	EIF5-001	eukaryotic translation initiation factor 5	protein_co	9606	14:103334	9
ENST0000(	ELAVL2-002	ELAV like neuron-specific RNA binding protein 2	protein_co	9606	9:2369010	3
ENST0000(	ELF1-003	E74-like factor 1 (ets domain transcription factor)	protein_co	9606	13:409320	40
ENST0000(	ELK3-001	ELK3, ETS-domain protein (SRF accessory protein 2)	protein_co	9606	12:961943	44
ENST0000(	ELL2-001	elongation factor, RNA polymerase II, 2	protein_co	9606	5:9588509	39
ENST0000(	EMC3-001	ER membrane protein complex subunit 3	protein_co	9606	3:9962537	25
ENST0000(	EML2-007	echinoderm microtubule associated protein like 2	protein_co	9606	19:456094	33
ENST0000(	ENO1-001	enolase 1, (alpha)	protein_co	9606	1:8861002	43
ENST0000(	EPB41L1-013	erythrocyte membrane protein band 4.1-like 1	protein_co	9606	20:360915	37
ENST0000(	EPDR1-001	ependymin related 1	protein_co	9606	7:3792056	30
ENST0000(	EPOR-001	erythropoietin receptor	protein_co	9606	19:113772	42
ENST0000(	EPS8L1-006	EPS8-like 1	protein_co	9606	19:550803	21
ENST0000(	EPX-001	eosinophil peroxidase	protein_co	9606	17:581927	19
ENST0000(	ERCC1-003	excision repair cross-complementation group 1	protein_co	9606	19:454134	20
ENST0000(	ERLEC1-001	endoplasmic reticulum lectin 1	protein_co	9606	2:5378708	44
ENST0000(	ESF1-001	ESF1, nucleolar pre-rRNA processing protein, homolog (S. cerevisiae)	protein_co	9606	20:137143	39
ENST0000(	ESRRA-002	estrogen-related receptor alpha	protein_co	9606	11:643055	25
ENST0000(	EXOC2-002	exocyst complex component 2	protein_co	9606	6:485133-6	43
ENST0000(	EXTL3-001	exostosin-like glycosyltransferase 3	protein_co	9606	8:2870132	43
ENST0000(	FAAH-001	fatty acid amide hydrolase	protein_co	9606	1:4639426	39
ENST0000(	FAM117A-001	family with sequence similarity 117, member A	protein_co	9606	17:497103	24
ENST0000(	FAM118A-001	family with sequence similarity 118, member A	protein_co	9606	22:453089	6
ENST0000(	FAM124B-001	family with sequence similarity 124B	protein_co	9606	2:2243995	22
ENST0000(	FAM136A-001	family with sequence similarity 136, member A	protein_co	9606	2:7029597	42
ENST0000(	FAM149B1-001	family with sequence similarity 149, member B1	protein_co	9606	10:731681	17
ENST0000(	FAM168A-003	family with sequence similarity 168, member A	protein_co	9606	11:734004	26
ENST0000(	FAM178A-001	family with sequence similarity 178, member A	protein_co	9606	10:100912	42
ENST0000(	FAM20A-003	family with sequence similarity 20, member A	processed_	9606	17:685351	5
ENST0000(	FAM53C-001	family with sequence similarity 53, member C	protein_co	9606	5:1383380	40
ENST0000(	FAM65C-201	family with sequence similarity 65, member C	protein_co	9606	20:505861	15
ENST0000(	FAM76A-003	family with sequence similarity 76, member A	protein_co	9606	1:2772605	8

ENST0000(	FAM83D-201	family with sequence similarity 83, member D	protein_co	9606	20:389263	32
ENST0000(	FAM98A-001	family with sequence similarity 98, member A	protein_co	9606	2:3358365	41
ENST0000(	FANCE-001	Fanconi anemia, complementation group E	protein_co	9606	6:3545236	39
ENST0000(	FANCL-001	Fanconi anemia, complementation group L	protein_co	9606	2:5815924	26
ENST0000(	FAP-001	fibroblast activation protein, alpha	protein_co	9606	2:1621706	15
ENST0000(	FBL-001	fibrillarin	protein_co	9606	19:398344	37
ENST0000(	FBXL12-001	F-box and leucine-rich repeat protein 12	protein_co	9606	19:981026	36
ENST0000(	FBXL4-201	F-box and leucine-rich repeat protein 4	protein_co	9606	6:9887372	36
ENST0000(	FBXO24-001	F-box protein 24	protein_co	9606	7:1005863	5
ENST0000(	FBXO30-001	F-box protein 30	protein_co	9606	6:1457935	41
ENST0000(	FERMT1-001	fermitin family member 1	protein_co	9606	20:607484	22
ENST0000(	FGFR1OP2-001	FGFR1 oncogene partner 2	protein_co	9606	12:269383	41
ENST0000(	FIG4-001	FIG4 phosphoinositide 5-phosphatase	protein_co	9606	6:1096913	40
ENST0000(	FIS1-001	fission 1 (mitochondrial outer membrane) homolog (S. cerevisiae)	protein_co	9606	7:1012396	43
ENST0000(	FKBP14-001	FK506 binding protein 14, 22 kDa	protein_co	9606	7:3001058	42
ENST0000(	FKBP4-001	FK506 binding protein 4, 59kDa	protein_co	9606	12:279495	43
ENST0000(	FKBP9-001	FK506 binding protein 9, 63 kDa	protein_co	9606	7:3295740	42
ENST0000(	FKTN-001	fukutin	protein_co	9606	9:1055581	37
ENST0000(	FLRT1-001	fibronectin leucine rich transmembrane protein 1	protein_co	9606	11:641031	31
ENST0000(	FNTB-001	farnesyltransferase, CAAX box, beta	protein_co	9606	14:649867	39
ENST0000(	FOXJ2-001	forkhead box J2	protein_co	9606	12:803270	42
ENST0000(	FSD1-001	fibronectin type III and SPRY domain containing 1	protein_co	9606	19:430460	40
ENST0000(	FSTL3-001	follistatin-like 3 (secreted glycoprotein)	protein_co	9606	19:676365	40
ENST0000(	FTSJ2-001	FtsJ RNA methyltransferase homolog 2 (E. coli)	protein_co	9606	7:2234291	39
ENST0000(	FYTTD1-001	forty-two-three domain containing 1	protein_co	9606	3:1977497	38
ENST0000(	G2E3-001	G2/M-phase specific E3 ubiquitin protein ligase	protein_co	9606	14:305591	38
ENST0000(	GABARAPL2-001	GABA(A) receptor-associated protein-like 2	protein_co	9606	16:755663	42
ENST0000(	GALK1-002	galactokinase 1	protein_co	9606	17:757515	44
ENST0000(	GAPDH-001	glyceraldehyde-3-phosphate dehydrogenase	protein_co	9606	12:653392	19
ENST0000(	GAR1-003	GAR1 ribonucleoprotein	protein_co	9606	4:1098155	40
ENST0000(	GCDH-001	glutaryl-CoA dehydrogenase	protein_co	9606	19:128910	34
ENST0000(	GCLC-001	glutamate-cysteine ligase, catalytic subunit	protein_co	9606	6:5349734	31
ENST0000(	GDAP1-001	ganglioside induced differentiation associated protein 1	protein_co	9606	8:7435038	34
ENST0000(	GHR-001	growth hormone receptor	protein_co	9606	5:4242377	13
ENST0000(	GIT1-001	G protein-coupled receptor kinase interacting ArfGAP 1	protein_co	9606	17:295734	32
ENST0000(	GJB6-001	gap junction protein, beta 6, 30kDa	protein_co	9606	13:202219	3
ENST0000(	GLA-001	galactosidase, alpha	protein_co	9606	X:1013978	41
ENST0000(	GLCCI1-001	glucocorticoid induced transcript 1	protein_co	9606	7:7968794	18
ENST0000(	GLG1-001	golgi glycoprotein 1	protein_co	9606	16:744519	21
ENST0000(	GLRA2-001	glycine receptor, alpha 2	protein_co	9606	X:1452952	6
ENST0000(	GMIP-001	GEM interacting protein	protein_co	9606	19:196294	33
ENST0000(	GMNN-001	geminin, DNA replication inhibitor	protein_co	9606	6:2477493	40
ENST0000(	GNA11-001	guanine nucleotide binding protein (G protein), alpha 11 (Gq class)	protein_co	9606	19:309441	41
ENST0000(	GNB4-001	guanine nucleotide binding protein (G protein), beta polypeptide 4	protein_co	9606	3:1793992	39
ENST0000(	GNPNAT1-001	glucosamine-phosphate N-acetyltransferase 1	protein_co	9606	14:527751	42
ENST0000(	GNPTG-001	N-acetylglucosamine-1-phosphate transferase, gamma subunit	protein_co	9606	16:135192	17
ENST0000(	GOLGA5-001	golgin A5	protein_co	9606	14:927942	43
ENST0000(	GOLT1B-001	golgi transport 1B	protein_co	9606	12:215018	37
ENST0000(	GOPC-001	golgi-associated PDZ and coiled-coil motif containing	protein_co	9606	6:1175602	37
ENST0000(	GORASP2-001	golgi reassembly stacking protein 2, 55kDa	protein_co	9606	2:1709285	17
ENST0000(	GOT2-001	glutamic-oxaloacetic transaminase 2, mitochondrial	protein_co	9606	16:587071	43
ENST0000(	GPN3-001	GPN-loop GTPase 3	protein_co	9606	12:110452	42
ENST0000(	GPR63-001	G protein-coupled receptor 63	protein_co	9606	6:9679412	36
ENST0000(	GPRC5A-001	G protein-coupled receptor, class C, group 5, member A	protein_co	9606	12:128907	40
ENST0000(	GRM6-201	glutamate receptor, metabotropic 6	protein_co	9606	5:1789783	32
ENST0000(	GRN-001	granulin	protein_co	9606	17:443452	42
ENST0000(	GS1-115G20.2-001		processed_	9606	1:1845665	11
ENST0000(	GSK3A-001	glycogen synthase kinase 3 alpha	protein_co	9606	19:422301	29
ENST0000(	GSR-001	glutathione reductase	protein_co	9606	8:3067806	29
ENST0000(	GSS-001	glutathione synthetase	protein_co	9606	20:349284	44
ENST0000(	GSTM2-002	glutathione S-transferase mu 2 (muscle)	protein_co	9606	1:1096680	20
ENST0000(	GSTZ1-001	glutathione S-transferase zeta 1	protein_co	9606	14:773208	19
ENST0000(	GTF2H3-002	general transcription factor IIH, polypeptide 3, 34kDa	protein_co	9606	12:123633	5
ENST0000(	GTPBP10-001	GTP-binding protein 10 (putative)	protein_co	9606	7:9034667	14
ENST0000(	GTPBP1-001	GTP binding protein 1	protein_co	9606	22:387057	27
ENST0000(	GUCY2F-001	guanylate cyclase 2F, retinal	protein_co	9606	X:1093729	20
ENST0000(	H2AFV-001	H2A histone family, member V	protein_co	9606	7:4482679	29
ENST0000(	H3F3AP6-001	H3 histone, family 3A, pseudogene 6	processed_	9606	4:1396981	30
ENST0000(	HARS2-001	histidyl-tRNA synthetase 2, mitochondrial	protein_co	9606	5:1406914	30
ENST0000(	HAS1-005	hyaluronan synthase 1	protein_co	9606	19:517131	9
ENST0000(	HAUS4-003	HAUS augmin-like complex, subunit 4	protein_co	9606	14:229462	8
ENST0000(	HBP1-001	HMG-box transcription factor 1	protein_co	9606	7:1071690	13
ENST0000(	HCFC2-001	host cell factor C2	protein_co	9606	12:104064	40
ENST0000(	HDAC10-001	histone deacetylase 10	protein_co	9606	22:502451	19
ENST0000(	HDAC7-001	histone deacetylase 7	protein_co	9606	12:477827	37
ENST0000(	HEATR6-001	HEAT repeat containing 6	protein_co	9606	17:600431	39
ENST0000(	HELLS-201	helicase, lymphoid-specific	protein_co	9606	10:945014	6
ENST0000(	HGF-001	hepatocyte growth factor (hepapoietin A; scatter factor)	protein_co	9606	7:8169900	26
ENST0000(	HIF3A-007	hypoxia inducible factor 3, alpha subunit	protein_co	9606	19:462983	9
ENST0000(	HIST1H1A-002	histone cluster 1, H1a	protein_co	9606	6:2601708	22
ENST0000(	HIST1H1D-001	histone cluster 1, H1d	protein_co	9606	6:2623426	22
ENST0000(	HIST1H4F-001	histone cluster 1, H4f	protein_co	9606	6:2624042	21
ENST0000(	HMGXB4-002	HMG box domain containing 4	protein_co	9606	22:352574	37
ENST0000(	HN1L-001	hematological and neurological expressed 1-like	protein_co	9606	16:167825	40

ENST0000(	HNRNPL-001	heterogeneous nuclear ribonucleoprotein L	protein_co	9606	19:388363	18
ENST0000(	HOXA2-001	homeobox A2	protein_co	9606	7:2710035	33
ENST0000(	HP-201	haptoglobin	protein_co	9606	16:720546	4
ENST0000(	HSCB-001	HscB mitochondrial iron-sulfur cluster co-chaperone	protein_co	9606	22:287420	27
ENST0000(	HSD17B10-001	hydroxysteroid (17-beta) dehydrogenase 10	protein_co	9606	X:5343125	43
ENST0000(	HSDL1-001	hydroxysteroid dehydrogenase like 1	protein_co	9606	16:841222	43
ENST0000(	HSP90AA1-002	heat shock protein 90kDa alpha (cytosolic), class A member 1	protein_co	9606	14:102080	43
ENST0000(	HSPA8-006	heat shock 70kDa protein 8	protein_co	9606	11:123057	38
ENST0000(	HSPB11-001	heat shock protein family B (small), member 11	protein_co	9606	1:5392156	41
ENST0000(	HSPE1-001	heat shock 10kDa protein 1	protein_co	9606	2:1974999	21
ENST0000(	HTATSF1-001	HIV-1 Tat specific factor 1	protein_co	9606	X:1364975	38
ENST0000(	HUWE1-007	HECT, UBA and WWE domain containing 1, E3 ubiquitin protein ligase	processed_	9606	X:5358421	5
ENST0000(	HVCN1-003	hydrogen voltage-gated channel 1	protein_co	9606	12:110648	19
ENST0000(	IDH3G-005	isocitrate dehydrogenase 3 (NAD+) gamma	protein_co	9606	X:1537857	26
ENST0000(	IFI35-007	interferon-induced protein 35	retained_ii	9606	17:430067	4
ENST0000(	IFRD1-001	interferon-related developmental regulator 1	protein_co	9606	7:1124231	10
ENST0000(	IFT81-003	intraflagellar transport 81	protein_co	9606	12:110124	24
ENST0000(	IL12B-001	interleukin 12B	protein_co	9606	5:1593147	8
ENST0000(	IL1RAP-001	interleukin 1 receptor accessory protein	protein_co	9606	3:1905140	26
ENST0000(	IL23A-001	interleukin 23, alpha subunit p19	protein_co	9606	12:563388	36
ENST0000(	IL32-010	interleukin 32	protein_co	9606	16:306563	10
ENST0000(	INHBA-001	inhibin, beta A	protein_co	9606	7:4168511	33
ENST0000(	INSIG2-001	insulin induced gene 2	protein_co	9606	2:1180884	41
ENST0000(	IRF1-001	interferon regulatory factor 1	protein_co	9606	5:1324816	34
ENST0000(	ISOC1-001	isochorismatase domain containing 1	protein_co	9606	5:1290947	42
ENST0000(	ITGB8-001	integrin, beta 8	protein_co	9606	7:2033112	41
ENST0000(	ITIH6-001	inter-alpha-trypsin inhibitor heavy chain family, member 6	protein_co	9606	X:5474889	8
ENST0000(	JADE1-201	jade family PHD finger 1	protein_co	9606	4:1288096	16
ENST0000(	JOSD1-001	Josephin domain containing 1	protein_co	9606	22:386855	41
ENST0000(	KALRN-005	kalirin, RhoGEF kinase	protein_co	9606	3:1240946	11
ENST0000(	KCNIP2-002	Kv channel interacting protein 2	protein_co	9606	10:101827	4
ENST0000(	KCNJ2-002	potassium inwardly-rectifying channel, subfamily J, member 2	protein_co	9606	17:701695	40
ENST0000(	KCTD10-001	potassium channel tetramerization domain containing 10	protein_co	9606	12:109448	21
ENST0000(	KCTD9-001	potassium channel tetramerization domain containing 9	protein_co	9606	8:2542784	38
ENST0000(	KDM5B-002	lysine (K)-specific demethylase 5B	protein_co	9606	1:2027299	14
ENST0000(	KEAP1-001	kelch-like ECH-associated protein 1	protein_co	9606	19:104861	16
ENST0000(	KIAA0141-003	KIAA0141	protein_co	9606	5:1419238	37
ENST0000(	KIAA0922-008	KIAA0922	protein_co	9606	4:1535558	19
ENST0000(	KIAA1467-001	KIAA1467	protein_co	9606	12:130442	40
ENST0000(	KIF22-001	kinesin family member 22	protein_co	9606	16:297907	44
ENST0000(	KIF6-007	kinesin family member 6	protein_co	9606	6:3933615	6
ENST0000(	KLC1-011	kinesin light chain 1	protein_co	9606	14:103629	6
ENST0000(	KLHL18-001	kelch-like family member 18	protein_co	9606	3:4728294	42
ENST0000(	KLHL20-001	kelch-like family member 20	protein_co	9606	1:1737149	41
ENST0000(	KLHL2-001	kelch-like family member 2	protein_co	9606	4:1652076	35
ENST0000(	KNOP1-001	lysine-rich nucleolar protein 1	protein_co	9606	16:197019	26
ENST0000(	KRR1-001	KRR1, small subunit (SSU) processome component, homolog (yeast)	protein_co	9606	12:754908	37
ENST0000(	KRT14-001	keratin 14	protein_co	9606	17:415822	16
ENST0000(	KRT20-001	keratin 20	protein_co	9606	17:408759	2
ENST0000(	KXD1-001	KxDL motif containing 1	protein_co	9606	19:185577	43
ENST0000(	L3HYPDH-003	L-3-hydroxyproline dehydratase (trans-)	protein_co	9606	14:594726	38
ENST0000(	L3MBTL2-001	l(3)mbt-like 2 (Drosophila)	protein_co	9606	22:412052	41
ENST0000(	LAG3-001	lymphocyte-activation gene 3	protein_co	9606	12:677252	17
ENST0000(	LAMA4-001	laminin, alpha 4	protein_co	9606	6:1121087	22
ENST0000(	LAMB1-001	laminin, beta 1	protein_co	9606	7:1079238	37
ENST0000(	LAMB4-001	laminin, beta 4	protein_co	9606	7:1080235	8
ENST0000(	LAMP2-001	lysosomal-associated membrane protein 2	protein_co	9606	X:1204308	42
ENST0000(	LGALS1-001	lectin, galactoside-binding, soluble, 1	protein_co	9606	22:376756	43
ENST0000(	LGALSL-001	lectin, galactoside-binding-like	protein_co	9606	2:6445409	25
ENST0000(	LIPE-001	lipase, hormone-sensitive	protein_co	9606	19:424015	10
ENST0000(	LMF2-001	lipase maturation factor 2	protein_co	9606	22:505029	4
ENST0000(	LNPEP-001	leucyl/cystinyl aminopeptidase	protein_co	9606	5:9693546	40
ENST0000(	LOX-001	lysyl oxidase	protein_co	9606	5:1220631	42
ENST0000(	LRP1-001	low density lipoprotein receptor-related protein 1	protein_co	9606	12:571284	16
ENST0000(	LRP11-001	low density lipoprotein receptor-related protein 11	protein_co	9606	6:1498187	41
ENST0000(	LRRC59-001	leucine rich repeat containing 59	protein_co	9606	17:503812	44
ENST0000(	LRRC7-001	leucine rich repeat containing 7	protein_co	9606	1:6976017	2
ENST0000(	LRRFIP1-001	leucine rich repeat (in FLII) interacting protein 1	protein_co	9606	2:2376922	10
ENST0000(	LST1-003	leukocyte specific transcript 1	protein_co	9606	6:3158684	6
ENST0000(	LTBP4-002	latent transforming growth factor beta binding protein 4	protein_co	9606	19:405931	10
ENST0000(	LZTR1-001	leucine-zipper-like transcription regulator 1	protein_co	9606	22:209820	24
ENST0000(	M6PR-001	mannose-6-phosphate receptor (cation dependent)	protein_co	9606	12:894036	25
ENST0000(	MAGED2-201	melanoma antigen family D, 2	protein_co	9606	X:5480833	4
ENST0000(	MANBA-001	mannosidase, beta A, lysosomal	protein_co	9606	4:1026318	37
ENST0000(	MAP2-003	microtubule-associated protein 2	protein_co	9606	2:2094240	23
ENST0000(	MAP2K5-001	mitogen-activated protein kinase kinase 5	protein_co	9606	15:675427	43
ENST0000(	MAP4K5-001	mitogen-activated protein kinase kinase kinase kinase 5	protein_co	9606	14:504185	33
ENST0000(	MAPK1-001	mitogen-activated protein kinase 1	protein_co	9606	22:217545	34
ENST0000(	MAPK14-001	mitogen-activated protein kinase 14	protein_co	9606	6:3602771	16
ENST0000(	MAPK14-002	mitogen-activated protein kinase 14	protein_co	9606	6:3602777	40
ENST0000(	MARK3-005	MAP/microtubule affinity-regulating kinase 3	protein_co	9606	14:103385	29
ENST0000(	MASP1-002	mannan-binding lectin serine peptidase 1 (C4/C2 activating component of Ra-reactive factor)	protein_co	9606	3:1872463	8
ENST0000(	MCM3-006	minichromosome maintenance complex component 3	protein_co	9606	6:5226400	36
ENST0000(	MCM5-001	minichromosome maintenance complex component 5	protein_co	9606	22:354000	19

ENST0000(	MDFI-001	MyoD family inhibitor	protein_co	9606	6:4163844	25
ENST0000(	MDH1-001	malate dehydrogenase 1, NAD (soluble)	protein_co	9606	2:6358860	41
ENST0000(	MED28-001	mediator complex subunit 28	protein_co	9606	4:1761463	36
ENST0000(	MED31-001	mediator complex subunit 31	protein_co	9606	17:664331	14
ENST0000(	MEST-001	mesoderm specific transcript	protein_co	9606	7:1304920	32
ENST0000(	METTL25-001	methyltransferase like 25	protein_co	9606	12:823584	31
ENST0000(	MFN2-001	mitofusin 2	protein_co	9606	1:1198031	44
ENST0000(	MGA-201	MGA, MAX dimerization protein	protein_co	9606	15:416604	31
ENST0000(	MGP-004	matrix Gla protein	protein_co	9606	12:148818	24
ENST0000(	MGST1-201	microsomal glutathione S-transferase 1	protein_co	9606	12:163471	3
ENST0000(	MICALL1-001	MICAL-like 1	protein_co	9606	22:379062	41
ENST0000(	MKRN2-001	makorin ring finger protein 2	protein_co	9606	3:1255701	38
ENST0000(	MLEC-001	malectin	protein_co	9606	12:120686	34
ENST0000(	MLH1-001	mutL homolog 1	protein_co	9606	3:3699333	37
ENST0000(	MMP2-001	matrix metallopeptidase 2 (gelatinase A, 72kDa gelatinase, 72kDa type IV collagenase)	protein_co	9606	16:554789	42
ENST0000(	MND1-001	meiotic nuclear divisions 1 homolog (S. cerevisiae)	protein_co	9606	4:1533446	33
ENST0000(	MNT-001	MAX network transcriptional repressor	protein_co	9606	17:238406	39
ENST0000(	MOCS3-001	molybdenum cofactor synthesis 3	protein_co	9606	20:509588	44
ENST0000(	MOGS-001	mannosyl-oligosaccharide glucosidase	protein_co	9606	2:7446105	39
ENST0000(	MON1B-001	MON1 secretory trafficking family member B	protein_co	9606	16:771909	40
ENST0000(	MORC2-002	MORC family CW-type zinc finger 2	protein_co	9606	22:309266	15
ENST0000(	MOSPD3-001	motile sperm domain containing 3	protein_co	9606	7:1006121	12
ENST0000(	MPP6-001	membrane protein, palmitoylated 6 (MAGUK p55 subfamily member 6)	protein_co	9606	7:2457334	33
ENST0000(	MRI1-002	methylthioribose-1-phosphate isomerase 1	protein_co	9606	19:137645	22
ENST0000(	MRPL27-001	mitochondrial ribosomal protein L27	protein_co	9606	17:503678	42
ENST0000(	MRPL28-006	mitochondrial ribosomal protein L28	protein_co	9606	16:367384	44
ENST0000(	MRPL32-001	mitochondrial ribosomal protein L32	protein_co	9606	7:4293220	36
ENST0000(	MRPL51-001	mitochondrial ribosomal protein L51	protein_co	9606	12:649188	38
ENST0000(	MRPS10-001	mitochondrial ribosomal protein S10	protein_co	9606	6:4220680	43
ENST0000(	MRPS2-201	mitochondrial ribosomal protein S2	protein_co	9606	9:1355006	42
ENST0000(	MRPS30-003	mitochondrial ribosomal protein S30	processed_	9606	5:4481521	13
ENST0000(	MRPS34-002	mitochondrial ribosomal protein S34	protein_co	9606	16:177189	8
ENST0000(	MRPS35-001	mitochondrial ribosomal protein S35	protein_co	9606	12:277107	42
ENST0000(	MRPS7-001	mitochondrial ribosomal protein S7	protein_co	9606	17:752616	39
ENST0000(	MSANTD2-001	Myb/SANT-like DNA-binding domain containing 2	protein_co	9606	11:124766	29
ENST0000(	MSH2-001	mutS homolog 2	protein_co	9606	2:4740296	32
ENST0000(	MT2A-001	metallothionein 2A	protein_co	9606	16:566081	42
ENST0000(	MTFMT-001	mitochondrial methionyl-tRNA formyltransferase	protein_co	9606	15:650015	27
ENST0000(	MTHFD1-019	methylenetetrahydrofolate dehydrogenase (NADP+ dependent) 1, methenyltetrahydrofolate cyclohydrolase, formyltetrahydrofolate syn	protein_co	9606	14:643883	40
ENST0000(	MTMR9-001	myotubularin related protein 9	protein_co	9606	8:1128441	39
ENST0000(	MTRF1-008	mitochondrial translational release factor 1	nonsense_	9606	13:412522	9
ENST0000(	MVK-001	mevalonate kinase	protein_co	9606	12:109573	40
ENST0000(	MYBL2-001	v-myb avian myeloblastosis viral oncogene homolog-like 2	protein_co	9606	20:436671	43
ENST0000(	MYF6-001	myogenic factor 6 (herculin)	protein_co	9606	12:807074	3
ENST0000(	MYH9-001	myosin, heavy chain 9, non-muscle	protein_co	9606	22:362812	42
ENST0000(	MYL12A-001	myosin, light chain 12A, regulatory, non-sarcomeric	protein_co	9606	18:324753	22
ENST0000(	MYL12B-001	myosin, light chain 12B, regulatory	protein_co	9606	18:326190	44
ENST0000(	MZF1-001	myeloid zinc finger 1	protein_co	9606	19:585619	13
ENST0000(	NAA50-001	N(alpha)-acetyltransferase 50, NatE catalytic subunit	protein_co	9606	3:1137164	24
ENST0000(	NAGK-032	N-acetylglucosamine kinase	protein_co	9606	2:7106862	35
ENST0000(	NAGLU-001	N-acetylglucosaminidase, alpha	protein_co	9606	17:425361	40
ENST0000(	NAMPT-001	nicotinamide phosphoribosyltransferase	protein_co	9606	7:1062482	41
ENST0000(	NANS-001	N-acetylneuraminic acid synthase	protein_co	9606	9:9805673	41
ENST0000(	NCAPH-001	non-SMC condensin I complex, subunit H	protein_co	9606	2:9633578	36
ENST0000(	NCK2-001	NCK adaptor protein 2	protein_co	9606	2:1057448	32
ENST0000(	NCLN-001	nicalin	protein_co	9606	19:318560	42
ENST0000(	NCOA7-012	nuclear receptor coactivator 7	protein_co	9606	6:1257909	31
ENST0000(	NDFIP2-001	Nedd4 family interacting protein 2	protein_co	9606	13:794811	38
ENST0000(	NDUFAB1-001	NADH dehydrogenase (ubiquinone) 1, alpha/beta subcomplex, 1, 8kDa	protein_co	9606	16:235810	43
ENST0000(	NDUFB2-001	NADH dehydrogenase (ubiquinone) 1 beta subcomplex, 2, 8kDa	protein_co	9606	7:1406966	34
ENST0000(	NDUFB3-001	NADH dehydrogenase (ubiquinone) 1 beta subcomplex, 3, 12kDa	protein_co	9606	2:2010717	37
ENST0000(	NDUFB7-001	NADH dehydrogenase (ubiquinone) 1 beta subcomplex, 7, 18kDa	protein_co	9606	19:145660	44
ENST0000(	NDUFS1-001	NADH dehydrogenase (ubiquinone) Fe-S protein 1, 75kDa (NADH-coenzyme Q reductase)	protein_co	9606	2:2061148	19
ENST0000(	NECAB3-010	N-terminal EF-hand calcium binding protein 3	protein_co	9606	20:336570	20
ENST0000(	NEK9-001	NIMA-related kinase 9	protein_co	9606	14:750793	14
ENST0000(	NFE2L3-001	nuclear factor, erythroid 2-like 3	protein_co	9606	7:2615224	40
ENST0000(	NFKB1-001	nuclear factor of kappa light polypeptide gene enhancer in B-cells 1	protein_co	9606	4:1025013	23
ENST0000(	NFYB-001	nuclear transcription factor Y, beta	protein_co	9606	12:104117	40
ENST0000(	NHP2L1-003	NHP2 non-histone chromosome protein 2-like 1 (S. cerevisiae)	protein_co	9606	22:416739	36
ENST0000(	NIN-201	ninein (GSK3B interacting protein)	protein_co	9606	14:507197	38
ENST0000(	NIPAL3-001	NIPA-like domain containing 3	protein_co	9606	1:2441580	13
ENST0000(	NIPSNAP1-001	nipsnap homolog 1 (C. elegans)	protein_co	9606	22:295548	42
ENST0000(	NKTR-001	natural killer cell triggering receptor	protein_co	9606	3:4260061	31
ENST0000(	NME3-001	NME/NM23 nucleoside diphosphate kinase 3	protein_co	9606	16:177028	16
ENST0000(	NME4-001	NME/NM23 nucleoside diphosphate kinase 4	protein_co	9606	16:397209	44
ENST0000(	NOD1-001	nucleotide-binding oligomerization domain containing 1	protein_co	9606	7:3042452	38
ENST0000(	NR5A2-003	nuclear receptor subfamily 5, group A, member 2	protein_co	9606	1:2000276	18
ENST0000(	NRBP1-001	nuclear receptor binding protein 1	protein_co	9606	2:2742779	18
ENST0000(	NRF1-001	nuclear respiratory factor 1	protein_co	9606	7:1296117	33
ENST0000(	NRK-001	Nik related kinase	protein_co	9606	X:1058225	38
ENST0000(	NSMAF-001	neutral sphingomyelinase (N-SMase) activation associated factor	protein_co	9606	8:5858350	40
ENST0000(	NSRP1-001	nuclear speckle splicing regulatory protein 1	protein_co	9606	17:301167	35
ENST0000(	NT5C3A-001	5'-nucleotidase, cytosolic IIIA	protein_co	9606	7:3301414	7
ENST0000(	NUDCD1-001	NudC domain containing 1	protein_co	9606	8:1092409	18

ENST0000(	NUFIP2-001	nuclear fragile X mental retardation protein interacting protein 2	protein_co	9606	17:292558	44
ENST0000(	NUP155-001	nucleoporin 155kDa	protein_co	9606	5:3728813	39
ENST0000(	NUP85-001	nucleoporin 85kDa	protein_co	9606	17:752056	34
ENST0000(	NUTF2-001	nuclear transport factor 2	protein_co	9606	16:678467	32
ENST0000(	OBFC1-001	oligonucleotide/oligosaccharide-binding fold containing 1	protein_co	9606	10:103882	39
ENST0000(	OCEL1-001	occludin/ELL domain containing 1	protein_co	9606	19:172262	30
ENST0000(	ODC1-001	ornithine decarboxylase 1	protein_co	9606	2:1043996	35
ENST0000(	OGDH-001	oxoglutarate (alpha-ketoglutarate) dehydrogenase (lipoamide)	protein_co	9606	7:4460663	39
ENST0000(	OIP5-001	Opa interacting protein 5	protein_co	9606	15:413092	13
ENST0000(	OMG-001	oligodendrocyte myelin glycoprotein	protein_co	9606	17:312946	14
ENST0000(	OR1E2-001	olfactory receptor, family 1, subfamily E, member 2	protein_co	9606	17:343287	3
ENST0000(	ORC2-001	origin recognition complex, subunit 2	protein_co	9606	2:2009089	37
ENST0000(	ORC6-001	origin recognition complex, subunit 6	protein_co	9606	16:466896	36
ENST0000(	OSBPL6-001	oxysterol binding protein-like 6	protein_co	9606	2:1781946	10
ENST0000(	OSBPL7-001	oxysterol binding protein-like 7	protein_co	9606	17:478073	37
ENST0000(	OSCP1-002	organic solute carrier partner 1	protein_co	9606	1:3641790	40
ENST0000(	OSGEP-001	O-sialoglycoprotein endopeptidase	protein_co	9606	14:204466	36
ENST0000(	OSTM1-001	osteopetrosis associated transmembrane protein 1	protein_co	9606	6:1080414	35
ENST0000(	OTUD4P1-001	OTUD4 pseudogene 1	processed_	9606	12:383945	22
ENST0000(	OXCT1-001	3-oxoacid CoA transferase 1	protein_co	9606	5:4173006	40
ENST0000(	OXT-001	oxytocin/neurophysin I prepropeptide	protein_co	9606	20:307162	17
ENST0000(	P4HA2-003	prolyl 4-hydroxylase, alpha polypeptide II	protein_co	9606	5:1321926	25
ENST0000(	PABPC1L-017	poly(A) binding protein, cytoplasmic 1-like	protein_co	9606	20:449100	23
ENST0000(	PANK3-001	pantothenate kinase 3	protein_co	9606	5:1685484	43
ENST0000(	PANX1-001	pannexin 1	protein_co	9606	11:941289	42
ENST0000(	PAPOLA-001	poly(A) polymerase alpha	protein_co	9606	14:965023	43
ENST0000(	PAPOLG-001	poly(A) polymerase gamma	protein_co	9606	2:6075623	42
ENST0000(	PASK-001	PAS domain containing serine/threonine kinase	protein_co	9606	2:2411060	13
ENST0000(	PATZ1-003	POZ (BTB) and AT hook containing zinc finger 1	protein_co	9606	22:313406	42
ENST0000(	PCDHB15-001	protocadherin beta 15	protein_co	9606	5:1412453	32
ENST0000(	PCDHB5-001	protocadherin beta 5	protein_co	9606	5:1411352	32
ENST0000(	PCDHB7-001	protocadherin beta 7	protein_co	9606	5:1411726	27
ENST0000(	PCDHB8-001	protocadherin beta 8	protein_co	9606	5:1411777	11
ENST0000(	PCK2-002	phosphoenolpyruvate carboxykinase 2 (mitochondrial)	protein_co	9606	14:240941	40
ENST0000(	PDCD2-009	programmed cell death 2	nonsense_	9606	6:1705774	33
ENST0000(	PDHX-001	pyruvate dehydrogenase complex, component X	protein_co	9606	11:349165	38
ENST0000(	PDK4-001	pyruvate dehydrogenase kinase, isozyme 4	protein_co	9606	7:9558349	39
ENST0000(	PDRG1-001	p53 and DNA-damage regulated 1	protein_co	9606	20:319443	41
ENST0000(	PDXP-001	pyridoxal (pyridoxine, vitamin B6) phosphatase	protein_co	9606	22:376587	39
ENST0000(	PDZD11-007	PDZ domain containing 11	protein_co	9606	X:7028659	43
ENST0000(	PEPD-001	peptidase D	protein_co	9606	19:333869	42
ENST0000(	PEX16-002	peroxisomal biogenesis factor 16	protein_co	9606	11:459096	22
ENST0000(	PFKFB4-001	6-phosphofructo-2-kinase/fructose-2,6-biphosphatase 4	protein_co	9606	3:4851768	35
ENST0000(	PFN2-001	profilin 2	protein_co	9606	3:1499649	6
ENST0000(	PGF-002	placental growth factor	protein_co	9606	14:749418	29
ENST0000(	PGRMC1-001	progesterone receptor membrane component 1	protein_co	9606	X:1192362	43
ENST0000(	PIGH-001	phosphatidylinositol glycan anchor biosynthesis, class H	protein_co	9606	14:675893	41
ENST0000(	PIGQ-002	phosphatidylinositol glycan anchor biosynthesis, class Q	protein_co	9606	16:570018	31
ENST0000(	PIGU-001	phosphatidylinositol glycan anchor biosynthesis, class U	protein_co	9606	20:345605	41
ENST0000(	PIGV-001	phosphatidylinositol glycan anchor biosynthesis, class V	protein_co	9606	1:2678799	34
ENST0000(	PIGZ-003	phosphatidylinositol glycan anchor biosynthesis, class Z	retained_ir	9606	3:1969515	12
ENST0000(	PIK3R2-001	phosphoinositide-3-kinase, regulatory subunit 2 (beta)	protein_co	9606	19:181531	41
ENST0000(	PIN4-002	protein (peptidylprolyl cis/trans isomerase) NIMA-interacting, 4 (parvulin)	protein_co	9606	X:7218167	7
ENST0000(	PITHD1-001	PITH (C-terminal proteasome-interacting domain of thioredoxin-like) domain containing 1	protein_co	9606	1:2377840	43
ENST0000(	PITRM1-007	pitrilysin metallopeptidase 1	protein_co	9606	10:313773	41
ENST0000(	PKD2-001	polycystic kidney disease 2 (autosomal dominant)	protein_co	9606	4:8800766	39
ENST0000(	PKN1-001	protein kinase N1	protein_co	9606	19:144333	41
ENST0000(	PLA2G12A-001	phospholipase A2, group XIIA	protein_co	9606	4:1097099	40
ENST0000(	PLA2G15-001	phospholipase A2, group XV	protein_co	9606	16:682453	41
ENST0000(	PLBD1-001	phospholipase B domain containing 1	protein_co	9606	12:145036	31
ENST0000(	PLEK2-001	pleckstrin 2	protein_co	9606	14:673869	38
ENST0000(	PLEKHA3-001	pleckstrin homology domain containing, family A (phosphoinositide binding specific) member 3	protein_co	9606	2:1784804	43
ENST0000(	PLEKHB2-001	pleckstrin homology domain containing, family B (evectins) member 2	protein_co	9606	2:1311049	41
ENST0000(	PLGRKT-001	plasminogen receptor, C-terminal lysine transmembrane protein	protein_co	9606	9:5357973	40
ENST0000(	PLIN3-001	perilipin 3	protein_co	9606	19:483834	44
ENST0000(	PLOD1-001	procollagen-lysine, 2-oxoglutarate 5-dioxygenase 1	protein_co	9606	1:1193475	44
ENST0000(	PLOD3-001	procollagen-lysine, 2-oxoglutarate 5-dioxygenase 3	protein_co	9606	7:1012059	39
ENST0000(	PNPLA6-001	patatin-like phospholipase domain containing 6	protein_co	9606	19:753400	9
ENST0000(	PNPO-001	pyridoxamine 5'-phosphate oxidase	protein_co	9606	17:479415	13
ENST0000(	POLE2-001	polymerase (DNA directed), epsilon 2, accessory subunit	protein_co	9606	14:496435	24
ENST0000(	POLE4-006	polymerase (DNA-directed), epsilon 4, accessory subunit	processed_	9606	2:7495879	6
ENST0000(	POLK-001	polymerase (DNA directed) kappa	protein_co	9606	5:7551175	13
ENST0000(	POLR2E-012	polymerase (RNA) II (DNA directed) polypeptide E, 25kDa	nonsense_	9606	19:108816	31
ENST0000(	POLR3B-001	polymerase (RNA) III (DNA directed) polypeptide B	protein_co	9606	12:106357	40
ENST0000(	PON2-002	paraoxonase 2	protein_co	9606	7:9540489	25
ENST0000(	PPFIBP1-001	PTPRF interacting protein, binding protein 1 (liprin beta 1)	protein_co	9606	12:275242	33
ENST0000(	PPIF-001	peptidylprolyl isomerase F	protein_co	9606	10:793474	43
ENST0000(	PPM1H-001	protein phosphatase, Mg2+/Mn2+ dependent, 1H	protein_co	9606	12:626439	38
ENST0000(	PPP1R13B-001	protein phosphatase 1, regulatory subunit 13B	protein_co	9606	14:103733	31
ENST0000(	PPP2CB-001	protein phosphatase 2, catalytic subunit, beta isozyme	protein_co	9606	8:3078561	38
ENST0000(	PPP3CC-002	protein phosphatase 3, catalytic subunit, gamma isozyme	protein_co	9606	8:2244108	34
ENST0000(	PPP4R1L-005	protein phosphatase 4, regulatory subunit 1-like (pseudogene)	processed_	9606	20:582457	31
ENST0000(	PPP5C-001	protein phosphatase 5, catalytic subunit	protein_co	9606	19:463469	42
ENST0000(	PRAMEF2-001	PRAME family member 2	protein_co	9606	1:1285708	2

ENST0000(	PRDM4-001	PR domain containing 4	protein_co	9606	12:107732	42
ENST0000(	PRKAB1-001	protein kinase, AMP-activated, beta 1 non-catalytic subunit	protein_co	9606	12:119667	41
ENST0000(	PRKAG3-001	protein kinase, AMP-activated, gamma 3 non-catalytic subunit	nonsense_	9606	2:2188223	7
ENST0000(	PRKD3-201	protein kinase D3	protein_co	9606	2:3725050	29
ENST0000(	PRLHR-001	prolactin releasing hormone receptor	protein_co	9606	10:118589	27
ENST0000(	PROCR-001	protein C receptor, endothelial	protein_co	9606	20:351720	44
ENST0000(	PRPF19-001	pre-mRNA processing factor 19	protein_co	9606	11:608907	42
ENST0000(	PRRG2-001	proline rich Gla (G-carboxyglutamic acid) 2	protein_co	9606	19:495813	24
ENST0000(	PRRX1-002	paired related homeobox 1	protein_co	9606	1:1706639	22
ENST0000(	PRSS21-001	protease, serine, 21 (testisin)	protein_co	9606	16:281722	21
ENST0000(	PSD4-001	pleckstrin and Sec7 domain containing 4	protein_co	9606	2:1131739	9
ENST0000(	PSG4-003	pregnancy specific beta-1-glycoprotein 4	protein_co	9606	19:431927	5
ENST0000(	PSMA2-001	proteasome (prosome, macropain) subunit, alpha type, 2	protein_co	9606	7:4291685	41
ENST0000(	PSMA3-001	proteasome (prosome, macropain) subunit, alpha type, 3	protein_co	9606	14:582448	42
ENST0000(	PSMC4-001	proteasome (prosome, macropain) 26S subunit, ATPase, 4	protein_co	9606	19:399710	30
ENST0000(	PSMD10-001	proteasome (prosome, macropain) 26S subunit, non-ATPase, 10	protein_co	9606	X:1080842	40
ENST0000(	PSMD5-001	proteasome (prosome, macropain) 26S subunit, non-ATPase, 5	protein_co	9606	9:1208160	42
ENST0000(	PSMD7-001	proteasome (prosome, macropain) 26S subunit, non-ATPase, 7	protein_co	9606	16:742967	16
ENST0000(	PSME1-001	proteasome (prosome, macropain) activator subunit 1 (PA28 alpha)	protein_co	9606	14:241361	44
ENST0000(	PSME2-001	proteasome (prosome, macropain) activator subunit 2 (PA28 beta)	protein_co	9606	14:241433	9
ENST0000(	PTGS1-002	prostaglandin-endoperoxide synthase 1 (prostaglandin G/H synthase and cyclooxygenase)	protein_co	9606	9:1223710	33
ENST0000(	PTK6-201	protein tyrosine kinase 6	protein_co	9606	20:635284	18
ENST0000(	PTK7-007	protein tyrosine kinase 7	nonsense_	9606	6:4307634	14
ENST0000(	PTPN18-001	protein tyrosine phosphatase, non-receptor type 18 (brain-derived)	protein_co	9606	2:1303560	31
ENST0000(	PUS3-201	pseudouridylate synthase 3	protein_co	9606	11:125893	10
ENST0000(	PXMP4-003	peroxisomal membrane protein 4, 24kDa	protein_co	9606	20:337074	32
ENST0000(	PYCRL-001	pyrroline-5-carboxylate reductase-like	protein_co	9606	8:1436039	30
ENST0000(	PYGB-001	phosphorylase, glycogen; brain	protein_co	9606	20:252480	44
ENST0000(	PYGL-001	phosphorylase, glycogen, liver	protein_co	9606	14:509052	31
ENST0000(	PYROXD1-001	pyridine nucleotide-disulphide oxidoreductase domain 1	protein_co	9606	12:214376	5
ENST0000(	QPCTL-001	glutaminyl-peptide cyclotransferase-like	protein_co	9606	19:456924	23
ENST0000(	R3HDML-001	R3H domain containing-like	protein_co	9606	20:443369	3
ENST0000(	RAB29-002	RAB29, member RAS oncogene family	protein_co	9606	1:2057694	14
ENST0000(	RAB35-001	RAB35, member RAS oncogene family	protein_co	9606	12:120095	43
ENST0000(	RAB3D-001	RAB3D, member RAS oncogene family	protein_co	9606	19:113220	43
ENST0000(	RAB3IP-003	RAB3A interacting protein	protein_co	9606	12:697386	3
ENST0000(	RAB9A-002	RAB9A, member RAS oncogene family	processed_	9606	X:1368918	6
ENST0000(	RABAC1-001	Rab acceptor 1 (prenylated)	protein_co	9606	19:419566	43
ENST0000(	RABGGTA-002	Rab geranylgeranyltransferase, alpha subunit	protein_co	9606	14:242655	16
ENST0000(	RAD51AP1-002	RAD51 associated protein 1	protein_co	9606	12:453889	19
ENST0000(	RALA-001	v-ral simian leukemia viral oncogene homolog A (ras related)	protein_co	9606	7:3962348	41
ENST0000(	RALBP1-001	ralA binding protein 1	protein_co	9606	18:947500	19
ENST0000(	RALGAPA2-008	Ral GTPase activating protein, alpha subunit 2 (catalytic)	protein_co	9606	20:203927	9
ENST0000(	RANBP3-003	RAN binding protein 3	protein_co	9606	19:591735	18
ENST0000(	RANBP9-001	RAN binding protein 9	protein_co	9606	6:1362149	38
ENST0000(	RAP2A-001	RAP2A, member of RAS oncogene family	protein_co	9606	13:974342	43
ENST0000(	RARS-001	arginyl-tRNA synthetase	protein_co	9606	5:1684864	39
ENST0000(	RB1CC1-001	RB1-inducible coiled-coil 1	protein_co	9606	8:5262245	32
ENST0000(	RBM22-001	RNA binding motif protein 22	protein_co	9606	5:1506907	39
ENST0000(	RCN1-002	reticulocalbin 1, EF-hand calcium binding domain	protein_co	9606	11:320909	39
ENST0000(	RDH10-001	retinol dehydrogenase 10 (all-trans)	protein_co	9606	8:7329461	43
ENST0000(	REEP1-001	receptor accessory protein 1	protein_co	9606	2:8621399	7
ENST0000(	RFC2-002	replication factor C (activator 1) 2, 40kDa	protein_co	9606	7:7423153	37
ENST0000(	RFX4-001	regulatory factor X, 4 (influences HLA class II expression)	protein_co	9606	12:106684	7
ENST0000(	RGS17-201	regulator of G-protein signaling 17	protein_co	9606	6:1530107	37
ENST0000(	RIF1-001	replication timing regulatory factor 1	protein_co	9606	2:1514099	37
ENST0000(	RIN3-001	Ras and Rab interactor 3	protein_co	9606	14:925137	30
ENST0000(	RIPK2-001	receptor-interacting serine-threonine kinase 2	protein_co	9606	8:8975774	41
ENST0000(	RNASEH2A-001	ribonuclease H2, subunit A	protein_co	9606	19:128065	44
ENST0000(	RNF103-001	ring finger protein 103	protein_co	9606	2:8660339	29
ENST0000(	RNF11-001	ring finger protein 11	protein_co	9606	1:5123627	44
ENST0000(	RNF19B-004	ring finger protein 19B	protein_co	9606	1:3293666	37
ENST0000(	RNF8-004	ring finger protein 8, E3 ubiquitin protein ligase	nonsense_	9606	6:3735401	41
ENST0000(	ROPN1-001	rhophilin associated tail protein 1	protein_co	9606	3:1239685	6
ENST0000(	RP1-001	retinitis pigmentosa 1 (autosomal dominant)	protein_co	9606	8:5461606	16
ENST0000(	RP2-001	retinitis pigmentosa 2 (X-linked recessive)	protein_co	9606	X:4683694	42
ENST0000(	RPAP3-001	RNA polymerase II associated protein 3	protein_co	9606	12:476612	34
ENST0000(	RPL18-006	ribosomal protein L18	protein_co	9606	19:486153	40
ENST0000(	RPL19-001	ribosomal protein L19	protein_co	9606	17:392002	44
ENST0000(	RPL3-001	ribosomal protein L3	protein_co	9606	22:393128	40
ENST0000(	RPLP0-201	ribosomal protein, large, P0	protein_co	9606	12:120196	16
ENST0000(	RPN2-001	ribophorin II	protein_co	9606	20:371790	43
ENST0000(	RPS12-001	ribosomal protein S12	protein_co	9606	6:1328144	42
ENST0000(	RPS13-010	ribosomal protein S13	protein_co	9606	11:170743	34
ENST0000(	RPS15-006	ribosomal protein S15	protein_co	9606	19:143835	11
ENST0000(	RPS5-201	ribosomal protein S5	protein_co	9606	19:583872	44
ENST0000(	RPS6KB1-001	ribosomal protein S6 kinase, 70kDa, polypeptide 1	protein_co	9606	17:598931	38
ENST0000(	RPUSD1-001	RNA pseudouridylate synthase domain containing 1	protein_co	9606	16:784974	39
ENST0000(	RRBP1-201	ribosome binding protein 1	protein_co	9606	20:176136	30
ENST0000(	RRN3-001	RRN3 RNA polymerase I transcription factor homolog (S. cerevisiae)	protein_co	9606	16:150600	36
ENST0000(	RSPH6A-001	radial spoke head 6 homolog A (Chlamydomonas)	protein_co	9606	19:457957	6
ENST0000(	RTCB-001	RNA 2',3'-cyclic phosphate and 5'-OH ligase	protein_co	9606	22:323875	42
ENST0000(	RTKN-004	rhotekin	protein_co	9606	2:7442588	39
ENST0000(	RTN2-001	reticulon 2	protein_co	9606	19:454852	14

ENST0000(	RUVBL2-006	RuvB-like AAA ATPase 2	nonsense_	9606	19:489938	43
ENST0000(	SAMD15-001	sterile alpha motif domain containing 15	protein_co	9606	14:773771	40
ENST0000(	SARS-001	seryl-tRNA synthetase	protein_co	9606	1:1092139	44
ENST0000(	SARS2-001	seryl-tRNA synthetase 2, mitochondrial	protein_co	9606	19:389152	11
ENST0000(	SART3-001	squamous cell carcinoma antigen recognized by T cells 3	protein_co	9606	12:108522	28
ENST0000(	SAYSD1-003	SAYSVFN motif domain containing 1	protein_co	9606	6:3910406	41
ENST0000(	SCRN1-001	secernin 1	protein_co	9606	7:2992010	41
ENST0000(	SEC61A1-001	Sec61 alpha 1 subunit (S. cerevisiae)	protein_co	9606	3:1280523	40
ENST0000(	SELPLG-002	selectin P ligand	protein_co	9606	12:108622	5
ENST0000(	SEMA3F-001	sema domain, immunoglobulin domain (Ig), short basic domain, secreted, (semaphorin) 3F	protein_co	9606	3:5015512	15
ENST0000(	SERPIND1-001	serpin peptidase inhibitor, clade D (heparin cofactor), member 1	protein_co	9606	22:207738	34
ENST0000(	SERPINE1-001	serpin peptidase inhibitor, clade E (nexin, plasminogen activator inhibitor type 1), member 1	protein_co	9606	7:1011270	43
ENST0000(	SETX-001	senataxin	protein_co	9606	9:1322613	39
ENST0000(	SF1-002	splicing factor 1	protein_co	9606	11:647646	31
ENST0000(	SF3A1-001	splicing factor 3a, subunit 1, 120kDa	protein_co	9606	22:303319	42
ENST0000(	SF3B6-001	splicing factor 3b, subunit 6, 14kDa	protein_co	9606	2:2406758	44
ENST0000(	SFXN3-201	sideroflexin 3	protein_co	9606	10:101031	42
ENST0000(	SGCG-001	sarcoglycan, gamma (35kDa dystrophin-associated glycoprotein)	protein_co	9606	13:231809	22
ENST0000(	SGK1-009	serum/glucocorticoid regulated kinase 1	protein_co	9606	6:1341692	38
ENST0000(	SGTA-001	small glutamine-rich tetratricopeptide repeat (TPR)-containing, alpha	protein_co	9606	19:275471	44
ENST0000(	SH3TC1-001	SH3 domain and tetratricopeptide repeats 1	protein_co	9606	4:8199367	38
ENST0000(	SHPK-001	sedoheptulokinase	protein_co	9606	17:360826	43
ENST0000(	SIKE1-001	suppressor of IKBKE 1	protein_co	9606	1:1147727	4
ENST0000(	SIPA1L3-001	signal-induced proliferation-associated 1 like 3	protein_co	9606	19:379072	43
ENST0000(	SIRT1-001	sirtuin 1	protein_co	9606	10:678846	40
ENST0000(	SIRT4-001	sirtuin 4	protein_co	9606	12:120302	36
ENST0000(	SIX1-001	SIX homeobox 1	protein_co	9606	14:606434	43
ENST0000(	SIX4-001	SIX homeobox 4	protein_co	9606	14:607095	30
ENST0000(	SLC10A2-001	solute carrier family 10 (sodium/bile acid cotransporter), member 2	protein_co	9606	13:103043	7
ENST0000(	SLC11A1-001	solute carrier family 11 (proton-coupled divalent metal ion transporter), member 1	protein_co	9606	2:2183820	33
ENST0000(	SLC19A2-001	solute carrier family 19 (thiamine transporter), member 2	protein_co	9606	1:1694639	42
ENST0000(	SLC1A4-001	solute carrier family 1 (glutamate/neutral amino acid transporter), member 4	protein_co	9606	2:6498940	42
ENST0000(	SLC22A4-001	solute carrier family 22 (organic cation/zwitterion transporter), member 4	protein_co	9606	5:1322944	35
ENST0000(	SLC22A5-001	solute carrier family 22 (organic cation/carnitine transporter), member 5	protein_co	9606	5:1323697	25
ENST0000(	SLC25A2-001	solute carrier family 25 (mitochondrial carrier; ornithine transporter) member 2	protein_co	9606	5:1413026	19
ENST0000(	SLC25A39-001	solute carrier family 25, member 39	protein_co	9606	17:443196	43
ENST0000(	SLC25A43-001	solute carrier family 25, member 43	protein_co	9606	X:1193990	38
ENST0000(	SLC35C2-002	solute carrier family 35 (GDP-fucose transporter), member C2	protein_co	9606	20:463495	14
ENST0000(	SLC35D1-001	solute carrier family 35 (UDP-GlcA/UDP-GalNAc transporter), member D1	protein_co	9606	1:6699933	44
ENST0000(	SLC35F5-001	solute carrier family 35, member F5	protein_co	9606	2:1137127	23
ENST0000(	SLC38A7-001	solute carrier family 38, member 7	protein_co	9606	16:586651	25
ENST0000(	SLC39A9-013	solute carrier family 39, member 9	protein_co	9606	14:693986	28
ENST0000(	SLC44A4-001	solute carrier family 44, member 4	protein_co	9606	6:3186319	15
ENST0000(	SLC7A2-201	solute carrier family 7 (cationic amino acid transporter, y+ system), member 2	protein_co	9606	8:1753877	11
ENST0000(	SLC7A6-001	solute carrier family 7 (amino acid transporter light chain, y+L system), member 6	protein_co	9606	16:682645	36
ENST0000(	SLCO4A1-201	solute carrier organic anion transporter family, member 4A1	protein_co	9606	20:626424	23
ENST0000(	SLURP1-001	secreted LY6/PLAUR domain containing 1	protein_co	9606	8:1427409	3
ENST0000(	SMARCD2-002	SWI/SNF related, matrix associated, actin dependent regulator of chromatin, subfamily d, member 2	protein_co	9606	17:638326	12
ENST0000(	SMIM8-001	small integral membrane protein 8	protein_co	9606	6:8732259	36
ENST0000(	SMUG1-008	single-strand-selective monofunctional uracil-DNA glycosylase 1	protein_co	9606	12:541814	35
ENST0000(	SNAI1-001	snail family zinc finger 1	protein_co	9606	20:499829	41
ENST0000(	SNAP29-001	synaptosomal-associated protein, 29kDa	protein_co	9606	22:208589	39
ENST0000(	SNRNP27-001	small nuclear ribonucleoprotein 27kDa (U4/U6.U5)	protein_co	9606	2:6989356	3
ENST0000(	SNRNP70-003	small nuclear ribonucleoprotein 70kDa (U1)	protein_co	9606	19:490854	38
ENST0000(	SNRPA-001	small nuclear ribonucleoprotein polypeptide A	protein_co	9606	19:407508	38
ENST0000(	SNRPB2-001	small nuclear ribonucleoprotein polypeptide B	protein_co	9606	20:167299	6
ENST0000(	SNRPC-001	small nuclear ribonucleoprotein polypeptide C	protein_co	9606	6:3475740	43
ENST0000(	SNRPD3-001	small nuclear ribonucleoprotein D3 polypeptide 18kDa	protein_co	9606	22:245555	32
ENST0000(	SNTA1-001	syntrophin, alpha 1	protein_co	9606	20:334079	42
ENST0000(	SNX17-001	sorting nexin 17	protein_co	9606	2:2737052	42
ENST0000(	SNX3-001	sorting nexin 3	protein_co	9606	6:1082112	43
ENST0000(	SOGA1-005	suppressor of glucose, autophagy associated 1	protein_co	9606	20:367774	39
ENST0000(	SOS2-001	son of sevenless homolog 2 (Drosophila)	protein_co	9606	14:501171	40
ENST0000(	SOX4-001	SRY (sex determining region Y)-box 4	protein_co	9606	6:2159276	43
ENST0000(	SP140L-201	SP140 nuclear body protein-like	protein_co	9606	2:2303272	7
ENST0000(	SPAG7-001	sperm associated antigen 7	protein_co	9606	17:495922	42
ENST0000(	SPARC-001	secreted protein, acidic, cysteine-rich (osteonectin)	protein_co	9606	5:1516610	27
ENST0000(	SPATA20-001	spermatogenesis associated 20	protein_co	9606	17:505470	29
ENST0000(	SPG21-001	spastic paraplegia 21 (autosomal recessive, Mast syndrome)	protein_co	9606	15:649630	36
ENST0000(	SPTLC2-001	serine palmitoyltransferase, long chain base subunit 2	protein_co	9606	14:775059	44
ENST0000(	SRGN-001	serglycin	protein_co	9606	10:690881	44
ENST0000(	SRP54-201	signal recognition particle 54kDa	protein_co	9606	14:349828	41
ENST0000(	SRRD-001	SRR1 domain containing	protein_co	9606	22:264838	27
ENST0000(	SRSF6-001	serine/arginine-rich splicing factor 6	protein_co	9606	20:434579	40
ENST0000(	SRSF9-001	serine/arginine-rich splicing factor 9	protein_co	9606	12:120461	42
ENST0000(	SS18L2-201	synovial sarcoma translocation gene on chromosome 18-like 2	protein_co	9606	3:4259080	17
ENST0000(	SSPN-001	sarcospan	protein_co	9606	12:261954	5
ENST0000(	SSR1-001	signal sequence receptor, alpha	protein_co	9606	6:7281143	29
ENST0000(	ST13-001	suppression of tumorigenicity 13 (colon carcinoma) (Hsp70 interacting protein)	protein_co	9606	22:408245	21
ENST0000(	ST3GAL4-008	ST3 beta-galactoside alpha-2,3-sialyltransferase 4	protein_co	9606	11:126355	19
ENST0000(	ST6GAL1-001	ST6 beta-galactosamide alpha-2,6-sialyltranferase 1	protein_co	9606	3:1869304	28
ENST0000(	STAG2-006	stromal antigen 2	protein_co	9606	X:1239605	3
ENST0000(	STARD3NL-001	STARD3 N-terminal like	protein_co	9606	7:3817822	41
ENST0000(	STATH-001	statherin	protein_co	9606	4:6999593	3

ENST0000(	STK10-001	serine/threonine kinase 10	protein_co	9606	5:1720420	39
ENST0000(	STK38-001	serine/threonine kinase 38	protein_co	9606	6:3649389	43
ENST0000(	STRADB-001	STE20-related kinase adaptor beta	protein_co	9606	2:2014516	17
ENST0000(	STS-001	steroid sulfatase (microsomal), isozyme S	protein_co	9606	X:7219456	29
ENST0000(	STUB1-001	STIP1 homology and U-box containing protein 1, E3 ubiquitin protein ligase	protein_co	9606	16:680276	9
ENST0000(	STX10-004	syntaxin 10	protein_co	9606	19:131440	19
ENST0000(	STX1A-001	syntaxin 1A (brain)	protein_co	9606	7:7369920	6
ENST0000(	STXBP2-001	syntaxin binding protein 2	protein_co	9606	19:763711	34
ENST0000(	STYK1-001	serine/threonine/tyrosine kinase 1	protein_co	9606	12:106189	37
ENST0000(	SUGP1-001	SURP and G patch domain containing 1	protein_co	9606	19:192765	7
ENST0000(	SULT1E1-001	sulfotransferase family 1E, estrogen-preferring, member 1	protein_co	9606	4:6984121	43
ENST0000(	SUPT16H-001	suppressor of Ty 16 homolog (S. cerevisiae)	protein_co	9606	14:213514	43
ENST0000(	SYDE2-001	synapse defective 1, Rho GTPase, homolog 2 (C. elegans)	retained_in	9606	1:8518115	36
ENST0000(	SYMPK-001	symplekin	protein_co	9606	19:458154	34
ENST0000(	SYNGR1-006	synaptogyrin 1	protein_co	9606	22:393499	7
ENST0000(	SYNGR2-001	synaptogyrin 2	protein_co	9606	17:781685	19
ENST0000(	SYPL1-001	synaptophysin-like 1	protein_co	9606	7:1060905	13
ENST0000(	SYS1-001	Sys1 golgi trafficking protein	protein_co	9606	20:453630	30
ENST0000(	TAB1-001	TGF-beta activated kinase 1/MAP3K7 binding protein 1	protein_co	9606	22:393997	41
ENST0000(	TARBP1-001	TAR (HIV-1) RNA binding protein 1	protein_co	9606	1:2343913	40
ENST0000(	TARDBP-001	TAR DNA binding protein	protein_co	9606	1:1101240	17
ENST0000(	TAS2R3-001	taste receptor, type 2, member 3	protein_co	9606	7:1417640	25
ENST0000(	TAS2R7-001	taste receptor, type 2, member 7	protein_co	9606	12:108015	7
ENST0000(	TAS2R8-001	taste receptor, type 2, member 8	protein_co	9606	12:108060	7
ENST0000(	TAS2R9-001	taste receptor, type 2, member 9	protein_co	9606	12:108091	9
ENST0000(	TAX1BP3-001	Tax1 (human T-cell leukemia virus type I) binding protein 3	protein_co	9606	17:366290	43
ENST0000(	TBC1D12-001	TBC1 domain family, member 12	protein_co	9606	10:944025	42
ENST0000(	TBC1D17-001	TBC1 domain family, member 17	protein_co	9606	19:498774	30
ENST0000(	TBL1X-003	transducin (beta)-like 1X-linked	protein_co	9606	X:9465008	17
ENST0000(	TBP-001	TATA box binding protein	protein_co	9606	6:1705543	30
ENST0000(	TBPL2-001	TATA box binding protein like 2	protein_co	9606	14:554135	3
ENST0000(	TBX15-001	T-box 15	protein_co	9606	1:1188830	10
ENST0000(	TCEAL3-201	transcription elongation factor A (SII)-like 3	protein_co	9606	X:1036079	24
ENST0000(	TCEANC2-001	transcription elongation factor A (SII) N-terminal and central domain containing 2	protein_co	9606	1:5405360	39
ENST0000(	TCFL5-201	transcription factor-like 5 (basic helix-loop-helix)	protein_co	9606	20:628451	13
ENST0000(	TFG-001	TRK-fused gene	protein_co	9606	3:1007094	36
ENST0000(	TFPI-201	tissue factor pathway inhibitor (lipoprotein-associated coagulation inhibitor)	protein_co	9606	2:1874642	17
ENST0000(	TGFB1-001	transforming growth factor, beta 1	protein_co	9606	19:413303	43
ENST0000(	TGFB3-001	transforming growth factor, beta 3	protein_co	9606	14:759582	30
ENST0000(	TGFBR3-201	transforming growth factor, beta receptor III	protein_co	9606	1:9168034	37
ENST0000(	THG1L-001	tRNA-histidine guanylyltransferase 1-like (S. cerevisiae)	protein_co	9606	5:1577311	39
ENST0000(	THOC2-201	THO complex 2	protein_co	9606	X:1236005	32
ENST0000(	THPO-001	thrombopoietin	protein_co	9606	3:1843719	9
ENST0000(	TIMELESS-002	timeless circadian clock	protein_co	9606	12:564171	42
ENST0000(	TIMM21-001	translocase of inner mitochondrial membrane 21 homolog (yeast)	protein_co	9606	18:741485	8
ENST0000(	TMED1-001	transmembrane emp24 protein transport domain containing 1	protein_co	9606	19:108324	24
ENST0000(	TMED8-001	transmembrane emp24 protein transport domain containing 8	protein_co	9606	14:773350	44
ENST0000(	TMEM101-201	transmembrane protein 101	protein_co	9606	17:440111	42
ENST0000(	TMEM109-001	transmembrane protein 109	protein_co	9606	11:609138	41
ENST0000(	TMEM131-001	transmembrane protein 131	protein_co	9606	2:9775634	41
ENST0000(	TMEM14A-001	transmembrane protein 14A	protein_co	9606	6:5267110	43
ENST0000(	TMEM14C-001	transmembrane protein 14C	protein_co	9606	6:1072297	42
ENST0000(	TMEM159-001	transmembrane protein 159	protein_co	9606	16:211583	9
ENST0000(	TMEM161A-004	transmembrane protein 161A	protein_co	9606	19:191196	41
ENST0000(	TMEM214-001	transmembrane protein 214	protein_co	9606	2:2703295	44
ENST0000(	TMEM30A-002	transmembrane protein 30A	protein_co	9606	6:7525292	39
ENST0000(	TMEM59-001	transmembrane protein 59	protein_co	9606	1:5403167	43
ENST0000(	TMEM97-001	transmembrane protein 97	protein_co	9606	17:283190	40
ENST0000(	TMX4-001	thioredoxin-related transmembrane protein 4	protein_co	9606	20:797734	41
ENST0000(	TNFAIP1-001	tumor necrosis factor, alpha-induced protein 1 (endothelial)	protein_co	9606	17:283357	42
ENST0000(	TNFRSF10A-001	tumor necrosis factor receptor superfamily, member 10a	protein_co	9606	8:2319045	42
ENST0000(	TNFRSF1A-001	tumor necrosis factor receptor superfamily, member 1A	protein_co	9606	12:632875	42
ENST0000(	TNNC1-001	troponin C type 1 (slow)	protein_co	9606	3:5245110	15
ENST0000(	TNS1-001	tensin 1	protein_co	9606	2:2177997	7
ENST0000(	TOMM22-001	translocase of outer mitochondrial membrane 22 homolog (yeast)	protein_co	9606	22:386819	43
ENST0000(	TRAF2-007	TNF receptor-associated factor 2	protein_co	9606	9:1368865	42
ENST0000(	TRAM2-001	translocation associated membrane protein 2	protein_co	9606	6:5249740	43
ENST0000(	TRAP1-001	TNF receptor-associated protein 1	protein_co	9606	16:365803	40
ENST0000(	TRIB3-001	tribbles pseudokinase 3	protein_co	9606	20:380617	43
ENST0000(	TRIM23-001	tripartite motif containing 23	protein_co	9606	5:6558968	39
ENST0000(	TRIP13-001	thyroid hormone receptor interactor 13	protein_co	9606	5:892643-	38
ENST0000(	TRIP6-001	thyroid hormone receptor interactor 6	protein_co	9606	7:1008671	36
ENST0000(	TRMT6-001	tRNA methyltransferase 6 homolog (S. cerevisiae)	protein_co	9606	20:593783	40
ENST0000(	TRPM5-001	transient receptor potential cation channel, subfamily M, member 5	protein_co	9606	11:240451	4
ENST0000(	TRPS1-002	trichorhinophalangeal syndrome I	protein_co	9606	8:1154125	27
ENST0000(	TSPAN12-001	tetraspanin 12	protein_co	9606	7:1207873	31
ENST0000(	TSPAN8-002	tetraspanin 8	protein_co	9606	12:711251	21
ENST0000(	TSPAN9-001	tetraspanin 9	protein_co	9606	12:307737	41
ENST0000(	TTC1-001	tetratricopeptide repeat domain 1	protein_co	9606	5:1600091	41
ENST0000(	TTC17-001	tetratricopeptide repeat domain 17	protein_co	9606	11:433589	16
ENST0000(	TTC31-001	tetratricopeptide repeat domain 31	protein_co	9606	2:7448308	20
ENST0000(	TTL-001	tubulin tyrosine ligase	protein_co	9606	2:1124821	25
ENST0000(	TTLL12-001	tubulin tyrosine ligase-like family, member 12	protein_co	9606	22:431666	42
ENST0000(	TTLL2-001	tubulin tyrosine ligase-like family, member 2	protein_co	9606	6:1673250	9

ENST0000(	TWIST1-001	twist family bHLH transcription factor 1	protein_co	9606	7:1911546	21
ENST0000(	TWISTNB-001	TWIST neighbor	protein_co	9606	7:1969546	42
ENST0000(	TYRL-002	tyrosinase-like (pseudogene)	unprocesse	9606	11:494050	2
ENST0000(	UBA2-001	ubiquitin-like modifier activating enzyme 2	protein_co	9606	19:344283	32
ENST0000(	UBR5-002	ubiquitin protein ligase E3 component n-recognin 5	protein_co	9606	8:1022537	33
ENST0000(	UBR7-001	ubiquitin protein ligase E3 component n-recognin 7 (putative)	protein_co	9606	14:932070	25
ENST0000(	UMPS-001	uridine monophosphate synthetase	protein_co	9606	3:1247303	41
ENST0000(	UNC13D-001	unc-13 homolog D (C. elegans)	protein_co	9606	17:758272	41
ENST0000(	UNG-001	uracil-DNA glycosylase	protein_co	9606	12:109097	36
ENST0000(	UNKL-201	unkempt family zinc finger-like	protein_co	9606	16:136320	4
ENST0000(	UQCRC1-001	ubiquinol-cytochrome c reductase core protein I	protein_co	9606	3:4859900	35
ENST0000(	UROD-001	uroporphyrinogen decarboxylase	protein_co	9606	1:4501214	44
ENST0000(	USP10-001	ubiquitin specific peptidase 10	protein_co	9606	16:846999	27
ENST0000(	USP11-201	ubiquitin specific peptidase 11	protein_co	9606	X:4723286	43
ENST0000(	USP18-001	ubiquitin specific peptidase 18	protein_co	9606	22:181498	43
ENST0000(	USP28-001	ubiquitin specific peptidase 28	protein_co	9606	11:113797	17
ENST0000(	USP31-001	ubiquitin specific peptidase 31	protein_co	9606	16:230614	41
ENST0000(	USP5-001	ubiquitin specific peptidase 5 (isopeptidase T)	protein_co	9606	12:685212	37
ENST0000(	UTP18-001	UTP18 small subunit (SSU) processome component homolog (yeast)	protein_co	9606	17:512605	41
ENST0000(	VAMP3-001	vesicle-associated membrane protein 3	protein_co	9606	1:7771269	44
ENST0000(	VASH1-001	vasohibin 1	protein_co	9606	14:767621	41
ENST0000(	VASP-001	vasodilator-stimulated phosphoprotein	protein_co	9606	19:455074	44
ENST0000(	VAX2-001	ventral anterior homeobox 2	protein_co	9606	2:7090059	40
ENST0000(	VCL-201	vinculin	protein_co	9606	10:739981	39
ENST0000(	VDAC3-003	voltage-dependent anion channel 3	protein_co	9606	8:4239190	43
ENST0000(	VDR-201	vitamin D (1,25- dihydroxyvitamin D3) receptor	protein_co	9606	12:478415	7
ENST0000(	VIM-001	vimentin	protein_co	9606	10:172292	44
ENST0000(	VPS13D-014	vacuolar protein sorting 13 homolog D (S. cerevisiae)	protein_co	9606	1:1227712	38
ENST0000(	VPS18-001	vacuolar protein sorting 18 homolog (S. cerevisiae)	protein_co	9606	15:408944	44
ENST0000(	VPS4B-001	vacuolar protein sorting 4 homolog B (S. cerevisiae)	protein_co	9606	18:633891	41
ENST0000(	VSIG1-001	V-set and immunoglobulin domain containing 1	protein_co	9606	X:1080450	32
ENST0000(	VTI1B-002	vesicle transport through interaction with t-SNAREs 1B	nonsense_	9606	14:676512	14
ENST0000(	WARS2-001	tryptophanyl tRNA synthetase 2, mitochondrial	protein_co	9606	1:1190312	41
ENST0000(	WDFY1-001	WD repeat and FYVE domain containing 1	protein_co	9606	2:2238753	43
ENST0000(	WDR24-201	WD repeat domain 24	protein_co	9606	16:684734	35
ENST0000(	WDR77-001	WD repeat domain 77	protein_co	9606	1:1114398	31
ENST0000(	WFS1-001	Wolfram syndrome 1 (wolframin)	protein_co	9606	4:6269850	41
ENST0000(	WNK4-001	WNK lysine deficient protein kinase 4	protein_co	9606	17:427806	20
ENST0000(	XBP1-001	X-box binding protein 1	protein_co	9606	22:287945	33
ENST0000(	XYLB-001	xylulokinase homolog (H. influenzae)	protein_co	9606	3:3834677	41
ENST0000(	YBX3-001	Y box binding protein 3	protein_co	9606	12:106992	25
ENST0000(	YIPF1-201	Yip1 domain family, member 1	protein_co	9606	1:5385172	18
ENST0000(	YKT6-001	YKT6 v-SNARE homolog (S. cerevisiae)	protein_co	9606	7:4420104	43
ENST0000(	YTHDC2-001	YTH domain containing 2	protein_co	9606	5:1135136	41
ENST0000(	YWHAQ-001	tyrosine 3-monooxygenase/tryptophan 5-monooxygenase activation protein, theta	protein_co	9606	2:9583972	44
ENST0000(	ZBTB47-002	zinc finger and BTB domain containing 47	protein_co	9606	3:4265368	32
ENST0000(	ZC3H13-001	zinc finger CCCH-type containing 13	protein_co	9606	13:459544	24
ENST0000(	ZC3HAV1-001	zinc finger CCCH-type, antiviral 1	protein_co	9606	7:1390435	43
ENST0000(	ZFP64-003	ZFP64 zinc finger protein	protein_co	9606	20:521512	10
ENST0000(	ZFY-201	zinc finger protein, Y-linked	protein_co	9606	Y:2935477	13
ENST0000(	ZNF141-001	zinc finger protein 141	protein_co	9606	4:337835-3	22
ENST0000(	ZNF211-007	zinc finger protein 211	protein_co	9606	19:576332	13
ENST0000(	ZNF263-001	zinc finger protein 263	protein_co	9606	16:328294	33
ENST0000(	ZNF268-002	zinc finger protein 268	protein_co	9606	12:133181	22
ENST0000(	ZNF317-001	zinc finger protein 317	protein_co	9606	19:914038	42
ENST0000(	ZNF415-001	zinc finger protein 415	protein_co	9606	19:531078	10
ENST0000(	ZNF468-002	zinc finger protein 468	protein_co	9606	19:528385	22
ENST0000(	ZNF500-001	zinc finger protein 500	protein_co	9606	16:474823	31
ENST0000(	ZNF510-201	zinc finger protein 510	protein_co	9606	9:9675586	33
ENST0000(	ZNF549-002	zinc finger protein 549	protein_co	9606	19:575273	21
ENST0000(	ZPR1-001	ZPR1 zinc finger	protein_co	9606	11:116777	44
ENST0000(	ZW10-001	zw10 kinetochore protein	protein_co	9606	11:113733	41

Table S7. Angiogenes, specific Heart not Liver

Accession	Name	Description	Biotype	Tax ID	Location	# of Detections
ENST00000607639	5S_rRNA.1-212	5S ribosomal RNA	rRNA	9606	14:676571	1
ENST00000539547	A2ML1-004	alpha-2-macroglobulin-like 1	protein_coding	9606	12:884500	1
ENST00000570932	AATK-003	apoptosis-associated tyrosine kinase	nonsense_mediated_decay	9606	17:811172	4
ENST00000615488	ABC7-481722F1.1-001		processed_pseudogene	9606	7:37032-3	1
ENST00000494046	ABCA2-017	ATP-binding cassette, sub-family A (ABC1), member 2	retained_intron	9606	9:1370201	2
ENST00000529442	ABCA7-009	ATP-binding cassette, sub-family A (ABC1), member 7	nonsense_mediated_decay	9606	19:105692	2
ENST00000295750	ABCB6-005	ATP-binding cassette, sub-family B (MDR/TAP), member 6 (Langereis blood group)	protein_coding	9606	2:2192097	2
ENST00000372515	ABCC10-009	ATP-binding cassette, sub-family C (CFTR/MRP), member 10	protein_coding	9606	6:4342736	1
ENST00000556119	ABCD4-020	ATP-binding cassette, sub-family D (ALD), member 4	nonsense_mediated_decay	9606	14:742923	2
ENST00000405322	ABCG5-002	ATP-binding cassette, sub-family G (WHITE), member 5	protein_coding	9606	2:4381256	1
ENST00000450061	AC000099.1-002		antisense	9606	7:1272151	1
ENST00000434698	AC000374.1-001		processed_pseudogene	9606	7:1265111	1
ENST00000413406	AC004014.3-001		lincRNA	9606	7:1089094	4
ENST00000563823	AC004158.2-006		lincRNA	9606	16:725233	2
ENST00000456537	AC004448.5-001		lincRNA	9606	17:194391	2
ENST00000421862	AC004540.5-004		lincRNA	9606	7:2639860	2
ENST00000418764	AC004543.2-001		lincRNA	9606	7:1981368	2
ENST00000448783	AC005041.11-001		processed_pseudogene	9606	2:7441302	1
ENST00000456995	AC005229.5-001		processed_pseudogene	9606	7:1485804	1
ENST00000592716	AC005307.3-005		lincRNA	9606	19:284376	2
ENST00000620402	AC005412.1-201		miRNA	9606	17:293011	2
ENST00000431878	AC005722.4-001		processed_pseudogene	9606	17:197552	1
ENST00000582731	AC005926.1-201		miRNA	9606	X:3031508	2
ENST00000616497	AC006288.1-201		miRNA	9606	9:1195116	2
ENST00000410616	AC006534.1-201		miRNA	9606	17:678135	2
ENST00000614674	AC006534.3-201		miRNA	9606	17:677205	4
ENST00000447461	AC007327.6-001		processed_pseudogene	9606	7:3622862	1
ENST00000620982	AC007339.2-201		miRNA	9606	16:496801	2
ENST00000418591	AC007563.1-001		lincRNA	9606	2:2168704	1
ENST00000447289	AC007563.5-001		antisense	9606	2:2166944	1
ENST00000434390	AC008079.9-001		antisense	9606	22:181780	1
ENST00000424916	AC008163.6-001		processed_pseudogene	9606	7:8146303	1
ENST00000439653	AC008175.1-002		lincRNA	9606	Y:2248053	4
ENST00000408143	AC008625.1-201		miRNA	9606	5:1543400	2
ENST00000458850	AC009009.1-201		miRNA	9606	5:1357131	2
ENST00000413944	AC010091.1-002		lincRNA	9606	7:8157638	1
ENST00000597623	AC010127.3-002		antisense	9606	2:1659574	2
ENST00000581768	AC010311.1-201		miRNA	9606	19:716042	2
ENST00000577852	AC010323.1-201		miRNA	9606	19:834601	2
ENST00000416320	AC010492.5-001		processed_pseudogene	9606	19:542680	2
ENST00000417035	AC010740.1-001		processed_pseudogene	9606	2:1408984	1
ENST00000448431	AC010744.1-001		lincRNA	9606	2:8352281	2
ENST00000407635	AC011298.2-001		lincRNA	9606	2:2406863	2
ENST00000583981	AC011379.1-201		miRNA	9606	5:1401957	1
ENST00000598042	AC011516.2-001		lincRNA	9606	19:226155	2
ENST00000617109	AC011841.2-201		miRNA	9606	GL000205	2
ENST00000454928	AC013271.3-002		lincRNA	9606	2:1099374	2
ENST00000578597	AC016727.1-201		miRNA	9606	2:6155536	2
ENST00000437979	AC016995.3-003		lincRNA	9606	2:3845863	2
ENST00000408375	AC018628.1-201		miRNA	9606	17:620158	2
ENST00000435682	AC018742.1-001		lincRNA	9606	2:2168743	3
ENST00000428171	AC018832.1-001		lincRNA	9606	3:8079322	2
ENST00000619792	AC023491.2-201		protein_coding	9606	KI270731.	1
ENST00000440535	AC024560.3-002		processed_transcript	9606	3:1976142	2
ENST00000413954	AC064871.3-002		antisense	9606	2:1830954	2
ENST00000459291	AC069082.1-201		miRNA	9606	15:791142	2
ENST00000599089	AC073043.1-006		lincRNA	9606	2:1998784	2
ENST00000600956	AC073043.1-010		lincRNA	9606	2:1998790	1
ENST00000600062	AC073043.1-020		retained_intron	9606	2:1998945	2
ENST00000382528	AC074389.6-001		lincRNA	9606	7:1692810	1
ENST00000447529	AC078842.4-001		lincRNA	9606	7:1373185	1
ENST00000422019	AC079199.2-001		processed_pseudogene	9606	17:398393	2
ENST00000408681	AC079235.1-201		miRNA	9606	2:2271242	2
ENST00000541917	AC079354.1-201	KIAA2012	protein_coding	9606	2:2020732	1
ENST00000615351	AC079768.1-201		miRNA	9606	4:1699242	1
ENST00000427064	AC090505.1-001		lincRNA	9606	3:1948278	2
ENST00000578625	AC090559.1-201		miRNA	9606	11:474858	2
ENST00000402115	AC091801.1-002		lincRNA	9606	7:3140500	2
ENST00000416748	AC091813.2-001		processed_pseudogene	9606	7:7954391	1
ENST00000425131	AC092066.6-001		processed_pseudogene	9606	19:447651	1
ENST00000408359	AC092289.1-201		miRNA	9606	16:725795	2
ENST00000598703	AC092316.1-001		antisense	9606	19:854950	3
ENST00000414446	AC092570.1-001		processed_pseudogene	9606	2:1013755	1
ENST00000433998	AC092580.1-001		processed_pseudogene	9606	2:7736438	1
ENST00000497017	AC093627.11-001		lincRNA	9606	7:174920-	2
ENST00000609486	AC097467.2-023		antisense	9606	4:1553050	4
ENST00000416350	AC104777.3-001		lincRNA	9606	2:1505661	1
ENST00000424170	AC104820.2-002		lincRNA	9606	2:1811238	2
ENST00000390165	AC110056.1-201		miRNA	9606	11:292766	2
ENST00000616561	AC113133.1-201		miRNA	9606	8:4173481	2
ENST00000509542	AC114812.9-001		unprocessed_pseudogene	9606	2:2337389	2
ENST00000427353	AC115283.1-001		processed_pseudogene	9606	3:3126900	3
ENST00000459309	AC117401.1-201		miRNA	9606	3:1240619	2
ENST00000583726	AC122714.1-201		miRNA	9606	5:1805824	2
ENST00000621140	AC129778.2-001		lincRNA	9606	9:4049785	2
ENST00000625083	AC139099.7-001		TEC	9606	17:831326	1
ENST00000459300	AC190387.2-201		miRNA	9606	12:108987	2
ENST00000544651	ACACB-008	acetyl-CoA carboxylase beta	retained_intron	9606	12:109191	2
ENST00000571220	ACAP1-007	ArfGAP with coiled-coil, ankyrin repeat and PH domains 1	retained_intron	9606	17:734293	2
ENST00000528370	ACAT1-009	acetyl-CoA acetyltransferase 1	protein_coding	9606	11:108133	2
ENST00000532792	ACAT1-011	acetyl-CoA acetyltransferase 1	retained_intron	9606	11:108138	4

ENST00000496890	ACBD7-002	acyl-CoA binding domain containing 7	processed_transcript	9606	10:150784	2
ENST00000461272	ACOT8-002	acyl-CoA thioesterase 8	nonsense_mediated_decay	9606	20:458417	2
ENST00000483689	ACPP-005	acid phosphatase, prostate	retained_intron	9606	3:1323283	1
ENST00000434099	ACSL6-005	acyl-CoA synthetase long-chain family member 6	protein_coding	9606	5:1319727	2
ENST00000572843	ACSM2A-009	acyl-CoA synthetase medium-chain family member 2A	retained_intron	9606	16:204753	2
ENST00000435871	ACTBP12-001	actin, beta pseudogene 12	transcribed_processed_pseudo	9606	1:9222901	1
ENST00000485601	ACTL6B-004	actin-like 6B	retained_intron	9606	7:1006482	2
ENST00000539352	ACTR3C-201	ARP3 actin-related protein 3 homolog C (yeast)	protein_coding	9606	7:1502847	2
ENST00000417441	ADAM5-002	ADAM metallopeptidase domain 5, pseudogene	processed_transcript	9606	8:3931466	2
ENST00000463298	ADAM8-003	ADAM metallopeptidase domain 8	retained_intron	9606	10:133272	1
ENST00000380566	ADAMTSL1-002	ADAMTS-like 1	protein_coding	9606	9:1847416	1
ENST00000474762	ADARB2-007	adenosine deaminase, RNA-specific, B2 (non-functional)	processed_transcript	9606	10:118284	2
ENST00000260600	ADCY3-001	adenylate cyclase 3	protein_coding	9606	2:2481916	2
ENST00000506651	ADH1B-004	alcohol dehydrogenase 1B (class I), beta polypeptide	nonsense_mediated_decay	9606	4:9930749	1
ENST00000478927	ADPRH-003	ADP-ribosylarginine hydrolase	protein_coding	9606	3:1195795	1
ENST00000545670	AE000661.37-004		antisense	9606	14:223821	1
ENST00000514473	AE000661.37-006		antisense	9606	14:223806	1
ENST00000435732	AF165138.7-002		protein_coding	9606	21:145919	2
ENST00000483600	AFF3-011	AF4/FMR2 family, member 3	processed_transcript	9606	2:1001098	4
ENST00000397238	AGAP3-001	ArfGAP with GTPase domain, ankyrin repeat and PH domain 3	protein_coding	9606	7:1510867	1
ENST00000375070	AGER-203	advanced glycosylation end product-specific receptor	protein_coding	9606	6:3218096	2
ENST00000355283	AK9-001	adenylate kinase 9	protein_coding	9606	6:1095276	6
ENST00000434743	AKAP8P1-001	A kinase (PRKA) anchor protein 8 pseudogene 1	processed_pseudogene	9606	9:1101174	2
ENST00000455190	AKR1C2-202	aldo-keto reductase family 1, member C2	protein_coding	9606	10:499991	1
ENST00000533295	AKR1E2-007	aldo-keto reductase family 1, member E2	protein_coding	9606	10:478662	2
ENST00000581611	AL008721.1-201		miRNA	9606	22:255327	2
ENST00000579493	AL020993.1-201		miRNA	9606	22:382743	2
ENST00000516820	AL050316.1-201		miRNA	9606	20:530626	1
ENST00000517228	AL109947.2-201		miRNA	9606	6:1095043	1
ENST00000408401	AL117333.1-201		miRNA	9606	20:139557	1
ENST00000577299	AL121655.1-201		miRNA	9606	2:3208830	2
ENST00000582803	AL390036.1-201		miRNA	9606	1:1080186	2
ENST00000408520	AL591415.1-201		miRNA	9606	1:4762109	2
ENST00000420153	AL928742.12-002		lincRNA	9606	14:105599	2
ENST00000430119	ALDH1A2-015	aldehyde dehydrogenase 1 family, member A2	nonsense_mediated_decay	9606	15:579538	1
ENST00000511283	ALDH1L1-014	aldehyde dehydrogenase 1 family, member L1	nonsense_mediated_decay	9606	3:1261585	2
ENST00000512384	ALDH1L1-AS1-001	ALDH1L1 antisense RNA 1	antisense	9606	3:1261036	2
ENST00000262374	ALG1-001	ALG1, chitobiosyldiphosphodolichol beta-mannosyltransferase	protein_coding	9606	16:507181	1
ENST00000591783	ALG1-010	ALG1, chitobiosyldiphosphodolichol beta-mannosyltransferase	protein_coding	9606	16:507220	4
ENST00000429370	ALKBH8-003	alkB, alkylation repair homolog 8 (E. coli)	protein_coding	9606	11:107503	2
ENST00000483623	ALOX5-003	arachidonate 5-lipoxygenase	processed_transcript	9606	10:454240	2
ENST00000509688	ALPK1-002	alpha-kinase 1	retained_intron	9606	4:1122973	1
ENST00000374832	ALPL-002	alkaline phosphatase, liver/bone/kidney	protein_coding	9606	1:2155127	2
ENST00000321420	AMER3-001	APC membrane recruitment protein 3	protein_coding	9606	2:1307554	1
ENST00000471913	AMPH-004	amphiphysin	processed_transcript	9606	7:3839198	1
ENST00000450124	AMPH-006	amphiphysin	nonsense_mediated_decay	9606	7:3839198	1
ENST00000491800	AMT-010	aminomethyltransferase	retained_intron	9606	3:4941820	2
ENST00000584836	AMZ2-012	archaelysin family metallopeptidase 2	nonsense_mediated_decay	9606	17:682502	2
ENST00000541652	ANAPC5-016	anaphase promoting complex subunit 5	retained_intron	9606	12:121335	1
ENST00000522400	ANGPT1-008	angiopoietin 1	retained_intron	9606	8:1072845	2
ENST00000514160	ANK2-017	ankyrin 2, neuronal	retained_intron	9606	4:1133329	2
ENST00000504887	ANK2-018	ankyrin 2, neuronal	processed_transcript	9606	4:1133363	2
ENST00000360839	ANKHD1-001	ankyrin repeat and KH domain containing 1	protein_coding	9606	5:1404018	2
ENST00000504641	ANKRA2-003	ankyrin repeat, family A (RFXANK-like), 2	protein_coding	9606	5:7355490	2
ENST00000622129	ANKRD20A1-008	ankyrin repeat domain 20 family, member A1	retained_intron	9606	9:6788045	1
ENST00000428576	ANKRD20A11P-003	ankyrin repeat domain 20 family, member A11, pseudogene	processed_transcript	9606	21:139379	1
ENST00000498524	ANKRD28-006	ankyrin repeat domain 28	retained_intron	9606	3:1568374	1
ENST00000519316	ANKRD46-011	ankyrin repeat domain 46	protein_coding	9606	8:1005225	1
ENST00000529636	ANO1-009	anoctamin 1, calcium activated chloride channel	retained_intron	9606	11:701653	2
ENST00000529913	ANO1-010	anoctamin 1, calcium activated chloride channel	retained_intron	9606	11:701041	2
ENST00000617537	AOAH-001	acyloxyacyl hydrolase (neutrophil)	protein_coding	9606	7:3651299	1
ENST00000452500	AP000472.3-001		lincRNA	9606	21:219333	1
ENST00000438328	AP000949.1-001		processed_pseudogene	9606	21:228825	3
ENST00000401378	AP001623.1-201		miRNA	9606	21:423002	2
ENST00000401135	AP002884.1-201		miRNA	9606	11:112247	2
ENST00000590238	APBA3-005	amyloid beta (A4) precursor protein-binding, family A, member 3	retained_intron	9606	19:375389	1
ENST00000589298	APLP1-007	amyloid beta (A4) precursor-like protein 1	processed_transcript	9606	19:358745	2
ENST00000613677	APOBEC3H-202	apolipoprotein B mRNA editing enzyme, catalytic polypeptide-like 3H	protein_coding	9606	22:390972	1
ENST00000590334	APOC1-009	apolipoprotein C-I	nonsense_mediated_decay	9606	19:449148	2
ENST00000551662	APPL2-004	adaptor protein, phosphotyrosine interaction, PH domain and leucine zipper containing 2	protein_coding	9606	12:105174	1
ENST00000519531	ARFGAP1-014	ADP-ribosylation factor GTPase activating protein 1	retained_intron	9606	20:632728	1
ENST00000549047	ARFGAP1-021	ADP-ribosylation factor GTPase activating protein 1	protein_coding	9606	20:632728	2
ENST00000519273	ARFGAP1-201	ADP-ribosylation factor GTPase activating protein 1	protein_coding	9606	20:632727	2
ENST00000423813	ARFIP2-010	ADP-ribosylation factor interacting protein 2	protein_coding	9606	11:647681	1
ENST00000541078	ARHGDIA-012	Rho GDP dissociation inhibitor (GDI) alpha	protein_coding	9606	17:818683	1
ENST00000471928	ARHGEF19-006	Rho guanine nucleotide exchange factor (GEF) 19	processed_transcript	9606	1:16620562	1
ENST00000441785	ARHGEF19-007	Rho guanine nucleotide exchange factor (GEF) 19	protein_coding	9606	1:1620584	1
ENST00000471370	ARHGEF25-004	Rho guanine nucleotide exchange factor (GEF) 25	retained_intron	9606	12:576139	2
ENST00000409978	ARHGEF33-001	Rho guanine nucleotide exchange factor (GEF) 33	protein_coding	9606	2:3888988	1
ENST00000495939	ARHGEF3-AS1-002	ARHGEF3 antisense RNA 1	antisense	9606	3:5694007	2
ENST00000555038	ARHGEF40-004	Rho guanine nucleotide exchange factor (GEF) 40	protein_coding	9606	14:210702	1
ENST00000357866	ARL6IP4-011	ADP-ribosylation factor-like 6 interacting protein 4	protein_coding	9606	12:122981	4
ENST00000491358	ARL8A-002	ADP-ribosylation factor-like 8A	processed_transcript	9606	1:2021343	2
ENST00000481659	ARMC4-006	armadillo repeat containing 4	processed_transcript	9606	10:278121	2
ENST00000485396	ARMC8-005	armadillo repeat containing 8	protein_coding	9606	3:1381875	2
ENST00000486832	ARMC8-011	armadillo repeat containing 8	retained_intron	9606	3:1382380	1
ENST00000463485	ARMC8-014	armadillo repeat containing 8	protein_coding	9606	3:1381875	2
ENST00000445416	ARMCX4-004	armadillo repeat containing, X-linked 4	nonsense_mediated_decay	9606	X:1014852	1
ENST00000602449	ARMCX7P-002	armadillo repeat containing, X-linked 7, pseudogene	processed_transcript	9606	X:1015980	2
ENST00000485273	ARPC4-009	actin related protein 2/3 complex, subunit 4, 20kDa	protein_coding	9606	3:9793096	4
ENST00000419386	ARRDC1-006	arrestin domain containing 1	protein_coding	9606	9:1376057	1
ENST00000565165	ARSB-006	arylsulfatase B	protein_coding	9606	5:7881605	1
ENST00000504873	ARSK-004	arylsulfatase family, member K	nonsense_mediated_decay	9606	5:9555518	2

ENST00000459912	ASB13-006	ankyrin repeat and SOCS box containing 13	nonsense_mediated_decay	9606	10:563886 2
ENST00000522650	ASCC3-004	activating signal cointegrator 1 complex subunit 3	protein_coding	9606	6:1007151 1
ENST00000551199	ASIC1-004	acid-sensing (proton-gated) ion channel 1	retained_intron	9606	12:500707 2
ENST00000568305	ASIP-002	agouti signaling protein	protein_coding	9606	20:341945 2
ENST00000467695	ASS1-009	argininosuccinate synthase 1	retained_intron	9606	9:1304585 2
ENST00000361209	ASTN2-001	astrotactin 2	protein_coding	9606	9:1164252 1
ENST00000593235	ASXL3-013	additional sex combs like transcriptional regulator 3	nonsense_mediated_decay	9606	18:336056 2
ENST00000330083	ATAT1-004	alpha tubulin acetyltransferase 1	protein_coding	9606	6:3062688 1
ENST00000601323	ATCAY-009	ataxia, cerebellar, Cayman type	processed_transcript	9606	19:392528 2
ENST00000426833	ATF2-201	activating transcription factor 2	protein_coding	9606	2:1750722 2
ENST00000366985	ATF3-007	activating transcription factor 3	protein_coding	9606	1:2126089 2
ENST00000406122	ATL2-013	atlastin GTPase 2	protein_coding	9606	2:3829515 2
ENST00000418678	ATP11A-003	ATPase, class VI, type 11A	protein_coding	9606	13:112785 2
ENST00000480680	ATP2B2-008	ATPase, Ca++ transporting, plasma membrane 2	retained_intron	9606	3:1038788 1
ENST00000565546	ATP2C2-007	ATPase, Ca++ transporting, type 2C, member 2	processed_transcript	9606	16:844065 2
ENST00000620894	ATP5A1P10-001	ATP synthase, H+ transporting, mitochondrial F1 complex, alpha subunit 1 pseudogene 10	processed_pseudogene	9606	9:6672458 2
ENST00000505852	ATP5I-002	ATP synthase, H+ transporting, mitochondrial Fo complex, subunit E	processed_transcript	9606	4:672438-(2
ENST00000449556	ATP6AP1-010	ATPase, H+ transporting, lysosomal accessory protein 1	protein_coding	9606	X:1544286 2
ENST00000473485	ATP6V0B-002	ATPase, H+ transporting, lysosomal 21kDa, V0 subunit b	retained_intron	9606	1:4397497 1
ENST00000396774	ATP6V1H-005	ATPase, H+ transporting, lysosomal 50/57kDa, V1 subunit H	protein_coding	9606	8:5371573 2
ENST00000546483	ATXN2-020	ataxin 2	retained_intron	9606	12:111488 2
ENST00000489774	AUTS2-006	autism susceptibility candidate 2	processed_transcript	9606	7:7069487 2
ENST00000508229	AXDND1-010	axonemal dynein light chain domain containing 1	protein_coding	9606	1:1793657 1
ENST00000334100	AZI2-014	5-azacytidine induced 2	protein_coding	9606	3:2833168 2
ENST00000483027	AZIN2-011	antizyme inhibitor 2	processed_transcript	9606	1:3308116 2
ENST00000497280	AZIN2-016	antizyme inhibitor 2	processed_transcript	9606	1:3309324 2
ENST00000535680	B4GALNT3-002	beta-1,4-N-acetyl-galactosaminyl transferase 3	retained_intron	9606	12:460470 2
ENST00000598567	BABAM1-012	BRISC and BRCA1 A complex member 1	protein_coding	9606	19:172689 2
ENST00000598382	BABAM1-017	BRISC and BRCA1 A complex member 1	retained_intron	9606	19:172764 2
ENST00000453877	BACH2-007	BTB and CNC homology 1, basic leucine zipper transcription factor 2	protein_coding	9606	6:9000860 2
ENST00000530134	BAI2-017	brain-specific angiogenesis inhibitor 2	processed_transcript	9606	1:3173951 2
ENST00000393207	BANP-002	BTG3 associated nuclear protein	protein_coding	9606	16:879700 3
ENST00000573319	BCAR4-001	breast cancer anti-estrogen resistance 4 (non-protein coding)	lincRNA	9606	16:118198 1
ENST00000433098	BCL2L11-002	BCL2-like 11 (apoptosis facilitator)	nonsense_mediated_decay	9606	2:1111226 1
ENST00000378463	BCOR-008	BCL6 corepressor	protein_coding	9606	X:4004981 1
ENST00000441294	BCORL1-004	BCL6 corepressor-like 1	protein_coding	9606	X:1300144 1
ENST00000446746	BDH1-008	3-hydroxybutyrate dehydrogenase, type 1	protein_coding	9606	3:1975121 1
ENST00000547208	BEST3-011	bestrophin 3	nonsense_mediated_decay	9606	12:696433 2
ENST00000486280	BET1L-004	Bet1 golgi vesicular membrane trafficking protein-like	protein_coding	9606	11:205169 1
ENST00000528940	BIRC3-005	baculoviral IAP repeat containing 3	processed_transcript	9606	11:102317 4
ENST00000527309	BIRC3-006	baculoviral IAP repeat containing 3	protein_coding	9606	11:102325 2
ENST00000587722	BLOC1S3-002	biogenesis of lysosomal organelles complex-1, subunit 3	protein_coding	9606	19:451792 1
ENST00000439052	BNIP2-006	BCL2/adenovirus E1B 19kDa interacting protein 2	protein_coding	9606	15:596633 1
ENST00000603076	BNIP3P36-001	BCL2/adenovirus E1B 19kDa interacting protein 3 pseudogene 36	processed_pseudogene	9606	19:229949 1
ENST00000605211	BPIFA4P-004	BPI fold containing family A, member 4, pseudogene	retained_intron	9606	20:331935 1
ENST00000431406	BRD7P6-001	bromodomain containing 7 pseudogene 6	processed_pseudogene	9606	2:7035301 1
ENST00000449261	BRPF3-009	bromodomain and PHD finger containing, 3	nonsense_mediated_decay	9606	6:3619696 2
ENST00000383680	BTLA-002	B and T lymphocyte associated	protein_coding	9606	3:1124661 1
ENST00000476549	BTN3A1-002	butyrophilin, subfamily 3, member A1	protein_coding	9606	6:2640223 1
ENST00000370228	C10orf2-002	chromosome 10 open reading frame 2	protein_coding	9606	10:100988 1
ENST00000470158	C10orf88-003	chromosome 10 open reading frame 88	processed_transcript	9606	10:122932 2
ENST00000532565	C11orf80-001	chromosome 11 open reading frame 80	protein_coding	9606	11:667448 2
ENST00000532727	C11orf80-003	chromosome 11 open reading frame 80	nonsense_mediated_decay	9606	11:667590 2
ENST00000623009	C14orf182-201	chromosome 14 open reading frame 182	protein_coding	9606	14:499923 2
ENST00000321731	C14orf39-001	chromosome 14 open reading frame 39	protein_coding	9606	14:604359 1
ENST00000557138	C14orf39-003	chromosome 14 open reading frame 39	nonsense_mediated_decay	9606	14:604366 1
ENST00000548920	C14orf80-007	chromosome 14 open reading frame 80	nonsense_mediated_decay	9606	14:105491 2
ENST00000334656	C14orf80-201	chromosome 14 open reading frame 80	protein_coding	9606	14:105491 2
ENST00000321691	C17orf47-001	chromosome 17 open reading frame 47	protein_coding	9606	17:585415 1
ENST00000434650	C17orf62-001	chromosome 17 open reading frame 62	protein_coding	9606	17:824426 2
ENST00000600373	C19orf48-013	chromosome 19 open reading frame 48	protein_coding	9606	19:507983 2
ENST00000286031	C1orf112-001	chromosome 1 open reading frame 112	protein_coding	9606	1:1697954 2
ENST00000486384	C1orf131-008	chromosome 1 open reading frame 131	processed_transcript	9606	1:2312255 2
ENST00000369942	C1orf194-006	chromosome 1 open reading frame 194	retained_intron	9606	1:1091060 1
ENST00000445071	C1orf228-017	chromosome 1 open reading frame 228	protein_coding	9606	1:4467472 1
ENST00000493883	C21orf33-003	chromosome 21 open reading frame 33	processed_transcript	9606	21:441336 1
ENST00000479548	C21orf62-002	chromosome 21 open reading frame 62	protein_coding	9606	21:327937 2
ENST00000400023	C22orf34-001	chromosome 22 open reading frame 34	lincRNA	9606	22:496196 2
ENST00000518401	C2orf81-004	chromosome 2 open reading frame 81	protein_coding	9606	2:7441601 2
ENST00000478197	C2orf88-002	chromosome 2 open reading frame 88	processed_transcript	9606	2:1898796 2
ENST00000450357	C2orf88-007	chromosome 2 open reading frame 88	protein_coding	9606	2:1900895 2
ENST00000599899	C3-002	complement component 3	retained_intron	9606	19:667784 2
ENST00000594005	C3-009	complement component 3	retained_intron	9606	19:669661 1
ENST00000492792	C3orf30-005	chromosome 3 open reading frame 30	protein_coding	9606	3:1191469 1
ENST00000545770	C3orf84-002	chromosome 3 open reading frame 84	protein_coding	9606	3:4917763 1
ENST00000451804	C4BPB-202	complement component 4 binding protein, beta	protein_coding	9606	1:2070068 1
ENST00000503001	C4orf36-012	chromosome 4 open reading frame 36	processed_transcript	9606	4:8688814 1
ENST00000505296	C4orf6-004	chromosome 4 open reading frame 6	lincRNA	9606	4:5525390 2
ENST00000503655	C5orf34-005	chromosome 5 open reading frame 34	nonsense_mediated_decay	9606	5:4349354 2
ENST00000424426	C6orf141-003	chromosome 6 open reading frame 141	processed_transcript	9606	6:4955102 2
ENST00000624695	C7orf66-001	chromosome 7 open reading frame 66	lincRNA	9606	7:1088839 1
ENST00000615071	C8orf59-011	chromosome 8 open reading frame 59	retained_intron	9606	8:8521450 2
ENST00000526458	C9orf135-AS1-003	C9orf135 antisense RNA 1 (head to head)	lincRNA	9606	9:6981982 4
ENST00000520996	CA2-003	carbonic anhydrase II	retained_intron	9606	8:8546400 2
ENST00000517946	CAAP1-004	caspase activity and apoptosis inhibitor 1	processed_transcript	9606	9:2686992 2
ENST00000445422	CABIN1-003	calcineurin binding protein 1	protein_coding	9606	22:240114 1
ENST00000542233	CABP4-007	calcium binding protein 4	processed_transcript	9606	11:674527 2
ENST00000540581	CACFD1-201	calcium channel flower domain containing 1	protein_coding	9606	9:1334599 1
ENST00000544415	CACNA1C-AS4-001	CACNA1C antisense RNA 4	antisense	9606	12:222053 1
ENST00000612134	CACNB2-201	calcium channel, voltage-dependent, beta 2 subunit	protein_coding	9606	10:183409 1
ENST00000587175	CACTIN-002	cactin, spliceosome C complex subunit	retained_intron	9606	19:361156 2
ENST00000283269	CADPS-001	Ca++-dependent secretion activator	protein_coding	9606	3:6239897 2
ENST00000472895	CAMKV-016	CaM kinase-like vesicle-associated	retained_intron	9606	3:4986095 1

ENST00000357048	CAPN10-003	calpain 10	nonsense_mediated_decay	9606	2:2405868 1
ENST00000586851	CAPNS1-012	calpain, small subunit 1	protein_coding	9606	19:361411 4
ENST00000415932	CASC20-001	cancer susceptibility candidate 20 (non-protein coding)	lincRNA	9606	20:644672 2
ENST00000375890	CASP9-008	caspase 9, apoptosis-related cysteine peptidase	protein_coding	9606	1:1549285 1
ENST00000490131	CASR-001	calcium-sensing receptor	protein_coding	9606	3:1221836 1
ENST00000480532	CATIP-004	ciliogenesis associated TTC17 interacting protein	retained_intron	9606	2:2183568 2
ENST00000557036	CATSPERB-002	catsper channel auxiliary subunit beta	nonsense_mediated_decay	9606	14:915806 1
ENST00000346541	CBFA2T2-201	core-binding factor, runt domain, alpha subunit 2; translocated to, 2	protein_coding	9606	20:335623 1
ENST00000490323	CBWD2-005	COBW domain containing 2	retained_intron	9606	2:1134378 2
ENST00000467791	CBWD7-012	COBW domain containing 7	nonsense_mediated_decay	9606	9:4114531 2
ENST00000424120	CC2D2A-201	coiled-coil and C2 domain containing 2A	protein_coding	9606	4:1546986 1
ENST00000513730	CCDC112-006	coiled-coil domain containing 112	retained_intron	9606	5:1152753 2
ENST00000470137	CCDC122-002	coiled-coil domain containing 122	processed_transcript	9606	13:438239 2
ENST00000512432	CCDC149-008	coiled-coil domain containing 149	processed_transcript	9606	4:2483643 2
ENST00000434846	CCDC158-005	coiled-coil domain containing 158	protein_coding	9606	4:7637517 2
ENST00000239859	CCDC169-014	coiled-coil domain containing 169	protein_coding	9606	13:362311 2
ENST00000466180	CCDC24-008	coiled-coil domain containing 24	protein_coding	9606	1:4399184 4
ENST00000398814	CCDC33-001	coiled-coil domain containing 33	protein_coding	9606	15:742362 1
ENST00000574799	CCDC40-004	coiled-coil domain containing 40	retained_intron	9606	17:800483 2
ENST00000529611	CCDC90B-004	coiled-coil domain containing 90B	protein_coding	9606	11:832614 2
ENST00000531712	CCKBR-002	cholecystokinin B receptor	protein_coding	9606	11:625992 1
ENST00000486428	CCNC-002	cyclin C	protein_coding	9606	6:9954346 1
ENST00000496007	CCNL2-003	cyclin L2	retained_intron	9606	1:1386499 1
ENST00000452312	CCNYL1-004	cyclin Y-like 1	nonsense_mediated_decay	9606	2:2077121 2
ENST00000233836	CCT4P2-001	chaperonin containing TCP1, subunit 4 (delta) pseudogene 2	processed_pseudogene	9606	X:6527091 3
ENST00000593867	CD22-007	CD22 molecule	protein_coding	9606	19:353291 3
ENST00000598028	CD22-018	CD22 molecule	processed_transcript	9606	19:353291 1
ENST00000599799	CD22-023	CD22 molecule	retained_intron	9606	19:353291 1
ENST00000392122	CD247-001	CD247 molecule	protein_coding	9606	1:1674306 1
ENST00000368045	CD48-002	CD48 molecule	protein_coding	9606	1:1606835 1
ENST00000488054	CD96-005	CD96 molecule	nonsense_mediated_decay	9606	3:1115421 2
ENST00000442050	CDC123-002	cell division cycle 123	protein_coding	9606	10:121962 2
ENST00000478882	CDC20-003	cell division cycle 20	processed_transcript	9606	1:4335976 4
ENST00000537909	CDH22-201	cadherin 22, type 2	protein_coding	9606	20:461737 1
ENST00000604175	CDK20-009	cyclin-dependent kinase 20	retained_intron	9606	9:8797044 4
ENST00000357886	CDK5RAP1-011	CDK5 regulatory subunit associated protein 1	protein_coding	9606	20:333588 2
ENST00000471157	CDKN1C-004	cyclin-dependent kinase inhibitor 1C (p57, Kip2)	retained_intron	9606	11:288367 3
ENST00000531830	CDON-006	cell adhesion associated, oncogene regulated	nonsense_mediated_decay	9606	11:126005 2
ENST00000404744	CDPF1-004	cysteine-rich, DPF motif domain containing 1	protein_coding	9606	22:462440 2
ENST00000447168	CDY11P-001	chromodomain protein, Y-linked 11 pseudogene	unprocessed_pseudogene	9606	Y:2169657 1
ENST00000221992	CEACAM5-001	carcinoembryonic antigen-related cell adhesion molecule 5	protein_coding	9606	19:417086 2
ENST00000621209	CEL-202	carboxyl ester lipase	protein_coding	9606	9:1330619 1
ENST00000531165	CELF1-001	CUGBP, Elav-like family member 1	protein_coding	9606	11:474703 1
ENST00000475662	CENPA-003	centromere protein A	processed_transcript	9606	2:2676428 2
ENST00000509397	CENPK-016	centromere protein K	protein_coding	9606	5:6552460 2
ENST00000367710	CENPL-002	centromere protein L	protein_coding	9606	1:1737998 2
ENST00000491031	CENPO-006	centromere protein O	retained_intron	9606	2:2479340 1
ENST00000378223	CEP104-002	centrosomal protein 104kDa	protein_coding	9606	1:3843109 1
ENST00000560322	CEP152-002	centrosomal protein 152kDa	non_stop_decay	9606	15:487725 1
ENST00000520610	CEP57L1-019	centrosomal protein 57kDa-like 1	nonsense_mediated_decay	9606	6:1090955 2
ENST00000462419	CEP70-011	centrosomal protein 70kDa	protein_coding	9606	3:1385292 2
ENST00000551596	CEP83-012	centrosomal protein 83kDa	retained_intron	9606	12:944029 2
ENST00000463535	CERCAM-006	cerebral endothelial cell adhesion molecule	retained_intron	9606	9:1284240 2
ENST00000565403	CES1-006	carboxylesterase 1	retained_intron	9606	16:558203 2
ENST00000594141	CFAP221-012	cilia and flagella associated protein 221	protein_coding	9606	2:1195871 1
ENST00000369703	CFAP58-002	cilia and flagella associated protein 58	protein_coding	9606	10:104376 4
ENST00000424759	CFL1P7-001	cofilin 1 (non-muscle) pseudogene 7	processed_pseudogene	9606	3:2532417 3
ENST00000611287	CH17-3B23.1-201	Uncharacterized protein {ECO:0000313|Ensembl:ENSP00000476257}	protein_coding	9606	1:1208054 1
ENST00000621489	CH17-408M7.1-001		lincRNA	9606	1:1465144 2
ENST00000622911	CH507-254M2.1-005		lincRNA	9606	21:704891 1
ENST00000420239	CHD2-202	chromodomain helicase DNA binding protein 2	protein_coding	9606	15:929012 1
ENST00000564582	CHD9-009	chromodomain helicase DNA binding protein 9	processed_transcript	9606	16:530991 2
ENST00000554701	CHEK2P2-001	checkpoint kinase 2 pseudogene 2	transcribed_unprocessed_pseu	9606	15:202834 2
ENST00000534159	CHRDL2-004	chordin-like 2	processed_transcript	9606	11:747009 1
ENST00000468573	CHRM3-004	cholinergic receptor, muscarinic 3	processed_transcript	9606	1:2393865 2
ENST00000529467	CHRNA6-002	cholinergic receptor, nicotinic, alpha 6 (neuronal)	retained_intron	9606	8:4276497 2
ENST00000409046	CHST10-005	carbohydrate sulfotransferase 10	protein_coding	9606	2:1003979 2
ENST00000591410	CLASRP-010	CLK4-associating serine/arginine rich protein	nonsense_mediated_decay	9606	19:450390 4
ENST00000455251	CLCP1-001	Charcot-Leyden crystal protein pseudogene 1	processed_pseudogene	9606	22:174243 1
ENST00000481904	CLPSL2-003	colipase-like 2	processed_transcript	9606	6:3577659 2
ENST00000295911	CLRN1-003	clarin 1	protein_coding	9606	3:1509261 1
ENST00000544584	CLSTN3-012	calsyntenin 3	processed_transcript	9606	12:713868 2
ENST00000574210	CLUH-002	clustered mitochondria (cluA/CLU1) homolog	retained_intron	9606	17:269155 2
ENST00000519777	CNGB3-002	cyclic nucleotide gated channel beta 3	processed_transcript	9606	8:8670394 2
ENST00000478120	CNIH3-003	cornichon family AMPA receptor auxiliary protein 3	processed_transcript	9606	1:2245291 1
ENST00000484374	CNTLN-003	centlein, centrosomal protein	processed_transcript	9606	9:1713498 6
ENST00000471618	CNTNAP4-002	contactin associated protein-like 4	retained_intron	9606	16:763098 2
ENST00000431571	CNTRL-005	centriolin	protein_coding	9606	9:1211440 2
ENST00000545127	COA4-005	cytochrome c oxidase assembly factor 4 homolog (S. cerevisiae)	protein_coding	9606	11:738726 2
ENST00000395540	COBL-006	cordon-bleu WH2 repeat protein	protein_coding	9606	7:5106439 1
ENST00000345356	COL12A1-002	collagen, type XII, alpha 1	protein_coding	9606	6:7508647 2
ENST00000419671	COL12A1-007	collagen, type XII, alpha 1	protein_coding	9606	6:7513337 2
ENST00000471582	COL20A1-002	collagen, type XX, alpha 1	retained_intron	9606	20:633243 1
ENST00000479501	COL20A1-005	collagen, type XX, alpha 1	retained_intron	9606	20:632932 1
ENST00000522546	COL22A1-007	collagen, type XXII, alpha 1	protein_coding	9606	8:1387779 2
ENST00000512961	COL25A1-007	collagen, type XXV, alpha 1	protein_coding	9606	4:1088136 4
ENST00000534234	COPB1-003	coatomer protein complex, subunit beta 1	protein_coding	9606	11:144769 1
ENST00000421578	CORO1C-009	coronin, actin binding protein, 1C	protein_coding	9606	12:108647 1
ENST00000287490	COX6A2-001	cytochrome c oxidase subunit VIa polypeptide 2	protein_coding	9606	16:314277 1
ENST00000590900	COX6B2-005	cytochrome c oxidase subunit VIb polypeptide 2 (testis)	protein_coding	9606	19:553504 1
ENST00000587854	COX6B2-008	cytochrome c oxidase subunit VIb polypeptide 2 (testis)	retained_intron	9606	19:553541 1
ENST00000603667	COX6CP16-001	cytochrome c oxidase subunit VIc pseudogene 16	processed_pseudogene	9606	16:852788 2
ENST00000580869	COX6CP3-001	cytochrome c oxidase subunit VIc pseudogene 3	processed_pseudogene	9606	18:117679 1

ENST00000597056	COX6CP7-001	cytochrome c oxidase subunit VIc pseudogene 7	processed_pseudogene	9606	19:495024 1
ENST00000505291	COX7A2P2-001	cytochrome c oxidase subunit VIIa polypeptide 2 (liver) pseudogene 2	processed_pseudogene	9606	4:9690280 2
ENST00000460674	CP-002	ceruloplasmin (ferroxidase)	retained_intron	9606	3:1491724 4
ENST00000491460	CPA1-002	carboxypeptidase A1 (pancreatic)	retained_intron	9606	7:1303804 1
ENST00000492072	CPA4-009	carboxypeptidase A4	protein_coding	9606	7:1302931 4
ENST00000601637	CPAMD8-006	C3 and PZP-like, alpha-2-macroglobulin domain containing 8	retained_intron	9606	19:170002 2
ENST00000480404	CPE-005	carboxypeptidase E	nonsense_mediated_decay	9606	4:1653792 1
ENST00000515025	CPLX2-005	complexin 2	protein_coding	9606	5:1758782 1
ENST00000503204	CPNE4-005	copine IV	processed_transcript	9606	3:1315347 2
ENST00000559778	CPNE6-016	copine VI (neuronal)	protein_coding	9606	14:240710 1
ENST00000517742	CPQ-006	carboxypeptidase Q	protein_coding	9606	8:9664537 1
ENST00000419704	CPSF3L-009	cleavage and polyadenylation specific factor 3-like	protein_coding	9606	1:1311606 4
ENST00000437527	CPVL-011	carboxypeptidase, vitellogenic-like	protein_coding	9606	7:2912104 1
ENST00000459523	CR383657.1-201		miRNA	9606	14:184773 2
ENST00000424918	CR848007.5-001		processed_pseudogene	9606	9:4295928 1
ENST00000480086	CRB1-007	crumbs family member 1, photoreceptor morphogenesis associated	processed_transcript	9606	1:1974228 1
ENST00000598894	CREB3L3-006	cAMP responsive element binding protein 3-like 3	retained_intron	9606	19:416444 1
ENST00000489674	CRELD1-010	cysteine-rich with EGF-like domains 1	processed_transcript	9606	3:9941124 2
ENST00000566151	CRISPLD2-010	cysteine-rich secretory protein LCCL domain containing 2	protein_coding	9606	16:848199 2
ENST00000566789	CRISPLD2-012	cysteine-rich secretory protein LCCL domain containing 2	protein_coding	9606	16:848492 3
ENST00000566165	CRISPLD2-013	cysteine-rich secretory protein LCCL domain containing 2	nonsense_mediated_decay	9606	16:848892 2
ENST00000525823	CRYAB-008	crystallin, alpha B	protein_coding	9606	11:111908 1
ENST00000549143	CS-008	citrate synthase	nonsense_mediated_decay	9606	12:562716 2
ENST00000513609	CSF1R-006	colony stimulating factor 1 receptor	retained_intron	9606	5:1500597 2
ENST00000475259	CSF2RA-016	colony stimulating factor 2 receptor, alpha, low-affinity (granulocyte-macrophage)	processed_transcript	9606	X:1290453 2
ENST00000569462	CSK-016	c-src tyrosine kinase	protein_coding	9606	15:747826 2
ENST00000532460	CSRP1-004	cysteine and glycine-rich protein 1	protein_coding	9606	1:2014839 1
ENST00000533432	CSRP1-006	cysteine and glycine-rich protein 1	protein_coding	9606	1:2014843 1
ENST00000569048	CTA-481E9.4-001		lincRNA	9606	16:179331 2
ENST00000314099	CTAGE9-001	CTAGE family, member 9	protein_coding	9606	6:1317086 1
ENST00000467206	CTB-49A3.1-001		processed_pseudogene	9606	5:1331722 1
ENST00000526402	CTBP2P6-001	C-terminal binding protein 2 pseudogene 6	processed_pseudogene	9606	11:435220 2
ENST00000611331	CTC-242N15.1-001		lincRNA	9606	5:8720540 1
ENST00000603941	CTC-447K7.1-001		processed_pseudogene	9606	5:1553972 2
ENST00000587645	CTC-454I21.4-001		antisense	9606	19:371286 2
ENST00000562262	CTC-457E21.1-001		sense_intronic	9606	19:225209 3
ENST00000607580	CTC-527H23.4-001		lincRNA	9606	16:486404 1
ENST00000590626	CTC-548K16.5-001		antisense	9606	19:144027 2
ENST00000605503	CTD-2026C7.1-002		processed_transcript	9606	2:5775542 2
ENST00000559211	CTD-2034I4.1-001		lincRNA	9606	15:814274 2
ENST00000452067	CTD-2090I13.2-001		processed_pseudogene	9606	1:2274106 3
ENST00000602740	CTD-2118P12.1-005		lincRNA	9606	5:2916442 1
ENST00000503953	CTD-2154B17.1-001		lincRNA	9606	5:1077505 1
ENST00000506336	CTD-2195M15.1-001		processed_pseudogene	9606	5:1274658 2
ENST00000509174	CTD-2207L17.2-001	UDP-GlcNAc:betaGal beta-1,3-N-acetylglucosaminyltransferase-like 1 pseudogene 1	processed_pseudogene	9606	5:5288966 1
ENST00000606074	CTD-2228K2.7-004		retained_intron	9606	5:477245-(3
ENST00000603743	CTD-2269E23.3-001		processed_pseudogene	9606	5:2889778 1
ENST00000556081	CTD-2298J14.1-001		processed_pseudogene	9606	14:415556 1
ENST00000519677	CTD-2299I21.1-001		processed_pseudogene	9606	5:7900011 1
ENST00000568646	CTD-2336H13.1-001		processed_pseudogene	9606	16:768782 1
ENST00000567777	CTD-2336H13.2-001		lincRNA	9606	16:767363 1
ENST00000587088	CTD-2369P2.4-001		antisense	9606	19:102591 2
ENST00000484315	CTD-2509G16.1-001		processed_pseudogene	9606	14:652688 2
ENST00000586871	CTD-2527I21.4-002		processed_transcript	9606	19:351388 2
ENST00000601692	CTD-2527I21.9-001		sense_intronic	9606	19:350363 2
ENST00000523800	CTD-2544L4.1-001		processed_pseudogene	9606	8:1105554 1
ENST00000569456	CTD-2547G23.2-001		antisense	9606	16:252383 2
ENST00000450909	CTD-2574D22.2-001		antisense	9606	16:299268 2
ENST00000567344	CTD-2576D5.4-001		antisense	9606	16:171345 2
ENST00000448269	CTD-3105H18.10-001		unprocessed_pseudogene	9606	19:125046 1
ENST00000610398	CTD-3194G12.2-001		lincRNA	9606	17:367225 1
ENST00000600135	CTD-3233P19.7-001		unprocessed_pseudogene	9606	19:212250 4
ENST00000570828	CTDNEP1-012	CTD nuclear envelope phosphatase 1	protein_coding	9606	17:724359 2
ENST00000540488	CTNNA2-202	catenin (cadherin-associated protein), alpha 2	protein_coding	9606	2:8031337 1
ENST00000373735	CTNNA3-005	catenin (cadherin-associated protein), alpha 3	processed_transcript	9606	10:659201 3
ENST00000534579	CTNND1-029	catenin (cadherin-associated protein), delta 1	protein_coding	9606	11:577618 2
ENST00000606788	CTSA-023	cathepsin A	nonsense_mediated_decay	9606	20:458901 1
ENST00000534268	CTSH-011	cathepsin H	processed_transcript	9606	15:789373 2
ENST00000529612	CTSH-016	cathepsin H	retained_intron	9606	15:789276 2
ENST00000534237	CTSH-017	cathepsin H	processed_transcript	9606	15:789221 2
ENST00000514851	CTXN3-003	cortexin 3	processed_transcript	9606	5:1276530 1
ENST00000564948	CX3CL1-004	chemokine (C-X3-C motif) ligand 1	protein_coding	9606	16:573725 2
ENST00000395793	CXCL12-005	chemokine (C-X-C motif) ligand 12	protein_coding	9606	10:443726 1
ENST00000477281	CXorf36-002	chromosome X open reading frame 36	processed_transcript	9606	X:4514990 1
ENST00000370540	CXorf66-001	chromosome X open reading frame 66	protein_coding	9606	X:1399557 1
ENST00000392975	CYB561-005	cytochrome b561	protein_coding	9606	17:634323 2
ENST00000533558	CYB5R2-001	cytochrome b5 reductase 2	protein_coding	9606	11:766510 1
ENST00000541131	CYFIP2-201	cytoplasmic FMR1 interacting protein 2	protein_coding	9606	5:1572661 1
ENST00000374798	CYLC2-001	cylicin, basic protein of sperm head cytoskeleton 2	protein_coding	9606	9:1029953 4
ENST00000371270	CYP2C8-001	cytochrome P450, family 2, subfamily C, polypeptide 8	protein_coding	9606	10:950367 1
ENST00000594920	CYP2G2P-001	cytochrome P450, family 2, subfamily G, polypeptide 2 pseudogene	unprocessed_pseudogene	9606	19:410504 1
ENST00000589654	CYP4F2-007	cytochrome P450, family 4, subfamily F, polypeptide 2	protein_coding	9606	19:158786 1
ENST00000397485	CYP4F30P-003	cytochrome P450, family 4, subfamily F, polypeptide 30, pseudogene	processed_transcript	9606	2:1306801 1
ENST00000450723	CYP51A1-002	cytochrome P450, family 51, subfamily A, polypeptide 1	protein_coding	9606	7:9211341 2
ENST00000316161	CYP8B1-001	cytochrome P450, family 8, subfamily B, polypeptide 1	protein_coding	9606	3:4287219 2
ENST00000405206	CYTH4-009	cytohesin 4	protein_coding	9606	22:372824 1
ENST00000592838	DAB1-AS1-015	DAB1 antisense RNA 1	antisense	9606	1:5786245 2
ENST00000469067	DAPK1-007	death-associated protein kinase 1	retained_intron	9606	9:8749773 1
ENST00000559897	DAPK2-006	death-associated protein kinase 2	processed_transcript	9606	15:639392 1
ENST00000514301	DAPP1-004	dual adaptor of phosphotyrosine and 3-phosphoinositides	retained_intron	9606	4:9986487 1
ENST00000406071	DCDC1-201	doublecortin domain containing 1	protein_coding	9606	11:308636 2
ENST00000503182	DCTD-006	dCMP deaminase	protein_coding	9606	4:1828914 2
ENST00000409567	DCTN1-002	dynactin 1	protein_coding	9606	2:7436149 1

ENST00000547345	DCTN2-013	dynactin 2 (p50)	retained_intron	9606	12:575349	1
ENST00000542129	DDX11-005	DEAD/H (Asp-Glu-Ala-Asp/His) box helicase 11	nonsense_mediated_decay	9606	12:310738	1
ENST00000543026	DDX11-014	DEAD/H (Asp-Glu-Ala-Asp/His) box helicase 11	retained_intron	9606	12:310876	2
ENST00000431908	DDX39B-019	DEAD (Asp-Glu-Ala-Asp) box polypeptide 39B	protein_coding	9606	6:3153130	4
ENST00000463199	DDX3Y-007	DEAD (Asp-Glu-Ala-Asp) box helicase 3, Y-linked	processed_transcript	9606	Y:1291300	1
ENST00000610822	DDX50-201	DEAD (Asp-Glu-Ala-Asp) box polypeptide 50	protein_coding	9606	10:689012	2
ENST00000519410	DECR1-008	2,4-dienoyl CoA reductase 1, mitochondrial	protein_coding	9606	8:9000147	2
ENST00000468102	DEF6-003	differentially expressed in FDCP 6 homolog (mouse)	processed_transcript	9606	6:3531760	2
ENST00000494124	DENND4C-009	DENN/MADD domain containing 4C	nonsense_mediated_decay	9606	9:1929075	4
ENST00000477548	DFFB-003	DNA fragmentation factor, 40kDa, beta polypeptide (caspase-activated DNase)	nonsense_mediated_decay	9606	1:3857473	1
ENST00000504396	DHFR-003	dihydrofolate reductase	protein_coding	9606	5:8062822	1
ENST00000558114	DHRS1-009	dehydrogenase/reductase (SDR family) member 1	retained_intron	9606	14:242987	2
ENST00000559483	DHRS1-010	dehydrogenase/reductase (SDR family) member 1	processed_transcript	9606	14:242922	2
ENST00000461948	DHRS12-005	dehydrogenase/reductase (SDR family) member 12	protein_coding	9606	13:517727	2
ENST00000591220	DHX58-009	DEXH (Asp-Glu-X-His) box polypeptide 58	protein_coding	9606	17:421107	2
ENST00000398557	DIAPH1-203	diaphanous-related formin 1	protein_coding	9606	5:1415150	1
ENST00000525044	DIO1-006	deiodinase, iodothyronine, type I	nonsense_mediated_decay	9606	1:5389418	2
ENST00000466140	DLG3-007	discs, large homolog 3 (Drosophila)	processed_transcript	9606	X:7047911	2
ENST00000582054	DLGAP1-AS4-001	DLGAP1 antisense RNA 4	antisense	9606	18:396235	1
ENST00000555071	DLST-003	dihydrolipoamide S-succinyltransferase (E2 component of 2-oxo-glutarate complex)	retained_intron	9606	14:748819	1
ENST00000470964	DMKN-060	dermokine	retained_intron	9606	19:354982	3
ENST00000532817	DNAAF3-014	dynein, axonemal, assembly factor 3	protein_coding	9606	19:551622	1
ENST00000406473	DNAJA1P4-001	DnaJ (Hsp40) homolog, subfamily A, member 1 pseudogene 4	processed_pseudogene	9606	6:1143494	1
ENST00000437347	DNAJA1P6-001	DnaJ (Hsp40) homolog, subfamily A, member 1 pseudogene 6	processed_pseudogene	9606	22:175186	1
ENST00000476396	DNAJB4-002	DnaJ (Hsp40) homolog, subfamily B, member 4	processed_transcript	9606	1:7800493	1
ENST00000559310	DNAJC17-006	DnaJ (Hsp40) homolog, subfamily C, member 17	nonsense_mediated_decay	9606	15:407743	2
ENST00000560065	DNAJC17-011	DnaJ (Hsp40) homolog, subfamily C, member 17	nonsense_mediated_decay	9606	15:407742	1
ENST00000402462	DNAJC5G-002	DnaJ (Hsp40) homolog, subfamily C, member 5 gamma	protein_coding	9606	2:2727546	4
ENST00000406962	DNAJC5G-003	DnaJ (Hsp40) homolog, subfamily C, member 5 gamma	protein_coding	9606	2:2727547	1
ENST00000463694	DNASE1L3-004	deoxyribonuclease I-like 3	retained_intron	9606	3:5820776	1
ENST00000463998	DNM1-011	dynamin 1	retained_intron	9606	9:1282335	1
ENST00000373966	DNPEP-003	aspartyl aminopeptidase	nonsense_mediated_decay	9606	2:2193741	1
ENST00000614472	DOCK7-202	dedicator of cytokinesis 7	protein_coding	9606	1:6254372	1
ENST00000478380	DOCK8-011	dedicator of cytokinesis 8	retained_intron	9606	9:273066-3	1
ENST00000418517	DPF1-006	D4, zinc and double PHD fingers family 1	nonsense_mediated_decay	9606	19:382117	4
ENST00000610283	DPF3-202	D4, zinc and double PHD fingers, family 3	protein_coding	9606	14:726610	1
ENST00000559233	DPP8-011	dipeptidyl-peptidase 8	protein_coding	9606	15:654467	3
ENST00000463966	DPPA4-002	developmental pluripotency associated 4	processed_transcript	9606	3:1093275	1
ENST00000487299	DPPA4-008	developmental pluripotency associated 4	retained_intron	9606	3:1093290	2
ENST00000605269	DPRXP2-001	divergent-paired related homeobox pseudogene 2	processed_pseudogene	9606	6:3598951	1
ENST00000344624	DROSHA-201	drosha, ribonuclease type III	protein_coding	9606	5:3140050	2
ENST00000422749	DSCAM-AS1-002	DSCAM antisense RNA 1	antisense	9606	21:403830	1
ENST00000495344	DSCR8-004	Down syndrome critical region gene 8	processed_transcript	9606	21:381214	2
ENST00000585206	DSG2-002	desmoglein 2	protein_coding	9606	18:314982	1
ENST00000456590	DTX2-011	deltex 2, E3 ubiquitin ligase	protein_coding	9606	7:7646169	2
ENST00000549583	DTX3-005	deltex 3, E3 ubiquitin ligase	protein_coding	9606	12:576046	2
ENST00000593229	DUS3L-009	dihydrouridine synthase 3-like (S. cerevisiae)	retained_intron	9606	19:578755	2
ENST00000601696	DYRK1B-004	dual-specificity tyrosine-(Y)-phosphorylation regulated kinase 1B	retained_intron	9606	19:398284	4
ENST00000367108	DYRK3-002	dual-specificity tyrosine-(Y)-phosphorylation regulated kinase 3	protein_coding	9606	1:2066355	2
ENST00000490477	EAF2-005	ELL associated factor 2	nonsense_mediated_decay	9606	3:1218352	2
ENST00000518323	EBF1-007	early B-cell factor 1	processed_transcript	9606	5:1586986	3
ENST00000536120	ECHDC2-201	enoyl CoA hydratase domain containing 2	protein_coding	9606	1:5289591	1
ENST00000252440	ECSIT-004	ECSIT signalling integrator	protein_coding	9606	19:115059	4
ENST00000512363	EEF1A1P21-001	eukaryotic translation elongation factor 1 alpha 1 pseudogene 21	processed_pseudogene	9606	4:2974875	2
ENST00000476600	EFCAB6-007	EF-hand calcium binding domain 6	processed_transcript	9606	22:437653	4
ENST00000618684	EFCAB8-201	EF-hand calcium binding domain 8	protein_coding	9606	20:328637	1
ENST00000511230	EGFEM1P-003	EGF-like and EMI domain containing 1, pseudogene	processed_transcript	9606	3:1682496	1
ENST00000502332	EGFEM1P-004	EGF-like and EMI domain containing 1, pseudogene	processed_transcript	9606	3:1682495	1
ENST00000405289	EHBP1-007	EH domain binding protein 1	protein_coding	9606	2:6270719	2
ENST00000421436	EIF1P1-001	eukaryotic translation initiation factor 1 pseudogene 1	processed_pseudogene	9606	9:659986-6	1
ENST00000461493	EIF2AK1-005	eukaryotic translation initiation factor 2-alpha kinase 1	retained_intron	9606	7:6049784	2
ENST00000395587	EIF3C-201	eukaryotic translation initiation factor 3, subunit C	protein_coding	9606	16:287114	1
ENST00000502290	ELMOD2-008	ELMO/CED-12 domain containing 2	retained_intron	9606	4:1405424	2
ENST00000590700	ELOF1-006	elongation factor 1 homolog (S. cerevisiae)	protein_coding	9606	19:115535	2
ENST00000505896	EMB-002	embigin	retained_intron	9606	5:5039802	1
ENST00000589876	EML2-008	echinoderm microtubule associated protein like 2	protein_coding	9606	19:456069	1
ENST00000394776	EML3-002	echinoderm microtubule associated protein like 3	protein_coding	9606	11:626022	2
ENST00000601198	EMR1-007	egf-like module containing, mucin-like, hormone receptor-like 1	processed_transcript	9606	19:689115	2
ENST00000616794	EMX2-002	empty spiracles homeobox 2	protein_coding	9606	10:117543	1
ENST00000546446	EMX2-003	empty spiracles homeobox 2	processed_transcript	9606	10:117544	2
ENST00000439162	ENTPD6-010	ectonucleoside triphosphate diphosphohydrolase 6 (putative)	protein_coding	9606	20:251957	2
ENST00000373945	EPB41L1-005	erythrocyte membrane protein band 4.1-like 1	protein_coding	9606	20:361547	1
ENST00000451082	EPB41L1-006	erythrocyte membrane protein band 4.1-like 1	protein_coding	9606	20:361907	2
ENST00000342933	EPB41L3-201	erythrocyte membrane protein band 4.1-like 3	protein_coding	9606	18:539238	1
ENST00000444950	EPHA10-007	EPH receptor A10	retained_intron	9606	1:3771389	1
ENST00000389672	EPHA6-002	EPH receptor A6	protein_coding	9606	3:9681458	1
ENST00000488154	EPHB1-009	EPH receptor B1	processed_transcript	9606	3:1347956	4
ENST00000609181	EPRS-007	glutamyl-prolyl-tRNA synthetase	protein_coding	9606	1:2199872	2
ENST00000540810	EPS8L1-004	EPS8-like 1	protein_coding	9606	19:550759	2
ENST00000537828	EPSTI1-008	epithelial stromal interaction 1 (breast)	protein_coding	9606	13:429263	1
ENST00000551767	EPYC-002	epiphycan	protein_coding	9606	12:909718	1
ENST00000380032	EQTN-001	equatorin, sperm acrosome associated	protein_coding	9606	9:2728465	1
ENST00000468118	ERC2-010	ELKS/RAB6-interacting/CAST family member 2	processed_transcript	9606	3:5551090	2
ENST00000587376	ERCC2-004	excision repair cross-complementation group 2	nonsense_mediated_decay	9606	19:453532	4
ENST00000404742	ESR1-004	estrogen receptor 1	protein_coding	9606	6:1516904	4
ENST00000554520	ESR2-006	estrogen receptor 2 (ER beta)	processed_transcript	9606	14:642330	1
ENST00000444817	ETNK2-007	ethanolamine kinase 2	protein_coding	9606	1:2041372	4
ENST00000510706	ETNPPL-001	ethanolamine-phosphate phospho-lyase	protein_coding	9606	4:1087424	1
ENST00000509451	EVC-002	Ellis van Creveld syndrome	protein_coding	9606	4:5711215	1
ENST00000490886	EXD3-012	exonuclease 3'-5' domain containing 3	processed_transcript	9606	9:1373539	2
ENST00000580616	EXOGP1-001	endo/exonuclease (5'-3'), endonuclease G-like pseudogene 1	processed_pseudogene	9606	18:211825	2
ENST00000592743	EZH1-002	enhancer of zeste 1 polycomb repressive complex 2 subunit	protein_coding	9606	17:427023	1
ENST00000498455	F10-004	coagulation factor X	retained_intron	9606	13:113140	1

ENST00000414279	F13A1-003	coagulation factor XIII, A1 polypeptide	protein_coding	9606	6:6266713 2
ENST00000479211	F13A1-005	coagulation factor XIII, A1 polypeptide	retained_intron	9606	6:6250907 2
ENST00000369505	F8A2-001	coagulation factor VIII-associated 2	protein_coding	9606	X:1553821 1
ENST00000394090	F9-201	coagulation factor IX	protein_coding	9606	X:1395307 1
ENST00000482018	FABP3-003	fatty acid binding protein 3, muscle and heart (mammary-derived growth inhibitor)	protein_coding	9606	1:3136589 1
ENST00000515543	FABP5P12-001	fatty acid binding protein 5 pseudogene 12	processed_pseudogene	9606	4:1590261 3
ENST00000487898	FAF1-004	Fas (TNFRSF6) associated factor 1	processed_transcript	9606	1:5070578 4
ENST00000463473	FAIM3-003	Fas apoptotic inhibitory molecule 3	nonsense_mediated_decay	9606	1:2069048 1
ENST00000291129	FAM115D-001	family with sequence similarity 115, member D (pseudogene)	unprocessed_pseudogene	9606	7:1438007 1
ENST00000500765	FAM13A-AS1-002	FAM13A antisense RNA 1	antisense	9606	4:8870978 1
ENST00000503146	FAM160A1-004	family with sequence similarity 160, member A1	protein_coding	9606	4:1514092 2
ENST00000511501	FAM160A1-005	family with sequence similarity 160, member A1	processed_transcript	9606	4:1514093 2
ENST00000442525	FAM170B-AS1-001	FAM170B antisense RNA 1	antisense	9606	10:491218 1
ENST00000555064	FAM174B-007	family with sequence similarity 174, member B	protein_coding	9606	15:926190 2
ENST00000410025	FAM183A-002	family with sequence similarity 183, member A	nonsense_mediated_decay	9606	1:4314791 5
ENST00000257515	FAM189A2-002	family with sequence similarity 189, member A2	protein_coding	9606	9:6932932 1
ENST00000551619	FAM19A2-003	family with sequence similarity 19 (chemokine (C-C motif)-like), member A2	protein_coding	9606	12:617082 2
ENST00000550396	FAM19A2-007	family with sequence similarity 19 (chemokine (C-C motif)-like), member A2	processed_transcript	9606	12:622587 3
ENST00000547919	FAM19A2-014	family with sequence similarity 19 (chemokine (C-C motif)-like), member A2	processed_transcript	9606	12:622068 1
ENST00000464043	FAM213B-009	family with sequence similarity 213, member B	processed_transcript	9606	1:2586817 2
ENST00000369641	FAM3A-012	family with sequence similarity 3, member A	protein_coding	9606	X:1545065 1
ENST00000571269	FAM57B-009	family with sequence similarity 57, member B	protein_coding	9606	16:300354 5
ENST00000435741	FAM58CP-001	family with sequence similarity 58, member C, pseudogene	processed_pseudogene	9606	Y:2662652 1
ENST00000566559	FAM65A-005	family with sequence similarity 65, member A	protein_coding	9606	16:675380 2
ENST00000566522	FAM65A-010	family with sequence similarity 65, member A	processed_transcript	9606	16:675288 2
ENST00000477515	FAM71F2-005	family with sequence similarity 71, member F2	protein_coding	9606	7:1286722 2
ENST00000345041	FAM83G-201	family with sequence similarity 83, member G	protein_coding	9606	17:189710 1
ENST00000474949	FANCC-005	Fanconi anemia, complementation group C	processed_transcript	9606	9:9517111 1
ENST00000431771	FBLIM1-007	filamin binding LIM protein 1	protein_coding	9606	1:1575879 1
ENST00000460538	FBLN1-013	fibulin 1	retained_intron	9606	22:455328 1
ENST00000465578	FBLN1-014	fibulin 1	retained_intron	9606	22:455331 1
ENST00000440407	FBXL15-006	F-box and leucine-rich repeat protein 15	protein_coding	9606	10:102420 2
ENST00000224862	FBXL15-201	F-box and leucine-rich repeat protein 15	protein_coding	9606	10:102419 1
ENST00000471231	FBXL19-004	F-box and leucine-rich repeat protein 19	protein_coding	9606	16:309230 1
ENST00000467490	FBXL21-004	F-box and leucine-rich repeat protein 21 (gene/pseudogene)	processed_transcript	9606	5:1359304 1
ENST00000478089	FBXO42-002	F-box protein 42	processed_transcript	9606	1:1629231 1
ENST00000251546	FBXO44-002	F-box protein 44	protein_coding	9606	1:1165437 1
ENST00000513696	FBXO8-004	F-box protein 8	protein_coding	9606	4:1742411 2
ENST00000604872	FBXW7-004	F-box and WD repeat domain containing 7, E3 ubiquitin protein ligase	protein_coding	9606	4:1524111 1
ENST00000496394	FCF1P3-001	FCF1 pseudogene 3	processed_pseudogene	9606	3:1068486 1
ENST00000367972	FCGR2A-002	Fc fragment of IgG, low affinity IIa, receptor (CD32)	protein_coding	9606	1:1615054 1
ENST00000349527	FCRLA-007	Fc receptor-like A	protein_coding	9606	1:1617072 2
ENST00000425091	FDPSP6-001	farnesyl diphosphate synthase pseudogene 6	processed_pseudogene	9606	21:203883 2
ENST00000440443	FER1L4-003	fer-1-like family member 4, pseudogene (functional)	processed_transcript	9606	20:355761 2
ENST00000412452	FER1L4-012	fer-1-like family member 4, pseudogene (functional)	retained_intron	9606	20:355997 2
ENST00000395631	FERMT2-002	fermitin family member 2	protein_coding	9606	14:528572 2
ENST00000343279	FERMT2-201	fermitin family member 2	protein_coding	9606	14:528572 2
ENST00000551984	FGD4-014	FYVE, RhoGEF and PH domain containing 4	nonsense_mediated_decay	9606	12:325021 2
ENST00000610990	FGF1-201	fibroblast growth factor 1 (acidic)	protein_coding	9606	5:1425921 3
ENST00000607251	FGF14-IT1-003	FGF14 intronic transcript 1 (non-protein coding)	sense_intronic	9606	13:102292 1
ENST00000619564	FGFR1-204	fibroblast growth factor receptor 1	protein_coding	9606	8:3841953 1
ENST00000491111	FGFR2-013	fibroblast growth factor receptor 2	processed_transcript	9606	10:121583 2
ENST00000507708	FGFR4-011	fibroblast growth factor receptor 4	processed_transcript	9606	5:1770869 2
ENST00000508139	FGFR4-013	fibroblast growth factor receptor 4	retained_intron	9606	5:1770917 2
ENST00000430447	FGGY-004	FGGY carbohydrate kinase domain containing	nonsense_mediated_decay	9606	1:5929701 1
ENST00000459961	FHAD1-009	forkhead-associated (FHA) phosphopeptide binding domain 1	retained_intron	9606	1:1532446 2
ENST00000533045	FIBP-007	fibroblast growth factor (acidic) intracellular binding protein	protein_coding	9606	11:658839 1
ENST00000620828	FMR1-013	fragile X mental retardation 1	retained_intron	9606	X:1479276 2
ENST00000370471	FMR1-202	fragile X mental retardation 1	protein_coding	9606	X:1479119 1
ENST00000613724	FMR1-AS1_2.1-201	FMR1 antisense RNA 1 conserved region 2	misc_RNA	9606	X:1479118 2
ENST00000480856	FNDC1-003	fibronectin type III domain containing 1	processed_transcript	9606	6:1592182 1
ENST00000555242	FOS-008	FBJ murine osteosarcoma viral oncogene homolog	protein_coding	9606	14:752789 2
ENST00000361953	FOXM1-001	forkhead box M1	protein_coding	9606	12:285768 2
ENST00000423577	FPGS-001	folylpolyglutamate synthase	protein_coding	9606	9:1278033 4
ENST00000479147	FPGS-015	folylpolyglutamate synthase	processed_transcript	9606	9:1277945 2
ENST00000443452	FUNDC2P2-002	FUN14 domain containing 2 pseudogene 2	transcribed_processed_pseudo	9606	2:8429077 2
ENST00000618099	FURIN-202	furin (paired basic amino acid cleaving enzyme)	protein_coding	9606	15:908728 1
ENST00000592563	FUT6-011	fucosyltransferase 6 (alpha (1,3) fucosyltransferase)	protein_coding	9606	19:583137 1
ENST00000604504	FXYD3-006	FXYD domain containing ion transport regulator 3	retained_intron	9606	19:351163 3
ENST00000342879	FXYD5-001	FXYD domain containing ion transport regulator 5	protein_coding	9606	19:351547 1
ENST00000484067	FYN-018	FYN proto-oncogene, Src family tyrosine kinase	protein_coding	9606	6:1117143 1
ENST00000517911	FZD3-003	frizzled class receptor 3	processed_transcript	9606	8:2856306 2
ENST00000395095	FZR1-002	fizzy/cell division cycle 20 related 1 (Drosophila)	protein_coding	9606	19:352299 2
ENST00000570803	GAA-005	glucosidase, alpha; acid	protein_coding	9606	17:801015 2
ENST00000557449	GABRA5-006	gamma-aminobutyric acid (GABA) A receptor, alpha 5	processed_transcript	9606	15:268672 2
ENST00000274545	GABRA6-001	gamma-aminobutyric acid (GABA) A receptor, alpha 6	protein_coding	9606	5:1616855 1
ENST00000557765	GABRB3-014	gamma-aminobutyric acid (GABA) A receptor, beta 3	retained_intron	9606	15:265611 2
ENST00000295452	GABRG1-001	gamma-aminobutyric acid (GABA) A receptor, gamma 1	protein_coding	9606	4:4603576 1
ENST00000445006	GAD1-008	glutamate decarboxylase 1 (brain, 67kDa)	protein_coding	9606	2:1708161 3
ENST00000456864	GAD1-010	glutamate decarboxylase 1 (brain, 67kDa)	protein_coding	9606	2:1708177 1
ENST00000428682	GAL3ST1-009	galactose-3-O-sulfotransferase 1	protein_coding	9606	22:305559 2
ENST00000393569	GALC-002	galactosylceramidase	protein_coding	9606	14:879343 1
ENST00000393568	GALC-005	galactosylceramidase	protein_coding	9606	14:879346 2
ENST00000472111	GALT-009	galactose-1-phosphate uridylyltransferase	retained_intron	9606	9:3464666 2
ENST00000436626	GAPDHP48-001	glyceraldehyde 3 phosphate dehydrogenase pseudogene 48	processed_pseudogene	9606	2:3688021 2
ENST00000433910	GAPDHP75-001	glyceraldehyde-3-phosphate dehydrogenase pseudogene 75	processed_pseudogene	9606	1:1891323 4
ENST00000469528	GAPVD1-018	GTPase activating protein and VPS9 domains 1	processed_transcript	9606	9:1252618 2
ENST00000464616	GARNL3-015	GTPase activating Rap/RanGAP domain-like 3	processed_transcript	9606	9:1272650 2
ENST00000478124	GARS-002	glycyl-tRNA synthetase	retained_intron	9606	7:3059488 1
ENST00000542249	GAS7-010	growth arrest-specific 7	protein_coding	9606	17:991683 1
ENST00000414723	GCA-010	grancalcin, EF-hand calcium binding protein	protein_coding	9606	2:1623564 1
ENST00000409896	GCC2-003	GRIP and coiled-coil domain containing 2	protein_coding	9606	2:1084498 1
ENST00000497568	GCG-003	glucagon	retained_intron	9606	2:1621446 2

ENST00000376986	GDA-002	guanine deaminase	protein_coding	9606	9:7214945 1
ENST00000445417	GEMIN8P3-001	gem (nuclear organelle) associated protein 8 pseudogene 3	processed_pseudogene	9606	X:8630436 1
ENST00000518906	GFPT2-005	glutamine-fructose-6-phosphate transaminase 2	protein_coding	9606	5:1803188 1
ENST00000566685	GGA2-016	golgi-associated, gamma adaptin ear containing, ARF binding protein 2	processed_transcript	9606	16:234666 2
ENST00000573591	GGT6-004	gamma-glutamyltransferase 6	protein_coding	9606	17:455782 1
ENST00000618759	GIMAP6-201	GTPase, IMAP family member 6	protein_coding	9606	7:1506253 1
ENST00000486459	GK5-013	glycerol kinase 5 (putative)	protein_coding	9606	3:1421701 2
ENST00000481498	GKN2-003	gastrokine 2	protein_coding	9606	2:6894529 1
ENST00000532985	GLB1L3-008	galactosidase, beta 1-like 3	retained_intron	9606	11:134287 2
ENST00000477960	GLDC-003	glycine dehydrogenase (decarboxylating)	processed_transcript	9606	9:6532899 2
ENST00000559317	GLDN-009	gliomedin	retained_intron	9606	15:513602 2
ENST00000490709	GLIS3-001	GLIS family zinc finger 3	processed_transcript	9606	9:4118530 2
ENST00000546851	GLT8D2-004	glycosyltransferase 8 domain containing 2	protein_coding	9606	12:103999 2
ENST00000599582	GLTSCR2-007	glioma tumor suppressor candidate region gene 2	processed_transcript	9606	19:477458 2
ENST00000319701	GM140-001	uncharacterized LOC100287948	lincRNA	9606	1:1812363 1
ENST00000534441	GMDS-AS1-010	GMDS antisense RNA 1 (head to head)	lincRNA	9606	6:2245842 2
ENST00000220940	GML-001	glycosylphosphatidylinositol anchored molecule like	protein_coding	9606	8:1428348 2
ENST00000492538	GNAZ-003	guanine nucleotide binding protein (G protein), alpha z polypeptide	processed_transcript	9606	22:230705 2
ENST00000503610	GOLPH3-004	golgi phosphoprotein 3 (coat-protein)	nonsense_mediated_decay	9606	5:3212636 2
ENST00000620426	GON4L-203	gon-4-like (C. elegans)	protein_coding	9606	1:1557503 3
ENST00000471559	GORASP2-004	golgi reassembly stacking protein 2, 55kDa	processed_transcript	9606	2:1709284 1
ENST00000471757	GPAT2-002	glycerol-3-phosphate acyltransferase 2, mitochondrial	retained_intron	9606	2:9602203 2
ENST00000468080	GPM6B-011	glycoprotein M6B	protein_coding	9606	X:1377990 2
ENST00000487890	GPNMB-006	glycoprotein (transmembrane) nmb	processed_transcript	9606	7:2324676 2
ENST00000507065	GPR111-003	G protein-coupled receptor 111	protein_coding	9606	6:4765643 1
ENST00000502482	GPR125-014	G protein-coupled receptor 125	protein_coding	9606	4:2242003 1
ENST00000543826	GPR133-009	G protein-coupled receptor 133	processed_transcript	9606	12:130953 2
ENST00000482813	GPR160-006	G protein-coupled receptor 160	processed_transcript	9606	3:1700381 2
ENST00000624973	GPR1-AS-002	GPR1 antisense RNA	retained_intron	9606	2:2062594 2
ENST00000534502	GPR89A-006	G protein-coupled receptor 89A	protein_coding	9606	1:1456080 1
ENST00000589115	GPX4-007	glutathione peroxidase 4	protein_coding	9606	19:110400 1
ENST00000562288	GRAMD2-006	GRAM domain containing 2	nonsense_mediated_decay	9606	15:721678 2
ENST00000564773	GRAMD2-011	GRAM domain containing 2	retained_intron	9606	15:721619 2
ENST00000567029	GRAMD2-012	GRAM domain containing 2	retained_intron	9606	15:721618 1
ENST00000578961	GRB2-008	growth factor receptor-bound protein 2	protein_coding	9606	17:753203 2
ENST00000473071	GRB7-007	growth factor receptor-bound protein 7	retained_intron	9606	17:397432 2
ENST00000584853	GRB7-010	growth factor receptor-bound protein 7	processed_transcript	9606	17:397379 4
ENST00000461318	GRHL3-004	grainyhead-like 3 (Drosophila)	retained_intron	9606	1:2434216 2
ENST00000602210	GRIK5-006	glutamate receptor, ionotropic, kainate 5	retained_intron	9606	19:419992 2
ENST00000619107	GRIPAP1-010	GRIP1 associated protein 1	protein_coding	9606	X:4897625 2
ENST00000445675	GRM7-AS3-001	GRM7 antisense RNA 3	lincRNA	9606	3:6631900 1
ENST00000588170	GRN-012	granulin	retained_intron	9606	17:443452 1
ENST00000414377	GS1-115G20.1-001		sense_intronic	9606	1:1844083 1
ENST00000422795	GS1-542M4.3-001		processed_pseudogene	9606	X:8856665 2
ENST00000464556	GSDMB-006	gasdermin B	retained_intron	9606	17:399094 2
ENST00000520542	GSDMB-010	gasdermin B	protein_coding	9606	17:399045 2
ENST00000521365	GSDMC-003	gasdermin C	retained_intron	9606	8:1297517 2
ENST00000438425	GSTA7P-001	glutathione S-transferase alpha 7, pseudogene	unprocessed_pseudogene	9606	6:5273959 1
ENST00000622534	GTF3C2-202	general transcription factor IIIC, polypeptide 2, beta 110kDa	protein_coding	9606	2:2733360 2
ENST00000470386	GTF3C3-011	general transcription factor IIIC, polypeptide 3, 102kDa	processed_transcript	9606	2:1967854 4
ENST00000483839	GTPBP4-005	GTP binding protein 4	processed_transcript	9606	10:101240 4
ENST00000492832	GUSBP2-001	glucuronidase, beta pseudogene 2	transcribed_unprocessed_pseu	9606	6:2687857 2
ENST00000446960	H2AFZP5-001	H2A histone family, member Z pseudogene 5	processed_pseudogene	9606	10:779534 1
ENST00000451445	HACL1-002	2-hydroxyacyl-CoA lyase 1	protein_coding	9606	3:1556074 2
ENST00000507260	HADH-009	hydroxyacyl-CoA dehydrogenase	retained_intron	9606	4:1080144 2
ENST00000502896	HAND2-AS1-008	HAND2 antisense RNA 1 (head to head)	antisense	9606	4:1735304 1
ENST00000616485	HAND2-AS1-201	HAND2 antisense RNA 1 (head to head)	antisense	9606	4:1735304 1
ENST00000562889	HAPLN3-003	hyaluronan and proteoglycan link protein 3	protein_coding	9606	15:888778 2
ENST00000428854	HAUS5-002	HAUS augmin-like complex, subunit 5	retained_intron	9606	19:356128 2
ENST00000485846	HBP1-003	HMG-box transcription factor 1	protein_coding	9606	7:1071697 4
ENST00000448756	HCG19P-001	HLA complex group 19 pseudogene	processed_pseudogene	9606	6:3035927 1
ENST00000424675	HCG27-002	HLA complex group 27 (non-protein coding)	protein_coding	9606	6:3119813 2
ENST00000425430	HDAC11-006	histone deacetylase 11	nonsense_mediated_decay	9606	3:1348030 3
ENST00000459727	HDAC3-012	histone deacetylase 3	processed_transcript	9606	5:1416214 4
ENST00000535493	HDAC4-016	histone deacetylase 4	retained_intron	9606	2:2390948 1
ENST00000482651	HDGF-006	hepatoma-derived growth factor	processed_transcript	9606	1:1567431 2
ENST00000588183	HDHD2-001	haloacid dehalogenase-like hydrolase domain containing 2	nonsense_mediated_decay	9606	18:471074 2
ENST00000370082	HELZ2-004	helicase with zinc finger 2, transcriptional coactivator	retained_intron	9606	20:635701 2
ENST00000519389	HEPH-001	hephaestin	protein_coding	9606	X:6616254 2
ENST00000440441	HERC2P3-011	hect domain and RLD 2 pseudogene 3	processed_transcript	9606	15:204442 2
ENST00000277817	HERC4-002	HECT and RLD domain containing E3 ubiquitin protein ligase 4	protein_coding	9606	10:679218 1
ENST00000427635	HERC4-010	HECT and RLD domain containing E3 ubiquitin protein ligase 4	nonsense_mediated_decay	9606	10:679224 1
ENST00000569569	HERPUD1-005	homocysteine-inducible, endoplasmic reticulum stress-inducible, ubiquitin-like domain member 1	retained_intron	9606	16:569321 1
ENST00000357836	HFE2-002	hemochromatosis type 2 (juvenile)	protein_coding	9606	1:1460174 1
ENST00000521576	HGSNAT-003	heparan-alpha-glucosaminide N-acetyltransferase	protein_coding	9606	8:4318028 1
ENST00000545781	HHAT-205	hedgehog acyltransferase	protein_coding	9606	1:2103697 1
ENST00000392332	HIBCH-010	3-hydroxyisobutyryl-CoA hydrolase	protein_coding	9606	2:1902039 1
ENST00000427832	HIGD1AP12-001	HIG1 hypoxia inducible domain family, member 1A pseudogene 12	processed_pseudogene	9606	1:1113802 2
ENST00000532312	HINFP-008	histone H4 transcription factor	protein_coding	9606	11:119123 1
ENST00000474908	HINT2-003	histidine triad nucleotide binding protein 2	processed_transcript	9606	9:3581296 2
ENST00000448609	HIST1H2APS6-001	HIST1H2A pseudogene 6	processed_pseudogene	9606	13:364900 1
ENST00000488706	HKDC1-003	hexokinase domain containing 1	processed_transcript	9606	10:692475 2
ENST00000519047	HMBOX1-005	homeobox containing 1	protein_coding	9606	8:2889062 2
ENST00000406019	HMGB1P13-001	high mobility group box 1 pseudogene 13	processed_pseudogene	9606	6:1328682 2
ENST00000520062	HMGB1P46-001	high mobility group box 1 pseudogene 46	processed_pseudogene	9606	8:1071732 2
ENST00000417997	HMGB3P20-001	high mobility group box 3 pseudogene 20	processed_pseudogene	9606	7:2698276 1
ENST00000428894	HMGB3P5-001	high mobility group box 3 pseudogene 5	processed_pseudogene	9606	10:110450 1
ENST00000463817	HMGN2-006	high mobility group nucleosomal binding domain 2	processed_transcript	9606	1:2647251 1
ENST00000433603	HMGN2P3-001	high mobility group nucleosomal binding domain 2 pseudogene 3	processed_pseudogene	9606	16:260325 1
ENST00000426409	HNRNPA1P29-001	heterogeneous nuclear ribonucleoprotein A1 pseudogene 29	processed_pseudogene	9606	13:933949 1
ENST00000446472	HNRNPA1P73-001	heterogeneous nuclear ribonucleoprotein A1 pseudogene 73	processed_pseudogene	9606	7:2732860 4
ENST00000313899	HNRNPD-001	heterogeneous nuclear ribonucleoprotein D (AU-rich element RNA binding protein 1, 37kDa)	protein_coding	9606	4:8235249 1
ENST00000534639	HNRNPKP3-001	heterogeneous nuclear ribonucleoprotein K pseudogene 3	transcribed_processed_pseudo	9606	11:432621 4

ENST00000489695	HOXA9-005	homeobox A9	processed_transcript	9606	7:2716368 1
ENST00000524304	HOXA-AS3-002	HOXA cluster antisense RNA 3	antisense	9606	7:2715005 1
ENST00000432796	HOXD3-002	homeobox D3	protein_coding	9606	2:1761369 4
ENST00000440016	HOXD-AS2-001	HOXD cluster antisense RNA 2	antisense	9606	2:1761216 4
ENST00000419810	HPCAL1-003	hippocalcin-like 1	nonsense_mediated_decay	9606	2:1032434 1
ENST00000423674	HPCAL1-004	hippocalcin-like 1	protein_coding	9606	2:1030288 1
ENST00000422379	HPS4-022	Hermansky-Pudlak syndrome 4	protein_coding	9606	22:264642 2
ENST00000586941	HRK-004	harakiri, BCL2 interacting protein	processed_transcript	9606	12:116869 4
ENST00000520507	HRSP12-007	heat-responsive protein 12	protein_coding	9606	8:9810243 2
ENST00000520216	HSD17B4-027	hydroxysteroid (17-beta) dehydrogenase 4	processed_transcript	9606	5:1194938 2
ENST00000412419	HSD17B7P1-001	hydroxysteroid (17-beta) dehydrogenase 7 pseudogene 1	processed_pseudogene	9606	1:2478274 1
ENST00000592607	HSD52-004	uncharacterized LOC729467	lincRNA	9606	1:5913237 2
ENST00000467434	HSDL2-004	hydroxysteroid dehydrogenase like 2	processed_transcript	9606	9:1123801 2
ENST00000530524	HSPD1P2-001	heat shock 60kDa protein 1 (chaperonin) pseudogene 2	processed_pseudogene	9606	8:7891003 2
ENST00000371951	HTR2C-001	5-hydroxytryptamine (serotonin) receptor 2C, G protein-coupled	protein_coding	9606	X:1145840 1
ENST00000521124	HTR4-005	5-hydroxytryptamine (serotonin) receptor 4, G protein-coupled	retained_intron	9606	5:1485093 2
ENST00000487366	HYI-006	hydroxypyruvate isomerase (putative)	protein_coding	9606	1:4345115 6
ENST00000532519	HYOU1-201	hypoxia up-regulated 1	protein_coding	9606	11:119045 2
ENST00000512800	ICE2P1-001	interactor of little elongator complex ELL subunit 2 pseudogene 1	processed_pseudogene	9606	4:1883874 2
ENST00000380851	IDH3B-010	isocitrate dehydrogenase 3 (NAD+) beta	protein_coding	9606	20:265839 1
ENST00000506561	IDUA-009	iduronidase, alpha-L-	retained_intron	9606	4:987076-: 2
ENST00000471408	IFFO1-004	intermediate filament family orphan 1	retained_intron	9606	12:653896 1
ENST00000553664	IFI27L1-007	interferon, alpha-inducible protein 27-like 1	protein_coding	9606	14:940813 2
ENST00000259555	IFNA6-201	interferon, alpha 6	protein_coding	9606	9:2135025 1
ENST00000466459	IFRD1-016	interferon-related developmental regulator 1	processed_transcript	9606	7:1124569 2
ENST00000546374	IFT81-004	intraflagellar transport 81	protein_coding	9606	12:110124 2
ENST00000551273	IFT81-005	intraflagellar transport 81	nonsense_mediated_decay	9606	12:110132 2
ENST00000561309	IGDCC4-004	immunoglobulin superfamily, DCC subclass, member 4	retained_intron	9606	15:653848 1
ENST00000601052	IGFL2-006	IGF-like family member 2	protein_coding	9606	19:461606 2
ENST00000568775	IGHV3OR16-6-001	immunoglobulin heavy variable 3/OR16-6 (pseudogene)	IG_V_pseudogene	9606	16:329150 1
ENST00000342002	IKZF2-002	IKAROS family zinc finger 2 (Helios)	protein_coding	9606	2:2130053 2
ENST00000454992	IL17RE-010	interleukin 17 receptor E	protein_coding	9606	3:9902882 2
ENST00000409329	IL1R1-004	interleukin 1 receptor, type I	protein_coding	9606	2:1021045 1
ENST00000413869	IL1RAP-004	interleukin 1 receptor accessory protein	nonsense_mediated_decay	9606	3:1905141 2
ENST00000429622	IL2RB-005	interleukin 2 receptor, beta	protein_coding	9606	22:371376 2
ENST00000531965	IL32-003	interleukin 32	protein_coding	9606	16:306532 1
ENST00000552664	IL32-031	interleukin 32	protein_coding	9606	16:306566 2
ENST00000462418	IL5-003	interleukin 5	retained_intron	9606	5:1325420 1
ENST00000336909	IL6ST-201	interleukin 6 signal transducer	protein_coding	9606	5:5593509 2
ENST00000614979	ILDR2-008	immunoglobulin-like domain containing receptor 2	processed_transcript	9606	1:1669080 3
ENST00000547970	INHBE-003	inhibin, beta E	protein_coding	9606	12:574547 1
ENST00000523221	INPP4A-009	inositol polyphosphate-4-phosphatase, type I, 107kDa	protein_coding	9606	2:9852004 2
ENST00000420017	INPP5J-011	inositol polyphosphate-5-phosphatase J	protein_coding	9606	22:311229 2
ENST00000576646	INPP5K-020	inositol polyphosphate-5-phosphatase K	nonsense_mediated_decay	9606	17:150708 4
ENST00000476665	IQCE-004	IQ motif containing E	protein_coding	9606	7:2559079 1
ENST00000416896	IQCG-008	IQ motif containing G	protein_coding	9606	3:1979260 2
ENST00000596822	IRF3-017	interferon regulatory factor 3	protein_coding	9606	19:496595 2
ENST00000464557	IRF5-010	interferon regulatory factor 5	protein_coding	9606	7:1289379 2
ENST00000532096	IRF7-011	interferon regulatory factor 7	retained_intron	9606	11:614090 1
ENST00000559510	ISLR-003	immunoglobulin superfamily containing leucine-rich repeat	protein_coding	9606	15:741741 3
ENST00000509814	ITGA2-004	integrin, alpha 2 (CD49B, alpha 2 subunit of VLA-2 receptor)	nonsense_mediated_decay	9606	5:5298944 1
ENST00000475438	ITGB6-003	integrin, beta 6	processed_transcript	9606	2:1601015 2
ENST00000274766	KAAG1-001	kidney associated antigen 1	protein_coding	9606	6:2435690 1
ENST00000543774	KAT8-001	K(lysine) acetyltransferase 8	protein_coding	9606	16:311157 1
ENST00000604350	KB-1410C5.4-001		processed_pseudogene	9606	8:1013374 2
ENST00000521884	KB-1460A1.2-001		lincRNA	9606	8:1011289 2
ENST00000403813	KCNQ5-204	potassium voltage-gated channel, KQT-like subfamily, member 5	protein_coding	9606	6:7262184 2
ENST00000600980	KDELR1-005	KDEL (Lys-Asp-Glu-Leu) endoplasmic reticulum protein retention receptor 1	protein_coding	9606	19:483842 2
ENST00000438023	KDM4C-011	lysine (K)-specific demethylase 4C	nonsense_mediated_decay	9606	9:6757641 1
ENST00000580882	KIAA0100-005	KIAA0100	retained_intron	9606	17:286390 1
ENST00000415042	KIF1A-012	kinesin family member 1A	protein_coding	9606	2:2407440 2
ENST00000422435	KIF21B-006	kinesin family member 21B	protein_coding	9606	1:2009738 1
ENST00000558346	KIF23-004	kinesin family member 23	nonsense_mediated_decay	9606	15:694143 1
ENST00000538893	KIF6-201	kinesin family member 6	protein_coding	9606	6:3934333 1
ENST00000458248	KLHL22-010	kelch-like family member 22	protein_coding	9606	22:204652 2
ENST00000326856	KLK15-001	kallikrein-related peptidase 15	protein_coding	9606	19:508253 1
ENST00000539370	KLRC4-KLRK1-001	KLRC4-KLRK1 readthrough	retained_intron	9606	12:103793 1
ENST00000559083	KNSTRN-014	kinetochore-localized astrin/SPAG5 binding protein	nonsense_mediated_decay	9606	15:403832 2
ENST00000468313	KRT13-003	keratin 13	retained_intron	9606	17:415009 1
ENST00000458290	KRT15-006	keratin 15	protein_coding	9606	17:415155 4
ENST00000580052	KRT16P4-002	keratin 16 pseudogene 4	processed_transcript	9606	17:184506 1
ENST00000264651	KRT24-001	keratin 24	protein_coding	9606	17:406979 1
ENST00000551956	KRT4-001	keratin 4	protein_coding	9606	12:528065 4
ENST00000267119	KRT71-001	keratin 71	protein_coding	9606	12:525439 2
ENST00000332411	KRT76-001	keratin 76	protein_coding	9606	12:527681 3
ENST00000257901	KRT85-001	keratin 85	protein_coding	9606	12:523600 2
ENST00000616689	KRTAP10-7-201	keratin associated protein 10-7	protein_coding	9606	21:446006 1
ENST00000391415	KRTAP4-9-001	keratin associated protein 4-9	protein_coding	9606	17:411053 1
ENST00000506319	KY-002	kyphoscoliosis peptidase	retained_intron	9606	3:1346070 1
ENST00000418998	L3MBTL1-201	l(3)mbt-like 1 (Drosophila)	protein_coding	9606	20:435076 2
ENST00000572086	LA16c-380H5.3-001		lincRNA	9606	16:299990 1
ENST00000417863	LA16c-60G3.8-001		processed_pseudogene	9606	22:155620 2
ENST00000517601	LACTB2-004	lactamase, beta 2	processed_transcript	9606	8:7063855 2
ENST00000393560	LAMB1-002	laminin, beta 1	protein_coding	9606	7:1079595 2
ENST00000434071	LARGE-005	like-glycosyltransferase	protein_coding	9606	22:336504 1
ENST00000528924	LAYN-010	layilin	processed_transcript	9606	11:111541 2
ENST00000528102	LAYN-011	layilin	protein_coding	9606	11:111541 1
ENST00000587600	LDLRAD4-014	low density lipoprotein receptor class A domain containing 4	processed_transcript	9606	18:134650 4
ENST00000505328	LEF1-015	lymphoid enhancer-binding factor 1	retained_intron	9606	4:1080704 1
ENST00000371060	LEPR-002	leptin receptor	protein_coding	9606	1:6542065 1
ENST00000489504	LILRA5-004	leukocyte immunoglobulin-like receptor, subfamily A (with TM domain), member 5	retained_intron	9606	19:543111 2
ENST00000452561	LINC00320-004	long intergenic non-protein coding RNA 320	lincRNA	9606	21:207429 1
ENST00000427717	LINC00379-002	long intergenic non-protein coding RNA 379	lincRNA	9606	13:911276 2

ENST00000452852	LINC00393-002	long intergenic non-protein coding RNA 393	lincRNA	9606	13:735463 2
ENST00000580336	LINC00470-007	long intergenic non-protein coding RNA 470	lincRNA	9606	18:136829 2
ENST00000582570	LINC00470-008	long intergenic non-protein coding RNA 470	lincRNA	9606	18:136808 1
ENST00000496285	LINC00518-001	long intergenic non-protein coding RNA 518	lincRNA	9606	6:1042925 1
ENST00000426364	LINC00633-002	long intergenic non-protein coding RNA 633	lincRNA	9606	X:1351189 1
ENST00000473528	LINC00635-005	long intergenic non-protein coding RNA 635	lincRNA	9606	3:1078673 2
ENST00000552608	LINC00661-003	long intergenic non-protein coding RNA 661	lincRNA	9606	19:160219 1
ENST00000596203	LINC00664-004	long intergenic non-protein coding RNA 664	lincRNA	9606	19:214942 1
ENST00000556886	LINC00871-002	long intergenic non-protein coding RNA 871	lincRNA	9606	14:460641 2
ENST00000487512	LINC00901-001	long intergenic non-protein coding RNA 901	antisense	9606	3:1169214 1
ENST00000558754	LINC00925-008	long intergenic non-protein coding RNA 925	lincRNA	9606	15:893785 2
ENST00000560917	LINC00933-003	long intergenic non-protein coding RNA 933	processed_transcript	9606	15:845706 2
ENST00000529135	LINC00964-004	long intergenic non-protein coding RNA 964	lincRNA	9606	8:1249403 2
ENST00000614200	LINC01002-006	long intergenic non-protein coding RNA 1002	lincRNA	9606	19:211647 2
ENST00000617826	LINC01002-026	long intergenic non-protein coding RNA 1002	lincRNA	9606	19:205170 2
ENST00000611441	LINC01002-038	long intergenic non-protein coding RNA 1002	lincRNA	9606	19:201404 2
ENST00000458683	LINC01036-001	long intergenic non-protein coding RNA 1036	lincRNA	9606	1:1870928 2
ENST00000514130	LINC01094-006	long intergenic non-protein coding RNA 1094	lincRNA	9606	4:7864608 2
ENST00000603837	LINC01109-001	long intergenic non-protein coding RNA 1109	lincRNA	9606	8:7640405 2
ENST00000415295	LINC01128-007	long intergenic non-protein coding RNA 1128	lincRNA	9606	1:831605- 1
ENST00000508878	LINC01184-004	long intergenic non-protein coding RNA 1184	lincRNA	9606	5:1280240 1
ENST00000594576	LINC01224-009	long intergenic non-protein coding RNA 1224	lincRNA	9606	19:234032 4
ENST00000563550	LINC01225-002	long intergenic non-protein coding RNA 1225	unitary_pseudogene	9606	1:3150139 1
ENST00000541162	LINC01252-002	long intergenic non-protein coding RNA 1252	lincRNA	9606	12:115569 2
ENST00000601505	LINC01435-002	long intergenic non-protein coding RNA 1435	lincRNA	9606	10:108039 2
ENST00000595603	LINC01435-014	long intergenic non-protein coding RNA 1435	lincRNA	9606	10:108040 1
ENST00000595737	LINC01515-008	long intergenic non-protein coding RNA 1515	lincRNA	9606	10:656157 1
ENST00000429510	LIPH-004	lipase, member H	nonsense_mediated_decay	9606	3:1855336 1
ENST00000620649	LL22NC03-23C6.13-001		lincRNA	9606	22:220363 2
ENST00000424192	LMO3-002	LIM domain only 3 (rhombotin-like 2)	nonsense_mediated_decay	9606	12:165509 2
ENST00000536509	LMO3-023	LIM domain only 3 (rhombotin-like 2)	processed_transcript	9606	12:165604 2
ENST00000537757	LMO3-024	LIM domain only 3 (rhombotin-like 2)	protein_coding	9606	12:165513 2
ENST00000544276	LMO3-026	LIM domain only 3 (rhombotin-like 2)	processed_transcript	9606	12:165988 2
ENST00000341547	LMO7-001	LIM domain 7	protein_coding	9606	13:756204 2
ENST00000510785	LNX1-AS1-001	LNX1 antisense RNA 1	antisense	9606	4:5350007 1
ENST00000398686	LOXHD1-008	lipoxygenase homology domains 1	protein_coding	9606	18:464772 1
ENST00000591528	LPHN1-007	latrophilin 1	processed_transcript	9606	19:141629 2
ENST00000485583	LRCH4-007	leucine-rich repeats and calponin homology (CH) domain containing 4	retained_intron	9606	7:1005821 1
ENST00000550945	LRMP-006	lymphoid-restricted membrane protein	protein_coding	9606	12:250522 1
ENST00000520770	LRP12-007	low density lipoprotein receptor-related protein 12	processed_transcript	9606	8:1044977 2
ENST00000442974	LRP1B-005	low density lipoprotein receptor-related protein 1B	protein_coding	9606	2:1402381 2
ENST00000559399	LRRC28-017	leucine rich repeat containing 28	processed_transcript	9606	15:992513 1
ENST00000563189	LRRC36-004	leucine rich repeat containing 36	protein_coding	9606	16:673473 2
ENST00000570311	LRRC37A17P-001	leucine rich repeat containing 37, member A17, pseudogene	transcribed_unprocessed_pseu	9606	17:470171 1
ENST00000478726	LRRC4-003	leucine rich repeat containing 4	protein_coding	9606	7:1280304 2
ENST00000588515	LRRC7-004	leucine rich repeat containing 7	nonsense_mediated_decay	9606	1:7010775 2
ENST00000216450	LRRC74A-001	leucine rich repeat containing 74A	nonsense_mediated_decay	9606	14:768263 2
ENST00000460005	LRRC74A-003	leucine rich repeat containing 74A	processed_transcript	9606	14:768263 1
ENST00000472068	LRSAM1-012	leucine rich repeat and sterile alpha motif containing 1	processed_transcript	9606	9:1274799 2
ENST00000543818	LRTM2-201	leucine-rich repeats and transmembrane domains 2	protein_coding	9606	12:182026 1
ENST00000490035	LSAMP-001	limbic system-associated membrane protein	protein_coding	9606	3:1158023 1
ENST00000436601	LSM3P3-001	LSM3 pseudogene 3	processed_pseudogene	9606	2:8510238 2
ENST00000419073	LST1-018	leukocyte specific transcript 1	processed_transcript	9606	6:3158708 2
ENST00000471842	LTA-004	lymphotoxin alpha	retained_intron	9606	6:3157229 1
ENST00000314174	LUZP1-001	leucine zipper protein 1	protein_coding	9606	1:2309042 1
ENST00000479606	LZTR1-002	leucine-zipper-like transcription regulator 1	processed_transcript	9606	22:209794 2
ENST00000375722	MAGED1-005	melanoma antigen family D, 1	protein_coding	9606	X:5189353 1
ENST00000463787	MAGED2-009	melanoma antigen family D, 2	processed_transcript	9606	X:5480775 1
ENST00000396224	MAGED2-203	melanoma antigen family D, 2	protein_coding	9606	X:5480906 1
ENST00000425952	MAP3K19-009	mitogen-activated protein kinase kinase kinase 19	protein_coding	9606	2:1349989 2
ENST00000399925	MAP3K7CL-011	MAP3K7 C-terminal like	protein_coding	9606	21:291309 2
ENST00000618822	MAP7-204	microtubule-associated protein 7	protein_coding	9606	6:1363422 2
ENST00000379248	MAP9-004	microtubule-associated protein 9	protein_coding	9606	4:1553681 2
ENST00000435438	MAPRE1P3-001	MAPRE1 pseudogene 3	processed_pseudogene	9606	2:1652852 2
ENST00000395769	MARCH8-001	membrane-associated ring finger (C3HC4) 8, E3 ubiquitin protein ligase	protein_coding	9606	10:454575 4
ENST00000425897	MARK2-012	MAP/microtubule affinity-regulating kinase 2	protein_coding	9606	11:638952 4
ENST00000447721	MARS-005	methionyl-tRNA synthetase	processed_transcript	9606	12:574880 1
ENST00000337774	MASP1-001	mannan-binding lectin serine peptidase 1 (C4/C2 activating component of Ra-reactive factor)	protein_coding	9606	3:1872172 2
ENST00000345429	MBNL2-001	muscleblind-like splicing regulator 2	protein_coding	9606	13:972223 2
ENST00000589410	MC5R-002	melanocortin 5 receptor	protein_coding	9606	18:138241 1
ENST00000512218	MCCC2-002	methylcrotonoyl-CoA carboxylase 2 (beta)	nonsense_mediated_decay	9606	5:7158728 1
ENST00000536274	MCF2-202	MCF.2 cell line derived transforming sequence	protein_coding	9606	X:1395817 1
ENST00000507214	MCTP1-016	multiple C2 domains, transmembrane 1	protein_coding	9606	5:9491285 2
ENST00000581054	MED24-028	mediator complex subunit 24	retained_intron	9606	17:400359 1
ENST00000523671	MEG3-014	maternally expressed 3 (non-protein coding)	lincRNA	9606	14:100829 1
ENST00000452349	MEG9-002	maternally expressed 9 (non-protein coding)	lincRNA	9606	14:101071 2
ENST00000561040	MEIS3-010	Meis homeobox 3	retained_intron	9606	19:474158 2
ENST00000619894	Metazoa_SRP.1-316	Metazoan signal recognition particle RNA	misc_RNA	9606	22:215551 2
ENST00000613101	Metazoa_SRP.1-362	Metazoan signal recognition particle RNA	misc_RNA	9606	7:1520251 2
ENST00000461754	Metazoa_SRP.1-382	Metazoan signal recognition particle RNA	misc_RNA	9606	2:2505803 2
ENST00000579643	Metazoa_SRP.1-417	Metazoan signal recognition particle RNA	misc_RNA	9606	17:470418 1
ENST00000621608	Metazoa_SRP.1-445	Metazoan signal recognition particle RNA	misc_RNA	9606	2:1306034 1
ENST00000466270	METTL10-002	methyltransferase like 10	nonsense_mediated_decay	9606	10:124757 1
ENST00000537825	METTL5-012	methyltransferase like 5	nonsense_mediated_decay	9606	2:1698117 2
ENST00000506764	MFAP3L-007	microfibrillar-associated protein 3-like	protein_coding	9606	4:1700056 2
ENST00000428085	MFSD9-005	major facilitator superfamily domain containing 9	nonsense_mediated_decay	9606	2:1027190 2
ENST00000482611	MGEA5-006	meningioma expressed antigen 5 (hyaluronidase)	processed_transcript	9606	10:101786 2
ENST00000449017	MIATNB-003	MIAT neighbor (non-protein coding)	lincRNA	9606	22:266727 2
ENST00000495076	MICAL3-005	microtubule associated monooxygenase, calponin and LIM domain containing 3	nonsense_mediated_decay	9606	22:178395 1
ENST00000408338	MIR1255A-201	microRNA 1255a	miRNA	9606	4:1013303 2
ENST00000524265	MIR143HG-005	MIR143 host gene (non-protein coding)	lincRNA	9606	5:1494069 1
ENST00000385240	MIR181B1-201	microRNA 181b-1	miRNA	9606	1:1988588 2
ENST00000579966	MIR378H-201	microRNA 378h	miRNA	9606	5:1548294 2

ENST00000566533	MIR3976HG-001	MIR3976 host gene (non-protein coding)	lincRNA	9606	18:574881 1
ENST00000390180	MIR454-201	microRNA 454	miRNA	9606	17:591377 1
ENST00000586239	MIR490-003	microRNA 490	antisense	9606	7:1366855 2
ENST00000583467	MIR5188-201	microRNA 5188	miRNA	9606	12:124915 1
ENST00000577370	MIR5189-201	microRNA 5189	miRNA	9606	16:884689 1
ENST00000579892	MIR5192-201	microRNA 5192	miRNA	9606	2:6220582 2
ENST00000408833	MIR548J-201	microRNA 548j	miRNA	9606	22:265552 1
ENST00000408742	MIR548N-201	microRNA 548n	miRNA	9606	7:3494076 2
ENST00000385017	MIR553-201	microRNA 553	miRNA	9606	1:1002812 2
ENST00000385256	MIR601-201	microRNA 601	miRNA	9606	9:1234025 2
ENST00000609914	MIR663AHG-031	MIR663A host gene (non-protein coding)	lincRNA	9606	20:261877 2
ENST00000618442	MIR6752-201	microRNA 6752	miRNA	9606	11:674902 2
ENST00000390702	MIR676-201	microRNA 676	miRNA	9606	X:7002282 2
ENST00000619908	MIR6783-201	microRNA 6783	miRNA	9606	17:449346 2
ENST00000614393	MIR7150-201	microRNA 7150	miRNA	9606	9:1234855 2
ENST00000401154	MIR933-201	microRNA 933	miRNA	9606	2:1751676 2
ENST00000362105	MIRLET7A2-201	microRNA let-7a-2	miRNA	9606	11:122146 2
ENST00000394351	MITF-004	microphthalmia-associated transcription factor	protein_coding	9606	3:6993660 2
ENST00000461511	MITF-011	microphthalmia-associated transcription factor	processed_transcript	9606	3:6973947 2
ENST00000484301	MKNK1-005	MAP kinase interacting serine/threonine kinase 1	retained_intron	9606	1:4657373 1
ENST00000542390	MMAB-010	methylmalonic aciduria (cobalamin deficiency) cblB type	retained_intron	9606	12:109561 2
ENST00000542305	MMP20-002	matrix metallopeptidase 20	processed_transcript	9606	11:102577 1
ENST00000441194	MMS19-009	MMS19 nucleotide excision repair homolog (S. cerevisiae)	nonsense_mediated_decay	9606	10:974696 1
ENST00000376894	MOG-002	myelin oligodendrocyte glycoprotein	protein_coding	9606	6:2965709 1
ENST00000414701	MOGS-007	mannosyl-oligosaccharide glucosidase	protein_coding	9606	2:7446299 2
ENST00000484985	MON1A-007	MON1 secretory trafficking family member A	processed_transcript	9606	3:4991153 1
ENST00000393529	MPP1-016	membrane protein, palmitoylated 1, 55kDa	protein_coding	9606	X:1547840 2
ENST00000528686	MPPED2-002	metallophosphoesterase domain containing 2	protein_coding	9606	11:305360 1
ENST00000402310	MPV17-004	MpV17 mitochondrial inner membrane protein	protein_coding	9606	2:2730949 2
ENST00000503890	MROH2B-004	maestro heat-like repeat family member 2B	retained_intron	9606	5:4099802 1
ENST00000467306	MRPL9-003	mitochondrial ribosomal protein L9	processed_transcript	9606	1:1517596 4
ENST00000492684	MRPL9-008	mitochondrial ribosomal protein L9	processed_transcript	9606	1:1517615 4
ENST00000515647	MRPS30-002	mitochondrial ribosomal protein S30	processed_transcript	9606	5:4480984 2
ENST00000606974	MRPS31P5-003	mitochondrial ribosomal protein S31 pseudogene 5	processed_transcript	9606	13:521712 2
ENST00000607588	MRPS31P5-005	mitochondrial ribosomal protein S31 pseudogene 5	processed_transcript	9606	13:521690 2
ENST00000512880	MRPS36-002	mitochondrial ribosomal protein S36	protein_coding	9606	5:6921783 2
ENST00000519284	MRPS36P3-001	mitochondrial ribosomal protein S36 pseudogene 3	processed_pseudogene	9606	8:1220914 2
ENST00000373729	MRRF-004	mitochondrial ribosome recycling factor	protein_coding	9606	9:1222649 2
ENST00000558540	MRVI1-015	murine retrovirus integration site 1 homolog	protein_coding	9606	11:105757 2
ENST00000300187	MS4A14-002	membrane-spanning 4-domains, subfamily A, member 14	protein_coding	9606	11:603963 1
ENST00000525686	MS4A3-005	membrane-spanning 4-domains, subfamily A, member 3 (hematopoietic cell-specific)	nonsense_mediated_decay	9606	11:600567 2
ENST00000521739	MSC-003	musculin	processed_transcript	9606	8:7184242 2
ENST00000565838	MT3-003	metallothionein 3	protein_coding	9606	16:565890 2
ENST00000400050	MTCL1-201	microtubule crosslinking factor 1	protein_coding	9606	18:871789 2
ENST00000422088	MTCO3P1-001	MT-CO3 pseudogene 1	unprocessed_pseudogene	9606	6:3270612 1
ENST00000401524	MTCYBP4-001	MT-CYB pseudogene 4	processed_pseudogene	9606	6:1331505 1
ENST00000374303	MTFR1L-201	mitochondrial fission regulator 1-like	protein_coding	9606	1:2581990 1
ENST00000527338	MTFR2-005	mitochondrial fission regulator 2	retained_intron	9606	6:1362486 1
ENST00000472005	MTG2-005	mitochondrial ribosome-associated GTPase 2	processed_transcript	9606	20:621935 2
ENST00000515074	MTHFD2P4-001	methylenetetrahydrofolate dehydrogenase (NADP+ dependent) 2, methenyltetrahydrofolate cyclohydrolase pseudogene 4	processed_pseudogene	9606	4:1623221 2
ENST00000320415	MTHFD2P5-001	methylenetetrahydrofolate dehydrogenase (NADP+ dependent) 2, methenyltetrahydrofolate cyclohydrolase pseudogene 5	processed_pseudogene	9606	7:8258984 3
ENST00000567539	MTHFSD-013	methenyltetrahydrofolate synthetase domain containing	nonsense_mediated_decay	9606	16:865323 2
ENST00000417741	MTIF2-006	mitochondrial translational initiation factor 2	processed_transcript	9606	2:5525254 2
ENST00000490530	MTM1-004	myotubularin 1	processed_transcript	9606	X:1505686 2
ENST00000465177	MTND1P16-001	MT-ND1 pseudogene 16	unprocessed_pseudogene	9606	3:1069010 1
ENST00000438556	MTND1P34-001	MT-ND1 pseudogene 34	transcribed_processed_pseudo	9606	1:4716451 1
ENST00000418575	MTND2P15-001	MT-ND2 pseudogene 15	processed_pseudogene	9606	10:695940 1
ENST00000453434	MTND2P24-001	MT-ND2 pseudogene 24	processed_pseudogene	9606	X:5517919 4
ENST00000449028	MTND2P25-001	MT-ND2 pseudogene 25	processed_pseudogene	9606	X:6284369 1
ENST00000438452	MTND3P2-001	MT-ND3 pseudogene 2	unprocessed_pseudogene	9606	7:6410565 1
ENST00000429526	MTND3P9-001	MT-ND3 pseudogene 9	processed_pseudogene	9606	2:1430984 1
ENST00000413116	MTND4P22-001	MT-ND4 pseudogene 22	processed_pseudogene	9606	2:1430968 1
ENST00000435821	MTND4P5-001	MT-ND4 pseudogene 5	unprocessed_pseudogene	9606	7:5719269 1
ENST00000513046	MTND5P13-001	MT-ND5 pseudogene 13	processed_pseudogene	9606	4:6460945 2
ENST00000430812	MTND5P23-001	MT-ND5 pseudogene 23	unprocessed_pseudogene	9606	2:1313704 1
ENST00000438536	MTND5P3-001	MT-ND5 pseudogene 3	processed_pseudogene	9606	13:845217 1
ENST00000562883	MTSS1L-003	metastasis suppressor 1-like	protein_coding	9606	16:706743 2
ENST00000304887	MUC7-001	mucin 7, secreted	protein_coding	9606	4:7047211 1
ENST00000357175	MUM1L1-002	melanoma associated antigen (mutated) 1-like 1	protein_coding	9606	X:1061683 2
ENST00000374439	MUSK-003	muscle, skeletal, receptor tyrosine kinase	protein_coding	9606	9:1106872 1
ENST00000529939	MVB12A-008	multivesicular body subunit 12A	protein_coding	9606	19:174200 2
ENST00000481838	MX2-003	MX dynamin-like GTPase 2	processed_transcript	9606	21:413989 1
ENST00000442647	MYB-009	v-myb avian myeloblastosis viral oncogene homolog	protein_coding	9606	6:1351813 2
ENST00000531737	MYB-028	v-myb avian myeloblastosis viral oncogene homolog	nonsense_mediated_decay	9606	6:1351815 1
ENST00000455534	MYB-AS1-001	MYB antisense RNA 1	antisense	9606	6:1351950 1
ENST00000596245	MYO1F-018	myosin IF	retained_intron	9606	19:852522 2
ENST00000598797	MYO1F-019	myosin IF	retained_intron	9606	19:853004 1
ENST00000409044	MYO3B-001	myosin IIIB	protein_coding	9606	2:1701781 2
ENST00000241651	MYOG-001	myogenin (myogenic factor 4)	protein_coding	9606	1:2030831 2
ENST00000581075	MYOM1-004	myomesin 1	nonsense_mediated_decay	9606	18:307573 2
ENST00000401044	MYSM1-002	Myb-like, SWIRM and MPN domains 1	processed_transcript	9606	1:5865915 2
ENST00000375944	NAALAD2-009	N-acetylated alpha-linked acidic dipeptidase 2	protein_coding	9606	11:901347 2
ENST00000414826	NAALADL2-005	N-acetylated alpha-linked acidic dipeptidase-like 2	nonsense_mediated_decay	9606	3:1752564 2
ENST00000409510	NABP1-010	nucleic acid binding protein 1	protein_coding	9606	2:1916782 4
ENST00000318548	NACAP2-001	nascent-polypeptide-associated complex alpha polypeptide pseudogene 2	processed_pseudogene	9606	10:119997 3
ENST00000490998	NAGK-010	N-acetylglucosamine kinase	retained_intron	9606	2:7107462 1
ENST00000470245	NAIF1-004	nuclear apoptosis inducing factor 1	processed_transcript	9606	9:1280612 2
ENST00000605043	NAMA-004	non-protein coding RNA, associated with MAP kinase pathway and growth arrest	lincRNA	9606	9:9937435 1
ENST00000526115	NAP1L4-002	nucleosome assembly protein 1-like 4	protein_coding	9606	11:294511 1
ENST00000523923	NCALD-016	neurocalcin delta	protein_coding	9606	8:1017193 1
ENST00000611284	NCAM1-007	neural cell adhesion molecule 1	processed_transcript	9606	11:113132 2
ENST00000533073	NCAM1-011	neural cell adhesion molecule 1	processed_transcript	9606	11:113232 2
ENST00000618266	NCAM1-203	neural cell adhesion molecule 1	protein_coding	9606	11:112961 3

ENST00000484983	NCAM2-002	neural cell adhesion molecule 2	processed_transcript	9606	21:214637 4
ENST00000520297	NCAPH2-014	non-SMC condensin II complex, subunit H2	processed_transcript	9606	22:505195 2
ENST00000612493	NCOA6-201	nuclear receptor coactivator 6	protein_coding	9606	20:347147 1
ENST00000420698	NCOR2-004	nuclear receptor corepressor 2	protein_coding	9606	12:124419 1
ENST00000455934	NDUFS1-201	NADH dehydrogenase (ubiquinone) Fe-S protein 1, 75kDa (NADH-coenzyme Q reductase)	protein_coding	9606	2:2061231 2
ENST00000450668	NDUFS5P3-001	NADH dehydrogenase (ubiquinone) Fe-S protein 5, 15kDa (NADH-coenzyme Q reductase) pseudogene 3	processed_pseudogene	9606	1:5270912 2
ENST00000437256	NDUFS5P5-001	NADH dehydrogenase (ubiquinone) Fe-S protein 5, 15kDa (NADH-coenzyme Q reductase) pseudogene 5	processed_pseudogene	9606	4:1184005 2
ENST00000532244	NDUFV1-008	NADH dehydrogenase (ubiquinone) flavoprotein 1, 51kDa	protein_coding	9606	11:676069 2
ENST00000356462	NEDD4L-005	neural precursor cell expressed, developmentally down-regulated 4-like, E3 ubiquitin protein ligase	protein_coding	9606	18:580443 1
ENST00000587881	NEDD4L-030	neural precursor cell expressed, developmentally down-regulated 4-like, E3 ubiquitin protein ligase	protein_coding	9606	18:583233 2
ENST00000435750	NEK10-005	NIMA-related kinase 10	protein_coding	9606	3:2732222 1
ENST00000550139	NELL2-015	NEL-like 2 (chicken)	protein_coding	9606	12:445227 1
ENST00000547636	NELL2-016	NEL-like 2 (chicken)	retained_intron	9606	12:445219 2
ENST00000547172	NELL2-018	NEL-like 2 (chicken)	processed_transcript	9606	12:447796 1
ENST00000558807	NEO1-009	neogenin 1	retained_intron	9606	15:732547 2
ENST00000588287	NF1P5-001	neurofibromin 1 pseudogene 5	processed_pseudogene	9606	18:141530 2
ENST00000553469	NFATC4-011	nuclear factor of activated T-cells, cytoplasmic, calcineurin-dependent 4	protein_coding	9606	14:243675 1
ENST00000557140	NFKBIA-002	nuclear factor of kappa light polypeptide gene enhancer in B-cells inhibitor, alpha	protein_coding	9606	14:354015 2
ENST00000531755	NFRKB-004	nuclear factor related to kappaB binding protein	protein_coding	9606	11:129870 1
ENST00000417799	NIFKP3-001	nucleolar protein interacting with the FHA domain of MKI67 pseudogene 3	processed_pseudogene	9606	12:760435 2
ENST00000513797	NIM1K-004	NIM1 serine/threonine protein kinase	processed_transcript	9606	5:4319223 2
ENST00000371527	NKRF-001	NFKB repressing factor	protein_coding	9606	X:1195883 2
ENST00000586379	NLRP7-004	NLR family, pyrin domain containing 7	nonsense_mediated_decay	9606	19:549236 2
ENST00000444498	NME4-012	NME/NM23 nucleoside diphosphate kinase 4	nonsense_mediated_decay	9606	16:397200 1
ENST00000505779	NMNAT3-011	nicotinamide nucleotide adenylyltransferase 3	processed_transcript	9606	3:1396343 2
ENST00000524712	NOD2-008	nucleotide-binding oligomerization domain containing 2	nonsense_mediated_decay	9606	16:507121 2
ENST00000535384	NOL4-006	nucleolar protein 4	protein_coding	9606	18:338522 2
ENST00000601015	NOSIP-007	nitric oxide synthase interacting protein	non_stop_decay	9606	19:495557 2
ENST00000378169	NPHP4-011	nephronophthisis 4	nonsense_mediated_decay	9606	1:5863265 2
ENST00000542133	NPIPB4-006	nuclear pore complex interacting protein family, member B4	nonsense_mediated_decay	9606	16:218376 2
ENST00000541674	NPIPB4-017	nuclear pore complex interacting protein family, member B4	protein_coding	9606	16:218572 2
ENST00000536620	NPIPB5-006	nuclear pore complex interacting protein family, member B5	protein_coding	9606	16:225130 2
ENST00000460690	NPL-006	N-acetylneuraminate pyruvate lyase (dihydrodipicolinate synthase)	processed_transcript	9606	1:1827894 2
ENST00000471010	NPL-007	N-acetylneuraminate pyruvate lyase (dihydrodipicolinate synthase)	processed_transcript	9606	1:1827922 2
ENST00000507141	NPR3-005	natriuretic peptide receptor 3	protein_coding	9606	5:3271233 3
ENST00000547206	NR4A1-009	nuclear receptor subfamily 4, group A, member 1	processed_transcript	9606	12:520438 2
ENST00000243050	NR4A1-201	nuclear receptor subfamily 4, group A, member 1	protein_coding	9606	12:520514 2
ENST00000429376	NR4A2-008	nuclear receptor subfamily 4, group A, member 2	protein_coding	9606	2:1563257 1
ENST00000622833	NTAN1-201	N-terminal asparagine amidase	protein_coding	9606	16:150378 2
ENST00000498764	NTM-016	neurotrimin	processed_transcript	9606	11:131910 1
ENST00000596844	NTN5-003	netrin 5	protein_coding	9606	19:486619 1
ENST00000480907	NUB1-010	negative regulator of ubiquitin-like proteins 1	retained_intron	9606	7:1513513 3
ENST00000301944	NXNL1-001	nucleoredoxin-like 1	protein_coding	9606	19:174554 1
ENST00000539878	NXPE1-006	neurexophilin and PC-esterase domain family, member 1	protein_coding	9606	11:114530 2
ENST00000366707	OBSCN-011	obscurin, cytoskeletal calmodulin and titin-interacting RhoGEF	protein_coding	9606	1:2282081 1
ENST00000570156	OBSCN-202	obscurin, cytoskeletal calmodulin and titin-interacting RhoGEF	protein_coding	9606	1:2282081 1
ENST00000487015	OFCC1-002	orofacial cleft 1 candidate 1	nonsense_mediated_decay	9606	6:9705425 1
ENST00000469421	OFCC1-022	orofacial cleft 1 candidate 1	processed_transcript	9606	6:9596110 2
ENST00000450048	OGFR-004	opioid growth factor receptor	protein_coding	9606	20:628072 2
ENST00000303532	OR10G3-001	olfactory receptor, family 10, subfamily G, member 3	protein_coding	9606	14:215698 1
ENST00000555298	OR11J2P-001	olfactory receptor, family 11, subfamily J, member 2 pseudogene	transcribed_unprocessed_pseu	9606	15:209606 1
ENST00000373686	OR1L1-201	olfactory receptor, family 1, subfamily L, member 1	protein_coding	9606	9:1226615 2
ENST00000408898	OR2A25-001	olfactory receptor, family 2, subfamily A, member 25	protein_coding	9606	7:1440741 1
ENST00000432841	OR2B8P-001	olfactory receptor, family 2, subfamily B, member 8 pseudogene	unprocessed_pseudogene	9606	6:2805322 1
ENST00000396792	OR2H1-007	olfactory receptor, family 2, subfamily H, member 1	protein_coding	9606	6:2946144 1
ENST00000318021	OR2T33-001	olfactory receptor, family 2, subfamily T, member 33	protein_coding	9606	1:2482728 1
ENST00000319968	OR2T8-001	olfactory receptor, family 2, subfamily T, member 8	protein_coding	9606	1:2479210 1
ENST00000529557	OR4A11P-001	olfactory receptor, family 4, subfamily A, member 11 pseudogene	unprocessed_pseudogene	9606	11:553185 1
ENST00000608504	OR51C1P-001	olfactory receptor, family 51, subfamily C, member 1 pseudogene	unprocessed_pseudogene	9606	11:469076 1
ENST00000452394	OR52E7P-001	olfactory receptor, family 52, subfamily E, member 7 pseudogene	unprocessed_pseudogene	9606	11:587396 1
ENST00000414298	OR52M2P-001	olfactory receptor, family 52, subfamily M, member 2 pseudogene	unprocessed_pseudogene	9606	11:451496 1
ENST00000616746	OR52Q1P-001	olfactory receptor, family 52, subfamily Q, member 1 pseudogene	unprocessed_pseudogene	9606	11:590376 3
ENST00000408922	OR6B1-001	olfactory receptor, family 6, subfamily B, member 1	protein_coding	9606	7:1440039 1
ENST00000402971	OR6B2-001	olfactory receptor, family 6, subfamily B, member 2	protein_coding	9606	2:2400289 1
ENST00000368145	OR6K3-001	olfactory receptor, family 6, subfamily K, member 3	protein_coding	9606	1:1587171 2
ENST00000531567	OR6M3P-001	olfactory receptor, family 6, subfamily M, member 3 pseudogene	unprocessed_pseudogene	9606	11:123861 1
ENST00000609216	OR6R2P-001	olfactory receptor, family 6, subfamily R, member 2 pseudogene	unprocessed_pseudogene	9606	8:2179746 2
ENST00000321252	OR6T1-001	olfactory receptor, family 6, subfamily T, member 1	protein_coding	9606	11:123942 2
ENST00000327930	OR6X1-001	olfactory receptor, family 6, subfamily X, member 1	protein_coding	9606	11:123753 2
ENST00000248072	OR7C2-001	olfactory receptor, family 7, subfamily C, member 2	protein_coding	9606	19:149414 1
ENST00000412208	OR7E155P-001	olfactory receptor, family 7, subfamily E, member 155 pseudogene	unprocessed_pseudogene	9606	13:414398 1
ENST00000533484	OR7E15P-001	olfactory receptor, family 7, subfamily E, member 15 pseudogene	unprocessed_pseudogene	9606	8:1269630 1
ENST00000437705	OR7E33P-001	olfactory receptor, family 7, subfamily E, member 33 pseudogene	unprocessed_pseudogene	9606	13:679109 1
ENST00000418098	OR7E53P-001	olfactory receptor, family 7, subfamily E, member 53 pseudogene	unprocessed_pseudogene	9606	3:1257342 2
ENST00000305456	OR7G2-001	olfactory receptor, family 7, subfamily G, member 2	protein_coding	9606	19:910226 1
ENST00000306842	OR8B12-001	olfactory receptor, family 8, subfamily B, member 12	protein_coding	9606	11:124542 1
ENST00000375013	OR8B2-001	olfactory receptor, family 8, subfamily B, member 2	protein_coding	9606	11:124382 1
ENST00000624268	OR8B4-201	olfactory receptor, family 8, subfamily B, member 4	protein_coding	9606	11:124423 1
ENST00000533932	OR8B5P-001	olfactory receptor, family 8, subfamily B, member 5 pseudogene	unprocessed_pseudogene	9606	11:124357 1
ENST00000525146	OR8B6P-001	olfactory receptor, family 8, subfamily B, member 6 pseudogene	unprocessed_pseudogene	9606	11:124338 1
ENST00000482472	OR9A3P-002	olfactory receptor, family 9, subfamily A, member 3 pseudogene	processed_transcript	9606	7:1418631 1
ENST00000580140	OSBPL7-002	oxysterol binding protein-like 7	retained_intron	9606	17:478152 1
ENST00000286149	OTOA-003	otoancorin	protein_coding	9606	16:216785 1
ENST00000319460	OTOS-201	otospiralin	protein_coding	9606	2:2401390 2
ENST00000545434	P2RX7-013	purinergic receptor P2X, ligand-gated ion channel, 7	nonsense_mediated_decay	9606	12:121132 2
ENST00000468596	P2RY12-003	purinergic receptor P2Y, G-protein coupled, 12	retained_intron	9606	3:1513381 1
ENST00000427714	P4HA3-004	prolyl 4-hydroxylase, alpha polypeptide III	protein_coding	9606	11:742666 2
ENST00000519596	PABPC1-013	poly(A) binding protein, cytoplasmic 1	processed_transcript	9606	8:1007066 1
ENST00000513861	PACRGL-026	PARK2 co-regulated-like	protein_coding	9606	4:2070045 4
ENST00000619888	PAMR1-001	peptidase domain containing associated with muscle regeneration 1	protein_coding	9606	11:354318 1
ENST00000527605	PAMR1-005	peptidase domain containing associated with muscle regeneration 1	protein_coding	9606	11:354324 2
ENST00000428308	PAPD4-201	PAP associated domain containing 4	protein_coding	9606	5:7961248 2
ENST00000483254	PAPPA-003	pregnancy-associated plasma protein A, pappalysin 1	processed_transcript	9606	9:1163731 2

ENST00000479836	PAPPA2-005	pappalysin 2	processed_transcript	9606	1:1767914	2
ENST00000463384	PARD6G-002	par-6 family cell polarity regulator gamma	protein_coding	9606	18:801828	1
ENST00000338468	PARK2-003	parkin RBR E3 ubiquitin protein ligase	protein_coding	9606	6:1613500	2
ENST00000510013	PCBD2-004	pterin-4 alpha-carbinolamine dehydratase/dimerization cofactor of hepatocyte nuclear factor 1 alpha (TCF1) 2	processed_transcript	9606	5:1349088	2
ENST00000465077	PCBP3-006	poly(rC) binding protein 3	processed_transcript	9606	21:456436	1
ENST00000361724	PCDH11X-004	protocadherin 11 X-linked	retained_intron	9606	X:9183466	1
ENST00000614895	PCDH15-205	protocadherin-related 15	protein_coding	9606	10:538027	2
ENST00000620262	PCDH18-008	protocadherin 18	processed_transcript	9606	4:1375215	1
ENST00000530304	PCF11-002	PCF11 cleavage and polyadenylation factor subunit	protein_coding	9606	11:831571	1
ENST00000530660	PCF11-003	PCF11 cleavage and polyadenylation factor subunit	protein_coding	9606	11:831571	1
ENST00000595406	PCGF7P-001	polycomb group ring finger 7 pseudogene	processed_pseudogene	9606	19:221379	2
ENST00000426442	PCLO-004	piccolo presynaptic cytomatrix protein	processed_transcript	9606	7:8282209	6
ENST00000480473	PCOLCE2-006	procollagen C-endopeptidase enhancer 2	nonsense_mediated_decay	9606	3:1428209	2
ENST00000588195	PCSK4-006	proprotein convertase subtilisin/kexin type 4	retained_intron	9606	19:148241	1
ENST00000465122	PDCD6IP-012	programmed cell death 6 interacting protein	retained_intron	9606	3:3384409	2
ENST00000392251	PDCL3P7-001	phosducin-like 3 pseudogene 7	processed_pseudogene	9606	12:662759	1
ENST00000358450	PDE11A-002	phosphodiesterase 11A	protein_coding	9606	2:1776280	1
ENST00000478646	PDE11A-011	phosphodiesterase 11A	retained_intron	9606	2:1776291	1
ENST00000611899	PDE1B-009	phosphodiesterase 1B, calmodulin-dependent	nonsense_mediated_decay	9606	12:545496	1
ENST00000396182	PDE1C-007	phosphodiesterase 1C, calmodulin-dependent 70kDa	protein_coding	9606	7:3179020	1
ENST00000539367	PDE2A-002	phosphodiesterase 2A, cGMP-stimulated	nonsense_mediated_decay	9606	11:725761	1
ENST00000593594	PDE4C-012	phosphodiesterase 4C, cAMP-specific	nonsense_mediated_decay	9606	19:182202	1
ENST00000295266	PDHA2-001	pyruvate dehydrogenase (lipoamide) alpha 2	protein_coding	9606	4:9584001	1
ENST00000397760	PDLIM2-020	PDZ and LIM domain 2 (mystique)	protein_coding	9606	8:2257913	2
ENST00000505886	PDLIM3-006	PDZ and LIM domain 3	nonsense_mediated_decay	9606	4:1855045	2
ENST00000513143	PDPN-007	podoplanin	protein_coding	9606	1:1358427	1
ENST00000451791	PENK-001	proenkephalin	protein_coding	9606	8:5644127	1
ENST00000314922	PENK-002	proenkephalin	protein_coding	9606	8:5644095	1
ENST00000525229	PEX16-008	peroxisomal biogenesis factor 16	nonsense_mediated_decay	9606	11:459154	2
ENST00000551018	PFDN5-001	prefoldin subunit 5	protein_coding	9606	12:532952	1
ENST00000312352	PFKM-201	phosphofructokinase, muscle	protein_coding	9606	12:481226	2
ENST00000378149	PGA4-001	pepsinogen 4, group I (pepsinogen A)	protein_coding	9606	11:612222	1
ENST00000496758	PGM5-005	phosphoglucomutase 5	retained_intron	9606	9:6849682	2
ENST00000421970	PGM5P4-001	phosphoglucomutase 5 pseudogene 4	processed_pseudogene	9606	2:1135419	1
ENST00000394276	PGRMC2-002	progesterone receptor membrane component 2	protein_coding	9606	4:1282692	2
ENST00000622263	PHF20L1-202	PHD finger protein 20-like 1	protein_coding	9606	8:1327753	2
ENST00000322659	PHF8-002	PHD finger protein 8	protein_coding	9606	X:5394325	1
ENST00000524713	PHLDB1-038	pleckstrin homology-like domain, family B, member 1	processed_transcript	9606	11:118641	1
ENST00000503902	PHOSPHO1-006	phosphatase, orphan 1	protein_coding	9606	17:492248	2
ENST00000528398	PICALM-003	phosphatidylinositol binding clathrin assembly protein	protein_coding	9606	11:859588	1
ENST00000356360	PICALM-006	phosphatidylinositol binding clathrin assembly protein	protein_coding	9606	11:859589	1
ENST00000484512	PIFO-002	primary cilia formation	processed_transcript	9606	1:1113466	4
ENST00000587942	PIGN-017	phosphatidylinositol glycan anchor biosynthesis, class N	retained_intron	9606	18:620453	4
ENST00000464016	PIK3R2-009	phosphoinositide-3-kinase, regulatory subunit 2 (beta)	nonsense_mediated_decay	9606	19:181662	4
ENST00000550465	PIP4K2C-003	phosphatidylinositol-5-phosphate 4-kinase, type II, gamma	protein_coding	9606	12:575912	1
ENST00000478500	PIP5K1B-002	phosphatidylinositol-4-phosphate 5-kinase, type I, beta	nonsense_mediated_decay	9606	9:6874225	2
ENST00000497234	PIP5KL1-001	phosphatidylinositol-4-phosphate 5-kinase-like 1	processed_transcript	9606	9:1279252	1
ENST00000454009	PIWIL2-003	piwi-like RNA-mediated gene silencing 2	protein_coding	9606	8:2227556	1
ENST00000337114	PKD1L2-201	polycystic kidney disease 1-like 2	protein_coding	9606	16:811702	1
ENST00000485301	PKN3-003	protein kinase N3	processed_transcript	9606	9:1287146	4
ENST00000559662	PKNOX2-014	PBX/knotted 1 homeobox 2	processed_transcript	9606	11:125164	1
ENST00000539875	PLCZ1-004	phospholipase C, zeta 1	protein_coding	9606	12:186831	1
ENST00000544849	PLCZ1-010	phospholipase C, zeta 1	retained_intron	9606	12:187357	1
ENST00000525581	PLEKHA7-012	pleckstrin homology domain containing, family A member 7	protein_coding	9606	11:167949	2
ENST00000544532	PLEKHB1-011	pleckstrin homology domain containing, family B (evectins) member 1	processed_transcript	9606	11:736476	2
ENST00000409612	PLEKHB2-004	pleckstrin homology domain containing, family B (evectins) member 2	protein_coding	9606	2:1311075	1
ENST00000567897	PLK1-007	polo-like kinase 1	protein_coding	9606	16:236795	2
ENST00000485046	PLOD1-004	procollagen-lysine, 2-oxoglutarate 5-dioxygenase 1	processed_transcript	9606	1:1193420	1
ENST00000358133	PLOD1-006	procollagen-lysine, 2-oxoglutarate 5-dioxygenase 1	processed_transcript	9606	1:1193473	1
ENST00000485980	PLXNB3-004	plexin B3	processed_transcript	9606	X:1537772	2
ENST00000567697	PMM2-007	phosphomannomutase 2	retained_intron	9606	16:880756	2
ENST00000412694	PMS2P6-001	postmeiotic segregation increased 2 pseudogene 6	unprocessed_pseudogene	9606	7:7531604	1
ENST00000579578	PNLIPRP2-005	pancreatic lipase-related protein 2	polymorphic_pseudogene	9606	10:116620	1
ENST00000502826	POC5-010	POC5 centriolar protein	protein_coding	9606	5:7570261	2
ENST00000518579	POLB-016	polymerase (DNA directed), beta	protein_coding	9606	8:4235717	2
ENST00000492605	POLM-009	polymerase (DNA directed), mu	retained_intron	9606	7:4407571	1
ENST00000621776	POLQ-201	polymerase (DNA directed), theta	protein_coding	9606	3:1214314	2
ENST00000531944	POLR2G-003	polymerase (RNA) II (DNA directed) polypeptide G	nonsense_mediated_decay	9606	11:627616	2
ENST00000361163	POTEF-202	POTE ankyrin domain family, member F	protein_coding	9606	2:1300752	1
ENST00000520104	POU6F2-007	POU class 6 homeobox 2	protein_coding	9606	7:3908505	2
ENST00000507724	PPAT-004	phosphoribosyl pyrophosphate amidotransferase	nonsense_mediated_decay	9606	4:5640307	4
ENST00000545381	PPFIBP1-004	PTPRF interacting protein, binding protein 1 (liprin beta 1)	non_stop_decay	9606	12:275241	1
ENST00000409361	PPIL3-007	peptidylprolyl isomerase (cyclophilin)-like 3	protein_coding	9606	2:2008712	2
ENST00000409432	PPM1B-004	protein phosphatase, Mg2+/Mn2+ dependent, 1B	protein_coding	9606	2:4416887	1
ENST00000396737	PPM1N-003	protein phosphatase, Mg2+/Mn2+ dependent, 1N (putative)	protein_coding	9606	19:454972	1
ENST00000376769	PPP1R11-001	protein phosphatase 1, regulatory (inhibitor) subunit 11	protein_coding	9606	6:3006720	2
ENST00000495799	PPP1R3F-003	protein phosphatase 1, regulatory subunit 3F	protein_coding	9606	X:4926987	2
ENST00000512984	PPP2R2B-013	protein phosphatase 2, regulatory subunit B, beta	protein_coding	9606	5:1465900	2
ENST00000456642	PPP6C-002	protein phosphatase 6, catalytic subunit	protein_coding	9606	9:1251539	2
ENST00000443648	PQBP1-011	polyglutamine binding protein 1	protein_coding	9606	X:4889826	2
ENST00000357367	PRAMEF8-001	PRAME family member 8	protein_coding	9606	1:1328103	1
ENST00000381842	PRB3-201	proline-rich protein BstNI subfamily 3	protein_coding	9606	12:112659	1
ENST00000489365	PRDM1-005	PR domain containing 1, with ZNF domain	retained_intron	9606	6:1060883	2
ENST00000463591	PRDM16-004	PR domain containing 16	protein_coding	9606	1:3244132	1
ENST00000509375	PRDM8-006	PR domain containing 8	processed_transcript	9606	4:8018388	2
ENST00000334482	PRDX2-002	peroxiredoxin 2	protein_coding	9606	19:127968	1
ENST00000260648	PREPL-003	prolyl endopeptidase-like	protein_coding	9606	2:4432082	2
ENST00000445766	PRICKLE1-005	prickle homolog 1 (Drosophila)	protein_coding	9606	12:424583	6
ENST00000484703	PRICKLE2-AS2-001	PRICKLE2 antisense RNA 2	antisense	9606	3:6410347	1
ENST00000614014	PRICKLE3-008	prickle homolog 3 (Drosophila)	retained_intron	9606	X:4917713	2
ENST00000376317	PRICKLE3-009	prickle homolog 3 (Drosophila)	protein_coding	9606	X:4917980	1
ENST00000430040	PRKAR1B-008	protein kinase, cAMP-dependent, regulatory, type I, beta	protein_coding	9606	7:579256-[illegible]	4
ENST00000545647	PRKG2-202	protein kinase, cGMP-dependent, type II	protein_coding	9606	4:8108737	1

ENST00000469763	PRKRIP1-008	PRKR interacting protein 1 (IL11 inducible)	processed_transcript	9606	7:1023638 1
ENST00000418528	PRNT-001	prion protein (testis specific)	protein_coding	9606	20:473128 2
ENST00000484434	PROSER1-004	proline and serine rich 1	processed_transcript	9606	13:390101 1
ENST00000413560	PROX1-AS1-002	PROX1 antisense RNA 1	antisense	9606	1:2138407 2
ENST00000601907	PROX1-AS1-011	PROX1 antisense RNA 1	antisense	9606	1:2138325 2
ENST00000605538	PRR13P7-001	proline rich 13 pseudogene 7	processed_pseudogene	9606	8:9190973 1
ENST00000571654	PRR14-016	proline rich 14	processed_transcript	9606	16:306548 2
ENST00000452758	PRSS37-003	protease, serine, 37	nonsense_mediated_decay	9606	7:1418363 1
ENST00000559842	PRTG-003	protogenin	retained_intron	9606	15:556780 2
ENST00000466500	PSAT1P4-001	phosphoserine aminotransferase 1 pseudogene 4	processed_pseudogene	9606	3:1666911 2
ENST00000286485	PSD3-003	pleckstrin and Sec7 domain containing 3	protein_coding	9606	8:1853071 1
ENST00000403486	PSG11-004	pregnancy specific beta-1-glycoprotein 11	protein_coding	9606	19:430076 1
ENST00000446844	PSG7-003	pregnancy specific beta-1-glycoprotein 7 (gene/pseudogene)	protein_coding	9606	19:429248 1
ENST00000596730	PSG9-006	pregnancy specific beta-1-glycoprotein 9	protein_coding	9606	19:432571 1
ENST00000477897	PSMA5-005	proteasome (prosome, macropain) subunit, alpha type, 5	processed_transcript	9606	1:1094118 2
ENST00000556349	PSMB7P1-001	PSMB7 pseudogene 1	unitary_pseudogene	9606	14:201492 2
ENST00000532097	PSMD13-001	proteasome (prosome, macropain) 26S subunit, non-ATPase, 13	protein_coding	9606	11:236546 1
ENST00000259881	PSORS1C1-001	psoriasis susceptibility 1 candidate 1	protein_coding	9606	6:3111483 1
ENST00000459765	PSRC1-008	proline/serine-rich coiled-coil 1	retained_intron	9606	1:1092814 1
ENST00000379595	PSTPIP1-002	proline-serine-threonine phosphatase interacting protein 1	protein_coding	9606	15:769951 2
ENST00000558407	PSTPIP1-012	proline-serine-threonine phosphatase interacting protein 1	protein_coding	9606	15:769933 2
ENST00000559856	PSTPIP1-015	proline-serine-threonine phosphatase interacting protein 1	protein_coding	9606	15:770184 2
ENST00000455399	PTCHD1-AS-001	PTCHD1 antisense RNA (head to head)	lincRNA	9606	X:2238931 1
ENST00000356595	PTGER3-004	prostaglandin E receptor 3 (subtype EP3)	protein_coding	9606	1:7095243 1
ENST00000471863	PTK7-003	protein tyrosine kinase 7	protein_coding	9606	6:4307633 2
ENST00000568793	PTPLAD1-005	protein tyrosine phosphatase-like A domain containing 1	protein_coding	9606	15:655305 2
ENST00000359785	PTPN22-001	protein tyrosine phosphatase, non-receptor type 22 (lymphoid)	protein_coding	9606	1:1138138 1
ENST00000412145	PTPN3-202	protein tyrosine phosphatase, non-receptor type 3	protein_coding	9606	9:1093756 1
ENST00000615445	PTPRJ-202	protein tyrosine phosphatase, receptor type, J	protein_coding	9606	11:479805 2
ENST00000412847	PTPRN-012	protein tyrosine phosphatase, receptor type, N	protein_coding	9606	2:2193024 1
ENST00000549107	PTPRR-010	protein tyrosine phosphatase, receptor type, R	processed_transcript	9606	12:706391 2
ENST00000517838	PVT1-015	Pvt1 oncogene (non-protein coding)	lincRNA	9606	8:1278908 4
ENST00000392252	PYHIN1-004	pyrin and HIN domain family, member 1	protein_coding	9606	1:1589315 1
ENST00000494941	PYROXD2-004	pyridine nucleotide-disulphide oxidoreductase domain 2	processed_transcript	9606	10:984139 1
ENST00000527788	QSER1-002	glutamine and serine rich 1	protein_coding	9606	11:329259 2
ENST00000518454	R3HCC1-002	R3H domain and coiled-coil containing 1	protein_coding	9606	8:2328790 2
ENST00000592334	RAB27B-002	RAB27B, member RAS oncogene family	protein_coding	9606	18:548843 2
ENST00000357573	RABEP2-006	rabaptin, RAB GTPase binding effector protein 2	protein_coding	9606	16:289044 1
ENST00000316856	RAD23A-001	RAD23 homolog A (S. cerevisiae)	protein_coding	9606	19:129458 2
ENST00000523727	RANBP17-020	RAN binding protein 17	retained_intron	9606	5:1708818 2
ENST00000475125	RARA-005	retinoic acid receptor, alpha	protein_coding	9606	17:403452 2
ENST00000416035	RBBP7-004	retinoblastoma binding protein 7	protein_coding	9606	X:1685204 4
ENST00000399725	RBBP8-002	retinoblastoma binding protein 8	protein_coding	9606	18:229338 1
ENST00000620507	RBFOX1-202	RNA binding protein, fox-1 homolog (C. elegans) 1	protein_coding	9606	16:733275 1
ENST00000513909	RCHY1-013	ring finger and CHY zinc finger domain containing 1, E3 ubiquitin protein ligase	nonsense_mediated_decay	9606	4:7548260 2
ENST00000400413	RCN1P1-001	reticulocalbin 1, EF-hand calcium binding domain pseudogene 1	processed_pseudogene	9606	6:8712169 2
ENST00000587721	RDH13-015	retinol dehydrogenase 13 (all-trans/9-cis)	nonsense_mediated_decay	9606	19:550474 2
ENST00000457282	RENBP-002	renin binding protein	nonsense_mediated_decay	9606	X:1539352 3
ENST00000475904	RENBP-010	renin binding protein	retained_intron	9606	X:1539436 1
ENST00000479668	REPIN1-001	replication initiator 1	protein_coding	9606	7:1503682 2
ENST00000473391	REPIN1-005	replication initiator 1	retained_intron	9606	7:1503688 1
ENST00000454447	RFTN2-002	raftlin family member 2	protein_coding	9606	2:1975720 2
ENST00000302303	RFX3-201	regulatory factor X, 3 (influences HLA class II expression)	protein_coding	9606	9:3270930 2
ENST00000357881	RFX4-003	regulatory factor X, 4 (influences HLA class II expression)	protein_coding	9606	12:106601 1
ENST00000330331	RGPD6-012	RANBP2-like and GRIP domain containing 6	protein_coding	9606	2:1105384 2
ENST00000493165	RGS19-002	regulator of G-protein signaling 19	processed_transcript	9606	20:640744 1
ENST00000519725	RGS22-017	regulator of G-protein signaling 22	nonsense_mediated_decay	9606	8:1000084 2
ENST00000511121	RHOH-014	ras homolog family member H	protein_coding	9606	4:4020034 1
ENST00000581094	RHOT1-006	ras homolog family member T1	protein_coding	9606	17:321426 1
ENST00000512598	RIMS2-010	regulating synaptic membrane exocytosis 2	retained_intron	9606	8:1039155 2
ENST00000548886	RMST-002	rhabdomyosarcoma 2 associated transcript (non-protein coding)	lincRNA	9606	12:974924 1
ENST00000363008	RN7SKP149-201	RNA, 7SK small nuclear pseudogene 149	misc_RNA	9606	X:1431972 2
ENST00000410170	RN7SKP232-201	RNA, 7SK small nuclear pseudogene 232	misc_RNA	9606	5:1517042 1
ENST00000411094	RN7SKP247-201	RNA, 7SK small nuclear pseudogene 247	misc_RNA	9606	1:8125178 2
ENST00000410685	RN7SKP254-201	RNA, 7SK small nuclear pseudogene 254	misc_RNA	9606	15:964865 2
ENST00000410520	RN7SKP59-201	RNA, 7SK small nuclear pseudogene 59	misc_RNA	9606	9:7816546 1
ENST00000487345	RN7SL147P-201	RNA, 7SL, cytoplasmic 147, pseudogene	misc_RNA	9606	3:1195278 2
ENST00000465197	RN7SL149P-201	RNA, 7SL, cytoplasmic 149, pseudogene	misc_RNA	9606	8:4184005 2
ENST00000490174	RN7SL205P-201	RNA, 7SL, cytoplasmic 205, pseudogene	misc_RNA	9606	4:1317202 2
ENST00000485107	RN7SL267P-201	RNA, 7SL, cytoplasmic 267, pseudogene	misc_RNA	9606	2:1823142 2
ENST00000481765	RN7SL293P-201	RNA, 7SL, cytoplasmic 293, pseudogene	misc_RNA	9606	8:1137939 2
ENST00000466002	RN7SL352P-201	RNA, 7SL, cytoplasmic 352, pseudogene	misc_RNA	9606	6:1507322 1
ENST00000584418	RN7SL435P-201	RNA, 7SL, cytoplasmic 435, pseudogene	misc_RNA	9606	11:592910 1
ENST00000476341	RN7SL497P-201	RNA, 7SL, cytoplasmic 497, pseudogene	misc_RNA	9606	15:411919 2
ENST00000464472	RN7SL508P-201	RNA, 7SL, cytoplasmic 508, pseudogene	misc_RNA	9606	12:118993 2
ENST00000467147	RN7SL542P-201	RNA, 7SL, cytoplasmic 542, pseudogene	misc_RNA	9606	7:2121553 2
ENST00000480810	RN7SL551P-201	RNA, 7SL, cytoplasmic 551, pseudogene	misc_RNA	9606	18:742275 1
ENST00000493933	RN7SL668P-201	RNA, 7SL, cytoplasmic 668, pseudogene	misc_RNA	9606	1:2349041 2
ENST00000460697	RN7SL72P-201	RNA, 7SL, cytoplasmic 72, pseudogene	misc_RNA	9606	7:1484383 2
ENST00000365061	RNA5SP172-201	RNA, 5S ribosomal pseudogene 172	rRNA	9606	4:1774571 2
ENST00000516938	RNA5SP364-201	RNA, 5S ribosomal pseudogene 364	rRNA	9606	12:880517 2
ENST00000363646	RNA5SP378-201	RNA, 5S ribosomal pseudogene 378	rRNA	9606	12:131801 1
ENST00000364599	RNA5SP379-201	RNA, 5S ribosomal pseudogene 379	rRNA	9606	12:132723 2
ENST00000591394	RNF126-002	ring finger protein 126	retained_intron	9606	19:651385 1
ENST00000474394	RNF220-017	ring finger protein 220	processed_transcript	9606	1:4463829 1
ENST00000399398	RNF222-001	ring finger protein 222	protein_coding	9606	17:839070 2
ENST00000525701	RNH1-002	ribonuclease/angiogenin inhibitor 1	nonsense_mediated_decay	9606	11:494523 2
ENST00000354420	RNH1-004	ribonuclease/angiogenin inhibitor 1	protein_coding	9606	11:494552 2
ENST00000543302	RNMT-003	RNA (guanine-7-) methyltransferase	protein_coding	9606	18:137266 1
ENST00000410144	RNU2-3P-201	RNA, U2 small nuclear 3, pseudogene	snRNA	9606	15:957458 3
ENST00000410697	RNU2-42P-201	RNA, U2 small nuclear 42, pseudogene	snRNA	9606	10:125890 1
ENST00000410494	RNU2-50P-201	RNA, U2 small nuclear 50, pseudogene	snRNA	9606	9:3428252 1
ENST00000517272	RNU4ATAC17P-201	RNA, U4atac small nuclear 17, pseudogene	snRNA	9606	3:8364529 1

ENST00000516802	RNU6-1121P-201	RNA, U6 small nuclear 1121, pseudogene	snRNA	9606	10:114969	2
ENST00000363795	RNU6-1188P-201	RNA, U6 small nuclear 1188, pseudogene	snRNA	9606	12:116082	3
ENST00000383958	RNU6-1189P-201	RNA, U6 small nuclear 1189, pseudogene	snRNA	9606	X:4708750	2
ENST00000384343	RNU6-1207P-201	RNA, U6 small nuclear 1207, pseudogene	snRNA	9606	10:451887	1
ENST00000459588	RNU6-1208P-201	RNA, U6 small nuclear 1208, pseudogene	snRNA	9606	1:2477787	1
ENST00000517185	RNU6-1231P-201	RNA, U6 small nuclear 1231, pseudogene	snRNA	9606	10:102803	2
ENST00000364841	RNU6-1303P-201	RNA, U6 small nuclear 1303, pseudogene	snRNA	9606	9:7763520	1
ENST00000517187	RNU6-1315P-201	RNA, U6 small nuclear 1315, pseudogene	snRNA	9606	12:295424	2
ENST00000384355	RNU6-22P-201	RNA, U6 small nuclear 22, pseudogene	snRNA	9606	16:691756	2
ENST00000517078	RNU6-430P-201	RNA, U6 small nuclear 430, pseudogene	snRNA	9606	16:894433	2
ENST00000391068	RNU6-482P-201	RNA, U6 small nuclear 482, pseudogene	snRNA	9606	5:1127783	1
ENST00000362356	RNU6-50P-201	RNA, U6 small nuclear 50, pseudogene	snRNA	9606	X:4651776	2
ENST00000363425	RNU6-527P-201	RNA, U6 small nuclear 527, pseudogene	snRNA	9606	6:1066077	2
ENST00000363354	RNU6-626P-201	RNA, U6 small nuclear 626, pseudogene	snRNA	9606	6:5694573	1
ENST00000516946	RNU6-703P-201	RNA, U6 small nuclear 703, pseudogene	snRNA	9606	8:9804885	2
ENST00000364837	RNU6-818P-201	RNA, U6 small nuclear 818, pseudogene	snRNA	9606	4:8820170	1
ENST00000364943	RNU6-915P-201	RNA, U6 small nuclear 915, pseudogene	snRNA	9606	2:1956703	2
ENST00000410432	RNU6-96P-201	RNA, U6 small nuclear 96, pseudogene	snRNA	9606	7:6639519	2
ENST00000459303	RNU7-170P-201	RNA, U7 small nuclear 170 pseudogene	snRNA	9606	12:121908	2
ENST00000363063	RNY1P16-201	RNA, Ro-associated Y1 pseudogene 16	misc_RNA	9606	12:101719	3
ENST00000401753	ROCK2-006	Rho-associated, coiled-coil containing protein kinase 2	protein_coding	9606	2:1118333	2
ENST00000413707	RP11-100E13.3-001		processed_pseudogene	9606	1:2246611	1
ENST00000524239	RP11-101E19.4-001		processed_pseudogene	9606	8:4683757	1
ENST00000549459	RP11-1079J22.1-001		processed_pseudogene	9606	12:849395	2
ENST00000414775	RP11-107G24.3-001		unprocessed_pseudogene	9606	1:2470853	1
ENST00000578367	RP11-107K17.1-001	long intergenic non-protein coding RNA 1543	lincRNA	9606	18:264229	1
ENST00000585536	RP11-1094M14.10-001		lincRNA	9606	17:355400	1
ENST00000478593	RP11-109G23.1-001		processed_pseudogene	9606	4:7876849	3
ENST00000518580	RP11-109J4.1-001		lincRNA	9606	5:1609317	1
ENST00000481228	RP11-10G15.4-001		processed_pseudogene	9606	3:1213569	2
ENST00000578881	RP11-1109M24.5-001		lincRNA	9606	17:222664	2
ENST00000519983	RP11-1114I9.1-001		lincRNA	9606	8:7929771	2
ENST00000538317	RP11-114G22.1-003		lincRNA	9606	12:228594	1
ENST00000450613	RP11-114J13.1-001		lincRNA	9606	5:1260768	2
ENST00000503691	RP11-116A1.1-001		lincRNA	9606	5:8958120	2
ENST00000589510	RP11-116O18.3-002		antisense	9606	18:456693	1
ENST00000503353	RP11-119H12.3-001		processed_pseudogene	9606	4:1127970	1
ENST00000504528	RP11-119H12.4-001		processed_pseudogene	9606	4:1128263	1
ENST00000511590	RP11-119H12.6-001		antisense	9606	4:1128802	3
ENST00000618187	RP11-120E5.1-001		processed_pseudogene	9606	9:6663575	2
ENST00000447087	RP11-1217F2.9-001		unprocessed_pseudogene	9606	7:5718789	2
ENST00000551699	RP11-121G22.3-008		lincRNA	9606	12:812791	6
ENST00000485829	RP11-123J14.1-001		processed_pseudogene	9606	4:7689103	2
ENST00000465411	RP11-124D2.1-001		processed_pseudogene	9606	14:232299	1
ENST00000432774	RP11-12D5.2-001		processed_pseudogene	9606	X:2783217	1
ENST00000624274	RP11-133K1.8-001		TEC	9606	15:402449	1
ENST00000451200	RP11-136C24.3-001	Uncharacterized protein {ECO:0000313|Ensembl:ENSP00000477043}	processed_transcript	9606	3:4280944	2
ENST00000555721	RP11-140I24.1-001		lincRNA	9606	16:733868	2
ENST00000567049	RP11-146F11.2-001		processed_pseudogene	9606	16:306269	2
ENST00000487111	RP11-147I3.1-003		processed_transcript	9606	11:749135	2
ENST00000468671	RP11-14K2.1-001		processed_pseudogene	9606	16:128811	2
ENST00000533300	RP11-159H10.3-002		antisense	9606	8:6983415	4
ENST00000589021	RP11-15A1.7-001		antisense	9606	19:439968	1
ENST00000588492	RP11-15B24.5-012		lincRNA	9606	9:8227345	1
ENST00000589973	RP11-15B24.5-016		lincRNA	9606	9:8249195	2
ENST00000570227	RP11-160C18.2-002		processed_transcript	9606	15:787336	2
ENST00000556228	RP11-163M18.1-003		antisense	9606	14:445090	2
ENST00000513415	RP11-167P23.2-002		processed_transcript	9606	X:5565472	2
ENST00000546865	RP11-171L9.1-001		lincRNA	9606	12:784489	1
ENST00000548782	RP11-175P13.3-001		transcribed_unprocessed_pseu	9606	12:100180	1
ENST00000445213	RP11-180I4.1-001		processed_pseudogene	9606	9:9565291	1
ENST00000606384	RP11-182L21.5-001		sense_intronic	9606	10:796631	2
ENST00000612699	RP11-189B4.7-001		lincRNA	9606	13:464742	2
ENST00000504144	RP11-18H21.1-003		lincRNA	9606	4:1521007	2
ENST00000604299	RP11-190G13.4-001		processed_pseudogene	9606	7:1302559	2
ENST00000433123	RP11-193H5.5-001		processed_pseudogene	9606	1:2379480	1
ENST00000579660	RP11-19P22.6-001		processed_pseudogene	9606	17:276778	1
ENST00000375867	RP11-1E11.1-001		processed_pseudogene	9606	9:8818067	2
ENST00000514650	RP11-1J11.1-001		processed_pseudogene	9606	4:7130025	2
ENST00000434264	RP11-206L10.9-017		lincRNA	9606	1:778770-[illegible]	1
ENST00000442302	RP11-208C17.4-001		processed_pseudogene	9606	10:950316	1
ENST00000624008	RP11-20J15.5-001	uncharacterized protein LOC100130539	lincRNA	9606	10:442927	2
ENST00000493084	RP11-216N21.1-001		processed_pseudogene	9606	8:8601681	1
ENST00000561413	RP11-219B17.2-002		antisense	9606	15:606816	1
ENST00000432108	RP11-219F10.1-001		lincRNA	9606	10:839116	2
ENST00000424997	RP11-219I21.1-001		processed_pseudogene	9606	3:4246964	2
ENST00000530418	RP11-222N13.1-001		lincRNA	9606	11:159109	2
ENST00000433646	RP11-236P24.3-001		lincRNA	9606	X:5309371	1
ENST00000613682	RP11-23B5.2-001		processed_pseudogene	9606	18:484782	2
ENST00000417800	RP11-240M16.1-001		lincRNA	9606	6:1379453	2
ENST00000618630	RP11-241M13.2-001		unprocessed_pseudogene	9606	2:8485071	1
ENST00000599172	RP11-247A12.8-001		antisense	9606	9:1291758	2
ENST00000605180	RP11-24C14.1-001		processed_pseudogene	9606	6:6671024	1
ENST00000618383	RP11-255H23.5-001		unprocessed_pseudogene	9606	19:238425	2
ENST00000548349	RP11-25J3.2-001		processed_pseudogene	9606	12:756000	2
ENST00000427176	RP11-261C10.1-001		lincRNA	9606	1:2430058	1
ENST00000437691	RP11-261C10.2-004		lincRNA	9606	1:2430477	1
ENST00000468625	RP11-274K13.5-001		processed_pseudogene	9606	X:1360978	2
ENST00000584916	RP11-277J6.3-001		lincRNA	9606	17:736625	2
ENST00000497218	RP11-286B5.1-001		processed_pseudogene	9606	14:484898	1
ENST00000580956	RP11-288C17.3-001		processed_pseudogene	9606	18:135765	1
ENST00000513711	RP11-292D4.1-001		lincRNA	9606	4:1469574	2
ENST00000448562	RP11-29O6.2-001		processed_pseudogene	9606	X:1438063	2

ENST00000505623	RP11-2O17.2-003		antisense	9606	5:6165847	1
ENST00000427435	RP11-2P2.1-001		processed_pseudogene	9606	1:2082552	2
ENST00000510964	RP11-305P14.1-001		lincRNA	9606	5:6629846	1
ENST00000447976	RP1-130H16.18-004	Uncharacterized protein {ECO:0000313|Ensembl:ENSP00000400319}	nonsense_mediated_decay	9606	22:302852	2
ENST00000504017	RP11-313E19.2-001		antisense	9606	4:1766696	2
ENST00000608056	RP11-314C9.2-001		lincRNA	9606	2:3819334	1
ENST00000567853	RP11-315F22.1-001		lincRNA	9606	2:2397349	1
ENST00000510068	RP11-319G6.1-001		antisense	9606	3:1393898	2
ENST00000593122	RP11-322E11.5-003		lincRNA	9606	18:354438	6
ENST00000556286	RP11-322L17.1-001		processed_pseudogene	9606	14:442909	2
ENST00000401776	RP11-323I14__A.1-001		processed_pseudogene	9606	6:5627652	1
ENST00000445455	RP11-325E14.2-001		processed_pseudogene	9606	X:8056014	3
ENST00000528510	RP11-334E6.3-001		antisense	9606	11:119380	2
ENST00000428146	RP11-340I6.5-001		processed_pseudogene	9606	7:6387634	1
ENST00000419853	RP11-343J18.1-001		lincRNA	9606	9:1262701	1
ENST00000426359	RP11-345I18.6-001		processed_pseudogene	9606	1:1792711	1
ENST00000511468	RP11-346J10.1-001		lincRNA	9606	5:1004014	1
ENST00000521984	RP11-348M17.2-001		antisense	9606	5:1616873	1
ENST00000537470	RP11-351O2.2-001		unprocessed_pseudogene	9606	12:127624	1
ENST00000485172	RP11-356M17.1-001		processed_pseudogene	9606	4:7640125	2
ENST00000557949	RP11-356M20.1-001		processed_pseudogene	9606	15:594069	2
ENST00000536931	RP11-361I14.2-001		antisense	9606	12:187147	4
ENST00000423044	RP11-361M4.1-001		processed_pseudogene	9606	9:8460411	1
ENST00000484765	RP11-368I23.2-001		lincRNA	9606	3:1689033	1
ENST00000579356	RP11-370A5.1-002		antisense	9606	18:227234	1
ENST00000418270	RP11-379F12.4-001		lincRNA	9606	10:805154	2
ENST00000433085	RP11-383C6.2-001		lincRNA	9606	10:114994	1
ENST00000562191	RP11-389G6.3-001		sense_overlapping	9606	3:9775952	1
ENST00000438139	RP11-38O14.5-001		transcribed_unprocessed_pseu	9606	1:2029865	1
ENST00000508655	RP11-392L5.2-001		processed_pseudogene	9606	4:1291331	2
ENST00000449954	RP11-395L14.3-001		lincRNA	9606	2:1135124	2
ENST00000472008	RP11-399D15.1-001		processed_pseudogene	9606	12:101228	2
ENST00000445073	RP11-399E6.1-002		lincRNA	9606	1:4124257	2
ENST00000413598	RP11-3L10.2-001		processed_pseudogene	9606	7:1214190	1
ENST00000422194	RP11-402P6.9-001		lincRNA	9606	X:7166299	3
ENST00000448475	RP11-403N16.2-001		lincRNA	9606	9:7987469	1
ENST00000440329	RP11-403P14.1-001		processed_pseudogene	9606	1:1649213	2
ENST00000605247	RP11-409K15.1-001		processed_pseudogene	9606	6:6745634	1
ENST00000602854	RP11-420L9.4-003		sense_overlapping	9606	6:3831933	1
ENST00000502634	RP11-420O16.2-001		processed_pseudogene	9606	5:2030404	3
ENST00000505613	RP11-432M8.8-001		processed_pseudogene	9606	5:1760478	1
ENST00000515227	RP11-434D9.1-006		lincRNA	9606	5:6779322	1
ENST00000607823	RP11-436D23.1-003		lincRNA	9606	6:9781670	1
ENST00000509718	RP11-438C19.2-001		lincRNA	9606	5:1150312	1
ENST00000529141	RP11-438N5.4-001		processed_pseudogene	9606	11:396127	1
ENST00000564851	RP11-439I14.1-001		transcribed_processed_pseudo	9606	16:647367	2
ENST00000515178	RP11-440I14.2-001		lincRNA	9606	4:1745237	1
ENST00000542812	RP11-444B24.2-001		processed_pseudogene	9606	12:681433	2
ENST00000450784	RP11-452F19.3-001		lincRNA	9606	1:2228150	2
ENST00000457636	RP11-452F19.3-004		lincRNA	9606	1:2228150	2
ENST00000515455	RP11-455F5.3-001		antisense	9606	16:300964	1
ENST00000442231	RP11-457D2.3-001		antisense	9606	10:178628	1
ENST00000535964	RP11-459D22.1-001		processed_pseudogene	9606	12:184246	1
ENST00000509711	RP11-463H12.2-001		lincRNA	9606	4:5494363	2
ENST00000477176	RP11-463H24.1-001		processed_pseudogene	9606	3:1362055	2
ENST00000556834	RP11-463J10.2-001		antisense	9606	14:519161	1
ENST00000507410	RP11-466J24.1-001		processed_pseudogene	9606	4:4358632	2
ENST00000564046	RP11-467L24.1-001		lincRNA	9606	16:643647	2
ENST00000576250	RP11-473M20.7-001		antisense	9606	16:305199	2
ENST00000542449	RP11-476M19.2-001		lincRNA	9606	12:336783	1
ENST00000509476	RP11-479O16.1-002		antisense	9606	5:5874158	1
ENST00000413757	RP11-482E14.2-002		lincRNA	9606	10:353511	1
ENST00000507389	RP11-485M7.3-001		lincRNA	9606	5:1331110	1
ENST00000561884	RP11-488I20.5-001		processed_pseudogene	9606	16:353473	2
ENST00000615076	RP11-493L12.7-001		lincRNA	9606	12:472656	1
ENST00000534576	RP11-510I21.1-001		processed_pseudogene	9606	8:1402268	2
ENST00000482216	RP11-510I6.1-001		processed_pseudogene	9606	5:1215758	2
ENST00000512500	RP11-517I3.1-002		antisense	9606	5:1264804	2
ENST00000437289	RP11-526P5.2-001		lincRNA	9606	10:250180	2
ENST00000511875	RP11-535C7.1-001		lincRNA	9606	4:5204480	1
ENST00000569661	RP11-548M13.1-001		lincRNA	9606	15:550567	1
ENST00000411879	RP1-154K9.2-001		lincRNA	9606	X:4225245	2
ENST00000443523	RP11-556E13.1-001		antisense	9606	10:525569	1
ENST00000562685	RP11-556H2.2-001		sense_intronic	9606	16:791059	1
ENST00000584105	RP11-557B23.3-001		unprocessed_pseudogene	9606	17:677745	2
ENST00000624276	RP11-560A15.6-001		TEC	9606	20:571618	1
ENST00000524017	RP11-566H8.2-001		lincRNA	9606	8:3133919	2
ENST00000568730	RP11-56L13.1-002		lincRNA	9606	16:322629	1
ENST00000455940	RP11-572H4.1-001		processed_pseudogene	9606	9:3116561	1
ENST00000583846	RP11-585K6.2-001		processed_pseudogene	9606	18:685279	2
ENST00000507903	RP11-586E1.1-001		processed_pseudogene	9606	5:6302291	1
ENST00000514187	RP11-588L15.2-001		antisense	9606	4:4042611	2
ENST00000471411	RP11-615I16.1-001		processed_pseudogene	9606	12:246426	2
ENST00000590085	RP11-622J9.1-001		processed_pseudogene	9606	18:607946	1
ENST00000519013	RP11-62H7.2-001		transcribed_processed_pseudo	9606	8:8961757	4
ENST00000623263	RP11-63I8.1-001		TEC	9606	18:463857	2
ENST00000603429	RP11-642D6.1-001		processed_pseudogene	9606	2:6234894	2
ENST00000443685	RP11-662M24.1-001		lincRNA	9606	12:130651	2
ENST00000528913	RP11-665E10.5-001		processed_pseudogene	9606	11:878150	1
ENST00000625137	RP11-678K21.3-001		TEC	9606	11:910938	2
ENST00000572530	RP11-683L23.1-004	Tubulin beta-8 chain-like protein LOC260334	processed_transcript	9606	18:49815-	1
ENST00000510731	RP11-692C23.1-001		processed_pseudogene	9606	5:2787451	1
ENST00000510764	RP11-696N14.1-006		antisense	9606	4:9926767	2

ENST00000522498	RP11-6I2.4-001		lincRNA	9606	8:7410351 3
ENST00000569328	RP11-700H13.1-001		lincRNA	9606	16:588478 2
ENST00000505122	RP11-700N1.1-001		lincRNA	9606	4:1199395 1
ENST00000547902	RP11-70F11.7-001		lincRNA	9606	12:498612 2
ENST00000423149	RP11-716O23.2-001		lincRNA	9606	6:1148722 1
ENST00000581173	RP11-728F11.4-003		antisense	9606	11:117833 4
ENST00000434426	RP11-73B2.6-001		processed_transcript	9606	7:6392484 1
ENST00000422707	RP11-748L13.2-001		unprocessed_pseudogene	9606	10:273313 2
ENST00000541797	RP11-749H20.1-001		lincRNA	9606	12:127793 2
ENST00000426027	RP11-760D2.4-001		processed_pseudogene	9606	7:5652542 1
ENST00000527351	RP11-762B21.5-001		processed_pseudogene	9606	11:123135 6
ENST00000569293	RP11-764E7.1-001		lincRNA	9606	2:1938983 1
ENST00000554187	RP11-783L4.1-001		lincRNA	9606	14:661115 1
ENST00000416507	RP11-787B4.2-001		lincRNA	9606	9:1160123 1
ENST00000592961	RP11-799D4.1-001		transcribed_processed_pseudo	9606	17:351909 1
ENST00000554043	RP11-7F17.4-001		lincRNA	9606	14:770481 1
ENST00000562617	RP11-809F4.3-001		lincRNA	9606	3:1759389 1
ENST00000591853	RP11-815J4.1-001		lincRNA	9606	18:120815 1
ENST00000428618	RP11-816B4.1-001		processed_pseudogene	9606	3:1728309 2
ENST00000531260	RP11-81M19.1-001		unprocessed_pseudogene	9606	11:898957 1
ENST00000581528	RP11-822E23.3-001		processed_pseudogene	9606	17:215525 1
ENST00000624018	RP11-826F13.1-001		TEC	9606	11:706033 2
ENST00000552441	RP11-845M18.6-001		antisense	9606	12:523066 1
ENST00000578634	RP11-846F4.1-001		processed_pseudogene	9606	17:225196 1
ENST00000607010	RP11-85G21.3-001		lincRNA	9606	1:1572807 2
ENST00000568867	RP11-883G10.1-001		unprocessed_pseudogene	9606	15:679850 2
ENST00000495382	RP11-88H10.2-001		lincRNA	9606	3:1459399 1
ENST00000455010	RP11-90C4.3-001		lincRNA	9606	1:5594508 1
ENST00000554319	RP11-945F5.1-001		lincRNA	9606	14:455679 1
ENST00000545739	RP11-955H22.3-001		lincRNA	9606	12:127324 1
ENST00000472881	RP11-959F10.5-001		processed_pseudogene	9606	11:630467 3
ENST00000545814	RP11-972L6.2-001		processed_pseudogene	9606	12:636516 1
ENST00000625026	RP11-9N12.2-001		antisense	9606	4:1542359 1
ENST00000456978	RP1-215K18.4-001		unitary_pseudogene	9606	X:8497321 1
ENST00000424283	RP1-261G23.5-001		lincRNA	9606	6:4372278 2
ENST00000424696	RP1-272L16.1-001		lincRNA	9606	1:2095284 2
ENST00000403583	RP1-288M22.1-001		processed_pseudogene	9606	6:7155095 2
ENST00000431947	RP1-297M16.2-001		lincRNA	9606	6:1511966 1
ENST00000412817	RP13-26D14.2-001		processed_pseudogene	9606	X:7036148 1
ENST00000427132	RP13-30A9.1-001		antisense	9606	20:626336 2
ENST00000616013	RP13-362E11.3-001		processed_pseudogene	9606	X:9020889 1
ENST00000449941	RP13-455A7.1-001		lincRNA	9606	22:478606 2
ENST00000405827	RP1-59B16.1-001		processed_pseudogene	9606	6:2397187 1
ENST00000476099	RP1-93H18.6-001		antisense	9606	6:1164607 1
ENST00000433173	RP3-323B6.1-001		processed_pseudogene	9606	X:6546908 1
ENST00000607703	RP3-337O18.9-001		antisense	9606	20:458926 4
ENST00000431968	RP3-477M7.6-001		processed_pseudogene	9606	1:8909742 1
ENST00000474641	RP3-523E19.2-001		antisense	9606	6:5391897 2
ENST00000610000	RP4-569D19.8-001		lincRNA	9606	22:355269 2
ENST00000425449	RP4-663N10.1-001		antisense	9606	1:1152830 2
ENST00000439662	RP4-700A9.1-001		processed_pseudogene	9606	1:6515448 3
ENST00000488389	RP4-710M3.1-001		processed_pseudogene	9606	11:303681 2
ENST00000432257	RP4-763G1.1-001		processed_pseudogene	9606	1:6727805 1
ENST00000450469	RP4-784A16.3-001		sense_intronic	9606	1:5329179 3
ENST00000412004	RP5-1052M9.1-001		processed_pseudogene	9606	X:1243261 2
ENST00000588260	RP5-1067M6.3-001		lincRNA	9606	17:443286 1
ENST00000413033	RP5-1106H14.1-001		processed_pseudogene	9606	7:1013888 2
ENST00000617644	RP5-1116H23.5-001		lincRNA	9606	20:504985 1
ENST00000562756	RP5-1119A7.17-001		sense_overlapping	9606	22:365608 1
ENST00000447169	RP5-1158E12.2-001		processed_pseudogene	9606	X:4591311 2
ENST00000610899	RP5-824H1.1-001		processed_pseudogene	9606	X:1474763 2
ENST00000620506	RP5-827E24.1-002		lincRNA	9606	20:614364 2
ENST00000419780	RP5-837O21.3-001		unprocessed_pseudogene	9606	1:9392661 1
ENST00000428673	RP5-865N13.2-001		lincRNA	9606	1:2321600 2
ENST00000433121	RP5-908M14.5-001		antisense	9606	20:624295 2
ENST00000536422	RP5-944M2.1-001		lincRNA	9606	12:126400 2
ENST00000419450	RP5-952N6.1-001		lincRNA	9606	1:7070645 1
ENST00000422038	RP6-102O10.1-001		lincRNA	9606	1:5738657 2
ENST00000546228	RPEP6-001	ribulose-5-phosphate-3-epimerase pseudogene 6	processed_pseudogene	9606	11:721312 1
ENST00000536489	RPH3AL-003	rabphilin 3A-like (without C2 domains)	protein_coding	9606	17:213645 3
ENST00000549273	RPL18-014	ribosomal protein L18	protein_coding	9606	19:486153 1
ENST00000466869	RPL19P18-001	ribosomal protein L19 pseudogene 18	processed_pseudogene	9606	17:958990 1
ENST00000491732	RPL21P129-001	ribosomal protein L21 pseudogene 129	processed_pseudogene	9606	19:775068 2
ENST00000418090	RPL21P38-001	ribosomal protein L21 pseudogene 38	processed_pseudogene	9606	2:1715870 2
ENST00000417197	RPL22P18-001	ribosomal protein L22 pseudogene 18	processed_pseudogene	9606	10:801259 2
ENST00000582485	RPL26-013	ribosomal protein L26	protein_coding	9606	17:837754 1
ENST00000498718	RPL29P22-001	ribosomal protein L29 pseudogene 22	processed_pseudogene	9606	11:631158 2
ENST00000491087	RPL31P56-001	ribosomal protein L31 pseudogene 56	processed_pseudogene	9606	16:553527 2
ENST00000394668	RPL34-003	ribosomal protein L34	protein_coding	9606	4:1086206 2
ENST00000484185	RPL35AP30-001	ribosomal protein L35a pseudogene 30	processed_pseudogene	9606	12:119959 2
ENST00000496996	RPL36P16-001	ribosomal protein L36 pseudogene 16	processed_pseudogene	9606	19:411698 2
ENST00000435890	RPL37P21-001	ribosomal protein L37 pseudogene 21	processed_pseudogene	9606	13:683313 1
ENST00000507308	RPL37P25-001	ribosomal protein L37 pseudogene 25	processed_pseudogene	9606	5:5439943 2
ENST00000448908	RPL37P4-001	ribosomal protein L37 pseudogene 4	processed_pseudogene	9606	21:195938 2
ENST00000478876	RPL7P43-001	ribosomal protein L7 pseudogene 43	processed_pseudogene	9606	12:767215 1
ENST00000546990	RPLP0-025	ribosomal protein, large, P0	protein_coding	9606	12:120196 4
ENST00000398220	RPLP2P1-001	ribosomal protein, large P2, pseudogene 1	processed_pseudogene	9606	6:2796517 2
ENST00000412619	RPS12P24-001	ribosomal protein S12 pseudogene 24	processed_pseudogene	9606	13:372208 1
ENST00000436276	RPS15AP5-001	ribosomal protein S15a pseudogene 5	processed_pseudogene	9606	10:121713 1
ENST00000396783	RPS17P1-001	ribosomal protein S17 pseudogene 1	processed_pseudogene	9606	6:2948927 2
ENST00000492356	RPS21-004	ribosomal protein S21	processed_transcript	9606	20:623871 1
ENST00000466210	RPS23P7-001	ribosomal protein S23 pseudogene 7	processed_pseudogene	9606	17:623973 4
ENST00000460178	RPS29P12-001	ribosomal protein S29 pseudogene 12	processed_pseudogene	9606	5:1809457 1

ENST00000402041	RPS29P13-001	ribosomal protein S29 pseudogene 13	processed_pseudogene	9606	6:1170486 2
ENST00000413190	RPS3AP1-001	ribosomal protein S3A pseudogene 1	processed_pseudogene	9606	21:204304 1
ENST00000472940	RPS6KB1-003	ribosomal protein S6 kinase, 70kDa, polypeptide 1	nonsense_mediated_decay	9606	17:598930 2
ENST00000435134	RPS7P2-001	ribosomal protein S7 pseudogene 2	processed_pseudogene	9606	1:1540788 3
ENST00000414704	RPS7P9-001	ribosomal protein S7 pseudogene 9	processed_pseudogene	9606	10:807164 2
ENST00000426177	RPS8P6-001	ribosomal protein S8 pseudogene 6	processed_pseudogene	9606	3:308714-: 2
ENST00000433555	RPUSD3-013	RNA pseudouridylate synthase domain containing 3	protein_coding	9606	3:9841408 2
ENST00000262850	RRAGB-001	Ras-related GTP binding B	protein_coding	9606	X:5571788 2
ENST00000339997	RTN3-001	reticulon 3	protein_coding	9606	11:636814 2
ENST00000474642	RTN4R-007	reticulon 4 receptor	processed_transcript	9606	22:202428 4
ENST00000591123	SAFB2-005	scaffold attachment factor B2	nonsense_mediated_decay	9606	19:559883 4
ENST00000314471	SAMD4B-001	sterile alpha motif domain containing 4B	protein_coding	9606	19:393424 1
ENST00000611159	SAMD4B-202	sterile alpha motif domain containing 4B	protein_coding	9606	19:393566 1
ENST00000429225	SAR1AP4-001	SAR1 homolog A (S. cerevisiae) pseudogene 4	processed_pseudogene	9606	X:7588454 2
ENST00000547018	SCAF11-008	SR-related CTD-associated factor 11	non_stop_decay	9606	12:459267 1
ENST00000554776	SCFD1-026	sec1 family domain containing 1	nonsense_mediated_decay	9606	14:306282 2
ENST00000445224	SCHIP1-004	schwannomin interacting protein 1	protein_coding	9606	3:1598398 2
ENST00000402904	SCMH1-205	sex comb on midleg homolog 1 (Drosophila)	protein_coding	9606	1:4102720 1
ENST00000507401	SCN1A-007	sodium channel, voltage-gated, type I, alpha subunit	retained_intron	9606	2:1660733 2
ENST00000530265	SCUBE2-009	signal peptide, CUB domain, EGF-like 2	processed_transcript	9606	11:904807 1
ENST00000533862	SCYL1-006	SCY1-like 1 (S. cerevisiae)	protein_coding	9606	11:655251 1
ENST00000427380	SDAD1P3-001	SDA1 domain containing 1 pseudogene 3	processed_pseudogene	9606	3:3377196 1
ENST00000396721	SDR16C5-003	short chain dehydrogenase/reductase family 16C, member 5	protein_coding	9606	8:5630001 1
ENST00000320982	SEC14L4-004	SEC14-like 4 (S. cerevisiae)	nonsense_mediated_decay	9606	22:304889 1
ENST00000371706	SEC16A-001	SEC16 homolog A (S. cerevisiae)	protein_coding	9606	9:1364401 2
ENST00000436790	SEC31A-006	SEC31 homolog A (S. cerevisiae)	processed_transcript	9606	4:8286321 2
ENST00000465273	SELENBP1-004	selenium binding protein 1	retained_intron	9606	1:1513666 2
ENST00000549518	SENP1-004	SUMO1/sentrin specific peptidase 1	protein_coding	9606	12:480453 1
ENST00000583114	SEPT4-017	septin 4	protein_coding	9606	17:585202 2
ENST00000578131	SEPT4-021	septin 4	nonsense_mediated_decay	9606	17:585220 2
ENST00000428867	SEPT7P2-009	septin 7 pseudogene 2	retained_intron	9606	7:4575798 2
ENST00000434048	SEPT7P5-001	septin 7 pseudogene 5	processed_pseudogene	9606	7:6349579 1
ENST00000354833	SERF1A-002	small EDRK-rich factor 1A (telomeric)	protein_coding	9606	5:7090068 1
ENST00000380750	SERF1B-001	small EDRK-rich factor 1B (centromeric)	protein_coding	9606	5:7002526 1
ENST00000525920	SERGEF-014	secretion regulating guanine nucleotide exchange factor	protein_coding	9606	11:177882 2
ENST00000438844	SERPINB13-002	serpin peptidase inhibitor, clade B (ovalbumin), member 13	nonsense_mediated_decay	9606	18:635874 1
ENST00000531605	SERPING1-010	serpin peptidase inhibitor, clade G (C1 inhibitor), member 1	processed_transcript	9606	11:576020 1
ENST00000461103	SETD8-003	SET domain containing (lysine methyltransferase) 8	retained_intron	9606	12:123389 1
ENST00000534805	SETDB1-013	SET domain, bifurcated 1	protein_coding	9606	1:1509263 1
ENST00000423837	SETP9-001	SET pseudogene 9	transcribed_processed_pseudo	9606	1:1606701 2
ENST00000489537	SF3A3-008	splicing factor 3a, subunit 3, 60kDa	processed_transcript	9606	1:3797872 2
ENST00000554600	SHMT2-034	serine hydroxymethyltransferase 2 (mitochondrial)	processed_transcript	9606	12:572296 1
ENST00000438092	SHPRH-005	SNF2 histone linker PHD RING helicase, E3 ubiquitin protein ligase	protein_coding	9606	6:1458848 2
ENST00000483946	SIDT1-003	SID1 transmembrane family, member 1	retained_intron	9606	3:1135331 1
ENST00000381621	SIRPD-003	signal-regulatory protein delta	protein_coding	9606	20:153425 1
ENST00000620437	SIRT2-203	sirtuin 2	protein_coding	9606	19:388790 2
ENST00000505131	SLAIN2-011	SLAIN motif family, member 2	nonsense_mediated_decay	9606	4:4837982 2
ENST00000616933	SLC12A5-203	solute carrier family 12 (potassium/chloride transporter), member 5	protein_coding	9606	20:460291 1
ENST00000561080	SLC12A6-020	solute carrier family 12 (potassium/chloride transporter), member 6	nonsense_mediated_decay	9606	15:342332 1
ENST00000344941	SLC15A5-001	solute carrier family 15, member 5	protein_coding	9606	12:161884 2
ENST00000581287	SLC16A3-001	solute carrier family 16 (monocarboxylate transporter), member 3	protein_coding	9606	17:822336 1
ENST00000369779	SLC16A4-001	solute carrier family 16, member 4	protein_coding	9606	1:1103628 1
ENST00000441077	SLC22A18-004	solute carrier family 22, member 18	processed_transcript	9606	11:291668 2
ENST00000463571	SLC22A18-007	solute carrier family 22, member 18	retained_intron	9606	11:291623 2
ENST00000539841	SLC22A8-005	solute carrier family 22 (organic anion transporter), member 8	retained_intron	9606	11:629891 2
ENST00000545862	SLC25A10-201	solute carrier family 25 (mitochondrial carrier; dicarboxylate transporter), member 10	protein_coding	9606	17:817122 2
ENST00000443844	SLC25A3P1-001	solute carrier family 25 (mitochondrial carrier; phosphate carrier), member 3 pseudogene 1	transcribed_processed_pseudo	9606	1:5343837 1
ENST00000398802	SLC25A45-201	solute carrier family 25, member 45	protein_coding	9606	11:653751 1
ENST00000468432	SLC25A51P1-001	solute carrier family 25, member 51 pseudogene 1	processed_pseudogene	9606	6:6578841 2
ENST00000411502	SLC26A11-005	solute carrier family 26 (anion exchanger), member 11	protein_coding	9606	17:802204 2
ENST00000393723	SLC26A5-010	solute carrier family 26 (anion exchanger), member 5	protein_coding	9606	7:1033743 1
ENST00000544936	SLC2A3-009	solute carrier family 2 (facilitated glucose transporter), member 3	nonsense_mediated_decay	9606	12:793135 2
ENST00000473157	SLC2A4RG-007	SLC2A4 regulator	processed_transcript	9606	20:637419 2
ENST00000508585	SLC2A9-012	solute carrier family 2 (facilitated glucose transporter), member 9	processed_transcript	9606	4:9771153 2
ENST00000507685	SLC34A1-003	solute carrier family 34 (type II sodium/phosphate contransporter), member 1	retained_intron	9606	5:1773844 2
ENST00000319429	SLC35E3-002	solute carrier family 35, member E3	processed_transcript	9606	12:687479 1
ENST00000547252	SLC38A2-006	solute carrier family 38, member 2	processed_transcript	9606	12:463646 2
ENST00000610458	SLC38A3-008	solute carrier family 38, member 3	protein_coding	9606	3:5020524 1
ENST00000621456	SLC38A3-009	solute carrier family 38, member 3	protein_coding	9606	3:5020768 1
ENST00000377891	SLC3A2-003	solute carrier family 3 (amino acid transporter heavy chain), member 2	protein_coding	9606	11:628561 1
ENST00000411411	SLC41A2-009	solute carrier family 41 (magnesium transporter), member 2	protein_coding	9606	12:104928 3
ENST00000457377	SLC4A7-008	solute carrier family 4, sodium bicarbonate cotransporter, member 7	nonsense_mediated_decay	9606	3:2737660 2
ENST00000484027	SLC50A1-012	solute carrier family 50 (sugar efflux transporter), member 1	processed_transcript	9606	1:1551363 4
ENST00000536262	SLC5A8-001	solute carrier family 5 (sodium/monocarboxylate cotransporter), member 8	protein_coding	9606	12:101155 2
ENST00000551612	SLC6A15-007	solute carrier family 6 (neutral amino acid transporter), member 15	protein_coding	9606	12:848731 2
ENST00000528860	SLC7A8-007	solute carrier family 7 (amino acid transporter light chain, L system), member 8	nonsense_mediated_decay	9606	14:231267 2
ENST00000528186	SLC7A8-012	solute carrier family 7 (amino acid transporter light chain, L system), member 8	retained_intron	9606	14:231295 2
ENST00000601757	SLC8A2-007	solute carrier family 8 (sodium/calcium exchanger), member 2	processed_transcript	9606	19:474324 5
ENST00000592108	SLFN11-007	schlafen family member 11	protein_coding	9606	17:353518 2
ENST00000416658	SLMAP-006	sarcolemma associated protein	protein_coding	9606	3:5786458 3
ENST00000509418	SMARCAD1-006	SWI/SNF-related, matrix-associated actin-dependent regulator of chromatin, subfamily a, containing DEAD/H box 1	protein_coding	9606	4:9425329 2
ENST00000551966	SMARCD1-011	SWI/SNF related, matrix associated, actin dependent regulator of chromatin, subfamily d, member 1	protein_coding	9606	12:500853 2
ENST00000543702	SMG1P4-006	SMG1 pseudogene 4	processed_transcript	9606	16:218807 1
ENST00000586005	SMIM22-008	small integral membrane protein 22	protein_coding	9606	16:479542 1
ENST00000462420	SMO-003	smoothened, frizzled class receptor	nonsense_mediated_decay	9606	7:1292061 2
ENST00000508027	SMR3B-004	submaxillary gland androgen regulated protein 3B	retained_intron	9606	4:7038313 1
ENST00000563830	SNAP23-017	synaptosomal-associated protein, 23kDa	protein_coding	9606	15:425152 2
ENST00000384676	SNORA1.1-204	Small nucleolar RNA SNORA1	snoRNA	9606	11:195911 1
ENST00000516634	SNORA62.1-205	Small nucleolar RNA SNORA62/SNORA6 family	snoRNA	9606	16:697859 1
ENST00000516293	SNORA63.1-211	Small nucleolar RNA SNORA63	snoRNA	9606	7:6532671 2
ENST00000383910	SNORA70.1-206	Small nucleolar RNA SNORA70	snoRNA	9606	1:2019784 2
ENST00000364915	SNORD117-201	small nucleolar RNA, C/D box 117	snoRNA	9606	6:3153637 2
ENST00000516231	snoU109.1-206	Small nucleolar RNA U109	snoRNA	9606	16:686302 2

ENST00000459483	snoU13.1-220	Small nucleolar RNA U13	snoRNA	9606	17:489566 2
ENST00000458945	snoU13.1-227	Small nucleolar RNA U13	snoRNA	9606	X:1361343 2
ENST00000409838	SNX10-004	sorting nexin 10	protein_coding	9606	7:2636432 2
ENST00000456554	SNX18P4-001	sorting nexin 18 pseudogene 4	processed_pseudogene	9606	9:6608235 2
ENST00000457286	SNX8-006	sorting nexin 8	protein_coding	9606	7:2264393 2
ENST00000613678	SOCS7-002	suppressor of cytokine signaling 7	protein_coding	9606	17:383522 1
ENST00000467753	SOGA3-002	SOGA family member 3	protein_coding	9606	6:1275165 2
ENST00000371245	SORBS1-006	sorbin and SH3 domain containing 1	protein_coding	9606	10:953150 2
ENST00000396652	SOSTDC1-002	sclerostin domain containing 1	protein_coding	9606	7:1646154 1
ENST00000458341	SP140L-001	SP140 nuclear body protein-like	protein_coding	9606	2:2303420 1
ENST00000414961	SPAG16-010	sperm associated antigen 16	processed_transcript	9606	2:2132960 2
ENST00000340011	SPAM1-002	sperm adhesion molecule 1 (PH-20 hyaluronidase, zona pellucida binding)	protein_coding	9606	7:1239253 1
ENST00000574457	SPATA22-012	spermatogenesis associated 22	protein_coding	9606	17:346274 2
ENST00000428649	SPATA31A3-001	SPATA31 subfamily A, member 3	protein_coding	9606	9:6698630 1
ENST00000619140	SPATA31A7-009	SPATA31 subfamily A, member 7	processed_transcript	9606	9:6119132 2
ENST00000434692	SPATA31B1P-001	SPATA31 subfamily B, member 1, pseudogene	unprocessed_pseudogene	9606	9:8205764 1
ENST00000508733	SPINK5-008	serine peptidase inhibitor, Kazal type 5	protein_coding	9606	5:1480639 1
ENST00000395389	SPN-001	sialophorin	protein_coding	9606	16:296629 4
ENST00000504871	SPON2-011	spondin 2, extracellular matrix protein	processed_transcript	9606	4:1172587 1
ENST00000436032	SRGAP3-AS2-002	SRGAP3 antisense RNA 2	antisense	9606	3:9192493 1
ENST00000432981	SRGAP3-AS3-001	SRGAP3 antisense RNA 3	antisense	9606	3:9216895 1
ENST00000576924	SRRM2-003	serine/arginine repetitive matrix 2	protein_coding	9606	16:275262 1
ENST00000504985	SSBP2-008	single-stranded DNA binding protein 2	protein_coding	9606	5:8141993 2
ENST00000527413	SSSCA1-003	Sjogren syndrome/scleroderma autoantigen 1	retained_intron	9606	11:655708 1
ENST00000522652	ST3GAL1-003	ST3 beta-galactoside alpha-2,3-sialyltransferase 1	protein_coding	9606	8:1334589 2
ENST00000537795	ST8SIA1-014	ST8 alpha-N-acetyl-neuraminide alpha-2,8-sialyltransferase 1	processed_transcript	9606	12:221071 2
ENST00000583884	STARD3-025	StAR-related lipid transfer (START) domain containing 3	nonsense_mediated_decay	9606	17:396590 2
ENST00000479417	STAT5A-005	signal transducer and activator of transcription 5A	retained_intron	9606	17:423041 2
ENST00000381060	STATH-003	statherin	protein_coding	9606	4:6999596 4
ENST00000423004	STK25-026	serine/threonine kinase 25	protein_coding	9606	2:2414963 2
ENST00000368620	STK32C-005	serine/threonine kinase 32C	protein_coding	9606	10:132226 2
ENST00000493263	STK35-003	serine/threonine kinase 35	nonsense_mediated_decay	9606	20:210289 1
ENST00000407753	STK38L-013	serine/threonine kinase 38 like	nonsense_mediated_decay	9606	12:273021 2
ENST00000540889	STX2-005	syntaxin 2	retained_intron	9606	12:130798 2
ENST00000564581	SUB1P3-001	SUB1 pseudogene 3	processed_pseudogene	9606	16:456281 2
ENST00000413931	SUGCT-004	succinyl-CoA:glutarate-CoA transferase	protein_coding	9606	7:4013501 2
ENST00000525999	SULF1-015	sulfatase 1	protein_coding	9606	8:6956386 2
ENST00000406622	SUN2-017	Sad1 and UNC84 domain containing 2	protein_coding	9606	22:387359 2
ENST00000453192	SUN3-202	Sad1 and UNC84 domain containing 3	protein_coding	9606	7:4798733 1
ENST00000494793	SUSD4-002	sushi domain containing 4	protein_coding	9606	1:2232208 1
ENST00000420416	SUV39H2-004	suppressor of variegation 3-9 homolog 2 (Drosophila)	protein_coding	9606	10:148789 2
ENST00000587442	SUV420H2-009	suppressor of variegation 4-20 homolog 2 (Drosophila)	processed_transcript	9606	19:553427 2
ENST00000372898	SWI5-003	SWI5 recombination repair homolog (yeast)	protein_coding	9606	9:1282754 2
ENST00000479535	SYCE1-002	synaptonemal complex central element protein 1	retained_intron	9606	10:133553 2
ENST00000343131	SYCE1-201	synaptonemal complex central element protein 1	protein_coding	9606	10:133554 4
ENST00000550658	SYF2P1-001	SYF2 pre-mRNA-splicing factor pseudogene 1	processed_pseudogene	9606	14:305417 1
ENST00000555612	SYNE2-008	spectrin repeat containing, nuclear envelope 2	nonsense_mediated_decay	9606	14:641414 2
ENST00000479510	SYNGAP1-002	synaptic Ras GTPase activating protein 1	retained_intron	9606	6:3342007 1
ENST00000381535	SYNGR1-005	synaptogyrin 1	protein_coding	9606	22:393641 2
ENST00000472598	SYP-002	synaptophysin	protein_coding	9606	X:4918913 1
ENST00000592956	SYT5-009	synaptotagmin V	retained_intron	9606	19:551735 1
ENST00000592935	SYT5-012	synaptotagmin V	processed_transcript	9606	19:551716 5
ENST00000615887	TAC3-201	tachykinin 3	protein_coding	9606	12:570100 1
ENST00000458173	TACC3-011	transforming, acidic coiled-coil containing protein 3	protein_coding	9606	4:1723110 1
ENST00000403609	TANK-003	TRAF family member-associated NFKB activator	protein_coding	9606	2:1611796 2
ENST00000429217	TANK-016	TRAF family member-associated NFKB activator	protein_coding	9606	2:1611603 2
ENST00000606292	TARID-006	TCF21 antisense RNA inducing promoter demethylation	antisense	9606	6:1335371 2
ENST00000451017	TARID-007	TCF21 antisense RNA inducing promoter demethylation	antisense	9606	6:1335022 2
ENST00000512840	TBC1D19-004	TBC1 domain family, member 19	protein_coding	9606	4:2657668 2
ENST00000617882	TBC1D3F-201	TBC1 domain family, member 3F	protein_coding	9606	17:363778 2
ENST00000459913	TBL2-007	transducin (beta)-like 2	processed_transcript	9606	7:7356966 1
ENST00000586706	TBX2-AS1-005	TBX2 antisense RNA 1	antisense	9606	17:613934 1
ENST00000587717	TBXA2R-002	thromboxane A2 receptor	processed_transcript	9606	19:359792 2
ENST00000554424	TC2N-006	tandem C2 domains, nuclear	retained_intron	9606	14:918120 1
ENST00000371331	TCEANC2-003	transcription elongation factor A (SII) N-terminal and central domain containing 2	protein_coding	9606	1:5405413 2
ENST00000459662	TCL6-003	T-cell leukemia/lymphoma 6 (non-protein coding)	lincRNA	9606	14:956511 4
ENST00000491611	TCTEX1D1-002	Tctex1 domain containing 1	processed_transcript	9606	1:6675253 2
ENST00000481318	TDRD9-006	tudor domain containing 9	retained_intron	9606	14:104049 2
ENST00000575592	TEKT1-002	tektin 1	nonsense_mediated_decay	9606	17:679966 1
ENST00000584699	TEX14-006	testis expressed 14	processed_transcript	9606	17:585566 2
ENST00000518257	TEX15-003	testis expressed 15	processed_transcript	9606	8:3087499 3
ENST00000310125	TFDP3-001	transcription factor Dp family, member 3	protein_coding	9606	X:1332166 2
ENST00000403298	TFEB-201	transcription factor EB	protein_coding	9606	6:4168397 2
ENST00000398431	TFF3-002	trefoil factor 3 (intestinal)	protein_coding	9606	21:423120 2
ENST00000620299	TFG-203	TRK-fused gene	protein_coding	9606	3:1007093 2
ENST00000453013	TFPI-005	tissue factor pathway inhibitor (lipoprotein-associated coagulation inhibitor)	protein_coding	9606	2:1874968 2
ENST00000518058	TG-004	thyroglobulin	protein_coding	9606	8:1329629 2
ENST00000556285	TGFB3-002	transforming growth factor, beta 3	protein_coding	9606	14:759630 1
ENST00000561067	TGM1-004	transglutaminase 1	protein_coding	9606	14:242623 2
ENST00000409232	TGOLN2-006	trans-golgi network protein 2	protein_coding	9606	2:8532189 2
ENST00000469226	TH-009	tyrosine hydroxylase	retained_intron	9606	11:216689 1
ENST00000476349	THNSL2-007	threonine synthase-like 2 (S. cerevisiae)	retained_intron	9606	2:8817895 1
ENST00000253952	THOC6-006	THO complex 6 homolog (Drosophila)	protein_coding	9606	16:302406 2
ENST00000546243	THRA-006	thyroid hormone receptor, alpha	protein_coding	9606	17:400689 1
ENST00000408005	THSD7A-004	thrombospondin, type I, domain containing 7A	retained_intron	9606	7:1137462 2
ENST00000612912	TJAP1-205	tight junction associated protein 1 (peripheral)	protein_coding	9606	6:4349698 2
ENST00000346128	TJP1-001	tight junction protein 1	protein_coding	9606	15:296993 1
ENST00000487660	TKT-013	transketolase	retained_intron	9606	3:5323485 1
ENST00000529148	TMA16P1-001	translation machinery associated 16 homolog pseudogene 1	processed_pseudogene	9606	11:587962 2
ENST00000514135	TMEM161B-006	transmembrane protein 161B	protein_coding	9606	5:8818963 2
ENST00000511218	TMEM161B-010	transmembrane protein 161B	protein_coding	9606	5:8819582 4
ENST00000296776	TMEM174-001	transmembrane protein 174	protein_coding	9606	5:7317319 1
ENST00000511737	TMEM174-002	transmembrane protein 174	processed_transcript	9606	5:7317329 1

ENST00000468449	TMEM183A-002	transmembrane protein 183A	processed_transcript	9606	1:2030077 1
ENST00000450925	TMEM191A-001	transmembrane protein 191A (pseudogene)	lincRNA	9606	22:207011 1
ENST00000545656	TMEM191C-202	transmembrane protein 191C	lincRNA	9606	22:214664 1
ENST00000444545	TMEM194B-003	transmembrane protein 194B	nonsense_mediated_decay	9606	2:1905144 2
ENST00000321326	TMEM214-003	transmembrane protein 214	nonsense_mediated_decay	9606	2:2703295 2
ENST00000478104	TMEM222-009	transmembrane protein 222	nonsense_mediated_decay	9606	1:2732228 2
ENST00000528595	TMEM225-002	transmembrane protein 225	protein_coding	9606	11:123883 1
ENST00000438392	TMEM244-201	transmembrane protein 244	protein_coding	9606	6:1298313 4
ENST00000449601	TMEM247-002	transmembrane protein 247	processed_transcript	9606	2:4642919 1
ENST00000462884	TMEM45A-003	transmembrane protein 45A	processed_transcript	9606	3:1005192 2
ENST00000468704	TMEM50A-005	transmembrane protein 50A	processed_transcript	9606	1:2533846 2
ENST00000522780	TMEM71-010	transmembrane protein 71	protein_coding	9606	8:1326850 2
ENST00000463427	TMEM87B-004	transmembrane protein 87B	processed_transcript	9606	2:1120860 2
ENST00000538566	TMIGD1-002	transmembrane and immunoglobulin domain containing 1	protein_coding	9606	17:303163 1
ENST00000343315	TMPO-014	thymopoietin	protein_coding	9606	12:985156 1
ENST00000422787	TMPRSS15-002	transmembrane protease, serine 15	protein_coding	9606	21:183651 2
ENST00000504642	TNFAIP8-004	tumor necrosis factor, alpha-induced protein 8	protein_coding	9606	5:1193553 2
ENST00000492571	TNFRSF9-002	tumor necrosis factor receptor superfamily, member 9	nonsense_mediated_decay	9606	1:7933127 2
ENST00000374044	TNFSF15-002	tumor necrosis factor (ligand) superfamily, member 15	protein_coding	9606	9:1147893 2
ENST00000367312	TNNI1-003	troponin I type 1 (skeletal, slow)	protein_coding	9606	1:2014098 1
ENST00000592920	TNNT1-008	troponin T type 1 (skeletal, slow)	processed_transcript	9606	19:551380 2
ENST00000560751	TNP2-002	transition protein 2 (during histone to protamine replacement)	retained_intron	9606	16:112677 1
ENST00000613741	TONSL-201	tonsoku-like, DNA repair protein	protein_coding	9606	8:1444366 2
ENST00000577934	TP53I13-007	tumor protein p53 inducible protein 13	nonsense_mediated_decay	9606	17:295685 2
ENST00000380148	TP53TG3D-001	TP53 target 3D	nonsense_mediated_decay	9606	16:322533 1
ENST00000538212	TPK1-014	thiamin pyrophosphokinase 1	protein_coding	9606	7:1444519 1
ENST00000469607	TPO-012	thyroid peroxidase	protein_coding	9606	2:1484665 1
ENST00000428886	TPP1-002	tripeptidyl peptidase I	retained_intron	9606	11:661699 1
ENST00000480281	TPST1-002	tyrosylprotein sulfotransferase 1	processed_transcript	9606	7:6620533 3
ENST00000530705	TPT1-001	tumor protein, translationally-controlled 1	protein_coding	9606	13:453334 1
ENST00000417243	TRAPPC12-018	trafficking protein particle complex 12	nonsense_mediated_decay	9606	2:3421883 2
ENST00000587818	TRAPPC6A-005	trafficking protein particle complex 6A	retained_intron	9606	19:451647 2
ENST00000610566	TRBC2-201	T cell receptor beta constant 2	TR_C_gene	9606	7:1426707 1
ENST00000390413	TRBJ2-2-001	T cell receptor beta joining 2-2	TR_J_gene	9606	7:1427965 2
ENST00000390363	TRBV9-001	T cell receptor beta variable 9	TR_V_gene	9606	7:1423918 2
ENST00000435350	TRHDE-AS1-001	TRHDE antisense RNA 1	antisense	9606	12:722535 1
ENST00000549957	TRHDE-AS1-003	TRHDE antisense RNA 1	retained_intron	9606	12:722535 2
ENST00000479800	TRIM17-004	tripartite motif containing 17	protein_coding	9606	1:2284091 2
ENST00000532195	TRIM29-014	tripartite motif containing 29	retained_intron	9606	11:120127 2
ENST00000272395	TRIM43-001	tripartite motif containing 43	protein_coding	9606	2:9559201 1
ENST00000448984	TRIM49C-001	tripartite motif containing 49C	protein_coding	9606	11:900311 1
ENST00000244891	TRIM51-001	tripartite motif-containing 51	protein_coding	9606	11:558859 1
ENST00000503141	TRIML2-005	tripartite motif family-like 2	nonsense_mediated_decay	9606	4:1880913 1
ENST00000417475	TRIP6-003	thyroid hormone receptor interactor 6	nonsense_mediated_decay	9606	7:1008673 1
ENST00000399736	TRO-011	trophinin	protein_coding	9606	X:5492081 2
ENST00000358082	TRPM3-015	transient receptor potential cation channel, subfamily M, member 3	protein_coding	9606	9:7053595 2
ENST00000360774	TRPM6-001	transient receptor potential cation channel, subfamily M, member 6	protein_coding	9606	9:7472249 1
ENST00000491992	TSPAN2-003	tetraspanin 2	processed_transcript	9606	1:1150504 2
ENST00000446063	TSPAN32-002	tetraspanin 32	nonsense_mediated_decay	9606	11:230201 1
ENST00000430181	TSPEAR-AS1-002	TSPEAR antisense RNA 1	antisense	9606	21:445106 2
ENST00000574792	TSSK3-004	testis-specific serine kinase 3	processed_transcript	9606	1:3235272 1
ENST00000465625	TTC14-010	tetratricopeptide repeat domain 14	processed_transcript	9606	3:1806081 2
ENST00000418766	TTC3-008	tetratricopeptide repeat domain 3	protein_coding	9606	21:370732 2
ENST00000477524	TTLL7-003	tubulin tyrosine ligase-like family, member 7	processed_transcript	9606	1:8386502 2
ENST00000482783	TTLL7-008	tubulin tyrosine ligase-like family, member 7	processed_transcript	9606	1:8391768 2
ENST00000597686	TUBB4A-005	tubulin, beta 4A class IVa	protein_coding	9606	19:649613 2
ENST00000594290	TUBB4A-012	tubulin, beta 4A class IVa	nonsense_mediated_decay	9606	19:649617 2
ENST00000561967	TUBB8-007	tubulin, beta 8 class VIII	protein_coding	9606	10:47057-‹ 2
ENST00000376094	TUBD1-003	tubulin, delta 1	protein_coding	9606	17:598602 1
ENST00000498606	TUFT1-012	tuftelin 1	retained_intron	9606	1:1515403 2
ENST00000540687	TUG1-003	taurine up-regulated 1 (non-protein coding)	antisense	9606	22:309706 2
ENST00000610453	TULP3-201	tubby like protein 3	protein_coding	9606	12:289090 2
ENST00000554321	TUNAR-003	TCL1 upstream neural differentiation-associated RNA	lincRNA	9606	14:958768 2
ENST00000509177	TUSC3-013	tumor suppressor candidate 3	retained_intron	9606	8:1562307 2
ENST00000477805	TUSC7-004	tumor suppressor candidate 7 (non-protein coding)	antisense	9606	3:1167097 1
ENST00000441872	TXNP1-001	thioredoxin pseudogene 1	processed_pseudogene	9606	10:119683 1
ENST00000412543	UBAP2-005	ubiquitin associated protein 2	protein_coding	9606	9:3394451 1
ENST00000414760	UBE2V1P9-001	ubiquitin-conjugating enzyme E2 variant 1 pseudogene 9	processed_pseudogene	9606	X:9028953 1
ENST00000434717	UBE2V2P1-001	ubiquitin-conjugating enzyme E2 variant 2 pseudogene 1	processed_pseudogene	9606	10:190514 2
ENST00000429033	UBE3AP2-001	ubiquitin protein ligase E3A pseudogene 2	processed_pseudogene	9606	21:310606 2
ENST00000590068	UBL5-009	ubiquitin-like 5	protein_coding	9606	19:982789 2
ENST00000609762	UBN2-004	ubinuclein 2	retained_intron	9606	7:1392936 1
ENST00000540917	UEVLD-014	UEV and lactate/malate dehyrogenase domains	processed_transcript	9606	11:185648 1
ENST00000623646	UG0898H09-001	uncharacterized LOC643763	lincRNA	9606	8:6297786 2
ENST00000453224	ULK4-007	unc-51 like kinase 4	nonsense_mediated_decay	9606	3:4193586 2
ENST00000508903	UNKL-002	unkempt family zinc finger-like	protein_coding	9606	16:136501 1
ENST00000625190	USP17L8-201	ubiquitin specific peptidase 17-like family member 8	protein_coding	9606	8:7971661 1
ENST00000598197	USP29-004	ubiquitin specific peptidase 29	protein_coding	9606	19:571286 1
ENST00000444558	USP32P1-006	ubiquitin specific peptidase 32 pseudogene 1	processed_transcript	9606	17:167944 2
ENST00000545955	UTY-205	ubiquitously transcribed tetratricopeptide repeat containing, Y-linked	protein_coding	9606	Y:1324837 1
ENST00000623120	UTY-209	Histone demethylase UTY {ECO:0000313|Ensembl:ENSP00000442047}	protein_coding	9606	Y:1324985 2
ENST00000477288	VARS2-002	valyl-tRNA synthetase 2, mitochondrial	retained_intron	9606	6:3090824 1
ENST00000493155	VASH2-008	vasohibin 2	processed_transcript	9606	1:2129515 2
ENST00000381029	VCX3B-201	variable charge, X-linked 3B	protein_coding	9606	X:8465426 1
ENST00000434069	VDAC1P3-001	voltage-dependent anion channel 1 pseudogene 3	processed_pseudogene	9606	X:9198265 1
ENST00000311170	VN1R4-001	vomeronasal 1 receptor 4	protein_coding	9606	19:532666 2
ENST00000412929	VN1R66P-001	vomeronasal 1 receptor 66 pseudogene	unprocessed_pseudogene	9606	16:316483 1
ENST00000497645	VWA7-003	von Willebrand factor A domain containing 7	processed_transcript	9606	6:3177605 3
ENST00000461942	WDR31-003	WD repeat domain 31	processed_transcript	9606	9:1133156 2
ENST00000472600	WDR49-003	WD repeat domain 49	protein_coding	9606	3:1674786 1
ENST00000466760	WDR49-005	WD repeat domain 49	protein_coding	9606	3:1675547 1
ENST00000420061	WDR90-005	WD repeat domain 90	retained_intron	9606	16:649353 2
ENST00000557825	WDR93-005	WD repeat domain 93	processed_transcript	9606	15:897196 2

ENST00000573440	WFIKKN1-002	WAP, follistatin/kazal, immunoglobulin, kunitz and netrin domain containing 1	retained_intron	9606	16:629239 1
ENST00000471243	WLS-012	wntless Wnt ligand secretion mediator	protein_coding	9606	1:6815517 2
ENST00000474009	WNK2-007	WNK lysine deficient protein kinase 2	processed_transcript	9606	9:9329308 1
ENST00000497817	WNT5A-008	wingless-type MMTV integration site family, member 5A	retained_intron	9606	3:5547904 1
ENST00000397196	WNT5B-001	wingless-type MMTV integration site family, member 5B	protein_coding	9606	12:162919 2
ENST00000538854	WNT5B-003	wingless-type MMTV integration site family, member 5B	processed_transcript	9606	12:161666 1
ENST00000583096	WSB1-017	WD repeat and SOCS box containing 1	retained_intron	9606	17:273033 2
ENST00000574232	WSCD1-004	WSC domain containing 1	protein_coding	9606	17:607110 2
ENST00000472697	XRN1-005	5'-3' exoribonuclease 1	retained_intron	9606	3:1423762 3
ENST00000575674	XYLT1-004	xylosyltransferase I	retained_intron	9606	16:171980 2
ENST00000362760	Y_RNA.1-252	Y RNA	misc_RNA	9606	6:8984132 2
ENST00000362996	Y_RNA.1-280	Y RNA	misc_RNA	9606	2:1725581 2
ENST00000363171	Y_RNA.1-303	Y RNA	misc_RNA	9606	9:7518152 2
ENST00000363391	Y_RNA.1-328	Y RNA	misc_RNA	9606	9:1121692 2
ENST00000363985	Y_RNA.1-395	Y RNA	misc_RNA	9606	11:108084 1
ENST00000364879	Y_RNA.1-492	Y RNA	misc_RNA	9606	9:1146154 2
ENST00000365043	Y_RNA.1-510	Y RNA	misc_RNA	9606	7:1003307 1
ENST00000365600	Y_RNA.1-575	Y RNA	misc_RNA	9606	12:952757 1
ENST00000383955	Y_RNA.1-601	Y RNA	misc_RNA	9606	5:3150568 2
ENST00000384097	Y_RNA.1-638	Y RNA	misc_RNA	9606	17:503861 4
ENST00000384333	Y_RNA.1-675	Y RNA	misc_RNA	9606	7:1561861 2
ENST00000384358	Y_RNA.1-680	Y RNA	misc_RNA	9606	2:9615267 1
ENST00000384586	Y_RNA.1-731	Y RNA	misc_RNA	9606	3:8843119 2
ENST00000459424	Y_RNA.1-840	Y RNA	misc_RNA	9606	4:5550159 1
ENST00000588002	YIF1B-007	Yip1 interacting factor homolog B (S. cerevisiae)	protein_coding	9606	19:383074 2
ENST00000460903	YIPF3-002	Yip1 domain family, member 3	retained_intron	9606	6:4351553 1
ENST00000434742	Z83001.1-001		antisense	9606	11:317277 2
ENST00000408372	Z83826.1-201		miRNA	9606	X:1348957 2
ENST00000426659	ZBTB32-003	zinc finger and BTB domain containing 32	protein_coding	9606	19:357045 2
ENST00000588028	ZBTB7C-018	zinc finger and BTB domain containing 7C	protein_coding	9606	18:480410 2
ENST00000590437	ZBTB7C-027	zinc finger and BTB domain containing 7C	protein_coding	9606	18:480409 2
ENST00000474453	ZCCHC11-016	zinc finger, CCHC domain containing 11	protein_coding	9606	1:5243124 2
ENST00000508058	ZCCHC4-005	zinc finger, CCHC domain containing 4	retained_intron	9606	4:2536170 2
ENST00000552693	ZDHHC17-014	zinc finger, DHHC-type containing 17	retained_intron	9606	12:768439 1
ENST00000517334	ZDHHC2-005	zinc finger, DHHC-type containing 2	retained_intron	9606	8:1720801 2
ENST00000369404	ZDHHC6-002	zinc finger, DHHC-type containing 6	protein_coding	9606	10:112430 4
ENST00000401903	ZFAND2A-005	zinc finger, AN1-type domain 2A	protein_coding	9606	7:1152071 2
ENST00000558087	ZFAND6-021	zinc finger, AN1-type domain 6	protein_coding	9606	15:800751 2
ENST00000571720	ZFP90-024	ZFP90 zinc finger protein	retained_intron	9606	16:685620 2
ENST00000515169	ZFYVE28-007	zinc finger, FYVE domain containing 28	protein_coding	9606	4:2328534 2
ENST00000361625	ZFYVE9-003	zinc finger, FYVE domain containing 9	retained_intron	9606	1:5214237 2
ENST00000369967	ZGPAT-003	zinc finger, CCCH-type with G patch domain	protein_coding	9606	20:637080 2
ENST00000264370	ZGRF1-006	zinc finger, GRF-type containing 1	retained_intron	9606	4:1126055 1
ENST00000543668	ZKSCAN7P1-001	zinc finger with KRAB and SCAN domains 7 pseudogene 1	processed_pseudogene	9606	12:184930 1
ENST00000615423	ZMIZ2-202	zinc finger, MIZ-type containing 2	protein_coding	9606	7:4475625 1
ENST00000373981	ZMYM3-003	zinc finger, MYM-type 3	protein_coding	9606	X:712492C 1
ENST00000317538	ZMYM6-002	zinc finger, MYM-type 6	protein_coding	9606	1:3501724 1
ENST00000531501	ZNF123P-001	zinc finger protein 123, pseudogene	processed_pseudogene	9606	11:129295 2
ENST00000324823	ZNF185-203	zinc finger protein 185 (LIM domain)	protein_coding	9606	X:1529181 2
ENST00000565100	ZNF19-004	zinc finger protein 19	protein_coding	9606	16:714743 2
ENST00000462724	ZNF212-002	zinc finger protein 212	nonsense_mediated_decay	9606	7:1492396 1
ENST00000579322	ZNF236-004	zinc finger protein 236	protein_coding	9606	18:768491 2
ENST00000535298	ZNF324-201	zinc finger protein 324	protein_coding	9606	19:584670 1
ENST00000476796	ZNF33B-003	zinc finger protein 33B	retained_intron	9606	10:426318 2
ENST00000534729	ZNF345-009	zinc finger protein 345	processed_transcript	9606	19:368516 2
ENST00000461412	ZNF365-007	zinc finger protein 365	processed_transcript	9606	10:626438 2
ENST00000589413	ZNF383-004	zinc finger protein 383	protein_coding	9606	19:372182 2
ENST00000551771	ZNF385A-008	zinc finger protein 385A	protein_coding	9606	12:543701 1
ENST00000582337	ZNF407-002	zinc finger protein 407	protein_coding	9606	18:745978 2
ENST00000600353	ZNF418-007	zinc finger protein 418	processed_transcript	9606	19:579348 2
ENST00000561648	ZNF423-001	zinc finger protein 423	protein_coding	9606	16:494875 2
ENST00000455504	ZNF433-002	zinc finger protein 433	protein_coding	9606	19:120164 2
ENST00000411443	ZNF502-003	zinc finger protein 502	protein_coding	9606	3:4471267 2
ENST00000590766	ZNF506-011	zinc finger protein 506	protein_coding	9606	19:197909 2
ENST00000217130	ZNF512B-201	zinc finger protein 512B	protein_coding	9606	20:639596 2
ENST00000528012	ZNF517-009	zinc finger protein 517	protein_coding	9606	8:1447989 2
ENST00000409544	ZNF638-002	zinc finger protein 638	protein_coding	9606	2:7133176 1
ENST00000467231	ZNF644-007	zinc finger protein 644	processed_transcript	9606	1:9091639 2
ENST00000595534	ZNF682-009	zinc finger protein 682	nonsense_mediated_decay	9606	19:200071 2
ENST00000468455	ZNF692-014	zinc finger protein 692	processed_transcript	9606	1:2488557 2
ENST00000507078	ZNF721-004	zinc finger protein 721	processed_transcript	9606	4:435770-· 2
ENST00000355095	ZNF736-201	zinc finger protein 736	protein_coding	9606	7:6431280 1
ENST00000252797	ZNF764-001	zinc finger protein 764	protein_coding	9606	16:305537 2
ENST00000560038	ZNF774-003	zinc finger protein 774	nonsense_mediated_decay	9606	15:903523 1
ENST00000558115	ZNF774-004	zinc finger protein 774	processed_transcript	9606	15:903523 1
ENST00000600457	ZNF83-013	zinc finger protein 83	processed_transcript	9606	19:526540 5
ENST00000301096	ZNF83-201	zinc finger protein 83	protein_coding	9606	19:526123 2
ENST00000592587	ZNF846-006	zinc finger protein 846	protein_coding	9606	19:975987 2
ENST00000596534	ZNF85-009	zinc finger protein 85	protein_coding	9606	19:209365 2
ENST00000598862	ZNF85-012	zinc finger protein 85	protein_coding	9606	19:209366 2
ENST00000434475	ZNF90-201	zinc finger protein 90	protein_coding	9606	19:201249 1
ENST00000595533	ZNF91-003	zinc finger protein 91	protein_coding	9606	19:233621 2
ENST00000599281	ZNF91-007	zinc finger protein 91	processed_transcript	9606	19:233388 2
ENST00000600543	ZNF92P2-001	zinc finger protein 92 pseudogene 2	processed_pseudogene	9606	19:221286 1
ENST00000622597	ZNRD1-AS1_2.1-202	ZNRD1 antisense RNA 1 conserved region 2	misc_RNA	9606	10:121949 2
ENST00000431012	ZNRD1-AS1-005	ZNRD1 antisense RNA 1	antisense	9606	6:3005460 2
ENST00000466960	ZP3-004	zona pellucida glycoprotein 3 (sperm receptor)	retained_intron	9606	7:7643395 1
ENST00000604524	ZSWIM8-019	zinc finger, SWIM-type containing 8	protein_coding	9606	10:737856 1

Table S8. Angiogenes, specific Liver not Heart

Accession	Name	Description	Biotype	Tax ID	Location	# of Detections
ENST0000(	AADAC-001	arylacetamide deacetylase	protein_cc	9606	3:1518140	36
ENST0000(	ABCD2-001	ATP-binding cassette, sub-family D (ALD), member 2	protein_cc	9606	12:395500	10
ENST0000(	ABCG5-001	ATP-binding cassette, sub-family G (WHITE), member 5	protein_cc	9606	2:4381247	9
ENST0000(	ABCG8-001	ATP-binding cassette, sub-family G (WHITE), member 8	protein_cc	9606	2:4383896	7
ENST0000(	ABHD6-002	abhydrolase domain containing 6	protein_cc	9606	3:5823753	19
ENST0000(	AC012074.2-002		lincRNA	9606	2:2536913	24
ENST0000(	ACADL-001	acyl-CoA dehydrogenase, long chain	protein_cc	9606	2:2101879	6
ENST0000(	ACKR3-001	atypical chemokine receptor 3	protein_cc	9606	2:2365696	39
ENST0000(	ACOT12-001	acyl-CoA thioesterase 12	protein_cc	9606	5:8133000	4
ENST0000(	ACPT-001	acid phosphatase, testicular	protein_cc	9606	19:507904	13
ENST0000(	ACR-001	acrosin	protein_cc	9606	22:507381	31
ENST0000(	ACSS3-002	acyl-CoA synthetase short-chain family member 3	protein_cc	9606	12:810780	10
ENST0000(	ACY3-001	aspartoacylase (aminocyclase) 3	protein_cc	9606	11:676425	2
ENST0000(	ADAD2-004	adenosine deaminase domain containing 2	protein_cc	9606	16:841911	12
ENST0000(	ADAMTS5-001	ADAM metallopeptidase with thrombospondin type 1 motif, 5	protein_cc	9606	21:269179	20
ENST0000(	ADH1A-001	alcohol dehydrogenase 1A (class I), alpha polypeptide	protein_cc	9606	4:9927636	2
ENST0000(	ADH1B-001	alcohol dehydrogenase 1B (class I), beta polypeptide	protein_cc	9606	4:9930496	15
ENST0000(	ADORA3-010	adenosine A3 receptor	protein_cc	9606	1:1114994	12
ENST0000(	AFAP1L2-002	actin filament associated protein 1-like 2	protein_cc	9606	10:114294	30
ENST0000(	AFF3-201	AF4/FMR2 family, member 3	protein_cc	9606	2:9954725	34
ENST0000(	AFM-001	afamin	protein_cc	9606	4:7348168	2
ENST0000(	AGAP4-201	ArfGAP with GTPase domain, ankyrin repeat and PH domain 4	protein_cc	9606	10:458256	9
ENST0000(	AGXT-001	alanine-glyoxylate aminotransferase	protein_cc	9606	2:2408684	2
ENST0000(	AHSG-002	alpha-2-HS-glycoprotein	protein_cc	9606	3:1866130	13
ENST0000(	AIP-001	aryl hydrocarbon receptor interacting protein	protein_cc	9606	11:674830	44
ENST0000(	AIRE-001	autoimmune regulator	protein_cc	9606	21:442858	8
ENST0000(	AK5-004	adenylate kinase 5	processed_	9606	1:7728205	2
ENST0000(	AK7-001	adenylate kinase 7	protein_cc	9606	14:963921	32
ENST0000(	AK9-007	adenylate kinase 9	protein_cc	9606	6:1096316	38
ENST0000(	ALKBH4-001	alkB, alkylation repair homolog 4 (E. coli)	protein_cc	9606	7:1024562	3
ENST0000(	ALLC-001	allantoicase	protein_cc	9606	2:3658195	2
ENST0000(	ALOX15-201	arachidonate 15-lipoxygenase	protein_cc	9606	17:463092	7
ENST0000(	AMBP-001	alpha-1-microglobulin/bikunin precursor	protein_cc	9606	9:1140601	8
ENST0000(	AMDHD1-001	amidohydrolase domain containing 1	protein_cc	9606	12:959432	13
ENST0000(	AMH-001	anti-Mullerian hormone	protein_cc	9606	19:224931	35
ENST0000(	AMHR2-001	anti-Mullerian hormone receptor, type II	protein_cc	9606	12:534238	5
ENST0000(	AMIGO2-003	adhesion molecule with Ig-like domain 2	protein_cc	9606	12:470757	7
ENST0000(	ANGPTL1-001	angiopoietin-like 1	protein_cc	9606	1:1788497	10
ENST0000(	ANKRD10-004	ankyrin repeat domain 10	protein_cc	9606	13:110892	18
ENST0000(	ANKRD12-001	ankyrin repeat domain 12	protein_cc	9606	18:913677	4
ENST0000(	AP1G2-002	adaptor-related protein complex 1, gamma 2 subunit	protein_cc	9606	14:235595	5
ENST0000(	APCS-001	amyloid P component, serum	protein_cc	9606	1:1595878	5
ENST0000(	APLF-001	aprataxin and PNKP like factor	protein_cc	9606	2:6846756	39
ENST0000(	APLNR-001	apelin receptor	nonsense_	9606	11:572335	15
ENST0000(	APOA5-001	apolipoprotein A-V	protein_cc	9606	11:116789	7
ENST0000(	APOBEC2-001	apolipoprotein B mRNA editing enzyme, catalytic polypeptide-like 2	protein_cc	9606	6:4105330	23
ENST0000(	APOC3-001	apolipoprotein C-III	protein_cc	9606	11:116829	3
ENST0000(	APOE-001	apolipoprotein E	protein_cc	9606	19:449057	35
ENST0000(	APOH-001	apolipoprotein H (beta-2-glycoprotein I)	protein_cc	9606	17:662120	3
ENST0000(	APOL5-001	apolipoprotein L, 5	protein_cc	9606	22:357178	8
ENST0000(	AQP11-001	aquaporin 11	protein_cc	9606	11:775896	39
ENST0000(	ARHGEF10L-005	Rho guanine nucleotide exchange factor (GEF) 10-like	nonsense_	9606	1:1762600	4
ENST0000(	ARHGEF25-001	Rho guanine nucleotide exchange factor (GEF) 25	protein_cc	9606	12:576114	28
ENST0000(	ARL11-001	ADP-ribosylation factor-like 11	protein_cc	9606	13:496282	41
ENST0000(	ARR3-002	arrestin 3, retinal (X-arrestin)	protein_cc	9606	X:7026835	12
ENST0000(	ARRDC2-002	arrestin domain containing 2	protein_cc	9606	19:180081	35
ENST0000(	ARRDC4-001	arrestin domain containing 4	protein_cc	9606	15:979606	42
ENST0000(	ASGR2-001	asialoglycoprotein receptor 2	protein_cc	9606	17:710132	3
ENST0000(	ASIC3-005	acid-sensing (proton-gated) ion channel 3	protein_cc	9606	7:1510488	9
ENST0000(	ATP10B-004	ATPase, class V, type 10B	retained_i	9606	5:1606326	37
ENST0000(	ATP5I-001	ATP synthase, H+ transporting, mitochondrial Fo complex, subunit E	protein_cc	9606	4:672436-(	41
ENST0000(	ATP8B3-002	ATPase, aminophospholipid transporter, class I, type 8B, member 3	protein_cc	9606	19:178207	4
ENST0000(	AVPR1A-001	arginine vasopressin receptor 1A	protein_cc	9606	12:631427	21
ENST0000(	AZGP1-001	alpha-2-glycoprotein 1, zinc-binding	protein_cc	9606	7:9996673	8
ENST0000(	B3GALNT2-002	beta-1,3-N-acetylgalactosaminyltransferase 2	protein_cc	9606	1:2354648	2
ENST0000(	B3GNT3-001	UDP-GlcNAc:betaGal beta-1,3-N-acetylglucosaminyltransferase 3	protein_cc	9606	19:177951	8
ENST0000(	B9D2-001	B9 protein domain 2	protein_cc	9606	19:413544	4
ENST0000(	BAALC-001	brain and acute leukemia, cytoplasmic	protein_cc	9606	8:1031407	25
ENST0000(	BAALC-003	brain and acute leukemia, cytoplasmic	protein_cc	9606	8:1031407	12
ENST0000(	BAAT-201	bile acid CoA:amino acid N-acyltransferase	protein_cc	9606	9:1013604	11
ENST0000(	BATF2-001	basic leucine zipper transcription factor, ATF-like 2	protein_cc	9606	11:649879	22
ENST0000(	BCAM-001	basal cell adhesion molecule (Lutheran blood group)	protein_cc	9606	19:448091	41
ENST0000(	BCHE-001	butyrylcholinesterase	protein_cc	9606	3:1657729	30
ENST0000(	BCL7C-001	B-cell CLL/lymphoma 7C	protein_cc	9606	16:308877	40
ENST0000(	BHMT-001	betaine--homocysteine S-methyltransferase	protein_cc	9606	5:7911177	3
ENST0000(	BIK-001	BCL2-interacting killer (apoptosis-inducing)	protein_cc	9606	22:431107	38
ENST0000(	BIRC3-002	baculoviral IAP repeat containing 3	protein_cc	9606	11:102317	37
ENST0000(	BMP3-001	bone morphogenetic protein 3	protein_cc	9606	4:8103096	22
ENST0000(	BMPER-001	BMP binding endothelial regulator	protein_cc	9606	7:3390491	34
ENST0000(	BRI3-001	brain protein I3	protein_cc	9606	7:9828167	39
ENST0000(	BRINP1-001	bone morphogenetic protein/retinoic acid inducible neural-specific 1	protein_cc	9606	9:1191666	15
ENST0000(	BST2-001	bone marrow stromal cell antigen 2	protein_cc	9606	19:174029	44
ENST0000(	BTK-006	Bruton agammaglobulinemia tyrosine kinase	protein_cc	9606	X:1013494	3
ENST0000(	C11orf52-001	chromosome 11 open reading frame 52	protein_cc	9606	11:111918	26
ENST0000(	C16orf87-001	chromosome 16 open reading frame 87	protein_cc	9606	16:468020	43

ENST0000(	C1QB-201	complement component 1, q subcomponent, B chain	protein_cc	9606	1:2265318	2
ENST0000(	C1QTNF1-002	C1q and tumor necrosis factor related protein 1	protein_cc	9606	17:790242	25
ENST0000(	C1QTNF4-001	C1q and tumor necrosis factor related protein 4	protein_cc	9606	11:475896	39
ENST0000(	C20orf197-201	chromosome 20 open reading frame 197	protein_cc	9606	20:600559	2
ENST0000(	C3-001	complement component 3	protein_cc	9606	19:667770	24
ENST0000(	C3AR1-001	complement component 3a receptor 1	protein_cc	9606	12:805830	40
ENST0000(	C5orf63-201	chromosome 5 open reading frame 63	protein_cc	9606	5:1270513	9
ENST0000(	C5orf64-201	chromosome 5 open reading frame 64	processed_	9606	5:6168607	26
ENST0000(	C6orf203-001	chromosome 6 open reading frame 203	protein_cc	9606	6:1070282	37
ENST0000(	C7-001	complement component 7	protein_cc	9606	5:4090925	36
ENST0000(	C8G-004	complement component 8, gamma polypeptide	protein_cc	9606	9:1369452	16
ENST0000(	C8orf34-004	chromosome 8 open reading frame 34	protein_cc	9606	8:6862545	29
ENST0000(	C8orf4-001	chromosome 8 open reading frame 4	protein_cc	9606	8:4015345	43
ENST0000(	C9orf106-001	chromosome 9 open reading frame 106	lincRNA	9606	9:1293210	24
ENST0000(	CA11-001	carbonic anhydrase XI	protein_cc	9606	19:486379	35
ENST0000(	CA3-001	carbonic anhydrase III, muscle specific	protein_cc	9606	8:8543882	13
ENST0000(	CA5A-001	carbonic anhydrase VA, mitochondrial	protein_cc	9606	16:878815	29
ENST0000(	CADM4-001	cell adhesion molecule 4	protein_cc	9606	19:436223	43
ENST0000(	CALCOCO1-004	calcium binding and coiled-coil domain 1	protein_cc	9606	12:537085	4
ENST0000(	CALML3-001	calmodulin-like 3	protein_cc	9606	10:552400	2
ENST0000(	CAPN6-001	calpain 6	protein_cc	9606	X:1112451	2
ENST0000(	CAPS-001	calcyphosine	protein_cc	9606	19:591423	3
ENST0000(	CATIP-001	ciliogenesis associated TTC17 interacting protein	protein_cc	9606	2:2183568	2
ENST0000(	CBX8-001	chromobox homolog 8	protein_cc	9606	17:797921	43
ENST0000(	CCDC116-001	coiled-coil domain containing 116	protein_cc	9606	22:216327	29
ENST0000(	CCDC184-001	coiled-coil domain containing 184	protein_cc	9606	12:481835	42
ENST0000(	CCDC58-001	coiled-coil domain containing 58	protein_cc	9606	3:1223595	37
ENST0000(	CCDC8-001	coiled-coil domain containing 8	protein_cc	9606	19:464103	41
ENST0000(	CCDC85B-001	coiled-coil domain containing 85B	protein_cc	9606	11:658901	44
ENST0000(	CCL21-002	chemokine (C-C motif) ligand 21	protein_cc	9606	9:3470900	22
ENST0000(	CCNF-002	cyclin F	nonsense_	9606	16:242939	16
ENST0000(	CCNL1-001	cyclin L1	protein_cc	9606	3:1571475	26
ENST0000(	CCR1-001	chemokine (C-C motif) receptor 1	protein_cc	9606	3:4620170	2
ENST0000(	CCR7-001	chemokine (C-C motif) receptor 7	protein_cc	9606	17:405537	12
ENST0000(	CCS-009	copper chaperone for superoxide dismutase	protein_cc	9606	11:665932	23
ENST0000(	CD101-201	CD101 molecule	protein_cc	9606	1:1170017	23
ENST0000(	CD180-001	CD180 molecule	protein_cc	9606	5:6718227	16
ENST0000(	CD200R1-001	CD200 receptor 1	protein_cc	9606	3:1129226	5
ENST0000(	CD209-001	CD209 molecule	protein_cc	9606	19:773999	20
ENST0000(	CD22-002	CD22 molecule	protein_cc	9606	19:353291	2
ENST0000(	CD3D-001	CD3d molecule, delta (CD3-TCR complex)	protein_cc	9606	11:118338	3
ENST0000(	CD69-001	CD69 molecule	protein_cc	9606	12:975248	32
ENST0000(	CD7-001	CD7 molecule	protein_cc	9606	17:823148	13
ENST0000(	CD79A-001	CD79a molecule, immunoglobulin-associated alpha	protein_cc	9606	19:418771	33
ENST0000(	CD97-003	CD97 molecule	protein_cc	9606	19:143814	6
ENST0000(	CDH19-001	cadherin 19, type 2	protein_cc	9606	18:665010	5
ENST0000(	CDH3-001	cadherin 3, type 1, P-cadherin (placental)	protein_cc	9606	16:686448	27
ENST0000(	CDH6-001	cadherin 6, type 2, K-cadherin (fetal kidney)	protein_cc	9606	5:3119375	33
ENST0000(	CDKN2C-001	cyclin-dependent kinase inhibitor 2C (p18, inhibits CDK4)	protein_cc	9606	1:5096074	5
ENST0000(	CEACAM8-001	carcinoembryonic antigen-related cell adhesion molecule 8	protein_cc	9606	19:425802	25
ENST0000(	CEBPB-001	CCAAT/enhancer binding protein (C/EBP), beta	protein_cc	9606	20:501907	37
ENST0000(	CEBPE-001	CCAAT/enhancer binding protein (C/EBP), epsilon	protein_cc	9606	14:231173	8
ENST0000(	CELA3A-001	chymotrypsin-like elastase family, member 3A	protein_cc	9606	1:2200165	16
ENST0000(	CEP120-201	centrosomal protein 120kDa	protein_cc	9606	5:1233448	9
ENST0000(	CEP135-001	centrosomal protein 135kDa	protein_cc	9606	4:5594898	36
ENST0000(	CEP44-201	centrosomal protein 44kDa	protein_cc	9606	4:1742990	15
ENST0000(	CERS3-001	ceramide synthase 3	protein_cc	9606	15:100400	2
ENST0000(	CFAP43-003	cilia and flagella associated protein 43	protein_cc	9606	10:104167	14
ENST0000(	CFHR1-001	complement factor H-related 1	protein_cc	9606	1:1968197	8
ENST0000(	CFHR4-002	complement factor H-related 4	protein_cc	9606	1:1968880	2
ENST0000(	CHD5-001	chromodomain helicase DNA binding protein 5	protein_cc	9606	1:6101793	21
ENST0000(	CHD9-019	chromodomain helicase DNA binding protein 9	retained_i	9606	16:532351	38
ENST0000(	CHID1-001	chitinase domain containing 1	protein_cc	9606	11:868396	5
ENST0000(	CHKA-001	choline kinase alpha	protein_cc	9606	11:680528	19
ENST0000(	CHRNA9-001	cholinergic receptor, nicotinic, alpha 9 (neuronal)	protein_cc	9606	4:4033532	11
ENST0000(	CHST12-002	carbohydrate (chondroitin 4) sulfotransferase 12	protein_cc	9606	7:2403588	43
ENST0000(	CHST13-001	carbohydrate (chondroitin 4) sulfotransferase 13	protein_cc	9606	3:1265242	11
ENST0000(	CILP-001	cartilage intermediate layer protein, nucleotide pyrophosphohydrolase	protein_cc	9606	15:651947	14
ENST0000(	CIT-001	citron rho-interacting serine/threonine kinase	protein_cc	9606	12:119685	3
ENST0000(	CLCA3P-002	chloride channel accessory 3, pseudogene	processed_	9606	1:8663427	7
ENST0000(	CLDN19-001	claudin 19	protein_cc	9606	1:4273309	17
ENST0000(	CLEC10A-001	C-type lectin domain family 10, member A	protein_cc	9606	17:707453	13
ENST0000(	CLEC11A-001	C-type lectin domain family 11, member A	protein_cc	9606	19:507233	39
ENST0000(	CLEC16A-005	C-type lectin domain family 16, member A	protein_cc	9606	16:111239	12
ENST0000(	CLEC1B-005	C-type lectin domain family 1, member B	protein_cc	9606	12:999306	4
ENST0000(	CLEC2B-001	C-type lectin domain family 2, member B	protein_cc	9606	12:985298	36
ENST0000(	CLEC3B-001	C-type lectin domain family 3, member B	protein_cc	9606	3:4502618	6
ENST0000(	CLEC4F-001	C-type lectin domain family 4, member F	protein_cc	9606	2:7080864	5
ENST0000(	CLEC4M-201	C-type lectin domain family 4, member M	protein_cc	9606	19:776314	10
ENST0000(	CLK4-001	CDC-like kinase 4	protein_cc	9606	5:1786026	26
ENST0000(	CMKLR1-001	chemokine-like receptor 1	protein_cc	9606	12:108288	12
ENST0000(	CMTM8-001	CKLF-like MARVEL transmembrane domain containing 8	protein_cc	9606	3:3223867	41
ENST0000(	CNFN-001	cornifelin	protein_cc	9606	19:423870	34
ENST0000(	CNOT1-201	CCR4-NOT transcription complex, subunit 1	protein_cc	9606	16:585199	9
ENST0000(	CNPY4-001	canopy FGF signaling regulator 4	protein_cc	9606	7:1001196	22
ENST0000(	COL10A1-201	collagen, type X, alpha 1	protein_cc	9606	6:1161189	8
ENST0000(	COL3A1-001	collagen, type III, alpha 1	protein_cc	9606	2:1889743	41

ENST0000(	COL6A2-001	collagen, type VI, alpha 2	protein_cc	9606	21:460980	40
ENST0000(	CORO1A-001	coronin, actin binding protein, 1A	protein_cc	9606	16:301834	25
ENST0000(	COX17-001	COX17 cytochrome c oxidase copper chaperone	protein_cc	9606	3:1196692	41
ENST0000(	COX7A1-001	cytochrome c oxidase subunit VIIa polypeptide 1 (muscle)	protein_cc	9606	19:361509	42
ENST0000(	CPB2-001	carboxypeptidase B2 (plasma)	protein_cc	9606	13:460531	2
ENST0000(	CPLX4-001	complexin 4	protein_cc	9606	18:592954	4
ENST0000(	CPN2-001	carboxypeptidase N, polypeptide 2	protein_cc	9606	3:1943397	11
ENST0000(	CPNE3-201	copine III	protein_cc	9606	8:8648483	3
ENST0000(	CPO-001	carboxypeptidase O	protein_cc	9606	2:2069395	17
ENST0000(	CPZ-001	carboxypeptidase Z	protein_cc	9606	4:8592756	9
ENST0000(	CR1L-001	complement component (3b/4b) receptor 1-like	nonsense_	9606	1:2076774	3
ENST0000(	CRHBP-001	corticotropin releasing hormone binding protein	protein_cc	9606	5:7695271	20
ENST0000(	CRIPAK-001	cysteine-rich PAK1 inhibitor	protein_cc	9606	4:1391552	38
ENST0000(	CRTC2-009	CREB regulated transcription coactivator 2	nonsense_	9606	1:1539476	5
ENST0000(	CSNK1D-001	casein kinase 1, delta	protein_cc	9606	17:822426	19
ENST0000(	CSPP1-001	centrosome and spindle pole associated protein 1	protein_cc	9606	8:6706436	10
ENST0000(	CSTA-001	cystatin A (stefin A)	protein_cc	9606	3:1223252	5
ENST0000(	CT55-002	cancer/testis antigen 55	protein_cc	9606	X:1351565	7
ENST0000(	CTD-2369P2.2-002		lincRNA	9606	19:102214	44
ENST0000(	CTLA4-001	cytotoxic T-lymphocyte-associated protein 4	protein_cc	9606	2:2038677	4
ENST0000(	CTSH-001	cathepsin H	protein_cc	9606	15:789210	19
ENST0000(	CUL3-001	cullin 3	protein_cc	9606	2:2244701	15
ENST0000(	CUL4A-201	cullin 4A	protein_cc	9606	13:113208	4
ENST0000(	CUX2-001	cut-like homeobox 2	protein_cc	9606	12:111034	22
ENST0000(	CXCL13-001	chemokine (C-X-C motif) ligand 13	protein_cc	9606	4:7751175	3
ENST0000(	CXCL3-001	chemokine (C-X-C motif) ligand 3	protein_cc	9606	4:7403658	38
ENST0000(	CXCL9-001	chemokine (C-X-C motif) ligand 9	protein_cc	9606	4:7600127	6
ENST0000(	CXXC5-001	CXXC finger protein 5	protein_cc	9606	5:1396482	35
ENST0000(	CYB5A-006	cytochrome b5 type A (microsomal)	protein_cc	9606	18:742532	33
ENST0000(	CYBA-001	cytochrome b-245, alpha polypeptide	protein_cc	9606	16:886432	43
ENST0000(	CYP2A7-201	cytochrome P450, family 2, subfamily A, polypeptide 7	protein_cc	9606	19:408754	7
ENST0000(	CYP2C18-001	cytochrome P450, family 2, subfamily C, polypeptide 18	protein_cc	9606	10:946836	4
ENST0000(	CYP2C9-001	cytochrome P450, family 2, subfamily C, polypeptide 9	protein_cc	9606	10:949386	2
ENST0000(	CYP2E1-201	cytochrome P450, family 2, subfamily E, polypeptide 1	protein_cc	9606	10:133527	9
ENST0000(	CYP2W1-001	cytochrome P450, family 2, subfamily W, polypeptide 1	protein_cc	9606	7:983199-[illegible]	14
ENST0000(	CYP3A5-001	cytochrome P450, family 3, subfamily A, polypeptide 5	protein_cc	9606	7:9964819	2
ENST0000(	CYP7A1-001	cytochrome P450, family 7, subfamily A, polypeptide 1	protein_cc	9606	8:5849017	12
ENST0000(	CYP7B1-001	cytochrome P450, family 7, subfamily B, polypeptide 1	protein_cc	9606	8:6459613	6
ENST0000(	CYSLTR2-001	cysteinyl leukotriene receptor 2	protein_cc	9606	13:487065	21
ENST0000(	CYTIP-001	cytohesin 1 interacting protein	protein_cc	9606	2:1574146	6
ENST0000(	DAAM2-001	dishevelled associated activator of morphogenesis 2	protein_cc	9606	6:3979236	4
ENST0000(	DAPP1-001	dual adaptor of phosphotyrosine and 3-phosphoinositides	protein_cc	9606	4:9981683	15
ENST0000(	DCAF4L2-001	DDB1 and CUL4 associated factor 4-like 2	protein_cc	9606	8:8787074	4
ENST0000(	DCDC1-005	doublecortin domain containing 1	nonsense_	9606	11:308636	13
ENST0000(	DDTL-001	D-dopachrome tautomerase-like	protein_cc	9606	22:239669	11
ENST0000(	DEF6-001	differentially expressed in FDCP 6 homolog (mouse)	protein_cc	9606	6:3529785	41
ENST0000(	DEPDC7-001	DEP domain containing 7	protein_cc	9606	11:330158	28
ENST0000(	DEPTOR-001	DEP domain containing MTOR-interacting protein	protein_cc	9606	8:1198737	33
ENST0000(	DFNB31-001	deafness, autosomal recessive 31	protein_cc	9606	9:1144020	27
ENST0000(	DHDH-001	dihydrodiol dehydrogenase (dimeric)	protein_cc	9606	19:489336	4
ENST0000(	DLGAP3-201	discs, large (Drosophila) homolog-associated protein 3	protein_cc	9606	1:3486543	39
ENST0000(	DLL4-001	delta-like 4 (Drosophila)	protein_cc	9606	15:409293	41
ENST0000(	DNAH6-001	dynein, axonemal, heavy chain 6	protein_cc	9606	2:8451781	21
ENST0000(	DNAI2-001	dynein, axonemal, intermediate chain 2	protein_cc	9606	17:742742	2
ENST0000(	DNAJC18-001	DnaJ (Hsp40) homolog, subfamily C, member 18	protein_cc	9606	5:1394102	7
ENST0000(	DNAJC7-003	DnaJ (Hsp40) homolog, subfamily C, member 7	protein_cc	9606	17:419766	4
ENST0000(	DPM2-001	dolichyl-phosphate mannosyltransferase polypeptide 2, regulatory subunit	protein_cc	9606	9:1279350	29
ENST0000(	DRD4-001	dopamine receptor D4	protein_cc	9606	11:637293	27
ENST0000(	DSC2-001	desmocollin 2	protein_cc	9606	18:310588	26
ENST0000(	DSC2-002	desmocollin 2	protein_cc	9606	18:310659	10
ENST0000(	DSCAML1-001	Down syndrome cell adhesion molecule like 1	protein_cc	9606	11:117427	7
ENST0000(	DSG1-001	desmoglein 1	protein_cc	9606	18:313180	2
ENST0000(	DSG2-001	desmoglein 2	protein_cc	9606	18:314980	40
ENST0000(	DTX1-001	deltex 1, E3 ubiquitin ligase	protein_cc	9606	12:113057	24
ENST0000(	DUS1L-001	dihydrouridine synthase 1-like (S. cerevisiae)	protein_cc	9606	17:820575	26
ENST0000(	DUSP1-001	dual specificity phosphatase 1	protein_cc	9606	5:1727680	43
ENST0000(	DUSP2-001	dual specificity phosphatase 2	protein_cc	9606	2:9614316	36
ENST0000(	DUSP26-001	dual specificity phosphatase 26 (putative)	protein_cc	9606	8:3359133	11
ENST0000(	DVL2-001	dishevelled segment polarity protein 2	protein_cc	9606	17:722534	41
ENST0000(	EAF2-001	ELL associated factor 2	protein_cc	9606	3:1218352	29
ENST0000(	EBI3-001	Epstein-Barr virus induced 3	protein_cc	9606	19:422949	41
ENST0000(	ECI1-001	enoyl-CoA delta isomerase 1	protein_cc	9606	16:223939	18
ENST0000(	EED-003	embryonic ectoderm development	protein_cc	9606	11:862451	8
ENST0000(	EEF1B2-001	eukaryotic translation elongation factor 1 beta 2	protein_cc	9606	2:2061595	7
ENST0000(	EFCAB1-001	EF-hand calcium binding domain 1	protein_cc	9606	8:4872340	6
ENST0000(	EFS-003	embryonal Fyn-associated substrate	protein_cc	9606	14:233564	9
ENST0000(	EGLN3-001	egl-9 family hypoxia-inducible factor 3	protein_cc	9606	14:339242	32
ENST0000(	EGR1-001	early growth response 1	protein_cc	9606	5:1384654	43
ENST0000(	EGR2-001	early growth response 2	protein_cc	9606	10:628119	18
ENST0000(	EHD3-001	EH-domain containing 3	protein_cc	9606	2:3123433	39
ENST0000(	ELOF1-201	elongation factor 1 homolog (S. cerevisiae)	protein_cc	9606	19:115530	44
ENST0000(	EMC2-001	ER membrane protein complex subunit 2	protein_cc	9606	8:1084436	40
ENST0000(	EMC6-001	ER membrane protein complex subunit 6	protein_cc	9606	17:366881	16
ENST0000(	EN2-001	engrailed homeobox 2	protein_cc	9606	7:1554581	19
ENST0000(	ENGASE-003	endo-beta-N-acetylglucosaminidase	nonsense_	9606	17:790803	31
ENST0000(	ENKD1-001	enkurin domain containing 1	protein_cc	9606	16:676629	43
ENST0000(	ENPEP-001	glutamyl aminopeptidase (aminopeptidase A)	protein_cc	9606	4:1104760	22

ENST0000(	EOMES-001	eomesodermin	protein_cc	9606	3:2771594	5
ENST0000(	EPCAM-001	epithelial cell adhesion molecule	protein_cc	9606	2:4736914	26
ENST0000(	EPO-001	erythropoietin	protein_cc	9606	7:1007208	15
ENST0000(	EPSTI1-001	epithelial stromal interaction 1 (breast)	protein_cc	9606	13:428863	34
ENST0000(	EREG-001	epiregulin	protein_cc	9606	4:7436514	32
ENST0000(	ERICH5-001	glutamate-rich 5	protein_cc	9606	8:9806431	26
ENST0000(	ERICH6-001	glutamate-rich 6	protein_cc	9606	3:1506598	7
ENST0000(	EXOC3L1-001	exocyst complex component 3-like 1	protein_cc	9606	16:671843	38
ENST0000(	EXPH5-001	exophilin 5	protein_cc	9606	11:108505	31
ENST0000(	F2-001	coagulation factor II (thrombin)	protein_cc	9606	11:467191	5
ENST0000(	F2RL3-001	coagulation factor II (thrombin) receptor-like 3	protein_cc	9606	19:168888	35
ENST0000(	F9-001	coagulation factor IX	protein_cc	9606	X:1395307	2
ENST0000(	FABP1-001	fatty acid binding protein 1, liver	protein_cc	9606	2:8812298	2
ENST0000(	FAHD2A-001	fumarylacetoacetate hydrolase domain containing 2A	protein_cc	9606	2:9540272	33
ENST0000(	FAM105A-001	family with sequence similarity 105, member A	protein_cc	9606	5:1458177	40
ENST0000(	FAM170B-001	family with sequence similarity 170, member B	protein_cc	9606	10:491311	2
ENST0000(	FAM177A1-001	family with sequence similarity 177, member A1	protein_cc	9606	14:350464	10
ENST0000(	FAM187B-001	family with sequence similarity 187, member B	protein_cc	9606	19:352248	4
ENST0000(	FAM189A1-001	family with sequence similarity 189, member A1	protein_cc	9606	15:291202	29
ENST0000(	FAM71A-001	family with sequence similarity 71, member A	protein_cc	9606	1:2126244	18
ENST0000(	FAM83B-001	family with sequence similarity 83, member B	protein_cc	9606	6:5484677	5
ENST0000(	FAM83E-001	family with sequence similarity 83, member E	protein_cc	9606	19:486008	34
ENST0000(	FAM84A-001	family with sequence similarity 84, member A	protein_cc	9606	2:1463268	12
ENST0000(	FAM86B3P-005	family with sequence similarity 86, member B3, pseudogene	processed_	9606	8:8240369	28
ENST0000(	FAM89B-001	family with sequence similarity 89, member B	protein_cc	9606	11:655723	4
ENST0000(	FBLN5-002	fibulin 5	protein_cc	9606	14:918694	6
ENST0000(	FBXO25-003	F-box protein 25	protein_cc	9606	8:413072-4	6
ENST0000(	FBXO9-002	F-box protein 9	protein_cc	9606	6:5307085	2
ENST0000(	FCER1G-001	Fc fragment of IgE, high affinity I, receptor for; gamma polypeptide	protein_cc	9606	1:1612152	25
ENST0000(	FCN3-001	ficolin (collagen/fibrinogen domain containing) 3	protein_cc	9606	1:2736911	17
ENST0000(	FGA-002	fibrinogen alpha chain	protein_cc	9606	4:1545831	2
ENST0000(	FGB-001	fibrinogen beta chain	protein_cc	9606	4:1545629	2
ENST0000(	FGF10-001	fibroblast growth factor 10	protein_cc	9606	5:4430354	5
ENST0000(	FGF18-001	fibroblast growth factor 18	protein_cc	9606	5:1714196	32
ENST0000(	FGF19-001	fibroblast growth factor 19	protein_cc	9606	11:696982	11
ENST0000(	FGF20-001	fibroblast growth factor 20	protein_cc	9606	8:1699216	12
ENST0000(	FGF21-201	fibroblast growth factor 21	protein_cc	9606	19:487560	3
ENST0000(	FGF23-001	fibroblast growth factor 23	protein_cc	9606	12:436822	20
ENST0000(	FGFBP3-001	fibroblast growth factor binding protein 3	protein_cc	9606	10:919065	42
ENST0000(	FGFR1OP2-004	FGFR1 oncogene partner 2	protein_cc	9606	12:269385	19
ENST0000(	FGFR4-001	fibroblast growth factor receptor 4	protein_cc	9606	5:1770868	26
ENST0000(	FGL2-001	fibrinogen-like 2	protein_cc	9606	7:7719337	29
ENST0000(	FHDC1-201	FH2 domain containing 1	protein_cc	9606	4:1529429	31
ENST0000(	FIBIN-001	fin bud initiation factor homolog (zebrafish)	protein_cc	9606	11:269941	5
ENST0000(	FIGF-001	c-fos induced growth factor (vascular endothelial growth factor D)	protein_cc	9606	X:1534559	38
ENST0000(	FILIP1-001	filamin A interacting protein 1	protein_cc	9606	6:7530808	11
ENST0000(	FIZ1-001	FLT3-interacting zinc finger 1	protein_cc	9606	19:555913	36
ENST0000(	FKBP2-003	FK506 binding protein 2, 13kDa	protein_cc	9606	11:642410	43
ENST0000(	FKRP-001	fukutin related protein	protein_cc	9606	19:467460	14
ENST0000(	FLJ34503-001	uncharacterized FLJ34503	antisense	9606	6:1139041	25
ENST0000(	FLJ40194-001	uncharacterized FLJ40194	lincRNA	9606	17:492482	30
ENST0000(	FLYWCH2-002	FLYWCH family member 2	protein_cc	9606	16:288321	9
ENST0000(	FMO6P-003	flavin containing monooxygenase 6 pseudogene	protein_cc	9606	1:1711381	2
ENST0000(	FOS-001	FBJ murine osteosarcoma viral oncogene homolog	protein_cc	9606	14:752787	38
ENST0000(	FOXD4L1-001	forkhead box D4-like 1	protein_cc	9606	2:1134986	29
ENST0000(	FOXJ1-001	forkhead box J1	protein_cc	9606	17:761363	9
ENST0000(	FOXQ1-001	forkhead box Q1	protein_cc	9606	6:1312473	8
ENST0000(	FPR1-001	formyl peptide receptor 1	protein_cc	9606	19:517451	18
ENST0000(	FRG2C-001	FSHD region gene 2 family, member C	protein_cc	9606	3:7566433	7
ENST0000(	FRZB-001	frizzled-related protein	protein_cc	9606	2:1828332	27
ENST0000(	FZD3-001	frizzled class receptor 3	protein_cc	9606	8:2849425	41
ENST0000(	GABARAPL1-001	GABA(A) receptor-associated protein like 1	protein_cc	9606	12:102128	37
ENST0000(	GABRB3-002	gamma-aminobutyric acid (GABA) A receptor, beta 3	protein_cc	9606	15:265435	15
ENST0000(	GABRP-201	gamma-aminobutyric acid (GABA) A receptor, pi	protein_cc	9606	5:1707837	12
ENST0000(	GADD45G-001	growth arrest and DNA-damage-inducible, gamma	protein_cc	9606	9:8960501	39
ENST0000(	GAL3ST2-001	galactose-3-O-sulfotransferase 2	protein_cc	9606	2:2417768	11
ENST0000(	GALNT18-001	polypeptide N-acetylgalactosaminyltransferase 18	protein_cc	9606	11:112708	43
ENST0000(	GAMT-001	guanidinoacetate N-methyltransferase	protein_cc	9606	19:139708	44
ENST0000(	GAP43-001	growth associated protein 43	protein_cc	9606	3:1156233	13
ENST0000(	GAS1-001	growth arrest-specific 1	protein_cc	9606	9:8694436	37
ENST0000(	GATA5-001	GATA binding protein 5	protein_cc	9606	20:624634	16
ENST0000(	GCA-003	grancalcin, EF-hand calcium binding protein	protein_cc	9606	2:1623443	24
ENST0000(	GCNT4-001	glucosaminyl (N-acetyl) transferase 4, core 2	protein_cc	9606	5:7502534	27
ENST0000(	GDF7-001	growth differentiation factor 7	protein_cc	9606	2:2066666	32
ENST0000(	GEM-001	GTP binding protein overexpressed in skeletal muscle	protein_cc	9606	8:9424925	24
ENST0000(	GFRA2-002	GDNF family receptor alpha 2	nonsense_	9606	8:2169201	8
ENST0000(	GGT6-001	gamma-glutamyltransferase 6	protein_cc	9606	17:455692	14
ENST0000(	GIGYF1-001	GRB10 interacting GYF protein 1	protein_cc	9606	7:1006795	40
ENST0000(	GIMAP4-001	GTPase, IMAP family member 4	protein_cc	9606	7:1505672	40
ENST0000(	GIPC3-001	GIPC PDZ domain containing family, member 3	protein_cc	9606	19:358555	42
ENST0000(	GJC3-001	gap junction protein, gamma 3, 30.2kDa	protein_cc	9606	7:9992326	37
ENST0000(	GLP2R-001	glucagon-like peptide 2 receptor	protein_cc	9606	17:982555	3
ENST0000(	GLTSCR2-001	glioma tumor suppressor candidate region gene 2	protein_cc	9606	19:477455	43
ENST0000(	GNAO1-001	guanine nucleotide binding protein (G protein), alpha activating activity polypeptide O	protein_cc	9606	16:561913	22
ENST0000(	GNAT1-001	guanine nucleotide binding protein (G protein), alpha transducing activity polypeptide 1	protein_cc	9606	3:5019161	26
ENST0000(	GNG8-001	guanine nucleotide binding protein (G protein), gamma 8	protein_cc	9606	19:466340	11
ENST0000(	GOLT1A-001	golgi transport 1A	protein_cc	9606	1:2041981	21

ENST0000(	GP9-001	glycoprotein IX (platelet)	protein_cc	9606	3:1290607	9
ENST0000(	GPATCH11-001	G patch domain containing 11	protein_cc	9606	2:3708451	3
ENST0000(	GPR137C-001	G protein-coupled receptor 137C	protein_cc	9606	14:525531	40
ENST0000(	GPR182-001	G protein-coupled receptor 182	protein_cc	9606	12:569944	29
ENST0000(	GPR82-001	G protein-coupled receptor 82	protein_cc	9606	X:4172415	32
ENST0000(	GPR83-001	G protein-coupled receptor 83	protein_cc	9606	11:943773	34
ENST0000(	GPR84-001	G protein-coupled receptor 84	protein_cc	9606	12:543624	14
ENST0000(	GPSM1-003	G-protein signaling modulator 1	protein_cc	9606	9:1363555	8
ENST0000(	GRHL1-001	grainyhead-like 1 (Drosophila)	protein_cc	9606	2:9951698	32
ENST0000(	GRHL2-001	grainyhead-like 2 (Drosophila)	protein_cc	9606	8:1014924	7
ENST0000(	GSC-001	goosecoid homeobox	protein_cc	9606	14:947682	8
ENST0000(	GUCA1B-001	guanylate cyclase activator 1B (retina)	protein_cc	9606	6:4218440	38
ENST0000(	GYS2-001	glycogen synthase 2 (liver)	protein_cc	9606	12:215361	17
ENST0000(	GZMM-001	granzyme M (lymphocyte met-ase 1)	protein_cc	9606	19:544035	22
ENST0000(	HAMP-002	hepcidin antimicrobial peptide	protein_cc	9606	19:352823	4
ENST0000(	HAS2-001	hyaluronan synthase 2	protein_cc	9606	8:1216121	37
ENST0000(	HDHD3-001	haloacid dehalogenase-like hydrolase domain containing 3	protein_cc	9606	9:1133734	39
ENST0000(	HERC5-001	HECT and RLD domain containing E3 ubiquitin protein ligase 5	protein_cc	9606	4:8845711	33
ENST0000(	HERC6-201	HECT and RLD domain containing E3 ubiquitin protein ligase family member 6	protein_cc	9606	4:8837873	6
ENST0000(	HES1-001	hes family bHLH transcription factor 1	protein_cc	9606	3:1941361	43
ENST0000(	HES4-001	hes family bHLH transcription factor 4	protein_cc	9606	1:998964-	38
ENST0000(	HGD-001	homogentisate 1,2-dioxygenase	protein_cc	9606	3:1206281	7
ENST0000(	HIGD2A-001	HIG1 hypoxia inducible domain family, member 2A	protein_cc	9606	5:1763887	40
ENST0000(	HIST1H2AC-001	histone cluster 1, H2ac	nonsense_	9606	6:2612414	14
ENST0000(	HIST1H2BC-003	histone cluster 1, H2bc	protein_cc	9606	6:2611487	28
ENST0000(	HK3-001	hexokinase 3 (white cell)	protein_cc	9606	5:1768808	7
ENST0000(	HLA-DOA-001	major histocompatibility complex, class II, DO alpha	protein_cc	9606	6:3300417	23
ENST0000(	HMGCL-010	3-hydroxymethyl-3-methylglutaryl-CoA lyase	protein_cc	9606	1:2380191	3
ENST0000(	HMGN1-004	high mobility group nucleosome binding domain 1	nonsense_	9606	21:393423	2
ENST0000(	HMGN3-002	high mobility group nucleosomal binding domain 3	protein_cc	9606	6:7920124	38
ENST0000(	HOXA5-001	homeobox A5	protein_cc	9606	7:2714105	41
ENST0000(	HPD-002	4-hydroxyphenylpyruvate dioxygenase	protein_cc	9606	12:121839	17
ENST0000(	HPN-002	hepsin	protein_cc	9606	19:350405	19
ENST0000(	HPX-001	hemopexin	protein_cc	9606	11:643104	22
ENST0000(	HRASLS2-001	HRAS-like suppressor 2	protein_cc	9606	11:635527	17
ENST0000(	HRG-001	histidine-rich glycoprotein	protein_cc	9606	3:1866659	2
ENST0000(	HRSP12-001	heat-responsive protein 12	protein_cc	9606	8:9810234	42
ENST0000(	HS1BP3-001	HCLS1 binding protein 3	protein_cc	9606	2:2061782	41
ENST0000(	HS3ST1-001	heparan sulfate (glucosamine) 3-O-sulfotransferase 1	protein_cc	9606	4:1139315	41
ENST0000(	HS3ST2-001	heparan sulfate (glucosamine) 3-O-sulfotransferase 2	protein_cc	9606	16:228141	4
ENST0000(	HS3ST3A1-001	heparan sulfate (glucosamine) 3-O-sulfotransferase 3A1	protein_cc	9606	17:134956	10
ENST0000(	HSPA2-201	heat shock 70kDa protein 2	protein_cc	9606	14:645404	44
ENST0000(	HSPA6-001	heat shock 70kDa protein 6 (HSP70B')	protein_cc	9606	1:1615245	36
ENST0000(	HSPB6-201	heat shock protein, alpha-crystallin-related, B6	protein_cc	9606	19:357545	44
ENST0000(	HYAL1-006	hyaluronoglucosaminidase 1	protein_cc	9606	3:5029989	5
ENST0000(	IDO1-002	indoleamine 2,3-dioxygenase 1	nonsense_	9606	8:3991386	5
ENST0000(	IDUA-001	iduronidase, alpha-L-	protein_cc	9606	4:986997-	37
ENST0000(	IER2-001	immediate early response 2	protein_cc	9606	19:131504	20
ENST0000(	IFNLR1-001	interferon, lambda receptor 1	protein_cc	9606	1:2415415	32
ENST0000(	IFT43-201	intraflagellar transport 43	protein_cc	9606	14:759857	15
ENST0000(	IGLON5-001	IgLON family member 5	protein_cc	9606	19:513118	25
ENST0000(	IGSF10-001	immunoglobulin superfamily, member 10	protein_cc	9606	3:1514334	33
ENST0000(	IGSF21-001	immunoglobin superfamily, member 21	protein_cc	9606	1:1810774	2
ENST0000(	IL13-001	interleukin 13	protein_cc	9606	5:1326581	4
ENST0000(	IL15-002	interleukin 15	protein_cc	9606	4:1416365	24
ENST0000(	IL18-001	interleukin 18	protein_cc	9606	11:112143	22
ENST0000(	IL18RAP-001	interleukin 18 receptor accessory protein	protein_cc	9606	2:1024186	10
ENST0000(	IL22RA1-001	interleukin 22 receptor, alpha 1	protein_cc	9606	1:2411977	13
ENST0000(	ILDR1-001	immunoglobulin-like domain containing receptor 1	protein_cc	9606	3:1219873	2
ENST0000(	IMPG2-001	interphotoreceptor matrix proteoglycan 2	protein_cc	9606	3:1012225	34
ENST0000(	INHA-001	inhibin, alpha	protein_cc	9606	2:2195721	13
ENST0000(	INHBB-001	inhibin, beta B	protein_cc	9606	2:1203461	38
ENST0000(	INHBE-001	inhibin, beta E	protein_cc	9606	12:574553	33
ENST0000(	INMT-001	indolethylamine N-methyltransferase	protein_cc	9606	7:3075213	3
ENST0000(	INO80B-001	INO80 complex subunit B	protein_cc	9606	2:7445502	23
ENST0000(	INSL3-001	insulin-like 3 (Leydig cell)	protein_cc	9606	19:178165	10
ENST0000(	IP6K3-002	inositol hexakisphosphate kinase 3	protein_cc	9606	6:3372167	7
ENST0000(	IRGC-001	immunity-related GTPase family, cinema	protein_cc	9606	19:437160	3
ENST0000(	IRS1-001	insulin receptor substrate 1	protein_cc	9606	2:2267313	37
ENST0000(	ISM1-001	isthmin 1, angiogenesis inhibitor	protein_cc	9606	20:132217	29
ENST0000(	ITGA9-001	integrin, alpha 9	protein_cc	9606	3:3745211	28
ENST0000(	ITM2C-006	integral membrane protein 2C	protein_cc	9606	2:2308649	5
ENST0000(	ITPKA-001	inositol-trisphosphate 3-kinase A	protein_cc	9606	15:414938	36
ENST0000(	JDP2-003	Jun dimerization protein 2	protein_cc	9606	14:754321	41
ENST0000(	JUNB-001	jun B proto-oncogene	protein_cc	9606	19:127914	44
ENST0000(	JUND-001	jun D proto-oncogene	protein_cc	9606	19:182797	44
ENST0000(	KBTBD11-001	kelch repeat and BTB (POZ) domain containing 11	protein_cc	9606	8:1973878	43
ENST0000(	KCNC1-002	potassium voltage-gated channel, Shaw-related subfamily, member 1	protein_cc	9606	11:177348	7
ENST0000(	KCND1-001	potassium voltage-gated channel, Shal-related subfamily, member 1	protein_cc	9606	X:4896138	43
ENST0000(	KCNE4-001	potassium voltage-gated channel, Isk-related family, member 4	protein_cc	9606	2:2230518	20
ENST0000(	KCNG4-001	potassium voltage-gated channel, subfamily G, member 4	protein_cc	9606	16:842186	30
ENST0000(	KCNJ1-002	potassium inwardly-rectifying channel, subfamily J, member 1	protein_cc	9606	11:128839	4
ENST0000(	KCNK13-001	potassium channel, subfamily K, member 13	protein_cc	9606	14:900617	17
ENST0000(	KCNMB4-001	potassium large conductance calcium-activated channel, subfamily M, beta member 4	protein_cc	9606	12:703662	40
ENST0000(	KDM6B-001	lysine (K)-specific demethylase 6B	protein_cc	9606	17:783990	27
ENST0000(	KHDRBS2-001	KH domain containing, RNA binding, signal transduction associated 2	protein_cc	9606	6:6167996	4
ENST0000(	KIAA1045-001	KIAA1045	protein_cc	9606	9:3495832	19

ENST0000(	KIAA1109-009	KIAA1109	protein_cc	9606	4:1223244	35
ENST0000(	KIF26A-001	kinesin family member 26A	protein_cc	9606	14:104139	35
ENST0000(	KIFC2-001	kinesin family member C2	protein_cc	9606	8:1444660	34
ENST0000(	KLF15-001	Kruppel-like factor 15	protein_cc	9606	3:1263426	36
ENST0000(	KLF2-001	Kruppel-like factor 2	protein_cc	9606	19:163248	41
ENST0000(	KLHDC3-201	kelch domain containing 3	protein_cc	9606	6:4301410	2
ENST0000(	KLRK1-001	killer cell lectin-like receptor subfamily K, member 1	protein_cc	9606	12:103723	8
ENST0000(	KMT2E-201	lysine (K)-specific methyltransferase 2E	protein_cc	9606	7:1050141	21
ENST0000(	KNDC1-006	kinase non-catalytic C-lobe domain (KIND) containing 1	protein_cc	9606	10:133160	17
ENST0000(	KNG1-001	kininogen 1	protein_cc	9606	3:1867172	4
ENST0000(	KNG1-002	kininogen 1	protein_cc	9606	3:1867173	12
ENST0000(	KRT5-001	keratin 5	protein_cc	9606	12:525145	2
ENST0000(	KRT73-001	keratin 73	protein_cc	9606	12:526075	2
ENST0000(	KRT8-002	keratin 8	protein_cc	9606	12:528971	3
ENST0000(	LAMTOR3-003	late endosomal/lysosomal adaptor, MAPK and MTOR activator 3	protein_cc	9606	4:9988120	36
ENST0000(	LBP-001	lipopolysaccharide binding protein	protein_cc	9606	20:383463	17
ENST0000(	LCN15-002	lipocalin 15	protein_cc	9606	9:1367596	19
ENST0000(	LCN2-001	lipocalin 2	protein_cc	9606	9:1281494	2
ENST0000(	LDHA-003	lactate dehydrogenase A	protein_cc	9606	11:183945	3
ENST0000(	LENG1-001	leukocyte receptor cluster (LRC) member 1	protein_cc	9606	19:541551	35
ENST0000(	LEPRE1-002	leucine proline-enriched proteoglycan (leprecan) 1	protein_cc	9606	1:4274637	5
ENST0000(	LGALS4-001	lectin, galactoside-binding, soluble, 4	protein_cc	9606	19:388016	15
ENST0000(	LGI4-001	leucine-rich repeat LGI family, member 4	protein_cc	9606	19:351245	11
ENST0000(	LIF-001	leukemia inhibitory factor	protein_cc	9606	22:302404	30
ENST0000(	LIN7B-001	lin-7 homolog B (C. elegans)	protein_cc	9606	19:491143	29
ENST0000(	LINC00324-001	long intergenic non-protein coding RNA 324	lincRNA	9606	17:822064	39
ENST0000(	LINC00471-001	long intergenic non-protein coding RNA 471	lincRNA	9606	2:2315084	38
ENST0000(	LINC00671-001	long intergenic non-protein coding RNA 671	lincRNA	9606	17:428746	15
ENST0000(	LIPH-001	lipase, member H	protein_cc	9606	3:1855062	25
ENST0000(	LMO7DN-001	LMO7 downstream neighbor	protein_cc	9606	13:758710	7
ENST0000(	LOH12CR1-002	loss of heterozygosity, 12, chromosomal region 1	protein_cc	9606	12:123574	7
ENST0000(	LPA-001	lipoprotein, Lp(a)	protein_cc	9606	6:1605314	2
ENST0000(	LPPR2-001	Lipid phosphate phosphatase-related protein type 2	protein_cc	9606	19:113553	43
ENST0000(	LRFN1-001	leucine rich repeat and fibronectin type III domain containing 1	protein_cc	9606	19:393065	28
ENST0000(	LRG1-001	leucine-rich alpha-2-glycoprotein 1	protein_cc	9606	19:453640	39
ENST0000(	LRGUK-001	leucine-rich repeats and guanylate kinase domain containing	protein_cc	9606	7:1341272	29
ENST0000(	LRP2-001	low density lipoprotein receptor-related protein 2	protein_cc	9606	2:1691271	12
ENST0000(	LRRC31-002	leucine rich repeat containing 31	protein_cc	9606	3:1698392	3
ENST0000(	LRRC32-001	leucine rich repeat containing 32	protein_cc	9606	11:766575	44
ENST0000(	LRRC56-001	leucine rich repeat containing 56	protein_cc	9606	11:537527	38
ENST0000(	LRRC75B-001	leucine rich repeat containing 75B	protein_cc	9606	22:245856	32
ENST0000(	LSM8-001	LSM8 homolog, U6 small nuclear RNA associated (S. cerevisiae)	protein_cc	9606	7:1181840	41
ENST0000(	LSP1-004	lymphocyte-specific protein 1	protein_cc	9606	11:185297	10
ENST0000(	LTBP4-008	latent transforming growth factor beta binding protein 4	protein_cc	9606	19:406097	2
ENST0000(	LTC4S-001	leukotriene C4 synthase	protein_cc	9606	5:1797939	2
ENST0000(	LYPD6B-201	LY6/PLAUR domain containing 6B	protein_cc	9606	2:1492045	8
ENST0000(	LYVE1-001	lymphatic vessel endothelial hyaluronan receptor 1	protein_cc	9606	11:105569	42
ENST0000(	LYZ-001	lysozyme	protein_cc	9606	12:693483	24
ENST0000(	MACROD1-001	MACRO domain containing 1	protein_cc	9606	11:639985	38
ENST0000(	MADCAM1-002	mucosal vascular addressin cell adhesion molecule 1	protein_cc	9606	19:496494	3
ENST0000(	MAP3K12-004	mitogen-activated protein kinase kinase kinase 12	protein_cc	9606	12:534796	5
ENST0000(	MAPK3-001	mitogen-activated protein kinase 3	protein_cc	9606	16:301141	27
ENST0000(	MAPK8IP2-002	mitogen-activated protein kinase 8 interacting protein 2	protein_cc	9606	22:506031	24
ENST0000(	MAPRE3-001	microtubule-associated protein, RP/EB family, member 3	protein_cc	9606	2:2697061	41
ENST0000(	MARCH9-001	membrane-associated ring finger (C3HC4) 9	protein_cc	9606	12:577550	43
ENST0000(	MAST3-001	microtubule associated serine/threonine kinase 3	protein_cc	9606	19:180977	36
ENST0000(	MATK-003	megakaryocyte-associated tyrosine kinase	protein_cc	9606	19:377797	7
ENST0000(	MATN2-201	matrilin 2	protein_cc	9606	8:9786908	14
ENST0000(	MCF2L-034	MCF.2 cell line derived transforming sequence-like	protein_cc	9606	13:113087	9
ENST0000(	MCHR2-201	melanin-concentrating hormone receptor 2	protein_cc	9606	6:9991991	6
ENST0000(	MED26-001	mediator complex subunit 26	protein_cc	9606	19:165749	2
ENST0000(	MED29-001	mediator complex subunit 29	protein_cc	9606	19:393913	11
ENST0000(	MEMO1-001	mediator of cell motility 1	protein_cc	9606	2:3186506	6
ENST0000(	MESP1-001	mesoderm posterior basic helix-loop-helix transcription factor 1	protein_cc	9606	15:897486	42
ENST0000(	METRNL-001	meteorin, glial cell differentiation regulator-like	protein_cc	9606	17:830796	44
ENST0000(	MGMT-001	O-6-methylguanine-DNA methyltransferase	protein_cc	9606	10:129467	41
ENST0000(	MGST2-001	microsomal glutathione S-transferase 2	protein_cc	9606	4:1396657	35
ENST0000(	MIDN-201	midnolin	protein_cc	9606	19:124855	42
ENST0000(	MIIP-001	migration and invasion inhibitory protein	protein_cc	9606	1:1201946	43
ENST0000(	MIPOL1-001	mirror-image polydactyly 1	protein_cc	9606	14:371979	9
ENST0000(	MISP-001	mitotic spindle positioning	protein_cc	9606	19:751126	18
ENST0000(	MMP15-001	matrix metallopeptidase 15 (membrane-inserted)	protein_cc	9606	16:580255	44
ENST0000(	MNS1-001	meiosis-specific nuclear structural 1	protein_cc	9606	15:564287	35
ENST0000(	MOB3B-001	MOB kinase activator 3B	protein_cc	9606	9:2732520	36
ENST0000(	MOB3C-001	MOB kinase activator 3C	protein_cc	9606	1:4660771	2
ENST0000(	MPV17L-001	MPV17 mitochondrial membrane protein-like	protein_cc	9606	16:153957	5
ENST0000(	MROH1-201	maestro heat-like repeat family member 1	protein_cc	9606	8:1441637	14
ENST0000(	MRPL10-003	mitochondrial ribosomal protein L10	protein_cc	9606	17:478234	5
ENST0000(	MRPL34-001	mitochondrial ribosomal protein L34	protein_cc	9606	19:173056	30
ENST0000(	MRPL43-007	mitochondrial ribosomal protein L43	protein_cc	9606	10:100981	6
ENST0000(	MRPS25-001	mitochondrial ribosomal protein S25	protein_cc	9606	3:1504851	3
ENST0000(	MS4A10-001	membrane-spanning 4-domains, subfamily A, member 10	protein_cc	9606	11:607853	21
ENST0000(	MSC-001	musculin	protein_cc	9606	8:7184154	20
ENST0000(	MSS51-201	MSS51 mitochondrial translational activator	protein_cc	9606	10:734235	20
ENST0000(	MSTN-001	myostatin	protein_cc	9606	2:1900556	23
ENST0000(	MXRA5-001	matrix-remodelling associated 5	protein_cc	9606	X:3308565	11
ENST0000(	MYB-201	v-myb avian myeloblastosis viral oncogene homolog	protein_cc	9606	6:1351813	12

ENST0000(	MYBPC3-201	myosin binding protein C, cardiac	protein_cc	9606	11:473314	4
ENST0000(	MYH14-201	myosin, heavy chain 14, non-muscle	protein_cc	9606	19:502683	16
ENST0000(	MYO1G-001	myosin IG	protein_cc	9606	7:4496266	2
ENST0000(	NAALAD2-002	N-acetylated alpha-linked acidic dipeptidase 2	protein_cc	9606	11:901347	9
ENST0000(	NAB2-001	NGFI-A binding protein 2 (EGR1 binding protein 2)	protein_cc	9606	12:570888	7
ENST0000(	NANOG-001	Nanog homeobox	protein_cc	9606	12:778939	31
ENST0000(	NAT2-001	N-acetyltransferase 2 (arylamine N-acetyltransferase)	protein_cc	9606	8:1839124	20
ENST0000(	NCALD-003	neurocalcin delta	protein_cc	9606	8:1016867	19
ENST0000(	NCAM2-201	neural cell adhesion molecule 2	protein_cc	9606	21:212805	39
ENST0000(	NCAN-001	neurocan	protein_cc	9606	19:192119	11
ENST0000(	NDRG2-004	NDRG family member 2	protein_cc	9606	14:210168	4
ENST0000(	NDUFA10-001	NADH dehydrogenase (ubiquinone) 1 alpha subcomplex, 10, 42kDa	protein_cc	9606	2:2399573	6
ENST0000(	NDUFA2-001	NADH dehydrogenase (ubiquinone) 1 alpha subcomplex, 2, 8kDa	protein_cc	9606	5:1406452	29
ENST0000(	NEURL3-002	neuralized E3 ubiquitin protein ligase 3	protein_cc	9606	2:9649764	16
ENST0000(	NFKBIA-001	nuclear factor of kappa light polypeptide gene enhancer in B-cells inhibitor, alpha	protein_cc	9606	14:354015	42
ENST0000(	NGFR-001	nerve growth factor receptor	protein_cc	9606	17:494952	22
ENST0000(	NHLH1-001	nescient helix loop helix 1	protein_cc	9606	1:1603670	40
ENST0000(	NHP2-002	NHP2 ribonucleoprotein	protein_cc	9606	5:1781494	4
ENST0000(	NINL-007	ninein-like	protein_cc	9606	20:254527	39
ENST0000(	NKD2-002	naked cuticle homolog 2 (Drosophila)	protein_cc	9606	5:1008962	20
ENST0000(	NKG7-001	natural killer cell granule protein 7	protein_cc	9606	19:513716	3
ENST0000(	NLRP12-001	NLR family, pyrin domain containing 12	protein_cc	9606	19:537936	31
ENST0000(	NLRP6-001	NLR family, pyrin domain containing 6	protein_cc	9606	11:278570	7
ENST0000(	NMUR1-001	neuromedin U receptor 1	protein_cc	9606	2:2315231	26
ENST0000(	NOV-001	nephroblastoma overexpressed	protein_cc	9606	8:1194163	41
ENST0000(	NOX5-010	NADPH oxidase, EF-hand calcium binding domain 5	protein_cc	9606	15:689305	11
ENST0000(	NPAS4-001	neuronal PAS domain protein 4	protein_cc	9606	11:664210	2
ENST0000(	NPFF-001	neuropeptide FF-amide peptide precursor	protein_cc	9606	12:535066	3
ENST0000(	NPL-201	N-acetylneuraminate pyruvate lyase (dihydrodipicolinate synthase)	protein_cc	9606	1:1827943	5
ENST0000(	NPR3-001	natriuretic peptide receptor 3	protein_cc	9606	5:3271143	11
ENST0000(	NPY1R-001	neuropeptide Y receptor Y1	protein_cc	9606	4:1633239	19
ENST0000(	NR0B2-001	nuclear receptor subfamily 0, group B, member 2	protein_cc	9606	1:2691148	9
ENST0000(	NRXN2-003	neurexin 2	protein_cc	9606	11:646061	2
ENST0000(	NRXN3-005	neurexin 3	protein_cc	9606	14:792794	8
ENST0000(	NSUN7-002	NOP2/Sun domain family, member 7	protein_cc	9606	4:4074992	24
ENST0000(	NTN1-001	netrin 1	protein_cc	9606	17:902154	36
ENST0000(	NTN5-001	netrin 5	protein_cc	9606	19:486614	8
ENST0000(	NTS-001	neurotensin	protein_cc	9606	12:858742	10
ENST0000(	NUPR1-001	nuclear protein, transcriptional regulator, 1	protein_cc	9606	16:285327	40
ENST0000(	NYAP1-001	neuronal tyrosine-phosphorylated phosphoinositide-3-kinase adaptor 1	protein_cc	9606	7:1004839	40
ENST0000(	OCM-001	oncomodulin	protein_cc	9606	7:5880798	3
ENST0000(	ODF2L-002	outer dense fiber of sperm tails 2-like	protein_cc	9606	1:8635009	18
ENST0000(	OLFM1-001	olfactomedin 1	protein_cc	9606	9:1350755	16
ENST0000(	ONECUT1-001	one cut homeobox 1	protein_cc	9606	15:527569	9
ENST0000(	OR1N1-001	olfactory receptor, family 1, subfamily N, member 1	protein_cc	9606	9:1225263	6
ENST0000(	OR52N2-001	olfactory receptor, family 52, subfamily N, member 2	protein_cc	9606	11:582031	2
ENST0000(	OR52N4-001	olfactory receptor, family 52, subfamily N, member 4 (gene/pseudogene)	protein_cc	9606	11:575469	8
ENST0000(	OR6F1-001	olfactory receptor, family 6, subfamily F, member 1	protein_cc	9606	1:2477118	2
ENST0000(	ORM1-001	orosomucoid 1	protein_cc	9606	9:1143230	3
ENST0000(	OSM-001	oncostatin M	protein_cc	9606	22:302628	4
ENST0000(	OSR1-001	odd-skipped related transciption factor 1	protein_cc	9606	2:1935148	20
ENST0000(	OTC-001	ornithine carbamoyltransferase	protein_cc	9606	X:3835254	10
ENST0000(	OTUB1-002	OTU deubiquitinase, ubiquitin aldehyde binding 1	retained_i	9606	11:639858	4
ENST0000(	OTUD7A-001	OTU deubiquitinase 7A	protein_cc	9606	15:314753	26
ENST0000(	P2RY13-001	purinergic receptor P2Y, G-protein coupled, 13	protein_cc	9606	3:1513263	5
ENST0000(	PAG1-001	phosphoprotein membrane anchor with glycosphingolipid microdomains 1	protein_cc	9606	8:8096781	37
ENST0000(	PAH-002	phenylalanine hydroxylase	protein_cc	9606	12:102838	3
ENST0000(	PAIP2B-001	poly(A) binding protein interacting protein 2B	protein_cc	9606	2:7118273	44
ENST0000(	PCDHB9-001	protocadherin beta 9	protein_cc	9606	5:1411871	28
ENST0000(	PCK1-001	phosphoenolpyruvate carboxykinase 1 (soluble)	protein_cc	9606	20:575610	7
ENST0000(	PCM1-201	pericentriolar material 1	protein_cc	9606	8:1792285	12
ENST0000(	PCSK7-001	proprotein convertase subtilisin/kexin type 7	protein_cc	9606	11:117204	14
ENST0000(	PCSK9-001	proprotein convertase subtilisin/kexin type 9	protein_cc	9606	1:5503954	24
ENST0000(	PDK1-001	pyruvate dehydrogenase kinase, isozyme 1	protein_cc	9606	2:1725559	8
ENST0000(	PF4-001	platelet factor 4	protein_cc	9606	4:7398107	15
ENST0000(	PGR-001	progesterone receptor	protein_cc	9606	11:101029	10
ENST0000(	PHYKPL-001	5-phosphohydroxy-L-lysine phospho-lyase	protein_cc	9606	5:1782084	5
ENST0000(	PIEZO1-201	piezo-type mechanosensitive ion channel component 1	protein_cc	9606	16:887154	2
ENST0000(	PIP5K1B-001	phosphatidylinositol-4-phosphate 5-kinase, type I, beta	protein_cc	9606	9:6870570	17
ENST0000(	PIWIL1-001	piwi-like RNA-mediated gene silencing 1	protein_cc	9606	12:130337	18
ENST0000(	PKDCC-001	protein kinase domain containing, cytoplasmic	protein_cc	9606	2:4204802	24
ENST0000(	PLEK-001	pleckstrin	protein_cc	9606	2:6836517	27
ENST0000(	PLEKHD1-002	pleckstrin homology domain containing, family D (with coiled-coil domains) member 1	protein_cc	9606	14:694847	34
ENST0000(	PLEKHG4B-001	pleckstrin homology domain containing, family G (with RhoGef domain) member 4B	protein_cc	9606	5:140258-:	22
ENST0000(	PLEKHG6-001	pleckstrin homology domain containing, family G (with RhoGef domain) member 6	protein_cc	9606	12:631043	14
ENST0000(	PLG-001	plasminogen	protein_cc	9606	6:1607022	2
ENST0000(	PLIN4-001	perilipin 4	protein_cc	9606	19:450219	4
ENST0000(	PNKD-003	paroxysmal nonkinesigenic dyskinesia	protein_cc	9606	2:2182703	42
ENST0000(	PNMT-001	phenylethanolamine N-methyltransferase	protein_cc	9606	17:396681	10
ENST0000(	PNN-001	pinin, desmosome associated protein	protein_cc	9606	14:391752	44
ENST0000(	PNPLA7-007	patatin-like phospholipase domain containing 7	protein_cc	9606	9:1374599	11
ENST0000(	POLR1D-003	polymerase (RNA) I polypeptide D, 16kDa	protein_cc	9606	13:276207	3
ENST0000(	PON1-001	paraoxonase 1	protein_cc	9606	7:9529767	13
ENST0000(	PPP1R14A-001	protein phosphatase 1, regulatory (inhibitor) subunit 14A	protein_cc	9606	19:382512	41
ENST0000(	PPP1R1A-001	protein phosphatase 1, regulatory (inhibitor) subunit 1A	protein_cc	9606	12:545792	8
ENST0000(	PPP1R3F-001	protein phosphatase 1, regulatory subunit 3F	protein_cc	9606	X:4926985	36
ENST0000(	PPP2R5B-001	protein phosphatase 2, regulatory subunit B', beta	protein_cc	9606	11:649246	34

ENST0000(	PPP4R4-001	protein phosphatase 4, regulatory subunit 4	protein_cc	9606	14:941743	11
ENST0000(	PRDM10-004	PR domain containing 10	protein_cc	9606	11:129899	6
ENST0000(	PRKD2-001	protein kinase D2	protein_cc	9606	19:466743	24
ENST0000(	PROCA1-001	protein interacting with cyclin A1	protein_cc	9606	17:287031	15
ENST0000(	PROM2-001	prominin 2	protein_cc	9606	2:9527445	4
ENST0000(	PRORSD1P-001	prolyl-tRNA synthetase associated domain containing 1, pseudogene	unitary_ps	9606	2:5528232	8
ENST0000(	PRR18-001	proline rich 18	protein_cc	9606	6:1663053	14
ENST0000(	PRRG4-001	proline rich Gla (G-carboxyglutamic acid) 4 (transmembrane)	protein_cc	9606	11:328299	41
ENST0000(	PRSS27-001	protease, serine 27	protein_cc	9606	16:271241	14
ENST0000(	PRSS8-001	protease, serine, 8	protein_cc	9606	16:311314	12
ENST0000(	PRTN3-001	proteinase 3	protein_cc	9606	19:840960	7
ENST0000(	PSD2-001	pleckstrin and Sec7 domain containing 2	protein_cc	9606	5:1397958	17
ENST0000(	PTCD1-001	pentatricopeptide repeat domain 1	protein_cc	9606	7:9941673	9
ENST0000(	PTGDR-001	prostaglandin D2 receptor (DP)	protein_cc	9606	14:522677	5
ENST0000(	PTGER2-001	prostaglandin E receptor 2 (subtype EP2), 53kDa	protein_cc	9606	14:523143	12
ENST0000(	PTPRR-001	protein tyrosine phosphatase, receptor type, R	protein_cc	9606	12:706380	2
ENST0000(	PXN-001	paxillin	protein_cc	9606	12:120210	4
ENST0000(	PYCARD-001	PYD and CARD domain containing	protein_cc	9606	16:312014	33
ENST0000(	RAB20-001	RAB20, member RAS oncogene family	protein_cc	9606	13:110523	44
ENST0000(	RAB24-001	RAB24, member RAS oncogene family	protein_cc	9606	5:1773014	8
ENST0000(	RAB39A-001	RAB39A, member RAS oncogene family	protein_cc	9606	11:107928	23
ENST0000(	RAB3C-001	RAB3C, member RAS oncogene family	protein_cc	9606	5:5858304	36
ENST0000(	RAB40AL-001	RAB40A, member RAS oncogene family-like	protein_cc	9606	X:1029372	15
ENST0000(	RAMP1-001	receptor (G protein-coupled) activity modifying protein 1	protein_cc	9606	2:2378595	13
ENST0000(	RAMP2-001	receptor (G protein-coupled) activity modifying protein 2	protein_cc	9606	17:427611	40
ENST0000(	RAMP3-001	receptor (G protein-coupled) activity modifying protein 3	protein_cc	9606	7:4515779	13
ENST0000(	RAPGEFL1-007	Rap guanine nucleotide exchange factor (GEF)-like 1	protein_cc	9606	17:401770	20
ENST0000(	RAPSN-001	receptor-associated protein of the synapse	protein_cc	9606	11:474377	12
ENST0000(	RARRES2-001	retinoic acid receptor responder (tazarotene induced) 2	protein_cc	9606	7:1503383	9
ENST0000(	RARRES3-001	retinoic acid receptor responder (tazarotene induced) 3	protein_cc	9606	11:635368	34
ENST0000(	RASA4-001	RAS p21 protein activator 4	protein_cc	9606	7:1025796	21
ENST0000(	RASD1-001	RAS, dexamethasone-induced 1	protein_cc	9606	17:174944	44
ENST0000(	RASGRP1-001	RAS guanyl releasing protein 1 (calcium and DAG-regulated)	protein_cc	9606	15:384881	21
ENST0000(	RASL10A-001	RAS-like, family 10, member A	protein_cc	9606	22:293129	32
ENST0000(	RBM46-003	RNA binding motif protein 46	protein_cc	9606	4:1547812	2
ENST0000(	RBM48-001	RNA binding motif protein 48	protein_cc	9606	7:9252877	39
ENST0000(	RBMY2FP-001	RNA binding motif protein, Y-linked, family 2, member F pseudogene	processed_	9606	Y:2230885	2
ENST0000(	RBP1-001	retinol binding protein 1, cellular	protein_cc	9606	3:1395174	30
ENST0000(	RBP5-001	retinol binding protein 5, cellular	protein_cc	9606	12:712368	32
ENST0000(	RBP7-001	retinol binding protein 7, cellular	protein_cc	9606	1:9997216	11
ENST0000(	RBP7-002	retinol binding protein 7, cellular	nonsense_	9606	1:9997206	17
ENST0000(	REEP6-001	receptor accessory protein 6	protein_cc	9606	19:149074	41
ENST0000(	RELB-001	v-rel avian reticuloendotheliosis viral oncogene homolog B	protein_cc	9606	19:450014	39
ENST0000(	RELL1-001	RELT-like 1	protein_cc	9606	4:3759080	7
ENST0000(	REM1-001	RAS (RAD and GEM)-like GTP-binding 1	protein_cc	9606	20:314752	39
ENST0000(	REM2-001	RAS (RAD and GEM)-like GTP binding 2	protein_cc	9606	14:228831	30
ENST0000(	RGS2-001	regulator of G-protein signaling 2	protein_cc	9606	1:1928090	42
ENST0000(	RHBDL1-001	rhomboid, veinlet-like 1 (Drosophila)	protein_cc	9606	16:676075	21
ENST0000(	RHOB-001	ras homolog family member B	protein_cc	9606	2:2044707	44
ENST0000(	RHOXF1-001	Rhox homeobox family, member 1	protein_cc	9606	X:1201090	3
ENST0000(	RHPN1-001	rhophilin, Rho GTPase binding protein 1	protein_cc	9606	8:1433688	28
ENST0000(	RIOK1-002	RIO kinase 1	retained_i	9606	6:7396752	3
ENST0000(	RNASE2-001	ribonuclease, RNase A family, 2 (liver, eosinophil-derived neurotoxin)	protein_cc	9606	14:209554	10
ENST0000(	RNASE6-002	ribonuclease, RNase A family, k6	protein_cc	9606	14:207810	39
ENST0000(	RNASE7-001	ribonuclease, RNase A family, 7	protein_cc	9606	14:210422	4
ENST0000(	RNF113B-001	ring finger protein 113B	protein_cc	9606	13:981757	15
ENST0000(	RNF114-001	ring finger protein 114	protein_cc	9606	20:499363	31
ENST0000(	RNF125-001	ring finger protein 125, E3 ubiquitin protein ligase	protein_cc	9606	18:320183	14
ENST0000(	RNF128-001	ring finger protein 128, E3 ubiquitin protein ligase	protein_cc	9606	X:1067266	13
ENST0000(	RNF144A-001	ring finger protein 144A	protein_cc	9606	2:6917392	37
ENST0000(	RNF157-010	ring finger protein 157	protein_cc	9606	17:761443	17
ENST0000(	RNF185-002	ring finger protein 185	protein_cc	9606	22:311602	3
ENST0000(	RNFT2-001	ring finger protein, transmembrane 2	protein_cc	9606	12:116738	37
ENST0000(	ROPN1L-001	rhophilin associated tail protein 1-like	protein_cc	9606	5:1044187	39
ENST0000(	RP11-15G8.1-001		processed_	9606	6:1547066	17
ENST0000(	RP11-16K12.1-001		antisense	9606	15:789788	24
ENST0000(	RP11-683L23.1-201	Tubulin beta-8 chain-like protein LOC260334	protein_cc	9606	18:47390-4	11
ENST0000(	RP4-763G1.2-001		lincRNA	9606	1:6752963	17
ENST0000(	RPL23P2-001	ribosomal protein L23 pseudogene 2	processed_	9606	21:289976	27
ENST0000(	RPS20-002	ribosomal protein S20	protein_cc	9606	8:5607294	43
ENST0000(	RPS27A-001	ribosomal protein S27a	protein_cc	9606	2:5523240	20
ENST0000(	RRAS-001	related RAS viral (r-ras) oncogene homolog	protein_cc	9606	19:496352	44
ENST0000(	RSPH4A-002	radial spoke head 4 homolog A (Chlamydomonas)	protein_cc	9606	6:1166164	11
ENST0000(	RSPO4-001	R-spondin 4	protein_cc	9606	20:958452	6
ENST0000(	RTKN2-003	rhotekin 2	protein_cc	9606	10:621830	5
ENST0000(	RTP3-001	receptor (chemosensory) transporter protein 3	protein_cc	9606	3:4649749	9
ENST0000(	RTP4-001	receptor (chemosensory) transporter protein 4	protein_cc	9606	3:1873683	31
ENST0000(	S100B-001	S100 calcium binding protein B	protein_cc	9606	21:465989	12
ENST0000(	S100P-001	S100 calcium binding protein P	protein_cc	9606	4:6693069	29
ENST0000(	S100Z-002	S100 calcium binding protein Z	protein_cc	9606	5:7685009	2
ENST0000(	SAA2-002	serum amyloid A2	protein_cc	9606	11:182452	2
ENST0000(	SAA4-001	serum amyloid A4, constitutive	protein_cc	9606	11:182313	4
ENST0000(	SALL1-001	spalt-like transcription factor 1	protein_cc	9606	16:511359	6
ENST0000(	SALL4-001	spalt-like transcription factor 4	protein_cc	9606	20:517840	20
ENST0000(	SAMD14-003	sterile alpha motif domain containing 14	protein_cc	9606	17:501113	3
ENST0000(	SAT2-002	spermidine/spermine N1-acetyltransferase family member 2	protein_cc	9606	17:762623	38
ENST0000(	SBSPON-001	somatomedin B and thrombospondin, type 1 domain containing	protein_cc	9606	8:7306454	22

ENST0000(	SCAND1-002	SCAN domain containing 1	protein_cc	9606	20:359536	43
ENST0000(	SCG2-001	secretogranin II	protein_cc	9606	2:2235969	25
ENST0000(	SCGB3A1-001	secretoglobin, family 3A, member 1	protein_cc	9606	5:1805901	27
ENST0000(	SCN3A-001	sodium channel, voltage-gated, type III, alpha subunit	protein_cc	9606	2:1650875	5
ENST0000(	SCNN1D-003	sodium channel, non-voltage-gated 1, delta subunit	protein_cc	9606	1:1282109	10
ENST0000(	SCT-001	secretin	protein_cc	9606	11:626431	8
ENST0000(	SCTR-001	secretin receptor	protein_cc	9606	2:1194398	6
ENST0000(	SDR9C7-001	short chain dehydrogenase/reductase family 9C, member 7	protein_cc	9606	12:569231	6
ENST0000(	SDS-001	serine dehydratase	protein_cc	9606	12:113392	15
ENST0000(	SEC16A-004	SEC16 homolog A (S. cerevisiae)	protein_cc	9606	9:1364401	8
ENST0000(	SEMA4C-001	sema domain, immunoglobulin domain (Ig), transmembrane domain (TM) and short cytoplasmic domain, (semaphorin) 4C	protein_cc	9606	2:9685971	39
ENST0000(	SEMA5B-201	sema domain, seven thrombospondin repeats (type 1 and type 1-like), transmembrane domain (TM) and short cytoplasmic domain, (sem	protein_cc	9606	3:1229091	5
ENST0000(	SEPW1P-001	selenoprotein W, 1 pseudogene	processed_	9606	1:3109498	22
ENST0000(	SERPINA10-001	serpin peptidase inhibitor, clade A (alpha-1 antiproteinase, antitrypsin), member 10	protein_cc	9606	14:942804	31
ENST0000(	SERPINF2-201	serpin peptidase inhibitor, clade F (alpha-2 antiplasmin, pigment epithelium derived factor), member 2	protein_cc	9606	17:174283	5
ENST0000(	SFRP5-001	secreted frizzled-related protein 5	protein_cc	9606	10:977667	21
ENST0000(	SH3GL3-001	SH3-domain GRB2-like 3	protein_cc	9606	15:834475	6
ENST0000(	SHANK2-AS3-001	SHANK2 antisense RNA 3	antisense	9606	11:708627	22
ENST0000(	SHC2-001	SHC (Src homology 2 domain containing) transforming protein 2	protein_cc	9606	19:416583	35
ENST0000(	SHOC2-201	soc-2 suppressor of clear homolog (C. elegans)	protein_cc	9606	10:110964	2
ENST0000(	SHROOM3-001	shroom family member 3	protein_cc	9606	4:7643510	6
ENST0000(	SIDT1-001	SID1 transmembrane family, member 1	protein_cc	9606	3:1135322	29
ENST0000(	SIGLEC12-001	sialic acid binding Ig-like lectin 12 (gene/pseudogene)	protein_cc	9606	19:514913	4
ENST0000(	SIGLEC9-001	sialic acid binding Ig-like lectin 9	protein_cc	9606	19:511249	8
ENST0000(	SIK1-001	salt-inducible kinase 1	protein_cc	9606	21:434145	36
ENST0000(	SIRPB1-201	signal-regulatory protein beta 1	protein_cc	9606	20:156438	7
ENST0000(	SLAMF1-001	signaling lymphocytic activation molecule family member 1	protein_cc	9606	1:1606081	3
ENST0000(	SLAMF8-001	SLAM family member 8	protein_cc	9606	1:1598267	3
ENST0000(	SLC12A5-002	solute carrier family 12 (potassium/chloride transporter), member 5	protein_cc	9606	20:460292	12
ENST0000(	SLC16A11-001	solute carrier family 16, member 11	protein_cc	9606	17:704163	11
ENST0000(	SLC22A17-003	solute carrier family 22, member 17	protein_cc	9606	14:233463	9
ENST0000(	SLC23A3-001	solute carrier family 23, member 3	protein_cc	9606	2:2191614	2
ENST0000(	SLC25A4-001	solute carrier family 25 (mitochondrial carrier; adenine nucleotide translocator), member 4	protein_cc	9606	4:1851432	32
ENST0000(	SLC27A1-001	solute carrier family 27 (fatty acid transporter), member 1	protein_cc	9606	19:174704	35
ENST0000(	SLC2A4-001	solute carrier family 2 (facilitated glucose transporter), member 4	protein_cc	9606	17:728166	13
ENST0000(	SLC35G3-001	solute carrier family 35, member G3	protein_cc	9606	17:351925	2
ENST0000(	SLC5A4-001	solute carrier family 5 (glucose activated ion channel), member 4	protein_cc	9606	22:322184	38
ENST0000(	SLC5A5-001	solute carrier family 5 (sodium/iodide cotransporter), member 5	protein_cc	9606	19:178719	29
ENST0000(	SLC6A19-001	solute carrier family 6 (neutral amino acid transporter), member 19	protein_cc	9606	5:1201595	2
ENST0000(	SLC7A7-007	solute carrier family 7 (amino acid transporter light chain, y+L system), member 7	protein_cc	9606	14:227732	13
ENST0000(	SLC8B1-201	solute carrier family 8 (sodium/lithium/calcium exchanger), member B1	protein_cc	9606	12:113298	10
ENST0000(	SMIM24-001	small integral membrane protein 24	protein_cc	9606	19:347398	11
ENST0000(	SNAI2-201	snail family zinc finger 2	protein_cc	9606	8:4891769	42
ENST0000(	SNRPF-001	small nuclear ribonucleoprotein polypeptide F	protein_cc	9606	12:958589	41
ENST0000(	SNX31-001	sorting nexin 31	protein_cc	9606	8:1005728	2
ENST0000(	SOAT2-001	sterol O-acyltransferase 2	protein_cc	9606	12:531035	3
ENST0000(	SORBS1-001	sorbin and SH3 domain containing 1	protein_cc	9606	10:953117	34
ENST0000(	SOSTDC1-001	sclerostin domain containing 1	protein_cc	9606	7:1646148	16
ENST0000(	SOX2-001	SRY (sex determining region Y)-box 2	protein_cc	9606	3:1817119	34
ENST0000(	SOX8-001	SRY (sex determining region Y)-box 8	protein_cc	9606	16:981808	39
ENST0000(	SOX9-001	SRY (sex determining region Y)-box 9	protein_cc	9606	17:721210	12
ENST0000(	SPATC1L-002	spermatogenesis and centriole associated 1-like	protein_cc	9606	21:461611	4
ENST0000(	SPI1-004	Spi-1 proto-oncogene	protein_cc	9606	11:473549	8
ENST0000(	SPINK1-001	serine peptidase inhibitor, Kazal type 1	protein_cc	9606	5:1478245	4
ENST0000(	SPINT2-001	serine peptidase inhibitor, Kunitz type, 2	protein_cc	9606	19:382644	31
ENST0000(	SPON2-001	spondin 2, extracellular matrix protein	protein_cc	9606	4:1166932	5
ENST0000(	SPP1-002	secreted phosphoprotein 1	protein_cc	9606	4:8797566	2
ENST0000(	SPSB3-007	splA/ryanodine receptor domain and SOCS box containing 3	protein_cc	9606	16:177674	31
ENST0000(	SPTBN5-001	spectrin, beta, non-erythrocytic 5	protein_cc	9606	15:418481	14
ENST0000(	SPX-001	spexin hormone	protein_cc	9606	12:215263	13
ENST0000(	SRP19-002	signal recognition particle 19kDa	protein_cc	9606	5:1128613	2
ENST0000(	SSTR1-002	somatostatin receptor 1	protein_cc	9606	14:382079	38
ENST0000(	ST8SIA3-001	ST8 alpha-N-acetyl-neuraminide alpha-2,8-sialyltransferase 3	protein_cc	9606	18:573508	9
ENST0000(	STAP1-001	signal transducing adaptor family member 1	protein_cc	9606	4:6755872	2
ENST0000(	STARD5-001	StAR-related lipid transfer (START) domain containing 5	protein_cc	9606	15:813090	19
ENST0000(	STOX1-002	storkhead box 1	protein_cc	9606	10:688275	11
ENST0000(	STPG1-001	sperm-tail PG-rich repeat containing 1	protein_cc	9606	1:2435700	3
ENST0000(	STRA8-001	stimulated by retinoic acid 8	protein_cc	9606	7:1352319	2
ENST0000(	STX19-001	syntaxin 19	protein_cc	9606	3:9401436	15
ENST0000(	STXBP6-006	syntaxin binding protein 6 (amisyn)	protein_cc	9606	14:248096	6
ENST0000(	SULT2A1-001	sulfotransferase family, cytosolic, 2A, dehydroepiandrosterone (DHEA)-preferring, member 1	protein_cc	9606	19:478704	22
ENST0000(	SUPT3H-201	suppressor of Ty 3 homolog (S. cerevisiae)	protein_cc	9606	6:4482673	12
ENST0000(	SYF2-001	SYF2 pre-mRNA-splicing factor	protein_cc	9606	1:2522267	42
ENST0000(	SYT7-001	synaptotagmin VII	protein_cc	9606	11:615153	37
ENST0000(	SYTL1-001	synaptotagmin-like 1	protein_cc	9606	1:2734202	5
ENST0000(	TACR1-001	tachykinin receptor 1	protein_cc	9606	2:7504646	12
ENST0000(	TAF1D-002	TATA box binding protein (TBP)-associated factor, RNA polymerase I, D, 41kDa	nonsense_	9606	11:937299	5
ENST0000(	TAS2R4-001	taste receptor, type 2, member 4	protein_cc	9606	7:1417784	39
ENST0000(	TBC1D10A-001	TBC1 domain family, member 10A	protein_cc	9606	22:302919	37
ENST0000(	TBC1D13-001	TBC1 domain family, member 13	protein_cc	9606	9:1287873	3
ENST0000(	TBC1D27-001	TBC1 domain family, member 27	processed_	9606	17:169229	2
ENST0000(	TBCA-002	tubulin folding cofactor A	protein_cc	9606	5:7769124	4
ENST0000(	TBX21-001	T-box 21	protein_cc	9606	17:477332	11
ENST0000(	TBX3-002	T-box 3	protein_cc	9606	12:114670	22
ENST0000(	TBX6-201	T-box 6	protein_cc	9606	16:300857	27
ENST0000(	TCF23-001	transcription factor 23	protein_cc	9606	2:2714900	35
ENST0000(	TCN2-001	transcobalamin II	protein_cc	9606	22:306068	14

ENST0000(	TDRD6-001	tudor domain containing 6	protein_cc	9606	6:4668812	19
ENST0000(	TEKT2-001	tektin 2 (testicular)	protein_cc	9606	1:3608407	23
ENST0000(	TENM4-001	teneurin transmembrane protein 4	protein_cc	9606	11:786528	2
ENST0000(	TEX29-002	testis expressed 29	protein_cc	9606	13:111320	15
ENST0000(	TFF3-201	trefoil factor 3 (intestinal)	protein_cc	9606	21:423120	9
ENST0000(	TGM1-001	transglutaminase 1	protein_cc	9606	14:242491	32
ENST0000(	THOP1-001	thimet oligopeptidase 1	protein_cc	9606	19:278546	5
ENST0000(	TIMP1-001	TIMP metallopeptidase inhibitor 1	protein_cc	9606	X:4758231	43
ENST0000(	TIMP3-001	TIMP metallopeptidase inhibitor 3	protein_cc	9606	22:328017	41
ENST0000(	TIMP4-001	TIMP metallopeptidase inhibitor 4	protein_cc	9606	3:1215305	26
ENST0000(	TLR2-001	toll-like receptor 2	protein_cc	9606	4:1537015	40
ENST0000(	TLR8-002	toll-like receptor 8	protein_cc	9606	X:1290662	3
ENST0000(	TM4SF18-001	transmembrane 4 L six family member 18	protein_cc	9606	3:1493184	35
ENST0000(	TM4SF5-001	transmembrane 4 L six family member 5	protein_cc	9606	17:477189	6
ENST0000(	TMEM132E-001	transmembrane protein 132E	protein_cc	9606	17:345807	11
ENST0000(	TMEM133-001	transmembrane protein 133	protein_cc	9606	11:100991	27
ENST0000(	TMEM145-001	transmembrane protein 145	protein_cc	9606	19:423133	14
ENST0000(	TMEM164-001	transmembrane protein 164	protein_cc	9606	X:1100031	2
ENST0000(	TMEM176A-001	transmembrane protein 176A	protein_cc	9606	7:1508005	2
ENST0000(	TMEM176B-004	transmembrane protein 176B	protein_cc	9606	7:1507912	6
ENST0000(	TMEM178A-001	transmembrane protein 178A	protein_cc	9606	2:3966591	8
ENST0000(	TMEM256-001	transmembrane protein 256	protein_cc	9606	17:740297	40
ENST0000(	TMEM38A-001	transmembrane protein 38A	protein_cc	9606	19:166611	42
ENST0000(	TMEM60-001	transmembrane protein 60	protein_cc	9606	7:7779372	42
ENST0000(	TMEM86A-001	transmembrane protein 86A	protein_cc	9606	11:186987	42
ENST0000(	TMEM88-001	transmembrane protein 88	protein_cc	9606	17:785506	43
ENST0000(	TMSB4XP4-001	thymosin beta 4, X-linked pseudogene 4	processed_	9606	9:1283421	43
ENST0000(	TMSB4Y-001	thymosin beta 4, Y-linked	protein_cc	9606	Y:1370356	25
ENST0000(	TMUB1-001	transmembrane and ubiquitin-like domain containing 1	protein_cc	9606	7:1510810	38
ENST0000(	TNFAIP3-001	tumor necrosis factor, alpha-induced protein 3	protein_cc	9606	6:1378674	33
ENST0000(	TNFAIP8L1-001	tumor necrosis factor, alpha-induced protein 8-like 1	protein_cc	9606	19:463951	43
ENST0000(	TNFRSF11A-002	tumor necrosis factor receptor superfamily, member 11a, NFKB activator	protein_cc	9606	18:623252	11
ENST0000(	TNFRSF17-001	tumor necrosis factor receptor superfamily, member 17	protein_cc	9606	16:119651	4
ENST0000(	TNIP3-001	TNFAIP3 interacting protein 3	protein_cc	9606	4:1211314	27
ENST0000(	TNNI2-201	troponin I type 2 (skeletal, fast)	protein_cc	9606	11:183969	5
ENST0000(	TP53I13-001	tumor protein p53 inducible protein 13	protein_cc	9606	17:295686	43
ENST0000(	TPH1-001	tryptophan hydroxylase 1	protein_cc	9606	11:180175	5
ENST0000(	TPRXL-001	tetra-peptide repeat homeobox-like	protein_cc	9606	3:1393740	5
ENST0000(	TRAF4-001	TNF receptor-associated factor 4	protein_cc	9606	17:287439	34
ENST0000(	TRAPPC2L-001	trafficking protein particle complex 2-like	protein_cc	9606	16:888570	15
ENST0000(	TRAPPC6A-002	trafficking protein particle complex 6A	protein_cc	9606	19:451629	27
ENST0000(	TRIB2-001	tribbles pseudokinase 2	protein_cc	9606	2:1271688	42
ENST0000(	TRIM45-001	tripartite motif containing 45	protein_cc	9606	1:1171110	34
ENST0000(	TRIM74-002	tripartite motif containing 74	protein_cc	9606	7:7295948	7
ENST0000(	TRIM9-001	tripartite motif containing 9	protein_cc	9606	14:509752	11
ENST0000(	TRIML2-003	tripartite motif family-like 2	protein_cc	9606	4:1880913	4
ENST0000(	TRIP10-003	thyroid hormone receptor interactor 10	protein_cc	9606	19:673972	6
ENST0000(	TRMT61A-003	tRNA methyltransferase 61 homolog A (S. cerevisiae)	protein_cc	9606	14:103529	3
ENST0000(	TRPA1-001	transient receptor potential cation channel, subfamily A, member 1	protein_cc	9606	8:7202125	2
ENST0000(	TSKS-001	testis-specific serine kinase substrate	protein_cc	9606	19:497397	9
ENST0000(	TSPYL5-001	TSPY-like 5	protein_cc	9606	8:9727347	43
ENST0000(	TTC9-001	tetratricopeptide repeat domain 9	protein_cc	9606	14:706417	39
ENST0000(	TTR-001	transthyretin	protein_cc	9606	18:315917	3
ENST0000(	TTTY14-001	testis-specific transcript, Y-linked 14 (non-protein coding)	lincRNA	9606	Y:1887250	22
ENST0000(	TXLNGY-012	taxilin gamma pseudogene, Y-linked	processed_	9606	Y:1959245	22
ENST0000(	UACA-001	uveal autoantigen with coiled-coil domains and ankyrin repeats	protein_cc	9606	15:706545	33
ENST0000(	UBA5-201	ubiquitin-like modifier activating enzyme 5	protein_cc	9606	3:1326544	2
ENST0000(	UBASH3A-001	ubiquitin associated and SH3 domain containing A	protein_cc	9606	21:424039	4
ENST0000(	UBE2N-001	ubiquitin-conjugating enzyme E2N	protein_cc	9606	12:934056	9
ENST0000(	UBE3D-004	ubiquitin protein ligase E3D	nonsense_	9606	6:8289239	3
ENST0000(	UBQLN3-001	ubiquilin 3	protein_cc	9606	11:550730	7
ENST0000(	UCN-001	urocortin	protein_cc	9606	2:2730740	35
ENST0000(	UCP3-001	uncoupling protein 3 (mitochondrial, proton carrier)	protein_cc	9606	11:740002	31
ENST0000(	UGT2B4-001	UDP glucuronosyltransferase 2 family, polypeptide B4	protein_cc	9606	4:6948016	2
ENST0000(	UGT2B7-001	UDP glucuronosyltransferase 2 family, polypeptide B7	protein_cc	9606	4:6909647	3
ENST0000(	UNC93B1-001	unc-93 homolog B1 (C. elegans)	protein_cc	9606	11:679911	43
ENST0000(	UNC93B2-001	unc-93 homolog B2 pseudogene (C. elegans)	processed_	9606	7:6855485	6
ENST0000(	UPK1A-001	uroplakin 1A	protein_cc	9606	19:356668	9
ENST0000(	UPP2-001	uridine phosphorylase 2	protein_cc	9606	2:1581018	3
ENST0000(	UROC1-001	urocanate hydratase 1	protein_cc	9606	3:1264812	6
ENST0000(	USP47-005	ubiquitin specific peptidase 47	processed_	9606	11:119504	15
ENST0000(	UTF1-001	undifferentiated embryonic cell transcription factor 1	protein_cc	9606	10:133230	6
ENST0000(	UTS2R-001	urotensin 2 receptor	protein_cc	9606	17:823742	7
ENST0000(	VAMP8-001	vesicle-associated membrane protein 8	protein_cc	9606	2:8557749	44
ENST0000(	VENTX-001	VENT homeobox	protein_cc	9606	10:133237	23
ENST0000(	VEZT-201	vezatin, adherens junctions transmembrane protein	protein_cc	9606	12:952663	3
ENST0000(	VN1R1-001	vomeronasal 1 receptor 1	protein_cc	9606	19:574547	5
ENST0000(	VPREB1-002	pre-B lymphocyte 1	protein_cc	9606	22:222447	2
ENST0000(	VPS28-201	vacuolar protein sorting 28 homolog (S. cerevisiae)	protein_cc	9606	8:1444236	23
ENST0000(	VSIG2-001	V-set and immunoglobulin domain containing 2	protein_cc	9606	11:124747	32
ENST0000(	VSTM4-002	V-set and transmembrane domain containing 4	protein_cc	9606	10:491021	16
ENST0000(	WNT11-001	wingless-type MMTV integration site family, member 11	protein_cc	9606	11:761863	21
ENST0000(	WNT2-002	wingless-type MMTV integration site family member 2	protein_cc	9606	7:1172766	7
ENST0000(	WNT4-001	wingless-type MMTV integration site family, member 4	protein_cc	9606	1:2211730	34
ENST0000(	WSB1-001	WD repeat and SOCS box containing 1	protein_cc	9606	17:272940	30
ENST0000(	WWC1-001	WW and C2 domain containing 1	protein_cc	9606	5:1682916	30
ENST0000(	XKR4-001	XK, Kell blood group complex subunit-related family, member 4	protein_cc	9606	8:5510238	42

ENST0000(	XYLT1-001	xylosyltransferase I	protein_cc	9606	16:171017	34
ENST0000(	YPEL5-001	yippee-like 5 (Drosophila)	protein_cc	9606	2:3014696	41
ENST0000(	ZAP70-001	zeta-chain (TCR) associated protein kinase 70kDa	protein_cc	9606	2:9771356	6
ENST0000(	ZBTB46-001	zinc finger and BTB domain containing 46	protein_cc	9606	20:637436	38
ENST0000(	ZCCHC6-003	zinc finger, CCHC domain containing 6	protein_cc	9606	9:8628773	33
ENST0000(	ZDHHC8P1-001	zinc finger, DHHC-type containing 8 pseudogene 1	processed_	9606	22:233906	4
ENST0000(	ZFPM1-001	zinc finger protein, FOG family member 1	protein_cc	9606	16:884533	42
ENST0000(	ZFYVE19-008	zinc finger, FYVE domain containing 19	protein_cc	9606	15:408075	13
ENST0000(	ZNF10-001	zinc finger protein 10	protein_cc	9606	12:133130	18
ENST0000(	ZNF276-001	zinc finger protein 276	protein_cc	9606	16:897209	10
ENST0000(	ZNF286B-003	zinc finger protein 286B	protein_cc	9606	17:186584	32
ENST0000(	ZNF350-001	zinc finger protein 350	protein_cc	9606	19:519643	42
ENST0000(	ZNF396-001	zinc finger protein 396	protein_cc	9606	18:353666	28
ENST0000(	ZNF404-201	zinc finger protein 404	protein_cc	9606	19:438723	31
ENST0000(	ZNF414-002	zinc finger protein 414	protein_cc	9606	19:851133	37
ENST0000(	ZNF423-201	zinc finger protein 423	protein_cc	9606	16:494906	15
ENST0000(	ZNF439-002	zinc finger protein 439	protein_cc	9606	19:118659	22
ENST0000(	ZNF497-002	zinc finger protein 497	protein_cc	9606	19:583543	36
ENST0000(	ZNF524-001	zinc finger protein 524	protein_cc	9606	19:556003	38
ENST0000(	ZNF544-201	zinc finger protein 544	protein_cc	9606	19:582439	5
ENST0000(	ZNF556-001	zinc finger protein 556	protein_cc	9606	19:286733	38
ENST0000(	ZNF579-001	zinc finger protein 579	protein_cc	9606	19:555767	38
ENST0000(	ZNF580-001	zinc finger protein 580	protein_cc	9606	19:556408	15
ENST0000(	ZNF688-001	zinc finger protein 688	protein_cc	9606	16:305693	32
ENST0000(	ZNF692-001	zinc finger protein 692	protein_cc	9606	1:2488500	26
ENST0000(	ZNF700-001	zinc finger protein 700	protein_cc	9606	19:119250	38
ENST0000(	ZNF771-001	zinc finger protein 771	protein_cc	9606	16:304072	5
ENST0000(	ZNF80-001	zinc finger protein 80	nonsense_	9606	3:1142346	2
ENST0000(	ZNF816-ZNF321P-001	ZNF816-ZNF321P readthrough	processed_	9606	19:529271	14
ENST0000(	ZNF862-001	zinc finger protein 862	protein_cc	9606	7:1498383	31
ENST0000(	ZNHIT2-001	zinc finger, HIT-type containing 2	protein_cc	9606	11:651164	44
ENST0000(	ZRSR2-001	zinc finger (CCCH type), RNA-binding motif and serine/arginine rich 2	protein_cc	9606	X:1579047	39

Table S8

Heart WCGNA Gene Color

	gene	hcolor	Name	Species
1	11715189_	turquoise	microRNA 4738(MIR4738)	Homo sapiens
2	11715193_	turquoise	UNC homeobox(UNCX)	Homo sapiens
3	11715207_	turquoise	WDFY family member 4(WDFY4)	Homo sapiens
4	11715239_	turquoise	interferon induced transmembrane protein 3(IFITM3)	Homo sapiens
5	11715291_	turquoise	T-box 5(TBX5)	Homo sapiens
6	11715320_	blue	titin(TTN)	Homo sapiens
7	11715339_	blue	titin(TTN)	Homo sapiens
8	11715346_	turquoise	Epstein-Barr virus induced 3(EBI3)	Homo sapiens
9	11715349_	brown	cytochrome c oxidase subunit 7C(COX7C)	Homo sapiens
10	11715359_	turquoise	TIMP metallopeptidase inhibitor 1(TIMP1)	Homo sapiens
11	11715360_	turquoise	TIMP metallopeptidase inhibitor 1(TIMP1)	Homo sapiens
12	11715363_	turquoise	alpha-2-macroglobulin(A2M)	Homo sapiens
13	11715367_	turquoise	septin 2(SEPT2)	Homo sapiens
14	11715369_	brown	NDUFA4, mitochondrial complex associated(NDUFA4)	Homo sapiens
15	11715370_	turquoise	galectin 3 binding protein(LGALS3BP)	Homo sapiens
16	11715377_	brown	cytochrome c oxidase subunit 4I1(COX4I1)	Homo sapiens
17	11715388_	turquoise	cyclin dependent kinase inhibitor 1A(CDKN1A)	Homo sapiens
18	11715390_	turquoise	FK506 binding protein 1A(FKBP1A)	Homo sapiens
19	11715401_	turquoise	interleukin enhancer binding factor 2(ILF2)	Homo sapiens
20	11715409_	blue	peroxiredoxin 2(PRDX2)	Homo sapiens
21	11715410_	blue	peroxiredoxin 2(PRDX2)	Homo sapiens
22	11715419_	turquoise	lymphocyte antigen 6 complex, locus E(LY6E)	Homo sapiens
23	11715439_	turquoise	death associated protein(DAP)	Homo sapiens
24	11715451_	turquoise	collagen type IV alpha 2 chain(COL4A2)	Homo sapiens
25	11715452_	turquoise	collagen type IV alpha 2 chain(COL4A2)	Homo sapiens
26	11715453_	turquoise	collagen type IV alpha 2 chain(COL4A2)	Homo sapiens
27	11715458_	brown	cytochrome c oxidase subunit 8A(COX8A)	Homo sapiens
28	11715459_	brown	cytochrome c oxidase subunit 8A(COX8A)	Homo sapiens
29	11715461_	turquoise	lysozyme(LYZ)	Homo sapiens
30	11715462_	turquoise	lysozyme(LYZ)	Homo sapiens
31	11715466_	turquoise	Rho GDP dissociation inhibitor beta(ARHGDIB)	Homo sapiens
32	11715467_	turquoise	Rho GDP dissociation inhibitor beta(ARHGDIB)	Homo sapiens
33	11715476_	turquoise	DEAD-box helicase 17(DDX17)	Homo sapiens
34	11715482_	turquoise	BCL2 family apoptosis regulator(MCL1)	Homo sapiens
35	11715483_	turquoise	BCL2 family apoptosis regulator(MCL1)	Homo sapiens
36	11715487_	turquoise	BCL2 family apoptosis regulator(MCL1)	Homo sapiens
37	11715497_	brown	OXA1L, mitochondrial inner membrane protein(OXA1L)	Homo sapiens
38	11715498_	blue	OXA1L, mitochondrial inner membrane protein(OXA1L)	Homo sapiens
39	11715503_	turquoise	LIM and SH3 protein 1(LASP1)	Homo sapiens
40	11715524_	turquoise	coactosin like F-actin binding protein 1(COTL1)	Homo sapiens
41	11715532_	turquoise	interferon gamma receptor 2 (interferon gamma transducer 1)(IFNGR2)	Homo sapiens
42	11715541_	turquoise	Thy-1 cell surface antigen(THY1)	Homo sapiens
43	11715542_	turquoise	Thy-1 cell surface antigen(THY1)	Homo sapiens
44	11715583_	turquoise	major histocompatibility complex, class II, DP alpha 1(HLA-DPA1)	Homo sapiens
45	11715593_	turquoise	tyrosine 3-monooxygenase/tryptophan 5-monooxygenase activation protein eta(YWHA	Homo sapiens
46	11715606_	turquoise	TSC22 domain family member 1(TSC22D1)	Homo sapiens
47	11715608_	turquoise	peptidylprolyl isomerase B(PPIB)	Homo sapiens
48	11715616_	brown	NADH:ubiquinone oxidoreductase subunit AB1(NDUFAB1)	Homo sapiens

49	11715620_ blue	crystallin alpha B(CRYAB)	Homo sapiens
50	11715623_ turquoise	ring finger protein 114(RNF114)	Homo sapiens
51	11715624_ turquoise	SH3 domain binding glutamate rich protein like(SH3BGRL)	Homo sapiens
52	11715625_ turquoise	SH3 domain binding glutamate rich protein like(SH3BGRL)	Homo sapiens
53	11715638_ turquoise	lysophosphatidylcholine acyltransferase 1(LPCAT1)	Homo sapiens
54	11715644_ turquoise	ATPase H+ transporting accessory protein 2(ATP6AP2)	Homo sapiens
55	11715655_ blue	voltage dependent anion channel 1(VDAC1)	Homo sapiens
56	11715656_ blue	cytochrome c oxidase subunit 5A(COX5A)	Homo sapiens
57	11715665_ turquoise	proteasome subunit beta 8(PSMB8)	Homo sapiens
58	11715667_ turquoise	tropomyosin 3(TPM3)	Homo sapiens
59	11715670_ turquoise	interferon induced transmembrane protein 1(IFITM1)	Homo sapiens
60	11715671_ turquoise	interferon induced transmembrane protein 1(IFITM1)	Homo sapiens
61	11715684_ turquoise	pyrophosphatase (inorganic) 1(PPA1)	Homo sapiens
62	11715685_ turquoise	actin related protein 2/3 complex subunit 5(ARPC5)	Homo sapiens
63	11715686_ turquoise	actin related protein 2/3 complex subunit 5(ARPC5)	Homo sapiens
64	11715687_ turquoise	actin related protein 2/3 complex subunit 5(ARPC5)	Homo sapiens
65	11715691_ turquoise	ZFP36 ring finger protein(ZFP36)	Homo sapiens
66	11715694_ brown	cytochrome c oxidase subunit 7B(COX7B)	Homo sapiens
67	11715695_ brown	cytochrome c oxidase subunit 7B(COX7B)	Homo sapiens
68	11715710_ turquoise	cathepsin C(CTSC)	Homo sapiens
69	11715716_ turquoise	moesin(MSN)	Homo sapiens
70	11715717_ turquoise	moesin(MSN)	Homo sapiens
71	11715722_ turquoise	golgi membrane protein 1(GOLM1)	Homo sapiens
72	11715742_ turquoise	regulator of chromosome condensation 2(RCC2)	Homo sapiens
73	11715743_ turquoise	regulator of chromosome condensation 2(RCC2)	Homo sapiens
74	11715744_ brown	ATP synthase, H+ transporting, mitochondrial Fo complex subunit F6(ATP5J)	Homo sapiens
75	11715767_ turquoise	acetyl-CoA acyltransferase 2(ACAA2)	Homo sapiens
76	11715770_ turquoise	SET nuclear proto-oncogene(SET)	Homo sapiens
77	11715772_ brown	mitochondrial ribosomal protein L13(MRPL13)	Homo sapiens
78	11715788_ turquoise	solute carrier family 40 member 1(SLC40A1)	Homo sapiens
79	11715817_ turquoise	ZFP36 ring finger protein like 2(ZFP36L2)	Homo sapiens
80	11715818_ turquoise	ZFP36 ring finger protein like 2(ZFP36L2)	Homo sapiens
81	11715827_ turquoise	polyhomeotic homolog 1(PHC1)	Homo sapiens
82	11715836_ turquoise	proteasome subunit alpha 3(PSMA3)	Homo sapiens
83	11715839_ turquoise	succinate dehydrogenase complex subunit C(SDHC)	Homo sapiens
84	11715855_ turquoise	prolylcarboxypeptidase(PRCP)	Homo sapiens
85	11715856_ turquoise	prolylcarboxypeptidase(PRCP)	Homo sapiens
86	11715867_ brown	mitochondrial ribosomal protein L41(MRPL41)	Homo sapiens
87	11715868_ turquoise	TERF1 interacting nuclear factor 2(TINF2)	Homo sapiens
88	11715869_ turquoise	TERF1 interacting nuclear factor 2(TINF2)	Homo sapiens
89	11715874_ brown	ATP synthase, H+ transporting, mitochondrial Fo complex subunit D(ATP5H)	Homo sapiens
90	11715881_ blue	death associated protein 3(DAP3)	Homo sapiens
91	11715883_ blue	death associated protein 3(DAP3)	Homo sapiens
92	11715884_ turquoise	transmembrane protein 248(TMEM248)	Homo sapiens
93	11715893_ turquoise	interferon alpha inducible protein 27(IFI27)	Homo sapiens
94	11715897_ turquoise	scavenger receptor class B member 2(SCARB2)	Homo sapiens
95	11715899_ brown	succinate-CoA ligase alpha subunit(SUCLG1)	Homo sapiens
96	11715908_ turquoise	myosin VI(MYO6)	Homo sapiens
97	11715915_ turquoise	CD44 molecule (Indian blood group)(CD44)	Homo sapiens
98	11715916_ turquoise	CD44 molecule (Indian blood group)(CD44)	Homo sapiens

99	11715917_	turquoise	CD44 molecule (Indian blood group)(CD44)	Homo sapiens
100	11715919_	turquoise	SH3 domain binding glutamate rich protein like 3(SH3BGRL3)	Homo sapiens
101	11715921_	turquoise	septin 9(SEPT9)	Homo sapiens
102	11715922_	turquoise	septin 9(SEPT9)	Homo sapiens
103	11715925_	turquoise	translocation associated membrane protein 1(TRAM1)	Homo sapiens
104	11715931_	turquoise	serum/glucocorticoid regulated kinase 1(SGK1)	Homo sapiens
105	11715935_	turquoise	neurobeachin like 2(NBEAL2)	Homo sapiens
106	11715936_	turquoise	neurobeachin like 2(NBEAL2)	Homo sapiens
107	11715948_	turquoise	potassium channel tetramerization domain containing 12(KCTD12)	Homo sapiens
108	11715949_	turquoise	potassium channel tetramerization domain containing 12(KCTD12)	Homo sapiens
109	11715966_	turquoise	abhydrolase domain containing 17A(ABHD17A)	Homo sapiens
110	11715973_	blue	mitochondrial fission regulator 1 like(MTFR1L)	Homo sapiens
111	11715988_	brown	ATP synthase, H+ transporting, mitochondrial Fo complex subunit C1 (subunit 9)(ATP5G	Homo sapiens
112	11715994_	turquoise	inhibitor of DNA binding 2, HLH protein(ID2)	Homo sapiens
113	11715995_	brown	mitochondrial ribosomal protein L16(MRPL16)	Homo sapiens
114	11715997_	brown	NADH:ubiquinone oxidoreductase subunit C1(NDUFC1)	Homo sapiens
115	11716012_	turquoise	TNF alpha induced protein 1(TNFAIP1)	Homo sapiens
116	11716015_	turquoise	CKLF like MARVEL transmembrane domain containing 3(CMTM3)	Homo sapiens
117	11716018_	turquoise	RAB31, member RAS oncogene family(RAB31)	Homo sapiens
118	11716019_	turquoise	RAB31, member RAS oncogene family(RAB31)	Homo sapiens
119	11716029_	brown	NADH:ubiquinone oxidoreductase subunit B9(NDUFB9)	Homo sapiens
120	11716030_	brown	NADH:ubiquinone oxidoreductase subunit B9(NDUFB9)	Homo sapiens
121	11716031_	turquoise	ADP ribosylation factor 3(ARF3)	Homo sapiens
122	11716034_	turquoise	bone marrow stromal cell antigen 2(BST2)	Homo sapiens
123	11716035_	turquoise	bone marrow stromal cell antigen 2(BST2)	Homo sapiens
124	11716036_	turquoise	bone marrow stromal cell antigen 2(BST2)	Homo sapiens
125	11716047_	brown	chromosome 21 open reading frame 33(C21orf33)	Homo sapiens
126	11716055_	turquoise	stathmin 3(STMN3)	Homo sapiens
127	11716061_	brown	family with sequence similarity 162 member A(FAM162A)	Homo sapiens
128	11716062_	turquoise	tenascin C(TNC)	Homo sapiens
129	11716063_	turquoise	tenascin C(TNC)	Homo sapiens
130	11716071_	turquoise	Pim-3 proto-oncogene, serine/threonine kinase(PIM3)	Homo sapiens
131	11716072_	turquoise	ATPase H+ transporting V1 subunit B2(ATP6V1B2)	Homo sapiens
132	11716093_	turquoise	Kruppel like factor 6(KLF6)	Homo sapiens
133	11716095_	turquoise	Kruppel like factor 6(KLF6)	Homo sapiens
134	11716103_	turquoise	proliferating cell nuclear antigen(PCNA)	Homo sapiens
135	11716115_	turquoise	ZFP36 ring finger protein like 1(ZFP36L1)	Homo sapiens
136	11716116_	brown	NADH:ubiquinone oxidoreductase subunit S6(NDUFS6)	Homo sapiens
137	11716118_	turquoise	protein disulfide isomerase family A member 4(PDIA4)	Homo sapiens
138	11716119_	turquoise	protein disulfide isomerase family A member 4(PDIA4)	Homo sapiens
139	11716121_	turquoise	apoptosis inhibitor 5(API5)	Homo sapiens
140	11716135_	turquoise	major vault protein(MVP)	Homo sapiens
141	11716167_	turquoise	MX dynamin like GTPase 1(MX1)	Homo sapiens
142	11716171_	turquoise	lamin B2(LMNB2)	Homo sapiens
143	11716181_	turquoise	mediator complex subunit 15(MED15)	Homo sapiens
144	11716186_	turquoise	activated leukocyte cell adhesion molecule(ALCAM)	Homo sapiens
145	11716202_	blue	phosphofructokinase, muscle(PFKM)	Homo sapiens
146	11716205_	blue	NADH:ubiquinone oxidoreductase subunit A8(NDUFA8)	Homo sapiens
147	11716211_	blue	3-hydroxyisobutyrate dehydrogenase(HIBADH)	Homo sapiens
148	11716212_	blue	3-hydroxyisobutyrate dehydrogenase(HIBADH)	Homo sapiens

149	11716226_ turquoise	LIM domain and actin binding 1(LIMA1)	Homo sapiens
150	11716243_ brown	protein phosphatase 1 regulatory subunit 16A(PPP1R16A)	Homo sapiens
151	11716245_ turquoise	WAS protein family member 2(WASF2)	Homo sapiens
152	11716249_ turquoise	transmembrane p24 trafficking protein 9(TMED9)	Homo sapiens
153	11716267_ turquoise	adenylate kinase 1(AK1)	Homo sapiens
154	11716268_ turquoise	adenylate kinase 1(AK1)	Homo sapiens
155	11716274_ turquoise	endosulfine alpha(ENSA)	Homo sapiens
156	11716275_ turquoise	chromobox 5(CBX5)	Homo sapiens
157	11716283_ turquoise	poly(A) RNA polymerase D7, non-canonical(PAPD7)	Homo sapiens
158	11716284_ turquoise	glyoxalase I(GLO1)	Homo sapiens
159	11716288_ turquoise	extended synaptotagmin 1(ESYT1)	Homo sapiens
160	11716294_ turquoise	cysteinyl-tRNA synthetase(CARS)	Homo sapiens
161	11716307_ turquoise	ubiquinol-cytochrome c reductase, complex III subunit XI(UQCR11)	Homo sapiens
162	11716315_ brown	mitochondrial ribosomal protein L15(MRPL15)	Homo sapiens
163	11716337_ turquoise	insulin induced gene 1(INSIG1)	Homo sapiens
164	11716339_ turquoise	insulin induced gene 1(INSIG1)	Homo sapiens
165	11716340_ brown	NADH:ubiquinone oxidoreductase subunit S4(NDUFS4)	Homo sapiens
166	11716350_ brown	HSPE1-MOB4 readthrough(HSPE1-MOB4)	Homo sapiens
167	11716355_ brown	NADH:ubiquinone oxidoreductase subunit A11(NDUFA11)	Homo sapiens
168	11716358_ turquoise	protein regulator of cytokinesis 1(PRC1)	Homo sapiens
169	11716367_ turquoise	proline rich 13(PRR13)	Homo sapiens
170	11716368_ turquoise	proline rich 13(PRR13)	Homo sapiens
171	11716377_ turquoise	granulin precursor(GRN)	Homo sapiens
172	11716380_ blue	mitochondrial pyruvate carrier 2(MPC2)	Homo sapiens
173	11716381_ blue	mitochondrial pyruvate carrier 2(MPC2)	Homo sapiens
174	11716384_ turquoise	C-C motif chemokine ligand 2(CCL2)	Homo sapiens
175	11716385_ turquoise	transforming growth factor beta 1(TGFB1)	Homo sapiens
176	11716392_ blue	alcohol dehydrogenase 5 (class III), chi polypeptide(ADH5)	Homo sapiens
177	11716395_ turquoise	adhesion G protein-coupled receptor G1(ADGRG1)	Homo sapiens
178	11716403_ turquoise	eukaryotic translation initiation factor 4E binding protein 2(EIF4EBP2)	Homo sapiens
179	11716415_ brown	NADH:ubiquinone oxidoreductase subunit B8(NDUFB8)	Homo sapiens
180	11716416_ turquoise	complement C1q A chain(C1QA)	Homo sapiens
181	11716432_ brown	MNAT1, CDK activating kinase assembly factor(MNAT1)	Homo sapiens
182	11716442_ brown	RNA polymerase II subunit L(POLR2L)	Homo sapiens
183	11716448_ blue	nudix hydrolase 9(NUDT9)	Homo sapiens
184	11716450_ turquoise	raftlin, lipid raft linker 1(RFTN1)	Homo sapiens
185	11716466_ turquoise	iron-sulfur cluster assembly 1(ISCA1)	Homo sapiens
186	11716467_ turquoise	iron-sulfur cluster assembly 1(ISCA1)	Homo sapiens
187	11716468_ blue	iron-sulfur cluster assembly 1(ISCA1)	Homo sapiens
188	11716470_ turquoise	ubiquinol-cytochrome c reductase binding protein(UQCRB)	Homo sapiens
189	11716472_ brown	Raf-1 proto-oncogene, serine/threonine kinase(RAF1)	Homo sapiens
190	11716479_ turquoise	coagulation factor XIII A chain(F13A1)	Homo sapiens
191	11716485_ turquoise	serine/threonine kinase 38(STK38)	Homo sapiens
192	11716513_ turquoise	molybdenum cofactor synthesis 2(MOCS2)	Homo sapiens
193	11716519_ brown	short chain dehydrogenase/reductase family 39U member 1(SDR39U1)	Homo sapiens
194	11716533_ turquoise	palmitoyl-protein thioesterase 1(PPT1)	Homo sapiens
195	11716534_ turquoise	palmitoyl-protein thioesterase 1(PPT1)	Homo sapiens
196	11716554_ turquoise	major histocompatibility complex, class II, DM alpha(HLA-DMA)	Homo sapiens
197	11716562_ blue	aminoacyl tRNA synthetase complex interacting multifunctional protein 2(AIMP2)	Homo sapiens
198	11716563_ brown	aminoacyl tRNA synthetase complex interacting multifunctional protein 2(AIMP2)	Homo sapiens

199	11716564_	turquoise	serologically defined colon cancer antigen 3(SDCCAG3)	Homo sapiens
200	11716567_	turquoise	proline rich coiled-coil 2C(PRRC2C)	Homo sapiens
201	11716598_	turquoise	charged multivesicular body protein 1A(CHMP1A)	Homo sapiens
202	11716599_	turquoise	charged multivesicular body protein 1A(CHMP1A)	Homo sapiens
203	11716606_	brown	stomatin like 2(STOML2)	Homo sapiens
204	11716617_	brown	mitochondrial ribosomal protein L36(MRPL36)	Homo sapiens
205	11716618_	turquoise	SS18 like 2(SS18L2)	Homo sapiens
206	11716633_	turquoise	transmembrane protein 50A(TMEM50A)	Homo sapiens
207	11716638_	turquoise	collagen type IV alpha 1 chain(COL4A1)	Homo sapiens
208	11716639_	turquoise	collagen type IV alpha 1 chain(COL4A1)	Homo sapiens
209	11716640_	turquoise	proteasome subunit beta 10(PSMB10)	Homo sapiens
210	11716641_	turquoise	proteasome subunit beta 10(PSMB10)	Homo sapiens
211	11716646_	turquoise	histocompatibility minor 13(HM13)	Homo sapiens
212	11716647_	turquoise	histocompatibility minor 13(HM13)	Homo sapiens
213	11716650_	turquoise	ubiquitin conjugating enzyme E2 Z(UBE2Z)	Homo sapiens
214	11716651_	turquoise	ubiquitin conjugating enzyme E2 Z(UBE2Z)	Homo sapiens
215	11716653_	turquoise	echinoderm microtubule associated protein like 4(EML4)	Homo sapiens
216	11716654_	turquoise	echinoderm microtubule associated protein like 4(EML4)	Homo sapiens
217	11716662_	turquoise	sushi domain containing 6(SUSD6)	Homo sapiens
218	11716666_	turquoise	inhibitor of DNA binding 3, HLH protein(ID3)	Homo sapiens
219	11716682_	turquoise	solute carrier family 39 member 9(SLC39A9)	Homo sapiens
220	11716683_	turquoise	solute carrier family 39 member 9(SLC39A9)	Homo sapiens
221	11716694_	grey	tetraspanin 6(TSPAN6)	Homo sapiens
222	11716700_	brown	SNAP associated protein(SNAPIN)	Homo sapiens
223	11716733_	turquoise	interferon regulatory factor 1(IRF1)	Homo sapiens
224	11716734_	turquoise	interferon regulatory factor 1(IRF1)	Homo sapiens
225	11716739_	turquoise	GRAM domain containing 1A(GRAMD1A)	Homo sapiens
226	11716746_	turquoise	family with sequence similarity 60 member A(FAM60A)	Homo sapiens
227	11716759_	turquoise	caspase 4(CASP4)	Homo sapiens
228	11716760_	turquoise	caspase 4(CASP4)	Homo sapiens
229	11716765_	turquoise	IQ motif containing GTPase activating protein 1(IQGAP1)	Homo sapiens
230	11716766_	turquoise	IQ motif containing GTPase activating protein 1(IQGAP1)	Homo sapiens
231	11716767_	turquoise	IQ motif containing GTPase activating protein 1(IQGAP1)	Homo sapiens
232	11716771_	turquoise	salt inducible kinase 1(SIK1)	Homo sapiens
233	11716774_	turquoise	retinoic acid induced 14(RAI14)	Homo sapiens
234	11716775_	turquoise	retinoic acid induced 14(RAI14)	Homo sapiens
235	11716787_	turquoise	beta-1,4-glucuronyltransferase 1(B4GAT1)	Homo sapiens
236	11716788_	turquoise	beta-1,4-glucuronyltransferase 1(B4GAT1)	Homo sapiens
237	11716792_	turquoise	uncharacterized LOC440034(DKFZp686K1684)	Homo sapiens
238	11716793_	turquoise	cyclin B2(CCNB2)	Homo sapiens
239	11716794_	turquoise	myosin light chain 6(MYL6)	Homo sapiens
240	11716795_	turquoise	interferon gamma inducible protein 16(IFI16)	Homo sapiens
241	11716796_	turquoise	interferon gamma inducible protein 16(IFI16)	Homo sapiens
242	11716797_	turquoise	lamin B receptor(LBR)	Homo sapiens
243	11716800_	turquoise	Fas associated via death domain(FADD)	Homo sapiens
244	11716828_	turquoise	NPC intracellular cholesterol transporter 2(NPC2)	Homo sapiens
245	11716842_	turquoise	CD53 molecule(CD53)	Homo sapiens
246	11716846_	turquoise	membrane spanning 4-domains A6A(MS4A6A)	Homo sapiens
247	11716847_	turquoise	solute carrier family 43 member 3(SLC43A3)	Homo sapiens
248	11716848_	turquoise	phosphatidylinositol-3,4,5-trisphosphate dependent Rac exchange factor 1(PREX1)	Homo sapiens

249	11716849_ turquoise	phosphatidylinositol-3,4,5-trisphosphate dependent Rac exchange factor 1(PREX1)	Homo sapiens
250	11716850_ turquoise	major facilitator superfamily domain containing 14B(MFSD14B)	Homo sapiens
251	11716859_ blue	electron transfer flavoprotein alpha subunit(ETFA)	Homo sapiens
252	11716865_ turquoise	solute carrier family 1 member 5(SLC1A5)	Homo sapiens
253	11716866_ turquoise	solute carrier family 1 member 5(SLC1A5)	Homo sapiens
254	11716867_ turquoise	fem-1 homolog A(FEM1A)	Homo sapiens
255	11716868_ turquoise	UTP18, small subunit processome component(UTP18)	Homo sapiens
256	11716883_ turquoise	RAP1B, member of RAS oncogene family(RAP1B)	Homo sapiens
257	11716888_ turquoise	hexosaminidase subunit alpha(HEXA)	Homo sapiens
258	11716890_ brown	transmembrane protein 261(TMEM261)	Homo sapiens
259	11716895_ turquoise	ISG15 ubiquitin-like modifier(ISG15)	Homo sapiens
260	11716900_ brown	ADP ribosylation factor like GTPase 3(ARL3)	Homo sapiens
261	11716901_ turquoise	trans-golgi network protein 2(TGOLN2)	Homo sapiens
262	11716904_ turquoise	trans-golgi network protein 2(TGOLN2)	Homo sapiens
263	11716908_ turquoise	ribonuclease T2(RNASET2)	Homo sapiens
264	11716918_ turquoise	N-acetylglucosamine kinase(NAGK)	Homo sapiens
265	11716920_ turquoise	plasmalemma vesicle associated protein(PLVAP)	Homo sapiens
266	11716935_ brown	superoxide dismutase 1, soluble(SOD1)	Homo sapiens
267	11716938_ turquoise	Sec61 translocon beta subunit(SEC61B)	Homo sapiens
268	11716939_ turquoise	heme oxygenase 1(HMOX1)	Homo sapiens
269	11716941_ turquoise	tribbles pseudokinase 2(TRIB2)	Homo sapiens
270	11716945_ turquoise	twinfilin actin binding protein 1(TWF1)	Homo sapiens
271	11716953_ brown	transmembrane protein 126A(TMEM126A)	Homo sapiens
272	11716962_ turquoise	protein phosphatase, Mg2+/Mn2+ dependent 1F(PPM1F)	Homo sapiens
273	11716963_ turquoise	protein phosphatase, Mg2+/Mn2+ dependent 1F(PPM1F)	Homo sapiens
274	11716972_ turquoise	N-ethylmaleimide sensitive factor, vesicle fusing ATPase(NSF)	Homo sapiens
275	11716984_ brown	paroxysmal nonkinesigenic dyskinesia(PNKD)	Homo sapiens
276	11716986_ turquoise	general transcription factor IIB(GTF2B)	Homo sapiens
277	11716993_ turquoise	colony stimulating factor 1 receptor(CSF1R)	Homo sapiens
278	11717012_ turquoise	vesicle associated membrane protein 8(VAMP8)	Homo sapiens
279	11717045_ turquoise	complement C2(C2)	Homo sapiens
280	11717051_ turquoise	signal sequence receptor subunit 1(SSR1)	Homo sapiens
281	11717060_ brown	2,4-dienoyl-CoA reductase 1, mitochondrial(DECR1)	Homo sapiens
282	11717104_ turquoise	glyoxylate and hydroxypyruvate reductase(GRHPR)	Homo sapiens
283	11717114_ turquoise	SPT16 homolog, facilitates chromatin remodeling subunit(SUPT16H)	Homo sapiens
284	11717127_ turquoise	alanyl aminopeptidase, membrane(ANPEP)	Homo sapiens
285	11717153_ turquoise	adipocyte plasma membrane associated protein(APMAP)	Homo sapiens
286	11717160_ turquoise	clathrin light chain A(CLTA)	Homo sapiens
287	11717163_ turquoise	cell division cycle 20(CDC20)	Homo sapiens
288	11717167_ turquoise	lysine demethylase 2A(KDM2A)	Homo sapiens
289	11717178_ turquoise	signal transducer and activator of transcription 5A(STAT5A)	Homo sapiens
290	11717188_ turquoise	DnaJ heat shock protein family (Hsp40) member A1(DNAJA1)	Homo sapiens
291	11717189_ turquoise	DnaJ heat shock protein family (Hsp40) member A1(DNAJA1)	Homo sapiens
292	11717211_ turquoise	neogenin 1(NEO1)	Homo sapiens
293	11717217_ turquoise	serine/threonine kinase 10(STK10)	Homo sapiens
294	11717218_ turquoise	serine/threonine kinase 10(STK10)	Homo sapiens
295	11717228_ turquoise	cerebellar degeneration-related protein 2(LOC101060399)	Homo sapiens
296	11717251_ brown	NADH:ubiquinone oxidoreductase subunit B11(NDUFB11)	Homo sapiens
297	11717252_ brown	NADH:ubiquinone oxidoreductase subunit B11(NDUFB11)	Homo sapiens
298	11717256_ turquoise	Pim-1 proto-oncogene, serine/threonine kinase(PIM1)	Homo sapiens

299	11717257_ turquoise	Pim-1 proto-oncogene, serine/threonine kinase(PIM1)	Homo sapiens
300	11717281_ turquoise	ArfGAP with SH3 domain, ankyrin repeat and PH domain 1(ASAP1)	Homo sapiens
301	11717282_ turquoise	ArfGAP with SH3 domain, ankyrin repeat and PH domain 1(ASAP1)	Homo sapiens
302	11717293_ turquoise	serine and arginine rich splicing factor 7(SRSF7)	Homo sapiens
303	11717296_ turquoise	leucine rich pentatricopeptide repeat containing(LRPPRC)	Homo sapiens
304	11717297_ blue	leucine rich pentatricopeptide repeat containing(LRPPRC)	Homo sapiens
305	11717299_ turquoise	major histocompatibility complex, class II, DP beta 1(HLA-DPB1)	Homo sapiens
306	11717300_ turquoise	major histocompatibility complex, class II, DP beta 1(HLA-DPB1)	Homo sapiens
307	11717311_ turquoise	F-box protein 7(FBXO7)	Homo sapiens
308	11717317_ turquoise	glycerol-3-phosphate acyltransferase 4(GPAT4)	Homo sapiens
309	11717319_ turquoise	SEC24 homolog D, COPII coat complex component(SEC24D)	Homo sapiens
310	11717325_ turquoise	nudix hydrolase 16 like 1(NUDT16L1)	Homo sapiens
311	11717337_ turquoise	integrin subunit beta 2(ITGB2)	Homo sapiens
312	11717365_ turquoise	syntrophin alpha 1(SNTA1)	Homo sapiens
313	11717366_ turquoise	zinc finger CCCH-type containing, antiviral 1(ZC3HAV1)	Homo sapiens
314	11717367_ turquoise	zinc finger CCCH-type containing, antiviral 1(ZC3HAV1)	Homo sapiens
315	11717368_ turquoise	zinc finger CCCH-type containing, antiviral 1(ZC3HAV1)	Homo sapiens
316	11717371_ turquoise	chromodomain helicase DNA binding protein 1 like(CHD1L)	Homo sapiens
317	11717375_ turquoise	solute carrier family 25 member 25(SLC25A25)	Homo sapiens
318	11717394_ turquoise	ariadne RBR E3 ubiquitin protein ligase 2(ARIH2)	Homo sapiens
319	11717397_ turquoise	damage specific DNA binding protein 2(DDB2)	Homo sapiens
320	11717401_ turquoise	ADP ribosylation factor 6(ARF6)	Homo sapiens
321	11717402_ turquoise	ADP ribosylation factor 6(ARF6)	Homo sapiens
322	11717407_ brown	ribonuclease P/MRP subunit p25 like(RPP25L)	Homo sapiens
323	11717409_ turquoise	SH2B adaptor protein 3(SH2B3)	Homo sapiens
324	11717418_ turquoise	dynein axonemal light chain 1(DNAL1)	Homo sapiens
325	11717423_ turquoise	RNA binding motif protein 8A(RBM8A)	Homo sapiens
326	11717425_ turquoise	RNF103-CHMP3 readthrough(RNF103-CHMP3)	Homo sapiens
327	11717427_ turquoise	G protein subunit beta 1(GNB1)	Homo sapiens
328	11717429_ brown	peroxiredoxin 5(PRDX5)	Homo sapiens
329	11717430_ blue	cytochrome c oxidase subunit 7A1(COX7A1)	Homo sapiens
330	11717438_ turquoise	topoisomerase (DNA) I(TOP1)	Homo sapiens
331	11717447_ turquoise	destrin, actin depolymerizing factor(DSTN)	Homo sapiens
332	11717453_ turquoise	SPARC/osteonectin, cwcv and kazal like domains proteoglycan 2(SPOCK2)	Homo sapiens
333	11717454_ turquoise	SPARC/osteonectin, cwcv and kazal like domains proteoglycan 2(SPOCK2)	Homo sapiens
334	11717464_ turquoise	latexin(LXN)	Homo sapiens
335	11717465_ turquoise	latexin(LXN)	Homo sapiens
336	11717479_ turquoise	lysosomal associated membrane protein 2(LAMP2)	Homo sapiens
337	11717482_ turquoise	pyruvate dehydrogenase (lipoamide) alpha 1(PDHA1)	Homo sapiens
338	11717483_ blue	pyruvate dehydrogenase (lipoamide) alpha 1(PDHA1)	Homo sapiens
339	11717484_ blue	pyruvate dehydrogenase (lipoamide) alpha 1(PDHA1)	Homo sapiens
340	11717496_ turquoise	inositol monophosphatase 2(IMPA2)	Homo sapiens
341	11717499_ blue	solute carrier family 25 member 36(SLC25A36)	Homo sapiens
342	11717501_ turquoise	hydroxyacylglutathione hydrolase(HAGH)	Homo sapiens
343	11717502_ turquoise	negative regulator of ubiquitin like proteins 1(NUB1)	Homo sapiens
344	11717503_ turquoise	negative regulator of ubiquitin like proteins 1(NUB1)	Homo sapiens
345	11717507_ turquoise	translocation associated membrane protein 2(TRAM2)	Homo sapiens
346	11717508_ turquoise	interferon regulatory factor 4(IRF4)	Homo sapiens
347	11717514_ turquoise	annexin A1(ANXA1)	Homo sapiens
348	11717517_ turquoise	RNA exonuclease 2(REXO2)	Homo sapiens

349	11717521_	turquoise	pituitary tumor-transforming 3, pseudogene(PTTG3P)	Homo sapiens
350	11717528_	turquoise	solute carrier family 25 member 20(SLC25A20)	Homo sapiens
351	11717529_	turquoise	solute carrier family 25 member 20(SLC25A20)	Homo sapiens
352	11717533_	turquoise	mannosidase alpha class 2A member 2(MAN2A2)	Homo sapiens
353	11717542_	turquoise	nuclear factor kappa B subunit 1(NFKB1)	Homo sapiens
354	11717561_	turquoise	deltex E3 ubiquitin ligase 3L(DTX3L)	Homo sapiens
355	11717562_	turquoise	deltex E3 ubiquitin ligase 3L(DTX3L)	Homo sapiens
356	11717574_	turquoise	profilin 1(PFN1)	Homo sapiens
357	11717580_	turquoise	CD52 molecule(CD52)	Homo sapiens
358	11717581_	turquoise	CD52 molecule(CD52)	Homo sapiens
359	11717611_	turquoise	microtubule associated serine/threonine kinase 2(MAST2)	Homo sapiens
360	11717634_	brown	cytochrome c oxidase subunit 6C(COX6C)	Homo sapiens
361	11717636_	turquoise	ras homolog family member G(RHOG)	Homo sapiens
362	11717637_	turquoise	ras homolog family member G(RHOG)	Homo sapiens
363	11717643_	brown	mitochondrial ribosomal protein L24(MRPL24)	Homo sapiens
364	11717654_	brown	C-X9-C motif containing 4(CMC4)	Homo sapiens
365	11717657_	turquoise	enhancer of zeste 2 polycomb repressive complex 2 subunit(EZH2)	Homo sapiens
366	11717661_	turquoise	protein phosphatase 1 regulatory subunit 16B(PPP1R16B)	Homo sapiens
367	11717662_	turquoise	piezo type mechanosensitive ion channel component 1(PIEZO1)	Homo sapiens
368	11717670_	brown	NADH:ubiquinone oxidoreductase subunit B6(NDUFB6)	Homo sapiens
369	11717672_	turquoise	ectonucleotide pyrophosphatase/phosphodiesterase 2(ENPP2)	Homo sapiens
370	11717679_	turquoise	unc-13 homolog B(UNC13B)	Homo sapiens
371	11717681_	turquoise	G protein subunit alpha 13(GNA13)	Homo sapiens
372	11717688_	turquoise	ring finger protein 19A, RBR E3 ubiquitin protein ligase(RNF19A)	Homo sapiens
373	11717691_	turquoise	ankyrin repeat domain 13A(ANKRD13A)	Homo sapiens
374	11717708_	turquoise	protein tyrosine phosphatase, non-receptor type 18(PTPN18)	Homo sapiens
375	11717714_	turquoise	carbohydrate sulfotransferase 10(CHST10)	Homo sapiens
376	11717721_	turquoise	ST6 beta-galactoside alpha-2,6-sialyltransferase 1(ST6GAL1)	Homo sapiens
377	11717726_	turquoise	hematological and neurological expressed 1(HN1)	Homo sapiens
378	11717727_	turquoise	hematological and neurological expressed 1(HN1)	Homo sapiens
379	11717730_	turquoise	squalene epoxidase(SQLE)	Homo sapiens
380	11717732_	turquoise	6-phosphofructo-2-kinase/fructose-2,6-biphosphatase 3(PFKFB3)	Homo sapiens
381	11717737_	turquoise	prenylcysteine oxidase 1(PCYOX1)	Homo sapiens
382	11717738_	turquoise	prenylcysteine oxidase 1(PCYOX1)	Homo sapiens
383	11717739_	turquoise	prenylcysteine oxidase 1(PCYOX1)	Homo sapiens
384	11717743_	turquoise	acyl-CoA thioesterase 7(ACOT7)	Homo sapiens
385	11717746_	turquoise	membrane associated ring-CH-type finger 6(MARCH6)	Homo sapiens
386	11717760_	turquoise	glia maturation factor gamma(GMFG)	Homo sapiens
387	11717800_	turquoise	activating transcription factor 5(ATF5)	Homo sapiens
388	11717801_	turquoise	activating transcription factor 5(ATF5)	Homo sapiens
389	11717823_	turquoise	TNF alpha induced protein 2(TNFAIP2)	Homo sapiens
390	11717836_	turquoise	allograft inflammatory factor 1(AIF1)	Homo sapiens
391	11717837_	turquoise	allograft inflammatory factor 1(AIF1)	Homo sapiens
392	11717839_	turquoise	programmed cell death 6(PDCD6)	Homo sapiens
393	11717840_	turquoise	ethanolamine kinase 1(ETNK1)	Homo sapiens
394	11717841_	turquoise	ethanolamine kinase 1(ETNK1)	Homo sapiens
395	11717858_	turquoise	CD37 molecule(CD37)	Homo sapiens
396	11717859_	brown	emopamil binding protein (sterol isomerase)(EBP)	Homo sapiens
397	11717865_	turquoise	proteasome activator subunit 4(PSME4)	Homo sapiens
398	11717872_	turquoise	interferon regulatory factor 8(IRF8)	Homo sapiens

399	11717873_ turquoise	interferon regulatory factor 8(IRF8)	Homo sapiens
400	11717874_ turquoise	interferon regulatory factor 8(IRF8)	Homo sapiens
401	11717883_ turquoise	zinc fingers and homeoboxes 3(ZHX3)	Homo sapiens
402	11717884_ turquoise	zinc fingers and homeoboxes 3(ZHX3)	Homo sapiens
403	11717886_ turquoise	plasminogen activator, urokinase(PLAU)	Homo sapiens
404	11717887_ turquoise	protein tyrosine phosphatase, non-receptor type 12(PTPN12)	Homo sapiens
405	11717889_ turquoise	G protein subunit alpha i2(GNAI2)	Homo sapiens
406	11717931_ turquoise	TANK binding kinase 1(TBK1)	Homo sapiens
407	11717936_ blue	endoplasmic reticulum metallopeptidase 1(ERMP1)	Homo sapiens
408	11717939_ turquoise	U2 small nuclear RNA auxiliary factor 2(U2AF2)	Homo sapiens
409	11717959_ turquoise	transcription factor 3(TCF3)	Homo sapiens
410	11717965_ turquoise	KRAS proto-oncogene, GTPase(KRAS)	Homo sapiens
411	11717972_ turquoise	family with sequence similarity 219 member B(FAM219B)	Homo sapiens
412	11717973_ turquoise	family with sequence similarity 219 member B(FAM219B)	Homo sapiens
413	11717974_ turquoise	family with sequence similarity 219 member B(FAM219B)	Homo sapiens
414	11717981_ turquoise	acid phosphatase 5, tartrate resistant(ACP5)	Homo sapiens
415	11717988_ turquoise	RNASEK-C17orf49 readthrough(RNASEK-C17orf49)	Homo sapiens
416	11718001_ turquoise	chromosome 10 open reading frame 54(C10orf54)	Homo sapiens
417	11718002_ turquoise	chromosome 10 open reading frame 54(C10orf54)	Homo sapiens
418	11718005_ turquoise	cell division cycle 25B(CDC25B)	Homo sapiens
419	11718008_ turquoise	copine 2(CPNE2)	Homo sapiens
420	11718010_ turquoise	solute carrier family 25 member 22(SLC25A22)	Homo sapiens
421	11718011_ turquoise	mitochondrial ribosomal protein L4(MRPL4)	Homo sapiens
422	11718020_ turquoise	leucine rich repeat containing 20(LRRC20)	Homo sapiens
423	11718026_ turquoise	nuclear receptor coactivator 7(NCOA7)	Homo sapiens
424	11718027_ turquoise	pseudouridylate synthase 1(PUS1)	Homo sapiens
425	11718028_ turquoise	pseudouridylate synthase 1(PUS1)	Homo sapiens
426	11718035_ blue	peptidylprolyl isomerase like 1(PPIL1)	Homo sapiens
427	11718036_ brown	peptidylprolyl isomerase like 1(PPIL1)	Homo sapiens
428	11718037_ turquoise	midkine (neurite growth-promoting factor 2)(MDK)	Homo sapiens
429	11718050_ turquoise	brain abundant membrane attached signal protein 1(BASP1)	Homo sapiens
430	11718058_ turquoise	thymidylate synthetase(TYMS)	Homo sapiens
431	11718060_ turquoise	tweety family member 2(TTYH2)	Homo sapiens
432	11718064_ turquoise	S100 calcium binding protein A4(S100A4)	Homo sapiens
433	11718065_ turquoise	nectin cell adhesion molecule 2(NECTIN2)	Homo sapiens
434	11718068_ turquoise	myosin IE(MYO1E)	Homo sapiens
435	11718073_ turquoise	interleukin 4 receptor(IL4R)	Homo sapiens
436	11718075_ turquoise	interleukin 4 receptor(IL4R)	Homo sapiens
437	11718076_ brown	mitogen-activated protein kinase-activated protein kinase 3(MAPKAPK3)	Homo sapiens
438	11718084_ turquoise	LYN proto-oncogene, Src family tyrosine kinase(LYN)	Homo sapiens
439	11718085_ turquoise	LYN proto-oncogene, Src family tyrosine kinase(LYN)	Homo sapiens
440	11718086_ turquoise	SH3KBP1 binding protein 1(SHKBP1)	Homo sapiens
441	11718099_ turquoise	intraflagellar transport 20(IFT20)	Homo sapiens
442	11718117_ blue	NFS1, cysteine desulfurase(NFS1)	Homo sapiens
443	11718121_ turquoise	ATPase H+ transporting V1 subunit A(ATP6V1A)	Homo sapiens
444	11718134_ turquoise	phosphoribosyl pyrophosphate synthetase 2(PRPS2)	Homo sapiens
445	11718135_ turquoise	phosphoribosyl pyrophosphate synthetase 2(PRPS2)	Homo sapiens
446	11718140_ turquoise	epithelial membrane protein 3(EMP3)	Homo sapiens
447	11718150_ brown	protease associated domain containing 1(PRADC1)	Homo sapiens
448	11718152_ turquoise	PPFIA binding protein 2(PPFIBP2)	Homo sapiens

449	11718158_ turquoise	N-myc and STAT interactor(NMI)	Homo sapiens
450	11718159_ turquoise	N-myc and STAT interactor(NMI)	Homo sapiens
451	11718184_ turquoise	FCH and double SH3 domains 2(FCHSD2)	Homo sapiens
452	11718186_ turquoise	ubiquitin like with PHD and ring finger domains 2(UHRF2)	Homo sapiens
453	11718204_ turquoise	REC8 meiotic recombination protein(REC8)	Homo sapiens
454	11718207_ blue	coenzyme Q6, monooxygenase(COQ6)	Homo sapiens
455	11718227_ blue	BCS1 homolog, ubiquinol-cytochrome c reductase complex chaperone(BCS1L)	Homo sapiens
456	11718230_ turquoise	major histocompatibility complex, class I, F(HLA-F)	Homo sapiens
457	11718231_ turquoise	major histocompatibility complex, class I, F(HLA-F)	Homo sapiens
458	11718239_ blue	cell cycle associated protein 1(CAPRIN1)	Homo sapiens
459	11718245_ turquoise	solute carrier family 25 member 42(SLC25A42)	Homo sapiens
460	11718265_ turquoise	SMAD family member 3(SMAD3)	Homo sapiens
461	11718266_ turquoise	SMAD family member 3(SMAD3)	Homo sapiens
462	11718276_ brown	interferon related developmental regulator 2(IFRD2)	Homo sapiens
463	11718278_ brown	NADH:ubiquinone oxidoreductase subunit B4(NDUFB4)	Homo sapiens
464	11718282_ brown	NADH:ubiquinone oxidoreductase core subunit V2(NDUFV2)	Homo sapiens
465	11718290_ turquoise	FXYD domain containing ion transport regulator 5(FXYD5)	Homo sapiens
466	11718293_ turquoise	cyclin dependent kinase inhibitor 2A(CDKN2A)	Homo sapiens
467	11718297_ turquoise	transforming acidic coiled-coil containing protein 3(TACC3)	Homo sapiens
468	11718303_ turquoise	platelet activating factor acetylhydrolase 1b catalytic subunit 3(PAFAH1B3)	Homo sapiens
469	11718312_ blue	acyl-CoA dehydrogenase, short/branched chain(ACADSB)	Homo sapiens
470	11718319_ turquoise	CD93 molecule(CD93)	Homo sapiens
471	11718322_ turquoise	ArfGAP with coiled-coil, ankyrin repeat and PH domains 2(ACAP2)	Homo sapiens
472	11718325_ turquoise	sestrin 2(SESN2)	Homo sapiens
473	11718340_ turquoise	DAZ associated protein 2(DAZAP2)	Homo sapiens
474	11718370_ turquoise	nuclear receptor binding factor 2(NRBF2)	Homo sapiens
475	11718381_ turquoise	pleckstrin and Sec7 domain containing 4(PSD4)	Homo sapiens
476	11718382_ turquoise	poly(ADP-ribose) polymerase family member 4(PARP4)	Homo sapiens
477	11718383_ blue	apoptosis inducing factor, mitochondria associated 1(AIFM1)	Homo sapiens
478	11718400_ turquoise	purine nucleoside phosphorylase(PNP)	Homo sapiens
479	11718415_ turquoise	non-POU domain containing, octamer-binding(NONO)	Homo sapiens
480	11718417_ turquoise	ECSIT signalling integrator(ECSIT)	Homo sapiens
481	11718418_ turquoise	thiosulfate sulfurtransferase(TST)	Homo sapiens
482	11718419_ turquoise	Fc fragment of IgE receptor Ig(FCER1G)	Homo sapiens
483	11718420_ turquoise	CLPTM1 like(CLPTM1L)	Homo sapiens
484	11718425_ turquoise	hydroxysteroid dehydrogenase like 2(HSDL2)	Homo sapiens
485	11718426_ blue	hydroxysteroid dehydrogenase like 2(HSDL2)	Homo sapiens
486	11718430_ brown	zinc finger protein 32(ZNF32)	Homo sapiens
487	11718436_ turquoise	ATP synthase mitochondrial F1 complex assembly factor 2(ATPAF2)	Homo sapiens
488	11718450_ turquoise	alpha-N-acetylgalactosaminidase(NAGA)	Homo sapiens
489	11718461_ turquoise	solute carrier family 39 member 11(SLC39A11)	Homo sapiens
490	11718468_ turquoise	N-terminal EF-hand calcium binding protein 3(NECAB3)	Homo sapiens
491	11718512_ turquoise	tetraspanin 14(TSPAN14)	Homo sapiens
492	11718513_ turquoise	tetraspanin 14(TSPAN14)	Homo sapiens
493	11718514_ turquoise	tetraspanin 14(TSPAN14)	Homo sapiens
494	11718515_ turquoise	sulfide quinone reductase-like (yeast)(SQRDL)	Homo sapiens
495	11718520_ turquoise	potassium channel modulatory factor 1(KCMF1)	Homo sapiens
496	11718525_ turquoise	non imprinted in Prader-Willi/Angelman syndrome 2(NIPA2)	Homo sapiens
497	11718528_ turquoise	torsin family 3 member A(TOR3A)	Homo sapiens
498	11718534_ turquoise	natural killer cell triggering receptor(NKTR)	Homo sapiens

499	11718537_ turquoise	fumarylacetoacetate hydrolase domain containing 2B(FAHD2B)	Homo sapiens
500	11718538_ turquoise	fumarylacetoacetate hydrolase domain containing 2A(FAHD2A)	Homo sapiens
501	11718540_ turquoise	transmembrane protein 243(TMEM243)	Homo sapiens
502	11718546_ turquoise	interleukin enhancer binding factor 3(ILF3)	Homo sapiens
503	11718559_ turquoise	nucleolar protein 4 like(NOL4L)	Homo sapiens
504	11718563_ turquoise	ankyrin repeat domain 10(ANKRD10)	Homo sapiens
505	11718568_ turquoise	WAS/WASL interacting protein family member 1(WIPF1)	Homo sapiens
506	11718569_ turquoise	WAS/WASL interacting protein family member 1(WIPF1)	Homo sapiens
507	11718580_ blue	coenzyme Q9(COQ9)	Homo sapiens
508	11718591_ turquoise	stromal cell derived factor 2 like 1(SDF2L1)	Homo sapiens
509	11718592_ turquoise	reticulon 4(RTN4)	Homo sapiens
510	11718594_ turquoise	cyclin L1(CCNL1)	Homo sapiens
511	11718608_ turquoise	microRNA 5193(MIR5193)	Homo sapiens
512	11718610_ turquoise	tumor protein p53 inducible nuclear protein 1(TP53INP1)	Homo sapiens
513	11718621_ turquoise	dual specificity phosphatase 12(DUSP12)	Homo sapiens
514	11718625_ turquoise	deoxyguanosine kinase(DGUOK)	Homo sapiens
515	11718631_ turquoise	SERTA domain containing 1(SERTAD1)	Homo sapiens
516	11718641_ turquoise	N-deacetylase and N-sulfotransferase 2(NDST2)	Homo sapiens
517	11718652_ turquoise	signal transducer and activator of transcription 2(STAT2)	Homo sapiens
518	11718655_ turquoise	charged multivesicular body protein 5(CHMP5)	Homo sapiens
519	11718657_ turquoise	microRNA 21(MIR21)	Homo sapiens
520	11718659_ turquoise	Mov10 RISC complex RNA helicase(MOV10)	Homo sapiens
521	11718665_ turquoise	glutamyl-tRNA amidotransferase subunit C(GATC)	Homo sapiens
522	11718666_ turquoise	glutamyl-tRNA amidotransferase subunit C(GATC)	Homo sapiens
523	11718669_ blue	mediator complex subunit 9(MED9)	Homo sapiens
524	11718670_ turquoise	mediator complex subunit 9(MED9)	Homo sapiens
525	11718672_ turquoise	interferon regulatory factor 3(IRF3)	Homo sapiens
526	11718674_ turquoise	transmembrane protein 245(TMEM245)	Homo sapiens
527	11718678_ turquoise	presenilin 1(PSEN1)	Homo sapiens
528	11718680_ turquoise	adhesion G protein-coupled receptor E5(ADGRE5)	Homo sapiens
529	11718699_ turquoise	chromosome 6 open reading frame 89(C6orf89)	Homo sapiens
530	11718757_ turquoise	hypoxia inducible factor 1 alpha subunit(HIF1A)	Homo sapiens
531	11718764_ brown	cytochrome c oxidase subunit 5B(COX5B)	Homo sapiens
532	11718765_ brown	NADH:ubiquinone oxidoreductase subunit A6(NDUFA6)	Homo sapiens
533	11718766_ turquoise	protease, serine 23(PRSS23)	Homo sapiens
534	11718767_ turquoise	protease, serine 23(PRSS23)	Homo sapiens
535	11718788_ turquoise	adenosine deaminase(ADA)	Homo sapiens
536	11718807_ turquoise	TNF receptor superfamily member 1B(TNFRSF1B)	Homo sapiens
537	11718809_ turquoise	protein kinase C eta(PRKCH)	Homo sapiens
538	11718810_ turquoise	protein kinase C eta(PRKCH)	Homo sapiens
539	11718811_ turquoise	protein kinase C eta(PRKCH)	Homo sapiens
540	11718830_ turquoise	EH domain containing 1(EHD1)	Homo sapiens
541	11718831_ brown	NADH:ubiquinone oxidoreductase subunit A2(NDUFA2)	Homo sapiens
542	11718832_ turquoise	lysyl oxidase like 2(LOXL2)	Homo sapiens
543	11718837_ blue	NADH:ubiquinone oxidoreductase core subunit S2(NDUFS2)	Homo sapiens
544	11718849_ turquoise	protein tyrosine phosphatase, receptor type C associated protein(PTPRCAP)	Homo sapiens
545	11718854_ blue	mitochondrial ribosomal protein S30(MRPS30)	Homo sapiens
546	11718860_ turquoise	Suv3 like RNA helicase(SUPV3L1)	Homo sapiens
547	11718861_ turquoise	HCK proto-oncogene, Src family tyrosine kinase(HCK)	Homo sapiens
548	11718865_ turquoise	COX10, heme A:farnesyltransferase cytochrome c oxidase assembly factor(COX10)	Homo sapiens

549	11718904_turquoise	filamin A interacting protein 1 like(FILIP1L)	Homo sapiens
550	11718908_turquoise	carbohydrate sulfotransferase 2(CHST2)	Homo sapiens
551	11718909_turquoise	carbohydrate sulfotransferase 2(CHST2)	Homo sapiens
552	11718912_turquoise	PDZ domain containing 2(PDZD2)	Homo sapiens
553	11718914_turquoise	PDZ domain containing 2(PDZD2)	Homo sapiens
554	11718915_turquoise	regulator of G-protein signaling 19(RGS19)	Homo sapiens
555	11718916_turquoise	interferon regulatory factor 7(IRF7)	Homo sapiens
556	11718924_turquoise	WW domain containing E3 ubiquitin protein ligase 2(WWP2)	Homo sapiens
557	11718929_turquoise	TGFB induced factor homeobox 2(TGIF2)	Homo sapiens
558	11718930_turquoise	TGFB induced factor homeobox 2(TGIF2)	Homo sapiens
559	11718935_turquoise	serine/threonine kinase 19(STK19)	Homo sapiens
560	11718939_turquoise	TNF alpha induced protein 3(TNFAIP3)	Homo sapiens
561	11718940_turquoise	TNF alpha induced protein 3(TNFAIP3)	Homo sapiens
562	11718948_blue	membrane palmitoylated protein 5(MPP5)	Homo sapiens
563	11718950_turquoise	membrane palmitoylated protein 5(MPP5)	Homo sapiens
564	11718954_turquoise	filamin binding LIM protein 1(FBLIM1)	Homo sapiens
565	11718958_turquoise	protein kinase AMP-activated non-catalytic subunit beta 2(PRKAB2)	Homo sapiens
566	11718981_turquoise	transgelin 2(TAGLN2)	Homo sapiens
567	11718982_turquoise	C-C motif chemokine ligand 4 like 1(CCL4L1)	Homo sapiens
568	11718983_turquoise	C-C motif chemokine ligand 4 like 1(CCL4L1)	Homo sapiens
569	11718986_turquoise	interferon alpha inducible protein 6(IFI6)	Homo sapiens
570	11718991_brown	NADH:ubiquinone oxidoreductase subunit B7(NDUFB7)	Homo sapiens
571	11718996_turquoise	methionine sulfoxide reductase B2(MSRB2)	Homo sapiens
572	11719016_turquoise	PQ loop repeat containing 3(PQLC3)	Homo sapiens
573	11719020_turquoise	WD repeat and FYVE domain containing 1(WDFY1)	Homo sapiens
574	11719021_turquoise	WD repeat and FYVE domain containing 1(WDFY1)	Homo sapiens
575	11719029_turquoise	phosphoinositide-3-kinase adaptor protein 1(PIK3AP1)	Homo sapiens
576	11719038_turquoise	ArfGAP with RhoGAP domain, ankyrin repeat and PH domain 1(ARAP1)	Homo sapiens
577	11719046_turquoise	SNF related kinase(SNRK)	Homo sapiens
578	11719050_turquoise	butyrophilin subfamily 2 member A1(BTN2A1)	Homo sapiens
579	11719065_blue	glyceronephosphate O-acyltransferase(GNPAT)	Homo sapiens
580	11719076_turquoise	receptor accessory protein 1(REEP1)	Homo sapiens
581	11719083_turquoise	TruB pseudouridine synthase family member 2(TRUB2)	Homo sapiens
582	11719089_turquoise	phosphoenolpyruvate carboxykinase 2, mitochondrial(PCK2)	Homo sapiens
583	11719111_brown	chromosome 11 open reading frame 1(C11orf1)	Homo sapiens
584	11719115_blue	glutamic-oxaloacetic transaminase 2(GOT2)	Homo sapiens
585	11719116_blue	glutamic-oxaloacetic transaminase 2(GOT2)	Homo sapiens
586	11719117_blue	glutamic-oxaloacetic transaminase 2(GOT2)	Homo sapiens
587	11719120_turquoise	kynureninase(KYNU)	Homo sapiens
588	11719123_turquoise	timeless circadian clock(TIMELESS)	Homo sapiens
589	11719126_turquoise	cell growth regulator with ring finger domain 1(CGRRF1)	Homo sapiens
590	11719128_turquoise	lipase maturation factor 2(LMF2)	Homo sapiens
591	11719132_turquoise	family with sequence similarity 49 member B(FAM49B)	Homo sapiens
592	11719154_turquoise	DNA damage regulated autophagy modulator 1(DRAM1)	Homo sapiens
593	11719155_turquoise	DNA damage regulated autophagy modulator 1(DRAM1)	Homo sapiens
594	11719164_turquoise	chloride voltage-gated channel 5(CLCN5)	Homo sapiens
595	11719182_turquoise	chloride voltage-gated channel 7(CLCN7)	Homo sapiens
596	11719188_turquoise	neuroblastoma RAS viral oncogene homolog(NRAS)	Homo sapiens
597	11719189_turquoise	neuroblastoma RAS viral oncogene homolog(NRAS)	Homo sapiens
598	11719193_turquoise	chibby family member 1, beta catenin antagonist(CBY1)	Homo sapiens

599	11719211_turquoise	heat shock transcription factor 2(HSF2)	Homo sapiens
600	11719216_turquoise	tudor domain containing 7(TDRD7)	Homo sapiens
601	11719218_turquoise	suppressor of cytokine signaling 3(SOCS3)	Homo sapiens
602	11719224_blue	coenzyme Q10A(COQ10A)	Homo sapiens
603	11719227_turquoise	potassium calcium-activated channel subfamily N member 4(KCNN4)	Homo sapiens
604	11719232_grey	protein tyrosine phosphatase, receptor type S(PTPRS)	Homo sapiens
605	11719245_turquoise	LARGE xylosyl- and glucuronyltransferase 1(LARGE1)	Homo sapiens
606	11719247_turquoise	absent in melanoma 1(AIM1)	Homo sapiens
607	11719248_turquoise	absent in melanoma 1(AIM1)	Homo sapiens
608	11719258_turquoise	toll like receptor adaptor molecule 1(TICAM1)	Homo sapiens
609	11719270_turquoise	RNA binding motif protein 22(RBM22)	Homo sapiens
610	11719271_turquoise	glucuronidase beta(GUSB)	Homo sapiens
611	11719304_brown	ER membrane protein complex subunit 8(EMC8)	Homo sapiens
612	11719305_turquoise	synaptopodin(SYNPO)	Homo sapiens
613	11719313_turquoise	family with sequence similarity 111 member A(FAM111A)	Homo sapiens
614	11719327_turquoise	cysteine rich with EGF like domains 2(CRELD2)	Homo sapiens
615	11719332_turquoise	uncharacterized LOC101928361(LOC101928361)	Homo sapiens
616	11719347_turquoise	Rho GTPase activating protein 19(ARHGAP19)	Homo sapiens
617	11719354_turquoise	SH3 domain binding protein 5(SH3BP5)	Homo sapiens
618	11719355_turquoise	SH3 domain binding protein 5(SH3BP5)	Homo sapiens
619	11719359_blue	aldehyde dehydrogenase 5 family member A1(ALDH5A1)	Homo sapiens
620	11719366_turquoise	C-X-C motif chemokine ligand 1(CXCL1)	Homo sapiens
621	11719367_turquoise	pleckstrin homology domain containing O2(PLEKHO2)	Homo sapiens
622	11719372_turquoise	brain expressed X-linked 3(BEX3)	Homo sapiens
623	11719377_turquoise	adenylate cyclase associated protein 1(CAP1)	Homo sapiens
624	11719378_turquoise	Rap1 GTPase-GDP dissociation stimulator 1(RAP1GDS1)	Homo sapiens
625	11719379_turquoise	Rap1 GTPase-GDP dissociation stimulator 1(RAP1GDS1)	Homo sapiens
626	11719398_turquoise	Ras related GTP binding C(RRAGC)	Homo sapiens
627	11719402_turquoise	RAP2A, member of RAS oncogene family(RAP2A)	Homo sapiens
628	11719411_turquoise	RAB32, member RAS oncogene family(RAB32)	Homo sapiens
629	11719413_turquoise	PBX homeobox 1(PBX1)	Homo sapiens
630	11719424_brown	family with sequence similarity 220 member A(FAM220A)	Homo sapiens
631	11719425_blue	family with sequence similarity 220 member A(FAM220A)	Homo sapiens
632	11719435_turquoise	LDL receptor related protein 4(LRP4)	Homo sapiens
633	11719438_turquoise	SLIT-ROBO Rho GTPase activating protein 2(SRGAP2)	Homo sapiens
634	11719447_turquoise	guanylate binding protein 2(GBP2)	Homo sapiens
635	11719465_turquoise	complement C1q B chain(C1QB)	Homo sapiens
636	11719466_turquoise	complement C1q B chain(C1QB)	Homo sapiens
637	11719479_turquoise	arachidonate 5-lipoxygenase activating protein(ALOX5AP)	Homo sapiens
638	11719480_turquoise	cystatin A(CSTA)	Homo sapiens
639	11719482_brown	mitochondrial ribosomal protein L21(MRPL21)	Homo sapiens
640	11719483_turquoise	purinergic receptor P2X 4(P2RX4)	Homo sapiens
641	11719491_turquoise	interferon induced protein 35(IFI35)	Homo sapiens
642	11719492_turquoise	interferon induced protein 35(IFI35)	Homo sapiens
643	11719499_turquoise	monoamine oxidase B(MAOB)	Homo sapiens
644	11719501_turquoise	nuclear factor of activated T-cells 3(NFATC3)	Homo sapiens
645	11719513_turquoise	ADAM metallopeptidase domain 15(ADAM15)	Homo sapiens
646	11719528_turquoise	nectin cell adhesion molecule 2(NECTIN2)	Homo sapiens
647	11719539_turquoise	nuclear envelope integral membrane protein 1(NEMP1)	Homo sapiens
648	11719543_turquoise	chromosome 3 open reading frame 18(C3orf18)	Homo sapiens

649	11719554_turquoise	cathepsin S(CTSS)	Homo sapiens
650	11719556_turquoise	pyruvate dehydrogenase kinase 2(PDK2)	Homo sapiens
651	11719566_brown	mitochondrial ribosomal protein S15(MRPS15)	Homo sapiens
652	11719579_turquoise	translational activator of cytochrome c oxidase I(TACO1)	Homo sapiens
653	11719587_turquoise	MICAL like 1(MICALL1)	Homo sapiens
654	11719588_turquoise	2'-5'-oligoadenylate synthetase 1(OAS1)	Homo sapiens
655	11719591_turquoise	glycolipid transfer protein(GLTP)	Homo sapiens
656	11719599_turquoise	homer scaffolding protein 2(HOMER2)	Homo sapiens
657	11719629_turquoise	bromodomain containing 2(BRD2)	Homo sapiens
658	11719630_turquoise	bromodomain containing 2(BRD2)	Homo sapiens
659	11719631_turquoise	bridging integrator 1(BIN1)	Homo sapiens
660	11719644_turquoise	GATA zinc finger domain containing 2A(GATAD2A)	Homo sapiens
661	11719648_turquoise	caspase 7(CASP7)	Homo sapiens
662	11719657_turquoise	matrix metallopeptidase 9(MMP9)	Homo sapiens
663	11719675_turquoise	vascular cell adhesion molecule 1(VCAM1)	Homo sapiens
664	11719676_turquoise	monoamine oxidase A(MAOA)	Homo sapiens
665	11719680_turquoise	TNF receptor superfamily member 10b(TNFRSF10B)	Homo sapiens
666	11719686_turquoise	microtubule associated monooxygenase, calponin and LIM domain containing 1(MICAL	Homo sapiens
667	11719687_turquoise	SET domain bifurcated 1(SETDB1)	Homo sapiens
668	11719692_turquoise	retinoic acid receptor responder 3(RARRES3)	Homo sapiens
669	11719718_turquoise	spleen associated tyrosine kinase(SYK)	Homo sapiens
670	11719728_turquoise	signal regulatory protein alpha(SIRPA)	Homo sapiens
671	11719738_turquoise	decapping mRNA 2(DCP2)	Homo sapiens
672	11719739_turquoise	decapping mRNA 2(DCP2)	Homo sapiens
673	11719745_turquoise	Rho GTPase activating protein 27(ARHGAP27)	Homo sapiens
674	11719749_turquoise	emopamil binding protein like(EBPL)	Homo sapiens
675	11719754_turquoise	interleukin 1 receptor antagonist(IL1RN)	Homo sapiens
676	11719763_turquoise	vacuolar protein sorting 13 homolog C(VPS13C)	Homo sapiens
677	11719780_turquoise	TNF alpha induced protein 8 like 2(TNFAIP8L2)	Homo sapiens
678	11719800_turquoise	chromosome 6 open reading frame 62(C6orf62)	Homo sapiens
679	11719801_turquoise	chromosome 6 open reading frame 62(C6orf62)	Homo sapiens
680	11719827_turquoise	glutaminyl-peptide cyclotransferase(QPCT)	Homo sapiens
681	11719828_turquoise	glutaminyl-peptide cyclotransferase(QPCT)	Homo sapiens
682	11719830_brown	ERCC excision repair 1, endonuclease non-catalytic subunit(ERCC1)	Homo sapiens
683	11719833_turquoise	myelin protein zero like 2(MPZL2)	Homo sapiens
684	11719838_turquoise	caspase 3(CASP3)	Homo sapiens
685	11719844_turquoise	small ArfGAP2(SMAP2)	Homo sapiens
686	11719845_turquoise	small ArfGAP2(SMAP2)	Homo sapiens
687	11719861_turquoise	SLC2A4 regulator(SLC2A4RG)	Homo sapiens
688	11719864_turquoise	small cell adhesion glycoprotein(SMAGP)	Homo sapiens
689	11719868_turquoise	v-myc avian myelocytomatosis viral oncogene neuroblastoma derived homolog(MYCN)	Homo sapiens
690	11719869_turquoise	v-myc avian myelocytomatosis viral oncogene neuroblastoma derived homolog(MYCN)	Homo sapiens
691	11719870_turquoise	v-myc avian myelocytomatosis viral oncogene neuroblastoma derived homolog(MYCN)	Homo sapiens
692	11719876_blue	creatine kinase, mitochondrial 2(CKMT2)	Homo sapiens
693	11719877_brown	tRNA phosphotransferase 1(TRPT1)	Homo sapiens
694	11719886_turquoise	interferon induced protein 44(IFI44)	Homo sapiens
695	11719887_turquoise	GRB10 interacting GYF protein 2(GIGYF2)	Homo sapiens
696	11719892_turquoise	vasohibin 1(VASH1)	Homo sapiens
697	11719894_turquoise	family with sequence similarity 91 member A1(FAM91A1)	Homo sapiens
698	11719895_turquoise	family with sequence similarity 91 member A1(FAM91A1)	Homo sapiens

699	11719897_turquoise	family with sequence similarity 91 member A1(FAM91A1)	Homo sapiens
700	11719902_turquoise	PHD finger protein 11(PHF11)	Homo sapiens
701	11719910_turquoise	phosphatidylinositol binding clathrin assembly protein(PICALM)	Homo sapiens
702	11719916_turquoise	interleukin 1 beta(IL1B)	Homo sapiens
703	11719938_turquoise	cullin associated and neddylation dissociated 1(CAND1)	Homo sapiens
704	11719939_turquoise	cullin associated and neddylation dissociated 1(CAND1)	Homo sapiens
705	11719943_turquoise	C-X-C motif chemokine ligand 9(CXCL9)	Homo sapiens
706	11719953_turquoise	FK506 binding protein 15(FKBP15)	Homo sapiens
707	11719972_turquoise	SCO2, cytochrome c oxidase assembly protein(SCO2)	Homo sapiens
708	11719973_turquoise	A-kinase anchoring protein 17A(AKAP17A)	Homo sapiens
709	11719976_turquoise	WAS protein family member 3(WASF3)	Homo sapiens
710	11719980_turquoise	formin like 2(FMNL2)	Homo sapiens
711	11719995_turquoise	transducin like enhancer of split 4(TLE4)	Homo sapiens
712	11719996_turquoise	transducin like enhancer of split 4(TLE4)	Homo sapiens
713	11720014_turquoise	ATM serine/threonine kinase(ATM)	Homo sapiens
714	11720029_turquoise	low density lipoprotein receptor(LDLR)	Homo sapiens
715	11720034_turquoise	caspase 2(CASP2)	Homo sapiens
716	11720035_turquoise	caspase 2(CASP2)	Homo sapiens
717	11720043_turquoise	abhydrolase domain containing 16A(ABHD16A)	Homo sapiens
718	11720044_turquoise	src kinase associated phosphoprotein 2(SKAP2)	Homo sapiens
719	11720063_turquoise	GLI pathogenesis related 2(GLIPR2)	Homo sapiens
720	11720064_turquoise	GLI pathogenesis related 2(GLIPR2)	Homo sapiens
721	11720096_turquoise	LIM domain containing 2(LIMD2)	Homo sapiens
722	11720098_turquoise	centrosomal protein 70(CEP70)	Homo sapiens
723	11720103_turquoise	p21 (RAC1) activated kinase 1(PAK1)	Homo sapiens
724	11720104_turquoise	p21 (RAC1) activated kinase 1(PAK1)	Homo sapiens
725	11720107_turquoise	kinesin family member 2A(KIF2A)	Homo sapiens
726	11720111_turquoise	syntrophin beta 2(SNTB2)	Homo sapiens
727	11720112_turquoise	syntrophin beta 2(SNTB2)	Homo sapiens
728	11720113_turquoise	syntrophin beta 2(SNTB2)	Homo sapiens
729	11720114_brown	ubiquitin conjugating enzyme E2 D4 (putative)(UBE2D4)	Homo sapiens
730	11720115_turquoise	serine/threonine protein kinase 26(STK26)	Homo sapiens
731	11720117_turquoise	signal-induced proliferation-associated 1(SIPA1)	Homo sapiens
732	11720140_brown	Rab interacting lysosomal protein(RILP)	Homo sapiens
733	11720143_turquoise	centriole, cilia and spindle associated protein(CCSAP)	Homo sapiens
734	11720146_turquoise	death associated protein kinase 1(DAPK1)	Homo sapiens
735	11720153_turquoise	nuclear receptor interacting protein 1(NRIP1)	Homo sapiens
736	11720161_turquoise	C-X-C motif chemokine ligand 13(CXCL13)	Homo sapiens
737	11720163_turquoise	vascular endothelial growth factor C(VEGFC)	Homo sapiens
738	11720167_turquoise	selectin L(SELL)	Homo sapiens
739	11720168_turquoise	selectin L(SELL)	Homo sapiens
740	11720171_brown	mitochondrial ribosomal protein S16(MRPS16)	Homo sapiens
741	11720187_turquoise	solute carrier family 27 member 3(SLC27A3)	Homo sapiens
742	11720188_brown	NADH:ubiquinone oxidoreductase subunit A7(NDUFA7)	Homo sapiens
743	11720191_blue	NADH:ubiquinone oxidoreductase complex assembly factor 4(NDUFAF4)	Homo sapiens
744	11720192_brown	NADH:ubiquinone oxidoreductase complex assembly factor 4(NDUFAF4)	Homo sapiens
745	11720193_brown	NADH:ubiquinone oxidoreductase complex assembly factor 4(NDUFAF4)	Homo sapiens
746	11720197_turquoise	translocase of outer mitochondrial membrane 40 like(TOMM40L)	Homo sapiens
747	11720204_turquoise	5'-nucleotidase domain containing 1(NT5DC1)	Homo sapiens
748	11720206_turquoise	claudin 12(CLDN12)	Homo sapiens

749	11720207_turquoise	interleukin 2 receptor subunit gamma(IL2RG)	Homo sapiens
750	11720208_turquoise	interferon regulatory factor 9(IRF9)	Homo sapiens
751	11720209_turquoise	interferon regulatory factor 9(IRF9)	Homo sapiens
752	11720218_turquoise	mitogen-activated protein kinase kinase kinase 3(MAP3K3)	Homo sapiens
753	11720219_turquoise	mitogen-activated protein kinase kinase kinase 3(MAP3K3)	Homo sapiens
754	11720230_turquoise	testin LIM domain protein(TES)	Homo sapiens
755	11720231_turquoise	testin LIM domain protein(TES)	Homo sapiens
756	11720237_turquoise	mitogen-activated protein kinase 9(MAPK9)	Homo sapiens
757	11720238_blue	mitogen-activated protein kinase 9(MAPK9)	Homo sapiens
758	11720243_turquoise	transcobalamin 2(TCN2)	Homo sapiens
759	11720244_turquoise	phospholipase C gamma 2(PLCG2)	Homo sapiens
760	11720247_turquoise	centrosomal protein 55(CEP55)	Homo sapiens
761	11720254_turquoise	cyclin and CBS domain divalent metal cation transport mediator 4(CNNM4)	Homo sapiens
762	11720264_turquoise	activating signal cointegrator 1 complex subunit 3(ASCC3)	Homo sapiens
763	11720280_turquoise	potassium calcium-activated channel subfamily M alpha 1(KCNMA1)	Homo sapiens
764	11720285_turquoise	trafficking kinesin protein 1(TRAK1)	Homo sapiens
765	11720298_turquoise	C-X-C motif chemokine ligand 10(CXCL10)	Homo sapiens
766	11720300_turquoise	Src-like-adaptor(SLA)	Homo sapiens
767	11720301_turquoise	Src-like-adaptor(SLA)	Homo sapiens
768	11720302_turquoise	Src-like-adaptor(SLA)	Homo sapiens
769	11720306_turquoise	stress associated endoplasmic reticulum protein 1(SERP1)	Homo sapiens
770	11720307_turquoise	stress associated endoplasmic reticulum protein 1(SERP1)	Homo sapiens
771	11720308_turquoise	stress associated endoplasmic reticulum protein 1(SERP1)	Homo sapiens
772	11720309_turquoise	stress associated endoplasmic reticulum protein 1(SERP1)	Homo sapiens
773	11720320_turquoise	adenylate kinase 2(AK2)	Homo sapiens
774	11720323_turquoise	adenylate kinase 2(AK2)	Homo sapiens
775	11720328_brown	ATP synthase, H+ transporting, mitochondrial F1 complex, O subunit(ATP5O)	Homo sapiens
776	11720364_turquoise	GIMAP1-GIMAP5 readthrough(GIMAP1-GIMAP5)	Homo sapiens
777	11720367_turquoise	transmembrane protein 2(TMEM2)	Homo sapiens
778	11720373_turquoise	prune exopolyphosphatase(PRUNE1)	Homo sapiens
779	11720380_turquoise	inosine monophosphate dehydrogenase 1(IMPDH1)	Homo sapiens
780	11720382_turquoise	syntaxin 6(STX6)	Homo sapiens
781	11720384_turquoise	syntaxin 6(STX6)	Homo sapiens
782	11720388_turquoise	complement C1q C chain(C1QC)	Homo sapiens
783	11720395_turquoise	nibrin(NBN)	Homo sapiens
784	11720396_turquoise	nibrin(NBN)	Homo sapiens
785	11720398_turquoise	nibrin(NBN)	Homo sapiens
786	11720427_turquoise	ilvB acetolactate synthase like(ILVBL)	Homo sapiens
787	11720430_turquoise	ORMDL sphingolipid biosynthesis regulator 1(ORMDL1)	Homo sapiens
788	11720441_turquoise	olfactomedin like 2B(OLFML2B)	Homo sapiens
789	11720443_turquoise	bromodomain adjacent to zinc finger domain 1A(BAZ1A)	Homo sapiens
790	11720444_turquoise	ER lipid raft associated 1(ERLIN1)	Homo sapiens
791	11720459_turquoise	cell cycle associated protein 1(CAPRIN1)	Homo sapiens
792	11720460_turquoise	F11 receptor(F11R)	Homo sapiens
793	11720464_turquoise	F11 receptor(F11R)	Homo sapiens
794	11720487_brown	heme binding protein 2(HEBP2)	Homo sapiens
795	11720493_turquoise	MOB kinase activator 3A(MOB3A)	Homo sapiens
796	11720496_turquoise	granzyme A(GZMA)	Homo sapiens
797	11720501_turquoise	ubiquitination factor E4B(UBE4B)	Homo sapiens
798	11720502_turquoise	ubiquitination factor E4B(UBE4B)	Homo sapiens

799	11720504_	turquoise	RAB6B, member RAS oncogene family(RAB6B)	Homo sapiens
800	11720510_	turquoise	apolipoprotein B mRNA editing enzyme catalytic subunit 3G(APOBEC3G)	Homo sapiens
801	11720511_	turquoise	calcium regulated heat stable protein 1(CARHSP1)	Homo sapiens
802	11720535_	brown	methylmalonic aciduria (cobalamin deficiency) cblB type(MMAB)	Homo sapiens
803	11720537_	turquoise	methylmalonic aciduria (cobalamin deficiency) cblB type(MMAB)	Homo sapiens
804	11720538_	turquoise	feline leukemia virus subgroup C cellular receptor family member 2(FLVCR2)	Homo sapiens
805	11720551_	turquoise	intermediate filament family orphan 1(IFFO1)	Homo sapiens
806	11720552_	turquoise	intermediate filament family orphan 1(IFFO1)	Homo sapiens
807	11720554_	turquoise	Rho/Rac guanine nucleotide exchange factor 18(ARHGEF18)	Homo sapiens
808	11720566_	turquoise	branched chain amino acid transaminase 1(BCAT1)	Homo sapiens
809	11720570_	turquoise	jade family PHD finger 2(JADE2)	Homo sapiens
810	11720573_	turquoise	ATPase phospholipid transporting 8B1(ATP8B1)	Homo sapiens
811	11720574_	turquoise	ATPase phospholipid transporting 8B1(ATP8B1)	Homo sapiens
812	11720587_	turquoise	structural maintenance of chromosomes flexible hinge domain containing 1(SMCHD1)	Homo sapiens
813	11720602_	turquoise	synaptotagmin 11(SYT11)	Homo sapiens
814	11720609_	turquoise	prohibitin(PHB)	Homo sapiens
815	11720610_	brown	prohibitin(PHB)	Homo sapiens
816	11720621_	turquoise	ATP citrate lyase(ACLY)	Homo sapiens
817	11720623_	turquoise	myeloid differentiation primary response 88(MYD88)	Homo sapiens
818	11720624_	turquoise	myeloid differentiation primary response 88(MYD88)	Homo sapiens
819	11720630_	brown	small nuclear ribonucleoprotein U11/U12 subunit 25(SNRNP25)	Homo sapiens
820	11720631_	brown	succinate dehydrogenase complex iron sulfur subunit B(SDHB)	Homo sapiens
821	11720632_	brown	succinate dehydrogenase complex iron sulfur subunit B(SDHB)	Homo sapiens
822	11720635_	brown	ATP synthase, H+ transporting, mitochondrial F1 complex, delta subunit(ATP5D)	Homo sapiens
823	11720644_	turquoise	platelet and endothelial cell adhesion molecule 1(PECAM1)	Homo sapiens
824	11720646_	turquoise	serine palmitoyltransferase long chain base subunit 2(SPTLC2)	Homo sapiens
825	11720657_	turquoise	major histocompatibility complex, class II, DR beta 5(HLA-DRB5)	Homo sapiens
826	11720672_	turquoise	protein phosphatase, Mg2+/Mn2+ dependent 1A(PPM1A)	Homo sapiens
827	11720673_	blue	protein phosphatase, Mg2+/Mn2+ dependent 1A(PPM1A)	Homo sapiens
828	11720674_	blue	protein phosphatase, Mg2+/Mn2+ dependent 1A(PPM1A)	Homo sapiens
829	11720679_	turquoise	CKLF-CMTM1 readthrough(CKLF-CMTM1)	Homo sapiens
830	11720680_	turquoise	diacylglycerol kinase alpha(DGKA)	Homo sapiens
831	11720694_	turquoise	HEN1 methyltransferase homolog 1(HENMT1)	Homo sapiens
832	11720695_	turquoise	chromosome 1 open reading frame 54(C1orf54)	Homo sapiens
833	11720725_	turquoise	zyg-11 family member B, cell cycle regulator(ZYG11B)	Homo sapiens
834	11720747_	turquoise	adenylate cyclase 7(ADCY7)	Homo sapiens
835	11720751_	turquoise	inositol polyphosphate-1-phosphatase(INPP1)	Homo sapiens
836	11720754_	turquoise	actin beta(ACTB)	Homo sapiens
837	11720755_	turquoise	signal transducer and activator of transcription 3(STAT3)	Homo sapiens
838	11720756_	turquoise	signal transducer and activator of transcription 3(STAT3)	Homo sapiens
839	11720763_	turquoise	selectin P ligand(SELPLG)	Homo sapiens
840	11720769_	turquoise	peptidylprolyl isomerase F(PPIF)	Homo sapiens
841	11720770_	blue	peptidylprolyl isomerase F(PPIF)	Homo sapiens
842	11720771_	blue	peptidylprolyl isomerase F(PPIF)	Homo sapiens
843	11720773_	brown	DnaJ heat shock protein family (Hsp40) member C19(DNAJC19)	Homo sapiens
844	11720774_	brown	DnaJ heat shock protein family (Hsp40) member C19(DNAJC19)	Homo sapiens
845	11720787_	blue	dynein cytoplasmic 1 light intermediate chain 1(DYNC1LI1)	Homo sapiens
846	11720793_	turquoise	acetoacetyl-CoA synthetase(AACS)	Homo sapiens
847	11720798_	turquoise	RAB8B, member RAS oncogene family(RAB8B)	Homo sapiens
848	11720799_	turquoise	RAB8B, member RAS oncogene family(RAB8B)	Homo sapiens

849	11720800_ turquoise	RAB8B, member RAS oncogene family(RAB8B)	Homo sapiens
850	11720802_ turquoise	bridging integrator 3(BIN3)	Homo sapiens
851	11720809_ brown	phosphoglycerate dehydrogenase(PHGDH)	Homo sapiens
852	11720821_ brown	FUN14 domain containing 2(FUNDC2)	Homo sapiens
853	11720827_ turquoise	centromere protein W(CENPW)	Homo sapiens
854	11720832_ turquoise	SRY-box 18(SOX18)	Homo sapiens
855	11720842_ turquoise	ras homolog family member D(RHOD)	Homo sapiens
856	11720847_ turquoise	PHD finger protein 6(PHF6)	Homo sapiens
857	11720859_ turquoise	abhydrolase domain containing 3(ABHD3)	Homo sapiens
858	11720860_ turquoise	Rho guanine nucleotide exchange factor 3(ARHGEF3)	Homo sapiens
859	11720861_ turquoise	Rho guanine nucleotide exchange factor 3(ARHGEF3)	Homo sapiens
860	11720863_ brown	patatin like phospholipase domain containing 4(PNPLA4)	Homo sapiens
861	11720867_ turquoise	placenta specific 8(PLAC8)	Homo sapiens
862	11720884_ brown	hydroxymethylbilane synthase(HMBS)	Homo sapiens
863	11720891_ turquoise	ubiquitin specific peptidase 20(USP20)	Homo sapiens
864	11720893_ turquoise	SOS Ras/Rac guanine nucleotide exchange factor 1(SOS1)	Homo sapiens
865	11720907_ turquoise	semaphorin 3F(SEMA3F)	Homo sapiens
866	11720909_ turquoise	transmembrane protein 44(TMEM44)	Homo sapiens
867	11720910_ turquoise	neural EGFL like 2(NELL2)	Homo sapiens
868	11720922_ turquoise	HLA complex P5 (non-protein coding)(HCP5)	Homo sapiens
869	11720923_ turquoise	HLA complex P5 (non-protein coding)(HCP5)	Homo sapiens
870	11720943_ turquoise	regulator of G-protein signaling 16(RGS16)	Homo sapiens
871	11720944_ turquoise	regulator of G-protein signaling 16(RGS16)	Homo sapiens
872	11720963_ turquoise	deoxycytidine kinase(DCK)	Homo sapiens
873	11720964_ turquoise	deoxycytidine kinase(DCK)	Homo sapiens
874	11720965_ turquoise	deoxycytidine kinase(DCK)	Homo sapiens
875	11720966_ turquoise	deoxycytidine kinase(DCK)	Homo sapiens
876	11720970_ turquoise	topoisomerase (DNA) II alpha(TOP2A)	Homo sapiens
877	11720971_ turquoise	topoisomerase (DNA) II alpha(TOP2A)	Homo sapiens
878	11720972_ turquoise	topoisomerase (DNA) II alpha(TOP2A)	Homo sapiens
879	11720973_ turquoise	ankyrin repeat and SOCS box containing 8(ASB8)	Homo sapiens
880	11720974_ brown	ankyrin repeat and SOCS box containing 8(ASB8)	Homo sapiens
881	11720977_ turquoise	tryptophanyl tRNA synthetase 2, mitochondrial(WARS2)	Homo sapiens
882	11720982_ turquoise	protein tyrosine kinase 2 beta(PTK2B)	Homo sapiens
883	11720989_ turquoise	solute carrier family 7 member 7(SLC7A7)	Homo sapiens
884	11720994_ turquoise	C-C motif chemokine ligand 3(CCL3)	Homo sapiens
885	11720998_ turquoise	bridging integrator 2(BIN2)	Homo sapiens
886	11720999_ turquoise	bridging integrator 2(BIN2)	Homo sapiens
887	11721027_ turquoise	cyclin G associated kinase(GAK)	Homo sapiens
888	11721028_ blue	malonyl-CoA decarboxylase(MLYCD)	Homo sapiens
889	11721029_ blue	phosphatidylinositol-4-phosphate 5-kinase type 1 beta(PIP5K1B)	Homo sapiens
890	11721030_ blue	phosphatidylinositol-4-phosphate 5-kinase type 1 beta(PIP5K1B)	Homo sapiens
891	11721043_ turquoise	exoribonuclease 1(ERI1)	Homo sapiens
892	11721044_ turquoise	exoribonuclease 1(ERI1)	Homo sapiens
893	11721053_ turquoise	kelch like family member 42(KLHL42)	Homo sapiens
894	11721054_ turquoise	kelch like family member 42(KLHL42)	Homo sapiens
895	11721055_ turquoise	protein kinase, X-linked(PRKX)	Homo sapiens
896	11721056_ turquoise	protein kinase, X-linked(PRKX)	Homo sapiens
897	11721061_ turquoise	IQ motif containing GTPase activating protein 2(IQGAP2)	Homo sapiens
898	11721077_ brown	esterase D(ESD)	Homo sapiens

899	11721096_ turquoise	thioredoxin related transmembrane protein 1(TMX1)	Homo sapiens
900	11721097_ turquoise	thioredoxin related transmembrane protein 1(TMX1)	Homo sapiens
901	11721098_ turquoise	thioredoxin related transmembrane protein 1(TMX1)	Homo sapiens
902	11721099_ turquoise	complement C3a receptor 1(C3AR1)	Homo sapiens
903	11721111_ brown	acyl-CoA thioesterase 13(ACOT13)	Homo sapiens
904	11721120_ turquoise	nudix hydrolase 5(NUDT5)	Homo sapiens
905	11721125_ turquoise	signal peptide peptidase like 2A(SPPL2A)	Homo sapiens
906	11721140_ turquoise	transcription elongation factor A3(TCEA3)	Homo sapiens
907	11721141_ turquoise	transcription elongation factor A3(TCEA3)	Homo sapiens
908	11721143_ turquoise	marker of proliferation Ki-67(MKI67)	Homo sapiens
909	11721145_ turquoise	marker of proliferation Ki-67(MKI67)	Homo sapiens
910	11721147_ turquoise	synaptogyrin 1(SYNGR1)	Homo sapiens
911	11721149_ turquoise	LysM domain containing 2(LYSMD2)	Homo sapiens
912	11721157_ turquoise	nth like DNA glycosylase 1(NTHL1)	Homo sapiens
913	11721163_ turquoise	KH and NYN domain containing(KHNYN)	Homo sapiens
914	11721169_ turquoise	PYD and CARD domain containing(PYCARD)	Homo sapiens
915	11721184_ turquoise	tetraspanin 33(TSPAN33)	Homo sapiens
916	11721202_ turquoise	ribonuclease A family member k6(RNASE6)	Homo sapiens
917	11721208_ turquoise	intermediate filament family orphan 2(IFFO2)	Homo sapiens
918	11721223_ turquoise	acyl-CoA oxidase 2(ACOX2)	Homo sapiens
919	11721243_ turquoise	leucine aminopeptidase 3(LAP3)	Homo sapiens
920	11721248_ turquoise	G protein subunit gamma 2(GNG2)	Homo sapiens
921	11721265_ brown	glutamyl-tRNA amidotransferase subunit B(GATB)	Homo sapiens
922	11721272_ turquoise	leucine rich repeat containing 59(LRRC59)	Homo sapiens
923	11721296_ brown	NADH:ubiquinone oxidoreductase subunit B1(NDUFB1)	Homo sapiens
924	11721297_ turquoise	ABRA C-terminal like(ABRACL)	Homo sapiens
925	11721302_ turquoise	C-type lectin domain family 2 member B(CLEC2B)	Homo sapiens
926	11721303_ turquoise	C-type lectin domain family 2 member B(CLEC2B)	Homo sapiens
927	11721313_ turquoise	extended synaptotagmin 2(ESYT2)	Homo sapiens
928	11721346_ turquoise	macrophage expressed 1(MPEG1)	Homo sapiens
929	11721347_ turquoise	macrophage expressed 1(MPEG1)	Homo sapiens
930	11721349_ turquoise	ubiquitin specific peptidase 18(USP18)	Homo sapiens
931	11721366_ turquoise	tripartite motif containing 47(TRIM47)	Homo sapiens
932	11721380_ turquoise	N-acetylglucosamine-1-phosphate transferase alpha and beta subunits(GNPTAB)	Homo sapiens
933	11721390_ turquoise	HtrA serine peptidase 3(HTRA3)	Homo sapiens
934	11721408_ turquoise	polyamine oxidase(PAOX)	Homo sapiens
935	11721432_ turquoise	microtubule associated protein 1S(MAP1S)	Homo sapiens
936	11721454_ turquoise	serpin family A member 1(SERPINA1)	Homo sapiens
937	11721455_ turquoise	serpin family A member 1(SERPINA1)	Homo sapiens
938	11721456_ turquoise	serpin family A member 1(SERPINA1)	Homo sapiens
939	11721460_ turquoise	TAP binding protein like(TAPBPL)	Homo sapiens
940	11721461_ brown	COP9 signalosome subunit 5(COPS5)	Homo sapiens
941	11721472_ blue	holocytochrome c synthase(HCCS)	Homo sapiens
942	11721473_ brown	holocytochrome c synthase(HCCS)	Homo sapiens
943	11721491_ turquoise	fructose-bisphosphatase 1(FBP1)	Homo sapiens
944	11721493_ turquoise	sulfatase 2(SULF2)	Homo sapiens
945	11721504_ turquoise	CKLF-CMTM1 readthrough(CKLF-CMTM1)	Homo sapiens
946	11721512_ turquoise	translocase of inner mitochondrial membrane 21(TIMM21)	Homo sapiens
947	11721554_ turquoise	collagen beta(1-O)galactosyltransferase 1(COLGALT1)	Homo sapiens
948	11721557_ turquoise	ATP binding cassette subfamily A member 8(ABCA8)	Homo sapiens

949	11721570_turquoise	family with sequence similarity 173 member B(FAM173B)	Homo sapiens
950	11721573_turquoise	activin A receptor like type 1(ACVRL1)	Homo sapiens
951	11721574_turquoise	E74 like ETS transcription factor 4(ELF4)	Homo sapiens
952	11721577_turquoise	tumor necrosis factor(TNF)	Homo sapiens
953	11721582_turquoise	lymphoid restricted membrane protein(LRMP)	Homo sapiens
954	11721583_turquoise	lymphoid restricted membrane protein(LRMP)	Homo sapiens
955	11721587_turquoise	TNF receptor associated factor 1(TRAF1)	Homo sapiens
956	11721588_turquoise	TNF receptor associated factor 1(TRAF1)	Homo sapiens
957	11721590_turquoise	solute carrier family 17 member 9(SLC17A9)	Homo sapiens
958	11721609_turquoise	poly(A) binding protein cytoplasmic 1 like(PABPC1L)	Homo sapiens
959	11721615_turquoise	thymocyte selection associated family member 2(THEMIS2)	Homo sapiens
960	11721623_turquoise	mesenchyme homeobox 1(MEOX1)	Homo sapiens
961	11721626_turquoise	septin 6(SEPT6)	Homo sapiens
962	11721627_turquoise	septin 6(SEPT6)	Homo sapiens
963	11721628_turquoise	septin 6(SEPT6)	Homo sapiens
964	11721629_turquoise	MAF bZIP transcription factor B(MAFB)	Homo sapiens
965	11721630_turquoise	MAF bZIP transcription factor B(MAFB)	Homo sapiens
966	11721651_turquoise	cadherin 5(CDH5)	Homo sapiens
967	11721661_turquoise	thromboxane A synthase 1(TBXAS1)	Homo sapiens
968	11721673_brown	glutaredoxin 5(GLRX5)	Homo sapiens
969	11721674_brown	glutaredoxin 5(GLRX5)	Homo sapiens
970	11721675_brown	glutaredoxin 5(GLRX5)	Homo sapiens
971	11721683_brown	mitochondrial ribosomal protein S9(MRPS9)	Homo sapiens
972	11721685_turquoise	C-C motif chemokine ligand 19(CCL19)	Homo sapiens
973	11721695_turquoise	dual specificity phosphatase 2(DUSP2)	Homo sapiens
974	11721702_turquoise	CKLF like MARVEL transmembrane domain containing 7(CMTM7)	Homo sapiens
975	11721704_turquoise	cysteine rich protein 1(CRIP1)	Homo sapiens
976	11721706_grey	glycerol-3-phosphate acyltransferase, mitochondrial(GPAM)	Homo sapiens
977	11721719_turquoise	N-acylethanolamine acid amidase(NAAA)	Homo sapiens
978	11721722_turquoise	MSH5-SAPCD1 readthrough (NMD candidate)(MSH5-SAPCD1)	Homo sapiens
979	11721728_turquoise	Rho GTPase activating protein 30(ARHGAP30)	Homo sapiens
980	11721733_turquoise	GTP cyclohydrolase 1(GCH1)	Homo sapiens
981	11721734_turquoise	GTP cyclohydrolase 1(GCH1)	Homo sapiens
982	11721755_turquoise	ALG13, UDP-N-acetylglucosaminyltransferase subunit(ALG13)	Homo sapiens
983	11721760_turquoise	tRNA splicing endonuclease subunit 54(TSEN54)	Homo sapiens
984	11721762_turquoise	phosphoinositide-3-kinase regulatory subunit 5(PIK3R5)	Homo sapiens
985	11721773_turquoise	WSC domain containing 1(WSCD1)	Homo sapiens
986	11721780_turquoise	LLGL2, scribble cell polarity complex component(LLGL2)	Homo sapiens
987	11721804_turquoise	BUB3, mitotic checkpoint protein(BUB3)	Homo sapiens
988	11721810_turquoise	Pim-2 proto-oncogene, serine/threonine kinase(PIM2)	Homo sapiens
989	11721815_turquoise	Fc fragment of IgM receptor(FCMR)	Homo sapiens
990	11721816_turquoise	Fc fragment of IgM receptor(FCMR)	Homo sapiens
991	11721827_turquoise	zwilch kinetochore protein(ZWILCH)	Homo sapiens
992	11721828_turquoise	zwilch kinetochore protein(ZWILCH)	Homo sapiens
993	11721836_brown	transmembrane protein 14B(TMEM14B)	Homo sapiens
994	11721838_turquoise	GLI pathogenesis related 1(GLIPR1)	Homo sapiens
995	11721839_turquoise	GLI pathogenesis related 1(GLIPR1)	Homo sapiens
996	11721840_turquoise	GLI pathogenesis related 1(GLIPR1)	Homo sapiens
997	11721841_turquoise	GLI pathogenesis related 1(GLIPR1)	Homo sapiens
998	11721842_turquoise	GLI pathogenesis related 1(GLIPR1)	Homo sapiens

999	11721843_ turquoise	atlastin GTPase 3(ATL3)	Homo sapiens
1000	11721851_ brown	coiled-coil-helix-coiled-coil-helix domain containing 6(CHCHD6)	Homo sapiens
1001	11721859_ turquoise	mitochondrial intermediate peptidase(MIPEP)	Homo sapiens
1002	11721860_ turquoise	syntaxin 12(STX12)	Homo sapiens
1003	11721861_ turquoise	syntaxin 12(STX12)	Homo sapiens
1004	11721872_ turquoise	family with sequence similarity 110 member A(FAM110A)	Homo sapiens
1005	11721873_ turquoise	interferon induced protein with tetratricopeptide repeats 2(IFIT2)	Homo sapiens
1006	11721874_ turquoise	interferon induced protein with tetratricopeptide repeats 2(IFIT2)	Homo sapiens
1007	11721879_ turquoise	AT-rich interaction domain 4B(ARID4B)	Homo sapiens
1008	11721880_ turquoise	AT-rich interaction domain 4B(ARID4B)	Homo sapiens
1009	11721885_ turquoise	cell division cycle 42(CDC42)	Homo sapiens
1010	11721897_ turquoise	DExH-box helicase 58(DHX58)	Homo sapiens
1011	11721900_ turquoise	ficolin 1(FCN1)	Homo sapiens
1012	11721906_ turquoise	proteasome subunit alpha 4(PSMA4)	Homo sapiens
1013	11721907_ turquoise	sphingosine-1-phosphate phosphatase 1(SGPP1)	Homo sapiens
1014	11721922_ turquoise	protein kinase C beta(PRKCB)	Homo sapiens
1015	11721923_ turquoise	protein kinase C beta(PRKCB)	Homo sapiens
1016	11721924_ turquoise	protein kinase C beta(PRKCB)	Homo sapiens
1017	11721927_ turquoise	transmembrane channel like 8(TMC8)	Homo sapiens
1018	11721935_ turquoise	DnaJ heat shock protein family (Hsp40) member C27(DNAJC27)	Homo sapiens
1019	11721954_ turquoise	ring finger protein 41(RNF41)	Homo sapiens
1020	11721960_ turquoise	sorting nexin family member 30(SNX30)	Homo sapiens
1021	11721974_ turquoise	sulfite oxidase(SUOX)	Homo sapiens
1022	11721988_ turquoise	coiled-coil domain containing 191(CCDC191)	Homo sapiens
1023	11721994_ turquoise	ubiquitin conjugating enzyme E2 L6(UBE2L6)	Homo sapiens
1024	11721996_ turquoise	poly(ADP-ribose) polymerase family member 14(PARP14)	Homo sapiens
1025	11721997_ turquoise	poly(ADP-ribose) polymerase family member 14(PARP14)	Homo sapiens
1026	11721998_ turquoise	poly(ADP-ribose) polymerase family member 14(PARP14)	Homo sapiens
1027	11722003_ turquoise	cytochrome c, somatic(CYCS)	Homo sapiens
1028	11722004_ blue	cytochrome c, somatic(CYCS)	Homo sapiens
1029	11722005_ turquoise	nuclear autoantigenic sperm protein(NASP)	Homo sapiens
1030	11722009_ turquoise	chloride intracellular channel 1(CLIC1)	Homo sapiens
1031	11722011_ turquoise	GTPase, IMAP family member 4(GIMAP4)	Homo sapiens
1032	11722012_ turquoise	GTPase, IMAP family member 4(GIMAP4)	Homo sapiens
1033	11722028_ blue	ubiquitin conjugating enzyme E2 G1(UBE2G1)	Homo sapiens
1034	11722038_ turquoise	FES proto-oncogene, tyrosine kinase(FES)	Homo sapiens
1035	11722039_ turquoise	FES proto-oncogene, tyrosine kinase(FES)	Homo sapiens
1036	11722040_ brown	eukaryotic translation initiation factor 2B subunit gamma(EIF2B3)	Homo sapiens
1037	11722049_ turquoise	dual specificity phosphatase 6(DUSP6)	Homo sapiens
1038	11722072_ turquoise	mesoderm development candidate 1(MESDC1)	Homo sapiens
1039	11722086_ turquoise	septin 1(SEPT1)	Homo sapiens
1040	11722089_ turquoise	echinoderm microtubule associated protein like 3(EML3)	Homo sapiens
1041	11722095_ turquoise	kinesin family member 21B(KIF21B)	Homo sapiens
1042	11722120_ turquoise	kinetochore associated 1(KNTC1)	Homo sapiens
1043	11722130_ turquoise	enoyl-CoA hydratase domain containing 2(ECHDC2)	Homo sapiens
1044	11722134_ turquoise	TNF receptor superfamily member 25(TNFRSF25)	Homo sapiens
1045	11722141_ turquoise	RELB proto-oncogene, NF-kB subunit(RELB)	Homo sapiens
1046	11722143_ turquoise	tetratricopeptide repeat and ankyrin repeat containing 1(TRANK1)	Homo sapiens
1047	11722147_ turquoise	poly(A) binding protein interacting protein 2B(PAIP2B)	Homo sapiens
1048	11722152_ turquoise	eukaryotic translation initiation factor 2 alpha kinase 3(EIF2AK3)	Homo sapiens

1049	11722170_ turquoise	coronin 7(CORO7)	Homo sapiens
1050	11722173_ blue	ubiquinol-cytochrome c reductase complex assembly factor 1(UQCC1)	Homo sapiens
1051	11722174_ turquoise	ubiquinol-cytochrome c reductase complex assembly factor 1(UQCC1)	Homo sapiens
1052	11722183_ turquoise	ATPase H+ transporting V1 subunit C1(ATP6V1C1)	Homo sapiens
1053	11722193_ turquoise	chromosome 12 open reading frame 75(C12orf75)	Homo sapiens
1054	11722194_ brown	microRNA 4691(MIR4691)	Homo sapiens
1055	11722225_ turquoise	ADAM metallopeptidase domain 8(ADAM8)	Homo sapiens
1056	11722226_ brown	succinate dehydrogenase complex assembly factor 3(SDHAF3)	Homo sapiens
1057	11722228_ turquoise	DnaJ heat shock protein family (Hsp40) member C30(DNAJC30)	Homo sapiens
1058	11722247_ turquoise	Ras association domain family member 1(RASSF1)	Homo sapiens
1059	11722248_ turquoise	Ras association domain family member 1(RASSF1)	Homo sapiens
1060	11722262_ turquoise	ER degradation enhancing alpha-mannosidase like protein 1(EDEM1)	Homo sapiens
1061	11722269_ turquoise	SAM and SH3 domain containing 3(SASH3)	Homo sapiens
1062	11722270_ turquoise	SAM and SH3 domain containing 3(SASH3)	Homo sapiens
1063	11722286_ turquoise	solute carrier family 30 member 7(SLC30A7)	Homo sapiens
1064	11722287_ turquoise	solute carrier family 30 member 7(SLC30A7)	Homo sapiens
1065	11722298_ turquoise	RAB29, member RAS oncogene family(RAB29)	Homo sapiens
1066	11722299_ turquoise	RAB29, member RAS oncogene family(RAB29)	Homo sapiens
1067	11722300_ turquoise	ETS proto-oncogene 1, transcription factor(ETS1)	Homo sapiens
1068	11722302_ turquoise	LRR binding FLII interacting protein 1(LRRFIP1)	Homo sapiens
1069	11722303_ turquoise	LRR binding FLII interacting protein 1(LRRFIP1)	Homo sapiens
1070	11722304_ turquoise	LRR binding FLII interacting protein 1(LRRFIP1)	Homo sapiens
1071	11722313_ turquoise	Rap guanine nucleotide exchange factor 6(RAPGEF6)	Homo sapiens
1072	11722314_ turquoise	Rap guanine nucleotide exchange factor 6(RAPGEF6)	Homo sapiens
1073	11722321_ turquoise	hematopoietically expressed homeobox(HHEX)	Homo sapiens
1074	11722333_ turquoise	fibronectin type III domain containing 5(FNDC5)	Homo sapiens
1075	11722334_ turquoise	fibronectin type III domain containing 5(FNDC5)	Homo sapiens
1076	11722335_ turquoise	fibronectin type III domain containing 5(FNDC5)	Homo sapiens
1077	11722338_ brown	peroxisomal biogenesis factor 7(PEX7)	Homo sapiens
1078	11722349_ turquoise	Lck interacting transmembrane adaptor 1(LIME1)	Homo sapiens
1079	11722355_ turquoise	DAB2, clathrin adaptor protein(DAB2)	Homo sapiens
1080	11722356_ turquoise	DAB2, clathrin adaptor protein(DAB2)	Homo sapiens
1081	11722364_ blue	cytochrome c oxidase subunit 6A2(COX6A2)	Homo sapiens
1082	11722368_ turquoise	tripartite motif containing 22(TRIM22)	Homo sapiens
1083	11722369_ turquoise	tripartite motif containing 22(TRIM22)	Homo sapiens
1084	11722370_ turquoise	tripartite motif containing 22(TRIM22)	Homo sapiens
1085	11722371_ turquoise	tripartite motif containing 22(TRIM22)	Homo sapiens
1086	11722403_ turquoise	pleckstrin homology domain containing O1(PLEKHO1)	Homo sapiens
1087	11722417_ turquoise	La ribonucleoprotein domain family member 6(LARP6)	Homo sapiens
1088	11722418_ turquoise	UTP3, small subunit processome component homolog (S. cerevisiae)(UTP3)	Homo sapiens
1089	11722419_ turquoise	UTP3, small subunit processome component homolog (S. cerevisiae)(UTP3)	Homo sapiens
1090	11722425_ turquoise	neural precursor cell expressed, developmentally down-regulated 4-like, E3 ubiquitin pı	Homo sapiens
1091	11722426_ turquoise	fibrinogen like 2(FGL2)	Homo sapiens
1092	11722427_ turquoise	fibrinogen like 2(FGL2)	Homo sapiens
1093	11722446_ turquoise	sirtuin 3(SIRT3)	Homo sapiens
1094	11722447_ turquoise	sirtuin 3(SIRT3)	Homo sapiens
1095	11722449_ turquoise	thymidine phosphorylase(TYMP)	Homo sapiens
1096	11722458_ turquoise	transmembrane protein 206(TMEM206)	Homo sapiens
1097	11722462_ turquoise	tankyrase 2(TNKS2)	Homo sapiens
1098	11722471_ turquoise	paternally expressed 3(PEG3)	Homo sapiens

1099	11722472_	turquoise	paternally expressed 3(PEG3)	Homo sapiens
1100	11722474_	turquoise	diacylglycerol kinase theta(DGKQ)	Homo sapiens
1101	11722480_	turquoise	FGR proto-oncogene, Src family tyrosine kinase(FGR)	Homo sapiens
1102	11722481_	turquoise	mitochondrial fission factor(MFF)	Homo sapiens
1103	11722482_	blue	mitochondrial fission factor(MFF)	Homo sapiens
1104	11722501_	turquoise	p21 (RAC1) activated kinase 6(PAK6)	Homo sapiens
1105	11722502_	turquoise	p21 (RAC1) activated kinase 6(PAK6)	Homo sapiens
1106	11722512_	turquoise	SP110 nuclear body protein(SP110)	Homo sapiens
1107	11722535_	turquoise	cyclin Y like 1(CCNYL1)	Homo sapiens
1108	11722538_	turquoise	transmembrane and coiled-coil domains 6(TMCO6)	Homo sapiens
1109	11722555_	blue	hydroxyacyl-CoA dehydrogenase(HADH)	Homo sapiens
1110	11722571_	turquoise	cyclin A2(CCNA2)	Homo sapiens
1111	11722573_	turquoise	endoplasmic reticulum aminopeptidase 2(ERAP2)	Homo sapiens
1112	11722574_	turquoise	adaptor related protein complex 1 gamma 1 subunit(AP1G1)	Homo sapiens
1113	11722577_	turquoise	prolyl 3-hydroxylase 1(P3H1)	Homo sapiens
1114	11722609_	turquoise	CDC42 small effector 1(CDC42SE1)	Homo sapiens
1115	11722620_	turquoise	aquaporin 4(AQP4)	Homo sapiens
1116	11722635_	turquoise	interleukin 2 receptor subunit beta(IL2RB)	Homo sapiens
1117	11722660_	turquoise	nuclear factor, erythroid 2 like 3(NFE2L3)	Homo sapiens
1118	11722679_	turquoise	poly(ADP-ribose) polymerase family member 8(PARP8)	Homo sapiens
1119	11722680_	turquoise	hematopoietic cell signal transducer(HCST)	Homo sapiens
1120	11722697_	turquoise	SFI1 centrin binding protein(SFI1)	Homo sapiens
1121	11722705_	turquoise	UDP-GlcNAc:betaGal beta-1,3-N-acetylglucosaminyltransferase 5(B3GNT5)	Homo sapiens
1122	11722708_	turquoise	coiled-coil serine rich protein 2(CCSER2)	Homo sapiens
1123	11722721_	turquoise	centromere protein V(CENPV)	Homo sapiens
1124	11722725_	turquoise	colony stimulating factor 2 receptor beta common subunit(CSF2RB)	Homo sapiens
1125	11722728_	turquoise	early growth response 2(EGR2)	Homo sapiens
1126	11722747_	turquoise	hook microtubule tethering protein 3(HOOK3)	Homo sapiens
1127	11722765_	turquoise	GIT ArfGAP 2(GIT2)	Homo sapiens
1128	11722768_	turquoise	Rap guanine nucleotide exchange factor 5(RAPGEF5)	Homo sapiens
1129	11722778_	turquoise	ectonucleotide pyrophosphatase/phosphodiesterase 2(ENPP2)	Homo sapiens
1130	11722794_	turquoise	glycerophosphodiester phosphodiesterase 1(GDE1)	Homo sapiens
1131	11722795_	blue	glycerophosphodiester phosphodiesterase 1(GDE1)	Homo sapiens
1132	11722806_	turquoise	RAB35, member RAS oncogene family(RAB35)	Homo sapiens
1133	11722807_	turquoise	WD repeat domain 54(WDR54)	Homo sapiens
1134	11722818_	turquoise	gamma-glutamyl hydrolase(GGH)	Homo sapiens
1135	11722826_	turquoise	non-SMC condensin I complex subunit G(NCAPG)	Homo sapiens
1136	11722841_	blue	transcription factor B2, mitochondrial(TFB2M)	Homo sapiens
1137	11722850_	turquoise	baculoviral IAP repeat containing 3(BIRC3)	Homo sapiens
1138	11722851_	turquoise	baculoviral IAP repeat containing 3(BIRC3)	Homo sapiens
1139	11722852_	turquoise	baculoviral IAP repeat containing 3(BIRC3)	Homo sapiens
1140	11722874_	turquoise	male-specific lethal 3 homolog (Drosophila)(MSL3)	Homo sapiens
1141	11722880_	turquoise	PHD finger protein 19(PHF19)	Homo sapiens
1142	11722886_	turquoise	embigin(EMB)	Homo sapiens
1143	11722887_	turquoise	embigin(EMB)	Homo sapiens
1144	11722888_	turquoise	embigin(EMB)	Homo sapiens
1145	11722904_	turquoise	TBC1 domain family member 9(TBC1D9)	Homo sapiens
1146	11722911_	blue	ubiquitin specific peptidase 13 (isopeptidase T-3)(USP13)	Homo sapiens
1147	11722912_	blue	ubiquitin specific peptidase 13 (isopeptidase T-3)(USP13)	Homo sapiens
1148	11722913_	blue	ubiquitin specific peptidase 13 (isopeptidase T-3)(USP13)	Homo sapiens

1149	11722923_turquoise	family with sequence similarity 122B(FAM122B)	Homo sapiens
1150	11722928_turquoise	microRNA 4656(MIR4656)	Homo sapiens
1151	11722940_turquoise	SLIT-ROBO Rho GTPase activating protein 2(SRGAP2)	Homo sapiens
1152	11722960_turquoise	cytochrome b561 family member D1(CYB561D1)	Homo sapiens
1153	11722964_turquoise	Alport syndrome, mental retardation, midface hypoplasia and elliptocytosis chromoson	Homo sapiens
1154	11722979_turquoise	coagulation factor VIII(F8)	Homo sapiens
1155	11722982_turquoise	lysosomal trafficking regulator(LYST)	Homo sapiens
1156	11723006_turquoise	S100 calcium binding protein A4(S100A4)	Homo sapiens
1157	11723010_turquoise	kinesin family member 20A(KIF20A)	Homo sapiens
1158	11723016_turquoise	histocompatibility minor 13(HM13)	Homo sapiens
1159	11723020_turquoise	receptor interacting serine/threonine kinase 3(RIPK3)	Homo sapiens
1160	11723021_turquoise	branched chain keto acid dehydrogenase E1 subunit beta(BCKDHB)	Homo sapiens
1161	11723025_turquoise	family with sequence similarity 26 member F(FAM26F)	Homo sapiens
1162	11723026_turquoise	family with sequence similarity 26 member F(FAM26F)	Homo sapiens
1163	11723046_brown	mitochondrial assembly of ribosomal large subunit 1(MALSU1)	Homo sapiens
1164	11723048_turquoise	C-X3-C motif chemokine receptor 1(CX3CR1)	Homo sapiens
1165	11723050_turquoise	heat shock protein family A (Hsp70) member 6(HSPA6)	Homo sapiens
1166	11723056_brown	mitochondrial ribosomal protein L33(MRPL33)	Homo sapiens
1167	11723057_brown	mitochondrial ribosomal protein L33(MRPL33)	Homo sapiens
1168	11723058_turquoise	transporter 2, ATP binding cassette subfamily B member(TAP2)	Homo sapiens
1169	11723059_turquoise	transporter 2, ATP binding cassette subfamily B member(TAP2)	Homo sapiens
1170	11723060_turquoise	Rab interacting lysosomal protein like 2(RILPL2)	Homo sapiens
1171	11723061_turquoise	Rab interacting lysosomal protein like 2(RILPL2)	Homo sapiens
1172	11723065_turquoise	endonuclease G(ENDOG)	Homo sapiens
1173	11723069_turquoise	ubiquitin D(UBD)	Homo sapiens
1174	11723080_brown	matrix AAA peptidase interacting protein 1(MAIP1)	Homo sapiens
1175	11723095_turquoise	CYLD lysine 63 deubiquitinase(CYLD)	Homo sapiens
1176	11723096_turquoise	CYLD lysine 63 deubiquitinase(CYLD)	Homo sapiens
1177	11723099_turquoise	carbonic anhydrase 14(CA14)	Homo sapiens
1178	11723100_turquoise	carbonic anhydrase 14(CA14)	Homo sapiens
1179	11723105_turquoise	cytidine/uridine monophosphate kinase 2(CMPK2)	Homo sapiens
1180	11723106_turquoise	neutrophil cytosolic factor 4(NCF4)	Homo sapiens
1181	11723110_turquoise	pleckstrin homology domain containing B2(PLEKHB2)	Homo sapiens
1182	11723116_turquoise	RAD51L3-RFFL readthrough(RAD51L3-RFFL)	Homo sapiens
1183	11723134_turquoise	solute carrier family 22 member 17(SLC22A17)	Homo sapiens
1184	11723144_turquoise	sodium leak channel, non-selective(NALCN)	Homo sapiens
1185	11723151_turquoise	succinyl-CoA:glutarate-CoA transferase(SUGCT)	Homo sapiens
1186	11723156_turquoise	lymphocyte-specific protein 1(LSP1)	Homo sapiens
1187	11723159_turquoise	transmembrane protein 229B(TMEM229B)	Homo sapiens
1188	11723169_turquoise	forkhead box N2(FOXN2)	Homo sapiens
1189	11723179_turquoise	intersectin 2(ITSN2)	Homo sapiens
1190	11723181_turquoise	mitogen-activated protein kinase 4(MAPK4)	Homo sapiens
1191	11723182_turquoise	CCR4-NOT transcription complex subunit 6 like(CNOT6L)	Homo sapiens
1192	11723183_turquoise	CCR4-NOT transcription complex subunit 6 like(CNOT6L)	Homo sapiens
1193	11723184_turquoise	CCR4-NOT transcription complex subunit 6 like(CNOT6L)	Homo sapiens
1194	11723185_turquoise	CCR4-NOT transcription complex subunit 6 like(CNOT6L)	Homo sapiens
1195	11723194_turquoise	major histocompatibility complex, class II, DQ beta 1(HLA-DQB1)	Homo sapiens
1196	11723195_turquoise	major histocompatibility complex, class I, E(HLA-E)	Homo sapiens
1197	11723197_turquoise	heterogeneous nuclear ribonucleoprotein A3(HNRNPA3)	Homo sapiens
1198	11723198_turquoise	heterogeneous nuclear ribonucleoprotein A3 pseudogene 1(HNRNPA3P1)	Homo sapiens

1199	11723199_ turquoise	heterogeneous nuclear ribonucleoprotein A3 pseudogene 1(HNRNPA3P1)	Homo sapiens
1200	11723217_ turquoise	sideroflexin 3(SFXN3)	Homo sapiens
1201	11723230_ turquoise	ring finger protein 138(RNF138)	Homo sapiens
1202	11723231_ turquoise	ring finger protein 138 pseudogene 1(RNF138P1)	Homo sapiens
1203	11723232_ turquoise	ring finger protein 138(RNF138)	Homo sapiens
1204	11723243_ turquoise	cyclin dependent kinase 6(CDK6)	Homo sapiens
1205	11723262_ turquoise	antagonist of mitotic exit network 1 homolog(AMN1)	Homo sapiens
1206	11723263_ blue	antagonist of mitotic exit network 1 homolog(AMN1)	Homo sapiens
1207	11723264_ turquoise	CD3d molecule(CD3D)	Homo sapiens
1208	11723287_ turquoise	TYRO protein tyrosine kinase binding protein(TYROBP)	Homo sapiens
1209	11723292_ turquoise	ORAI calcium release-activated calcium modulator 1(ORAI1)	Homo sapiens
1210	11723310_ turquoise	TNF receptor superfamily member 10d(TNFRSF10D)	Homo sapiens
1211	11723313_ brown	peroxisomal membrane protein 2(PXMP2)	Homo sapiens
1212	11723335_ turquoise	GEM interacting protein(GMIP)	Homo sapiens
1213	11723346_ turquoise	LYL1, basic helix-loop-helix family member(LYL1)	Homo sapiens
1214	11723372_ turquoise	dedicator of cytokinesis 11(DOCK11)	Homo sapiens
1215	11723378_ turquoise	centriolin(CNTRL)	Homo sapiens
1216	11723400_ turquoise	serine/threonine kinase 4(STK4)	Homo sapiens
1217	11723401_ turquoise	serine/threonine kinase 4(STK4)	Homo sapiens
1218	11723402_ turquoise	serine/threonine kinase 4(STK4)	Homo sapiens
1219	11723404_ blue	PPARG coactivator 1 alpha(PPARGC1A)	Homo sapiens
1220	11723408_ turquoise	McKusick-Kaufman syndrome(MKKS)	Homo sapiens
1221	11723409_ turquoise	McKusick-Kaufman syndrome(MKKS)	Homo sapiens
1222	11723419_ turquoise	collagen type IX alpha 3 chain(COL9A3)	Homo sapiens
1223	11723424_ turquoise	interferon alpha and beta receptor subunit 1(IFNAR1)	Homo sapiens
1224	11723425_ turquoise	interferon alpha and beta receptor subunit 1(IFNAR1)	Homo sapiens
1225	11723426_ brown	electron transfer flavoprotein beta subunit(ETFB)	Homo sapiens
1226	11723437_ turquoise	centrosomal protein 41(CEP41)	Homo sapiens
1227	11723447_ turquoise	mal, T-cell differentiation protein like(MALL)	Homo sapiens
1228	11723448_ turquoise	mal, T-cell differentiation protein like(MALL)	Homo sapiens
1229	11723449_ brown	NADH:ubiquinone oxidoreductase subunit V3(NDUFV3)	Homo sapiens
1230	11723467_ brown	NADH:ubiquinone oxidoreductase subunit A3(NDUFA3)	Homo sapiens
1231	11723469_ turquoise	lysophosphatidylglycerol acyltransferase 1(LPGAT1)	Homo sapiens
1232	11723491_ turquoise	Rho guanine nucleotide exchange factor 3(ARHGEF3)	Homo sapiens
1233	11723492_ turquoise	Rho guanine nucleotide exchange factor 3(ARHGEF3)	Homo sapiens
1234	11723494_ turquoise	ring finger protein 166(RNF166)	Homo sapiens
1235	11723516_ turquoise	calcium binding protein 39 like(CAB39L)	Homo sapiens
1236	11723534_ turquoise	breast cancer metastasis-suppressor 1-like(BRMS1L)	Homo sapiens
1237	11723537_ turquoise	Rho GTPase activating protein 45(ARHGAP45)	Homo sapiens
1238	11723545_ turquoise	phospholipase D1(PLD1)	Homo sapiens
1239	11723546_ turquoise	phospholipase D1(PLD1)	Homo sapiens
1240	11723566_ blue	calpain 7(CAPN7)	Homo sapiens
1241	11723590_ turquoise	tetratricopeptide repeat domain 13(TTC13)	Homo sapiens
1242	11723591_ turquoise	leucine rich repeat containing 8 family member C(LRRC8C)	Homo sapiens
1243	11723592_ turquoise	leucine rich repeat containing 8 family member C(LRRC8C)	Homo sapiens
1244	11723604_ turquoise	inositol polyphosphate-5-phosphatase J(INPP5J)	Homo sapiens
1245	11723605_ turquoise	PC-esterase domain containing 1B(PCED1B)	Homo sapiens
1246	11723623_ turquoise	tripartite motif containing 21(TRIM21)	Homo sapiens
1247	11723638_ turquoise	chromosome 11 open reading frame 58(C11orf58)	Homo sapiens
1248	11723639_ turquoise	chromosome 11 open reading frame 58(C11orf58)	Homo sapiens

1249	11723679_ turquoise	CD69 molecule(CD69)	Homo sapiens
1250	11723689_ turquoise	cAMP responsive element binding protein like 2(CREBL2)	Homo sapiens
1251	11723690_ turquoise	cAMP responsive element binding protein like 2(CREBL2)	Homo sapiens
1252	11723695_ brown	heat shock transcription factor 1(HSF1)	Homo sapiens
1253	11723697_ turquoise	Fli-1 proto-oncogene, ETS transcription factor(FLI1)	Homo sapiens
1254	11723698_ turquoise	2'-5'-oligoadenylate synthetase 3(OAS3)	Homo sapiens
1255	11723699_ turquoise	2'-5'-oligoadenylate synthetase 3(OAS3)	Homo sapiens
1256	11723771_ turquoise	microtubule associated serine/threonine kinase like(MASTL)	Homo sapiens
1257	11723795_ turquoise	solute carrier family 29 member 2(SLC29A2)	Homo sapiens
1258	11723821_ turquoise	SMAD specific E3 ubiquitin protein ligase 2(SMURF2)	Homo sapiens
1259	11723826_ turquoise	chromosome 2 open reading frame 88(C2orf88)	Homo sapiens
1260	11723847_ turquoise	ATPase phospholipid transporting 11C(ATP11C)	Homo sapiens
1261	11723849_ turquoise	membrane spanning 4-domains A6A(MS4A6A)	Homo sapiens
1262	11723850_ turquoise	outer dense fiber of sperm tails 2 like(ODF2L)	Homo sapiens
1263	11723851_ turquoise	outer dense fiber of sperm tails 2 like(ODF2L)	Homo sapiens
1264	11723853_ turquoise	B-cell CLL/lymphoma 11A(BCL11A)	Homo sapiens
1265	11723854_ turquoise	sterile alpha motif domain containing 9(SAMD9)	Homo sapiens
1266	11723863_ turquoise	dedicator of cytokinesis 10(DOCK10)	Homo sapiens
1267	11723871_ turquoise	dedicator of cytokinesis 2(DOCK2)	Homo sapiens
1268	11723894_ turquoise	telomerase associated protein 1(TEP1)	Homo sapiens
1269	11723895_ turquoise	telomerase associated protein 1(TEP1)	Homo sapiens
1270	11723899_ turquoise	dehydrogenase/reductase 9(DHRS9)	Homo sapiens
1271	11723902_ turquoise	pleckstrin homology and RhoGEF domain containing G1(PLEKHG1)	Homo sapiens
1272	11723903_ turquoise	pleckstrin homology and RhoGEF domain containing G1(PLEKHG1)	Homo sapiens
1273	11723910_ turquoise	chemerin chemokine-like receptor 1(CMKLR1)	Homo sapiens
1274	11723946_ turquoise	acyl-CoA synthetase long-chain family member 5(ACSL5)	Homo sapiens
1275	11723947_ turquoise	acyl-CoA synthetase long-chain family member 5(ACSL5)	Homo sapiens
1276	11723964_ turquoise	aspartylglucosaminidase(AGA)	Homo sapiens
1277	11723973_ turquoise	carbohydrate sulfotransferase 12(CHST12)	Homo sapiens
1278	11723974_ turquoise	apolipoprotein L3(APOL3)	Homo sapiens
1279	11723975_ turquoise	apolipoprotein L3(APOL3)	Homo sapiens
1280	11723977_ turquoise	RAB3A, member RAS oncogene family(RAB3A)	Homo sapiens
1281	11723978_ turquoise	RAB3A, member RAS oncogene family(RAB3A)	Homo sapiens
1282	11723979_ turquoise	phosphorylase kinase regulatory subunit alpha 2(PHKA2)	Homo sapiens
1283	11723991_ turquoise	ALG6, alpha-1,3-glucosyltransferase(ALG6)	Homo sapiens
1284	11723995_ turquoise	glucocorticoid induced 1(GLCCI1)	Homo sapiens
1285	11723996_ turquoise	glucocorticoid induced 1(GLCCI1)	Homo sapiens
1286	11723997_ turquoise	NFKB inhibitor interacting Ras like 1(NKIRAS1)	Homo sapiens
1287	11724004_ turquoise	FYN binding protein(FYB)	Homo sapiens
1288	11724005_ turquoise	FYN binding protein(FYB)	Homo sapiens
1289	11724006_ turquoise	ectonucleoside triphosphate diphosphohydrolase 1(ENTPD1)	Homo sapiens
1290	11724008_ turquoise	ectonucleoside triphosphate diphosphohydrolase 1(ENTPD1)	Homo sapiens
1291	11724013_ turquoise	sterol O-acyltransferase 1(SOAT1)	Homo sapiens
1292	11724014_ turquoise	sterol O-acyltransferase 1(SOAT1)	Homo sapiens
1293	11724033_ turquoise	CD2 associated protein(CD2AP)	Homo sapiens
1294	11724037_ turquoise	prostaglandin-endoperoxide synthase 2(PTGS2)	Homo sapiens
1295	11724058_ turquoise	ceramide-1-phosphate transfer protein(CPTP)	Homo sapiens
1296	11724061_ turquoise	oxysterol binding protein like 1A(OSBPL1A)	Homo sapiens
1297	11724082_ turquoise	torsin family 2 member A(TOR2A)	Homo sapiens
1298	11724102_ turquoise	PDZ domain containing ring finger 3(PDZRN3)	Homo sapiens

1299	11724103_ turquoise	C8orf44-SGK3 readthrough(C8orf44-SGK3)	Homo sapiens
1300	11724104_ turquoise	C8orf44-SGK3 readthrough(C8orf44-SGK3)	Homo sapiens
1301	11724106_ turquoise	basic leucine zipper ATF-like transcription factor 2(BATF2)	Homo sapiens
1302	11724117_ turquoise	sterile alpha motif domain containing 9 like(SAMD9L)	Homo sapiens
1303	11724118_ turquoise	sterile alpha motif domain containing 9 like(SAMD9L)	Homo sapiens
1304	11724119_ turquoise	sterile alpha motif domain containing 9 like(SAMD9L)	Homo sapiens
1305	11724145_ turquoise	solute carrier family 37 member 1(SLC37A1)	Homo sapiens
1306	11724149_ turquoise	src kinase associated phosphoprotein 1(SKAP1)	Homo sapiens
1307	11724159_ turquoise	endothelial cell surface expressed chemotaxis and apoptosis regulator(ECSCR)	Homo sapiens
1308	11724163_ turquoise	major histocompatibility complex, class II, DO beta(HLA-DOB)	Homo sapiens
1309	11724166_ turquoise	collagen beta(1-O)galactosyltransferase 2(COLGALT2)	Homo sapiens
1310	11724167_ turquoise	collagen beta(1-O)galactosyltransferase 2(COLGALT2)	Homo sapiens
1311	11724172_ turquoise	FCH domain only 2(FCHO2)	Homo sapiens
1312	11724188_ turquoise	poly(ADP-ribose) polymerase family member 10(PARP10)	Homo sapiens
1313	11724192_ turquoise	COP9 signalosome subunit 2(COPS2)	Homo sapiens
1314	11724201_ turquoise	transmembrane protein 143(TMEM143)	Homo sapiens
1315	11724214_ turquoise	SPRY domain containing 4(SPRYD4)	Homo sapiens
1316	11724224_ turquoise	adenosine monophosphate deaminase 2(AMPD2)	Homo sapiens
1317	11724226_ turquoise	microRNA 6748(MIR6748)	Homo sapiens
1318	11724236_ turquoise	receptor interacting serine/threonine kinase 2(RIPK2)	Homo sapiens
1319	11724237_ turquoise	linker for activation of T-cells family member 2(LAT2)	Homo sapiens
1320	11724255_ turquoise	2'-5'-oligoadenylate synthetase 1(OAS1)	Homo sapiens
1321	11724256_ turquoise	2'-5'-oligoadenylate synthetase 1(OAS1)	Homo sapiens
1322	11724264_ brown	intraflagellar transport 22(IFT22)	Homo sapiens
1323	11724271_ turquoise	HLF, PAR bZIP transcription factor(HLF)	Homo sapiens
1324	11724272_ turquoise	HLF, PAR bZIP transcription factor(HLF)	Homo sapiens
1325	11724296_ turquoise	bone morphogenetic protein 7(BMP7)	Homo sapiens
1326	11724297_ turquoise	bone morphogenetic protein 7(BMP7)	Homo sapiens
1327	11724310_ turquoise	SH3 domain binding protein 2(SH3BP2)	Homo sapiens
1328	11724311_ turquoise	SH3 domain binding protein 2(SH3BP2)	Homo sapiens
1329	11724312_ turquoise	SH3 domain binding protein 2(SH3BP2)	Homo sapiens
1330	11724326_ turquoise	Rho GTPase activating protein 25(ARHGAP25)	Homo sapiens
1331	11724328_ turquoise	ubiquitin conjugating enzyme E2 C(UBE2C)	Homo sapiens
1332	11724343_ turquoise	integrin subunit alpha 4(ITGA4)	Homo sapiens
1333	11724344_ turquoise	integrin subunit alpha 4(ITGA4)	Homo sapiens
1334	11724345_ turquoise	integrin subunit alpha 4(ITGA4)	Homo sapiens
1335	11724346_ turquoise	interferon induced with helicase C domain 1(IFIH1)	Homo sapiens
1336	11724347_ turquoise	astrotactin 2(ASTN2)	Homo sapiens
1337	11724348_ turquoise	astrotactin 2(ASTN2)	Homo sapiens
1338	11724350_ blue	protein phosphatase 1 regulatory inhibitor subunit 14C(PPP1R14C)	Homo sapiens
1339	11724354_ turquoise	autophagy related 16 like 2(ATG16L2)	Homo sapiens
1340	11724356_ brown	transmembrane protein 177(TMEM177)	Homo sapiens
1341	11724357_ turquoise	myosin VA(MYO5A)	Homo sapiens
1342	11724358_ turquoise	myosin VA(MYO5A)	Homo sapiens
1343	11724360_ turquoise	beta-transducin repeat containing E3 ubiquitin protein ligase(BTRC)	Homo sapiens
1344	11724361_ turquoise	phosphoinositide kinase, FYVE-type zinc finger containing(PIKFYVE)	Homo sapiens
1345	11724374_ turquoise	basic leucine zipper ATF-like transcription factor(BATF)	Homo sapiens
1346	11724375_ turquoise	phosphoprotein membrane anchor with glycosphingolipid microdomains 1(PAG1)	Homo sapiens
1347	11724377_ turquoise	phosphoprotein membrane anchor with glycosphingolipid microdomains 1(PAG1)	Homo sapiens
1348	11724378_ turquoise	phosphoprotein membrane anchor with glycosphingolipid microdomains 1(PAG1)	Homo sapiens

1349	11724381_turquoise	family with sequence similarity 65 member B(FAM65B)	Homo sapiens
1350	11724391_turquoise	aspartate beta-hydroxylase domain containing 2(ASPHD2)	Homo sapiens
1351	11724392_turquoise	aspartate beta-hydroxylase domain containing 2(ASPHD2)	Homo sapiens
1352	11724399_turquoise	DEAD-box helicase 60-like(DDX60L)	Homo sapiens
1353	11724404_turquoise	Wilms tumor 1 associated protein(WTAP)	Homo sapiens
1354	11724424_turquoise	G protein-coupled receptor 68(GPR68)	Homo sapiens
1355	11724432_turquoise	trafficking protein particle complex 2(TRAPPC2)	Homo sapiens
1356	11724435_turquoise	thiamin pyrophosphokinase 1(TPK1)	Homo sapiens
1357	11724465_turquoise	anillin actin binding protein(ANLN)	Homo sapiens
1358	11724485_turquoise	c-src tyrosine kinase(CSK)	Homo sapiens
1359	11724499_turquoise	MFNG O-fucosylpeptide 3-beta-N-acetylglucosaminyltransferase(MFNG)	Homo sapiens
1360	11724509_turquoise	phorbol-12-myristate-13-acetate-induced protein 1(PMAIP1)	Homo sapiens
1361	11724510_turquoise	phorbol-12-myristate-13-acetate-induced protein 1(PMAIP1)	Homo sapiens
1362	11724559_turquoise	G protein subunit alpha 15(GNA15)	Homo sapiens
1363	11724565_turquoise	talin 2(TLN2)	Homo sapiens
1364	11724594_brown	HRAS like suppressor(HRASLS)	Homo sapiens
1365	11724595_turquoise	retinitis pigmentosa 2 (X-linked recessive)(RP2)	Homo sapiens
1366	11724596_turquoise	TRAF family member associated NFKB activator(TANK)	Homo sapiens
1367	11724601_turquoise	endoplasmic reticulum aminopeptidase 1(ERAP1)	Homo sapiens
1368	11724602_turquoise	endoplasmic reticulum aminopeptidase 1(ERAP1)	Homo sapiens
1369	11724640_turquoise	Rho GTPase activating protein 44(ARHGAP44)	Homo sapiens
1370	11724656_turquoise	mitogen-activated protein kinase kinase kinase kinase 1(MAP4K1)	Homo sapiens
1371	11724669_brown	mitochondrial ribosomal protein S22(MRPS22)	Homo sapiens
1372	11724673_turquoise	branched chain keto acid dehydrogenase E1 subunit beta(BCKDHB)	Homo sapiens
1373	11724678_turquoise	lymphocyte antigen 86(LY86)	Homo sapiens
1374	11724682_turquoise	transforming growth factor beta receptor 2(TGFBR2)	Homo sapiens
1375	11724683_turquoise	CD300a molecule(CD300A)	Homo sapiens
1376	11724685_turquoise	interferon alpha and beta receptor subunit 2(IFNAR2)	Homo sapiens
1377	11724686_turquoise	interferon alpha and beta receptor subunit 2(IFNAR2)	Homo sapiens
1378	11724701_turquoise	BORCS7-ASMT readthrough (NMD candidate)(BORCS7-ASMT)	Homo sapiens
1379	11724728_turquoise	CD8a molecule(CD8A)	Homo sapiens
1380	11724729_turquoise	CD8a molecule(CD8A)	Homo sapiens
1381	11724732_turquoise	tubulin beta class I(TUBB)	Homo sapiens
1382	11724765_blue	FAST kinase domains 2(FASTKD2)	Homo sapiens
1383	11724766_turquoise	FAST kinase domains 2(FASTKD2)	Homo sapiens
1384	11724768_turquoise	Fc fragment of IgG receptor IIa(FCGR2A)	Homo sapiens
1385	11724769_turquoise	Fc fragment of IgG receptor IIa(FCGR2A)	Homo sapiens
1386	11724770_turquoise	Fc fragment of IgG receptor IIa(FCGR2A)	Homo sapiens
1387	11724771_turquoise	Fc fragment of IgG receptor IIa(FCGR2A)	Homo sapiens
1388	11724775_turquoise	ferredoxin 1(FDX1)	Homo sapiens
1389	11724776_turquoise	phosphoglucomutase 2 like 1(PGM2L1)	Homo sapiens
1390	11724780_brown	mitochondrial ribosomal protein S17(MRPS17)	Homo sapiens
1391	11724793_turquoise	phosphatidylglycerophosphate synthase 1(PGS1)	Homo sapiens
1392	11724799_turquoise	major histocompatibility complex, class II, DQ alpha 1(HLA-DQA1)	Homo sapiens
1393	11724800_turquoise	quinolinate phosphoribosyltransferase(QPRT)	Homo sapiens
1394	11724804_turquoise	leukocyte associated immunoglobulin like receptor 1(LAIR1)	Homo sapiens
1395	11724805_turquoise	leukocyte associated immunoglobulin like receptor 1(LAIR1)	Homo sapiens
1396	11724807_turquoise	Cbl proto-oncogene(CBL)	Homo sapiens
1397	11724810_turquoise	Cbl proto-oncogene(CBL)	Homo sapiens
1398	11724817_turquoise	SH3 domain containing kinase binding protein 1(SH3KBP1)	Homo sapiens

1399	11724820_	turquoise	NIMA related kinase 6(NEK6)	Homo sapiens
1400	11724821_	turquoise	NIMA related kinase 6(NEK6)	Homo sapiens
1401	11724844_	turquoise	abhydrolase domain containing 17A(ABHD17A)	Homo sapiens
1402	11724851_	turquoise	syntaxin binding protein 6(STXBP6)	Homo sapiens
1403	11724853_	turquoise	LDL receptor related protein 3(LRP3)	Homo sapiens
1404	11724857_	turquoise	centrosomal protein 63(CEP63)	Homo sapiens
1405	11724862_	turquoise	threonine synthase like 1(THNSL1)	Homo sapiens
1406	11724881_	turquoise	zinc finger protein 217(ZNF217)	Homo sapiens
1407	11724883_	turquoise	hyaluronan and proteoglycan link protein 3(HAPLN3)	Homo sapiens
1408	11724884_	turquoise	hyaluronan and proteoglycan link protein 3(HAPLN3)	Homo sapiens
1409	11724900_	turquoise	granzyme B(GZMB)	Homo sapiens
1410	11724904_	turquoise	TIMP metallopeptidase inhibitor 4(TIMP4)	Homo sapiens
1411	11724908_	turquoise	dynein cytoplasmic 1 intermediate chain 1(DYNC1I1)	Homo sapiens
1412	11724921_	turquoise	vacuolar protein sorting 13 homolog B(VPS13B)	Homo sapiens
1413	11724944_	turquoise	calsyntenin 2(CLSTN2)	Homo sapiens
1414	11724947_	turquoise	membrane associated guanylate kinase, WW and PDZ domain containing 2(MAGI2)	Homo sapiens
1415	11724954_	turquoise	mannose receptor, C type 1(MRC1)	Homo sapiens
1416	11724958_	turquoise	5',3'-nucleotidase, mitochondrial(NT5M)	Homo sapiens
1417	11724963_	turquoise	F-box protein 5(FBXO5)	Homo sapiens
1418	11724979_	turquoise	dipeptidase 2(DPEP2)	Homo sapiens
1419	11724997_	turquoise	CD86 molecule(CD86)	Homo sapiens
1420	11724998_	turquoise	CD86 molecule(CD86)	Homo sapiens
1421	11725011_	turquoise	centrosomal protein 78(CEP78)	Homo sapiens
1422	11725013_	turquoise	methylmalonic aciduria (cobalamin deficiency) cblC type, with homocystinuria(MMACH	Homo sapiens
1423	11725042_	turquoise	tyrosine 3-monooxygenase/tryptophan 5-monooxygenase activation protein beta(YWH	Homo sapiens
1424	11725044_	turquoise	tyrosine 3-monooxygenase/tryptophan 5-monooxygenase activation protein beta(YWH	Homo sapiens
1425	11725084_	blue	ubiquitin C-terminal hydrolase L5(UCHL5)	Homo sapiens
1426	11725091_	turquoise	regulatory factor X5(RFX5)	Homo sapiens
1427	11725092_	turquoise	regulatory factor X5(RFX5)	Homo sapiens
1428	11725093_	turquoise	regulatory factor X5(RFX5)	Homo sapiens
1429	11725095_	turquoise	tetratricopeptide repeat domain 33(TTC33)	Homo sapiens
1430	11725110_	turquoise	nudE neurodevelopment protein 1(NDE1)	Homo sapiens
1431	11725124_	turquoise	cystatin F(CST7)	Homo sapiens
1432	11725135_	turquoise	VPS16, CORVET/HOPS core subunit(VPS16)	Homo sapiens
1433	11725137_	brown	synaptogyrin 1(SYNGR1)	Homo sapiens
1434	11725144_	turquoise	zinc finger protein 784(ZNF784)	Homo sapiens
1435	11725153_	turquoise	nuclear factor kappa B subunit 2(NFKB2)	Homo sapiens
1436	11725155_	turquoise	four jointed box 1(FJX1)	Homo sapiens
1437	11725174_	turquoise	ArfGAP with dual PH domains 2(ADAP2)	Homo sapiens
1438	11725175_	turquoise	ArfGAP with dual PH domains 2(ADAP2)	Homo sapiens
1439	11725180_	turquoise	runt related transcription factor 2(RUNX2)	Homo sapiens
1440	11725228_	turquoise	pyrimidinergic receptor P2Y6(P2RY6)	Homo sapiens
1441	11725232_	turquoise	ATPase phospholipid transporting 8B2(ATP8B2)	Homo sapiens
1442	11725237_	blue	RNA binding protein with multiple splicing 2(RBPMS2)	Homo sapiens
1443	11725238_	turquoise	RNA binding protein with multiple splicing 2(RBPMS2)	Homo sapiens
1444	11725240_	turquoise	receptor transporter protein 4(RTP4)	Homo sapiens
1445	11725249_	turquoise	lymphocyte antigen 75(LY75)	Homo sapiens
1446	11725255_	turquoise	interleukin 15(IL15)	Homo sapiens
1447	11725280_	turquoise	PR/SET domain 8(PRDM8)	Homo sapiens
1448	11725315_	turquoise	PRELI domain containing 1(PRELID1)	Homo sapiens

1449	11725351_	turquoise	KIAA0930(KIAA0930)	Homo sapiens
1450	11725352_	turquoise	KIAA0930(KIAA0930)	Homo sapiens
1451	11725373_	turquoise	TNFRSF1A associated via death domain(TRADD)	Homo sapiens
1452	11725379_	turquoise	protein O-glucosyltransferase 1(POGLUT1)	Homo sapiens
1453	11725380_	turquoise	protein O-glucosyltransferase 1(POGLUT1)	Homo sapiens
1454	11725385_	turquoise	UDP-glucose ceramide glucosyltransferase(UGCG)	Homo sapiens
1455	11725389_	turquoise	CD6 molecule(CD6)	Homo sapiens
1456	11725403_	turquoise	transmembrane 4 L six family member 18(TM4SF18)	Homo sapiens
1457	11725412_	turquoise	tripartite motif containing 14(TRIM14)	Homo sapiens
1458	11725413_	turquoise	tripartite motif containing 14(TRIM14)	Homo sapiens
1459	11725416_	turquoise	natural killer cell granule protein 7(NKG7)	Homo sapiens
1460	11725419_	turquoise	lysophosphatidylcholine acyltransferase 2(LPCAT2)	Homo sapiens
1461	11725424_	turquoise	RAB27A, member RAS oncogene family(RAB27A)	Homo sapiens
1462	11725425_	turquoise	RAB27A, member RAS oncogene family(RAB27A)	Homo sapiens
1463	11725426_	turquoise	RAB27A, member RAS oncogene family(RAB27A)	Homo sapiens
1464	11725429_	turquoise	lysosomal associated membrane protein 3(LAMP3)	Homo sapiens
1465	11725437_	turquoise	SPRY domain containing 7(SPRYD7)	Homo sapiens
1466	11725440_	turquoise	lysophosphatidic acid receptor 2(LPAR2)	Homo sapiens
1467	11725444_	turquoise	C-C motif chemokine receptor 1(CCR1)	Homo sapiens
1468	11725445_	turquoise	microRNA 8085(MIR8085)	Homo sapiens
1469	11725448_	turquoise	folliculin(FLCN)	Homo sapiens
1470	11725455_	turquoise	CD247 molecule(CD247)	Homo sapiens
1471	11725471_	turquoise	mannosidase alpha class 2A member 1(MAN2A1)	Homo sapiens
1472	11725472_	turquoise	progestin and adipoQ receptor family member 8(PAQR8)	Homo sapiens
1473	11725473_	turquoise	progestin and adipoQ receptor family member 8(PAQR8)	Homo sapiens
1474	11725476_	turquoise	ArfGAP with coiled-coil, ankyrin repeat and PH domains 1(ACAP1)	Homo sapiens
1475	11725481_	turquoise	CD27 molecule(CD27)	Homo sapiens
1476	11725485_	turquoise	disrupted in renal carcinoma 2(DIRC2)	Homo sapiens
1477	11725492_	turquoise	RAB20, member RAS oncogene family(RAB20)	Homo sapiens
1478	11725498_	turquoise	transmembrane protein 117(TMEM117)	Homo sapiens
1479	11725522_	turquoise	mesoderm posterior bHLH transcription factor 1(MESP1)	Homo sapiens
1480	11725523_	turquoise	desmoglein 2(DSG2)	Homo sapiens
1481	11725524_	turquoise	desmoglein 2(DSG2)	Homo sapiens
1482	11725525_	blue	desmoglein 2(DSG2)	Homo sapiens
1483	11725537_	turquoise	zinc and ring finger 3(ZNRF3)	Homo sapiens
1484	11725563_	brown	chromosome 12 open reading frame 60(C12orf60)	Homo sapiens
1485	11725590_	brown	nudix hydrolase 6(NUDT6)	Homo sapiens
1486	11725612_	brown	ARMC2 antisense RNA 1(ARMC2-AS1)	Homo sapiens
1487	11725617_	turquoise	heterogeneous nuclear ribonucleoprotein R(HNRNPR)	Homo sapiens
1488	11725620_	blue	pyruvate dehydrogenase (lipoamide) beta(PDHB)	Homo sapiens
1489	11725629_	turquoise	2',3'-cyclic nucleotide 3' phosphodiesterase(CNP)	Homo sapiens
1490	11725634_	turquoise	mitochondrial ribosomal protein S25(MRPS25)	Homo sapiens
1491	11725635_	brown	mitochondrial ribosomal protein S25(MRPS25)	Homo sapiens
1492	11725636_	turquoise	MOB kinase activator 1A(MOB1A)	Homo sapiens
1493	11725641_	turquoise	EF-hand domain family member D2(EFHD2)	Homo sapiens
1494	11725642_	turquoise	EF-hand domain family member D2(EFHD2)	Homo sapiens
1495	11725643_	brown	mitochondrial ribosomal protein S26(MRPS26)	Homo sapiens
1496	11725650_	turquoise	DDB1 and CUL4 associated factor 5(DCAF5)	Homo sapiens
1497	11725657_	turquoise	zinc finger with UFM1 specific peptidase domain(ZUFSP)	Homo sapiens
1498	11725682_	blue	MRS2, magnesium transporter(MRS2)	Homo sapiens

1499	11725683_	turquoise	MRS2, magnesium transporter(MRS2)	Homo sapiens
1500	11725684_	turquoise	protein tyrosine phosphatase, receptor type O(PTPRO)	Homo sapiens
1501	11725685_	turquoise	protein tyrosine phosphatase, receptor type O(PTPRO)	Homo sapiens
1502	11725697_	turquoise	tripartite motif containing 56(TRIM56)	Homo sapiens
1503	11725712_	turquoise	peptidyl-prolyl cis-trans isomerase A pseudogene(LOC101060363)	Homo sapiens
1504	11725729_	turquoise	chromosome 1 open reading frame 56(C1orf56)	Homo sapiens
1505	11725746_	turquoise	phosphatidylinositol transfer protein, cytoplasmic 1(PITPNC1)	Homo sapiens
1506	11725755_	turquoise	butyrophilin subfamily 2 member A2(BTN2A2)	Homo sapiens
1507	11725782_	turquoise	annexin A2 receptor(ANXA2R)	Homo sapiens
1508	11725789_	turquoise	dihydropyrimidine dehydrogenase(DPYD)	Homo sapiens
1509	11725790_	turquoise	dihydropyrimidine dehydrogenase(DPYD)	Homo sapiens
1510	11725793_	turquoise	prostaglandin E receptor 4(PTGER4)	Homo sapiens
1511	11725794_	turquoise	prostaglandin E receptor 4(PTGER4)	Homo sapiens
1512	11725803_	turquoise	chromosome 21 open reading frame 91(C21orf91)	Homo sapiens
1513	11725804_	turquoise	chromosome 21 open reading frame 91(C21orf91)	Homo sapiens
1514	11725811_	turquoise	phospholipase A2 group V(PLA2G5)	Homo sapiens
1515	11725813_	turquoise	microtubule associated serine/threonine kinase 3(MAST3)	Homo sapiens
1516	11725842_	turquoise	PIF1 5'-to-3' DNA helicase(PIF1)	Homo sapiens
1517	11725856_	turquoise	dynein axonemal heavy chain 1(DNAH1)	Homo sapiens
1518	11725861_	turquoise	MYB proto-oncogene like 1(MYBL1)	Homo sapiens
1519	11725876_	turquoise	glycosylphosphatidylinositol anchored high density lipoprotein binding protein 1(GPIHB	Homo sapiens
1520	11725887_	turquoise	family with sequence similarity 167 member B(FAM167B)	Homo sapiens
1521	11725894_	blue	taxilin beta(TXLNB)	Homo sapiens
1522	11725899_	turquoise	leucine rich repeat containing 8 family member D(LRRC8D)	Homo sapiens
1523	11725910_	turquoise	collagen type IV alpha 6 chain(COL4A6)	Homo sapiens
1524	11725912_	turquoise	erb-b2 receptor tyrosine kinase 4(ERBB4)	Homo sapiens
1525	11725917_	turquoise	ring finger protein 19B(RNF19B)	Homo sapiens
1526	11725924_	turquoise	Ras association domain family member 5(RASSF5)	Homo sapiens
1527	11725935_	turquoise	calcium/calmodulin dependent protein kinase II alpha(CAMK2A)	Homo sapiens
1528	11725957_	blue	translocase of inner mitochondrial membrane 17 homolog A (yeast)(TIMM17A)	Homo sapiens
1529	11725966_	turquoise	integrin subunit beta 7(ITGB7)	Homo sapiens
1530	11725970_	turquoise	heterogeneous nuclear ribonucleoprotein F(HNRNPF)	Homo sapiens
1531	11725971_	turquoise	heterogeneous nuclear ribonucleoprotein F(HNRNPF)	Homo sapiens
1532	11725972_	turquoise	heterogeneous nuclear ribonucleoprotein F(HNRNPF)	Homo sapiens
1533	11725973_	turquoise	heterogeneous nuclear ribonucleoprotein F(HNRNPF)	Homo sapiens
1534	11725981_	turquoise	C-C motif chemokine receptor 7(CCR7)	Homo sapiens
1535	11725983_	turquoise	basic helix-loop-helix family member e40(BHLHE40)	Homo sapiens
1536	11725991_	brown	regulator of microtubule dynamics 1(RMDN1)	Homo sapiens
1537	11725998_	turquoise	Fas cell surface death receptor(FAS)	Homo sapiens
1538	11725999_	turquoise	Fas cell surface death receptor(FAS)	Homo sapiens
1539	11726000_	turquoise	copine 4(CPNE4)	Homo sapiens
1540	11726004_	turquoise	layilin(LAYN)	Homo sapiens
1541	11726034_	turquoise	protein tyrosine phosphatase, non-receptor type 6(PTPN6)	Homo sapiens
1542	11726035_	blue	phosphoglycerate mutase 2(PGAM2)	Homo sapiens
1543	11726040_	turquoise	serine/threonine kinase 17b(STK17B)	Homo sapiens
1544	11726041_	turquoise	serine/threonine kinase 17b(STK17B)	Homo sapiens
1545	11726042_	turquoise	serine/threonine kinase 17b(STK17B)	Homo sapiens
1546	11726056_	turquoise	protein phosphatase 4 regulatory subunit 3B(PPP4R3B)	Homo sapiens
1547	11726087_	blue	heat shock protein family B (small) member 3(HSPB3)	Homo sapiens
1548	11726088_	turquoise	phosphatase and actin regulator 3(PHACTR3)	Homo sapiens

1549	11726089_turquoise	VPS53, GARP complex subunit(VPS53)	Homo sapiens
1550	11726104_blue	protein phosphatase 2 regulatory subunit B''alpha(PPP2R3A)	Homo sapiens
1551	11726120_turquoise	BMP2 inducible kinase(BMP2K)	Homo sapiens
1552	11726122_turquoise	myosin IF(MYO1F)	Homo sapiens
1553	11726153_yellow	POU class 2 associating factor 1(POU2AF1)	Homo sapiens
1554	11726158_turquoise	spindlin family member 4(SPIN4)	Homo sapiens
1555	11726168_turquoise	progesterone receptor(PGR)	Homo sapiens
1556	11726178_turquoise	SRY-box 6(SOX6)	Homo sapiens
1557	11726179_turquoise	SRY-box 6(SOX6)	Homo sapiens
1558	11726180_turquoise	SRY-box 6(SOX6)	Homo sapiens
1559	11726187_turquoise	shisa family member 3(SHISA3)	Homo sapiens
1560	11726188_turquoise	shisa family member 3(SHISA3)	Homo sapiens
1561	11726190_turquoise	dedicator of cytokinesis 4(DOCK4)	Homo sapiens
1562	11726201_turquoise	2'-5'-oligoadenylate synthetase 2(OAS2)	Homo sapiens
1563	11726217_turquoise	PR/SET domain 1(PRDM1)	Homo sapiens
1564	11726218_turquoise	PR/SET domain 1(PRDM1)	Homo sapiens
1565	11726242_turquoise	transketolase like 1(TKTL1)	Homo sapiens
1566	11726254_turquoise	CD74 molecule(CD74)	Homo sapiens
1567	11726255_turquoise	CD74 molecule(CD74)	Homo sapiens
1568	11726264_brown	STE20-related kinase adaptor beta(STRADB)	Homo sapiens
1569	11726286_turquoise	tryptophanyl-tRNA synthetase(WARS)	Homo sapiens
1570	11726287_turquoise	tryptophanyl-tRNA synthetase(WARS)	Homo sapiens
1571	11726292_turquoise	WD repeat domain 44(WDR44)	Homo sapiens
1572	11726293_turquoise	WD repeat domain 44(WDR44)	Homo sapiens
1573	11726294_turquoise	CCAAT/enhancer binding protein alpha(CEBPA)	Homo sapiens
1574	11726309_turquoise	glycine receptor beta(GLRB)	Homo sapiens
1575	11726310_turquoise	glycine receptor beta(GLRB)	Homo sapiens
1576	11726311_turquoise	glycine receptor beta(GLRB)	Homo sapiens
1577	11726315_turquoise	methyltransferase like 22(METTL22)	Homo sapiens
1578	11726316_turquoise	selectin E(SELE)	Homo sapiens
1579	11726321_turquoise	endoplasmic reticulum oxidoreductase 1 alpha(ERO1A)	Homo sapiens
1580	11726324_turquoise	cytokine receptor like factor 3(CRLF3)	Homo sapiens
1581	11726328_turquoise	guanylate binding protein 1(GBP1)	Homo sapiens
1582	11726329_turquoise	guanylate binding protein 1(GBP1)	Homo sapiens
1583	11726333_turquoise	lymphoid enhancer binding factor 1(LEF1)	Homo sapiens
1584	11726335_brown	cardiolipin synthase 1(CRLS1)	Homo sapiens
1585	11726337_turquoise	arachidonate 5-lipoxygenase(ALOX5)	Homo sapiens
1586	11726338_turquoise	arachidonate 5-lipoxygenase(ALOX5)	Homo sapiens
1587	11726347_turquoise	linker for activation of T-cells family member 2(LAT2)	Homo sapiens
1588	11726350_brown	ankyrin repeat domain 39(ANKRD39)	Homo sapiens
1589	11726353_turquoise	CD180 molecule(CD180)	Homo sapiens
1590	11726364_turquoise	2'-5'-oligoadenylate synthetase like(OASL)	Homo sapiens
1591	11726366_turquoise	glutamate rich 1(ERICH1)	Homo sapiens
1592	11726368_turquoise	glutamate rich 1(ERICH1)	Homo sapiens
1593	11726369_turquoise	GRIK1 antisense RNA 2(GRIK1-AS2)	Homo sapiens
1594	11726375_turquoise	G protein-coupled receptor 34(GPR34)	Homo sapiens
1595	11726378_turquoise	Wolf-Hirschhorn syndrome candidate 1(WHSC1)	Homo sapiens
1596	11726382_turquoise	signaling lymphocytic activation molecule family member 1(SLAMF1)	Homo sapiens
1597	11726392_turquoise	GTPase, IMAP family member 2(GIMAP2)	Homo sapiens
1598	11726410_turquoise	GID complex subunit 4 homolog(GID4)	Homo sapiens

1599	11726416_turquoise	abl interactor 1(ABI1)	Homo sapiens
1600	11726417_turquoise	abl interactor 1(ABI1)	Homo sapiens
1601	11726418_turquoise	abl interactor 1(ABI1)	Homo sapiens
1602	11726421_turquoise	amyloid beta precursor protein binding family B member 1 interacting protein(APBB1IP	Homo sapiens
1603	11726432_turquoise	oxysterol binding protein like 3(OSBPL3)	Homo sapiens
1604	11726433_turquoise	oxysterol binding protein like 3(OSBPL3)	Homo sapiens
1605	11726464_turquoise	IGF like family receptor 1(IGFLR1)	Homo sapiens
1606	11726473_turquoise	docking protein 3(DOK3)	Homo sapiens
1607	11726479_turquoise	MX dynamin like GTPase 2(MX2)	Homo sapiens
1608	11726494_turquoise	SUMO/sentrin peptidase family member, NEDD8 specific(SENP8)	Homo sapiens
1609	11726496_blue	ryanodine receptor 2(RYR2)	Homo sapiens
1610	11726517_turquoise	WD repeat domain 11(WDR11)	Homo sapiens
1611	11726528_turquoise	family with sequence similarity 81 member A(FAM81A)	Homo sapiens
1612	11726532_turquoise	cytohesin 4(CYTH4)	Homo sapiens
1613	11726542_turquoise	transmembrane protein 132C(TMEM132C)	Homo sapiens
1614	11726557_turquoise	gasdermin B(GSDMB)	Homo sapiens
1615	11726559_turquoise	lymphocyte cytosolic protein 1(LCP1)	Homo sapiens
1616	11726579_turquoise	proteasome activator subunit 1(PSME1)	Homo sapiens
1617	11726580_turquoise	proteasome activator subunit 1(PSME1)	Homo sapiens
1618	11726594_turquoise	cathepsin Z(CTSZ)	Homo sapiens
1619	11726608_turquoise	metal response element binding transcription factor 2(MTF2)	Homo sapiens
1620	11726610_turquoise	metal response element binding transcription factor 2(MTF2)	Homo sapiens
1621	11726611_turquoise	MAF bZIP transcription factor F(MAFF)	Homo sapiens
1622	11726617_turquoise	cyclin dependent kinase 1(CDK1)	Homo sapiens
1623	11726625_turquoise	cyclin G2(CCNG2)	Homo sapiens
1624	11726628_turquoise	semaphorin 4A(SEMA4A)	Homo sapiens
1625	11726647_turquoise	butyrophilin subfamily 3 member A2(BTN3A2)	Homo sapiens
1626	11726655_turquoise	interferon induced protein with tetratricopeptide repeats 5(IFIT5)	Homo sapiens
1627	11726656_turquoise	interferon induced protein with tetratricopeptide repeats 5(IFIT5)	Homo sapiens
1628	11726662_brown	apolipoprotein O(APOO)	Homo sapiens
1629	11726671_turquoise	DNA cross-link repair 1B(DCLRE1B)	Homo sapiens
1630	11726676_blue	apolipoprotein B mRNA editing enzyme catalytic subunit 2(APOBEC2)	Homo sapiens
1631	11726677_turquoise	proteasome subunit beta 9(PSMB9)	Homo sapiens
1632	11726689_turquoise	signal transducer and activator of transcription 1(STAT1)	Homo sapiens
1633	11726690_turquoise	signal transducer and activator of transcription 1(STAT1)	Homo sapiens
1634	11726700_turquoise	aftiphilin(AFTPH)	Homo sapiens
1635	11726701_turquoise	aftiphilin(AFTPH)	Homo sapiens
1636	11726702_turquoise	aftiphilin(AFTPH)	Homo sapiens
1637	11726703_turquoise	B-cell CLL/lymphoma 10(BCL10)	Homo sapiens
1638	11726704_turquoise	B-cell CLL/lymphoma 10(BCL10)	Homo sapiens
1639	11726707_turquoise	interleukin 34(IL34)	Homo sapiens
1640	11726723_turquoise	WD repeat and FYVE domain containing 2(WDFY2)	Homo sapiens
1641	11726725_turquoise	nucleic acid binding protein 1(NABP1)	Homo sapiens
1642	11726726_turquoise	nucleic acid binding protein 1(NABP1)	Homo sapiens
1643	11726727_turquoise	nucleic acid binding protein 1(NABP1)	Homo sapiens
1644	11726769_turquoise	XIAP associated factor 1(XAF1)	Homo sapiens
1645	11726770_turquoise	XIAP associated factor 1(XAF1)	Homo sapiens
1646	11726771_turquoise	XIAP associated factor 1(XAF1)	Homo sapiens
1647	11726777_turquoise	linker for activation of T-cells(LAT)	Homo sapiens
1648	11726779_turquoise	IKBKB interacting protein(IKBIP)	Homo sapiens

1649	11726803_turquoise	helicase with zinc finger 2(HELZ2)	Homo sapiens
1650	11726820_turquoise	Rho GTPase activating protein 9(ARHGAP9)	Homo sapiens
1651	11726822_turquoise	chromosome X open reading frame 36(CXorf36)	Homo sapiens
1652	11726839_turquoise	RELT like 1(RELL1)	Homo sapiens
1653	11726869_turquoise	major facilitator superfamily domain containing 12(MFSD12)	Homo sapiens
1654	11726875_turquoise	promyelocytic leukemia(PML)	Homo sapiens
1655	11726876_turquoise	promyelocytic leukemia(PML)	Homo sapiens
1656	11726883_turquoise	ATPase family, AAA domain containing 2B(ATAD2B)	Homo sapiens
1657	11726893_turquoise	microRNA 6132(MIR6132)	Homo sapiens
1658	11726928_turquoise	interleukin 18 binding protein(IL18BP)	Homo sapiens
1659	11726947_turquoise	toll like receptor 8(TLR8)	Homo sapiens
1660	11726948_turquoise	toll like receptor 8(TLR8)	Homo sapiens
1661	11726963_turquoise	small glutamine rich tetratricopeptide repeat containing beta(SGTB)	Homo sapiens
1662	11726974_blue	PERP, TP53 apoptosis effector(PERP)	Homo sapiens
1663	11726975_blue	PERP, TP53 apoptosis effector(PERP)	Homo sapiens
1664	11727005_turquoise	adhesion G protein-coupled receptor L4(ADGRL4)	Homo sapiens
1665	11727006_turquoise	T-cell activation RhoGTPase activating protein(TAGAP)	Homo sapiens
1666	11727009_turquoise	aldehyde dehydrogenase 7 family member A1(ALDH7A1)	Homo sapiens
1667	11727022_turquoise	transmembrane protein 64(TMEM64)	Homo sapiens
1668	11727044_turquoise	transmembrane protein 173(TMEM173)	Homo sapiens
1669	11727058_turquoise	mitochondrial ribosomal protein S36(MRPS36)	Homo sapiens
1670	11727059_turquoise	mitochondrial ribosomal protein S36(MRPS36)	Homo sapiens
1671	11727060_brown	mitochondrial ribosomal protein S36(MRPS36)	Homo sapiens
1672	11727062_turquoise	PIR-FIGF readthrough(PIR-FIGF)	Homo sapiens
1673	11727080_turquoise	NUFIP1, FMR1 interacting protein 1(NUFIP1)	Homo sapiens
1674	11727092_turquoise	interleukin 18(IL18)	Homo sapiens
1675	11727111_turquoise	formin binding protein 1(FNBP1)	Homo sapiens
1676	11727112_turquoise	signaling threshold regulating transmembrane adaptor 1(SIT1)	Homo sapiens
1677	11727116_turquoise	phospholipase A1 member A(PLA1A)	Homo sapiens
1678	11727118_turquoise	complement factor properdin(CFP)	Homo sapiens
1679	11727120_turquoise	MYC binding protein 2, E3 ubiquitin protein ligase(MYCBP2)	Homo sapiens
1680	11727121_turquoise	MYC binding protein 2, E3 ubiquitin protein ligase(MYCBP2)	Homo sapiens
1681	11727141_turquoise	erythrocyte membrane protein band 4.1 like 3(EPB41L3)	Homo sapiens
1682	11727142_turquoise	erythrocyte membrane protein band 4.1 like 3(EPB41L3)	Homo sapiens
1683	11727143_turquoise	erythrocyte membrane protein band 4.1 like 3(EPB41L3)	Homo sapiens
1684	11727146_turquoise	G protein-coupled receptor 160(GPR160)	Homo sapiens
1685	11727186_turquoise	Wnt family member 5A(WNT5A)	Homo sapiens
1686	11727210_turquoise	Purkinje cell protein 2(PCP2)	Homo sapiens
1687	11727219_turquoise	dermatan sulfate epimerase(DSE)	Homo sapiens
1688	11727224_turquoise	polypeptide N-acetylgalactosaminyltransferase 16(GALNT16)	Homo sapiens
1689	11727225_turquoise	polypeptide N-acetylgalactosaminyltransferase 16(GALNT16)	Homo sapiens
1690	11727254_turquoise	transmembrane protein 51(TMEM51)	Homo sapiens
1691	11727257_turquoise	Janus kinase 3(JAK3)	Homo sapiens
1692	11727291_turquoise	TEN1-CDK3 readthrough (NMD candidate)(TEN1-CDK3)	Homo sapiens
1693	11727292_turquoise	chromosome 1 open reading frame 162(C1orf162)	Homo sapiens
1694	11727321_turquoise	elastin microfibril interfacer 3(EMILIN3)	Homo sapiens
1695	11727327_turquoise	family with sequence similarity 131 member C(FAM131C)	Homo sapiens
1696	11727360_turquoise	gap junction protein delta 3(GJD3)	Homo sapiens
1697	11727372_turquoise	3'-phosphoadenosine 5'-phosphosulfate synthase 1(PAPSS1)	Homo sapiens
1698	11727373_turquoise	3'-phosphoadenosine 5'-phosphosulfate synthase 1(PAPSS1)	Homo sapiens

1699	11727404_ grey	glypican 4(GPC4)	Homo sapiens
1700	11727417_ blue	ATP5S like(ATP5SL)	Homo sapiens
1701	11727428_ turquoise	G protein-coupled receptor 183(GPR183)	Homo sapiens
1702	11727456_ turquoise	OFD1, centriole and centriolar satellite protein(OFD1)	Homo sapiens
1703	11727467_ turquoise	interleukin 10 receptor subunit beta(IL10RB)	Homo sapiens
1704	11727478_ turquoise	angiotensin II receptor associated protein(AGTRAP)	Homo sapiens
1705	11727489_ turquoise	kinesin family member 11(KIF11)	Homo sapiens
1706	11727497_ turquoise	zinc finger RANBP2-type containing 1(ZRANB1)	Homo sapiens
1707	11727499_ turquoise	zinc finger RANBP2-type containing 1(ZRANB1)	Homo sapiens
1708	11727516_ turquoise	ribonucleoprotein, PTB binding 2(RAVER2)	Homo sapiens
1709	11727522_ turquoise	zinc finger protein 267(ZNF267)	Homo sapiens
1710	11727523_ turquoise	zinc finger protein 267(ZNF267)	Homo sapiens
1711	11727532_ turquoise	fasciculation and elongation protein zeta 2(FEZ2)	Homo sapiens
1712	11727533_ turquoise	fasciculation and elongation protein zeta 2(FEZ2)	Homo sapiens
1713	11727538_ turquoise	NME/NM23 family member 5(NME5)	Homo sapiens
1714	11727539_ turquoise	iron-sulfur cluster assembly enzyme(ISCU)	Homo sapiens
1715	11727547_ turquoise	RB binding protein 8, endonuclease(RBBP8)	Homo sapiens
1716	11727561_ turquoise	transcription factor 7 (T-cell specific, HMG-box)(TCF7)	Homo sapiens
1717	11727565_ turquoise	tumor necrosis factor superfamily member 13b(TNFSF13B)	Homo sapiens
1718	11727572_ turquoise	kelch like family member 6(KLHL6)	Homo sapiens
1719	11727574_ turquoise	retinol binding protein 5(RBP5)	Homo sapiens
1720	11727577_ turquoise	transmembrane protein 237(TMEM237)	Homo sapiens
1721	11727600_ turquoise	TNF receptor superfamily member 4(TNFRSF4)	Homo sapiens
1722	11727609_ turquoise	killer cell lectin like receptor B1(KLRB1)	Homo sapiens
1723	11727622_ turquoise	vitrin(VIT)	Homo sapiens
1724	11727643_ turquoise	transcriptional regulating factor 1(TRERF1)	Homo sapiens
1725	11727677_ turquoise	biogenesis of lysosomal organelles complex 1 subunit 3(BLOC1S3)	Homo sapiens
1726	11727694_ turquoise	KLRC4-KLRK1 readthrough(KLRC4-KLRK1)	Homo sapiens
1727	11727695_ turquoise	KLRC4-KLRK1 readthrough(KLRC4-KLRK1)	Homo sapiens
1728	11727701_ turquoise	family with sequence similarity 185 member A(FAM185A)	Homo sapiens
1729	11727707_ turquoise	microRNA 3191(MIR3191)	Homo sapiens
1730	11727740_ turquoise	CD300 molecule like family member f(CD300LF)	Homo sapiens
1731	11727775_ turquoise	G protein subunit alpha i3(GNAI3)	Homo sapiens
1732	11727776_ turquoise	G protein subunit alpha i3(GNAI3)	Homo sapiens
1733	11727778_ turquoise	G protein subunit alpha i3(GNAI3)	Homo sapiens
1734	11727779_ turquoise	growth hormone inducible transmembrane protein(GHITM)	Homo sapiens
1735	11727782_ turquoise	tropomyosin 4(TPM4)	Homo sapiens
1736	11727783_ turquoise	tropomyosin 4(TPM4)	Homo sapiens
1737	11727791_ blue	dihydrolipoamide S-acetyltransferase(DLAT)	Homo sapiens
1738	11727792_ blue	dihydrolipoamide S-acetyltransferase(DLAT)	Homo sapiens
1739	11727793_ blue	dihydrolipoamide S-acetyltransferase(DLAT)	Homo sapiens
1740	11727797_ turquoise	Nedd4 family interacting protein 2(NDFIP2)	Homo sapiens
1741	11727816_ turquoise	ubiquitin associated and SH3 domain containing B(UBASH3B)	Homo sapiens
1742	11727817_ turquoise	ubiquitin associated and SH3 domain containing B(UBASH3B)	Homo sapiens
1743	11727820_ brown	CDGSH iron sulfur domain 1(CISD1)	Homo sapiens
1744	11727821_ turquoise	CDGSH iron sulfur domain 1(CISD1)	Homo sapiens
1745	11727835_ turquoise	Enah/Vasp-like(EVL)	Homo sapiens
1746	11727841_ turquoise	protein kinase C delta(PRKCD)	Homo sapiens
1747	11727853_ turquoise	nucleoporin 50(NUP50)	Homo sapiens
1748	11727854_ turquoise	nucleoporin 50(NUP50)	Homo sapiens

1749	11727855_	turquoise	nucleoporin 50(NUP50)	Homo sapiens
1750	11727856_	turquoise	nucleoporin 50(NUP50)	Homo sapiens
1751	11727876_	turquoise	cytochrome b-245 beta chain(CYBB)	Homo sapiens
1752	11727877_	turquoise	MIA-RAB4B readthrough (NMD candidate)(MIA-RAB4B)	Homo sapiens
1753	11727891_	turquoise	proline and arginine rich end leucine rich repeat protein(PRELP)	Homo sapiens
1754	11727892_	turquoise	proline and arginine rich end leucine rich repeat protein(PRELP)	Homo sapiens
1755	11727894_	turquoise	G kinase anchoring protein 1(GKAP1)	Homo sapiens
1756	11727904_	turquoise	interleukin 13 receptor subunit alpha 1(IL13RA1)	Homo sapiens
1757	11727905_	turquoise	interleukin 13 receptor subunit alpha 1(IL13RA1)	Homo sapiens
1758	11727913_	turquoise	formin like 1(FMNL1)	Homo sapiens
1759	11727920_	turquoise	osteoclast stimulating factor 1(OSTF1)	Homo sapiens
1760	11727930_	turquoise	ATPase plasma membrane Ca2+ transporting 1(ATP2B1)	Homo sapiens
1761	11727936_	turquoise	SLC9A3 regulator 1(SLC9A3R1)	Homo sapiens
1762	11727942_	turquoise	formyl peptide receptor 3(FPR3)	Homo sapiens
1763	11727943_	turquoise	formyl peptide receptor 3(FPR3)	Homo sapiens
1764	11727970_	turquoise	solute carrier family 14 member 1 (Kidd blood group)(SLC14A1)	Homo sapiens
1765	11727992_	turquoise	syntaxin 11(STX11)	Homo sapiens
1766	11727994_	turquoise	syntaxin 11(STX11)	Homo sapiens
1767	11728003_	blue	chromosome 4 open reading frame 48(C4orf48)	Homo sapiens
1768	11728021_	brown	coiled-coil-helix-coiled-coil-helix domain containing 4(CHCHD4)	Homo sapiens
1769	11728027_	brown	coiled-coil domain containing 58(CCDC58)	Homo sapiens
1770	11728033_	turquoise	SFT2 domain containing 2(SFT2D2)	Homo sapiens
1771	11728038_	turquoise	C-C motif chemokine ligand 8(CCL8)	Homo sapiens
1772	11728039_	turquoise	C-C motif chemokine ligand 8(CCL8)	Homo sapiens
1773	11728040_	brown	peroxiredoxin 5(PRDX5)	Homo sapiens
1774	11728055_	turquoise	growth hormone receptor(GHR)	Homo sapiens
1775	11728056_	turquoise	caspase 10(CASP10)	Homo sapiens
1776	11728062_	turquoise	SH3 domain binding protein 1(SH3BP1)	Homo sapiens
1777	11728063_	turquoise	SH3 domain binding protein 1(SH3BP1)	Homo sapiens
1778	11728077_	turquoise	protein kinase C theta(PRKCQ)	Homo sapiens
1779	11728093_	turquoise	mitogen-activated protein kinase kinase kinase 1(MAP3K1)	Homo sapiens
1780	11728094_	turquoise	mitogen-activated protein kinase kinase kinase 1(MAP3K1)	Homo sapiens
1781	11728125_	turquoise	granzyme H(GZMH)	Homo sapiens
1782	11728137_	turquoise	X-linked Kx blood group(XK)	Homo sapiens
1783	11728146_	turquoise	aquaporin 11(AQP11)	Homo sapiens
1784	11728152_	brown	histone cluster 1 H2B family member d(HIST1H2BD)	Homo sapiens
1785	11728154_	turquoise	fucosyltransferase 4(FUT4)	Homo sapiens
1786	11728157_	turquoise	ectodysplasin A(EDA)	Homo sapiens
1787	11728178_	turquoise	sirtuin 4(SIRT4)	Homo sapiens
1788	11728189_	turquoise	C-X-C motif chemokine receptor 4(CXCR4)	Homo sapiens
1789	11728190_	turquoise	C-X-C motif chemokine receptor 4(CXCR4)	Homo sapiens
1790	11728191_	turquoise	C-X-C motif chemokine receptor 4(CXCR4)	Homo sapiens
1791	11728207_	turquoise	XPC complex subunit, DNA damage recognition and repair factor(XPC)	Homo sapiens
1792	11728223_	turquoise	ubiquitin conjugating enzyme E2 J1(UBE2J1)	Homo sapiens
1793	11728224_	turquoise	ubiquitin conjugating enzyme E2 J1(UBE2J1)	Homo sapiens
1794	11728225_	turquoise	ubiquitin conjugating enzyme E2 J1(UBE2J1)	Homo sapiens
1795	11728228_	turquoise	hepatitis A virus cellular receptor 2(HAVCR2)	Homo sapiens
1796	11728229_	turquoise	hepatitis A virus cellular receptor 2(HAVCR2)	Homo sapiens
1797	11728236_	turquoise	CD5 molecule(CD5)	Homo sapiens
1798	11728245_	turquoise	angiopoietin 2(ANGPT2)	Homo sapiens

1799	11728265_	turquoise	leukocyte immunoglobulin like receptor B2(LILRB2)	Homo sapiens
1800	11728266_	turquoise	leukocyte immunoglobulin like receptor B2(LILRB2)	Homo sapiens
1801	11728267_	turquoise	leukocyte immunoglobulin like receptor B2(LILRB2)	Homo sapiens
1802	11728276_	turquoise	phospholipase A2 group XIIA(PLA2G12A)	Homo sapiens
1803	11728277_	turquoise	phospholipase A2 group XIIA(PLA2G12A)	Homo sapiens
1804	11728278_	turquoise	phospholipase A2 group XIIA(PLA2G12A)	Homo sapiens
1805	11728279_	turquoise	phospholipase A2 group XIIA(PLA2G12A)	Homo sapiens
1806	11728291_	turquoise	SH2 domain containing 1A(SH2D1A)	Homo sapiens
1807	11728292_	turquoise	SH2 domain containing 1A(SH2D1A)	Homo sapiens
1808	11728294_	brown	mitochondrial ribosomal protein L58(MRPL58)	Homo sapiens
1809	11728311_	brown	ERCC excision repair 1, endonuclease non-catalytic subunit(ERCC1)	Homo sapiens
1810	11728317_	brown	family with sequence similarity 122A(FAM122A)	Homo sapiens
1811	11728347_	turquoise	ABI family member 3(ABI3)	Homo sapiens
1812	11728350_	turquoise	stromal interaction molecule 2(STIM2)	Homo sapiens
1813	11728373_	turquoise	ERG, ETS transcription factor(ERG)	Homo sapiens
1814	11728374_	turquoise	actinin alpha 1(ACTN1)	Homo sapiens
1815	11728375_	turquoise	actinin alpha 1(ACTN1)	Homo sapiens
1816	11728378_	turquoise	embryonic ectoderm development(EED)	Homo sapiens
1817	11728392_	blue	LDL receptor related protein 12(LRP12)	Homo sapiens
1818	11728396_	turquoise	sorting nexin 14(SNX14)	Homo sapiens
1819	11728401_	turquoise	ATPase H+ transporting V0 subunit a2(ATP6V0A2)	Homo sapiens
1820	11728405_	turquoise	negative regulator of reactive oxygen species(NRROS)	Homo sapiens
1821	11728413_	turquoise	serpin family B member 8(SERPINB8)	Homo sapiens
1822	11728421_	turquoise	phospholipase A2 group VII(PLA2G7)	Homo sapiens
1823	11728423_	turquoise	paired immunoglobin like type 2 receptor alpha(PILRA)	Homo sapiens
1824	11728424_	turquoise	paired immunoglobin like type 2 receptor alpha(PILRA)	Homo sapiens
1825	11728425_	brown	bolA family member 3(BOLA3)	Homo sapiens
1826	11728430_	turquoise	hes related family bHLH transcription factor with YRPW motif 2(HEY2)	Homo sapiens
1827	11728446_	turquoise	phospholipase C eta 1(PLCH1)	Homo sapiens
1828	11728489_	turquoise	C-type lectin domain family 4 member A(CLEC4A)	Homo sapiens
1829	11728503_	turquoise	CD300c molecule(CD300C)	Homo sapiens
1830	11728507_	turquoise	lymphocyte antigen 96(LY96)	Homo sapiens
1831	11728512_	turquoise	TNF receptor associated factor 5(TRAF5)	Homo sapiens
1832	11728513_	brown	NADH:ubiquinone oxidoreductase complex assembly factor 6(NDUFAF6)	Homo sapiens
1833	11728514_	turquoise	NADH:ubiquinone oxidoreductase complex assembly factor 6(NDUFAF6)	Homo sapiens
1834	11728515_	turquoise	SP140 nuclear body protein(SP140)	Homo sapiens
1835	11728523_	turquoise	v-myc avian myelocytomatosis viral oncogene lung carcinoma derived homolog(MYCL)	Homo sapiens
1836	11728530_	turquoise	B-cell linker(BLNK)	Homo sapiens
1837	11728543_	turquoise	maestro(MRO)	Homo sapiens
1838	11728560_	turquoise	granzyme K(GZMK)	Homo sapiens
1839	11728565_	turquoise	family with sequence similarity 210 member A(FAM210A)	Homo sapiens
1840	11728566_	blue	family with sequence similarity 210 member A(FAM210A)	Homo sapiens
1841	11728570_	turquoise	zinc finger protein, FOG family member 2(ZFPM2)	Homo sapiens
1842	11728592_	turquoise	leucine rich repeat containing 70(LRRC70)	Homo sapiens
1843	11728595_	turquoise	caveolin 3(CAV3)	Homo sapiens
1844	11728617_	turquoise	caspase recruitment domain family member 6(CARD6)	Homo sapiens
1845	11728640_	turquoise	EDAR associated death domain(EDARADD)	Homo sapiens
1846	11728643_	turquoise	ARP3 actin related protein 3 homolog(ACTR3)	Homo sapiens
1847	11728673_	brown	kinesin family member 22(KIF22)	Homo sapiens
1848	11728676_	turquoise	mediator complex subunit 14(MED14)	Homo sapiens

1849	11728679_ turquoise	CD163 molecule(CD163)	Homo sapiens
1850	11728680_ turquoise	poly(A) binding protein interacting protein 1(PAIP1)	Homo sapiens
1851	11728702_ turquoise	KIAA0232(KIAA0232)	Homo sapiens
1852	11728706_ turquoise	epithelial membrane protein 2(EMP2)	Homo sapiens
1853	11728711_ turquoise	nuclear factor of activated T-cells 2 interacting protein(NFATC2IP)	Homo sapiens
1854	11728720_ turquoise	ras homolog family member H(RHOH)	Homo sapiens
1855	11728741_ brown	coenzyme Q3, methyltransferase(COQ3)	Homo sapiens
1856	11728749_ turquoise	transcription factor 19(TCF19)	Homo sapiens
1857	11728761_ turquoise	uncharacterized LOC100506403(LOC100506403)	Homo sapiens
1858	11728766_ turquoise	kelch like family member 5(KLHL5)	Homo sapiens
1859	11728767_ turquoise	kelch like family member 5(KLHL5)	Homo sapiens
1860	11728768_ turquoise	kelch like family member 5(KLHL5)	Homo sapiens
1861	11728776_ turquoise	vitamin D (1,25- dihydroxyvitamin D3) receptor(VDR)	Homo sapiens
1862	11728780_ turquoise	zeta chain of T cell receptor associated protein kinase 70(ZAP70)	Homo sapiens
1863	11728781_ turquoise	CD1c molecule(CD1C)	Homo sapiens
1864	11728810_ turquoise	enoyl-CoA hydratase domain containing 3(ECHDC3)	Homo sapiens
1865	11728811_ turquoise	OTU deubiquitinase 7B(OTUD7B)	Homo sapiens
1866	11728823_ blue	solute carrier family 25 member 3(SLC25A3)	Homo sapiens
1867	11728824_ blue	solute carrier family 25 member 3(SLC25A3)	Homo sapiens
1868	11728841_ blue	pleckstrin homology domain containing A5(PLEKHA5)	Homo sapiens
1869	11728846_ turquoise	heart and neural crest derivatives expressed 1(HAND1)	Homo sapiens
1870	11728852_ turquoise	tubby bipartite transcription factor(TUB)	Homo sapiens
1871	11728863_ turquoise	GINS complex subunit 1(GINS1)	Homo sapiens
1872	11728892_ turquoise	tubulin polymerization promoting protein(TPPP)	Homo sapiens
1873	11728901_ turquoise	dual adaptor of phosphotyrosine and 3-phosphoinositides 1(DAPP1)	Homo sapiens
1874	11728920_ turquoise	RAS guanyl releasing protein 1(RASGRP1)	Homo sapiens
1875	11728926_ turquoise	NFAT activating protein with ITAM motif 1(NFAM1)	Homo sapiens
1876	11728937_ turquoise	GDNF family receptor alpha 2(GFRA2)	Homo sapiens
1877	11728944_ turquoise	leukocyte specific transcript 1(LST1)	Homo sapiens
1878	11728945_ turquoise	leukocyte specific transcript 1(LST1)	Homo sapiens
1879	11728961_ turquoise	TSNAX-DISC1 readthrough (NMD candidate)(TSNAX-DISC1)	Homo sapiens
1880	11728978_ turquoise	caspase 8(CASP8)	Homo sapiens
1881	11728984_ turquoise	monofunctional C1-tetrahydrofolate synthase, mitochondrial-like(LOC100996643)	Homo sapiens
1882	11728985_ turquoise	monofunctional C1-tetrahydrofolate synthase, mitochondrial-like(LOC100996643)	Homo sapiens
1883	11728986_ turquoise	monofunctional C1-tetrahydrofolate synthase, mitochondrial-like(LOC100996643)	Homo sapiens
1884	11729000_ turquoise	CD84 molecule(CD84)	Homo sapiens
1885	11729023_ turquoise	potassium voltage-gated channel subfamily A member 5(KCNA5)	Homo sapiens
1886	11729033_ turquoise	inositol 1,4,5-trisphosphate receptor interacting protein like 2(ITPRIPL2)	Homo sapiens
1887	11729035_ turquoise	inositol 1,4,5-trisphosphate receptor interacting protein like 2(ITPRIPL2)	Homo sapiens
1888	11729074_ turquoise	hydroxylysine kinase(HYKK)	Homo sapiens
1889	11729101_ turquoise	aldo-keto reductase family 1 member C2(AKR1C2)	Homo sapiens
1890	11729110_ turquoise	ADAM like decysin 1(ADAMDEC1)	Homo sapiens
1891	11729111_ turquoise	ADAM like decysin 1(ADAMDEC1)	Homo sapiens
1892	11729121_ brown	McKusick-Kaufman syndrome(MKKS)	Homo sapiens
1893	11729122_ brown	McKusick-Kaufman syndrome(MKKS)	Homo sapiens
1894	11729126_ blue	solute carrier family 25 member 3(SLC25A3)	Homo sapiens
1895	11729127_ blue	solute carrier family 25 member 3(SLC25A3)	Homo sapiens
1896	11729131_ brown	proteasome activator subunit 3(PSME3)	Homo sapiens
1897	11729138_ turquoise	SLAM family member 7(SLAMF7)	Homo sapiens
1898	11729139_ turquoise	SLAM family member 7(SLAMF7)	Homo sapiens

1899	11729140_ turquoise	SLAM family member 7(SLAMF7)	Homo sapiens
1900	11729149_ brown	mitochondrial ribosomal protein S23(MRPS23)	Homo sapiens
1901	11729163_ turquoise	BTG anti-proliferation factor 3(BTG3)	Homo sapiens
1902	11729191_ turquoise	MYC associated factor X(MAX)	Homo sapiens
1903	11729202_ turquoise	Kazal type serine peptidase inhibitor domain 1(KAZALD1)	Homo sapiens
1904	11729203_ turquoise	plexin D1(PLXND1)	Homo sapiens
1905	11729219_ turquoise	NCK adaptor protein 1(NCK1)	Homo sapiens
1906	11729220_ turquoise	carboxymethylenebutenolidase homolog(CMBL)	Homo sapiens
1907	11729222_ turquoise	GATA binding protein 3(GATA3)	Homo sapiens
1908	11729223_ turquoise	GATA binding protein 3(GATA3)	Homo sapiens
1909	11729226_ turquoise	integrin subunit alpha L(ITGAL)	Homo sapiens
1910	11729237_ blue	methylcrotonoyl-CoA carboxylase 2(MCCC2)	Homo sapiens
1911	11729247_ turquoise	sirtuin 5(SIRT5)	Homo sapiens
1912	11729251_ turquoise	myeloid cell nuclear differentiation antigen(MNDA)	Homo sapiens
1913	11729263_ turquoise	serpin family B member 9(SERPINB9)	Homo sapiens
1914	11729272_ turquoise	ankyrin repeat and SOCS box containing 1(ASB1)	Homo sapiens
1915	11729274_ turquoise	guanylate binding protein 5(GBP5)	Homo sapiens
1916	11729311_ brown	spexin hormone(SPX)	Homo sapiens
1917	11729312_ blue	nicotinamide nucleotide adenylyltransferase 1(NMNAT1)	Homo sapiens
1918	11729338_ turquoise	proline-serine-threonine phosphatase interacting protein 2(PSTPIP2)	Homo sapiens
1919	11729357_ turquoise	poly(A) RNA polymerase D4, non-canonical(PAPD4)	Homo sapiens
1920	11729410_ turquoise	zinc finger protein 415(ZNF415)	Homo sapiens
1921	11729419_ turquoise	ATPase sarcoplasmic/endoplasmic reticulum Ca2+ transporting 3(ATP2A3)	Homo sapiens
1922	11729422_ brown	popeye domain containing 3(POPDC3)	Homo sapiens
1923	11729424_ turquoise	C-C motif chemokine receptor like 2(CCRL2)	Homo sapiens
1924	11729431_ turquoise	phosphatase domain containing, paladin 1(PALD1)	Homo sapiens
1925	11729442_ turquoise	TEPSIN, adaptor related protein complex 4 accessory protein(TEPSIN)	Homo sapiens
1926	11729443_ turquoise	promyelocytic leukemia(PML)	Homo sapiens
1927	11729449_ turquoise	TERF1 interacting nuclear factor 2(TINF2)	Homo sapiens
1928	11729458_ turquoise	Fanconi anemia complementation group I(FANCI)	Homo sapiens
1929	11729479_ turquoise	C-type lectin domain family 12 member A(CLEC12A)	Homo sapiens
1930	11729481_ turquoise	guanylate kinase 1(GUK1)	Homo sapiens
1931	11729485_ turquoise	regulator of G-protein signaling 6(RGS6)	Homo sapiens
1932	11729487_ turquoise	regulator of G-protein signaling 6(RGS6)	Homo sapiens
1933	11729520_ turquoise	chromosome X open reading frame 65(CXorf65)	Homo sapiens
1934	11729523_ turquoise	NLR family CARD domain containing 5(NLRC5)	Homo sapiens
1935	11729542_ turquoise	calcium/calmodulin dependent protein kinase kinase 2(CAMKK2)	Homo sapiens
1936	11729570_ turquoise	mitogen-activated protein kinase kinase kinase kinase 1(MAP4K1)	Homo sapiens
1937	11729574_ turquoise	ectodermal-neural cortex 1(ENC1)	Homo sapiens
1938	11729603_ turquoise	minichromosome maintenance complex component 7(MCM7)	Homo sapiens
1939	11729610_ turquoise	engulfment and cell motility 1(ELMO1)	Homo sapiens
1940	11729611_ turquoise	actin related protein 2/3 complex subunit 4(ARPC4)	Homo sapiens
1941	11729619_ turquoise	chloride voltage-gated channel 4(CLCN4)	Homo sapiens
1942	11729620_ turquoise	chloride voltage-gated channel 4(CLCN4)	Homo sapiens
1943	11729621_ blue	chloride voltage-gated channel 4(CLCN4)	Homo sapiens
1944	11729622_ turquoise	chloride voltage-gated channel 4(CLCN4)	Homo sapiens
1945	11729649_ turquoise	perforin 1(PRF1)	Homo sapiens
1946	11729657_ turquoise	SP110 nuclear body protein(SP110)	Homo sapiens
1947	11729658_ turquoise	SP110 nuclear body protein(SP110)	Homo sapiens
1948	11729659_ blue	transmembrane protein 69(TMEM69)	Homo sapiens

1949	11729667_	turquoise	nucleolar protein 3(NOL3)	Homo sapiens
1950	11729669_	brown	eukaryotic translation initiation factor 3 subunit K(EIF3K)	Homo sapiens
1951	11729676_	turquoise	cyclin dependent kinase 17(CDK17)	Homo sapiens
1952	11729687_	turquoise	LYR motif containing 7(LYRM7)	Homo sapiens
1953	11729689_	turquoise	LYR motif containing 7(LYRM7)	Homo sapiens
1954	11729721_	turquoise	leukocyte immunoglobulin like receptor B3(LILRB3)	Homo sapiens
1955	11729741_	turquoise	CD68 molecule(CD68)	Homo sapiens
1956	11729747_	turquoise	interleukin 10 receptor subunit alpha(IL10RA)	Homo sapiens
1957	11729752_	turquoise	epidermal growth factor receptor pathway substrate 15 like 1(EPS15L1)	Homo sapiens
1958	11729754_	turquoise	unc-51 like autophagy activating kinase 2(ULK2)	Homo sapiens
1959	11729758_	turquoise	regulator of G-protein signaling 18(RGS18)	Homo sapiens
1960	11729759_	turquoise	regulator of G-protein signaling 18(RGS18)	Homo sapiens
1961	11729769_	turquoise	CD38 molecule(CD38)	Homo sapiens
1962	11729807_	brown	cytochrome c oxidase subunit 6B1(COX6B1)	Homo sapiens
1963	11729821_	turquoise	interleukin 27 receptor subunit alpha(IL27RA)	Homo sapiens
1964	11729851_	turquoise	Rap associating with DIL domain(RADIL)	Homo sapiens
1965	11729855_	turquoise	CD3e molecule(CD3E)	Homo sapiens
1966	11729886_	turquoise	chromosome 18 open reading frame 54(C18orf54)	Homo sapiens
1967	11729889_	turquoise	ZFP3 zinc finger protein(ZFP3)	Homo sapiens
1968	11729924_	turquoise	tet methylcytosine dioxygenase 3(TET3)	Homo sapiens
1969	11729954_	turquoise	fragile histidine triad(FHIT)	Homo sapiens
1970	11729955_	turquoise	fragile histidine triad(FHIT)	Homo sapiens
1971	11729976_	turquoise	protein tyrosine phosphatase, receptor type N2(PTPRN2)	Homo sapiens
1972	11729977_	turquoise	C-X-C motif chemokine receptor 6(CXCR6)	Homo sapiens
1973	11729980_	turquoise	phosphatidylinositol specific phospholipase C X domain containing 3(PLCXD3)	Homo sapiens
1974	11729981_	turquoise	poly(ADP-ribose) polymerase family member 11(PARP11)	Homo sapiens
1975	11729996_	turquoise	schlafen family member 12(SLFN12)	Homo sapiens
1976	11730005_	turquoise	semaphorin 4D(SEMA4D)	Homo sapiens
1977	11730010_	blue	microtubule associated tumor suppressor candidate 2(MTUS2)	Homo sapiens
1978	11730044_	brown	chromosome 9 open reading frame 24(C9orf24)	Homo sapiens
1979	11730060_	turquoise	lymphocyte activating 3(LAG3)	Homo sapiens
1980	11730067_	turquoise	granzyme M(GZMM)	Homo sapiens
1981	11730090_	turquoise	TNFSF12-TNFSF13 readthrough(TNFSF12-TNFSF13)	Homo sapiens
1982	11730096_	turquoise	BCL2 related protein A1(BCL2A1)	Homo sapiens
1983	11730099_	turquoise	fms related tyrosine kinase 3 ligand(FLT3LG)	Homo sapiens
1984	11730100_	turquoise	fms related tyrosine kinase 3 ligand(FLT3LG)	Homo sapiens
1985	11730109_	turquoise	NLR family CARD domain containing 3(NLRC3)	Homo sapiens
1986	11730145_	turquoise	kelch like family member 31(KLHL31)	Homo sapiens
1987	11730146_	blue	kelch like family member 31(KLHL31)	Homo sapiens
1988	11730148_	turquoise	adhesion G protein-coupled receptor G5(ADGRG5)	Homo sapiens
1989	11730155_	turquoise	sarcoglycan delta(SGCD)	Homo sapiens
1990	11730159_	blue	RNA binding motif protein 20(RBM20)	Homo sapiens
1991	11730168_	turquoise	lipopolysaccharide induced TNF factor(LITAF)	Homo sapiens
1992	11730186_	brown	isocitrate dehydrogenase 3 (NAD(+)) beta(IDH3B)	Homo sapiens
1993	11730190_	turquoise	Obg like ATPase 1(OLA1)	Homo sapiens
1994	11730191_	turquoise	SEC14 like lipid binding 2(SEC14L2)	Homo sapiens
1995	11730207_	turquoise	ArfGAP with RhoGAP domain, ankyrin repeat and PH domain 2(ARAP2)	Homo sapiens
1996	11730212_	blue	mitochondrial ribosomal protein S7(MRPS7)	Homo sapiens
1997	11730213_	blue	mitochondrial ribosomal protein S7(MRPS7)	Homo sapiens
1998	11730217_	turquoise	ceramide synthase 5(CERS5)	Homo sapiens

1999	11730218_	turquoise	ceramide synthase 5(CERS5)	Homo sapiens
2000	11730231_	turquoise	TIA1 cytotoxic granule associated RNA binding protein like 1(TIAL1)	Homo sapiens
2001	11730237_	turquoise	abhydrolase domain containing 10(ABHD10)	Homo sapiens
2002	11730244_	turquoise	flavin containing monooxygenase 2(FMO2)	Homo sapiens
2003	11730247_	turquoise	stromal antigen 3(STAG3)	Homo sapiens
2004	11730250_	turquoise	ligand of numb-protein X 1(LNX1)	Homo sapiens
2005	11730251_	turquoise	ligand of numb-protein X 1(LNX1)	Homo sapiens
2006	11730254_	turquoise	ELK3, ETS transcription factor(ELK3)	Homo sapiens
2007	11730259_	turquoise	zinc finger C2HC-type containing 1C(ZC2HC1C)	Homo sapiens
2008	11730269_	turquoise	NIPBL, cohesin loading factor(NIPBL)	Homo sapiens
2009	11730274_	turquoise	calcitonin receptor like receptor(CALCRL)	Homo sapiens
2010	11730296_	turquoise	toll like receptor 3(TLR3)	Homo sapiens
2011	11730301_	turquoise	lipoic acid synthetase(LIAS)	Homo sapiens
2012	11730322_	turquoise	C-type lectin domain family 2 member D(CLEC2D)	Homo sapiens
2013	11730342_	turquoise	Src like adaptor 2(SLA2)	Homo sapiens
2014	11730372_	turquoise	FYN binding protein(FYB)	Homo sapiens
2015	11730373_	turquoise	FYN binding protein(FYB)	Homo sapiens
2016	11730374_	turquoise	FYN binding protein(FYB)	Homo sapiens
2017	11730399_	turquoise	AT-rich interaction domain 3A(ARID3A)	Homo sapiens
2018	11730403_	turquoise	MRE11 homolog, double strand break repair nuclease(MRE11)	Homo sapiens
2019	11730424_	turquoise	APC down-regulated 1 like(APCDD1L)	Homo sapiens
2020	11730428_	turquoise	adaptor related protein complex 1 beta 1 subunit(AP1B1)	Homo sapiens
2021	11730451_	brown	succinate dehydrogenase complex assembly factor 4(SDHAF4)	Homo sapiens
2022	11730457_	turquoise	absent in melanoma 2(AIM2)	Homo sapiens
2023	11730458_	turquoise	absent in melanoma 2(AIM2)	Homo sapiens
2024	11730469_	turquoise	LIM domain only 2(LMO2)	Homo sapiens
2025	11730478_	turquoise	ring finger protein 125(RNF125)	Homo sapiens
2026	11730542_	blue	acetyl-CoA acyltransferase 1(ACAA1)	Homo sapiens
2027	11730555_	turquoise	TRIM6-TRIM34 readthrough(TRIM6-TRIM34)	Homo sapiens
2028	11730556_	turquoise	TRIM6-TRIM34 readthrough(TRIM6-TRIM34)	Homo sapiens
2029	11730600_	turquoise	putative aquaporin-7-like protein 3(LOC100509620)	Homo sapiens
2030	11730611_	turquoise	mucolipin 2(MCOLN2)	Homo sapiens
2031	11730637_	turquoise	cytotoxic T-lymphocyte associated protein 4(CTLA4)	Homo sapiens
2032	11730659_	brown	3-oxoacyl-ACP synthase, mitochondrial(OXSM)	Homo sapiens
2033	11730690_	turquoise	family with sequence similarity 111 member A(FAM111A)	Homo sapiens
2034	11730729_	turquoise	transporter 1, ATP binding cassette subfamily B member(TAP1)	Homo sapiens
2035	11730752_	turquoise	1-acylglycerol-3-phosphate O-acyltransferase 5(AGPAT5)	Homo sapiens
2036	11730754_	turquoise	1-acylglycerol-3-phosphate O-acyltransferase 5(AGPAT5)	Homo sapiens
2037	11730779_	turquoise	alcohol dehydrogenase 1C (class I), gamma polypeptide(ADH1C)	Homo sapiens
2038	11730780_	turquoise	alcohol dehydrogenase 1C (class I), gamma polypeptide(ADH1C)	Homo sapiens
2039	11730782_	turquoise	solute carrier family 2 member 6(SLC2A6)	Homo sapiens
2040	11730784_	blue	origin recognition complex subunit 4(ORC4)	Homo sapiens
2041	11730795_	turquoise	mitochondrial ribosomal protein L4(MRPL4)	Homo sapiens
2042	11730804_	turquoise	pre-mRNA processing factor 38B(PRPF38B)	Homo sapiens
2043	11730806_	turquoise	butyrophilin subfamily 3 member A2(BTN3A2)	Homo sapiens
2044	11730807_	turquoise	butyrophilin subfamily 3 member A2(BTN3A2)	Homo sapiens
2045	11730813_	turquoise	leukocyte immunoglobulin like receptor B4(LILRB4)	Homo sapiens
2046	11730815_	turquoise	leukocyte immunoglobulin like receptor B4(LILRB4)	Homo sapiens
2047	11730817_	turquoise	CTD small phosphatase like 2(CTDSPL2)	Homo sapiens
2048	11730835_	blue	metaxin 2(MTX2)	Homo sapiens

2049	11730837_turquoise	WD repeat domain 55(WDR55)	Homo sapiens
2050	11730862_turquoise	CTTNBP2 N-terminal like(CTTNBP2NL)	Homo sapiens
2051	11730864_turquoise	CTTNBP2 N-terminal like(CTTNBP2NL)	Homo sapiens
2052	11730873_turquoise	Ras association domain family member 5(RASSF5)	Homo sapiens
2053	11730875_turquoise	Fanconi anemia complementation group I(FANCI)	Homo sapiens
2054	11730894_turquoise	ubiquitin like modifier activating enzyme 6(UBA6)	Homo sapiens
2055	11730909_turquoise	C-C motif chemokine receptor 5 (gene/pseudogene)(CCR5)	Homo sapiens
2056	11730914_turquoise	ER lipid raft associated 1(ERLIN1)	Homo sapiens
2057	11730923_turquoise	ADAM metallopeptidase with thrombospondin type 1 motif 4(ADAMTS4)	Homo sapiens
2058	11730928_turquoise	inhibitor of kappa light polypeptide gene enhancer in B-cells, kinase beta(IKBKB)	Homo sapiens
2059	11730929_turquoise	inhibitor of kappa light polypeptide gene enhancer in B-cells, kinase beta(IKBKB)	Homo sapiens
2060	11730930_turquoise	inhibitor of kappa light polypeptide gene enhancer in B-cells, kinase beta(IKBKB)	Homo sapiens
2061	11730931_turquoise	acyl-CoA synthetase short-chain family member 3(ACSS3)	Homo sapiens
2062	11730932_turquoise	acyl-CoA synthetase short-chain family member 3(ACSS3)	Homo sapiens
2063	11730935_turquoise	AT-hook transcription factor(AKNA)	Homo sapiens
2064	11730936_turquoise	AT-hook transcription factor(AKNA)	Homo sapiens
2065	11730943_turquoise	osteoclast associated, immunoglobulin-like receptor(OSCAR)	Homo sapiens
2066	11730947_turquoise	interferon gamma(IFNG)	Homo sapiens
2067	11730958_blue	coiled-coil-helix-coiled-coil-helix domain containing 4(CHCHD4)	Homo sapiens
2068	11730962_turquoise	choline/ethanolamine phosphotransferase 1(CEPT1)	Homo sapiens
2069	11730966_turquoise	mitogen-activated protein kinase 11(MAPK11)	Homo sapiens
2070	11730968_turquoise	RIC8 guanine nucleotide exchange factor B(RIC8B)	Homo sapiens
2071	11730994_turquoise	sphingosine-1-phosphate receptor 4(S1PR4)	Homo sapiens
2072	11730996_turquoise	junction adhesion molecule like(JAML)	Homo sapiens
2073	11731004_turquoise	MIS18 binding protein 1(MIS18BP1)	Homo sapiens
2074	11731005_turquoise	MIS18 binding protein 1(MIS18BP1)	Homo sapiens
2075	11731006_turquoise	MIS18 binding protein 1(MIS18BP1)	Homo sapiens
2076	11731019_turquoise	allograft inflammatory factor 1(AIF1)	Homo sapiens
2077	11731023_turquoise	ataxin 7(ATXN7)	Homo sapiens
2078	11731024_turquoise	ataxin 7(ATXN7)	Homo sapiens
2079	11731036_turquoise	fibrinogen C domain containing 1(FIBCD1)	Homo sapiens
2080	11731039_turquoise	interleukin 15 receptor subunit alpha(IL15RA)	Homo sapiens
2081	11731066_turquoise	GTPase, IMAP family member 1(GIMAP1)	Homo sapiens
2082	11731110_turquoise	ADAM metallopeptidase domain 17(ADAM17)	Homo sapiens
2083	11731147_turquoise	toll like receptor 7(TLR7)	Homo sapiens
2084	11731148_turquoise	toll like receptor 7(TLR7)	Homo sapiens
2085	11731181_turquoise	epithelial stromal interaction 1(EPSTI1)	Homo sapiens
2086	11731196_turquoise	proline rich 5 like(PRR5L)	Homo sapiens
2087	11731197_turquoise	proline rich 5 like(PRR5L)	Homo sapiens
2088	11731200_turquoise	toll like receptor 5(TLR5)	Homo sapiens
2089	11731209_turquoise	chromosome 15 open reading frame 59(C15orf59)	Homo sapiens
2090	11731227_turquoise	megakaryocyte-associated tyrosine kinase(MATK)	Homo sapiens
2091	11731230_turquoise	RNA pseudouridylate synthase domain containing 3(RPUSD3)	Homo sapiens
2092	11731248_brown	transmembrane protein 116(TMEM116)	Homo sapiens
2093	11731281_turquoise	nuclear factor of activated T-cells 1(NFATC1)	Homo sapiens
2094	11731309_turquoise	chromosome 15 open reading frame 56(C15orf56)	Homo sapiens
2095	11731340_turquoise	nucleotide binding oligomerization domain containing 2(NOD2)	Homo sapiens
2096	11731346_turquoise	diacylglycerol kinase zeta(DGKZ)	Homo sapiens
2097	11731356_turquoise	inositol hexakisphosphate kinase 3(IP6K3)	Homo sapiens
2098	11731360_turquoise	inducible T-cell costimulator(ICOS)	Homo sapiens

2099	11731394_ turquoise	vimentin(VIM)	Homo sapiens
2100	11731407_ turquoise	interferon induced protein with tetratricopeptide repeats 3(IFIT3)	Homo sapiens
2101	11731417_ blue	NADH:ubiquinone oxidoreductase core subunit S1(NDUFS1)	Homo sapiens
2102	11731422_ turquoise	Fc fragment of IgG receptor IIIa(FCGR3A)	Homo sapiens
2103	11731433_ turquoise	selenoprotein P(SELENOP)	Homo sapiens
2104	11731465_ turquoise	cathepsin C(CTSC)	Homo sapiens
2105	11731466_ turquoise	cathepsin C(CTSC)	Homo sapiens
2106	11731485_ turquoise	nuclear factor I C(NFIC)	Homo sapiens
2107	11731504_ turquoise	chromosome 22 open reading frame 39(C22orf39)	Homo sapiens
2108	11731505_ turquoise	chromosome 22 open reading frame 39(C22orf39)	Homo sapiens
2109	11731518_ turquoise	microfibrillar associated protein 3(MFAP3)	Homo sapiens
2110	11731531_ turquoise	docking protein 1(DOK1)	Homo sapiens
2111	11731555_ turquoise	synaptopodin 2(SYNPO2)	Homo sapiens
2112	11731556_ turquoise	synaptopodin 2(SYNPO2)	Homo sapiens
2113	11731557_ blue	synaptopodin 2(SYNPO2)	Homo sapiens
2114	11731575_ turquoise	chitobiase(CTBS)	Homo sapiens
2115	11731613_ turquoise	Ras homolog enriched in brain like 1(RHEBL1)	Homo sapiens
2116	11731614_ blue	electron transfer flavoprotein alpha subunit(ETFA)	Homo sapiens
2117	11731617_ turquoise	SID1 transmembrane family member 1(SIDT1)	Homo sapiens
2118	11731620_ turquoise	arrestin beta 2(ARRB2)	Homo sapiens
2119	11731641_ turquoise	trafficking protein particle complex subunit 10-like(LOC102724200)	Homo sapiens
2120	11731644_ turquoise	trafficking protein particle complex subunit 10-like(LOC102724200)	Homo sapiens
2121	11731657_ turquoise	vav guanine nucleotide exchange factor 3(VAV3)	Homo sapiens
2122	11731667_ turquoise	platelet derived growth factor subunit B(PDGFB)	Homo sapiens
2123	11731669_ turquoise	G protein-coupled receptor 65(GPR65)	Homo sapiens
2124	11731671_ brown	FUN14 domain containing 1(FUNDC1)	Homo sapiens
2125	11731676_ turquoise	C-C motif chemokine receptor 2(CCR2)	Homo sapiens
2126	11731702_ turquoise	C-type lectin domain family 2 member D(CLEC2D)	Homo sapiens
2127	11731722_ turquoise	aldehyde dehydrogenase 3 family member B1(ALDH3B1)	Homo sapiens
2128	11731728_ turquoise	purinergic receptor P2Y13(P2RY13)	Homo sapiens
2129	11731729_ turquoise	purinergic receptor P2Y13(P2RY13)	Homo sapiens
2130	11731755_ turquoise	synaptotagmin like 2(SYTL2)	Homo sapiens
2131	11731764_ turquoise	CD3g molecule(CD3G)	Homo sapiens
2132	11731770_ turquoise	dermatan sulfate epimerase(DSE)	Homo sapiens
2133	11731777_ turquoise	protein O-linked mannose N-acetylglucosaminyltransferase 2 (beta 1,4-)(POMGNT2)	Homo sapiens
2134	11731783_ turquoise	RAS protein activator like 3(RASAL3)	Homo sapiens
2135	11731798_ turquoise	galectin 2(LGALS2)	Homo sapiens
2136	11731799_ turquoise	urocortin 3(UCN3)	Homo sapiens
2137	11731818_ turquoise	CD40 molecule(CD40)	Homo sapiens
2138	11731820_ turquoise	ADAM metallopeptidase domain 11(ADAM11)	Homo sapiens
2139	11731821_ turquoise	ADAM metallopeptidase domain 11(ADAM11)	Homo sapiens
2140	11731845_ turquoise	immunoglobulin superfamily member 3(IGSF3)	Homo sapiens
2141	11731848_ turquoise	killer cell lectin like receptor C1(KLRC1)	Homo sapiens
2142	11731873_ turquoise	CD209 molecule(CD209)	Homo sapiens
2143	11731879_ turquoise	NLR family pyrin domain containing 1(NLRP1)	Homo sapiens
2144	11731880_ turquoise	NLR family pyrin domain containing 1(NLRP1)	Homo sapiens
2145	11731914_ turquoise	adhesion molecule with Ig like domain 1(AMIGO1)	Homo sapiens
2146	11731932_ blue	KN motif and ankyrin repeat domains 1(KANK1)	Homo sapiens
2147	11731942_ turquoise	PR/SET domain 1(PRDM1)	Homo sapiens
2148	11731949_ turquoise	sterile alpha motif domain containing 3(SAMD3)	Homo sapiens

2149	11731950_ turquoise	sterile alpha motif domain containing 3(SAMD3)	Homo sapiens
2150	11731978_ turquoise	proprotein convertase subtilisin/kexin type 7(PCSK7)	Homo sapiens
2151	11731998_ turquoise	coiled-coil domain containing 88C(CCDC88C)	Homo sapiens
2152	11732004_ turquoise	adenosine deaminase like(ADAL)	Homo sapiens
2153	11732006_ turquoise	adenosine deaminase like(ADAL)	Homo sapiens
2154	11732017_ turquoise	interleukin 18 receptor accessory protein(IL18RAP)	Homo sapiens
2155	11732058_ turquoise	cell division cycle 7(CDC7)	Homo sapiens
2156	11732084_ turquoise	transcription factor EC(TFEC)	Homo sapiens
2157	11732110_ turquoise	interleukin 4 induced 1(IL4I1)	Homo sapiens
2158	11732153_ turquoise	zinc finger DHHC-type containing 6(ZDHHC6)	Homo sapiens
2159	11732155_ turquoise	O-linked N-acetylglucosamine (GlcNAc) transferase(OGT)	Homo sapiens
2160	11732169_ turquoise	protein tyrosine phosphatase, non-receptor type 7(PTPN7)	Homo sapiens
2161	11732171_ turquoise	G-rich RNA sequence binding factor 1(GRSF1)	Homo sapiens
2162	11732180_ turquoise	platelet activating factor receptor(PTAFR)	Homo sapiens
2163	11732181_ turquoise	platelet activating factor receptor(PTAFR)	Homo sapiens
2164	11732193_ blue	myeloid leukemia factor 1(MLF1)	Homo sapiens
2165	11732218_ turquoise	BH3 interacting domain death agonist(BID)	Homo sapiens
2166	11732220_ turquoise	enoyl-CoA delta isomerase 1(ECI1)	Homo sapiens
2167	11732232_ turquoise	ORAI calcium release-activated calcium modulator 2(ORAI2)	Homo sapiens
2168	11732243_ turquoise	apolipoprotein L6(APOL6)	Homo sapiens
2169	11732244_ turquoise	apolipoprotein L6(APOL6)	Homo sapiens
2170	11732247_ turquoise	heterogeneous nuclear ribonucleoprotein C (C1/C2)(HNRNPC)	Homo sapiens
2171	11732265_ turquoise	leukocyte immunoglobulin like receptor B1(LILRB1)	Homo sapiens
2172	11732266_ turquoise	leukocyte immunoglobulin like receptor B1(LILRB1)	Homo sapiens
2173	11732273_ turquoise	cytochrome c oxidase assembly factor 7 (putative)(COA7)	Homo sapiens
2174	11732275_ turquoise	C-C motif chemokine ligand 5(CCL5)	Homo sapiens
2175	11732276_ turquoise	C-C motif chemokine ligand 5(CCL5)	Homo sapiens
2176	11732278_ turquoise	forkhead box P1(FOXP1)	Homo sapiens
2177	11732315_ turquoise	sarcoglycan delta(SGCD)	Homo sapiens
2178	11732322_ brown	frataxin(FXN)	Homo sapiens
2179	11732323_ turquoise	frataxin(FXN)	Homo sapiens
2180	11732331_ turquoise	ADP ribosylation factor like GTPase 4C(ARL4C)	Homo sapiens
2181	11732349_ turquoise	leukocyte immunoglobulin like receptor A6(LILRA6)	Homo sapiens
2182	11732355_ turquoise	major histocompatibility complex, class I, F(HLA-F)	Homo sapiens
2183	11732366_ turquoise	S-phase cyclin A associated protein in the ER(SCAPER)	Homo sapiens
2184	11732369_ turquoise	fibroblast growth factor 14(FGF14)	Homo sapiens
2185	11732402_ turquoise	UBA domain containing 1(UBAC1)	Homo sapiens
2186	11732414_ turquoise	leucine rich repeat containing 8 family member B(LRRC8B)	Homo sapiens
2187	11732418_ turquoise	family with sequence similarity 105 member A(FAM105A)	Homo sapiens
2188	11732424_ turquoise	ankyrin repeat domain 22(ANKRD22)	Homo sapiens
2189	11732425_ turquoise	ankyrin repeat domain 22(ANKRD22)	Homo sapiens
2190	11732435_ turquoise	SLP adaptor and CSK interacting membrane protein(SCIMP)	Homo sapiens
2191	11732450_ turquoise	agrin(AGRN)	Homo sapiens
2192	11732456_ turquoise	membrane associated ring-CH-type finger 1(MARCH1)	Homo sapiens
2193	11732466_ turquoise	C-X-C motif chemokine ligand 11(CXCL11)	Homo sapiens
2194	11732467_ turquoise	C-X-C motif chemokine ligand 11(CXCL11)	Homo sapiens
2195	11732469_ turquoise	interleukin 3 receptor subunit alpha(IL3RA)	Homo sapiens
2196	11732479_ turquoise	integrin subunit alpha M(ITGAM)	Homo sapiens
2197	11732480_ turquoise	integrin subunit alpha M(ITGAM)	Homo sapiens
2198	11732481_ turquoise	integrin subunit alpha M(ITGAM)	Homo sapiens

2199	11732492_	turquoise	KIAA1549(KIAA1549)	Homo sapiens
2200	11732501_	blue	tropomodulin 1(TMOD1)	Homo sapiens
2201	11732514_	turquoise	LYN proto-oncogene, Src family tyrosine kinase(LYN)	Homo sapiens
2202	11732519_	turquoise	cholesterol 25-hydroxylase(CH25H)	Homo sapiens
2203	11732520_	brown	RAD51 paralog C(RAD51C)	Homo sapiens
2204	11732524_	blue	carnitine palmitoyltransferase 1B(CPT1B)	Homo sapiens
2205	11732526_	turquoise	chondroitin sulfate N-acetylgalactosaminyltransferase 1(CSGALNACT1)	Homo sapiens
2206	11732528_	turquoise	T-box 5(TBX5)	Homo sapiens
2207	11732529_	turquoise	T-box 5(TBX5)	Homo sapiens
2208	11732531_	turquoise	NEDD4 binding protein 2 like 1(N4BP2L1)	Homo sapiens
2209	11732533_	turquoise	lamin B1(LMNB1)	Homo sapiens
2210	11732538_	turquoise	T-box 21(TBX21)	Homo sapiens
2211	11732544_	turquoise	G protein-coupled receptor 18(GPR18)	Homo sapiens
2212	11732550_	turquoise	prostaglandin E receptor 2(PTGER2)	Homo sapiens
2213	11732555_	turquoise	Fas ligand(FASLG)	Homo sapiens
2214	11732580_	turquoise	malic enzyme 3(ME3)	Homo sapiens
2215	11732582_	brown	nudix hydrolase 22(NUDT22)	Homo sapiens
2216	11732583_	brown	nudix hydrolase 22(NUDT22)	Homo sapiens
2217	11732589_	turquoise	zinc finger protein 467(ZNF467)	Homo sapiens
2218	11732601_	turquoise	centrosomal protein 135(CEP135)	Homo sapiens
2219	11732602_	turquoise	centrosomal protein 135(CEP135)	Homo sapiens
2220	11732650_	turquoise	hexosaminidase D(HEXDC)	Homo sapiens
2221	11732661_	turquoise	SCL/TAL1 interrupting locus(STIL)	Homo sapiens
2222	11732701_	turquoise	chondroitin sulfate N-acetylgalactosaminyltransferase 2(CSGALNACT2)	Homo sapiens
2223	11732709_	turquoise	sarcoglycan alpha(SGCA)	Homo sapiens
2224	11732759_	turquoise	GRB2 binding adaptor protein, transmembrane(GAPT)	Homo sapiens
2225	11732798_	turquoise	mirror-image polydactyly 1(MIPOL1)	Homo sapiens
2226	11732813_	turquoise	dendritic cell associated nuclear protein(DCANP1)	Homo sapiens
2227	11732827_	turquoise	renalase, FAD dependent amine oxidase(RNLS)	Homo sapiens
2228	11732854_	turquoise	scavenger receptor class F member 1(SCARF1)	Homo sapiens
2229	11732857_	blue	THAP domain containing 1(THAP1)	Homo sapiens
2230	11732870_	turquoise	caspase 1(CASP1)	Homo sapiens
2231	11732873_	turquoise	mitochondrial translation release factor 1(MTRF1)	Homo sapiens
2232	11732894_	turquoise	cyclin dependent kinase like 3(CDKL3)	Homo sapiens
2233	11732896_	turquoise	hydroxysteroid 11-beta dehydrogenase 1 like(HSD11B1L)	Homo sapiens
2234	11732901_	turquoise	APOBEC3A and APOBEC3B deletion hybrid(APOBEC3A_B)	Homo sapiens
2235	11732902_	turquoise	apolipoprotein B mRNA editing enzyme catalytic subunit 3B(APOBEC3B)	Homo sapiens
2236	11732913_	turquoise	SP140 nuclear body protein(SP140)	Homo sapiens
2237	11732927_	turquoise	killer cell lectin like receptor C1(KLRC1)	Homo sapiens
2238	11732936_	turquoise	TBC1 domain family member 2B(TBC1D2B)	Homo sapiens
2239	11732956_	turquoise	TCR gamma alternate reading frame protein(TARP)	Homo sapiens
2240	11732999_	turquoise	intercellular adhesion molecule 1(ICAM1)	Homo sapiens
2241	11733000_	turquoise	intercellular adhesion molecule 1(ICAM1)	Homo sapiens
2242	11733004_	turquoise	Fc fragment of IgG receptor IIIa(FCGR3A)	Homo sapiens
2243	11733006_	turquoise	angiomotin like 2(AMOTL2)	Homo sapiens
2244	11733014_	turquoise	ring finger protein 213(RNF213)	Homo sapiens
2245	11733021_	turquoise	lactate dehydrogenase A(LDHA)	Homo sapiens
2246	11733023_	turquoise	BTG anti-proliferation factor 1(BTG1)	Homo sapiens
2247	11733024_	turquoise	BTG anti-proliferation factor 1(BTG1)	Homo sapiens
2248	11733030_	turquoise	axin interactor, dorsalization associated(AIDA)	Homo sapiens

2249	11733032_ blue	RNA binding motif protein 18(RBM18)	Homo sapiens
2250	11733033_ turquoise	family with sequence similarity 32 member A(FAM32A)	Homo sapiens
2251	11733046_ turquoise	abl interactor 1(ABI1)	Homo sapiens
2252	11733047_ turquoise	abl interactor 1(ABI1)	Homo sapiens
2253	11733052_ turquoise	secretory carrier membrane protein 1(SCAMP1)	Homo sapiens
2254	11733056_ turquoise	polypyrimidine tract binding protein 3(PTBP3)	Homo sapiens
2255	11733057_ turquoise	polypyrimidine tract binding protein 3(PTBP3)	Homo sapiens
2256	11733058_ turquoise	polypyrimidine tract binding protein 3(PTBP3)	Homo sapiens
2257	11733060_ turquoise	pellino E3 ubiquitin protein ligase 1(PELI1)	Homo sapiens
2258	11733061_ turquoise	pellino E3 ubiquitin protein ligase 1(PELI1)	Homo sapiens
2259	11733087_ turquoise	MAF bZIP transcription factor(MAF)	Homo sapiens
2260	11733088_ turquoise	MAF bZIP transcription factor(MAF)	Homo sapiens
2261	11733127_ turquoise	solute carrier family 25 member 26(SLC25A26)	Homo sapiens
2262	11733134_ brown	mitochondrial ribosomal protein S33(MRPS33)	Homo sapiens
2263	11733140_ turquoise	ADP ribosylation factor like GTPase 4A(ARL4A)	Homo sapiens
2264	11733148_ turquoise	annexin A4(ANXA4)	Homo sapiens
2265	11733149_ turquoise	DExD/H-box helicase 58(DDX58)	Homo sapiens
2266	11733161_ turquoise	TNF alpha induced protein 8(TNFAIP8)	Homo sapiens
2267	11733162_ turquoise	proline-serine-threonine phosphatase interacting protein 1(PSTPIP1)	Homo sapiens
2268	11733187_ turquoise	interleukin 7 receptor(IL7R)	Homo sapiens
2269	11733194_ turquoise	formin like 3(FMNL3)	Homo sapiens
2270	11733198_ turquoise	DExD-box helicase 39A(DDX39A)	Homo sapiens
2271	11733213_ turquoise	schlafen family member 5(SLFN5)	Homo sapiens
2272	11733214_ turquoise	schlafen family member 5(SLFN5)	Homo sapiens
2273	11733215_ turquoise	signal transducer and activator of transcription 4(STAT4)	Homo sapiens
2274	11733246_ turquoise	dystrobrevin binding protein 1(DTNBP1)	Homo sapiens
2275	11733255_ blue	carnitine palmitoyltransferase 1B(CPT1B)	Homo sapiens
2276	11733264_ turquoise	echinoderm microtubule associated protein like 1(EML1)	Homo sapiens
2277	11733334_ turquoise	neuropilin 2(NRP2)	Homo sapiens
2278	11733343_ turquoise	UTP6, small subunit processome component(UTP6)	Homo sapiens
2279	11733353_ turquoise	cytotoxic and regulatory T-cell molecule(CRTAM)	Homo sapiens
2280	11733355_ turquoise	complement C5a receptor 1(C5AR1)	Homo sapiens
2281	11733360_ turquoise	solute carrier family 14 member 1 (Kidd blood group)(SLC14A1)	Homo sapiens
2282	11733370_ turquoise	proteasome activator subunit 1(PSME1)	Homo sapiens
2283	11733389_ turquoise	DEF6, guanine nucleotide exchange factor(DEF6)	Homo sapiens
2284	11733402_ turquoise	C-type lectin domain family 7 member A(CLEC7A)	Homo sapiens
2285	11733403_ turquoise	C-type lectin domain family 7 member A(CLEC7A)	Homo sapiens
2286	11733411_ turquoise	transcription factor 15 (basic helix-loop-helix)(TCF15)	Homo sapiens
2287	11733439_ turquoise	guanylate binding protein 5(GBP5)	Homo sapiens
2288	11733450_ turquoise	schlafen family member 11(SLFN11)	Homo sapiens
2289	11733477_ turquoise	succinate receptor 1(SUCNR1)	Homo sapiens
2290	11733485_ turquoise	calcium voltage-gated channel subunit alpha1 C(CACNA1C)	Homo sapiens
2291	11733494_ turquoise	NEDD4 binding protein 2 like 2(N4BP2L2)	Homo sapiens
2292	11733511_ turquoise	myotubularin related protein 1(MTMR1)	Homo sapiens
2293	11733512_ turquoise	myotubularin related protein 1(MTMR1)	Homo sapiens
2294	11733523_ blue	protein phosphatase 1 regulatory subunit 3A(PPP1R3A)	Homo sapiens
2295	11733525_ turquoise	chromosome 2 open reading frame 71(C2orf71)	Homo sapiens
2296	11733530_ turquoise	complement C1r subcomponent like(C1RL)	Homo sapiens
2297	11733535_ brown	nudix hydrolase 7(NUDT7)	Homo sapiens
2298	11733576_ brown	isocitrate dehydrogenase 3 (NAD(+)) beta(IDH3B)	Homo sapiens

2299	11733579_ turquoise	chromosome 19 open reading frame 47(C19orf47)	Homo sapiens
2300	11733611_ turquoise	dedicator of cytokinesis 8(DOCK8)	Homo sapiens
2301	11733612_ turquoise	dedicator of cytokinesis 8(DOCK8)	Homo sapiens
2302	11733616_ turquoise	histamine N-methyltransferase(HNMT)	Homo sapiens
2303	11733620_ turquoise	B-cell CLL/lymphoma 11B(BCL11B)	Homo sapiens
2304	11733632_ turquoise	purinergic receptor P2Y14(P2RY14)	Homo sapiens
2305	11733641_ turquoise	kinesin light chain 4(KLC4)	Homo sapiens
2306	11733642_ turquoise	NECAP endocytosis associated 2(NECAP2)	Homo sapiens
2307	11733643_ turquoise	NECAP endocytosis associated 2(NECAP2)	Homo sapiens
2308	11733644_ turquoise	NECAP endocytosis associated 2(NECAP2)	Homo sapiens
2309	11733671_ blue	EYA transcriptional coactivator and phosphatase 1(EYA1)	Homo sapiens
2310	11733695_ turquoise	ubiquitin conjugating enzyme E2 C(UBE2C)	Homo sapiens
2311	11733696_ turquoise	ubiquitin conjugating enzyme E2 C(UBE2C)	Homo sapiens
2312	11733698_ turquoise	serum/glucocorticoid regulated kinase 1(SGK1)	Homo sapiens
2313	11733699_ turquoise	TEA domain transcription factor 4(TEAD4)	Homo sapiens
2314	11733701_ turquoise	FK506 binding protein 11(FKBP11)	Homo sapiens
2315	11733702_ turquoise	ubiquitin conjugating enzyme E2 C(UBE2C)	Homo sapiens
2316	11733718_ turquoise	leukocyte associated immunoglobulin like receptor 2(LAIR2)	Homo sapiens
2317	11733725_ turquoise	complement factor B(CFB)	Homo sapiens
2318	11733736_ turquoise	CD2 molecule(CD2)	Homo sapiens
2319	11733754_ turquoise	pleckstrin homology domain containing A4(PLEKHA4)	Homo sapiens
2320	11733767_ turquoise	leukocyte immunoglobulin like receptor A2(LILRA2)	Homo sapiens
2321	11733768_ turquoise	leukocyte immunoglobulin like receptor A2(LILRA2)	Homo sapiens
2322	11733784_ turquoise	protein phosphatase 2 scaffold subunit Abeta(PPP2R1B)	Homo sapiens
2323	11733792_ blue	solute carrier family 25 member 12(SLC25A12)	Homo sapiens
2324	11733809_ turquoise	ARMCX5-GPRASP2 readthrough(ARMCX5-GPRASP2)	Homo sapiens
2325	11733841_ turquoise	ecotropic viral integration site 2A(EVI2A)	Homo sapiens
2326	11733845_ turquoise	G protein-coupled receptor kinase 6(GRK6)	Homo sapiens
2327	11733846_ turquoise	G protein-coupled receptor kinase 6(GRK6)	Homo sapiens
2328	11733855_ turquoise	apolipoprotein L1(APOL1)	Homo sapiens
2329	11733860_ blue	cytoplasmic linker associated protein 2(CLASP2)	Homo sapiens
2330	11733867_ turquoise	major histocompatibility complex, class II, DO alpha(HLA-DOA)	Homo sapiens
2331	11733868_ turquoise	major histocompatibility complex, class II, DO alpha(HLA-DOA)	Homo sapiens
2332	11733869_ turquoise	major histocompatibility complex, class II, DO alpha(HLA-DOA)	Homo sapiens
2333	11733870_ turquoise	major histocompatibility complex, class II, DO alpha(HLA-DOA)	Homo sapiens
2334	11733878_ turquoise	LIM domain 7(LMO7)	Homo sapiens
2335	11733894_ turquoise	transcriptional adaptor 2B(TADA2B)	Homo sapiens
2336	11733901_ brown	ubiquinol-cytochrome c reductase complex III subunit VII(UQCRQ)	Homo sapiens
2337	11733934_ turquoise	primary cilia formation(PIFO)	Homo sapiens
2338	11733946_ turquoise	RAP2B, member of RAS oncogene family(RAP2B)	Homo sapiens
2339	11733950_ turquoise	guanylate binding protein 4(GBP4)	Homo sapiens
2340	11733951_ turquoise	guanylate binding protein 4(GBP4)	Homo sapiens
2341	11733952_ turquoise	guanylate binding protein 4(GBP4)	Homo sapiens
2342	11733953_ turquoise	guanylate binding protein 4(GBP4)	Homo sapiens
2343	11733960_ turquoise	USH1 protein network component sans(USH1G)	Homo sapiens
2344	11733979_ turquoise	CD28 molecule(CD28)	Homo sapiens
2345	11733986_ turquoise	tubulin alpha 3e(TUBA3E)	Homo sapiens
2346	11733992_ turquoise	transmembrane protein 150C(TMEM150C)	Homo sapiens
2347	11734006_ turquoise	interleukin 1 receptor associated kinase 1(IRAK1)	Homo sapiens
2348	11734033_ brown	chromosome 15 open reading frame 61(C15orf61)	Homo sapiens

2349	11734035_	turquoise	formyl peptide receptor 2(FPR2)	Homo sapiens
2350	11734050_	turquoise	T cell receptor associated transmembrane adaptor 1(TRAT1)	Homo sapiens
2351	11734051_	turquoise	T cell receptor associated transmembrane adaptor 1(TRAT1)	Homo sapiens
2352	11734056_	turquoise	prostaglandin reductase 2(PTGR2)	Homo sapiens
2353	11734064_	turquoise	uncharacterized LOC100130460(CAND1.11)	Homo sapiens
2354	11734065_	turquoise	uncharacterized LOC100130460(CAND1.11)	Homo sapiens
2355	11734066_	turquoise	interleukin 1 receptor associated kinase 4(IRAK4)	Homo sapiens
2356	11734067_	turquoise	interleukin 1 receptor associated kinase 4(IRAK4)	Homo sapiens
2357	11734084_	turquoise	sushi domain containing 3(SUSD3)	Homo sapiens
2358	11734110_	turquoise	T-cell immunoreceptor with Ig and ITIM domains(TIGIT)	Homo sapiens
2359	11734112_	turquoise	T-cell immunoreceptor with Ig and ITIM domains(TIGIT)	Homo sapiens
2360	11734117_	turquoise	butyrophilin subfamily 3 member A3(BTN3A3)	Homo sapiens
2361	11734118_	turquoise	butyrophilin subfamily 3 member A3(BTN3A3)	Homo sapiens
2362	11734126_	turquoise	nucleotide binding oligomerization domain containing 1(NOD1)	Homo sapiens
2363	11734139_	turquoise	TBC1 domain family member 10C(TBC1D10C)	Homo sapiens
2364	11734162_	turquoise	transcription factor EC(TFEC)	Homo sapiens
2365	11734185_	turquoise	zinc finger protein 677(ZNF677)	Homo sapiens
2366	11734259_	turquoise	sterol carrier protein 2(SCP2)	Homo sapiens
2367	11734273_	turquoise	G protein-coupled receptor 18(GPR18)	Homo sapiens
2368	11734359_	turquoise	ribonuclease L(RNASEL)	Homo sapiens
2369	11734394_	turquoise	killer cell lectin like receptor C3(KLRC3)	Homo sapiens
2370	11734395_	turquoise	killer cell lectin like receptor C3(KLRC3)	Homo sapiens
2371	11734491_	brown	spermatogenesis associated 24(SPATA24)	Homo sapiens
2372	11734521_	turquoise	sodium channel and clathrin linker 1(SCLT1)	Homo sapiens
2373	11734526_	turquoise	uncharacterized LOC100507472(LOC100507472)	Homo sapiens
2374	11734529_	turquoise	paired immunoglobin like type 2 receptor alpha(PILRA)	Homo sapiens
2375	11734530_	turquoise	major histocompatibility complex, class I, F(HLA-F)	Homo sapiens
2376	11734536_	turquoise	glycoprotein Ib platelet alpha subunit(GP1BA)	Homo sapiens
2377	11734548_	turquoise	transforming growth factor beta induced(TGFBI)	Homo sapiens
2378	11734549_	turquoise	transforming growth factor beta induced(TGFBI)	Homo sapiens
2379	11734550_	turquoise	transforming growth factor beta induced(TGFBI)	Homo sapiens
2380	11734552_	turquoise	C-X3-C motif chemokine ligand 1(CX3CL1)	Homo sapiens
2381	11734567_	turquoise	RAS guanyl releasing protein 2(RASGRP2)	Homo sapiens
2382	11734577_	turquoise	sodium voltage-gated channel alpha subunit 5(SCN5A)	Homo sapiens
2383	11734606_	turquoise	C-X-C motif chemokine receptor 3(CXCR3)	Homo sapiens
2384	11734652_	blue	ATP synthase, H+ transporting, mitochondrial Fo complex subunit B1(ATP5F1)	Homo sapiens
2385	11734653_	blue	ATP synthase, H+ transporting, mitochondrial Fo complex subunit B1(ATP5F1)	Homo sapiens
2386	11734657_	turquoise	solute carrier family 2 member 14(SLC2A14)	Homo sapiens
2387	11734659_	turquoise	Fos proto-oncogene, AP-1 transcription factor subunit(FOS)	Homo sapiens
2388	11734661_	turquoise	calsyntenin 3(CLSTN3)	Homo sapiens
2389	11734672_	turquoise	nuclear factor of activated T-cells 4(NFATC4)	Homo sapiens
2390	11734684_	turquoise	uridine monophosphate synthetase(UMPS)	Homo sapiens
2391	11734687_	turquoise	methyl-CpG binding domain protein 2(MBD2)	Homo sapiens
2392	11734690_	turquoise	cytohesin 1 interacting protein(CYTIP)	Homo sapiens
2393	11734695_	turquoise	protein phosphatase 4 catalytic subunit(PPP4C)	Homo sapiens
2394	11734715_	turquoise	DBF4 zinc finger(DBF4)	Homo sapiens
2395	11734727_	turquoise	interleukin 16(IL16)	Homo sapiens
2396	11734731_	brown	mitochondrial ribosomal protein S12(MRPS12)	Homo sapiens
2397	11734746_	brown	CDKN2A interacting protein N-terminal like(CDKN2AIPNL)	Homo sapiens
2398	11734747_	turquoise	complement component 4B (Chido blood group), copy 2(C4B_2)	Homo sapiens

2399	11734772_	turquoise	synergin gamma(SYNRG)	Homo sapiens
2400	11734783_	turquoise	chloride intracellular channel 2(CLIC2)	Homo sapiens
2401	11734784_	turquoise	chloride intracellular channel 2(CLIC2)	Homo sapiens
2402	11734785_	turquoise	chloride intracellular channel 2(CLIC2)	Homo sapiens
2403	11734791_	turquoise	septin 6(SEPT6)	Homo sapiens
2404	11734798_	turquoise	ring finger protein, LIM domain interacting(RLIM)	Homo sapiens
2405	11734825_	turquoise	sprouty related EVH1 domain containing 1(SPRED1)	Homo sapiens
2406	11734826_	turquoise	sprouty related EVH1 domain containing 1(SPRED1)	Homo sapiens
2407	11734852_	turquoise	RUN and cysteine rich domain containing beclin 1 interacting protein like(RUBCNL)	Homo sapiens
2408	11734862_	blue	trimethyllysine hydroxylase, epsilon(TMLHE)	Homo sapiens
2409	11734870_	turquoise	NAD kinase(NADK)	Homo sapiens
2410	11734873_	turquoise	S-phase cyclin A associated protein in the ER(SCAPER)	Homo sapiens
2411	11734890_	turquoise	interleukin 32(IL32)	Homo sapiens
2412	11734894_	turquoise	CST telomere replication complex component 1(CTC1)	Homo sapiens
2413	11734921_	turquoise	zinc finger protein 276(ZNF276)	Homo sapiens
2414	11734922_	turquoise	zinc finger protein 276(ZNF276)	Homo sapiens
2415	11734924_	turquoise	ATPase H+ transporting V1 subunit G2(ATP6V1G2)	Homo sapiens
2416	11734938_	turquoise	colony stimulating factor 2 receptor alpha subunit(CSF2RA)	Homo sapiens
2417	11734943_	turquoise	SLAM family member 6(SLAMF6)	Homo sapiens
2418	11734944_	turquoise	SLAM family member 6(SLAMF6)	Homo sapiens
2419	11734948_	turquoise	fibroblast growth factor 7(FGF7)	Homo sapiens
2420	11734962_	turquoise	ADAM metallopeptidase domain 33(ADAM33)	Homo sapiens
2421	11734967_	turquoise	thymocyte selection associated(THEMIS)	Homo sapiens
2422	11735003_	turquoise	suppression of tumorigenicity 7 like(ST7L)	Homo sapiens
2423	11735026_	turquoise	sialophorin(SPN)	Homo sapiens
2424	11735027_	turquoise	sialophorin(SPN)	Homo sapiens
2425	11735039_	turquoise	shootin 1(SHTN1)	Homo sapiens
2426	11735040_	blue	ribosomal protein L3 like(RPL3L)	Homo sapiens
2427	11735044_	turquoise	tripartite motif containing 5(TRIM5)	Homo sapiens
2428	11735096_	brown	MLX, MAX dimerization protein(MLX)	Homo sapiens
2429	11735097_	blue	MLX, MAX dimerization protein(MLX)	Homo sapiens
2430	11735124_	turquoise	interaction protein for cytohesin exchange factors 1(IPCEF1)	Homo sapiens
2431	11735157_	turquoise	cysteinyl leukotriene receptor 1(CYSLTR1)	Homo sapiens
2432	11735173_	turquoise	small nuclear ribonucleoprotein polypeptide N(SNRPN)	Homo sapiens
2433	11735174_	turquoise	interleukin 32(IL32)	Homo sapiens
2434	11735189_	turquoise	H2A histone family member Y(H2AFY)	Homo sapiens
2435	11735192_	turquoise	adhesion G protein-coupled receptor E2(ADGRE2)	Homo sapiens
2436	11735204_	turquoise	methylmalonic aciduria (cobalamin deficiency) cblA type(MMAA)	Homo sapiens
2437	11735205_	turquoise	methylmalonic aciduria (cobalamin deficiency) cblA type(MMAA)	Homo sapiens
2438	11735220_	turquoise	lysophosphatidic acid receptor 3(LPAR3)	Homo sapiens
2439	11735221_	turquoise	B and T lymphocyte associated(BTLA)	Homo sapiens
2440	11735222_	turquoise	B and T lymphocyte associated(BTLA)	Homo sapiens
2441	11735223_	turquoise	killer cell lectin like receptor G1(KLRG1)	Homo sapiens
2442	11735224_	turquoise	killer cell lectin like receptor G1(KLRG1)	Homo sapiens
2443	11735225_	turquoise	guanidinoacetate N-methyltransferase(GAMT)	Homo sapiens
2444	11735249_	turquoise	SP140 nuclear body protein like(SP140L)	Homo sapiens
2445	11735270_	turquoise	glucosaminyl (N-acetyl) transferase 1, core 2(GCNT1)	Homo sapiens
2446	11735271_	turquoise	glucosaminyl (N-acetyl) transferase 1, core 2(GCNT1)	Homo sapiens
2447	11735275_	turquoise	interleukin 18 receptor 1(IL18R1)	Homo sapiens
2448	11735284_	turquoise	lysine methyltransferase 2E(KMT2E)	Homo sapiens

2449	11735290_	turquoise	TNFAIP3 interacting protein 2(TNIP2)	Homo sapiens
2450	11735329_	turquoise	CD47 molecule(CD47)	Homo sapiens
2451	11735362_	blue	AKT interacting protein(AKTIP)	Homo sapiens
2452	11735394_	turquoise	X-C motif chemokine ligand 1(XCL1)	Homo sapiens
2453	11735416_	turquoise	DNA cross-link repair 1C(DCLRE1C)	Homo sapiens
2454	11735429_	turquoise	ring finger protein 135(RNF135)	Homo sapiens
2455	11735474_	turquoise	CD8b molecule(CD8B)	Homo sapiens
2456	11735502_	turquoise	TAP binding protein(TAPBP)	Homo sapiens
2457	11735504_	turquoise	NLR family CARD domain containing 4(NLRC4)	Homo sapiens
2458	11735551_	turquoise	Fc receptor like 6(FCRL6)	Homo sapiens
2459	11735552_	turquoise	Fc receptor like 6(FCRL6)	Homo sapiens
2460	11735623_	turquoise	proteasome subunit beta 9(PSMB9)	Homo sapiens
2461	11735710_	turquoise	SAM domain, SH3 domain and nuclear localization signals 1(SAMSN1)	Homo sapiens
2462	11735711_	turquoise	casein kinase 1 gamma 2(CSNK1G2)	Homo sapiens
2463	11735712_	turquoise	casein kinase 1 gamma 2(CSNK1G2)	Homo sapiens
2464	11735713_	turquoise	casein kinase 1 gamma 2(CSNK1G2)	Homo sapiens
2465	11735732_	turquoise	ribonuclease P/MRP subunit p14(RPP14)	Homo sapiens
2466	11735740_	turquoise	RAS p21 protein activator 3(RASA3)	Homo sapiens
2467	11735741_	turquoise	RAS p21 protein activator 3(RASA3)	Homo sapiens
2468	11735767_	turquoise	chromosome 16 open reading frame 54(C16orf54)	Homo sapiens
2469	11735768_	turquoise	chromosome 16 open reading frame 54(C16orf54)	Homo sapiens
2470	11735790_	turquoise	protein tyrosine phosphatase, non-receptor type 18(PTPN18)	Homo sapiens
2471	11735864_	turquoise	CD160 molecule(CD160)	Homo sapiens
2472	11735937_	turquoise	CD48 molecule(CD48)	Homo sapiens
2473	11735956_	turquoise	methyltransferase like 11B(METTL11B)	Homo sapiens
2474	11735989_	yellow	joining chain of multimeric IgA and IgM(JCHAIN)	Homo sapiens
2475	11735990_	yellow	joining chain of multimeric IgA and IgM(JCHAIN)	Homo sapiens
2476	11735997_	turquoise	apolipoprotein L1(APOL1)	Homo sapiens
2477	11735998_	turquoise	apolipoprotein L1(APOL1)	Homo sapiens
2478	11736030_	turquoise	microRNA 6787(MIR6787)	Homo sapiens
2479	11736049_	brown	family with sequence similarity 229 member B(FAM229B)	Homo sapiens
2480	11736050_	brown	family with sequence similarity 229 member B(FAM229B)	Homo sapiens
2481	11736064_	turquoise	purinergic receptor P2X 7(P2RX7)	Homo sapiens
2482	11736065_	turquoise	purinergic receptor P2X 7(P2RX7)	Homo sapiens
2483	11736094_	turquoise	dCMP deaminase(DCTD)	Homo sapiens
2484	11736097_	turquoise	golgi associated, gamma adaptin ear containing, ARF binding protein 1(GGA1)	Homo sapiens
2485	11736111_	turquoise	Rho GTPase activating protein 18(ARHGAP18)	Homo sapiens
2486	11736112_	turquoise	Rho GTPase activating protein 18(ARHGAP18)	Homo sapiens
2487	11736113_	turquoise	Rho GTPase activating protein 18(ARHGAP18)	Homo sapiens
2488	11736132_	turquoise	discoidin, CUB and LCCL domain containing 2(DCBLD2)	Homo sapiens
2489	11736133_	turquoise	PHD finger protein 19(PHF19)	Homo sapiens
2490	11736135_	turquoise	2'-5'-oligoadenylate synthetase 2(OAS2)	Homo sapiens
2491	11736136_	turquoise	2'-5'-oligoadenylate synthetase 2(OAS2)	Homo sapiens
2492	11736207_	turquoise	plexin C1(PLXNC1)	Homo sapiens
2493	11736217_	turquoise	mitogen-activated protein kinase kinase kinase 8(MAP3K8)	Homo sapiens
2494	11736224_	turquoise	spermatogenesis associated serine rich 2(SPATS2)	Homo sapiens
2495	11736225_	turquoise	spermatogenesis associated serine rich 2(SPATS2)	Homo sapiens
2496	11736233_	turquoise	phosphotriesterase related(PTER)	Homo sapiens
2497	11736241_	turquoise	ORMDL sphingolipid biosynthesis regulator 2(ORMDL2)	Homo sapiens
2498	11736247_	turquoise	adhesion molecule with Ig like domain 2(AMIGO2)	Homo sapiens

2499	11736255_ turquoise	inositol polyphosphate-4-phosphatase type I A(INPP4A)	Homo sapiens
2500	11736273_ turquoise	toll like receptor 2(TLR2)	Homo sapiens
2501	11736296_ blue	solute carrier family 5 member 1(SLC5A1)	Homo sapiens
2502	11736297_ turquoise	solute carrier family 5 member 1(SLC5A1)	Homo sapiens
2503	11736311_ turquoise	Fc fragment of IgG receptor Ic, pseudogene(FCGR1CP)	Homo sapiens
2504	11736324_ turquoise	CD96 molecule(CD96)	Homo sapiens
2505	11736330_ turquoise	solute carrier family 2 member 11(SLC2A11)	Homo sapiens
2506	11736342_ turquoise	early growth response 3(EGR3)	Homo sapiens
2507	11736375_ turquoise	CD86 molecule(CD86)	Homo sapiens
2508	11736376_ turquoise	membrane palmitoylated protein 3(MPP3)	Homo sapiens
2509	11736378_ turquoise	male-specific lethal 2 homolog (Drosophila)(MSL2)	Homo sapiens
2510	11736394_ turquoise	interleukin 32(IL32)	Homo sapiens
2511	11736405_ turquoise	DNA methyltransferase 1(DNMT1)	Homo sapiens
2512	11736409_ turquoise	nucleoporin 62(NUP62)	Homo sapiens
2513	11736414_ turquoise	melanotransferrin(MELTF)	Homo sapiens
2514	11736419_ turquoise	zinc finger protein 800(ZNF800)	Homo sapiens
2515	11736430_ turquoise	nucleolar protein 8(NOL8)	Homo sapiens
2516	11736448_ blue	gypsy retrotransposon integrase 1(GIN1)	Homo sapiens
2517	11736457_ turquoise	potassium two pore domain channel subfamily K member 6(KCNK6)	Homo sapiens
2518	11736458_ turquoise	potassium two pore domain channel subfamily K member 6(KCNK6)	Homo sapiens
2519	11736467_ turquoise	T-cell activation RhoGTPase activating protein(TAGAP)	Homo sapiens
2520	11736499_ turquoise	transmembrane protein 38A(TMEM38A)	Homo sapiens
2521	11736519_ turquoise	chromosome X open reading frame 38(CXorf38)	Homo sapiens
2522	11736526_ turquoise	caspase recruitment domain family member 9(CARD9)	Homo sapiens
2523	11736539_ turquoise	RAB29, member RAS oncogene family(RAB29)	Homo sapiens
2524	11736550_ turquoise	RAB29, member RAS oncogene family(RAB29)	Homo sapiens
2525	11736551_ turquoise	REL proto-oncogene, NF-kB subunit(REL)	Homo sapiens
2526	11736555_ turquoise	solute carrier family 2 member 14(SLC2A14)	Homo sapiens
2527	11736556_ turquoise	solute carrier family 2 member 14(SLC2A14)	Homo sapiens
2528	11736567_ turquoise	lymphoid enhancer binding factor 1(LEF1)	Homo sapiens
2529	11736568_ turquoise	lymphoid enhancer binding factor 1(LEF1)	Homo sapiens
2530	11736573_ turquoise	protein kinase C delta(PRKCD)	Homo sapiens
2531	11736578_ turquoise	adaptor related protein complex 1 gamma 2 subunit(AP1G2)	Homo sapiens
2532	11736581_ turquoise	glucosaminyl (N-acetyl) transferase 1, core 2(GCNT1)	Homo sapiens
2533	11736589_ blue	de-etiolated homolog 1 (Arabidopsis)(DET1)	Homo sapiens
2534	11736590_ turquoise	receptor activity modifying protein 3(RAMP3)	Homo sapiens
2535	11736594_ turquoise	zinc finger and BTB domain containing 24(ZBTB24)	Homo sapiens
2536	11736619_ turquoise	aldehyde dehydrogenase 5 family member A1(ALDH5A1)	Homo sapiens
2537	11736637_ turquoise	DENN domain containing 4A(DENND4A)	Homo sapiens
2538	11736641_ turquoise	mitochondrial trans-2-enoyl-CoA reductase(MECR)	Homo sapiens
2539	11736683_ turquoise	cytoplasmic FMR1 interacting protein 1(CYFIP1)	Homo sapiens
2540	11736693_ turquoise	tripartite motif containing 38(TRIM38)	Homo sapiens
2541	11736694_ turquoise	tripartite motif containing 38(TRIM38)	Homo sapiens
2542	11736705_ turquoise	HPS5, biogenesis of lysosomal organelles complex 2 subunit 2(HPS5)	Homo sapiens
2543	11736710_ turquoise	DENN domain containing 4A(DENND4A)	Homo sapiens
2544	11736742_ turquoise	chromosome 1 open reading frame 168(C1orf168)	Homo sapiens
2545	11736746_ turquoise	ADAM metallopeptidase domain 28(ADAM28)	Homo sapiens
2546	11736747_ turquoise	ADAM metallopeptidase domain 28(ADAM28)	Homo sapiens
2547	11736760_ turquoise	FK506 binding protein 11(FKBP11)	Homo sapiens
2548	11736761_ turquoise	caspase recruitment domain family member 16(CARD16)	Homo sapiens

2549	11736770_ blue	ADAM metallopeptidase domain 23(ADAM23)	Homo sapiens
2550	11736813_ turquoise	programmed cell death 1 ligand 2(PDCD1LG2)	Homo sapiens
2551	11736831_ turquoise	Sec23 homolog B, coat complex II component(SEC23B)	Homo sapiens
2552	11736877_ blue	DTW domain containing 2(DTWD2)	Homo sapiens
2553	11736888_ turquoise	integrin subunit alpha L(ITGAL)	Homo sapiens
2554	11736965_ blue	F-box protein 40(FBXO40)	Homo sapiens
2555	11736973_ turquoise	T-box 5(TBX5)	Homo sapiens
2556	11737026_ turquoise	iroquois homeobox 4(IRX4)	Homo sapiens
2557	11737027_ turquoise	fms related tyrosine kinase 3(FLT3)	Homo sapiens
2558	11737038_ turquoise	chromosome 19 open reading frame 38(C19orf38)	Homo sapiens
2559	11737050_ turquoise	histamine receptor H1(HRH1)	Homo sapiens
2560	11737058_ turquoise	pantothenate kinase 2(PANK2)	Homo sapiens
2561	11737131_ turquoise	fat storage inducing transmembrane protein 2(FITM2)	Homo sapiens
2562	11737146_ turquoise	suppressor of cytokine signaling 1(SOCS1)	Homo sapiens
2563	11737147_ turquoise	C-type lectin domain family 7 member A(CLEC7A)	Homo sapiens
2564	11737148_ turquoise	C-type lectin domain family 7 member A(CLEC7A)	Homo sapiens
2565	11737160_ turquoise	perilipin 5(PLIN5)	Homo sapiens
2566	11737171_ turquoise	CD80 molecule(CD80)	Homo sapiens
2567	11737237_ turquoise	eomesodermin(EOMES)	Homo sapiens
2568	11737238_ turquoise	eomesodermin(EOMES)	Homo sapiens
2569	11737239_ turquoise	eomesodermin(EOMES)	Homo sapiens
2570	11737243_ blue	fibronectin type III and SPRY domain containing 2(FSD2)	Homo sapiens
2571	11737250_ turquoise	interleukin 1 receptor accessory protein like 1(IL1RAPL1)	Homo sapiens
2572	11737256_ turquoise	SP100 nuclear antigen(SP100)	Homo sapiens
2573	11737309_ brown	chromosome 14 open reading frame 2(C14orf2)	Homo sapiens
2574	11737322_ turquoise	phospholipase D1(PLD1)	Homo sapiens
2575	11737423_ turquoise	NLR family pyrin domain containing 1(NLRP1)	Homo sapiens
2576	11737428_ turquoise	macrophage scavenger receptor 1(MSR1)	Homo sapiens
2577	11737429_ turquoise	macrophage scavenger receptor 1(MSR1)	Homo sapiens
2578	11737464_ turquoise	interleukin 11 receptor subunit alpha(IL11RA)	Homo sapiens
2579	11737484_ turquoise	chromosome 9 open reading frame 129(C9orf129)	Homo sapiens
2580	11737496_ turquoise	CD200 receptor 1(CD200R1)	Homo sapiens
2581	11737504_ turquoise	signal regulatory protein beta 2(SIRPB2)	Homo sapiens
2582	11737511_ turquoise	SP100 nuclear antigen(SP100)	Homo sapiens
2583	11737555_ turquoise	nudix hydrolase 17(NUDT17)	Homo sapiens
2584	11737750_ turquoise	serum/glucocorticoid regulated kinase 1(SGK1)	Homo sapiens
2585	11737757_ turquoise	cysteinyl-tRNA synthetase(CARS)	Homo sapiens
2586	11737758_ turquoise	cysteinyl-tRNA synthetase(CARS)	Homo sapiens
2587	11737770_ turquoise	apolipoprotein L4(APOL4)	Homo sapiens
2588	11737791_ turquoise	apolipoprotein L2(APOL2)	Homo sapiens
2589	11737811_ blue	nicotinamide nucleotide transhydrogenase(NNT)	Homo sapiens
2590	11737847_ turquoise	major histocompatibility complex, class I-related(MR1)	Homo sapiens
2591	11737851_ turquoise	protein phosphatase 1 regulatory subunit 18(PPP1R18)	Homo sapiens
2592	11737852_ turquoise	protein phosphatase 1 regulatory subunit 18(PPP1R18)	Homo sapiens
2593	11737890_ turquoise	linker for activation of T-cells family member 2(LAT2)	Homo sapiens
2594	11737907_ turquoise	protein phosphatase 1 catalytic subunit alpha(PPP1CA)	Homo sapiens
2595	11737911_ turquoise	G protein-coupled receptor 84(GPR84)	Homo sapiens
2596	11737940_ turquoise	receptor interacting serine/threonine kinase 1(RIPK1)	Homo sapiens
2597	11737952_ turquoise	ral guanine nucleotide dissociation stimulator(RALGDS)	Homo sapiens
2598	11737975_ turquoise	p21 (RAC1) activated kinase 6(PAK6)	Homo sapiens

2599	11737980_ turquoise	DAN domain BMP antagonist family member 5(DAND5)	Homo sapiens
2600	11738018_ brown	ARV1 homolog, fatty acid homeostasis modulator(ARV1)	Homo sapiens
2601	11738021_ turquoise	phospholipase C beta 4(PLCB4)	Homo sapiens
2602	11738053_ turquoise	plasminogen activator, urokinase receptor(PLAUR)	Homo sapiens
2603	11738054_ blue	integrin subunit beta 1 binding protein 2(ITGB1BP2)	Homo sapiens
2604	11738124_ turquoise	sialic acid binding Ig like lectin 10(SIGLEC10)	Homo sapiens
2605	11738139_ turquoise	kelch like family member 33(KLHL33)	Homo sapiens
2606	11738168_ turquoise	basic leucine zipper ATF-like transcription factor 3(BATF3)	Homo sapiens
2607	11738183_ turquoise	NLR family pyrin domain containing 3(NLRP3)	Homo sapiens
2608	11738193_ turquoise	paired like homeodomain 3(PITX3)	Homo sapiens
2609	11738202_ turquoise	death domain associated protein(DAXX)	Homo sapiens
2610	11738203_ turquoise	inosine monophosphate dehydrogenase 1(IMPDH1)	Homo sapiens
2611	11738268_ turquoise	family with sequence similarity 122C(FAM122C)	Homo sapiens
2612	11738270_ brown	stress associated endoplasmic reticulum protein family member 2(SERP2)	Homo sapiens
2613	11738335_ turquoise	tumor protein p53(TP53)	Homo sapiens
2614	11738435_ turquoise	CD74 molecule(CD74)	Homo sapiens
2615	11738460_ turquoise	NUT family member 2G(NUTM2G)	Homo sapiens
2616	11738516_ turquoise	zinc finger CCHC-type containing 6(ZCCHC6)	Homo sapiens
2617	11738523_ turquoise	NLR family pyrin domain containing 3(NLRP3)	Homo sapiens
2618	11738540_ turquoise	HHIP like 1(HHIPL1)	Homo sapiens
2619	11738656_ turquoise	PARK2 coregulated(PACRG)	Homo sapiens
2620	11738799_ turquoise	ninein(NIN)	Homo sapiens
2621	11738807_ turquoise	c-Maf inducing protein(CMIP)	Homo sapiens
2622	11738858_ turquoise	colony stimulating factor 2 receptor alpha subunit(CSF2RA)	Homo sapiens
2623	11738863_ turquoise	protein tyrosine phosphatase, non-receptor type 2(PTPN2)	Homo sapiens
2624	11738883_ turquoise	tumor necrosis factor superfamily member 14(TNFSF14)	Homo sapiens
2625	11738884_ turquoise	caspase 1(CASP1)	Homo sapiens
2626	11738900_ turquoise	small nucleolar RNA, C/D box 139(SNORD139)	Homo sapiens
2627	11738935_ turquoise	killer cell lectin like receptor C3(KLRC3)	Homo sapiens
2628	11738980_ turquoise	CD4 molecule(CD4)	Homo sapiens
2629	11738981_ turquoise	CD4 molecule(CD4)	Homo sapiens
2630	11738982_ turquoise	CD4 molecule(CD4)	Homo sapiens
2631	11739005_ turquoise	capping actin protein of muscle Z-line alpha subunit 1(CAPZA1)	Homo sapiens
2632	11739006_ turquoise	capping actin protein of muscle Z-line alpha subunit 1(CAPZA1)	Homo sapiens
2633	11739007_ turquoise	capping actin protein of muscle Z-line alpha subunit 1(CAPZA1)	Homo sapiens
2634	11739008_ turquoise	capping actin protein of muscle Z-line alpha subunit 1(CAPZA1)	Homo sapiens
2635	11739009_ turquoise	myosin heavy chain 9(MYH9)	Homo sapiens
2636	11739019_ turquoise	RAB5C, member RAS oncogene family(RAB5C)	Homo sapiens
2637	11739024_ turquoise	adenosine deaminase, RNA specific(ADAR)	Homo sapiens
2638	11739028_ turquoise	clathrin heavy chain(CLTC)	Homo sapiens
2639	11739031_ turquoise	ARP2 actin related protein 2 homolog(ACTR2)	Homo sapiens
2640	11739032_ turquoise	ARP2 actin related protein 2 homolog(ACTR2)	Homo sapiens
2641	11739043_ turquoise	LIM domain kinase 2(LIMK2)	Homo sapiens
2642	11739046_ turquoise	multimerin 2(MMRN2)	Homo sapiens
2643	11739052_ turquoise	solute carrier organic anion transporter family member 2B1(SLCO2B1)	Homo sapiens
2644	11739053_ turquoise	solute carrier organic anion transporter family member 2B1(SLCO2B1)	Homo sapiens
2645	11739054_ turquoise	protein tyrosine phosphatase, non-receptor type 6(PTPN6)	Homo sapiens
2646	11739055_ turquoise	protein tyrosine phosphatase, non-receptor type 6(PTPN6)	Homo sapiens
2647	11739059_ blue	OCIA domain containing 1(OCIAD1)	Homo sapiens
2648	11739075_ turquoise	nuclear factor, erythroid 2 like 1(NFE2L1)	Homo sapiens

2649	11739094_ turquoise	C-X-C motif chemokine receptor 4(CXCR4)	Homo sapiens
2650	11739113_ turquoise	ADP dependent glucokinase(ADPGK)	Homo sapiens
2651	11739118_ turquoise	canopy FGF signaling regulator 3(CNPY3)	Homo sapiens
2652	11739119_ turquoise	canopy FGF signaling regulator 3(CNPY3)	Homo sapiens
2653	11739142_ turquoise	heterogeneous nuclear ribonucleoprotein F(HNRNPF)	Homo sapiens
2654	11739148_ turquoise	SAM and HD domain containing deoxynucleoside triphosphate triphosphohydrolase 1(S	Homo sapiens
2655	11739149_ turquoise	SAM and HD domain containing deoxynucleoside triphosphate triphosphohydrolase 1(S	Homo sapiens
2656	11739157_ turquoise	thioredoxin related transmembrane protein 4(TMX4)	Homo sapiens
2657	11739168_ turquoise	GTPase, IMAP family member 6(GIMAP6)	Homo sapiens
2658	11739201_ brown	ATP synthase, H+ transporting, mitochondrial Fo complex subunit C3 (subunit 9)(ATP5G	Homo sapiens
2659	11739202_ turquoise	ATP synthase, H+ transporting, mitochondrial Fo complex subunit C3 (subunit 9)(ATP5G	Homo sapiens
2660	11739223_ turquoise	neurofibromin 1 pseudogene 9(NF1P9)	Homo sapiens
2661	11739230_ turquoise	ADP ribosylation factor like GTPase 4A(ARL4A)	Homo sapiens
2662	11739232_ turquoise	Ras association domain family member 2(RASSF2)	Homo sapiens
2663	11739262_ turquoise	vacuolar protein sorting 4 homolog B(VPS4B)	Homo sapiens
2664	11739280_ turquoise	C-X-C motif chemokine ligand 16(CXCL16)	Homo sapiens
2665	11739285_ turquoise	ADP ribosylation factor like GTPase 6 interacting protein 6(ARL6IP6)	Homo sapiens
2666	11739298_ turquoise	LRR binding FLII interacting protein 1(LRRFIP1)	Homo sapiens
2667	11739300_ brown	CDGSH iron sulfur domain 3(CISD3)	Homo sapiens
2668	11739334_ turquoise	protein tyrosine phosphatase, receptor type C(PTPRC)	Homo sapiens
2669	11739345_ blue	calmegin(CLGN)	Homo sapiens
2670	11739346_ turquoise	TNF receptor superfamily member 1A(TNFRSF1A)	Homo sapiens
2671	11739347_ turquoise	phosphatidylinositol-5-phosphate 4-kinase type 2 alpha(PIP4K2A)	Homo sapiens
2672	11739348_ turquoise	phosphatidylinositol-5-phosphate 4-kinase type 2 alpha(PIP4K2A)	Homo sapiens
2673	11739349_ turquoise	angiomotin(AMOT)	Homo sapiens
2674	11739364_ turquoise	family with sequence similarity 92 member A(FAM92A)	Homo sapiens
2675	11739375_ turquoise	synaptotagmin 7(SYT7)	Homo sapiens
2676	11739392_ turquoise	recombination signal binding protein for immunoglobulin kappa J region(RBPJ)	Homo sapiens
2677	11739400_ turquoise	F-box protein 7(FBXO7)	Homo sapiens
2678	11739401_ turquoise	CD74 molecule(CD74)	Homo sapiens
2679	11739419_ turquoise	uncharacterized LOC100130460(CAND1.11)	Homo sapiens
2680	11739437_ blue	PTC7 protein phosphatase homolog(PPTC7)	Homo sapiens
2681	11739452_ turquoise	malic enzyme 2(ME2)	Homo sapiens
2682	11739453_ blue	malic enzyme 2(ME2)	Homo sapiens
2683	11739465_ turquoise	alkB homolog 4, lysine demethylase(ALKBH4)	Homo sapiens
2684	11739470_ turquoise	transmembrane protein 87B(TMEM87B)	Homo sapiens
2685	11739482_ turquoise	placenta specific 8(PLAC8)	Homo sapiens
2686	11739484_ turquoise	coiled-coil domain containing 88A(CCDC88A)	Homo sapiens
2687	11739487_ turquoise	SUZ12 polycomb repressive complex 2 subunit(SUZ12)	Homo sapiens
2688	11739489_ turquoise	ATPase phospholipid transporting 10D (putative)(ATP10D)	Homo sapiens
2689	11739492_ turquoise	mannosyl (alpha-1,3-)-glycoprotein beta-1,4-N-acetylglucosaminyltransferase, isozyme	Homo sapiens
2690	11739493_ turquoise	mannosyl (alpha-1,3-)-glycoprotein beta-1,4-N-acetylglucosaminyltransferase, isozyme	Homo sapiens
2691	11739494_ turquoise	mannosyl (alpha-1,3-)-glycoprotein beta-1,4-N-acetylglucosaminyltransferase, isozyme	Homo sapiens
2692	11739501_ blue	desmoplakin(DSP)	Homo sapiens
2693	11739506_ blue	LON peptidase N-terminal domain and ring finger 2(LONRF2)	Homo sapiens
2694	11739507_ turquoise	PBX homeobox 3(PBX3)	Homo sapiens
2695	11739508_ blue	N(alpha)-acetyltransferase 30, NatC catalytic subunit(NAA30)	Homo sapiens
2696	11739512_ brown	translocase of inner mitochondrial membrane 50(TIMM50)	Homo sapiens
2697	11739521_ turquoise	ST8 alpha-N-acetyl-neuraminide alpha-2,8-sialyltransferase 4(ST8SIA4)	Homo sapiens
2698	11739522_ turquoise	ST8 alpha-N-acetyl-neuraminide alpha-2,8-sialyltransferase 4(ST8SIA4)	Homo sapiens

2699	11739527_turquoise	secreted and transmembrane 1(SECTM1)	Homo sapiens
2700	11739557_turquoise	vesicle associated membrane protein 5(VAMP5)	Homo sapiens
2701	11739558_turquoise	ring finger and WD repeat domain 3(RFWD3)	Homo sapiens
2702	11739576_turquoise	HPS5, biogenesis of lysosomal organelles complex 2 subunit 2(HPS5)	Homo sapiens
2703	11739581_turquoise	protein kinase D3(PRKD3)	Homo sapiens
2704	11739583_turquoise	protein kinase D3(PRKD3)	Homo sapiens
2705	11739584_turquoise	protein kinase D3(PRKD3)	Homo sapiens
2706	11739586_turquoise	chloride voltage-gated channel 3(CLCN3)	Homo sapiens
2707	11739606_turquoise	coiled-coil domain containing 88A(CCDC88A)	Homo sapiens
2708	11739610_turquoise	calmodulin like 4(CALML4)	Homo sapiens
2709	11739617_turquoise	CD44 molecule (Indian blood group)(CD44)	Homo sapiens
2710	11739654_turquoise	TNF receptor superfamily member 10a(TNFRSF10A)	Homo sapiens
2711	11739657_turquoise	lymphotoxin beta(LTB)	Homo sapiens
2712	11739658_turquoise	lymphotoxin beta(LTB)	Homo sapiens
2713	11739666_turquoise	RUN and cysteine rich domain containing beclin 1 interacting protein(RUBCN)	Homo sapiens
2714	11739667_turquoise	polypeptide N-acetylgalactosaminyltransferase 6(GALNT6)	Homo sapiens
2715	11739670_turquoise	GRB2-related adaptor protein(GRAP)	Homo sapiens
2716	11739672_turquoise	zinc finger protein 253(ZNF253)	Homo sapiens
2717	11739681_turquoise	aryl hydrocarbon receptor(AHR)	Homo sapiens
2718	11739687_turquoise	family with sequence similarity 107 member B(FAM107B)	Homo sapiens
2719	11739688_turquoise	family with sequence similarity 107 member B(FAM107B)	Homo sapiens
2720	11739730_turquoise	casein kinase 1 gamma 1(CSNK1G1)	Homo sapiens
2721	11739731_turquoise	casein kinase 1 gamma 1(CSNK1G1)	Homo sapiens
2722	11739739_turquoise	interleukin 21 receptor(IL21R)	Homo sapiens
2723	11739740_turquoise	interleukin 21 receptor(IL21R)	Homo sapiens
2724	11739747_blue	kelch like family member 7(KLHL7)	Homo sapiens
2725	11739753_turquoise	SLAM family member 8(SLAMF8)	Homo sapiens
2726	11739754_turquoise	SLAM family member 8(SLAMF8)	Homo sapiens
2727	11739767_turquoise	protein tyrosine phosphatase, receptor type E(PTPRE)	Homo sapiens
2728	11739768_turquoise	protein tyrosine phosphatase, receptor type E(PTPRE)	Homo sapiens
2729	11739796_turquoise	rhomboid 5 homolog 2(RHBDF2)	Homo sapiens
2730	11739811_blue	sirtuin 3(SIRT3)	Homo sapiens
2731	11739825_turquoise	DnaJ heat shock protein family (Hsp40) member B12(DNAJB12)	Homo sapiens
2732	11739828_turquoise	cystin 1(CYS1)	Homo sapiens
2733	11739845_turquoise	macrophage scavenger receptor 1(MSR1)	Homo sapiens
2734	11739846_turquoise	macrophage scavenger receptor 1(MSR1)	Homo sapiens
2735	11739847_turquoise	macrophage scavenger receptor 1(MSR1)	Homo sapiens
2736	11739854_brown	LYR motif containing 1(LYRM1)	Homo sapiens
2737	11739859_turquoise	protein kinase C and casein kinase substrate in neurons 1(PACSIN1)	Homo sapiens
2738	11739862_turquoise	integrator complex subunit 6 like(INTS6L)	Homo sapiens
2739	11739863_turquoise	transcription termination factor 2(TTF2)	Homo sapiens
2740	11739864_turquoise	transcription termination factor 2(TTF2)	Homo sapiens
2741	11739877_turquoise	basigin (Ok blood group)(BSG)	Homo sapiens
2742	11739911_brown	pterin-4 alpha-carbinolamine dehydratase 2(PCBD2)	Homo sapiens
2743	11739917_turquoise	casein kinase 1 delta(CSNK1D)	Homo sapiens
2744	11739941_turquoise	SUMO1/sentrin specific peptidase 1(SENP1)	Homo sapiens
2745	11739944_turquoise	neuroblastoma breakpoint family member 10(NBPF10)	Homo sapiens
2746	11740006_turquoise	KIAA0895(KIAA0895)	Homo sapiens
2747	11740035_turquoise	caspase recruitment domain family member 8(CARD8)	Homo sapiens
2748	11740036_turquoise	caspase recruitment domain family member 8(CARD8)	Homo sapiens

2749	11740038_	turquoise	ankyrin repeat domain 36(ANKRD36)	Homo sapiens
2750	11740043_	blue	mitochondrial methionyl-tRNA formyltransferase(MTFMT)	Homo sapiens
2751	11740044_	blue	mitochondrial methionyl-tRNA formyltransferase(MTFMT)	Homo sapiens
2752	11740061_	turquoise	c-src tyrosine kinase(CSK)	Homo sapiens
2753	11740065_	turquoise	interleukin 17 receptor A(IL17RA)	Homo sapiens
2754	11740077_	turquoise	discs large MAGUK scaffold protein 2(DLG2)	Homo sapiens
2755	11740080_	turquoise	chromosome X open reading frame 21(CXorf21)	Homo sapiens
2756	11740144_	turquoise	centrosomal protein 164(CEP164)	Homo sapiens
2757	11740145_	turquoise	centrosomal protein 164(CEP164)	Homo sapiens
2758	11740162_	turquoise	ArfGAP with RhoGAP domain, ankyrin repeat and PH domain 1(ARAP1)	Homo sapiens
2759	11740197_	turquoise	isoprenoid synthase domain containing(ISPD)	Homo sapiens
2760	11740212_	turquoise	phosphatidylinositol-4,5-bisphosphate 3-kinase catalytic subunit gamma(PIK3CG)	Homo sapiens
2761	11740238_	turquoise	sulfatase 2(SULF2)	Homo sapiens
2762	11740282_	turquoise	tRNA splicing endonuclease subunit 2(TSEN2)	Homo sapiens
2763	11740302_	turquoise	parvin gamma(PARVG)	Homo sapiens
2764	11740370_	turquoise	RAS guanyl releasing protein 1(RASGRP1)	Homo sapiens
2765	11740375_	turquoise	regulator of G-protein signaling 10(RGS10)	Homo sapiens
2766	11740378_	turquoise	aftiphilin(AFTPH)	Homo sapiens
2767	11740390_	turquoise	RNA binding motif protein 47(RBM47)	Homo sapiens
2768	11740393_	turquoise	TNF receptor superfamily member 9(TNFRSF9)	Homo sapiens
2769	11740411_	turquoise	protein kinase D2(PRKD2)	Homo sapiens
2770	11740412_	turquoise	protein kinase D2(PRKD2)	Homo sapiens
2771	11740429_	turquoise	5'-3' exoribonuclease 1(XRN1)	Homo sapiens
2772	11740444_	turquoise	E74 like ETS transcription factor 1(ELF1)	Homo sapiens
2773	11740447_	turquoise	butyrophilin subfamily 3 member A1(BTN3A1)	Homo sapiens
2774	11740450_	turquoise	killer cell lectin like receptor D1(KLRD1)	Homo sapiens
2775	11740451_	turquoise	killer cell lectin like receptor D1(KLRD1)	Homo sapiens
2776	11740452_	turquoise	killer cell lectin like receptor D1(KLRD1)	Homo sapiens
2777	11740465_	turquoise	G protein-coupled receptor 171(GPR171)	Homo sapiens
2778	11740486_	turquoise	runt related transcription factor 3(RUNX3)	Homo sapiens
2779	11740499_	turquoise	chromobox 5(CBX5)	Homo sapiens
2780	11740513_	blue	phosphodiesterase 4D(PDE4D)	Homo sapiens
2781	11740531_	turquoise	C-type lectin domain family 10 member A(CLEC10A)	Homo sapiens
2782	11740594_	turquoise	histone cluster 1 H3 family member e(HIST1H3E)	Homo sapiens
2783	11740606_	turquoise	hydroxylysine kinase(HYKK)	Homo sapiens
2784	11740616_	turquoise	parkin RBR E3 ubiquitin protein ligase(PARK2)	Homo sapiens
2785	11740617_	turquoise	parkin RBR E3 ubiquitin protein ligase(PARK2)	Homo sapiens
2786	11740621_	turquoise	lysine demethylase 2B(KDM2B)	Homo sapiens
2787	11740657_	turquoise	solute carrier family 22 member 5(SLC22A5)	Homo sapiens
2788	11740672_	turquoise	COX15, cytochrome c oxidase assembly homolog(COX15)	Homo sapiens
2789	11740677_	turquoise	GDP dissociation inhibitor 2(GDI2)	Homo sapiens
2790	11740681_	turquoise	TGFB induced factor homeobox 1(TGIF1)	Homo sapiens
2791	11740691_	turquoise	allograft inflammatory factor 1(AIF1)	Homo sapiens
2792	11740745_	blue	OPA1, mitochondrial dynamin like GTPase(OPA1)	Homo sapiens
2793	11740746_	blue	OPA1, mitochondrial dynamin like GTPase(OPA1)	Homo sapiens
2794	11740748_	turquoise	MYC associated factor X(MAX)	Homo sapiens
2795	11740752_	turquoise	folate receptor beta(FOLR2)	Homo sapiens
2796	11740758_	turquoise	mir-99a-let-7c cluster host gene(MIR99AHG)	Homo sapiens
2797	11740801_	turquoise	acyl-CoA dehydrogenase family member 10(ACAD10)	Homo sapiens
2798	11740821_	turquoise	CD1e molecule(CD1E)	Homo sapiens

2799	11740825_ turquoise	thymus, brain and testes associated(TBATA)	Homo sapiens
2800	11740829_ turquoise	synapse defective Rho GTPase homolog 2(SYDE2)	Homo sapiens
2801	11740871_ turquoise	membrane spanning 4-domains A7(MS4A7)	Homo sapiens
2802	11740872_ turquoise	membrane spanning 4-domains A7(MS4A7)	Homo sapiens
2803	11740873_ turquoise	membrane spanning 4-domains A7(MS4A7)	Homo sapiens
2804	11740874_ turquoise	FES proto-oncogene, tyrosine kinase(FES)	Homo sapiens
2805	11740879_ turquoise	annexin A2(ANXA2)	Homo sapiens
2806	11740880_ turquoise	annexin A2(ANXA2)	Homo sapiens
2807	11740881_ turquoise	interleukin 15(IL15)	Homo sapiens
2808	11740885_ turquoise	oxysterol binding protein like 3(OSBPL3)	Homo sapiens
2809	11740890_ turquoise	ADAM metallopeptidase domain 28(ADAM28)	Homo sapiens
2810	11740891_ turquoise	2'-5'-oligoadenylate synthetase 1(OAS1)	Homo sapiens
2811	11740934_ turquoise	nucleolar and spindle associated protein 1(NUSAP1)	Homo sapiens
2812	11740937_ turquoise	lipoic acid synthetase(LIAS)	Homo sapiens
2813	11740938_ turquoise	TGFB induced factor homeobox 1(TGIF1)	Homo sapiens
2814	11740957_ turquoise	solute carrier family 2 member 6(SLC2A6)	Homo sapiens
2815	11740958_ turquoise	centrosomal protein 63(CEP63)	Homo sapiens
2816	11740961_ turquoise	CD33 molecule(CD33)	Homo sapiens
2817	11740971_ turquoise	Ras and Rab interactor like(RINL)	Homo sapiens
2818	11740974_ turquoise	cytoplasmic FMR1 interacting protein 1(CYFIP1)	Homo sapiens
2819	11740979_ turquoise	LCK proto-oncogene, Src family tyrosine kinase(LCK)	Homo sapiens
2820	11740990_ blue	hyaluronoglucosaminidase 1(HYAL1)	Homo sapiens
2821	11740992_ turquoise	microRNA 6837(MIR6837)	Homo sapiens
2822	11740997_ turquoise	TRAF-type zinc finger domain containing 1(TRAFD1)	Homo sapiens
2823	11740998_ turquoise	diacylglycerol kinase alpha(DGKA)	Homo sapiens
2824	11741016_ turquoise	adaptor related protein complex 2 alpha 2 subunit(AP2A2)	Homo sapiens
2825	11741030_ turquoise	pentatricopeptide repeat domain 2(PTCD2)	Homo sapiens
2826	11741031_ brown	HCLS1 associated protein X-1(HAX1)	Homo sapiens
2827	11741035_ turquoise	ribosomal protein S6 kinase A1(RPS6KA1)	Homo sapiens
2828	11741039_ blue	death associated protein 3(DAP3)	Homo sapiens
2829	11741040_ blue	death associated protein 3(DAP3)	Homo sapiens
2830	11741047_ turquoise	vav guanine nucleotide exchange factor 1(VAV1)	Homo sapiens
2831	11741063_ turquoise	uncharacterized LOC101928143(LOC101928143)	Homo sapiens
2832	11741067_ turquoise	sulfatase modifying factor 2(SUMF2)	Homo sapiens
2833	11741076_ turquoise	caspase 1(CASP1)	Homo sapiens
2834	11741121_ turquoise	zinc finger protein 700(ZNF700)	Homo sapiens
2835	11741126_ turquoise	schlafen family member 11(SLFN11)	Homo sapiens
2836	11741129_ turquoise	calpain 2(CAPN2)	Homo sapiens
2837	11741133_ turquoise	heterogeneous nuclear ribonucleoprotein F(HNRNPF)	Homo sapiens
2838	11741138_ turquoise	gasdermin D(GSDMD)	Homo sapiens
2839	11741148_ blue	peroxiredoxin 2(PRDX2)	Homo sapiens
2840	11741153_ turquoise	neutrophil cytosolic factor 2(NCF2)	Homo sapiens
2841	11741158_ turquoise	renalase, FAD dependent amine oxidase(RNLS)	Homo sapiens
2842	11741184_ turquoise	uncharacterized LOC100130460(CAND1.11)	Homo sapiens
2843	11741187_ turquoise	apolipoprotein L1(APOL1)	Homo sapiens
2844	11741190_ turquoise	purinergic receptor P2Y10(P2RY10)	Homo sapiens
2845	11741192_ brown	ribokinase(RBKS)	Homo sapiens
2846	11741195_ turquoise	sialic acid binding Ig like lectin 14(SIGLEC14)	Homo sapiens
2847	11741202_ turquoise	XIAP associated factor 1(XAF1)	Homo sapiens
2848	11741226_ turquoise	EYA transcriptional coactivator and phosphatase 1(EYA1)	Homo sapiens

2849	11741229_ turquoise	family with sequence similarity 111 member A(FAM111A)	Homo sapiens
2850	11741250_ turquoise	SH2 domain containing 3C(SH2D3C)	Homo sapiens
2851	11741255_ turquoise	apolipoprotein B mRNA editing enzyme catalytic subunit 3F(APOBEC3F)	Homo sapiens
2852	11741257_ turquoise	apolipoprotein B mRNA editing enzyme catalytic subunit 3F(APOBEC3F)	Homo sapiens
2853	11741290_ turquoise	tumor necrosis factor superfamily member 14(TNFSF14)	Homo sapiens
2854	11741309_ turquoise	serine/threonine protein kinase 26(STK26)	Homo sapiens
2855	11741319_ turquoise	small nucleolar RNA, H/ACA box 29(SNORA29)	Homo sapiens
2856	11741326_ turquoise	endosulfine alpha(ENSA)	Homo sapiens
2857	11741365_ brown	intraflagellar transport 22(IFT22)	Homo sapiens
2858	11741402_ blue	chromosome 21 open reading frame 33(C21orf33)	Homo sapiens
2859	11741428_ blue	RNA binding protein, fox-1 homolog 1(RBFOX1)	Homo sapiens
2860	11741435_ turquoise	growth factor receptor bound protein 2(GRB2)	Homo sapiens
2861	11741438_ blue	calmegin(CLGN)	Homo sapiens
2862	11741477_ turquoise	nephrocystin 1(NPHP1)	Homo sapiens
2863	11741493_ turquoise	peroxisomal biogenesis factor 5(PEX5)	Homo sapiens
2864	11741519_ turquoise	CD14 molecule(CD14)	Homo sapiens
2865	11741520_ turquoise	CD14 molecule(CD14)	Homo sapiens
2866	11741535_ turquoise	transmembrane protein 2(TMEM2)	Homo sapiens
2867	11741536_ turquoise	transmembrane protein 2(TMEM2)	Homo sapiens
2868	11741554_ turquoise	caspase 8(CASP8)	Homo sapiens
2869	11741555_ turquoise	lipase A, lysosomal acid type(LIPA)	Homo sapiens
2870	11741567_ turquoise	solute carrier organic anion transporter family member 2B1(SLCO2B1)	Homo sapiens
2871	11741581_ turquoise	synaptosome associated protein 23(SNAP23)	Homo sapiens
2872	11741584_ turquoise	ankyrin repeat and SOCS box containing 10(ASB10)	Homo sapiens
2873	11741594_ turquoise	leukocyte specific transcript 1(LST1)	Homo sapiens
2874	11741605_ turquoise	chromodomain helicase DNA binding protein 3(CHD3)	Homo sapiens
2875	11741606_ turquoise	chromodomain helicase DNA binding protein 3(CHD3)	Homo sapiens
2876	11741619_ turquoise	sodium voltage-gated channel alpha subunit 7(SCN7A)	Homo sapiens
2877	11741632_ turquoise	transporter 2, ATP binding cassette subfamily B member(TAP2)	Homo sapiens
2878	11741650_ blue	LDL receptor related protein 12(LRP12)	Homo sapiens
2879	11741660_ turquoise	T-cell activation RhoGTPase activating protein(TAGAP)	Homo sapiens
2880	11741667_ turquoise	lymphocyte transmembrane adaptor 1(LAX1)	Homo sapiens
2881	11741686_ blue	family with sequence similarity 185 member A(FAM185A)	Homo sapiens
2882	11741689_ turquoise	sulfatase modifying factor 2(SUMF2)	Homo sapiens
2883	11741719_ turquoise	adenosine deaminase, RNA specific(ADAR)	Homo sapiens
2884	11741720_ turquoise	adenosine deaminase, RNA specific(ADAR)	Homo sapiens
2885	11741723_ turquoise	DENN domain containing 2C(DENND2C)	Homo sapiens
2886	11741749_ blue	leucine rich repeat containing 2(LRRC2)	Homo sapiens
2887	11741769_ turquoise	sorting nexin family member 21(SNX21)	Homo sapiens
2888	11741785_ turquoise	SH3 domain binding kinase family member 2(SBK2)	Homo sapiens
2889	11741789_ brown	NADH:ubiquinone oxidoreductase subunit B6(NDUFB6)	Homo sapiens
2890	11741793_ turquoise	fucose mutarotase(FUOM)	Homo sapiens
2891	11741796_ turquoise	SH3 domain binding protein 2(SH3BP2)	Homo sapiens
2892	11741797_ turquoise	collagen type IV alpha 6 chain(COL4A6)	Homo sapiens
2893	11741856_ turquoise	zinc finger protein 658(ZNF658)	Homo sapiens
2894	11741860_ turquoise	caveolin 3(CAV3)	Homo sapiens
2895	11741870_ turquoise	parkin RBR E3 ubiquitin protein ligase(PARK2)	Homo sapiens
2896	11741878_ turquoise	promyelocytic leukemia(PML)	Homo sapiens
2897	11741879_ turquoise	promyelocytic leukemia(PML)	Homo sapiens
2898	11741885_ turquoise	caspase 1(CASP1)	Homo sapiens

2899	11741911_ turquoise	guanylate binding protein 3(GBP3)	Homo sapiens
2900	11741980_ turquoise	dual specificity phosphatase 6(DUSP6)	Homo sapiens
2901	11741981_ turquoise	C-type lectin domain family 7 member A(CLEC7A)	Homo sapiens
2902	11741990_ turquoise	C-C motif chemokine receptor like 2(CCRL2)	Homo sapiens
2903	11742022_ turquoise	IKAROS family zinc finger 1(IKZF1)	Homo sapiens
2904	11742027_ turquoise	angiopoietin 2(ANGPT2)	Homo sapiens
2905	11742028_ turquoise	angiopoietin 2(ANGPT2)	Homo sapiens
2906	11742035_ blue	phospholipase C like 1(PLCL1)	Homo sapiens
2907	11742042_ turquoise	interleukin 1 receptor associated kinase 4(IRAK4)	Homo sapiens
2908	11742043_ turquoise	CD44 molecule (Indian blood group)(CD44)	Homo sapiens
2909	11742063_ turquoise	butyrophilin subfamily 3 member A1(BTN3A1)	Homo sapiens
2910	11742071_ turquoise	RAB27A, member RAS oncogene family(RAB27A)	Homo sapiens
2911	11742078_ turquoise	family with sequence similarity 222 member A(FAM222A)	Homo sapiens
2912	11742107_ turquoise	palmitoyl-protein thioesterase 1(PPT1)	Homo sapiens
2913	11742116_ turquoise	lactate dehydrogenase A(LDHA)	Homo sapiens
2914	11742119_ turquoise	nuclear transcription factor Y subunit gamma(NFYC)	Homo sapiens
2915	11742173_ turquoise	leukocyte specific transcript 1(LST1)	Homo sapiens
2916	11742182_ turquoise	POTE ankyrin domain family member E(POTEE)	Homo sapiens
2917	11742191_ turquoise	uncharacterized LOC100506403(LOC100506403)	Homo sapiens
2918	11742216_ turquoise	killer cell lectin like receptor D1(KLRD1)	Homo sapiens
2919	11742217_ turquoise	protein tyrosine phosphatase, receptor type A(PTPRA)	Homo sapiens
2920	11742241_ turquoise	IKAROS family zinc finger 3(IKZF3)	Homo sapiens
2921	11742273_ blue	brain and reproductive organ-expressed (TNFRSF1A modulator)(BRE)	Homo sapiens
2922	11742282_ turquoise	CD40 molecule(CD40)	Homo sapiens
2923	11742303_ turquoise	aspartate beta-hydroxylase(ASPH)	Homo sapiens
2924	11742331_ turquoise	ectodysplasin A(EDA)	Homo sapiens
2925	11742378_ turquoise	aldo-keto reductase family 1 member B15(AKR1B15)	Homo sapiens
2926	11742386_ turquoise	caspase 1(CASP1)	Homo sapiens
2927	11742439_ turquoise	adenosine deaminase like(ADAL)	Homo sapiens
2928	11742449_ turquoise	apolipoprotein L1(APOL1)	Homo sapiens
2929	11742456_ turquoise	microRNA 4745(MIR4745)	Homo sapiens
2930	11742457_ turquoise	microRNA 4745(MIR4745)	Homo sapiens
2931	11742482_ turquoise	actin related protein 2/3 complex subunit 4(ARPC4)	Homo sapiens
2932	11742537_ turquoise	BCL2 associated X, apoptosis regulator(BAX)	Homo sapiens
2933	11742678_ turquoise	microRNA 4745(MIR4745)	Homo sapiens
2934	11742680_ turquoise	heterogeneous nuclear ribonucleoprotein A2/B1(HNRNPA2B1)	Homo sapiens
2935	11742681_ turquoise	endoplasmic reticulum protein 29(ERP29)	Homo sapiens
2936	11742686_ turquoise	Fc fragment of IgG receptor and transporter(FCGRT)	Homo sapiens
2937	11742706_ turquoise	RAB8A, member RAS oncogene family(RAB8A)	Homo sapiens
2938	11742710_ turquoise	serglycin(SRGN)	Homo sapiens
2939	11742717_ turquoise	transforming growth factor beta regulator 1(TBRG1)	Homo sapiens
2940	11742723_ turquoise	signal transducer and activator of transcription 6(STAT6)	Homo sapiens
2941	11742724_ turquoise	signal transducer and activator of transcription 6(STAT6)	Homo sapiens
2942	11742735_ turquoise	nucleolar and spindle associated protein 1(NUSAP1)	Homo sapiens
2943	11742736_ brown	methylmalonyl-CoA epimerase(MCEE)	Homo sapiens
2944	11742740_ turquoise	gem nuclear organelle associated protein 5(GEMIN5)	Homo sapiens
2945	11742752_ turquoise	ETS proto-oncogene 2, transcription factor(ETS2)	Homo sapiens
2946	11742765_ turquoise	regulator of G-protein signaling 1(RGS1)	Homo sapiens
2947	11742784_ turquoise	senataxin(SETX)	Homo sapiens
2948	11742785_ turquoise	senataxin(SETX)	Homo sapiens

2949	11742798_ blue	chloride voltage-gated channel 3(CLCN3)	Homo sapiens
2950	11742800_ turquoise	E2F transcription factor 3(E2F3)	Homo sapiens
2951	11742832_ turquoise	abnormal spindle microtubule assembly(ASPM)	Homo sapiens
2952	11742833_ turquoise	family with sequence similarity 149 member A(FAM149A)	Homo sapiens
2953	11742838_ turquoise	arrestin beta 1(ARRB1)	Homo sapiens
2954	11742849_ turquoise	ral guanine nucleotide dissociation stimulator like 3(RGL3)	Homo sapiens
2955	11742850_ turquoise	major histocompatibility complex, class II, DR alpha(HLA-DRA)	Homo sapiens
2956	11742859_ turquoise	pleckstrin(PLEK)	Homo sapiens
2957	11742863_ turquoise	CD200 molecule(CD200)	Homo sapiens
2958	11742870_ turquoise	actin related protein 2/3 complex subunit 2(ARPC2)	Homo sapiens
2959	11742881_ turquoise	ubiquitin conjugating enzyme E2 B(UBE2B)	Homo sapiens
2960	11742889_ turquoise	F-box protein 6(FBXO6)	Homo sapiens
2961	11742892_ turquoise	proteasome subunit alpha 4(PSMA4)	Homo sapiens
2962	11742904_ turquoise	BCL2 associated athanogene 2(BAG2)	Homo sapiens
2963	11742905_ turquoise	DnaJ heat shock protein family (Hsp40) member C1(DNAJC1)	Homo sapiens
2964	11742906_ turquoise	DnaJ heat shock protein family (Hsp40) member C1(DNAJC1)	Homo sapiens
2965	11742911_ turquoise	immunoglobulin superfamily member 6(IGSF6)	Homo sapiens
2966	11742922_ turquoise	lysosomal protein transmembrane 5(LAPTM5)	Homo sapiens
2967	11742923_ turquoise	lysosomal protein transmembrane 5(LAPTM5)	Homo sapiens
2968	11742926_ turquoise	carnitine palmitoyltransferase 2(CPT2)	Homo sapiens
2969	11742943_ blue	creatine kinase, M-type(CKM)	Homo sapiens
2970	11742944_ turquoise	coronin 1A(CORO1A)	Homo sapiens
2971	11742948_ turquoise	myristoylated alanine rich protein kinase C substrate(MARCKS)	Homo sapiens
2972	11742950_ turquoise	CCR4-NOT transcription complex subunit 8(CNOT8)	Homo sapiens
2973	11742964_ turquoise	inositol 1,4,5-trisphosphate receptor type 3(ITPR3)	Homo sapiens
2974	11742973_ blue	acetyl-CoA acetyltransferase 1(ACAT1)	Homo sapiens
2975	11742981_ blue	fatty acid binding protein 3(FABP3)	Homo sapiens
2976	11742985_ turquoise	lymphocyte antigen 9(LY9)	Homo sapiens
2977	11742999_ turquoise	peroxiredoxin 6(PRDX6)	Homo sapiens
2978	11743000_ turquoise	CD83 molecule(CD83)	Homo sapiens
2979	11743002_ brown	dynactin subunit 6(DCTN6)	Homo sapiens
2980	11743003_ turquoise	cytochrome P450 family 4 subfamily B member 1(CYP4B1)	Homo sapiens
2981	11743007_ turquoise	NFKB inhibitor epsilon(NFKBIE)	Homo sapiens
2982	11743008_ turquoise	uncharacterized LOC100505585(LOC100505585)	Homo sapiens
2983	11743009_ turquoise	uncharacterized LOC100505585(LOC100505585)	Homo sapiens
2984	11743014_ turquoise	disco interacting protein 2 homolog C(DIP2C)	Homo sapiens
2985	11743015_ blue	disco interacting protein 2 homolog C(DIP2C)	Homo sapiens
2986	11743016_ turquoise	disco interacting protein 2 homolog C(DIP2C)	Homo sapiens
2987	11743036_ turquoise	spermidine/spermine N1-acetyltransferase 1(SAT1)	Homo sapiens
2988	11743048_ blue	aconitase 2(ACO2)	Homo sapiens
2989	11743055_ turquoise	phospholipase A2 activating protein(PLAA)	Homo sapiens
2990	11743062_ turquoise	plasminogen activator, urokinase receptor(PLAUR)	Homo sapiens
2991	11743063_ turquoise	plasminogen activator, urokinase receptor(PLAUR)	Homo sapiens
2992	11743065_ turquoise	cell division cycle 6(CDC6)	Homo sapiens
2993	11743071_ turquoise	lysophosphatidic acid receptor 6(LPAR6)	Homo sapiens
2994	11743076_ blue	protein activator of interferon induced protein kinase EIF2AK2(PRKRA)	Homo sapiens
2995	11743105_ turquoise	pentatricopeptide repeat domain 3(PTCD3)	Homo sapiens
2996	11743120_ turquoise	SENP3-EIF4A1 readthrough (NMD candidate)(SENP3-EIF4A1)	Homo sapiens
2997	11743127_ turquoise	coiled-coil-helix-coiled-coil-helix domain containing 10(CHCHD10)	Homo sapiens
2998	11743129_ turquoise	sortilin related receptor 1(SORL1)	Homo sapiens

2999	11743130_ turquoise	sortilin related receptor 1(SORL1)	Homo sapiens
3000	11743157_ turquoise	electron transfer flavoprotein dehydrogenase(ETFDH)	Homo sapiens
3001	11743166_ turquoise	ETS variant 6(ETV6)	Homo sapiens
3002	11743167_ turquoise	ETS variant 6(ETV6)	Homo sapiens
3003	11743168_ turquoise	indoleamine 2,3-dioxygenase 1(IDO1)	Homo sapiens
3004	11743169_ turquoise	leupaxin(LPXN)	Homo sapiens
3005	11743180_ turquoise	nuclear protein, coactivator of histone transcription(NPAT)	Homo sapiens
3006	11743194_ turquoise	TNF receptor superfamily member 14(TNFRSF14)	Homo sapiens
3007	11743196_ turquoise	toll like receptor 4(TLR4)	Homo sapiens
3008	11743197_ turquoise	toll like receptor 4(TLR4)	Homo sapiens
3009	11743264_ brown	chromosome 14 open reading frame 2(C14orf2)	Homo sapiens
3010	11743290_ turquoise	polypeptide N-acetylgalactosaminyltransferase 1(GALNT1)	Homo sapiens
3011	11743299_ turquoise	signal recognition particle 72(SRP72)	Homo sapiens
3012	11743301_ turquoise	signal recognition particle 72(SRP72)	Homo sapiens
3013	11743321_ turquoise	cathepsin O(CTSO)	Homo sapiens
3014	11743330_ brown	phosphoglycolate phosphatase(PGP)	Homo sapiens
3015	11743332_ turquoise	mitochondrial ribosomal protein L35(MRPL35)	Homo sapiens
3016	11743333_ turquoise	mitochondrial ribosomal protein L35(MRPL35)	Homo sapiens
3017	11743334_ turquoise	mitochondrial ribosomal protein L35(MRPL35)	Homo sapiens
3018	11743348_ turquoise	CD47 molecule(CD47)	Homo sapiens
3019	11743350_ turquoise	chromosome 15 open reading frame 48(C15orf48)	Homo sapiens
3020	11743352_ turquoise	cordon-bleu WH2 repeat protein like 1(COBLL1)	Homo sapiens
3021	11743353_ turquoise	signal transducer and activator of transcription 1(STAT1)	Homo sapiens
3022	11743361_ turquoise	adenylosuccinate synthase(ADSS)	Homo sapiens
3023	11743362_ turquoise	adenylosuccinate synthase(ADSS)	Homo sapiens
3024	11743377_ turquoise	ninein(NIN)	Homo sapiens
3025	11743379_ turquoise	abl interactor 1(ABI1)	Homo sapiens
3026	11743386_ turquoise	pre-mRNA processing factor 40 homolog A(PRPF40A)	Homo sapiens
3027	11743394_ turquoise	androgen induced 1(AIG1)	Homo sapiens
3028	11743406_ turquoise	small integral membrane protein 14(SMIM14)	Homo sapiens
3029	11743428_ turquoise	runt related transcription factor 3(RUNX3)	Homo sapiens
3030	11743433_ turquoise	6-phosphogluconolactonase(PGLS)	Homo sapiens
3031	11743452_ turquoise	cell division cycle associated 4(CDCA4)	Homo sapiens
3032	11743458_ turquoise	family with sequence similarity 49 member A(FAM49A)	Homo sapiens
3033	11743474_ brown	small integral membrane protein 4(SMIM4)	Homo sapiens
3034	11743475_ turquoise	integrin subunit alpha X(ITGAX)	Homo sapiens
3035	11743501_ turquoise	TNF receptor associated factor 3(TRAF3)	Homo sapiens
3036	11743505_ blue	asparaginyl-tRNA synthetase 2, mitochondrial (putative)(NARS2)	Homo sapiens
3037	11743517_ blue	nicotinamide nucleotide transhydrogenase(NNT)	Homo sapiens
3038	11743530_ turquoise	centromere protein K(CENPK)	Homo sapiens
3039	11743533_ turquoise	RUN and FYVE domain containing 3(RUFY3)	Homo sapiens
3040	11743555_ turquoise	queuine tRNA-ribosyltransferase accessory subunit 2(QTRT2)	Homo sapiens
3041	11743559_ turquoise	protein tyrosine phosphatase, receptor type C(PTPRC)	Homo sapiens
3042	11743560_ turquoise	protein tyrosine phosphatase, receptor type C(PTPRC)	Homo sapiens
3043	11743561_ turquoise	protein tyrosine phosphatase, receptor type C(PTPRC)	Homo sapiens
3044	11743570_ turquoise	echinoderm microtubule associated protein like 1(EML1)	Homo sapiens
3045	11743581_ turquoise	ARP2 actin related protein 2 homolog(ACTR2)	Homo sapiens
3046	11743586_ turquoise	poly(A) RNA polymerase D4, non-canonical(PAPD4)	Homo sapiens
3047	11743596_ turquoise	protein tyrosine phosphatase, receptor type E(PTPRE)	Homo sapiens
3048	11743613_ turquoise	tropomodulin 3(TMOD3)	Homo sapiens

3049	11743614_ turquoise	ubiquitin specific peptidase 47(USP47)	Homo sapiens
3050	11743619_ turquoise	dihydrouridine synthase 4 like(DUS4L)	Homo sapiens
3051	11743624_ turquoise	translocase of inner mitochondrial membrane 22(TIMM22)	Homo sapiens
3052	11743635_ turquoise	zinc and ring finger 2(ZNRF2)	Homo sapiens
3053	11743643_ turquoise	dihydroorotate dehydrogenase (quinone)(DHODH)	Homo sapiens
3054	11743647_ turquoise	high mobility group nucleosomal binding domain 4(HMGN4)	Homo sapiens
3055	11743648_ blue	DDB1 and CUL4 associated factor 6(DCAF6)	Homo sapiens
3056	11743658_ turquoise	H2A histone family member Y2(H2AFY2)	Homo sapiens
3057	11743677_ turquoise	purinergic receptor P2Y8(P2RY8)	Homo sapiens
3058	11743691_ turquoise	mitochondrial ribosomal protein L30(MRPL30)	Homo sapiens
3059	11743704_ turquoise	abhydrolase domain containing 11(ABHD11)	Homo sapiens
3060	11743709_ turquoise	N(alpha)-acetyltransferase 15, NatA auxiliary subunit(NAA15)	Homo sapiens
3061	11743722_ turquoise	MARCKS like 1(MARCKSL1)	Homo sapiens
3062	11743725_ turquoise	major facilitator superfamily domain containing 1(MFSD1)	Homo sapiens
3063	11743726_ turquoise	ras homolog family member A(RHOA)	Homo sapiens
3064	11743728_ blue	coiled-coil domain containing 47(CCDC47)	Homo sapiens
3065	11743730_ turquoise	tumor necrosis factor superfamily member 10(TNFSF10)	Homo sapiens
3066	11743731_ turquoise	tumor necrosis factor superfamily member 10(TNFSF10)	Homo sapiens
3067	11743736_ turquoise	motile sperm domain containing 2(MOSPD2)	Homo sapiens
3068	11743748_ turquoise	linker for activation of T-cells(LAT)	Homo sapiens
3069	11743759_ turquoise	CDC42 small effector 2(CDC42SE2)	Homo sapiens
3070	11743760_ turquoise	CDC42 small effector 2(CDC42SE2)	Homo sapiens
3071	11743771_ turquoise	GTPase, IMAP family member 7(GIMAP7)	Homo sapiens
3072	11743781_ turquoise	5'-3' exoribonuclease 1(XRN1)	Homo sapiens
3073	11743808_ turquoise	7-dehydrocholesterol reductase(DHCR7)	Homo sapiens
3074	11743813_ turquoise	ecotropic viral integration site 2B(EVI2B)	Homo sapiens
3075	11743824_ turquoise	topoisomerase (DNA) II binding protein 1(TOPBP1)	Homo sapiens
3076	11743841_ turquoise	phosphatidylinositol specific phospholipase C X domain containing 2(PLCXD2)	Homo sapiens
3077	11743843_ turquoise	DnaJ heat shock protein family (Hsp40) member C28(DNAJC28)	Homo sapiens
3078	11743856_ turquoise	collagen triple helix repeat containing 1(CTHRC1)	Homo sapiens
3079	11743863_ turquoise	ATPase plasma membrane Ca2+ transporting 1(ATP2B1)	Homo sapiens
3080	11743864_ turquoise	ATPase plasma membrane Ca2+ transporting 1(ATP2B1)	Homo sapiens
3081	11743865_ turquoise	ATPase plasma membrane Ca2+ transporting 1(ATP2B1)	Homo sapiens
3082	11743881_ turquoise	melanoregulin(MREG)	Homo sapiens
3083	11743887_ blue	S-phase response (cyclin related)(SPHAR)	Homo sapiens
3084	11743888_ turquoise	zinc finger protein 10(ZNF10)	Homo sapiens
3085	11743890_ turquoise	zinc finger protein 10(ZNF10)	Homo sapiens
3086	11743900_ turquoise	class II major histocompatibility complex transactivator(CIITA)	Homo sapiens
3087	11743911_ turquoise	phospholipid scramblase 1(PLSCR1)	Homo sapiens
3088	11743915_ turquoise	SEL1L family member 3(SEL1L3)	Homo sapiens
3089	11743916_ turquoise	NPC intracellular cholesterol transporter 1(NPC1)	Homo sapiens
3090	11743931_ turquoise	endoplasmic reticulum protein 29(ERP29)	Homo sapiens
3091	11743960_ turquoise	microRNA 6787(MIR6787)	Homo sapiens
3092	11743968_ turquoise	cytochrome b-245 alpha chain(CYBA)	Homo sapiens
3093	11743979_ blue	ubiquinol-cytochrome c reductase core protein I(UQCRC1)	Homo sapiens
3094	11743984_ turquoise	A-kinase anchoring protein 1(AKAP1)	Homo sapiens
3095	11743985_ turquoise	A-kinase anchoring protein 1(AKAP1)	Homo sapiens
3096	11743986_ turquoise	A-kinase anchoring protein 1(AKAP1)	Homo sapiens
3097	11744000_ turquoise	NFKB inhibitor alpha(NFKBIA)	Homo sapiens
3098	11744002_ turquoise	methylenetetrahydrofolate dehydrogenase (NADP+ dependent) 2, methenyltetrahydro	Homo sapiens

3099	11744006_ turquoise	vinculin(VCL)	Homo sapiens
3100	11744017_ turquoise	fermitin family member 3(FERMT3)	Homo sapiens
3101	11744020_ turquoise	nucleoporin 210(NUP210)	Homo sapiens
3102	11744027_ turquoise	transmembrane protein 181(TMEM181)	Homo sapiens
3103	11744033_ turquoise	S100 calcium binding protein A11(S100A11)	Homo sapiens
3104	11744034_ turquoise	vasodilator-stimulated phosphoprotein(VASP)	Homo sapiens
3105	11744037_ turquoise	c-Maf inducing protein(CMIP)	Homo sapiens
3106	11744040_ brown	cytochrome c oxidase assembly factor 3(COA3)	Homo sapiens
3107	11744054_ turquoise	zinc finger NFX1-type containing 1(ZNFX1)	Homo sapiens
3108	11744059_ blue	ubiquinol-cytochrome c reductase core protein II(UQCRC2)	Homo sapiens
3109	11744060_ blue	ubiquinol-cytochrome c reductase core protein II(UQCRC2)	Homo sapiens
3110	11744077_ turquoise	actin related protein 2/3 complex subunit 1B(ARPC1B)	Homo sapiens
3111	11744082_ turquoise	mitochondrial ribosomal protein S31(MRPS31)	Homo sapiens
3112	11744118_ turquoise	mitochondrial ribosomal protein L10(MRPL10)	Homo sapiens
3113	11744119_ turquoise	microRNA 6734(MIR6734)	Homo sapiens
3114	11744129_ turquoise	myosin IXB(MYO9B)	Homo sapiens
3115	11744134_ turquoise	zinc finger SWIM-type containing 6(ZSWIM6)	Homo sapiens
3116	11744137_ turquoise	calcium/calmodulin dependent protein kinase ID(CAMK1D)	Homo sapiens
3117	11744138_ turquoise	calcium/calmodulin dependent protein kinase ID(CAMK1D)	Homo sapiens
3118	11744148_ brown	solute carrier family 25 member 10(SLC25A10)	Homo sapiens
3119	11744149_ turquoise	lipin 2(LPIN2)	Homo sapiens
3120	11744150_ turquoise	lipin 2(LPIN2)	Homo sapiens
3121	11744151_ turquoise	lipin 2(LPIN2)	Homo sapiens
3122	11744153_ turquoise	reticulon 1(RTN1)	Homo sapiens
3123	11744156_ turquoise	ribosomal protein S6 kinase A1(RPS6KA1)	Homo sapiens
3124	11744162_ turquoise	dual specificity phosphatase 4(DUSP4)	Homo sapiens
3125	11744166_ blue	fumarate hydratase(FH)	Homo sapiens
3126	11744172_ brown	DnaJ heat shock protein family (Hsp40) member C4(DNAJC4)	Homo sapiens
3127	11744176_ turquoise	protein phosphatase 1 regulatory subunit 9B(PPP1R9B)	Homo sapiens
3128	11744178_ turquoise	chromosome 19 open reading frame 66(C19orf66)	Homo sapiens
3129	11744180_ turquoise	T-cell immune regulator 1, ATPase H+ transporting V0 subunit a3(TCIRG1)	Homo sapiens
3130	11744181_ brown	phenylalanyl-tRNA synthetase 2, mitochondrial(FARS2)	Homo sapiens
3131	11744182_ blue	phenylalanyl-tRNA synthetase 2, mitochondrial(FARS2)	Homo sapiens
3132	11744197_ turquoise	myosin light chain 12B(MYL12B)	Homo sapiens
3133	11744202_ brown	chromosome 19 open reading frame 70(C19orf70)	Homo sapiens
3134	11744207_ brown	isocitrate dehydrogenase 3 (NAD(+)) beta(IDH3B)	Homo sapiens
3135	11744209_ turquoise	serpin family H member 1(SERPINH1)	Homo sapiens
3136	11744214_ turquoise	TRAF-type zinc finger domain containing 1(TRAFD1)	Homo sapiens
3137	11744215_ turquoise	ring finger protein 149(RNF149)	Homo sapiens
3138	11744222_ brown	ATP synthase, H+ transporting, mitochondrial F1 complex, delta subunit(ATP5D)	Homo sapiens
3139	11744223_ brown	ATP synthase, H+ transporting, mitochondrial F1 complex, delta subunit(ATP5D)	Homo sapiens
3140	11744227_ brown	MACRO domain containing 1(MACROD1)	Homo sapiens
3141	11744236_ turquoise	DExD/H-box helicase 60(DDX60)	Homo sapiens
3142	11744258_ turquoise	ATPase Na+/K+ transporting subunit beta 3(ATP1B3)	Homo sapiens
3143	11744259_ turquoise	ATPase Na+/K+ transporting subunit beta 3(ATP1B3)	Homo sapiens
3144	11744261_ blue	solute carrier family 25 member 11(SLC25A11)	Homo sapiens
3145	11744263_ turquoise	ras-related C3 botulinum toxin substrate 2 (rho family, small GTP binding protein Rac2)(	Homo sapiens
3146	11744273_ turquoise	stem-loop binding protein(SLBP)	Homo sapiens
3147	11744274_ turquoise	NDC80, kinetochore complex component(NDC80)	Homo sapiens
3148	11744275_ blue	WD repeat domain 12(WDR12)	Homo sapiens

3149	11744276_	turquoise	capping actin protein, gelsolin like(CAPG)	Homo sapiens
3150	11744284_	brown	mitochondrial ribosomal protein S12(MRPS12)	Homo sapiens
3151	11744300_	turquoise	carbohydrate sulfotransferase 15(CHST15)	Homo sapiens
3152	11744323_	turquoise	PWWP domain containing 2A(PWWP2A)	Homo sapiens
3153	11744331_	turquoise	complement factor B(CFB)	Homo sapiens
3154	11744345_	turquoise	tubulin alpha 1b(TUBA1B)	Homo sapiens
3155	11744351_	turquoise	NLR family pyrin domain containing 1(NLRP1)	Homo sapiens
3156	11744353_	turquoise	TatD DNase domain containing 2(TATDN2)	Homo sapiens
3157	11744360_	turquoise	reticulon 4(RTN4)	Homo sapiens
3158	11744364_	turquoise	inositol polyphosphate-5-phosphatase D(INPP5D)	Homo sapiens
3159	11744374_	turquoise	major histocompatibility complex, class II, DR beta 1(HLA-DRB1)	Homo sapiens
3160	11744384_	brown	up-regulated during skeletal muscle growth 5 homolog (mouse)(USMG5)	Homo sapiens
3161	11744400_	brown	myosin light chain 6B(MYL6B)	Homo sapiens
3162	11744401_	brown	calmodulin binding transcription activator 1(CAMTA1)	Homo sapiens
3163	11744425_	turquoise	KIAA0101(KIAA0101)	Homo sapiens
3164	11744426_	turquoise	KIAA0101(KIAA0101)	Homo sapiens
3165	11744430_	brown	kinesin family member 9(KIF9)	Homo sapiens
3166	11744434_	turquoise	poly(ADP-ribose) polymerase family member 9(PARP9)	Homo sapiens
3167	11744435_	turquoise	dual specificity phosphatase 6(DUSP6)	Homo sapiens
3168	11744436_	turquoise	microRNA 1178(MIR1178)	Homo sapiens
3169	11744441_	turquoise	brain abundant membrane attached signal protein 1(BASP1)	Homo sapiens
3170	11744444_	brown	NADH:ubiquinone oxidoreductase subunit B10(NDUFB10)	Homo sapiens
3171	11744449_	turquoise	Rho GTPase activating protein 4(ARHGAP4)	Homo sapiens
3172	11744450_	turquoise	Rho GTPase activating protein 4(ARHGAP4)	Homo sapiens
3173	11744455_	brown	ubiquinol-cytochrome c reductase hinge protein like(UQCRHL)	Homo sapiens
3174	11744462_	blue	PCI domain containing 2(PCID2)	Homo sapiens
3175	11744482_	turquoise	transmembrane protein 246(TMEM246)	Homo sapiens
3176	11744484_	turquoise	major histocompatibility complex, class II, DR alpha(HLA-DRA)	Homo sapiens
3177	11744485_	turquoise	WD repeat domain 81(WDR81)	Homo sapiens
3178	11744486_	turquoise	WD repeat domain 81(WDR81)	Homo sapiens
3179	11744495_	turquoise	intraflagellar transport 46(IFT46)	Homo sapiens
3180	11744503_	turquoise	arginine and serine rich protein 1(RSRP1)	Homo sapiens
3181	11744512_	turquoise	TraB domain containing 2A(TRABD2A)	Homo sapiens
3182	11744525_	turquoise	mitochondrial transcription termination factor 2(MTERF2)	Homo sapiens
3183	11744545_	brown	GTF2I repeat domain containing 2B(GTF2IRD2B)	Homo sapiens
3184	11744560_	turquoise	microRNA 4723(MIR4723)	Homo sapiens
3185	11744562_	turquoise	eva-1 homolog B(EVA1B)	Homo sapiens
3186	11744567_	turquoise	CD72 molecule(CD72)	Homo sapiens
3187	11744599_	turquoise	nucleoporin 62(NUP62)	Homo sapiens
3188	11744608_	turquoise	poly(ADP-ribose) polymerase family member 3(PARP3)	Homo sapiens
3189	11744609_	turquoise	ataxin 7(ATXN7)	Homo sapiens
3190	11744611_	turquoise	paternally expressed 3(PEG3)	Homo sapiens
3191	11744612_	turquoise	NudC domain containing 1(NUDCD1)	Homo sapiens
3192	11744613_	turquoise	solute carrier family 6 member 13(SLC6A13)	Homo sapiens
3193	11744618_	turquoise	dual specificity phosphatase 6(DUSP6)	Homo sapiens
3194	11744632_	blue	COX11, cytochrome c oxidase copper chaperone(COX11)	Homo sapiens
3195	11744638_	turquoise	SND1 intronic transcript 1(SND1-IT1)	Homo sapiens
3196	11744645_	turquoise	transcobalamin 2(TCN2)	Homo sapiens
3197	11744652_	turquoise	hyaluronan and proteoglycan link protein 2(HAPLN2)	Homo sapiens
3198	11744654_	turquoise	CD44 molecule (Indian blood group)(CD44)	Homo sapiens

3199	11744660_turquoise	C-C motif chemokine ligand 4 like 1(CCL4L1)	Homo sapiens
3200	11744663_turquoise	target of myb1 like 1 membrane trafficking protein(TOM1L1)	Homo sapiens
3201	11744672_brown	MORN repeat containing 4(MORN4)	Homo sapiens
3202	11744673_turquoise	SP100 nuclear antigen(SP100)	Homo sapiens
3203	11744675_turquoise	family with sequence similarity 208 member A(FAM208A)	Homo sapiens
3204	11744699_turquoise	tyrosyl-DNA phosphodiesterase 1(TDP1)	Homo sapiens
3205	11744700_turquoise	tyrosyl-DNA phosphodiesterase 1(TDP1)	Homo sapiens
3206	11744715_turquoise	solute carrier family 38 member 6(SLC38A6)	Homo sapiens
3207	11744717_turquoise	chromosome 18 open reading frame 8(C18orf8)	Homo sapiens
3208	11744718_turquoise	collagen triple helix repeat containing 1(CTHRC1)	Homo sapiens
3209	11744728_turquoise	golgin A4(GOLGA4)	Homo sapiens
3210	11744735_turquoise	transmembrane protein 200A(TMEM200A)	Homo sapiens
3211	11744749_turquoise	DnaJ heat shock protein family (Hsp40) member C11(DNAJC11)	Homo sapiens
3212	11744751_turquoise	serpin family B member 9(SERPINB9)	Homo sapiens
3213	11744761_turquoise	uncharacterized LOC100505585(LOC100505585)	Homo sapiens
3214	11744776_turquoise	nicotinamide N-methyltransferase(NNMT)	Homo sapiens
3215	11744783_turquoise	SP100 nuclear antigen(SP100)	Homo sapiens
3216	11744786_turquoise	opioid growth factor receptor(OGFR)	Homo sapiens
3217	11744789_turquoise	centromere protein U(CENPU)	Homo sapiens
3218	11744791_turquoise	fibronectin type III domain containing 5(FNDC5)	Homo sapiens
3219	11744793_turquoise	DLG associated protein 5(DLGAP5)	Homo sapiens
3220	11744796_turquoise	DAB2, clathrin adaptor protein(DAB2)	Homo sapiens
3221	11744797_turquoise	DAB2, clathrin adaptor protein(DAB2)	Homo sapiens
3222	11744800_turquoise	fms related tyrosine kinase 3 ligand(FLT3LG)	Homo sapiens
3223	11744809_turquoise	BORCS7-ASMT readthrough (NMD candidate)(BORCS7-ASMT)	Homo sapiens
3224	11744822_brown	NADH:ubiquinone oxidoreductase subunit B2(NDUFB2)	Homo sapiens
3225	11744829_turquoise	major histocompatibility complex, class I, E(HLA-E)	Homo sapiens
3226	11744830_turquoise	nuclear pore complex interacting protein family member B5(NPIPB5)	Homo sapiens
3227	11744838_brown	dual specificity phosphatase 28(DUSP28)	Homo sapiens
3228	11744887_turquoise	zwilch kinetochore protein(ZWILCH)	Homo sapiens
3229	11744888_turquoise	lamin B1(LMNB1)	Homo sapiens
3230	11744889_turquoise	lamin B1(LMNB1)	Homo sapiens
3231	11744896_turquoise	G-protein signaling modulator 3(GPSM3)	Homo sapiens
3232	11744898_turquoise	proline rich coiled-coil 2C(PRRC2C)	Homo sapiens
3233	11744901_blue	NADH:ubiquinone oxidoreductase core subunit S1(NDUFS1)	Homo sapiens
3234	11744920_turquoise	outer dense fiber of sperm tails 3B(ODF3B)	Homo sapiens
3235	11744940_turquoise	abhydrolase domain containing 17A(ABHD17A)	Homo sapiens
3236	11744948_turquoise	semaphorin 3F(SEMA3F)	Homo sapiens
3237	11744953_turquoise	annexin A1(ANXA1)	Homo sapiens
3238	11744954_turquoise	annexin A1(ANXA1)	Homo sapiens
3239	11744955_turquoise	annexin A1(ANXA1)	Homo sapiens
3240	11744962_turquoise	ADP-ribosyltransferase 5(ART5)	Homo sapiens
3241	11744978_brown	origin recognition complex subunit 4(ORC4)	Homo sapiens
3242	11744991_turquoise	acetyl-CoA carboxylase beta(ACACB)	Homo sapiens
3243	11744993_turquoise	sirtuin 7(SIRT7)	Homo sapiens
3244	11744995_turquoise	pantothenate kinase 1(PANK1)	Homo sapiens
3245	11745015_turquoise	SP100 nuclear antigen(SP100)	Homo sapiens
3246	11745021_turquoise	v-myc avian myelocytomatosis viral oncogene homolog(MYC)	Homo sapiens
3247	11745063_turquoise	leucine rich repeat containing 8 family member B(LRRC8B)	Homo sapiens
3248	11745084_turquoise	family with sequence similarity 168 member B(FAM168B)	Homo sapiens

3249	11745105_ turquoise	meiotic double-stranded break formation protein 1(MEI1)	Homo sapiens
3250	11745114_ turquoise	adhesion G protein-coupled receptor E2(ADGRE2)	Homo sapiens
3251	11745124_ brown	3-hydroxybutyrate dehydrogenase, type 1(BDH1)	Homo sapiens
3252	11745144_ turquoise	C-type lectin domain family 4 member E(CLEC4E)	Homo sapiens
3253	11745163_ blue	hydroxyacyl-CoA dehydrogenase/3-ketoacyl-CoA thiolase/enoyl-CoA hydratase (trifunct	Homo sapiens
3254	11745165_ turquoise	TRAF3 interacting protein 3(TRAF3IP3)	Homo sapiens
3255	11745171_ turquoise	diaphanous related formin 1(DIAPH1)	Homo sapiens
3256	11745185_ blue	mitochondrial fission factor(MFF)	Homo sapiens
3257	11745189_ turquoise	apolipoprotein L1(APOL1)	Homo sapiens
3258	11745190_ turquoise	FUS RNA binding protein(FUS)	Homo sapiens
3259	11745192_ turquoise	erythrocyte membrane protein band 4.1 like 3(EPB41L3)	Homo sapiens
3260	11745230_ blue	T-cell activation inhibitor, mitochondrial(TCAIM)	Homo sapiens
3261	11745232_ turquoise	N-6 adenine-specific DNA methyltransferase 1 (putative)(N6AMT1)	Homo sapiens
3262	11745234_ turquoise	3-hydroxyacyl-CoA dehydratase 4(HACD4)	Homo sapiens
3263	11745243_ turquoise	neuroblastoma breakpoint family member 11(NBPF11)	Homo sapiens
3264	11745244_ yellow	microRNA 8071-1(MIR8071-1)	Homo sapiens
3265	11745248_ turquoise	ilvB acetolactate synthase like(ILVBL)	Homo sapiens
3266	11745259_ brown	chromosome 11 open reading frame 21(C11orf21)	Homo sapiens
3267	11745266_ turquoise	transporter 2, ATP binding cassette subfamily B member(TAP2)	Homo sapiens
3268	11745268_ turquoise	tensin 1(TNS1)	Homo sapiens
3269	11745274_ turquoise	par-3 family cell polarity regulator(PARD3)	Homo sapiens
3270	11745276_ turquoise	sterol O-acyltransferase 1(SOAT1)	Homo sapiens
3271	11745289_ turquoise	LIM domain and actin binding 1(LIMA1)	Homo sapiens
3272	11745302_ turquoise	StAR related lipid transfer domain containing 3(STARD3)	Homo sapiens
3273	11745313_ turquoise	synergin gamma(SYNRG)	Homo sapiens
3274	11745364_ turquoise	signal transducer and activator of transcription 5A(STAT5A)	Homo sapiens
3275	11745376_ turquoise	apolipoprotein L6(APOL6)	Homo sapiens
3276	11745384_ brown	NADH:ubiquinone oxidoreductase subunit B4(NDUFB4)	Homo sapiens
3277	11745403_ turquoise	zinc finger protein 683(ZNF683)	Homo sapiens
3278	11745406_ turquoise	MICAL like 2(MICALL2)	Homo sapiens
3279	11745409_ turquoise	La ribonucleoprotein domain family member 1B(LARP1B)	Homo sapiens
3280	11745415_ turquoise	diaphanous related formin 1(DIAPH1)	Homo sapiens
3281	11745421_ turquoise	BH3 interacting domain death agonist(BID)	Homo sapiens
3282	11745440_ turquoise	RELA proto-oncogene, NF-kB subunit(RELA)	Homo sapiens
3283	11745460_ brown	NADH:ubiquinone oxidoreductase subunit V3(NDUFV3)	Homo sapiens
3284	11745462_ blue	peroxiredoxin 2(PRDX2)	Homo sapiens
3285	11745468_ turquoise	gamma-aminobutyric acid type B receptor subunit 2(GABBR2)	Homo sapiens
3286	11745471_ turquoise	zinc finger protein 276(ZNF276)	Homo sapiens
3287	11745477_ turquoise	tweety family member 1(TTYH1)	Homo sapiens
3288	11745479_ turquoise	glycerate kinase(GLYCTK)	Homo sapiens
3289	11745496_ turquoise	serine peptidase inhibitor, Kunitz type 2(SPINT2)	Homo sapiens
3290	11745498_ turquoise	interferon regulatory factor 3(IRF3)	Homo sapiens
3291	11745499_ turquoise	interferon regulatory factor 3(IRF3)	Homo sapiens
3292	11745500_ turquoise	C-type lectin domain family 10 member A(CLEC10A)	Homo sapiens
3293	11745501_ turquoise	endothelial cell surface expressed chemotaxis and apoptosis regulator(ECSCR)	Homo sapiens
3294	11745509_ turquoise	IQ motif containing GTPase activating protein 2(IQGAP2)	Homo sapiens
3295	11745517_ blue	succinate dehydrogenase complex iron sulfur subunit B(SDHB)	Homo sapiens
3296	11745524_ turquoise	catenin beta interacting protein 1(CTNNBIP1)	Homo sapiens
3297	11745527_ blue	heat shock transcription factor 2(HSF2)	Homo sapiens
3298	11745537_ turquoise	nuclear factor of activated T-cells 3(NFATC3)	Homo sapiens

3299	11745555_ brown	chromosome 11 open reading frame 74(C11orf74)	Homo sapiens
3300	11745556_ brown	chromosome 11 open reading frame 74(C11orf74)	Homo sapiens
3301	11745560_ turquoise	dedicator of cytokinesis 8(DOCK8)	Homo sapiens
3302	11745602_ turquoise	clathrin light chain A(CLTA)	Homo sapiens
3303	11745648_ turquoise	eukaryotic translation elongation factor 1 alpha 1(EEF1A1)	Homo sapiens
3304	11745737_ turquoise	acyl-CoA dehydrogenase, very long chain(ACADVL)	Homo sapiens
3305	11745742_ turquoise	FYVE, RhoGEF and PH domain containing 3(FGD3)	Homo sapiens
3306	11745772_ turquoise	TNF receptor superfamily member 14(TNFRSF14)	Homo sapiens
3307	11745773_ turquoise	TNF receptor superfamily member 14(TNFRSF14)	Homo sapiens
3308	11745775_ turquoise	lipase A, lysosomal acid type(LIPA)	Homo sapiens
3309	11745801_ turquoise	adenylate cyclase associated protein 1(CAP1)	Homo sapiens
3310	11745812_ blue	isocitrate dehydrogenase 3 (NAD(+)) beta(IDH3B)	Homo sapiens
3311	11745833_ turquoise	epithelial cell transforming 2(ECT2)	Homo sapiens
3312	11745840_ turquoise	progestin and adipoQ receptor family member 8(PAQR8)	Homo sapiens
3313	11745841_ turquoise	CNDP dipeptidase 2 (metallopeptidase M20 family)(CNDP2)	Homo sapiens
3314	11745854_ turquoise	ring finger protein 145(RNF145)	Homo sapiens
3315	11745858_ blue	ATP5S like(ATP5SL)	Homo sapiens
3316	11745859_ blue	ATP5S like(ATP5SL)	Homo sapiens
3317	11745860_ turquoise	ATP5S like(ATP5SL)	Homo sapiens
3318	11745869_ blue	mitochondrial pyruvate carrier 2(MPC2)	Homo sapiens
3319	11745884_ turquoise	ubiquitin conjugating enzyme E2 Z(UBE2Z)	Homo sapiens
3320	11745892_ turquoise	methylenetetrahydrofolate dehydrogenase (NADP+ dependent) 2, methenyltetrahydro	Homo sapiens
3321	11745893_ turquoise	methylenetetrahydrofolate dehydrogenase (NADP+ dependent) 2, methenyltetrahydro	Homo sapiens
3322	11745894_ turquoise	TNF receptor superfamily member 14(TNFRSF14)	Homo sapiens
3323	11745902_ turquoise	NPC intracellular cholesterol transporter 2(NPC2)	Homo sapiens
3324	11745903_ turquoise	SLAM family member 7(SLAMF7)	Homo sapiens
3325	11745912_ turquoise	pellino E3 ubiquitin protein ligase 1(PELI1)	Homo sapiens
3326	11745922_ turquoise	monoamine oxidase A(MAOA)	Homo sapiens
3327	11745923_ turquoise	monoamine oxidase A(MAOA)	Homo sapiens
3328	11745926_ turquoise	ADAM metallopeptidase domain 15(ADAM15)	Homo sapiens
3329	11745927_ turquoise	ADAM metallopeptidase domain 15(ADAM15)	Homo sapiens
3330	11745930_ turquoise	p21 (RAC1) activated kinase 6(PAK6)	Homo sapiens
3331	11745932_ turquoise	thymosin beta 10(TMSB10)	Homo sapiens
3332	11745936_ turquoise	solute carrier family 50 member 1(SLC50A1)	Homo sapiens
3333	11745938_ turquoise	absent in melanoma 1(AIM1)	Homo sapiens
3334	11745988_ turquoise	ADP dependent glucokinase(ADPGK)	Homo sapiens
3335	11745989_ turquoise	major histocompatibility complex, class II, DM alpha(HLA-DMA)	Homo sapiens
3336	11745991_ turquoise	UV radiation resistance associated(UVRAG)	Homo sapiens
3337	11745998_ turquoise	zinc finger protein 252, pseudogene(ZNF252P)	Homo sapiens
3338	11746002_ turquoise	caspase 4(CASP4)	Homo sapiens
3339	11746003_ turquoise	DNA polymerase delta interacting protein 2(POLDIP2)	Homo sapiens
3340	11746014_ turquoise	neutrophil cytosolic factor 1(NCF1)	Homo sapiens
3341	11746027_ turquoise	N-myc and STAT interactor(NMI)	Homo sapiens
3342	11746028_ turquoise	N-myc and STAT interactor(NMI)	Homo sapiens
3343	11746041_ turquoise	transformer 2 beta homolog (Drosophila)(TRA2B)	Homo sapiens
3344	11746042_ turquoise	transformer 2 beta homolog (Drosophila)(TRA2B)	Homo sapiens
3345	11746048_ turquoise	adenosine deaminase like(ADAL)	Homo sapiens
3346	11746053_ turquoise	uncharacterized LOC100996740(LOC100996740)	Homo sapiens
3347	11746060_ turquoise	TNF alpha induced protein 2(TNFAIP2)	Homo sapiens
3348	11746079_ turquoise	adenylate cyclase 7(ADCY7)	Homo sapiens

3349	11746084_blue	protein-L-isoaspartate (D-aspartate) O-methyltransferase(PCMT1)	Homo sapiens
3350	11746087_turquoise	CD84 molecule(CD84)	Homo sapiens
3351	11746088_turquoise	interferon induced protein 44(IFI44)	Homo sapiens
3352	11746092_turquoise	rogdi homolog(ROGDI)	Homo sapiens
3353	11746135_turquoise	microRNA 1292(MIR1292)	Homo sapiens
3354	11746149_turquoise	butyrylcholinesterase(BCHE)	Homo sapiens
3355	11746155_turquoise	interferon regulatory factor 9(IRF9)	Homo sapiens
3356	11746156_turquoise	interferon regulatory factor 9(IRF9)	Homo sapiens
3357	11746159_turquoise	PBX homeobox 3(PBX3)	Homo sapiens
3358	11746162_turquoise	family with sequence similarity 49 member B(FAM49B)	Homo sapiens
3359	11746175_turquoise	apolipoprotein L4(APOL4)	Homo sapiens
3360	11746217_turquoise	HPS3, biogenesis of lysosomal organelles complex 2 subunit 1(HPS3)	Homo sapiens
3361	11746242_turquoise	aryl hydrocarbon receptor nuclear translocator like 2(ARNTL2)	Homo sapiens
3362	11746251_turquoise	chloride intracellular channel 1(CLIC1)	Homo sapiens
3363	11746269_turquoise	abhydrolase domain containing 3(ABHD3)	Homo sapiens
3364	11746273_turquoise	dihydrouridine synthase 4 like(DUS4L)	Homo sapiens
3365	11746286_turquoise	butyrophilin subfamily 3 member A2(BTN3A2)	Homo sapiens
3366	11746309_turquoise	solute carrier family 15 member 3(SLC15A3)	Homo sapiens
3367	11746321_turquoise	serpin family H member 1(SERPINH1)	Homo sapiens
3368	11746331_brown	adenosine kinase(ADK)	Homo sapiens
3369	11746340_turquoise	nucleolar protein 8(NOL8)	Homo sapiens
3370	11746347_turquoise	beta-1,3-glucuronyltransferase 3(B3GAT3)	Homo sapiens
3371	11746350_turquoise	Pim-1 proto-oncogene, serine/threonine kinase(PIM1)	Homo sapiens
3372	11746370_turquoise	ectonucleoside triphosphate diphosphohydrolase 6 (putative)(ENTPD6)	Homo sapiens
3373	11746372_brown	nudix hydrolase 6(NUDT6)	Homo sapiens
3374	11746376_turquoise	interleukin 16(IL16)	Homo sapiens
3375	11746400_turquoise	myosin light chain kinase 3(MYLK3)	Homo sapiens
3376	11746407_turquoise	toll like receptor 10(TLR10)	Homo sapiens
3377	11746414_turquoise	family with sequence similarity 107 member B(FAM107B)	Homo sapiens
3378	11746418_turquoise	vitrin(VIT)	Homo sapiens
3379	11746439_turquoise	glyoxalase I(GLO1)	Homo sapiens
3380	11746449_turquoise	Meis homeobox 2(MEIS2)	Homo sapiens
3381	11746450_turquoise	Meis homeobox 2(MEIS2)	Homo sapiens
3382	11746465_turquoise	epidermal growth factor receptor pathway substrate 15 like 1(EPS15L1)	Homo sapiens
3383	11746516_turquoise	ADAM metallopeptidase domain 15(ADAM15)	Homo sapiens
3384	11746524_turquoise	family with sequence similarity 81 member A(FAM81A)	Homo sapiens
3385	11746529_turquoise	TNF receptor superfamily member 14(TNFRSF14)	Homo sapiens
3386	11746557_turquoise	galectin 9B(LGALS9B)	Homo sapiens
3387	11746558_turquoise	galectin 9(LGALS9)	Homo sapiens
3388	11746568_turquoise	hypoxia inducible factor 3 alpha subunit(HIF3A)	Homo sapiens
3389	11746584_turquoise	metal response element binding transcription factor 2(MTF2)	Homo sapiens
3390	11746586_brown	NADH:ubiquinone oxidoreductase core subunit S7(NDUFS7)	Homo sapiens
3391	11746596_turquoise	NAD kinase(NADK)	Homo sapiens
3392	11746609_turquoise	cullin associated and neddylation dissociated 2 (putative)(CAND2)	Homo sapiens
3393	11746626_turquoise	hydroxyacylglutathione hydrolase(HAGH)	Homo sapiens
3394	11746631_turquoise	centrosomal protein 41(CEP41)	Homo sapiens
3395	11746635_turquoise	lymphoid enhancer binding factor 1(LEF1)	Homo sapiens
3396	11746638_turquoise	dehydrogenase/reductase 11(DHRS11)	Homo sapiens
3397	11746639_turquoise	Ras association domain family member 2(RASSF2)	Homo sapiens
3398	11746655_blue	acetyl-CoA acyltransferase 1(ACAA1)	Homo sapiens

3399	11746658_turquoise	CD86 molecule(CD86)	Homo sapiens
3400	11746659_turquoise	nuclear receptor subfamily 2 group F member 6(NR2F6)	Homo sapiens
3401	11746686_turquoise	G kinase anchoring protein 1(GKAP1)	Homo sapiens
3402	11746692_turquoise	bromodomain adjacent to zinc finger domain 1A(BAZ1A)	Homo sapiens
3403	11746705_turquoise	regulator of G-protein signaling 16(RGS16)	Homo sapiens
3404	11746710_turquoise	dedicator of cytokinesis 10(DOCK10)	Homo sapiens
3405	11746729_turquoise	lymphoid restricted membrane protein(LRMP)	Homo sapiens
3406	11746736_turquoise	calcium voltage-gated channel auxiliary subunit alpha2delta 3(CACNA2D3)	Homo sapiens
3407	11746767_turquoise	integrin subunit alpha 4(ITGA4)	Homo sapiens
3408	11746778_turquoise	intersectin 1(ITSN1)	Homo sapiens
3409	11746779_turquoise	nuclear receptor coactivator 7(NCOA7)	Homo sapiens
3410	11746804_turquoise	major histocompatibility complex, class II, DQ beta 1(HLA-DQB1)	Homo sapiens
3411	11746844_blue	transmembrane protein 143(TMEM143)	Homo sapiens
3412	11746845_blue	transmembrane protein 143(TMEM143)	Homo sapiens
3413	11746855_brown	NAD(P)HX epimerase(NAXE)	Homo sapiens
3414	11746868_turquoise	GIT ArfGAP 2(GIT2)	Homo sapiens
3415	11746874_turquoise	G protein subunit alpha i2(GNAI2)	Homo sapiens
3416	11746875_turquoise	G protein subunit alpha i2(GNAI2)	Homo sapiens
3417	11746878_turquoise	inhibitor of DNA binding 2, HLH protein(ID2)	Homo sapiens
3418	11746880_turquoise	filamin A(FLNA)	Homo sapiens
3419	11746906_turquoise	caspase recruitment domain family member 8(CARD8)	Homo sapiens
3420	11746918_blue	L-2-hydroxyglutarate dehydrogenase(L2HGDH)	Homo sapiens
3421	11746935_turquoise	tryptophanyl tRNA synthetase 2, mitochondrial(WARS2)	Homo sapiens
3422	11746954_turquoise	C-C motif chemokine ligand 4 like 1(CCL4L1)	Homo sapiens
3423	11746961_turquoise	major histocompatibility complex, class II, DM beta(HLA-DMB)	Homo sapiens
3424	11746962_turquoise	major histocompatibility complex, class II, DM beta(HLA-DMB)	Homo sapiens
3425	11746965_turquoise	alanyl aminopeptidase, membrane(ANPEP)	Homo sapiens
3426	11746972_turquoise	membrane associated ring-CH-type finger 1(MARCH1)	Homo sapiens
3427	11746995_turquoise	N-acetylneuraminate pyruvate lyase(NPL)	Homo sapiens
3428	11747004_turquoise	sulfite oxidase(SUOX)	Homo sapiens
3429	11747013_blue	pyrophosphatase (inorganic) 2(PPA2)	Homo sapiens
3430	11747019_turquoise	Rho GTPase activating protein 45(ARHGAP45)	Homo sapiens
3431	11747020_turquoise	Rho GTPase activating protein 45(ARHGAP45)	Homo sapiens
3432	11747076_turquoise	microRNA 6837(MIR6837)	Homo sapiens
3433	11747077_turquoise	eukaryotic translation elongation factor 1 alpha 1(EEF1A1)	Homo sapiens
3434	11747086_turquoise	grancalcin(GCA)	Homo sapiens
3435	11747110_turquoise	PAS domain containing serine/threonine kinase(PASK)	Homo sapiens
3436	11747113_turquoise	CD160 molecule(CD160)	Homo sapiens
3437	11747126_blue	ADP-ribosyltransferase 3(ART3)	Homo sapiens
3438	11747135_turquoise	centriolin(CNTRL)	Homo sapiens
3439	11747137_turquoise	endoplasmic reticulum aminopeptidase 2(ERAP2)	Homo sapiens
3440	11747147_turquoise	protein phosphatase 2 scaffold subunit Abeta(PPP2R1B)	Homo sapiens
3441	11747189_turquoise	B-cell CLL/lymphoma 11A(BCL11A)	Homo sapiens
3442	11747190_turquoise	FCH and double SH3 domains 1(FCHSD1)	Homo sapiens
3443	11747212_turquoise	zinc finger protein 174(ZNF174)	Homo sapiens
3444	11747230_turquoise	BUB1 mitotic checkpoint serine/threonine kinase(BUB1)	Homo sapiens
3445	11747247_turquoise	RAP1B, member of RAS oncogene family(RAP1B)	Homo sapiens
3446	11747249_turquoise	adenylosuccinate synthase(ADSS)	Homo sapiens
3447	11747276_turquoise	A-kinase anchoring protein 1(AKAP1)	Homo sapiens
3448	11747278_brown	malate dehydrogenase 2(MDH2)	Homo sapiens

3449	11747282_turquoise	annexin A2 pseudogene 2(ANXA2P2)	Homo sapiens
3450	11747285_turquoise	target of myb1 like 1 membrane trafficking protein(TOM1L1)	Homo sapiens
3451	11747295_turquoise	perforin 1(PRF1)	Homo sapiens
3452	11747301_turquoise	SP100 nuclear antigen(SP100)	Homo sapiens
3453	11747313_blue	OXA1L, mitochondrial inner membrane protein(OXA1L)	Homo sapiens
3454	11747315_blue	ectonucleoside triphosphate diphosphohydrolase 6 (putative)(ENTPD6)	Homo sapiens
3455	11747345_blue	acyl-CoA dehydrogenase, C-4 to C-12 straight chain(ACADM)	Homo sapiens
3456	11747346_blue	acyl-CoA dehydrogenase, C-4 to C-12 straight chain(ACADM)	Homo sapiens
3457	11747381_turquoise	septin 1(SEPT1)	Homo sapiens
3458	11747401_blue	COP9 signalosome subunit 7A(COPS7A)	Homo sapiens
3459	11747408_turquoise	filamin binding LIM protein 1(FBLIM1)	Homo sapiens
3460	11747418_turquoise	napsin B aspartic peptidase, pseudogene(NAPSB)	Homo sapiens
3461	11747437_turquoise	caspase recruitment domain family member 8(CARD8)	Homo sapiens
3462	11747438_turquoise	GTPase, IMAP family member 4(GIMAP4)	Homo sapiens
3463	11747439_turquoise	myosin light chain 6(MYL6)	Homo sapiens
3464	11747446_turquoise	major histocompatibility complex, class II, DO beta(HLA-DOB)	Homo sapiens
3465	11747448_turquoise	butyrophilin subfamily 3 member A2(BTN3A2)	Homo sapiens
3466	11747449_turquoise	D-2-hydroxyglutarate dehydrogenase(D2HGDH)	Homo sapiens
3467	11747453_turquoise	protein phosphatase 1 regulatory subunit 18(PPP1R18)	Homo sapiens
3468	11747460_turquoise	transgelin 2(TAGLN2)	Homo sapiens
3469	11747467_turquoise	protein kinase C theta(PRKCQ)	Homo sapiens
3470	11747483_turquoise	TNF alpha induced protein 8(TNFAIP8)	Homo sapiens
3471	11747499_turquoise	intercellular adhesion molecule 1(ICAM1)	Homo sapiens
3472	11747502_turquoise	mitochondrial translational release factor 1 like(MTRF1L)	Homo sapiens
3473	11747508_turquoise	erythrocyte membrane protein band 4.1 like 3(EPB41L3)	Homo sapiens
3474	11747509_turquoise	family with sequence similarity 107 member B(FAM107B)	Homo sapiens
3475	11747523_turquoise	TNF receptor associated factor 4(TRAF4)	Homo sapiens
3476	11747533_blue	G-rich RNA sequence binding factor 1(GRSF1)	Homo sapiens
3477	11747538_turquoise	myeloid differentiation primary response 88(MYD88)	Homo sapiens
3478	11747539_turquoise	myeloid differentiation primary response 88(MYD88)	Homo sapiens
3479	11747546_blue	phosphatidylinositol-4-phosphate 5-kinase type 1 beta(PIP5K1B)	Homo sapiens
3480	11747559_blue	PDZ domain containing ring finger 3(PDZRN3)	Homo sapiens
3481	11747589_blue	glutamic-oxaloacetic transaminase 2(GOT2)	Homo sapiens
3482	11747590_turquoise	Fc fragment of IgM receptor(FCMR)	Homo sapiens
3483	11747591_turquoise	GLI pathogenesis related 1(GLIPR1)	Homo sapiens
3484	11747593_turquoise	SH3 domain binding glutamate rich protein like(SH3BGRL)	Homo sapiens
3485	11747594_turquoise	SH3 domain binding glutamate rich protein like(SH3BGRL)	Homo sapiens
3486	11747597_turquoise	C-type lectin domain family 2 member D(CLEC2D)	Homo sapiens
3487	11747602_turquoise	mitogen-activated protein kinase kinase kinase 8(MAP3K8)	Homo sapiens
3488	11747607_turquoise	purinergic receptor P2X 7(P2RX7)	Homo sapiens
3489	11747623_turquoise	Rho GTPase activating protein 25(ARHGAP25)	Homo sapiens
3490	11747627_turquoise	tRNA-yW synthesizing protein 1 homolog B(TYW1B)	Homo sapiens
3491	11747630_turquoise	XIAP associated factor 1(XAF1)	Homo sapiens
3492	11747631_turquoise	XIAP associated factor 1(XAF1)	Homo sapiens
3493	11747643_turquoise	BUB3, mitotic checkpoint protein(BUB3)	Homo sapiens
3494	11747652_turquoise	cell division cycle 25B(CDC25B)	Homo sapiens
3495	11747653_turquoise	cell division cycle 25B(CDC25B)	Homo sapiens
3496	11747666_turquoise	tryptophanyl-tRNA synthetase(WARS)	Homo sapiens
3497	11747677_blue	basic leucine zipper and W2 domains 2(BZW2)	Homo sapiens
3498	11747680_turquoise	thyroid hormone receptor, alpha(THRA)	Homo sapiens

3499	11747681_ turquoise	thyroid hormone receptor, alpha(THRA)	Homo sapiens
3500	11747714_ turquoise	ATP binding cassette subfamily A member 8(ABCA8)	Homo sapiens
3501	11747723_ turquoise	death domain associated protein(DAXX)	Homo sapiens
3502	11747726_ turquoise	syntaxin binding protein 6(STXBP6)	Homo sapiens
3503	11747731_ turquoise	DBF4 zinc finger(DBF4)	Homo sapiens
3504	11747742_ turquoise	metal response element binding transcription factor 2(MTF2)	Homo sapiens
3505	11747743_ turquoise	metal response element binding transcription factor 2(MTF2)	Homo sapiens
3506	11747772_ blue	pyruvate dehydrogenase (lipoamide) alpha 1(PDHA1)	Homo sapiens
3507	11747785_ turquoise	lymphocyte cytosolic protein 1(LCP1)	Homo sapiens
3508	11747799_ turquoise	enolase 1(ENO1)	Homo sapiens
3509	11747803_ turquoise	CDC like kinase 2(CLK2)	Homo sapiens
3510	11747811_ turquoise	GRINL1A complex locus 1(GCOM1)	Homo sapiens
3511	11747820_ turquoise	uridine phosphorylase 1(UPP1)	Homo sapiens
3512	11747895_ turquoise	tectonic family member 1(TCTN1)	Homo sapiens
3513	11747902_ blue	echinoderm microtubule associated protein like 1(EML1)	Homo sapiens
3514	11747926_ turquoise	interferon gamma inducible protein 16(IFI16)	Homo sapiens
3515	11747935_ turquoise	tenascin C(TNC)	Homo sapiens
3516	11747948_ turquoise	small ArfGAP2(SMAP2)	Homo sapiens
3517	11747952_ turquoise	tumor necrosis factor superfamily member 10(TNFSF10)	Homo sapiens
3518	11747961_ turquoise	proteasome subunit beta 8(PSMB8)	Homo sapiens
3519	11747981_ turquoise	APC down-regulated 1(APCDD1)	Homo sapiens
3520	11748003_ turquoise	interferon induced protein 44(IFI44)	Homo sapiens
3521	11748014_ blue	voltage dependent anion channel 3(VDAC3)	Homo sapiens
3522	11748015_ blue	voltage dependent anion channel 3(VDAC3)	Homo sapiens
3523	11748034_ turquoise	cytidine monophospho-N-acetylneuraminic acid hydroxylase, pseudogene(CMAHP)	Homo sapiens
3524	11748088_ turquoise	A-kinase anchoring protein 1(AKAP1)	Homo sapiens
3525	11748097_ turquoise	poly(A) binding protein cytoplasmic 1(PABPC1)	Homo sapiens
3526	11748099_ turquoise	phosphatase and actin regulator 3(PHACTR3)	Homo sapiens
3527	11748120_ turquoise	lymphocyte cytosolic protein 2(LCP2)	Homo sapiens
3528	11748148_ blue	coenzyme Q6, monooxygenase(COQ6)	Homo sapiens
3529	11748149_ turquoise	formin binding protein 1(FNBP1)	Homo sapiens
3530	11748188_ turquoise	alpha-N-acetylgalactosaminidase(NAGA)	Homo sapiens
3531	11748216_ blue	staufen double-stranded RNA binding protein 2(STAU2)	Homo sapiens
3532	11748229_ turquoise	interleukin 15 receptor subunit alpha(IL15RA)	Homo sapiens
3533	11748230_ turquoise	coronin 1A(CORO1A)	Homo sapiens
3534	11748249_ turquoise	NDRG family member 3(NDRG3)	Homo sapiens
3535	11748250_ turquoise	NDRG family member 3(NDRG3)	Homo sapiens
3536	11748253_ blue	solute carrier family 5 member 1(SLC5A1)	Homo sapiens
3537	11748254_ turquoise	male-specific lethal 3 homolog (Drosophila)(MSL3)	Homo sapiens
3538	11748273_ blue	Raf-1 proto-oncogene, serine/threonine kinase(RAF1)	Homo sapiens
3539	11748280_ turquoise	serpin family B member 1(SERPINB1)	Homo sapiens
3540	11748281_ turquoise	cyclin and CBS domain divalent metal cation transport mediator 4(CNNM4)	Homo sapiens
3541	11748301_ turquoise	transcription factor Dp-2(TFDP2)	Homo sapiens
3542	11748304_ turquoise	ectonucleoside triphosphate diphosphohydrolase 6 (putative)(ENTPD6)	Homo sapiens
3543	11748325_ blue	mitochondrial ribosomal protein S22(MRPS22)	Homo sapiens
3544	11748332_ turquoise	major histocompatibility complex, class I, E(HLA-E)	Homo sapiens
3545	11748339_ turquoise	leukocyte associated immunoglobulin like receptor 1(LAIR1)	Homo sapiens
3546	11748340_ turquoise	leukocyte associated immunoglobulin like receptor 1(LAIR1)	Homo sapiens
3547	11748343_ brown	intraflagellar transport 22(IFT22)	Homo sapiens
3548	11748344_ brown	intraflagellar transport 22(IFT22)	Homo sapiens

3549	11748359_turquoise	BCR, RhoGEF and GTPase activating protein(BCR)	Homo sapiens
3550	11748362_turquoise	ATP binding cassette subfamily C member 3(ABCC3)	Homo sapiens
3551	11748400_turquoise	tropomyosin 4(TPM4)	Homo sapiens
3552	11748401_turquoise	tropomyosin 4(TPM4)	Homo sapiens
3553	11748423_blue	phosphoglucomutase 1(PGM1)	Homo sapiens
3554	11748483_turquoise	purinergic receptor P2X 7(P2RX7)	Homo sapiens
3555	11748492_turquoise	layilin(LAYN)	Homo sapiens
3556	11748497_turquoise	grancalcin(GCA)	Homo sapiens
3557	11748509_blue	voltage dependent anion channel 2(VDAC2)	Homo sapiens
3558	11748529_turquoise	caspase 4(CASP4)	Homo sapiens
3559	11748531_turquoise	calcium channel flower domain containing 1(CACFD1)	Homo sapiens
3560	11748541_blue	protease associated domain containing 1(PRADC1)	Homo sapiens
3561	11748548_turquoise	purinergic receptor P2X 4(P2RX4)	Homo sapiens
3562	11748553_blue	ubiquinol-cytochrome c reductase complex assembly factor 1(UQCC1)	Homo sapiens
3563	11748555_turquoise	cyclin dependent kinase like 3(CDKL3)	Homo sapiens
3564	11748582_turquoise	ADP-ribosyltransferase 3(ART3)	Homo sapiens
3565	11748583_blue	ADP-ribosyltransferase 3(ART3)	Homo sapiens
3566	11748604_turquoise	death associated protein kinase 2(DAPK2)	Homo sapiens
3567	11748620_blue	succinate-CoA ligase ADP-forming beta subunit(SUCLA2)	Homo sapiens
3568	11748650_turquoise	ADAM metallopeptidase domain 33(ADAM33)	Homo sapiens
3569	11748654_turquoise	protein tyrosine phosphatase, non-receptor type 22(PTPN22)	Homo sapiens
3570	11748671_blue	cullin 4A(CUL4A)	Homo sapiens
3571	11748695_brown	synaptogyrin 1(SYNGR1)	Homo sapiens
3572	11748702_turquoise	annexin A4(ANXA4)	Homo sapiens
3573	11748713_turquoise	abnormal spindle microtubule assembly(ASPM)	Homo sapiens
3574	11748747_turquoise	Rho GTPase activating protein 25(ARHGAP25)	Homo sapiens
3575	11748755_turquoise	Meis homeobox 2(MEIS2)	Homo sapiens
3576	11748775_turquoise	uridine phosphorylase 1(UPP1)	Homo sapiens
3577	11748780_turquoise	target of myb1 like 1 membrane trafficking protein(TOM1L1)	Homo sapiens
3578	11748786_turquoise	thymocyte expressed, positive selection associated 1(TESPA1)	Homo sapiens
3579	11748808_turquoise	AT-rich interaction domain 3B(ARID3B)	Homo sapiens
3580	11748839_blue	NFS1, cysteine desulfurase(NFS1)	Homo sapiens
3581	11748841_turquoise	SAM and HD domain containing deoxynucleoside triphosphate triphosphohydrolase 1(S	Homo sapiens
3582	11748845_turquoise	SLAM family member 7(SLAMF7)	Homo sapiens
3583	11748854_blue	RNA pseudouridylate synthase domain containing 4(RPUSD4)	Homo sapiens
3584	11748859_turquoise	transcription factor EC(TFEC)	Homo sapiens
3585	11748860_blue	apolipoprotein B mRNA editing enzyme catalytic subunit 2(APOBEC2)	Homo sapiens
3586	11748874_blue	mitochondrial ribosomal protein S25(MRPS25)	Homo sapiens
3587	11748897_turquoise	putative aquaporin-7-like protein 3(LOC100509620)	Homo sapiens
3588	11748905_turquoise	collagen triple helix repeat containing 1(CTHRC1)	Homo sapiens
3589	11748907_turquoise	retinoic acid receptor responder 3(RARRES3)	Homo sapiens
3590	11748915_turquoise	glutamic--pyruvic transaminase(GPT)	Homo sapiens
3591	11748972_turquoise	cell division cycle 25B(CDC25B)	Homo sapiens
3592	11748973_turquoise	cell division cycle 25B(CDC25B)	Homo sapiens
3593	11749002_turquoise	Rho GTPase activating protein 45(ARHGAP45)	Homo sapiens
3594	11749003_turquoise	endoplasmic reticulum aminopeptidase 1(ERAP1)	Homo sapiens
3595	11749016_turquoise	uncharacterized LOC100130460(CAND1.11)	Homo sapiens
3596	11749026_turquoise	protein tyrosine phosphatase, receptor type A(PTPRA)	Homo sapiens
3597	11749040_turquoise	phosphoglucomutase 2(PGM2)	Homo sapiens
3598	11749059_turquoise	reticulon 4(RTN4)	Homo sapiens

3599	11749092_	turquoise	NLR family member X1(NLRX1)	Homo sapiens
3600	11749094_	turquoise	dermatan sulfate epimerase(DSE)	Homo sapiens
3601	11749097_	turquoise	ATP binding cassette subfamily A member 8(ABCA8)	Homo sapiens
3602	11749104_	turquoise	Rho GTPase activating protein 45(ARHGAP45)	Homo sapiens
3603	11749121_	turquoise	interferon alpha and beta receptor subunit 2(IFNAR2)	Homo sapiens
3604	11749132_	turquoise	solute carrier organic anion transporter family member 2B1(SLCO2B1)	Homo sapiens
3605	11749141_	turquoise	TNF receptor associated factor 5(TRAF5)	Homo sapiens
3606	11749144_	blue	electron transfer flavoprotein dehydrogenase(ETFDH)	Homo sapiens
3607	11749164_	turquoise	transmembrane protein 206(TMEM206)	Homo sapiens
3608	11749171_	turquoise	beta-ureidopropionase 1(UPB1)	Homo sapiens
3609	11749188_	turquoise	ectonucleoside triphosphate diphosphohydrolase 1(ENTPD1)	Homo sapiens
3610	11749201_	blue	IQCJ-SCHIP1 readthrough(IQCJ-SCHIP1)	Homo sapiens
3611	11749214_	turquoise	selectin P ligand(SELPLG)	Homo sapiens
3612	11749225_	turquoise	butyrophilin subfamily 2 member A2(BTN2A2)	Homo sapiens
3613	11749229_	brown	sirtuin 5(SIRT5)	Homo sapiens
3614	11749245_	turquoise	C-X-C motif chemokine ligand 11(CXCL11)	Homo sapiens
3615	11749261_	blue	tubulin folding cofactor E(TBCE)	Homo sapiens
3616	11749265_	turquoise	putative aquaporin-7-like protein 3(LOC100509620)	Homo sapiens
3617	11749275_	turquoise	src kinase associated phosphoprotein 2(SKAP2)	Homo sapiens
3618	11749293_	turquoise	membrane spanning 4-domains A6A(MS4A6A)	Homo sapiens
3619	11749309_	turquoise	prune exopolyphosphatase(PRUNE1)	Homo sapiens
3620	11749310_	turquoise	prune exopolyphosphatase(PRUNE1)	Homo sapiens
3621	11749311_	blue	GrpE like 1, mitochondrial(GRPEL1)	Homo sapiens
3622	11749326_	turquoise	olfactomedin like 2B(OLFML2B)	Homo sapiens
3623	11749329_	turquoise	Rho GTPase activating protein 9(ARHGAP9)	Homo sapiens
3624	11749367_	turquoise	FXYD domain containing ion transport regulator 5(FXYD5)	Homo sapiens
3625	11749369_	blue	VAMP associated protein B and C(VAPB)	Homo sapiens
3626	11749370_	turquoise	XIAP associated factor 1(XAF1)	Homo sapiens
3627	11749377_	turquoise	cat eye syndrome chromosome region, candidate 1(CECR1)	Homo sapiens
3628	11749391_	turquoise	quinolinate phosphoribosyltransferase(QPRT)	Homo sapiens
3629	11749392_	blue	ATP synthase, H+ transporting, mitochondrial F1 complex, gamma polypeptide 1(ATP5C	Homo sapiens
3630	11749398_	brown	B9 domain containing 1(B9D1)	Homo sapiens
3631	11749399_	brown	MAPK regulated corepressor interacting protein 2(MCRIP2)	Homo sapiens
3632	11749402_	turquoise	integrin subunit alpha L(ITGAL)	Homo sapiens
3633	11749412_	turquoise	poly(ADP-ribose) polymerase family member 14(PARP14)	Homo sapiens
3634	11749416_	turquoise	small nucleolar RNA host gene 4(SNHG4)	Homo sapiens
3635	11749430_	brown	NADH:ubiquinone oxidoreductase complex assembly factor 6(NDUFAF6)	Homo sapiens
3636	11749445_	turquoise	uncharacterized LOC101928361(LOC101928361)	Homo sapiens
3637	11749492_	turquoise	ribosomal protein S6 kinase A1(RPS6KA1)	Homo sapiens
3638	11749497_	turquoise	DExH-box helicase 58(DHX58)	Homo sapiens
3639	11749510_	turquoise	SAM and HD domain containing deoxynucleoside triphosphate triphosphohydrolase 1(S	Homo sapiens
3640	11749512_	turquoise	signal transducer and activator of transcription 4(STAT4)	Homo sapiens
3641	11749522_	turquoise	lymphocyte activating 3(LAG3)	Homo sapiens
3642	11749541_	turquoise	sushi domain containing 4(SUSD4)	Homo sapiens
3643	11749550_	turquoise	serine carboxypeptidase 1(SCPEP1)	Homo sapiens
3644	11749573_	turquoise	arrestin beta 2(ARRB2)	Homo sapiens
3645	11749578_	turquoise	COX10, heme A:farnesyltransferase cytochrome c oxidase assembly factor(COX10)	Homo sapiens
3646	11749587_	turquoise	Fc fragment of IgG receptor IIa(FCGR2A)	Homo sapiens
3647	11749589_	turquoise	cathepsin S(CTSS)	Homo sapiens
3648	11749595_	turquoise	guanylate binding protein 4(GBP4)	Homo sapiens

3649	11749613_turquoise	OCIA domain containing 1(OCIAD1)	Homo sapiens
3650	11749625_turquoise	PQ loop repeat containing 3(PQLC3)	Homo sapiens
3651	11749660_turquoise	Mov10 RISC complex RNA helicase(MOV10)	Homo sapiens
3652	11749674_turquoise	shootin 1(SHTN1)	Homo sapiens
3653	11749676_turquoise	B-cell CLL/lymphoma 11A(BCL11A)	Homo sapiens
3654	11749690_blue	LARGE xylosyl- and glucuronyltransferase 1(LARGE1)	Homo sapiens
3655	11749697_blue	NADH:ubiquinone oxidoreductase core subunit S1(NDUFS1)	Homo sapiens
3656	11749708_turquoise	Fli-1 proto-oncogene, ETS transcription factor(FLI1)	Homo sapiens
3657	11749722_blue	NADH:ubiquinone oxidoreductase core subunit S1(NDUFS1)	Homo sapiens
3658	11749740_turquoise	ubiquitin specific peptidase 3(USP3)	Homo sapiens
3659	11749750_turquoise	WAS/WASL interacting protein family member 1(WIPF1)	Homo sapiens
3660	11749751_turquoise	WAS/WASL interacting protein family member 1(WIPF1)	Homo sapiens
3661	11749752_turquoise	WAS/WASL interacting protein family member 1(WIPF1)	Homo sapiens
3662	11749761_turquoise	PC-esterase domain containing 1A(PCED1A)	Homo sapiens
3663	11749773_turquoise	guanylate binding protein 1(GBP1)	Homo sapiens
3664	11749786_turquoise	heterogeneous nuclear ribonucleoprotein F(HNRNPF)	Homo sapiens
3665	11749788_turquoise	L-2-hydroxyglutarate dehydrogenase(L2HGDH)	Homo sapiens
3666	11749790_turquoise	caspase 8(CASP8)	Homo sapiens
3667	11749792_turquoise	PBX homeobox 1(PBX1)	Homo sapiens
3668	11749793_turquoise	annexin A4(ANXA4)	Homo sapiens
3669	11749794_turquoise	annexin A4(ANXA4)	Homo sapiens
3670	11749827_turquoise	cytochrome b-245 beta chain(CYBB)	Homo sapiens
3671	11749833_turquoise	angiotensinogen(AGT)	Homo sapiens
3672	11749844_turquoise	colony stimulating factor 2 receptor alpha subunit(CSF2RA)	Homo sapiens
3673	11749862_turquoise	apolipoprotein L3(APOL3)	Homo sapiens
3674	11749879_turquoise	post-GPI attachment to proteins 2(PGAP2)	Homo sapiens
3675	11749880_turquoise	post-GPI attachment to proteins 2(PGAP2)	Homo sapiens
3676	11749893_turquoise	carbonic anhydrase 14(CA14)	Homo sapiens
3677	11749894_turquoise	deoxycytidine kinase(DCK)	Homo sapiens
3678	11749914_blue	single-pass membrane protein with coiled-coil domains 1(SMCO1)	Homo sapiens
3679	11749917_turquoise	hes related family bHLH transcription factor with YRPW motif 2(HEY2)	Homo sapiens
3680	11749924_turquoise	mitochondrial ribosomal protein S25(MRPS25)	Homo sapiens
3681	11749935_turquoise	tropomyosin 3(TPM3)	Homo sapiens
3682	11749978_turquoise	major vault protein(MVP)	Homo sapiens
3683	11749990_turquoise	major histocompatibility complex, class I, A(HLA-A)	Homo sapiens
3684	11750000_turquoise	C-type lectin domain family 7 member A(CLEC7A)	Homo sapiens
3685	11750001_turquoise	C-type lectin domain family 7 member A(CLEC7A)	Homo sapiens
3686	11750006_brown	cytochrome c oxidase assembly factor 1 homolog(COA1)	Homo sapiens
3687	11750038_blue	mitochondrial trans-2-enoyl-CoA reductase(MECR)	Homo sapiens
3688	11750044_blue	NFS1, cysteine desulfurase(NFS1)	Homo sapiens
3689	11750045_turquoise	tropomyosin 3(TPM3)	Homo sapiens
3690	11750059_blue	MLX, MAX dimerization protein(MLX)	Homo sapiens
3691	11750080_turquoise	damage specific DNA binding protein 2(DDB2)	Homo sapiens
3692	11750103_blue	hydroxyacyl-CoA dehydrogenase(HADH)	Homo sapiens
3693	11750107_turquoise	SEL1L family member 3(SEL1L3)	Homo sapiens
3694	11750111_turquoise	vinculin(VCL)	Homo sapiens
3695	11750135_blue	monoamine oxidase B(MAOB)	Homo sapiens
3696	11750143_turquoise	phosphatidylinositol glycan anchor biosynthesis class Z(PIGZ)	Homo sapiens
3697	11750146_turquoise	transketolase like 1(TKTL1)	Homo sapiens
3698	11750159_turquoise	basic leucine zipper ATF-like transcription factor 2(BATF2)	Homo sapiens

3699	11750170_ turquoise	tripartite motif containing 22(TRIM22)	Homo sapiens
3700	11750171_ turquoise	transducin like enhancer of split 2(TLE2)	Homo sapiens
3701	11750178_ turquoise	enoyl-CoA hydratase domain containing 2(ECHDC2)	Homo sapiens
3702	11750179_ turquoise	enoyl-CoA hydratase domain containing 2(ECHDC2)	Homo sapiens
3703	11750185_ turquoise	coiled-coil domain containing 71-like(CCDC71L)	Homo sapiens
3704	11750189_ turquoise	CD44 molecule (Indian blood group)(CD44)	Homo sapiens
3705	11750190_ turquoise	CD44 molecule (Indian blood group)(CD44)	Homo sapiens
3706	11750196_ turquoise	annexin A4(ANXA4)	Homo sapiens
3707	11750198_ turquoise	caspase 7(CASP7)	Homo sapiens
3708	11750199_ turquoise	hematopoietic cell-specific Lyn substrate 1(HCLS1)	Homo sapiens
3709	11750223_ brown	NAD(P)HX epimerase(NAXE)	Homo sapiens
3710	11750231_ yellow	microRNA 8071-1(MIR8071-1)	Homo sapiens
3711	11750250_ turquoise	protein phosphatase 1 regulatory subunit 13 like(PPP1R13L)	Homo sapiens
3712	11750267_ turquoise	inositol polyphosphate-5-phosphatase J(INPP5J)	Homo sapiens
3713	11750282_ turquoise	microRNA 6837(MIR6837)	Homo sapiens
3714	11750284_ turquoise	lines homolog 1(LINS1)	Homo sapiens
3715	11750293_ brown	RAD51 paralog C(RAD51C)	Homo sapiens
3716	11750301_ turquoise	family with sequence similarity 92 member A(FAM92A)	Homo sapiens
3717	11750334_ turquoise	REL proto-oncogene, NF-kB subunit(REL)	Homo sapiens
3718	11750359_ turquoise	small nucleolar RNA, H/ACA box 67(SNORA67)	Homo sapiens
3719	11750374_ turquoise	Rap guanine nucleotide exchange factor 6(RAPGEF6)	Homo sapiens
3720	11750376_ turquoise	potassium calcium-activated channel subfamily M alpha 1(KCNMA1)	Homo sapiens
3721	11750408_ turquoise	adhesion G protein-coupled receptor E5(ADGRE5)	Homo sapiens
3722	11750453_ turquoise	parkin RBR E3 ubiquitin protein ligase(PARK2)	Homo sapiens
3723	11750454_ turquoise	ETS variant 7(ETV7)	Homo sapiens
3724	11750455_ turquoise	ETS variant 7(ETV7)	Homo sapiens
3725	11750527_ turquoise	major histocompatibility complex, class II, DQ alpha 1(HLA-DQA1)	Homo sapiens
3726	11750528_ turquoise	major histocompatibility complex, class II, DQ alpha 1(HLA-DQA1)	Homo sapiens
3727	11750531_ turquoise	PYD and CARD domain containing(PYCARD)	Homo sapiens
3728	11750551_ turquoise	serine/threonine kinase 4(STK4)	Homo sapiens
3729	11750552_ turquoise	caspase recruitment domain family member 16(CARD16)	Homo sapiens
3730	11750553_ turquoise	caspase 1(CASP1)	Homo sapiens
3731	11750575_ turquoise	zinc finger protein, FOG family member 2(ZFPM2)	Homo sapiens
3732	11750585_ turquoise	septin 9(SEPT9)	Homo sapiens
3733	11750594_ blue	phospholipase C like 1(PLCL1)	Homo sapiens
3734	11750598_ turquoise	TPX2, microtubule nucleation factor(TPX2)	Homo sapiens
3735	11750629_ turquoise	C1q and tumor necrosis factor related protein 8(C1QTNF8)	Homo sapiens
3736	11750636_ turquoise	acetyl-CoA carboxylase beta(ACACB)	Homo sapiens
3737	11750674_ turquoise	signal transducer and activator of transcription 3(STAT3)	Homo sapiens
3738	11750677_ turquoise	family with sequence similarity 65 member C(FAM65C)	Homo sapiens
3739	11750687_ turquoise	ADAM metallopeptidase domain 28(ADAM28)	Homo sapiens
3740	11750704_ turquoise	sex comb on midleg homolog 1 (Drosophila)(SCMH1)	Homo sapiens
3741	11750723_ turquoise	MHC class I polypeptide-related sequence B(MICB)	Homo sapiens
3742	11750740_ turquoise	ST3 beta-galactoside alpha-2,3-sialyltransferase 5(ST3GAL5)	Homo sapiens
3743	11750766_ turquoise	transforming acidic coiled-coil containing protein 2(TACC2)	Homo sapiens
3744	11750769_ turquoise	C-C motif chemokine receptor 2(CCR2)	Homo sapiens
3745	11750773_ turquoise	kinesin family member 2A(KIF2A)	Homo sapiens
3746	11750778_ turquoise	macrophage scavenger receptor 1(MSR1)	Homo sapiens
3747	11750800_ turquoise	ligand of numb-protein X 1(LNX1)	Homo sapiens
3748	11750811_ turquoise	eomesodermin(EOMES)	Homo sapiens

3749	11750812_turquoise	Fli-1 proto-oncogene, ETS transcription factor(FLI1)	Homo sapiens
3750	11750815_turquoise	microRNA 3064(MIR3064)	Homo sapiens
3751	11750816_turquoise	CD96 molecule(CD96)	Homo sapiens
3752	11750821_turquoise	mirror-image polydactyly 1(MIPOL1)	Homo sapiens
3753	11750826_turquoise	ADAM like decysin 1(ADAMDEC1)	Homo sapiens
3754	11750847_turquoise	ankyrin repeat domain 44(ANKRD44)	Homo sapiens
3755	11750856_turquoise	C-C motif chemokine receptor 2(CCR2)	Homo sapiens
3756	11750860_turquoise	cytidine monophospho-N-acetylneuraminic acid hydroxylase, pseudogene(CMAHP)	Homo sapiens
3757	11750861_turquoise	cytidine monophospho-N-acetylneuraminic acid hydroxylase, pseudogene(CMAHP)	Homo sapiens
3758	11750894_turquoise	T-box 5(TBX5)	Homo sapiens
3759	11750914_blue	G1 to S phase transition 2(GSPT2)	Homo sapiens
3760	11750932_turquoise	epoxide hydrolase 2(EPHX2)	Homo sapiens
3761	11750984_turquoise	mannosyl (alpha-1,6-)-glycoprotein beta-1,2-N-acetylglucosaminyltransferase(MGAT2)	Homo sapiens
3762	11751002_blue	dihydrolipoamide S-acetyltransferase(DLAT)	Homo sapiens
3763	11751013_turquoise	glycine receptor beta(GLRB)	Homo sapiens
3764	11751031_turquoise	c-src tyrosine kinase(CSK)	Homo sapiens
3765	11751084_turquoise	plexin D1(PLXND1)	Homo sapiens
3766	11751096_turquoise	sorting nexin 10(SNX10)	Homo sapiens
3767	11751097_turquoise	sorting nexin 10(SNX10)	Homo sapiens
3768	11751105_turquoise	wolframin ER transmembrane glycoprotein(WFS1)	Homo sapiens
3769	11751135_turquoise	CD86 molecule(CD86)	Homo sapiens
3770	11751141_turquoise	ARP3 actin related protein 3 homolog(ACTR3)	Homo sapiens
3771	11751162_turquoise	tweety family member 1(TTYH1)	Homo sapiens
3772	11751178_turquoise	ATPase H+ transporting V1 subunit A(ATP6V1A)	Homo sapiens
3773	11751202_turquoise	chromosome 19 open reading frame 66(C19orf66)	Homo sapiens
3774	11751225_turquoise	colony stimulating factor 2 receptor alpha subunit(CSF2RA)	Homo sapiens
3775	11751242_turquoise	Fc fragment of IgG receptor IIa(FCGR2A)	Homo sapiens
3776	11751288_turquoise	lysyl oxidase like 2(LOXL2)	Homo sapiens
3777	11751299_turquoise	apolipoprotein L2(APOL2)	Homo sapiens
3778	11751304_turquoise	integrin subunit alpha L(ITGAL)	Homo sapiens
3779	11751305_blue	beta-1,4-glucuronyltransferase 1(B4GAT1)	Homo sapiens
3780	11751318_turquoise	SENP3-EIF4A1 readthrough (NMD candidate)(SENP3-EIF4A1)	Homo sapiens
3781	11751319_turquoise	SENP3-EIF4A1 readthrough (NMD candidate)(SENP3-EIF4A1)	Homo sapiens
3782	11751332_turquoise	cytokine receptor like factor 3(CRLF3)	Homo sapiens
3783	11751399_turquoise	NDRG family member 3(NDRG3)	Homo sapiens
3784	11751422_turquoise	choline dehydrogenase(CHDH)	Homo sapiens
3785	11751424_turquoise	TRAF3 interacting protein 3(TRAF3IP3)	Homo sapiens
3786	11751425_turquoise	TRAF3 interacting protein 3(TRAF3IP3)	Homo sapiens
3787	11751440_blue	microtubule associated tumor suppressor candidate 2(MTUS2)	Homo sapiens
3788	11751465_turquoise	growth factor receptor bound protein 14(GRB14)	Homo sapiens
3789	11751467_turquoise	phosphatidylinositol glycan anchor biosynthesis class U(PIGU)	Homo sapiens
3790	11751482_turquoise	butyrophilin subfamily 3 member A2(BTN3A2)	Homo sapiens
3791	11751501_turquoise	fatty acid hydroxylase domain containing 2(FAXDC2)	Homo sapiens
3792	11751511_turquoise	ETS variant 7(ETV7)	Homo sapiens
3793	11751544_turquoise	myosin IF(MYO1F)	Homo sapiens
3794	11751571_turquoise	sarcoglycan alpha(SGCA)	Homo sapiens
3795	11751585_blue	ankyrin repeat and SOCS box containing 8(ASB8)	Homo sapiens
3796	11751596_turquoise	abl interactor 1(ABI1)	Homo sapiens
3797	11751597_turquoise	abl interactor 1(ABI1)	Homo sapiens
3798	11751600_turquoise	dual adaptor of phosphotyrosine and 3-phosphoinositides 1(DAPP1)	Homo sapiens

3799	11751602_turquoise	serine and arginine rich splicing factor 7(SRSF7)	Homo sapiens
3800	11751603_turquoise	serine and arginine rich splicing factor 7(SRSF7)	Homo sapiens
3801	11751611_turquoise	TRAF-type zinc finger domain containing 1(TRAFD1)	Homo sapiens
3802	11751616_blue	lactamase beta 2(LACTB2)	Homo sapiens
3803	11751618_turquoise	major histocompatibility complex, class I, E(HLA-E)	Homo sapiens
3804	11751619_turquoise	apolipoprotein L1(APOL1)	Homo sapiens
3805	11751628_turquoise	transmembrane protein 140(TMEM140)	Homo sapiens
3806	11751641_turquoise	PHD finger protein 11(PHF11)	Homo sapiens
3807	11751647_turquoise	interleukin 7 receptor(IL7R)	Homo sapiens
3808	11751651_turquoise	phospholipid scramblase 1(PLSCR1)	Homo sapiens
3809	11751652_turquoise	phospholipid scramblase 1(PLSCR1)	Homo sapiens
3810	11751680_brown	ubiquinol-cytochrome c reductase binding protein(UQCRB)	Homo sapiens
3811	11751688_turquoise	glycoprotein integral membrane 1(GINM1)	Homo sapiens
3812	11751690_turquoise	putative aquaporin-7-like protein 3(LOC100509620)	Homo sapiens
3813	11751702_blue	PATJ, crumbs cell polarity complex component(PATJ)	Homo sapiens
3814	11751713_turquoise	chromosome 6 open reading frame 62(C6orf62)	Homo sapiens
3815	11751724_turquoise	zinc finger protein 542, pseudogene(ZNF542P)	Homo sapiens
3816	11751746_brown	guanidinoacetate N-methyltransferase(GAMT)	Homo sapiens
3817	11751765_turquoise	ribonucleoprotein, PTB binding 2(RAVER2)	Homo sapiens
3818	11751766_turquoise	enoyl-CoA hydratase domain containing 2(ECHDC2)	Homo sapiens
3819	11751768_turquoise	sorting nexin 19(SNX19)	Homo sapiens
3820	11751776_blue	prohibitin(PHB)	Homo sapiens
3821	11751778_brown	translocase of inner mitochondrial membrane 23(TIMM23)	Homo sapiens
3822	11751782_turquoise	EF-hand domain containing 2(EFHC2)	Homo sapiens
3823	11751799_turquoise	peroxiredoxin 6(PRDX6)	Homo sapiens
3824	11751805_turquoise	thymidylate synthetase(TYMS)	Homo sapiens
3825	11751821_turquoise	cytochrome b-245 beta chain(CYBB)	Homo sapiens
3826	11751833_turquoise	butyrylcholinesterase(BCHE)	Homo sapiens
3827	11751857_turquoise	granulysin(GNLY)	Homo sapiens
3828	11751861_blue	hyaluronoglucosaminidase 1(HYAL1)	Homo sapiens
3829	11751888_turquoise	collagen type IV alpha 6 chain(COL4A6)	Homo sapiens
3830	11751890_turquoise	sodium voltage-gated channel alpha subunit 5(SCN5A)	Homo sapiens
3831	11751893_turquoise	collagen type IV alpha 6 chain(COL4A6)	Homo sapiens
3832	11751917_turquoise	myotubularin related protein 12(MTMR12)	Homo sapiens
3833	11751921_turquoise	aryl hydrocarbon receptor(AHR)	Homo sapiens
3834	11752003_turquoise	IQ motif containing GTPase activating protein 1(IQGAP1)	Homo sapiens
3835	11752009_turquoise	tenascin C(TNC)	Homo sapiens
3836	11752036_turquoise	transmembrane protein 123(TMEM123)	Homo sapiens
3837	11752051_turquoise	myosin heavy chain 9(MYH9)	Homo sapiens
3838	11752052_turquoise	myosin heavy chain 9(MYH9)	Homo sapiens
3839	11752065_turquoise	CD6 molecule(CD6)	Homo sapiens
3840	11752095_turquoise	protein tyrosine phosphatase, receptor type C(PTPRC)	Homo sapiens
3841	11752142_turquoise	filamin A(FLNA)	Homo sapiens
3842	11752149_turquoise	ubiquitin associated and SH3 domain containing A(UBASH3A)	Homo sapiens
3843	11752153_turquoise	neurobeachin like 2(NBEAL2)	Homo sapiens
3844	11752154_turquoise	neurobeachin like 2(NBEAL2)	Homo sapiens
3845	11752163_turquoise	lysine demethylase 2B(KDM2B)	Homo sapiens
3846	11752164_turquoise	lysine demethylase 2B(KDM2B)	Homo sapiens
3847	11752171_turquoise	serine palmitoyltransferase long chain base subunit 1(SPTLC1)	Homo sapiens
3848	11752173_turquoise	transmembrane protein 2(TMEM2)	Homo sapiens

3849	11752175_ turquoise	DBF4 zinc finger(DBF4)	Homo sapiens
3850	11752180_ turquoise	von Hippel-Lindau tumor suppressor(VHL)	Homo sapiens
3851	11752224_ turquoise	schlafen family member 5(SLFN5)	Homo sapiens
3852	11752233_ turquoise	copine 4(CPNE4)	Homo sapiens
3853	11752235_ turquoise	inhibitor of kappa light polypeptide gene enhancer in B-cells, kinase beta(IKBKB)	Homo sapiens
3854	11752265_ turquoise	androgen receptor(AR)	Homo sapiens
3855	11752267_ turquoise	ribosomal protein S6 kinase A4(RPS6KA4)	Homo sapiens
3856	11752273_ turquoise	colony stimulating factor 1 receptor(CSF1R)	Homo sapiens
3857	11752278_ turquoise	poly(A) binding protein cytoplasmic 1(PABPC1)	Homo sapiens
3858	11752297_ turquoise	class II major histocompatibility complex transactivator(CIITA)	Homo sapiens
3859	11752318_ turquoise	butyrophilin subfamily 3 member A3(BTN3A3)	Homo sapiens
3860	11752331_ turquoise	SLX1A-SULT1A3 readthrough (NMD candidate)(SLX1A-SULT1A3)	Homo sapiens
3861	11752341_ turquoise	coagulation factor VIII(F8)	Homo sapiens
3862	11752379_ turquoise	multiple C2 and transmembrane domain containing 1(MCTP1)	Homo sapiens
3863	11752387_ turquoise	ETS proto-oncogene 1, transcription factor(ETS1)	Homo sapiens
3864	11752398_ turquoise	chromosome 1 open reading frame 228(C1orf228)	Homo sapiens
3865	11752411_ turquoise	coiled-coil domain containing 8(CCDC8)	Homo sapiens
3866	11752467_ turquoise	inositol polyphosphate-5-phosphatase D(INPP5D)	Homo sapiens
3867	11752482_ turquoise	fibrillin 2(FBN2)	Homo sapiens
3868	11752535_ turquoise	cyclin L1(CCNL1)	Homo sapiens
3869	11752568_ turquoise	LCK proto-oncogene, Src family tyrosine kinase(LCK)	Homo sapiens
3870	11752569_ turquoise	LCK proto-oncogene, Src family tyrosine kinase(LCK)	Homo sapiens
3871	11752606_ turquoise	microRNA 6845(MIR6845)	Homo sapiens
3872	11752607_ turquoise	microRNA 6132(MIR6132)	Homo sapiens
3873	11752608_ turquoise	microRNA 6132(MIR6132)	Homo sapiens
3874	11752610_ turquoise	thymocyte selection associated family member 2(THEMIS2)	Homo sapiens
3875	11752611_ turquoise	signal sequence receptor subunit 1(SSR1)	Homo sapiens
3876	11752631_ turquoise	SAM and SH3 domain containing 3(SASH3)	Homo sapiens
3877	11752635_ turquoise	potassium voltage-gated channel subfamily H member 2(KCNH2)	Homo sapiens
3878	11752644_ turquoise	TIA1 cytotoxic granule associated RNA binding protein like 1(TIAL1)	Homo sapiens
3879	11752649_ blue	aconitase 2(ACO2)	Homo sapiens
3880	11752697_ turquoise	PC-esterase domain containing 1A(PCED1A)	Homo sapiens
3881	11752698_ turquoise	PC-esterase domain containing 1A(PCED1A)	Homo sapiens
3882	11752723_ turquoise	CD200 receptor 1(CD200R1)	Homo sapiens
3883	11752732_ turquoise	dihydrouridine synthase 4 like(DUS4L)	Homo sapiens
3884	11752737_ turquoise	C-type lectin domain family 7 member A(CLEC7A)	Homo sapiens
3885	11752747_ blue	MRS2, magnesium transporter(MRS2)	Homo sapiens
3886	11752748_ blue	MRS2, magnesium transporter(MRS2)	Homo sapiens
3887	11752769_ turquoise	piggyBac transposable element derived 5(PGBD5)	Homo sapiens
3888	11752771_ turquoise	carbohydrate sulfotransferase 15(CHST15)	Homo sapiens
3889	11752794_ turquoise	signal transducer and activator of transcription 2(STAT2)	Homo sapiens
3890	11752795_ turquoise	signal transducer and activator of transcription 2(STAT2)	Homo sapiens
3891	11752796_ turquoise	chromosome 3 open reading frame 33(C3orf33)	Homo sapiens
3892	11752801_ turquoise	SP110 nuclear body protein(SP110)	Homo sapiens
3893	11752802_ turquoise	SP110 nuclear body protein(SP110)	Homo sapiens
3894	11752812_ turquoise	tweety family member 1(TTYH1)	Homo sapiens
3895	11752814_ turquoise	TNF receptor superfamily member 1A(TNFRSF1A)	Homo sapiens
3896	11752817_ turquoise	tripeptidyl peptidase 1(TPP1)	Homo sapiens
3897	11752833_ turquoise	post-GPI attachment to proteins 2(PGAP2)	Homo sapiens
3898	11752865_ turquoise	actin beta(ACTB)	Homo sapiens

3899	11752873_turquoise	src kinase associated phosphoprotein 1(SKAP1)	Homo sapiens
3900	11752877_turquoise	tubulin beta class I(TUBB)	Homo sapiens
3901	11752890_turquoise	syntrophin alpha 1(SNTA1)	Homo sapiens
3902	11752898_turquoise	peptidylprolyl isomerase like 6(PPIL6)	Homo sapiens
3903	11752910_turquoise	ADP ribosylation factor like GTPase 6 interacting protein 1(ARL6IP1)	Homo sapiens
3904	11752915_blue	ryanodine receptor 2(RYR2)	Homo sapiens
3905	11752918_turquoise	Fc receptor like 6(FCRL6)	Homo sapiens
3906	11752919_blue	acyl-CoA dehydrogenase, C-4 to C-12 straight chain(ACADM)	Homo sapiens
3907	11752930_turquoise	guanylate binding protein 1(GBP1)	Homo sapiens
3908	11752931_turquoise	guanylate binding protein 1(GBP1)	Homo sapiens
3909	11752948_turquoise	prolylcarboxypeptidase(PRCP)	Homo sapiens
3910	11752959_turquoise	actin beta(ACTB)	Homo sapiens
3911	11752964_blue	solute carrier family 25 member 20(SLC25A20)	Homo sapiens
3912	11752987_turquoise	EPN2 intronic transcript 1(EPN2-IT1)	Homo sapiens
3913	11753025_turquoise	TNF receptor superfamily member 1B(TNFRSF1B)	Homo sapiens
3914	11753034_turquoise	zinc finger NFX1-type containing 1(ZNFX1)	Homo sapiens
3915	11753055_turquoise	RAP1B, member of RAS oncogene family(RAP1B)	Homo sapiens
3916	11753056_turquoise	zinc finger protein 667(ZNF667)	Homo sapiens
3917	11753061_turquoise	schlafen family member 5(SLFN5)	Homo sapiens
3918	11753088_turquoise	multiple C2 and transmembrane domain containing 1(MCTP1)	Homo sapiens
3919	11753090_turquoise	opioid growth factor receptor(OGFR)	Homo sapiens
3920	11753106_turquoise	target of myb1 like 2 membrane trafficking protein(TOM1L2)	Homo sapiens
3921	11753111_turquoise	interleukin 10 receptor subunit beta(IL10RB)	Homo sapiens
3922	11753129_turquoise	transmembrane 4 L six family member 1(TM4SF1)	Homo sapiens
3923	11753132_turquoise	solute carrier family 50 member 1(SLC50A1)	Homo sapiens
3924	11753151_turquoise	histone deacetylase 8(HDAC8)	Homo sapiens
3925	11753165_turquoise	RAB27A, member RAS oncogene family(RAB27A)	Homo sapiens
3926	11753213_turquoise	G protein subunit alpha i2(GNAI2)	Homo sapiens
3927	11753219_turquoise	killer cell lectin like receptor F1(KLRF1)	Homo sapiens
3928	11753239_turquoise	scavenger receptor class A member 5(SCARA5)	Homo sapiens
3929	11753241_turquoise	C-type lectin domain family 12 member A(CLEC12A)	Homo sapiens
3930	11753266_turquoise	transcription elongation factor A3(TCEA3)	Homo sapiens
3931	11753291_turquoise	CD44 molecule (Indian blood group)(CD44)	Homo sapiens
3932	11753292_turquoise	CD44 molecule (Indian blood group)(CD44)	Homo sapiens
3933	11753312_turquoise	DnaJ heat shock protein family (Hsp40) member C5 gamma(DNAJC5G)	Homo sapiens
3934	11753321_turquoise	TNFSF12-TNFSF13 readthrough(TNFSF12-TNFSF13)	Homo sapiens
3935	11753322_brown	diphthamide biosynthesis 5(DPH5)	Homo sapiens
3936	11753334_turquoise	BCL2 associated athanogene 1(BAG1)	Homo sapiens
3937	11753335_turquoise	transmembrane protein 177(TMEM177)	Homo sapiens
3938	11753345_blue	cutC copper transporter(CUTC)	Homo sapiens
3939	11753348_turquoise	SAM domain, SH3 domain and nuclear localization signals 1(SAMSN1)	Homo sapiens
3940	11753399_turquoise	annexin A2(ANXA2)	Homo sapiens
3941	11753404_turquoise	major histocompatibility complex, class I, E(HLA-E)	Homo sapiens
3942	11753445_turquoise	heme oxygenase 1(HMOX1)	Homo sapiens
3943	11753446_turquoise	heme oxygenase 1(HMOX1)	Homo sapiens
3944	11753467_blue	aminomethyltransferase(AMT)	Homo sapiens
3945	11753480_blue	reticulon 4 interacting protein 1(RTN4IP1)	Homo sapiens
3946	11753484_turquoise	killer cell lectin like receptor D1(KLRD1)	Homo sapiens
3947	11753505_blue	post-GPI attachment to proteins 2(PGAP2)	Homo sapiens
3948	11753515_turquoise	interleukin 32(IL32)	Homo sapiens

3949	11753519_turquoise	gamma-secretase activating protein(GSAP)	Homo sapiens
3950	11753521_turquoise	RAB5C, member RAS oncogene family(RAB5C)	Homo sapiens
3951	11753534_turquoise	killer cell lectin like receptor D1(KLRD1)	Homo sapiens
3952	11753535_turquoise	killer cell lectin like receptor D1(KLRD1)	Homo sapiens
3953	11753549_turquoise	CKLF like MARVEL transmembrane domain containing 3(CMTM3)	Homo sapiens
3954	11753555_turquoise	CD53 molecule(CD53)	Homo sapiens
3955	11753569_turquoise	leucine zipper tumor suppressor 1(LZTS1)	Homo sapiens
3956	11753575_turquoise	coenzyme Q7, hydroxylase(COQ7)	Homo sapiens
3957	11753623_turquoise	RAP1B, member of RAS oncogene family(RAP1B)	Homo sapiens
3958	11753628_blue	abhydrolase domain containing 11(ABHD11)	Homo sapiens
3959	11753675_turquoise	profilin 2(PFN2)	Homo sapiens
3960	11753722_turquoise	profilin 1(PFN1)	Homo sapiens
3961	11753727_turquoise	microRNA 6805(MIR6805)	Homo sapiens
3962	11753732_turquoise	polyamine oxidase(PAOX)	Homo sapiens
3963	11753735_turquoise	thymosin beta 4, X-linked(TMSB4X)	Homo sapiens
3964	11753755_brown	ATP synthase, H+ transporting, mitochondrial Fo complex subunit F2(ATP5J2)	Homo sapiens
3965	11753776_turquoise	leukocyte associated immunoglobulin like receptor 2(LAIR2)	Homo sapiens
3966	11753799_brown	small integral membrane protein 11A(SMIM11A)	Homo sapiens
3967	11753800_brown	small integral membrane protein 11A(SMIM11A)	Homo sapiens
3968	11753803_blue	cytochrome c, somatic(CYCS)	Homo sapiens
3969	11753810_turquoise	C-C motif chemokine ligand 5(CCL5)	Homo sapiens
3970	11753832_yellow	immunoglobulin kappa constant(IGKC)	Homo sapiens
3971	11753843_brown	NADH:ubiquinone oxidoreductase subunit A3(NDUFA3)	Homo sapiens
3972	11753844_brown	NADH:ubiquinone oxidoreductase subunit A3(NDUFA3)	Homo sapiens
3973	11753856_turquoise	CDC28 protein kinase regulatory subunit 2(CKS2)	Homo sapiens
3974	11753858_turquoise	small nucleolar RNA, H/ACA box 44(SNORA44)	Homo sapiens
3975	11753859_turquoise	small nucleolar RNA, H/ACA box 44(SNORA44)	Homo sapiens
3976	11753868_turquoise	leukocyte specific transcript 1(LST1)	Homo sapiens
3977	11753871_brown	G protein subunit gamma 7(GNG7)	Homo sapiens
3978	11753872_brown	metallothionein 3(MT3)	Homo sapiens
3979	11753882_turquoise	interleukin 2 receptor subunit gamma(IL2RG)	Homo sapiens
3980	11753883_turquoise	interleukin 2 receptor subunit gamma(IL2RG)	Homo sapiens
3981	11753896_blue	pyruvate dehydrogenase (lipoamide) alpha 1(PDHA1)	Homo sapiens
3982	11753898_turquoise	major histocompatibility complex, class II, DQ alpha 1(HLA-DQA1)	Homo sapiens
3983	11753902_turquoise	actinin alpha 4(ACTN4)	Homo sapiens
3984	11753911_turquoise	transmembrane protein 242(TMEM242)	Homo sapiens
3985	11753915_turquoise	SEL1L family member 3(SEL1L3)	Homo sapiens
3986	11753918_turquoise	nucleoporin 85(NUP85)	Homo sapiens
3987	11753920_turquoise	apolipoprotein L1(APOL1)	Homo sapiens
3988	11753923_turquoise	MBNL1 antisense RNA 1(MBNL1-AS1)	Homo sapiens
3989	11753929_turquoise	ST3 beta-galactoside alpha-2,3-sialyltransferase 2(ST3GAL2)	Homo sapiens
3990	11753936_turquoise	transient receptor potential cation channel subfamily M member 2(TRPM2)	Homo sapiens
3991	11753939_turquoise	proteasome subunit beta 10(PSMB10)	Homo sapiens
3992	11753988_turquoise	sprouty RTK signaling antagonist 2(SPRY2)	Homo sapiens
3993	11753996_brown	heat shock protein family E (Hsp10) member 1(HSPE1)	Homo sapiens
3994	11754015_brown	translocase of inner mitochondrial membrane 50(TIMM50)	Homo sapiens
3995	11754016_brown	translocase of inner mitochondrial membrane 50(TIMM50)	Homo sapiens
3996	11754023_blue	cytochrome c oxidase subunit 7A2 like(COX7A2L)	Homo sapiens
3997	11754024_blue	solute carrier family 25 member 11(SLC25A11)	Homo sapiens
3998	11754027_blue	cytochrome c1(CYC1)	Homo sapiens

3999	11754032_	yellow	microRNA 8071-1(MIR8071-1)	Homo sapiens
4000	11754034_	brown	reactive intermediate imine deaminase A homolog(RIDA)	Homo sapiens
4001	11754035_	turquoise	interferon regulatory factor 1(IRF1)	Homo sapiens
4002	11754064_	turquoise	annexin A2(ANXA2)	Homo sapiens
4003	11754071_	turquoise	transgelin 2(TAGLN2)	Homo sapiens
4004	11754105_	turquoise	small nuclear ribonucleoprotein polypeptides B and B1(SNRPB)	Homo sapiens
4005	11754124_	turquoise	coactosin like F-actin binding protein 1(COTL1)	Homo sapiens
4006	11754125_	turquoise	coactosin like F-actin binding protein 1(COTL1)	Homo sapiens
4007	11754142_	turquoise	microRNA 1244-1(MIR1244-1)	Homo sapiens
4008	11754145_	yellow	NA	NA
4009	11754153_	turquoise	caspase 8(CASP8)	Homo sapiens
4010	11754161_	brown	phosphatidylinositol glycan anchor biosynthesis class P(PIGP)	Homo sapiens
4011	11754170_	turquoise	transforming growth factor beta regulator 1(TBRG1)	Homo sapiens
4012	11754181_	turquoise	interleukin 2 receptor subunit gamma(IL2RG)	Homo sapiens
4013	11754182_	turquoise	interleukin 2 receptor subunit gamma(IL2RG)	Homo sapiens
4014	11754202_	turquoise	capping actin protein, gelsolin like(CAPG)	Homo sapiens
4015	11754204_	turquoise	peroxisomal biogenesis factor 11 alpha(PEX11A)	Homo sapiens
4016	11754205_	turquoise	lysine acetyltransferase 6B(KAT6B)	Homo sapiens
4017	11754217_	turquoise	actin beta(ACTB)	Homo sapiens
4018	11754219_	turquoise	signal induced proliferation associated 1 like 1(SIPA1L1)	Homo sapiens
4019	11754223_	turquoise	transmembrane protein 35B(TMEM35B)	Homo sapiens
4020	11754224_	turquoise	RAS p21 protein activator 4B(RASA4B)	Homo sapiens
4021	11754225_	turquoise	Sec23 homolog B, coat complex II component(SEC23B)	Homo sapiens
4022	11754243_	turquoise	H2A histone family member X(H2AFX)	Homo sapiens
4023	11754246_	turquoise	poly(A) binding protein cytoplasmic 1(PABPC1)	Homo sapiens
4024	11754258_	turquoise	lipoic acid synthetase(LIAS)	Homo sapiens
4025	11754266_	turquoise	E2F transcription factor 3(E2F3)	Homo sapiens
4026	11754270_	turquoise	complement component 4B (Chido blood group), copy 2(C4B_2)	Homo sapiens
4027	11754272_	blue	small nuclear ribonucleoprotein polypeptide N(SNRPN)	Homo sapiens
4028	11754278_	turquoise	cyclin G associated kinase(GAK)	Homo sapiens
4029	11754295_	turquoise	family with sequence similarity 107 member B(FAM107B)	Homo sapiens
4030	11754297_	turquoise	TNF receptor superfamily member 1A(TNFRSF1A)	Homo sapiens
4031	11754298_	turquoise	casein kinase 1 delta(CSNK1D)	Homo sapiens
4032	11754313_	turquoise	ADP ribosylation factor like GTPase 4C(ARL4C)	Homo sapiens
4033	11754315_	turquoise	thymosin beta 4, X-linked(TMSB4X)	Homo sapiens
4034	11754317_	turquoise	annexin A4(ANXA4)	Homo sapiens
4035	11754320_	turquoise	structural maintenance of chromosomes 4(SMC4)	Homo sapiens
4036	11754321_	turquoise	solute carrier family 12 member 9(SLC12A9)	Homo sapiens
4037	11754330_	turquoise	interferon regulatory factor 2(IRF2)	Homo sapiens
4038	11754339_	turquoise	NAD synthetase 1(NADSYN1)	Homo sapiens
4039	11754348_	turquoise	kelch domain containing 4(KLHDC4)	Homo sapiens
4040	11754351_	turquoise	cytohesin 1(CYTH1)	Homo sapiens
4041	11754352_	turquoise	ring finger protein 213(RNF213)	Homo sapiens
4042	11754358_	turquoise	small nucleolar RNA host gene 4(SNHG4)	Homo sapiens
4043	11754360_	turquoise	ribonucleotide reductase regulatory subunit M2(RRM2)	Homo sapiens
4044	11754370_	turquoise	synaptotagmin like 1(SYTL1)	Homo sapiens
4045	11754375_	turquoise	sorting nexin 10(SNX10)	Homo sapiens
4046	11754379_	turquoise	poly(ADP-ribose) polymerase family member 9(PARP9)	Homo sapiens
4047	11754381_	blue	tropomodulin 1(TMOD1)	Homo sapiens
4048	11754382_	turquoise	small integral membrane protein 5(SMIM5)	Homo sapiens

4049	11754403_turquoise	poly(ADP-ribose) polymerase family member 14(PARP14)	Homo sapiens
4050	11754404_turquoise	poly(ADP-ribose) polymerase family member 14(PARP14)	Homo sapiens
4051	11754419_turquoise	nucleolus and neural progenitor protein(NEPRO)	Homo sapiens
4052	11754434_turquoise	cyclin D3(CCND3)	Homo sapiens
4053	11754444_brown	cytochrome c oxidase subunit 6C(COX6C)	Homo sapiens
4054	11754454_turquoise	LDL receptor related protein 10(LRP10)	Homo sapiens
4055	11754461_turquoise	uncoupling protein 2(UCP2)	Homo sapiens
4056	11754471_turquoise	major histocompatibility complex, class II, DM beta(HLA-DMB)	Homo sapiens
4057	11754474_turquoise	pleckstrin(PLEK)	Homo sapiens
4058	11754481_turquoise	coronin 7(CORO7)	Homo sapiens
4059	11754489_brown	NADH:ubiquinone oxidoreductase complex assembly factor 5(NDUFAF5)	Homo sapiens
4060	11754491_turquoise	chromosome 16 open reading frame 46(C16orf46)	Homo sapiens
4061	11754492_turquoise	phospholipase C beta 2(PLCB2)	Homo sapiens
4062	11754503_turquoise	choline/ethanolamine phosphotransferase 1(CEPT1)	Homo sapiens
4063	11754510_blue	voltage dependent anion channel 3(VDAC3)	Homo sapiens
4064	11754524_turquoise	tissue factor pathway inhibitor 2(TFPI2)	Homo sapiens
4065	11754551_turquoise	enolase 1(ENO1)	Homo sapiens
4066	11754554_turquoise	enoyl-CoA hydratase domain containing 2(ECHDC2)	Homo sapiens
4067	11754579_turquoise	pappalysin 1(PAPPA)	Homo sapiens
4068	11754584_turquoise	RAD9-HUS1-RAD1 interacting nuclear orphan 1(RHNO1)	Homo sapiens
4069	11754587_turquoise	FYN proto-oncogene, Src family tyrosine kinase(FYN)	Homo sapiens
4070	11754589_turquoise	exportin 6(XPO6)	Homo sapiens
4071	11754592_turquoise	activating transcription factor 5(ATF5)	Homo sapiens
4072	11754593_turquoise	tubulin beta 3 class III(TUBB3)	Homo sapiens
4073	11754598_blue	DnaJ heat shock protein family (Hsp40) member A3(DNAJA3)	Homo sapiens
4074	11754603_turquoise	dual specificity phosphatase 18(DUSP18)	Homo sapiens
4075	11754609_turquoise	stathmin 3(STMN3)	Homo sapiens
4076	11754615_turquoise	ubiquitin fold modifier 1(UFM1)	Homo sapiens
4077	11754623_brown	choline phosphotransferase 1(CHPT1)	Homo sapiens
4078	11754627_turquoise	RAB37, member RAS oncogene family(RAB37)	Homo sapiens
4079	11754630_turquoise	mixed lineage kinase domain like(MLKL)	Homo sapiens
4080	11754637_turquoise	synaptotagmin like 1(SYTL1)	Homo sapiens
4081	11754644_turquoise	calponin 2(CNN2)	Homo sapiens
4082	11754649_turquoise	IL2 inducible T-cell kinase(ITK)	Homo sapiens
4083	11754652_brown	enoyl-CoA delta isomerase 2(ECI2)	Homo sapiens
4084	11754666_turquoise	coronin 1A(CORO1A)	Homo sapiens
4085	11754672_turquoise	checkpoint with forkhead and ring finger domains(CHFR)	Homo sapiens
4086	11754678_turquoise	solute carrier family 25 member 43(SLC25A43)	Homo sapiens
4087	11754680_turquoise	mitogen-activated protein kinase kinase kinase 2(MAP3K2)	Homo sapiens
4088	11754681_turquoise	major histocompatibility complex, class II, DM beta(HLA-DMB)	Homo sapiens
4089	11754695_turquoise	phospholipase A2 group V(PLA2G5)	Homo sapiens
4090	11754707_brown	CUE domain containing 2(CUEDC2)	Homo sapiens
4091	11754712_turquoise	TBC1 domain family member 10A(TBC1D10A)	Homo sapiens
4092	11754716_turquoise	GATA zinc finger domain containing 2A(GATAD2A)	Homo sapiens
4093	11754722_turquoise	SEPSECS antisense RNA 1 (head to head)(SEPSECS-AS1)	Homo sapiens
4094	11754724_turquoise	coiled-coil domain containing 88B(CCDC88B)	Homo sapiens
4095	11754751_turquoise	BCL2 associated athanogene 1(BAG1)	Homo sapiens
4096	11754752_turquoise	outer dense fiber of sperm tails 3B(ODF3B)	Homo sapiens
4097	11754759_turquoise	chromatin target of PRMT1(CHTOP)	Homo sapiens
4098	11754760_turquoise	GRAM domain containing 1A(GRAMD1A)	Homo sapiens

4099	11754761_turquoise	high mobility group nucleosome binding domain 1(HMGN1)	Homo sapiens
4100	11754762_turquoise	T-cell immune regulator 1, ATPase H+ transporting V0 subunit a3(TCIRG1)	Homo sapiens
4101	11754765_turquoise	microRNA 6080(MIR6080)	Homo sapiens
4102	11754769_turquoise	BEN domain containing 5(BEND5)	Homo sapiens
4103	11754777_turquoise	SH2 domain containing 2A(SH2D2A)	Homo sapiens
4104	11754783_brown	COMM domain containing 3(COMMD3)	Homo sapiens
4105	11754798_turquoise	CD164 molecule(CD164)	Homo sapiens
4106	11754802_turquoise	chromosome 12 open reading frame 76(C12orf76)	Homo sapiens
4107	11754808_turquoise	C3 and PZP like, alpha-2-macroglobulin domain containing 8(CPAMD8)	Homo sapiens
4108	11754814_turquoise	nucleoporin 93(NUP93)	Homo sapiens
4109	11754822_turquoise	tyrosyl-DNA phosphodiesterase 1(TDP1)	Homo sapiens
4110	11754828_turquoise	butyrophilin subfamily 2 member A2(BTN2A2)	Homo sapiens
4111	11754833_turquoise	toll like receptor 2(TLR2)	Homo sapiens
4112	11754835_turquoise	immunoglobin superfamily member 21(IGSF21)	Homo sapiens
4113	11754839_turquoise	ubiquitin specific peptidase 54(USP54)	Homo sapiens
4114	11754842_turquoise	meiotic double-stranded break formation protein 1(MEI1)	Homo sapiens
4115	11754844_blue	COP9 signalosome subunit 5(COPS5)	Homo sapiens
4116	11754859_turquoise	RUN and cysteine rich domain containing beclin 1 interacting protein like(RUBCNL)	Homo sapiens
4117	11754861_brown	succinate-CoA ligase alpha subunit(SUCLG1)	Homo sapiens
4118	11754869_turquoise	zinc finger protein 267(ZNF267)	Homo sapiens
4119	11754870_turquoise	Mab-21 domain containing 1(MB21D1)	Homo sapiens
4120	11754881_turquoise	signal regulatory protein gamma(SIRPG)	Homo sapiens
4121	11754884_turquoise	WD repeat and FYVE domain containing 1(WDFY1)	Homo sapiens
4122	11754887_turquoise	methionine sulfoxide reductase B3(MSRB3)	Homo sapiens
4123	11754888_blue	adenylosuccinate synthase like 1(ADSSL1)	Homo sapiens
4124	11754910_turquoise	MICAL like 2(MICALL2)	Homo sapiens
4125	11754917_turquoise	SP100 nuclear antigen(SP100)	Homo sapiens
4126	11754920_turquoise	vesicular, overexpressed in cancer, prosurvival protein 1(VOPP1)	Homo sapiens
4127	11754925_turquoise	spleen associated tyrosine kinase(SYK)	Homo sapiens
4128	11754942_turquoise	chromosome 17 open reading frame 62(C17orf62)	Homo sapiens
4129	11754945_turquoise	transketolase(TKT)	Homo sapiens
4130	11754946_turquoise	chromosome 7 open reading frame 49(C7orf49)	Homo sapiens
4131	11754951_turquoise	nuclear receptor coactivator 7(NCOA7)	Homo sapiens
4132	11754964_brown	NADH:ubiquinone oxidoreductase subunit A10(NDUFA10)	Homo sapiens
4133	11754972_turquoise	bromodomain adjacent to zinc finger domain 2A(BAZ2A)	Homo sapiens
4134	11754976_turquoise	canopy FGF signaling regulator 3(CNPY3)	Homo sapiens
4135	11754989_turquoise	Dmx like 2(DMXL2)	Homo sapiens
4136	11754992_turquoise	chromodomain helicase DNA binding protein 1(CHD1)	Homo sapiens
4137	11754998_turquoise	multiple C2 and transmembrane domain containing 1(MCTP1)	Homo sapiens
4138	11755010_turquoise	POC1B-GALNT4 readthrough(POC1B-GALNT4)	Homo sapiens
4139	11755012_turquoise	CDC42 small effector 2(CDC42SE2)	Homo sapiens
4140	11755013_turquoise	TNF receptor superfamily member 1B(TNFRSF1B)	Homo sapiens
4141	11755018_blue	protein phosphatase 1 catalytic subunit gamma(PPP1CC)	Homo sapiens
4142	11755022_turquoise	ADP ribosylation factor like GTPase 6 interacting protein 1(ARL6IP1)	Homo sapiens
4143	11755032_turquoise	ArfGAP with SH3 domain, ankyrin repeat and PH domain 1(ASAP1)	Homo sapiens
4144	11755036_turquoise	2',3'-cyclic nucleotide 3' phosphodiesterase(CNP)	Homo sapiens
4145	11755043_turquoise	gamma-secretase activating protein(GSAP)	Homo sapiens
4146	11755044_turquoise	gamma-secretase activating protein(GSAP)	Homo sapiens
4147	11755045_turquoise	ankyrin repeat domain 13A(ANKRD13A)	Homo sapiens
4148	11755054_turquoise	runt related transcription factor 3(RUNX3)	Homo sapiens

4149	11755058_	turquoise	bromodomain adjacent to zinc finger domain 1A(BAZ1A)	Homo sapiens
4150	11755059_	turquoise	transcription factor 3(TCF3)	Homo sapiens
4151	11755070_	turquoise	caspase recruitment domain family member 8(CARD8)	Homo sapiens
4152	11755075_	turquoise	NAD synthetase 1(NADSYN1)	Homo sapiens
4153	11755076_	turquoise	ORAI calcium release-activated calcium modulator 2(ORAI2)	Homo sapiens
4154	11755078_	turquoise	transcriptional adaptor 2B(TADA2B)	Homo sapiens
4155	11755091_	turquoise	ATP binding cassette subfamily A member 7(ABCA7)	Homo sapiens
4156	11755105_	turquoise	diacylglycerol kinase zeta(DGKZ)	Homo sapiens
4157	11755116_	turquoise	abhydrolase domain containing 11(ABHD11)	Homo sapiens
4158	11755117_	turquoise	F-box and leucine rich repeat protein 22(FBXL22)	Homo sapiens
4159	11755121_	turquoise	organic solute carrier partner 1(OSCP1)	Homo sapiens
4160	11755135_	turquoise	abhydrolase domain containing 1(ABHD1)	Homo sapiens
4161	11755137_	turquoise	mitochondrial calcium uniporter dominant negative beta subunit(MCUB)	Homo sapiens
4162	11755147_	turquoise	signal transducer and activator of transcription 2(STAT2)	Homo sapiens
4163	11755148_	turquoise	Dmx like 2(DMXL2)	Homo sapiens
4164	11755151_	turquoise	aryl hydrocarbon receptor nuclear translocator like(ARNTL)	Homo sapiens
4165	11755175_	turquoise	heat shock protein family A (Hsp70) member 12A(HSPA12A)	Homo sapiens
4166	11755180_	turquoise	transcription factor 7 (T-cell specific, HMG-box)(TCF7)	Homo sapiens
4167	11755222_	turquoise	G protein subunit alpha 13(GNA13)	Homo sapiens
4168	11755231_	turquoise	LCK proto-oncogene, Src family tyrosine kinase(LCK)	Homo sapiens
4169	11755235_	turquoise	fatty acyl-CoA reductase 2(FAR2)	Homo sapiens
4170	11755237_	turquoise	protein phosphatase, Mg2+/Mn2+ dependent 1M(PPM1M)	Homo sapiens
4171	11755246_	turquoise	growth regulation by estrogen in breast cancer 1(GREB1)	Homo sapiens
4172	11755247_	turquoise	DAN domain BMP antagonist family member 5(DAND5)	Homo sapiens
4173	11755265_	turquoise	5'-nucleotidase domain containing 1(NT5DC1)	Homo sapiens
4174	11755266_	blue	succinate-CoA ligase ADP-forming beta subunit(SUCLA2)	Homo sapiens
4175	11755269_	brown	URGCP-MRPS24 readthrough(URGCP-MRPS24)	Homo sapiens
4176	11755279_	turquoise	major histocompatibility complex, class II, DP beta 1(HLA-DPB1)	Homo sapiens
4177	11755280_	turquoise	ferritin light chain(FTL)	Homo sapiens
4178	11755281_	turquoise	transforming growth factor beta induced(TGFBI)	Homo sapiens
4179	11755291_	turquoise	DENN domain containing 2D(DENND2D)	Homo sapiens
4180	11755292_	turquoise	cytochrome b561 family member A3(CYB561A3)	Homo sapiens
4181	11755303_	turquoise	major histocompatibility complex, class I, A(HLA-A)	Homo sapiens
4182	11755315_	turquoise	arginyl aminopeptidase(RNPEP)	Homo sapiens
4183	11755319_	turquoise	ankyrin repeat domain 13D(ANKRD13D)	Homo sapiens
4184	11755320_	turquoise	ankyrin repeat domain 13D(ANKRD13D)	Homo sapiens
4185	11755326_	turquoise	abhydrolase domain containing 16A(ABHD16A)	Homo sapiens
4186	11755327_	turquoise	family with sequence similarity 115, member C pseudogene(LOC154761)	Homo sapiens
4187	11755331_	turquoise	ankyrin 2(ANK2)	Homo sapiens
4188	11755342_	turquoise	microRNA 1292(MIR1292)	Homo sapiens
4189	11755343_	turquoise	SET domain and mariner transposase fusion gene(SETMAR)	Homo sapiens
4190	11755344_	turquoise	major histocompatibility complex, class I, A(HLA-A)	Homo sapiens
4191	11755346_	brown	ATP synthase, H+ transporting, mitochondrial F1 complex, O subunit(ATP5O)	Homo sapiens
4192	11755347_	brown	secretion associated Ras related GTPase 1B(SAR1B)	Homo sapiens
4193	11755355_	turquoise	RCSD domain containing 1(RCSD1)	Homo sapiens
4194	11755357_	turquoise	aconitase 1(ACO1)	Homo sapiens
4195	11755361_	turquoise	de-etiolated homolog 1 (Arabidopsis)(DET1)	Homo sapiens
4196	11755373_	turquoise	enoyl-CoA hydratase domain containing 2(ECHDC2)	Homo sapiens
4197	11755374_	turquoise	HECT and RLD domain containing E3 ubiquitin protein ligase 5(HERC5)	Homo sapiens
4198	11755382_	yellow	immunoglobulin lambda variable 1-44(IGLV1-44)	Homo sapiens

4199	11755387_	turquoise	SAC1 suppressor of actin mutations 1-like (yeast)(SACM1L)	Homo sapiens
4200	11755391_	turquoise	H2A histone family member Y(H2AFY)	Homo sapiens
4201	11755405_	turquoise	RUN and cysteine rich domain containing beclin 1 interacting protein like(RUBCNL)	Homo sapiens
4202	11755411_	turquoise	inhibitor of kappa light polypeptide gene enhancer in B-cells, kinase beta(IKBKB)	Homo sapiens
4203	11755412_	turquoise	inhibitor of kappa light polypeptide gene enhancer in B-cells, kinase beta(IKBKB)	Homo sapiens
4204	11755417_	turquoise	KIAA0922(KIAA0922)	Homo sapiens
4205	11755422_	turquoise	CD6 molecule(CD6)	Homo sapiens
4206	11755425_	blue	family with sequence similarity 179 member B(FAM179B)	Homo sapiens
4207	11755426_	turquoise	Sec61 translocon alpha 1 subunit(SEC61A1)	Homo sapiens
4208	11755433_	turquoise	uncharacterized LOC100505585(LOC100505585)	Homo sapiens
4209	11755443_	turquoise	monoamine oxidase A(MAOA)	Homo sapiens
4210	11755458_	brown	HD domain containing 2(HDDC2)	Homo sapiens
4211	11755469_	turquoise	minichromosome maintenance complex component 7(MCM7)	Homo sapiens
4212	11755474_	turquoise	ADAM metallopeptidase domain 15(ADAM15)	Homo sapiens
4213	11755476_	turquoise	nicotinamide nucleotide adenylyltransferase 3(NMNAT3)	Homo sapiens
4214	11755481_	turquoise	DNA helicase B(HELB)	Homo sapiens
4215	11755482_	turquoise	G protein-coupled receptor 132(GPR132)	Homo sapiens
4216	11755483_	turquoise	protein phosphatase 1 regulatory subunit 21(PPP1R21)	Homo sapiens
4217	11755522_	turquoise	leupaxin(LPXN)	Homo sapiens
4218	11755523_	turquoise	RNA binding motif protein 47(RBM47)	Homo sapiens
4219	11755533_	turquoise	adhesion G protein-coupled receptor A3(ADGRA3)	Homo sapiens
4220	11755546_	turquoise	LFNG O-fucosylpeptide 3-beta-N-acetylglucosaminyltransferase(LFNG)	Homo sapiens
4221	11755547_	turquoise	serine/threonine kinase 33(STK33)	Homo sapiens
4222	11755549_	turquoise	semaphorin 6D(SEMA6D)	Homo sapiens
4223	11755556_	turquoise	exocyst complex component 2(EXOC2)	Homo sapiens
4224	11755564_	turquoise	C-C motif chemokine ligand 3 like 3(CCL3L3)	Homo sapiens
4225	11755577_	turquoise	meiotic double-stranded break formation protein 1(MEI1)	Homo sapiens
4226	11755587_	turquoise	tripartite motif containing 22(TRIM22)	Homo sapiens
4227	11755606_	turquoise	IFI30, lysosomal thiol reductase(IFI30)	Homo sapiens
4228	11755615_	turquoise	Janus kinase and microtubule interacting protein 3(JAKMIP3)	Homo sapiens
4229	11755616_	turquoise	solute carrier family 8 member B1(SLC8B1)	Homo sapiens
4230	11755622_	turquoise	roundabout guidance receptor 4(ROBO4)	Homo sapiens
4231	11755631_	turquoise	required for meiotic nuclear division 1 homolog(RMND1)	Homo sapiens
4232	11755641_	turquoise	post-GPI attachment to proteins 1(PGAP1)	Homo sapiens
4233	11755646_	turquoise	sialic acid binding Ig like lectin 1(SIGLEC1)	Homo sapiens
4234	11755671_	turquoise	synaptotagmin like 2(SYTL2)	Homo sapiens
4235	11755676_	turquoise	interaction protein for cytohesin exchange factors 1(IPCEF1)	Homo sapiens
4236	11755703_	turquoise	zinc finger protein 287(ZNF287)	Homo sapiens
4237	11755705_	turquoise	small integral membrane protein 5(SMIM5)	Homo sapiens
4238	11755709_	turquoise	La ribonucleoprotein domain family member 6(LARP6)	Homo sapiens
4239	11755710_	brown	GTP binding protein 8 (putative)(GTPBP8)	Homo sapiens
4240	11755717_	turquoise	docking protein 2(DOK2)	Homo sapiens
4241	11755718_	turquoise	histone deacetylase 1(HDAC1)	Homo sapiens
4242	11755747_	turquoise	placenta specific 8(PLAC8)	Homo sapiens
4243	11755748_	turquoise	placenta specific 8(PLAC8)	Homo sapiens
4244	11755755_	turquoise	integrator complex subunit 6 like(INTS6L)	Homo sapiens
4245	11755758_	turquoise	NLR family CARD domain containing 5(NLRC5)	Homo sapiens
4246	11755762_	turquoise	transmembrane protein 2(TMEM2)	Homo sapiens
4247	11755766_	turquoise	sorting nexin family member 30(SNX30)	Homo sapiens
4248	11755778_	turquoise	roundabout guidance receptor 4(ROBO4)	Homo sapiens

4249	11755788_	blue	kinesin family member 21A(KIF21A)	Homo sapiens
4250	11755811_	turquoise	zinc finger protein 266(ZNF266)	Homo sapiens
4251	11755818_	turquoise	FYVE and coiled-coil domain containing 1(FYCO1)	Homo sapiens
4252	11755819_	turquoise	DExD/H-box helicase 58(DDX58)	Homo sapiens
4253	11755830_	turquoise	myosin IG(MYO1G)	Homo sapiens
4254	11755858_	turquoise	membrane spanning 4-domains A1(MS4A1)	Homo sapiens
4255	11755874_	turquoise	bora, aurora kinase A activator(BORA)	Homo sapiens
4256	11755876_	brown	interferon related developmental regulator 2(IFRD2)	Homo sapiens
4257	11755882_	turquoise	solute carrier family 35 member B3(SLC35B3)	Homo sapiens
4258	11755888_	turquoise	regucalcin(RGN)	Homo sapiens
4259	11755902_	turquoise	transient receptor potential cation channel subfamily V member 2(TRPV2)	Homo sapiens
4260	11755903_	turquoise	NEDD4 binding protein 2 like 1(N4BP2L1)	Homo sapiens
4261	11755908_	turquoise	immunoglobulin superfamily member 3(IGSF3)	Homo sapiens
4262	11755912_	turquoise	YEATS domain containing 2(YEATS2)	Homo sapiens
4263	11755915_	turquoise	HPS3, biogenesis of lysosomal organelles complex 2 subunit 1(HPS3)	Homo sapiens
4264	11755932_	turquoise	hematopoietic cell-specific Lyn substrate 1(HCLS1)	Homo sapiens
4265	11755933_	turquoise	major histocompatibility complex, class I, B(HLA-B)	Homo sapiens
4266	11755943_	turquoise	vimentin(VIM)	Homo sapiens
4267	11755952_	turquoise	arrestin beta 2(ARRB2)	Homo sapiens
4268	11755960_	blue	leucine rich repeat containing 39(LRRC39)	Homo sapiens
4269	11755966_	turquoise	major histocompatibility complex, class I, B(HLA-B)	Homo sapiens
4270	11755980_	blue	solute carrier family 25 member 3(SLC25A3)	Homo sapiens
4271	11755990_	turquoise	calpain 12(CAPN12)	Homo sapiens
4272	11755997_	turquoise	sterile alpha motif domain containing 9 like(SAMD9L)	Homo sapiens
4273	11756006_	turquoise	LCK proto-oncogene, Src family tyrosine kinase(LCK)	Homo sapiens
4274	11756011_	turquoise	protein tyrosine phosphatase, receptor type J(PTPRJ)	Homo sapiens
4275	11756061_	turquoise	small nucleolar RNA, H/ACA box 64(SNORA64)	Homo sapiens
4276	11756068_	turquoise	beta-site APP-cleaving enzyme 2(BACE2)	Homo sapiens
4277	11756071_	turquoise	actin beta(ACTB)	Homo sapiens
4278	11756073_	turquoise	major histocompatibility complex, class II, DP beta 1(HLA-DPB1)	Homo sapiens
4279	11756077_	turquoise	nudE neurodevelopment protein 1(NDE1)	Homo sapiens
4280	11756083_	turquoise	major histocompatibility complex, class II, DQ alpha 1(HLA-DQA1)	Homo sapiens
4281	11756089_	turquoise	macrophage scavenger receptor 1(MSR1)	Homo sapiens
4282	11756096_	blue	solute carrier family 25 member 4(SLC25A4)	Homo sapiens
4283	11756099_	blue	lactate dehydrogenase B(LDHB)	Homo sapiens
4284	11756109_	turquoise	mannosyl (alpha-1,6-)-glycoprotein beta-1,2-N-acetylglucosaminyltransferase(MGAT2)	Homo sapiens
4285	11756125_	brown	NADH:ubiquinone oxidoreductase subunit A1(NDUFA1)	Homo sapiens
4286	11756138_	turquoise	hexokinase 2(HK2)	Homo sapiens
4287	11756146_	turquoise	S100 calcium binding protein A4(S100A4)	Homo sapiens
4288	11756150_	turquoise	beta-2-microglobulin(B2M)	Homo sapiens
4289	11756151_	turquoise	beta-2-microglobulin(B2M)	Homo sapiens
4290	11756168_	turquoise	RAB13, member RAS oncogene family(RAB13)	Homo sapiens
4291	11756170_	turquoise	chromosome X open reading frame 38(CXorf38)	Homo sapiens
4292	11756175_	turquoise	NSE4 homolog A, SMC5-SMC6 complex component(NSMCE4A)	Homo sapiens
4293	11756176_	turquoise	eukaryotic translation initiation factor 4A3(EIF4A3)	Homo sapiens
4294	11756177_	turquoise	CKLF like MARVEL transmembrane domain containing 6(CMTM6)	Homo sapiens
4295	11756178_	turquoise	CKLF like MARVEL transmembrane domain containing 6(CMTM6)	Homo sapiens
4296	11756181_	turquoise	tyrosine 3-monooxygenase/tryptophan 5-monooxygenase activation protein zeta(YWH/	Homo sapiens
4297	11756183_	blue	solute carrier family 25 member 11(SLC25A11)	Homo sapiens
4298	11756211_	brown	Hikeshi, heat shock protein nuclear import factor(HIKESHI)	Homo sapiens

4299	11756223_turquoise	DExD-box helicase 21(DDX21)	Homo sapiens
4300	11756225_turquoise	DnaJ heat shock protein family (Hsp40) member B6(DNAJB6)	Homo sapiens
4301	11756226_turquoise	GRIP1 associated protein 1(GRIPAP1)	Homo sapiens
4302	11756228_turquoise	NECAP endocytosis associated 2(NECAP2)	Homo sapiens
4303	11756232_blue	voltage dependent anion channel 2(VDAC2)	Homo sapiens
4304	11756239_blue	mitochondrial ribosomal protein S35(MRPS35)	Homo sapiens
4305	11756243_turquoise	dual adaptor of phosphotyrosine and 3-phosphoinositides 1(DAPP1)	Homo sapiens
4306	11756245_turquoise	annexin A5(ANXA5)	Homo sapiens
4307	11756255_turquoise	tyrosine 3-monooxygenase/tryptophan 5-monooxygenase activation protein eta(YWHA	Homo sapiens
4308	11756280_turquoise	nudix hydrolase 5(NUDT5)	Homo sapiens
4309	11756281_turquoise	CD7 molecule(CD7)	Homo sapiens
4310	11756283_turquoise	cell division cycle and apoptosis regulator 1(CCAR1)	Homo sapiens
4311	11756287_turquoise	acyloxyacyl hydrolase(AOAH)	Homo sapiens
4312	11756289_turquoise	CCM2 scaffolding protein(CCM2)	Homo sapiens
4313	11756292_turquoise	cathepsin W(CTSW)	Homo sapiens
4314	11756296_blue	mitochondrial fission factor(MFF)	Homo sapiens
4315	11756302_turquoise	CD37 molecule(CD37)	Homo sapiens
4316	11756303_turquoise	SH3 domain and tetratricopeptide repeats 1(SH3TC1)	Homo sapiens
4317	11756306_turquoise	poly(ADP-ribose) polymerase family member 9(PARP9)	Homo sapiens
4318	11756309_turquoise	RAB24, member RAS oncogene family(RAB24)	Homo sapiens
4319	11756317_turquoise	zinc finger protein 276(ZNF276)	Homo sapiens
4320	11756320_turquoise	nudix hydrolase 13(NUDT13)	Homo sapiens
4321	11756322_turquoise	CNDP dipeptidase 2 (metallopeptidase M20 family)(CNDP2)	Homo sapiens
4322	11756327_turquoise	ribosomal protein S19(RPS19)	Homo sapiens
4323	11756330_turquoise	DExD/H-box helicase 58(DDX58)	Homo sapiens
4324	11756351_brown	superoxide dismutase 1, soluble(SOD1)	Homo sapiens
4325	11756358_turquoise	polo like kinase 3(PLK3)	Homo sapiens
4326	11756362_turquoise	Fc fragment of IgG receptor Ic, pseudogene(FCGR1CP)	Homo sapiens
4327	11756363_turquoise	NCK associated protein 1 like(NCKAP1L)	Homo sapiens
4328	11756367_blue	solute carrier family 25 member 4(SLC25A4)	Homo sapiens
4329	11756369_turquoise	mitochondrial trans-2-enoyl-CoA reductase(MECR)	Homo sapiens
4330	11756375_grey	ETS variant 7(ETV7)	Homo sapiens
4331	11756386_turquoise	WD repeat domain 1(WDR1)	Homo sapiens
4332	11756387_turquoise	ADP ribosylation factor like GTPase 4A(ARL4A)	Homo sapiens
4333	11756391_turquoise	microtubule interacting and trafficking domain containing 1(MITD1)	Homo sapiens
4334	11756393_turquoise	coronin 1A(CORO1A)	Homo sapiens
4335	11756401_turquoise	family with sequence similarity 49 member B(FAM49B)	Homo sapiens
4336	11756411_turquoise	stabilin 1(STAB1)	Homo sapiens
4337	11756417_turquoise	kizuna centrosomal protein(KIZ)	Homo sapiens
4338	11756429_turquoise	oligosaccharyltransferase complex non-catalytic subunit(OSTC)	Homo sapiens
4339	11756434_turquoise	DNA methyltransferase 1(DNMT1)	Homo sapiens
4340	11756449_turquoise	RANBP2-type and C3HC4-type zinc finger containing 1(RBCK1)	Homo sapiens
4341	11756453_turquoise	SH2 domain containing 3C(SH2D3C)	Homo sapiens
4342	11756467_turquoise	transmembrane protein 50A(TMEM50A)	Homo sapiens
4343	11756479_turquoise	ribonuclease T2(RNASET2)	Homo sapiens
4344	11756485_turquoise	taxilin alpha(TXLNA)	Homo sapiens
4345	11756499_blue	kelch domain containing 2(KLHDC2)	Homo sapiens
4346	11756500_brown	diphthamide biosynthesis 5(DPH5)	Homo sapiens
4347	11756501_blue	transmembrane protein 38B(TMEM38B)	Homo sapiens
4348	11756512_turquoise	zinc fingers and homeoboxes 3(ZHX3)	Homo sapiens

4349	11756523_	turquoise	shisa family member 5(SHISA5)	Homo sapiens
4350	11756525_	turquoise	IDI2 antisense RNA 1(IDI2-AS1)	Homo sapiens
4351	11756526_	brown	mitochondrial ribosomal protein L48(MRPL48)	Homo sapiens
4352	11756532_	turquoise	phosphatidylinositol-4,5-bisphosphate 3-kinase catalytic subunit delta(PIK3CD)	Homo sapiens
4353	11756535_	turquoise	solute carrier family 25 member 45(SLC25A45)	Homo sapiens
4354	11756543_	turquoise	nuclear RNA export factor 1(NXF1)	Homo sapiens
4355	11756564_	turquoise	ancient ubiquitous protein 1(AUP1)	Homo sapiens
4356	11756572_	turquoise	interferon regulatory factor 3(IRF3)	Homo sapiens
4357	11756581_	turquoise	protease, serine 23(PRSS23)	Homo sapiens
4358	11756593_	blue	uroporphyrinogen decarboxylase(UROD)	Homo sapiens
4359	11756603_	turquoise	family with sequence similarity 206 member A(FAM206A)	Homo sapiens
4360	11756613_	turquoise	semaphorin 6A(SEMA6A)	Homo sapiens
4361	11756617_	turquoise	TAP binding protein(TAPBP)	Homo sapiens
4362	11756618_	turquoise	TAP binding protein(TAPBP)	Homo sapiens
4363	11756626_	turquoise	ZW10 interacting kinetochore protein(ZWINT)	Homo sapiens
4364	11756632_	turquoise	granulysin(GNLY)	Homo sapiens
4365	11756636_	turquoise	PC-esterase domain containing 1A(PCED1A)	Homo sapiens
4366	11756641_	turquoise	zinc finger protein 256(ZNF256)	Homo sapiens
4367	11756645_	turquoise	C-X-C motif chemokine ligand 16(CXCL16)	Homo sapiens
4368	11756652_	brown	N-terminal Xaa-Pro-Lys N-methyltransferase 1(NTMT1)	Homo sapiens
4369	11756655_	turquoise	malonyl-CoA-acyl carrier protein transacylase(MCAT)	Homo sapiens
4370	11756658_	yellow	marginal zone B and B1 cell specific protein(MZB1)	Homo sapiens
4371	11756668_	turquoise	family with sequence similarity 213 member A(FAM213A)	Homo sapiens
4372	11756683_	turquoise	CD1e molecule(CD1E)	Homo sapiens
4373	11756687_	brown	mitochondrial ribosomal protein L46(MRPL46)	Homo sapiens
4374	11756694_	turquoise	ring finger protein 44(RNF44)	Homo sapiens
4375	11756702_	turquoise	cytochrome P450 family 39 subfamily A member 1(CYP39A1)	Homo sapiens
4376	11756709_	turquoise	Bruton tyrosine kinase(BTK)	Homo sapiens
4377	11756712_	turquoise	acid phosphatase 2, lysosomal(ACP2)	Homo sapiens
4378	11756722_	blue	stomatin like 2(STOML2)	Homo sapiens
4379	11756725_	turquoise	shisa family member 5(SHISA5)	Homo sapiens
4380	11756741_	turquoise	SH3 domain binding glutamate rich protein like(SH3BGRL)	Homo sapiens
4381	11756746_	turquoise	A-kinase interacting protein 1(AKIP1)	Homo sapiens
4382	11756757_	brown	short chain dehydrogenase/reductase family 39U member 1(SDR39U1)	Homo sapiens
4383	11756765_	turquoise	BCL2 like 13(BCL2L13)	Homo sapiens
4384	11756766_	turquoise	CD74 molecule(CD74)	Homo sapiens
4385	11756767_	turquoise	primary cilia formation(PIFO)	Homo sapiens
4386	11756780_	turquoise	membrane spanning 4-domains A7(MS4A7)	Homo sapiens
4387	11756786_	turquoise	solute carrier family 38 member 9(SLC38A9)	Homo sapiens
4388	11756787_	turquoise	hyaluronan and proteoglycan link protein 3(HAPLN3)	Homo sapiens
4389	11756792_	turquoise	microRNA 6837(MIR6837)	Homo sapiens
4390	11756793_	turquoise	vanin 2(VNN2)	Homo sapiens
4391	11756806_	turquoise	interferon stimulated exonuclease gene 20(ISG20)	Homo sapiens
4392	11756807_	turquoise	receptor interacting serine/threonine kinase 1(RIPK1)	Homo sapiens
4393	11756809_	turquoise	EF-hand domain containing 2(EFHC2)	Homo sapiens
4394	11756818_	turquoise	PAT1 homolog 2(PATL2)	Homo sapiens
4395	11756822_	turquoise	lymphatic vessel endothelial hyaluronan receptor 1(LYVE1)	Homo sapiens
4396	11756827_	turquoise	reticulon 1(RTN1)	Homo sapiens
4397	11756839_	turquoise	zinc finger CCCH-type containing, antiviral 1(ZC3HAV1)	Homo sapiens
4398	11756847_	turquoise	C-X3-C motif chemokine receptor 1(CX3CR1)	Homo sapiens

4399	11756854_grey	EH domain binding protein 1(EHBP1)	Homo sapiens
4400	11756867_turquoise	tumor necrosis factor superfamily member 13b(TNFSF13B)	Homo sapiens
4401	11756869_turquoise	PDZ domain containing 2(PDZD2)	Homo sapiens
4402	11756871_turquoise	sorting nexin 10(SNX10)	Homo sapiens
4403	11756883_turquoise	proline rich coiled-coil 2C(PRRC2C)	Homo sapiens
4404	11756892_turquoise	C2 calcium dependent domain containing 2(C2CD2)	Homo sapiens
4405	11756913_turquoise	nucleotide binding oligomerization domain containing 1(NOD1)	Homo sapiens
4406	11756924_turquoise	ATPase H+ transporting V1 subunit A(ATP6V1A)	Homo sapiens
4407	11756946_turquoise	TBC1 domain family member 10C(TBC1D10C)	Homo sapiens
4408	11756952_turquoise	transketolase(TKT)	Homo sapiens
4409	11756959_turquoise	protein kinase D2(PRKD2)	Homo sapiens
4410	11756976_turquoise	LON peptidase N-terminal domain and ring finger 2(LONRF2)	Homo sapiens
4411	11756977_turquoise	phytanoyl-CoA dioxygenase domain containing 1(PHYHD1)	Homo sapiens
4412	11756979_turquoise	tubulin alpha 1b(TUBA1B)	Homo sapiens
4413	11756984_turquoise	G protein subunit beta 2(GNB2)	Homo sapiens
4414	11756994_turquoise	G protein subunit beta 2(GNB2)	Homo sapiens
4415	11756999_turquoise	cytochrome b561 family member D2(CYB561D2)	Homo sapiens
4416	11757011_blue	SPRY domain containing 7(SPRYD7)	Homo sapiens
4417	11757022_turquoise	tissue factor pathway inhibitor 2(TFPI2)	Homo sapiens
4418	11757030_blue	enoyl-CoA hydratase 1(ECH1)	Homo sapiens
4419	11757035_turquoise	atypical chemokine receptor 1 (Duffy blood group)(ACKR1)	Homo sapiens
4420	11757037_turquoise	microRNA 4738(MIR4738)	Homo sapiens
4421	11757038_turquoise	microRNA 4738(MIR4738)	Homo sapiens
4422	11757049_turquoise	CTD phosphatase subunit 1(CTDP1)	Homo sapiens
4423	11757050_turquoise	transgelin 2(TAGLN2)	Homo sapiens
4424	11757056_turquoise	microRNA 1244-1(MIR1244-1)	Homo sapiens
4425	11757059_turquoise	RPL36A-HNRNPH2 readthrough(RPL36A-HNRNPH2)	Homo sapiens
4426	11757073_brown	THAP domain containing 4(THAP4)	Homo sapiens
4427	11757083_brown	coiled-coil-helix-coiled-coil-helix domain containing 3(CHCHD3)	Homo sapiens
4428	11757085_turquoise	N-acylsphingosine amidohydrolase 2(ASAH2)	Homo sapiens
4429	11757141_turquoise	microRNA 1304(MIR1304)	Homo sapiens
4430	11757156_turquoise	small nucleolar RNA, H/ACA box 44(SNORA44)	Homo sapiens
4431	11757164_turquoise	microRNA 1304(MIR1304)	Homo sapiens
4432	11757180_turquoise	casein kinase 1 gamma 2(CSNK1G2)	Homo sapiens
4433	11757186_turquoise	hexokinase 3(HK3)	Homo sapiens
4434	11757190_turquoise	lipopolysaccharide induced TNF factor(LITAF)	Homo sapiens
4435	11757194_turquoise	leishmanolysin like peptidase(LMLN)	Homo sapiens
4436	11757213_turquoise	uncharacterized FLJ34503(FLJ34503)	Homo sapiens
4437	11757259_turquoise	small Cajal body-specific RNA 9-like(SCARNA9L)	Homo sapiens
4438	11757265_brown	eukaryotic translation initiation factor 3 subunit K(EIF3K)	Homo sapiens
4439	11757267_brown	sirtuin 2(SIRT2)	Homo sapiens
4440	11757269_turquoise	ferritin light chain(FTL)	Homo sapiens
4441	11757271_turquoise	sorting nexin 6(SNX6)	Homo sapiens
4442	11757274_turquoise	arginine and glutamate rich 1(ARGLU1)	Homo sapiens
4443	11757275_turquoise	arginine and glutamate rich 1(ARGLU1)	Homo sapiens
4444	11757279_brown	translocase of inner mitochondrial membrane domain containing 1(TIMMDC1)	Homo sapiens
4445	11757281_brown	ATP synthase, H+ transporting, mitochondrial Fo complex subunit C3 (subunit 9)(ATP5G	Homo sapiens
4446	11757282_brown	ATP synthase, H+ transporting, mitochondrial Fo complex subunit C3 (subunit 9)(ATP5G	Homo sapiens
4447	11757284_turquoise	myeloid derived growth factor(MYDGF)	Homo sapiens
4448	11757289_turquoise	clathrin light chain A(CLTA)	Homo sapiens

4449	11757291_ turquoise	iron-sulfur cluster assembly enzyme(ISCU)	Homo sapiens
4450	11757300_ turquoise	ELOVL fatty acid elongase 5(ELOVL5)	Homo sapiens
4451	11757321_ turquoise	TAP binding protein(TAPBP)	Homo sapiens
4452	11757326_ turquoise	ferritin light chain(FTL)	Homo sapiens
4453	11757327_ turquoise	ferritin light chain(FTL)	Homo sapiens
4454	11757330_ brown	small nuclear ribonucleoprotein polypeptide C(SNRPC)	Homo sapiens
4455	11757332_ turquoise	microRNA 3064(MIR3064)	Homo sapiens
4456	11757334_ brown	ubiquinol-cytochrome c reductase, complex III subunit XI(UQCR11)	Homo sapiens
4457	11757335_ turquoise	mitochondrial translational initiation factor 3(MTIF3)	Homo sapiens
4458	11757337_ turquoise	microRNA 6734(MIR6734)	Homo sapiens
4459	11757338_ turquoise	glyoxylate and hydroxypyruvate reductase(GRHPR)	Homo sapiens
4460	11757342_ turquoise	dCMP deaminase(DCTD)	Homo sapiens
4461	11757344_ turquoise	small nuclear ribonucleoprotein polypeptides B and B1(SNRPB)	Homo sapiens
4462	11757346_ turquoise	ferritin light chain(FTL)	Homo sapiens
4463	11757351_ turquoise	transporter 1, ATP binding cassette subfamily B member(TAP1)	Homo sapiens
4464	11757367_ turquoise	heat shock protein family A (Hsp70) member 6(HSPA6)	Homo sapiens
4465	11757368_ turquoise	annexin A4(ANXA4)	Homo sapiens
4466	11757369_ turquoise	mannosidase alpha class 2B member 1(MAN2B1)	Homo sapiens
4467	11757373_ turquoise	apolipoprotein C1(APOC1)	Homo sapiens
4468	11757379_ turquoise	tyrosine kinase 2(TYK2)	Homo sapiens
4469	11757383_ turquoise	cathepsin H(CTSH)	Homo sapiens
4470	11757404_ turquoise	inhibitor of DNA binding 3, HLH protein(ID3)	Homo sapiens
4471	11757405_ brown	methionine sulfoxide reductase B2(MSRB2)	Homo sapiens
4472	11757409_ turquoise	thymosin beta 10(TMSB10)	Homo sapiens
4473	11757413_ brown	NADH:ubiquinone oxidoreductase core subunit S3(NDUFS3)	Homo sapiens
4474	11757415_ turquoise	solute carrier family 5 member 3(SLC5A3)	Homo sapiens
4475	11757420_ turquoise	ZFP36 ring finger protein like 2(ZFP36L2)	Homo sapiens
4476	11757425_ turquoise	vascular cell adhesion molecule 1(VCAM1)	Homo sapiens
4477	11757429_ brown	cytochrome c1(CYC1)	Homo sapiens
4478	11757435_ turquoise	bridging integrator 1(BIN1)	Homo sapiens
4479	11757436_ turquoise	interferon induced transmembrane protein 2(IFITM2)	Homo sapiens
4480	11757438_ turquoise	destrin, actin depolymerizing factor(DSTN)	Homo sapiens
4481	11757439_ turquoise	GDP dissociation inhibitor 2(GDI2)	Homo sapiens
4482	11757443_ blue	translocase of inner mitochondrial membrane 21(TIMM21)	Homo sapiens
4483	11757446_ brown	MINOS1-NBL1 readthrough(MINOS1-NBL1)	Homo sapiens
4484	11757454_ turquoise	cAMP responsive element binding protein like 2(CREBL2)	Homo sapiens
4485	11757455_ turquoise	glutathione peroxidase 1(GPX1)	Homo sapiens
4486	11757459_ turquoise	WAS/WASL interacting protein family member 1(WIPF1)	Homo sapiens
4487	11757464_ turquoise	plexin B2(PLXNB2)	Homo sapiens
4488	11757465_ blue	glioblastoma amplified sequence(GBAS)	Homo sapiens
4489	11757469_ turquoise	tripeptidyl peptidase 1(TPP1)	Homo sapiens
4490	11757470_ turquoise	transcription factor 25(TCF25)	Homo sapiens
4491	11757474_ turquoise	chloride intracellular channel 1(CLIC1)	Homo sapiens
4492	11757477_ turquoise	LIM and SH3 protein 1(LASP1)	Homo sapiens
4493	11757480_ turquoise	interferon alpha inducible protein 27(IFI27)	Homo sapiens
4494	11757483_ turquoise	von Willebrand factor A domain containing 1(VWA1)	Homo sapiens
4495	11757485_ brown	family with sequence similarity 129 member B(FAM129B)	Homo sapiens
4496	11757511_ turquoise	major histocompatibility complex, class II, DP alpha 1(HLA-DPA1)	Homo sapiens
4497	11757513_ turquoise	NFKB inhibitor zeta(NFKBIZ)	Homo sapiens
4498	11757519_ turquoise	capping actin protein, gelsolin like(CAPG)	Homo sapiens

4499	11757524_blue	cytochrome c1(CYC1)	Homo sapiens
4500	11757529_turquoise	lysosomal associated membrane protein 1(LAMP1)	Homo sapiens
4501	11757532_turquoise	RNA binding motif protein 47(RBM47)	Homo sapiens
4502	11757533_turquoise	phospholipid scramblase 1(PLSCR1)	Homo sapiens
4503	11757538_blue	glutamic-oxaloacetic transaminase 1(GOT1)	Homo sapiens
4504	11757539_turquoise	stromal interaction molecule 2(STIM2)	Homo sapiens
4505	11757544_turquoise	intercellular adhesion molecule 2(ICAM2)	Homo sapiens
4506	11757545_turquoise	platelet and endothelial cell adhesion molecule 1(PECAM1)	Homo sapiens
4507	11757546_turquoise	MICAL like 2(MICALL2)	Homo sapiens
4508	11757552_turquoise	actin related protein 2/3 complex subunit 3(ARPC3)	Homo sapiens
4509	11757554_turquoise	EGF like domain multiple 8(EGFL8)	Homo sapiens
4510	11757556_turquoise	bleomycin hydrolase(BLMH)	Homo sapiens
4511	11757563_turquoise	protein phosphatase 1 regulatory subunit 35(PPP1R35)	Homo sapiens
4512	11757566_turquoise	nucleolar protein 8(NOL8)	Homo sapiens
4513	11757567_brown	NADH:ubiquinone oxidoreductase subunit B5(NDUFB5)	Homo sapiens
4514	11757574_turquoise	transmembrane protein 123(TMEM123)	Homo sapiens
4515	11757588_turquoise	ALG5, dolichyl-phosphate beta-glucosyltransferase(ALG5)	Homo sapiens
4516	11757589_brown	NADH:ubiquinone oxidoreductase subunit A12(NDUFA12)	Homo sapiens
4517	11757590_turquoise	ISY1-RAB43 readthrough(ISY1-RAB43)	Homo sapiens
4518	11757591_turquoise	PAN3 poly(A) specific ribonuclease subunit(PAN3)	Homo sapiens
4519	11757601_brown	COP9 signalosome subunit 3(COPS3)	Homo sapiens
4520	11757602_turquoise	endoplasmic reticulum protein 29(ERP29)	Homo sapiens
4521	11757604_turquoise	SAMM50 sorting and assembly machinery component(SAMM50)	Homo sapiens
4522	11757613_brown	eukaryotic translation initiation factor 3 subunit K(EIF3K)	Homo sapiens
4523	11757624_turquoise	poly(ADP-ribose) polymerase family member 12(PARP12)	Homo sapiens
4524	11757625_turquoise	CD200 molecule(CD200)	Homo sapiens
4525	11757630_turquoise	HERPUD family member 2(HERPUD2)	Homo sapiens
4526	11757632_turquoise	LIM domain kinase 2(LIMK2)	Homo sapiens
4527	11757635_turquoise	TYRO protein tyrosine kinase binding protein(TYROBP)	Homo sapiens
4528	11757638_turquoise	CD93 molecule(CD93)	Homo sapiens
4529	11757649_brown	CORO7-PAM16 readthrough(CORO7-PAM16)	Homo sapiens
4530	11757652_turquoise	prolyl 3-hydroxylase 1(P3H1)	Homo sapiens
4531	11757653_turquoise	CDK5 regulatory subunit associated protein 3(CDK5RAP3)	Homo sapiens
4532	11757654_turquoise	uncoupling protein 2(UCP2)	Homo sapiens
4533	11757661_turquoise	small nuclear ribonucleoprotein polypeptides B and B1(SNRPB)	Homo sapiens
4534	11757669_turquoise	peptidylprolyl isomerase like 1(PPIL1)	Homo sapiens
4535	11757670_brown	ubiquinol-cytochrome c reductase, complex III subunit X(UQCR10)	Homo sapiens
4536	11757672_brown	mitochondrial ribosomal protein S17(MRPS17)	Homo sapiens
4537	11757692_turquoise	APC, WNT signaling pathway regulator(APC)	Homo sapiens
4538	11757713_turquoise	ataxin 2 like(ATXN2L)	Homo sapiens
4539	11757722_turquoise	septin 8(SEPT8)	Homo sapiens
4540	11757727_brown	ATP synthase, H+ transporting, mitochondrial F1 complex, O subunit(ATP5O)	Homo sapiens
4541	11757728_turquoise	ferritin light chain(FTL)	Homo sapiens
4542	11757731_turquoise	RAB31, member RAS oncogene family(RAB31)	Homo sapiens
4543	11757732_turquoise	G protein subunit alpha i2(GNAI2)	Homo sapiens
4544	11757737_turquoise	recombination signal binding protein for immunoglobulin kappa J region(RBPJ)	Homo sapiens
4545	11757756_turquoise	additional sex combs like 2, transcriptional regulator(ASXL2)	Homo sapiens
4546	11757765_turquoise	sarcoglycan delta(SGCD)	Homo sapiens
4547	11757787_turquoise	ferritin light chain(FTL)	Homo sapiens
4548	11757798_turquoise	MAF bZIP transcription factor B(MAFB)	Homo sapiens

4549	11757801_ turquoise	major histocompatibility complex, class II, DP beta 1(HLA-DPB1)	Homo sapiens
4550	11757806_ turquoise	peroxiredoxin 6(PRDX6)	Homo sapiens
4551	11757807_ turquoise	actin beta(ACTB)	Homo sapiens
4552	11757816_ turquoise	myristoylated alanine rich protein kinase C substrate(MARCKS)	Homo sapiens
4553	11757817_ turquoise	brain abundant membrane attached signal protein 1(BASP1)	Homo sapiens
4554	11757818_ turquoise	cofilin 1(CFL1)	Homo sapiens
4555	11757825_ brown	C7orf55-LUC7L2 readthrough(C7orf55-LUC7L2)	Homo sapiens
4556	11757831_ turquoise	synaptosome associated protein 23(SNAP23)	Homo sapiens
4557	11757833_ turquoise	RAB31, member RAS oncogene family(RAB31)	Homo sapiens
4558	11757839_ turquoise	NECAP endocytosis associated 2(NECAP2)	Homo sapiens
4559	11757847_ brown	ubiquinol-cytochrome c reductase, Rieske iron-sulfur polypeptide 1(UQCRFS1)	Homo sapiens
4560	11757863_ brown	transmembrane protein 261(TMEM261)	Homo sapiens
4561	11757867_ turquoise	basic leucine zipper and W2 domains 1(BZW1)	Homo sapiens
4562	11757872_ turquoise	phospholipid scramblase 1(PLSCR1)	Homo sapiens
4563	11757873_ blue	ATP synthase, H+ transporting, mitochondrial F1 complex, beta polypeptide(ATP5B)	Homo sapiens
4564	11757875_ turquoise	Lck interacting transmembrane adaptor 1(LIME1)	Homo sapiens
4565	11757879_ turquoise	microRNA 6837(MIR6837)	Homo sapiens
4566	11757883_ turquoise	ring finger protein 4(RNF4)	Homo sapiens
4567	11757887_ turquoise	tyrosine 3-monooxygenase/tryptophan 5-monooxygenase activation protein beta(YWH	Homo sapiens
4568	11757890_ turquoise	mitochondrial transcription termination factor 2(MTERF2)	Homo sapiens
4569	11757894_ turquoise	NFKB inhibitor alpha(NFKBIA)	Homo sapiens
4570	11757896_ turquoise	arginine and serine rich protein 1(RSRP1)	Homo sapiens
4571	11757904_ turquoise	microtubule associated serine/threonine kinase 3(MAST3)	Homo sapiens
4572	11757908_ turquoise	ral guanine nucleotide dissociation stimulator(RALGDS)	Homo sapiens
4573	11757917_ turquoise	CCR4-NOT transcription complex subunit 6 like(CNOT6L)	Homo sapiens
4574	11757920_ turquoise	zyg-11 family member B, cell cycle regulator(ZYG11B)	Homo sapiens
4575	11757931_ turquoise	KIAA0930(KIAA0930)	Homo sapiens
4576	11757934_ turquoise	KIAA1551(KIAA1551)	Homo sapiens
4577	11757935_ turquoise	KIAA1551(KIAA1551)	Homo sapiens
4578	11757936_ brown	glycine cleavage system protein H pseudogene 3(GCSHP3)	Homo sapiens
4579	11757938_ turquoise	N-myc and STAT interactor(NMI)	Homo sapiens
4580	11757940_ turquoise	placental growth factor(PGF)	Homo sapiens
4581	11757942_ turquoise	utrophin(UTRN)	Homo sapiens
4582	11757950_ turquoise	chondroitin sulfate synthase 1(CHSY1)	Homo sapiens
4583	11757953_ turquoise	tetratricopeptide repeat domain 39C(TTC39C)	Homo sapiens
4584	11757957_ turquoise	SRY-box 6(SOX6)	Homo sapiens
4585	11757960_ turquoise	ArfGAP with RhoGAP domain, ankyrin repeat and PH domain 2(ARAP2)	Homo sapiens
4586	11757966_ turquoise	zinc finger protein 75D(ZNF75D)	Homo sapiens
4587	11757983_ turquoise	translocation associated membrane protein 1(TRAM1)	Homo sapiens
4588	11757986_ turquoise	MIS12, kinetochore complex component(MIS12)	Homo sapiens
4589	11757989_ turquoise	ankyrin repeat domain 46(ANKRD46)	Homo sapiens
4590	11758007_ turquoise	YY1 associated factor 2(YAF2)	Homo sapiens
4591	11758008_ turquoise	patatin like phospholipase domain containing 4(PNPLA4)	Homo sapiens
4592	11758009_ turquoise	uncharacterized LOC100130460(CAND1.11)	Homo sapiens
4593	11758011_ turquoise	eukaryotic translation elongation factor 1 alpha 1(EEF1A1)	Homo sapiens
4594	11758014_ turquoise	transforming growth factor beta receptor 1(TGFBR1)	Homo sapiens
4595	11758017_ turquoise	peptidyl-prolyl cis-trans isomerase A pseudogene(LOC101060363)	Homo sapiens
4596	11758025_ turquoise	inositol polyphosphate-5-phosphatase F(INPP5F)	Homo sapiens
4597	11758037_ turquoise	myotubularin related protein 12(MTMR12)	Homo sapiens
4598	11758042_ turquoise	actin beta(ACTB)	Homo sapiens

4599	11758043_turquoise	immediate early response 2(IER2)	Homo sapiens
4600	11758047_turquoise	major facilitator superfamily domain containing 14A(MFSD14A)	Homo sapiens
4601	11758056_blue	threonyl-tRNA synthetase like 2(TARSL2)	Homo sapiens
4602	11758059_turquoise	ankyrin repeat domain 49(ANKRD49)	Homo sapiens
4603	11758067_turquoise	peptidyl-prolyl cis-trans isomerase A pseudogene(LOC101060363)	Homo sapiens
4604	11758089_turquoise	hyaluronan mediated motility receptor(HMMR)	Homo sapiens
4605	11758090_turquoise	interleukin 16(IL16)	Homo sapiens
4606	11758092_turquoise	ephrin A5(EFNA5)	Homo sapiens
4607	11758094_turquoise	nuclear factor of activated T-cells 2 interacting protein(NFATC2IP)	Homo sapiens
4608	11758101_blue	eukaryotic translation initiation factor 4E binding protein 2(EIF4EBP2)	Homo sapiens
4609	11758113_turquoise	actin related protein 2/3 complex subunit 4(ARPC4)	Homo sapiens
4610	11758114_turquoise	src kinase associated phosphoprotein 2(SKAP2)	Homo sapiens
4611	11758126_turquoise	ELL associated factor 1(EAF1)	Homo sapiens
4612	11758132_blue	trimethyllysine hydroxylase, epsilon(TMLHE)	Homo sapiens
4613	11758135_brown	chromosome 14 open reading frame 2(C14orf2)	Homo sapiens
4614	11758144_turquoise	heterogeneous nuclear ribonucleoprotein C (C1/C2)(HNRNPC)	Homo sapiens
4615	11758148_turquoise	beta-2-microglobulin(B2M)	Homo sapiens
4616	11758149_turquoise	Rac GTPase activating protein 1(RACGAP1)	Homo sapiens
4617	11758158_turquoise	forkhead box P1(FOXP1)	Homo sapiens
4618	11758162_turquoise	ferredoxin 1(FDX1)	Homo sapiens
4619	11758176_turquoise	ATPase phospholipid transporting 10D (putative)(ATP10D)	Homo sapiens
4620	11758178_turquoise	coronin 1B(CORO1B)	Homo sapiens
4621	11758182_turquoise	ferritin light chain(FTL)	Homo sapiens
4622	11758190_blue	succinate dehydrogenase complex subunit D(SDHD)	Homo sapiens
4623	11758191_turquoise	mex-3 RNA binding family member C(MEX3C)	Homo sapiens
4624	11758192_turquoise	beta-2-microglobulin(B2M)	Homo sapiens
4625	11758193_turquoise	beta-2-microglobulin(B2M)	Homo sapiens
4626	11758208_turquoise	Kruppel like factor 2(KLF2)	Homo sapiens
4627	11758210_turquoise	tRNA methyltransferase 10C, mitochondrial RNase P subunit(TRMT10C)	Homo sapiens
4628	11758215_turquoise	transducin like enhancer of split 3(TLE3)	Homo sapiens
4629	11758218_blue	motile sperm domain containing 1(MOSPD1)	Homo sapiens
4630	11758219_turquoise	ribonucleotide reductase regulatory subunit M2(RRM2)	Homo sapiens
4631	11758222_turquoise	solute carrier family 20 member 1(SLC20A1)	Homo sapiens
4632	11758225_turquoise	IQ motif containing GTPase activating protein 2(IQGAP2)	Homo sapiens
4633	11758226_turquoise	ubiquitin conjugating enzyme E2 J1(UBE2J1)	Homo sapiens
4634	11758231_turquoise	major histocompatibility complex, class II, DP alpha 1(HLA-DPA1)	Homo sapiens
4635	11758238_turquoise	ATP binding cassette subfamily C member 4(ABCC4)	Homo sapiens
4636	11758244_blue	A-kinase anchoring protein 6(AKAP6)	Homo sapiens
4637	11758248_blue	succinate dehydrogenase complex subunit D(SDHD)	Homo sapiens
4638	11758259_turquoise	solute carrier organic anion transporter family member 2B1(SLCO2B1)	Homo sapiens
4639	11758260_turquoise	beta-2-microglobulin(B2M)	Homo sapiens
4640	11758261_turquoise	centrosomal protein 55(CEP55)	Homo sapiens
4641	11758263_turquoise	cytochrome b-245 beta chain(CYBB)	Homo sapiens
4642	11758269_turquoise	methionine adenosyltransferase 2B(MAT2B)	Homo sapiens
4643	11758273_turquoise	ADP ribosylation factor 6(ARF6)	Homo sapiens
4644	11758277_turquoise	neuroblastoma breakpoint family member 10(NBPF10)	Homo sapiens
4645	11758279_turquoise	coronin 1C(CORO1C)	Homo sapiens
4646	11758282_turquoise	tubulin alpha 1c(TUBA1C)	Homo sapiens
4647	11758297_turquoise	SHC adaptor protein 1(SHC1)	Homo sapiens
4648	11758299_turquoise	beta-2-microglobulin(B2M)	Homo sapiens

4649	11758300_ turquoise	beta-2-microglobulin(B2M)	Homo sapiens
4650	11758311_ blue	succinate dehydrogenase complex subunit D(SDHD)	Homo sapiens
4651	11758314_ brown	NADH:ubiquinone oxidoreductase complex assembly factor 2(NDUFAF2)	Homo sapiens
4652	11758327_ turquoise	bromodomain adjacent to zinc finger domain 1A(BAZ1A)	Homo sapiens
4653	11758330_ blue	succinate dehydrogenase complex subunit D(SDHD)	Homo sapiens
4654	11758331_ turquoise	uncharacterized LOC101929823(LOC101929823)	Homo sapiens
4655	11758335_ blue	ferredoxin 1(FDX1)	Homo sapiens
4656	11758337_ turquoise	adenosine deaminase(ADA)	Homo sapiens
4657	11758340_ turquoise	major histocompatibility complex, class II, DQ alpha 1(HLA-DQA1)	Homo sapiens
4658	11758343_ brown	up-regulated during skeletal muscle growth 5 homolog (mouse)(USMG5)	Homo sapiens
4659	11758365_ turquoise	tubulin tyrosine ligase like 8(TTLL8)	Homo sapiens
4660	11758369_ turquoise	major histocompatibility complex, class II, DP beta 1(HLA-DPB1)	Homo sapiens
4661	11758377_ turquoise	toll like receptor 1(TLR1)	Homo sapiens
4662	11758385_ turquoise	hes related family bHLH transcription factor with YRPW motif 2(HEY2)	Homo sapiens
4663	11758401_ turquoise	zinc finger protein 148(ZNF148)	Homo sapiens
4664	11758402_ blue	family with sequence similarity 179 member B(FAM179B)	Homo sapiens
4665	11758409_ turquoise	microRNA 6805(MIR6805)	Homo sapiens
4666	11758417_ turquoise	major histocompatibility complex, class II, DP alpha 1(HLA-DPA1)	Homo sapiens
4667	11758418_ turquoise	family with sequence similarity 219 member B(FAM219B)	Homo sapiens
4668	11758419_ turquoise	transmembrane protein 106A(TMEM106A)	Homo sapiens
4669	11758447_ turquoise	nucleoporin 50(NUP50)	Homo sapiens
4670	11758448_ turquoise	TMED7-TICAM2 readthrough(TMED7-TICAM2)	Homo sapiens
4671	11758478_ turquoise	cell division cycle associated 7(CDCA7)	Homo sapiens
4672	11758486_ turquoise	beta-site APP-cleaving enzyme 2(BACE2)	Homo sapiens
4673	11758500_ turquoise	transmembrane protein 140(TMEM140)	Homo sapiens
4674	11758501_ turquoise	nicotinamide nucleotide adenylyltransferase 1(NMNAT1)	Homo sapiens
4675	11758527_ turquoise	inositol 1,4,5-trisphosphate receptor type 2(ITPR2)	Homo sapiens
4676	11758537_ turquoise	tripartite motif containing 25(TRIM25)	Homo sapiens
4677	11758539_ turquoise	dedicator of cytokinesis 10(DOCK10)	Homo sapiens
4678	11758542_ turquoise	zinc finger CCCH-type, RNA binding motif and serine/arginine rich 2(ZRSR2)	Homo sapiens
4679	11758547_ turquoise	echinoderm microtubule associated protein like 1(EML1)	Homo sapiens
4680	11758550_ turquoise	transmembrane protein 268(TMEM268)	Homo sapiens
4681	11758554_ turquoise	ring finger and WD repeat domain 2(RFWD2)	Homo sapiens
4682	11758555_ turquoise	G protein-coupled receptor 183(GPR183)	Homo sapiens
4683	11758557_ turquoise	ZFP36 ring finger protein like 1(ZFP36L1)	Homo sapiens
4684	11758558_ turquoise	ATPase sarcoplasmic/endoplasmic reticulum Ca2+ transporting 2(ATP2A2)	Homo sapiens
4685	11758566_ brown	BCS1 homolog, ubiquinol-cytochrome c reductase complex chaperone(BCS1L)	Homo sapiens
4686	11758595_ turquoise	neurobeachin(NBEA)	Homo sapiens
4687	11758607_ turquoise	von Willebrand factor A domain containing 8(VWA8)	Homo sapiens
4688	11758608_ turquoise	colony stimulating factor 2 receptor beta common subunit(CSF2RB)	Homo sapiens
4689	11758620_ turquoise	methylmalonic aciduria (cobalamin deficiency) cblB type(MMAB)	Homo sapiens
4690	11758630_ turquoise	fibrinogen like 2(FGL2)	Homo sapiens
4691	11758645_ turquoise	eukaryotic translation initiation factor 4E binding protein 2(EIF4EBP2)	Homo sapiens
4692	11758653_ turquoise	adhesion G protein-coupled receptor L4(ADGRL4)	Homo sapiens
4693	11758663_ turquoise	small nucleolar RNA host gene 4(SNHG4)	Homo sapiens
4694	11758667_ turquoise	tyrosine 3-monooxygenase/tryptophan 5-monooxygenase activation protein zeta(YWH/	Homo sapiens
4695	11758671_ turquoise	IKAROS family zinc finger 1(IKZF1)	Homo sapiens
4696	11758679_ turquoise	microRNA 1292(MIR1292)	Homo sapiens
4697	11758683_ turquoise	jade family PHD finger 2(JADE2)	Homo sapiens
4698	11758693_ turquoise	microRNA 7703(MIR7703)	Homo sapiens

4699	11758709_turquoise	retinol dehydrogenase 11 (all-trans/9-cis/11-cis)(RDH11)	Homo sapiens
4700	11758711_turquoise	tyrosine 3-monooxygenase/tryptophan 5-monooxygenase activation protein zeta(YWH/	Homo sapiens
4701	11758716_turquoise	NDUFA4, mitochondrial complex associated(NDUFA4)	Homo sapiens
4702	11758717_brown	NDUFA4, mitochondrial complex associated(NDUFA4)	Homo sapiens
4703	11758727_brown	ATP synthase, H+ transporting, mitochondrial F1 complex, gamma polypeptide 1(ATP5C	Homo sapiens
4704	11758750_turquoise	tyrosine 3-monooxygenase/tryptophan 5-monooxygenase activation protein zeta(YWH/	Homo sapiens
4705	11758753_turquoise	StAR related lipid transfer domain containing 3(STARD3)	Homo sapiens
4706	11758754_turquoise	Fas associated factor 1(FAF1)	Homo sapiens
4707	11758755_blue	mitochondrial pyruvate carrier 2(MPC2)	Homo sapiens
4708	11758757_turquoise	alcohol dehydrogenase 5 (class III), chi polypeptide(ADH5)	Homo sapiens
4709	11758771_turquoise	major histocompatibility complex, class II, DP beta 1(HLA-DPB1)	Homo sapiens
4710	11758772_turquoise	major histocompatibility complex, class II, DP beta 1(HLA-DPB1)	Homo sapiens
4711	11758793_turquoise	mitochondrial calcium uniporter dominant negative beta subunit(MCUB)	Homo sapiens
4712	11758795_brown	mitochondrial ribosomal protein L45(MRPL45)	Homo sapiens
4713	11758821_turquoise	major histocompatibility complex, class II, DQ beta 1(HLA-DQB1)	Homo sapiens
4714	11758831_turquoise	RNA polymerase II subunit C(POLR2C)	Homo sapiens
4715	11758838_turquoise	serine palmitoyltransferase long chain base subunit 2(SPTLC2)	Homo sapiens
4716	11758854_turquoise	atlastin GTPase 3(ATL3)	Homo sapiens
4717	11758862_turquoise	poly(A) binding protein interacting protein 2B(PAIP2B)	Homo sapiens
4718	11758868_turquoise	Rap guanine nucleotide exchange factor 6(RAPGEF6)	Homo sapiens
4719	11758873_turquoise	heparanase(HPSE)	Homo sapiens
4720	11758879_turquoise	major histocompatibility complex, class I, E(HLA-E)	Homo sapiens
4721	11758882_turquoise	CD3d molecule(CD3D)	Homo sapiens
4722	11758897_turquoise	apolipoprotein B mRNA editing enzyme catalytic subunit 3C(APOBEC3C)	Homo sapiens
4723	11758898_turquoise	apolipoprotein B mRNA editing enzyme catalytic subunit 3C(APOBEC3C)	Homo sapiens
4724	11758901_turquoise	receptor interacting serine/threonine kinase 2(RIPK2)	Homo sapiens
4725	11758921_turquoise	mitochondrial ribosomal protein S17(MRPS17)	Homo sapiens
4726	11758922_turquoise	syntaxin binding protein 6(STXBP6)	Homo sapiens
4727	11758933_turquoise	transmembrane 4 L six family member 18(TM4SF18)	Homo sapiens
4728	11758934_turquoise	RAB27A, member RAS oncogene family(RAB27A)	Homo sapiens
4729	11758940_turquoise	MOB kinase activator 1A(MOB1A)	Homo sapiens
4730	11758941_turquoise	MOB kinase activator 1A(MOB1A)	Homo sapiens
4731	11758942_turquoise	MOB kinase activator 1A(MOB1A)	Homo sapiens
4732	11758947_turquoise	copine 4(CPNE4)	Homo sapiens
4733	11758984_turquoise	zinc finger RANBP2-type containing 1(ZRANB1)	Homo sapiens
4734	11758990_turquoise	SLC9A3 regulator 1(SLC9A3R1)	Homo sapiens
4735	11758994_turquoise	fatty acyl-CoA reductase 2(FAR2)	Homo sapiens
4736	11759022_turquoise	ELK3, ETS transcription factor(ELK3)	Homo sapiens
4737	11759049_turquoise	acyl-CoA synthetase short-chain family member 3(ACSS3)	Homo sapiens
4738	11759087_turquoise	NHL repeat containing 2(NHLRC2)	Homo sapiens
4739	11759088_turquoise	membrane associated ring-CH-type finger 1(MARCH1)	Homo sapiens
4740	11759093_turquoise	G protein subunit alpha q(GNAQ)	Homo sapiens
4741	11759094_turquoise	G protein subunit alpha q(GNAQ)	Homo sapiens
4742	11759095_turquoise	G protein subunit alpha q(GNAQ)	Homo sapiens
4743	11759144_turquoise	zinc finger protein 710(ZNF710)	Homo sapiens
4744	11759177_turquoise	mannosidase alpha class 1A member 1(MAN1A1)	Homo sapiens
4745	11759179_blue	transmembrane protein 38B(TMEM38B)	Homo sapiens
4746	11759184_turquoise	plexin C1(PLXNC1)	Homo sapiens
4747	11759187_turquoise	TNF alpha induced protein 8 like 1(TNFAIP8L1)	Homo sapiens
4748	11759288_blue	thioredoxin related transmembrane protein 4(TMX4)	Homo sapiens

4749	11759292_ turquoise	family with sequence similarity 63 member A(FAM63A)	Homo sapiens
4750	11759294_ turquoise	lysine methyltransferase 2A(KMT2A)	Homo sapiens
4751	11759296_ turquoise	mitogen-activated protein kinase kinase kinase 2(MAP3K2)	Homo sapiens
4752	11759300_ turquoise	A-kinase anchoring protein 10(AKAP10)	Homo sapiens
4753	11759320_ turquoise	myelin protein zero like 3(MPZL3)	Homo sapiens
4754	11759328_ turquoise	kinetochore scaffold 1(KNL1)	Homo sapiens
4755	11759347_ turquoise	TXK tyrosine kinase(TXK)	Homo sapiens
4756	11759352_ turquoise	Fc receptor like 3(FCRL3)	Homo sapiens
4757	11759414_ turquoise	glycine cleavage system protein H(GCSH)	Homo sapiens
4758	11759417_ blue	heat shock protein family A (Hsp70) member 9(HSPA9)	Homo sapiens
4759	11759444_ turquoise	BCL2 associated X, apoptosis regulator(BAX)	Homo sapiens
4760	11759477_ turquoise	ADAM metallopeptidase with thrombospondin type 1 motif 15(ADAMTS15)	Homo sapiens
4761	11759496_ turquoise	carbohydrate sulfotransferase 11(CHST11)	Homo sapiens
4762	11759497_ turquoise	carbohydrate sulfotransferase 11(CHST11)	Homo sapiens
4763	11759498_ turquoise	family with sequence similarity 49 member A(FAM49A)	Homo sapiens
4764	11759499_ turquoise	family with sequence similarity 49 member A(FAM49A)	Homo sapiens
4765	11759502_ turquoise	TNF receptor associated factor 3(TRAF3)	Homo sapiens
4766	11759511_ turquoise	motile sperm domain containing 2(MOSPD2)	Homo sapiens
4767	11759512_ turquoise	CWC25 spliceosome associated protein homolog(CWC25)	Homo sapiens
4768	11759519_ blue	succinate-CoA ligase ADP-forming beta subunit(SUCLA2)	Homo sapiens
4769	11759522_ brown	alkB homolog 7(ALKBH7)	Homo sapiens
4770	11759523_ brown	alkB homolog 7(ALKBH7)	Homo sapiens
4771	11759550_ turquoise	ZNFX1 antisense RNA 1(ZFAS1)	Homo sapiens
4772	11759553_ brown	translocase of inner mitochondrial membrane 8 homolog B(TIMM8B)	Homo sapiens
4773	11759566_ turquoise	N-acetylneuraminate pyruvate lyase(NPL)	Homo sapiens
4774	11759569_ turquoise	CDK5 regulatory subunit associated protein 3(CDK5RAP3)	Homo sapiens
4775	11759572_ turquoise	staufen double-stranded RNA binding protein 2(STAU2)	Homo sapiens
4776	11759573_ turquoise	meningioma expressed antigen 5 (hyaluronidase)(MGEA5)	Homo sapiens
4777	11759575_ brown	ubiquinol-cytochrome c reductase hinge protein like(UQCRHL)	Homo sapiens
4778	11759581_ turquoise	major histocompatibility complex, class I, B(HLA-B)	Homo sapiens
4779	11759587_ turquoise	HtrA serine peptidase 3(HTRA3)	Homo sapiens
4780	11759615_ turquoise	pre-mRNA processing factor 38B(PRPF38B)	Homo sapiens
4781	11759619_ turquoise	trafficking protein particle complex 10(TRAPPC10)	Homo sapiens
4782	11759622_ turquoise	DExD-box helicase 21(DDX21)	Homo sapiens
4783	11759627_ turquoise	microRNA 6125(MIR6125)	Homo sapiens
4784	11759629_ turquoise	T cell receptor delta variable 2(TRDV2)	Homo sapiens
4785	11759635_ turquoise	THO complex 2(THOC2)	Homo sapiens
4786	11759642_ turquoise	major histocompatibility complex, class II, DR beta 1(HLA-DRB1)	Homo sapiens
4787	11759645_ brown	COX11, cytochrome c oxidase copper chaperone(COX11)	Homo sapiens
4788	11759652_ yellow	immunoglobulin kappa constant(IGKC)	Homo sapiens
4789	11759657_ turquoise	target of myb1 like 1 membrane trafficking protein(TOM1L1)	Homo sapiens
4790	11759663_ turquoise	SP100 nuclear antigen(SP100)	Homo sapiens
4791	11759666_ turquoise	major histocompatibility complex, class II, DP beta 1(HLA-DPB1)	Homo sapiens
4792	11759671_ turquoise	potassium calcium-activated channel subfamily M alpha 1(KCNMA1)	Homo sapiens
4793	11759678_ turquoise	RAB12, member RAS oncogene family(RAB12)	Homo sapiens
4794	11759687_ brown	small nuclear RNA activating complex polypeptide 5(SNAPC5)	Homo sapiens
4795	11759729_ turquoise	tetratricopeptide repeat domain 14(TTC14)	Homo sapiens
4796	11759759_ turquoise	cyclin L1(CCNL1)	Homo sapiens
4797	11759766_ turquoise	major histocompatibility complex, class I-related(MR1)	Homo sapiens
4798	11759767_ turquoise	major histocompatibility complex, class I-related(MR1)	Homo sapiens

4799	11759815_ yellow	immunoglobulin heavy constant mu(IGHM)	Homo sapiens
4800	11759816_ yellow	immunoglobulin heavy constant mu(IGHM)	Homo sapiens
4801	11759828_ turquoise	SP100 nuclear antigen(SP100)	Homo sapiens
4802	11759833_ turquoise	NADH:ubiquinone oxidoreductase core subunit S7(NDUFS7)	Homo sapiens
4803	11759852_ yellow	microRNA 8071-1(MIR8071-1)	Homo sapiens
4804	11759854_ turquoise	ribosomal protein S27a(RPS27A)	Homo sapiens
4805	11759866_ turquoise	NA	NA
4806	11759922_ turquoise	par-3 family cell polarity regulator(PARD3)	Homo sapiens
4807	11759939_ turquoise	uncharacterized LOC100131541(LOC100131541)	Homo sapiens
4808	11759973_ turquoise	prenyl (decaprenyl) diphosphate synthase, subunit 1(PDSS1)	Homo sapiens
4809	11760005_ turquoise	DnaJ heat shock protein family (Hsp40) member A1(DNAJA1)	Homo sapiens
4810	11760009_ turquoise	NA	NA
4811	11760018_ turquoise	C-type lectin domain family 10 member A(CLEC10A)	Homo sapiens
4812	11760037_ turquoise	glutaminase(GLS)	Homo sapiens
4813	11760038_ turquoise	glutaminase(GLS)	Homo sapiens
4814	11760085_ turquoise	heterogeneous nuclear ribonucleoprotein A1(HNRNPA1)	Homo sapiens
4815	11760103_ turquoise	protein kinase cAMP-dependent type II regulatory subunit alpha(PRKAR2A)	Homo sapiens
4816	11760124_ turquoise	Kruppel like factor 6(KLF6)	Homo sapiens
4817	11760137_ yellow	immunoglobulin heavy constant alpha 1(IGHA1)	Homo sapiens
4818	11760143_ turquoise	major histocompatibility complex, class I, F(HLA-F)	Homo sapiens
4819	11760144_ turquoise	major histocompatibility complex, class I, F(HLA-F)	Homo sapiens
4820	11760156_ turquoise	Enah/Vasp-like(EVL)	Homo sapiens
4821	11760198_ turquoise	eukaryotic translation initiation factor 4E family member 2(EIF4E2)	Homo sapiens
4822	11760211_ turquoise	CD48 molecule(CD48)	Homo sapiens
4823	11760221_ turquoise	caspase 4(CASP4)	Homo sapiens
4824	11760222_ turquoise	caspase 4(CASP4)	Homo sapiens
4825	11760244_ turquoise	caspase recruitment domain family member 8(CARD8)	Homo sapiens
4826	11760280_ turquoise	epidermal growth factor receptor pathway substrate 15 like 1(EPS15L1)	Homo sapiens
4827	11760356_ turquoise	peptidase, mitochondrial processing beta subunit(PMPCB)	Homo sapiens
4828	11760380_ turquoise	septin 6(SEPT6)	Homo sapiens
4829	11760387_ turquoise	interleukin 16(IL16)	Homo sapiens
4830	11760406_ turquoise	major histocompatibility complex, class I, G(HLA-G)	Homo sapiens
4831	11760426_ turquoise	epidermal growth factor receptor pathway substrate 15 like 1(EPS15L1)	Homo sapiens
4832	11760444_ turquoise	major histocompatibility complex, class I, C(HLA-C)	Homo sapiens
4833	11760475_ turquoise	serine/threonine kinase 4(STK4)	Homo sapiens
4834	11760482_ brown	NADH:ubiquinone oxidoreductase core subunit S7(NDUFS7)	Homo sapiens
4835	11760584_ turquoise	YME1 like 1 ATPase(YME1L1)	Homo sapiens
4836	11760710_ turquoise	membrane spanning 4-domains A6A(MS4A6A)	Homo sapiens
4837	11760776_ turquoise	dermokine(DMKN)	Homo sapiens
4838	11760819_ yellow	immunoglobulin heavy variable 4-31(IGHV4-31)	Homo sapiens
4839	11760822_ turquoise	sodium voltage-gated channel beta subunit 1(SCN1B)	Homo sapiens
4840	11760850_ turquoise	PATJ, crumbs cell polarity complex component(PATJ)	Homo sapiens
4841	11760878_ turquoise	major histocompatibility complex, class II, DP beta 1(HLA-DPB1)	Homo sapiens
4842	11760881_ turquoise	transcription termination factor 2(TTF2)	Homo sapiens
4843	11760902_ turquoise	napsin B aspartic peptidase, pseudogene(NAPSB)	Homo sapiens
4844	11760929_ yellow	microRNA 8071-1(MIR8071-1)	Homo sapiens
4845	11760994_ turquoise	tryptophanyl-tRNA synthetase(WARS)	Homo sapiens
4846	11761076_ brown	acyl-CoA thioesterase 8(ACOT8)	Homo sapiens
4847	11761116_ turquoise	family with sequence similarity 65 member B(FAM65B)	Homo sapiens
4848	11761194_ turquoise	trans-golgi network protein 2(TGOLN2)	Homo sapiens

4849	11761343_	turquoise	ADAM metallopeptidase domain 8(ADAM8)	Homo sapiens
4850	11761390_	turquoise	ankyrin repeat domain 10(ANKRD10)	Homo sapiens
4851	11761400_	turquoise	nucleolar protein interacting with the FHA domain of MKI67(NIFK)	Homo sapiens
4852	11761418_	turquoise	interleukin 23 subunit alpha(IL23A)	Homo sapiens
4853	11761427_	turquoise	dedicator of cytokinesis 8(DOCK8)	Homo sapiens
4854	11761446_	blue	staufen double-stranded RNA binding protein 2(STAU2)	Homo sapiens
4855	11761465_	turquoise	NA	NA
4856	11761467_	yellow	immunoglobulin heavy constant gamma 3 (G3m marker)(IGHG3)	Homo sapiens
4857	11761506_	turquoise	malic enzyme 3(ME3)	Homo sapiens
4858	11761525_	turquoise	interleukin 23 subunit alpha(IL23A)	Homo sapiens
4859	11761604_	turquoise	RNA binding protein, fox-1 homolog 1(RBFOX1)	Homo sapiens
4860	11761671_	turquoise	ETS variant 7(ETV7)	Homo sapiens
4861	11761683_	turquoise	pyrin and HIN domain family member 1(PYHIN1)	Homo sapiens
4862	11761703_	turquoise	DnaJ heat shock protein family (Hsp40) member A1(DNAJA1)	Homo sapiens
4863	11761727_	turquoise	aquaporin 7(AQP7)	Homo sapiens
4864	11761758_	turquoise	proteasome subunit beta 9(PSMB9)	Homo sapiens
4865	11761790_	turquoise	YME1 like 1 ATPase(YME1L1)	Homo sapiens
4866	11761796_	turquoise	calcitonin receptor like receptor(CALCRL)	Homo sapiens
4867	11761918_	turquoise	T cell receptor beta constant 1(TRBC1)	Homo sapiens
4868	11761959_	turquoise	T cell receptor delta variable 2(TRDV2)	Homo sapiens
4869	11761960_	turquoise	T cell receptor delta variable 2(TRDV2)	Homo sapiens
4870	11762018_	turquoise	DNA cross-link repair 1C(DCLRE1C)	Homo sapiens
4871	11762038_	turquoise	zinc finger protein 844(ZNF844)	Homo sapiens
4872	11762069_	turquoise	septin 6(SEPT6)	Homo sapiens
4873	11762091_	yellow	immunoglobulin heavy locus(IGH)	Homo sapiens
4874	11762099_	yellow	immunoglobulin heavy constant alpha 1(IGHA1)	Homo sapiens
4875	11762119_	turquoise	major histocompatibility complex, class I, A(HLA-A)	Homo sapiens
4876	11762140_	turquoise	butyrophilin like 9(BTNL9)	Homo sapiens
4877	11762150_	turquoise	major histocompatibility complex, class I, A(HLA-A)	Homo sapiens
4878	11762172_	turquoise	ETS variant 7(ETV7)	Homo sapiens
4879	11762266_	turquoise	interleukin 7 receptor(IL7R)	Homo sapiens
4880	11762274_	turquoise	major histocompatibility complex, class II, DM beta(HLA-DMB)	Homo sapiens
4881	11762275_	brown	ubiquinol-cytochrome c reductase binding protein(UQCRB)	Homo sapiens
4882	11762282_	turquoise	major histocompatibility complex, class I, A(HLA-A)	Homo sapiens
4883	11762287_	turquoise	interleukin 23 subunit alpha(IL23A)	Homo sapiens
4884	11762294_	turquoise	T cell receptor beta constant 1(TRBC1)	Homo sapiens
4885	11762313_	turquoise	enoyl-CoA hydratase domain containing 2(ECHDC2)	Homo sapiens
4886	11762318_	turquoise	interleukin 23 subunit alpha(IL23A)	Homo sapiens
4887	11762339_	turquoise	aldo-keto reductase family 7 member A2(AKR7A2)	Homo sapiens
4888	11762366_	turquoise	recombination signal binding protein for immunoglobulin kappa J region(RBPJ)	Homo sapiens
4889	11762406_	turquoise	guanylate binding protein 2(GBP2)	Homo sapiens
4890	11762432_	turquoise	WD repeat domain 31(WDR31)	Homo sapiens
4891	11762456_	turquoise	NA	NA
4892	11762480_	turquoise	SP140 nuclear body protein(SP140)	Homo sapiens
4893	11762486_	turquoise	fucosyltransferase 6(FUT6)	Homo sapiens
4894	11762641_	turquoise	major histocompatibility complex, class II, DQ beta 1(HLA-DQB1)	Homo sapiens
4895	11762736_	turquoise	butyrophilin like 9(BTNL9)	Homo sapiens
4896	11762785_	turquoise	actin gamma 1(ACTG1)	Homo sapiens
4897	11762851_	turquoise	major histocompatibility complex, class I, B(HLA-B)	Homo sapiens
4898	11762908_	turquoise	TNF receptor superfamily member 14(TNFRSF14)	Homo sapiens

4899	11762936_	turquoise	aquaporin 7(AQP7)	Homo sapiens
4900	11762940_	turquoise	major histocompatibility complex, class I, C(HLA-C)	Homo sapiens
4901	11762993_	turquoise	YME1 like 1 ATPase(YME1L1)	Homo sapiens
4902	11762996_	turquoise	YME1 like 1 ATPase(YME1L1)	Homo sapiens
4903	11762998_	turquoise	YME1 like 1 ATPase(YME1L1)	Homo sapiens
4904	11763032_	brown	adipogenesis associated Mth938 domain containing(AAMDC)	Homo sapiens
4905	11763101_	brown	NADH:ubiquinone oxidoreductase subunit B9(NDUFB9)	Homo sapiens
4906	11763144_	yellow	immunoglobulin kappa constant(IGKC)	Homo sapiens
4907	11763159_	turquoise	YME1 like 1 ATPase(YME1L1)	Homo sapiens
4908	11763182_	turquoise	CCR4-NOT transcription complex subunit 9(CNOT9)	Homo sapiens
4909	11763184_	turquoise	insulin degrading enzyme(IDE)	Homo sapiens
4910	11763186_	turquoise	caspase 1(CASP1)	Homo sapiens
4911	11763196_	turquoise	dymeclin(DYM)	Homo sapiens
4912	11763197_	turquoise	dymeclin(DYM)	Homo sapiens
4913	11763201_	turquoise	family with sequence similarity 65 member B(FAM65B)	Homo sapiens
4914	11763202_	turquoise	family with sequence similarity 65 member B(FAM65B)	Homo sapiens
4915	11763215_	turquoise	dermokine(DMKN)	Homo sapiens
4916	11763222_	yellow	immunoglobulin kappa constant(IGKC)	Homo sapiens
4917	11763233_	turquoise	T cell receptor delta variable 2(TRDV2)	Homo sapiens
4918	11763246_	turquoise	major histocompatibility complex, class II, DQ beta 1(HLA-DQB1)	Homo sapiens
4919	11763249_	yellow	immunoglobulin heavy locus(IGH)	Homo sapiens
4920	11763253_	yellow	NA	NA
4921	11763271_	turquoise	peroxisomal biogenesis factor 11 alpha(PEX11A)	Homo sapiens
4922	11763274_	turquoise	signal induced proliferation associated 1 like 1(SIPA1L1)	Homo sapiens
4923	11763307_	turquoise	nuclear factor of activated T-cells 2(NFATC2)	Homo sapiens
4924	11763310_	turquoise	microRNA 21(MIR21)	Homo sapiens
4925	11763314_	turquoise	ADP ribosylation factor like GTPase 4C(ARL4C)	Homo sapiens
4926	11763320_	turquoise	glutaminase(GLS)	Homo sapiens
4927	11763322_	turquoise	casein kinase 2 alpha 2(CSNK2A2)	Homo sapiens
4928	11763329_	turquoise	docking protein 1(DOK1)	Homo sapiens
4929	11763335_	blue	malate dehydrogenase 2(MDH2)	Homo sapiens
4930	11763336_	turquoise	uncharacterized protein DKFZp586I1420(DKFZP586I1420)	Homo sapiens
4931	11763353_	turquoise	NA	NA
4932	11763357_	turquoise	transmembrane protein 170A(TMEM170A)	Homo sapiens
4933	11763359_	turquoise	NA	NA
4934	11763365_	turquoise	caspase recruitment domain family member 8(CARD8)	Homo sapiens
4935	11763367_	turquoise	nucleic acid binding protein 1(NABP1)	Homo sapiens
4936	11763382_	turquoise	uncharacterized LOC101928143(LOC101928143)	Homo sapiens
4937	11763403_	turquoise	CUGBP, Elav-like family member 1(CELF1)	Homo sapiens
4938	11763408_	yellow	ankyrin repeat domain 36B pseudogene 2(ANKRD36BP2)	Homo sapiens
4939	11763426_	turquoise	TRAF3 interacting protein 3(TRAF3IP3)	Homo sapiens
4940	11763446_	turquoise	T cell receptor delta variable 3(TRDV3)	Homo sapiens
4941	11763447_	turquoise	YME1 like 1 ATPase(YME1L1)	Homo sapiens
4942	11763472_	turquoise	zinc finger CCCH-type containing 12D(ZC3H12D)	Homo sapiens
4943	11763494_	turquoise	FERM domain containing 8(FRMD8)	Homo sapiens
4944	11763522_	blue	ryanodine receptor 2(RYR2)	Homo sapiens
4945	11763550_	yellow	immunoglobulin kappa constant(IGKC)	Homo sapiens
4946	11763556_	turquoise	eukaryotic translation initiation factor 4A1(EIF4A1)	Homo sapiens
4947	11763557_	turquoise	T cell receptor beta constant 1(TRBC1)	Homo sapiens
4948	11763585_	turquoise	thymopoietin(TMPO)	Homo sapiens

4949	11763605_	turquoise	Wnt family member 11(WNT11)	Homo sapiens
4950	11763606_	turquoise	vav guanine nucleotide exchange factor 1(VAV1)	Homo sapiens
4951	11763627_	blue	solute carrier family 25 member 10(SLC25A10)	Homo sapiens
4952	11763640_	yellow	immunoglobulin kappa constant(IGKC)	Homo sapiens
4953	11763663_	yellow	immunoglobulin kappa constant(IGKC)	Homo sapiens
4954	11763684_	yellow	immunoglobulin kappa constant(IGKC)	Homo sapiens
4955	11763693_	turquoise	glutaminase(GLS)	Homo sapiens
4956	11763699_	blue	cytoplasmic linker associated protein 2(CLASP2)	Homo sapiens
4957	11763704_	turquoise	spermidine/spermine N1-acetyltransferase 1(SAT1)	Homo sapiens
4958	11763715_	turquoise	granulysin(GNLY)	Homo sapiens
4959	11763726_	turquoise	small nucleolar RNA, H/ACA box 70(SNORA70)	Homo sapiens
4960	11763735_	turquoise	Fc receptor like 3(FCRL3)	Homo sapiens
4961	11763756_	turquoise	granulysin(GNLY)	Homo sapiens
4962	11763776_	turquoise	thymocyte selection associated family member 2(THEMIS2)	Homo sapiens
4963	11763862_	turquoise	SH3 domain binding glutamate rich protein like(SH3BGRL)	Homo sapiens
4964	11763960_	brown	NADH:ubiquinone oxidoreductase subunit B3(NDUFB3)	Homo sapiens
4965	11763986_	turquoise	apolipoprotein L1(APOL1)	Homo sapiens
4966	11763990_	brown	alkB homolog 7(ALKBH7)	Homo sapiens
4967	11764017_	turquoise	aph-1 homolog A, gamma-secretase subunit(APH1A)	Homo sapiens
4968	11764037_	turquoise	DnaJ heat shock protein family (Hsp40) member A1(DNAJA1)	Homo sapiens
4969	11764046_	yellow	immunoglobulin heavy constant mu(IGHM)	Homo sapiens
4970	11764053_	turquoise	recombination signal binding protein for immunoglobulin kappa J region(RBPJ)	Homo sapiens
4971	11764085_	turquoise	NA	NA
4972	11764130_	yellow	immunoglobulin heavy locus(IGH)	Homo sapiens
4973	11764146_	turquoise	motile sperm domain containing 2(MOSPD2)	Homo sapiens
4974	11764172_	turquoise	uncharacterized LOC102724880(LOC102724880)	Homo sapiens
4975	11764239_	turquoise	transforming growth factor beta regulator 1(TBRG1)	Homo sapiens
4976	11764248_	turquoise	low density lipoprotein receptor class A domain containing 3(LDLRAD3)	Homo sapiens
4977	200003_PI	turquoise	microRNA 6805(MIR6805)	Homo sapiens
4978	200009_PI	turquoise	GDP dissociation inhibitor 2(GDI2)	Homo sapiens
4979	200011_PI	turquoise	ADP ribosylation factor 3(ARF3)	Homo sapiens
4980	200015_PI	turquoise	septin 2(SEPT2)	Homo sapiens
4981	200021_PI	turquoise	cofilin 1(CFL1)	Homo sapiens
4982	200030_PI	blue	solute carrier family 25 member 3(SLC25A3)	Homo sapiens
4983	200033_PI	turquoise	microRNA 3064(MIR3064)	Homo sapiens
4984	200037_PI	turquoise	chromobox 3(CBX3)	Homo sapiens
4985	200044_PI	turquoise	glutamyl-tRNA amidotransferase subunit C(GATC)	Homo sapiens
4986	200057_PI	turquoise	non-POU domain containing, octamer-binding(NONO)	Homo sapiens
4987	200059_PI	turquoise	ras homolog family member A(RHOA)	Homo sapiens
4988	200086_PI	brown	cytochrome c oxidase subunit 4I1(COX4I1)	Homo sapiens
4989	AFFX-BkGr	turquoise	NA	NA
4990	AFFX-BkGr	turquoise	NA	NA
4991	AFFX-BkGr	turquoise	NA	NA
4992	AFFX-BkGr	turquoise	NA	NA
4993	AFFX-BkGr	turquoise	NA	NA
4994	AFFX-BkGr	turquoise	NA	NA
4995	AFFX-HSAC	turquoise	actin beta(ACTB)	Homo sapiens
4996	AFFX-HSAC	turquoise	actin beta(ACTB)	Homo sapiens
4997	AFFX-HUM	turquoise	signal transducer and activator of transcription 1(STAT1)	Homo sapiens
4998	AFFX-HUM	turquoise	signal transducer and activator of transcription 1(STAT1)	Homo sapiens

4999	AFFX-HUM	turquoise	signal transducer and activator of transcription 1(STAT1)	Homo sapiens
5000	AFFX-HUM	turquoise	signal transducer and activator of transcription 1(STAT1)	Homo sapiens

Module Color	Module Size	Correlation with binary trait rejection vs non rejection
Turquoise	4314	0.77
Yellow	31	0.51
Blue	317	-0.5
Brown	332	-0.53
Unassigned gene	6	

WGCNA module assignment for the top 5,000 most significantly differentially expressed genes comparing rejection vs. non-rejection by Wilcoxon testing

Turquoise Module

	gene	hcolor	Name	Species
1	11715189_s_at	turquoise	microRNA 4738(MIR4738)	Homo sapiens
2	11715193_s_at	turquoise	UNC homeobox(UNCX)	Homo sapiens
3	11715207_at	turquoise	WDFY family member 4(WDFY4)	Homo sapiens
4	11715239_x_at	turquoise	interferon induced transmembrane protein 3(IFITM3)	Homo sapiens
5	11715291_s_at	turquoise	T-box 5(TBX5)	Homo sapiens
8	11715346_at	turquoise	Epstein-Barr virus induced 3(EBI3)	Homo sapiens
10	11715359_a_at	turquoise	TIMP metallopeptidase inhibitor 1(TIMP1)	Homo sapiens
11	11715360_x_at	turquoise	TIMP metallopeptidase inhibitor 1(TIMP1)	Homo sapiens
12	11715363_a_at	turquoise	alpha-2-macroglobulin(A2M)	Homo sapiens
13	11715367_s_at	turquoise	septin 2(SEPT2)	Homo sapiens
15	11715370_s_at	turquoise	galectin 3 binding protein(LGALS3BP)	Homo sapiens
17	11715388_s_at	turquoise	cyclin dependent kinase inhibitor 1A(CDKN1A)	Homo sapiens
18	11715390_s_at	turquoise	FK506 binding protein 1A(FKBP1A)	Homo sapiens
19	11715401_s_at	turquoise	interleukin enhancer binding factor 2(ILF2)	Homo sapiens
22	11715419_at	turquoise	lymphocyte antigen 6 complex, locus E(LY6E)	Homo sapiens
23	11715439_at	turquoise	death associated protein(DAP)	Homo sapiens
24	11715451_a_at	turquoise	collagen type IV alpha 2 chain(COL4A2)	Homo sapiens
25	11715452_s_at	turquoise	collagen type IV alpha 2 chain(COL4A2)	Homo sapiens
26	11715453_a_at	turquoise	collagen type IV alpha 2 chain(COL4A2)	Homo sapiens
29	11715461_at	turquoise	lysozyme(LYZ)	Homo sapiens
30	11715462_x_at	turquoise	lysozyme(LYZ)	Homo sapiens
31	11715466_at	turquoise	Rho GDP dissociation inhibitor beta(ARHGDIB)	Homo sapiens
32	11715467_s_at	turquoise	Rho GDP dissociation inhibitor beta(ARHGDIB)	Homo sapiens
33	11715476_s_at	turquoise	DEAD-box helicase 17(DDX17)	Homo sapiens
34	11715482_a_at	turquoise	BCL2 family apoptosis regulator(MCL1)	Homo sapiens
35	11715483_s_at	turquoise	BCL2 family apoptosis regulator(MCL1)	Homo sapiens
36	11715487_a_at	turquoise	BCL2 family apoptosis regulator(MCL1)	Homo sapiens
39	11715503_a_at	turquoise	LIM and SH3 protein 1(LASP1)	Homo sapiens
40	11715524_a_at	turquoise	coactosin like F-actin binding protein 1(COTL1)	Homo sapiens
41	11715532_a_at	turquoise	interferon gamma receptor 2 (interferon gamma transducer 1)(IFNGR2)	Homo sapiens
42	11715541_a_at	turquoise	Thy-1 cell surface antigen(THY1)	Homo sapiens
43	11715542_s_at	turquoise	Thy-1 cell surface antigen(THY1)	Homo sapiens
44	11715583_s_at	turquoise	major histocompatibility complex, class II, DP alpha 1(HLA-DPA1)	Homo sapiens
45	11715593_s_at	turquoise	tyrosine 3-monooxygenase/tryptophan 5-monooxygenase activation protein eta(YWHAH)	Homo sapiens
46	11715606_a_at	turquoise	TSC22 domain family member 1(TSC22D1)	Homo sapiens
47	11715608_x_at	turquoise	peptidylprolyl isomerase B(PPIB)	Homo sapiens
50	11715623_s_at	turquoise	ring finger protein 114(RNF114)	Homo sapiens
51	11715624_at	turquoise	SH3 domain binding glutamate rich protein like(SH3BGRL)	Homo sapiens
52	11715625_a_at	turquoise	SH3 domain binding glutamate rich protein like(SH3BGRL)	Homo sapiens
53	11715638_s_at	turquoise	lysophosphatidylcholine acyltransferase 1(LPCAT1)	Homo sapiens
54	11715644_s_at	turquoise	ATPase H+ transporting accessory protein 2(ATP6AP2)	Homo sapiens
57	11715665_a_at	turquoise	proteasome subunit beta 8(PSMB8)	Homo sapiens
58	11715667_s_at	turquoise	tropomyosin 3(TPM3)	Homo sapiens
59	11715670_a_at	turquoise	interferon induced transmembrane protein 1(IFITM1)	Homo sapiens
60	11715671_x_at	turquoise	interferon induced transmembrane protein 1(IFITM1)	Homo sapiens
61	11715684_a_at	turquoise	pyrophosphatase (inorganic) 1(PPA1)	Homo sapiens
62	11715685_s_at	turquoise	actin related protein 2/3 complex subunit 5(ARPC5)	Homo sapiens
63	11715686_s_at	turquoise	actin related protein 2/3 complex subunit 5(ARPC5)	Homo sapiens
64	11715687_x_at	turquoise	actin related protein 2/3 complex subunit 5(ARPC5)	Homo sapiens
65	11715691_s_at	turquoise	ZFP36 ring finger protein(ZFP36)	Homo sapiens
68	11715710_s_at	turquoise	cathepsin C(CTSC)	Homo sapiens
69	11715716_at	turquoise	moesin(MSN)	Homo sapiens
70	11715717_s_at	turquoise	moesin(MSN)	Homo sapiens
71	11715722_a_at	turquoise	golgi membrane protein 1(GOLM1)	Homo sapiens
72	11715742_s_at	turquoise	regulator of chromosome condensation 2(RCC2)	Homo sapiens
73	11715743_x_at	turquoise	regulator of chromosome condensation 2(RCC2)	Homo sapiens
75	11715767_s_at	turquoise	acetyl-CoA acyltransferase 2(ACAA2)	Homo sapiens
76	11715770_x_at	turquoise	SET nuclear proto-oncogene(SET)	Homo sapiens
78	11715788_a_at	turquoise	solute carrier family 40 member 1(SLC40A1)	Homo sapiens
79	11715817_at	turquoise	ZFP36 ring finger protein like 2(ZFP36L2)	Homo sapiens
80	11715818_at	turquoise	ZFP36 ring finger protein like 2(ZFP36L2)	Homo sapiens
81	11715827_s_at	turquoise	polyhomeotic homolog 1(PHC1)	Homo sapiens
82	11715836_x_at	turquoise	proteasome subunit alpha 3(PSMA3)	Homo sapiens
83	11715839_a_at	turquoise	succinate dehydrogenase complex subunit C(SDHC)	Homo sapiens
84	11715855_a_at	turquoise	prolylcarboxypeptidase(PRCP)	Homo sapiens
85	11715856_s_at	turquoise	prolylcarboxypeptidase(PRCP)	Homo sapiens
87	11715868_a_at	turquoise	TERF1 interacting nuclear factor 2(TINF2)	Homo sapiens
88	11715869_x_at	turquoise	TERF1 interacting nuclear factor 2(TINF2)	Homo sapiens
92	11715884_a_at	turquoise	transmembrane protein 248(TMEM248)	Homo sapiens
93	11715893_s_at	turquoise	interferon alpha inducible protein 27(IFI27)	Homo sapiens
94	11715897_at	turquoise	scavenger receptor class B member 2(SCARB2)	Homo sapiens
96	11715908_at	turquoise	myosin VI(MYO6)	Homo sapiens
97	11715915_a_at	turquoise	CD44 molecule (Indian blood group)(CD44)	Homo sapiens
98	11715916_a_at	turquoise	CD44 molecule (Indian blood group)(CD44)	Homo sapiens
99	11715917_a_at	turquoise	CD44 molecule (Indian blood group)(CD44)	Homo sapiens
100	11715919_a_at	turquoise	SH3 domain binding glutamate rich protein like 3(SH3BGRL3)	Homo sapiens
101	11715921_a_at	turquoise	septin 9(SEPT9)	Homo sapiens
102	11715922_x_at	turquoise	septin 9(SEPT9)	Homo sapiens
103	11715925_a_at	turquoise	translocation associated membrane protein 1(TRAM1)	Homo sapiens
104	11715931_s_at	turquoise	serum/glucocorticoid regulated kinase 1(SGK1)	Homo sapiens

105	11715935_a_at	turquoise	neurobeachin like 2(NBEAL2)	Homo sapiens
106	11715936_x_at	turquoise	neurobeachin like 2(NBEAL2)	Homo sapiens
107	11715948_at	turquoise	potassium channel tetramerization domain containing 12(KCTD12)	Homo sapiens
108	11715949_s_at	turquoise	potassium channel tetramerization domain containing 12(KCTD12)	Homo sapiens
109	11715966_s_at	turquoise	abhydrolase domain containing 17A(ABHD17A)	Homo sapiens
112	11715994_x_at	turquoise	inhibitor of DNA binding 2, HLH protein(ID2)	Homo sapiens
115	11716012_at	turquoise	TNF alpha induced protein 1(TNFAIP1)	Homo sapiens
116	11716015_a_at	turquoise	CKLF like MARVEL transmembrane domain containing 3(CMTM3)	Homo sapiens
117	11716018_s_at	turquoise	RAB31, member RAS oncogene family(RAB31)	Homo sapiens
118	11716019_at	turquoise	RAB31, member RAS oncogene family(RAB31)	Homo sapiens
121	11716031_s_at	turquoise	ADP ribosylation factor 3(ARF3)	Homo sapiens
122	11716034_a_at	turquoise	bone marrow stromal cell antigen 2(BST2)	Homo sapiens
123	11716035_at	turquoise	bone marrow stromal cell antigen 2(BST2)	Homo sapiens
124	11716036_x_at	turquoise	bone marrow stromal cell antigen 2(BST2)	Homo sapiens
126	11716055_s_at	turquoise	stathmin 3(STMN3)	Homo sapiens
128	11716062_a_at	turquoise	tenascin C(TNC)	Homo sapiens
129	11716063_at	turquoise	tenascin C(TNC)	Homo sapiens
130	11716071_s_at	turquoise	Pim-3 proto-oncogene, serine/threonine kinase(PIM3)	Homo sapiens
131	11716072_s_at	turquoise	ATPase H+ transporting V1 subunit B2(ATP6V1B2)	Homo sapiens
132	11716093_a_at	turquoise	Kruppel like factor 6(KLF6)	Homo sapiens
133	11716095_s_at	turquoise	Kruppel like factor 6(KLF6)	Homo sapiens
134	11716103_a_at	turquoise	proliferating cell nuclear antigen(PCNA)	Homo sapiens
135	11716115_s_at	turquoise	ZFP36 ring finger protein like 1(ZFP36L1)	Homo sapiens
137	11716118_a_at	turquoise	protein disulfide isomerase family A member 4(PDIA4)	Homo sapiens
138	11716119_x_at	turquoise	protein disulfide isomerase family A member 4(PDIA4)	Homo sapiens
139	11716121_s_at	turquoise	apoptosis inhibitor 5(API5)	Homo sapiens
140	11716135_a_at	turquoise	major vault protein(MVP)	Homo sapiens
141	11716167_a_at	turquoise	MX dynamin like GTPase 1(MX1)	Homo sapiens
142	11716171_s_at	turquoise	lamin B2(LMNB2)	Homo sapiens
143	11716181_x_at	turquoise	mediator complex subunit 15(MED15)	Homo sapiens
144	11716186_s_at	turquoise	activated leukocyte cell adhesion molecule(ALCAM)	Homo sapiens
149	11716226_a_at	turquoise	LIM domain and actin binding 1(LIMA1)	Homo sapiens
151	11716245_at	turquoise	WAS protein family member 2(WASF2)	Homo sapiens
152	11716249_x_at	turquoise	transmembrane p24 trafficking protein 9(TMED9)	Homo sapiens
153	11716267_at	turquoise	adenylate kinase 1(AK1)	Homo sapiens
154	11716268_at	turquoise	adenylate kinase 1(AK1)	Homo sapiens
155	11716274_a_at	turquoise	endosulfine alpha(ENSA)	Homo sapiens
156	11716275_a_at	turquoise	chromobox 5(CBX5)	Homo sapiens
157	11716283_at	turquoise	poly(A) RNA polymerase D7, non-canonical(PAPD7)	Homo sapiens
158	11716284_s_at	turquoise	glyoxalase I(GLO1)	Homo sapiens
159	11716288_s_at	turquoise	extended synaptotagmin 1(ESYT1)	Homo sapiens
160	11716294_a_at	turquoise	cysteinyl-tRNA synthetase(CARS)	Homo sapiens
161	11716307_at	turquoise	ubiquinol-cytochrome c reductase, complex III subunit XI(UQCR11)	Homo sapiens
163	11716337_s_at	turquoise	insulin induced gene 1(INSIG1)	Homo sapiens
164	11716339_a_at	turquoise	insulin induced gene 1(INSIG1)	Homo sapiens
168	11716358_s_at	turquoise	protein regulator of cytokinesis 1(PRC1)	Homo sapiens
169	11716367_s_at	turquoise	proline rich 13(PRR13)	Homo sapiens
170	11716368_x_at	turquoise	proline rich 13(PRR13)	Homo sapiens
171	11716377_s_at	turquoise	granulin precursor(GRN)	Homo sapiens
174	11716384_at	turquoise	C-C motif chemokine ligand 2(CCL2)	Homo sapiens
175	11716385_at	turquoise	transforming growth factor beta 1(TGFB1)	Homo sapiens
177	11716395_a_at	turquoise	adhesion G protein-coupled receptor G1(ADGRG1)	Homo sapiens
178	11716403_at	turquoise	eukaryotic translation initiation factor 4E binding protein 2(EIF4EBP2)	Homo sapiens
180	11716416_at	turquoise	complement C1q A chain(C1QA)	Homo sapiens
184	11716450_s_at	turquoise	raftlin, lipid raft linker 1(RFTN1)	Homo sapiens
185	11716466_s_at	turquoise	iron-sulfur cluster assembly 1(ISCA1)	Homo sapiens
186	11716467_x_at	turquoise	iron-sulfur cluster assembly 1(ISCA1)	Homo sapiens
188	11716470_at	turquoise	ubiquinol-cytochrome c reductase binding protein(UQCRB)	Homo sapiens
190	11716479_s_at	turquoise	coagulation factor XIII A chain(F13A1)	Homo sapiens
191	11716485_at	turquoise	serine/threonine kinase 38(STK38)	Homo sapiens
192	11716513_a_at	turquoise	molybdenum cofactor synthesis 2(MOCS2)	Homo sapiens
194	11716533_a_at	turquoise	palmitoyl-protein thioesterase 1(PPT1)	Homo sapiens
195	11716534_s_at	turquoise	palmitoyl-protein thioesterase 1(PPT1)	Homo sapiens
196	11716554_a_at	turquoise	major histocompatibility complex, class II, DM alpha(HLA-DMA)	Homo sapiens
199	11716564_a_at	turquoise	serologically defined colon cancer antigen 3(SDCCAG3)	Homo sapiens
200	11716567_a_at	turquoise	proline rich coiled-coil 2C(PRRC2C)	Homo sapiens
201	11716598_a_at	turquoise	charged multivesicular body protein 1A(CHMP1A)	Homo sapiens
202	11716599_s_at	turquoise	charged multivesicular body protein 1A(CHMP1A)	Homo sapiens
205	11716618_at	turquoise	SS18 like 2(SS18L2)	Homo sapiens
206	11716633_a_at	turquoise	transmembrane protein 50A(TMEM50A)	Homo sapiens
207	11716638_s_at	turquoise	collagen type IV alpha 1 chain(COL4A1)	Homo sapiens
208	11716639_a_at	turquoise	collagen type IV alpha 1 chain(COL4A1)	Homo sapiens
209	11716640_a_at	turquoise	proteasome subunit beta 10(PSMB10)	Homo sapiens
210	11716641_x_at	turquoise	proteasome subunit beta 10(PSMB10)	Homo sapiens
211	11716646_a_at	turquoise	histocompatibility minor 13(HM13)	Homo sapiens
212	11716647_x_at	turquoise	histocompatibility minor 13(HM13)	Homo sapiens
213	11716650_a_at	turquoise	ubiquitin conjugating enzyme E2 Z(UBE2Z)	Homo sapiens
214	11716651_a_at	turquoise	ubiquitin conjugating enzyme E2 Z(UBE2Z)	Homo sapiens
215	11716653_x_at	turquoise	echinoderm microtubule associated protein like 4(EML4)	Homo sapiens
216	11716654_a_at	turquoise	echinoderm microtubule associated protein like 4(EML4)	Homo sapiens
217	11716662_at	turquoise	sushi domain containing 6(SUSD6)	Homo sapiens
218	11716666_a_at	turquoise	inhibitor of DNA binding 3, HLH protein(ID3)	Homo sapiens

219	11716682_s_at	turquoise	solute carrier family 39 member 9(SLC39A9)	Homo sapiens
220	11716683_x_at	turquoise	solute carrier family 39 member 9(SLC39A9)	Homo sapiens
223	11716733_at	turquoise	interferon regulatory factor 1(IRF1)	Homo sapiens
224	11716734_at	turquoise	interferon regulatory factor 1(IRF1)	Homo sapiens
225	11716739_x_at	turquoise	GRAM domain containing 1A(GRAMD1A)	Homo sapiens
226	11716746_a_at	turquoise	family with sequence similarity 60 member A(FAM60A)	Homo sapiens
227	11716759_a_at	turquoise	caspase 4(CASP4)	Homo sapiens
228	11716760_x_at	turquoise	caspase 4(CASP4)	Homo sapiens
229	11716765_s_at	turquoise	IQ motif containing GTPase activating protein 1(IQGAP1)	Homo sapiens
230	11716766_a_at	turquoise	IQ motif containing GTPase activating protein 1(IQGAP1)	Homo sapiens
231	11716767_at	turquoise	IQ motif containing GTPase activating protein 1(IQGAP1)	Homo sapiens
232	11716771_s_at	turquoise	salt inducible kinase 1(SIK1)	Homo sapiens
233	11716774_a_at	turquoise	retinoic acid induced 14(RAI14)	Homo sapiens
234	11716775_a_at	turquoise	retinoic acid induced 14(RAI14)	Homo sapiens
235	11716787_a_at	turquoise	beta-1,4-glucuronyltransferase 1(B4GAT1)	Homo sapiens
236	11716788_at	turquoise	beta-1,4-glucuronyltransferase 1(B4GAT1)	Homo sapiens
237	11716792_s_at	turquoise	uncharacterized LOC440034(DKFZp686K1684)	Homo sapiens
238	11716793_a_at	turquoise	cyclin B2(CCNB2)	Homo sapiens
239	11716794_a_at	turquoise	myosin light chain 6(MYL6)	Homo sapiens
240	11716795_s_at	turquoise	interferon gamma inducible protein 16(IFI16)	Homo sapiens
241	11716796_s_at	turquoise	interferon gamma inducible protein 16(IFI16)	Homo sapiens
242	11716797_s_at	turquoise	lamin B receptor(LBR)	Homo sapiens
243	11716800_at	turquoise	Fas associated via death domain(FADD)	Homo sapiens
244	11716828_a_at	turquoise	NPC intracellular cholesterol transporter 2(NPC2)	Homo sapiens
245	11716842_a_at	turquoise	CD53 molecule(CD53)	Homo sapiens
246	11716846_a_at	turquoise	membrane spanning 4-domains A6A(MS4A6A)	Homo sapiens
247	11716847_a_at	turquoise	solute carrier family 43 member 3(SLC43A3)	Homo sapiens
248	11716848_a_at	turquoise	phosphatidylinositol-3,4,5-trisphosphate dependent Rac exchange factor 1(PREX1)	Homo sapiens
249	11716849_s_at	turquoise	phosphatidylinositol-3,4,5-trisphosphate dependent Rac exchange factor 1(PREX1)	Homo sapiens
250	11716850_s_at	turquoise	major facilitator superfamily domain containing 14B(MFSD14B)	Homo sapiens
252	11716865_a_at	turquoise	solute carrier family 1 member 5(SLC1A5)	Homo sapiens
253	11716866_x_at	turquoise	solute carrier family 1 member 5(SLC1A5)	Homo sapiens
254	11716867_at	turquoise	fem-1 homolog A(FEM1A)	Homo sapiens
255	11716868_at	turquoise	UTP18, small subunit processome component(UTP18)	Homo sapiens
256	11716883_s_at	turquoise	RAP1B, member of RAS oncogene family(RAP1B)	Homo sapiens
257	11716888_s_at	turquoise	hexosaminidase subunit alpha(HEXA)	Homo sapiens
259	11716895_s_at	turquoise	ISG15 ubiquitin-like modifier(ISG15)	Homo sapiens
261	11716901_s_at	turquoise	trans-golgi network protein 2(TGOLN2)	Homo sapiens
262	11716904_s_at	turquoise	trans-golgi network protein 2(TGOLN2)	Homo sapiens
263	11716908_a_at	turquoise	ribonuclease T2(RNASET2)	Homo sapiens
264	11716918_a_at	turquoise	N-acetylglucosamine kinase(NAGK)	Homo sapiens
265	11716920_at	turquoise	plasmalemma vesicle associated protein(PLVAP)	Homo sapiens
267	11716938_a_at	turquoise	Sec61 translocon beta subunit(SEC61B)	Homo sapiens
268	11716939_a_at	turquoise	heme oxygenase 1(HMOX1)	Homo sapiens
269	11716941_at	turquoise	tribbles pseudokinase 2(TRIB2)	Homo sapiens
270	11716945_s_at	turquoise	twinfilin actin binding protein 1(TWF1)	Homo sapiens
272	11716962_a_at	turquoise	protein phosphatase, Mg2+/Mn2+ dependent 1F(PPM1F)	Homo sapiens
273	11716963_a_at	turquoise	protein phosphatase, Mg2+/Mn2+ dependent 1F(PPM1F)	Homo sapiens
274	11716972_s_at	turquoise	N-ethylmaleimide sensitive factor, vesicle fusing ATPase(NSF)	Homo sapiens
276	11716986_a_at	turquoise	general transcription factor IIB(GTF2B)	Homo sapiens
277	11716993_a_at	turquoise	colony stimulating factor 1 receptor(CSF1R)	Homo sapiens
278	11717012_s_at	turquoise	vesicle associated membrane protein 8(VAMP8)	Homo sapiens
279	11717045_a_at	turquoise	complement C2(C2)	Homo sapiens
280	11717051_s_at	turquoise	signal sequence receptor subunit 1(SSR1)	Homo sapiens
282	11717104_a_at	turquoise	glyoxylate and hydroxypyruvate reductase(GRHPR)	Homo sapiens
283	11717114_a_at	turquoise	SPT16 homolog, facilitates chromatin remodeling subunit(SUPT16H)	Homo sapiens
284	11717127_a_at	turquoise	alanyl aminopeptidase, membrane(ANPEP)	Homo sapiens
285	11717153_a_at	turquoise	adipocyte plasma membrane associated protein(APMAP)	Homo sapiens
286	11717160_a_at	turquoise	clathrin light chain A(CLTA)	Homo sapiens
287	11717163_s_at	turquoise	cell division cycle 20(CDC20)	Homo sapiens
288	11717167_a_at	turquoise	lysine demethylase 2A(KDM2A)	Homo sapiens
289	11717178_a_at	turquoise	signal transducer and activator of transcription 5A(STAT5A)	Homo sapiens
290	11717188_s_at	turquoise	DnaJ heat shock protein family (Hsp40) member A1(DNAJA1)	Homo sapiens
291	11717189_x_at	turquoise	DnaJ heat shock protein family (Hsp40) member A1(DNAJA1)	Homo sapiens
292	11717211_a_at	turquoise	neogenin 1(NEO1)	Homo sapiens
293	11717217_at	turquoise	serine/threonine kinase 10(STK10)	Homo sapiens
294	11717218_a_at	turquoise	serine/threonine kinase 10(STK10)	Homo sapiens
295	11717228_at	turquoise	cerebellar degeneration-related protein 2(LOC101060399)	Homo sapiens
298	11717256_at	turquoise	Pim-1 proto-oncogene, serine/threonine kinase(PIM1)	Homo sapiens
299	11717257_s_at	turquoise	Pim-1 proto-oncogene, serine/threonine kinase(PIM1)	Homo sapiens
300	11717281_a_at	turquoise	ArfGAP with SH3 domain, ankyrin repeat and PH domain 1(ASAP1)	Homo sapiens
301	11717282_at	turquoise	ArfGAP with SH3 domain, ankyrin repeat and PH domain 1(ASAP1)	Homo sapiens
302	11717293_x_at	turquoise	serine and arginine rich splicing factor 7(SRSF7)	Homo sapiens
303	11717296_s_at	turquoise	leucine rich pentatricopeptide repeat containing(LRPPRC)	Homo sapiens
305	11717299_at	turquoise	major histocompatibility complex, class II, DP beta 1(HLA-DPB1)	Homo sapiens
306	11717300_x_at	turquoise	major histocompatibility complex, class II, DP beta 1(HLA-DPB1)	Homo sapiens
307	11717311_s_at	turquoise	F-box protein 7(FBXO7)	Homo sapiens
308	11717317_at	turquoise	glycerol-3-phosphate acyltransferase 4(GPAT4)	Homo sapiens
309	11717319_a_at	turquoise	SEC24 homolog D, COPII coat complex component(SEC24D)	Homo sapiens
310	11717325_at	turquoise	nudix hydrolase 16 like 1(NUDT16L1)	Homo sapiens
311	11717337_a_at	turquoise	integrin subunit beta 2(ITGB2)	Homo sapiens
312	11717365_x_at	turquoise	syntrophin alpha 1(SNTA1)	Homo sapiens

313	11717366_at	turquoise	zinc finger CCCH-type containing, antiviral 1(ZC3HAV1)	Homo sapiens
314	11717367_at	turquoise	zinc finger CCCH-type containing, antiviral 1(ZC3HAV1)	Homo sapiens
315	11717368_x_at	turquoise	zinc finger CCCH-type containing, antiviral 1(ZC3HAV1)	Homo sapiens
316	11717371_a_at	turquoise	chromodomain helicase DNA binding protein 1 like(CHD1L)	Homo sapiens
317	11717375_a_at	turquoise	solute carrier family 25 member 25(SLC25A25)	Homo sapiens
318	11717394_at	turquoise	ariadne RBR E3 ubiquitin protein ligase 2(ARIH2)	Homo sapiens
319	11717397_a_at	turquoise	damage specific DNA binding protein 2(DDB2)	Homo sapiens
320	11717401_at	turquoise	ADP ribosylation factor 6(ARF6)	Homo sapiens
321	11717402_s_at	turquoise	ADP ribosylation factor 6(ARF6)	Homo sapiens
323	11717409_at	turquoise	SH2B adaptor protein 3(SH2B3)	Homo sapiens
324	11717418_at	turquoise	dynein axonemal light chain 1(DNAL1)	Homo sapiens
325	11717423_s_at	turquoise	RNA binding motif protein 8A(RBM8A)	Homo sapiens
326	11717425_a_at	turquoise	RNF103-CHMP3 readthrough(RNF103-CHMP3)	Homo sapiens
327	11717427_a_at	turquoise	G protein subunit beta 1(GNB1)	Homo sapiens
330	11717438_x_at	turquoise	topoisomerase (DNA) I(TOP1)	Homo sapiens
331	11717447_s_at	turquoise	destrin, actin depolymerizing factor(DSTN)	Homo sapiens
332	11717453_at	turquoise	SPARC/osteonectin, cwcv and kazal like domains proteoglycan 2(SPOCK2)	Homo sapiens
333	11717454_at	turquoise	SPARC/osteonectin, cwcv and kazal like domains proteoglycan 2(SPOCK2)	Homo sapiens
334	11717464_at	turquoise	latexin(LXN)	Homo sapiens
335	11717465_s_at	turquoise	latexin(LXN)	Homo sapiens
336	11717479_at	turquoise	lysosomal associated membrane protein 2(LAMP2)	Homo sapiens
337	11717482_at	turquoise	pyruvate dehydrogenase (lipoamide) alpha 1(PDHA1)	Homo sapiens
340	11717496_a_at	turquoise	inositol monophosphatase 2(IMPA2)	Homo sapiens
342	11717501_a_at	turquoise	hydroxyacylglutathione hydrolase(HAGH)	Homo sapiens
343	11717502_a_at	turquoise	negative regulator of ubiquitin like proteins 1(NUB1)	Homo sapiens
344	11717503_s_at	turquoise	negative regulator of ubiquitin like proteins 1(NUB1)	Homo sapiens
345	11717507_at	turquoise	translocation associated membrane protein 2(TRAM2)	Homo sapiens
346	11717508_at	turquoise	interferon regulatory factor 4(IRF4)	Homo sapiens
347	11717514_a_at	turquoise	annexin A1(ANXA1)	Homo sapiens
348	11717517_s_at	turquoise	RNA exonuclease 2(REXO2)	Homo sapiens
349	11717521_x_at	turquoise	pituitary tumor-transforming 3, pseudogene(PTTG3P)	Homo sapiens
350	11717528_a_at	turquoise	solute carrier family 25 member 20(SLC25A20)	Homo sapiens
351	11717529_at	turquoise	solute carrier family 25 member 20(SLC25A20)	Homo sapiens
352	11717533_at	turquoise	mannosidase alpha class 2A member 2(MAN2A2)	Homo sapiens
353	11717542_a_at	turquoise	nuclear factor kappa B subunit 1(NFKB1)	Homo sapiens
354	11717561_s_at	turquoise	deltex E3 ubiquitin ligase 3L(DTX3L)	Homo sapiens
355	11717562_x_at	turquoise	deltex E3 ubiquitin ligase 3L(DTX3L)	Homo sapiens
356	11717574_s_at	turquoise	profilin 1(PFN1)	Homo sapiens
357	11717580_a_at	turquoise	CD52 molecule(CD52)	Homo sapiens
358	11717581_s_at	turquoise	CD52 molecule(CD52)	Homo sapiens
359	11717611_a_at	turquoise	microtubule associated serine/threonine kinase 2(MAST2)	Homo sapiens
361	11717636_s_at	turquoise	ras homolog family member G(RHOG)	Homo sapiens
362	11717637_x_at	turquoise	ras homolog family member G(RHOG)	Homo sapiens
365	11717657_a_at	turquoise	enhancer of zeste 2 polycomb repressive complex 2 subunit(EZH2)	Homo sapiens
366	11717661_a_at	turquoise	protein phosphatase 1 regulatory subunit 16B(PPP1R16B)	Homo sapiens
367	11717662_a_at	turquoise	piezo type mechanosensitive ion channel component 1(PIEZO1)	Homo sapiens
369	11717672_s_at	turquoise	ectonucleotide pyrophosphatase/phosphodiesterase 2(ENPP2)	Homo sapiens
370	11717679_a_at	turquoise	unc-13 homolog B(UNC13B)	Homo sapiens
371	11717681_at	turquoise	G protein subunit alpha 13(GNA13)	Homo sapiens
372	11717688_a_at	turquoise	ring finger protein 19A, RBR E3 ubiquitin protein ligase(RNF19A)	Homo sapiens
373	11717691_a_at	turquoise	ankyrin repeat domain 13A(ANKRD13A)	Homo sapiens
374	11717708_s_at	turquoise	protein tyrosine phosphatase, non-receptor type 18(PTPN18)	Homo sapiens
375	11717714_at	turquoise	carbohydrate sulfotransferase 10(CHST10)	Homo sapiens
376	11717721_s_at	turquoise	ST6 beta-galactoside alpha-2,6-sialyltransferase 1(ST6GAL1)	Homo sapiens
377	11717726_s_at	turquoise	hematological and neurological expressed 1(HN1)	Homo sapiens
378	11717727_s_at	turquoise	hematological and neurological expressed 1(HN1)	Homo sapiens
379	11717730_s_at	turquoise	squalene epoxidase(SQLE)	Homo sapiens
380	11717732_s_at	turquoise	6-phosphofructo-2-kinase/fructose-2,6-biphosphatase 3(PFKFB3)	Homo sapiens
381	11717737_at	turquoise	prenylcysteine oxidase 1(PCYOX1)	Homo sapiens
382	11717738_at	turquoise	prenylcysteine oxidase 1(PCYOX1)	Homo sapiens
383	11717739_at	turquoise	prenylcysteine oxidase 1(PCYOX1)	Homo sapiens
384	11717743_a_at	turquoise	acyl-CoA thioesterase 7(ACOT7)	Homo sapiens
385	11717746_a_at	turquoise	membrane associated ring-CH-type finger 6(MARCH6)	Homo sapiens
386	11717760_a_at	turquoise	glia maturation factor gamma(GMFG)	Homo sapiens
387	11717800_a_at	turquoise	activating transcription factor 5(ATF5)	Homo sapiens
388	11717801_a_at	turquoise	activating transcription factor 5(ATF5)	Homo sapiens
389	11717823_s_at	turquoise	TNF alpha induced protein 2(TNFAIP2)	Homo sapiens
390	11717836_a_at	turquoise	allograft inflammatory factor 1(AIF1)	Homo sapiens
391	11717837_x_at	turquoise	allograft inflammatory factor 1(AIF1)	Homo sapiens
392	11717839_a_at	turquoise	programmed cell death 6(PDCD6)	Homo sapiens
393	11717840_at	turquoise	ethanolamine kinase 1(ETNK1)	Homo sapiens
394	11717841_at	turquoise	ethanolamine kinase 1(ETNK1)	Homo sapiens
395	11717858_a_at	turquoise	CD37 molecule(CD37)	Homo sapiens
397	11717865_at	turquoise	proteasome activator subunit 4(PSME4)	Homo sapiens
398	11717872_a_at	turquoise	interferon regulatory factor 8(IRF8)	Homo sapiens
399	11717873_x_at	turquoise	interferon regulatory factor 8(IRF8)	Homo sapiens
400	11717874_a_at	turquoise	interferon regulatory factor 8(IRF8)	Homo sapiens
401	11717883_x_at	turquoise	zinc fingers and homeoboxes 3(ZHX3)	Homo sapiens
402	11717884_a_at	turquoise	zinc fingers and homeoboxes 3(ZHX3)	Homo sapiens
403	11717886_a_at	turquoise	plasminogen activator, urokinase(PLAU)	Homo sapiens
404	11717887_s_at	turquoise	protein tyrosine phosphatase, non-receptor type 12(PTPN12)	Homo sapiens
405	11717889_s_at	turquoise	G protein subunit alpha i2(GNAI2)	Homo sapiens

406	11717931_at	turquoise	TANK binding kinase 1(TBK1)	Homo sapiens
408	11717939_a_at	turquoise	U2 small nuclear RNA auxiliary factor 2(U2AF2)	Homo sapiens
409	11717959_a_at	turquoise	transcription factor 3(TCF3)	Homo sapiens
410	11717965_s_at	turquoise	KRAS proto-oncogene, GTPase(KRAS)	Homo sapiens
411	11717972_a_at	turquoise	family with sequence similarity 219 member B(FAM219B)	Homo sapiens
412	11717973_x_at	turquoise	family with sequence similarity 219 member B(FAM219B)	Homo sapiens
413	11717974_x_at	turquoise	family with sequence similarity 219 member B(FAM219B)	Homo sapiens
414	11717981_a_at	turquoise	acid phosphatase 5, tartrate resistant(ACP5)	Homo sapiens
415	11717988_s_at	turquoise	RNASEK-C17orf49 readthrough(RNASEK-C17orf49)	Homo sapiens
416	11718001_a_at	turquoise	chromosome 10 open reading frame 54(C10orf54)	Homo sapiens
417	11718002_at	turquoise	chromosome 10 open reading frame 54(C10orf54)	Homo sapiens
418	11718005_a_at	turquoise	cell division cycle 25B(CDC25B)	Homo sapiens
419	11718008_a_at	turquoise	copine 2(CPNE2)	Homo sapiens
420	11718010_at	turquoise	solute carrier family 25 member 22(SLC25A22)	Homo sapiens
421	11718011_a_at	turquoise	mitochondrial ribosomal protein L4(MRPL4)	Homo sapiens
422	11718020_a_at	turquoise	leucine rich repeat containing 20(LRRC20)	Homo sapiens
423	11718026_a_at	turquoise	nuclear receptor coactivator 7(NCOA7)	Homo sapiens
424	11718027_a_at	turquoise	pseudouridylate synthase 1(PUS1)	Homo sapiens
425	11718028_x_at	turquoise	pseudouridylate synthase 1(PUS1)	Homo sapiens
428	11718037_x_at	turquoise	midkine (neurite growth-promoting factor 2)(MDK)	Homo sapiens
429	11718050_a_at	turquoise	brain abundant membrane attached signal protein 1(BASP1)	Homo sapiens
430	11718058_a_at	turquoise	thymidylate synthetase(TYMS)	Homo sapiens
431	11718060_a_at	turquoise	tweety family member 2(TTYH2)	Homo sapiens
432	11718064_a_at	turquoise	S100 calcium binding protein A4(S100A4)	Homo sapiens
433	11718065_a_at	turquoise	nectin cell adhesion molecule 2(NECTIN2)	Homo sapiens
434	11718068_at	turquoise	myosin IE(MYO1E)	Homo sapiens
435	11718073_at	turquoise	interleukin 4 receptor(IL4R)	Homo sapiens
436	11718075_at	turquoise	interleukin 4 receptor(IL4R)	Homo sapiens
438	11718084_a_at	turquoise	LYN proto-oncogene, Src family tyrosine kinase(LYN)	Homo sapiens
439	11718085_s_at	turquoise	LYN proto-oncogene, Src family tyrosine kinase(LYN)	Homo sapiens
440	11718086_s_at	turquoise	SH3KBP1 binding protein 1(SHKBP1)	Homo sapiens
441	11718099_a_at	turquoise	intraflagellar transport 20(IFT20)	Homo sapiens
443	11718121_x_at	turquoise	ATPase H+ transporting V1 subunit A(ATP6V1A)	Homo sapiens
444	11718134_a_at	turquoise	phosphoribosyl pyrophosphate synthetase 2(PRPS2)	Homo sapiens
445	11718135_at	turquoise	phosphoribosyl pyrophosphate synthetase 2(PRPS2)	Homo sapiens
446	11718140_a_at	turquoise	epithelial membrane protein 3(EMP3)	Homo sapiens
448	11718152_a_at	turquoise	PPFIA binding protein 2(PPFIBP2)	Homo sapiens
449	11718158_a_at	turquoise	N-myc and STAT interactor(NMI)	Homo sapiens
450	11718159_at	turquoise	N-myc and STAT interactor(NMI)	Homo sapiens
451	11718184_a_at	turquoise	FCH and double SH3 domains 2(FCHSD2)	Homo sapiens
452	11718186_a_at	turquoise	ubiquitin like with PHD and ring finger domains 2(UHRF2)	Homo sapiens
453	11718204_a_at	turquoise	REC8 meiotic recombination protein(REC8)	Homo sapiens
456	11718230_a_at	turquoise	major histocompatibility complex, class I, F(HLA-F)	Homo sapiens
457	11718231_x_at	turquoise	major histocompatibility complex, class I, F(HLA-F)	Homo sapiens
459	11718245_at	turquoise	solute carrier family 25 member 42(SLC25A42)	Homo sapiens
460	11718265_a_at	turquoise	SMAD family member 3(SMAD3)	Homo sapiens
461	11718266_s_at	turquoise	SMAD family member 3(SMAD3)	Homo sapiens
465	11718290_a_at	turquoise	FXYD domain containing ion transport regulator 5(FXYD5)	Homo sapiens
466	11718293_a_at	turquoise	cyclin dependent kinase inhibitor 2A(CDKN2A)	Homo sapiens
467	11718297_a_at	turquoise	transforming acidic coiled-coil containing protein 3(TACC3)	Homo sapiens
468	11718303_a_at	turquoise	platelet activating factor acetylhydrolase 1b catalytic subunit 3(PAFAH1B3)	Homo sapiens
470	11718319_at	turquoise	CD93 molecule(CD93)	Homo sapiens
471	11718322_at	turquoise	ArfGAP with coiled-coil, ankyrin repeat and PH domains 2(ACAP2)	Homo sapiens
472	11718325_at	turquoise	sestrin 2(SESN2)	Homo sapiens
473	11718340_x_at	turquoise	DAZ associated protein 2(DAZAP2)	Homo sapiens
474	11718370_a_at	turquoise	nuclear receptor binding factor 2(NRBF2)	Homo sapiens
475	11718381_a_at	turquoise	pleckstrin and Sec7 domain containing 4(PSD4)	Homo sapiens
476	11718382_x_at	turquoise	poly(ADP-ribose) polymerase family member 4(PARP4)	Homo sapiens
478	11718400_a_at	turquoise	purine nucleoside phosphorylase(PNP)	Homo sapiens
479	11718415_x_at	turquoise	non-POU domain containing, octamer-binding(NONO)	Homo sapiens
480	11718417_a_at	turquoise	ECSIT signalling integrator(ECSIT)	Homo sapiens
481	11718418_a_at	turquoise	thiosulfate sulfurtransferase(TST)	Homo sapiens
482	11718419_at	turquoise	Fc fragment of IgE receptor Ig(FCER1G)	Homo sapiens
483	11718420_a_at	turquoise	CLPTM1 like(CLPTM1L)	Homo sapiens
484	11718425_a_at	turquoise	hydroxysteroid dehydrogenase like 2(HSDL2)	Homo sapiens
487	11718436_a_at	turquoise	ATP synthase mitochondrial F1 complex assembly factor 2(ATPAF2)	Homo sapiens
488	11718450_at	turquoise	alpha-N-acetylgalactosaminidase(NAGA)	Homo sapiens
489	11718461_at	turquoise	solute carrier family 39 member 11(SLC39A11)	Homo sapiens
490	11718468_s_at	turquoise	N-terminal EF-hand calcium binding protein 3(NECAB3)	Homo sapiens
491	11718512_x_at	turquoise	tetraspanin 14(TSPAN14)	Homo sapiens
492	11718513_x_at	turquoise	tetraspanin 14(TSPAN14)	Homo sapiens
493	11718514_s_at	turquoise	tetraspanin 14(TSPAN14)	Homo sapiens
494	11718515_a_at	turquoise	sulfide quinone reductase-like (yeast)(SQRDL)	Homo sapiens
495	11718520_at	turquoise	potassium channel modulatory factor 1(KCMF1)	Homo sapiens
496	11718525_s_at	turquoise	non imprinted in Prader-Willi/Angelman syndrome 2(NIPA2)	Homo sapiens
497	11718528_a_at	turquoise	torsin family 3 member A(TOR3A)	Homo sapiens
498	11718534_at	turquoise	natural killer cell triggering receptor(NKTR)	Homo sapiens
499	11718537_s_at	turquoise	fumarylacetoacetate hydrolase domain containing 2B(FAHD2B)	Homo sapiens
500	11718538_x_at	turquoise	fumarylacetoacetate hydrolase domain containing 2A(FAHD2A)	Homo sapiens
501	11718540_at	turquoise	transmembrane protein 243(TMEM243)	Homo sapiens
502	11718546_s_at	turquoise	interleukin enhancer binding factor 3(ILF3)	Homo sapiens
503	11718559_at	turquoise	nucleolar protein 4 like(NOL4L)	Homo sapiens

504	11718563_a_at	turquoise	ankyrin repeat domain 10(ANKRD10)	Homo sapiens
505	11718568_a_at	turquoise	WAS/WASL interacting protein family member 1(WIPF1)	Homo sapiens
506	11718569_a_at	turquoise	WAS/WASL interacting protein family member 1(WIPF1)	Homo sapiens
508	11718591_at	turquoise	stromal cell derived factor 2 like 1(SDF2L1)	Homo sapiens
509	11718592_s_at	turquoise	reticulon 4(RTN4)	Homo sapiens
510	11718594_a_at	turquoise	cyclin L1(CCNL1)	Homo sapiens
511	11718608_at	turquoise	microRNA 5193(MIR5193)	Homo sapiens
512	11718610_at	turquoise	tumor protein p53 inducible nuclear protein 1(TP53INP1)	Homo sapiens
513	11718621_at	turquoise	dual specificity phosphatase 12(DUSP12)	Homo sapiens
514	11718625_a_at	turquoise	deoxyguanosine kinase(DGUOK)	Homo sapiens
515	11718631_at	turquoise	SERTA domain containing 1(SERTAD1)	Homo sapiens
516	11718641_a_at	turquoise	N-deacetylase and N-sulfotransferase 2(NDST2)	Homo sapiens
517	11718652_a_at	turquoise	signal transducer and activator of transcription 2(STAT2)	Homo sapiens
518	11718655_s_at	turquoise	charged multivesicular body protein 5(CHMP5)	Homo sapiens
519	11718657_a_at	turquoise	microRNA 21(MIR21)	Homo sapiens
520	11718659_x_at	turquoise	Mov10 RISC complex RNA helicase(MOV10)	Homo sapiens
521	11718665_s_at	turquoise	glutamyl-tRNA amidotransferase subunit C(GATC)	Homo sapiens
522	11718666_x_at	turquoise	glutamyl-tRNA amidotransferase subunit C(GATC)	Homo sapiens
524	11718670_at	turquoise	mediator complex subunit 9(MED9)	Homo sapiens
525	11718672_x_at	turquoise	interferon regulatory factor 3(IRF3)	Homo sapiens
526	11718674_a_at	turquoise	transmembrane protein 245(TMEM245)	Homo sapiens
527	11718678_a_at	turquoise	presenilin 1(PSEN1)	Homo sapiens
528	11718680_a_at	turquoise	adhesion G protein-coupled receptor E5(ADGRE5)	Homo sapiens
529	11718699_at	turquoise	chromosome 6 open reading frame 89(C6orf89)	Homo sapiens
530	11718757_s_at	turquoise	hypoxia inducible factor 1 alpha subunit(HIF1A)	Homo sapiens
533	11718766_at	turquoise	protease, serine 23(PRSS23)	Homo sapiens
534	11718767_x_at	turquoise	protease, serine 23(PRSS23)	Homo sapiens
535	11718788_a_at	turquoise	adenosine deaminase(ADA)	Homo sapiens
536	11718807_at	turquoise	TNF receptor superfamily member 1B(TNFRSF1B)	Homo sapiens
537	11718809_a_at	turquoise	protein kinase C eta(PRKCH)	Homo sapiens
538	11718810_at	turquoise	protein kinase C eta(PRKCH)	Homo sapiens
539	11718811_s_at	turquoise	protein kinase C eta(PRKCH)	Homo sapiens
540	11718830_s_at	turquoise	EH domain containing 1(EHD1)	Homo sapiens
542	11718832_a_at	turquoise	lysyl oxidase like 2(LOXL2)	Homo sapiens
544	11718849_at	turquoise	protein tyrosine phosphatase, receptor type C associated protein(PTPRCAP)	Homo sapiens
546	11718860_a_at	turquoise	Suv3 like RNA helicase(SUPV3L1)	Homo sapiens
547	11718861_a_at	turquoise	HCK proto-oncogene, Src family tyrosine kinase(HCK)	Homo sapiens
548	11718865_at	turquoise	COX10, heme A:farnesyltransferase cytochrome c oxidase assembly factor(COX10)	Homo sapiens
549	11718904_s_at	turquoise	filamin A interacting protein 1 like(FILIP1L)	Homo sapiens
550	11718908_s_at	turquoise	carbohydrate sulfotransferase 2(CHST2)	Homo sapiens
551	11718909_x_at	turquoise	carbohydrate sulfotransferase 2(CHST2)	Homo sapiens
552	11718912_a_at	turquoise	PDZ domain containing 2(PDZD2)	Homo sapiens
553	11718914_x_at	turquoise	PDZ domain containing 2(PDZD2)	Homo sapiens
554	11718915_a_at	turquoise	regulator of G-protein signaling 19(RGS19)	Homo sapiens
555	11718916_a_at	turquoise	interferon regulatory factor 7(IRF7)	Homo sapiens
556	11718924_a_at	turquoise	WW domain containing E3 ubiquitin protein ligase 2(WWP2)	Homo sapiens
557	11718929_a_at	turquoise	TGFB induced factor homeobox 2(TGIF2)	Homo sapiens
558	11718930_a_at	turquoise	TGFB induced factor homeobox 2(TGIF2)	Homo sapiens
559	11718935_x_at	turquoise	serine/threonine kinase 19(STK19)	Homo sapiens
560	11718939_s_at	turquoise	TNF alpha induced protein 3(TNFAIP3)	Homo sapiens
561	11718940_a_at	turquoise	TNF alpha induced protein 3(TNFAIP3)	Homo sapiens
563	11718950_s_at	turquoise	membrane palmitoylated protein 5(MPP5)	Homo sapiens
564	11718954_a_at	turquoise	filamin binding LIM protein 1(FBLIM1)	Homo sapiens
565	11718958_s_at	turquoise	protein kinase AMP-activated non-catalytic subunit beta 2(PRKAB2)	Homo sapiens
566	11718981_a_at	turquoise	transgelin 2(TAGLN2)	Homo sapiens
567	11718982_s_at	turquoise	C-C motif chemokine ligand 4 like 1(CCL4L1)	Homo sapiens
568	11718983_x_at	turquoise	C-C motif chemokine ligand 4 like 1(CCL4L1)	Homo sapiens
569	11718986_a_at	turquoise	interferon alpha inducible protein 6(IFI6)	Homo sapiens
571	11718996_at	turquoise	methionine sulfoxide reductase B2(MSRB2)	Homo sapiens
572	11719016_at	turquoise	PQ loop repeat containing 3(PQLC3)	Homo sapiens
573	11719020_at	turquoise	WD repeat and FYVE domain containing 1(WDFY1)	Homo sapiens
574	11719021_at	turquoise	WD repeat and FYVE domain containing 1(WDFY1)	Homo sapiens
575	11719029_at	turquoise	phosphoinositide-3-kinase adaptor protein 1(PIK3AP1)	Homo sapiens
576	11719038_a_at	turquoise	ArfGAP with RhoGAP domain, ankyrin repeat and PH domain 1(ARAP1)	Homo sapiens
577	11719046_a_at	turquoise	SNF related kinase(SNRK)	Homo sapiens
578	11719050_a_at	turquoise	butyrophilin subfamily 2 member A1(BTN2A1)	Homo sapiens
580	11719076_a_at	turquoise	receptor accessory protein 1(REEP1)	Homo sapiens
581	11719083_x_at	turquoise	TruB pseudouridine synthase family member 2(TRUB2)	Homo sapiens
582	11719089_a_at	turquoise	phosphoenolpyruvate carboxykinase 2, mitochondrial(PCK2)	Homo sapiens
587	11719120_a_at	turquoise	kynureninase(KYNU)	Homo sapiens
588	11719123_a_at	turquoise	timeless circadian clock(TIMELESS)	Homo sapiens
589	11719126_a_at	turquoise	cell growth regulator with ring finger domain 1(CGRRF1)	Homo sapiens
590	11719128_a_at	turquoise	lipase maturation factor 2(LMF2)	Homo sapiens
591	11719132_s_at	turquoise	family with sequence similarity 49 member B(FAM49B)	Homo sapiens
592	11719154_a_at	turquoise	DNA damage regulated autophagy modulator 1(DRAM1)	Homo sapiens
593	11719155_a_at	turquoise	DNA damage regulated autophagy modulator 1(DRAM1)	Homo sapiens
594	11719164_a_at	turquoise	chloride voltage-gated channel 5(CLCN5)	Homo sapiens
595	11719182_a_at	turquoise	chloride voltage-gated channel 7(CLCN7)	Homo sapiens
596	11719188_at	turquoise	neuroblastoma RAS viral oncogene homolog(NRAS)	Homo sapiens
597	11719189_at	turquoise	neuroblastoma RAS viral oncogene homolog(NRAS)	Homo sapiens
598	11719193_a_at	turquoise	chibby family member 1, beta catenin antagonist(CBY1)	Homo sapiens
599	11719211_a_at	turquoise	heat shock transcription factor 2(HSF2)	Homo sapiens

600	11719216_a_at	turquoise	tudor domain containing 7(TDRD7)	Homo sapiens
601	11719218_at	turquoise	suppressor of cytokine signaling 3(SOCS3)	Homo sapiens
603	11719227_x_at	turquoise	potassium calcium-activated channel subfamily N member 4(KCNN4)	Homo sapiens
605	11719245_a_at	turquoise	LARGE xylosyl- and glucuronyltransferase 1(LARGE1)	Homo sapiens
606	11719247_at	turquoise	absent in melanoma 1(AIM1)	Homo sapiens
607	11719248_a_at	turquoise	absent in melanoma 1(AIM1)	Homo sapiens
608	11719258_at	turquoise	toll like receptor adaptor molecule 1(TICAM1)	Homo sapiens
609	11719270_s_at	turquoise	RNA binding motif protein 22(RBM22)	Homo sapiens
610	11719271_s_at	turquoise	glucuronidase beta(GUSB)	Homo sapiens
612	11719305_at	turquoise	synaptopodin(SYNPO)	Homo sapiens
613	11719313_a_at	turquoise	family with sequence similarity 111 member A(FAM111A)	Homo sapiens
614	11719327_a_at	turquoise	cysteine rich with EGF like domains 2(CRELD2)	Homo sapiens
615	11719332_at	turquoise	uncharacterized LOC101928361(LOC101928361)	Homo sapiens
616	11719347_a_at	turquoise	Rho GTPase activating protein 19(ARHGAP19)	Homo sapiens
617	11719354_s_at	turquoise	SH3 domain binding protein 5(SH3BP5)	Homo sapiens
618	11719355_s_at	turquoise	SH3 domain binding protein 5(SH3BP5)	Homo sapiens
620	11719366_s_at	turquoise	C-X-C motif chemokine ligand 1(CXCL1)	Homo sapiens
621	11719367_a_at	turquoise	pleckstrin homology domain containing O2(PLEKHO2)	Homo sapiens
622	11719372_s_at	turquoise	brain expressed X-linked 3(BEX3)	Homo sapiens
623	11719377_x_at	turquoise	adenylate cyclase associated protein 1(CAP1)	Homo sapiens
624	11719378_a_at	turquoise	Rap1 GTPase-GDP dissociation stimulator 1(RAP1GDS1)	Homo sapiens
625	11719379_a_at	turquoise	Rap1 GTPase-GDP dissociation stimulator 1(RAP1GDS1)	Homo sapiens
626	11719398_s_at	turquoise	Ras related GTP binding C(RRAGC)	Homo sapiens
627	11719402_a_at	turquoise	RAP2A, member of RAS oncogene family(RAP2A)	Homo sapiens
628	11719411_at	turquoise	RAB32, member RAS oncogene family(RAB32)	Homo sapiens
629	11719413_a_at	turquoise	PBX homeobox 1(PBX1)	Homo sapiens
632	11719435_a_at	turquoise	LDL receptor related protein 4(LRP4)	Homo sapiens
633	11719438_at	turquoise	SLIT-ROBO Rho GTPase activating protein 2(SRGAP2)	Homo sapiens
634	11719447_s_at	turquoise	guanylate binding protein 2(GBP2)	Homo sapiens
635	11719465_a_at	turquoise	complement C1q B chain(C1QB)	Homo sapiens
636	11719466_s_at	turquoise	complement C1q B chain(C1QB)	Homo sapiens
637	11719479_at	turquoise	arachidonate 5-lipoxygenase activating protein(ALOX5AP)	Homo sapiens
638	11719480_a_at	turquoise	cystatin A(CSTA)	Homo sapiens
640	11719483_a_at	turquoise	purinergic receptor P2X 4(P2RX4)	Homo sapiens
641	11719491_a_at	turquoise	interferon induced protein 35(IFI35)	Homo sapiens
642	11719492_s_at	turquoise	interferon induced protein 35(IFI35)	Homo sapiens
643	11719499_at	turquoise	monoamine oxidase B(MAOB)	Homo sapiens
644	11719501_a_at	turquoise	nuclear factor of activated T-cells 3(NFATC3)	Homo sapiens
645	11719513_a_at	turquoise	ADAM metallopeptidase domain 15(ADAM15)	Homo sapiens
646	11719528_at	turquoise	nectin cell adhesion molecule 2(NECTIN2)	Homo sapiens
647	11719539_a_at	turquoise	nuclear envelope integral membrane protein 1(NEMP1)	Homo sapiens
648	11719543_a_at	turquoise	chromosome 3 open reading frame 18(C3orf18)	Homo sapiens
649	11719554_at	turquoise	cathepsin S(CTSS)	Homo sapiens
650	11719556_a_at	turquoise	pyruvate dehydrogenase kinase 2(PDK2)	Homo sapiens
652	11719579_a_at	turquoise	translational activator of cytochrome c oxidase I(TACO1)	Homo sapiens
653	11719587_a_at	turquoise	MICAL like 1(MICALL1)	Homo sapiens
654	11719588_a_at	turquoise	2'-5'-oligoadenylate synthetase 1(OAS1)	Homo sapiens
655	11719591_s_at	turquoise	glycolipid transfer protein(GLTP)	Homo sapiens
656	11719599_a_at	turquoise	homer scaffolding protein 2(HOMER2)	Homo sapiens
657	11719629_a_at	turquoise	bromodomain containing 2(BRD2)	Homo sapiens
658	11719630_a_at	turquoise	bromodomain containing 2(BRD2)	Homo sapiens
659	11719631_s_at	turquoise	bridging integrator 1(BIN1)	Homo sapiens
660	11719644_a_at	turquoise	GATA zinc finger domain containing 2A(GATAD2A)	Homo sapiens
661	11719648_a_at	turquoise	caspase 7(CASP7)	Homo sapiens
662	11719657_a_at	turquoise	matrix metallopeptidase 9(MMP9)	Homo sapiens
663	11719675_a_at	turquoise	vascular cell adhesion molecule 1(VCAM1)	Homo sapiens
664	11719676_s_at	turquoise	monoamine oxidase A(MAOA)	Homo sapiens
665	11719680_x_at	turquoise	TNF receptor superfamily member 10b(TNFRSF10B)	Homo sapiens
666	11719686_a_at	turquoise	microtubule associated monooxygenase, calponin and LIM domain containing 1(MICAL1)	Homo sapiens
667	11719687_at	turquoise	SET domain bifurcated 1(SETDB1)	Homo sapiens
668	11719692_a_at	turquoise	retinoic acid receptor responder 3(RARRES3)	Homo sapiens
669	11719718_a_at	turquoise	spleen associated tyrosine kinase(SYK)	Homo sapiens
670	11719728_s_at	turquoise	signal regulatory protein alpha(SIRPA)	Homo sapiens
671	11719738_at	turquoise	decapping mRNA 2(DCP2)	Homo sapiens
672	11719739_s_at	turquoise	decapping mRNA 2(DCP2)	Homo sapiens
673	11719745_s_at	turquoise	Rho GTPase activating protein 27(ARHGAP27)	Homo sapiens
674	11719749_a_at	turquoise	emopamil binding protein like(EBPL)	Homo sapiens
675	11719754_s_at	turquoise	interleukin 1 receptor antagonist(IL1RN)	Homo sapiens
676	11719763_a_at	turquoise	vacuolar protein sorting 13 homolog C(VPS13C)	Homo sapiens
677	11719780_at	turquoise	TNF alpha induced protein 8 like 2(TNFAIP8L2)	Homo sapiens
678	11719800_at	turquoise	chromosome 6 open reading frame 62(C6orf62)	Homo sapiens
679	11719801_a_at	turquoise	chromosome 6 open reading frame 62(C6orf62)	Homo sapiens
680	11719827_a_at	turquoise	glutaminyl-peptide cyclotransferase(QPCT)	Homo sapiens
681	11719828_x_at	turquoise	glutaminyl-peptide cyclotransferase(QPCT)	Homo sapiens
683	11719833_at	turquoise	myelin protein zero like 2(MPZL2)	Homo sapiens
684	11719838_a_at	turquoise	caspase 3(CASP3)	Homo sapiens
685	11719844_at	turquoise	small ArfGAP2(SMAP2)	Homo sapiens
686	11719845_a_at	turquoise	small ArfGAP2(SMAP2)	Homo sapiens
687	11719861_x_at	turquoise	SLC2A4 regulator(SLC2A4RG)	Homo sapiens
688	11719864_a_at	turquoise	small cell adhesion glycoprotein(SMAGP)	Homo sapiens
689	11719868_a_at	turquoise	v-myc avian myelocytomatosis viral oncogene neuroblastoma derived homolog(MYCN)	Homo sapiens
690	11719869_a_at	turquoise	v-myc avian myelocytomatosis viral oncogene neuroblastoma derived homolog(MYCN)	Homo sapiens

691	11719870_s_at	turquoise	v-myc avian myelocytomatosis viral oncogene neuroblastoma derived homolog(MYCN)	Homo sapiens
694	11719886_a_at	turquoise	interferon induced protein 44(IFI44)	Homo sapiens
695	11719887_a_at	turquoise	GRB10 interacting GYF protein 2(GIGYF2)	Homo sapiens
696	11719892_at	turquoise	vasohibin 1(VASH1)	Homo sapiens
697	11719894_s_at	turquoise	family with sequence similarity 91 member A1(FAM91A1)	Homo sapiens
698	11719895_x_at	turquoise	family with sequence similarity 91 member A1(FAM91A1)	Homo sapiens
699	11719897_x_at	turquoise	family with sequence similarity 91 member A1(FAM91A1)	Homo sapiens
700	11719902_a_at	turquoise	PHD finger protein 11(PHF11)	Homo sapiens
701	11719910_a_at	turquoise	phosphatidylinositol binding clathrin assembly protein(PICALM)	Homo sapiens
702	11719916_at	turquoise	interleukin 1 beta(IL1B)	Homo sapiens
703	11719938_s_at	turquoise	cullin associated and neddylation dissociated 1(CAND1)	Homo sapiens
704	11719939_a_at	turquoise	cullin associated and neddylation dissociated 1(CAND1)	Homo sapiens
705	11719943_at	turquoise	C-X-C motif chemokine ligand 9(CXCL9)	Homo sapiens
706	11719953_s_at	turquoise	FK506 binding protein 15(FKBP15)	Homo sapiens
707	11719972_at	turquoise	SCO2, cytochrome c oxidase assembly protein(SCO2)	Homo sapiens
708	11719973_a_at	turquoise	A-kinase anchoring protein 17A(AKAP17A)	Homo sapiens
709	11719976_at	turquoise	WAS protein family member 3(WASF3)	Homo sapiens
710	11719980_a_at	turquoise	formin like 2(FMNL2)	Homo sapiens
711	11719995_a_at	turquoise	transducin like enhancer of split 4(TLE4)	Homo sapiens
712	11719996_at	turquoise	transducin like enhancer of split 4(TLE4)	Homo sapiens
713	11720014_a_at	turquoise	ATM serine/threonine kinase(ATM)	Homo sapiens
714	11720029_a_at	turquoise	low density lipoprotein receptor(LDLR)	Homo sapiens
715	11720034_x_at	turquoise	caspase 2(CASP2)	Homo sapiens
716	11720035_s_at	turquoise	caspase 2(CASP2)	Homo sapiens
717	11720043_a_at	turquoise	abhydrolase domain containing 16A(ABHD16A)	Homo sapiens
718	11720044_at	turquoise	src kinase associated phosphoprotein 2(SKAP2)	Homo sapiens
719	11720063_a_at	turquoise	GLI pathogenesis related 2(GLIPR2)	Homo sapiens
720	11720064_a_at	turquoise	GLI pathogenesis related 2(GLIPR2)	Homo sapiens
721	11720096_a_at	turquoise	LIM domain containing 2(LIMD2)	Homo sapiens
722	11720098_a_at	turquoise	centrosomal protein 70(CEP70)	Homo sapiens
723	11720103_a_at	turquoise	p21 (RAC1) activated kinase 1(PAK1)	Homo sapiens
724	11720104_a_at	turquoise	p21 (RAC1) activated kinase 1(PAK1)	Homo sapiens
725	11720107_s_at	turquoise	kinesin family member 2A(KIF2A)	Homo sapiens
726	11720111_at	turquoise	syntrophin beta 2(SNTB2)	Homo sapiens
727	11720112_at	turquoise	syntrophin beta 2(SNTB2)	Homo sapiens
728	11720113_at	turquoise	syntrophin beta 2(SNTB2)	Homo sapiens
730	11720115_a_at	turquoise	serine/threonine protein kinase 26(STK26)	Homo sapiens
731	11720117_a_at	turquoise	signal-induced proliferation-associated 1(SIPA1)	Homo sapiens
733	11720143_at	turquoise	centriole, cilia and spindle associated protein(CCSAP)	Homo sapiens
734	11720146_a_at	turquoise	death associated protein kinase 1(DAPK1)	Homo sapiens
735	11720153_s_at	turquoise	nuclear receptor interacting protein 1(NRIP1)	Homo sapiens
736	11720161_at	turquoise	C-X-C motif chemokine ligand 13(CXCL13)	Homo sapiens
737	11720163_at	turquoise	vascular endothelial growth factor C(VEGFC)	Homo sapiens
738	11720167_at	turquoise	selectin L(SELL)	Homo sapiens
739	11720168_at	turquoise	selectin L(SELL)	Homo sapiens
741	11720187_a_at	turquoise	solute carrier family 27 member 3(SLC27A3)	Homo sapiens
746	11720197_a_at	turquoise	translocase of outer mitochondrial membrane 40 like(TOMM40L)	Homo sapiens
747	11720204_at	turquoise	5'-nucleotidase domain containing 1(NT5DC1)	Homo sapiens
748	11720206_s_at	turquoise	claudin 12(CLDN12)	Homo sapiens
749	11720207_a_at	turquoise	interleukin 2 receptor subunit gamma(IL2RG)	Homo sapiens
750	11720208_a_at	turquoise	interferon regulatory factor 9(IRF9)	Homo sapiens
751	11720209_at	turquoise	interferon regulatory factor 9(IRF9)	Homo sapiens
752	11720218_a_at	turquoise	mitogen-activated protein kinase kinase kinase 3(MAP3K3)	Homo sapiens
753	11720219_a_at	turquoise	mitogen-activated protein kinase kinase kinase 3(MAP3K3)	Homo sapiens
754	11720230_at	turquoise	testin LIM domain protein(TES)	Homo sapiens
755	11720231_s_at	turquoise	testin LIM domain protein(TES)	Homo sapiens
756	11720237_s_at	turquoise	mitogen-activated protein kinase 9(MAPK9)	Homo sapiens
758	11720243_x_at	turquoise	transcobalamin 2(TCN2)	Homo sapiens
759	11720244_at	turquoise	phospholipase C gamma 2(PLCG2)	Homo sapiens
760	11720247_a_at	turquoise	centrosomal protein 55(CEP55)	Homo sapiens
761	11720254_a_at	turquoise	cyclin and CBS domain divalent metal cation transport mediator 4(CNNM4)	Homo sapiens
762	11720264_at	turquoise	activating signal cointegrator 1 complex subunit 3(ASCC3)	Homo sapiens
763	11720280_at	turquoise	potassium calcium-activated channel subfamily M alpha 1(KCNMA1)	Homo sapiens
764	11720285_s_at	turquoise	trafficking kinesin protein 1(TRAK1)	Homo sapiens
765	11720298_at	turquoise	C-X-C motif chemokine ligand 10(CXCL10)	Homo sapiens
766	11720300_a_at	turquoise	Src-like-adaptor(SLA)	Homo sapiens
767	11720301_a_at	turquoise	Src-like-adaptor(SLA)	Homo sapiens
768	11720302_a_at	turquoise	Src-like-adaptor(SLA)	Homo sapiens
769	11720306_at	turquoise	stress associated endoplasmic reticulum protein 1(SERP1)	Homo sapiens
770	11720307_s_at	turquoise	stress associated endoplasmic reticulum protein 1(SERP1)	Homo sapiens
771	11720308_x_at	turquoise	stress associated endoplasmic reticulum protein 1(SERP1)	Homo sapiens
772	11720309_s_at	turquoise	stress associated endoplasmic reticulum protein 1(SERP1)	Homo sapiens
773	11720320_at	turquoise	adenylate kinase 2(AK2)	Homo sapiens
774	11720323_s_at	turquoise	adenylate kinase 2(AK2)	Homo sapiens
776	11720364_a_at	turquoise	GIMAP1-GIMAP5 readthrough(GIMAP1-GIMAP5)	Homo sapiens
777	11720367_a_at	turquoise	transmembrane protein 2(TMEM2)	Homo sapiens
778	11720373_a_at	turquoise	prune exopolyphosphatase(PRUNE1)	Homo sapiens
779	11720380_a_at	turquoise	inosine monophosphate dehydrogenase 1(IMPDH1)	Homo sapiens
780	11720382_at	turquoise	syntaxin 6(STX6)	Homo sapiens
781	11720384_at	turquoise	syntaxin 6(STX6)	Homo sapiens
782	11720388_s_at	turquoise	complement C1q C chain(C1QC)	Homo sapiens
783	11720395_a_at	turquoise	nibrin(NBN)	Homo sapiens

784	11720396_x_at	turquoise	nibrin(NBN)	Homo sapiens
785	11720398_a_at	turquoise	nibrin(NBN)	Homo sapiens
786	11720427_a_at	turquoise	ilvB acetolactate synthase like(ILVBL)	Homo sapiens
787	11720430_a_at	turquoise	ORMDL sphingolipid biosynthesis regulator 1(ORMDL1)	Homo sapiens
788	11720441_x_at	turquoise	olfactomedin like 2B(OLFML2B)	Homo sapiens
789	11720443_s_at	turquoise	bromodomain adjacent to zinc finger domain 1A(BAZ1A)	Homo sapiens
790	11720444_s_at	turquoise	ER lipid raft associated 1(ERLIN1)	Homo sapiens
791	11720459_s_at	turquoise	cell cycle associated protein 1(CAPRIN1)	Homo sapiens
792	11720460_x_at	turquoise	F11 receptor(F11R)	Homo sapiens
793	11720464_x_at	turquoise	F11 receptor(F11R)	Homo sapiens
795	11720493_s_at	turquoise	MOB kinase activator 3A(MOB3A)	Homo sapiens
796	11720496_at	turquoise	granzyme A(GZMA)	Homo sapiens
797	11720501_a_at	turquoise	ubiquitination factor E4B(UBE4B)	Homo sapiens
798	11720502_s_at	turquoise	ubiquitination factor E4B(UBE4B)	Homo sapiens
799	11720504_s_at	turquoise	RAB6B, member RAS oncogene family(RAB6B)	Homo sapiens
800	11720510_a_at	turquoise	apolipoprotein B mRNA editing enzyme catalytic subunit 3G(APOBEC3G)	Homo sapiens
801	11720511_a_at	turquoise	calcium regulated heat stable protein 1(CARHSP1)	Homo sapiens
803	11720537_at	turquoise	methylmalonic aciduria (cobalamin deficiency) cblB type(MMAB)	Homo sapiens
804	11720538_at	turquoise	feline leukemia virus subgroup C cellular receptor family member 2(FLVCR2)	Homo sapiens
805	11720551_a_at	turquoise	intermediate filament family orphan 1(IFFO1)	Homo sapiens
806	11720552_a_at	turquoise	intermediate filament family orphan 1(IFFO1)	Homo sapiens
807	11720554_a_at	turquoise	Rho/Rac guanine nucleotide exchange factor 18(ARHGEF18)	Homo sapiens
808	11720566_at	turquoise	branched chain amino acid transaminase 1(BCAT1)	Homo sapiens
809	11720570_a_at	turquoise	jade family PHD finger 2(JADE2)	Homo sapiens
810	11720573_at	turquoise	ATPase phospholipid transporting 8B1(ATP8B1)	Homo sapiens
811	11720574_s_at	turquoise	ATPase phospholipid transporting 8B1(ATP8B1)	Homo sapiens
812	11720587_s_at	turquoise	structural maintenance of chromosomes flexible hinge domain containing 1(SMCHD1)	Homo sapiens
813	11720602_at	turquoise	synaptotagmin 11(SYT11)	Homo sapiens
814	11720609_at	turquoise	prohibitin(PHB)	Homo sapiens
816	11720621_s_at	turquoise	ATP citrate lyase(ACLY)	Homo sapiens
817	11720623_a_at	turquoise	myeloid differentiation primary response 88(MYD88)	Homo sapiens
818	11720624_a_at	turquoise	myeloid differentiation primary response 88(MYD88)	Homo sapiens
823	11720644_a_at	turquoise	platelet and endothelial cell adhesion molecule 1(PECAM1)	Homo sapiens
824	11720646_at	turquoise	serine palmitoyltransferase long chain base subunit 2(SPTLC2)	Homo sapiens
825	11720657_x_at	turquoise	major histocompatibility complex, class II, DR beta 5(HLA-DRB5)	Homo sapiens
826	11720672_a_at	turquoise	protein phosphatase, Mg2+/Mn2+ dependent 1A(PPM1A)	Homo sapiens
829	11720679_a_at	turquoise	CKLF-CMTM1 readthrough(CKLF-CMTM1)	Homo sapiens
830	11720680_a_at	turquoise	diacylglycerol kinase alpha(DGKA)	Homo sapiens
831	11720694_a_at	turquoise	HEN1 methyltransferase homolog 1(HENMT1)	Homo sapiens
832	11720695_at	turquoise	chromosome 1 open reading frame 54(C1orf54)	Homo sapiens
833	11720725_at	turquoise	zyg-11 family member B, cell cycle regulator(ZYG11B)	Homo sapiens
834	11720747_a_at	turquoise	adenylate cyclase 7(ADCY7)	Homo sapiens
835	11720751_a_at	turquoise	inositol polyphosphate-1-phosphatase(INPP1)	Homo sapiens
836	11720754_x_at	turquoise	actin beta(ACTB)	Homo sapiens
837	11720755_at	turquoise	signal transducer and activator of transcription 3(STAT3)	Homo sapiens
838	11720756_at	turquoise	signal transducer and activator of transcription 3(STAT3)	Homo sapiens
839	11720763_a_at	turquoise	selectin P ligand(SELPLG)	Homo sapiens
840	11720769_a_at	turquoise	peptidylprolyl isomerase F(PPIF)	Homo sapiens
846	11720793_x_at	turquoise	acetoacetyl-CoA synthetase(AACS)	Homo sapiens
847	11720798_at	turquoise	RAB8B, member RAS oncogene family(RAB8B)	Homo sapiens
848	11720799_s_at	turquoise	RAB8B, member RAS oncogene family(RAB8B)	Homo sapiens
849	11720800_a_at	turquoise	RAB8B, member RAS oncogene family(RAB8B)	Homo sapiens
850	11720802_s_at	turquoise	bridging integrator 3(BIN3)	Homo sapiens
853	11720827_a_at	turquoise	centromere protein W(CENPW)	Homo sapiens
854	11720832_x_at	turquoise	SRY-box 18(SOX18)	Homo sapiens
855	11720842_at	turquoise	ras homolog family member D(RHOD)	Homo sapiens
856	11720847_s_at	turquoise	PHD finger protein 6(PHF6)	Homo sapiens
857	11720859_s_at	turquoise	abhydrolase domain containing 3(ABHD3)	Homo sapiens
858	11720860_s_at	turquoise	Rho guanine nucleotide exchange factor 3(ARHGEF3)	Homo sapiens
859	11720861_a_at	turquoise	Rho guanine nucleotide exchange factor 3(ARHGEF3)	Homo sapiens
861	11720867_x_at	turquoise	placenta specific 8(PLAC8)	Homo sapiens
863	11720891_a_at	turquoise	ubiquitin specific peptidase 20(USP20)	Homo sapiens
864	11720893_s_at	turquoise	SOS Ras/Rac guanine nucleotide exchange factor 1(SOS1)	Homo sapiens
865	11720907_x_at	turquoise	semaphorin 3F(SEMA3F)	Homo sapiens
866	11720909_a_at	turquoise	transmembrane protein 44(TMEM44)	Homo sapiens
867	11720910_a_at	turquoise	neural EGFL like 2(NELL2)	Homo sapiens
868	11720922_a_at	turquoise	HLA complex P5 (non-protein coding)(HCP5)	Homo sapiens
869	11720923_a_at	turquoise	HLA complex P5 (non-protein coding)(HCP5)	Homo sapiens
870	11720943_a_at	turquoise	regulator of G-protein signaling 16(RGS16)	Homo sapiens
871	11720944_at	turquoise	regulator of G-protein signaling 16(RGS16)	Homo sapiens
872	11720963_a_at	turquoise	deoxycytidine kinase(DCK)	Homo sapiens
873	11720964_s_at	turquoise	deoxycytidine kinase(DCK)	Homo sapiens
874	11720965_at	turquoise	deoxycytidine kinase(DCK)	Homo sapiens
875	11720966_x_at	turquoise	deoxycytidine kinase(DCK)	Homo sapiens
876	11720970_at	turquoise	topoisomerase (DNA) II alpha(TOP2A)	Homo sapiens
877	11720971_at	turquoise	topoisomerase (DNA) II alpha(TOP2A)	Homo sapiens
878	11720972_at	turquoise	topoisomerase (DNA) II alpha(TOP2A)	Homo sapiens
879	11720973_x_at	turquoise	ankyrin repeat and SOCS box containing 8(ASB8)	Homo sapiens
881	11720977_a_at	turquoise	tryptophanyl tRNA synthetase 2, mitochondrial(WARS2)	Homo sapiens
882	11720982_s_at	turquoise	protein tyrosine kinase 2 beta(PTK2B)	Homo sapiens
883	11720989_a_at	turquoise	solute carrier family 7 member 7(SLC7A7)	Homo sapiens
884	11720994_x_at	turquoise	C-C motif chemokine ligand 3(CCL3)	Homo sapiens

885	11720998_a_at	turquoise	bridging integrator 2(BIN2)	Homo sapiens
886	11720999_a_at	turquoise	bridging integrator 2(BIN2)	Homo sapiens
887	11721027_a_at	turquoise	cyclin G associated kinase(GAK)	Homo sapiens
891	11721043_at	turquoise	exoribonuclease 1(ERI1)	Homo sapiens
892	11721044_a_at	turquoise	exoribonuclease 1(ERI1)	Homo sapiens
893	11721053_s_at	turquoise	kelch like family member 42(KLHL42)	Homo sapiens
894	11721054_at	turquoise	kelch like family member 42(KLHL42)	Homo sapiens
895	11721055_x_at	turquoise	protein kinase, X-linked(PRKX)	Homo sapiens
896	11721056_x_at	turquoise	protein kinase, X-linked(PRKX)	Homo sapiens
897	11721061_a_at	turquoise	IQ motif containing GTPase activating protein 2(IQGAP2)	Homo sapiens
899	11721096_a_at	turquoise	thioredoxin related transmembrane protein 1(TMX1)	Homo sapiens
900	11721097_at	turquoise	thioredoxin related transmembrane protein 1(TMX1)	Homo sapiens
901	11721098_x_at	turquoise	thioredoxin related transmembrane protein 1(TMX1)	Homo sapiens
902	11721099_at	turquoise	complement C3a receptor 1(C3AR1)	Homo sapiens
904	11721120_a_at	turquoise	nudix hydrolase 5(NUDT5)	Homo sapiens
905	11721125_at	turquoise	signal peptide peptidase like 2A(SPPL2A)	Homo sapiens
906	11721140_a_at	turquoise	transcription elongation factor A3(TCEA3)	Homo sapiens
907	11721141_x_at	turquoise	transcription elongation factor A3(TCEA3)	Homo sapiens
908	11721143_a_at	turquoise	marker of proliferation Ki-67(MKI67)	Homo sapiens
909	11721145_s_at	turquoise	marker of proliferation Ki-67(MKI67)	Homo sapiens
910	11721147_at	turquoise	synaptogyrin 1(SYNGR1)	Homo sapiens
911	11721149_a_at	turquoise	LysM domain containing 2(LYSMD2)	Homo sapiens
912	11721157_at	turquoise	nth like DNA glycosylase 1(NTHL1)	Homo sapiens
913	11721163_a_at	turquoise	KH and NYN domain containing(KHNYN)	Homo sapiens
914	11721169_a_at	turquoise	PYD and CARD domain containing(PYCARD)	Homo sapiens
915	11721184_at	turquoise	tetraspanin 33(TSPAN33)	Homo sapiens
916	11721202_at	turquoise	ribonuclease A family member k6(RNASE6)	Homo sapiens
917	11721208_at	turquoise	intermediate filament family orphan 2(IFFO2)	Homo sapiens
918	11721223_at	turquoise	acyl-CoA oxidase 2(ACOX2)	Homo sapiens
919	11721243_a_at	turquoise	leucine aminopeptidase 3(LAP3)	Homo sapiens
920	11721248_s_at	turquoise	G protein subunit gamma 2(GNG2)	Homo sapiens
922	11721272_at	turquoise	leucine rich repeat containing 59(LRRC59)	Homo sapiens
924	11721297_at	turquoise	ABRA C-terminal like(ABRACL)	Homo sapiens
925	11721302_a_at	turquoise	C-type lectin domain family 2 member B(CLEC2B)	Homo sapiens
926	11721303_at	turquoise	C-type lectin domain family 2 member B(CLEC2B)	Homo sapiens
927	11721313_a_at	turquoise	extended synaptotagmin 2(ESYT2)	Homo sapiens
928	11721346_a_at	turquoise	macrophage expressed 1(MPEG1)	Homo sapiens
929	11721347_a_at	turquoise	macrophage expressed 1(MPEG1)	Homo sapiens
930	11721349_s_at	turquoise	ubiquitin specific peptidase 18(USP18)	Homo sapiens
931	11721366_at	turquoise	tripartite motif containing 47(TRIM47)	Homo sapiens
932	11721380_x_at	turquoise	N-acetylglucosamine-1-phosphate transferase alpha and beta subunits(GNPTAB)	Homo sapiens
933	11721390_at	turquoise	HtrA serine peptidase 3(HTRA3)	Homo sapiens
934	11721408_a_at	turquoise	polyamine oxidase(PAOX)	Homo sapiens
935	11721432_a_at	turquoise	microtubule associated protein 1S(MAP1S)	Homo sapiens
936	11721454_a_at	turquoise	serpin family A member 1(SERPINA1)	Homo sapiens
937	11721455_s_at	turquoise	serpin family A member 1(SERPINA1)	Homo sapiens
938	11721456_x_at	turquoise	serpin family A member 1(SERPINA1)	Homo sapiens
939	11721460_s_at	turquoise	TAP binding protein like(TAPBPL)	Homo sapiens
943	11721491_a_at	turquoise	fructose-bisphosphatase 1(FBP1)	Homo sapiens
944	11721493_a_at	turquoise	sulfatase 2(SULF2)	Homo sapiens
945	11721504_a_at	turquoise	CKLF-CMTM1 readthrough(CKLF-CMTM1)	Homo sapiens
946	11721512_a_at	turquoise	translocase of inner mitochondrial membrane 21(TIMM21)	Homo sapiens
947	11721554_a_at	turquoise	collagen beta(1-O)galactosyltransferase 1(COLGALT1)	Homo sapiens
948	11721557_a_at	turquoise	ATP binding cassette subfamily A member 8(ABCA8)	Homo sapiens
949	11721570_a_at	turquoise	family with sequence similarity 173 member B(FAM173B)	Homo sapiens
950	11721573_a_at	turquoise	activin A receptor like type 1(ACVRL1)	Homo sapiens
951	11721574_a_at	turquoise	E74 like ETS transcription factor 4(ELF4)	Homo sapiens
952	11721577_at	turquoise	tumor necrosis factor(TNF)	Homo sapiens
953	11721582_a_at	turquoise	lymphoid restricted membrane protein(LRMP)	Homo sapiens
954	11721583_x_at	turquoise	lymphoid restricted membrane protein(LRMP)	Homo sapiens
955	11721587_at	turquoise	TNF receptor associated factor 1(TRAF1)	Homo sapiens
956	11721588_at	turquoise	TNF receptor associated factor 1(TRAF1)	Homo sapiens
957	11721590_a_at	turquoise	solute carrier family 17 member 9(SLC17A9)	Homo sapiens
958	11721609_a_at	turquoise	poly(A) binding protein cytoplasmic 1 like(PABPC1L)	Homo sapiens
959	11721615_a_at	turquoise	thymocyte selection associated family member 2(THEMIS2)	Homo sapiens
960	11721623_a_at	turquoise	mesenchyme homeobox 1(MEOX1)	Homo sapiens
961	11721626_a_at	turquoise	septin 6(SEPT6)	Homo sapiens
962	11721627_a_at	turquoise	septin 6(SEPT6)	Homo sapiens
963	11721628_a_at	turquoise	septin 6(SEPT6)	Homo sapiens
964	11721629_a_at	turquoise	MAF bZIP transcription factor B(MAFB)	Homo sapiens
965	11721630_at	turquoise	MAF bZIP transcription factor B(MAFB)	Homo sapiens
966	11721651_at	turquoise	cadherin 5(CDH5)	Homo sapiens
967	11721661_a_at	turquoise	thromboxane A synthase 1(TBXAS1)	Homo sapiens
972	11721685_at	turquoise	C-C motif chemokine ligand 19(CCL19)	Homo sapiens
973	11721695_s_at	turquoise	dual specificity phosphatase 2(DUSP2)	Homo sapiens
974	11721702_a_at	turquoise	CKLF like MARVEL transmembrane domain containing 7(CMTM7)	Homo sapiens
975	11721704_a_at	turquoise	cysteine rich protein 1(CRIP1)	Homo sapiens
977	11721719_at	turquoise	N-acylethanolamine acid amidase(NAAA)	Homo sapiens
978	11721722_x_at	turquoise	MSH5-SAPCD1 readthrough (NMD candidate)(MSH5-SAPCD1)	Homo sapiens
979	11721728_a_at	turquoise	Rho GTPase activating protein 30(ARHGAP30)	Homo sapiens
980	11721733_a_at	turquoise	GTP cyclohydrolase 1(GCH1)	Homo sapiens
981	11721734_s_at	turquoise	GTP cyclohydrolase 1(GCH1)	Homo sapiens

982	11721755_at	turquoise	ALG13, UDP-N-acetylglucosaminyltransferase subunit(ALG13)	Homo sapiens
983	11721760_a_at	turquoise	tRNA splicing endonuclease subunit 54(TSEN54)	Homo sapiens
984	11721762_a_at	turquoise	phosphoinositide-3-kinase regulatory subunit 5(PIK3R5)	Homo sapiens
985	11721773_at	turquoise	WSC domain containing 1(WSCD1)	Homo sapiens
986	11721780_a_at	turquoise	LLGL2, scribble cell polarity complex component(LLGL2)	Homo sapiens
987	11721804_a_at	turquoise	BUB3, mitotic checkpoint protein(BUB3)	Homo sapiens
988	11721810_at	turquoise	Pim-2 proto-oncogene, serine/threonine kinase(PIM2)	Homo sapiens
989	11721815_a_at	turquoise	Fc fragment of IgM receptor(FCMR)	Homo sapiens
990	11721816_a_at	turquoise	Fc fragment of IgM receptor(FCMR)	Homo sapiens
991	11721827_a_at	turquoise	zwilch kinetochore protein(ZWILCH)	Homo sapiens
992	11721828_x_at	turquoise	zwilch kinetochore protein(ZWILCH)	Homo sapiens
994	11721838_a_at	turquoise	GLI pathogenesis related 1(GLIPR1)	Homo sapiens
995	11721839_at	turquoise	GLI pathogenesis related 1(GLIPR1)	Homo sapiens
996	11721840_at	turquoise	GLI pathogenesis related 1(GLIPR1)	Homo sapiens
997	11721841_at	turquoise	GLI pathogenesis related 1(GLIPR1)	Homo sapiens
998	11721842_a_at	turquoise	GLI pathogenesis related 1(GLIPR1)	Homo sapiens
999	11721843_a_at	turquoise	atlastin GTPase 3(ATL3)	Homo sapiens
1001	11721859_at	turquoise	mitochondrial intermediate peptidase(MIPEP)	Homo sapiens
1002	11721860_s_at	turquoise	syntaxin 12(STX12)	Homo sapiens
1003	11721861_at	turquoise	syntaxin 12(STX12)	Homo sapiens
1004	11721872_at	turquoise	family with sequence similarity 110 member A(FAM110A)	Homo sapiens
1005	11721873_at	turquoise	interferon induced protein with tetratricopeptide repeats 2(IFIT2)	Homo sapiens
1006	11721874_at	turquoise	interferon induced protein with tetratricopeptide repeats 2(IFIT2)	Homo sapiens
1007	11721879_s_at	turquoise	AT-rich interaction domain 4B(ARID4B)	Homo sapiens
1008	11721880_s_at	turquoise	AT-rich interaction domain 4B(ARID4B)	Homo sapiens
1009	11721885_s_at	turquoise	cell division cycle 42(CDC42)	Homo sapiens
1010	11721897_a_at	turquoise	DExH-box helicase 58(DHX58)	Homo sapiens
1011	11721900_at	turquoise	ficolin 1(FCN1)	Homo sapiens
1012	11721906_s_at	turquoise	proteasome subunit alpha 4(PSMA4)	Homo sapiens
1013	11721907_at	turquoise	sphingosine-1-phosphate phosphatase 1(SGPP1)	Homo sapiens
1014	11721922_at	turquoise	protein kinase C beta(PRKCB)	Homo sapiens
1015	11721923_a_at	turquoise	protein kinase C beta(PRKCB)	Homo sapiens
1016	11721924_s_at	turquoise	protein kinase C beta(PRKCB)	Homo sapiens
1017	11721927_at	turquoise	transmembrane channel like 8(TMC8)	Homo sapiens
1018	11721935_at	turquoise	DnaJ heat shock protein family (Hsp40) member C27(DNAJC27)	Homo sapiens
1019	11721954_s_at	turquoise	ring finger protein 41(RNF41)	Homo sapiens
1020	11721960_a_at	turquoise	sorting nexin family member 30(SNX30)	Homo sapiens
1021	11721974_a_at	turquoise	sulfite oxidase(SUOX)	Homo sapiens
1022	11721988_at	turquoise	coiled-coil domain containing 191(CCDC191)	Homo sapiens
1023	11721994_s_at	turquoise	ubiquitin conjugating enzyme E2 L6(UBE2L6)	Homo sapiens
1024	11721996_a_at	turquoise	poly(ADP-ribose) polymerase family member 14(PARP14)	Homo sapiens
1025	11721997_s_at	turquoise	poly(ADP-ribose) polymerase family member 14(PARP14)	Homo sapiens
1026	11721998_a_at	turquoise	poly(ADP-ribose) polymerase family member 14(PARP14)	Homo sapiens
1027	11722003_at	turquoise	cytochrome c, somatic(CYCS)	Homo sapiens
1029	11722005_a_at	turquoise	nuclear autoantigenic sperm protein(NASP)	Homo sapiens
1030	11722009_a_at	turquoise	chloride intracellular channel 1(CLIC1)	Homo sapiens
1031	11722011_a_at	turquoise	GTPase, IMAP family member 4(GIMAP4)	Homo sapiens
1032	11722012_a_at	turquoise	GTPase, IMAP family member 4(GIMAP4)	Homo sapiens
1034	11722038_a_at	turquoise	FES proto-oncogene, tyrosine kinase(FES)	Homo sapiens
1035	11722039_x_at	turquoise	FES proto-oncogene, tyrosine kinase(FES)	Homo sapiens
1037	11722049_a_at	turquoise	dual specificity phosphatase 6(DUSP6)	Homo sapiens
1038	11722072_at	turquoise	mesoderm development candidate 1(MESDC1)	Homo sapiens
1039	11722086_x_at	turquoise	septin 1(SEPT1)	Homo sapiens
1040	11722089_a_at	turquoise	echinoderm microtubule associated protein like 3(EML3)	Homo sapiens
1041	11722095_a_at	turquoise	kinesin family member 21B(KIF21B)	Homo sapiens
1042	11722120_a_at	turquoise	kinetochore associated 1(KNTC1)	Homo sapiens
1043	11722130_s_at	turquoise	enoyl-CoA hydratase domain containing 2(ECHDC2)	Homo sapiens
1044	11722134_a_at	turquoise	TNF receptor superfamily member 25(TNFRSF25)	Homo sapiens
1045	11722141_at	turquoise	RELB proto-oncogene, NF-kB subunit(RELB)	Homo sapiens
1046	11722143_at	turquoise	tetratricopeptide repeat and ankyrin repeat containing 1(TRANK1)	Homo sapiens
1047	11722147_at	turquoise	poly(A) binding protein interacting protein 2B(PAIP2B)	Homo sapiens
1048	11722152_s_at	turquoise	eukaryotic translation initiation factor 2 alpha kinase 3(EIF2AK3)	Homo sapiens
1049	11722170_a_at	turquoise	coronin 7(CORO7)	Homo sapiens
1051	11722174_a_at	turquoise	ubiquinol-cytochrome c reductase complex assembly factor 1(UQCC1)	Homo sapiens
1052	11722183_s_at	turquoise	ATPase H+ transporting V1 subunit C1(ATP6V1C1)	Homo sapiens
1053	11722193_a_at	turquoise	chromosome 12 open reading frame 75(C12orf75)	Homo sapiens
1055	11722225_a_at	turquoise	ADAM metallopeptidase domain 8(ADAM8)	Homo sapiens
1057	11722228_at	turquoise	DnaJ heat shock protein family (Hsp40) member C30(DNAJC30)	Homo sapiens
1058	11722247_a_at	turquoise	Ras association domain family member 1(RASSF1)	Homo sapiens
1059	11722248_a_at	turquoise	Ras association domain family member 1(RASSF1)	Homo sapiens
1060	11722262_at	turquoise	ER degradation enhancing alpha-mannosidase like protein 1(EDEM1)	Homo sapiens
1061	11722269_a_at	turquoise	SAM and SH3 domain containing 3(SASH3)	Homo sapiens
1062	11722270_at	turquoise	SAM and SH3 domain containing 3(SASH3)	Homo sapiens
1063	11722286_a_at	turquoise	solute carrier family 30 member 7(SLC30A7)	Homo sapiens
1064	11722287_a_at	turquoise	solute carrier family 30 member 7(SLC30A7)	Homo sapiens
1065	11722298_a_at	turquoise	RAB29, member RAS oncogene family(RAB29)	Homo sapiens
1066	11722299_a_at	turquoise	RAB29, member RAS oncogene family(RAB29)	Homo sapiens
1067	11722300_a_at	turquoise	ETS proto-oncogene 1, transcription factor(ETS1)	Homo sapiens
1068	11722302_a_at	turquoise	LRR binding FLII interacting protein 1(LRRFIP1)	Homo sapiens
1069	11722303_x_at	turquoise	LRR binding FLII interacting protein 1(LRRFIP1)	Homo sapiens
1070	11722304_a_at	turquoise	LRR binding FLII interacting protein 1(LRRFIP1)	Homo sapiens
1071	11722313_a_at	turquoise	Rap guanine nucleotide exchange factor 6(RAPGEF6)	Homo sapiens

1072	11722314_a_at	turquoise	Rap guanine nucleotide exchange factor 6(RAPGEF6)	Homo sapiens
1073	11722321_at	turquoise	hematopoietically expressed homeobox(HHEX)	Homo sapiens
1074	11722333_a_at	turquoise	fibronectin type III domain containing 5(FNDC5)	Homo sapiens
1075	11722334_at	turquoise	fibronectin type III domain containing 5(FNDC5)	Homo sapiens
1076	11722335_a_at	turquoise	fibronectin type III domain containing 5(FNDC5)	Homo sapiens
1078	11722349_a_at	turquoise	Lck interacting transmembrane adaptor 1(LIME1)	Homo sapiens
1079	11722355_s_at	turquoise	DAB2, clathrin adaptor protein(DAB2)	Homo sapiens
1080	11722356_a_at	turquoise	DAB2, clathrin adaptor protein(DAB2)	Homo sapiens
1082	11722368_a_at	turquoise	tripartite motif containing 22(TRIM22)	Homo sapiens
1083	11722369_x_at	turquoise	tripartite motif containing 22(TRIM22)	Homo sapiens
1084	11722370_a_at	turquoise	tripartite motif containing 22(TRIM22)	Homo sapiens
1085	11722371_x_at	turquoise	tripartite motif containing 22(TRIM22)	Homo sapiens
1086	11722403_a_at	turquoise	pleckstrin homology domain containing O1(PLEKHO1)	Homo sapiens
1087	11722417_a_at	turquoise	La ribonucleoprotein domain family member 6(LARP6)	Homo sapiens
1088	11722418_at	turquoise	UTP3, small subunit processome component homolog (S. cerevisiae)(UTP3)	Homo sapiens
1089	11722419_s_at	turquoise	UTP3, small subunit processome component homolog (S. cerevisiae)(UTP3)	Homo sapiens
1090	11722425_s_at	turquoise	neural precursor cell expressed, developmentally down-regulated 4-like, E3 ubiquitin protein ligase(NEDD4L)	Homo sapiens
1091	11722426_a_at	turquoise	fibrinogen like 2(FGL2)	Homo sapiens
1092	11722427_at	turquoise	fibrinogen like 2(FGL2)	Homo sapiens
1093	11722446_a_at	turquoise	sirtuin 3(SIRT3)	Homo sapiens
1094	11722447_a_at	turquoise	sirtuin 3(SIRT3)	Homo sapiens
1095	11722449_x_at	turquoise	thymidine phosphorylase(TYMP)	Homo sapiens
1096	11722458_a_at	turquoise	transmembrane protein 206(TMEM206)	Homo sapiens
1097	11722462_a_at	turquoise	tankyrase 2(TNKS2)	Homo sapiens
1098	11722471_a_at	turquoise	paternally expressed 3(PEG3)	Homo sapiens
1099	11722472_a_at	turquoise	paternally expressed 3(PEG3)	Homo sapiens
1100	11722474_a_at	turquoise	diacylglycerol kinase theta(DGKQ)	Homo sapiens
1101	11722480_a_at	turquoise	FGR proto-oncogene, Src family tyrosine kinase(FGR)	Homo sapiens
1102	11722481_a_at	turquoise	mitochondrial fission factor(MFF)	Homo sapiens
1104	11722501_a_at	turquoise	p21 (RAC1) activated kinase 6(PAK6)	Homo sapiens
1105	11722502_a_at	turquoise	p21 (RAC1) activated kinase 6(PAK6)	Homo sapiens
1106	11722512_a_at	turquoise	SP110 nuclear body protein(SP110)	Homo sapiens
1107	11722535_a_at	turquoise	cyclin Y like 1(CCNYL1)	Homo sapiens
1108	11722538_a_at	turquoise	transmembrane and coiled-coil domains 6(TMCO6)	Homo sapiens
1110	11722571_at	turquoise	cyclin A2(CCNA2)	Homo sapiens
1111	11722573_a_at	turquoise	endoplasmic reticulum aminopeptidase 2(ERAP2)	Homo sapiens
1112	11722574_at	turquoise	adaptor related protein complex 1 gamma 1 subunit(AP1G1)	Homo sapiens
1113	11722577_a_at	turquoise	prolyl 3-hydroxylase 1(P3H1)	Homo sapiens
1114	11722609_a_at	turquoise	CDC42 small effector 1(CDC42SE1)	Homo sapiens
1115	11722620_a_at	turquoise	aquaporin 4(AQP4)	Homo sapiens
1116	11722635_at	turquoise	interleukin 2 receptor subunit beta(IL2RB)	Homo sapiens
1117	11722660_a_at	turquoise	nuclear factor, erythroid 2 like 3(NFE2L3)	Homo sapiens
1118	11722679_a_at	turquoise	poly(ADP-ribose) polymerase family member 8(PARP8)	Homo sapiens
1119	11722680_at	turquoise	hematopoietic cell signal transducer(HCST)	Homo sapiens
1120	11722697_x_at	turquoise	SFI1 centrin binding protein(SFI1)	Homo sapiens
1121	11722705_at	turquoise	UDP-GlcNAc:betaGal beta-1,3-N-acetylglucosaminyltransferase 5(B3GNT5)	Homo sapiens
1122	11722708_a_at	turquoise	coiled-coil serine rich protein 2(CCSER2)	Homo sapiens
1123	11722721_a_at	turquoise	centromere protein V(CENPV)	Homo sapiens
1124	11722725_a_at	turquoise	colony stimulating factor 2 receptor beta common subunit(CSF2RB)	Homo sapiens
1125	11722728_a_at	turquoise	early growth response 2(EGR2)	Homo sapiens
1126	11722747_at	turquoise	hook microtubule tethering protein 3(HOOK3)	Homo sapiens
1127	11722765_a_at	turquoise	GIT ArfGAP 2(GIT2)	Homo sapiens
1128	11722768_s_at	turquoise	Rap guanine nucleotide exchange factor 5(RAPGEF5)	Homo sapiens
1129	11722778_s_at	turquoise	ectonucleotide pyrophosphatase/phosphodiesterase 2(ENPP2)	Homo sapiens
1130	11722794_at	turquoise	glycerophosphodiester phosphodiesterase 1(GDE1)	Homo sapiens
1132	11722806_at	turquoise	RAB35, member RAS oncogene family(RAB35)	Homo sapiens
1133	11722807_a_at	turquoise	WD repeat domain 54(WDR54)	Homo sapiens
1134	11722818_a_at	turquoise	gamma-glutamyl hydrolase(GGH)	Homo sapiens
1135	11722826_a_at	turquoise	non-SMC condensin I complex subunit G(NCAPG)	Homo sapiens
1137	11722850_a_at	turquoise	baculoviral IAP repeat containing 3(BIRC3)	Homo sapiens
1138	11722851_at	turquoise	baculoviral IAP repeat containing 3(BIRC3)	Homo sapiens
1139	11722852_s_at	turquoise	baculoviral IAP repeat containing 3(BIRC3)	Homo sapiens
1140	11722874_s_at	turquoise	male-specific lethal 3 homolog (Drosophila)(MSL3)	Homo sapiens
1141	11722880_a_at	turquoise	PHD finger protein 19(PHF19)	Homo sapiens
1142	11722886_s_at	turquoise	embigin(EMB)	Homo sapiens
1143	11722887_x_at	turquoise	embigin(EMB)	Homo sapiens
1144	11722888_s_at	turquoise	embigin(EMB)	Homo sapiens
1145	11722904_a_at	turquoise	TBC1 domain family member 9(TBC1D9)	Homo sapiens
1149	11722923_a_at	turquoise	family with sequence similarity 122B(FAM122B)	Homo sapiens
1150	11722928_a_at	turquoise	microRNA 4656(MIR4656)	Homo sapiens
1151	11722940_a_at	turquoise	SLIT-ROBO Rho GTPase activating protein 2(SRGAP2)	Homo sapiens
1152	11722960_a_at	turquoise	cytochrome b561 family member D1(CYB561D1)	Homo sapiens
1153	11722964_a_at	turquoise	Alport syndrome, mental retardation, midface hypoplasia and elliptocytosis chromosomal region gene 1(AMMECR1)	Homo sapiens
1154	11722979_a_at	turquoise	coagulation factor VIII(F8)	Homo sapiens
1155	11722982_a_at	turquoise	lysosomal trafficking regulator(LYST)	Homo sapiens
1156	11723006_a_at	turquoise	S100 calcium binding protein A4(S100A4)	Homo sapiens
1157	11723010_a_at	turquoise	kinesin family member 20A(KIF20A)	Homo sapiens
1158	11723016_a_at	turquoise	histocompatibility minor 13(HM13)	Homo sapiens
1159	11723020_a_at	turquoise	receptor interacting serine/threonine kinase 3(RIPK3)	Homo sapiens
1160	11723021_a_at	turquoise	branched chain keto acid dehydrogenase E1 subunit beta(BCKDHB)	Homo sapiens
1161	11723025_at	turquoise	family with sequence similarity 26 member F(FAM26F)	Homo sapiens
1162	11723026_at	turquoise	family with sequence similarity 26 member F(FAM26F)	Homo sapiens

1164	11723048_at	turquoise	C-X3-C motif chemokine receptor 1(CX3CR1)	Homo sapiens
1165	11723050_a_at	turquoise	heat shock protein family A (Hsp70) member 6(HSPA6)	Homo sapiens
1168	11723058_a_at	turquoise	transporter 2, ATP binding cassette subfamily B member(TAP2)	Homo sapiens
1169	11723059_a_at	turquoise	transporter 2, ATP binding cassette subfamily B member(TAP2)	Homo sapiens
1170	11723060_at	turquoise	Rab interacting lysosomal protein like 2(RILPL2)	Homo sapiens
1171	11723061_s_at	turquoise	Rab interacting lysosomal protein like 2(RILPL2)	Homo sapiens
1172	11723065_at	turquoise	endonuclease G(ENDOG)	Homo sapiens
1173	11723069_at	turquoise	ubiquitin D(UBD)	Homo sapiens
1175	11723095_a_at	turquoise	CYLD lysine 63 deubiquitinase(CYLD)	Homo sapiens
1176	11723096_a_at	turquoise	CYLD lysine 63 deubiquitinase(CYLD)	Homo sapiens
1177	11723099_a_at	turquoise	carbonic anhydrase 14(CA14)	Homo sapiens
1178	11723100_at	turquoise	carbonic anhydrase 14(CA14)	Homo sapiens
1179	11723105_at	turquoise	cytidine/uridine monophosphate kinase 2(CMPK2)	Homo sapiens
1180	11723106_a_at	turquoise	neutrophil cytosolic factor 4(NCF4)	Homo sapiens
1181	11723110_at	turquoise	pleckstrin homology domain containing B2(PLEKHB2)	Homo sapiens
1182	11723116_a_at	turquoise	RAD51L3-RFFL readthrough(RAD51L3-RFFL)	Homo sapiens
1183	11723134_a_at	turquoise	solute carrier family 22 member 17(SLC22A17)	Homo sapiens
1184	11723144_at	turquoise	sodium leak channel, non-selective(NALCN)	Homo sapiens
1185	11723151_s_at	turquoise	succinyl-CoA:glutarate-CoA transferase(SUGCT)	Homo sapiens
1186	11723156_a_at	turquoise	lymphocyte-specific protein 1(LSP1)	Homo sapiens
1187	11723159_at	turquoise	transmembrane protein 229B(TMEM229B)	Homo sapiens
1188	11723169_s_at	turquoise	forkhead box N2(FOXN2)	Homo sapiens
1189	11723179_a_at	turquoise	intersectin 2(ITSN2)	Homo sapiens
1190	11723181_a_at	turquoise	mitogen-activated protein kinase 4(MAPK4)	Homo sapiens
1191	11723182_at	turquoise	CCR4-NOT transcription complex subunit 6 like(CNOT6L)	Homo sapiens
1192	11723183_s_at	turquoise	CCR4-NOT transcription complex subunit 6 like(CNOT6L)	Homo sapiens
1193	11723184_x_at	turquoise	CCR4-NOT transcription complex subunit 6 like(CNOT6L)	Homo sapiens
1194	11723185_s_at	turquoise	CCR4-NOT transcription complex subunit 6 like(CNOT6L)	Homo sapiens
1195	11723194_x_at	turquoise	major histocompatibility complex, class II, DQ beta 1(HLA-DQB1)	Homo sapiens
1196	11723195_x_at	turquoise	major histocompatibility complex, class I, E(HLA-E)	Homo sapiens
1197	11723197_at	turquoise	heterogeneous nuclear ribonucleoprotein A3(HNRNPA3)	Homo sapiens
1198	11723198_s_at	turquoise	heterogeneous nuclear ribonucleoprotein A3 pseudogene 1(HNRNPA3P1)	Homo sapiens
1199	11723199_s_at	turquoise	heterogeneous nuclear ribonucleoprotein A3 pseudogene 1(HNRNPA3P1)	Homo sapiens
1200	11723217_x_at	turquoise	sideroflexin 3(SFXN3)	Homo sapiens
1201	11723230_a_at	turquoise	ring finger protein 138(RNF138)	Homo sapiens
1202	11723231_s_at	turquoise	ring finger protein 138 pseudogene 1(RNF138P1)	Homo sapiens
1203	11723232_x_at	turquoise	ring finger protein 138(RNF138)	Homo sapiens
1204	11723243_at	turquoise	cyclin dependent kinase 6(CDK6)	Homo sapiens
1205	11723262_at	turquoise	antagonist of mitotic exit network 1 homolog(AMN1)	Homo sapiens
1207	11723264_a_at	turquoise	CD3d molecule(CD3D)	Homo sapiens
1208	11723287_at	turquoise	TYRO protein tyrosine kinase binding protein(TYROBP)	Homo sapiens
1209	11723292_at	turquoise	ORAI calcium release-activated calcium modulator 1(ORAI1)	Homo sapiens
1210	11723310_at	turquoise	TNF receptor superfamily member 10d(TNFRSF10D)	Homo sapiens
1212	11723335_s_at	turquoise	GEM interacting protein(GMIP)	Homo sapiens
1213	11723346_at	turquoise	LYL1, basic helix-loop-helix family member(LYL1)	Homo sapiens
1214	11723372_a_at	turquoise	dedicator of cytokinesis 11(DOCK11)	Homo sapiens
1215	11723378_a_at	turquoise	centriolin(CNTRL)	Homo sapiens
1216	11723400_a_at	turquoise	serine/threonine kinase 4(STK4)	Homo sapiens
1217	11723401_x_at	turquoise	serine/threonine kinase 4(STK4)	Homo sapiens
1218	11723402_a_at	turquoise	serine/threonine kinase 4(STK4)	Homo sapiens
1220	11723408_a_at	turquoise	McKusick-Kaufman syndrome(MKKS)	Homo sapiens
1221	11723409_s_at	turquoise	McKusick-Kaufman syndrome(MKKS)	Homo sapiens
1222	11723419_a_at	turquoise	collagen type IX alpha 3 chain(COL9A3)	Homo sapiens
1223	11723424_at	turquoise	interferon alpha and beta receptor subunit 1(IFNAR1)	Homo sapiens
1224	11723425_at	turquoise	interferon alpha and beta receptor subunit 1(IFNAR1)	Homo sapiens
1226	11723437_at	turquoise	centrosomal protein 41(CEP41)	Homo sapiens
1227	11723447_at	turquoise	mal, T-cell differentiation protein like(MALL)	Homo sapiens
1228	11723448_x_at	turquoise	mal, T-cell differentiation protein like(MALL)	Homo sapiens
1231	11723469_s_at	turquoise	lysophosphatidylglycerol acyltransferase 1(LPGAT1)	Homo sapiens
1232	11723491_a_at	turquoise	Rho guanine nucleotide exchange factor 3(ARHGEF3)	Homo sapiens
1233	11723492_a_at	turquoise	Rho guanine nucleotide exchange factor 3(ARHGEF3)	Homo sapiens
1234	11723494_a_at	turquoise	ring finger protein 166(RNF166)	Homo sapiens
1235	11723516_a_at	turquoise	calcium binding protein 39 like(CAB39L)	Homo sapiens
1236	11723534_at	turquoise	breast cancer metastasis-suppressor 1-like(BRMS1L)	Homo sapiens
1237	11723537_a_at	turquoise	Rho GTPase activating protein 45(ARHGAP45)	Homo sapiens
1238	11723545_a_at	turquoise	phospholipase D1(PLD1)	Homo sapiens
1239	11723546_s_at	turquoise	phospholipase D1(PLD1)	Homo sapiens
1241	11723590_a_at	turquoise	tetratricopeptide repeat domain 13(TTC13)	Homo sapiens
1242	11723591_at	turquoise	leucine rich repeat containing 8 family member C(LRRC8C)	Homo sapiens
1243	11723592_at	turquoise	leucine rich repeat containing 8 family member C(LRRC8C)	Homo sapiens
1244	11723604_a_at	turquoise	inositol polyphosphate-5-phosphatase J(INPP5J)	Homo sapiens
1245	11723605_a_at	turquoise	PC-esterase domain containing 1B(PCED1B)	Homo sapiens
1246	11723623_s_at	turquoise	tripartite motif containing 21(TRIM21)	Homo sapiens
1247	11723638_at	turquoise	chromosome 11 open reading frame 58(C11orf58)	Homo sapiens
1248	11723639_s_at	turquoise	chromosome 11 open reading frame 58(C11orf58)	Homo sapiens
1249	11723679_s_at	turquoise	CD69 molecule(CD69)	Homo sapiens
1250	11723689_at	turquoise	cAMP responsive element binding protein like 2(CREBL2)	Homo sapiens
1251	11723690_at	turquoise	cAMP responsive element binding protein like 2(CREBL2)	Homo sapiens
1253	11723697_s_at	turquoise	Fli-1 proto-oncogene, ETS transcription factor(FLI1)	Homo sapiens
1254	11723698_a_at	turquoise	2'-5'-oligoadenylate synthetase 3(OAS3)	Homo sapiens
1255	11723699_s_at	turquoise	2'-5'-oligoadenylate synthetase 3(OAS3)	Homo sapiens
1256	11723771_a_at	turquoise	microtubule associated serine/threonine kinase like(MASTL)	Homo sapiens

1257	11723795_a_at	turquoise	solute carrier family 29 member 2(SLC29A2)	Homo sapiens
1258	11723821_a_at	turquoise	SMAD specific E3 ubiquitin protein ligase 2(SMURF2)	Homo sapiens
1259	11723826_a_at	turquoise	chromosome 2 open reading frame 88(C2orf88)	Homo sapiens
1260	11723847_a_at	turquoise	ATPase phospholipid transporting 11C(ATP11C)	Homo sapiens
1261	11723849_a_at	turquoise	membrane spanning 4-domains A6A(MS4A6A)	Homo sapiens
1262	11723850_a_at	turquoise	outer dense fiber of sperm tails 2 like(ODF2L)	Homo sapiens
1263	11723851_x_at	turquoise	outer dense fiber of sperm tails 2 like(ODF2L)	Homo sapiens
1264	11723853_a_at	turquoise	B-cell CLL/lymphoma 11A(BCL11A)	Homo sapiens
1265	11723854_at	turquoise	sterile alpha motif domain containing 9(SAMD9)	Homo sapiens
1266	11723863_a_at	turquoise	dedicator of cytokinesis 10(DOCK10)	Homo sapiens
1267	11723871_a_at	turquoise	dedicator of cytokinesis 2(DOCK2)	Homo sapiens
1268	11723894_a_at	turquoise	telomerase associated protein 1(TEP1)	Homo sapiens
1269	11723895_a_at	turquoise	telomerase associated protein 1(TEP1)	Homo sapiens
1270	11723899_a_at	turquoise	dehydrogenase/reductase 9(DHRS9)	Homo sapiens
1271	11723902_at	turquoise	pleckstrin homology and RhoGEF domain containing G1(PLEKHG1)	Homo sapiens
1272	11723903_at	turquoise	pleckstrin homology and RhoGEF domain containing G1(PLEKHG1)	Homo sapiens
1273	11723910_a_at	turquoise	chemerin chemokine-like receptor 1(CMKLR1)	Homo sapiens
1274	11723946_a_at	turquoise	acyl-CoA synthetase long-chain family member 5(ACSL5)	Homo sapiens
1275	11723947_a_at	turquoise	acyl-CoA synthetase long-chain family member 5(ACSL5)	Homo sapiens
1276	11723964_at	turquoise	aspartylglucosaminidase(AGA)	Homo sapiens
1277	11723973_at	turquoise	carbohydrate sulfotransferase 12(CHST12)	Homo sapiens
1278	11723974_a_at	turquoise	apolipoprotein L3(APOL3)	Homo sapiens
1279	11723975_x_at	turquoise	apolipoprotein L3(APOL3)	Homo sapiens
1280	11723977_at	turquoise	RAB3A, member RAS oncogene family(RAB3A)	Homo sapiens
1281	11723978_x_at	turquoise	RAB3A, member RAS oncogene family(RAB3A)	Homo sapiens
1282	11723979_s_at	turquoise	phosphorylase kinase regulatory subunit alpha 2(PHKA2)	Homo sapiens
1283	11723991_a_at	turquoise	ALG6, alpha-1,3-glucosyltransferase(ALG6)	Homo sapiens
1284	11723995_a_at	turquoise	glucocorticoid induced 1(GLCCI1)	Homo sapiens
1285	11723996_a_at	turquoise	glucocorticoid induced 1(GLCCI1)	Homo sapiens
1286	11723997_a_at	turquoise	NFKB inhibitor interacting Ras like 1(NKIRAS1)	Homo sapiens
1287	11724004_a_at	turquoise	FYN binding protein(FYB)	Homo sapiens
1288	11724005_s_at	turquoise	FYN binding protein(FYB)	Homo sapiens
1289	11724006_a_at	turquoise	ectonucleoside triphosphate diphosphohydrolase 1(ENTPD1)	Homo sapiens
1290	11724008_a_at	turquoise	ectonucleoside triphosphate diphosphohydrolase 1(ENTPD1)	Homo sapiens
1291	11724013_a_at	turquoise	sterol O-acyltransferase 1(SOAT1)	Homo sapiens
1292	11724014_a_at	turquoise	sterol O-acyltransferase 1(SOAT1)	Homo sapiens
1293	11724033_s_at	turquoise	CD2 associated protein(CD2AP)	Homo sapiens
1294	11724037_at	turquoise	prostaglandin-endoperoxide synthase 2(PTGS2)	Homo sapiens
1295	11724058_x_at	turquoise	ceramide-1-phosphate transfer protein(CPTP)	Homo sapiens
1296	11724061_s_at	turquoise	oxysterol binding protein like 1A(OSBPL1A)	Homo sapiens
1297	11724082_x_at	turquoise	torsin family 2 member A(TOR2A)	Homo sapiens
1298	11724102_s_at	turquoise	PDZ domain containing ring finger 3(PDZRN3)	Homo sapiens
1299	11724103_x_at	turquoise	C8orf44-SGK3 readthrough(C8orf44-SGK3)	Homo sapiens
1300	11724104_s_at	turquoise	C8orf44-SGK3 readthrough(C8orf44-SGK3)	Homo sapiens
1301	11724106_a_at	turquoise	basic leucine zipper ATF-like transcription factor 2(BATF2)	Homo sapiens
1302	11724117_x_at	turquoise	sterile alpha motif domain containing 9 like(SAMD9L)	Homo sapiens
1303	11724118_a_at	turquoise	sterile alpha motif domain containing 9 like(SAMD9L)	Homo sapiens
1304	11724119_at	turquoise	sterile alpha motif domain containing 9 like(SAMD9L)	Homo sapiens
1305	11724145_a_at	turquoise	solute carrier family 37 member 1(SLC37A1)	Homo sapiens
1306	11724149_a_at	turquoise	src kinase associated phosphoprotein 1(SKAP1)	Homo sapiens
1307	11724159_x_at	turquoise	endothelial cell surface expressed chemotaxis and apoptosis regulator(ECSCR)	Homo sapiens
1308	11724163_at	turquoise	major histocompatibility complex, class II, DO beta(HLA-DOB)	Homo sapiens
1309	11724166_a_at	turquoise	collagen beta(1-O)galactosyltransferase 2(COLGALT2)	Homo sapiens
1310	11724167_s_at	turquoise	collagen beta(1-O)galactosyltransferase 2(COLGALT2)	Homo sapiens
1311	11724172_s_at	turquoise	FCH domain only 2(FCHO2)	Homo sapiens
1312	11724188_x_at	turquoise	poly(ADP-ribose) polymerase family member 10(PARP10)	Homo sapiens
1313	11724192_at	turquoise	COP9 signalosome subunit 2(COPS2)	Homo sapiens
1314	11724201_a_at	turquoise	transmembrane protein 143(TMEM143)	Homo sapiens
1315	11724214_a_at	turquoise	SPRY domain containing 4(SPRYD4)	Homo sapiens
1316	11724224_a_at	turquoise	adenosine monophosphate deaminase 2(AMPD2)	Homo sapiens
1317	11724226_at	turquoise	microRNA 6748(MIR6748)	Homo sapiens
1318	11724236_a_at	turquoise	receptor interacting serine/threonine kinase 2(RIPK2)	Homo sapiens
1319	11724237_a_at	turquoise	linker for activation of T-cells family member 2(LAT2)	Homo sapiens
1320	11724255_a_at	turquoise	2'-5'-oligoadenylate synthetase 1(OAS1)	Homo sapiens
1321	11724256_s_at	turquoise	2'-5'-oligoadenylate synthetase 1(OAS1)	Homo sapiens
1323	11724271_a_at	turquoise	HLF, PAR bZIP transcription factor(HLF)	Homo sapiens
1324	11724272_at	turquoise	HLF, PAR bZIP transcription factor(HLF)	Homo sapiens
1325	11724296_a_at	turquoise	bone morphogenetic protein 7(BMP7)	Homo sapiens
1326	11724297_a_at	turquoise	bone morphogenetic protein 7(BMP7)	Homo sapiens
1327	11724310_a_at	turquoise	SH3 domain binding protein 2(SH3BP2)	Homo sapiens
1328	11724311_a_at	turquoise	SH3 domain binding protein 2(SH3BP2)	Homo sapiens
1329	11724312_a_at	turquoise	SH3 domain binding protein 2(SH3BP2)	Homo sapiens
1330	11724326_a_at	turquoise	Rho GTPase activating protein 25(ARHGAP25)	Homo sapiens
1331	11724328_a_at	turquoise	ubiquitin conjugating enzyme E2 C(UBE2C)	Homo sapiens
1332	11724343_at	turquoise	integrin subunit alpha 4(ITGA4)	Homo sapiens
1333	11724344_a_at	turquoise	integrin subunit alpha 4(ITGA4)	Homo sapiens
1334	11724345_at	turquoise	integrin subunit alpha 4(ITGA4)	Homo sapiens
1335	11724346_a_at	turquoise	interferon induced with helicase C domain 1(IFIH1)	Homo sapiens
1336	11724347_a_at	turquoise	astrotactin 2(ASTN2)	Homo sapiens
1337	11724348_s_at	turquoise	astrotactin 2(ASTN2)	Homo sapiens
1339	11724354_x_at	turquoise	autophagy related 16 like 2(ATG16L2)	Homo sapiens
1341	11724357_a_at	turquoise	myosin VA(MYO5A)	Homo sapiens

1342	11724358_s_at	turquoise	myosin VA(MYO5A)	Homo sapiens
1343	11724360_s_at	turquoise	beta-transducin repeat containing E3 ubiquitin protein ligase(BTRC)	Homo sapiens
1344	11724361_s_at	turquoise	phosphoinositide kinase, FYVE-type zinc finger containing(PIKFYVE)	Homo sapiens
1345	11724374_at	turquoise	basic leucine zipper ATF-like transcription factor(BATF)	Homo sapiens
1346	11724375_at	turquoise	phosphoprotein membrane anchor with glycosphingolipid microdomains 1(PAG1)	Homo sapiens
1347	11724377_at	turquoise	phosphoprotein membrane anchor with glycosphingolipid microdomains 1(PAG1)	Homo sapiens
1348	11724378_s_at	turquoise	phosphoprotein membrane anchor with glycosphingolipid microdomains 1(PAG1)	Homo sapiens
1349	11724381_at	turquoise	family with sequence similarity 65 member B(FAM65B)	Homo sapiens
1350	11724391_at	turquoise	aspartate beta-hydroxylase domain containing 2(ASPHD2)	Homo sapiens
1351	11724392_at	turquoise	aspartate beta-hydroxylase domain containing 2(ASPHD2)	Homo sapiens
1352	11724399_a_at	turquoise	DEAD-box helicase 60-like(DDX60L)	Homo sapiens
1353	11724404_a_at	turquoise	Wilms tumor 1 associated protein(WTAP)	Homo sapiens
1354	11724424_at	turquoise	G protein-coupled receptor 68(GPR68)	Homo sapiens
1355	11724432_x_at	turquoise	trafficking protein particle complex 2(TRAPPC2)	Homo sapiens
1356	11724435_a_at	turquoise	thiamin pyrophosphokinase 1(TPK1)	Homo sapiens
1357	11724465_a_at	turquoise	anillin actin binding protein(ANLN)	Homo sapiens
1358	11724485_x_at	turquoise	c-src tyrosine kinase(CSK)	Homo sapiens
1359	11724499_at	turquoise	MFNG O-fucosylpeptide 3-beta-N-acetylglucosaminyltransferase(MFNG)	Homo sapiens
1360	11724509_a_at	turquoise	phorbol-12-myristate-13-acetate-induced protein 1(PMAIP1)	Homo sapiens
1361	11724510_at	turquoise	phorbol-12-myristate-13-acetate-induced protein 1(PMAIP1)	Homo sapiens
1362	11724559_at	turquoise	G protein subunit alpha 15(GNA15)	Homo sapiens
1363	11724565_at	turquoise	talin 2(TLN2)	Homo sapiens
1365	11724595_at	turquoise	retinitis pigmentosa 2 (X-linked recessive)(RP2)	Homo sapiens
1366	11724596_s_at	turquoise	TRAF family member associated NFKB activator(TANK)	Homo sapiens
1367	11724601_s_at	turquoise	endoplasmic reticulum aminopeptidase 1(ERAP1)	Homo sapiens
1368	11724602_a_at	turquoise	endoplasmic reticulum aminopeptidase 1(ERAP1)	Homo sapiens
1369	11724640_a_at	turquoise	Rho GTPase activating protein 44(ARHGAP44)	Homo sapiens
1370	11724656_a_at	turquoise	mitogen-activated protein kinase kinase kinase kinase 1(MAP4K1)	Homo sapiens
1372	11724673_a_at	turquoise	branched chain keto acid dehydrogenase E1 subunit beta(BCKDHB)	Homo sapiens
1373	11724678_a_at	turquoise	lymphocyte antigen 86(LY86)	Homo sapiens
1374	11724682_s_at	turquoise	transforming growth factor beta receptor 2(TGFBR2)	Homo sapiens
1375	11724683_a_at	turquoise	CD300a molecule(CD300A)	Homo sapiens
1376	11724685_x_at	turquoise	interferon alpha and beta receptor subunit 2(IFNAR2)	Homo sapiens
1377	11724686_x_at	turquoise	interferon alpha and beta receptor subunit 2(IFNAR2)	Homo sapiens
1378	11724701_x_at	turquoise	BORCS7-ASMT readthrough (NMD candidate)(BORCS7-ASMT)	Homo sapiens
1379	11724728_a_at	turquoise	CD8a molecule(CD8A)	Homo sapiens
1380	11724729_a_at	turquoise	CD8a molecule(CD8A)	Homo sapiens
1381	11724732_s_at	turquoise	tubulin beta class I(TUBB)	Homo sapiens
1383	11724766_a_at	turquoise	FAST kinase domains 2(FASTKD2)	Homo sapiens
1384	11724768_s_at	turquoise	Fc fragment of IgG receptor IIa(FCGR2A)	Homo sapiens
1385	11724769_x_at	turquoise	Fc fragment of IgG receptor IIa(FCGR2A)	Homo sapiens
1386	11724770_s_at	turquoise	Fc fragment of IgG receptor IIa(FCGR2A)	Homo sapiens
1387	11724771_x_at	turquoise	Fc fragment of IgG receptor IIa(FCGR2A)	Homo sapiens
1388	11724775_at	turquoise	ferredoxin 1(FDX1)	Homo sapiens
1389	11724776_at	turquoise	phosphoglucomutase 2 like 1(PGM2L1)	Homo sapiens
1391	11724793_s_at	turquoise	phosphatidylglycerophosphate synthase 1(PGS1)	Homo sapiens
1392	11724799_x_at	turquoise	major histocompatibility complex, class II, DQ alpha 1(HLA-DQA1)	Homo sapiens
1393	11724800_a_at	turquoise	quinolinate phosphoribosyltransferase(QPRT)	Homo sapiens
1394	11724804_x_at	turquoise	leukocyte associated immunoglobulin like receptor 1(LAIR1)	Homo sapiens
1395	11724805_a_at	turquoise	leukocyte associated immunoglobulin like receptor 1(LAIR1)	Homo sapiens
1396	11724807_at	turquoise	Cbl proto-oncogene(CBL)	Homo sapiens
1397	11724810_at	turquoise	Cbl proto-oncogene(CBL)	Homo sapiens
1398	11724817_a_at	turquoise	SH3 domain containing kinase binding protein 1(SH3KBP1)	Homo sapiens
1399	11724820_a_at	turquoise	NIMA related kinase 6(NEK6)	Homo sapiens
1400	11724821_a_at	turquoise	NIMA related kinase 6(NEK6)	Homo sapiens
1401	11724844_s_at	turquoise	abhydrolase domain containing 17A(ABHD17A)	Homo sapiens
1402	11724851_a_at	turquoise	syntaxin binding protein 6(STXBP6)	Homo sapiens
1403	11724853_at	turquoise	LDL receptor related protein 3(LRP3)	Homo sapiens
1404	11724857_s_at	turquoise	centrosomal protein 63(CEP63)	Homo sapiens
1405	11724862_at	turquoise	threonine synthase like 1(THNSL1)	Homo sapiens
1406	11724881_at	turquoise	zinc finger protein 217(ZNF217)	Homo sapiens
1407	11724883_a_at	turquoise	hyaluronan and proteoglycan link protein 3(HAPLN3)	Homo sapiens
1408	11724884_x_at	turquoise	hyaluronan and proteoglycan link protein 3(HAPLN3)	Homo sapiens
1409	11724900_a_at	turquoise	granzyme B(GZMB)	Homo sapiens
1410	11724904_at	turquoise	TIMP metallopeptidase inhibitor 4(TIMP4)	Homo sapiens
1411	11724908_a_at	turquoise	dynein cytoplasmic 1 intermediate chain 1(DYNC1I1)	Homo sapiens
1412	11724921_x_at	turquoise	vacuolar protein sorting 13 homolog B(VPS13B)	Homo sapiens
1413	11724944_at	turquoise	calsyntenin 2(CLSTN2)	Homo sapiens
1414	11724947_a_at	turquoise	membrane associated guanylate kinase, WW and PDZ domain containing 2(MAGI2)	Homo sapiens
1415	11724954_s_at	turquoise	mannose receptor, C type 1(MRC1)	Homo sapiens
1416	11724958_at	turquoise	5',3'-nucleotidase, mitochondrial(NT5M)	Homo sapiens
1417	11724963_s_at	turquoise	F-box protein 5(FBXO5)	Homo sapiens
1418	11724979_a_at	turquoise	dipeptidase 2(DPEP2)	Homo sapiens
1419	11724997_a_at	turquoise	CD86 molecule(CD86)	Homo sapiens
1420	11724998_a_at	turquoise	CD86 molecule(CD86)	Homo sapiens
1421	11725011_a_at	turquoise	centrosomal protein 78(CEP78)	Homo sapiens
1422	11725013_at	turquoise	methylmalonic aciduria (cobalamin deficiency) cblC type, with homocystinuria(MMACHC)	Homo sapiens
1423	11725042_a_at	turquoise	tyrosine 3-monooxygenase/tryptophan 5-monooxygenase activation protein beta(YWHAB)	Homo sapiens
1424	11725044_s_at	turquoise	tyrosine 3-monooxygenase/tryptophan 5-monooxygenase activation protein beta(YWHAB)	Homo sapiens
1426	11725091_at	turquoise	regulatory factor X5(RFX5)	Homo sapiens
1427	11725092_s_at	turquoise	regulatory factor X5(RFX5)	Homo sapiens
1428	11725093_a_at	turquoise	regulatory factor X5(RFX5)	Homo sapiens

1429	11725095_at	turquoise	tetratricopeptide repeat domain 33(TTC33)	Homo sapiens
1430	11725110_a_at	turquoise	nudE neurodevelopment protein 1(NDE1)	Homo sapiens
1431	11725124_at	turquoise	cystatin F(CST7)	Homo sapiens
1432	11725135_a_at	turquoise	VPS16, CORVET/HOPS core subunit(VPS16)	Homo sapiens
1434	11725144_a_at	turquoise	zinc finger protein 784(ZNF784)	Homo sapiens
1435	11725153_a_at	turquoise	nuclear factor kappa B subunit 2(NFKB2)	Homo sapiens
1436	11725155_at	turquoise	four jointed box 1(FJX1)	Homo sapiens
1437	11725174_a_at	turquoise	ArfGAP with dual PH domains 2(ADAP2)	Homo sapiens
1438	11725175_x_at	turquoise	ArfGAP with dual PH domains 2(ADAP2)	Homo sapiens
1439	11725180_a_at	turquoise	runt related transcription factor 2(RUNX2)	Homo sapiens
1440	11725228_a_at	turquoise	pyrimidinergic receptor P2Y6(P2RY6)	Homo sapiens
1441	11725232_at	turquoise	ATPase phospholipid transporting 8B2(ATP8B2)	Homo sapiens
1443	11725238_a_at	turquoise	RNA binding protein with multiple splicing 2(RBPMS2)	Homo sapiens
1444	11725240_at	turquoise	receptor transporter protein 4(RTP4)	Homo sapiens
1445	11725249_at	turquoise	lymphocyte antigen 75(LY75)	Homo sapiens
1446	11725255_a_at	turquoise	interleukin 15(IL15)	Homo sapiens
1447	11725280_a_at	turquoise	PR/SET domain 8(PRDM8)	Homo sapiens
1448	11725315_x_at	turquoise	PRELI domain containing 1(PRELID1)	Homo sapiens
1449	11725351_x_at	turquoise	KIAA0930(KIAA0930)	Homo sapiens
1450	11725352_a_at	turquoise	KIAA0930(KIAA0930)	Homo sapiens
1451	11725373_a_at	turquoise	TNFRSF1A associated via death domain(TRADD)	Homo sapiens
1452	11725379_at	turquoise	protein O-glucosyltransferase 1(POGLUT1)	Homo sapiens
1453	11725380_at	turquoise	protein O-glucosyltransferase 1(POGLUT1)	Homo sapiens
1454	11725385_at	turquoise	UDP-glucose ceramide glucosyltransferase(UGCG)	Homo sapiens
1455	11725389_a_at	turquoise	CD6 molecule(CD6)	Homo sapiens
1456	11725403_at	turquoise	transmembrane 4 L six family member 18(TM4SF18)	Homo sapiens
1457	11725412_s_at	turquoise	tripartite motif containing 14(TRIM14)	Homo sapiens
1458	11725413_x_at	turquoise	tripartite motif containing 14(TRIM14)	Homo sapiens
1459	11725416_at	turquoise	natural killer cell granule protein 7(NKG7)	Homo sapiens
1460	11725419_a_at	turquoise	lysophosphatidylcholine acyltransferase 2(LPCAT2)	Homo sapiens
1461	11725424_a_at	turquoise	RAB27A, member RAS oncogene family(RAB27A)	Homo sapiens
1462	11725425_s_at	turquoise	RAB27A, member RAS oncogene family(RAB27A)	Homo sapiens
1463	11725426_a_at	turquoise	RAB27A, member RAS oncogene family(RAB27A)	Homo sapiens
1464	11725429_at	turquoise	lysosomal associated membrane protein 3(LAMP3)	Homo sapiens
1465	11725437_x_at	turquoise	SPRY domain containing 7(SPRYD7)	Homo sapiens
1466	11725440_a_at	turquoise	lysophosphatidic acid receptor 2(LPAR2)	Homo sapiens
1467	11725444_at	turquoise	C-C motif chemokine receptor 1(CCR1)	Homo sapiens
1468	11725445_x_at	turquoise	microRNA 8085(MIR8085)	Homo sapiens
1469	11725448_at	turquoise	folliculin(FLCN)	Homo sapiens
1470	11725455_at	turquoise	CD247 molecule(CD247)	Homo sapiens
1471	11725471_a_at	turquoise	mannosidase alpha class 2A member 1(MAN2A1)	Homo sapiens
1472	11725472_a_at	turquoise	progestin and adipoQ receptor family member 8(PAQR8)	Homo sapiens
1473	11725473_a_at	turquoise	progestin and adipoQ receptor family member 8(PAQR8)	Homo sapiens
1474	11725476_at	turquoise	ArfGAP with coiled-coil, ankyrin repeat and PH domains 1(ACAP1)	Homo sapiens
1475	11725481_a_at	turquoise	CD27 molecule(CD27)	Homo sapiens
1476	11725485_at	turquoise	disrupted in renal carcinoma 2(DIRC2)	Homo sapiens
1477	11725492_at	turquoise	RAB20, member RAS oncogene family(RAB20)	Homo sapiens
1478	11725498_a_at	turquoise	transmembrane protein 117(TMEM117)	Homo sapiens
1479	11725522_at	turquoise	mesoderm posterior bHLH transcription factor 1(MESP1)	Homo sapiens
1480	11725523_at	turquoise	desmoglein 2(DSG2)	Homo sapiens
1481	11725524_s_at	turquoise	desmoglein 2(DSG2)	Homo sapiens
1483	11725537_at	turquoise	zinc and ring finger 3(ZNRF3)	Homo sapiens
1487	11725617_s_at	turquoise	heterogeneous nuclear ribonucleoprotein R(HNRNPR)	Homo sapiens
1489	11725629_s_at	turquoise	2',3'-cyclic nucleotide 3' phosphodiesterase(CNP)	Homo sapiens
1490	11725634_at	turquoise	mitochondrial ribosomal protein S25(MRPS25)	Homo sapiens
1492	11725636_a_at	turquoise	MOB kinase activator 1A(MOB1A)	Homo sapiens
1493	11725641_at	turquoise	EF-hand domain family member D2(EFHD2)	Homo sapiens
1494	11725642_at	turquoise	EF-hand domain family member D2(EFHD2)	Homo sapiens
1496	11725650_s_at	turquoise	DDB1 and CUL4 associated factor 5(DCAF5)	Homo sapiens
1497	11725657_a_at	turquoise	zinc finger with UFM1 specific peptidase domain(ZUFSP)	Homo sapiens
1499	11725683_at	turquoise	MRS2, magnesium transporter(MRS2)	Homo sapiens
1500	11725684_a_at	turquoise	protein tyrosine phosphatase, receptor type O(PTPRO)	Homo sapiens
1501	11725685_a_at	turquoise	protein tyrosine phosphatase, receptor type O(PTPRO)	Homo sapiens
1502	11725697_at	turquoise	tripartite motif containing 56(TRIM56)	Homo sapiens
1503	11725712_s_at	turquoise	peptidyl-prolyl cis-trans isomerase A pseudogene(LOC101060363)	Homo sapiens
1504	11725729_s_at	turquoise	chromosome 1 open reading frame 56(C1orf56)	Homo sapiens
1505	11725746_a_at	turquoise	phosphatidylinositol transfer protein, cytoplasmic 1(PITPNC1)	Homo sapiens
1506	11725755_a_at	turquoise	butyrophilin subfamily 2 member A2(BTN2A2)	Homo sapiens
1507	11725782_at	turquoise	annexin A2 receptor(ANXA2R)	Homo sapiens
1508	11725789_at	turquoise	dihydropyrimidine dehydrogenase(DPYD)	Homo sapiens
1509	11725790_at	turquoise	dihydropyrimidine dehydrogenase(DPYD)	Homo sapiens
1510	11725793_s_at	turquoise	prostaglandin E receptor 4(PTGER4)	Homo sapiens
1511	11725794_at	turquoise	prostaglandin E receptor 4(PTGER4)	Homo sapiens
1512	11725803_a_at	turquoise	chromosome 21 open reading frame 91(C21orf91)	Homo sapiens
1513	11725804_a_at	turquoise	chromosome 21 open reading frame 91(C21orf91)	Homo sapiens
1514	11725811_at	turquoise	phospholipase A2 group V(PLA2G5)	Homo sapiens
1515	11725813_at	turquoise	microtubule associated serine/threonine kinase 3(MAST3)	Homo sapiens
1516	11725842_a_at	turquoise	PIF1 5'-to-3' DNA helicase(PIF1)	Homo sapiens
1517	11725856_a_at	turquoise	dynein axonemal heavy chain 1(DNAH1)	Homo sapiens
1518	11725861_a_at	turquoise	MYB proto-oncogene like 1(MYBL1)	Homo sapiens
1519	11725876_at	turquoise	glycosylphosphatidylinositol anchored high density lipoprotein binding protein 1(GPIHBP1)	Homo sapiens
1520	11725887_at	turquoise	family with sequence similarity 167 member B(FAM167B)	Homo sapiens

1522	11725899_a_at	turquoise	leucine rich repeat containing 8 family member D(LRRC8D)	Homo sapiens
1523	11725910_a_at	turquoise	collagen type IV alpha 6 chain(COL4A6)	Homo sapiens
1524	11725912_a_at	turquoise	erb-b2 receptor tyrosine kinase 4(ERBB4)	Homo sapiens
1525	11725917_s_at	turquoise	ring finger protein 19B(RNF19B)	Homo sapiens
1526	11725924_a_at	turquoise	Ras association domain family member 5(RASSF5)	Homo sapiens
1527	11725935_at	turquoise	calcium/calmodulin dependent protein kinase II alpha(CAMK2A)	Homo sapiens
1529	11725966_a_at	turquoise	integrin subunit beta 7(ITGB7)	Homo sapiens
1530	11725970_x_at	turquoise	heterogeneous nuclear ribonucleoprotein F(HNRNPF)	Homo sapiens
1531	11725971_a_at	turquoise	heterogeneous nuclear ribonucleoprotein F(HNRNPF)	Homo sapiens
1532	11725972_s_at	turquoise	heterogeneous nuclear ribonucleoprotein F(HNRNPF)	Homo sapiens
1533	11725973_x_at	turquoise	heterogeneous nuclear ribonucleoprotein F(HNRNPF)	Homo sapiens
1534	11725981_at	turquoise	C-C motif chemokine receptor 7(CCR7)	Homo sapiens
1535	11725983_at	turquoise	basic helix-loop-helix family member e40(BHLHE40)	Homo sapiens
1537	11725998_a_at	turquoise	Fas cell surface death receptor(FAS)	Homo sapiens
1538	11725999_s_at	turquoise	Fas cell surface death receptor(FAS)	Homo sapiens
1539	11726000_x_at	turquoise	copine 4(CPNE4)	Homo sapiens
1540	11726004_a_at	turquoise	layilin(LAYN)	Homo sapiens
1541	11726034_a_at	turquoise	protein tyrosine phosphatase, non-receptor type 6(PTPN6)	Homo sapiens
1543	11726040_at	turquoise	serine/threonine kinase 17b(STK17B)	Homo sapiens
1544	11726041_x_at	turquoise	serine/threonine kinase 17b(STK17B)	Homo sapiens
1545	11726042_a_at	turquoise	serine/threonine kinase 17b(STK17B)	Homo sapiens
1546	11726056_a_at	turquoise	protein phosphatase 4 regulatory subunit 3B(PPP4R3B)	Homo sapiens
1548	11726088_s_at	turquoise	phosphatase and actin regulator 3(PHACTR3)	Homo sapiens
1549	11726089_at	turquoise	VPS53, GARP complex subunit(VPS53)	Homo sapiens
1551	11726120_a_at	turquoise	BMP2 inducible kinase(BMP2K)	Homo sapiens
1552	11726122_at	turquoise	myosin IF(MYO1F)	Homo sapiens
1554	11726158_at	turquoise	spindlin family member 4(SPIN4)	Homo sapiens
1555	11726168_at	turquoise	progesterone receptor(PGR)	Homo sapiens
1556	11726178_a_at	turquoise	SRY-box 6(SOX6)	Homo sapiens
1557	11726179_a_at	turquoise	SRY-box 6(SOX6)	Homo sapiens
1558	11726180_a_at	turquoise	SRY-box 6(SOX6)	Homo sapiens
1559	11726187_at	turquoise	shisa family member 3(SHISA3)	Homo sapiens
1560	11726188_at	turquoise	shisa family member 3(SHISA3)	Homo sapiens
1561	11726190_a_at	turquoise	dedicator of cytokinesis 4(DOCK4)	Homo sapiens
1562	11726201_a_at	turquoise	2'-5'-oligoadenylate synthetase 2(OAS2)	Homo sapiens
1563	11726217_a_at	turquoise	PR/SET domain 1(PRDM1)	Homo sapiens
1564	11726218_a_at	turquoise	PR/SET domain 1(PRDM1)	Homo sapiens
1565	11726242_a_at	turquoise	transketolase like 1(TKTL1)	Homo sapiens
1566	11726254_s_at	turquoise	CD74 molecule(CD74)	Homo sapiens
1567	11726255_x_at	turquoise	CD74 molecule(CD74)	Homo sapiens
1569	11726286_a_at	turquoise	tryptophanyl-tRNA synthetase(WARS)	Homo sapiens
1570	11726287_a_at	turquoise	tryptophanyl-tRNA synthetase(WARS)	Homo sapiens
1571	11726292_s_at	turquoise	WD repeat domain 44(WDR44)	Homo sapiens
1572	11726293_x_at	turquoise	WD repeat domain 44(WDR44)	Homo sapiens
1573	11726294_s_at	turquoise	CCAAT/enhancer binding protein alpha(CEBPA)	Homo sapiens
1574	11726309_a_at	turquoise	glycine receptor beta(GLRB)	Homo sapiens
1575	11726310_at	turquoise	glycine receptor beta(GLRB)	Homo sapiens
1576	11726311_at	turquoise	glycine receptor beta(GLRB)	Homo sapiens
1577	11726315_a_at	turquoise	methyltransferase like 22(METTL22)	Homo sapiens
1578	11726316_at	turquoise	selectin E(SELE)	Homo sapiens
1579	11726321_a_at	turquoise	endoplasmic reticulum oxidoreductase 1 alpha(ERO1A)	Homo sapiens
1580	11726324_at	turquoise	cytokine receptor like factor 3(CRLF3)	Homo sapiens
1581	11726328_x_at	turquoise	guanylate binding protein 1(GBP1)	Homo sapiens
1582	11726329_x_at	turquoise	guanylate binding protein 1(GBP1)	Homo sapiens
1583	11726333_s_at	turquoise	lymphoid enhancer binding factor 1(LEF1)	Homo sapiens
1585	11726337_a_at	turquoise	arachidonate 5-lipoxygenase(ALOX5)	Homo sapiens
1586	11726338_a_at	turquoise	arachidonate 5-lipoxygenase(ALOX5)	Homo sapiens
1587	11726347_x_at	turquoise	linker for activation of T-cells family member 2(LAT2)	Homo sapiens
1589	11726353_at	turquoise	CD180 molecule(CD180)	Homo sapiens
1590	11726364_x_at	turquoise	2'-5'-oligoadenylate synthetase like(OASL)	Homo sapiens
1591	11726366_s_at	turquoise	glutamate rich 1(ERICH1)	Homo sapiens
1592	11726368_x_at	turquoise	glutamate rich 1(ERICH1)	Homo sapiens
1593	11726369_at	turquoise	GRIK1 antisense RNA 2(GRIK1-AS2)	Homo sapiens
1594	11726375_x_at	turquoise	G protein-coupled receptor 34(GPR34)	Homo sapiens
1595	11726378_a_at	turquoise	Wolf-Hirschhorn syndrome candidate 1(WHSC1)	Homo sapiens
1596	11726382_a_at	turquoise	signaling lymphocytic activation molecule family member 1(SLAMF1)	Homo sapiens
1597	11726392_at	turquoise	GTPase, IMAP family member 2(GIMAP2)	Homo sapiens
1598	11726410_at	turquoise	GID complex subunit 4 homolog(GID4)	Homo sapiens
1599	11726416_a_at	turquoise	abl interactor 1(ABI1)	Homo sapiens
1600	11726417_x_at	turquoise	abl interactor 1(ABI1)	Homo sapiens
1601	11726418_x_at	turquoise	abl interactor 1(ABI1)	Homo sapiens
1602	11726421_a_at	turquoise	amyloid beta precursor protein binding family B member 1 interacting protein(APBB1IP)	Homo sapiens
1603	11726432_a_at	turquoise	oxysterol binding protein like 3(OSBPL3)	Homo sapiens
1604	11726433_a_at	turquoise	oxysterol binding protein like 3(OSBPL3)	Homo sapiens
1605	11726464_a_at	turquoise	IGF like family receptor 1(IGFLR1)	Homo sapiens
1606	11726473_a_at	turquoise	docking protein 3(DOK3)	Homo sapiens
1607	11726479_a_at	turquoise	MX dynamin like GTPase 2(MX2)	Homo sapiens
1608	11726494_a_at	turquoise	SUMO/sentrin peptidase family member, NEDD8 specific(SENP8)	Homo sapiens
1610	11726517_s_at	turquoise	WD repeat domain 11(WDR11)	Homo sapiens
1611	11726528_at	turquoise	family with sequence similarity 81 member A(FAM81A)	Homo sapiens
1612	11726532_a_at	turquoise	cytohesin 4(CYTH4)	Homo sapiens
1613	11726542_at	turquoise	transmembrane protein 132C(TMEM132C)	Homo sapiens

1614	11726557_a_at	turquoise	gasdermin B(GSDMB)	Homo sapiens
1615	11726559_s_at	turquoise	lymphocyte cytosolic protein 1(LCP1)	Homo sapiens
1616	11726579_a_at	turquoise	proteasome activator subunit 1(PSME1)	Homo sapiens
1617	11726580_x_at	turquoise	proteasome activator subunit 1(PSME1)	Homo sapiens
1618	11726594_at	turquoise	cathepsin Z(CTSZ)	Homo sapiens
1619	11726608_s_at	turquoise	metal response element binding transcription factor 2(MTF2)	Homo sapiens
1620	11726610_s_at	turquoise	metal response element binding transcription factor 2(MTF2)	Homo sapiens
1621	11726611_x_at	turquoise	MAF bZIP transcription factor F(MAFF)	Homo sapiens
1622	11726617_s_at	turquoise	cyclin dependent kinase 1(CDK1)	Homo sapiens
1623	11726625_at	turquoise	cyclin G2(CCNG2)	Homo sapiens
1624	11726628_a_at	turquoise	semaphorin 4A(SEMA4A)	Homo sapiens
1625	11726647_s_at	turquoise	butyrophilin subfamily 3 member A2(BTN3A2)	Homo sapiens
1626	11726655_at	turquoise	interferon induced protein with tetratricopeptide repeats 5(IFIT5)	Homo sapiens
1627	11726656_a_at	turquoise	interferon induced protein with tetratricopeptide repeats 5(IFIT5)	Homo sapiens
1629	11726671_a_at	turquoise	DNA cross-link repair 1B(DCLRE1B)	Homo sapiens
1631	11726677_a_at	turquoise	proteasome subunit beta 9(PSMB9)	Homo sapiens
1632	11726689_a_at	turquoise	signal transducer and activator of transcription 1(STAT1)	Homo sapiens
1633	11726690_a_at	turquoise	signal transducer and activator of transcription 1(STAT1)	Homo sapiens
1634	11726700_a_at	turquoise	aftiphilin(AFTPH)	Homo sapiens
1635	11726701_x_at	turquoise	aftiphilin(AFTPH)	Homo sapiens
1636	11726702_s_at	turquoise	aftiphilin(AFTPH)	Homo sapiens
1637	11726703_a_at	turquoise	B-cell CLL/lymphoma 10(BCL10)	Homo sapiens
1638	11726704_x_at	turquoise	B-cell CLL/lymphoma 10(BCL10)	Homo sapiens
1639	11726707_x_at	turquoise	interleukin 34(IL34)	Homo sapiens
1640	11726723_a_at	turquoise	WD repeat and FYVE domain containing 2(WDFY2)	Homo sapiens
1641	11726725_a_at	turquoise	nucleic acid binding protein 1(NABP1)	Homo sapiens
1642	11726726_s_at	turquoise	nucleic acid binding protein 1(NABP1)	Homo sapiens
1643	11726727_a_at	turquoise	nucleic acid binding protein 1(NABP1)	Homo sapiens
1644	11726769_a_at	turquoise	XIAP associated factor 1(XAF1)	Homo sapiens
1645	11726770_x_at	turquoise	XIAP associated factor 1(XAF1)	Homo sapiens
1646	11726771_a_at	turquoise	XIAP associated factor 1(XAF1)	Homo sapiens
1647	11726777_a_at	turquoise	linker for activation of T-cells(LAT)	Homo sapiens
1648	11726779_a_at	turquoise	IKBKB interacting protein(IKBIP)	Homo sapiens
1649	11726803_x_at	turquoise	helicase with zinc finger 2(HELZ2)	Homo sapiens
1650	11726820_a_at	turquoise	Rho GTPase activating protein 9(ARHGAP9)	Homo sapiens
1651	11726822_a_at	turquoise	chromosome X open reading frame 36(CXorf36)	Homo sapiens
1652	11726839_s_at	turquoise	RELT like 1(RELL1)	Homo sapiens
1653	11726869_a_at	turquoise	major facilitator superfamily domain containing 12(MFSD12)	Homo sapiens
1654	11726875_a_at	turquoise	promyelocytic leukemia(PML)	Homo sapiens
1655	11726876_a_at	turquoise	promyelocytic leukemia(PML)	Homo sapiens
1656	11726883_s_at	turquoise	ATPase family, AAA domain containing 2B(ATAD2B)	Homo sapiens
1657	11726893_a_at	turquoise	microRNA 6132(MIR6132)	Homo sapiens
1658	11726928_s_at	turquoise	interleukin 18 binding protein(IL18BP)	Homo sapiens
1659	11726947_a_at	turquoise	toll like receptor 8(TLR8)	Homo sapiens
1660	11726948_at	turquoise	toll like receptor 8(TLR8)	Homo sapiens
1661	11726963_s_at	turquoise	small glutamine rich tetratricopeptide repeat containing beta(SGTB)	Homo sapiens
1664	11727005_at	turquoise	adhesion G protein-coupled receptor L4(ADGRL4)	Homo sapiens
1665	11727006_a_at	turquoise	T-cell activation RhoGTPase activating protein(TAGAP)	Homo sapiens
1666	11727009_at	turquoise	aldehyde dehydrogenase 7 family member A1(ALDH7A1)	Homo sapiens
1667	11727022_at	turquoise	transmembrane protein 64(TMEM64)	Homo sapiens
1668	11727044_a_at	turquoise	transmembrane protein 173(TMEM173)	Homo sapiens
1669	11727058_at	turquoise	mitochondrial ribosomal protein S36(MRPS36)	Homo sapiens
1670	11727059_x_at	turquoise	mitochondrial ribosomal protein S36(MRPS36)	Homo sapiens
1672	11727062_at	turquoise	PIR-FIGF readthrough(PIR-FIGF)	Homo sapiens
1673	11727080_at	turquoise	NUFIP1, FMR1 interacting protein 1(NUFIP1)	Homo sapiens
1674	11727092_x_at	turquoise	interleukin 18(IL18)	Homo sapiens
1675	11727111_a_at	turquoise	formin binding protein 1(FNBP1)	Homo sapiens
1676	11727112_a_at	turquoise	signaling threshold regulating transmembrane adaptor 1(SIT1)	Homo sapiens
1677	11727116_a_at	turquoise	phospholipase A1 member A(PLA1A)	Homo sapiens
1678	11727118_a_at	turquoise	complement factor properdin(CFP)	Homo sapiens
1679	11727120_a_at	turquoise	MYC binding protein 2, E3 ubiquitin protein ligase(MYCBP2)	Homo sapiens
1680	11727121_at	turquoise	MYC binding protein 2, E3 ubiquitin protein ligase(MYCBP2)	Homo sapiens
1681	11727141_s_at	turquoise	erythrocyte membrane protein band 4.1 like 3(EPB41L3)	Homo sapiens
1682	11727142_a_at	turquoise	erythrocyte membrane protein band 4.1 like 3(EPB41L3)	Homo sapiens
1683	11727143_x_at	turquoise	erythrocyte membrane protein band 4.1 like 3(EPB41L3)	Homo sapiens
1684	11727146_at	turquoise	G protein-coupled receptor 160(GPR160)	Homo sapiens
1685	11727186_a_at	turquoise	Wnt family member 5A(WNT5A)	Homo sapiens
1686	11727210_at	turquoise	Purkinje cell protein 2(PCP2)	Homo sapiens
1687	11727219_a_at	turquoise	dermatan sulfate epimerase(DSE)	Homo sapiens
1688	11727224_a_at	turquoise	polypeptide N-acetylgalactosaminyltransferase 16(GALNT16)	Homo sapiens
1689	11727225_a_at	turquoise	polypeptide N-acetylgalactosaminyltransferase 16(GALNT16)	Homo sapiens
1690	11727254_a_at	turquoise	transmembrane protein 51(TMEM51)	Homo sapiens
1691	11727257_a_at	turquoise	Janus kinase 3(JAK3)	Homo sapiens
1692	11727291_at	turquoise	TEN1-CDK3 readthrough (NMD candidate)(TEN1-CDK3)	Homo sapiens
1693	11727292_a_at	turquoise	chromosome 1 open reading frame 162(C1orf162)	Homo sapiens
1694	11727321_at	turquoise	elastin microfibril interfacer 3(EMILIN3)	Homo sapiens
1695	11727327_x_at	turquoise	family with sequence similarity 131 member C(FAM131C)	Homo sapiens
1696	11727360_a_at	turquoise	gap junction protein delta 3(GJD3)	Homo sapiens
1697	11727372_at	turquoise	3'-phosphoadenosine 5'-phosphosulfate synthase 1(PAPSS1)	Homo sapiens
1698	11727373_s_at	turquoise	3'-phosphoadenosine 5'-phosphosulfate synthase 1(PAPSS1)	Homo sapiens
1701	11727428_at	turquoise	G protein-coupled receptor 183(GPR183)	Homo sapiens
1702	11727456_a_at	turquoise	OFD1, centriole and centriolar satellite protein(OFD1)	Homo sapiens

1703	11727467_x_at	turquoise	interleukin 10 receptor subunit beta(IL10RB)	Homo sapiens
1704	11727478_a_at	turquoise	angiotensin II receptor associated protein(AGTRAP)	Homo sapiens
1705	11727489_a_at	turquoise	kinesin family member 11(KIF11)	Homo sapiens
1706	11727497_x_at	turquoise	zinc finger RANBP2-type containing 1(ZRANB1)	Homo sapiens
1707	11727499_s_at	turquoise	zinc finger RANBP2-type containing 1(ZRANB1)	Homo sapiens
1708	11727516_at	turquoise	ribonucleoprotein, PTB binding 2(RAVER2)	Homo sapiens
1709	11727522_a_at	turquoise	zinc finger protein 267(ZNF267)	Homo sapiens
1710	11727523_x_at	turquoise	zinc finger protein 267(ZNF267)	Homo sapiens
1711	11727532_a_at	turquoise	fasciculation and elongation protein zeta 2(FEZ2)	Homo sapiens
1712	11727533_a_at	turquoise	fasciculation and elongation protein zeta 2(FEZ2)	Homo sapiens
1713	11727538_at	turquoise	NME/NM23 family member 5(NME5)	Homo sapiens
1714	11727539_x_at	turquoise	iron-sulfur cluster assembly enzyme(ISCU)	Homo sapiens
1715	11727547_s_at	turquoise	RB binding protein 8, endonuclease(RBBP8)	Homo sapiens
1716	11727561_x_at	turquoise	transcription factor 7 (T-cell specific, HMG-box)(TCF7)	Homo sapiens
1717	11727565_a_at	turquoise	tumor necrosis factor superfamily member 13b(TNFSF13B)	Homo sapiens
1718	11727572_s_at	turquoise	kelch like family member 6(KLHL6)	Homo sapiens
1719	11727574_at	turquoise	retinol binding protein 5(RBP5)	Homo sapiens
1720	11727577_a_at	turquoise	transmembrane protein 237(TMEM237)	Homo sapiens
1721	11727600_a_at	turquoise	TNF receptor superfamily member 4(TNFRSF4)	Homo sapiens
1722	11727609_at	turquoise	killer cell lectin like receptor B1(KLRB1)	Homo sapiens
1723	11727622_a_at	turquoise	vitrin(VIT)	Homo sapiens
1724	11727643_s_at	turquoise	transcriptional regulating factor 1(TRERF1)	Homo sapiens
1725	11727677_at	turquoise	biogenesis of lysosomal organelles complex 1 subunit 3(BLOC1S3)	Homo sapiens
1726	11727694_a_at	turquoise	KLRC4-KLRK1 readthrough(KLRC4-KLRK1)	Homo sapiens
1727	11727695_a_at	turquoise	KLRC4-KLRK1 readthrough(KLRC4-KLRK1)	Homo sapiens
1728	11727701_a_at	turquoise	family with sequence similarity 185 member A(FAM185A)	Homo sapiens
1729	11727707_a_at	turquoise	microRNA 3191(MIR3191)	Homo sapiens
1730	11727740_a_at	turquoise	CD300 molecule like family member f(CD300LF)	Homo sapiens
1731	11727775_s_at	turquoise	G protein subunit alpha i3(GNAI3)	Homo sapiens
1732	11727776_at	turquoise	G protein subunit alpha i3(GNAI3)	Homo sapiens
1733	11727778_a_at	turquoise	G protein subunit alpha i3(GNAI3)	Homo sapiens
1734	11727779_at	turquoise	growth hormone inducible transmembrane protein(GHITM)	Homo sapiens
1735	11727782_a_at	turquoise	tropomyosin 4(TPM4)	Homo sapiens
1736	11727783_s_at	turquoise	tropomyosin 4(TPM4)	Homo sapiens
1740	11727797_at	turquoise	Nedd4 family interacting protein 2(NDFIP2)	Homo sapiens
1741	11727816_at	turquoise	ubiquitin associated and SH3 domain containing B(UBASH3B)	Homo sapiens
1742	11727817_at	turquoise	ubiquitin associated and SH3 domain containing B(UBASH3B)	Homo sapiens
1744	11727821_s_at	turquoise	CDGSH iron sulfur domain 1(CISD1)	Homo sapiens
1745	11727835_a_at	turquoise	Enah/Vasp-like(EVL)	Homo sapiens
1746	11727841_a_at	turquoise	protein kinase C delta(PRKCD)	Homo sapiens
1747	11727853_a_at	turquoise	nucleoporin 50(NUP50)	Homo sapiens
1748	11727854_s_at	turquoise	nucleoporin 50(NUP50)	Homo sapiens
1749	11727855_a_at	turquoise	nucleoporin 50(NUP50)	Homo sapiens
1750	11727856_s_at	turquoise	nucleoporin 50(NUP50)	Homo sapiens
1751	11727876_at	turquoise	cytochrome b-245 beta chain(CYBB)	Homo sapiens
1752	11727877_a_at	turquoise	MIA-RAB4B readthrough (NMD candidate)(MIA-RAB4B)	Homo sapiens
1753	11727891_at	turquoise	proline and arginine rich end leucine rich repeat protein(PRELP)	Homo sapiens
1754	11727892_at	turquoise	proline and arginine rich end leucine rich repeat protein(PRELP)	Homo sapiens
1755	11727894_a_at	turquoise	G kinase anchoring protein 1(GKAP1)	Homo sapiens
1756	11727904_s_at	turquoise	interleukin 13 receptor subunit alpha 1(IL13RA1)	Homo sapiens
1757	11727905_a_at	turquoise	interleukin 13 receptor subunit alpha 1(IL13RA1)	Homo sapiens
1758	11727913_a_at	turquoise	formin like 1(FMNL1)	Homo sapiens
1759	11727920_a_at	turquoise	osteoclast stimulating factor 1(OSTF1)	Homo sapiens
1760	11727930_a_at	turquoise	ATPase plasma membrane Ca2+ transporting 1(ATP2B1)	Homo sapiens
1761	11727936_a_at	turquoise	SLC9A3 regulator 1(SLC9A3R1)	Homo sapiens
1762	11727942_a_at	turquoise	formyl peptide receptor 3(FPR3)	Homo sapiens
1763	11727943_a_at	turquoise	formyl peptide receptor 3(FPR3)	Homo sapiens
1764	11727970_a_at	turquoise	solute carrier family 14 member 1 (Kidd blood group)(SLC14A1)	Homo sapiens
1765	11727992_at	turquoise	syntaxin 11(STX11)	Homo sapiens
1766	11727994_at	turquoise	syntaxin 11(STX11)	Homo sapiens
1770	11728033_at	turquoise	SFT2 domain containing 2(SFT2D2)	Homo sapiens
1771	11728038_at	turquoise	C-C motif chemokine ligand 8(CCL8)	Homo sapiens
1772	11728039_s_at	turquoise	C-C motif chemokine ligand 8(CCL8)	Homo sapiens
1774	11728055_at	turquoise	growth hormone receptor(GHR)	Homo sapiens
1775	11728056_a_at	turquoise	caspase 10(CASP10)	Homo sapiens
1776	11728062_a_at	turquoise	SH3 domain binding protein 1(SH3BP1)	Homo sapiens
1777	11728063_x_at	turquoise	SH3 domain binding protein 1(SH3BP1)	Homo sapiens
1778	11728077_s_at	turquoise	protein kinase C theta(PRKCQ)	Homo sapiens
1779	11728093_a_at	turquoise	mitogen-activated protein kinase kinase kinase 1(MAP3K1)	Homo sapiens
1780	11728094_s_at	turquoise	mitogen-activated protein kinase kinase kinase 1(MAP3K1)	Homo sapiens
1781	11728125_a_at	turquoise	granzyme H(GZMH)	Homo sapiens
1782	11728137_at	turquoise	X-linked Kx blood group(XK)	Homo sapiens
1783	11728146_a_at	turquoise	aquaporin 11(AQP11)	Homo sapiens
1785	11728154_s_at	turquoise	fucosyltransferase 4(FUT4)	Homo sapiens
1786	11728157_at	turquoise	ectodysplasin A(EDA)	Homo sapiens
1787	11728178_at	turquoise	sirtuin 4(SIRT4)	Homo sapiens
1788	11728189_a_at	turquoise	C-X-C motif chemokine receptor 4(CXCR4)	Homo sapiens
1789	11728190_s_at	turquoise	C-X-C motif chemokine receptor 4(CXCR4)	Homo sapiens
1790	11728191_x_at	turquoise	C-X-C motif chemokine receptor 4(CXCR4)	Homo sapiens
1791	11728207_a_at	turquoise	XPC complex subunit, DNA damage recognition and repair factor(XPC)	Homo sapiens
1792	11728223_at	turquoise	ubiquitin conjugating enzyme E2 J1(UBE2J1)	Homo sapiens
1793	11728224_a_at	turquoise	ubiquitin conjugating enzyme E2 J1(UBE2J1)	Homo sapiens

1794	11728225_at	turquoise	ubiquitin conjugating enzyme E2 J1(UBE2J1)	Homo sapiens
1795	11728228_at	turquoise	hepatitis A virus cellular receptor 2(HAVCR2)	Homo sapiens
1796	11728229_a_at	turquoise	hepatitis A virus cellular receptor 2(HAVCR2)	Homo sapiens
1797	11728236_at	turquoise	CD5 molecule(CD5)	Homo sapiens
1798	11728245_a_at	turquoise	angiopoietin 2(ANGPT2)	Homo sapiens
1799	11728265_a_at	turquoise	leukocyte immunoglobulin like receptor B2(LILRB2)	Homo sapiens
1800	11728266_a_at	turquoise	leukocyte immunoglobulin like receptor B2(LILRB2)	Homo sapiens
1801	11728267_s_at	turquoise	leukocyte immunoglobulin like receptor B2(LILRB2)	Homo sapiens
1802	11728276_s_at	turquoise	phospholipase A2 group XIIA(PLA2G12A)	Homo sapiens
1803	11728277_x_at	turquoise	phospholipase A2 group XIIA(PLA2G12A)	Homo sapiens
1804	11728278_at	turquoise	phospholipase A2 group XIIA(PLA2G12A)	Homo sapiens
1805	11728279_at	turquoise	phospholipase A2 group XIIA(PLA2G12A)	Homo sapiens
1806	11728291_at	turquoise	SH2 domain containing 1A(SH2D1A)	Homo sapiens
1807	11728292_at	turquoise	SH2 domain containing 1A(SH2D1A)	Homo sapiens
1811	11728347_at	turquoise	ABI family member 3(ABI3)	Homo sapiens
1812	11728350_s_at	turquoise	stromal interaction molecule 2(STIM2)	Homo sapiens
1813	11728373_a_at	turquoise	ERG, ETS transcription factor(ERG)	Homo sapiens
1814	11728374_s_at	turquoise	actinin alpha 1(ACTN1)	Homo sapiens
1815	11728375_x_at	turquoise	actinin alpha 1(ACTN1)	Homo sapiens
1816	11728378_a_at	turquoise	embryonic ectoderm development(EED)	Homo sapiens
1818	11728396_a_at	turquoise	sorting nexin 14(SNX14)	Homo sapiens
1819	11728401_at	turquoise	ATPase H+ transporting V0 subunit a2(ATP6V0A2)	Homo sapiens
1820	11728405_s_at	turquoise	negative regulator of reactive oxygen species(NRROS)	Homo sapiens
1821	11728413_at	turquoise	serpin family B member 8(SERPINB8)	Homo sapiens
1822	11728421_a_at	turquoise	phospholipase A2 group VII(PLA2G7)	Homo sapiens
1823	11728423_a_at	turquoise	paired immunoglobin like type 2 receptor alpha(PILRA)	Homo sapiens
1824	11728424_x_at	turquoise	paired immunoglobin like type 2 receptor alpha(PILRA)	Homo sapiens
1826	11728430_a_at	turquoise	hes related family bHLH transcription factor with YRPW motif 2(HEY2)	Homo sapiens
1827	11728446_a_at	turquoise	phospholipase C eta 1(PLCH1)	Homo sapiens
1828	11728489_a_at	turquoise	C-type lectin domain family 4 member A(CLEC4A)	Homo sapiens
1829	11728503_at	turquoise	CD300c molecule(CD300C)	Homo sapiens
1830	11728507_at	turquoise	lymphocyte antigen 96(LY96)	Homo sapiens
1831	11728512_a_at	turquoise	TNF receptor associated factor 5(TRAF5)	Homo sapiens
1833	11728514_a_at	turquoise	NADH:ubiquinone oxidoreductase complex assembly factor 6(NDUFAF6)	Homo sapiens
1834	11728515_a_at	turquoise	SP140 nuclear body protein(SP140)	Homo sapiens
1835	11728523_a_at	turquoise	v-myc avian myelocytomatosis viral oncogene lung carcinoma derived homolog(MYCL)	Homo sapiens
1836	11728530_a_at	turquoise	B-cell linker(BLNK)	Homo sapiens
1837	11728543_a_at	turquoise	maestro(MRO)	Homo sapiens
1838	11728560_at	turquoise	granzyme K(GZMK)	Homo sapiens
1839	11728565_x_at	turquoise	family with sequence similarity 210 member A(FAM210A)	Homo sapiens
1841	11728570_at	turquoise	zinc finger protein, FOG family member 2(ZFPM2)	Homo sapiens
1842	11728592_at	turquoise	leucine rich repeat containing 70(LRRC70)	Homo sapiens
1843	11728595_a_at	turquoise	caveolin 3(CAV3)	Homo sapiens
1844	11728617_at	turquoise	caspase recruitment domain family member 6(CARD6)	Homo sapiens
1845	11728640_s_at	turquoise	EDAR associated death domain(EDARADD)	Homo sapiens
1846	11728643_s_at	turquoise	ARP3 actin related protein 3 homolog(ACTR3)	Homo sapiens
1848	11728676_at	turquoise	mediator complex subunit 14(MED14)	Homo sapiens
1849	11728679_a_at	turquoise	CD163 molecule(CD163)	Homo sapiens
1850	11728680_s_at	turquoise	poly(A) binding protein interacting protein 1(PAIP1)	Homo sapiens
1851	11728702_s_at	turquoise	KIAA0232(KIAA0232)	Homo sapiens
1852	11728706_x_at	turquoise	epithelial membrane protein 2(EMP2)	Homo sapiens
1853	11728711_a_at	turquoise	nuclear factor of activated T-cells 2 interacting protein(NFATC2IP)	Homo sapiens
1854	11728720_at	turquoise	ras homolog family member H(RHOH)	Homo sapiens
1856	11728749_a_at	turquoise	transcription factor 19(TCF19)	Homo sapiens
1857	11728761_a_at	turquoise	uncharacterized LOC100506403(LOC100506403)	Homo sapiens
1858	11728766_a_at	turquoise	kelch like family member 5(KLHL5)	Homo sapiens
1859	11728767_s_at	turquoise	kelch like family member 5(KLHL5)	Homo sapiens
1860	11728768_s_at	turquoise	kelch like family member 5(KLHL5)	Homo sapiens
1861	11728776_s_at	turquoise	vitamin D (1,25- dihydroxyvitamin D3) receptor(VDR)	Homo sapiens
1862	11728780_a_at	turquoise	zeta chain of T cell receptor associated protein kinase 70(ZAP70)	Homo sapiens
1863	11728781_a_at	turquoise	CD1c molecule(CD1C)	Homo sapiens
1864	11728810_a_at	turquoise	enoyl-CoA hydratase domain containing 3(ECHDC3)	Homo sapiens
1865	11728811_at	turquoise	OTU deubiquitinase 7B(OTUD7B)	Homo sapiens
1869	11728846_at	turquoise	heart and neural crest derivatives expressed 1(HAND1)	Homo sapiens
1870	11728852_a_at	turquoise	tubby bipartite transcription factor(TUB)	Homo sapiens
1871	11728863_at	turquoise	GINS complex subunit 1(GINS1)	Homo sapiens
1872	11728892_at	turquoise	tubulin polymerization promoting protein(TPPP)	Homo sapiens
1873	11728901_a_at	turquoise	dual adaptor of phosphotyrosine and 3-phosphoinositides 1(DAPP1)	Homo sapiens
1874	11728920_a_at	turquoise	RAS guanyl releasing protein 1(RASGRP1)	Homo sapiens
1875	11728926_at	turquoise	NFAT activating protein with ITAM motif 1(NFAM1)	Homo sapiens
1876	11728937_at	turquoise	GDNF family receptor alpha 2(GFRA2)	Homo sapiens
1877	11728944_a_at	turquoise	leukocyte specific transcript 1(LST1)	Homo sapiens
1878	11728945_x_at	turquoise	leukocyte specific transcript 1(LST1)	Homo sapiens
1879	11728961_a_at	turquoise	TSNAX-DISC1 readthrough (NMD candidate)(TSNAX-DISC1)	Homo sapiens
1880	11728978_x_at	turquoise	caspase 8(CASP8)	Homo sapiens
1881	11728984_a_at	turquoise	monofunctional C1-tetrahydrofolate synthase, mitochondrial-like(LOC100996643)	Homo sapiens
1882	11728985_s_at	turquoise	monofunctional C1-tetrahydrofolate synthase, mitochondrial-like(LOC100996643)	Homo sapiens
1883	11728986_x_at	turquoise	monofunctional C1-tetrahydrofolate synthase, mitochondrial-like(LOC100996643)	Homo sapiens
1884	11729000_x_at	turquoise	CD84 molecule(CD84)	Homo sapiens
1885	11729023_at	turquoise	potassium voltage-gated channel subfamily A member 5(KCNA5)	Homo sapiens
1886	11729033_at	turquoise	inositol 1,4,5-trisphosphate receptor interacting protein like 2(ITPRIPL2)	Homo sapiens
1887	11729035_at	turquoise	inositol 1,4,5-trisphosphate receptor interacting protein like 2(ITPRIPL2)	Homo sapiens

1888	11729074_a_at	turquoise	hydroxylysine kinase(HYKK)	Homo sapiens
1889	11729101_a_at	turquoise	aldo-keto reductase family 1 member C2(AKR1C2)	Homo sapiens
1890	11729110_s_at	turquoise	ADAM like decysin 1(ADAMDEC1)	Homo sapiens
1891	11729111_x_at	turquoise	ADAM like decysin 1(ADAMDEC1)	Homo sapiens
1897	11729138_a_at	turquoise	SLAM family member 7(SLAMF7)	Homo sapiens
1898	11729139_x_at	turquoise	SLAM family member 7(SLAMF7)	Homo sapiens
1899	11729140_x_at	turquoise	SLAM family member 7(SLAMF7)	Homo sapiens
1901	11729163_a_at	turquoise	BTG anti-proliferation factor 3(BTG3)	Homo sapiens
1902	11729191_s_at	turquoise	MYC associated factor X(MAX)	Homo sapiens
1903	11729202_at	turquoise	Kazal type serine peptidase inhibitor domain 1(KAZALD1)	Homo sapiens
1904	11729203_a_at	turquoise	plexin D1(PLXND1)	Homo sapiens
1905	11729219_at	turquoise	NCK adaptor protein 1(NCK1)	Homo sapiens
1906	11729220_at	turquoise	carboxymethylenebutenolidase homolog(CMBL)	Homo sapiens
1907	11729222_a_at	turquoise	GATA binding protein 3(GATA3)	Homo sapiens
1908	11729223_at	turquoise	GATA binding protein 3(GATA3)	Homo sapiens
1909	11729226_x_at	turquoise	integrin subunit alpha L(ITGAL)	Homo sapiens
1911	11729247_at	turquoise	sirtuin 5(SIRT5)	Homo sapiens
1912	11729251_at	turquoise	myeloid cell nuclear differentiation antigen(MNDA)	Homo sapiens
1913	11729263_x_at	turquoise	serpin family B member 9(SERPINB9)	Homo sapiens
1914	11729272_a_at	turquoise	ankyrin repeat and SOCS box containing 1(ASB1)	Homo sapiens
1915	11729274_a_at	turquoise	guanylate binding protein 5(GBP5)	Homo sapiens
1918	11729338_x_at	turquoise	proline-serine-threonine phosphatase interacting protein 2(PSTPIP2)	Homo sapiens
1919	11729357_x_at	turquoise	poly(A) RNA polymerase D4, non-canonical(PAPD4)	Homo sapiens
1920	11729410_a_at	turquoise	zinc finger protein 415(ZNF415)	Homo sapiens
1921	11729419_a_at	turquoise	ATPase sarcoplasmic/endoplasmic reticulum Ca2+ transporting 3(ATP2A3)	Homo sapiens
1923	11729424_s_at	turquoise	C-C motif chemokine receptor like 2(CCRL2)	Homo sapiens
1924	11729431_a_at	turquoise	phosphatase domain containing, paladin 1(PALD1)	Homo sapiens
1925	11729442_a_at	turquoise	TEPSIN, adaptor related protein complex 4 accessory protein(TEPSIN)	Homo sapiens
1926	11729443_a_at	turquoise	promyelocytic leukemia(PML)	Homo sapiens
1927	11729449_s_at	turquoise	TERF1 interacting nuclear factor 2(TINF2)	Homo sapiens
1928	11729458_a_at	turquoise	Fanconi anemia complementation group I(FANCI)	Homo sapiens
1929	11729479_a_at	turquoise	C-type lectin domain family 12 member A(CLEC12A)	Homo sapiens
1930	11729481_s_at	turquoise	guanylate kinase 1(GUK1)	Homo sapiens
1931	11729485_a_at	turquoise	regulator of G-protein signaling 6(RGS6)	Homo sapiens
1932	11729487_at	turquoise	regulator of G-protein signaling 6(RGS6)	Homo sapiens
1933	11729520_at	turquoise	chromosome X open reading frame 65(CXorf65)	Homo sapiens
1934	11729523_a_at	turquoise	NLR family CARD domain containing 5(NLRC5)	Homo sapiens
1935	11729542_a_at	turquoise	calcium/calmodulin dependent protein kinase kinase 2(CAMKK2)	Homo sapiens
1936	11729570_x_at	turquoise	mitogen-activated protein kinase kinase kinase kinase 1(MAP4K1)	Homo sapiens
1937	11729574_at	turquoise	ectodermal-neural cortex 1(ENC1)	Homo sapiens
1938	11729603_a_at	turquoise	minichromosome maintenance complex component 7(MCM7)	Homo sapiens
1939	11729610_a_at	turquoise	engulfment and cell motility 1(ELMO1)	Homo sapiens
1940	11729611_a_at	turquoise	actin related protein 2/3 complex subunit 4(ARPC4)	Homo sapiens
1941	11729619_at	turquoise	chloride voltage-gated channel 4(CLCN4)	Homo sapiens
1942	11729620_at	turquoise	chloride voltage-gated channel 4(CLCN4)	Homo sapiens
1944	11729622_at	turquoise	chloride voltage-gated channel 4(CLCN4)	Homo sapiens
1945	11729649_at	turquoise	perforin 1(PRF1)	Homo sapiens
1946	11729657_a_at	turquoise	SP110 nuclear body protein(SP110)	Homo sapiens
1947	11729658_a_at	turquoise	SP110 nuclear body protein(SP110)	Homo sapiens
1949	11729667_a_at	turquoise	nucleolar protein 3(NOL3)	Homo sapiens
1951	11729676_s_at	turquoise	cyclin dependent kinase 17(CDK17)	Homo sapiens
1952	11729687_at	turquoise	LYR motif containing 7(LYRM7)	Homo sapiens
1953	11729689_s_at	turquoise	LYR motif containing 7(LYRM7)	Homo sapiens
1954	11729721_s_at	turquoise	leukocyte immunoglobulin like receptor B3(LILRB3)	Homo sapiens
1955	11729741_x_at	turquoise	CD68 molecule(CD68)	Homo sapiens
1956	11729747_a_at	turquoise	interleukin 10 receptor subunit alpha(IL10RA)	Homo sapiens
1957	11729752_a_at	turquoise	epidermal growth factor receptor pathway substrate 15 like 1(EPS15L1)	Homo sapiens
1958	11729754_at	turquoise	unc-51 like autophagy activating kinase 2(ULK2)	Homo sapiens
1959	11729758_at	turquoise	regulator of G-protein signaling 18(RGS18)	Homo sapiens
1960	11729759_at	turquoise	regulator of G-protein signaling 18(RGS18)	Homo sapiens
1961	11729769_a_at	turquoise	CD38 molecule(CD38)	Homo sapiens
1963	11729821_at	turquoise	interleukin 27 receptor subunit alpha(IL27RA)	Homo sapiens
1964	11729851_a_at	turquoise	Rap associating with DIL domain(RADIL)	Homo sapiens
1965	11729855_at	turquoise	CD3e molecule(CD3E)	Homo sapiens
1966	11729886_at	turquoise	chromosome 18 open reading frame 54(C18orf54)	Homo sapiens
1967	11729889_at	turquoise	ZFP3 zinc finger protein(ZFP3)	Homo sapiens
1968	11729924_at	turquoise	tet methylcytosine dioxygenase 3(TET3)	Homo sapiens
1969	11729954_a_at	turquoise	fragile histidine triad(FHIT)	Homo sapiens
1970	11729955_x_at	turquoise	fragile histidine triad(FHIT)	Homo sapiens
1971	11729976_a_at	turquoise	protein tyrosine phosphatase, receptor type N2(PTPRN2)	Homo sapiens
1972	11729977_a_at	turquoise	C-X-C motif chemokine receptor 6(CXCR6)	Homo sapiens
1973	11729980_a_at	turquoise	phosphatidylinositol specific phospholipase C X domain containing 3(PLCXD3)	Homo sapiens
1974	11729981_s_at	turquoise	poly(ADP-ribose) polymerase family member 11(PARP11)	Homo sapiens
1975	11729996_a_at	turquoise	schlafen family member 12(SLFN12)	Homo sapiens
1976	11730005_a_at	turquoise	semaphorin 4D(SEMA4D)	Homo sapiens
1979	11730060_a_at	turquoise	lymphocyte activating 3(LAG3)	Homo sapiens
1980	11730067_at	turquoise	granzyme M(GZMM)	Homo sapiens
1981	11730090_a_at	turquoise	TNFSF12-TNFSF13 readthrough(TNFSF12-TNFSF13)	Homo sapiens
1982	11730096_a_at	turquoise	BCL2 related protein A1(BCL2A1)	Homo sapiens
1983	11730099_a_at	turquoise	fms related tyrosine kinase 3 ligand(FLT3LG)	Homo sapiens
1984	11730100_x_at	turquoise	fms related tyrosine kinase 3 ligand(FLT3LG)	Homo sapiens
1985	11730109_a_at	turquoise	NLR family CARD domain containing 3(NLRC3)	Homo sapiens

1986	11730145_s_at	turquoise	kelch like family member 31(KLHL31)	Homo sapiens
1988	11730148_a_at	turquoise	adhesion G protein-coupled receptor G5(ADGRG5)	Homo sapiens
1989	11730155_a_at	turquoise	sarcoglycan delta(SGCD)	Homo sapiens
1991	11730168_s_at	turquoise	lipopolysaccharide induced TNF factor(LITAF)	Homo sapiens
1993	11730190_a_at	turquoise	Obg like ATPase 1(OLA1)	Homo sapiens
1994	11730191_a_at	turquoise	SEC14 like lipid binding 2(SEC14L2)	Homo sapiens
1995	11730207_at	turquoise	ArfGAP with RhoGAP domain, ankyrin repeat and PH domain 2(ARAP2)	Homo sapiens
1998	11730217_a_at	turquoise	ceramide synthase 5(CERS5)	Homo sapiens
1999	11730218_s_at	turquoise	ceramide synthase 5(CERS5)	Homo sapiens
2000	11730231_s_at	turquoise	TIA1 cytotoxic granule associated RNA binding protein like 1(TIAL1)	Homo sapiens
2001	11730237_at	turquoise	abhydrolase domain containing 10(ABHD10)	Homo sapiens
2002	11730244_at	turquoise	flavin containing monooxygenase 2(FMO2)	Homo sapiens
2003	11730247_a_at	turquoise	stromal antigen 3(STAG3)	Homo sapiens
2004	11730250_a_at	turquoise	ligand of numb-protein X 1(LNX1)	Homo sapiens
2005	11730251_x_at	turquoise	ligand of numb-protein X 1(LNX1)	Homo sapiens
2006	11730254_at	turquoise	ELK3, ETS transcription factor(ELK3)	Homo sapiens
2007	11730259_a_at	turquoise	zinc finger C2HC-type containing 1C(ZC2HC1C)	Homo sapiens
2008	11730269_at	turquoise	NIPBL, cohesin loading factor(NIPBL)	Homo sapiens
2009	11730274_at	turquoise	calcitonin receptor like receptor(CALCRL)	Homo sapiens
2010	11730296_a_at	turquoise	toll like receptor 3(TLR3)	Homo sapiens
2011	11730301_a_at	turquoise	lipoic acid synthetase(LIAS)	Homo sapiens
2012	11730322_a_at	turquoise	C-type lectin domain family 2 member D(CLEC2D)	Homo sapiens
2013	11730342_x_at	turquoise	Src like adaptor 2(SLA2)	Homo sapiens
2014	11730372_a_at	turquoise	FYN binding protein(FYB)	Homo sapiens
2015	11730373_a_at	turquoise	FYN binding protein(FYB)	Homo sapiens
2016	11730374_a_at	turquoise	FYN binding protein(FYB)	Homo sapiens
2017	11730399_at	turquoise	AT-rich interaction domain 3A(ARID3A)	Homo sapiens
2018	11730403_a_at	turquoise	MRE11 homolog, double strand break repair nuclease(MRE11)	Homo sapiens
2019	11730424_at	turquoise	APC down-regulated 1 like(APCDD1L)	Homo sapiens
2020	11730428_a_at	turquoise	adaptor related protein complex 1 beta 1 subunit(AP1B1)	Homo sapiens
2022	11730457_a_at	turquoise	absent in melanoma 2(AIM2)	Homo sapiens
2023	11730458_at	turquoise	absent in melanoma 2(AIM2)	Homo sapiens
2024	11730469_s_at	turquoise	LIM domain only 2(LMO2)	Homo sapiens
2025	11730478_at	turquoise	ring finger protein 125(RNF125)	Homo sapiens
2027	11730555_a_at	turquoise	TRIM6-TRIM34 readthrough(TRIM6-TRIM34)	Homo sapiens
2028	11730556_s_at	turquoise	TRIM6-TRIM34 readthrough(TRIM6-TRIM34)	Homo sapiens
2029	11730600_s_at	turquoise	putative aquaporin-7-like protein 3(LOC100509620)	Homo sapiens
2030	11730611_at	turquoise	mucolipin 2(MCOLN2)	Homo sapiens
2031	11730637_a_at	turquoise	cytotoxic T-lymphocyte associated protein 4(CTLA4)	Homo sapiens
2033	11730690_a_at	turquoise	family with sequence similarity 111 member A(FAM111A)	Homo sapiens
2034	11730729_a_at	turquoise	transporter 1, ATP binding cassette subfamily B member(TAP1)	Homo sapiens
2035	11730752_s_at	turquoise	1-acylglycerol-3-phosphate O-acyltransferase 5(AGPAT5)	Homo sapiens
2036	11730754_s_at	turquoise	1-acylglycerol-3-phosphate O-acyltransferase 5(AGPAT5)	Homo sapiens
2037	11730779_at	turquoise	alcohol dehydrogenase 1C (class I), gamma polypeptide(ADH1C)	Homo sapiens
2038	11730780_x_at	turquoise	alcohol dehydrogenase 1C (class I), gamma polypeptide(ADH1C)	Homo sapiens
2039	11730782_x_at	turquoise	solute carrier family 2 member 6(SLC2A6)	Homo sapiens
2041	11730795_at	turquoise	mitochondrial ribosomal protein L4(MRPL4)	Homo sapiens
2042	11730804_s_at	turquoise	pre-mRNA processing factor 38B(PRPF38B)	Homo sapiens
2043	11730806_at	turquoise	butyrophilin subfamily 3 member A2(BTN3A2)	Homo sapiens
2044	11730807_x_at	turquoise	butyrophilin subfamily 3 member A2(BTN3A2)	Homo sapiens
2045	11730813_at	turquoise	leukocyte immunoglobulin like receptor B4(LILRB4)	Homo sapiens
2046	11730815_x_at	turquoise	leukocyte immunoglobulin like receptor B4(LILRB4)	Homo sapiens
2047	11730817_a_at	turquoise	CTD small phosphatase like 2(CTDSPL2)	Homo sapiens
2049	11730837_x_at	turquoise	WD repeat domain 55(WDR55)	Homo sapiens
2050	11730862_at	turquoise	CTTNBP2 N-terminal like(CTTNBP2NL)	Homo sapiens
2051	11730864_s_at	turquoise	CTTNBP2 N-terminal like(CTTNBP2NL)	Homo sapiens
2052	11730873_a_at	turquoise	Ras association domain family member 5(RASSF5)	Homo sapiens
2053	11730875_a_at	turquoise	Fanconi anemia complementation group I(FANCI)	Homo sapiens
2054	11730894_a_at	turquoise	ubiquitin like modifier activating enzyme 6(UBA6)	Homo sapiens
2055	11730909_s_at	turquoise	C-C motif chemokine receptor 5 (gene/pseudogene)(CCR5)	Homo sapiens
2056	11730914_a_at	turquoise	ER lipid raft associated 1(ERLIN1)	Homo sapiens
2057	11730923_x_at	turquoise	ADAM metallopeptidase with thrombospondin type 1 motif 4(ADAMTS4)	Homo sapiens
2058	11730928_a_at	turquoise	inhibitor of kappa light polypeptide gene enhancer in B-cells, kinase beta(IKBKB)	Homo sapiens
2059	11730929_s_at	turquoise	inhibitor of kappa light polypeptide gene enhancer in B-cells, kinase beta(IKBKB)	Homo sapiens
2060	11730930_a_at	turquoise	inhibitor of kappa light polypeptide gene enhancer in B-cells, kinase beta(IKBKB)	Homo sapiens
2061	11730931_at	turquoise	acyl-CoA synthetase short-chain family member 3(ACSS3)	Homo sapiens
2062	11730932_at	turquoise	acyl-CoA synthetase short-chain family member 3(ACSS3)	Homo sapiens
2063	11730935_a_at	turquoise	AT-hook transcription factor(AKNA)	Homo sapiens
2064	11730936_s_at	turquoise	AT-hook transcription factor(AKNA)	Homo sapiens
2065	11730943_x_at	turquoise	osteoclast associated, immunoglobulin-like receptor(OSCAR)	Homo sapiens
2066	11730947_at	turquoise	interferon gamma(IFNG)	Homo sapiens
2068	11730962_a_at	turquoise	choline/ethanolamine phosphotransferase 1(CEPT1)	Homo sapiens
2069	11730966_at	turquoise	mitogen-activated protein kinase 11(MAPK11)	Homo sapiens
2070	11730968_at	turquoise	RIC8 guanine nucleotide exchange factor B(RIC8B)	Homo sapiens
2071	11730994_at	turquoise	sphingosine-1-phosphate receptor 4(S1PR4)	Homo sapiens
2072	11730996_a_at	turquoise	junction adhesion molecule like(JAML)	Homo sapiens
2073	11731004_at	turquoise	MIS18 binding protein 1(MIS18BP1)	Homo sapiens
2074	11731005_at	turquoise	MIS18 binding protein 1(MIS18BP1)	Homo sapiens
2075	11731006_s_at	turquoise	MIS18 binding protein 1(MIS18BP1)	Homo sapiens
2076	11731019_x_at	turquoise	allograft inflammatory factor 1(AIF1)	Homo sapiens
2077	11731023_a_at	turquoise	ataxin 7(ATXN7)	Homo sapiens
2078	11731024_s_at	turquoise	ataxin 7(ATXN7)	Homo sapiens

2079	11731036_a_at	turquoise	fibrinogen C domain containing 1(FIBCD1)	Homo sapiens
2080	11731039_s_at	turquoise	interleukin 15 receptor subunit alpha(IL15RA)	Homo sapiens
2081	11731066_x_at	turquoise	GTPase, IMAP family member 1(GIMAP1)	Homo sapiens
2082	11731110_at	turquoise	ADAM metallopeptidase domain 17(ADAM17)	Homo sapiens
2083	11731147_at	turquoise	toll like receptor 7(TLR7)	Homo sapiens
2084	11731148_at	turquoise	toll like receptor 7(TLR7)	Homo sapiens
2085	11731181_a_at	turquoise	epithelial stromal interaction 1(EPSTI1)	Homo sapiens
2086	11731196_a_at	turquoise	proline rich 5 like(PRR5L)	Homo sapiens
2087	11731197_a_at	turquoise	proline rich 5 like(PRR5L)	Homo sapiens
2088	11731200_a_at	turquoise	toll like receptor 5(TLR5)	Homo sapiens
2089	11731209_s_at	turquoise	chromosome 15 open reading frame 59(C15orf59)	Homo sapiens
2090	11731227_a_at	turquoise	megakaryocyte-associated tyrosine kinase(MATK)	Homo sapiens
2091	11731230_a_at	turquoise	RNA pseudouridylate synthase domain containing 3(RPUSD3)	Homo sapiens
2093	11731281_a_at	turquoise	nuclear factor of activated T-cells 1(NFATC1)	Homo sapiens
2094	11731309_at	turquoise	chromosome 15 open reading frame 56(C15orf56)	Homo sapiens
2095	11731340_at	turquoise	nucleotide binding oligomerization domain containing 2(NOD2)	Homo sapiens
2096	11731346_a_at	turquoise	diacylglycerol kinase zeta(DGKZ)	Homo sapiens
2097	11731356_a_at	turquoise	inositol hexakisphosphate kinase 3(IP6K3)	Homo sapiens
2098	11731360_a_at	turquoise	inducible T-cell costimulator(ICOS)	Homo sapiens
2099	11731394_a_at	turquoise	vimentin(VIM)	Homo sapiens
2100	11731407_x_at	turquoise	interferon induced protein with tetratricopeptide repeats 3(IFIT3)	Homo sapiens
2102	11731422_s_at	turquoise	Fc fragment of IgG receptor IIIa(FCGR3A)	Homo sapiens
2103	11731433_a_at	turquoise	selenoprotein P(SELENOP)	Homo sapiens
2104	11731465_a_at	turquoise	cathepsin C(CTSC)	Homo sapiens
2105	11731466_a_at	turquoise	cathepsin C(CTSC)	Homo sapiens
2106	11731485_x_at	turquoise	nuclear factor I C(NFIC)	Homo sapiens
2107	11731504_a_at	turquoise	chromosome 22 open reading frame 39(C22orf39)	Homo sapiens
2108	11731505_a_at	turquoise	chromosome 22 open reading frame 39(C22orf39)	Homo sapiens
2109	11731518_at	turquoise	microfibrillar associated protein 3(MFAP3)	Homo sapiens
2110	11731531_x_at	turquoise	docking protein 1(DOK1)	Homo sapiens
2111	11731555_at	turquoise	synaptopodin 2(SYNPO2)	Homo sapiens
2112	11731556_a_at	turquoise	synaptopodin 2(SYNPO2)	Homo sapiens
2114	11731575_a_at	turquoise	chitobiase(CTBS)	Homo sapiens
2115	11731613_a_at	turquoise	Ras homolog enriched in brain like 1(RHEBL1)	Homo sapiens
2117	11731617_x_at	turquoise	SID1 transmembrane family member 1(SIDT1)	Homo sapiens
2118	11731620_a_at	turquoise	arrestin beta 2(ARRB2)	Homo sapiens
2119	11731641_s_at	turquoise	trafficking protein particle complex subunit 10-like(LOC102724200)	Homo sapiens
2120	11731644_s_at	turquoise	trafficking protein particle complex subunit 10-like(LOC102724200)	Homo sapiens
2121	11731657_s_at	turquoise	vav guanine nucleotide exchange factor 3(VAV3)	Homo sapiens
2122	11731667_s_at	turquoise	platelet derived growth factor subunit B(PDGFB)	Homo sapiens
2123	11731669_at	turquoise	G protein-coupled receptor 65(GPR65)	Homo sapiens
2125	11731676_s_at	turquoise	C-C motif chemokine receptor 2(CCR2)	Homo sapiens
2126	11731702_a_at	turquoise	C-type lectin domain family 2 member D(CLEC2D)	Homo sapiens
2127	11731722_a_at	turquoise	aldehyde dehydrogenase 3 family member B1(ALDH3B1)	Homo sapiens
2128	11731728_at	turquoise	purinergic receptor P2Y13(P2RY13)	Homo sapiens
2129	11731729_at	turquoise	purinergic receptor P2Y13(P2RY13)	Homo sapiens
2130	11731755_a_at	turquoise	synaptotagmin like 2(SYTL2)	Homo sapiens
2131	11731764_a_at	turquoise	CD3g molecule(CD3G)	Homo sapiens
2132	11731770_a_at	turquoise	dermatan sulfate epimerase(DSE)	Homo sapiens
2133	11731777_a_at	turquoise	protein O-linked mannose N-acetylglucosaminyltransferase 2 (beta 1,4-)(POMGNT2)	Homo sapiens
2134	11731783_a_at	turquoise	RAS protein activator like 3(RASAL3)	Homo sapiens
2135	11731798_a_at	turquoise	galectin 2(LGALS2)	Homo sapiens
2136	11731799_at	turquoise	urocortin 3(UCN3)	Homo sapiens
2137	11731818_a_at	turquoise	CD40 molecule(CD40)	Homo sapiens
2138	11731820_at	turquoise	ADAM metallopeptidase domain 11(ADAM11)	Homo sapiens
2139	11731821_at	turquoise	ADAM metallopeptidase domain 11(ADAM11)	Homo sapiens
2140	11731845_a_at	turquoise	immunoglobulin superfamily member 3(IGSF3)	Homo sapiens
2141	11731848_s_at	turquoise	killer cell lectin like receptor C1(KLRC1)	Homo sapiens
2142	11731873_a_at	turquoise	CD209 molecule(CD209)	Homo sapiens
2143	11731879_a_at	turquoise	NLR family pyrin domain containing 1(NLRP1)	Homo sapiens
2144	11731880_at	turquoise	NLR family pyrin domain containing 1(NLRP1)	Homo sapiens
2145	11731914_at	turquoise	adhesion molecule with Ig like domain 1(AMIGO1)	Homo sapiens
2147	11731942_a_at	turquoise	PR/SET domain 1(PRDM1)	Homo sapiens
2148	11731949_a_at	turquoise	sterile alpha motif domain containing 3(SAMD3)	Homo sapiens
2149	11731950_x_at	turquoise	sterile alpha motif domain containing 3(SAMD3)	Homo sapiens
2150	11731978_s_at	turquoise	proprotein convertase subtilisin/kexin type 7(PCSK7)	Homo sapiens
2151	11731998_s_at	turquoise	coiled-coil domain containing 88C(CCDC88C)	Homo sapiens
2152	11732004_a_at	turquoise	adenosine deaminase like(ADAL)	Homo sapiens
2153	11732006_s_at	turquoise	adenosine deaminase like(ADAL)	Homo sapiens
2154	11732017_a_at	turquoise	interleukin 18 receptor accessory protein(IL18RAP)	Homo sapiens
2155	11732058_a_at	turquoise	cell division cycle 7(CDC7)	Homo sapiens
2156	11732084_a_at	turquoise	transcription factor EC(TFEC)	Homo sapiens
2157	11732110_a_at	turquoise	interleukin 4 induced 1(IL4I1)	Homo sapiens
2158	11732153_a_at	turquoise	zinc finger DHHC-type containing 6(ZDHHC6)	Homo sapiens
2159	11732155_a_at	turquoise	O-linked N-acetylglucosamine (GlcNAc) transferase(OGT)	Homo sapiens
2160	11732169_a_at	turquoise	protein tyrosine phosphatase, non-receptor type 7(PTPN7)	Homo sapiens
2161	11732171_x_at	turquoise	G-rich RNA sequence binding factor 1(GRSF1)	Homo sapiens
2162	11732180_a_at	turquoise	platelet activating factor receptor(PTAFR)	Homo sapiens
2163	11732181_x_at	turquoise	platelet activating factor receptor(PTAFR)	Homo sapiens
2165	11732218_x_at	turquoise	BH3 interacting domain death agonist(BID)	Homo sapiens
2166	11732220_a_at	turquoise	enoyl-CoA delta isomerase 1(ECI1)	Homo sapiens
2167	11732232_x_at	turquoise	ORAI calcium release-activated calcium modulator 2(ORAI2)	Homo sapiens

2168	11732243_at	turquoise	apolipoprotein L6(APOL6)	Homo sapiens
2169	11732244_at	turquoise	apolipoprotein L6(APOL6)	Homo sapiens
2170	11732247_s_at	turquoise	heterogeneous nuclear ribonucleoprotein C (C1/C2)(HNRNPC)	Homo sapiens
2171	11732265_at	turquoise	leukocyte immunoglobulin like receptor B1(LILRB1)	Homo sapiens
2172	11732266_x_at	turquoise	leukocyte immunoglobulin like receptor B1(LILRB1)	Homo sapiens
2173	11732273_at	turquoise	cytochrome c oxidase assembly factor 7 (putative)(COA7)	Homo sapiens
2174	11732275_at	turquoise	C-C motif chemokine ligand 5(CCL5)	Homo sapiens
2175	11732276_x_at	turquoise	C-C motif chemokine ligand 5(CCL5)	Homo sapiens
2176	11732278_at	turquoise	forkhead box P1(FOXP1)	Homo sapiens
2177	11732315_a_at	turquoise	sarcoglycan delta(SGCD)	Homo sapiens
2179	11732323_at	turquoise	frataxin(FXN)	Homo sapiens
2180	11732331_s_at	turquoise	ADP ribosylation factor like GTPase 4C(ARL4C)	Homo sapiens
2181	11732349_at	turquoise	leukocyte immunoglobulin like receptor A6(LILRA6)	Homo sapiens
2182	11732355_x_at	turquoise	major histocompatibility complex, class I, F(HLA-F)	Homo sapiens
2183	11732366_a_at	turquoise	S-phase cyclin A associated protein in the ER(SCAPER)	Homo sapiens
2184	11732369_a_at	turquoise	fibroblast growth factor 14(FGF14)	Homo sapiens
2185	11732402_a_at	turquoise	UBA domain containing 1(UBAC1)	Homo sapiens
2186	11732414_a_at	turquoise	leucine rich repeat containing 8 family member B(LRRC8B)	Homo sapiens
2187	11732418_at	turquoise	family with sequence similarity 105 member A(FAM105A)	Homo sapiens
2188	11732424_at	turquoise	ankyrin repeat domain 22(ANKRD22)	Homo sapiens
2189	11732425_at	turquoise	ankyrin repeat domain 22(ANKRD22)	Homo sapiens
2190	11732435_at	turquoise	SLP adaptor and CSK interacting membrane protein(SCIMP)	Homo sapiens
2191	11732450_s_at	turquoise	agrin(AGRN)	Homo sapiens
2192	11732456_a_at	turquoise	membrane associated ring-CH-type finger 1(MARCH1)	Homo sapiens
2193	11732466_a_at	turquoise	C-X-C motif chemokine ligand 11(CXCL11)	Homo sapiens
2194	11732467_x_at	turquoise	C-X-C motif chemokine ligand 11(CXCL11)	Homo sapiens
2195	11732469_at	turquoise	interleukin 3 receptor subunit alpha(IL3RA)	Homo sapiens
2196	11732479_a_at	turquoise	integrin subunit alpha M(ITGAM)	Homo sapiens
2197	11732480_s_at	turquoise	integrin subunit alpha M(ITGAM)	Homo sapiens
2198	11732481_a_at	turquoise	integrin subunit alpha M(ITGAM)	Homo sapiens
2199	11732492_at	turquoise	KIAA1549(KIAA1549)	Homo sapiens
2201	11732514_s_at	turquoise	LYN proto-oncogene, Src family tyrosine kinase(LYN)	Homo sapiens
2202	11732519_at	turquoise	cholesterol 25-hydroxylase(CH25H)	Homo sapiens
2205	11732526_s_at	turquoise	chondroitin sulfate N-acetylgalactosaminyltransferase 1(CSGALNACT1)	Homo sapiens
2206	11732528_s_at	turquoise	T-box 5(TBX5)	Homo sapiens
2207	11732529_a_at	turquoise	T-box 5(TBX5)	Homo sapiens
2208	11732531_s_at	turquoise	NEDD4 binding protein 2 like 1(N4BP2L1)	Homo sapiens
2209	11732533_a_at	turquoise	lamin B1(LMNB1)	Homo sapiens
2210	11732538_at	turquoise	T-box 21(TBX21)	Homo sapiens
2211	11732544_a_at	turquoise	G protein-coupled receptor 18(GPR18)	Homo sapiens
2212	11732550_at	turquoise	prostaglandin E receptor 2(PTGER2)	Homo sapiens
2213	11732555_at	turquoise	Fas ligand(FASLG)	Homo sapiens
2214	11732580_a_at	turquoise	malic enzyme 3(ME3)	Homo sapiens
2217	11732589_a_at	turquoise	zinc finger protein 467(ZNF467)	Homo sapiens
2218	11732601_at	turquoise	centrosomal protein 135(CEP135)	Homo sapiens
2219	11732602_at	turquoise	centrosomal protein 135(CEP135)	Homo sapiens
2220	11732650_a_at	turquoise	hexosaminidase D(HEXDC)	Homo sapiens
2221	11732661_x_at	turquoise	SCL/TAL1 interrupting locus(STIL)	Homo sapiens
2222	11732701_a_at	turquoise	chondroitin sulfate N-acetylgalactosaminyltransferase 2(CSGALNACT2)	Homo sapiens
2223	11732709_x_at	turquoise	sarcoglycan alpha(SGCA)	Homo sapiens
2224	11732759_at	turquoise	GRB2 binding adaptor protein, transmembrane(GAPT)	Homo sapiens
2225	11732798_a_at	turquoise	mirror-image polydactyly 1(MIPOL1)	Homo sapiens
2226	11732813_at	turquoise	dendritic cell associated nuclear protein(DCANP1)	Homo sapiens
2227	11732827_a_at	turquoise	renalase, FAD dependent amine oxidase(RNLS)	Homo sapiens
2228	11732854_x_at	turquoise	scavenger receptor class F member 1(SCARF1)	Homo sapiens
2230	11732870_a_at	turquoise	caspase 1(CASP1)	Homo sapiens
2231	11732873_x_at	turquoise	mitochondrial translation release factor 1(MTRF1)	Homo sapiens
2232	11732894_x_at	turquoise	cyclin dependent kinase like 3(CDKL3)	Homo sapiens
2233	11732896_a_at	turquoise	hydroxysteroid 11-beta dehydrogenase 1 like(HSD11B1L)	Homo sapiens
2234	11732901_a_at	turquoise	APOBEC3A and APOBEC3B deletion hybrid(APOBEC3A_B)	Homo sapiens
2235	11732902_x_at	turquoise	apolipoprotein B mRNA editing enzyme catalytic subunit 3B(APOBEC3B)	Homo sapiens
2236	11732913_a_at	turquoise	SP140 nuclear body protein(SP140)	Homo sapiens
2237	11732927_x_at	turquoise	killer cell lectin like receptor C1(KLRC1)	Homo sapiens
2238	11732936_a_at	turquoise	TBC1 domain family member 2B(TBC1D2B)	Homo sapiens
2239	11732956_a_at	turquoise	TCR gamma alternate reading frame protein(TARP)	Homo sapiens
2240	11732999_a_at	turquoise	intercellular adhesion molecule 1(ICAM1)	Homo sapiens
2241	11733000_at	turquoise	intercellular adhesion molecule 1(ICAM1)	Homo sapiens
2242	11733004_s_at	turquoise	Fc fragment of IgG receptor IIIa(FCGR3A)	Homo sapiens
2243	11733006_at	turquoise	angiomotin like 2(AMOTL2)	Homo sapiens
2244	11733014_a_at	turquoise	ring finger protein 213(RNF213)	Homo sapiens
2245	11733021_x_at	turquoise	lactate dehydrogenase A(LDHA)	Homo sapiens
2246	11733023_s_at	turquoise	BTG anti-proliferation factor 1(BTG1)	Homo sapiens
2247	11733024_x_at	turquoise	BTG anti-proliferation factor 1(BTG1)	Homo sapiens
2248	11733030_s_at	turquoise	axin interactor, dorsalization associated(AIDA)	Homo sapiens
2250	11733033_a_at	turquoise	family with sequence similarity 32 member A(FAM32A)	Homo sapiens
2251	11733046_x_at	turquoise	abl interactor 1(ABI1)	Homo sapiens
2252	11733047_a_at	turquoise	abl interactor 1(ABI1)	Homo sapiens
2253	11733052_s_at	turquoise	secretory carrier membrane protein 1(SCAMP1)	Homo sapiens
2254	11733056_s_at	turquoise	polypyrimidine tract binding protein 3(PTBP3)	Homo sapiens
2255	11733057_a_at	turquoise	polypyrimidine tract binding protein 3(PTBP3)	Homo sapiens
2256	11733058_a_at	turquoise	polypyrimidine tract binding protein 3(PTBP3)	Homo sapiens
2257	11733060_a_at	turquoise	pellino E3 ubiquitin protein ligase 1(PELI1)	Homo sapiens

2258	11733061_s_at	turquoise	pellino E3 ubiquitin protein ligase 1(PELI1)	Homo sapiens
2259	11733087_a_at	turquoise	MAF bZIP transcription factor(MAF)	Homo sapiens
2260	11733088_at	turquoise	MAF bZIP transcription factor(MAF)	Homo sapiens
2261	11733127_at	turquoise	solute carrier family 25 member 26(SLC25A26)	Homo sapiens
2263	11733140_s_at	turquoise	ADP ribosylation factor like GTPase 4A(ARL4A)	Homo sapiens
2264	11733148_a_at	turquoise	annexin A4(ANXA4)	Homo sapiens
2265	11733149_a_at	turquoise	DExD/H-box helicase 58(DDX58)	Homo sapiens
2266	11733161_a_at	turquoise	TNF alpha induced protein 8(TNFAIP8)	Homo sapiens
2267	11733162_a_at	turquoise	proline-serine-threonine phosphatase interacting protein 1(PSTPIP1)	Homo sapiens
2268	11733187_a_at	turquoise	interleukin 7 receptor(IL7R)	Homo sapiens
2269	11733194_s_at	turquoise	formin like 3(FMNL3)	Homo sapiens
2270	11733198_a_at	turquoise	DExD-box helicase 39A(DDX39A)	Homo sapiens
2271	11733213_x_at	turquoise	schlafen family member 5(SLFN5)	Homo sapiens
2272	11733214_a_at	turquoise	schlafen family member 5(SLFN5)	Homo sapiens
2273	11733215_a_at	turquoise	signal transducer and activator of transcription 4(STAT4)	Homo sapiens
2274	11733246_at	turquoise	dystrobrevin binding protein 1(DTNBP1)	Homo sapiens
2276	11733264_a_at	turquoise	echinoderm microtubule associated protein like 1(EML1)	Homo sapiens
2277	11733334_a_at	turquoise	neuropilin 2(NRP2)	Homo sapiens
2278	11733343_a_at	turquoise	UTP6, small subunit processome component(UTP6)	Homo sapiens
2279	11733353_at	turquoise	cytotoxic and regulatory T-cell molecule(CRTAM)	Homo sapiens
2280	11733355_x_at	turquoise	complement C5a receptor 1(C5AR1)	Homo sapiens
2281	11733360_x_at	turquoise	solute carrier family 14 member 1 (Kidd blood group)(SLC14A1)	Homo sapiens
2282	11733370_a_at	turquoise	proteasome activator subunit 1(PSME1)	Homo sapiens
2283	11733389_a_at	turquoise	DEF6, guanine nucleotide exchange factor(DEF6)	Homo sapiens
2284	11733402_a_at	turquoise	C-type lectin domain family 7 member A(CLEC7A)	Homo sapiens
2285	11733403_x_at	turquoise	C-type lectin domain family 7 member A(CLEC7A)	Homo sapiens
2286	11733411_at	turquoise	transcription factor 15 (basic helix-loop-helix)(TCF15)	Homo sapiens
2287	11733439_a_at	turquoise	guanylate binding protein 5(GBP5)	Homo sapiens
2288	11733450_a_at	turquoise	schlafen family member 11(SLFN11)	Homo sapiens
2289	11733477_at	turquoise	succinate receptor 1(SUCNR1)	Homo sapiens
2290	11733485_a_at	turquoise	calcium voltage-gated channel subunit alpha1 C(CACNA1C)	Homo sapiens
2291	11733494_a_at	turquoise	NEDD4 binding protein 2 like 2(N4BP2L2)	Homo sapiens
2292	11733511_a_at	turquoise	myotubularin related protein 1(MTMR1)	Homo sapiens
2293	11733512_s_at	turquoise	myotubularin related protein 1(MTMR1)	Homo sapiens
2295	11733525_at	turquoise	chromosome 2 open reading frame 71(C2orf71)	Homo sapiens
2296	11733530_at	turquoise	complement C1r subcomponent like(C1RL)	Homo sapiens
2299	11733579_a_at	turquoise	chromosome 19 open reading frame 47(C19orf47)	Homo sapiens
2300	11733611_a_at	turquoise	dedicator of cytokinesis 8(DOCK8)	Homo sapiens
2301	11733612_s_at	turquoise	dedicator of cytokinesis 8(DOCK8)	Homo sapiens
2302	11733616_at	turquoise	histamine N-methyltransferase(HNMT)	Homo sapiens
2303	11733620_a_at	turquoise	B-cell CLL/lymphoma 11B(BCL11B)	Homo sapiens
2304	11733632_s_at	turquoise	purinergic receptor P2Y14(P2RY14)	Homo sapiens
2305	11733641_a_at	turquoise	kinesin light chain 4(KLC4)	Homo sapiens
2306	11733642_s_at	turquoise	NECAP endocytosis associated 2(NECAP2)	Homo sapiens
2307	11733643_x_at	turquoise	NECAP endocytosis associated 2(NECAP2)	Homo sapiens
2308	11733644_s_at	turquoise	NECAP endocytosis associated 2(NECAP2)	Homo sapiens
2310	11733695_a_at	turquoise	ubiquitin conjugating enzyme E2 C(UBE2C)	Homo sapiens
2311	11733696_x_at	turquoise	ubiquitin conjugating enzyme E2 C(UBE2C)	Homo sapiens
2312	11733698_s_at	turquoise	serum/glucocorticoid regulated kinase 1(SGK1)	Homo sapiens
2313	11733699_a_at	turquoise	TEA domain transcription factor 4(TEAD4)	Homo sapiens
2314	11733701_a_at	turquoise	FK506 binding protein 11(FKBP11)	Homo sapiens
2315	11733702_x_at	turquoise	ubiquitin conjugating enzyme E2 C(UBE2C)	Homo sapiens
2316	11733718_x_at	turquoise	leukocyte associated immunoglobulin like receptor 2(LAIR2)	Homo sapiens
2317	11733725_a_at	turquoise	complement factor B(CFB)	Homo sapiens
2318	11733736_a_at	turquoise	CD2 molecule(CD2)	Homo sapiens
2319	11733754_a_at	turquoise	pleckstrin homology domain containing A4(PLEKHA4)	Homo sapiens
2320	11733767_a_at	turquoise	leukocyte immunoglobulin like receptor A2(LILRA2)	Homo sapiens
2321	11733768_x_at	turquoise	leukocyte immunoglobulin like receptor A2(LILRA2)	Homo sapiens
2322	11733784_at	turquoise	protein phosphatase 2 scaffold subunit Abeta(PPP2R1B)	Homo sapiens
2324	11733809_s_at	turquoise	ARMCX5-GPRASP2 readthrough(ARMCX5-GPRASP2)	Homo sapiens
2325	11733841_a_at	turquoise	ecotropic viral integration site 2A(EVI2A)	Homo sapiens
2326	11733845_at	turquoise	G protein-coupled receptor kinase 6(GRK6)	Homo sapiens
2327	11733846_a_at	turquoise	G protein-coupled receptor kinase 6(GRK6)	Homo sapiens
2328	11733855_a_at	turquoise	apolipoprotein L1(APOL1)	Homo sapiens
2330	11733867_a_at	turquoise	major histocompatibility complex, class II, DO alpha(HLA-DOA)	Homo sapiens
2331	11733868_at	turquoise	major histocompatibility complex, class II, DO alpha(HLA-DOA)	Homo sapiens
2332	11733869_a_at	turquoise	major histocompatibility complex, class II, DO alpha(HLA-DOA)	Homo sapiens
2333	11733870_at	turquoise	major histocompatibility complex, class II, DO alpha(HLA-DOA)	Homo sapiens
2334	11733878_a_at	turquoise	LIM domain 7(LMO7)	Homo sapiens
2335	11733894_x_at	turquoise	transcriptional adaptor 2B(TADA2B)	Homo sapiens
2337	11733934_a_at	turquoise	primary cilia formation(PIFO)	Homo sapiens
2338	11733946_at	turquoise	RAP2B, member of RAS oncogene family(RAP2B)	Homo sapiens
2339	11733950_at	turquoise	guanylate binding protein 4(GBP4)	Homo sapiens
2340	11733951_at	turquoise	guanylate binding protein 4(GBP4)	Homo sapiens
2341	11733952_at	turquoise	guanylate binding protein 4(GBP4)	Homo sapiens
2342	11733953_x_at	turquoise	guanylate binding protein 4(GBP4)	Homo sapiens
2343	11733960_a_at	turquoise	USH1 protein network component sans(USH1G)	Homo sapiens
2344	11733979_at	turquoise	CD28 molecule(CD28)	Homo sapiens
2345	11733986_x_at	turquoise	tubulin alpha 3e(TUBA3E)	Homo sapiens
2346	11733992_a_at	turquoise	transmembrane protein 150C(TMEM150C)	Homo sapiens
2347	11734006_a_at	turquoise	interleukin 1 receptor associated kinase 1(IRAK1)	Homo sapiens
2349	11734035_a_at	turquoise	formyl peptide receptor 2(FPR2)	Homo sapiens

2350	11734050_a_at	turquoise	T cell receptor associated transmembrane adaptor 1(TRAT1)	Homo sapiens
2351	11734051_a_at	turquoise	T cell receptor associated transmembrane adaptor 1(TRAT1)	Homo sapiens
2352	11734056_at	turquoise	prostaglandin reductase 2(PTGR2)	Homo sapiens
2353	11734064_a_at	turquoise	uncharacterized LOC100130460(CAND1.11)	Homo sapiens
2354	11734065_a_at	turquoise	uncharacterized LOC100130460(CAND1.11)	Homo sapiens
2355	11734066_x_at	turquoise	interleukin 1 receptor associated kinase 4(IRAK4)	Homo sapiens
2356	11734067_a_at	turquoise	interleukin 1 receptor associated kinase 4(IRAK4)	Homo sapiens
2357	11734084_a_at	turquoise	sushi domain containing 3(SUSD3)	Homo sapiens
2358	11734110_at	turquoise	T-cell immunoreceptor with Ig and ITIM domains(TIGIT)	Homo sapiens
2359	11734112_at	turquoise	T-cell immunoreceptor with Ig and ITIM domains(TIGIT)	Homo sapiens
2360	11734117_a_at	turquoise	butyrophilin subfamily 3 member A3(BTN3A3)	Homo sapiens
2361	11734118_a_at	turquoise	butyrophilin subfamily 3 member A3(BTN3A3)	Homo sapiens
2362	11734126_a_at	turquoise	nucleotide binding oligomerization domain containing 1(NOD1)	Homo sapiens
2363	11734139_a_at	turquoise	TBC1 domain family member 10C(TBC1D10C)	Homo sapiens
2364	11734162_a_at	turquoise	transcription factor EC(TFEC)	Homo sapiens
2365	11734185_at	turquoise	zinc finger protein 677(ZNF677)	Homo sapiens
2366	11734259_a_at	turquoise	sterol carrier protein 2(SCP2)	Homo sapiens
2367	11734273_s_at	turquoise	G protein-coupled receptor 18(GPR18)	Homo sapiens
2368	11734359_a_at	turquoise	ribonuclease L(RNASEL)	Homo sapiens
2369	11734394_at	turquoise	killer cell lectin like receptor C3(KLRC3)	Homo sapiens
2370	11734395_x_at	turquoise	killer cell lectin like receptor C3(KLRC3)	Homo sapiens
2372	11734521_x_at	turquoise	sodium channel and clathrin linker 1(SCLT1)	Homo sapiens
2373	11734526_a_at	turquoise	uncharacterized LOC100507472(LOC100507472)	Homo sapiens
2374	11734529_x_at	turquoise	paired immunoglobin like type 2 receptor alpha(PILRA)	Homo sapiens
2375	11734530_x_at	turquoise	major histocompatibility complex, class I, F(HLA-F)	Homo sapiens
2376	11734536_a_at	turquoise	glycoprotein Ib platelet alpha subunit(GP1BA)	Homo sapiens
2377	11734548_a_at	turquoise	transforming growth factor beta induced(TGFBI)	Homo sapiens
2378	11734549_s_at	turquoise	transforming growth factor beta induced(TGFBI)	Homo sapiens
2379	11734550_x_at	turquoise	transforming growth factor beta induced(TGFBI)	Homo sapiens
2380	11734552_a_at	turquoise	C-X3-C motif chemokine ligand 1(CX3CL1)	Homo sapiens
2381	11734567_a_at	turquoise	RAS guanyl releasing protein 2(RASGRP2)	Homo sapiens
2382	11734577_a_at	turquoise	sodium voltage-gated channel alpha subunit 5(SCN5A)	Homo sapiens
2383	11734606_a_at	turquoise	C-X-C motif chemokine receptor 3(CXCR3)	Homo sapiens
2386	11734657_s_at	turquoise	solute carrier family 2 member 14(SLC2A14)	Homo sapiens
2387	11734659_a_at	turquoise	Fos proto-oncogene, AP-1 transcription factor subunit(FOS)	Homo sapiens
2388	11734661_a_at	turquoise	calsyntenin 3(CLSTN3)	Homo sapiens
2389	11734672_a_at	turquoise	nuclear factor of activated T-cells 4(NFATC4)	Homo sapiens
2390	11734684_s_at	turquoise	uridine monophosphate synthetase(UMPS)	Homo sapiens
2391	11734687_at	turquoise	methyl-CpG binding domain protein 2(MBD2)	Homo sapiens
2392	11734690_a_at	turquoise	cytohesin 1 interacting protein(CYTIP)	Homo sapiens
2393	11734695_a_at	turquoise	protein phosphatase 4 catalytic subunit(PPP4C)	Homo sapiens
2394	11734715_a_at	turquoise	DBF4 zinc finger(DBF4)	Homo sapiens
2395	11734727_a_at	turquoise	interleukin 16(IL16)	Homo sapiens
2398	11734747_a_at	turquoise	complement component 4B (Chido blood group), copy 2(C4B_2)	Homo sapiens
2399	11734772_a_at	turquoise	synergin gamma(SYNRG)	Homo sapiens
2400	11734783_at	turquoise	chloride intracellular channel 2(CLIC2)	Homo sapiens
2401	11734784_a_at	turquoise	chloride intracellular channel 2(CLIC2)	Homo sapiens
2402	11734785_at	turquoise	chloride intracellular channel 2(CLIC2)	Homo sapiens
2403	11734791_x_at	turquoise	septin 6(SEPT6)	Homo sapiens
2404	11734798_x_at	turquoise	ring finger protein, LIM domain interacting(RLIM)	Homo sapiens
2405	11734825_at	turquoise	sprouty related EVH1 domain containing 1(SPRED1)	Homo sapiens
2406	11734826_s_at	turquoise	sprouty related EVH1 domain containing 1(SPRED1)	Homo sapiens
2407	11734852_a_at	turquoise	RUN and cysteine rich domain containing beclin 1 interacting protein like(RUBCNL)	Homo sapiens
2409	11734870_a_at	turquoise	NAD kinase(NADK)	Homo sapiens
2410	11734873_a_at	turquoise	S-phase cyclin A associated protein in the ER(SCAPER)	Homo sapiens
2411	11734890_a_at	turquoise	interleukin 32(IL32)	Homo sapiens
2412	11734894_at	turquoise	CST telomere replication complex component 1(CTC1)	Homo sapiens
2413	11734921_a_at	turquoise	zinc finger protein 276(ZNF276)	Homo sapiens
2414	11734922_x_at	turquoise	zinc finger protein 276(ZNF276)	Homo sapiens
2415	11734924_a_at	turquoise	ATPase H+ transporting V1 subunit G2(ATP6V1G2)	Homo sapiens
2416	11734938_x_at	turquoise	colony stimulating factor 2 receptor alpha subunit(CSF2RA)	Homo sapiens
2417	11734943_a_at	turquoise	SLAM family member 6(SLAMF6)	Homo sapiens
2418	11734944_at	turquoise	SLAM family member 6(SLAMF6)	Homo sapiens
2419	11734948_x_at	turquoise	fibroblast growth factor 7(FGF7)	Homo sapiens
2420	11734962_x_at	turquoise	ADAM metallopeptidase domain 33(ADAM33)	Homo sapiens
2421	11734967_a_at	turquoise	thymocyte selection associated(THEMIS)	Homo sapiens
2422	11735003_a_at	turquoise	suppression of tumorigenicity 7 like(ST7L)	Homo sapiens
2423	11735026_at	turquoise	sialophorin(SPN)	Homo sapiens
2424	11735027_x_at	turquoise	sialophorin(SPN)	Homo sapiens
2425	11735039_s_at	turquoise	shootin 1(SHTN1)	Homo sapiens
2427	11735044_x_at	turquoise	tripartite motif containing 5(TRIM5)	Homo sapiens
2430	11735124_a_at	turquoise	interaction protein for cytohesin exchange factors 1(IPCEF1)	Homo sapiens
2431	11735157_at	turquoise	cysteinyl leukotriene receptor 1(CYSLTR1)	Homo sapiens
2432	11735173_a_at	turquoise	small nuclear ribonucleoprotein polypeptide N(SNRPN)	Homo sapiens
2433	11735174_a_at	turquoise	interleukin 32(IL32)	Homo sapiens
2434	11735189_s_at	turquoise	H2A histone family member Y(H2AFY)	Homo sapiens
2435	11735192_a_at	turquoise	adhesion G protein-coupled receptor E2(ADGRE2)	Homo sapiens
2436	11735204_a_at	turquoise	methylmalonic aciduria (cobalamin deficiency) cblA type(MMAA)	Homo sapiens
2437	11735205_s_at	turquoise	methylmalonic aciduria (cobalamin deficiency) cblA type(MMAA)	Homo sapiens
2438	11735220_at	turquoise	lysophosphatidic acid receptor 3(LPAR3)	Homo sapiens
2439	11735221_a_at	turquoise	B and T lymphocyte associated(BTLA)	Homo sapiens
2440	11735222_a_at	turquoise	B and T lymphocyte associated(BTLA)	Homo sapiens

2441	11735223_a_at	turquoise	killer cell lectin like receptor G1(KLRG1)	Homo sapiens
2442	11735224_a_at	turquoise	killer cell lectin like receptor G1(KLRG1)	Homo sapiens
2443	11735225_x_at	turquoise	guanidinoacetate N-methyltransferase(GAMT)	Homo sapiens
2444	11735249_x_at	turquoise	SP140 nuclear body protein like(SP140L)	Homo sapiens
2445	11735270_a_at	turquoise	glucosaminyl (N-acetyl) transferase 1, core 2(GCNT1)	Homo sapiens
2446	11735271_a_at	turquoise	glucosaminyl (N-acetyl) transferase 1, core 2(GCNT1)	Homo sapiens
2447	11735275_at	turquoise	interleukin 18 receptor 1(IL18R1)	Homo sapiens
2448	11735284_x_at	turquoise	lysine methyltransferase 2E(KMT2E)	Homo sapiens
2449	11735290_a_at	turquoise	TNFAIP3 interacting protein 2(TNIP2)	Homo sapiens
2450	11735329_a_at	turquoise	CD47 molecule(CD47)	Homo sapiens
2452	11735394_s_at	turquoise	X-C motif chemokine ligand 1(XCL1)	Homo sapiens
2453	11735416_x_at	turquoise	DNA cross-link repair 1C(DCLRE1C)	Homo sapiens
2454	11735429_x_at	turquoise	ring finger protein 135(RNF135)	Homo sapiens
2455	11735474_a_at	turquoise	CD8b molecule(CD8B)	Homo sapiens
2456	11735502_a_at	turquoise	TAP binding protein(TAPBP)	Homo sapiens
2457	11735504_a_at	turquoise	NLR family CARD domain containing 4(NLRC4)	Homo sapiens
2458	11735551_a_at	turquoise	Fc receptor like 6(FCRL6)	Homo sapiens
2459	11735552_at	turquoise	Fc receptor like 6(FCRL6)	Homo sapiens
2460	11735623_a_at	turquoise	proteasome subunit beta 9(PSMB9)	Homo sapiens
2461	11735710_s_at	turquoise	SAM domain, SH3 domain and nuclear localization signals 1(SAMSN1)	Homo sapiens
2462	11735711_a_at	turquoise	casein kinase 1 gamma 2(CSNK1G2)	Homo sapiens
2463	11735712_x_at	turquoise	casein kinase 1 gamma 2(CSNK1G2)	Homo sapiens
2464	11735713_x_at	turquoise	casein kinase 1 gamma 2(CSNK1G2)	Homo sapiens
2465	11735732_a_at	turquoise	ribonuclease P/MRP subunit p14(RPP14)	Homo sapiens
2466	11735740_a_at	turquoise	RAS p21 protein activator 3(RASA3)	Homo sapiens
2467	11735741_s_at	turquoise	RAS p21 protein activator 3(RASA3)	Homo sapiens
2468	11735767_at	turquoise	chromosome 16 open reading frame 54(C16orf54)	Homo sapiens
2469	11735768_x_at	turquoise	chromosome 16 open reading frame 54(C16orf54)	Homo sapiens
2470	11735790_x_at	turquoise	protein tyrosine phosphatase, non-receptor type 18(PTPN18)	Homo sapiens
2471	11735864_a_at	turquoise	CD160 molecule(CD160)	Homo sapiens
2472	11735937_a_at	turquoise	CD48 molecule(CD48)	Homo sapiens
2473	11735956_at	turquoise	methyltransferase like 11B(METTL11B)	Homo sapiens
2476	11735997_x_at	turquoise	apolipoprotein L1(APOL1)	Homo sapiens
2477	11735998_a_at	turquoise	apolipoprotein L1(APOL1)	Homo sapiens
2478	11736030_a_at	turquoise	microRNA 6787(MIR6787)	Homo sapiens
2481	11736064_x_at	turquoise	purinergic receptor P2X 7(P2RX7)	Homo sapiens
2482	11736065_x_at	turquoise	purinergic receptor P2X 7(P2RX7)	Homo sapiens
2483	11736094_a_at	turquoise	dCMP deaminase(DCTD)	Homo sapiens
2484	11736097_a_at	turquoise	golgi associated, gamma adaptin ear containing, ARF binding protein 1(GGA1)	Homo sapiens
2485	11736111_a_at	turquoise	Rho GTPase activating protein 18(ARHGAP18)	Homo sapiens
2486	11736112_a_at	turquoise	Rho GTPase activating protein 18(ARHGAP18)	Homo sapiens
2487	11736113_at	turquoise	Rho GTPase activating protein 18(ARHGAP18)	Homo sapiens
2488	11736132_s_at	turquoise	discoidin, CUB and LCCL domain containing 2(DCBLD2)	Homo sapiens
2489	11736133_s_at	turquoise	PHD finger protein 19(PHF19)	Homo sapiens
2490	11736135_at	turquoise	2'-5'-oligoadenylate synthetase 2(OAS2)	Homo sapiens
2491	11736136_s_at	turquoise	2'-5'-oligoadenylate synthetase 2(OAS2)	Homo sapiens
2492	11736207_a_at	turquoise	plexin C1(PLXNC1)	Homo sapiens
2493	11736217_at	turquoise	mitogen-activated protein kinase kinase kinase 8(MAP3K8)	Homo sapiens
2494	11736224_x_at	turquoise	spermatogenesis associated serine rich 2(SPATS2)	Homo sapiens
2495	11736225_a_at	turquoise	spermatogenesis associated serine rich 2(SPATS2)	Homo sapiens
2496	11736233_s_at	turquoise	phosphotriesterase related(PTER)	Homo sapiens
2497	11736241_a_at	turquoise	ORMDL sphingolipid biosynthesis regulator 2(ORMDL2)	Homo sapiens
2498	11736247_x_at	turquoise	adhesion molecule with Ig like domain 2(AMIGO2)	Homo sapiens
2499	11736255_a_at	turquoise	inositol polyphosphate-4-phosphatase type I A(INPP4A)	Homo sapiens
2500	11736273_a_at	turquoise	toll like receptor 2(TLR2)	Homo sapiens
2502	11736297_a_at	turquoise	solute carrier family 5 member 1(SLC5A1)	Homo sapiens
2503	11736311_x_at	turquoise	Fc fragment of IgG receptor Ic, pseudogene(FCGR1CP)	Homo sapiens
2504	11736324_x_at	turquoise	CD96 molecule(CD96)	Homo sapiens
2505	11736330_a_at	turquoise	solute carrier family 2 member 11(SLC2A11)	Homo sapiens
2506	11736342_at	turquoise	early growth response 3(EGR3)	Homo sapiens
2507	11736375_a_at	turquoise	CD86 molecule(CD86)	Homo sapiens
2508	11736376_a_at	turquoise	membrane palmitoylated protein 3(MPP3)	Homo sapiens
2509	11736378_at	turquoise	male-specific lethal 2 homolog (Drosophila)(MSL2)	Homo sapiens
2510	11736394_x_at	turquoise	interleukin 32(IL32)	Homo sapiens
2511	11736405_a_at	turquoise	DNA methyltransferase 1(DNMT1)	Homo sapiens
2512	11736409_a_at	turquoise	nucleoporin 62(NUP62)	Homo sapiens
2513	11736414_a_at	turquoise	melanotransferrin(MELTF)	Homo sapiens
2514	11736419_a_at	turquoise	zinc finger protein 800(ZNF800)	Homo sapiens
2515	11736430_a_at	turquoise	nucleolar protein 8(NOL8)	Homo sapiens
2517	11736457_at	turquoise	potassium two pore domain channel subfamily K member 6(KCNK6)	Homo sapiens
2518	11736458_x_at	turquoise	potassium two pore domain channel subfamily K member 6(KCNK6)	Homo sapiens
2519	11736467_a_at	turquoise	T-cell activation RhoGTPase activating protein(TAGAP)	Homo sapiens
2520	11736499_at	turquoise	transmembrane protein 38A(TMEM38A)	Homo sapiens
2521	11736519_x_at	turquoise	chromosome X open reading frame 38(CXorf38)	Homo sapiens
2522	11736526_a_at	turquoise	caspase recruitment domain family member 9(CARD9)	Homo sapiens
2523	11736539_x_at	turquoise	RAB29, member RAS oncogene family(RAB29)	Homo sapiens
2524	11736550_x_at	turquoise	RAB29, member RAS oncogene family(RAB29)	Homo sapiens
2525	11736551_x_at	turquoise	REL proto-oncogene, NF-kB subunit(REL)	Homo sapiens
2526	11736555_s_at	turquoise	solute carrier family 2 member 14(SLC2A14)	Homo sapiens
2527	11736556_s_at	turquoise	solute carrier family 2 member 14(SLC2A14)	Homo sapiens
2528	11736567_a_at	turquoise	lymphoid enhancer binding factor 1(LEF1)	Homo sapiens
2529	11736568_x_at	turquoise	lymphoid enhancer binding factor 1(LEF1)	Homo sapiens

2530	11736573_a_at	turquoise	protein kinase C delta(PRKCD)	Homo sapiens
2531	11736578_a_at	turquoise	adaptor related protein complex 1 gamma 2 subunit(AP1G2)	Homo sapiens
2532	11736581_a_at	turquoise	glucosaminyl (N-acetyl) transferase 1, core 2(GCNT1)	Homo sapiens
2534	11736590_a_at	turquoise	receptor activity modifying protein 3(RAMP3)	Homo sapiens
2535	11736594_x_at	turquoise	zinc finger and BTB domain containing 24(ZBTB24)	Homo sapiens
2536	11736619_a_at	turquoise	aldehyde dehydrogenase 5 family member A1(ALDH5A1)	Homo sapiens
2537	11736637_s_at	turquoise	DENN domain containing 4A(DENND4A)	Homo sapiens
2538	11736641_x_at	turquoise	mitochondrial trans-2-enoyl-CoA reductase(MECR)	Homo sapiens
2539	11736683_a_at	turquoise	cytoplasmic FMR1 interacting protein 1(CYFIP1)	Homo sapiens
2540	11736693_a_at	turquoise	tripartite motif containing 38(TRIM38)	Homo sapiens
2541	11736694_x_at	turquoise	tripartite motif containing 38(TRIM38)	Homo sapiens
2542	11736705_x_at	turquoise	HPS5, biogenesis of lysosomal organelles complex 2 subunit 2(HPS5)	Homo sapiens
2543	11736710_a_at	turquoise	DENN domain containing 4A(DENND4A)	Homo sapiens
2544	11736742_a_at	turquoise	chromosome 1 open reading frame 168(C1orf168)	Homo sapiens
2545	11736746_a_at	turquoise	ADAM metallopeptidase domain 28(ADAM28)	Homo sapiens
2546	11736747_x_at	turquoise	ADAM metallopeptidase domain 28(ADAM28)	Homo sapiens
2547	11736760_a_at	turquoise	FK506 binding protein 11(FKBP11)	Homo sapiens
2548	11736761_at	turquoise	caspase recruitment domain family member 16(CARD16)	Homo sapiens
2550	11736813_at	turquoise	programmed cell death 1 ligand 2(PDCD1LG2)	Homo sapiens
2551	11736831_a_at	turquoise	Sec23 homolog B, coat complex II component(SEC23B)	Homo sapiens
2553	11736888_x_at	turquoise	integrin subunit alpha L(ITGAL)	Homo sapiens
2555	11736973_a_at	turquoise	T-box 5(TBX5)	Homo sapiens
2556	11737026_at	turquoise	iroquois homeobox 4(IRX4)	Homo sapiens
2557	11737027_a_at	turquoise	fms related tyrosine kinase 3(FLT3)	Homo sapiens
2558	11737038_at	turquoise	chromosome 19 open reading frame 38(C19orf38)	Homo sapiens
2559	11737050_x_at	turquoise	histamine receptor H1(HRH1)	Homo sapiens
2560	11737058_a_at	turquoise	pantothenate kinase 2(PANK2)	Homo sapiens
2561	11737131_at	turquoise	fat storage inducing transmembrane protein 2(FITM2)	Homo sapiens
2562	11737146_a_at	turquoise	suppressor of cytokine signaling 1(SOCS1)	Homo sapiens
2563	11737147_a_at	turquoise	C-type lectin domain family 7 member A(CLEC7A)	Homo sapiens
2564	11737148_x_at	turquoise	C-type lectin domain family 7 member A(CLEC7A)	Homo sapiens
2565	11737160_at	turquoise	perilipin 5(PLIN5)	Homo sapiens
2566	11737171_at	turquoise	CD80 molecule(CD80)	Homo sapiens
2567	11737237_a_at	turquoise	eomesodermin(EOMES)	Homo sapiens
2568	11737238_s_at	turquoise	eomesodermin(EOMES)	Homo sapiens
2569	11737239_x_at	turquoise	eomesodermin(EOMES)	Homo sapiens
2571	11737250_at	turquoise	interleukin 1 receptor accessory protein like 1(IL1RAPL1)	Homo sapiens
2572	11737256_at	turquoise	SP100 nuclear antigen(SP100)	Homo sapiens
2574	11737322_a_at	turquoise	phospholipase D1(PLD1)	Homo sapiens
2575	11737423_a_at	turquoise	NLR family pyrin domain containing 1(NLRP1)	Homo sapiens
2576	11737428_a_at	turquoise	macrophage scavenger receptor 1(MSR1)	Homo sapiens
2577	11737429_x_at	turquoise	macrophage scavenger receptor 1(MSR1)	Homo sapiens
2578	11737464_a_at	turquoise	interleukin 11 receptor subunit alpha(IL11RA)	Homo sapiens
2579	11737484_at	turquoise	chromosome 9 open reading frame 129(C9orf129)	Homo sapiens
2580	11737496_a_at	turquoise	CD200 receptor 1(CD200R1)	Homo sapiens
2581	11737504_x_at	turquoise	signal regulatory protein beta 2(SIRPB2)	Homo sapiens
2582	11737511_a_at	turquoise	SP100 nuclear antigen(SP100)	Homo sapiens
2583	11737555_s_at	turquoise	nudix hydrolase 17(NUDT17)	Homo sapiens
2584	11737750_s_at	turquoise	serum/glucocorticoid regulated kinase 1(SGK1)	Homo sapiens
2585	11737757_a_at	turquoise	cysteinyl-tRNA synthetase(CARS)	Homo sapiens
2586	11737758_x_at	turquoise	cysteinyl-tRNA synthetase(CARS)	Homo sapiens
2587	11737770_a_at	turquoise	apolipoprotein L4(APOL4)	Homo sapiens
2588	11737791_s_at	turquoise	apolipoprotein L2(APOL2)	Homo sapiens
2590	11737847_a_at	turquoise	major histocompatibility complex, class I-related(MR1)	Homo sapiens
2591	11737851_a_at	turquoise	protein phosphatase 1 regulatory subunit 18(PPP1R18)	Homo sapiens
2592	11737852_s_at	turquoise	protein phosphatase 1 regulatory subunit 18(PPP1R18)	Homo sapiens
2593	11737890_x_at	turquoise	linker for activation of T-cells family member 2(LAT2)	Homo sapiens
2594	11737907_s_at	turquoise	protein phosphatase 1 catalytic subunit alpha(PPP1CA)	Homo sapiens
2595	11737911_a_at	turquoise	G protein-coupled receptor 84(GPR84)	Homo sapiens
2596	11737940_s_at	turquoise	receptor interacting serine/threonine kinase 1(RIPK1)	Homo sapiens
2597	11737952_a_at	turquoise	ral guanine nucleotide dissociation stimulator(RALGDS)	Homo sapiens
2598	11737975_x_at	turquoise	p21 (RAC1) activated kinase 6(PAK6)	Homo sapiens
2599	11737980_a_at	turquoise	DAN domain BMP antagonist family member 5(DAND5)	Homo sapiens
2601	11738021_a_at	turquoise	phospholipase C beta 4(PLCB4)	Homo sapiens
2602	11738053_x_at	turquoise	plasminogen activator, urokinase receptor(PLAUR)	Homo sapiens
2604	11738124_x_at	turquoise	sialic acid binding Ig like lectin 10(SIGLEC10)	Homo sapiens
2605	11738139_s_at	turquoise	kelch like family member 33(KLHL33)	Homo sapiens
2606	11738168_a_at	turquoise	basic leucine zipper ATF-like transcription factor 3(BATF3)	Homo sapiens
2607	11738183_a_at	turquoise	NLR family pyrin domain containing 3(NLRP3)	Homo sapiens
2608	11738193_a_at	turquoise	paired like homeodomain 3(PITX3)	Homo sapiens
2609	11738202_x_at	turquoise	death domain associated protein(DAXX)	Homo sapiens
2610	11738203_x_at	turquoise	inosine monophosphate dehydrogenase 1(IMPDH1)	Homo sapiens
2611	11738268_a_at	turquoise	family with sequence similarity 122C(FAM122C)	Homo sapiens
2613	11738335_x_at	turquoise	tumor protein p53(TP53)	Homo sapiens
2614	11738435_x_at	turquoise	CD74 molecule(CD74)	Homo sapiens
2615	11738460_at	turquoise	NUT family member 2G(NUTM2G)	Homo sapiens
2616	11738516_s_at	turquoise	zinc finger CCHC-type containing 6(ZCCHC6)	Homo sapiens
2617	11738523_a_at	turquoise	NLR family pyrin domain containing 3(NLRP3)	Homo sapiens
2618	11738540_a_at	turquoise	HHIP like 1(HHIPL1)	Homo sapiens
2619	11738656_a_at	turquoise	PARK2 coregulated(PACRG)	Homo sapiens
2620	11738799_x_at	turquoise	ninein(NIN)	Homo sapiens
2621	11738807_s_at	turquoise	c-Maf inducing protein(CMIP)	Homo sapiens

2622	11738858_x_at	turquoise	colony stimulating factor 2 receptor alpha subunit(CSF2RA)	Homo sapiens
2623	11738863_a_at	turquoise	protein tyrosine phosphatase, non-receptor type 2(PTPN2)	Homo sapiens
2624	11738883_x_at	turquoise	tumor necrosis factor superfamily member 14(TNFSF14)	Homo sapiens
2625	11738884_x_at	turquoise	caspase 1(CASP1)	Homo sapiens
2626	11738900_x_at	turquoise	small nucleolar RNA, C/D box 139(SNORD139)	Homo sapiens
2627	11738935_a_at	turquoise	killer cell lectin like receptor C3(KLRC3)	Homo sapiens
2628	11738980_a_at	turquoise	CD4 molecule(CD4)	Homo sapiens
2629	11738981_x_at	turquoise	CD4 molecule(CD4)	Homo sapiens
2630	11738982_at	turquoise	CD4 molecule(CD4)	Homo sapiens
2631	11739005_at	turquoise	capping actin protein of muscle Z-line alpha subunit 1(CAPZA1)	Homo sapiens
2632	11739006_x_at	turquoise	capping actin protein of muscle Z-line alpha subunit 1(CAPZA1)	Homo sapiens
2633	11739007_at	turquoise	capping actin protein of muscle Z-line alpha subunit 1(CAPZA1)	Homo sapiens
2634	11739008_x_at	turquoise	capping actin protein of muscle Z-line alpha subunit 1(CAPZA1)	Homo sapiens
2635	11739009_a_at	turquoise	myosin heavy chain 9(MYH9)	Homo sapiens
2636	11739019_x_at	turquoise	RAB5C, member RAS oncogene family(RAB5C)	Homo sapiens
2637	11739024_s_at	turquoise	adenosine deaminase, RNA specific(ADAR)	Homo sapiens
2638	11739028_s_at	turquoise	clathrin heavy chain(CLTC)	Homo sapiens
2639	11739031_s_at	turquoise	ARP2 actin related protein 2 homolog(ACTR2)	Homo sapiens
2640	11739032_x_at	turquoise	ARP2 actin related protein 2 homolog(ACTR2)	Homo sapiens
2641	11739043_a_at	turquoise	LIM domain kinase 2(LIMK2)	Homo sapiens
2642	11739046_s_at	turquoise	multimerin 2(MMRN2)	Homo sapiens
2643	11739052_a_at	turquoise	solute carrier organic anion transporter family member 2B1(SLCO2B1)	Homo sapiens
2644	11739053_a_at	turquoise	solute carrier organic anion transporter family member 2B1(SLCO2B1)	Homo sapiens
2645	11739054_a_at	turquoise	protein tyrosine phosphatase, non-receptor type 6(PTPN6)	Homo sapiens
2646	11739055_x_at	turquoise	protein tyrosine phosphatase, non-receptor type 6(PTPN6)	Homo sapiens
2648	11739075_a_at	turquoise	nuclear factor, erythroid 2 like 1(NFE2L1)	Homo sapiens
2649	11739094_a_at	turquoise	C-X-C motif chemokine receptor 4(CXCR4)	Homo sapiens
2650	11739113_a_at	turquoise	ADP dependent glucokinase(ADPGK)	Homo sapiens
2651	11739118_a_at	turquoise	canopy FGF signaling regulator 3(CNPY3)	Homo sapiens
2652	11739119_s_at	turquoise	canopy FGF signaling regulator 3(CNPY3)	Homo sapiens
2653	11739142_x_at	turquoise	heterogeneous nuclear ribonucleoprotein F(HNRNPF)	Homo sapiens
2654	11739148_a_at	turquoise	SAM and HD domain containing deoxynucleoside triphosphate triphosphohydrolase 1(SAMHD1)	Homo sapiens
2655	11739149_a_at	turquoise	SAM and HD domain containing deoxynucleoside triphosphate triphosphohydrolase 1(SAMHD1)	Homo sapiens
2656	11739157_at	turquoise	thioredoxin related transmembrane protein 4(TMX4)	Homo sapiens
2657	11739168_a_at	turquoise	GTPase, IMAP family member 6(GIMAP6)	Homo sapiens
2659	11739202_a_at	turquoise	ATP synthase, H+ transporting, mitochondrial Fo complex subunit C3 (subunit 9)(ATP5G3)	Homo sapiens
2660	11739223_s_at	turquoise	neurofibromin 1 pseudogene 9(NF1P9)	Homo sapiens
2661	11739230_a_at	turquoise	ADP ribosylation factor like GTPase 4A(ARL4A)	Homo sapiens
2662	11739232_x_at	turquoise	Ras association domain family member 2(RASSF2)	Homo sapiens
2663	11739262_at	turquoise	vacuolar protein sorting 4 homolog B(VPS4B)	Homo sapiens
2664	11739280_a_at	turquoise	C-X-C motif chemokine ligand 16(CXCL16)	Homo sapiens
2665	11739285_a_at	turquoise	ADP ribosylation factor like GTPase 6 interacting protein 6(ARL6IP6)	Homo sapiens
2666	11739298_a_at	turquoise	LRR binding FLII interacting protein 1(LRRFIP1)	Homo sapiens
2668	11739334_a_at	turquoise	protein tyrosine phosphatase, receptor type C(PTPRC)	Homo sapiens
2670	11739346_s_at	turquoise	TNF receptor superfamily member 1A(TNFRSF1A)	Homo sapiens
2671	11739347_a_at	turquoise	phosphatidylinositol-5-phosphate 4-kinase type 2 alpha(PIP4K2A)	Homo sapiens
2672	11739348_at	turquoise	phosphatidylinositol-5-phosphate 4-kinase type 2 alpha(PIP4K2A)	Homo sapiens
2673	11739349_a_at	turquoise	angiomotin(AMOT)	Homo sapiens
2674	11739364_s_at	turquoise	family with sequence similarity 92 member A(FAM92A)	Homo sapiens
2675	11739375_at	turquoise	synaptotagmin 7(SYT7)	Homo sapiens
2676	11739392_a_at	turquoise	recombination signal binding protein for immunoglobulin kappa J region(RBPJ)	Homo sapiens
2677	11739400_s_at	turquoise	F-box protein 7(FBXO7)	Homo sapiens
2678	11739401_a_at	turquoise	CD74 molecule(CD74)	Homo sapiens
2679	11739419_a_at	turquoise	uncharacterized LOC100130460(CAND1.11)	Homo sapiens
2681	11739452_a_at	turquoise	malic enzyme 2(ME2)	Homo sapiens
2683	11739465_x_at	turquoise	alkB homolog 4, lysine demethylase(ALKBH4)	Homo sapiens
2684	11739470_at	turquoise	transmembrane protein 87B(TMEM87B)	Homo sapiens
2685	11739482_x_at	turquoise	placenta specific 8(PLAC8)	Homo sapiens
2686	11739484_a_at	turquoise	coiled-coil domain containing 88A(CCDC88A)	Homo sapiens
2687	11739487_s_at	turquoise	SUZ12 polycomb repressive complex 2 subunit(SUZ12)	Homo sapiens
2688	11739489_a_at	turquoise	ATPase phospholipid transporting 10D (putative)(ATP10D)	Homo sapiens
2689	11739492_a_at	turquoise	mannosyl (alpha-1,3-)-glycoprotein beta-1,4-N-acetylglucosaminyltransferase, isozyme A(MGAT4A)	Homo sapiens
2690	11739493_at	turquoise	mannosyl (alpha-1,3-)-glycoprotein beta-1,4-N-acetylglucosaminyltransferase, isozyme A(MGAT4A)	Homo sapiens
2691	11739494_at	turquoise	mannosyl (alpha-1,3-)-glycoprotein beta-1,4-N-acetylglucosaminyltransferase, isozyme A(MGAT4A)	Homo sapiens
2694	11739507_a_at	turquoise	PBX homeobox 3(PBX3)	Homo sapiens
2697	11739521_at	turquoise	ST8 alpha-N-acetyl-neuraminide alpha-2,8-sialyltransferase 4(ST8SIA4)	Homo sapiens
2698	11739522_at	turquoise	ST8 alpha-N-acetyl-neuraminide alpha-2,8-sialyltransferase 4(ST8SIA4)	Homo sapiens
2699	11739527_a_at	turquoise	secreted and transmembrane 1(SECTM1)	Homo sapiens
2700	11739557_a_at	turquoise	vesicle associated membrane protein 5(VAMP5)	Homo sapiens
2701	11739558_at	turquoise	ring finger and WD repeat domain 3(RFWD3)	Homo sapiens
2702	11739576_x_at	turquoise	HPS5, biogenesis of lysosomal organelles complex 2 subunit 2(HPS5)	Homo sapiens
2703	11739581_at	turquoise	protein kinase D3(PRKD3)	Homo sapiens
2704	11739583_s_at	turquoise	protein kinase D3(PRKD3)	Homo sapiens
2705	11739584_s_at	turquoise	protein kinase D3(PRKD3)	Homo sapiens
2706	11739586_a_at	turquoise	chloride voltage-gated channel 3(CLCN3)	Homo sapiens
2707	11739606_x_at	turquoise	coiled-coil domain containing 88A(CCDC88A)	Homo sapiens
2708	11739610_a_at	turquoise	calmodulin like 4(CALML4)	Homo sapiens
2709	11739617_x_at	turquoise	CD44 molecule (Indian blood group)(CD44)	Homo sapiens
2710	11739654_at	turquoise	TNF receptor superfamily member 10a(TNFRSF10A)	Homo sapiens
2711	11739657_a_at	turquoise	lymphotoxin beta(LTB)	Homo sapiens
2712	11739658_a_at	turquoise	lymphotoxin beta(LTB)	Homo sapiens
2713	11739666_a_at	turquoise	RUN and cysteine rich domain containing beclin 1 interacting protein(RUBCN)	Homo sapiens

2714	11739667_at	turquoise	polypeptide N-acetylgalactosaminyltransferase 6(GALNT6)	Homo sapiens
2715	11739670_at	turquoise	GRB2-related adaptor protein(GRAP)	Homo sapiens
2716	11739672_x_at	turquoise	zinc finger protein 253(ZNF253)	Homo sapiens
2717	11739681_x_at	turquoise	aryl hydrocarbon receptor(AHR)	Homo sapiens
2718	11739687_a_at	turquoise	family with sequence similarity 107 member B(FAM107B)	Homo sapiens
2719	11739688_a_at	turquoise	family with sequence similarity 107 member B(FAM107B)	Homo sapiens
2720	11739730_at	turquoise	casein kinase 1 gamma 1(CSNK1G1)	Homo sapiens
2721	11739731_s_at	turquoise	casein kinase 1 gamma 1(CSNK1G1)	Homo sapiens
2722	11739739_a_at	turquoise	interleukin 21 receptor(IL21R)	Homo sapiens
2723	11739740_a_at	turquoise	interleukin 21 receptor(IL21R)	Homo sapiens
2725	11739753_at	turquoise	SLAM family member 8(SLAMF8)	Homo sapiens
2726	11739754_at	turquoise	SLAM family member 8(SLAMF8)	Homo sapiens
2727	11739767_a_at	turquoise	protein tyrosine phosphatase, receptor type E(PTPRE)	Homo sapiens
2728	11739768_a_at	turquoise	protein tyrosine phosphatase, receptor type E(PTPRE)	Homo sapiens
2729	11739796_a_at	turquoise	rhomboid 5 homolog 2(RHBDF2)	Homo sapiens
2731	11739825_a_at	turquoise	DnaJ heat shock protein family (Hsp40) member B12(DNAJB12)	Homo sapiens
2732	11739828_s_at	turquoise	cystin 1(CYS1)	Homo sapiens
2733	11739845_a_at	turquoise	macrophage scavenger receptor 1(MSR1)	Homo sapiens
2734	11739846_a_at	turquoise	macrophage scavenger receptor 1(MSR1)	Homo sapiens
2735	11739847_x_at	turquoise	macrophage scavenger receptor 1(MSR1)	Homo sapiens
2737	11739859_a_at	turquoise	protein kinase C and casein kinase substrate in neurons 1(PACSIN1)	Homo sapiens
2738	11739862_a_at	turquoise	integrator complex subunit 6 like(INTS6L)	Homo sapiens
2739	11739863_a_at	turquoise	transcription termination factor 2(TTF2)	Homo sapiens
2740	11739864_x_at	turquoise	transcription termination factor 2(TTF2)	Homo sapiens
2741	11739877_x_at	turquoise	basigin (Ok blood group)(BSG)	Homo sapiens
2743	11739917_a_at	turquoise	casein kinase 1 delta(CSNK1D)	Homo sapiens
2744	11739941_s_at	turquoise	SUMO1/sentrin specific peptidase 1(SENP1)	Homo sapiens
2745	11739944_x_at	turquoise	neuroblastoma breakpoint family member 10(NBPF10)	Homo sapiens
2746	11740006_a_at	turquoise	KIAA0895(KIAA0895)	Homo sapiens
2747	11740035_a_at	turquoise	caspase recruitment domain family member 8(CARD8)	Homo sapiens
2748	11740036_at	turquoise	caspase recruitment domain family member 8(CARD8)	Homo sapiens
2749	11740038_s_at	turquoise	ankyrin repeat domain 36(ANKRD36)	Homo sapiens
2752	11740061_a_at	turquoise	c-src tyrosine kinase(CSK)	Homo sapiens
2753	11740065_at	turquoise	interleukin 17 receptor A(IL17RA)	Homo sapiens
2754	11740077_a_at	turquoise	discs large MAGUK scaffold protein 2(DLG2)	Homo sapiens
2755	11740080_at	turquoise	chromosome X open reading frame 21(CXorf21)	Homo sapiens
2756	11740144_a_at	turquoise	centrosomal protein 164(CEP164)	Homo sapiens
2757	11740145_a_at	turquoise	centrosomal protein 164(CEP164)	Homo sapiens
2758	11740162_a_at	turquoise	ArfGAP with RhoGAP domain, ankyrin repeat and PH domain 1(ARAP1)	Homo sapiens
2759	11740197_s_at	turquoise	isoprenoid synthase domain containing(ISPD)	Homo sapiens
2760	11740212_x_at	turquoise	phosphatidylinositol-4,5-bisphosphate 3-kinase catalytic subunit gamma(PIK3CG)	Homo sapiens
2761	11740238_a_at	turquoise	sulfatase 2(SULF2)	Homo sapiens
2762	11740282_a_at	turquoise	tRNA splicing endonuclease subunit 2(TSEN2)	Homo sapiens
2763	11740302_a_at	turquoise	parvin gamma(PARVG)	Homo sapiens
2764	11740370_a_at	turquoise	RAS guanyl releasing protein 1(RASGRP1)	Homo sapiens
2765	11740375_a_at	turquoise	regulator of G-protein signaling 10(RGS10)	Homo sapiens
2766	11740378_a_at	turquoise	aftiphilin(AFTPH)	Homo sapiens
2767	11740390_a_at	turquoise	RNA binding motif protein 47(RBM47)	Homo sapiens
2768	11740393_at	turquoise	TNF receptor superfamily member 9(TNFRSF9)	Homo sapiens
2769	11740411_a_at	turquoise	protein kinase D2(PRKD2)	Homo sapiens
2770	11740412_x_at	turquoise	protein kinase D2(PRKD2)	Homo sapiens
2771	11740429_s_at	turquoise	5'-3' exoribonuclease 1(XRN1)	Homo sapiens
2772	11740444_s_at	turquoise	E74 like ETS transcription factor 1(ELF1)	Homo sapiens
2773	11740447_x_at	turquoise	butyrophilin subfamily 3 member A1(BTN3A1)	Homo sapiens
2774	11740450_a_at	turquoise	killer cell lectin like receptor D1(KLRD1)	Homo sapiens
2775	11740451_a_at	turquoise	killer cell lectin like receptor D1(KLRD1)	Homo sapiens
2776	11740452_x_at	turquoise	killer cell lectin like receptor D1(KLRD1)	Homo sapiens
2777	11740465_at	turquoise	G protein-coupled receptor 171(GPR171)	Homo sapiens
2778	11740486_a_at	turquoise	runt related transcription factor 3(RUNX3)	Homo sapiens
2779	11740499_a_at	turquoise	chromobox 5(CBX5)	Homo sapiens
2781	11740531_a_at	turquoise	C-type lectin domain family 10 member A(CLEC10A)	Homo sapiens
2782	11740594_s_at	turquoise	histone cluster 1 H3 family member e(HIST1H3E)	Homo sapiens
2783	11740606_a_at	turquoise	hydroxylysine kinase(HYKK)	Homo sapiens
2784	11740616_a_at	turquoise	parkin RBR E3 ubiquitin protein ligase(PARK2)	Homo sapiens
2785	11740617_x_at	turquoise	parkin RBR E3 ubiquitin protein ligase(PARK2)	Homo sapiens
2786	11740621_a_at	turquoise	lysine demethylase 2B(KDM2B)	Homo sapiens
2787	11740657_a_at	turquoise	solute carrier family 22 member 5(SLC22A5)	Homo sapiens
2788	11740672_x_at	turquoise	COX15, cytochrome c oxidase assembly homolog(COX15)	Homo sapiens
2789	11740677_a_at	turquoise	GDP dissociation inhibitor 2(GDI2)	Homo sapiens
2790	11740681_a_at	turquoise	TGFB induced factor homeobox 1(TGIF1)	Homo sapiens
2791	11740691_x_at	turquoise	allograft inflammatory factor 1(AIF1)	Homo sapiens
2794	11740748_s_at	turquoise	MYC associated factor X(MAX)	Homo sapiens
2795	11740752_a_at	turquoise	folate receptor beta(FOLR2)	Homo sapiens
2796	11740758_a_at	turquoise	mir-99a-let-7c cluster host gene(MIR99AHG)	Homo sapiens
2797	11740801_a_at	turquoise	acyl-CoA dehydrogenase family member 10(ACAD10)	Homo sapiens
2798	11740821_a_at	turquoise	CD1e molecule(CD1E)	Homo sapiens
2799	11740825_a_at	turquoise	thymus, brain and testes associated(TBATA)	Homo sapiens
2800	11740829_at	turquoise	synapse defective Rho GTPase homolog 2(SYDE2)	Homo sapiens
2801	11740871_a_at	turquoise	membrane spanning 4-domains A7(MS4A7)	Homo sapiens
2802	11740872_a_at	turquoise	membrane spanning 4-domains A7(MS4A7)	Homo sapiens
2803	11740873_x_at	turquoise	membrane spanning 4-domains A7(MS4A7)	Homo sapiens
2804	11740874_a_at	turquoise	FES proto-oncogene, tyrosine kinase(FES)	Homo sapiens

2805	11740879_s_at	turquoise	annexin A2(ANXA2)	Homo sapiens
2806	11740880_x_at	turquoise	annexin A2(ANXA2)	Homo sapiens
2807	11740881_x_at	turquoise	interleukin 15(IL15)	Homo sapiens
2808	11740885_a_at	turquoise	oxysterol binding protein like 3(OSBPL3)	Homo sapiens
2809	11740890_a_at	turquoise	ADAM metallopeptidase domain 28(ADAM28)	Homo sapiens
2810	11740891_a_at	turquoise	2'-5'-oligoadenylate synthetase 1(OAS1)	Homo sapiens
2811	11740934_a_at	turquoise	nucleolar and spindle associated protein 1(NUSAP1)	Homo sapiens
2812	11740937_x_at	turquoise	lipoic acid synthetase(LIAS)	Homo sapiens
2813	11740938_a_at	turquoise	TGFB induced factor homeobox 1(TGIF1)	Homo sapiens
2814	11740957_x_at	turquoise	solute carrier family 2 member 6(SLC2A6)	Homo sapiens
2815	11740958_x_at	turquoise	centrosomal protein 63(CEP63)	Homo sapiens
2816	11740961_a_at	turquoise	CD33 molecule(CD33)	Homo sapiens
2817	11740971_x_at	turquoise	Ras and Rab interactor like(RINL)	Homo sapiens
2818	11740974_a_at	turquoise	cytoplasmic FMR1 interacting protein 1(CYFIP1)	Homo sapiens
2819	11740979_x_at	turquoise	LCK proto-oncogene, Src family tyrosine kinase(LCK)	Homo sapiens
2821	11740992_x_at	turquoise	microRNA 6837(MIR6837)	Homo sapiens
2822	11740997_a_at	turquoise	TRAF-type zinc finger domain containing 1(TRAFD1)	Homo sapiens
2823	11740998_a_at	turquoise	diacylglycerol kinase alpha(DGKA)	Homo sapiens
2824	11741016_a_at	turquoise	adaptor related protein complex 2 alpha 2 subunit(AP2A2)	Homo sapiens
2825	11741030_a_at	turquoise	pentatricopeptide repeat domain 2(PTCD2)	Homo sapiens
2827	11741035_a_at	turquoise	ribosomal protein S6 kinase A1(RPS6KA1)	Homo sapiens
2830	11741047_a_at	turquoise	vav guanine nucleotide exchange factor 1(VAV1)	Homo sapiens
2831	11741063_a_at	turquoise	uncharacterized LOC101928143(LOC101928143)	Homo sapiens
2832	11741067_x_at	turquoise	sulfatase modifying factor 2(SUMF2)	Homo sapiens
2833	11741076_a_at	turquoise	caspase 1(CASP1)	Homo sapiens
2834	11741121_a_at	turquoise	zinc finger protein 700(ZNF700)	Homo sapiens
2835	11741126_s_at	turquoise	schlafen family member 11(SLFN11)	Homo sapiens
2836	11741129_s_at	turquoise	calpain 2(CAPN2)	Homo sapiens
2837	11741133_s_at	turquoise	heterogeneous nuclear ribonucleoprotein F(HNRNPF)	Homo sapiens
2838	11741138_a_at	turquoise	gasdermin D(GSDMD)	Homo sapiens
2840	11741153_a_at	turquoise	neutrophil cytosolic factor 2(NCF2)	Homo sapiens
2841	11741158_at	turquoise	renalase, FAD dependent amine oxidase(RNLS)	Homo sapiens
2842	11741184_a_at	turquoise	uncharacterized LOC100130460(CAND1.11)	Homo sapiens
2843	11741187_a_at	turquoise	apolipoprotein L1(APOL1)	Homo sapiens
2844	11741190_a_at	turquoise	purinergic receptor P2Y10(P2RY10)	Homo sapiens
2846	11741195_x_at	turquoise	sialic acid binding Ig like lectin 14(SIGLEC14)	Homo sapiens
2847	11741202_x_at	turquoise	XIAP associated factor 1(XAF1)	Homo sapiens
2848	11741226_a_at	turquoise	EYA transcriptional coactivator and phosphatase 1(EYA1)	Homo sapiens
2849	11741229_a_at	turquoise	family with sequence similarity 111 member A(FAM111A)	Homo sapiens
2850	11741250_a_at	turquoise	SH2 domain containing 3C(SH2D3C)	Homo sapiens
2851	11741255_at	turquoise	apolipoprotein B mRNA editing enzyme catalytic subunit 3F(APOBEC3F)	Homo sapiens
2852	11741257_x_at	turquoise	apolipoprotein B mRNA editing enzyme catalytic subunit 3F(APOBEC3F)	Homo sapiens
2853	11741290_a_at	turquoise	tumor necrosis factor superfamily member 14(TNFSF14)	Homo sapiens
2854	11741309_x_at	turquoise	serine/threonine protein kinase 26(STK26)	Homo sapiens
2855	11741319_a_at	turquoise	small nucleolar RNA, H/ACA box 29(SNORA29)	Homo sapiens
2856	11741326_x_at	turquoise	endosulfine alpha(ENSA)	Homo sapiens
2860	11741435_s_at	turquoise	growth factor receptor bound protein 2(GRB2)	Homo sapiens
2862	11741477_a_at	turquoise	nephrocystin 1(NPHP1)	Homo sapiens
2863	11741493_a_at	turquoise	peroxisomal biogenesis factor 5(PEX5)	Homo sapiens
2864	11741519_s_at	turquoise	CD14 molecule(CD14)	Homo sapiens
2865	11741520_x_at	turquoise	CD14 molecule(CD14)	Homo sapiens
2866	11741535_a_at	turquoise	transmembrane protein 2(TMEM2)	Homo sapiens
2867	11741536_x_at	turquoise	transmembrane protein 2(TMEM2)	Homo sapiens
2868	11741554_x_at	turquoise	caspase 8(CASP8)	Homo sapiens
2869	11741555_s_at	turquoise	lipase A, lysosomal acid type(LIPA)	Homo sapiens
2870	11741567_x_at	turquoise	solute carrier organic anion transporter family member 2B1(SLCO2B1)	Homo sapiens
2871	11741581_s_at	turquoise	synaptosome associated protein 23(SNAP23)	Homo sapiens
2872	11741584_a_at	turquoise	ankyrin repeat and SOCS box containing 10(ASB10)	Homo sapiens
2873	11741594_a_at	turquoise	leukocyte specific transcript 1(LST1)	Homo sapiens
2874	11741605_a_at	turquoise	chromodomain helicase DNA binding protein 3(CHD3)	Homo sapiens
2875	11741606_s_at	turquoise	chromodomain helicase DNA binding protein 3(CHD3)	Homo sapiens
2876	11741619_a_at	turquoise	sodium voltage-gated channel alpha subunit 7(SCN7A)	Homo sapiens
2877	11741632_a_at	turquoise	transporter 2, ATP binding cassette subfamily B member(TAP2)	Homo sapiens
2879	11741660_a_at	turquoise	T-cell activation RhoGTPase activating protein(TAGAP)	Homo sapiens
2880	11741667_x_at	turquoise	lymphocyte transmembrane adaptor 1(LAX1)	Homo sapiens
2882	11741689_s_at	turquoise	sulfatase modifying factor 2(SUMF2)	Homo sapiens
2883	11741719_a_at	turquoise	adenosine deaminase, RNA specific(ADAR)	Homo sapiens
2884	11741720_s_at	turquoise	adenosine deaminase, RNA specific(ADAR)	Homo sapiens
2885	11741723_x_at	turquoise	DENN domain containing 2C(DENND2C)	Homo sapiens
2887	11741769_s_at	turquoise	sorting nexin family member 21(SNX21)	Homo sapiens
2888	11741785_at	turquoise	SH3 domain binding kinase family member 2(SBK2)	Homo sapiens
2890	11741793_a_at	turquoise	fucose mutarotase(FUOM)	Homo sapiens
2891	11741796_a_at	turquoise	SH3 domain binding protein 2(SH3BP2)	Homo sapiens
2892	11741797_a_at	turquoise	collagen type IV alpha 6 chain(COL4A6)	Homo sapiens
2893	11741856_s_at	turquoise	zinc finger protein 658(ZNF658)	Homo sapiens
2894	11741860_a_at	turquoise	caveolin 3(CAV3)	Homo sapiens
2895	11741870_a_at	turquoise	parkin RBR E3 ubiquitin protein ligase(PARK2)	Homo sapiens
2896	11741878_a_at	turquoise	promyelocytic leukemia(PML)	Homo sapiens
2897	11741879_x_at	turquoise	promyelocytic leukemia(PML)	Homo sapiens
2898	11741885_x_at	turquoise	caspase 1(CASP1)	Homo sapiens
2899	11741911_a_at	turquoise	guanylate binding protein 3(GBP3)	Homo sapiens
2900	11741980_a_at	turquoise	dual specificity phosphatase 6(DUSP6)	Homo sapiens

2901	11741981_x_at	turquoise	C-type lectin domain family 7 member A(CLEC7A)	Homo sapiens
2902	11741990_s_at	turquoise	C-C motif chemokine receptor like 2(CCRL2)	Homo sapiens
2903	11742022_s_at	turquoise	IKAROS family zinc finger 1(IKZF1)	Homo sapiens
2904	11742027_a_at	turquoise	angiopoietin 2(ANGPT2)	Homo sapiens
2905	11742028_a_at	turquoise	angiopoietin 2(ANGPT2)	Homo sapiens
2907	11742042_a_at	turquoise	interleukin 1 receptor associated kinase 4(IRAK4)	Homo sapiens
2908	11742043_a_at	turquoise	CD44 molecule (Indian blood group)(CD44)	Homo sapiens
2909	11742063_a_at	turquoise	butyrophilin subfamily 3 member A1(BTN3A1)	Homo sapiens
2910	11742071_x_at	turquoise	RAB27A, member RAS oncogene family(RAB27A)	Homo sapiens
2911	11742078_a_at	turquoise	family with sequence similarity 222 member A(FAM222A)	Homo sapiens
2912	11742107_a_at	turquoise	palmitoyl-protein thioesterase 1(PPT1)	Homo sapiens
2913	11742116_x_at	turquoise	lactate dehydrogenase A(LDHA)	Homo sapiens
2914	11742119_a_at	turquoise	nuclear transcription factor Y subunit gamma(NFYC)	Homo sapiens
2915	11742173_x_at	turquoise	leukocyte specific transcript 1(LST1)	Homo sapiens
2916	11742182_x_at	turquoise	POTE ankyrin domain family member E(POTEE)	Homo sapiens
2917	11742191_a_at	turquoise	uncharacterized LOC100506403(LOC100506403)	Homo sapiens
2918	11742216_a_at	turquoise	killer cell lectin like receptor D1(KLRD1)	Homo sapiens
2919	11742217_a_at	turquoise	protein tyrosine phosphatase, receptor type A(PTPRA)	Homo sapiens
2920	11742241_a_at	turquoise	IKAROS family zinc finger 3(IKZF3)	Homo sapiens
2922	11742282_a_at	turquoise	CD40 molecule(CD40)	Homo sapiens
2923	11742303_a_at	turquoise	aspartate beta-hydroxylase(ASPH)	Homo sapiens
2924	11742331_x_at	turquoise	ectodysplasin A(EDA)	Homo sapiens
2925	11742378_a_at	turquoise	aldo-keto reductase family 1 member B15(AKR1B15)	Homo sapiens
2926	11742386_a_at	turquoise	caspase 1(CASP1)	Homo sapiens
2927	11742439_s_at	turquoise	adenosine deaminase like(ADAL)	Homo sapiens
2928	11742449_a_at	turquoise	apolipoprotein L1(APOL1)	Homo sapiens
2929	11742456_a_at	turquoise	microRNA 4745(MIR4745)	Homo sapiens
2930	11742457_s_at	turquoise	microRNA 4745(MIR4745)	Homo sapiens
2931	11742482_a_at	turquoise	actin related protein 2/3 complex subunit 4(ARPC4)	Homo sapiens
2932	11742537_a_at	turquoise	BCL2 associated X, apoptosis regulator(BAX)	Homo sapiens
2933	11742678_s_at	turquoise	microRNA 4745(MIR4745)	Homo sapiens
2934	11742680_at	turquoise	heterogeneous nuclear ribonucleoprotein A2/B1(HNRNPA2B1)	Homo sapiens
2935	11742681_s_at	turquoise	endoplasmic reticulum protein 29(ERP29)	Homo sapiens
2936	11742686_a_at	turquoise	Fc fragment of IgG receptor and transporter(FCGRT)	Homo sapiens
2937	11742706_s_at	turquoise	RAB8A, member RAS oncogene family(RAB8A)	Homo sapiens
2938	11742710_a_at	turquoise	serglycin(SRGN)	Homo sapiens
2939	11742717_a_at	turquoise	transforming growth factor beta regulator 1(TBRG1)	Homo sapiens
2940	11742723_a_at	turquoise	signal transducer and activator of transcription 6(STAT6)	Homo sapiens
2941	11742724_x_at	turquoise	signal transducer and activator of transcription 6(STAT6)	Homo sapiens
2942	11742735_a_at	turquoise	nucleolar and spindle associated protein 1(NUSAP1)	Homo sapiens
2944	11742740_at	turquoise	gem nuclear organelle associated protein 5(GEMIN5)	Homo sapiens
2945	11742752_a_at	turquoise	ETS proto-oncogene 2, transcription factor(ETS2)	Homo sapiens
2946	11742765_at	turquoise	regulator of G-protein signaling 1(RGS1)	Homo sapiens
2947	11742784_at	turquoise	senataxin(SETX)	Homo sapiens
2948	11742785_a_at	turquoise	senataxin(SETX)	Homo sapiens
2950	11742800_s_at	turquoise	E2F transcription factor 3(E2F3)	Homo sapiens
2951	11742832_a_at	turquoise	abnormal spindle microtubule assembly(ASPM)	Homo sapiens
2952	11742833_a_at	turquoise	family with sequence similarity 149 member A(FAM149A)	Homo sapiens
2953	11742838_at	turquoise	arrestin beta 1(ARRB1)	Homo sapiens
2954	11742849_a_at	turquoise	ral guanine nucleotide dissociation stimulator like 3(RGL3)	Homo sapiens
2955	11742850_x_at	turquoise	major histocompatibility complex, class II, DR alpha(HLA-DRA)	Homo sapiens
2956	11742859_a_at	turquoise	pleckstrin(PLEK)	Homo sapiens
2957	11742863_a_at	turquoise	CD200 molecule(CD200)	Homo sapiens
2958	11742870_at	turquoise	actin related protein 2/3 complex subunit 2(ARPC2)	Homo sapiens
2959	11742881_s_at	turquoise	ubiquitin conjugating enzyme E2 B(UBE2B)	Homo sapiens
2960	11742889_at	turquoise	F-box protein 6(FBXO6)	Homo sapiens
2961	11742892_s_at	turquoise	proteasome subunit alpha 4(PSMA4)	Homo sapiens
2962	11742904_s_at	turquoise	BCL2 associated athanogene 2(BAG2)	Homo sapiens
2963	11742905_a_at	turquoise	DnaJ heat shock protein family (Hsp40) member C1(DNAJC1)	Homo sapiens
2964	11742906_a_at	turquoise	DnaJ heat shock protein family (Hsp40) member C1(DNAJC1)	Homo sapiens
2965	11742911_at	turquoise	immunoglobulin superfamily member 6(IGSF6)	Homo sapiens
2966	11742922_at	turquoise	lysosomal protein transmembrane 5(LAPTM5)	Homo sapiens
2967	11742923_a_at	turquoise	lysosomal protein transmembrane 5(LAPTM5)	Homo sapiens
2968	11742926_at	turquoise	carnitine palmitoyltransferase 2(CPT2)	Homo sapiens
2970	11742944_a_at	turquoise	coronin 1A(CORO1A)	Homo sapiens
2971	11742948_at	turquoise	myristoylated alanine rich protein kinase C substrate(MARCKS)	Homo sapiens
2972	11742950_s_at	turquoise	CCR4-NOT transcription complex subunit 8(CNOT8)	Homo sapiens
2973	11742964_a_at	turquoise	inositol 1,4,5-trisphosphate receptor type 3(ITPR3)	Homo sapiens
2976	11742985_at	turquoise	lymphocyte antigen 9(LY9)	Homo sapiens
2977	11742999_x_at	turquoise	peroxiredoxin 6(PRDX6)	Homo sapiens
2978	11743000_at	turquoise	CD83 molecule(CD83)	Homo sapiens
2980	11743003_a_at	turquoise	cytochrome P450 family 4 subfamily B member 1(CYP4B1)	Homo sapiens
2981	11743007_at	turquoise	NFKB inhibitor epsilon(NFKBIE)	Homo sapiens
2982	11743008_a_at	turquoise	uncharacterized LOC100505585(LOC100505585)	Homo sapiens
2983	11743009_x_at	turquoise	uncharacterized LOC100505585(LOC100505585)	Homo sapiens
2984	11743014_a_at	turquoise	disco interacting protein 2 homolog C(DIP2C)	Homo sapiens
2986	11743016_at	turquoise	disco interacting protein 2 homolog C(DIP2C)	Homo sapiens
2987	11743036_s_at	turquoise	spermidine/spermine N1-acetyltransferase 1(SAT1)	Homo sapiens
2989	11743055_at	turquoise	phospholipase A2 activating protein(PLAA)	Homo sapiens
2990	11743062_a_at	turquoise	plasminogen activator, urokinase receptor(PLAUR)	Homo sapiens
2991	11743063_x_at	turquoise	plasminogen activator, urokinase receptor(PLAUR)	Homo sapiens
2992	11743065_at	turquoise	cell division cycle 6(CDC6)	Homo sapiens

2993	11743071_a_at	turquoise	lysophosphatidic acid receptor 6(LPAR6)	Homo sapiens
2995	11743105_at	turquoise	pentatricopeptide repeat domain 3(PTCD3)	Homo sapiens
2996	11743120_s_at	turquoise	SENP3-EIF4A1 readthrough (NMD candidate)(SENP3-EIF4A1)	Homo sapiens
2997	11743127_x_at	turquoise	coiled-coil-helix-coiled-coil-helix domain containing 10(CHCHD10)	Homo sapiens
2998	11743129_at	turquoise	sortilin related receptor 1(SORL1)	Homo sapiens
2999	11743130_a_at	turquoise	sortilin related receptor 1(SORL1)	Homo sapiens
3000	11743157_a_at	turquoise	electron transfer flavoprotein dehydrogenase(ETFDH)	Homo sapiens
3001	11743166_at	turquoise	ETS variant 6(ETV6)	Homo sapiens
3002	11743167_at	turquoise	ETS variant 6(ETV6)	Homo sapiens
3003	11743168_at	turquoise	indoleamine 2,3-dioxygenase 1(IDO1)	Homo sapiens
3004	11743169_a_at	turquoise	leupaxin(LPXN)	Homo sapiens
3005	11743180_at	turquoise	nuclear protein, coactivator of histone transcription(NPAT)	Homo sapiens
3006	11743194_x_at	turquoise	TNF receptor superfamily member 14(TNFRSF14)	Homo sapiens
3007	11743196_a_at	turquoise	toll like receptor 4(TLR4)	Homo sapiens
3008	11743197_at	turquoise	toll like receptor 4(TLR4)	Homo sapiens
3010	11743290_at	turquoise	polypeptide N-acetylgalactosaminyltransferase 1(GALNT1)	Homo sapiens
3011	11743299_at	turquoise	signal recognition particle 72(SRP72)	Homo sapiens
3012	11743301_a_at	turquoise	signal recognition particle 72(SRP72)	Homo sapiens
3013	11743321_at	turquoise	cathepsin O(CTSO)	Homo sapiens
3015	11743332_a_at	turquoise	mitochondrial ribosomal protein L35(MRPL35)	Homo sapiens
3016	11743333_a_at	turquoise	mitochondrial ribosomal protein L35(MRPL35)	Homo sapiens
3017	11743334_a_at	turquoise	mitochondrial ribosomal protein L35(MRPL35)	Homo sapiens
3018	11743348_a_at	turquoise	CD47 molecule(CD47)	Homo sapiens
3019	11743350_a_at	turquoise	chromosome 15 open reading frame 48(C15orf48)	Homo sapiens
3020	11743352_s_at	turquoise	cordon-bleu WH2 repeat protein like 1(COBLL1)	Homo sapiens
3021	11743353_s_at	turquoise	signal transducer and activator of transcription 1(STAT1)	Homo sapiens
3022	11743361_at	turquoise	adenylosuccinate synthase(ADSS)	Homo sapiens
3023	11743362_s_at	turquoise	adenylosuccinate synthase(ADSS)	Homo sapiens
3024	11743377_a_at	turquoise	ninein(NIN)	Homo sapiens
3025	11743379_a_at	turquoise	abl interactor 1(ABI1)	Homo sapiens
3026	11743386_s_at	turquoise	pre-mRNA processing factor 40 homolog A(PRPF40A)	Homo sapiens
3027	11743394_a_at	turquoise	androgen induced 1(AIG1)	Homo sapiens
3028	11743406_x_at	turquoise	small integral membrane protein 14(SMIM14)	Homo sapiens
3029	11743428_a_at	turquoise	runt related transcription factor 3(RUNX3)	Homo sapiens
3030	11743433_a_at	turquoise	6-phosphogluconolactonase(PGLS)	Homo sapiens
3031	11743452_s_at	turquoise	cell division cycle associated 4(CDCA4)	Homo sapiens
3032	11743458_a_at	turquoise	family with sequence similarity 49 member A(FAM49A)	Homo sapiens
3034	11743475_a_at	turquoise	integrin subunit alpha X(ITGAX)	Homo sapiens
3035	11743501_a_at	turquoise	TNF receptor associated factor 3(TRAF3)	Homo sapiens
3038	11743530_a_at	turquoise	centromere protein K(CENPK)	Homo sapiens
3039	11743533_x_at	turquoise	RUN and FYVE domain containing 3(RUFY3)	Homo sapiens
3040	11743555_a_at	turquoise	queuine tRNA-ribosyltransferase accessory subunit 2(QTRT2)	Homo sapiens
3041	11743559_a_at	turquoise	protein tyrosine phosphatase, receptor type C(PTPRC)	Homo sapiens
3042	11743560_a_at	turquoise	protein tyrosine phosphatase, receptor type C(PTPRC)	Homo sapiens
3043	11743561_a_at	turquoise	protein tyrosine phosphatase, receptor type C(PTPRC)	Homo sapiens
3044	11743570_s_at	turquoise	echinoderm microtubule associated protein like 1(EML1)	Homo sapiens
3045	11743581_s_at	turquoise	ARP2 actin related protein 2 homolog(ACTR2)	Homo sapiens
3046	11743586_a_at	turquoise	poly(A) RNA polymerase D4, non-canonical(PAPD4)	Homo sapiens
3047	11743596_a_at	turquoise	protein tyrosine phosphatase, receptor type E(PTPRE)	Homo sapiens
3048	11743613_a_at	turquoise	tropomodulin 3(TMOD3)	Homo sapiens
3049	11743614_a_at	turquoise	ubiquitin specific peptidase 47(USP47)	Homo sapiens
3050	11743619_a_at	turquoise	dihydrouridine synthase 4 like(DUS4L)	Homo sapiens
3051	11743624_a_at	turquoise	translocase of inner mitochondrial membrane 22(TIMM22)	Homo sapiens
3052	11743635_at	turquoise	zinc and ring finger 2(ZNRF2)	Homo sapiens
3053	11743643_a_at	turquoise	dihydroorotate dehydrogenase (quinone)(DHODH)	Homo sapiens
3054	11743647_at	turquoise	high mobility group nucleosomal binding domain 4(HMGN4)	Homo sapiens
3056	11743658_at	turquoise	H2A histone family member Y2(H2AFY2)	Homo sapiens
3057	11743677_at	turquoise	purinergic receptor P2Y8(P2RY8)	Homo sapiens
3058	11743691_a_at	turquoise	mitochondrial ribosomal protein L30(MRPL30)	Homo sapiens
3059	11743704_x_at	turquoise	abhydrolase domain containing 11(ABHD11)	Homo sapiens
3060	11743709_at	turquoise	N(alpha)-acetyltransferase 15, NatA auxiliary subunit(NAA15)	Homo sapiens
3061	11743722_x_at	turquoise	MARCKS like 1(MARCKSL1)	Homo sapiens
3062	11743725_s_at	turquoise	major facilitator superfamily domain containing 1(MFSD1)	Homo sapiens
3063	11743726_s_at	turquoise	ras homolog family member A(RHOA)	Homo sapiens
3065	11743730_at	turquoise	tumor necrosis factor superfamily member 10(TNFSF10)	Homo sapiens
3066	11743731_a_at	turquoise	tumor necrosis factor superfamily member 10(TNFSF10)	Homo sapiens
3067	11743736_at	turquoise	motile sperm domain containing 2(MOSPD2)	Homo sapiens
3068	11743748_a_at	turquoise	linker for activation of T-cells(LAT)	Homo sapiens
3069	11743759_a_at	turquoise	CDC42 small effector 2(CDC42SE2)	Homo sapiens
3070	11743760_s_at	turquoise	CDC42 small effector 2(CDC42SE2)	Homo sapiens
3071	11743771_at	turquoise	GTPase, IMAP family member 7(GIMAP7)	Homo sapiens
3072	11743781_a_at	turquoise	5'-3' exoribonuclease 1(XRN1)	Homo sapiens
3073	11743808_a_at	turquoise	7-dehydrocholesterol reductase(DHCR7)	Homo sapiens
3074	11743813_a_at	turquoise	ecotropic viral integration site 2B(EVI2B)	Homo sapiens
3075	11743824_s_at	turquoise	topoisomerase (DNA) II binding protein 1(TOPBP1)	Homo sapiens
3076	11743841_at	turquoise	phosphatidylinositol specific phospholipase C X domain containing 2(PLCXD2)	Homo sapiens
3077	11743843_a_at	turquoise	DnaJ heat shock protein family (Hsp40) member C28(DNAJC28)	Homo sapiens
3078	11743856_a_at	turquoise	collagen triple helix repeat containing 1(CTHRC1)	Homo sapiens
3079	11743863_a_at	turquoise	ATPase plasma membrane Ca2+ transporting 1(ATP2B1)	Homo sapiens
3080	11743864_a_at	turquoise	ATPase plasma membrane Ca2+ transporting 1(ATP2B1)	Homo sapiens
3081	11743865_s_at	turquoise	ATPase plasma membrane Ca2+ transporting 1(ATP2B1)	Homo sapiens
3082	11743881_s_at	turquoise	melanoregulin(MREG)	Homo sapiens

3084	11743888_at	turquoise	zinc finger protein 10(ZNF10)	Homo sapiens
3085	11743890_at	turquoise	zinc finger protein 10(ZNF10)	Homo sapiens
3086	11743900_at	turquoise	class II major histocompatibility complex transactivator(CIITA)	Homo sapiens
3087	11743911_a_at	turquoise	phospholipid scramblase 1(PLSCR1)	Homo sapiens
3088	11743915_s_at	turquoise	SEL1L family member 3(SEL1L3)	Homo sapiens
3089	11743916_a_at	turquoise	NPC intracellular cholesterol transporter 1(NPC1)	Homo sapiens
3090	11743931_x_at	turquoise	endoplasmic reticulum protein 29(ERP29)	Homo sapiens
3091	11743960_a_at	turquoise	microRNA 6787(MIR6787)	Homo sapiens
3092	11743968_a_at	turquoise	cytochrome b-245 alpha chain(CYBA)	Homo sapiens
3094	11743984_a_at	turquoise	A-kinase anchoring protein 1(AKAP1)	Homo sapiens
3095	11743985_s_at	turquoise	A-kinase anchoring protein 1(AKAP1)	Homo sapiens
3096	11743986_at	turquoise	A-kinase anchoring protein 1(AKAP1)	Homo sapiens
3097	11744000_a_at	turquoise	NFKB inhibitor alpha(NFKBIA)	Homo sapiens
3098	11744002_s_at	turquoise	methylenetetrahydrofolate dehydrogenase (NADP+ dependent) 2, methenyltetrahydrofolate cyclohydrolase(MTHFD2)	Homo sapiens
3099	11744006_a_at	turquoise	vinculin(VCL)	Homo sapiens
3100	11744017_at	turquoise	fermitin family member 3(FERMT3)	Homo sapiens
3101	11744020_at	turquoise	nucleoporin 210(NUP210)	Homo sapiens
3102	11744027_s_at	turquoise	transmembrane protein 181(TMEM181)	Homo sapiens
3103	11744033_s_at	turquoise	S100 calcium binding protein A11(S100A11)	Homo sapiens
3104	11744034_a_at	turquoise	vasodilator-stimulated phosphoprotein(VASP)	Homo sapiens
3105	11744037_a_at	turquoise	c-Maf inducing protein(CMIP)	Homo sapiens
3107	11744054_a_at	turquoise	zinc finger NFX1-type containing 1(ZNFX1)	Homo sapiens
3110	11744077_at	turquoise	actin related protein 2/3 complex subunit 1B(ARPC1B)	Homo sapiens
3111	11744082_at	turquoise	mitochondrial ribosomal protein S31(MRPS31)	Homo sapiens
3112	11744118_a_at	turquoise	mitochondrial ribosomal protein L10(MRPL10)	Homo sapiens
3113	11744119_a_at	turquoise	microRNA 6734(MIR6734)	Homo sapiens
3114	11744129_at	turquoise	myosin IXB(MYO9B)	Homo sapiens
3115	11744134_at	turquoise	zinc finger SWIM-type containing 6(ZSWIM6)	Homo sapiens
3116	11744137_a_at	turquoise	calcium/calmodulin dependent protein kinase ID(CAMK1D)	Homo sapiens
3117	11744138_at	turquoise	calcium/calmodulin dependent protein kinase ID(CAMK1D)	Homo sapiens
3119	11744149_at	turquoise	lipin 2(LPIN2)	Homo sapiens
3120	11744150_s_at	turquoise	lipin 2(LPIN2)	Homo sapiens
3121	11744151_x_at	turquoise	lipin 2(LPIN2)	Homo sapiens
3122	11744153_a_at	turquoise	reticulon 1(RTN1)	Homo sapiens
3123	11744156_a_at	turquoise	ribosomal protein S6 kinase A1(RPS6KA1)	Homo sapiens
3124	11744162_a_at	turquoise	dual specificity phosphatase 4(DUSP4)	Homo sapiens
3127	11744176_at	turquoise	protein phosphatase 1 regulatory subunit 9B(PPP1R9B)	Homo sapiens
3128	11744178_a_at	turquoise	chromosome 19 open reading frame 66(C19orf66)	Homo sapiens
3129	11744180_a_at	turquoise	T-cell immune regulator 1, ATPase H+ transporting V0 subunit a3(TCIRG1)	Homo sapiens
3132	11744197_s_at	turquoise	myosin light chain 12B(MYL12B)	Homo sapiens
3135	11744209_s_at	turquoise	serpin family H member 1(SERPINH1)	Homo sapiens
3136	11744214_s_at	turquoise	TRAF-type zinc finger domain containing 1(TRAFD1)	Homo sapiens
3137	11744215_at	turquoise	ring finger protein 149(RNF149)	Homo sapiens
3141	11744236_a_at	turquoise	DExD/H-box helicase 60(DDX60)	Homo sapiens
3142	11744258_a_at	turquoise	ATPase Na+/K+ transporting subunit beta 3(ATP1B3)	Homo sapiens
3143	11744259_a_at	turquoise	ATPase Na+/K+ transporting subunit beta 3(ATP1B3)	Homo sapiens
3145	11744263_s_at	turquoise	ras-related C3 botulinum toxin substrate 2 (rho family, small GTP binding protein Rac2)(RAC2)	Homo sapiens
3146	11744273_a_at	turquoise	stem-loop binding protein(SLBP)	Homo sapiens
3147	11744274_at	turquoise	NDC80, kinetochore complex component(NDC80)	Homo sapiens
3149	11744276_a_at	turquoise	capping actin protein, gelsolin like(CAPG)	Homo sapiens
3151	11744300_at	turquoise	carbohydrate sulfotransferase 15(CHST15)	Homo sapiens
3152	11744323_s_at	turquoise	PWWP domain containing 2A(PWWP2A)	Homo sapiens
3153	11744331_a_at	turquoise	complement factor B(CFB)	Homo sapiens
3154	11744345_x_at	turquoise	tubulin alpha 1b(TUBA1B)	Homo sapiens
3155	11744351_a_at	turquoise	NLR family pyrin domain containing 1(NLRP1)	Homo sapiens
3156	11744353_at	turquoise	TatD DNase domain containing 2(TATDN2)	Homo sapiens
3157	11744360_x_at	turquoise	reticulon 4(RTN4)	Homo sapiens
3158	11744364_s_at	turquoise	inositol polyphosphate-5-phosphatase D(INPP5D)	Homo sapiens
3159	11744374_x_at	turquoise	major histocompatibility complex, class II, DR beta 1(HLA-DRB1)	Homo sapiens
3163	11744425_a_at	turquoise	KIAA0101(KIAA0101)	Homo sapiens
3164	11744426_x_at	turquoise	KIAA0101(KIAA0101)	Homo sapiens
3166	11744434_a_at	turquoise	poly(ADP-ribose) polymerase family member 9(PARP9)	Homo sapiens
3167	11744435_a_at	turquoise	dual specificity phosphatase 6(DUSP6)	Homo sapiens
3168	11744436_a_at	turquoise	microRNA 1178(MIR1178)	Homo sapiens
3169	11744441_a_at	turquoise	brain abundant membrane attached signal protein 1(BASP1)	Homo sapiens
3171	11744449_a_at	turquoise	Rho GTPase activating protein 4(ARHGAP4)	Homo sapiens
3172	11744450_x_at	turquoise	Rho GTPase activating protein 4(ARHGAP4)	Homo sapiens
3175	11744482_a_at	turquoise	transmembrane protein 246(TMEM246)	Homo sapiens
3176	11744484_s_at	turquoise	major histocompatibility complex, class II, DR alpha(HLA-DRA)	Homo sapiens
3177	11744485_a_at	turquoise	WD repeat domain 81(WDR81)	Homo sapiens
3178	11744486_x_at	turquoise	WD repeat domain 81(WDR81)	Homo sapiens
3179	11744495_a_at	turquoise	intraflagellar transport 46(IFT46)	Homo sapiens
3180	11744503_x_at	turquoise	arginine and serine rich protein 1(RSRP1)	Homo sapiens
3181	11744512_a_at	turquoise	TraB domain containing 2A(TRABD2A)	Homo sapiens
3182	11744525_s_at	turquoise	mitochondrial transcription termination factor 2(MTERF2)	Homo sapiens
3184	11744560_s_at	turquoise	microRNA 4723(MIR4723)	Homo sapiens
3185	11744562_x_at	turquoise	eva-1 homolog B(EVA1B)	Homo sapiens
3186	11744567_a_at	turquoise	CD72 molecule(CD72)	Homo sapiens
3187	11744599_x_at	turquoise	nucleoporin 62(NUP62)	Homo sapiens
3188	11744608_a_at	turquoise	poly(ADP-ribose) polymerase family member 3(PARP3)	Homo sapiens
3189	11744609_a_at	turquoise	ataxin 7(ATXN7)	Homo sapiens
3190	11744611_x_at	turquoise	paternally expressed 3(PEG3)	Homo sapiens

3191	11744612_a_at	turquoise	NudC domain containing 1(NUDCD1)	Homo sapiens
3192	11744613_a_at	turquoise	solute carrier family 6 member 13(SLC6A13)	Homo sapiens
3193	11744618_a_at	turquoise	dual specificity phosphatase 6(DUSP6)	Homo sapiens
3195	11744638_at	turquoise	SND1 intronic transcript 1(SND1-IT1)	Homo sapiens
3196	11744645_a_at	turquoise	transcobalamin 2(TCN2)	Homo sapiens
3197	11744652_a_at	turquoise	hyaluronan and proteoglycan link protein 2(HAPLN2)	Homo sapiens
3198	11744654_s_at	turquoise	CD44 molecule (Indian blood group)(CD44)	Homo sapiens
3199	11744660_s_at	turquoise	C-C motif chemokine ligand 4 like 1(CCL4L1)	Homo sapiens
3200	11744663_a_at	turquoise	target of myb1 like 1 membrane trafficking protein(TOM1L1)	Homo sapiens
3202	11744673_a_at	turquoise	SP100 nuclear antigen(SP100)	Homo sapiens
3203	11744675_a_at	turquoise	family with sequence similarity 208 member A(FAM208A)	Homo sapiens
3204	11744699_a_at	turquoise	tyrosyl-DNA phosphodiesterase 1(TDP1)	Homo sapiens
3205	11744700_x_at	turquoise	tyrosyl-DNA phosphodiesterase 1(TDP1)	Homo sapiens
3206	11744715_a_at	turquoise	solute carrier family 38 member 6(SLC38A6)	Homo sapiens
3207	11744717_a_at	turquoise	chromosome 18 open reading frame 8(C18orf8)	Homo sapiens
3208	11744718_a_at	turquoise	collagen triple helix repeat containing 1(CTHRC1)	Homo sapiens
3209	11744728_a_at	turquoise	golgin A4(GOLGA4)	Homo sapiens
3210	11744735_a_at	turquoise	transmembrane protein 200A(TMEM200A)	Homo sapiens
3211	11744749_a_at	turquoise	DnaJ heat shock protein family (Hsp40) member C11(DNAJC11)	Homo sapiens
3212	11744751_a_at	turquoise	serpin family B member 9(SERPINB9)	Homo sapiens
3213	11744761_x_at	turquoise	uncharacterized LOC100505585(LOC100505585)	Homo sapiens
3214	11744776_a_at	turquoise	nicotinamide N-methyltransferase(NNMT)	Homo sapiens
3215	11744783_a_at	turquoise	SP100 nuclear antigen(SP100)	Homo sapiens
3216	11744786_x_at	turquoise	opioid growth factor receptor(OGFR)	Homo sapiens
3217	11744789_a_at	turquoise	centromere protein U(CENPU)	Homo sapiens
3218	11744791_a_at	turquoise	fibronectin type III domain containing 5(FNDC5)	Homo sapiens
3219	11744793_x_at	turquoise	DLG associated protein 5(DLGAP5)	Homo sapiens
3220	11744796_a_at	turquoise	DAB2, clathrin adaptor protein(DAB2)	Homo sapiens
3221	11744797_s_at	turquoise	DAB2, clathrin adaptor protein(DAB2)	Homo sapiens
3222	11744800_x_at	turquoise	fms related tyrosine kinase 3 ligand(FLT3LG)	Homo sapiens
3223	11744809_a_at	turquoise	BORCS7-ASMT readthrough (NMD candidate)(BORCS7-ASMT)	Homo sapiens
3225	11744829_s_at	turquoise	major histocompatibility complex, class I, E(HLA-E)	Homo sapiens
3226	11744830_x_at	turquoise	nuclear pore complex interacting protein family member B5(NPIPB5)	Homo sapiens
3228	11744887_s_at	turquoise	zwilch kinetochore protein(ZWILCH)	Homo sapiens
3229	11744888_a_at	turquoise	lamin B1(LMNB1)	Homo sapiens
3230	11744889_a_at	turquoise	lamin B1(LMNB1)	Homo sapiens
3231	11744896_a_at	turquoise	G-protein signaling modulator 3(GPSM3)	Homo sapiens
3232	11744898_a_at	turquoise	proline rich coiled-coil 2C(PRRC2C)	Homo sapiens
3234	11744920_a_at	turquoise	outer dense fiber of sperm tails 3B(ODF3B)	Homo sapiens
3235	11744940_s_at	turquoise	abhydrolase domain containing 17A(ABHD17A)	Homo sapiens
3236	11744948_x_at	turquoise	semaphorin 3F(SEMA3F)	Homo sapiens
3237	11744953_a_at	turquoise	annexin A1(ANXA1)	Homo sapiens
3238	11744954_x_at	turquoise	annexin A1(ANXA1)	Homo sapiens
3239	11744955_a_at	turquoise	annexin A1(ANXA1)	Homo sapiens
3240	11744962_a_at	turquoise	ADP-ribosyltransferase 5(ART5)	Homo sapiens
3242	11744991_a_at	turquoise	acetyl-CoA carboxylase beta(ACACB)	Homo sapiens
3243	11744993_s_at	turquoise	sirtuin 7(SIRT7)	Homo sapiens
3244	11744995_s_at	turquoise	pantothenate kinase 1(PANK1)	Homo sapiens
3245	11745015_a_at	turquoise	SP100 nuclear antigen(SP100)	Homo sapiens
3246	11745021_a_at	turquoise	v-myc avian myelocytomatosis viral oncogene homolog(MYC)	Homo sapiens
3247	11745063_x_at	turquoise	leucine rich repeat containing 8 family member B(LRRC8B)	Homo sapiens
3248	11745084_a_at	turquoise	family with sequence similarity 168 member B(FAM168B)	Homo sapiens
3249	11745105_a_at	turquoise	meiotic double-stranded break formation protein 1(MEI1)	Homo sapiens
3250	11745114_a_at	turquoise	adhesion G protein-coupled receptor E2(ADGRE2)	Homo sapiens
3252	11745144_a_at	turquoise	C-type lectin domain family 4 member E(CLEC4E)	Homo sapiens
3254	11745165_a_at	turquoise	TRAF3 interacting protein 3(TRAF3IP3)	Homo sapiens
3255	11745171_x_at	turquoise	diaphanous related formin 1(DIAPH1)	Homo sapiens
3257	11745189_a_at	turquoise	apolipoprotein L1(APOL1)	Homo sapiens
3258	11745190_a_at	turquoise	FUS RNA binding protein(FUS)	Homo sapiens
3259	11745192_a_at	turquoise	erythrocyte membrane protein band 4.1 like 3(EPB41L3)	Homo sapiens
3261	11745232_s_at	turquoise	N-6 adenine-specific DNA methyltransferase 1 (putative)(N6AMT1)	Homo sapiens
3262	11745234_a_at	turquoise	3-hydroxyacyl-CoA dehydratase 4(HACD4)	Homo sapiens
3263	11745243_x_at	turquoise	neuroblastoma breakpoint family member 11(NBPF11)	Homo sapiens
3265	11745248_a_at	turquoise	ilvB acetolactate synthase like(ILVBL)	Homo sapiens
3267	11745266_x_at	turquoise	transporter 2, ATP binding cassette subfamily B member(TAP2)	Homo sapiens
3268	11745268_a_at	turquoise	tensin 1(TNS1)	Homo sapiens
3269	11745274_a_at	turquoise	par-3 family cell polarity regulator(PARD3)	Homo sapiens
3270	11745276_a_at	turquoise	sterol O-acyltransferase 1(SOAT1)	Homo sapiens
3271	11745289_a_at	turquoise	LIM domain and actin binding 1(LIMA1)	Homo sapiens
3272	11745302_a_at	turquoise	StAR related lipid transfer domain containing 3(STARD3)	Homo sapiens
3273	11745313_a_at	turquoise	synergin gamma(SYNRG)	Homo sapiens
3274	11745364_a_at	turquoise	signal transducer and activator of transcription 5A(STAT5A)	Homo sapiens
3275	11745376_a_at	turquoise	apolipoprotein L6(APOL6)	Homo sapiens
3277	11745403_a_at	turquoise	zinc finger protein 683(ZNF683)	Homo sapiens
3278	11745406_a_at	turquoise	MICAL like 2(MICALL2)	Homo sapiens
3279	11745409_a_at	turquoise	La ribonucleoprotein domain family member 1B(LARP1B)	Homo sapiens
3280	11745415_a_at	turquoise	diaphanous related formin 1(DIAPH1)	Homo sapiens
3281	11745421_a_at	turquoise	BH3 interacting domain death agonist(BID)	Homo sapiens
3282	11745440_s_at	turquoise	RELA proto-oncogene, NF-kB subunit(RELA)	Homo sapiens
3285	11745468_a_at	turquoise	gamma-aminobutyric acid type B receptor subunit 2(GABBR2)	Homo sapiens
3286	11745471_a_at	turquoise	zinc finger protein 276(ZNF276)	Homo sapiens
3287	11745477_x_at	turquoise	tweety family member 1(TTYH1)	Homo sapiens

3288	11745479_a_at	turquoise	glycerate kinase(GLYCTK)	Homo sapiens
3289	11745496_a_at	turquoise	serine peptidase inhibitor, Kunitz type 2(SPINT2)	Homo sapiens
3290	11745498_a_at	turquoise	interferon regulatory factor 3(IRF3)	Homo sapiens
3291	11745499_x_at	turquoise	interferon regulatory factor 3(IRF3)	Homo sapiens
3292	11745500_a_at	turquoise	C-type lectin domain family 10 member A(CLEC10A)	Homo sapiens
3293	11745501_s_at	turquoise	endothelial cell surface expressed chemotaxis and apoptosis regulator(ECSCR)	Homo sapiens
3294	11745509_a_at	turquoise	IQ motif containing GTPase activating protein 2(IQGAP2)	Homo sapiens
3296	11745524_a_at	turquoise	catenin beta interacting protein 1(CTNNBIP1)	Homo sapiens
3298	11745537_a_at	turquoise	nuclear factor of activated T-cells 3(NFATC3)	Homo sapiens
3301	11745560_a_at	turquoise	dedicator of cytokinesis 8(DOCK8)	Homo sapiens
3302	11745602_s_at	turquoise	clathrin light chain A(CLTA)	Homo sapiens
3303	11745648_x_at	turquoise	eukaryotic translation elongation factor 1 alpha 1(EEF1A1)	Homo sapiens
3304	11745737_x_at	turquoise	acyl-CoA dehydrogenase, very long chain(ACADVL)	Homo sapiens
3305	11745742_s_at	turquoise	FYVE, RhoGEF and PH domain containing 3(FGD3)	Homo sapiens
3306	11745772_x_at	turquoise	TNF receptor superfamily member 14(TNFRSF14)	Homo sapiens
3307	11745773_a_at	turquoise	TNF receptor superfamily member 14(TNFRSF14)	Homo sapiens
3308	11745775_a_at	turquoise	lipase A, lysosomal acid type(LIPA)	Homo sapiens
3309	11745801_s_at	turquoise	adenylate cyclase associated protein 1(CAP1)	Homo sapiens
3311	11745833_s_at	turquoise	epithelial cell transforming 2(ECT2)	Homo sapiens
3312	11745840_a_at	turquoise	progestin and adipoQ receptor family member 8(PAQR8)	Homo sapiens
3313	11745841_a_at	turquoise	CNDP dipeptidase 2 (metallopeptidase M20 family)(CNDP2)	Homo sapiens
3314	11745854_a_at	turquoise	ring finger protein 145(RNF145)	Homo sapiens
3317	11745860_x_at	turquoise	ATP5S like(ATP5SL)	Homo sapiens
3319	11745884_a_at	turquoise	ubiquitin conjugating enzyme E2 Z(UBE2Z)	Homo sapiens
3320	11745892_a_at	turquoise	methylenetetrahydrofolate dehydrogenase (NADP+ dependent) 2, methenyltetrahydrofolate cyclohydrolase(MTHFD2)	Homo sapiens
3321	11745893_s_at	turquoise	methylenetetrahydrofolate dehydrogenase (NADP+ dependent) 2, methenyltetrahydrofolate cyclohydrolase(MTHFD2)	Homo sapiens
3322	11745894_x_at	turquoise	TNF receptor superfamily member 14(TNFRSF14)	Homo sapiens
3323	11745902_a_at	turquoise	NPC intracellular cholesterol transporter 2(NPC2)	Homo sapiens
3324	11745903_a_at	turquoise	SLAM family member 7(SLAMF7)	Homo sapiens
3325	11745912_a_at	turquoise	pellino E3 ubiquitin protein ligase 1(PELI1)	Homo sapiens
3326	11745922_a_at	turquoise	monoamine oxidase A(MAOA)	Homo sapiens
3327	11745923_x_at	turquoise	monoamine oxidase A(MAOA)	Homo sapiens
3328	11745926_a_at	turquoise	ADAM metallopeptidase domain 15(ADAM15)	Homo sapiens
3329	11745927_x_at	turquoise	ADAM metallopeptidase domain 15(ADAM15)	Homo sapiens
3330	11745930_a_at	turquoise	p21 (RAC1) activated kinase 6(PAK6)	Homo sapiens
3331	11745932_x_at	turquoise	thymosin beta 10(TMSB10)	Homo sapiens
3332	11745936_a_at	turquoise	solute carrier family 50 member 1(SLC50A1)	Homo sapiens
3333	11745938_a_at	turquoise	absent in melanoma 1(AIM1)	Homo sapiens
3334	11745988_a_at	turquoise	ADP dependent glucokinase(ADPGK)	Homo sapiens
3335	11745989_a_at	turquoise	major histocompatibility complex, class II, DM alpha(HLA-DMA)	Homo sapiens
3336	11745991_a_at	turquoise	UV radiation resistance associated(UVRAG)	Homo sapiens
3337	11745998_at	turquoise	zinc finger protein 252, pseudogene(ZNF252P)	Homo sapiens
3338	11746002_a_at	turquoise	caspase 4(CASP4)	Homo sapiens
3339	11746003_a_at	turquoise	DNA polymerase delta interacting protein 2(POLDIP2)	Homo sapiens
3340	11746014_s_at	turquoise	neutrophil cytosolic factor 1(NCF1)	Homo sapiens
3341	11746027_a_at	turquoise	N-myc and STAT interactor(NMI)	Homo sapiens
3342	11746028_x_at	turquoise	N-myc and STAT interactor(NMI)	Homo sapiens
3343	11746041_a_at	turquoise	transformer 2 beta homolog (Drosophila)(TRA2B)	Homo sapiens
3344	11746042_s_at	turquoise	transformer 2 beta homolog (Drosophila)(TRA2B)	Homo sapiens
3345	11746048_s_at	turquoise	adenosine deaminase like(ADAL)	Homo sapiens
3346	11746053_x_at	turquoise	uncharacterized LOC100996740(LOC100996740)	Homo sapiens
3347	11746060_a_at	turquoise	TNF alpha induced protein 2(TNFAIP2)	Homo sapiens
3348	11746079_a_at	turquoise	adenylate cyclase 7(ADCY7)	Homo sapiens
3350	11746087_a_at	turquoise	CD84 molecule(CD84)	Homo sapiens
3351	11746088_a_at	turquoise	interferon induced protein 44(IFI44)	Homo sapiens
3352	11746092_a_at	turquoise	rogdi homolog(ROGDI)	Homo sapiens
3353	11746135_x_at	turquoise	microRNA 1292(MIR1292)	Homo sapiens
3354	11746149_x_at	turquoise	butyrylcholinesterase(BCHE)	Homo sapiens
3355	11746155_a_at	turquoise	interferon regulatory factor 9(IRF9)	Homo sapiens
3356	11746156_x_at	turquoise	interferon regulatory factor 9(IRF9)	Homo sapiens
3357	11746159_a_at	turquoise	PBX homeobox 3(PBX3)	Homo sapiens
3358	11746162_a_at	turquoise	family with sequence similarity 49 member B(FAM49B)	Homo sapiens
3359	11746175_a_at	turquoise	apolipoprotein L4(APOL4)	Homo sapiens
3360	11746217_a_at	turquoise	HPS3, biogenesis of lysosomal organelles complex 2 subunit 1(HPS3)	Homo sapiens
3361	11746242_a_at	turquoise	aryl hydrocarbon receptor nuclear translocator like 2(ARNTL2)	Homo sapiens
3362	11746251_x_at	turquoise	chloride intracellular channel 1(CLIC1)	Homo sapiens
3363	11746269_a_at	turquoise	abhydrolase domain containing 3(ABHD3)	Homo sapiens
3364	11746273_x_at	turquoise	dihydrouridine synthase 4 like(DUS4L)	Homo sapiens
3365	11746286_s_at	turquoise	butyrophilin subfamily 3 member A2(BTN3A2)	Homo sapiens
3366	11746309_a_at	turquoise	solute carrier family 15 member 3(SLC15A3)	Homo sapiens
3367	11746321_s_at	turquoise	serpin family H member 1(SERPINH1)	Homo sapiens
3369	11746340_a_at	turquoise	nucleolar protein 8(NOL8)	Homo sapiens
3370	11746347_s_at	turquoise	beta-1,3-glucuronyltransferase 3(B3GAT3)	Homo sapiens
3371	11746350_a_at	turquoise	Pim-1 proto-oncogene, serine/threonine kinase(PIM1)	Homo sapiens
3372	11746370_a_at	turquoise	ectonucleoside triphosphate diphosphohydrolase 6 (putative)(ENTPD6)	Homo sapiens
3374	11746376_a_at	turquoise	interleukin 16(IL16)	Homo sapiens
3375	11746400_a_at	turquoise	myosin light chain kinase 3(MYLK3)	Homo sapiens
3376	11746407_x_at	turquoise	toll like receptor 10(TLR10)	Homo sapiens
3377	11746414_a_at	turquoise	family with sequence similarity 107 member B(FAM107B)	Homo sapiens
3378	11746418_a_at	turquoise	vitrin(VIT)	Homo sapiens
3379	11746439_a_at	turquoise	glyoxalase I(GLO1)	Homo sapiens
3380	11746449_a_at	turquoise	Meis homeobox 2(MEIS2)	Homo sapiens

3381	11746450_x_at	turquoise	Meis homeobox 2(MEIS2)	Homo sapiens
3382	11746465_a_at	turquoise	epidermal growth factor receptor pathway substrate 15 like 1(EPS15L1)	Homo sapiens
3383	11746516_a_at	turquoise	ADAM metallopeptidase domain 15(ADAM15)	Homo sapiens
3384	11746524_a_at	turquoise	family with sequence similarity 81 member A(FAM81A)	Homo sapiens
3385	11746529_x_at	turquoise	TNF receptor superfamily member 14(TNFRSF14)	Homo sapiens
3386	11746557_s_at	turquoise	galectin 9B(LGALS9B)	Homo sapiens
3387	11746558_x_at	turquoise	galectin 9(LGALS9)	Homo sapiens
3388	11746568_x_at	turquoise	hypoxia inducible factor 3 alpha subunit(HIF3A)	Homo sapiens
3389	11746584_x_at	turquoise	metal response element binding transcription factor 2(MTF2)	Homo sapiens
3391	11746596_a_at	turquoise	NAD kinase(NADK)	Homo sapiens
3392	11746609_a_at	turquoise	cullin associated and neddylation dissociated 2 (putative)(CAND2)	Homo sapiens
3393	11746626_a_at	turquoise	hydroxyacylglutathione hydrolase(HAGH)	Homo sapiens
3394	11746631_a_at	turquoise	centrosomal protein 41(CEP41)	Homo sapiens
3395	11746635_a_at	turquoise	lymphoid enhancer binding factor 1(LEF1)	Homo sapiens
3396	11746638_a_at	turquoise	dehydrogenase/reductase 11(DHRS11)	Homo sapiens
3397	11746639_a_at	turquoise	Ras association domain family member 2(RASSF2)	Homo sapiens
3399	11746658_a_at	turquoise	CD86 molecule(CD86)	Homo sapiens
3400	11746659_a_at	turquoise	nuclear receptor subfamily 2 group F member 6(NR2F6)	Homo sapiens
3401	11746686_a_at	turquoise	G kinase anchoring protein 1(GKAP1)	Homo sapiens
3402	11746692_a_at	turquoise	bromodomain adjacent to zinc finger domain 1A(BAZ1A)	Homo sapiens
3403	11746705_a_at	turquoise	regulator of G-protein signaling 16(RGS16)	Homo sapiens
3404	11746710_a_at	turquoise	dedicator of cytokinesis 10(DOCK10)	Homo sapiens
3405	11746729_a_at	turquoise	lymphoid restricted membrane protein(LRMP)	Homo sapiens
3406	11746736_a_at	turquoise	calcium voltage-gated channel auxiliary subunit alpha2delta 3(CACNA2D3)	Homo sapiens
3407	11746767_a_at	turquoise	integrin subunit alpha 4(ITGA4)	Homo sapiens
3408	11746778_a_at	turquoise	intersectin 1(ITSN1)	Homo sapiens
3409	11746779_a_at	turquoise	nuclear receptor coactivator 7(NCOA7)	Homo sapiens
3410	11746804_x_at	turquoise	major histocompatibility complex, class II, DQ beta 1(HLA-DQB1)	Homo sapiens
3414	11746868_a_at	turquoise	GIT ArfGAP 2(GIT2)	Homo sapiens
3415	11746874_a_at	turquoise	G protein subunit alpha i2(GNAI2)	Homo sapiens
3416	11746875_x_at	turquoise	G protein subunit alpha i2(GNAI2)	Homo sapiens
3417	11746878_s_at	turquoise	inhibitor of DNA binding 2, HLH protein(ID2)	Homo sapiens
3418	11746880_x_at	turquoise	filamin A(FLNA)	Homo sapiens
3419	11746906_s_at	turquoise	caspase recruitment domain family member 8(CARD8)	Homo sapiens
3421	11746935_a_at	turquoise	tryptophanyl tRNA synthetase 2, mitochondrial(WARS2)	Homo sapiens
3422	11746954_s_at	turquoise	C-C motif chemokine ligand 4 like 1(CCL4L1)	Homo sapiens
3423	11746961_a_at	turquoise	major histocompatibility complex, class II, DM beta(HLA-DMB)	Homo sapiens
3424	11746962_x_at	turquoise	major histocompatibility complex, class II, DM beta(HLA-DMB)	Homo sapiens
3425	11746965_a_at	turquoise	alanyl aminopeptidase, membrane(ANPEP)	Homo sapiens
3426	11746972_a_at	turquoise	membrane associated ring-CH-type finger 1(MARCH1)	Homo sapiens
3427	11746995_a_at	turquoise	N-acetylneuraminate pyruvate lyase(NPL)	Homo sapiens
3428	11747004_a_at	turquoise	sulfite oxidase(SUOX)	Homo sapiens
3430	11747019_a_at	turquoise	Rho GTPase activating protein 45(ARHGAP45)	Homo sapiens
3431	11747020_x_at	turquoise	Rho GTPase activating protein 45(ARHGAP45)	Homo sapiens
3432	11747076_a_at	turquoise	microRNA 6837(MIR6837)	Homo sapiens
3433	11747077_x_at	turquoise	eukaryotic translation elongation factor 1 alpha 1(EEF1A1)	Homo sapiens
3434	11747086_a_at	turquoise	grancalcin(GCA)	Homo sapiens
3435	11747110_a_at	turquoise	PAS domain containing serine/threonine kinase(PASK)	Homo sapiens
3436	11747113_a_at	turquoise	CD160 molecule(CD160)	Homo sapiens
3438	11747135_x_at	turquoise	centriolin(CNTRL)	Homo sapiens
3439	11747137_x_at	turquoise	endoplasmic reticulum aminopeptidase 2(ERAP2)	Homo sapiens
3440	11747147_a_at	turquoise	protein phosphatase 2 scaffold subunit Abeta(PPP2R1B)	Homo sapiens
3441	11747189_a_at	turquoise	B-cell CLL/lymphoma 11A(BCL11A)	Homo sapiens
3442	11747190_a_at	turquoise	FCH and double SH3 domains 1(FCHSD1)	Homo sapiens
3443	11747212_a_at	turquoise	zinc finger protein 174(ZNF174)	Homo sapiens
3444	11747230_a_at	turquoise	BUB1 mitotic checkpoint serine/threonine kinase(BUB1)	Homo sapiens
3445	11747247_x_at	turquoise	RAP1B, member of RAS oncogene family(RAP1B)	Homo sapiens
3446	11747249_a_at	turquoise	adenylosuccinate synthase(ADSS)	Homo sapiens
3447	11747276_a_at	turquoise	A-kinase anchoring protein 1(AKAP1)	Homo sapiens
3449	11747282_x_at	turquoise	annexin A2 pseudogene 2(ANXA2P2)	Homo sapiens
3450	11747285_a_at	turquoise	target of myb1 like 1 membrane trafficking protein(TOM1L1)	Homo sapiens
3451	11747295_a_at	turquoise	perforin 1(PRF1)	Homo sapiens
3452	11747301_a_at	turquoise	SP100 nuclear antigen(SP100)	Homo sapiens
3457	11747381_a_at	turquoise	septin 1(SEPT1)	Homo sapiens
3459	11747408_x_at	turquoise	filamin binding LIM protein 1(FBLIM1)	Homo sapiens
3460	11747418_a_at	turquoise	napsin B aspartic peptidase, pseudogene(NAPSB)	Homo sapiens
3461	11747437_a_at	turquoise	caspase recruitment domain family member 8(CARD8)	Homo sapiens
3462	11747438_a_at	turquoise	GTPase, IMAP family member 4(GIMAP4)	Homo sapiens
3463	11747439_a_at	turquoise	myosin light chain 6(MYL6)	Homo sapiens
3464	11747446_s_at	turquoise	major histocompatibility complex, class II, DO beta(HLA-DOB)	Homo sapiens
3465	11747448_x_at	turquoise	butyrophilin subfamily 3 member A2(BTN3A2)	Homo sapiens
3466	11747449_s_at	turquoise	D-2-hydroxyglutarate dehydrogenase(D2HGDH)	Homo sapiens
3467	11747453_a_at	turquoise	protein phosphatase 1 regulatory subunit 18(PPP1R18)	Homo sapiens
3468	11747460_x_at	turquoise	transgelin 2(TAGLN2)	Homo sapiens
3469	11747467_a_at	turquoise	protein kinase C theta(PRKCQ)	Homo sapiens
3470	11747483_a_at	turquoise	TNF alpha induced protein 8(TNFAIP8)	Homo sapiens
3471	11747499_a_at	turquoise	intercellular adhesion molecule 1(ICAM1)	Homo sapiens
3472	11747502_s_at	turquoise	mitochondrial translational release factor 1 like(MTRF1L)	Homo sapiens
3473	11747508_a_at	turquoise	erythrocyte membrane protein band 4.1 like 3(EPB41L3)	Homo sapiens
3474	11747509_a_at	turquoise	family with sequence similarity 107 member B(FAM107B)	Homo sapiens
3475	11747523_a_at	turquoise	TNF receptor associated factor 4(TRAF4)	Homo sapiens
3477	11747538_a_at	turquoise	myeloid differentiation primary response 88(MYD88)	Homo sapiens

3478	11747539_x_at	turquoise	myeloid differentiation primary response 88(MYD88)	Homo sapiens
3482	11747590_a_at	turquoise	Fc fragment of IgM receptor(FCMR)	Homo sapiens
3483	11747591_a_at	turquoise	GLI pathogenesis related 1(GLIPR1)	Homo sapiens
3484	11747593_a_at	turquoise	SH3 domain binding glutamate rich protein like(SH3BGRL)	Homo sapiens
3485	11747594_x_at	turquoise	SH3 domain binding glutamate rich protein like(SH3BGRL)	Homo sapiens
3486	11747597_x_at	turquoise	C-type lectin domain family 2 member D(CLEC2D)	Homo sapiens
3487	11747602_a_at	turquoise	mitogen-activated protein kinase kinase kinase 8(MAP3K8)	Homo sapiens
3488	11747607_a_at	turquoise	purinergic receptor P2X 7(P2RX7)	Homo sapiens
3489	11747623_a_at	turquoise	Rho GTPase activating protein 25(ARHGAP25)	Homo sapiens
3490	11747627_s_at	turquoise	tRNA-yW synthesizing protein 1 homolog B(TYW1B)	Homo sapiens
3491	11747630_a_at	turquoise	XIAP associated factor 1(XAF1)	Homo sapiens
3492	11747631_x_at	turquoise	XIAP associated factor 1(XAF1)	Homo sapiens
3493	11747643_s_at	turquoise	BUB3, mitotic checkpoint protein(BUB3)	Homo sapiens
3494	11747652_a_at	turquoise	cell division cycle 25B(CDC25B)	Homo sapiens
3495	11747653_x_at	turquoise	cell division cycle 25B(CDC25B)	Homo sapiens
3496	11747666_a_at	turquoise	tryptophanyl-tRNA synthetase(WARS)	Homo sapiens
3498	11747680_a_at	turquoise	thyroid hormone receptor, alpha(THRA)	Homo sapiens
3499	11747681_x_at	turquoise	thyroid hormone receptor, alpha(THRA)	Homo sapiens
3500	11747714_a_at	turquoise	ATP binding cassette subfamily A member 8(ABCA8)	Homo sapiens
3501	11747723_a_at	turquoise	death domain associated protein(DAXX)	Homo sapiens
3502	11747726_s_at	turquoise	syntaxin binding protein 6(STXBP6)	Homo sapiens
3503	11747731_a_at	turquoise	DBF4 zinc finger(DBF4)	Homo sapiens
3504	11747742_a_at	turquoise	metal response element binding transcription factor 2(MTF2)	Homo sapiens
3505	11747743_x_at	turquoise	metal response element binding transcription factor 2(MTF2)	Homo sapiens
3507	11747785_s_at	turquoise	lymphocyte cytosolic protein 1(LCP1)	Homo sapiens
3508	11747799_x_at	turquoise	enolase 1(ENO1)	Homo sapiens
3509	11747803_a_at	turquoise	CDC like kinase 2(CLK2)	Homo sapiens
3510	11747811_x_at	turquoise	GRINL1A complex locus 1(GCOM1)	Homo sapiens
3511	11747820_x_at	turquoise	uridine phosphorylase 1(UPP1)	Homo sapiens
3512	11747895_a_at	turquoise	tectonic family member 1(TCTN1)	Homo sapiens
3514	11747926_s_at	turquoise	interferon gamma inducible protein 16(IFI16)	Homo sapiens
3515	11747935_a_at	turquoise	tenascin C(TNC)	Homo sapiens
3516	11747948_a_at	turquoise	small ArfGAP2(SMAP2)	Homo sapiens
3517	11747952_x_at	turquoise	tumor necrosis factor superfamily member 10(TNFSF10)	Homo sapiens
3518	11747961_a_at	turquoise	proteasome subunit beta 8(PSMB8)	Homo sapiens
3519	11747981_a_at	turquoise	APC down-regulated 1(APCDD1)	Homo sapiens
3520	11748003_a_at	turquoise	interferon induced protein 44(IFI44)	Homo sapiens
3523	11748034_a_at	turquoise	cytidine monophospho-N-acetylneuraminic acid hydroxylase, pseudogene(CMAHP)	Homo sapiens
3524	11748088_a_at	turquoise	A-kinase anchoring protein 1(AKAP1)	Homo sapiens
3525	11748097_x_at	turquoise	poly(A) binding protein cytoplasmic 1(PABPC1)	Homo sapiens
3526	11748099_a_at	turquoise	phosphatase and actin regulator 3(PHACTR3)	Homo sapiens
3527	11748120_a_at	turquoise	lymphocyte cytosolic protein 2(LCP2)	Homo sapiens
3529	11748149_a_at	turquoise	formin binding protein 1(FNBP1)	Homo sapiens
3530	11748188_a_at	turquoise	alpha-N-acetylgalactosaminidase(NAGA)	Homo sapiens
3532	11748229_x_at	turquoise	interleukin 15 receptor subunit alpha(IL15RA)	Homo sapiens
3533	11748230_a_at	turquoise	coronin 1A(CORO1A)	Homo sapiens
3534	11748249_a_at	turquoise	NDRG family member 3(NDRG3)	Homo sapiens
3535	11748250_x_at	turquoise	NDRG family member 3(NDRG3)	Homo sapiens
3537	11748254_s_at	turquoise	male-specific lethal 3 homolog (Drosophila)(MSL3)	Homo sapiens
3539	11748280_a_at	turquoise	serpin family B member 1(SERPINB1)	Homo sapiens
3540	11748281_s_at	turquoise	cyclin and CBS domain divalent metal cation transport mediator 4(CNNM4)	Homo sapiens
3541	11748301_a_at	turquoise	transcription factor Dp-2(TFDP2)	Homo sapiens
3542	11748304_a_at	turquoise	ectonucleoside triphosphate diphosphohydrolase 6 (putative)(ENTPD6)	Homo sapiens
3544	11748332_x_at	turquoise	major histocompatibility complex, class I, E(HLA-E)	Homo sapiens
3545	11748339_a_at	turquoise	leukocyte associated immunoglobulin like receptor 1(LAIR1)	Homo sapiens
3546	11748340_x_at	turquoise	leukocyte associated immunoglobulin like receptor 1(LAIR1)	Homo sapiens
3549	11748359_s_at	turquoise	BCR, RhoGEF and GTPase activating protein(BCR)	Homo sapiens
3550	11748362_s_at	turquoise	ATP binding cassette subfamily C member 3(ABCC3)	Homo sapiens
3551	11748400_s_at	turquoise	tropomyosin 4(TPM4)	Homo sapiens
3552	11748401_x_at	turquoise	tropomyosin 4(TPM4)	Homo sapiens
3554	11748483_a_at	turquoise	purinergic receptor P2X 7(P2RX7)	Homo sapiens
3555	11748492_a_at	turquoise	layilin(LAYN)	Homo sapiens
3556	11748497_s_at	turquoise	grancalcin(GCA)	Homo sapiens
3558	11748529_x_at	turquoise	caspase 4(CASP4)	Homo sapiens
3559	11748531_x_at	turquoise	calcium channel flower domain containing 1(CACFD1)	Homo sapiens
3561	11748548_a_at	turquoise	purinergic receptor P2X 4(P2RX4)	Homo sapiens
3563	11748555_a_at	turquoise	cyclin dependent kinase like 3(CDKL3)	Homo sapiens
3564	11748582_a_at	turquoise	ADP-ribosyltransferase 3(ART3)	Homo sapiens
3566	11748604_a_at	turquoise	death associated protein kinase 2(DAPK2)	Homo sapiens
3568	11748650_a_at	turquoise	ADAM metallopeptidase domain 33(ADAM33)	Homo sapiens
3569	11748654_a_at	turquoise	protein tyrosine phosphatase, non-receptor type 22(PTPN22)	Homo sapiens
3572	11748702_x_at	turquoise	annexin A4(ANXA4)	Homo sapiens
3573	11748713_a_at	turquoise	abnormal spindle microtubule assembly(ASPM)	Homo sapiens
3574	11748747_a_at	turquoise	Rho GTPase activating protein 25(ARHGAP25)	Homo sapiens
3575	11748755_a_at	turquoise	Meis homeobox 2(MEIS2)	Homo sapiens
3576	11748775_a_at	turquoise	uridine phosphorylase 1(UPP1)	Homo sapiens
3577	11748780_a_at	turquoise	target of myb1 like 1 membrane trafficking protein(TOM1L1)	Homo sapiens
3578	11748786_a_at	turquoise	thymocyte expressed, positive selection associated 1(TESPA1)	Homo sapiens
3579	11748808_a_at	turquoise	AT-rich interaction domain 3B(ARID3B)	Homo sapiens
3581	11748841_a_at	turquoise	SAM and HD domain containing deoxynucleoside triphosphate triphosphohydrolase 1(SAMHD1)	Homo sapiens
3582	11748845_a_at	turquoise	SLAM family member 7(SLAMF7)	Homo sapiens
3584	11748859_a_at	turquoise	transcription factor EC(TFEC)	Homo sapiens

3587	11748897_a_at	turquoise	putative aquaporin-7-like protein 3(LOC100509620)	Homo sapiens
3588	11748905_a_at	turquoise	collagen triple helix repeat containing 1(CTHRC1)	Homo sapiens
3589	11748907_a_at	turquoise	retinoic acid receptor responder 3(RARRES3)	Homo sapiens
3590	11748915_a_at	turquoise	glutamic--pyruvic transaminase(GPT)	Homo sapiens
3591	11748972_a_at	turquoise	cell division cycle 25B(CDC25B)	Homo sapiens
3592	11748973_x_at	turquoise	cell division cycle 25B(CDC25B)	Homo sapiens
3593	11749002_a_at	turquoise	Rho GTPase activating protein 45(ARHGAP45)	Homo sapiens
3594	11749003_a_at	turquoise	endoplasmic reticulum aminopeptidase 1(ERAP1)	Homo sapiens
3595	11749016_a_at	turquoise	uncharacterized LOC100130460(CAND1.11)	Homo sapiens
3596	11749026_a_at	turquoise	protein tyrosine phosphatase, receptor type A(PTPRA)	Homo sapiens
3597	11749040_a_at	turquoise	phosphoglucomutase 2(PGM2)	Homo sapiens
3598	11749059_x_at	turquoise	reticulon 4(RTN4)	Homo sapiens
3599	11749092_a_at	turquoise	NLR family member X1(NLRX1)	Homo sapiens
3600	11749094_a_at	turquoise	dermatan sulfate epimerase(DSE)	Homo sapiens
3601	11749097_a_at	turquoise	ATP binding cassette subfamily A member 8(ABCA8)	Homo sapiens
3602	11749104_x_at	turquoise	Rho GTPase activating protein 45(ARHGAP45)	Homo sapiens
3603	11749121_a_at	turquoise	interferon alpha and beta receptor subunit 2(IFNAR2)	Homo sapiens
3604	11749132_a_at	turquoise	solute carrier organic anion transporter family member 2B1(SLCO2B1)	Homo sapiens
3605	11749141_a_at	turquoise	TNF receptor associated factor 5(TRAF5)	Homo sapiens
3607	11749164_a_at	turquoise	transmembrane protein 206(TMEM206)	Homo sapiens
3608	11749171_a_at	turquoise	beta-ureidopropionase 1(UPB1)	Homo sapiens
3609	11749188_a_at	turquoise	ectonucleoside triphosphate diphosphohydrolase 1(ENTPD1)	Homo sapiens
3611	11749214_a_at	turquoise	selectin P ligand(SELPLG)	Homo sapiens
3612	11749225_a_at	turquoise	butyrophilin subfamily 2 member A2(BTN2A2)	Homo sapiens
3614	11749245_a_at	turquoise	C-X-C motif chemokine ligand 11(CXCL11)	Homo sapiens
3616	11749265_a_at	turquoise	putative aquaporin-7-like protein 3(LOC100509620)	Homo sapiens
3617	11749275_a_at	turquoise	src kinase associated phosphoprotein 2(SKAP2)	Homo sapiens
3618	11749293_x_at	turquoise	membrane spanning 4-domains A6A(MS4A6A)	Homo sapiens
3619	11749309_a_at	turquoise	prune exopolyphosphatase(PRUNE1)	Homo sapiens
3620	11749310_x_at	turquoise	prune exopolyphosphatase(PRUNE1)	Homo sapiens
3622	11749326_s_at	turquoise	olfactomedin like 2B(OLFML2B)	Homo sapiens
3623	11749329_a_at	turquoise	Rho GTPase activating protein 9(ARHGAP9)	Homo sapiens
3624	11749367_a_at	turquoise	FXYD domain containing ion transport regulator 5(FXYD5)	Homo sapiens
3626	11749370_x_at	turquoise	XIAP associated factor 1(XAF1)	Homo sapiens
3627	11749377_a_at	turquoise	cat eye syndrome chromosome region, candidate 1(CECR1)	Homo sapiens
3628	11749391_x_at	turquoise	quinolinate phosphoribosyltransferase(QPRT)	Homo sapiens
3632	11749402_a_at	turquoise	integrin subunit alpha L(ITGAL)	Homo sapiens
3633	11749412_a_at	turquoise	poly(ADP-ribose) polymerase family member 14(PARP14)	Homo sapiens
3634	11749416_a_at	turquoise	small nucleolar RNA host gene 4(SNHG4)	Homo sapiens
3636	11749445_a_at	turquoise	uncharacterized LOC101928361(LOC101928361)	Homo sapiens
3637	11749492_a_at	turquoise	ribosomal protein S6 kinase A1(RPS6KA1)	Homo sapiens
3638	11749497_a_at	turquoise	DExH-box helicase 58(DHX58)	Homo sapiens
3639	11749510_a_at	turquoise	SAM and HD domain containing deoxynucleoside triphosphate triphosphohydrolase 1(SAMHD1)	Homo sapiens
3640	11749512_a_at	turquoise	signal transducer and activator of transcription 4(STAT4)	Homo sapiens
3641	11749522_a_at	turquoise	lymphocyte activating 3(LAG3)	Homo sapiens
3642	11749541_a_at	turquoise	sushi domain containing 4(SUSD4)	Homo sapiens
3643	11749550_a_at	turquoise	serine carboxypeptidase 1(SCPEP1)	Homo sapiens
3644	11749573_a_at	turquoise	arrestin beta 2(ARRB2)	Homo sapiens
3645	11749578_a_at	turquoise	COX10, heme A:farnesyltransferase cytochrome c oxidase assembly factor(COX10)	Homo sapiens
3646	11749587_x_at	turquoise	Fc fragment of IgG receptor IIa(FCGR2A)	Homo sapiens
3647	11749589_x_at	turquoise	cathepsin S(CTSS)	Homo sapiens
3648	11749595_a_at	turquoise	guanylate binding protein 4(GBP4)	Homo sapiens
3649	11749613_a_at	turquoise	OCIA domain containing 1(OCIAD1)	Homo sapiens
3650	11749625_a_at	turquoise	PQ loop repeat containing 3(PQLC3)	Homo sapiens
3651	11749660_a_at	turquoise	Mov10 RISC complex RNA helicase(MOV10)	Homo sapiens
3652	11749674_a_at	turquoise	shootin 1(SHTN1)	Homo sapiens
3653	11749676_x_at	turquoise	B-cell CLL/lymphoma 11A(BCL11A)	Homo sapiens
3656	11749708_a_at	turquoise	Fli-1 proto-oncogene, ETS transcription factor(FLI1)	Homo sapiens
3658	11749740_a_at	turquoise	ubiquitin specific peptidase 3(USP3)	Homo sapiens
3659	11749750_a_at	turquoise	WAS/WASL interacting protein family member 1(WIPF1)	Homo sapiens
3660	11749751_s_at	turquoise	WAS/WASL interacting protein family member 1(WIPF1)	Homo sapiens
3661	11749752_x_at	turquoise	WAS/WASL interacting protein family member 1(WIPF1)	Homo sapiens
3662	11749761_a_at	turquoise	PC-esterase domain containing 1A(PCED1A)	Homo sapiens
3663	11749773_x_at	turquoise	guanylate binding protein 1(GBP1)	Homo sapiens
3664	11749786_x_at	turquoise	heterogeneous nuclear ribonucleoprotein F(HNRNPF)	Homo sapiens
3665	11749788_x_at	turquoise	L-2-hydroxyglutarate dehydrogenase(L2HGDH)	Homo sapiens
3666	11749790_a_at	turquoise	caspase 8(CASP8)	Homo sapiens
3667	11749792_a_at	turquoise	PBX homeobox 1(PBX1)	Homo sapiens
3668	11749793_a_at	turquoise	annexin A4(ANXA4)	Homo sapiens
3669	11749794_x_at	turquoise	annexin A4(ANXA4)	Homo sapiens
3670	11749827_a_at	turquoise	cytochrome b-245 beta chain(CYBB)	Homo sapiens
3671	11749833_s_at	turquoise	angiotensinogen(AGT)	Homo sapiens
3672	11749844_x_at	turquoise	colony stimulating factor 2 receptor alpha subunit(CSF2RA)	Homo sapiens
3673	11749862_a_at	turquoise	apolipoprotein L3(APOL3)	Homo sapiens
3674	11749879_a_at	turquoise	post-GPI attachment to proteins 2(PGAP2)	Homo sapiens
3675	11749880_x_at	turquoise	post-GPI attachment to proteins 2(PGAP2)	Homo sapiens
3676	11749893_a_at	turquoise	carbonic anhydrase 14(CA14)	Homo sapiens
3677	11749894_a_at	turquoise	deoxycytidine kinase(DCK)	Homo sapiens
3679	11749917_a_at	turquoise	hes related family bHLH transcription factor with YRPW motif 2(HEY2)	Homo sapiens
3680	11749924_a_at	turquoise	mitochondrial ribosomal protein S25(MRPS25)	Homo sapiens
3681	11749935_s_at	turquoise	tropomyosin 3(TPM3)	Homo sapiens
3682	11749978_a_at	turquoise	major vault protein(MVP)	Homo sapiens

3683	11749990_x_at	turquoise	major histocompatibility complex, class I, A(HLA-A)	Homo sapiens
3684	11750000_a_at	turquoise	C-type lectin domain family 7 member A(CLEC7A)	Homo sapiens
3685	11750001_x_at	turquoise	C-type lectin domain family 7 member A(CLEC7A)	Homo sapiens
3689	11750045_x_at	turquoise	tropomyosin 3(TPM3)	Homo sapiens
3691	11750080_a_at	turquoise	damage specific DNA binding protein 2(DDB2)	Homo sapiens
3693	11750107_a_at	turquoise	SEL1L family member 3(SEL1L3)	Homo sapiens
3694	11750111_a_at	turquoise	vinculin(VCL)	Homo sapiens
3696	11750143_a_at	turquoise	phosphatidylinositol glycan anchor biosynthesis class Z(PIGZ)	Homo sapiens
3697	11750146_a_at	turquoise	transketolase like 1(TKTL1)	Homo sapiens
3698	11750159_a_at	turquoise	basic leucine zipper ATF-like transcription factor 2(BATF2)	Homo sapiens
3699	11750170_a_at	turquoise	tripartite motif containing 22(TRIM22)	Homo sapiens
3700	11750171_a_at	turquoise	transducin like enhancer of split 2(TLE2)	Homo sapiens
3701	11750178_a_at	turquoise	enoyl-CoA hydratase domain containing 2(ECHDC2)	Homo sapiens
3702	11750179_s_at	turquoise	enoyl-CoA hydratase domain containing 2(ECHDC2)	Homo sapiens
3703	11750185_a_at	turquoise	coiled-coil domain containing 71-like(CCDC71L)	Homo sapiens
3704	11750189_s_at	turquoise	CD44 molecule (Indian blood group)(CD44)	Homo sapiens
3705	11750190_x_at	turquoise	CD44 molecule (Indian blood group)(CD44)	Homo sapiens
3706	11750196_a_at	turquoise	annexin A4(ANXA4)	Homo sapiens
3707	11750198_a_at	turquoise	caspase 7(CASP7)	Homo sapiens
3708	11750199_a_at	turquoise	hematopoietic cell-specific Lyn substrate 1(HCLS1)	Homo sapiens
3711	11750250_a_at	turquoise	protein phosphatase 1 regulatory subunit 13 like(PPP1R13L)	Homo sapiens
3712	11750267_x_at	turquoise	inositol polyphosphate-5-phosphatase J(INPP5J)	Homo sapiens
3713	11750282_x_at	turquoise	microRNA 6837(MIR6837)	Homo sapiens
3714	11750284_a_at	turquoise	lines homolog 1(LINS1)	Homo sapiens
3716	11750301_x_at	turquoise	family with sequence similarity 92 member A(FAM92A)	Homo sapiens
3717	11750334_a_at	turquoise	REL proto-oncogene, NF-kB subunit(REL)	Homo sapiens
3718	11750359_x_at	turquoise	small nucleolar RNA, H/ACA box 67(SNORA67)	Homo sapiens
3719	11750374_a_at	turquoise	Rap guanine nucleotide exchange factor 6(RAPGEF6)	Homo sapiens
3720	11750376_a_at	turquoise	potassium calcium-activated channel subfamily M alpha 1(KCNMA1)	Homo sapiens
3721	11750408_a_at	turquoise	adhesion G protein-coupled receptor E5(ADGRE5)	Homo sapiens
3722	11750453_s_at	turquoise	parkin RBR E3 ubiquitin protein ligase(PARK2)	Homo sapiens
3723	11750454_a_at	turquoise	ETS variant 7(ETV7)	Homo sapiens
3724	11750455_x_at	turquoise	ETS variant 7(ETV7)	Homo sapiens
3725	11750527_s_at	turquoise	major histocompatibility complex, class II, DQ alpha 1(HLA-DQA1)	Homo sapiens
3726	11750528_x_at	turquoise	major histocompatibility complex, class II, DQ alpha 1(HLA-DQA1)	Homo sapiens
3727	11750531_a_at	turquoise	PYD and CARD domain containing(PYCARD)	Homo sapiens
3728	11750551_a_at	turquoise	serine/threonine kinase 4(STK4)	Homo sapiens
3729	11750552_s_at	turquoise	caspase recruitment domain family member 16(CARD16)	Homo sapiens
3730	11750553_x_at	turquoise	caspase 1(CASP1)	Homo sapiens
3731	11750575_a_at	turquoise	zinc finger protein, FOG family member 2(ZFPM2)	Homo sapiens
3732	11750585_a_at	turquoise	septin 9(SEPT9)	Homo sapiens
3734	11750598_s_at	turquoise	TPX2, microtubule nucleation factor(TPX2)	Homo sapiens
3735	11750629_a_at	turquoise	C1q and tumor necrosis factor related protein 8(C1QTNF8)	Homo sapiens
3736	11750636_a_at	turquoise	acetyl-CoA carboxylase beta(ACACB)	Homo sapiens
3737	11750674_a_at	turquoise	signal transducer and activator of transcription 3(STAT3)	Homo sapiens
3738	11750677_a_at	turquoise	family with sequence similarity 65 member C(FAM65C)	Homo sapiens
3739	11750687_a_at	turquoise	ADAM metallopeptidase domain 28(ADAM28)	Homo sapiens
3740	11750704_a_at	turquoise	sex comb on midleg homolog 1 (Drosophila)(SCMH1)	Homo sapiens
3741	11750723_x_at	turquoise	MHC class I polypeptide-related sequence B(MICB)	Homo sapiens
3742	11750740_a_at	turquoise	ST3 beta-galactoside alpha-2,3-sialyltransferase 5(ST3GAL5)	Homo sapiens
3743	11750766_a_at	turquoise	transforming acidic coiled-coil containing protein 2(TACC2)	Homo sapiens
3744	11750769_s_at	turquoise	C-C motif chemokine receptor 2(CCR2)	Homo sapiens
3745	11750773_a_at	turquoise	kinesin family member 2A(KIF2A)	Homo sapiens
3746	11750778_a_at	turquoise	macrophage scavenger receptor 1(MSR1)	Homo sapiens
3747	11750800_a_at	turquoise	ligand of numb-protein X 1(LNX1)	Homo sapiens
3748	11750811_x_at	turquoise	eomesodermin(EOMES)	Homo sapiens
3749	11750812_a_at	turquoise	Fli-1 proto-oncogene, ETS transcription factor(FLI1)	Homo sapiens
3750	11750815_s_at	turquoise	microRNA 3064(MIR3064)	Homo sapiens
3751	11750816_a_at	turquoise	CD96 molecule(CD96)	Homo sapiens
3752	11750821_x_at	turquoise	mirror-image polydactyly 1(MIPOL1)	Homo sapiens
3753	11750826_x_at	turquoise	ADAM like decysin 1(ADAMDEC1)	Homo sapiens
3754	11750847_a_at	turquoise	ankyrin repeat domain 44(ANKRD44)	Homo sapiens
3755	11750856_s_at	turquoise	C-C motif chemokine receptor 2(CCR2)	Homo sapiens
3756	11750860_a_at	turquoise	cytidine monophospho-N-acetylneuraminic acid hydroxylase, pseudogene(CMAHP)	Homo sapiens
3757	11750861_x_at	turquoise	cytidine monophospho-N-acetylneuraminic acid hydroxylase, pseudogene(CMAHP)	Homo sapiens
3758	11750894_a_at	turquoise	T-box 5(TBX5)	Homo sapiens
3760	11750932_a_at	turquoise	epoxide hydrolase 2(EPHX2)	Homo sapiens
3761	11750984_a_at	turquoise	mannosyl (alpha-1,6-)-glycoprotein beta-1,2-N-acetylglucosaminyltransferase(MGAT2)	Homo sapiens
3763	11751013_a_at	turquoise	glycine receptor beta(GLRB)	Homo sapiens
3764	11751031_a_at	turquoise	c-src tyrosine kinase(CSK)	Homo sapiens
3765	11751084_s_at	turquoise	plexin D1(PLXND1)	Homo sapiens
3766	11751096_a_at	turquoise	sorting nexin 10(SNX10)	Homo sapiens
3767	11751097_x_at	turquoise	sorting nexin 10(SNX10)	Homo sapiens
3768	11751105_s_at	turquoise	wolframin ER transmembrane glycoprotein(WFS1)	Homo sapiens
3769	11751135_a_at	turquoise	CD86 molecule(CD86)	Homo sapiens
3770	11751141_a_at	turquoise	ARP3 actin related protein 3 homolog(ACTR3)	Homo sapiens
3771	11751162_x_at	turquoise	tweety family member 1(TTYH1)	Homo sapiens
3772	11751178_a_at	turquoise	ATPase H+ transporting V1 subunit A(ATP6V1A)	Homo sapiens
3773	11751202_a_at	turquoise	chromosome 19 open reading frame 66(C19orf66)	Homo sapiens
3774	11751225_s_at	turquoise	colony stimulating factor 2 receptor alpha subunit(CSF2RA)	Homo sapiens
3775	11751242_s_at	turquoise	Fc fragment of IgG receptor IIa(FCGR2A)	Homo sapiens
3776	11751288_a_at	turquoise	lysyl oxidase like 2(LOXL2)	Homo sapiens

3777	11751299_a_at	turquoise	apolipoprotein L2(APOL2)	Homo sapiens
3778	11751304_a_at	turquoise	integrin subunit alpha L(ITGAL)	Homo sapiens
3780	11751318_s_at	turquoise	SENP3-EIF4A1 readthrough (NMD candidate)(SENP3-EIF4A1)	Homo sapiens
3781	11751319_x_at	turquoise	SENP3-EIF4A1 readthrough (NMD candidate)(SENP3-EIF4A1)	Homo sapiens
3782	11751332_a_at	turquoise	cytokine receptor like factor 3(CRLF3)	Homo sapiens
3783	11751399_x_at	turquoise	NDRG family member 3(NDRG3)	Homo sapiens
3784	11751422_a_at	turquoise	choline dehydrogenase(CHDH)	Homo sapiens
3785	11751424_a_at	turquoise	TRAF3 interacting protein 3(TRAF3IP3)	Homo sapiens
3786	11751425_x_at	turquoise	TRAF3 interacting protein 3(TRAF3IP3)	Homo sapiens
3788	11751465_a_at	turquoise	growth factor receptor bound protein 14(GRB14)	Homo sapiens
3789	11751467_a_at	turquoise	phosphatidylinositol glycan anchor biosynthesis class U(PIGU)	Homo sapiens
3790	11751482_s_at	turquoise	butyrophilin subfamily 3 member A2(BTN3A2)	Homo sapiens
3791	11751501_a_at	turquoise	fatty acid hydroxylase domain containing 2(FAXDC2)	Homo sapiens
3792	11751511_a_at	turquoise	ETS variant 7(ETV7)	Homo sapiens
3793	11751544_a_at	turquoise	myosin IF(MYO1F)	Homo sapiens
3794	11751571_x_at	turquoise	sarcoglycan alpha(SGCA)	Homo sapiens
3796	11751596_s_at	turquoise	abl interactor 1(ABI1)	Homo sapiens
3797	11751597_x_at	turquoise	abl interactor 1(ABI1)	Homo sapiens
3798	11751600_a_at	turquoise	dual adaptor of phosphotyrosine and 3-phosphoinositides 1(DAPP1)	Homo sapiens
3799	11751602_a_at	turquoise	serine and arginine rich splicing factor 7(SRSF7)	Homo sapiens
3800	11751603_x_at	turquoise	serine and arginine rich splicing factor 7(SRSF7)	Homo sapiens
3801	11751611_a_at	turquoise	TRAF-type zinc finger domain containing 1(TRAFD1)	Homo sapiens
3803	11751618_x_at	turquoise	major histocompatibility complex, class I, E(HLA-E)	Homo sapiens
3804	11751619_a_at	turquoise	apolipoprotein L1(APOL1)	Homo sapiens
3805	11751628_a_at	turquoise	transmembrane protein 140(TMEM140)	Homo sapiens
3806	11751641_x_at	turquoise	PHD finger protein 11(PHF11)	Homo sapiens
3807	11751647_a_at	turquoise	interleukin 7 receptor(IL7R)	Homo sapiens
3808	11751651_s_at	turquoise	phospholipid scramblase 1(PLSCR1)	Homo sapiens
3809	11751652_x_at	turquoise	phospholipid scramblase 1(PLSCR1)	Homo sapiens
3811	11751688_a_at	turquoise	glycoprotein integral membrane 1(GINM1)	Homo sapiens
3812	11751690_x_at	turquoise	putative aquaporin-7-like protein 3(LOC100509620)	Homo sapiens
3814	11751713_a_at	turquoise	chromosome 6 open reading frame 62(C6orf62)	Homo sapiens
3815	11751724_a_at	turquoise	zinc finger protein 542, pseudogene(ZNF542P)	Homo sapiens
3817	11751765_a_at	turquoise	ribonucleoprotein, PTB binding 2(RAVER2)	Homo sapiens
3818	11751766_a_at	turquoise	enoyl-CoA hydratase domain containing 2(ECHDC2)	Homo sapiens
3819	11751768_a_at	turquoise	sorting nexin 19(SNX19)	Homo sapiens
3822	11751782_a_at	turquoise	EF-hand domain containing 2(EFHC2)	Homo sapiens
3823	11751799_a_at	turquoise	peroxiredoxin 6(PRDX6)	Homo sapiens
3824	11751805_a_at	turquoise	thymidylate synthetase(TYMS)	Homo sapiens
3825	11751821_a_at	turquoise	cytochrome b-245 beta chain(CYBB)	Homo sapiens
3826	11751833_a_at	turquoise	butyrylcholinesterase(BCHE)	Homo sapiens
3827	11751857_a_at	turquoise	granulysin(GNLY)	Homo sapiens
3829	11751888_x_at	turquoise	collagen type IV alpha 6 chain(COL4A6)	Homo sapiens
3830	11751890_a_at	turquoise	sodium voltage-gated channel alpha subunit 5(SCN5A)	Homo sapiens
3831	11751893_a_at	turquoise	collagen type IV alpha 6 chain(COL4A6)	Homo sapiens
3832	11751917_a_at	turquoise	myotubularin related protein 12(MTMR12)	Homo sapiens
3833	11751921_s_at	turquoise	aryl hydrocarbon receptor(AHR)	Homo sapiens
3834	11752003_a_at	turquoise	IQ motif containing GTPase activating protein 1(IQGAP1)	Homo sapiens
3835	11752009_a_at	turquoise	tenascin C(TNC)	Homo sapiens
3836	11752036_s_at	turquoise	transmembrane protein 123(TMEM123)	Homo sapiens
3837	11752051_a_at	turquoise	myosin heavy chain 9(MYH9)	Homo sapiens
3838	11752052_x_at	turquoise	myosin heavy chain 9(MYH9)	Homo sapiens
3839	11752065_a_at	turquoise	CD6 molecule(CD6)	Homo sapiens
3840	11752095_a_at	turquoise	protein tyrosine phosphatase, receptor type C(PTPRC)	Homo sapiens
3841	11752142_a_at	turquoise	filamin A(FLNA)	Homo sapiens
3842	11752149_a_at	turquoise	ubiquitin associated and SH3 domain containing A(UBASH3A)	Homo sapiens
3843	11752153_a_at	turquoise	neurobeachin like 2(NBEAL2)	Homo sapiens
3844	11752154_x_at	turquoise	neurobeachin like 2(NBEAL2)	Homo sapiens
3845	11752163_a_at	turquoise	lysine demethylase 2B(KDM2B)	Homo sapiens
3846	11752164_x_at	turquoise	lysine demethylase 2B(KDM2B)	Homo sapiens
3847	11752171_s_at	turquoise	serine palmitoyltransferase long chain base subunit 1(SPTLC1)	Homo sapiens
3848	11752173_a_at	turquoise	transmembrane protein 2(TMEM2)	Homo sapiens
3849	11752175_x_at	turquoise	DBF4 zinc finger(DBF4)	Homo sapiens
3850	11752180_x_at	turquoise	von Hippel-Lindau tumor suppressor(VHL)	Homo sapiens
3851	11752224_a_at	turquoise	schlafen family member 5(SLFN5)	Homo sapiens
3852	11752233_a_at	turquoise	copine 4(CPNE4)	Homo sapiens
3853	11752235_x_at	turquoise	inhibitor of kappa light polypeptide gene enhancer in B-cells, kinase beta(IKBKB)	Homo sapiens
3854	11752265_a_at	turquoise	androgen receptor(AR)	Homo sapiens
3855	11752267_a_at	turquoise	ribosomal protein S6 kinase A4(RPS6KA4)	Homo sapiens
3856	11752273_a_at	turquoise	colony stimulating factor 1 receptor(CSF1R)	Homo sapiens
3857	11752278_x_at	turquoise	poly(A) binding protein cytoplasmic 1(PABPC1)	Homo sapiens
3858	11752297_x_at	turquoise	class II major histocompatibility complex transactivator(CIITA)	Homo sapiens
3859	11752318_a_at	turquoise	butyrophilin subfamily 3 member A3(BTN3A3)	Homo sapiens
3860	11752331_s_at	turquoise	SLX1A-SULT1A3 readthrough (NMD candidate)(SLX1A-SULT1A3)	Homo sapiens
3861	11752341_a_at	turquoise	coagulation factor VIII(F8)	Homo sapiens
3862	11752379_a_at	turquoise	multiple C2 and transmembrane domain containing 1(MCTP1)	Homo sapiens
3863	11752387_a_at	turquoise	ETS proto-oncogene 1, transcription factor(ETS1)	Homo sapiens
3864	11752398_a_at	turquoise	chromosome 1 open reading frame 228(C1orf228)	Homo sapiens
3865	11752411_a_at	turquoise	coiled-coil domain containing 8(CCDC8)	Homo sapiens
3866	11752467_s_at	turquoise	inositol polyphosphate-5-phosphatase D(INPP5D)	Homo sapiens
3867	11752482_a_at	turquoise	fibrillin 2(FBN2)	Homo sapiens
3868	11752535_a_at	turquoise	cyclin L1(CCNL1)	Homo sapiens

3869	11752568_a_at	turquoise	LCK proto-oncogene, Src family tyrosine kinase(LCK)	Homo sapiens
3870	11752569_x_at	turquoise	LCK proto-oncogene, Src family tyrosine kinase(LCK)	Homo sapiens
3871	11752606_x_at	turquoise	microRNA 6845(MIR6845)	Homo sapiens
3872	11752607_a_at	turquoise	microRNA 6132(MIR6132)	Homo sapiens
3873	11752608_x_at	turquoise	microRNA 6132(MIR6132)	Homo sapiens
3874	11752610_a_at	turquoise	thymocyte selection associated family member 2(THEMIS2)	Homo sapiens
3875	11752611_s_at	turquoise	signal sequence receptor subunit 1(SSR1)	Homo sapiens
3876	11752631_a_at	turquoise	SAM and SH3 domain containing 3(SASH3)	Homo sapiens
3877	11752635_x_at	turquoise	potassium voltage-gated channel subfamily H member 2(KCNH2)	Homo sapiens
3878	11752644_a_at	turquoise	TIA1 cytotoxic granule associated RNA binding protein like 1(TIAL1)	Homo sapiens
3880	11752697_a_at	turquoise	PC-esterase domain containing 1A(PCED1A)	Homo sapiens
3881	11752698_x_at	turquoise	PC-esterase domain containing 1A(PCED1A)	Homo sapiens
3882	11752723_a_at	turquoise	CD200 receptor 1(CD200R1)	Homo sapiens
3883	11752732_x_at	turquoise	dihydrouridine synthase 4 like(DUS4L)	Homo sapiens
3884	11752737_a_at	turquoise	C-type lectin domain family 7 member A(CLEC7A)	Homo sapiens
3887	11752769_a_at	turquoise	piggyBac transposable element derived 5(PGBD5)	Homo sapiens
3888	11752771_a_at	turquoise	carbohydrate sulfotransferase 15(CHST15)	Homo sapiens
3889	11752794_a_at	turquoise	signal transducer and activator of transcription 2(STAT2)	Homo sapiens
3890	11752795_x_at	turquoise	signal transducer and activator of transcription 2(STAT2)	Homo sapiens
3891	11752796_x_at	turquoise	chromosome 3 open reading frame 33(C3orf33)	Homo sapiens
3892	11752801_a_at	turquoise	SP110 nuclear body protein(SP110)	Homo sapiens
3893	11752802_x_at	turquoise	SP110 nuclear body protein(SP110)	Homo sapiens
3894	11752812_x_at	turquoise	tweety family member 1(TTYH1)	Homo sapiens
3895	11752814_s_at	turquoise	TNF receptor superfamily member 1A(TNFRSF1A)	Homo sapiens
3896	11752817_s_at	turquoise	tripeptidyl peptidase 1(TPP1)	Homo sapiens
3897	11752833_x_at	turquoise	post-GPI attachment to proteins 2(PGAP2)	Homo sapiens
3898	11752865_x_at	turquoise	actin beta(ACTB)	Homo sapiens
3899	11752873_a_at	turquoise	src kinase associated phosphoprotein 1(SKAP1)	Homo sapiens
3900	11752877_x_at	turquoise	tubulin beta class I(TUBB)	Homo sapiens
3901	11752890_a_at	turquoise	syntrophin alpha 1(SNTA1)	Homo sapiens
3902	11752898_a_at	turquoise	peptidylprolyl isomerase like 6(PPIL6)	Homo sapiens
3903	11752910_a_at	turquoise	ADP ribosylation factor like GTPase 6 interacting protein 1(ARL6IP1)	Homo sapiens
3905	11752918_a_at	turquoise	Fc receptor like 6(FCRL6)	Homo sapiens
3907	11752930_a_at	turquoise	guanylate binding protein 1(GBP1)	Homo sapiens
3908	11752931_x_at	turquoise	guanylate binding protein 1(GBP1)	Homo sapiens
3909	11752948_a_at	turquoise	prolylcarboxypeptidase(PRCP)	Homo sapiens
3910	11752959_x_at	turquoise	actin beta(ACTB)	Homo sapiens
3912	11752987_a_at	turquoise	EPN2 intronic transcript 1(EPN2-IT1)	Homo sapiens
3913	11753025_a_at	turquoise	TNF receptor superfamily member 1B(TNFRSF1B)	Homo sapiens
3914	11753034_a_at	turquoise	zinc finger NFX1-type containing 1(ZNFX1)	Homo sapiens
3915	11753055_s_at	turquoise	RAP1B, member of RAS oncogene family(RAP1B)	Homo sapiens
3916	11753056_a_at	turquoise	zinc finger protein 667(ZNF667)	Homo sapiens
3917	11753061_a_at	turquoise	schlafen family member 5(SLFN5)	Homo sapiens
3918	11753088_a_at	turquoise	multiple C2 and transmembrane domain containing 1(MCTP1)	Homo sapiens
3919	11753090_x_at	turquoise	opioid growth factor receptor(OGFR)	Homo sapiens
3920	11753106_a_at	turquoise	target of myb1 like 2 membrane trafficking protein(TOM1L2)	Homo sapiens
3921	11753111_x_at	turquoise	interleukin 10 receptor subunit beta(IL10RB)	Homo sapiens
3922	11753129_a_at	turquoise	transmembrane 4 L six family member 1(TM4SF1)	Homo sapiens
3923	11753132_x_at	turquoise	solute carrier family 50 member 1(SLC50A1)	Homo sapiens
3924	11753151_a_at	turquoise	histone deacetylase 8(HDAC8)	Homo sapiens
3925	11753165_a_at	turquoise	RAB27A, member RAS oncogene family(RAB27A)	Homo sapiens
3926	11753213_x_at	turquoise	G protein subunit alpha i2(GNAI2)	Homo sapiens
3927	11753219_a_at	turquoise	killer cell lectin like receptor F1(KLRF1)	Homo sapiens
3928	11753239_a_at	turquoise	scavenger receptor class A member 5(SCARA5)	Homo sapiens
3929	11753241_s_at	turquoise	C-type lectin domain family 12 member A(CLEC12A)	Homo sapiens
3930	11753266_a_at	turquoise	transcription elongation factor A3(TCEA3)	Homo sapiens
3931	11753291_a_at	turquoise	CD44 molecule (Indian blood group)(CD44)	Homo sapiens
3932	11753292_x_at	turquoise	CD44 molecule (Indian blood group)(CD44)	Homo sapiens
3933	11753312_a_at	turquoise	DnaJ heat shock protein family (Hsp40) member C5 gamma(DNAJC5G)	Homo sapiens
3934	11753321_a_at	turquoise	TNFSF12-TNFSF13 readthrough(TNFSF12-TNFSF13)	Homo sapiens
3936	11753334_x_at	turquoise	BCL2 associated athanogene 1(BAG1)	Homo sapiens
3937	11753335_a_at	turquoise	transmembrane protein 177(TMEM177)	Homo sapiens
3939	11753348_a_at	turquoise	SAM domain, SH3 domain and nuclear localization signals 1(SAMSN1)	Homo sapiens
3940	11753399_x_at	turquoise	annexin A2(ANXA2)	Homo sapiens
3941	11753404_x_at	turquoise	major histocompatibility complex, class I, E(HLA-E)	Homo sapiens
3942	11753445_a_at	turquoise	heme oxygenase 1(HMOX1)	Homo sapiens
3943	11753446_x_at	turquoise	heme oxygenase 1(HMOX1)	Homo sapiens
3946	11753484_x_at	turquoise	killer cell lectin like receptor D1(KLRD1)	Homo sapiens
3948	11753515_a_at	turquoise	interleukin 32(IL32)	Homo sapiens
3949	11753519_a_at	turquoise	gamma-secretase activating protein(GSAP)	Homo sapiens
3950	11753521_a_at	turquoise	RAB5C, member RAS oncogene family(RAB5C)	Homo sapiens
3951	11753534_a_at	turquoise	killer cell lectin like receptor D1(KLRD1)	Homo sapiens
3952	11753535_x_at	turquoise	killer cell lectin like receptor D1(KLRD1)	Homo sapiens
3953	11753549_a_at	turquoise	CKLF like MARVEL transmembrane domain containing 3(CMTM3)	Homo sapiens
3954	11753555_a_at	turquoise	CD53 molecule(CD53)	Homo sapiens
3955	11753569_a_at	turquoise	leucine zipper tumor suppressor 1(LZTS1)	Homo sapiens
3956	11753575_at	turquoise	coenzyme Q7, hydroxylase(COQ7)	Homo sapiens
3957	11753623_s_at	turquoise	RAP1B, member of RAS oncogene family(RAP1B)	Homo sapiens
3959	11753675_x_at	turquoise	profilin 2(PFN2)	Homo sapiens
3960	11753722_x_at	turquoise	profilin 1(PFN1)	Homo sapiens
3961	11753727_x_at	turquoise	microRNA 6805(MIR6805)	Homo sapiens
3962	11753732_a_at	turquoise	polyamine oxidase(PAOX)	Homo sapiens

3963	11753735_x_at	turquoise	thymosin beta 4, X-linked(TMSB4X)	Homo sapiens
3965	11753776_x_at	turquoise	leukocyte associated immunoglobulin like receptor 2(LAIR2)	Homo sapiens
3969	11753810_a_at	turquoise	C-C motif chemokine ligand 5(CCL5)	Homo sapiens
3973	11753856_a_at	turquoise	CDC28 protein kinase regulatory subunit 2(CKS2)	Homo sapiens
3974	11753858_a_at	turquoise	small nucleolar RNA, H/ACA box 44(SNORA44)	Homo sapiens
3975	11753859_x_at	turquoise	small nucleolar RNA, H/ACA box 44(SNORA44)	Homo sapiens
3976	11753868_x_at	turquoise	leukocyte specific transcript 1(LST1)	Homo sapiens
3979	11753882_a_at	turquoise	interleukin 2 receptor subunit gamma(IL2RG)	Homo sapiens
3980	11753883_x_at	turquoise	interleukin 2 receptor subunit gamma(IL2RG)	Homo sapiens
3982	11753898_x_at	turquoise	major histocompatibility complex, class II, DQ alpha 1(HLA-DQA1)	Homo sapiens
3983	11753902_a_at	turquoise	actinin alpha 4(ACTN4)	Homo sapiens
3984	11753911_s_at	turquoise	transmembrane protein 242(TMEM242)	Homo sapiens
3985	11753915_a_at	turquoise	SEL1L family member 3(SEL1L3)	Homo sapiens
3986	11753918_a_at	turquoise	nucleoporin 85(NUP85)	Homo sapiens
3987	11753920_a_at	turquoise	apolipoprotein L1(APOL1)	Homo sapiens
3988	11753923_at	turquoise	MBNL1 antisense RNA 1(MBNL1-AS1)	Homo sapiens
3989	11753929_x_at	turquoise	ST3 beta-galactoside alpha-2,3-sialyltransferase 2(ST3GAL2)	Homo sapiens
3990	11753936_x_at	turquoise	transient receptor potential cation channel subfamily M member 2(TRPM2)	Homo sapiens
3991	11753939_x_at	turquoise	proteasome subunit beta 10(PSMB10)	Homo sapiens
3992	11753988_a_at	turquoise	sprouty RTK signaling antagonist 2(SPRY2)	Homo sapiens
4001	11754035_a_at	turquoise	interferon regulatory factor 1(IRF1)	Homo sapiens
4002	11754064_x_at	turquoise	annexin A2(ANXA2)	Homo sapiens
4003	11754071_x_at	turquoise	transgelin 2(TAGLN2)	Homo sapiens
4004	11754105_x_at	turquoise	small nuclear ribonucleoprotein polypeptides B and B1(SNRPB)	Homo sapiens
4005	11754124_a_at	turquoise	coactosin like F-actin binding protein 1(COTL1)	Homo sapiens
4006	11754125_x_at	turquoise	coactosin like F-actin binding protein 1(COTL1)	Homo sapiens
4007	11754142_x_at	turquoise	microRNA 1244-1(MIR1244-1)	Homo sapiens
4009	11754153_x_at	turquoise	caspase 8(CASP8)	Homo sapiens
4011	11754170_a_at	turquoise	transforming growth factor beta regulator 1(TBRG1)	Homo sapiens
4012	11754181_a_at	turquoise	interleukin 2 receptor subunit gamma(IL2RG)	Homo sapiens
4013	11754182_x_at	turquoise	interleukin 2 receptor subunit gamma(IL2RG)	Homo sapiens
4014	11754202_a_at	turquoise	capping actin protein, gelsolin like(CAPG)	Homo sapiens
4015	11754204_a_at	turquoise	peroxisomal biogenesis factor 11 alpha(PEX11A)	Homo sapiens
4016	11754205_a_at	turquoise	lysine acetyltransferase 6B(KAT6B)	Homo sapiens
4017	11754217_x_at	turquoise	actin beta(ACTB)	Homo sapiens
4018	11754219_a_at	turquoise	signal induced proliferation associated 1 like 1(SIPA1L1)	Homo sapiens
4019	11754223_at	turquoise	transmembrane protein 35B(TMEM35B)	Homo sapiens
4020	11754224_x_at	turquoise	RAS p21 protein activator 4B(RASA4B)	Homo sapiens
4021	11754225_a_at	turquoise	Sec23 homolog B, coat complex II component(SEC23B)	Homo sapiens
4022	11754243_a_at	turquoise	H2A histone family member X(H2AFX)	Homo sapiens
4023	11754246_x_at	turquoise	poly(A) binding protein cytoplasmic 1(PABPC1)	Homo sapiens
4024	11754258_a_at	turquoise	lipoic acid synthetase(LIAS)	Homo sapiens
4025	11754266_s_at	turquoise	E2F transcription factor 3(E2F3)	Homo sapiens
4026	11754270_a_at	turquoise	complement component 4B (Chido blood group), copy 2(C4B_2)	Homo sapiens
4028	11754278_x_at	turquoise	cyclin G associated kinase(GAK)	Homo sapiens
4029	11754295_a_at	turquoise	family with sequence similarity 107 member B(FAM107B)	Homo sapiens
4030	11754297_s_at	turquoise	TNF receptor superfamily member 1A(TNFRSF1A)	Homo sapiens
4031	11754298_x_at	turquoise	casein kinase 1 delta(CSNK1D)	Homo sapiens
4032	11754313_s_at	turquoise	ADP ribosylation factor like GTPase 4C(ARL4C)	Homo sapiens
4033	11754315_x_at	turquoise	thymosin beta 4, X-linked(TMSB4X)	Homo sapiens
4034	11754317_a_at	turquoise	annexin A4(ANXA4)	Homo sapiens
4035	11754320_a_at	turquoise	structural maintenance of chromosomes 4(SMC4)	Homo sapiens
4036	11754321_x_at	turquoise	solute carrier family 12 member 9(SLC12A9)	Homo sapiens
4037	11754330_a_at	turquoise	interferon regulatory factor 2(IRF2)	Homo sapiens
4038	11754339_a_at	turquoise	NAD synthetase 1(NADSYN1)	Homo sapiens
4039	11754348_s_at	turquoise	kelch domain containing 4(KLHDC4)	Homo sapiens
4040	11754351_a_at	turquoise	cytohesin 1(CYTH1)	Homo sapiens
4041	11754352_a_at	turquoise	ring finger protein 213(RNF213)	Homo sapiens
4042	11754358_x_at	turquoise	small nucleolar RNA host gene 4(SNHG4)	Homo sapiens
4043	11754360_a_at	turquoise	ribonucleotide reductase regulatory subunit M2(RRM2)	Homo sapiens
4044	11754370_x_at	turquoise	synaptotagmin like 1(SYTL1)	Homo sapiens
4045	11754375_s_at	turquoise	sorting nexin 10(SNX10)	Homo sapiens
4046	11754379_at	turquoise	poly(ADP-ribose) polymerase family member 9(PARP9)	Homo sapiens
4048	11754382_a_at	turquoise	small integral membrane protein 5(SMIM5)	Homo sapiens
4049	11754403_x_at	turquoise	poly(ADP-ribose) polymerase family member 14(PARP14)	Homo sapiens
4050	11754404_a_at	turquoise	poly(ADP-ribose) polymerase family member 14(PARP14)	Homo sapiens
4051	11754419_a_at	turquoise	nucleolus and neural progenitor protein(NEPRO)	Homo sapiens
4052	11754434_a_at	turquoise	cyclin D3(CCND3)	Homo sapiens
4054	11754454_s_at	turquoise	LDL receptor related protein 10(LRP10)	Homo sapiens
4055	11754461_s_at	turquoise	uncoupling protein 2(UCP2)	Homo sapiens
4056	11754471_a_at	turquoise	major histocompatibility complex, class II, DM beta(HLA-DMB)	Homo sapiens
4057	11754474_a_at	turquoise	pleckstrin(PLEK)	Homo sapiens
4058	11754481_x_at	turquoise	coronin 7(CORO7)	Homo sapiens
4060	11754491_a_at	turquoise	chromosome 16 open reading frame 46(C16orf46)	Homo sapiens
4061	11754492_x_at	turquoise	phospholipase C beta 2(PLCB2)	Homo sapiens
4062	11754503_a_at	turquoise	choline/ethanolamine phosphotransferase 1(CEPT1)	Homo sapiens
4064	11754524_x_at	turquoise	tissue factor pathway inhibitor 2(TFPI2)	Homo sapiens
4065	11754551_x_at	turquoise	enolase 1(ENO1)	Homo sapiens
4066	11754554_x_at	turquoise	enoyl-CoA hydratase domain containing 2(ECHDC2)	Homo sapiens
4067	11754579_at	turquoise	pappalysin 1(PAPPA)	Homo sapiens
4068	11754584_a_at	turquoise	RAD9-HUS1-RAD1 interacting nuclear orphan 1(RHNO1)	Homo sapiens
4069	11754587_a_at	turquoise	FYN proto-oncogene, Src family tyrosine kinase(FYN)	Homo sapiens

4070	11754589_a_at	turquoise	exportin 6(XPO6)	Homo sapiens
4071	11754592_x_at	turquoise	activating transcription factor 5(ATF5)	Homo sapiens
4072	11754593_x_at	turquoise	tubulin beta 3 class III(TUBB3)	Homo sapiens
4074	11754603_a_at	turquoise	dual specificity phosphatase 18(DUSP18)	Homo sapiens
4075	11754609_x_at	turquoise	stathmin 3(STMN3)	Homo sapiens
4076	11754615_x_at	turquoise	ubiquitin fold modifier 1(UFM1)	Homo sapiens
4078	11754627_s_at	turquoise	RAB37, member RAS oncogene family(RAB37)	Homo sapiens
4079	11754630_a_at	turquoise	mixed lineage kinase domain like(MLKL)	Homo sapiens
4080	11754637_a_at	turquoise	synaptotagmin like 1(SYTL1)	Homo sapiens
4081	11754644_x_at	turquoise	calponin 2(CNN2)	Homo sapiens
4082	11754649_s_at	turquoise	IL2 inducible T-cell kinase(ITK)	Homo sapiens
4084	11754666_a_at	turquoise	coronin 1A(CORO1A)	Homo sapiens
4085	11754672_a_at	turquoise	checkpoint with forkhead and ring finger domains(CHFR)	Homo sapiens
4086	11754678_a_at	turquoise	solute carrier family 25 member 43(SLC25A43)	Homo sapiens
4087	11754680_a_at	turquoise	mitogen-activated protein kinase kinase kinase 2(MAP3K2)	Homo sapiens
4088	11754681_s_at	turquoise	major histocompatibility complex, class II, DM beta(HLA-DMB)	Homo sapiens
4089	11754695_a_at	turquoise	phospholipase A2 group V(PLA2G5)	Homo sapiens
4091	11754712_a_at	turquoise	TBC1 domain family member 10A(TBC1D10A)	Homo sapiens
4092	11754716_s_at	turquoise	GATA zinc finger domain containing 2A(GATAD2A)	Homo sapiens
4093	11754722_s_at	turquoise	SEPSECS antisense RNA 1 (head to head)(SEPSECS-AS1)	Homo sapiens
4094	11754724_a_at	turquoise	coiled-coil domain containing 88B(CCDC88B)	Homo sapiens
4095	11754751_x_at	turquoise	BCL2 associated athanogene 1(BAG1)	Homo sapiens
4096	11754752_a_at	turquoise	outer dense fiber of sperm tails 3B(ODF3B)	Homo sapiens
4097	11754759_a_at	turquoise	chromatin target of PRMT1(CHTOP)	Homo sapiens
4098	11754760_a_at	turquoise	GRAM domain containing 1A(GRAMD1A)	Homo sapiens
4099	11754761_x_at	turquoise	high mobility group nucleosome binding domain 1(HMGN1)	Homo sapiens
4100	11754762_x_at	turquoise	T-cell immune regulator 1, ATPase H+ transporting V0 subunit a3(TCIRG1)	Homo sapiens
4101	11754765_s_at	turquoise	microRNA 6080(MIR6080)	Homo sapiens
4102	11754769_a_at	turquoise	BEN domain containing 5(BEND5)	Homo sapiens
4103	11754777_a_at	turquoise	SH2 domain containing 2A(SH2D2A)	Homo sapiens
4105	11754798_s_at	turquoise	CD164 molecule(CD164)	Homo sapiens
4106	11754802_s_at	turquoise	chromosome 12 open reading frame 76(C12orf76)	Homo sapiens
4107	11754808_s_at	turquoise	C3 and PZP like, alpha-2-macroglobulin domain containing 8(CPAMD8)	Homo sapiens
4108	11754814_a_at	turquoise	nucleoporin 93(NUP93)	Homo sapiens
4109	11754822_x_at	turquoise	tyrosyl-DNA phosphodiesterase 1(TDP1)	Homo sapiens
4110	11754828_a_at	turquoise	butyrophilin subfamily 2 member A2(BTN2A2)	Homo sapiens
4111	11754833_a_at	turquoise	toll like receptor 2(TLR2)	Homo sapiens
4112	11754835_a_at	turquoise	immunoglobin superfamily member 21(IGSF21)	Homo sapiens
4113	11754839_x_at	turquoise	ubiquitin specific peptidase 54(USP54)	Homo sapiens
4114	11754842_x_at	turquoise	meiotic double-stranded break formation protein 1(MEI1)	Homo sapiens
4116	11754859_x_at	turquoise	RUN and cysteine rich domain containing beclin 1 interacting protein like(RUBCNL)	Homo sapiens
4118	11754869_s_at	turquoise	zinc finger protein 267(ZNF267)	Homo sapiens
4119	11754870_x_at	turquoise	Mab-21 domain containing 1(MB21D1)	Homo sapiens
4120	11754881_a_at	turquoise	signal regulatory protein gamma(SIRPG)	Homo sapiens
4121	11754884_s_at	turquoise	WD repeat and FYVE domain containing 1(WDFY1)	Homo sapiens
4122	11754887_a_at	turquoise	methionine sulfoxide reductase B3(MSRB3)	Homo sapiens
4124	11754910_x_at	turquoise	MICAL like 2(MICALL2)	Homo sapiens
4125	11754917_a_at	turquoise	SP100 nuclear antigen(SP100)	Homo sapiens
4126	11754920_s_at	turquoise	vesicular, overexpressed in cancer, prosurvival protein 1(VOPP1)	Homo sapiens
4127	11754925_a_at	turquoise	spleen associated tyrosine kinase(SYK)	Homo sapiens
4128	11754942_x_at	turquoise	chromosome 17 open reading frame 62(C17orf62)	Homo sapiens
4129	11754945_x_at	turquoise	transketolase(TKT)	Homo sapiens
4130	11754946_a_at	turquoise	chromosome 7 open reading frame 49(C7orf49)	Homo sapiens
4131	11754951_a_at	turquoise	nuclear receptor coactivator 7(NCOA7)	Homo sapiens
4133	11754972_s_at	turquoise	bromodomain adjacent to zinc finger domain 2A(BAZ2A)	Homo sapiens
4134	11754976_x_at	turquoise	canopy FGF signaling regulator 3(CNPY3)	Homo sapiens
4135	11754989_a_at	turquoise	Dmx like 2(DMXL2)	Homo sapiens
4136	11754992_a_at	turquoise	chromodomain helicase DNA binding protein 1(CHD1)	Homo sapiens
4137	11754998_a_at	turquoise	multiple C2 and transmembrane domain containing 1(MCTP1)	Homo sapiens
4138	11755010_s_at	turquoise	POC1B-GALNT4 readthrough(POC1B-GALNT4)	Homo sapiens
4139	11755012_a_at	turquoise	CDC42 small effector 2(CDC42SE2)	Homo sapiens
4140	11755013_x_at	turquoise	TNF receptor superfamily member 1B(TNFRSF1B)	Homo sapiens
4142	11755022_a_at	turquoise	ADP ribosylation factor like GTPase 6 interacting protein 1(ARL6IP1)	Homo sapiens
4143	11755032_s_at	turquoise	ArfGAP with SH3 domain, ankyrin repeat and PH domain 1(ASAP1)	Homo sapiens
4144	11755036_a_at	turquoise	2',3'-cyclic nucleotide 3' phosphodiesterase(CNP)	Homo sapiens
4145	11755043_a_at	turquoise	gamma-secretase activating protein(GSAP)	Homo sapiens
4146	11755044_x_at	turquoise	gamma-secretase activating protein(GSAP)	Homo sapiens
4147	11755045_s_at	turquoise	ankyrin repeat domain 13A(ANKRD13A)	Homo sapiens
4148	11755054_x_at	turquoise	runt related transcription factor 3(RUNX3)	Homo sapiens
4149	11755058_a_at	turquoise	bromodomain adjacent to zinc finger domain 1A(BAZ1A)	Homo sapiens
4150	11755059_s_at	turquoise	transcription factor 3(TCF3)	Homo sapiens
4151	11755070_a_at	turquoise	caspase recruitment domain family member 8(CARD8)	Homo sapiens
4152	11755075_s_at	turquoise	NAD synthetase 1(NADSYN1)	Homo sapiens
4153	11755076_x_at	turquoise	ORAI calcium release-activated calcium modulator 2(ORAI2)	Homo sapiens
4154	11755078_a_at	turquoise	transcriptional adaptor 2B(TADA2B)	Homo sapiens
4155	11755091_a_at	turquoise	ATP binding cassette subfamily A member 7(ABCA7)	Homo sapiens
4156	11755105_a_at	turquoise	diacylglycerol kinase zeta(DGKZ)	Homo sapiens
4157	11755116_x_at	turquoise	abhydrolase domain containing 11(ABHD11)	Homo sapiens
4158	11755117_x_at	turquoise	F-box and leucine rich repeat protein 22(FBXL22)	Homo sapiens
4159	11755121_x_at	turquoise	organic solute carrier partner 1(OSCP1)	Homo sapiens
4160	11755135_a_at	turquoise	abhydrolase domain containing 1(ABHD1)	Homo sapiens
4161	11755137_a_at	turquoise	mitochondrial calcium uniporter dominant negative beta subunit(MCUB)	Homo sapiens

4162	11755147_s_at	turquoise	signal transducer and activator of transcription 2(STAT2)	Homo sapiens
4163	11755148_a_at	turquoise	Dmx like 2(DMXL2)	Homo sapiens
4164	11755151_a_at	turquoise	aryl hydrocarbon receptor nuclear translocator like(ARNTL)	Homo sapiens
4165	11755175_a_at	turquoise	heat shock protein family A (Hsp70) member 12A(HSPA12A)	Homo sapiens
4166	11755180_x_at	turquoise	transcription factor 7 (T-cell specific, HMG-box)(TCF7)	Homo sapiens
4167	11755222_a_at	turquoise	G protein subunit alpha 13(GNA13)	Homo sapiens
4168	11755231_a_at	turquoise	LCK proto-oncogene, Src family tyrosine kinase(LCK)	Homo sapiens
4169	11755235_a_at	turquoise	fatty acyl-CoA reductase 2(FAR2)	Homo sapiens
4170	11755237_a_at	turquoise	protein phosphatase, Mg2+/Mn2+ dependent 1M(PPM1M)	Homo sapiens
4171	11755246_x_at	turquoise	growth regulation by estrogen in breast cancer 1(GREB1)	Homo sapiens
4172	11755247_x_at	turquoise	DAN domain BMP antagonist family member 5(DAND5)	Homo sapiens
4173	11755265_s_at	turquoise	5'-nucleotidase domain containing 1(NT5DC1)	Homo sapiens
4176	11755279_x_at	turquoise	major histocompatibility complex, class II, DP beta 1(HLA-DPB1)	Homo sapiens
4177	11755280_x_at	turquoise	ferritin light chain(FTL)	Homo sapiens
4178	11755281_a_at	turquoise	transforming growth factor beta induced(TGFBI)	Homo sapiens
4179	11755291_a_at	turquoise	DENN domain containing 2D(DENND2D)	Homo sapiens
4180	11755292_a_at	turquoise	cytochrome b561 family member A3(CYB561A3)	Homo sapiens
4181	11755303_x_at	turquoise	major histocompatibility complex, class I, A(HLA-A)	Homo sapiens
4182	11755315_a_at	turquoise	arginyl aminopeptidase(RNPEP)	Homo sapiens
4183	11755319_a_at	turquoise	ankyrin repeat domain 13D(ANKRD13D)	Homo sapiens
4184	11755320_x_at	turquoise	ankyrin repeat domain 13D(ANKRD13D)	Homo sapiens
4185	11755326_x_at	turquoise	abhydrolase domain containing 16A(ABHD16A)	Homo sapiens
4186	11755327_s_at	turquoise	family with sequence similarity 115, member C pseudogene(LOC154761)	Homo sapiens
4187	11755331_a_at	turquoise	ankyrin 2(ANK2)	Homo sapiens
4188	11755342_x_at	turquoise	microRNA 1292(MIR1292)	Homo sapiens
4189	11755343_s_at	turquoise	SET domain and mariner transposase fusion gene(SETMAR)	Homo sapiens
4190	11755344_x_at	turquoise	major histocompatibility complex, class I, A(HLA-A)	Homo sapiens
4193	11755355_s_at	turquoise	RCSD domain containing 1(RCSD1)	Homo sapiens
4194	11755357_s_at	turquoise	aconitase 1(ACO1)	Homo sapiens
4195	11755361_a_at	turquoise	de-etiolated homolog 1 (Arabidopsis)(DET1)	Homo sapiens
4196	11755373_x_at	turquoise	enoyl-CoA hydratase domain containing 2(ECHDC2)	Homo sapiens
4197	11755374_a_at	turquoise	HECT and RLD domain containing E3 ubiquitin protein ligase 5(HERC5)	Homo sapiens
4199	11755387_a_at	turquoise	SAC1 suppressor of actin mutations 1-like (yeast)(SACM1L)	Homo sapiens
4200	11755391_x_at	turquoise	H2A histone family member Y(H2AFY)	Homo sapiens
4201	11755405_a_at	turquoise	RUN and cysteine rich domain containing beclin 1 interacting protein like(RUBCNL)	Homo sapiens
4202	11755411_a_at	turquoise	inhibitor of kappa light polypeptide gene enhancer in B-cells, kinase beta(IKBKB)	Homo sapiens
4203	11755412_x_at	turquoise	inhibitor of kappa light polypeptide gene enhancer in B-cells, kinase beta(IKBKB)	Homo sapiens
4204	11755417_a_at	turquoise	KIAA0922(KIAA0922)	Homo sapiens
4205	11755422_s_at	turquoise	CD6 molecule(CD6)	Homo sapiens
4207	11755426_x_at	turquoise	Sec61 translocon alpha 1 subunit(SEC61A1)	Homo sapiens
4208	11755433_x_at	turquoise	uncharacterized LOC100505585(LOC100505585)	Homo sapiens
4209	11755443_a_at	turquoise	monoamine oxidase A(MAOA)	Homo sapiens
4211	11755469_x_at	turquoise	minichromosome maintenance complex component 7(MCM7)	Homo sapiens
4212	11755474_a_at	turquoise	ADAM metallopeptidase domain 15(ADAM15)	Homo sapiens
4213	11755476_a_at	turquoise	nicotinamide nucleotide adenylyltransferase 3(NMNAT3)	Homo sapiens
4214	11755481_a_at	turquoise	DNA helicase B(HELB)	Homo sapiens
4215	11755482_x_at	turquoise	G protein-coupled receptor 132(GPR132)	Homo sapiens
4216	11755483_x_at	turquoise	protein phosphatase 1 regulatory subunit 21(PPP1R21)	Homo sapiens
4217	11755522_a_at	turquoise	leupaxin(LPXN)	Homo sapiens
4218	11755523_a_at	turquoise	RNA binding motif protein 47(RBM47)	Homo sapiens
4219	11755533_a_at	turquoise	adhesion G protein-coupled receptor A3(ADGRA3)	Homo sapiens
4220	11755546_a_at	turquoise	LFNG O-fucosylpeptide 3-beta-N-acetylglucosaminyltransferase(LFNG)	Homo sapiens
4221	11755547_a_at	turquoise	serine/threonine kinase 33(STK33)	Homo sapiens
4222	11755549_a_at	turquoise	semaphorin 6D(SEMA6D)	Homo sapiens
4223	11755556_a_at	turquoise	exocyst complex component 2(EXOC2)	Homo sapiens
4224	11755564_x_at	turquoise	C-C motif chemokine ligand 3 like 3(CCL3L3)	Homo sapiens
4225	11755577_x_at	turquoise	meiotic double-stranded break formation protein 1(MEI1)	Homo sapiens
4226	11755587_a_at	turquoise	tripartite motif containing 22(TRIM22)	Homo sapiens
4227	11755606_x_at	turquoise	IFI30, lysosomal thiol reductase(IFI30)	Homo sapiens
4228	11755615_a_at	turquoise	Janus kinase and microtubule interacting protein 3(JAKMIP3)	Homo sapiens
4229	11755616_a_at	turquoise	solute carrier family 8 member B1(SLC8B1)	Homo sapiens
4230	11755622_x_at	turquoise	roundabout guidance receptor 4(ROBO4)	Homo sapiens
4231	11755631_x_at	turquoise	required for meiotic nuclear division 1 homolog(RMND1)	Homo sapiens
4232	11755641_s_at	turquoise	post-GPI attachment to proteins 1(PGAP1)	Homo sapiens
4233	11755646_a_at	turquoise	sialic acid binding Ig like lectin 1(SIGLEC1)	Homo sapiens
4234	11755671_a_at	turquoise	synaptotagmin like 2(SYTL2)	Homo sapiens
4235	11755676_a_at	turquoise	interaction protein for cytohesin exchange factors 1(IPCEF1)	Homo sapiens
4236	11755703_a_at	turquoise	zinc finger protein 287(ZNF287)	Homo sapiens
4237	11755705_a_at	turquoise	small integral membrane protein 5(SMIM5)	Homo sapiens
4238	11755709_s_at	turquoise	La ribonucleoprotein domain family member 6(LARP6)	Homo sapiens
4240	11755717_a_at	turquoise	docking protein 2(DOK2)	Homo sapiens
4241	11755718_s_at	turquoise	histone deacetylase 1(HDAC1)	Homo sapiens
4242	11755747_a_at	turquoise	placenta specific 8(PLAC8)	Homo sapiens
4243	11755748_s_at	turquoise	placenta specific 8(PLAC8)	Homo sapiens
4244	11755755_a_at	turquoise	integrator complex subunit 6 like(INTS6L)	Homo sapiens
4245	11755758_s_at	turquoise	NLR family CARD domain containing 5(NLRC5)	Homo sapiens
4246	11755762_a_at	turquoise	transmembrane protein 2(TMEM2)	Homo sapiens
4247	11755766_x_at	turquoise	sorting nexin family member 30(SNX30)	Homo sapiens
4248	11755778_a_at	turquoise	roundabout guidance receptor 4(ROBO4)	Homo sapiens
4250	11755811_a_at	turquoise	zinc finger protein 266(ZNF266)	Homo sapiens
4251	11755818_a_at	turquoise	FYVE and coiled-coil domain containing 1(FYCO1)	Homo sapiens
4252	11755819_a_at	turquoise	DExD/H-box helicase 58(DDX58)	Homo sapiens

4253	11755830_a_at	turquoise	myosin IG(MYO1G)	Homo sapiens
4254	11755858_a_at	turquoise	membrane spanning 4-domains A1(MS4A1)	Homo sapiens
4255	11755874_a_at	turquoise	bora, aurora kinase A activator(BORA)	Homo sapiens
4257	11755882_a_at	turquoise	solute carrier family 35 member B3(SLC35B3)	Homo sapiens
4258	11755888_a_at	turquoise	regucalcin(RGN)	Homo sapiens
4259	11755902_a_at	turquoise	transient receptor potential cation channel subfamily V member 2(TRPV2)	Homo sapiens
4260	11755903_x_at	turquoise	NEDD4 binding protein 2 like 1(N4BP2L1)	Homo sapiens
4261	11755908_s_at	turquoise	immunoglobulin superfamily member 3(IGSF3)	Homo sapiens
4262	11755912_s_at	turquoise	YEATS domain containing 2(YEATS2)	Homo sapiens
4263	11755915_a_at	turquoise	HPS3, biogenesis of lysosomal organelles complex 2 subunit 1(HPS3)	Homo sapiens
4264	11755932_a_at	turquoise	hematopoietic cell-specific Lyn substrate 1(HCLS1)	Homo sapiens
4265	11755933_x_at	turquoise	major histocompatibility complex, class I, B(HLA-B)	Homo sapiens
4266	11755943_x_at	turquoise	vimentin(VIM)	Homo sapiens
4267	11755952_x_at	turquoise	arrestin beta 2(ARRB2)	Homo sapiens
4269	11755966_x_at	turquoise	major histocompatibility complex, class I, B(HLA-B)	Homo sapiens
4271	11755990_a_at	turquoise	calpain 12(CAPN12)	Homo sapiens
4272	11755997_a_at	turquoise	sterile alpha motif domain containing 9 like(SAMD9L)	Homo sapiens
4273	11756006_x_at	turquoise	LCK proto-oncogene, Src family tyrosine kinase(LCK)	Homo sapiens
4274	11756011_a_at	turquoise	protein tyrosine phosphatase, receptor type J(PTPRJ)	Homo sapiens
4275	11756061_x_at	turquoise	small nucleolar RNA, H/ACA box 64(SNORA64)	Homo sapiens
4276	11756068_a_at	turquoise	beta-site APP-cleaving enzyme 2(BACE2)	Homo sapiens
4277	11756071_x_at	turquoise	actin beta(ACTB)	Homo sapiens
4278	11756073_x_at	turquoise	major histocompatibility complex, class II, DP beta 1(HLA-DPB1)	Homo sapiens
4279	11756077_a_at	turquoise	nudE neurodevelopment protein 1(NDE1)	Homo sapiens
4280	11756083_x_at	turquoise	major histocompatibility complex, class II, DQ alpha 1(HLA-DQA1)	Homo sapiens
4281	11756089_s_at	turquoise	macrophage scavenger receptor 1(MSR1)	Homo sapiens
4284	11756109_s_at	turquoise	mannosyl (alpha-1,6-)-glycoprotein beta-1,2-N-acetylglucosaminyltransferase(MGAT2)	Homo sapiens
4286	11756138_a_at	turquoise	hexokinase 2(HK2)	Homo sapiens
4287	11756146_x_at	turquoise	S100 calcium binding protein A4(S100A4)	Homo sapiens
4288	11756150_at	turquoise	beta-2-microglobulin(B2M)	Homo sapiens
4289	11756151_x_at	turquoise	beta-2-microglobulin(B2M)	Homo sapiens
4290	11756168_x_at	turquoise	RAB13, member RAS oncogene family(RAB13)	Homo sapiens
4291	11756170_a_at	turquoise	chromosome X open reading frame 38(CXorf38)	Homo sapiens
4292	11756175_a_at	turquoise	NSE4 homolog A, SMC5-SMC6 complex component(NSMCE4A)	Homo sapiens
4293	11756176_a_at	turquoise	eukaryotic translation initiation factor 4A3(EIF4A3)	Homo sapiens
4294	11756177_s_at	turquoise	CKLF like MARVEL transmembrane domain containing 6(CMTM6)	Homo sapiens
4295	11756178_s_at	turquoise	CKLF like MARVEL transmembrane domain containing 6(CMTM6)	Homo sapiens
4296	11756181_x_at	turquoise	tyrosine 3-monooxygenase/tryptophan 5-monooxygenase activation protein zeta(YWHAZ)	Homo sapiens
4299	11756223_s_at	turquoise	DExD-box helicase 21(DDX21)	Homo sapiens
4300	11756225_s_at	turquoise	DnaJ heat shock protein family (Hsp40) member B6(DNAJB6)	Homo sapiens
4301	11756226_a_at	turquoise	GRIP1 associated protein 1(GRIPAP1)	Homo sapiens
4302	11756228_s_at	turquoise	NECAP endocytosis associated 2(NECAP2)	Homo sapiens
4305	11756243_a_at	turquoise	dual adaptor of phosphotyrosine and 3-phosphoinositides 1(DAPP1)	Homo sapiens
4306	11756245_s_at	turquoise	annexin A5(ANXA5)	Homo sapiens
4307	11756255_x_at	turquoise	tyrosine 3-monooxygenase/tryptophan 5-monooxygenase activation protein eta(YWHAH)	Homo sapiens
4308	11756280_a_at	turquoise	nudix hydrolase 5(NUDT5)	Homo sapiens
4309	11756281_a_at	turquoise	CD7 molecule(CD7)	Homo sapiens
4310	11756283_s_at	turquoise	cell division cycle and apoptosis regulator 1(CCAR1)	Homo sapiens
4311	11756287_a_at	turquoise	acyloxyacyl hydrolase(AOAH)	Homo sapiens
4312	11756289_x_at	turquoise	CCM2 scaffolding protein(CCM2)	Homo sapiens
4313	11756292_a_at	turquoise	cathepsin W(CTSW)	Homo sapiens
4315	11756302_x_at	turquoise	CD37 molecule(CD37)	Homo sapiens
4316	11756303_a_at	turquoise	SH3 domain and tetratricopeptide repeats 1(SH3TC1)	Homo sapiens
4317	11756306_a_at	turquoise	poly(ADP-ribose) polymerase family member 9(PARP9)	Homo sapiens
4318	11756309_x_at	turquoise	RAB24, member RAS oncogene family(RAB24)	Homo sapiens
4319	11756317_a_at	turquoise	zinc finger protein 276(ZNF276)	Homo sapiens
4320	11756320_a_at	turquoise	nudix hydrolase 13(NUDT13)	Homo sapiens
4321	11756322_x_at	turquoise	CNDP dipeptidase 2 (metallopeptidase M20 family)(CNDP2)	Homo sapiens
4322	11756327_x_at	turquoise	ribosomal protein S19(RPS19)	Homo sapiens
4323	11756330_a_at	turquoise	DExD/H-box helicase 58(DDX58)	Homo sapiens
4325	11756358_a_at	turquoise	polo like kinase 3(PLK3)	Homo sapiens
4326	11756362_x_at	turquoise	Fc fragment of IgG receptor Ic, pseudogene(FCGR1CP)	Homo sapiens
4327	11756363_a_at	turquoise	NCK associated protein 1 like(NCKAP1L)	Homo sapiens
4329	11756369_a_at	turquoise	mitochondrial trans-2-enoyl-CoA reductase(MECR)	Homo sapiens
4331	11756386_s_at	turquoise	WD repeat domain 1(WDR1)	Homo sapiens
4332	11756387_x_at	turquoise	ADP ribosylation factor like GTPase 4A(ARL4A)	Homo sapiens
4333	11756391_a_at	turquoise	microtubule interacting and trafficking domain containing 1(MITD1)	Homo sapiens
4334	11756393_x_at	turquoise	coronin 1A(CORO1A)	Homo sapiens
4335	11756401_x_at	turquoise	family with sequence similarity 49 member B(FAM49B)	Homo sapiens
4336	11756411_a_at	turquoise	stabilin 1(STAB1)	Homo sapiens
4337	11756417_x_at	turquoise	kizuna centrosomal protein(KIZ)	Homo sapiens
4338	11756429_x_at	turquoise	oligosaccharyltransferase complex non-catalytic subunit(OSTC)	Homo sapiens
4339	11756434_a_at	turquoise	DNA methyltransferase 1(DNMT1)	Homo sapiens
4340	11756449_x_at	turquoise	RANBP2-type and C3HC4-type zinc finger containing 1(RBCK1)	Homo sapiens
4341	11756453_a_at	turquoise	SH2 domain containing 3C(SH2D3C)	Homo sapiens
4342	11756467_x_at	turquoise	transmembrane protein 50A(TMEM50A)	Homo sapiens
4343	11756479_x_at	turquoise	ribonuclease T2(RNASET2)	Homo sapiens
4344	11756485_a_at	turquoise	taxilin alpha(TXLNA)	Homo sapiens
4348	11756512_a_at	turquoise	zinc fingers and homeoboxes 3(ZHX3)	Homo sapiens
4349	11756523_x_at	turquoise	shisa family member 5(SHISA5)	Homo sapiens
4350	11756525_a_at	turquoise	IDI2 antisense RNA 1(IDI2-AS1)	Homo sapiens
4352	11756532_a_at	turquoise	phosphatidylinositol-4,5-bisphosphate 3-kinase catalytic subunit delta(PIK3CD)	Homo sapiens

4353	11756535_a_at	turquoise	solute carrier family 25 member 45(SLC25A45)	Homo sapiens
4354	11756543_a_at	turquoise	nuclear RNA export factor 1(NXF1)	Homo sapiens
4355	11756564_a_at	turquoise	ancient ubiquitous protein 1(AUP1)	Homo sapiens
4356	11756572_x_at	turquoise	interferon regulatory factor 3(IRF3)	Homo sapiens
4357	11756581_s_at	turquoise	protease, serine 23(PRSS23)	Homo sapiens
4359	11756603_a_at	turquoise	family with sequence similarity 206 member A(FAM206A)	Homo sapiens
4360	11756613_a_at	turquoise	semaphorin 6A(SEMA6A)	Homo sapiens
4361	11756617_a_at	turquoise	TAP binding protein(TAPBP)	Homo sapiens
4362	11756618_x_at	turquoise	TAP binding protein(TAPBP)	Homo sapiens
4363	11756626_s_at	turquoise	ZW10 interacting kinetochore protein(ZWINT)	Homo sapiens
4364	11756632_a_at	turquoise	granulysin(GNLY)	Homo sapiens
4365	11756636_x_at	turquoise	PC-esterase domain containing 1A(PCED1A)	Homo sapiens
4366	11756641_a_at	turquoise	zinc finger protein 256(ZNF256)	Homo sapiens
4367	11756645_x_at	turquoise	C-X-C motif chemokine ligand 16(CXCL16)	Homo sapiens
4369	11756655_a_at	turquoise	malonyl-CoA-acyl carrier protein transacylase(MCAT)	Homo sapiens
4371	11756668_x_at	turquoise	family with sequence similarity 213 member A(FAM213A)	Homo sapiens
4372	11756683_a_at	turquoise	CD1e molecule(CD1E)	Homo sapiens
4374	11756694_a_at	turquoise	ring finger protein 44(RNF44)	Homo sapiens
4375	11756702_a_at	turquoise	cytochrome P450 family 39 subfamily A member 1(CYP39A1)	Homo sapiens
4376	11756709_x_at	turquoise	Bruton tyrosine kinase(BTK)	Homo sapiens
4377	11756712_a_at	turquoise	acid phosphatase 2, lysosomal(ACP2)	Homo sapiens
4379	11756725_x_at	turquoise	shisa family member 5(SHISA5)	Homo sapiens
4380	11756741_x_at	turquoise	SH3 domain binding glutamate rich protein like(SH3BGRL)	Homo sapiens
4381	11756746_s_at	turquoise	A-kinase interacting protein 1(AKIP1)	Homo sapiens
4383	11756765_x_at	turquoise	BCL2 like 13(BCL2L13)	Homo sapiens
4384	11756766_x_at	turquoise	CD74 molecule(CD74)	Homo sapiens
4385	11756767_x_at	turquoise	primary cilia formation(PIFO)	Homo sapiens
4386	11756780_a_at	turquoise	membrane spanning 4-domains A7(MS4A7)	Homo sapiens
4387	11756786_x_at	turquoise	solute carrier family 38 member 9(SLC38A9)	Homo sapiens
4388	11756787_a_at	turquoise	hyaluronan and proteoglycan link protein 3(HAPLN3)	Homo sapiens
4389	11756792_x_at	turquoise	microRNA 6837(MIR6837)	Homo sapiens
4390	11756793_a_at	turquoise	vanin 2(VNN2)	Homo sapiens
4391	11756806_a_at	turquoise	interferon stimulated exonuclease gene 20(ISG20)	Homo sapiens
4392	11756807_a_at	turquoise	receptor interacting serine/threonine kinase 1(RIPK1)	Homo sapiens
4393	11756809_a_at	turquoise	EF-hand domain containing 2(EFHC2)	Homo sapiens
4394	11756818_a_at	turquoise	PAT1 homolog 2(PATL2)	Homo sapiens
4395	11756822_s_at	turquoise	lymphatic vessel endothelial hyaluronan receptor 1(LYVE1)	Homo sapiens
4396	11756827_a_at	turquoise	reticulon 1(RTN1)	Homo sapiens
4397	11756839_x_at	turquoise	zinc finger CCCH-type containing, antiviral 1(ZC3HAV1)	Homo sapiens
4398	11756847_a_at	turquoise	C-X3-C motif chemokine receptor 1(CX3CR1)	Homo sapiens
4400	11756867_a_at	turquoise	tumor necrosis factor superfamily member 13b(TNFSF13B)	Homo sapiens
4401	11756869_a_at	turquoise	PDZ domain containing 2(PDZD2)	Homo sapiens
4402	11756871_x_at	turquoise	sorting nexin 10(SNX10)	Homo sapiens
4403	11756883_s_at	turquoise	proline rich coiled-coil 2C(PRRC2C)	Homo sapiens
4404	11756892_a_at	turquoise	C2 calcium dependent domain containing 2(C2CD2)	Homo sapiens
4405	11756913_s_at	turquoise	nucleotide binding oligomerization domain containing 1(NOD1)	Homo sapiens
4406	11756924_s_at	turquoise	ATPase H+ transporting V1 subunit A(ATP6V1A)	Homo sapiens
4407	11756946_x_at	turquoise	TBC1 domain family member 10C(TBC1D10C)	Homo sapiens
4408	11756952_a_at	turquoise	transketolase(TKT)	Homo sapiens
4409	11756959_x_at	turquoise	protein kinase D2(PRKD2)	Homo sapiens
4410	11756976_a_at	turquoise	LON peptidase N-terminal domain and ring finger 2(LONRF2)	Homo sapiens
4411	11756977_a_at	turquoise	phytanoyl-CoA dioxygenase domain containing 1(PHYHD1)	Homo sapiens
4412	11756979_x_at	turquoise	tubulin alpha 1b(TUBA1B)	Homo sapiens
4413	11756984_a_at	turquoise	G protein subunit beta 2(GNB2)	Homo sapiens
4414	11756994_a_at	turquoise	G protein subunit beta 2(GNB2)	Homo sapiens
4415	11756999_a_at	turquoise	cytochrome b561 family member D2(CYB561D2)	Homo sapiens
4417	11757022_x_at	turquoise	tissue factor pathway inhibitor 2(TFPI2)	Homo sapiens
4419	11757035_a_at	turquoise	atypical chemokine receptor 1 (Duffy blood group)(ACKR1)	Homo sapiens
4420	11757037_s_at	turquoise	microRNA 4738(MIR4738)	Homo sapiens
4421	11757038_x_at	turquoise	microRNA 4738(MIR4738)	Homo sapiens
4422	11757049_a_at	turquoise	CTD phosphatase subunit 1(CTDP1)	Homo sapiens
4423	11757050_x_at	turquoise	transgelin 2(TAGLN2)	Homo sapiens
4424	11757056_x_at	turquoise	microRNA 1244-1(MIR1244-1)	Homo sapiens
4425	11757059_x_at	turquoise	RPL36A-HNRNPH2 readthrough(RPL36A-HNRNPH2)	Homo sapiens
4428	11757085_s_at	turquoise	N-acylsphingosine amidohydrolase 2(ASAH2)	Homo sapiens
4429	11757141_s_at	turquoise	microRNA 1304(MIR1304)	Homo sapiens
4430	11757156_s_at	turquoise	small nucleolar RNA, H/ACA box 44(SNORA44)	Homo sapiens
4431	11757164_s_at	turquoise	microRNA 1304(MIR1304)	Homo sapiens
4432	11757180_a_at	turquoise	casein kinase 1 gamma 2(CSNK1G2)	Homo sapiens
4433	11757186_a_at	turquoise	hexokinase 3(HK3)	Homo sapiens
4434	11757190_s_at	turquoise	lipopolysaccharide induced TNF factor(LITAF)	Homo sapiens
4435	11757194_a_at	turquoise	leishmanolysin like peptidase(LMLN)	Homo sapiens
4436	11757213_at	turquoise	uncharacterized FLJ34503(FLJ34503)	Homo sapiens
4437	11757259_x_at	turquoise	small Cajal body-specific RNA 9-like(SCARNA9L)	Homo sapiens
4440	11757269_s_at	turquoise	ferritin light chain(FTL)	Homo sapiens
4441	11757271_s_at	turquoise	sorting nexin 6(SNX6)	Homo sapiens
4442	11757274_s_at	turquoise	arginine and glutamate rich 1(ARGLU1)	Homo sapiens
4443	11757275_x_at	turquoise	arginine and glutamate rich 1(ARGLU1)	Homo sapiens
4447	11757284_x_at	turquoise	myeloid derived growth factor(MYDGF)	Homo sapiens
4448	11757289_x_at	turquoise	clathrin light chain A(CLTA)	Homo sapiens
4449	11757291_s_at	turquoise	iron-sulfur cluster assembly enzyme(ISCU)	Homo sapiens
4450	11757300_s_at	turquoise	ELOVL fatty acid elongase 5(ELOVL5)	Homo sapiens

4451	11757321_a_at	turquoise	TAP binding protein(TAPBP)	Homo sapiens
4452	11757326_s_at	turquoise	ferritin light chain(FTL)	Homo sapiens
4453	11757327_x_at	turquoise	ferritin light chain(FTL)	Homo sapiens
4455	11757332_x_at	turquoise	microRNA 3064(MIR3064)	Homo sapiens
4457	11757335_s_at	turquoise	mitochondrial translational initiation factor 3(MTIF3)	Homo sapiens
4458	11757337_x_at	turquoise	microRNA 6734(MIR6734)	Homo sapiens
4459	11757338_x_at	turquoise	glyoxylate and hydroxypyruvate reductase(GRHPR)	Homo sapiens
4460	11757342_x_at	turquoise	dCMP deaminase(DCTD)	Homo sapiens
4461	11757344_x_at	turquoise	small nuclear ribonucleoprotein polypeptides B and B1(SNRPB)	Homo sapiens
4462	11757346_s_at	turquoise	ferritin light chain(FTL)	Homo sapiens
4463	11757351_a_at	turquoise	transporter 1, ATP binding cassette subfamily B member(TAP1)	Homo sapiens
4464	11757367_s_at	turquoise	heat shock protein family A (Hsp70) member 6(HSPA6)	Homo sapiens
4465	11757368_x_at	turquoise	annexin A4(ANXA4)	Homo sapiens
4466	11757369_s_at	turquoise	mannosidase alpha class 2B member 1(MAN2B1)	Homo sapiens
4467	11757373_x_at	turquoise	apolipoprotein C1(APOC1)	Homo sapiens
4468	11757379_a_at	turquoise	tyrosine kinase 2(TYK2)	Homo sapiens
4469	11757383_a_at	turquoise	cathepsin H(CTSH)	Homo sapiens
4470	11757404_x_at	turquoise	inhibitor of DNA binding 3, HLH protein(ID3)	Homo sapiens
4472	11757409_x_at	turquoise	thymosin beta 10(TMSB10)	Homo sapiens
4474	11757415_s_at	turquoise	solute carrier family 5 member 3(SLC5A3)	Homo sapiens
4475	11757420_s_at	turquoise	ZFP36 ring finger protein like 2(ZFP36L2)	Homo sapiens
4476	11757425_s_at	turquoise	vascular cell adhesion molecule 1(VCAM1)	Homo sapiens
4478	11757435_x_at	turquoise	bridging integrator 1(BIN1)	Homo sapiens
4479	11757436_x_at	turquoise	interferon induced transmembrane protein 2(IFITM2)	Homo sapiens
4480	11757438_x_at	turquoise	destrin, actin depolymerizing factor(DSTN)	Homo sapiens
4481	11757439_s_at	turquoise	GDP dissociation inhibitor 2(GDI2)	Homo sapiens
4484	11757454_s_at	turquoise	cAMP responsive element binding protein like 2(CREBL2)	Homo sapiens
4485	11757455_x_at	turquoise	glutathione peroxidase 1(GPX1)	Homo sapiens
4486	11757459_s_at	turquoise	WAS/WASL interacting protein family member 1(WIPF1)	Homo sapiens
4487	11757464_x_at	turquoise	plexin B2(PLXNB2)	Homo sapiens
4489	11757469_s_at	turquoise	tripeptidyl peptidase 1(TPP1)	Homo sapiens
4490	11757470_x_at	turquoise	transcription factor 25(TCF25)	Homo sapiens
4491	11757474_x_at	turquoise	chloride intracellular channel 1(CLIC1)	Homo sapiens
4492	11757477_s_at	turquoise	LIM and SH3 protein 1(LASP1)	Homo sapiens
4493	11757480_x_at	turquoise	interferon alpha inducible protein 27(IFI27)	Homo sapiens
4494	11757483_s_at	turquoise	von Willebrand factor A domain containing 1(VWA1)	Homo sapiens
4496	11757511_x_at	turquoise	major histocompatibility complex, class II, DP alpha 1(HLA-DPA1)	Homo sapiens
4497	11757513_at	turquoise	NFKB inhibitor zeta(NFKBIZ)	Homo sapiens
4498	11757519_x_at	turquoise	capping actin protein, gelsolin like(CAPG)	Homo sapiens
4500	11757529_s_at	turquoise	lysosomal associated membrane protein 1(LAMP1)	Homo sapiens
4501	11757532_s_at	turquoise	RNA binding motif protein 47(RBM47)	Homo sapiens
4502	11757533_x_at	turquoise	phospholipid scramblase 1(PLSCR1)	Homo sapiens
4504	11757539_s_at	turquoise	stromal interaction molecule 2(STIM2)	Homo sapiens
4505	11757544_s_at	turquoise	intercellular adhesion molecule 2(ICAM2)	Homo sapiens
4506	11757545_x_at	turquoise	platelet and endothelial cell adhesion molecule 1(PECAM1)	Homo sapiens
4507	11757546_x_at	turquoise	MICAL like 2(MICALL2)	Homo sapiens
4508	11757552_x_at	turquoise	actin related protein 2/3 complex subunit 3(ARPC3)	Homo sapiens
4509	11757554_s_at	turquoise	EGF like domain multiple 8(EGFL8)	Homo sapiens
4510	11757556_s_at	turquoise	bleomycin hydrolase(BLMH)	Homo sapiens
4511	11757563_a_at	turquoise	protein phosphatase 1 regulatory subunit 35(PPP1R35)	Homo sapiens
4512	11757566_x_at	turquoise	nucleolar protein 8(NOL8)	Homo sapiens
4514	11757574_s_at	turquoise	transmembrane protein 123(TMEM123)	Homo sapiens
4515	11757588_a_at	turquoise	ALG5, dolichyl-phosphate beta-glucosyltransferase(ALG5)	Homo sapiens
4517	11757590_s_at	turquoise	ISY1-RAB43 readthrough(ISY1-RAB43)	Homo sapiens
4518	11757591_s_at	turquoise	PAN3 poly(A) specific ribonuclease subunit(PAN3)	Homo sapiens
4520	11757602_x_at	turquoise	endoplasmic reticulum protein 29(ERP29)	Homo sapiens
4521	11757604_a_at	turquoise	SAMM50 sorting and assembly machinery component(SAMM50)	Homo sapiens
4523	11757624_s_at	turquoise	poly(ADP-ribose) polymerase family member 12(PARP12)	Homo sapiens
4524	11757625_s_at	turquoise	CD200 molecule(CD200)	Homo sapiens
4525	11757630_s_at	turquoise	HERPUD family member 2(HERPUD2)	Homo sapiens
4526	11757632_s_at	turquoise	LIM domain kinase 2(LIMK2)	Homo sapiens
4527	11757635_a_at	turquoise	TYRO protein tyrosine kinase binding protein(TYROBP)	Homo sapiens
4528	11757638_s_at	turquoise	CD93 molecule(CD93)	Homo sapiens
4530	11757652_x_at	turquoise	prolyl 3-hydroxylase 1(P3H1)	Homo sapiens
4531	11757653_s_at	turquoise	CDK5 regulatory subunit associated protein 3(CDK5RAP3)	Homo sapiens
4532	11757654_x_at	turquoise	uncoupling protein 2(UCP2)	Homo sapiens
4533	11757661_x_at	turquoise	small nuclear ribonucleoprotein polypeptides B and B1(SNRPB)	Homo sapiens
4534	11757669_s_at	turquoise	peptidylprolyl isomerase like 1(PPIL1)	Homo sapiens
4537	11757692_s_at	turquoise	APC, WNT signaling pathway regulator(APC)	Homo sapiens
4538	11757713_x_at	turquoise	ataxin 2 like(ATXN2L)	Homo sapiens
4539	11757722_s_at	turquoise	septin 8(SEPT8)	Homo sapiens
4541	11757728_s_at	turquoise	ferritin light chain(FTL)	Homo sapiens
4542	11757731_s_at	turquoise	RAB31, member RAS oncogene family(RAB31)	Homo sapiens
4543	11757732_x_at	turquoise	G protein subunit alpha i2(GNAI2)	Homo sapiens
4544	11757737_s_at	turquoise	recombination signal binding protein for immunoglobulin kappa J region(RBPJ)	Homo sapiens
4545	11757756_s_at	turquoise	additional sex combs like 2, transcriptional regulator(ASXL2)	Homo sapiens
4546	11757765_s_at	turquoise	sarcoglycan delta(SGCD)	Homo sapiens
4547	11757787_x_at	turquoise	ferritin light chain(FTL)	Homo sapiens
4548	11757798_s_at	turquoise	MAF bZIP transcription factor B(MAFB)	Homo sapiens
4549	11757801_x_at	turquoise	major histocompatibility complex, class II, DP beta 1(HLA-DPB1)	Homo sapiens
4550	11757806_x_at	turquoise	peroxiredoxin 6(PRDX6)	Homo sapiens
4551	11757807_x_at	turquoise	actin beta(ACTB)	Homo sapiens

4552	11757816_s_at	turquoise	myristoylated alanine rich protein kinase C substrate(MARCKS)	Homo sapiens
4553	11757817_s_at	turquoise	brain abundant membrane attached signal protein 1(BASP1)	Homo sapiens
4554	11757818_x_at	turquoise	cofilin 1(CFL1)	Homo sapiens
4556	11757831_x_at	turquoise	synaptosome associated protein 23(SNAP23)	Homo sapiens
4557	11757833_a_at	turquoise	RAB31, member RAS oncogene family(RAB31)	Homo sapiens
4558	11757839_s_at	turquoise	NECAP endocytosis associated 2(NECAP2)	Homo sapiens
4561	11757867_s_at	turquoise	basic leucine zipper and W2 domains 1(BZW1)	Homo sapiens
4562	11757872_s_at	turquoise	phospholipid scramblase 1(PLSCR1)	Homo sapiens
4564	11757875_s_at	turquoise	Lck interacting transmembrane adaptor 1(LIME1)	Homo sapiens
4565	11757879_s_at	turquoise	microRNA 6837(MIR6837)	Homo sapiens
4566	11757883_s_at	turquoise	ring finger protein 4(RNF4)	Homo sapiens
4567	11757887_x_at	turquoise	tyrosine 3-monooxygenase/tryptophan 5-monooxygenase activation protein beta(YWHAB)	Homo sapiens
4568	11757890_a_at	turquoise	mitochondrial transcription termination factor 2(MTERF2)	Homo sapiens
4569	11757894_x_at	turquoise	NFKB inhibitor alpha(NFKBIA)	Homo sapiens
4570	11757896_s_at	turquoise	arginine and serine rich protein 1(RSRP1)	Homo sapiens
4571	11757904_s_at	turquoise	microtubule associated serine/threonine kinase 3(MAST3)	Homo sapiens
4572	11757908_x_at	turquoise	ral guanine nucleotide dissociation stimulator(RALGDS)	Homo sapiens
4573	11757917_s_at	turquoise	CCR4-NOT transcription complex subunit 6 like(CNOT6L)	Homo sapiens
4574	11757920_s_at	turquoise	zyg-11 family member B, cell cycle regulator(ZYG11B)	Homo sapiens
4575	11757931_s_at	turquoise	KIAA0930(KIAA0930)	Homo sapiens
4576	11757934_at	turquoise	KIAA1551(KIAA1551)	Homo sapiens
4577	11757935_x_at	turquoise	KIAA1551(KIAA1551)	Homo sapiens
4579	11757938_s_at	turquoise	N-myc and STAT interactor(NMI)	Homo sapiens
4580	11757940_at	turquoise	placental growth factor(PGF)	Homo sapiens
4581	11757942_s_at	turquoise	utrophin(UTRN)	Homo sapiens
4582	11757950_s_at	turquoise	chondroitin sulfate synthase 1(CHSY1)	Homo sapiens
4583	11757953_x_at	turquoise	tetratricopeptide repeat domain 39C(TTC39C)	Homo sapiens
4584	11757957_s_at	turquoise	SRY-box 6(SOX6)	Homo sapiens
4585	11757960_s_at	turquoise	ArfGAP with RhoGAP domain, ankyrin repeat and PH domain 2(ARAP2)	Homo sapiens
4586	11757966_s_at	turquoise	zinc finger protein 75D(ZNF75D)	Homo sapiens
4587	11757983_s_at	turquoise	translocation associated membrane protein 1(TRAM1)	Homo sapiens
4588	11757986_s_at	turquoise	MIS12, kinetochore complex component(MIS12)	Homo sapiens
4589	11757989_s_at	turquoise	ankyrin repeat domain 46(ANKRD46)	Homo sapiens
4590	11758007_s_at	turquoise	YY1 associated factor 2(YAF2)	Homo sapiens
4591	11758008_s_at	turquoise	patatin like phospholipase domain containing 4(PNPLA4)	Homo sapiens
4592	11758009_s_at	turquoise	uncharacterized LOC100130460(CAND1.11)	Homo sapiens
4593	11758011_x_at	turquoise	eukaryotic translation elongation factor 1 alpha 1(EEF1A1)	Homo sapiens
4594	11758014_s_at	turquoise	transforming growth factor beta receptor 1(TGFBR1)	Homo sapiens
4595	11758017_x_at	turquoise	peptidyl-prolyl cis-trans isomerase A pseudogene(LOC101060363)	Homo sapiens
4596	11758025_x_at	turquoise	inositol polyphosphate-5-phosphatase F(INPP5F)	Homo sapiens
4597	11758037_s_at	turquoise	myotubularin related protein 12(MTMR12)	Homo sapiens
4598	11758042_x_at	turquoise	actin beta(ACTB)	Homo sapiens
4599	11758043_s_at	turquoise	immediate early response 2(IER2)	Homo sapiens
4600	11758047_s_at	turquoise	major facilitator superfamily domain containing 14A(MFSD14A)	Homo sapiens
4602	11758059_s_at	turquoise	ankyrin repeat domain 49(ANKRD49)	Homo sapiens
4603	11758067_x_at	turquoise	peptidyl-prolyl cis-trans isomerase A pseudogene(LOC101060363)	Homo sapiens
4604	11758089_s_at	turquoise	hyaluronan mediated motility receptor(HMMR)	Homo sapiens
4605	11758090_s_at	turquoise	interleukin 16(IL16)	Homo sapiens
4606	11758092_s_at	turquoise	ephrin A5(EFNA5)	Homo sapiens
4607	11758094_s_at	turquoise	nuclear factor of activated T-cells 2 interacting protein(NFATC2IP)	Homo sapiens
4609	11758113_s_at	turquoise	actin related protein 2/3 complex subunit 4(ARPC4)	Homo sapiens
4610	11758114_x_at	turquoise	src kinase associated phosphoprotein 2(SKAP2)	Homo sapiens
4611	11758126_s_at	turquoise	ELL associated factor 1(EAF1)	Homo sapiens
4614	11758144_x_at	turquoise	heterogeneous nuclear ribonucleoprotein C (C1/C2)(HNRNPC)	Homo sapiens
4615	11758148_s_at	turquoise	beta-2-microglobulin(B2M)	Homo sapiens
4616	11758149_s_at	turquoise	Rac GTPase activating protein 1(RACGAP1)	Homo sapiens
4617	11758158_s_at	turquoise	forkhead box P1(FOXP1)	Homo sapiens
4618	11758162_s_at	turquoise	ferredoxin 1(FDX1)	Homo sapiens
4619	11758176_s_at	turquoise	ATPase phospholipid transporting 10D (putative)(ATP10D)	Homo sapiens
4620	11758178_s_at	turquoise	coronin 1B(CORO1B)	Homo sapiens
4621	11758182_x_at	turquoise	ferritin light chain(FTL)	Homo sapiens
4623	11758191_s_at	turquoise	mex-3 RNA binding family member C(MEX3C)	Homo sapiens
4624	11758192_s_at	turquoise	beta-2-microglobulin(B2M)	Homo sapiens
4625	11758193_x_at	turquoise	beta-2-microglobulin(B2M)	Homo sapiens
4626	11758208_s_at	turquoise	Kruppel like factor 2(KLF2)	Homo sapiens
4627	11758210_s_at	turquoise	tRNA methyltransferase 10C, mitochondrial RNase P subunit(TRMT10C)	Homo sapiens
4628	11758215_s_at	turquoise	transducin like enhancer of split 3(TLE3)	Homo sapiens
4630	11758219_x_at	turquoise	ribonucleotide reductase regulatory subunit M2(RRM2)	Homo sapiens
4631	11758222_s_at	turquoise	solute carrier family 20 member 1(SLC20A1)	Homo sapiens
4632	11758225_s_at	turquoise	IQ motif containing GTPase activating protein 2(IQGAP2)	Homo sapiens
4633	11758226_s_at	turquoise	ubiquitin conjugating enzyme E2 J1(UBE2J1)	Homo sapiens
4634	11758231_x_at	turquoise	major histocompatibility complex, class II, DP alpha 1(HLA-DPA1)	Homo sapiens
4635	11758238_s_at	turquoise	ATP binding cassette subfamily C member 4(ABCC4)	Homo sapiens
4638	11758259_s_at	turquoise	solute carrier organic anion transporter family member 2B1(SLCO2B1)	Homo sapiens
4639	11758260_x_at	turquoise	beta-2-microglobulin(B2M)	Homo sapiens
4640	11758261_s_at	turquoise	centrosomal protein 55(CEP55)	Homo sapiens
4641	11758263_s_at	turquoise	cytochrome b-245 beta chain(CYBB)	Homo sapiens
4642	11758269_s_at	turquoise	methionine adenosyltransferase 2B(MAT2B)	Homo sapiens
4643	11758273_s_at	turquoise	ADP ribosylation factor 6(ARF6)	Homo sapiens
4644	11758277_x_at	turquoise	neuroblastoma breakpoint family member 10(NBPF10)	Homo sapiens
4645	11758279_s_at	turquoise	coronin 1C(CORO1C)	Homo sapiens
4646	11758282_s_at	turquoise	tubulin alpha 1c(TUBA1C)	Homo sapiens

4647	11758297_s_at	turquoise	SHC adaptor protein 1(SHC1)	Homo sapiens
4648	11758299_a_at	turquoise	beta-2-microglobulin(B2M)	Homo sapiens
4649	11758300_x_at	turquoise	beta-2-microglobulin(B2M)	Homo sapiens
4652	11758327_s_at	turquoise	bromodomain adjacent to zinc finger domain 1A(BAZ1A)	Homo sapiens
4654	11758331_s_at	turquoise	uncharacterized LOC101929823(LOC101929823)	Homo sapiens
4656	11758337_s_at	turquoise	adenosine deaminase(ADA)	Homo sapiens
4657	11758340_x_at	turquoise	major histocompatibility complex, class II, DQ alpha 1(HLA-DQA1)	Homo sapiens
4659	11758365_s_at	turquoise	tubulin tyrosine ligase like 8(TTLL8)	Homo sapiens
4660	11758369_x_at	turquoise	major histocompatibility complex, class II, DP beta 1(HLA-DPB1)	Homo sapiens
4661	11758377_s_at	turquoise	toll like receptor 1(TLR1)	Homo sapiens
4662	11758385_s_at	turquoise	hes related family bHLH transcription factor with YRPW motif 2(HEY2)	Homo sapiens
4663	11758401_s_at	turquoise	zinc finger protein 148(ZNF148)	Homo sapiens
4665	11758409_s_at	turquoise	microRNA 6805(MIR6805)	Homo sapiens
4666	11758417_s_at	turquoise	major histocompatibility complex, class II, DP alpha 1(HLA-DPA1)	Homo sapiens
4667	11758418_s_at	turquoise	family with sequence similarity 219 member B(FAM219B)	Homo sapiens
4668	11758419_s_at	turquoise	transmembrane protein 106A(TMEM106A)	Homo sapiens
4669	11758447_s_at	turquoise	nucleoporin 50(NUP50)	Homo sapiens
4670	11758448_s_at	turquoise	TMED7-TICAM2 readthrough(TMED7-TICAM2)	Homo sapiens
4671	11758478_s_at	turquoise	cell division cycle associated 7(CDCA7)	Homo sapiens
4672	11758486_s_at	turquoise	beta-site APP-cleaving enzyme 2(BACE2)	Homo sapiens
4673	11758500_s_at	turquoise	transmembrane protein 140(TMEM140)	Homo sapiens
4674	11758501_s_at	turquoise	nicotinamide nucleotide adenylyltransferase 1(NMNAT1)	Homo sapiens
4675	11758527_s_at	turquoise	inositol 1,4,5-trisphosphate receptor type 2(ITPR2)	Homo sapiens
4676	11758537_s_at	turquoise	tripartite motif containing 25(TRIM25)	Homo sapiens
4677	11758539_s_at	turquoise	dedicator of cytokinesis 10(DOCK10)	Homo sapiens
4678	11758542_x_at	turquoise	zinc finger CCCH-type, RNA binding motif and serine/arginine rich 2(ZRSR2)	Homo sapiens
4679	11758547_s_at	turquoise	echinoderm microtubule associated protein like 1(EML1)	Homo sapiens
4680	11758550_x_at	turquoise	transmembrane protein 268(TMEM268)	Homo sapiens
4681	11758554_s_at	turquoise	ring finger and WD repeat domain 2(RFWD2)	Homo sapiens
4682	11758555_s_at	turquoise	G protein-coupled receptor 183(GPR183)	Homo sapiens
4683	11758557_s_at	turquoise	ZFP36 ring finger protein like 1(ZFP36L1)	Homo sapiens
4684	11758558_s_at	turquoise	ATPase sarcoplasmic/endoplasmic reticulum Ca2+ transporting 2(ATP2A2)	Homo sapiens
4686	11758595_at	turquoise	neurobeachin(NBEA)	Homo sapiens
4687	11758607_s_at	turquoise	von Willebrand factor A domain containing 8(VWA8)	Homo sapiens
4688	11758608_s_at	turquoise	colony stimulating factor 2 receptor beta common subunit(CSF2RB)	Homo sapiens
4689	11758620_s_at	turquoise	methylmalonic aciduria (cobalamin deficiency) cblB type(MMAB)	Homo sapiens
4690	11758630_s_at	turquoise	fibrinogen like 2(FGL2)	Homo sapiens
4691	11758645_s_at	turquoise	eukaryotic translation initiation factor 4E binding protein 2(EIF4EBP2)	Homo sapiens
4692	11758653_s_at	turquoise	adhesion G protein-coupled receptor L4(ADGRL4)	Homo sapiens
4693	11758663_s_at	turquoise	small nucleolar RNA host gene 4(SNHG4)	Homo sapiens
4694	11758667_x_at	turquoise	tyrosine 3-monooxygenase/tryptophan 5-monooxygenase activation protein zeta(YWHAZ)	Homo sapiens
4695	11758671_s_at	turquoise	IKAROS family zinc finger 1(IKZF1)	Homo sapiens
4696	11758679_s_at	turquoise	microRNA 1292(MIR1292)	Homo sapiens
4697	11758683_s_at	turquoise	jade family PHD finger 2(JADE2)	Homo sapiens
4698	11758693_s_at	turquoise	microRNA 7703(MIR7703)	Homo sapiens
4699	11758709_s_at	turquoise	retinol dehydrogenase 11 (all-trans/9-cis/11-cis)(RDH11)	Homo sapiens
4700	11758711_x_at	turquoise	tyrosine 3-monooxygenase/tryptophan 5-monooxygenase activation protein zeta(YWHAZ)	Homo sapiens
4701	11758716_at	turquoise	NDUFA4, mitochondrial complex associated(NDUFA4)	Homo sapiens
4704	11758750_x_at	turquoise	tyrosine 3-monooxygenase/tryptophan 5-monooxygenase activation protein zeta(YWHAZ)	Homo sapiens
4705	11758753_a_at	turquoise	StAR related lipid transfer domain containing 3(STARD3)	Homo sapiens
4706	11758754_a_at	turquoise	Fas associated factor 1(FAF1)	Homo sapiens
4708	11758757_at	turquoise	alcohol dehydrogenase 5 (class III), chi polypeptide(ADH5)	Homo sapiens
4709	11758771_at	turquoise	major histocompatibility complex, class II, DP beta 1(HLA-DPB1)	Homo sapiens
4710	11758772_x_at	turquoise	major histocompatibility complex, class II, DP beta 1(HLA-DPB1)	Homo sapiens
4711	11758793_at	turquoise	mitochondrial calcium uniporter dominant negative beta subunit(MCUB)	Homo sapiens
4713	11758821_x_at	turquoise	major histocompatibility complex, class II, DQ beta 1(HLA-DQB1)	Homo sapiens
4714	11758831_at	turquoise	RNA polymerase II subunit C(POLR2C)	Homo sapiens
4715	11758838_at	turquoise	serine palmitoyltransferase long chain base subunit 2(SPTLC2)	Homo sapiens
4716	11758854_at	turquoise	atlastin GTPase 3(ATL3)	Homo sapiens
4717	11758862_x_at	turquoise	poly(A) binding protein interacting protein 2B(PAIP2B)	Homo sapiens
4718	11758868_at	turquoise	Rap guanine nucleotide exchange factor 6(RAPGEF6)	Homo sapiens
4719	11758873_a_at	turquoise	heparanase(HPSE)	Homo sapiens
4720	11758879_s_at	turquoise	major histocompatibility complex, class I, E(HLA-E)	Homo sapiens
4721	11758882_at	turquoise	CD3d molecule(CD3D)	Homo sapiens
4722	11758897_at	turquoise	apolipoprotein B mRNA editing enzyme catalytic subunit 3C(APOBEC3C)	Homo sapiens
4723	11758898_x_at	turquoise	apolipoprotein B mRNA editing enzyme catalytic subunit 3C(APOBEC3C)	Homo sapiens
4724	11758901_at	turquoise	receptor interacting serine/threonine kinase 2(RIPK2)	Homo sapiens
4725	11758921_x_at	turquoise	mitochondrial ribosomal protein S17(MRPS17)	Homo sapiens
4726	11758922_at	turquoise	syntaxin binding protein 6(STXBP6)	Homo sapiens
4727	11758933_at	turquoise	transmembrane 4 L six family member 18(TM4SF18)	Homo sapiens
4728	11758934_x_at	turquoise	RAB27A, member RAS oncogene family(RAB27A)	Homo sapiens
4729	11758940_at	turquoise	MOB kinase activator 1A(MOB1A)	Homo sapiens
4730	11758941_x_at	turquoise	MOB kinase activator 1A(MOB1A)	Homo sapiens
4731	11758942_s_at	turquoise	MOB kinase activator 1A(MOB1A)	Homo sapiens
4732	11758947_a_at	turquoise	copine 4(CPNE4)	Homo sapiens
4733	11758984_at	turquoise	zinc finger RANBP2-type containing 1(ZRANB1)	Homo sapiens
4734	11758990_x_at	turquoise	SLC9A3 regulator 1(SLC9A3R1)	Homo sapiens
4735	11758994_at	turquoise	fatty acyl-CoA reductase 2(FAR2)	Homo sapiens
4736	11759022_s_at	turquoise	ELK3, ETS transcription factor(ELK3)	Homo sapiens
4737	11759049_at	turquoise	acyl-CoA synthetase short-chain family member 3(ACSS3)	Homo sapiens
4738	11759087_a_at	turquoise	NHL repeat containing 2(NHLRC2)	Homo sapiens
4739	11759088_at	turquoise	membrane associated ring-CH-type finger 1(MARCH1)	Homo sapiens

4740	11759093_s_at	turquoise	G protein subunit alpha q(GNAQ)	Homo sapiens
4741	11759094_a_at	turquoise	G protein subunit alpha q(GNAQ)	Homo sapiens
4742	11759095_at	turquoise	G protein subunit alpha q(GNAQ)	Homo sapiens
4743	11759144_at	turquoise	zinc finger protein 710(ZNF710)	Homo sapiens
4744	11759177_at	turquoise	mannosidase alpha class 1A member 1(MAN1A1)	Homo sapiens
4746	11759184_at	turquoise	plexin C1(PLXNC1)	Homo sapiens
4747	11759187_at	turquoise	TNF alpha induced protein 8 like 1(TNFAIP8L1)	Homo sapiens
4749	11759292_at	turquoise	family with sequence similarity 63 member A(FAM63A)	Homo sapiens
4750	11759294_s_at	turquoise	lysine methyltransferase 2A(KMT2A)	Homo sapiens
4751	11759296_at	turquoise	mitogen-activated protein kinase kinase kinase 2(MAP3K2)	Homo sapiens
4752	11759300_s_at	turquoise	A-kinase anchoring protein 10(AKAP10)	Homo sapiens
4753	11759320_at	turquoise	myelin protein zero like 3(MPZL3)	Homo sapiens
4754	11759328_at	turquoise	kinetochore scaffold 1(KNL1)	Homo sapiens
4755	11759347_x_at	turquoise	TXK tyrosine kinase(TXK)	Homo sapiens
4756	11759352_a_at	turquoise	Fc receptor like 3(FCRL3)	Homo sapiens
4757	11759414_at	turquoise	glycine cleavage system protein H(GCSH)	Homo sapiens
4759	11759444_x_at	turquoise	BCL2 associated X, apoptosis regulator(BAX)	Homo sapiens
4760	11759477_at	turquoise	ADAM metallopeptidase with thrombospondin type 1 motif 15(ADAMTS15)	Homo sapiens
4761	11759496_at	turquoise	carbohydrate sulfotransferase 11(CHST11)	Homo sapiens
4762	11759497_at	turquoise	carbohydrate sulfotransferase 11(CHST11)	Homo sapiens
4763	11759498_at	turquoise	family with sequence similarity 49 member A(FAM49A)	Homo sapiens
4764	11759499_at	turquoise	family with sequence similarity 49 member A(FAM49A)	Homo sapiens
4765	11759502_at	turquoise	TNF receptor associated factor 3(TRAF3)	Homo sapiens
4766	11759511_at	turquoise	motile sperm domain containing 2(MOSPD2)	Homo sapiens
4767	11759512_x_at	turquoise	CWC25 spliceosome associated protein homolog(CWC25)	Homo sapiens
4771	11759550_at	turquoise	ZNFX1 antisense RNA 1(ZFAS1)	Homo sapiens
4773	11759566_a_at	turquoise	N-acetylneuraminate pyruvate lyase(NPL)	Homo sapiens
4774	11759569_a_at	turquoise	CDK5 regulatory subunit associated protein 3(CDK5RAP3)	Homo sapiens
4775	11759572_a_at	turquoise	staufen double-stranded RNA binding protein 2(STAU2)	Homo sapiens
4776	11759573_at	turquoise	meningioma expressed antigen 5 (hyaluronidase)(MGEA5)	Homo sapiens
4778	11759581_x_at	turquoise	major histocompatibility complex, class I, B(HLA-B)	Homo sapiens
4779	11759587_at	turquoise	HtrA serine peptidase 3(HTRA3)	Homo sapiens
4780	11759615_x_at	turquoise	pre-mRNA processing factor 38B(PRPF38B)	Homo sapiens
4781	11759619_at	turquoise	trafficking protein particle complex 10(TRAPPC10)	Homo sapiens
4782	11759622_s_at	turquoise	DExD-box helicase 21(DDX21)	Homo sapiens
4783	11759627_at	turquoise	microRNA 6125(MIR6125)	Homo sapiens
4784	11759629_a_at	turquoise	T cell receptor delta variable 2(TRDV2)	Homo sapiens
4785	11759635_x_at	turquoise	THO complex 2(THOC2)	Homo sapiens
4786	11759642_x_at	turquoise	major histocompatibility complex, class II, DR beta 1(HLA-DRB1)	Homo sapiens
4789	11759657_at	turquoise	target of myb1 like 1 membrane trafficking protein(TOM1L1)	Homo sapiens
4790	11759663_x_at	turquoise	SP100 nuclear antigen(SP100)	Homo sapiens
4791	11759666_x_at	turquoise	major histocompatibility complex, class II, DP beta 1(HLA-DPB1)	Homo sapiens
4792	11759671_s_at	turquoise	potassium calcium-activated channel subfamily M alpha 1(KCNMA1)	Homo sapiens
4793	11759678_at	turquoise	RAB12, member RAS oncogene family(RAB12)	Homo sapiens
4795	11759729_a_at	turquoise	tetratricopeptide repeat domain 14(TTC14)	Homo sapiens
4796	11759759_s_at	turquoise	cyclin L1(CCNL1)	Homo sapiens
4797	11759766_at	turquoise	major histocompatibility complex, class I-related(MR1)	Homo sapiens
4798	11759767_x_at	turquoise	major histocompatibility complex, class I-related(MR1)	Homo sapiens
4801	11759828_s_at	turquoise	SP100 nuclear antigen(SP100)	Homo sapiens
4802	11759833_at	turquoise	NADH:ubiquinone oxidoreductase core subunit S7(NDUFS7)	Homo sapiens
4804	11759854_x_at	turquoise	ribosomal protein S27a(RPS27A)	Homo sapiens
4805	11759866_at	turquoise	NA	NA
4806	11759922_a_at	turquoise	par-3 family cell polarity regulator(PARD3)	Homo sapiens
4807	11759939_x_at	turquoise	uncharacterized LOC100131541(LOC100131541)	Homo sapiens
4808	11759973_at	turquoise	prenyl (decaprenyl) diphosphate synthase, subunit 1(PDSS1)	Homo sapiens
4809	11760005_x_at	turquoise	DnaJ heat shock protein family (Hsp40) member A1(DNAJA1)	Homo sapiens
4810	11760009_at	turquoise	NA	NA
4811	11760018_at	turquoise	C-type lectin domain family 10 member A(CLEC10A)	Homo sapiens
4812	11760037_a_at	turquoise	glutaminase(GLS)	Homo sapiens
4813	11760038_a_at	turquoise	glutaminase(GLS)	Homo sapiens
4814	11760085_s_at	turquoise	heterogeneous nuclear ribonucleoprotein A1(HNRNPA1)	Homo sapiens
4815	11760103_at	turquoise	protein kinase cAMP-dependent type II regulatory subunit alpha(PRKAR2A)	Homo sapiens
4816	11760124_a_at	turquoise	Kruppel like factor 6(KLF6)	Homo sapiens
4818	11760143_at	turquoise	major histocompatibility complex, class I, F(HLA-F)	Homo sapiens
4819	11760144_x_at	turquoise	major histocompatibility complex, class I, F(HLA-F)	Homo sapiens
4820	11760156_at	turquoise	Enah/Vasp-like(EVL)	Homo sapiens
4821	11760198_at	turquoise	eukaryotic translation initiation factor 4E family member 2(EIF4E2)	Homo sapiens
4822	11760211_at	turquoise	CD48 molecule(CD48)	Homo sapiens
4823	11760221_at	turquoise	caspase 4(CASP4)	Homo sapiens
4824	11760222_x_at	turquoise	caspase 4(CASP4)	Homo sapiens
4825	11760244_a_at	turquoise	caspase recruitment domain family member 8(CARD8)	Homo sapiens
4826	11760280_a_at	turquoise	epidermal growth factor receptor pathway substrate 15 like 1(EPS15L1)	Homo sapiens
4827	11760356_at	turquoise	peptidase, mitochondrial processing beta subunit(PMPCB)	Homo sapiens
4828	11760380_x_at	turquoise	septin 6(SEPT6)	Homo sapiens
4829	11760387_at	turquoise	interleukin 16(IL16)	Homo sapiens
4830	11760406_x_at	turquoise	major histocompatibility complex, class I, G(HLA-G)	Homo sapiens
4831	11760426_a_at	turquoise	epidermal growth factor receptor pathway substrate 15 like 1(EPS15L1)	Homo sapiens
4832	11760444_x_at	turquoise	major histocompatibility complex, class I, C(HLA-C)	Homo sapiens
4833	11760475_at	turquoise	serine/threonine kinase 4(STK4)	Homo sapiens
4835	11760584_a_at	turquoise	YME1 like 1 ATPase(YME1L1)	Homo sapiens
4836	11760710_a_at	turquoise	membrane spanning 4-domains A6A(MS4A6A)	Homo sapiens
4837	11760776_a_at	turquoise	dermokine(DMKN)	Homo sapiens

4839	11760822_at	turquoise	sodium voltage-gated channel beta subunit 1(SCN1B)	Homo sapiens
4840	11760850_x_at	turquoise	PATJ, crumbs cell polarity complex component(PATJ)	Homo sapiens
4841	11760878_x_at	turquoise	major histocompatibility complex, class II, DP beta 1(HLA-DPB1)	Homo sapiens
4842	11760881_x_at	turquoise	transcription termination factor 2(TTF2)	Homo sapiens
4843	11760902_x_at	turquoise	napsin B aspartic peptidase, pseudogene(NAPSB)	Homo sapiens
4845	11760994_at	turquoise	tryptophanyl-tRNA synthetase(WARS)	Homo sapiens
4847	11761116_a_at	turquoise	family with sequence similarity 65 member B(FAM65B)	Homo sapiens
4848	11761194_at	turquoise	trans-golgi network protein 2(TGOLN2)	Homo sapiens
4849	11761343_a_at	turquoise	ADAM metallopeptidase domain 8(ADAM8)	Homo sapiens
4850	11761390_a_at	turquoise	ankyrin repeat domain 10(ANKRD10)	Homo sapiens
4851	11761400_at	turquoise	nucleolar protein interacting with the FHA domain of MKI67(NIFK)	Homo sapiens
4852	11761418_x_at	turquoise	interleukin 23 subunit alpha(IL23A)	Homo sapiens
4853	11761427_at	turquoise	dedicator of cytokinesis 8(DOCK8)	Homo sapiens
4855	11761465_at	turquoise	NA	NA
4857	11761506_at	turquoise	malic enzyme 3(ME3)	Homo sapiens
4858	11761525_a_at	turquoise	interleukin 23 subunit alpha(IL23A)	Homo sapiens
4859	11761604_x_at	turquoise	RNA binding protein, fox-1 homolog 1(RBFOX1)	Homo sapiens
4860	11761671_a_at	turquoise	ETS variant 7(ETV7)	Homo sapiens
4861	11761683_x_at	turquoise	pyrin and HIN domain family member 1(PYHIN1)	Homo sapiens
4862	11761703_s_at	turquoise	DnaJ heat shock protein family (Hsp40) member A1(DNAJA1)	Homo sapiens
4863	11761727_x_at	turquoise	aquaporin 7(AQP7)	Homo sapiens
4864	11761758_at	turquoise	proteasome subunit beta 9(PSMB9)	Homo sapiens
4865	11761790_x_at	turquoise	YME1 like 1 ATPase(YME1L1)	Homo sapiens
4866	11761796_at	turquoise	calcitonin receptor like receptor(CALCRL)	Homo sapiens
4867	11761918_x_at	turquoise	T cell receptor beta constant 1(TRBC1)	Homo sapiens
4868	11761959_x_at	turquoise	T cell receptor delta variable 2(TRDV2)	Homo sapiens
4869	11761960_x_at	turquoise	T cell receptor delta variable 2(TRDV2)	Homo sapiens
4870	11762018_at	turquoise	DNA cross-link repair 1C(DCLRE1C)	Homo sapiens
4871	11762038_at	turquoise	zinc finger protein 844(ZNF844)	Homo sapiens
4872	11762069_a_at	turquoise	septin 6(SEPT6)	Homo sapiens
4875	11762119_x_at	turquoise	major histocompatibility complex, class I, A(HLA-A)	Homo sapiens
4876	11762140_a_at	turquoise	butyrophilin like 9(BTNL9)	Homo sapiens
4877	11762150_x_at	turquoise	major histocompatibility complex, class I, A(HLA-A)	Homo sapiens
4878	11762172_x_at	turquoise	ETS variant 7(ETV7)	Homo sapiens
4879	11762266_x_at	turquoise	interleukin 7 receptor(IL7R)	Homo sapiens
4880	11762274_x_at	turquoise	major histocompatibility complex, class II, DM beta(HLA-DMB)	Homo sapiens
4882	11762282_x_at	turquoise	major histocompatibility complex, class I, A(HLA-A)	Homo sapiens
4883	11762287_x_at	turquoise	interleukin 23 subunit alpha(IL23A)	Homo sapiens
4884	11762294_x_at	turquoise	T cell receptor beta constant 1(TRBC1)	Homo sapiens
4885	11762313_x_at	turquoise	enoyl-CoA hydratase domain containing 2(ECHDC2)	Homo sapiens
4886	11762318_x_at	turquoise	interleukin 23 subunit alpha(IL23A)	Homo sapiens
4887	11762339_x_at	turquoise	aldo-keto reductase family 7 member A2(AKR7A2)	Homo sapiens
4888	11762366_s_at	turquoise	recombination signal binding protein for immunoglobulin kappa J region(RBPJ)	Homo sapiens
4889	11762406_s_at	turquoise	guanylate binding protein 2(GBP2)	Homo sapiens
4890	11762432_a_at	turquoise	WD repeat domain 31(WDR31)	Homo sapiens
4891	11762456_at	turquoise	NA	NA
4892	11762480_x_at	turquoise	SP140 nuclear body protein(SP140)	Homo sapiens
4893	11762486_x_at	turquoise	fucosyltransferase 6(FUT6)	Homo sapiens
4894	11762641_x_at	turquoise	major histocompatibility complex, class II, DQ beta 1(HLA-DQB1)	Homo sapiens
4895	11762736_at	turquoise	butyrophilin like 9(BTNL9)	Homo sapiens
4896	11762785_x_at	turquoise	actin gamma 1(ACTG1)	Homo sapiens
4897	11762851_at	turquoise	major histocompatibility complex, class I, B(HLA-B)	Homo sapiens
4898	11762908_x_at	turquoise	TNF receptor superfamily member 14(TNFRSF14)	Homo sapiens
4899	11762936_x_at	turquoise	aquaporin 7(AQP7)	Homo sapiens
4900	11762940_x_at	turquoise	major histocompatibility complex, class I, C(HLA-C)	Homo sapiens
4901	11762993_x_at	turquoise	YME1 like 1 ATPase(YME1L1)	Homo sapiens
4902	11762996_x_at	turquoise	YME1 like 1 ATPase(YME1L1)	Homo sapiens
4903	11762998_x_at	turquoise	YME1 like 1 ATPase(YME1L1)	Homo sapiens
4907	11763159_at	turquoise	YME1 like 1 ATPase(YME1L1)	Homo sapiens
4908	11763182_at	turquoise	CCR4-NOT transcription complex subunit 9(CNOT9)	Homo sapiens
4909	11763184_at	turquoise	insulin degrading enzyme(IDE)	Homo sapiens
4910	11763186_at	turquoise	caspase 1(CASP1)	Homo sapiens
4911	11763196_at	turquoise	dymeclin(DYM)	Homo sapiens
4912	11763197_x_at	turquoise	dymeclin(DYM)	Homo sapiens
4913	11763201_at	turquoise	family with sequence similarity 65 member B(FAM65B)	Homo sapiens
4914	11763202_x_at	turquoise	family with sequence similarity 65 member B(FAM65B)	Homo sapiens
4915	11763215_a_at	turquoise	dermokine(DMKN)	Homo sapiens
4917	11763233_x_at	turquoise	T cell receptor delta variable 2(TRDV2)	Homo sapiens
4918	11763246_x_at	turquoise	major histocompatibility complex, class II, DQ beta 1(HLA-DQB1)	Homo sapiens
4921	11763271_at	turquoise	peroxisomal biogenesis factor 11 alpha(PEX11A)	Homo sapiens
4922	11763274_a_at	turquoise	signal induced proliferation associated 1 like 1(SIPA1L1)	Homo sapiens
4923	11763307_s_at	turquoise	nuclear factor of activated T-cells 2(NFATC2)	Homo sapiens
4924	11763310_at	turquoise	microRNA 21(MIR21)	Homo sapiens
4925	11763314_s_at	turquoise	ADP ribosylation factor like GTPase 4C(ARL4C)	Homo sapiens
4926	11763320_x_at	turquoise	glutaminase(GLS)	Homo sapiens
4927	11763322_s_at	turquoise	casein kinase 2 alpha 2(CSNK2A2)	Homo sapiens
4928	11763329_a_at	turquoise	docking protein 1(DOK1)	Homo sapiens
4930	11763336_at	turquoise	uncharacterized protein DKFZp586I1420(DKFZP586I1420)	Homo sapiens
4931	11763353_at	turquoise	NA	NA
4932	11763357_a_at	turquoise	transmembrane protein 170A(TMEM170A)	Homo sapiens
4933	11763359_at	turquoise	NA	NA
4934	11763365_x_at	turquoise	caspase recruitment domain family member 8(CARD8)	Homo sapiens

4935	11763367_at	turquoise	nucleic acid binding protein 1(NABP1)	Homo sapiens
4936	11763382_a_at	turquoise	uncharacterized LOC101928143(LOC101928143)	Homo sapiens
4937	11763403_a_at	turquoise	CUGBP, Elav-like family member 1(CELF1)	Homo sapiens
4939	11763426_a_at	turquoise	TRAF3 interacting protein 3(TRAF3IP3)	Homo sapiens
4940	11763446_s_at	turquoise	T cell receptor delta variable 3(TRDV3)	Homo sapiens
4941	11763447_x_at	turquoise	YME1 like 1 ATPase(YME1L1)	Homo sapiens
4942	11763472_x_at	turquoise	zinc finger CCCH-type containing 12D(ZC3H12D)	Homo sapiens
4943	11763494_x_at	turquoise	FERM domain containing 8(FRMD8)	Homo sapiens
4946	11763556_at	turquoise	eukaryotic translation initiation factor 4A1(EIF4A1)	Homo sapiens
4947	11763557_x_at	turquoise	T cell receptor beta constant 1(TRBC1)	Homo sapiens
4948	11763585_s_at	turquoise	thymopoietin(TMPO)	Homo sapiens
4949	11763605_a_at	turquoise	Wnt family member 11(WNT11)	Homo sapiens
4950	11763606_a_at	turquoise	vav guanine nucleotide exchange factor 1(VAV1)	Homo sapiens
4955	11763693_a_at	turquoise	glutaminase(GLS)	Homo sapiens
4957	11763704_a_at	turquoise	spermidine/spermine N1-acetyltransferase 1(SAT1)	Homo sapiens
4958	11763715_a_at	turquoise	granulysin(GNLY)	Homo sapiens
4959	11763726_a_at	turquoise	small nucleolar RNA, H/ACA box 70(SNORA70)	Homo sapiens
4960	11763735_a_at	turquoise	Fc receptor like 3(FCRL3)	Homo sapiens
4961	11763756_x_at	turquoise	granulysin(GNLY)	Homo sapiens
4962	11763776_a_at	turquoise	thymocyte selection associated family member 2(THEMIS2)	Homo sapiens
4963	11763862_x_at	turquoise	SH3 domain binding glutamate rich protein like(SH3BGRL)	Homo sapiens
4965	11763986_a_at	turquoise	apolipoprotein L1(APOL1)	Homo sapiens
4967	11764017_x_at	turquoise	aph-1 homolog A, gamma-secretase subunit(APH1A)	Homo sapiens
4968	11764037_s_at	turquoise	DnaJ heat shock protein family (Hsp40) member A1(DNAJA1)	Homo sapiens
4970	11764053_a_at	turquoise	recombination signal binding protein for immunoglobulin kappa J region(RBPJ)	Homo sapiens
4971	11764085_at	turquoise	NA	NA
4973	11764146_s_at	turquoise	motile sperm domain containing 2(MOSPD2)	Homo sapiens
4974	11764172_a_at	turquoise	uncharacterized LOC102724880(LOC102724880)	Homo sapiens
4975	11764239_s_at	turquoise	transforming growth factor beta regulator 1(TBRG1)	Homo sapiens
4976	11764248_s_at	turquoise	low density lipoprotein receptor class A domain containing 3(LDLRAD3)	Homo sapiens
4977	200003_PM_s_at	turquoise	microRNA 6805(MIR6805)	Homo sapiens
4978	200009_PM_at	turquoise	GDP dissociation inhibitor 2(GDI2)	Homo sapiens
4979	200011_PM_s_at	turquoise	ADP ribosylation factor 3(ARF3)	Homo sapiens
4980	200015_PM_s_at	turquoise	septin 2(SEPT2)	Homo sapiens
4981	200021_PM_at	turquoise	cofilin 1(CFL1)	Homo sapiens
4983	200033_PM_at	turquoise	microRNA 3064(MIR3064)	Homo sapiens
4984	200037_PM_s_at	turquoise	chromobox 3(CBX3)	Homo sapiens
4985	200044_PM_at	turquoise	glutamyl-tRNA amidotransferase subunit C(GATC)	Homo sapiens
4986	200057_PM_s_at	turquoise	non-POU domain containing, octamer-binding(NONO)	Homo sapiens
4987	200059_PM_s_at	turquoise	ras homolog family member A(RHOA)	Homo sapiens
4989	AFFX-BkGr-GC17_at	turquoise	NA	NA
4990	AFFX-BkGr-GC18_at	turquoise	NA	NA
4991	AFFX-BkGr-GC19_at	turquoise	NA	NA
4992	AFFX-BkGr-GC20_at	turquoise	NA	NA
4993	AFFX-BkGr-GC21_at	turquoise	NA	NA
4994	AFFX-BkGr-GC22_at	turquoise	NA	NA
4995	AFFX-HSAC07/X00351_3_at	turquoise	actin beta(ACTB)	Homo sapiens
4996	AFFX-HSAC07/X00351_M_at	turquoise	actin beta(ACTB)	Homo sapiens
4997	AFFX-HUMISGF3A/M97935_3_at	turquoise	signal transducer and activator of transcription 1(STAT1)	Homo sapiens
4998	AFFX-HUMISGF3A/M97935_5_at	turquoise	signal transducer and activator of transcription 1(STAT1)	Homo sapiens
4999	AFFX-HUMISGF3A/M97935_MA_at	turquoise	signal transducer and activator of transcription 1(STAT1)	Homo sapiens
5000	AFFX-HUMISGF3A/M97935_MB_at	turquoise	signal transducer and activator of transcription 1(STAT1)	Homo sapiens

Yellow Module

	gene	hcolor	Name	Species
1553	11726153_at	yellow	POU class 2 associating factor 1(POU2AF1)	Homo sapiens
2474	11735989_s_at	yellow	joining chain of multimeric IgA and IgM(JCHAIN)	Homo sapiens
2475	11735990_x_at	yellow	joining chain of multimeric IgA and IgM(JCHAIN)	Homo sapiens
3264	11745244_x_at	yellow	microRNA 8071-1(MIR8071-1)	Homo sapiens
3710	11750231_x_at	yellow	microRNA 8071-1(MIR8071-1)	Homo sapiens
3970	11753832_x_at	yellow	immunoglobulin kappa constant(IGKC)	Homo sapiens
3999	11754032_x_at	yellow	microRNA 8071-1(MIR8071-1)	Homo sapiens
4008	11754145_x_at	yellow	NA	NA
4198	11755382_x_at	yellow	immunoglobulin lambda variable 1-44(IGLV1-44)	Homo sapiens
4370	11756658_a_at	yellow	marginal zone B and B1 cell specific protein(MZB1)	Homo sapiens
4788	11759652_x_at	yellow	immunoglobulin kappa constant(IGKC)	Homo sapiens
4799	11759815_a_at	yellow	immunoglobulin heavy constant mu(IGHM)	Homo sapiens
4800	11759816_x_at	yellow	immunoglobulin heavy constant mu(IGHM)	Homo sapiens
4803	11759852_x_at	yellow	microRNA 8071-1(MIR8071-1)	Homo sapiens
4817	11760137_x_at	yellow	immunoglobulin heavy constant alpha 1(IGHA1)	Homo sapiens
4838	11760819_x_at	yellow	immunoglobulin heavy variable 4-31(IGHV4-31)	Homo sapiens
4844	11760929_x_at	yellow	microRNA 8071-1(MIR8071-1)	Homo sapiens
4856	11761467_x_at	yellow	immunoglobulin heavy constant gamma 3 (G3m marker)(IGHG3)	Homo sapiens
4873	11762091_x_at	yellow	immunoglobulin heavy locus(IGH)	Homo sapiens
4874	11762099_x_at	yellow	immunoglobulin heavy constant alpha 1(IGHA1)	Homo sapiens
4906	11763144_x_at	yellow	immunoglobulin kappa constant(IGKC)	Homo sapiens
4916	11763222_x_at	yellow	immunoglobulin kappa constant(IGKC)	Homo sapiens
4919	11763249_x_at	yellow	immunoglobulin heavy locus(IGH)	Homo sapiens
4920	11763253_x_at	yellow	NA	NA
4938	11763408_x_at	yellow	ankyrin repeat domain 36B pseudogene 2(ANKRD36BP2)	Homo sapiens
4945	11763550_x_at	yellow	immunoglobulin kappa constant(IGKC)	Homo sapiens
4952	11763640_x_at	yellow	immunoglobulin kappa constant(IGKC)	Homo sapiens
4953	11763663_x_at	yellow	immunoglobulin kappa constant(IGKC)	Homo sapiens
4954	11763684_x_at	yellow	immunoglobulin kappa constant(IGKC)	Homo sapiens
4969	11764046_x_at	yellow	immunoglobulin heavy constant mu(IGHM)	Homo sapiens
4972	11764130_s_at	yellow	immunoglobulin heavy locus(IGH)	Homo sapiens

Blue Module

	gene	hcolor	Name	Species
6	11715320_s_at	blue	titin(TTN)	Homo sapiens
7	11715339_at	blue	titin(TTN)	Homo sapiens
20	11715409_a_at	blue	peroxiredoxin 2(PRDX2)	Homo sapiens
21	11715410_x_at	blue	peroxiredoxin 2(PRDX2)	Homo sapiens
38	11715498_a_at	blue	OXA1L, mitochondrial inner membrane protein(OXA1L)	Homo sapiens
49	11715620_a_at	blue	crystallin alpha B(CRYAB)	Homo sapiens
55	11715655_s_at	blue	voltage dependent anion channel 1(VDAC1)	Homo sapiens
56	11715656_at	blue	cytochrome c oxidase subunit 5A(COX5A)	Homo sapiens
90	11715881_a_at	blue	death associated protein 3(DAP3)	Homo sapiens
91	11715883_x_at	blue	death associated protein 3(DAP3)	Homo sapiens
110	11715973_a_at	blue	mitochondrial fission regulator 1 like(MTFR1L)	Homo sapiens
145	11716202_a_at	blue	phosphofructokinase, muscle(PFKM)	Homo sapiens
146	11716205_a_at	blue	NADH:ubiquinone oxidoreductase subunit A8(NDUFA8)	Homo sapiens
147	11716211_a_at	blue	3-hydroxyisobutyrate dehydrogenase(HIBADH)	Homo sapiens
148	11716212_a_at	blue	3-hydroxyisobutyrate dehydrogenase(HIBADH)	Homo sapiens
172	11716380_a_at	blue	mitochondrial pyruvate carrier 2(MPC2)	Homo sapiens
173	11716381_x_at	blue	mitochondrial pyruvate carrier 2(MPC2)	Homo sapiens
176	11716392_s_at	blue	alcohol dehydrogenase 5 (class III), chi polypeptide(ADH5)	Homo sapiens
183	11716448_a_at	blue	nudix hydrolase 9(NUDT9)	Homo sapiens
187	11716468_s_at	blue	iron-sulfur cluster assembly 1(ISCA1)	Homo sapiens
197	11716562_at	blue	aminoacyl tRNA synthetase complex interacting multifunctional protein 2(AIMP2)	Homo sapiens
251	11716859_a_at	blue	electron transfer flavoprotein alpha subunit(ETFA)	Homo sapiens
304	11717297_at	blue	leucine rich pentatricopeptide repeat containing(LRPPRC)	Homo sapiens
329	11717430_s_at	blue	cytochrome c oxidase subunit 7A1(COX7A1)	Homo sapiens
338	11717483_a_at	blue	pyruvate dehydrogenase (lipoamide) alpha 1(PDHA1)	Homo sapiens
339	11717484_x_at	blue	pyruvate dehydrogenase (lipoamide) alpha 1(PDHA1)	Homo sapiens
341	11717499_at	blue	solute carrier family 25 member 36(SLC25A36)	Homo sapiens
407	11717936_a_at	blue	endoplasmic reticulum metallopeptidase 1(ERMP1)	Homo sapiens
426	11718035_at	blue	peptidylprolyl isomerase like 1(PPIL1)	Homo sapiens
442	11718117_a_at	blue	NFS1, cysteine desulfurase(NFS1)	Homo sapiens
454	11718207_a_at	blue	coenzyme Q6, monooxygenase(COQ6)	Homo sapiens
455	11718227_a_at	blue	BCS1 homolog, ubiquinol-cytochrome c reductase complex chaperone(BCS1L)	Homo sapiens
458	11718239_s_at	blue	cell cycle associated protein 1(CAPRIN1)	Homo sapiens
469	11718312_at	blue	acyl-CoA dehydrogenase, short/branched chain(ACADSB)	Homo sapiens
477	11718383_a_at	blue	apoptosis inducing factor, mitochondria associated 1(AIFM1)	Homo sapiens
485	11718426_a_at	blue	hydroxysteroid dehydrogenase like 2(HSDL2)	Homo sapiens
507	11718580_a_at	blue	coenzyme Q9(COQ9)	Homo sapiens
523	11718669_x_at	blue	mediator complex subunit 9(MED9)	Homo sapiens
543	11718837_x_at	blue	NADH:ubiquinone oxidoreductase core subunit S2(NDUFS2)	Homo sapiens
545	11718854_a_at	blue	mitochondrial ribosomal protein S30(MRPS30)	Homo sapiens
562	11718948_a_at	blue	membrane palmitoylated protein 5(MPP5)	Homo sapiens
579	11719065_x_at	blue	glyceronephosphate O-acyltransferase(GNPAT)	Homo sapiens
584	11719115_a_at	blue	glutamic-oxaloacetic transaminase 2(GOT2)	Homo sapiens
585	11719116_a_at	blue	glutamic-oxaloacetic transaminase 2(GOT2)	Homo sapiens
586	11719117_x_at	blue	glutamic-oxaloacetic transaminase 2(GOT2)	Homo sapiens
602	11719224_s_at	blue	coenzyme Q10A(COQ10A)	Homo sapiens
619	11719359_a_at	blue	aldehyde dehydrogenase 5 family member A1(ALDH5A1)	Homo sapiens
631	11719425_at	blue	family with sequence similarity 220 member A(FAM220A)	Homo sapiens
692	11719876_at	blue	creatine kinase, mitochondrial 2(CKMT2)	Homo sapiens
743	11720191_s_at	blue	NADH:ubiquinone oxidoreductase complex assembly factor 4(NDUFAF4)	Homo sapiens
757	11720238_at	blue	mitogen-activated protein kinase 9(MAPK9)	Homo sapiens
827	11720673_a_at	blue	protein phosphatase, Mg2+/Mn2+ dependent 1A(PPM1A)	Homo sapiens
828	11720674_a_at	blue	protein phosphatase, Mg2+/Mn2+ dependent 1A(PPM1A)	Homo sapiens
841	11720770_a_at	blue	peptidylprolyl isomerase F(PPIF)	Homo sapiens
842	11720771_x_at	blue	peptidylprolyl isomerase F(PPIF)	Homo sapiens
845	11720787_a_at	blue	dynein cytoplasmic 1 light intermediate chain 1(DYNC1LI1)	Homo sapiens
888	11721028_at	blue	malonyl-CoA decarboxylase(MLYCD)	Homo sapiens
889	11721029_a_at	blue	phosphatidylinositol-4-phosphate 5-kinase type 1 beta(PIP5K1B)	Homo sapiens
890	11721030_a_at	blue	phosphatidylinositol-4-phosphate 5-kinase type 1 beta(PIP5K1B)	Homo sapiens
941	11721472_a_at	blue	holocytochrome c synthase(HCCS)	Homo sapiens
1028	11722004_s_at	blue	cytochrome c, somatic(CYCS)	Homo sapiens
1033	11722028_at	blue	ubiquitin conjugating enzyme E2 G1(UBE2G1)	Homo sapiens
1050	11722173_x_at	blue	ubiquinol-cytochrome c reductase complex assembly factor 1(UQCC1)	Homo sapiens
1081	11722364_at	blue	cytochrome c oxidase subunit 6A2(COX6A2)	Homo sapiens
1103	11722482_x_at	blue	mitochondrial fission factor(MFF)	Homo sapiens
1109	11722555_s_at	blue	hydroxyacyl-CoA dehydrogenase(HADH)	Homo sapiens
1131	11722795_a_at	blue	glycerophosphodiester phosphodiesterase 1(GDE1)	Homo sapiens
1136	11722841_s_at	blue	transcription factor B2, mitochondrial(TFB2M)	Homo sapiens
1146	11722911_at	blue	ubiquitin specific peptidase 13 (isopeptidase T-3)(USP13)	Homo sapiens
1147	11722912_at	blue	ubiquitin specific peptidase 13 (isopeptidase T-3)(USP13)	Homo sapiens
1148	11722913_s_at	blue	ubiquitin specific peptidase 13 (isopeptidase T-3)(USP13)	Homo sapiens
1206	11723263_a_at	blue	antagonist of mitotic exit network 1 homolog(AMN1)	Homo sapiens
1219	11723404_at	blue	PPARG coactivator 1 alpha(PPARGC1A)	Homo sapiens
1240	11723566_a_at	blue	calpain 7(CAPN7)	Homo sapiens
1338	11724350_s_at	blue	protein phosphatase 1 regulatory inhibitor subunit 14C(PPP1R14C)	Homo sapiens
1382	11724765_a_at	blue	FAST kinase domains 2(FASTKD2)	Homo sapiens
1425	11725084_a_at	blue	ubiquitin C-terminal hydrolase L5(UCHL5)	Homo sapiens
1442	11725237_a_at	blue	RNA binding protein with multiple splicing 2(RBPMS2)	Homo sapiens
1482	11725525_at	blue	desmoglein 2(DSG2)	Homo sapiens
1488	11725620_a_at	blue	pyruvate dehydrogenase (lipoamide) beta(PDHB)	Homo sapiens

1498	11725682_x_at	blue	MRS2, magnesium transporter(MRS2)	Homo sapiens
1521	11725894_at	blue	taxilin beta(TXLNB)	Homo sapiens
1528	11725957_x_at	blue	translocase of inner mitochondrial membrane 17 homolog A (yeast)(TIMM17A)	Homo sapiens
1542	11726035_at	blue	phosphoglycerate mutase 2(PGAM2)	Homo sapiens
1547	11726087_at	blue	heat shock protein family B (small) member 3(HSPB3)	Homo sapiens
1550	11726104_a_at	blue	protein phosphatase 2 regulatory subunit B''alpha(PPP2R3A)	Homo sapiens
1609	11726496_at	blue	ryanodine receptor 2(RYR2)	Homo sapiens
1630	11726676_a_at	blue	apolipoprotein B mRNA editing enzyme catalytic subunit 2(APOBEC2)	Homo sapiens
1662	11726974_a_at	blue	PERP, TP53 apoptosis effector(PERP)	Homo sapiens
1663	11726975_x_at	blue	PERP, TP53 apoptosis effector(PERP)	Homo sapiens
1700	11727417_a_at	blue	ATP5S like(ATP5SL)	Homo sapiens
1737	11727791_s_at	blue	dihydrolipoamide S-acetyltransferase(DLAT)	Homo sapiens
1738	11727792_at	blue	dihydrolipoamide S-acetyltransferase(DLAT)	Homo sapiens
1739	11727793_x_at	blue	dihydrolipoamide S-acetyltransferase(DLAT)	Homo sapiens
1767	11728003_a_at	blue	chromosome 4 open reading frame 48(C4orf48)	Homo sapiens
1817	11728392_a_at	blue	LDL receptor related protein 12(LRP12)	Homo sapiens
1840	11728566_x_at	blue	family with sequence similarity 210 member A(FAM210A)	Homo sapiens
1866	11728823_a_at	blue	solute carrier family 25 member 3(SLC25A3)	Homo sapiens
1867	11728824_s_at	blue	solute carrier family 25 member 3(SLC25A3)	Homo sapiens
1868	11728841_a_at	blue	pleckstrin homology domain containing A5(PLEKHA5)	Homo sapiens
1894	11729126_a_at	blue	solute carrier family 25 member 3(SLC25A3)	Homo sapiens
1895	11729127_x_at	blue	solute carrier family 25 member 3(SLC25A3)	Homo sapiens
1910	11729237_a_at	blue	methylcrotonoyl-CoA carboxylase 2(MCCC2)	Homo sapiens
1917	11729312_at	blue	nicotinamide nucleotide adenylyltransferase 1(NMNAT1)	Homo sapiens
1943	11729621_at	blue	chloride voltage-gated channel 4(CLCN4)	Homo sapiens
1948	11729659_s_at	blue	transmembrane protein 69(TMEM69)	Homo sapiens
1977	11730010_a_at	blue	microtubule associated tumor suppressor candidate 2(MTUS2)	Homo sapiens
1987	11730146_at	blue	kelch like family member 31(KLHL31)	Homo sapiens
1990	11730159_at	blue	RNA binding motif protein 20(RBM20)	Homo sapiens
1996	11730212_a_at	blue	mitochondrial ribosomal protein S7(MRPS7)	Homo sapiens
1997	11730213_s_at	blue	mitochondrial ribosomal protein S7(MRPS7)	Homo sapiens
2026	11730542_x_at	blue	acetyl-CoA acyltransferase 1(ACAA1)	Homo sapiens
2040	11730784_x_at	blue	origin recognition complex subunit 4(ORC4)	Homo sapiens
2048	11730835_a_at	blue	metaxin 2(MTX2)	Homo sapiens
2067	11730958_a_at	blue	coiled-coil-helix-coiled-coil-helix domain containing 4(CHCHD4)	Homo sapiens
2101	11731417_a_at	blue	NADH:ubiquinone oxidoreductase core subunit S1(NDUFS1)	Homo sapiens
2113	11731557_at	blue	synaptopodin 2(SYNPO2)	Homo sapiens
2116	11731614_x_at	blue	electron transfer flavoprotein alpha subunit(ETFA)	Homo sapiens
2146	11731932_a_at	blue	KN motif and ankyrin repeat domains 1(KANK1)	Homo sapiens
2164	11732193_a_at	blue	myeloid leukemia factor 1(MLF1)	Homo sapiens
2200	11732501_a_at	blue	tropomodulin 1(TMOD1)	Homo sapiens
2204	11732524_a_at	blue	carnitine palmitoyltransferase 1B(CPT1B)	Homo sapiens
2229	11732857_a_at	blue	THAP domain containing 1(THAP1)	Homo sapiens
2249	11733032_at	blue	RNA binding motif protein 18(RBM18)	Homo sapiens
2275	11733255_a_at	blue	carnitine palmitoyltransferase 1B(CPT1B)	Homo sapiens
2294	11733523_at	blue	protein phosphatase 1 regulatory subunit 3A(PPP1R3A)	Homo sapiens
2309	11733671_a_at	blue	EYA transcriptional coactivator and phosphatase 1(EYA1)	Homo sapiens
2323	11733792_a_at	blue	solute carrier family 25 member 12(SLC25A12)	Homo sapiens
2329	11733860_a_at	blue	cytoplasmic linker associated protein 2(CLASP2)	Homo sapiens
2384	11734652_s_at	blue	ATP synthase, H+ transporting, mitochondrial Fo complex subunit B1(ATP5F1)	Homo sapiens
2385	11734653_x_at	blue	ATP synthase, H+ transporting, mitochondrial Fo complex subunit B1(ATP5F1)	Homo sapiens
2408	11734862_a_at	blue	trimethyllysine hydroxylase, epsilon(TMLHE)	Homo sapiens
2426	11735040_at	blue	ribosomal protein L3 like(RPL3L)	Homo sapiens
2429	11735097_x_at	blue	MLX, MAX dimerization protein(MLX)	Homo sapiens
2451	11735362_s_at	blue	AKT interacting protein(AKTIP)	Homo sapiens
2501	11736296_at	blue	solute carrier family 5 member 1(SLC5A1)	Homo sapiens
2516	11736448_a_at	blue	gypsy retrotransposon integrase 1(GIN1)	Homo sapiens
2533	11736589_a_at	blue	de-etiolated homolog 1 (Arabidopsis)(DET1)	Homo sapiens
2549	11736770_a_at	blue	ADAM metallopeptidase domain 23(ADAM23)	Homo sapiens
2552	11736877_a_at	blue	DTW domain containing 2(DTWD2)	Homo sapiens
2554	11736965_at	blue	F-box protein 40(FBXO40)	Homo sapiens
2570	11737243_a_at	blue	fibronectin type III and SPRY domain containing 2(FSD2)	Homo sapiens
2589	11737811_a_at	blue	nicotinamide nucleotide transhydrogenase(NNT)	Homo sapiens
2603	11738054_a_at	blue	integrin subunit beta 1 binding protein 2(ITGB1BP2)	Homo sapiens
2647	11739059_a_at	blue	OCIA domain containing 1(OCIAD1)	Homo sapiens
2669	11739345_x_at	blue	calmegin(CLGN)	Homo sapiens
2680	11739437_at	blue	PTC7 protein phosphatase homolog(PPTC7)	Homo sapiens
2682	11739453_a_at	blue	malic enzyme 2(ME2)	Homo sapiens
2692	11739501_a_at	blue	desmoplakin(DSP)	Homo sapiens
2693	11739506_a_at	blue	LON peptidase N-terminal domain and ring finger 2(LONRF2)	Homo sapiens
2695	11739508_a_at	blue	N(alpha)-acetyltransferase 30, NatC catalytic subunit(NAA30)	Homo sapiens
2724	11739747_a_at	blue	kelch like family member 7(KLHL7)	Homo sapiens
2730	11739811_a_at	blue	sirtuin 3(SIRT3)	Homo sapiens
2750	11740043_a_at	blue	mitochondrial methionyl-tRNA formyltransferase(MTFMT)	Homo sapiens
2751	11740044_x_at	blue	mitochondrial methionyl-tRNA formyltransferase(MTFMT)	Homo sapiens
2780	11740513_a_at	blue	phosphodiesterase 4D(PDE4D)	Homo sapiens
2792	11740745_a_at	blue	OPA1, mitochondrial dynamin like GTPase(OPA1)	Homo sapiens
2793	11740746_s_at	blue	OPA1, mitochondrial dynamin like GTPase(OPA1)	Homo sapiens
2820	11740990_x_at	blue	hyaluronoglucosaminidase 1(HYAL1)	Homo sapiens
2828	11741039_a_at	blue	death associated protein 3(DAP3)	Homo sapiens
2829	11741040_x_at	blue	death associated protein 3(DAP3)	Homo sapiens
2839	11741148_a_at	blue	peroxiredoxin 2(PRDX2)	Homo sapiens

2858	11741402_a_at	blue	chromosome 21 open reading frame 33(C21orf33)	Homo sapiens
2859	11741428_a_at	blue	RNA binding protein, fox-1 homolog 1(RBFOX1)	Homo sapiens
2861	11741438_a_at	blue	calmegin(CLGN)	Homo sapiens
2878	11741650_a_at	blue	LDL receptor related protein 12(LRP12)	Homo sapiens
2881	11741686_a_at	blue	family with sequence similarity 185 member A(FAM185A)	Homo sapiens
2886	11741749_x_at	blue	leucine rich repeat containing 2(LRRC2)	Homo sapiens
2906	11742035_a_at	blue	phospholipase C like 1(PLCL1)	Homo sapiens
2921	11742273_a_at	blue	brain and reproductive organ-expressed (TNFRSF1A modulator)(BRE)	Homo sapiens
2949	11742798_a_at	blue	chloride voltage-gated channel 3(CLCN3)	Homo sapiens
2969	11742943_a_at	blue	creatine kinase, M-type(CKM)	Homo sapiens
2974	11742973_at	blue	acetyl-CoA acetyltransferase 1(ACAT1)	Homo sapiens
2975	11742981_a_at	blue	fatty acid binding protein 3(FABP3)	Homo sapiens
2985	11743015_a_at	blue	disco interacting protein 2 homolog C(DIP2C)	Homo sapiens
2988	11743048_s_at	blue	aconitase 2(ACO2)	Homo sapiens
2994	11743076_a_at	blue	protein activator of interferon induced protein kinase EIF2AK2(PRKRA)	Homo sapiens
3036	11743505_a_at	blue	asparaginyl-tRNA synthetase 2, mitochondrial (putative)(NARS2)	Homo sapiens
3037	11743517_a_at	blue	nicotinamide nucleotide transhydrogenase(NNT)	Homo sapiens
3055	11743648_a_at	blue	DDB1 and CUL4 associated factor 6(DCAF6)	Homo sapiens
3064	11743728_a_at	blue	coiled-coil domain containing 47(CCDC47)	Homo sapiens
3083	11743887_s_at	blue	S-phase response (cyclin related)(SPHAR)	Homo sapiens
3093	11743979_a_at	blue	ubiquinol-cytochrome c reductase core protein I(UQCRC1)	Homo sapiens
3108	11744059_a_at	blue	ubiquinol-cytochrome c reductase core protein II(UQCRC2)	Homo sapiens
3109	11744060_s_at	blue	ubiquinol-cytochrome c reductase core protein II(UQCRC2)	Homo sapiens
3125	11744166_at	blue	fumarate hydratase(FH)	Homo sapiens
3131	11744182_a_at	blue	phenylalanyl-tRNA synthetase 2, mitochondrial(FARS2)	Homo sapiens
3144	11744261_a_at	blue	solute carrier family 25 member 11(SLC25A11)	Homo sapiens
3148	11744275_a_at	blue	WD repeat domain 12(WDR12)	Homo sapiens
3174	11744462_x_at	blue	PCI domain containing 2(PCID2)	Homo sapiens
3194	11744632_a_at	blue	COX11, cytochrome c oxidase copper chaperone(COX11)	Homo sapiens
3233	11744901_s_at	blue	NADH:ubiquinone oxidoreductase core subunit S1(NDUFS1)	Homo sapiens
3253	11745163_s_at	blue	hydroxyacyl-CoA dehydrogenase/3-ketoacyl-CoA thiolase/enoyl-CoA hydratase (trifunctional protein), beta subunit(HADHB)	Homo sapiens
3256	11745185_s_at	blue	mitochondrial fission factor(MFF)	Homo sapiens
3260	11745230_a_at	blue	T-cell activation inhibitor, mitochondrial(TCAIM)	Homo sapiens
3284	11745462_a_at	blue	peroxiredoxin 2(PRDX2)	Homo sapiens
3295	11745517_s_at	blue	succinate dehydrogenase complex iron sulfur subunit B(SDHB)	Homo sapiens
3297	11745527_a_at	blue	heat shock transcription factor 2(HSF2)	Homo sapiens
3310	11745812_x_at	blue	isocitrate dehydrogenase 3 (NAD(+)) beta(IDH3B)	Homo sapiens
3315	11745858_a_at	blue	ATP5S like(ATP5SL)	Homo sapiens
3316	11745859_x_at	blue	ATP5S like(ATP5SL)	Homo sapiens
3318	11745869_a_at	blue	mitochondrial pyruvate carrier 2(MPC2)	Homo sapiens
3349	11746084_a_at	blue	protein-L-isoaspartate (D-aspartate) O-methyltransferase(PCMT1)	Homo sapiens
3398	11746655_a_at	blue	acetyl-CoA acyltransferase 1(ACAA1)	Homo sapiens
3411	11746844_a_at	blue	transmembrane protein 143(TMEM143)	Homo sapiens
3412	11746845_a_at	blue	transmembrane protein 143(TMEM143)	Homo sapiens
3420	11746918_a_at	blue	L-2-hydroxyglutarate dehydrogenase(L2HGDH)	Homo sapiens
3429	11747013_a_at	blue	pyrophosphatase (inorganic) 2(PPA2)	Homo sapiens
3437	11747126_x_at	blue	ADP-ribosyltransferase 3(ART3)	Homo sapiens
3453	11747313_a_at	blue	OXA1L, mitochondrial inner membrane protein(OXA1L)	Homo sapiens
3454	11747315_a_at	blue	ectonucleoside triphosphate diphosphohydrolase 6 (putative)(ENTPD6)	Homo sapiens
3455	11747345_s_at	blue	acyl-CoA dehydrogenase, C-4 to C-12 straight chain(ACADM)	Homo sapiens
3456	11747346_x_at	blue	acyl-CoA dehydrogenase, C-4 to C-12 straight chain(ACADM)	Homo sapiens
3458	11747401_s_at	blue	COP9 signalosome subunit 7A(COPS7A)	Homo sapiens
3476	11747533_a_at	blue	G-rich RNA sequence binding factor 1(GRSF1)	Homo sapiens
3479	11747546_a_at	blue	phosphatidylinositol-4-phosphate 5-kinase type 1 beta(PIP5K1B)	Homo sapiens
3480	11747559_a_at	blue	PDZ domain containing ring finger 3(PDZRN3)	Homo sapiens
3481	11747589_a_at	blue	glutamic-oxaloacetic transaminase 2(GOT2)	Homo sapiens
3497	11747677_a_at	blue	basic leucine zipper and W2 domains 2(BZW2)	Homo sapiens
3506	11747772_x_at	blue	pyruvate dehydrogenase (lipoamide) alpha 1(PDHA1)	Homo sapiens
3513	11747902_a_at	blue	echinoderm microtubule associated protein like 1(EML1)	Homo sapiens
3521	11748014_s_at	blue	voltage dependent anion channel 3(VDAC3)	Homo sapiens
3522	11748015_x_at	blue	voltage dependent anion channel 3(VDAC3)	Homo sapiens
3528	11748148_a_at	blue	coenzyme Q6, monooxygenase(COQ6)	Homo sapiens
3531	11748216_a_at	blue	staufen double-stranded RNA binding protein 2(STAU2)	Homo sapiens
3536	11748253_a_at	blue	solute carrier family 5 member 1(SLC5A1)	Homo sapiens
3538	11748273_a_at	blue	Raf-1 proto-oncogene, serine/threonine kinase(RAF1)	Homo sapiens
3543	11748325_x_at	blue	mitochondrial ribosomal protein S22(MRPS22)	Homo sapiens
3553	11748423_a_at	blue	phosphoglucomutase 1(PGM1)	Homo sapiens
3557	11748509_s_at	blue	voltage dependent anion channel 2(VDAC2)	Homo sapiens
3560	11748541_s_at	blue	protease associated domain containing 1(PRADC1)	Homo sapiens
3562	11748553_x_at	blue	ubiquinol-cytochrome c reductase complex assembly factor 1(UQCC1)	Homo sapiens
3565	11748583_x_at	blue	ADP-ribosyltransferase 3(ART3)	Homo sapiens
3567	11748620_x_at	blue	succinate-CoA ligase ADP-forming beta subunit(SUCLA2)	Homo sapiens
3570	11748671_a_at	blue	cullin 4A(CUL4A)	Homo sapiens
3580	11748839_a_at	blue	NFS1, cysteine desulfurase(NFS1)	Homo sapiens
3583	11748854_a_at	blue	RNA pseudouridylate synthase domain containing 4(RPUSD4)	Homo sapiens
3585	11748860_a_at	blue	apolipoprotein B mRNA editing enzyme catalytic subunit 2(APOBEC2)	Homo sapiens
3586	11748874_a_at	blue	mitochondrial ribosomal protein S25(MRPS25)	Homo sapiens
3606	11749144_s_at	blue	electron transfer flavoprotein dehydrogenase(ETFDH)	Homo sapiens
3610	11749201_a_at	blue	IQCJ-SCHIP1 readthrough(IQCJ-SCHIP1)	Homo sapiens
3615	11749261_a_at	blue	tubulin folding cofactor E(TBCE)	Homo sapiens
3621	11749311_a_at	blue	GrpE like 1, mitochondrial(GRPEL1)	Homo sapiens
3625	11749369_a_at	blue	VAMP associated protein B and C(VAPB)	Homo sapiens

3629	11749392_a_at	blue	ATP synthase, H+ transporting, mitochondrial F1 complex, gamma polypeptide 1(ATP5C1)	Homo sapiens
3654	11749690_a_at	blue	LARGE xylosyl- and glucuronyltransferase 1(LARGE1)	Homo sapiens
3655	11749697_a_at	blue	NADH:ubiquinone oxidoreductase core subunit S1(NDUFS1)	Homo sapiens
3657	11749722_a_at	blue	NADH:ubiquinone oxidoreductase core subunit S1(NDUFS1)	Homo sapiens
3678	11749914_a_at	blue	single-pass membrane protein with coiled-coil domains 1(SMCO1)	Homo sapiens
3687	11750038_a_at	blue	mitochondrial trans-2-enoyl-CoA reductase(MECR)	Homo sapiens
3688	11750044_a_at	blue	NFS1, cysteine desulfurase(NFS1)	Homo sapiens
3690	11750059_a_at	blue	MLX, MAX dimerization protein(MLX)	Homo sapiens
3692	11750103_a_at	blue	hydroxyacyl-CoA dehydrogenase(HADH)	Homo sapiens
3695	11750135_a_at	blue	monoamine oxidase B(MAOB)	Homo sapiens
3733	11750594_a_at	blue	phospholipase C like 1(PLCL1)	Homo sapiens
3759	11750914_a_at	blue	G1 to S phase transition 2(GSPT2)	Homo sapiens
3762	11751002_x_at	blue	dihydrolipoamide S-acetyltransferase(DLAT)	Homo sapiens
3779	11751305_a_at	blue	beta-1,4-glucuronyltransferase 1(B4GAT1)	Homo sapiens
3787	11751440_a_at	blue	microtubule associated tumor suppressor candidate 2(MTUS2)	Homo sapiens
3795	11751585_a_at	blue	ankyrin repeat and SOCS box containing 8(ASB8)	Homo sapiens
3802	11751616_a_at	blue	lactamase beta 2(LACTB2)	Homo sapiens
3813	11751702_a_at	blue	PATJ, crumbs cell polarity complex component(PATJ)	Homo sapiens
3820	11751776_a_at	blue	prohibitin(PHB)	Homo sapiens
3828	11751861_x_at	blue	hyaluronoglucosaminidase 1(HYAL1)	Homo sapiens
3879	11752649_s_at	blue	aconitase 2(ACO2)	Homo sapiens
3885	11752747_a_at	blue	MRS2, magnesium transporter(MRS2)	Homo sapiens
3886	11752748_x_at	blue	MRS2, magnesium transporter(MRS2)	Homo sapiens
3904	11752915_a_at	blue	ryanodine receptor 2(RYR2)	Homo sapiens
3906	11752919_x_at	blue	acyl-CoA dehydrogenase, C-4 to C-12 straight chain(ACADM)	Homo sapiens
3911	11752964_a_at	blue	solute carrier family 25 member 20(SLC25A20)	Homo sapiens
3938	11753345_a_at	blue	cutC copper transporter(CUTC)	Homo sapiens
3944	11753467_a_at	blue	aminomethyltransferase(AMT)	Homo sapiens
3945	11753480_a_at	blue	reticulon 4 interacting protein 1(RTN4IP1)	Homo sapiens
3947	11753505_a_at	blue	post-GPI attachment to proteins 2(PGAP2)	Homo sapiens
3958	11753628_a_at	blue	abhydrolase domain containing 11(ABHD11)	Homo sapiens
3968	11753803_x_at	blue	cytochrome c, somatic(CYCS)	Homo sapiens
3981	11753896_a_at	blue	pyruvate dehydrogenase (lipoamide) alpha 1(PDHA1)	Homo sapiens
3996	11754023_a_at	blue	cytochrome c oxidase subunit 7A2 like(COX7A2L)	Homo sapiens
3997	11754024_a_at	blue	solute carrier family 25 member 11(SLC25A11)	Homo sapiens
3998	11754027_x_at	blue	cytochrome c1(CYC1)	Homo sapiens
4027	11754272_x_at	blue	small nuclear ribonucleoprotein polypeptide N(SNRPN)	Homo sapiens
4047	11754381_a_at	blue	tropomodulin 1(TMOD1)	Homo sapiens
4063	11754510_s_at	blue	voltage dependent anion channel 3(VDAC3)	Homo sapiens
4073	11754598_s_at	blue	DnaJ heat shock protein family (Hsp40) member A3(DNAJA3)	Homo sapiens
4115	11754844_x_at	blue	COP9 signalosome subunit 5(COPS5)	Homo sapiens
4123	11754888_a_at	blue	adenylosuccinate synthase like 1(ADSSL1)	Homo sapiens
4141	11755018_a_at	blue	protein phosphatase 1 catalytic subunit gamma(PPP1CC)	Homo sapiens
4174	11755266_x_at	blue	succinate-CoA ligase ADP-forming beta subunit(SUCLA2)	Homo sapiens
4206	11755425_a_at	blue	family with sequence similarity 179 member B(FAM179B)	Homo sapiens
4249	11755788_a_at	blue	kinesin family member 21A(KIF21A)	Homo sapiens
4268	11755960_a_at	blue	leucine rich repeat containing 39(LRRC39)	Homo sapiens
4270	11755980_x_at	blue	solute carrier family 25 member 3(SLC25A3)	Homo sapiens
4282	11756096_a_at	blue	solute carrier family 25 member 4(SLC25A4)	Homo sapiens
4283	11756099_a_at	blue	lactate dehydrogenase B(LDHB)	Homo sapiens
4297	11756183_x_at	blue	solute carrier family 25 member 11(SLC25A11)	Homo sapiens
4303	11756232_s_at	blue	voltage dependent anion channel 2(VDAC2)	Homo sapiens
4304	11756239_s_at	blue	mitochondrial ribosomal protein S35(MRPS35)	Homo sapiens
4314	11756296_x_at	blue	mitochondrial fission factor(MFF)	Homo sapiens
4328	11756367_x_at	blue	solute carrier family 25 member 4(SLC25A4)	Homo sapiens
4345	11756499_a_at	blue	kelch domain containing 2(KLHDC2)	Homo sapiens
4347	11756501_a_at	blue	transmembrane protein 38B(TMEM38B)	Homo sapiens
4358	11756593_a_at	blue	uroporphyrinogen decarboxylase(UROD)	Homo sapiens
4378	11756722_a_at	blue	stomatin like 2(STOML2)	Homo sapiens
4416	11757011_a_at	blue	SPRY domain containing 7(SPRYD7)	Homo sapiens
4418	11757030_at	blue	enoyl-CoA hydratase 1(ECH1)	Homo sapiens
4482	11757443_s_at	blue	translocase of inner mitochondrial membrane 21(TIMM21)	Homo sapiens
4488	11757465_s_at	blue	glioblastoma amplified sequence(GBAS)	Homo sapiens
4499	11757524_x_at	blue	cytochrome c1(CYC1)	Homo sapiens
4503	11757538_a_at	blue	glutamic-oxaloacetic transaminase 1(GOT1)	Homo sapiens
4563	11757873_x_at	blue	ATP synthase, H+ transporting, mitochondrial F1 complex, beta polypeptide(ATP5B)	Homo sapiens
4601	11758056_s_at	blue	threonyl-tRNA synthetase like 2(TARSL2)	Homo sapiens
4608	11758101_s_at	blue	eukaryotic translation initiation factor 4E binding protein 2(EIF4EBP2)	Homo sapiens
4612	11758132_s_at	blue	trimethyllysine hydroxylase, epsilon(TMLHE)	Homo sapiens
4622	11758190_s_at	blue	succinate dehydrogenase complex subunit D(SDHD)	Homo sapiens
4629	11758218_s_at	blue	motile sperm domain containing 1(MOSPD1)	Homo sapiens
4636	11758244_s_at	blue	A-kinase anchoring protein 6(AKAP6)	Homo sapiens
4637	11758248_s_at	blue	succinate dehydrogenase complex subunit D(SDHD)	Homo sapiens
4650	11758311_s_at	blue	succinate dehydrogenase complex subunit D(SDHD)	Homo sapiens
4653	11758330_x_at	blue	succinate dehydrogenase complex subunit D(SDHD)	Homo sapiens
4655	11758335_s_at	blue	ferredoxin 1(FDX1)	Homo sapiens
4664	11758402_s_at	blue	family with sequence similarity 179 member B(FAM179B)	Homo sapiens
4707	11758755_at	blue	mitochondrial pyruvate carrier 2(MPC2)	Homo sapiens
4745	11759179_at	blue	transmembrane protein 38B(TMEM38B)	Homo sapiens
4748	11759288_at	blue	thioredoxin related transmembrane protein 4(TMX4)	Homo sapiens
4758	11759417_s_at	blue	heat shock protein family A (Hsp70) member 9(HSPA9)	Homo sapiens
4768	11759519_a_at	blue	succinate-CoA ligase ADP-forming beta subunit(SUCLA2)	Homo sapiens

4854	11761446_a_at	blue	staufen double-stranded RNA binding protein 2(STAU2)	Homo sapiens
4929	11763335_a_at	blue	malate dehydrogenase 2(MDH2)	Homo sapiens
4944	11763522_a_at	blue	ryanodine receptor 2(RYR2)	Homo sapiens
4951	11763627_a_at	blue	solute carrier family 25 member 10(SLC25A10)	Homo sapiens
4956	11763699_a_at	blue	cytoplasmic linker associated protein 2(CLASP2)	Homo sapiens
4982	200030_PM_s_at	blue	solute carrier family 25 member 3(SLC25A3)	Homo sapiens

Brown Module

	gene	hcolor	Name	Species
9	11715349_a_at	brown	cytochrome c oxidase subunit 7C(COX7C)	Homo sapiens
14	11715369_s_at	brown	NDUFA4, mitochondrial complex associated(NDUFA4)	Homo sapiens
16	11715377_x_at	brown	cytochrome c oxidase subunit 4I1(COX4I1)	Homo sapiens
27	11715458_a_at	brown	cytochrome c oxidase subunit 8A(COX8A)	Homo sapiens
28	11715459_x_at	brown	cytochrome c oxidase subunit 8A(COX8A)	Homo sapiens
37	11715497_a_at	brown	OXA1L, mitochondrial inner membrane protein(OXA1L)	Homo sapiens
48	11715616_a_at	brown	NADH:ubiquinone oxidoreductase subunit AB1(NDUFAB1)	Homo sapiens
66	11715694_at	brown	cytochrome c oxidase subunit 7B(COX7B)	Homo sapiens
67	11715695_x_at	brown	cytochrome c oxidase subunit 7B(COX7B)	Homo sapiens
74	11715744_a_at	brown	ATP synthase, H+ transporting, mitochondrial Fo complex subunit F6(ATP5J)	Homo sapiens
77	11715772_x_at	brown	mitochondrial ribosomal protein L13(MRPL13)	Homo sapiens
86	11715867_x_at	brown	mitochondrial ribosomal protein L41(MRPL41)	Homo sapiens
89	11715874_s_at	brown	ATP synthase, H+ transporting, mitochondrial Fo complex subunit D(ATP5H)	Homo sapiens
95	11715899_a_at	brown	succinate-CoA ligase alpha subunit(SUCLG1)	Homo sapiens
111	11715988_x_at	brown	ATP synthase, H+ transporting, mitochondrial Fo complex subunit C1 (subunit 9)(ATP5G1)	Homo sapiens
113	11715995_at	brown	mitochondrial ribosomal protein L16(MRPL16)	Homo sapiens
114	11715997_a_at	brown	NADH:ubiquinone oxidoreductase subunit C1(NDUFC1)	Homo sapiens
119	11716029_a_at	brown	NADH:ubiquinone oxidoreductase subunit B9(NDUFB9)	Homo sapiens
120	11716030_x_at	brown	NADH:ubiquinone oxidoreductase subunit B9(NDUFB9)	Homo sapiens
125	11716047_a_at	brown	chromosome 21 open reading frame 33(C21orf33)	Homo sapiens
127	11716061_a_at	brown	family with sequence similarity 162 member A(FAM162A)	Homo sapiens
136	11716116_at	brown	NADH:ubiquinone oxidoreductase subunit S6(NDUFS6)	Homo sapiens
150	11716243_a_at	brown	protein phosphatase 1 regulatory subunit 16A(PPP1R16A)	Homo sapiens
162	11716315_at	brown	mitochondrial ribosomal protein L15(MRPL15)	Homo sapiens
165	11716340_a_at	brown	NADH:ubiquinone oxidoreductase subunit S4(NDUFS4)	Homo sapiens
166	11716350_a_at	brown	HSPE1-MOB4 readthrough(HSPE1-MOB4)	Homo sapiens
167	11716355_s_at	brown	NADH:ubiquinone oxidoreductase subunit A11(NDUFA11)	Homo sapiens
179	11716415_s_at	brown	NADH:ubiquinone oxidoreductase subunit B8(NDUFB8)	Homo sapiens
181	11716432_a_at	brown	MNAT1, CDK activating kinase assembly factor(MNAT1)	Homo sapiens
182	11716442_a_at	brown	RNA polymerase II subunit L(POLR2L)	Homo sapiens
189	11716472_a_at	brown	Raf-1 proto-oncogene, serine/threonine kinase(RAF1)	Homo sapiens
193	11716519_s_at	brown	short chain dehydrogenase/reductase family 39U member 1(SDR39U1)	Homo sapiens
198	11716563_s_at	brown	aminoacyl tRNA synthetase complex interacting multifunctional protein 2(AIMP2)	Homo sapiens
203	11716606_s_at	brown	stomatin like 2(STOML2)	Homo sapiens
204	11716617_at	brown	mitochondrial ribosomal protein L36(MRPL36)	Homo sapiens
222	11716700_at	brown	SNAP associated protein(SNAPIN)	Homo sapiens
258	11716890_a_at	brown	transmembrane protein 261(TMEM261)	Homo sapiens
260	11716900_at	brown	ADP ribosylation factor like GTPase 3(ARL3)	Homo sapiens
266	11716935_a_at	brown	superoxide dismutase 1, soluble(SOD1)	Homo sapiens
271	11716953_at	brown	transmembrane protein 126A(TMEM126A)	Homo sapiens
275	11716984_a_at	brown	paroxysmal nonkinesigenic dyskinesia(PNKD)	Homo sapiens
281	11717060_a_at	brown	2,4-dienoyl-CoA reductase 1, mitochondrial(DECR1)	Homo sapiens
296	11717251_s_at	brown	NADH:ubiquinone oxidoreductase subunit B11(NDUFB11)	Homo sapiens
297	11717252_x_at	brown	NADH:ubiquinone oxidoreductase subunit B11(NDUFB11)	Homo sapiens
322	11717407_a_at	brown	ribonuclease P/MRP subunit p25 like(RPP25L)	Homo sapiens
328	11717429_x_at	brown	peroxiredoxin 5(PRDX5)	Homo sapiens
360	11717634_a_at	brown	cytochrome c oxidase subunit 6C(COX6C)	Homo sapiens
363	11717643_a_at	brown	mitochondrial ribosomal protein L24(MRPL24)	Homo sapiens
364	11717654_a_at	brown	C-X9-C motif containing 4(CMC4)	Homo sapiens
368	11717670_a_at	brown	NADH:ubiquinone oxidoreductase subunit B6(NDUFB6)	Homo sapiens
396	11717859_a_at	brown	emopamil binding protein (sterol isomerase)(EBP)	Homo sapiens
427	11718036_at	brown	peptidylprolyl isomerase like 1(PPIL1)	Homo sapiens
437	11718076_at	brown	mitogen-activated protein kinase-activated protein kinase 3(MAPKAPK3)	Homo sapiens
447	11718150_at	brown	protease associated domain containing 1(PRADC1)	Homo sapiens
462	11718276_x_at	brown	interferon related developmental regulator 2(IFRD2)	Homo sapiens
463	11718278_s_at	brown	NADH:ubiquinone oxidoreductase subunit B4(NDUFB4)	Homo sapiens
464	11718282_s_at	brown	NADH:ubiquinone oxidoreductase core subunit V2(NDUFV2)	Homo sapiens
486	11718430_a_at	brown	zinc finger protein 32(ZNF32)	Homo sapiens
531	11718764_at	brown	cytochrome c oxidase subunit 5B(COX5B)	Homo sapiens
532	11718765_a_at	brown	NADH:ubiquinone oxidoreductase subunit A6(NDUFA6)	Homo sapiens
541	11718831_at	brown	NADH:ubiquinone oxidoreductase subunit A2(NDUFA2)	Homo sapiens
570	11718991_a_at	brown	NADH:ubiquinone oxidoreductase subunit B7(NDUFB7)	Homo sapiens
583	11719111_a_at	brown	chromosome 11 open reading frame 1(C11orf1)	Homo sapiens
611	11719304_a_at	brown	ER membrane protein complex subunit 8(EMC8)	Homo sapiens
630	11719424_at	brown	family with sequence similarity 220 member A(FAM220A)	Homo sapiens
639	11719482_a_at	brown	mitochondrial ribosomal protein L21(MRPL21)	Homo sapiens
651	11719566_a_at	brown	mitochondrial ribosomal protein S15(MRPS15)	Homo sapiens
682	11719830_a_at	brown	ERCC excision repair 1, endonuclease non-catalytic subunit(ERCC1)	Homo sapiens
693	11719877_a_at	brown	tRNA phosphotransferase 1(TRPT1)	Homo sapiens
729	11720114_a_at	brown	ubiquitin conjugating enzyme E2 D4 (putative)(UBE2D4)	Homo sapiens
732	11720140_at	brown	Rab interacting lysosomal protein(RILP)	Homo sapiens
740	11720171_s_at	brown	mitochondrial ribosomal protein S16(MRPS16)	Homo sapiens
742	11720188_a_at	brown	NADH:ubiquinone oxidoreductase subunit A7(NDUFA7)	Homo sapiens
744	11720192_at	brown	NADH:ubiquinone oxidoreductase complex assembly factor 4(NDUFAF4)	Homo sapiens
745	11720193_x_at	brown	NADH:ubiquinone oxidoreductase complex assembly factor 4(NDUFAF4)	Homo sapiens
775	11720328_a_at	brown	ATP synthase, H+ transporting, mitochondrial F1 complex, O subunit(ATP5O)	Homo sapiens
794	11720487_a_at	brown	heme binding protein 2(HEBP2)	Homo sapiens
802	11720535_a_at	brown	methylmalonic aciduria (cobalamin deficiency) cblB type(MMAB)	Homo sapiens
815	11720610_x_at	brown	prohibitin(PHB)	Homo sapiens
819	11720630_a_at	brown	small nuclear ribonucleoprotein U11/U12 subunit 25(SNRNP25)	Homo sapiens

820	11720631_a_at	brown	succinate dehydrogenase complex iron sulfur subunit B(SDHB)	Homo sapiens
821	11720632_x_at	brown	succinate dehydrogenase complex iron sulfur subunit B(SDHB)	Homo sapiens
822	11720635_a_at	brown	ATP synthase, H+ transporting, mitochondrial F1 complex, delta subunit(ATP5D)	Homo sapiens
843	11720773_x_at	brown	DnaJ heat shock protein family (Hsp40) member C19(DNAJC19)	Homo sapiens
844	11720774_s_at	brown	DnaJ heat shock protein family (Hsp40) member C19(DNAJC19)	Homo sapiens
851	11720809_x_at	brown	phosphoglycerate dehydrogenase(PHGDH)	Homo sapiens
852	11720821_x_at	brown	FUN14 domain containing 2(FUNDC2)	Homo sapiens
860	11720863_at	brown	patatin like phospholipase domain containing 4(PNPLA4)	Homo sapiens
862	11720884_a_at	brown	hydroxymethylbilane synthase(HMBS)	Homo sapiens
880	11720974_at	brown	ankyrin repeat and SOCS box containing 8(ASB8)	Homo sapiens
898	11721077_a_at	brown	esterase D(ESD)	Homo sapiens
903	11721111_a_at	brown	acyl-CoA thioesterase 13(ACOT13)	Homo sapiens
921	11721265_s_at	brown	glutamyl-tRNA amidotransferase subunit B(GATB)	Homo sapiens
923	11721296_a_at	brown	NADH:ubiquinone oxidoreductase subunit B1(NDUFB1)	Homo sapiens
940	11721461_a_at	brown	COP9 signalosome subunit 5(COPS5)	Homo sapiens
942	11721473_a_at	brown	holocytochrome c synthase(HCCS)	Homo sapiens
968	11721673_at	brown	glutaredoxin 5(GLRX5)	Homo sapiens
969	11721674_s_at	brown	glutaredoxin 5(GLRX5)	Homo sapiens
970	11721675_x_at	brown	glutaredoxin 5(GLRX5)	Homo sapiens
971	11721683_a_at	brown	mitochondrial ribosomal protein S9(MRPS9)	Homo sapiens
993	11721836_x_at	brown	transmembrane protein 14B(TMEM14B)	Homo sapiens
1000	11721851_x_at	brown	coiled-coil-helix-coiled-coil-helix domain containing 6(CHCHD6)	Homo sapiens
1036	11722040_a_at	brown	eukaryotic translation initiation factor 2B subunit gamma(EIF2B3)	Homo sapiens
1054	11722194_a_at	brown	microRNA 4691(MIR4691)	Homo sapiens
1056	11722226_s_at	brown	succinate dehydrogenase complex assembly factor 3(SDHAF3)	Homo sapiens
1077	11722338_at	brown	peroxisomal biogenesis factor 7(PEX7)	Homo sapiens
1163	11723046_a_at	brown	mitochondrial assembly of ribosomal large subunit 1(MALSU1)	Homo sapiens
1166	11723056_a_at	brown	mitochondrial ribosomal protein L33(MRPL33)	Homo sapiens
1167	11723057_x_at	brown	mitochondrial ribosomal protein L33(MRPL33)	Homo sapiens
1174	11723080_at	brown	matrix AAA peptidase interacting protein 1(MAIP1)	Homo sapiens
1211	11723313_s_at	brown	peroxisomal membrane protein 2(PXMP2)	Homo sapiens
1225	11723426_a_at	brown	electron transfer flavoprotein beta subunit(ETFB)	Homo sapiens
1229	11723449_a_at	brown	NADH:ubiquinone oxidoreductase subunit V3(NDUFV3)	Homo sapiens
1230	11723467_a_at	brown	NADH:ubiquinone oxidoreductase subunit A3(NDUFA3)	Homo sapiens
1252	11723695_s_at	brown	heat shock transcription factor 1(HSF1)	Homo sapiens
1322	11724264_a_at	brown	intraflagellar transport 22(IFT22)	Homo sapiens
1340	11724356_a_at	brown	transmembrane protein 177(TMEM177)	Homo sapiens
1364	11724594_at	brown	HRAS like suppressor(HRASLS)	Homo sapiens
1371	11724669_a_at	brown	mitochondrial ribosomal protein S22(MRPS22)	Homo sapiens
1390	11724780_x_at	brown	mitochondrial ribosomal protein S17(MRPS17)	Homo sapiens
1433	11725137_x_at	brown	synaptogyrin 1(SYNGR1)	Homo sapiens
1484	11725563_at	brown	chromosome 12 open reading frame 60(C12orf60)	Homo sapiens
1485	11725590_a_at	brown	nudix hydrolase 6(NUDT6)	Homo sapiens
1486	11725612_x_at	brown	ARMC2 antisense RNA 1(ARMC2-AS1)	Homo sapiens
1491	11725635_a_at	brown	mitochondrial ribosomal protein S25(MRPS25)	Homo sapiens
1495	11725643_a_at	brown	mitochondrial ribosomal protein S26(MRPS26)	Homo sapiens
1536	11725991_a_at	brown	regulator of microtubule dynamics 1(RMDN1)	Homo sapiens
1568	11726264_s_at	brown	STE20-related kinase adaptor beta(STRADB)	Homo sapiens
1584	11726335_a_at	brown	cardiolipin synthase 1(CRLS1)	Homo sapiens
1588	11726350_at	brown	ankyrin repeat domain 39(ANKRD39)	Homo sapiens
1628	11726662_a_at	brown	apolipoprotein O(APOO)	Homo sapiens
1671	11727060_a_at	brown	mitochondrial ribosomal protein S36(MRPS36)	Homo sapiens
1743	11727820_a_at	brown	CDGSH iron sulfur domain 1(CISD1)	Homo sapiens
1768	11728021_a_at	brown	coiled-coil-helix-coiled-coil-helix domain containing 4(CHCHD4)	Homo sapiens
1769	11728027_x_at	brown	coiled-coil domain containing 58(CCDC58)	Homo sapiens
1773	11728040_x_at	brown	peroxiredoxin 5(PRDX5)	Homo sapiens
1784	11728152_a_at	brown	histone cluster 1 H2B family member d(HIST1H2BD)	Homo sapiens
1808	11728294_at	brown	mitochondrial ribosomal protein L58(MRPL58)	Homo sapiens
1809	11728311_x_at	brown	ERCC excision repair 1, endonuclease non-catalytic subunit(ERCC1)	Homo sapiens
1810	11728317_a_at	brown	family with sequence similarity 122A(FAM122A)	Homo sapiens
1825	11728425_s_at	brown	bolA family member 3(BOLA3)	Homo sapiens
1832	11728513_a_at	brown	NADH:ubiquinone oxidoreductase complex assembly factor 6(NDUFAF6)	Homo sapiens
1847	11728673_s_at	brown	kinesin family member 22(KIF22)	Homo sapiens
1855	11728741_at	brown	coenzyme Q3, methyltransferase(COQ3)	Homo sapiens
1892	11729121_a_at	brown	McKusick-Kaufman syndrome(MKKS)	Homo sapiens
1893	11729122_x_at	brown	McKusick-Kaufman syndrome(MKKS)	Homo sapiens
1896	11729131_a_at	brown	proteasome activator subunit 3(PSME3)	Homo sapiens
1900	11729149_a_at	brown	mitochondrial ribosomal protein S23(MRPS23)	Homo sapiens
1916	11729311_a_at	brown	spexin hormone(SPX)	Homo sapiens
1922	11729422_a_at	brown	popeye domain containing 3(POPDC3)	Homo sapiens
1950	11729669_a_at	brown	eukaryotic translation initiation factor 3 subunit K(EIF3K)	Homo sapiens
1962	11729807_a_at	brown	cytochrome c oxidase subunit 6B1(COX6B1)	Homo sapiens
1978	11730044_a_at	brown	chromosome 9 open reading frame 24(C9orf24)	Homo sapiens
1992	11730186_a_at	brown	isocitrate dehydrogenase 3 (NAD(+)) beta(IDH3B)	Homo sapiens
2021	11730451_at	brown	succinate dehydrogenase complex assembly factor 4(SDHAF4)	Homo sapiens
2032	11730659_s_at	brown	3-oxoacyl-ACP synthase, mitochondrial(OXSM)	Homo sapiens
2092	11731248_a_at	brown	transmembrane protein 116(TMEM116)	Homo sapiens
2124	11731671_at	brown	FUN14 domain containing 1(FUNDC1)	Homo sapiens
2178	11732322_at	brown	frataxin(FXN)	Homo sapiens
2203	11732520_a_at	brown	RAD51 paralog C(RAD51C)	Homo sapiens
2215	11732582_a_at	brown	nudix hydrolase 22(NUDT22)	Homo sapiens
2216	11732583_x_at	brown	nudix hydrolase 22(NUDT22)	Homo sapiens

2262	11733134_a_at	brown	mitochondrial ribosomal protein S33(MRPS33)	Homo sapiens
2297	11733535_a_at	brown	nudix hydrolase 7(NUDT7)	Homo sapiens
2298	11733576_a_at	brown	isocitrate dehydrogenase 3 (NAD(+)) beta(IDH3B)	Homo sapiens
2336	11733901_a_at	brown	ubiquinol-cytochrome c reductase complex III subunit VII(UQCRQ)	Homo sapiens
2348	11734033_at	brown	chromosome 15 open reading frame 61(C15orf61)	Homo sapiens
2371	11734491_a_at	brown	spermatogenesis associated 24(SPATA24)	Homo sapiens
2396	11734731_x_at	brown	mitochondrial ribosomal protein S12(MRPS12)	Homo sapiens
2397	11734746_x_at	brown	CDKN2A interacting protein N-terminal like(CDKN2AIPNL)	Homo sapiens
2428	11735096_a_at	brown	MLX, MAX dimerization protein(MLX)	Homo sapiens
2479	11736049_at	brown	family with sequence similarity 229 member B(FAM229B)	Homo sapiens
2480	11736050_s_at	brown	family with sequence similarity 229 member B(FAM229B)	Homo sapiens
2573	11737309_x_at	brown	chromosome 14 open reading frame 2(C14orf2)	Homo sapiens
2600	11738018_a_at	brown	ARV1 homolog, fatty acid homeostasis modulator(ARV1)	Homo sapiens
2612	11738270_a_at	brown	stress associated endoplasmic reticulum protein family member 2(SERP2)	Homo sapiens
2658	11739201_a_at	brown	ATP synthase, H+ transporting, mitochondrial Fo complex subunit C3 (subunit 9)(ATP5G3)	Homo sapiens
2667	11739300_a_at	brown	CDGSH iron sulfur domain 3(CISD3)	Homo sapiens
2696	11739512_a_at	brown	translocase of inner mitochondrial membrane 50(TIMM50)	Homo sapiens
2736	11739854_s_at	brown	LYR motif containing 1(LYRM1)	Homo sapiens
2742	11739911_at	brown	pterin-4 alpha-carbinolamine dehydratase 2(PCBD2)	Homo sapiens
2826	11741031_a_at	brown	HCLS1 associated protein X-1(HAX1)	Homo sapiens
2845	11741192_a_at	brown	ribokinase(RBKS)	Homo sapiens
2857	11741365_x_at	brown	intraflagellar transport 22(IFT22)	Homo sapiens
2889	11741789_a_at	brown	NADH:ubiquinone oxidoreductase subunit B6(NDUFB6)	Homo sapiens
2943	11742736_at	brown	methylmalonyl-CoA epimerase(MCEE)	Homo sapiens
2979	11743002_at	brown	dynactin subunit 6(DCTN6)	Homo sapiens
3009	11743264_x_at	brown	chromosome 14 open reading frame 2(C14orf2)	Homo sapiens
3014	11743330_a_at	brown	phosphoglycolate phosphatase(PGP)	Homo sapiens
3033	11743474_a_at	brown	small integral membrane protein 4(SMIM4)	Homo sapiens
3106	11744040_a_at	brown	cytochrome c oxidase assembly factor 3(COA3)	Homo sapiens
3118	11744148_at	brown	solute carrier family 25 member 10(SLC25A10)	Homo sapiens
3126	11744172_s_at	brown	DnaJ heat shock protein family (Hsp40) member C4(DNAJC4)	Homo sapiens
3130	11744181_a_at	brown	phenylalanyl-tRNA synthetase 2, mitochondrial(FARS2)	Homo sapiens
3133	11744202_at	brown	chromosome 19 open reading frame 70(C19orf70)	Homo sapiens
3134	11744207_a_at	brown	isocitrate dehydrogenase 3 (NAD(+)) beta(IDH3B)	Homo sapiens
3138	11744222_a_at	brown	ATP synthase, H+ transporting, mitochondrial F1 complex, delta subunit(ATP5D)	Homo sapiens
3139	11744223_x_at	brown	ATP synthase, H+ transporting, mitochondrial F1 complex, delta subunit(ATP5D)	Homo sapiens
3140	11744227_at	brown	MACRO domain containing 1(MACROD1)	Homo sapiens
3150	11744284_a_at	brown	mitochondrial ribosomal protein S12(MRPS12)	Homo sapiens
3160	11744384_x_at	brown	up-regulated during skeletal muscle growth 5 homolog (mouse)(USMG5)	Homo sapiens
3161	11744400_a_at	brown	myosin light chain 6B(MYL6B)	Homo sapiens
3162	11744401_a_at	brown	calmodulin binding transcription activator 1(CAMTA1)	Homo sapiens
3165	11744430_a_at	brown	kinesin family member 9(KIF9)	Homo sapiens
3170	11744444_a_at	brown	NADH:ubiquinone oxidoreductase subunit B10(NDUFB10)	Homo sapiens
3173	11744455_s_at	brown	ubiquinol-cytochrome c reductase hinge protein like(UQCRHL)	Homo sapiens
3183	11744545_s_at	brown	GTF2I repeat domain containing 2B(GTF2IRD2B)	Homo sapiens
3201	11744672_a_at	brown	MORN repeat containing 4(MORN4)	Homo sapiens
3224	11744822_a_at	brown	NADH:ubiquinone oxidoreductase subunit B2(NDUFB2)	Homo sapiens
3227	11744838_a_at	brown	dual specificity phosphatase 28(DUSP28)	Homo sapiens
3241	11744978_a_at	brown	origin recognition complex subunit 4(ORC4)	Homo sapiens
3251	11745124_a_at	brown	3-hydroxybutyrate dehydrogenase, type 1(BDH1)	Homo sapiens
3266	11745259_a_at	brown	chromosome 11 open reading frame 21(C11orf21)	Homo sapiens
3276	11745384_s_at	brown	NADH:ubiquinone oxidoreductase subunit B4(NDUFB4)	Homo sapiens
3283	11745460_at	brown	NADH:ubiquinone oxidoreductase subunit V3(NDUFV3)	Homo sapiens
3299	11745555_a_at	brown	chromosome 11 open reading frame 74(C11orf74)	Homo sapiens
3300	11745556_x_at	brown	chromosome 11 open reading frame 74(C11orf74)	Homo sapiens
3368	11746331_a_at	brown	adenosine kinase(ADK)	Homo sapiens
3373	11746372_a_at	brown	nudix hydrolase 6(NUDT6)	Homo sapiens
3390	11746586_a_at	brown	NADH:ubiquinone oxidoreductase core subunit S7(NDUFS7)	Homo sapiens
3413	11746855_a_at	brown	NAD(P)HX epimerase(NAXE)	Homo sapiens
3448	11747278_a_at	brown	malate dehydrogenase 2(MDH2)	Homo sapiens
3547	11748343_a_at	brown	intraflagellar transport 22(IFT22)	Homo sapiens
3548	11748344_x_at	brown	intraflagellar transport 22(IFT22)	Homo sapiens
3571	11748695_a_at	brown	synaptogyrin 1(SYNGR1)	Homo sapiens
3613	11749229_a_at	brown	sirtuin 5(SIRT5)	Homo sapiens
3630	11749398_a_at	brown	B9 domain containing 1(B9D1)	Homo sapiens
3631	11749399_x_at	brown	MAPK regulated corepressor interacting protein 2(MCRIP2)	Homo sapiens
3635	11749430_a_at	brown	NADH:ubiquinone oxidoreductase complex assembly factor 6(NDUFAF6)	Homo sapiens
3686	11750006_a_at	brown	cytochrome c oxidase assembly factor 1 homolog(COA1)	Homo sapiens
3709	11750223_a_at	brown	NAD(P)HX epimerase(NAXE)	Homo sapiens
3715	11750293_a_at	brown	RAD51 paralog C(RAD51C)	Homo sapiens
3810	11751680_a_at	brown	ubiquinol-cytochrome c reductase binding protein(UQCRB)	Homo sapiens
3816	11751746_a_at	brown	guanidinoacetate N-methyltransferase(GAMT)	Homo sapiens
3821	11751778_s_at	brown	translocase of inner mitochondrial membrane 23(TIMM23)	Homo sapiens
3935	11753322_a_at	brown	diphthamide biosynthesis 5(DPH5)	Homo sapiens
3964	11753755_x_at	brown	ATP synthase, H+ transporting, mitochondrial Fo complex subunit F2(ATP5J2)	Homo sapiens
3966	11753799_a_at	brown	small integral membrane protein 11A(SMIM11A)	Homo sapiens
3967	11753800_x_at	brown	small integral membrane protein 11A(SMIM11A)	Homo sapiens
3971	11753843_a_at	brown	NADH:ubiquinone oxidoreductase subunit A3(NDUFA3)	Homo sapiens
3972	11753844_x_at	brown	NADH:ubiquinone oxidoreductase subunit A3(NDUFA3)	Homo sapiens
3977	11753871_a_at	brown	G protein subunit gamma 7(GNG7)	Homo sapiens
3978	11753872_x_at	brown	metallothionein 3(MT3)	Homo sapiens
3993	11753996_x_at	brown	heat shock protein family E (Hsp10) member 1(HSPE1)	Homo sapiens

3994	11754015_a_at	brown	translocase of inner mitochondrial membrane 50(TIMM50)	Homo sapiens
3995	11754016_x_at	brown	translocase of inner mitochondrial membrane 50(TIMM50)	Homo sapiens
4000	11754034_a_at	brown	reactive intermediate imine deaminase A homolog(RIDA)	Homo sapiens
4010	11754161_s_at	brown	phosphatidylinositol glycan anchor biosynthesis class P(PIGP)	Homo sapiens
4053	11754444_x_at	brown	cytochrome c oxidase subunit 6C(COX6C)	Homo sapiens
4059	11754489_a_at	brown	NADH:ubiquinone oxidoreductase complex assembly factor 5(NDUFAF5)	Homo sapiens
4077	11754623_x_at	brown	choline phosphotransferase 1(CHPT1)	Homo sapiens
4083	11754652_a_at	brown	enoyl-CoA delta isomerase 2(ECI2)	Homo sapiens
4090	11754707_a_at	brown	CUE domain containing 2(CUEDC2)	Homo sapiens
4104	11754783_x_at	brown	COMM domain containing 3(COMMD3)	Homo sapiens
4117	11754861_s_at	brown	succinate-CoA ligase alpha subunit(SUCLG1)	Homo sapiens
4132	11754964_a_at	brown	NADH:ubiquinone oxidoreductase subunit A10(NDUFA10)	Homo sapiens
4175	11755269_a_at	brown	URGCP-MRPS24 readthrough(URGCP-MRPS24)	Homo sapiens
4191	11755346_x_at	brown	ATP synthase, H+ transporting, mitochondrial F1 complex, O subunit(ATP5O)	Homo sapiens
4192	11755347_x_at	brown	secretion associated Ras related GTPase 1B(SAR1B)	Homo sapiens
4210	11755458_a_at	brown	HD domain containing 2(HDDC2)	Homo sapiens
4239	11755710_a_at	brown	GTP binding protein 8 (putative)(GTPBP8)	Homo sapiens
4256	11755876_a_at	brown	interferon related developmental regulator 2(IFRD2)	Homo sapiens
4285	11756125_x_at	brown	NADH:ubiquinone oxidoreductase subunit A1(NDUFA1)	Homo sapiens
4298	11756211_s_at	brown	Hikeshi, heat shock protein nuclear import factor(HIKESHI)	Homo sapiens
4324	11756351_x_at	brown	superoxide dismutase 1, soluble(SOD1)	Homo sapiens
4346	11756500_x_at	brown	diphthamide biosynthesis 5(DPH5)	Homo sapiens
4351	11756526_a_at	brown	mitochondrial ribosomal protein L48(MRPL48)	Homo sapiens
4368	11756652_a_at	brown	N-terminal Xaa-Pro-Lys N-methyltransferase 1(NTMT1)	Homo sapiens
4373	11756687_a_at	brown	mitochondrial ribosomal protein L46(MRPL46)	Homo sapiens
4382	11756757_x_at	brown	short chain dehydrogenase/reductase family 39U member 1(SDR39U1)	Homo sapiens
4426	11757073_s_at	brown	THAP domain containing 4(THAP4)	Homo sapiens
4427	11757083_s_at	brown	coiled-coil-helix-coiled-coil-helix domain containing 3(CHCHD3)	Homo sapiens
4438	11757265_x_at	brown	eukaryotic translation initiation factor 3 subunit K(EIF3K)	Homo sapiens
4439	11757267_x_at	brown	sirtuin 2(SIRT2)	Homo sapiens
4444	11757279_a_at	brown	translocase of inner mitochondrial membrane domain containing 1(TIMMDC1)	Homo sapiens
4445	11757281_a_at	brown	ATP synthase, H+ transporting, mitochondrial Fo complex subunit C3 (subunit 9)(ATP5G3)	Homo sapiens
4446	11757282_x_at	brown	ATP synthase, H+ transporting, mitochondrial Fo complex subunit C3 (subunit 9)(ATP5G3)	Homo sapiens
4454	11757330_x_at	brown	small nuclear ribonucleoprotein polypeptide C(SNRPC)	Homo sapiens
4456	11757334_a_at	brown	ubiquinol-cytochrome c reductase, complex III subunit XI(UQCR11)	Homo sapiens
4471	11757405_a_at	brown	methionine sulfoxide reductase B2(MSRB2)	Homo sapiens
4473	11757413_a_at	brown	NADH:ubiquinone oxidoreductase core subunit S3(NDUFS3)	Homo sapiens
4477	11757429_a_at	brown	cytochrome c1(CYC1)	Homo sapiens
4483	11757446_x_at	brown	MINOS1-NBL1 readthrough(MINOS1-NBL1)	Homo sapiens
4495	11757485_x_at	brown	family with sequence similarity 129 member B(FAM129B)	Homo sapiens
4513	11757567_x_at	brown	NADH:ubiquinone oxidoreductase subunit B5(NDUFB5)	Homo sapiens
4516	11757589_a_at	brown	NADH:ubiquinone oxidoreductase subunit A12(NDUFA12)	Homo sapiens
4519	11757601_a_at	brown	COP9 signalosome subunit 3(COPS3)	Homo sapiens
4522	11757613_x_at	brown	eukaryotic translation initiation factor 3 subunit K(EIF3K)	Homo sapiens
4529	11757649_a_at	brown	CORO7-PAM16 readthrough(CORO7-PAM16)	Homo sapiens
4535	11757670_a_at	brown	ubiquinol-cytochrome c reductase, complex III subunit X(UQCR10)	Homo sapiens
4536	11757672_s_at	brown	mitochondrial ribosomal protein S17(MRPS17)	Homo sapiens
4540	11757727_x_at	brown	ATP synthase, H+ transporting, mitochondrial F1 complex, O subunit(ATP5O)	Homo sapiens
4555	11757825_a_at	brown	C7orf55-LUC7L2 readthrough(C7orf55-LUC7L2)	Homo sapiens
4559	11757847_s_at	brown	ubiquinol-cytochrome c reductase, Rieske iron-sulfur polypeptide 1(UQCRFS1)	Homo sapiens
4560	11757863_x_at	brown	transmembrane protein 261(TMEM261)	Homo sapiens
4578	11757936_s_at	brown	glycine cleavage system protein H pseudogene 3(GCSHP3)	Homo sapiens
4613	11758135_s_at	brown	chromosome 14 open reading frame 2(C14orf2)	Homo sapiens
4651	11758314_s_at	brown	NADH:ubiquinone oxidoreductase complex assembly factor 2(NDUFAF2)	Homo sapiens
4658	11758343_s_at	brown	up-regulated during skeletal muscle growth 5 homolog (mouse)(USMG5)	Homo sapiens
4685	11758566_s_at	brown	BCS1 homolog, ubiquinol-cytochrome c reductase complex chaperone(BCS1L)	Homo sapiens
4702	11758717_x_at	brown	NDUFA4, mitochondrial complex associated(NDUFA4)	Homo sapiens
4703	11758727_x_at	brown	ATP synthase, H+ transporting, mitochondrial F1 complex, gamma polypeptide 1(ATP5C1)	Homo sapiens
4712	11758795_at	brown	mitochondrial ribosomal protein L45(MRPL45)	Homo sapiens
4769	11759522_a_at	brown	alkB homolog 7(ALKBH7)	Homo sapiens
4770	11759523_x_at	brown	alkB homolog 7(ALKBH7)	Homo sapiens
4772	11759553_at	brown	translocase of inner mitochondrial membrane 8 homolog B(TIMM8B)	Homo sapiens
4777	11759575_at	brown	ubiquinol-cytochrome c reductase hinge protein like(UQCRHL)	Homo sapiens
4787	11759645_x_at	brown	COX11, cytochrome c oxidase copper chaperone(COX11)	Homo sapiens
4794	11759687_a_at	brown	small nuclear RNA activating complex polypeptide 5(SNAPC5)	Homo sapiens
4834	11760482_x_at	brown	NADH:ubiquinone oxidoreductase core subunit S7(NDUFS7)	Homo sapiens
4846	11761076_x_at	brown	acyl-CoA thioesterase 8(ACOT8)	Homo sapiens
4881	11762275_x_at	brown	ubiquinol-cytochrome c reductase binding protein(UQCRB)	Homo sapiens
4904	11763032_x_at	brown	adipogenesis associated Mth938 domain containing(AAMDC)	Homo sapiens
4905	11763101_x_at	brown	NADH:ubiquinone oxidoreductase subunit B9(NDUFB9)	Homo sapiens
4964	11763960_a_at	brown	NADH:ubiquinone oxidoreductase subunit B3(NDUFB3)	Homo sapiens
4966	11763990_a_at	brown	alkB homolog 7(ALKBH7)	Homo sapiens
4988	200086_PM_s_at	brown	cytochrome c oxidase subunit 4I1(COX4I1)	Homo sapiens

Grey (unassigned) Module

	gene	hcolor	Name	Species
221	11716694_at	grey	tetraspanin 6(TSPAN6)	Homo sapiens
604	11719232_x_at	grey	protein tyrosine phosphatase, receptor type S(PTPRS)	Homo sapiens
976	11721706_at	grey	glycerol-3-phosphate acyltransferase, mitochondrial(GPAM)	Homo sapiens
1699	11727404_a_at	grey	glypican 4(GPC4)	Homo sapiens
4330	11756375_x_at	grey	ETS variant 7(ETV7)	Homo sapiens
4399	11756854_a_at	grey	EH domain binding protein 1(EHBP1)	Homo sapiens
